# Identification of quantitative trait loci and candidate genes for pod shatter resistance in *Brassica carinata*

**DOI:** 10.1186/s12870-024-05596-2

**Published:** 2024-09-30

**Authors:** Rosy Raman, Zun Xu Zhang, Simon Diffey, Yu Qiu, Yan Niu, Jun Zou, Harsh Raman

**Affiliations:** 1 NSW Department of Primary Industries and Regional Development, Wagga Wagga Agricultural Institute, Wagga Wagga, NSW 2650 Australia; 2 National Key Laboratory of Crop Genetic Improvement, College of Plant Science & Technology, Huazhong Agricultural University, Wuhan, Hubei 430070 China; 3 Apex Biometry Pty. Ltd., South Freemantle, WA 6162 Australia

**Keywords:** *Brassica carinata*, Pod shattering, Domestication, Genetic mapping, Ethiopian mustard, Genetic analysis, Sequence variation

## Abstract

**Background:**

Understanding the genetic control of pod shatter resistance and its association with pod length is crucial for breeding improved pod shatter resistance and reducing pre-harvest yield losses due to extensive shattering in cultivars of *Brassica species*. In this study, we evaluated a doubled haploid (DH) mapping population derived from an F_1_ cross between two *Brassica carinata* parental lines Y-BcDH64 and W-BcDH76 (YWDH), originating from Ethiopia and determined genetic bases of variation in pod length and pod shatter resistance, measured as rupture energy. The YWDH population, its parental lines and 11 controls were grown across three years for genetic analysis.

**Results:**

By using three quantitative trait loci (QTL) analytic approaches, we identified nine genomic regions on B02, B03, B04, B06, B07 and C01 chromosomes for rupture energy that were repeatedly detected across three growing environments. One of the QTL on chromosome B07, flanked with DArTseq markers 100,046,735 and 100,022,658, accounted for up to 27.6% of genetic variance in rupture energy. We observed no relationship between pod length and rupture energy, suggesting that pod length does not contribute to variation in pod shatter resistance. Comparative mapping identified six candidate genes; *SHP1 on B6*, *FUL* and *MAN* on chromosomes B07, *IND* and *NST2* on B08, and *MAN7* on C07 that mapped within 0.2 Mb from the QTL for rupture energy.

**Conclusion:**

The results suggest that favourable alleles of stable QTL on B06, B07, B08 and C01 for pod shatter resistance can be incorporated into the shatter-prone *B. carinata* and its related species to improve final seed yield at harvest.

**Supplementary Information:**

The online version contains supplementary material available at 10.1186/s12870-024-05596-2.

## Background

Ethiopian mustard (*Brassica carinata* A. Braun, 2*n* = 4*x* = 34, genome: B^c^B^c^C^c^C^c^ ) is an allotetraploid member of the family Brassicaceae and formed as a result of interspecific hybridisation between ancestors of diploid *Brassica nigra* (2*n* = 16, genome BB) and *Brassica oleracea* (2*n* = 18, genome CC) [1, 2]. It is believed to have originated in Ethiopia and possibly in East Africa and the Mediterranean coast [3]. *B carinata* is grown as a cover and cash crop, a leafy vegetable, and for oil, medicines, and condiments [4]. However, due to the presence of high erucic acid (> 40%), its oil is considered unhealthy for human consumption. In recent years, *B. carinata* has been exploited as a dedicated feedstock for renewable jet fuel, biodiesel and other byproducts [5]. Compared to other members of oilseed Brassica crops such as *Brassica rapa*, *Brassica napus*, and *Brassica juncea*, little research has been conducted on the genetic improvement in *B. carinata* and only limited cultivars have been released for commercial cultivation worldwide. Research has shown that there is a limited genetic variation in *B. carinata* germplasm due to stronger domestication bottlenecks [1, 6, 7]. Reduced pod shattering is one of the domestication traits in several crops including domesticated members of Brassicaceae, Fabaceae and Gramineae, which suffer serious yield losses due to seed shattering.

Genetic improvement for pod shatter resistance is one of the major objectives of several brassica breeding programs, including *B. carinata*. Previous research has shown that *B. carinata* is generally more resistant to pod shatter compared to other oilseed brassicas [8]. However, different accessions of *B. carinata* show a range of variations in pod shatter resistance. For example, Raman et al. [8] showed a range of variation for pod shatter resistance; based on pod rupture energy (RE, varying from 2.53 to 20.82 mJ), measured using a pendulum test developed by [9].

To expedite the allele introgression for valuable traits for the industry and understand the genetic architecture of traits, genome assemblies, genotyping platforms and genetic analysis methods have been developed in *B. carinata* [7, 8, 10–15]. In general, *B. carinata* accessions show a greater level of pod shatter resistance than other *Brassica species* [8, 16]. Raman et al. (8) investigated the genetic control of shatter resistance using the pendulum test [9, 17] in an F_2:3_ population from two contrasting *B. carinata* parental lines, BC73526 (shatter resistant with high RE) and BC73524 (shatter prone with low RE) and identified five statistically significant QTL on chromosomes B01, B03, B08, and C05. The QTL on B01, B03, and B08 were recently remapped and anchored to the B07, B08, and B02 pseudomolecules respectively on the pan-genome of *B. carinata* [12]. Based on the phenotypic data published (8), Niu et al., [12] verified QTL associated with pod shatter resistance using a whole genome resequencing (WGS)-based BSA approach and identified a major locus on B07, along with two minor QTL on B02 and B08 chromosomes. Further, Niu et al., [12] prioritised a candidate gene for pod shatter resistance, *FRUITFUL* (*FUL*; *BcaFUL.B7*) that had the highest expression in pods, within the overlapping major QTL region in the BC73526/BC73524 population.

The *B. carinata* DH mapping population derived from Y-BcDH64 (yellow petal and yellow seed coat; Yellowcross) and W-BcDH76 (white petal, Whiteban and brown seed coat), also referred to as a YWDH population show substantial genetic variation for budding and flowering time, seed yield, and yield-related traits (pod width, pod length, seed number per pod, seed weight, pod number on main inflorescence, and length of main inflorescence) and seed quality traits (protein content, oil content, erucic acid, linolenic acid, linoleic acid, and oleic acid) [7, 10, 18]. However, the genetic architecture of pod-shatter resistance in this population has not been reported and it deserves further testing if this population could provide novel QTL for genetic improvement of *B. carinata*. This YWDH population (*n* = 93 to 185 lines) has been previously mapped with 214 conventional markers (151 SSR markers; 44 AFLP makers; five IBP markers; 12 SRAP markers; and two morphological markers based on anther colour and seed colour), 4,031 high-density DArTseq and 16,321 WGS-based markers [7, 10, 16, 19]. Utilising these available genetic resources, we investigated the (i) extent of genetic variation for pod shatter resistance, measured as RE, and pod length (ii) identification of genomic regions associated with RE and pod length, and (iii) prioritising candidate genes associated with pod shatter resistance in the YWDH population. In addition, we compared the QTL for pod shatter resistance across two *B. carinata* populations that have been mapped so far, based on the physical position of markers using the recently published pan-genome [12].

## Materials and methods

### Mapping population

The YWDH population consisting of 188 lines was used in this study and the parental lines, Yellocross and Whiteban were obtained from the Centre for Genetic Resources, Wageningen, The Netherlands and Germany, respectively [11]. To compare the level of pod shatter resistance in the YWDH population with other related species and determine the stability of phenotypes across environments, we used 11 controls comprising *B. rapa* (Torch), *B. napus* (BLN2762, BLN3614, Chon nam, OasisCL, Surpass400, ) and *B. carinata* (ATC93184-1, ATC94126, ATC93883, ATC94114, ATC94348, accessed from the Australian Grains Genebank, Horsham) along with the parental lines and YWDH population, for field trials.

### Evaluation for pod shatter resistance

We conducted three experiments: two in pots (2013, 2014) and one under field conditions (2023). Both parental lines and 186 lines of the YWDH population were grown in 2013, and 2014 in white plastic pots (Garden City Plastics, NSW, Australia) under birdcage conditions, whereas, the YWDH population was sown in two-row plots (6 m long x 75 cm wide) in the field at the Wagga Wagga Agricultural Institute, New South Wales, Australia. Five plants of each accession were raised per pot whereas 50 seeds were sown in each row plot. All birdcage and field experiments comprised DH lines of the YW population, two parental lines and 11 controls of *B. rapa*, *B. napus* and *B. carinata* and were arranged in randomized complete block designs, with two (2013, 2014) to three replicates (2023). Standard practices for the cultivation of canola plants and pod collection for pendulum test to detect genetic variation in rupture energy - a measure for pod strength/resistance to shattering [9, 17] were followed as described previously [16].

### Statistical and QTL analysis

The linkage map of the YWDH population, constructed previously [10] was used for QTL analysis using the WGAIM: Whole Genome Average Interval Mapping [20] as described in detail by [21], IciMapping version 4.2 [22] and WinQTL cartographer version 2.5 (http://statgen.ncsu.edu/qtlcart/WQTLCart.htm). For the WinQTL cartographer, a composite interval mapping model was used for QTL identification [23] as described in [10]. The inclusive composite interval mapping (ICIM-ADD) method was used to identify QTL using IciMapping software. Marker intervals that map within 2 cM across environments (years) were considered the same QTL.

A linear mixed model was developed for the phenotypic data for each trait as described in [24]. Field or birdcage spatial variability and temporal variation in the laboratory were considered for each trait using the methods described by [25]. Broad-sense heritability (*h*^2^ ), the coefficient of variation (CV) and line-best linear unbiased predictions (BLUPs) for RE and pod length were calculated from the final model developed for each trait and experiment combination.

### Alignment of markers with the pan-genome

We used the pan-genome of *B. carinata* [12] to align the physical positions of the 1366 markers which were genotyped in the earlier studies [10] and QTL that were identified in this study using BLASTN (v2.5.0+) [26]. When the QTL regions of the YWDH and BC73526/BC73524 population showed alignment to the same physical position on the pan-genome, we assumed the homologous QTL control variation in the trait(s) of interest. To prioritise the candidate genes, we used the *Arabidopsis thaliana* protein sequences of the *priori* pod dehiscence genes from the Arabidopsis Information Resource (TAIR) and searched the homologues in the *B. carinata* pan*-*genome.

## Results

### Inheritance of pod shatter resistance and pod length

The estimated mean of parental lines, range, coefficient of variation and *h*^*2*^ values of pod length and rupture energy measured in DH lines across three growing environments are given in Table 1. There were high values for *h*^*2*^ (71.1 to 85.7%) for rupture energy and pod length (85.2 to 92.8%), suggesting that both traits are genetically determined and are stable across environments.

**Table 1:** Descriptive statistics for genetic variation for rupture energy and pod length traits of the YW doubled haploid population grown across three growing seasons (2013, 2014 and 2023) at the Wagga Wagga Agricultural Institute, Australia

Trait	Growing season	Mean	Range	Coefficient of variation		Broad sense heritability (h^2^; %)
Pod Length (mm)	2013	55.6	46.30–70.46	8.07		85.20
Pod Length (mm)	2014	57.4	42.65–74.50	7.06		90.10
Pod Length (mm)	2023	54.43	41.74–72.89	5.35		92.80
Rupture Energy (mJ)	2013	10.52	4.40–20.30	34.17		71.10
Rupture Energy (mJ)	2014	12.91	4.14–22.70	29.83		76.40
Rupture Energy (mJ)	2023	13.33	4.00–20.30	17.40		85.70

**Fig. 1:**
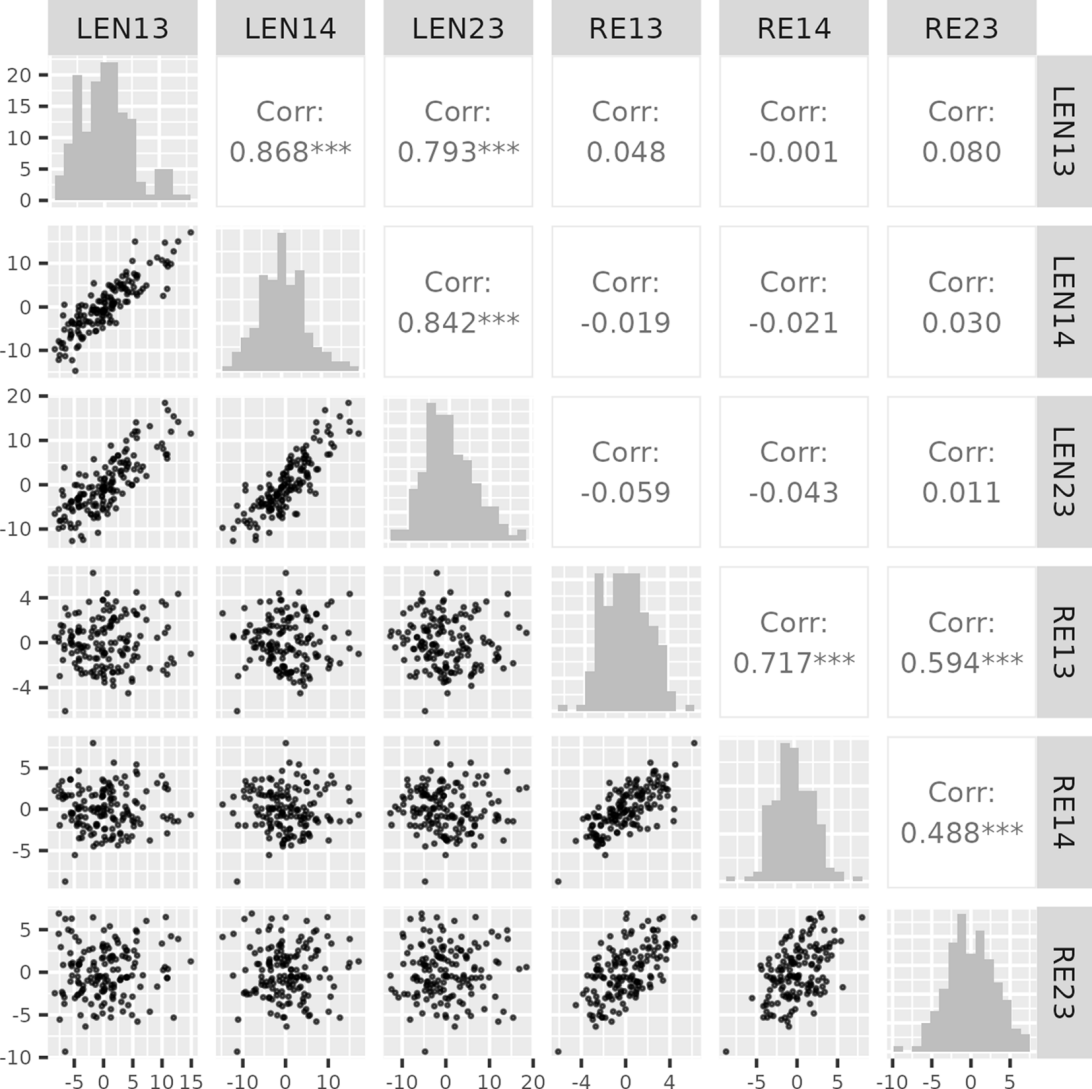
Scatter plots (lower triangles and correlations (upper triangles ) among traits (pod length: LEN; Rupture energy: RE) in the doubled haploid population derived from Yellowcross/Whiteban population grown across three environments (2023, 2014 and 2013). *** indicates P < 0.001, no stars Indicates P > 0.1”. Note that two stars would be P < 0.01 and one star P < 0.1 but these values are not required for this figure

Both parental lines ‘Yellowcross’ (Y) and ‘Whiteban’ (W) differed significantly in pod shatter resistance scores (Table S1; Fig. S1). The paternal parent, Whiteban required a higher level of RE (20.30–22.71 mJ) to break up the pod and release seed than the maternal line, Yellowcross (6.33–10.90 mJ). A wide range of phenotypic variation was observed for pod length (41.74 mm to 74.50 mm) and RE (4.0 to 22.7 mj) among the DH lines under different environments (Table S1, Table S2, Fig. 1). Among the checks, *B. carinata* accession ATC94126 (BC73526) had the maximum pod strength (RE: 19.33 ± 3.27) while *B. napus* varieties OasisCL and Surpass400 had the minimum pod strength (RE: 2.04–2.43) Table S1b. Frequency distribution analysis for trait means clearly showed segregation in the DH lines across environments (Fig. 1, Fig. S1).

In the 2023 environment, 13 lines (YW016, YW064, YW080, YW088, YW117, YW126, YW143, YW145, YW152, YW160, YW179, YW183, and YW186) had more extreme values than Yellowcross, suggesting transgressive segregation (Table S1). Whiteban had a higher pod shatter resistance compared to different controls tested (Figure S1), suggesting that it has superior alleles for resistance and can be exploited in the breeding program to develop improved cultivars for pod shatter resistance. Pod shatter resistance was positively correlated (0.488 to 0.717) across three environments suggesting that the genetic variation in pod shatter resistance is stable (Fig. 1). There were also higher correlation values for pod length (0.793 to 0.868) across environments compared to RE (Fig. 1).

### Correlation between pod length and rupture energy

Previous studies showed an inconsistent relationship between pod length and RE [16, 27, 28]. Therefore, we collected data on the length of pods from the test lines that were used to measure RE. Our results showed no relationship (*r* = 0.04) between pod length and pod shatter resistance based on RE (Fig. 1), suggesting that the two traits develop independently of each other in the YW population.

### Alignment of YWDH genetic map to pangenome of *B. carinata*

Earlier, Zou et al., [10] constructed an integrated linkage map (2,048 cM) of the YWDH population (*n* = 185) that comprised 4,031 DArTseq and other conventional markers corresponding to 1,366 unique loci. After the *B. nigra* Ni100 and *B. oleracea TO1000* genomes were published and made available in the public domain, several linkage groups of the YW DH genetic map have been renamed and oriented according to the alignment with the pan-genome of *B. carinata* [12] (Table S3). The marker sequences that aligned to multiple locations or returned no hits (no positions) were discarded. Of the 1,366 unique marker loci, 1,105 were mapped to the reference pan-genome (Table S4). Alignment between the genetic and physical map also hinted at potential genetic rearrangements on chromosomes B01, C05 and C06 (Fig. S2). We used the corrected genetic map in this study for the identification of QTL and candidate genes for pod shatter resistance.

### QTL associated with resistance to pod shatter

In this study, three approaches based on WGAIM, WINQTL and IciMapping were used for identifying QTL associated with pod shatter resistance and pod length (Table S5). A total of 21 QTL that had a LOD score ≥ 3 were identified for RE in the YWDH population across three analytic approaches (Table 2). Of them, 10 putative QTL were repeatedly detected (within 2 cM) across three growing environments and the different analytical approaches on chromosomes B02, B03, B06, B07, B08, C01, C06 and C07 (Table 2). We observed that some of the QTL on B07, B08, and C07 chromosomes had LOD scores < 3 were repeatedly detected (Table 2. Table S5), suggesting that these genomic regions could be of significance for pod shatter trait enhancement in breeding programs.

**Table 2:** Quantitative trait loci associated with pod shatter resistance were measured using a pendulum test based on rupture energy (RE) across three environments (2013, 2014 and 2023). QTL were identified using the whole genome average interval mapping (WGAIM). Marker intervals that are in bold represent genomic regions that consistently detected and those are in italics and bold may represent the same genomic region (QTL). A detailed QTL summary is given in Table S4

	Chromosome	Left marker	Left marker position (cM)	Right marker	Right marker position (cM)	Allelic effect size	Genotypic variance (%)	LOD
RE 2013	B02	100031592	24.23	100047102	24.53	0.65	6.10	2.96
RE 2014	B02	100019770	49.36	100019364	49.78	0.70	6.00	3.04
RE 2023	B03	100022962	13.77	100052281	14.15	-1.00	9.00	4.63
RE 2023	B04	100043850	33.95	100078929	34.38	0.85	6.50	3.17
**RE 2013**	**B06**	**100013746**	**94.53**	**100001380**	**94.8**	**0.78**	**8.50**	**4.79**
**RE 2023**	**B06**	**100001380**	**94.8**	**100025434**	**101.81**	**0.92**	**7.60**	**3.62**
***RE 2013***	***B07***	***100046735***	***20.82***	***100022658***	***21.56***	***1.43***	***27.60***	***15.63***
***RE 2023***	***B07***	***100053770***	***28.09***	***100019213***	***29.28***	***0.72***	***4.60***	***2.42***
RE 2014	B07	100043465	42.9	100071901	43.31	1.22	17.30	10.21
***RE 2014***	***B08***	***100024082***	***12.78***	***100042491***	***12.98***	***0.55***	***3.80***	***2.29***
***RE 2023***	***B08***	***100002494***	***18.41***	***100024018***	***19.01***	***0.90***	***7.30***	***3.76***
RE 2013	B08	100064677	110.55	100024312	111.38	-0.58	4.90	2.66
***RE 2013***	***C01***	***Na10H06***	***76.11***	***100031795***	***87.7***	***0.75***	***6.80***	***3.90***
***RE 2014***	***C01***	***100062611***	***89.8***	***100059879***	***98.74***	***0.75***	***6.10***	***3.71***
RE 2013	C03	100055560	67.23	100052674	76.3	0.65	5.20	3.01
RE 2023	C04	100023632	2.88	100019506	3.09	-0.75	5.00	2.52
RE 2014	C04	100008104	140.45	Ol11D12	141.22	0.84	8.30	4.93
RE 2023	C06	HG-FT-C6a	67.92	100017755	69.32	1.12	11.20	5.67
RE 2014	C07	100065291	22.83	100037708	26.01	0.67	5.40	3.09

Our analysis showed that different mapping approaches vary in terms of QTL detection. For example, IciMapping detected eight QTL having LOD scores of ≥ 3 on B02, B03, B07, B08 and C06, while WinQTL mapping detected 13 QTL (with LOD of ≥ 3) on B02, B03, B06, and B07, B08, C01, C03, C04, C06 and C07 chromosomes (Table S5). WGAIM detected 16 QTL for pod shatter resistance on B02, B03, B04, B06, B07, B08, C01, C03, C04, C06 and C07 across three environments (Table S5). Based on the genetic positions of significantly associated markers, we identified two stable QTL regions for pod shatter resistance detected with WGAIM in at least two growing seasons. These were located on B06 (100013746–100025434 marker interval) and C01 (*Na10H06*- 100059879 marker interval) and accounted for up to 8.50% of the genotypic variance (Table 2). Among QTL detected, the genomic region flanked with DArTseq markers 100046735 and 100022658 with a LOD score of 15.63 on B07 accounted for the maximum (27.6%) of the genotypic variance in RE (Table 2, Table S5). The direction of the allelic effect of QTL suggested that both parental lines; Yellowcross and Whiteban contribute to the variation in RE, ranging from − 1.0 to 1.43 mJ (Table 2). The pod shatter-resistant parent, Whiteban contributed alleles for pod shatter resistance on QTL mapped on the B02, B04, B06, B07, B08 and C01, C03, C04, C06 and C07 chromosomes (Table 2) whereas the Yellowcross parental line contributed alleles for resistance on the B03, B08, and C04 chromosomes.

 In a previous study, 93 YWDH lines were mapped with 16,632 markers using a whole genome resequencing approach [19]. To determine whether this dataset of selective genotyping from a few lines (93 DH lines, small population size) using high marker density can be used as an alternative approach to classical QTL analysis using moderate population size and marker density used in this study. Based on the LD, a total of 2,833 haplotype blocks (HB) were detected (Table S6) and then performed genome-wide association analysis. Haplotype Trend Regression (HTR) analysis revealed that 56 SNP HB of chromosome B07 had a significant association (LOD ≥ 3) with pod shatter resistance in the 2013 growing season, while in 2014, four genomic regions delimited with HB on B07 and one of B02 chromosome showed a significant association with pod shatter resistance (Table S7a, b). In the 2023 environment, 23 HB mapped to B01, B02, B03, B07 and C06 chromosomes showed statistical association with pod shatter resistance (Table S7c). Across three environments, HB #1362 marked with M9620644, M9626667, M9633923, M9635330, M9635946, M9663678, and M9667355 had a significant association (LOD 5.85) with shatter resistance. Based on the haplotypes, we binned WGS markers into 2,833 unique loci (Table S6), constructed a synthetic map based on physical position on the pan-genome (Table S8a) and performed QTL analysis using the IciMapping approach which identified six significant genomic regions associated with RE on B03, B07, C01, C05, and C06, with LOD scores of 4.07 to 10.63 across three environments in 93 DH lines (Table S8b-c). Of them, the genomic region flanked with SNP1338 (M5120118)/1339(M5220242) which were mapped onto 40.9–51.9 Mb on chromosome B07 of the pan-genome sequence accounted for 20.18% of the variation in pod shatter resistance (Table S8). These binned loci SNP1338 (M5120118/M5336932/M534422/M55346221)/1339(M5220242/M5375029/M5385024,) represent the 4.62/5.09 Mb to 4.69/5.34 Mb of the chromosome B07 sequence of pan-genome.

### QTL associated with pod length

We further detected QTL associated with pod length using WGAIM and identified 29 significant genomic regions (LOD ≥ 3) on all chromosomes of the B subgenome of *B. carinata* but not on C02, C05, C06 and C08 (Table 3, Table S2). There were 12 QTL that were localised on chromosomes B01, B02, B03, B04, B06, B07, B08, C01 C03 and C07 across at least two environments (Table 3). QTL accounted for 0.8–10.9% of genotypic variance (Table 3). Both parental lines contributed alleles for pod length, Yellowcross contributed favourable alleles for pod length on B01, B07, B08, and C01 whereas Whiteban contributed variation in pod length on B02, C01 and C03 (Table 3C).

**Table 3:** Quantitative trait loci associated with pod length (mm) across three environments (2013, 2014 and 2023). QTL were identified using the whole genome average interval mapping. Marker intervals that are in bold represent genomic regions that consistently detected and those are in italics and bold may represent the same genomic region (QTL). A detailed QTL summary is given in Table S9

Trait	Chromosome	Left marker	Left marker position (cM)	Right marker	Right marker position (cM)	Allelic effect size	Genotypic variance (%)	LOD
Pod Length 2023	B01	100011720	2.47	100029508	6.24	1.08	2.50	3.02
Pod Length 2013	B01	100074174	24.89	100020829	26.58	1.20	4.60	4.36
Pod Length 2014	B01	100001950	52.28	100062079	53.35	1.88	9.30	9.87
*Pod Length 2023*	*B01*	*100058331*	*69.61*	*100019767*	*70.03*	*1.87*	*7.60*	*8.01*
*Pod Length 2013*	*B01*	*100079097*	*74.92*	*100027560*	*77.76*	*1.44*	*6.50*	*5.59*
**Pod Length 2023**	**B02**	**100020984**	**23.67**	**100031592**	**24.23**	**-1.46**	**4.60**	**4.30**
**Pod Length 2013**	**B02**	**100047102**	**24.53**	**NA12H07**	**24.89**	**-1.60**	**8.00**	**7.24**
**Pod Length 2014**	**B02**	**NA12H07**	**24.89**	**100033803**	**25.4**	**-1.99**	**10.20**	**9.05**
*Pod Length 2023*	*B02*	*100014832*	*74.31*	*100030187*	*75.31*	*-2.23*	*10.80*	*11.36*
*Pod Length 2014*	*B02*	*100007738*	*75.83*	*100033564*	*76.6*	*-1.54*	*6.30*	*6.28*
Pod Length 2014	B02	100067615	136.49	100058792	137.84	1.12	3.40	3.77
**Pod Length 2013**	**B03**	**100008060**	**47.01**	**100047311**	**47.55**	**-1.20**	**4.70**	**4.72**
**Pod Length 2014**	**B03**	**100008060**	**47.01**	**100047311**	**47.55**	**-0.50**	**0.80**	**0.88**
**Pod Length 2013**	**B04**	**100045241**	**32.51**	**100006091**	**33.62**	**-1.00**	**3.30**	**3.12**
**Pod Length 2014**	**B04**	**100045241**	**32.51**	**100006091**	**33.62**	**-1.04**	**3.00**	**3.19**
Pod Length 2023	B05	100064697	3.48	100008226	4.12	-1.37	4.10	4.58
Pod Length 2013	B05	100004698	141.39	100071120	141.78	1.10	4.00	4.14
**Pod Length 2013**	**B06**	**100037101**	**92.45**	**BRAS116**	**92.88**	**-0.87**	**2.50**	**2.38**
**Pod Length 2014**	**B06**	**100037101**	**92.45**	**BRAS116**	**92.88**	**-0.99**	**2.60**	**2.94**
**Pod Length 2023**	**B06**	**100037101**	**92.45**	**BRAS116**	**92.88**	**-1.75**	**6.60**	**7.66**
**Pod Length 2013**	**B07**	**100053770**	**28.09**	**100019213**	**29.28**	**1.54**	**7.50**	**7.60**
**Pod Length 2023**	**B07**	**100053770**	**28.09**	**100019213**	**29.28**	**1.49**	**4.80**	**5.64**
Pod Length 2013	B07	100021591	84.94	100055759	85.17	-1.16	4.30	4.48
**Pod Length 2014**	**B08**	**100025362**	**1.49**	**100013294**	**3.28**	**1.24**	**4.20**	**4.39**
**Pod Length 2023**	**B08**	**100025362**	**1.49**	**100013294**	**3.28**	**1.54**	**5.10**	**5.62**
**Pod Length 2013**	**B08**	**100013294**	**3.28**	**100071515**	**4.11**	**1.25**	**5.10**	**5.29**
Pod Length 2023	B08	100045154	95.3	100030257	95.98	-1.30	3.60	4.28
**Pod Length 2013**	**C01**	**100011545**	**16.46**	**CB10587**	**19.39**	**0.98**	**3.00**	**3.06**
**Pod Length 2014**	**C01**	**CB10587**	**19.39**	**100011069**	**26.28**	**1.42**	**5.00**	**5.47**
**Pod Length 2023**	**C01**	**CB10587**	**19.39**	**100011069**	**26.28**	**1.51**	**5.00**	**5.52**
Pod Length 2013	C01	100016232	99.2	100020118	100.57	-1.01	3.30	3.35
**Pod Length 2023**	**C01**	**100020118**	**100.57**	**100017613**	**103.13**	**-1.66**	**5.90**	**6.42**
**Pod Length 2014**	**C01**	**100048162**	**109.47**	**100010271**	**114.25**	**-1.60**	**6.60**	**7.08**
**Pod Length 2013**	**C03**	**100058609**	**11.57**	**100028512**	**22.99**	**-1.27**	**4.70**	**4.56**
**Pod Length 2014**	**C03**	**100058609**	**11.57**	**100028512**	**22.99**	**-2.17**	**10.90**	**11.62**
**Pod Length 2023**	**C03**	**100058609**	**11.57**	**100028512**	**22.99**	**-2.00**	**8.70**	**8.70**
Pod Length 2013	C04	100023632	2.88	100019506	3.09	0.95	3.00	2.83
Pod Length 2013	C04	100044151	71.03	100013195	71.52	1.04	3.60	3.73
Pod Length 2023	C04	ks20640-A	122.49	100027194	131.98	-1.16	2.90	2.91
**Pod Length 2023**	**C07**	**100019077**	**21.69**	**100062030**	**21.98**	**-1.18**	**3.00**	**3.72**
**Pod Length 2013**	**C07**	**100062030**	**21.98**	**100065291**	**22.83**	**-1.07**	**3.80**	**3.88**
Pod Length 2014	C07	BnGMS357	67.13	100071984	68.63	-1.05	3.00	3.06
Pod Length 2023	C09	100052899	34.44	3078850S	36.62	-1.11	2.70	3.00

WINQTL analysis identified 19 QTL for pod length, of which five were detected across environments; these were mapped to the same genomic region on chromosomes B01, B02, B08 and C03. Five marker intervals on B01, B07, C03 and C07 were repeatedly detected across WGAIM and WinQTL (Table S9).

### Colocation of QTL for pod shatter resistance and pod length

We compared the genetic position of QTL associated with RE and pod length and identified seven significant QTL regions on chromosomes B02, B04, B06, B07, C01, C04, and C07 which showed association with both traits investigated suggesting that pod length is somewhat associated with RE (Table 2, Table S10a). Opposite allelic effects for pod length and RE were detected for all QTL on B02, B04, B06, C01, C04, and C07, suggesting that pod length and RE are controlled by alternative/opposite alleles. Whiteban alleles on B07 contributed favourable alleles for pod shatter resistance and pod length.

### Prioritised candidate genes underlying QTLs for pod shatter resistance

We searched for the physical locations of significant markers flanking QTL for RE (Tables 2 and 3) using the recently published *B. carinata* pan-genome (Supplementary Tables). Annotated genes in the reference assemblies located within QTL intervals in reference assemblies were prioritised as candidates for pod shatter resistance.

**Fig. 2:**
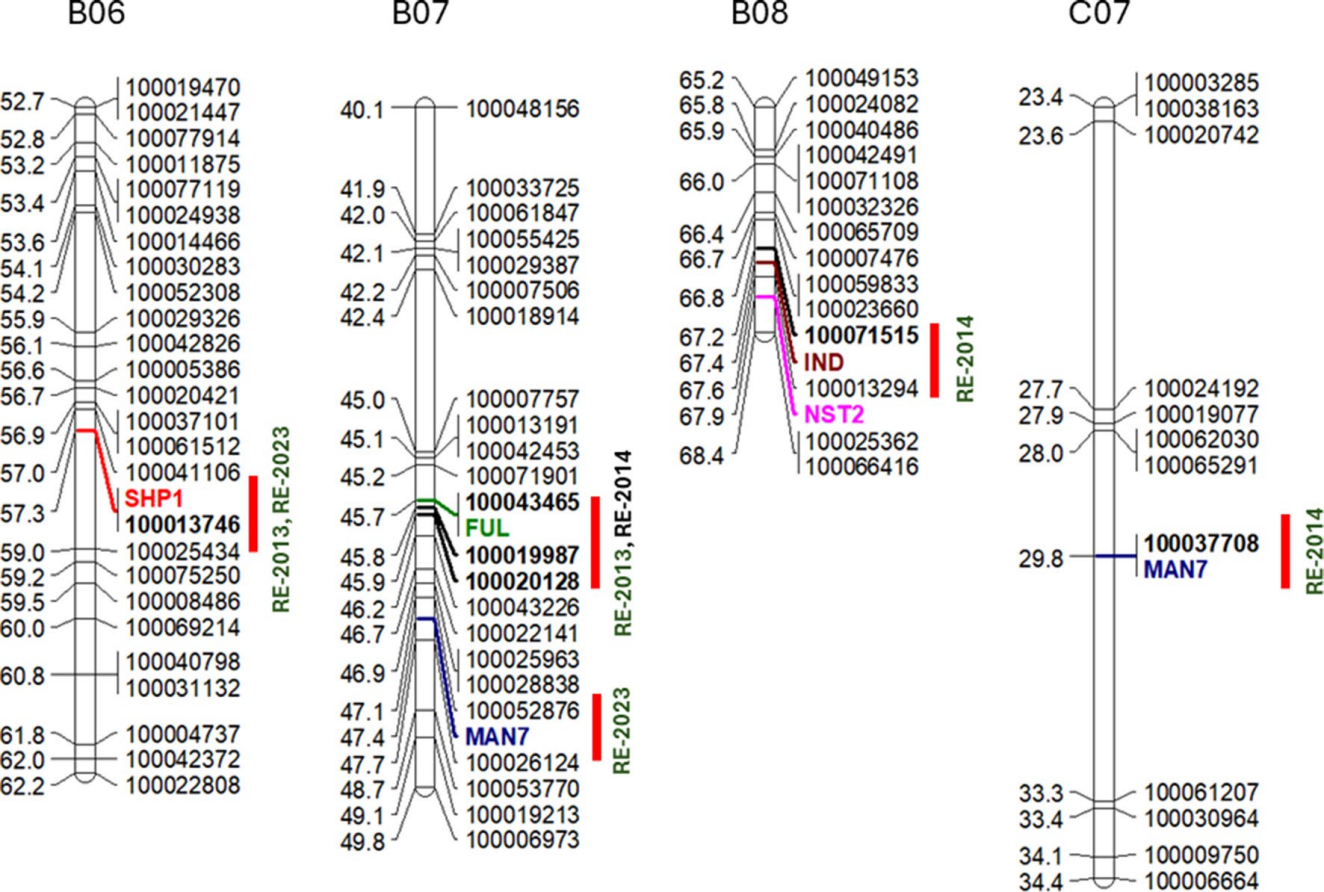
Physical locations of the* priori* candidate genes that map in the vicinity of DArTseq markers (within 200 Kb) associated with pod shatter resistance in the doubled haploid population derived from Yellowcross/Whiteban. The physical locations are based on the pan-genome of *B. carinata* and are in Mb. Chromosomal positions (B06, B07, B08 and C07) are given on the top of the linkage groups, and candidate genes are highlighted in colours. Details of markers, QTL and their genetic position are given in Table 2

Comparative mapping identified at least six candidate genes for pod shatter resistance in the YWDH population and included *SHP1 on B06*, *FUL* and *MAN* on chromosomes B07, *IND* and *NST2* on B08, *NST1* on C04 and *MAN7* on C07 that mapped within 0.5 Mb from the QTL for pod shatter resistance. *FUL* gene was located within 28.1 kb from the highly significant marker interval 100043465 (100007757/100006973 100046735 − 100022658, 45,711,486 bp) which accounted for the 27.6% genetic variation for pod shatter resistance on B07 (Fig. 2, Table S10).

The sequence of marker 5863583 showed significant association in the intercross population derived from BC73526 and BC73524 was localised to the 46,394,072 − 46,394,140 bp of the reference sequence; this interval encompasses also the *FUL* homologue previously mapped on B01 [8] and recently on B07 [12]. However, the WGS SNP marker interval was 43.3 to 46.1 Mb away from the marker interval that was associated with pod shatter resistance and *FUL* gene, which was identified with DArTseq markers on B07 in the larger set of DH lines (Table S10).

## Discussion

Although *B. carinata* is an old crop, its use in biodiesel and aviation fuel is relatively new. Considering its narrow genetic base [1], there is a need for targeted breeding of *B. carinata* varieties especially to improve key traits such as plant height, flowering time, root system, pod shatter resistance, pod length, seed yield and erucic acid content suitable for biodiesel and jet fuel markets. Understanding the genetic control of pod shatter resistance and pod length is a key approach for breeding pod shatter-resistant and high-yielding *B. carinata* cultivars for emerging markets. Longer pods could accommodate more and/or bolder seeds due to the increased pod area for photosynthesis. Previous research has localised several QTL associated with pod shatter resistance in *B. rapa* [29, 30], *B. juncea* [31] and *B. napus* [16, 27, 32–36]. However, studies describing the genetic architecture of pod shatter resistance in *B*. *carinata* are limited [8]. Our results have shown that a considerable genetic variation exists among the parental lines and DH lines from the YW cross that could be harnessed in breeding programs.

In this study, we employed WGAIM, WinQTL, IciMapping and haplotype trend regression methods to detect robust QTL for pod shatter resistance in *B. carinata*. In contrast to other approaches followed in this study, WGAIM enabled the simultaneous modelling of genetic and non-genetic variation through extensions of the linear mixed model that allows complex extraneous variation to be captured as well as simultaneously incorporating a whole genome analysis to detect and select QTL while controlling the number of false positive QTL [20]. In this study, WGAIM detected 16 QTL for pod shatter resistance on B02, B03, B04, B06, B07, B08, C01, C03, C04, C06 and C07 across three environments and accounted for a higher proportion of genotypic variance (Table 2). However, several QTL do not precisely overlap across phenotypic environments, suggesting the role of the growing environment in modulating phenotypic trait (pod shatter resistance) expression. Significant variations due to environment and genotype interactions were reported in *B. rapa* and *B. juncea* [31]. Nonetheless, allelic effects (from the Whiteban parent) for pod shatter resistance were consistent especially for four QTL, suggesting that the Whiteban parent possesses higher pod shatter resistance compared to the maternal parent, Yellowcross. This observation is consistent with higher pod rupture energy values (20–22 mJ) of Whiteban across three environments. Across statistical packages, we repeatedly detected six QTL for pod shatter resistance on B02, B03, B06, B07, B08, and C06 chromosomes (Table S4). These results suggest that these QTLs are reliable for research and development activities such as introducing appropriate favourable alleles into pod shatter prone varieties of *B carinata* and related species and might play a significant role in improving resistance in shatter-prone germplasm.

Previous studies also reported QTL associated with pod shatter resistance in the *B. carinata* population. For example, Raman et al., [8]) reported five QTL for rupture energy on chromosomes B1, B3, B8 and C5. These chromosomal locations were based on the reference genomes of *B. nigra* and *B. oleracea*. Recently, the pan-genome of *B. carinata* was published, and QTL mapped by Raman et al., [8] were re-anchored onto the pan-genome of *B. carinata* on chromosomes that correspond to chromosome B07(B01), B03(B08), B08 (B02) and C05(C05) respectively [12]. The physical position of markers flanking QTL is shown (Table S10). The study by Niu et al. [12] also mapped QTL for pod shatter resistance using whole genome resequencing-based bulked segregant analysis of selected lines from the same population [8] on chromosomes B02, B07 and B08. The B7 QTL was mapped to the 46394072 − 46394140 bp region on the reference pan-genome. In the YW DH population, the B07 QTL region was mapped to the 45711486–45805435 bp; likely, populations derived from Yellowcross/Whiteban (this study) and BC73526/BC73524 [8] may have the same gene controlling-pod shatter resistance. Further work needs to be done to test this hypothesis using allelism tests. In a GWAS panel of *B. juncea (AABB)*, Kaur et al., [31] identified QTL for rupture energy on chromosome B5 and identified *RAP2.4* and *LEUNIG* candidate genes that alter AP2 expression in Arabidopsis [37]. Out of the repeatedly detected QTL across environments/QTL mapping approaches, no QTL were detected on chromosome B05 in the YWDH population. It is possible that the usage of parents of the mapping populations may not have captured the same level of allelic variation compared to the GWAS panel of *B. juncea*. In the YW population, we detected a significant QTL for pod shatter resistance on chromosome C01 across 2013 and 2014 environments, this novel QTL has not been detected in earlier studies. Therefore, this novel locus could be exploited in the C genome species (*B. carinata*,* B. napus* and *B. oleracea*) in the breeding programs. However, stable introgression of B genome QTL into the A^n^C^n^ genome may be difficult, although not impossible due to genomic instability.

Using the selective genotyping of the 83 DH lines which had the higher recombination rate [19] and higher marker density, we located only 7 QTL; of them, one major QTL was located approximately 43 Mb apart from the markers and the candidate gene identified in the moderately larger population, with moderate marker density. Previous studies have shown that marker density, recombination rate and effective population size play an important role in resolving the precise location of QTL [38]. Physical mapping of marker-interval underlying B7 QTL indicated that gene-controlling pod shatter resistance, *FRUITFUL* gene is localised approximately 28.1 kb on the pan-genome of *B. carinata*. This is much closer than the QTL interval detected using 16,632 WGS SNP markers, hinting that a linkage map with more recombination events due to a larger population is more useful in identifying QTL and candidate genes for trait variation, due to the increased power of detecting recombinant events.

Some *priori* genes for pod shatter resistance such as *SHP1*, *FUL*, *MAN7*, *NST1*, *NST2* and *IND* were localised within 0.5 Mb from significant QTL regions. Small populations with low-density markers cannot resolve recombination between markers and candidate genes [39]. However, the homologs of pod-shatter resistance genes that map further apart (more than 0.5 Mb) from significantly associated markers on other chromosomes could also regulate genetic variation in pod-shatter resistance. Further research is required to substantiate this hypothesis. In Arabidopsis and related Brassica species, several genes involved in pod dehiscence such as four master valve margin identity genes: two MADS-box transcription factors *SHATTERPROOF 1*/*2* (*SHP1*/*SHP2*) [40, 41] and two bHLH family transcription factors *INDEHISCENT* and *ALCATRAZ* [42] have been reported. *FRUITFUL* (*FUL*), a MADS-box gene in the valves [40] and *REPLUMLESS* (*RPL*) homeodomain gene in the replum [43] negatively restrict the pod dehiscence. *FUL* has been prioritised as a candidate gene for pod shatter resistance in *B. napus*/*B.rapa* and *B. carinata* populations. In addition, *NST1* and *NST2* have also been implicated in modulating variation in pod shatter resistance in *B. juncea* and *B. napus* [31, 44].

## Conclusion

We identified several genomic regions associated with pod rupture energy and pod length in the YWDH population. Three QTL regions on B06, B07 and C07 were mapped near (within 100 Kb) the *priori* genes for pod dehiscence in Arabidopsis. Our research provides a valuable genetic resource for improving pod shatter resistance in *B. carinata* and related species such as *B. juncea* and *B. napus* - the major oilseed crops and for future studies on understanding molecular mechanisms underlying pod shatter resistance. The markers flanking stable QTL regions, which account for a higher proportion of genotypic variance, could accelerate Brassica breeding programs using marker-assisted selection, backcross, and genomic selection pipelines.

## Electronic supplementary material

Below is the link to the electronic supplementary material.

Supplementary Material 1

## Data Availability

All the data generated or analysed in this study are available in the manuscript and the supplementary materials.

## Abbreviations

ALC: ALCATRAZ
cM: CentiMorgan
FUL: FRUITFUL
IND: INDEHISCENT
Kb: Killobase pair
LOD: Logarithm of the likelihood ratio
MAN7: MANNANASE 7
Mb: Megabase pair
NST: NAC SECONDARY WALL THICKENING PROMOTING FACTOR
RPL: REPLUMLESS
QTL: Quantitative trait loci
RE: rupture energy
SHP1: SHATTERPROOF 1
SHP2: SHATTERPROOF 2
WGAIM: Whole genome average interval mapping
WGR: Whole genome resequencing

## References

[1] Khedikar Y, Clarke WE, Chen L, Higgins EE, Kagale S, Koh CS, Bennett R, Parkin IAP. Narrow genetic base shapes population structure and linkage disequilibrium in an industrial oilseed crop, *Brassica carinata* A. Braun. Sci Rep. 2020;10(1):12629. 32724070 10.1038/s41598-020-69255-w PMC7387349

[2] Nagaharu U. Genome analysis in Brassica with special reference to the experimental formation of B. napus and peculiar mode of fertilization. Jpn JBot. 1935;7.

[3] Gómez-Campo C, Prakash S. Origin and domestication. In: C.Gómez-Campo, editor. Biology of Brassica Coenospecies. Netherlands: Elsevier; 1999. pp. 33–58.

[4] Alemayehu N, Becker H. Genotypic diversity and patterns of variation in a germplasm material of Ethiopian mustard (*Brassica carinata* A. Braun). Genet Resour Crop Evol. 2002;49(6):573–82.

[5] Seepaul R, Kumar S, Iboyi JE, Bashyal M, Stansly TL, Bennett R, Boote KJ, Mulvaney MJ, Small IM, George S, et al. *Brassica carinata*: Biology and agronomy as a biofuel crop. GCB Bioenergy. 2021;13(4):582–99.

[6] Jiang Y, Tian E, Li R, Chen L, Meng J. Genetic diversity of *Brassica carinata* with emphasis on the interspecific crossability with *B. rapa*. Plant Breeding. 2007;126(5):487–91.

[7] Guo S, Zou J, Li R, Long Y, Chen S, Meng J. A genetic linkage map of *Brassica carinata* constructed with a doubled haploid population. Theor Appl Genet. 2012;125. 10.1007/s00122-012-1898-3 22669300

[8] Raman R, Qiu Y, Coombes N, Song J, Kilian A, Raman H. Molecular diversity analysis and genetic mapping of pod shatter resistance loci in *Brassica carinata* L. Front Plant Sci. 2017;8:1765. 10.3389/fpls.2017.01765. 10.3389/fpls.2017.01765 PMC5716317 29250080

[9] Liu XY, Macmillan RH, Burrow RP, Kadkol GP, Halloran GM. Pendulum test for evaluation of rupture strength of seed pods. J Texture Stud. 1994;25:179–89.

[10] Zou J, Raman H, Guo S, Hu D, Wei Z, Luo Z, Long Y, Shi W, Fu Z, Du D, et al. Constructing a dense genetic linkage map and mapping QTL for the traits of flower development in Brassica carinata. Theor Appl Genet. 2014;1–13. 10.1007/s00122-014-2321-z. 10.1007/s00122-014-2321-z 24824567

[11] Zhang W, Hu D, Raman R, Guo S, Wei Z, Shen X, Meng J, Raman H, Zou J. Investigation of the genetic diversity and quantitative trait loci accounting for important agronomic and seed quality traits in *Brassica carinata*. 2017;8(615). 10.3389/fpls201701765. 10.3389/fpls.2017.00615 PMC5401912 28484482

[12] Niu Y, Liu Q, He Z, Raman R, Wang H, Long X, Qin H, Raman H, Parkin IAP, Bancroft I et al. A *Brassica carinata* pan-genome platform for *Brassica* crop improvement. Plant Commun. 2024;5(1). 10.1016/j.xplc.2023.100725 PMC10811369 37803826

[13] Banga S, Kaur G, Grewal N, Salisbury PA, Banga SS. Transfer of resistance to seed shattering from *Brassica carinata* to *B. napus*. In: *13th International Rapeseed Congress: 5–9 June, 2011 2011; Prague, Czhech Republic*. 863–865.

[14] Song X, Wei Y, Xiao D, Gong K, Sun P, Ren Y, Yuan J, Wu T, Yang Q, Li X, et al. *Brassica carinata* genome characterization clarifies U’s triangle model of evolution and polyploidy in Brassica. Plant Physiol. 2021;186(1):388–406. 33599732 10.1093/plphys/kiab048 PMC8154070

[15] Yim WC, Swain ML, Ma D, An H, Bird KA, Curdie DD, Wang S, Ham HD, Luzuriaga-Neira A, Kirkwood JS, et al. The final piece of the triangle of U: evolution of the tetraploid *Brassica carinata* genome. Plant Cell. 2022;34(11):4143–72. 35961044 10.1093/plcell/koac249 PMC9614464

[16] Raman H, Raman R, Kilian A, Detering F, Carling J, Coombes N, Diffey S, Kadkol G, Edwards D, McCully M et al. Genome-wide delineation of natural variation for pod shatter resistance in *Brassica napus*. PLoS ONE. 2014;9(7):e101673. 10.1371/journal.pone.0101673. 10.1371/journal.pone.0101673 PMC4090071 25006804

[17] Kadkol GP, Macmillan RH, Burrow RP, Halloran GM. Evaluation of Brassica genotypes for resistance to shatter. I. Development of a laboratory test. Euphytica. 1984;33(1):63–73.

[18] Zhang Y, Thomas CL, Xiang J, Long Y, Wang X, Zou J et al. QTL meta-analysis of root traits in *Brassica napus* under contrasting phosphorus supply in two growth systems. Sci Rep. 2016;6:33113. 10.1038/srep33113. 10.1038/srep33113 PMC5021999 27624881

[19] He Z, Ji R, Havlickova L, Wang L, Li Y, Lee HT, Song J, Koh C, Yang J, Zhang M, et al. Genome structural evolution in Brassica crops. Nat Plants. 2021;7(6):757–65. 34045706 10.1038/s41477-021-00928-8

[20] Taylor J. A Verbyla. R package Wgaim: QTL analysis in bi-parental populations using linear mixed models. J Stat Softw. 2011;40(7):1–18 10.18637/jss.v040.i07.

[21] Raman H, Raman R, Mathews K, Diffey S, Salisbury P. QTL mapping reveals genomic regions for yield based on an incremental tolerance index to drought stress and related agronomic traits in canola. Crop Pasture Sci. 2020;71(6):562–577. 10.1071/CP20046

[22] Meng L, Li H, Zhang L, Wang J. QTL IciMapping: Integrated software for genetic linkage map construction and quantitative trait locus mapping in biparental populations. Crop J. 2015;3(3):269–83.

[23] Wang S, Basten CJ, Zeng ZB. Windows QTL cartographer 2.5. Department of Statistics, North Carolina State University, Raleigh, NC; 2010. http://statgen.ncsu.edu/qtlcart/WinQTLCart.pdf.

[24] Raman R, Diffey S, Carling J, Cowley R, Kilian A, Luckett D, Raman H. Quantitative genetic analysis of yield in an Australian *Brassica napus* doubled haploid population. Crop Pasture Sci. 2016;67(4):298–307.

[25] Gilmour AR, Cullis BR, Verbyla AP. Accounting for natural and extraneous variation in the analysis of field experiments. J Agricultural Biol Environ Stat. 1997;2(3):269–93.

[26] Altschul S, Gish W, Miller W, Myers E, Lipman D. Basic local alignment search tool. J Mole Biol. 1990;215:403–10. 10.1016/S0022-2836(05)80360-2 2231712

[27] Raman H, Raman R, Sharma N, Cui X, McVittie B, Qiu Y, Zhang Y, Hu Q, Liu S, Gororo N. Novel quantitative trait loci from an interspecific *Brassica rapa* derivative improve pod shatter resistance in *Brassica napus*. Front Plant Sci. 2023;14:1233996. 37736615 10.3389/fpls.2023.1233996 PMC10510201

[28] Summers JEB, Vancanneyt DM, Redig G, Werner P, Morgan CP, Child C. Pod shatter resistance in the resynthesized *Brassica napus* line DK142. J Agric Sci. 2003;140:43–52.

[29] Kadkol G, Halloran G, MacMillan R. Inheritance of siliqua strength in *Brassica campestris* L. I. studies of F_2_ and backcross populations. Can J Genetical Cytol. 1986;28:365–73.

[30] Mongkolporn O, Kadkol GP, Pang CK, Taylor PWJ. Identification of RAPD markers linked to recessive genes conferring siliqua shatter resistance in *Brassica rapa*. Plant Breeding. 2003;122:479–84.

[31] Kaur J, Akhatar J, Goyal A, Kaur N, Kaur S, Mittal M, Kumar N, Sharma H, Banga S, Banga SS. Genome wide association mapping and candidate gene analysis for pod shatter resistance in *Brassica juncea* and its progenitor species. Mol Biol Rep. 2020;47(4):2963–74. 32219770 10.1007/s11033-020-05384-9

[32] Dhaliwal I, Banga S, Kumar N, Salisbury P, Banga S. A candidate gene-based association study of introgressed pod shatter resistance in Brassica napus. Indian J Tradit Knowl. 2021;20:267–76. 10.56042/ijtkv20i130783.

[33] Hu Z, Hua W, Huang S, Yang H, Zhan G, Wang X, Liu G, Wang H. Discovery of pod shatter-resistant associated SNPs by deep sequencing of a representative library followed by bulk segregant analysis in rapeseed. PLoS ONE. 2012;7(4):e34253. 22529909 10.1371/journal.pone.0034253 PMC3328475

[34] Wen YC, Zhang SF, Yi B, Wen J, Wang JP, Zhu JC, He JP, Cao JH. Identification of QTLs involved in pod-shatter resistance in *Brassica napus* L. Crop Pasture Sci. 2013;63(12):1082–9.

[35] Liu J, Wang J, Wang H, Wang W, Zhou R, Mei D, Cheng H, Yang J, Raman H, Hu Q. Multigenic control of pod shattering resistance in chinese rapeseed germplasm revealed by genome-wide association and linkage analyses. Frontiers Plant Sci. 2016;7(1058). https://doi.org/103389/fpls201601058 10.3389/fpls.2016.01058 PMC4954820 27493651

[36] Liu J, Rijin Z, Wang W, Wang H, Qiu Y, Rosy R, Mei D. Raman Harsh, Qiong Hu: A copia-like retrotransposon insertion in the upstream region of the SHATTERPROOF1 gene, BnSHP1.A9, is associated with quantitative variation in pod shattering resistance in oilseed rape. J Exp Bot. 2020;19;71(18):5402–5413. 10.1093/jxb/eraa281. PMC7501816 32525990

[37] Liu Z, Meyerowitz EM. LEUNIG regulates AGAMOUS expression in Arabidopsis flowers. Development. 1995;121(4):975–91. 7743940 10.1242/dev.121.4.975

[38] Xu X, Zeng L, Tao Y, Vuong T, Wan J, Boerma R, Noe JLZ, Finnerty S, Pathan SM, Shannon JG, et al. Pinpointing genes underlying the quantitative trait loci for root-knot nematode resistance in palaeopolyploid soybean by whole genome resequencing. Proc Natl Acad Sci U S A. 2013;33:13469–74. 10.1073/pnas.1222368110 PMC3746920 23898176

[39] Raman H, Raman R, Coombes N, Song J, Prangnell R, Bandaranayake C, Tahira R, Sundaramoorthi V, Killian A, Meng J, et al. Genome-wide association analyses reveal complex genetic architecture underlying natural variation for flowering time in canola. Plant Cell Environ. 2016;39(6):1228–39. 26428711 10.1111/pce.12644

[40] Ferrandiz C, Liljegren SJ, Yanofsky MF. Negative regulation of the *SHATTERPROOF* genes by *FRUITFULL* during Arabidopsis fruit development. Science. 2000;289(5478):436–8. 10903201 10.1126/science.289.5478.436

[41] Liiljegren SJ, Ditta GS, Eshed Y, Savidge B, Bowman JL, Yanofsky MF. *SHATTERPROOF* MADS-box genes control seed dispersal in *Arabidopsis*. Nature. 2000;404:766–70. 10783890 10.1038/35008089

[42] Rajani S, Sundaresan V. The Arabidopsis myc/bHLH gene *ALCATRAZ* enables cell separation in fruit dehiscence. Curr Biol. 2001;11(24):1914–22. 11747817 10.1016/s0960-9822(01)00593-0

[43] Roeder AHK, Ferrándiz C, Yanofsky MF. The role of REPLUMLESS Homeodomain protein in patterning the Arabidopsis Fruit. Curr Biol. 2003;13(18):1630–5. 13678595 10.1016/j.cub.2003.08.027

[44] Zhou R, Cheng H, Wang W, Chu W, Wang H, Hao M, Mei D, Fu L, Li C et al. Wenkai Yu : *BnNST2.A9* targeting *BnMYB46* modulates pod-shattering resistance in *Brassica napus* L. *Submitted* 2024.

