# Supplementary material for: Identification of quantitative trait loci and candidate genes for pod shatter resistance in Brassica carinata
Source: BMC Plant Biol. 2024 Sep 30;24:892. doi: 10.1186/s12870-024-05596-2 (PMC11441008; doi:10.1186/s12870-024-05596-2)
Supplement: Supplementary file 1 — Supplementary Material 1 [file 12870_2024_5596_MOESM1_ESM.pdf]

Figure S1. Genetic analysis of the Yellowcross (shatter prone)/Whiteban (shatter resistant) DH population for pod shatter resistance. Frequency distribution of rupture energy scores in a segregation DH population in 2013 (A), 2014 (B) and 2023 (C). The predicted mean score of rupture energy for check and parental lines of Yellowcross/Whiteban are shown (2013, D; 2014, E). Frequency distribution of pod length scores in a segregation DH population in 2013 (F), 2014 (G) and 2023 (H). Solid arrows indicate the average RE/pod length scores of the maternal parent Yellowcross and the paternal Whiteban.

A.

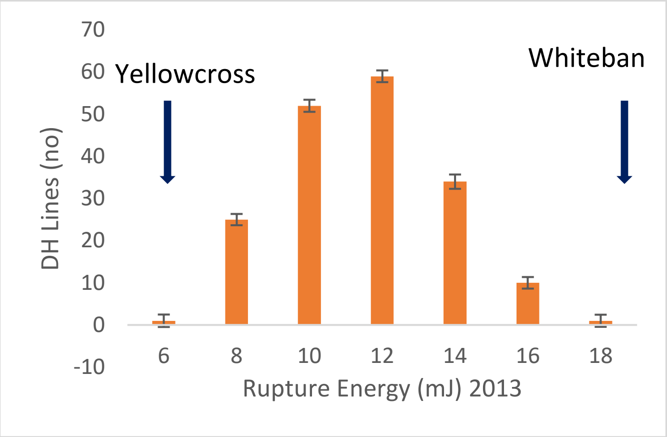

B.

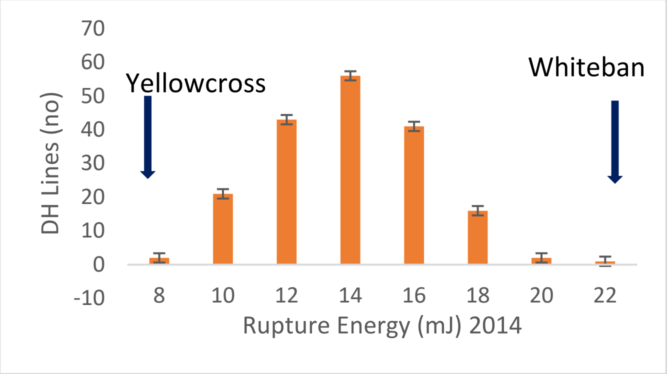

C.

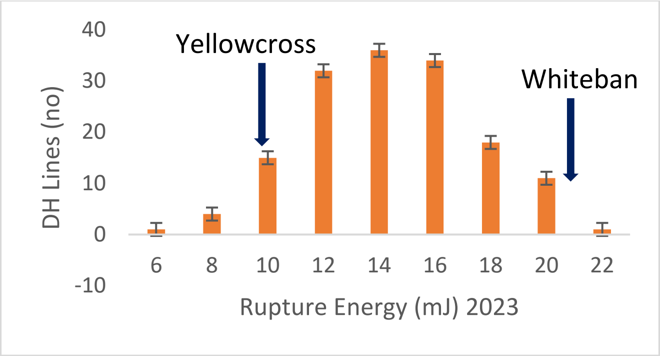

DH Lines (no)
40
30
20
10
0
-10
Yellowcross
Whiteban
6
8
10
12
14
16
18
20
22
Rupture Energy (mJ) 2023

D.

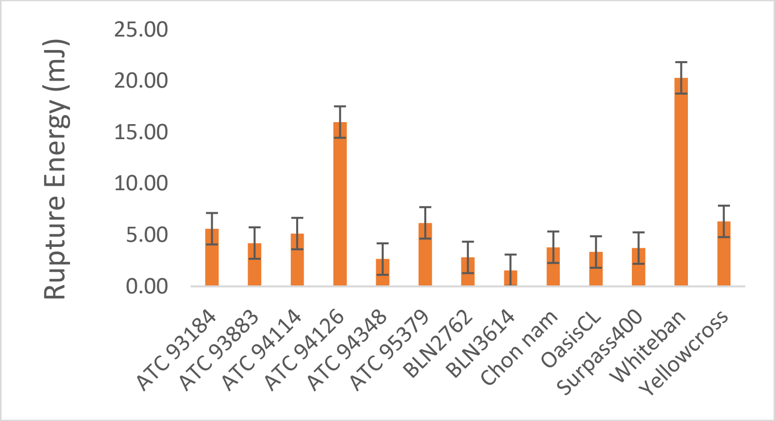

Rupture Energy (mJ)
25.00
20.00
15.00
10.00
5.00
0.00
ATC 93184
ATC 93883
ATC 94114
ATC 94126
ATC 94348
ATC 95379
BLN2762
BLN3614
Chon nam
OasisCL
Surpass400
Whiteban
Yellowcross

E.

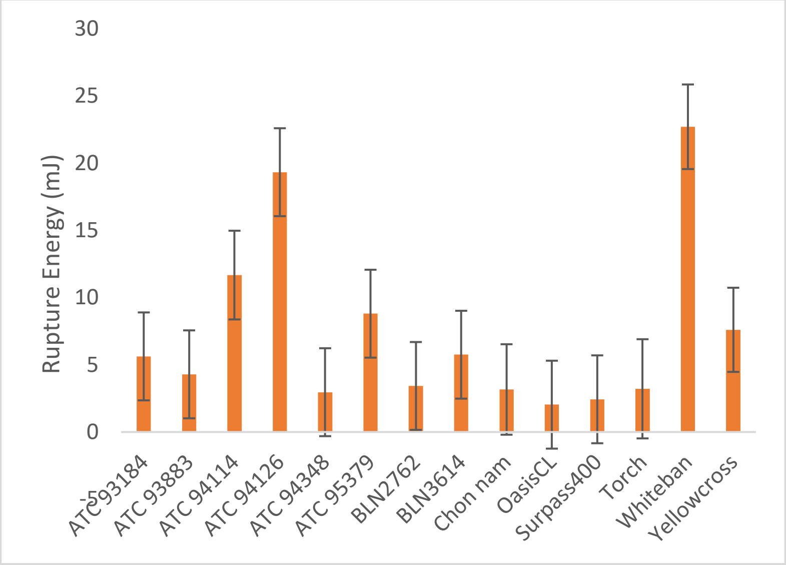

Rupture Energy (mJ)
30
25
20
15
10
5
0
ATC 93184
ATC 93883
ATC 94114
ATC 94126
ATC 94348
ATC 95379
BLN2762
BLN3614
Chon nam
OasisCL
Surpass400
Torch
Whiteban
Yellowcross

F.

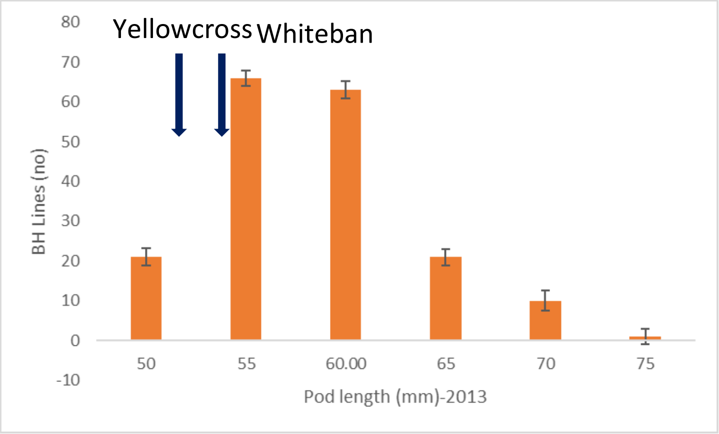
Yellowcross
Whiteban
BH Lines (no)
80
70
60
50
40
30
20
10
0
-10
50
55
60.00
65
70
75
Pod length (mm)-2013

F.

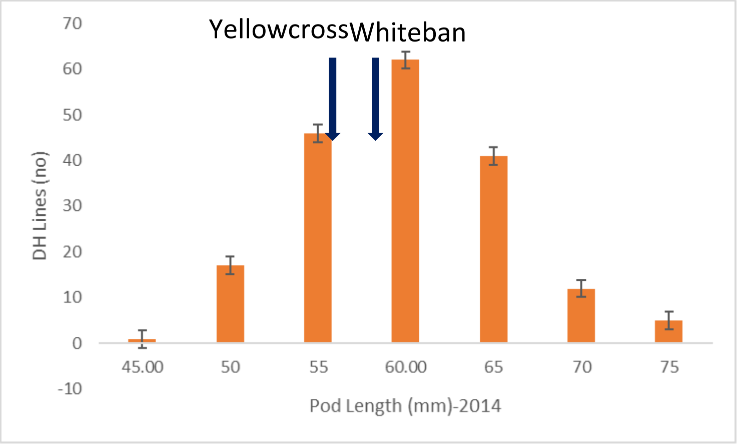
Yellowcross
Whiteban
DH Lines (no)
70
60
50
40
30
20
10
0
-10
45.00
50
55
60.00
65
70
75
Pod Length (mm)-2014

G.

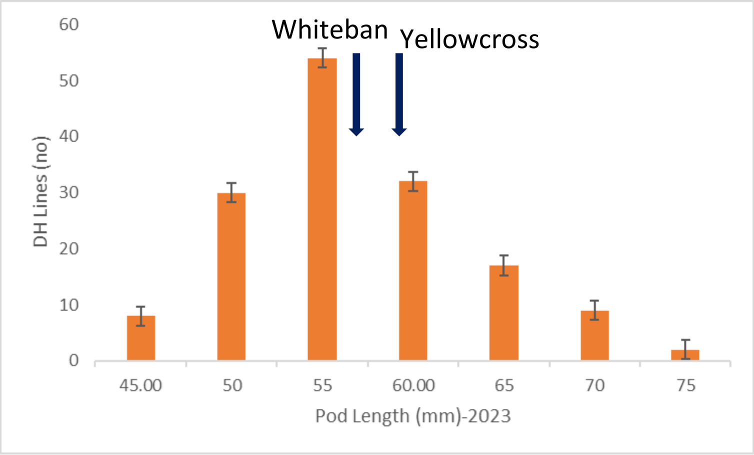
Whiteban
Yellowcross
DH Lines (no)
60
50
40
30
20
10
0
45.00
50
55
60.00
65
70
75
Pod Length (mm)-2023

Figure S2. Relationship between genetic and physical mapp positions of markers on the pan-genome of *Brassica carinata.*

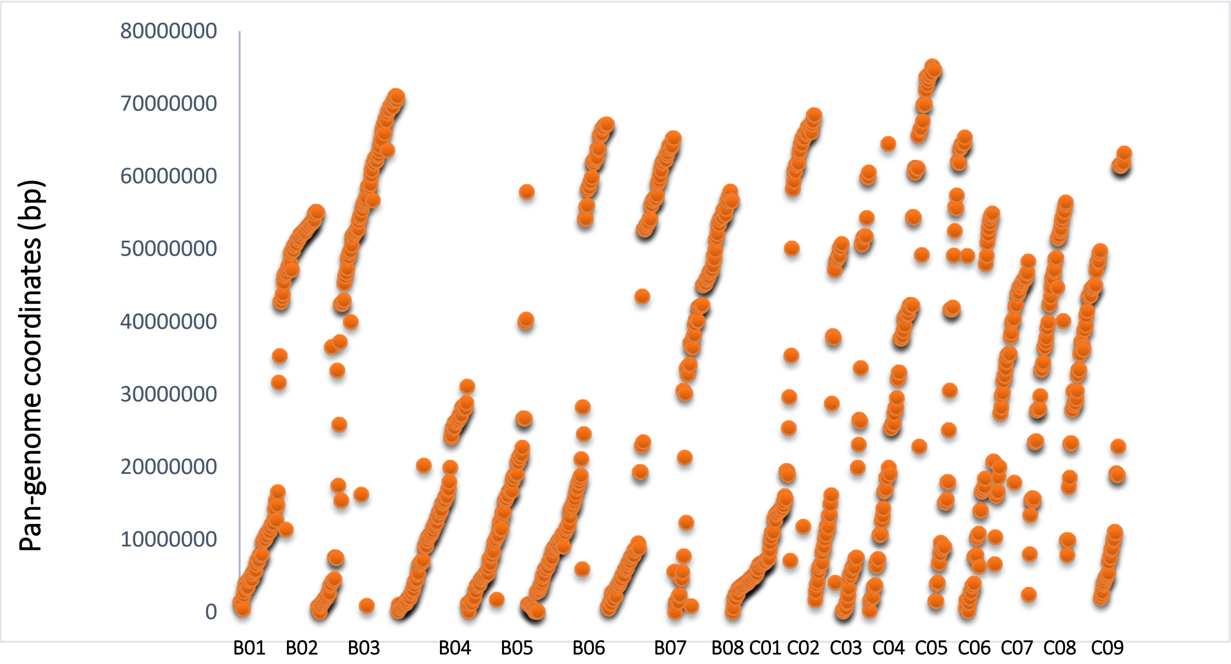

Figure S3 Whole Genome Average Interval Mapping-based detection of QTL for pod shatter resistance, measured as rupture energy (mj) by the pendulum test in the YWDH population. Manhattan plots showing genomic regions associated with resistance to pod shatter: highly significant regions are labelled (A: 2013, B: 2014).

A B

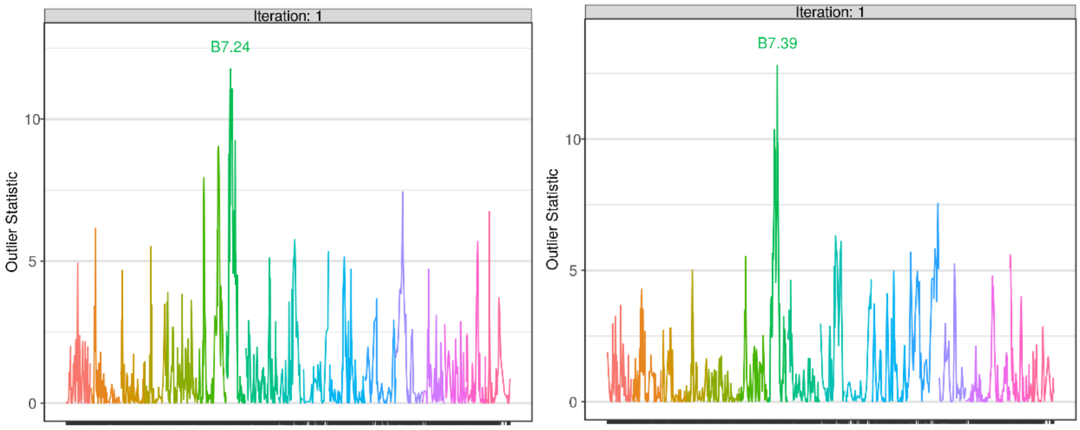

Table S1a. Predicted mean values for rupture energy (mJ) in the YWDH population and i
environments in 2013, 2014 and 2023 seasons. Rupture energy was measured by the pendu
predictions

2013			
Accession	EBLUP	EBLUP withTrial Mean	EBLUP Standard Error
YW001	-3.16	7.36	1.48
YW002	0.49	11.01	1.34
YW003	-0.51	10.01	1.43
YW005	-2.09	8.43	1.39
YW006	-2.11	8.41	1.70
YW007	0.54	11.06	1.37
YW008	-0.93	9.59	1.44
YW009	3.08	13.60	1.70
YW010	-2.45	8.07	1.39
YW011	3.11	13.63	1.35
YW012	2.86	13.39	1.38
YW013	1.36	11.88	1.39
YW014	2.17	12.69	1.36
YW015	3.14	13.66	1.47
YW016	-2.13	8.39	1.57
YW017	2.89	13.41	1.47
YW018	-0.11	10.41	1.34
YW019	3.79	14.31	1.62
YW020	-1.40	9.12	1.37
YW022	1.03	11.55	1.42
YW023	-0.92	9.60	1.42
YW024	-0.78	9.74	1.44
YW025	3.81	14.33	1.38
YW026	-1.40	9.13	1.69
YW027	1.47	11.99	1.54
YW028	-0.94	9.58	1.38
YW029	-0.24	10.28	1.65
YW030	-0.78	9.74	1.42
YW031	-3.46	7.06	1.47
YW032	0.13	10.65	1.39
YW033	2.30	12.83	1.44
YW034	-3.54	6.98	1.39
YW035	-0.01	10.51	1.37
YW036	1.36	11.88	1.63
YW037	4.34	14.86	1.43
YW038	4.39	14.91	1.40
YW039	3.36	13.88	1.39
YW040	-0.09	10.43	1.47
YW041	-2.65	7.87	1.70

2014			
Accession	EBLUP	EBLUP with Trial Mean	EBLUP Standard Error
YW001	-2.40	10.51	1.38
YW002	2.24	15.15	1.40
YW003	0.96	13.87	1.39
YW004	-1.47	11.44	1.35
YW005	-4.15	8.76	1.38
YW006	0.46	13.37	1.38
YW007	-0.70	12.21	1.38
YW008	0.26	13.17	1.38
YW009	1.46	14.37	1.41
YW010	0.21	13.12	1.42
YW011	-1.59	11.33	1.37
YW012	2.91	15.82	1.39
YW013	1.19	14.10	1.41
YW014	0.14	13.05	1.38
YW015	0.85	13.76	1.40
YW016	-4.15	8.76	1.38
YW017	3.38	16.29	1.40
YW018	1.47	14.38	1.38
YW019	1.61	14.52	1.40
YW020	-0.43	12.48	1.38
YW021	0.77	13.68	1.39
YW022	3.61	16.53	1.39
YW023	2.35	15.26	1.40
YW024	-2.78	10.13	1.38
YW025	2.38	15.29	1.44
YW026	1.74	14.65	1.40
YW027	1.78	14.69	1.39
YW028	-0.74	12.17	1.39
YW029	1.61	14.52	1.38
YW030	-0.82	12.10	1.38
YW031	-0.91	12.00	1.38
YW032	-0.10	12.81	1.38
YW033	3.18	16.10	1.38
YW034	-3.33	9.58	1.38
YW035	-0.22	12.69	1.38
YW036	2.42	15.33	1.38
YW037	-1.46	11.45	1.38
YW038	-0.01	12.90	1.39
YW039	3.03	15.94	1.38

YW042	0.00	10.52	1.41
YW043	2.24	12.76	1.34
YW044	3.67	14.19	1.39
YW045	2.05	12.58	1.39
YW046	3.63	14.16	1.64
YW047	2.57	13.09	1.38
YW048	-3.03	7.49	1.53
YW049	-0.64	9.88	1.65
YW050	-1.00	9.52	1.35
YW051	1.99	12.51	1.70
YW052	2.47	12.99	1.62
YW053	0.60	11.12	1.39
YW054	-4.50	6.02	1.34
YW055	-1.84	8.68	1.39
YW056	2.95	13.47	1.35
YW057	0.04	10.56	1.36
YW058	0.18	10.70	1.40
YW059	-1.39	9.13	1.44
YW060	-0.36	10.16	1.39
YW061	0.44	10.96	1.70
YW062	-0.58	9.94	1.70
YW063	2.61	13.13	1.43
YW064	-2.98	7.54	1.38
YW065	-2.82	7.70	1.40
YW066	-0.94	9.58	1.39
YW067	-0.55	9.97	1.42
YW068	-0.24	10.28	1.34
YW069	0.40	10.92	1.35
YW070	0.87	11.39	1.34
YW071	2.92	13.44	1.36
YW072	3.13	13.65	1.40
YW073	2.53	13.05	1.39
YW074	-1.42	9.10	1.47
YW075	-2.52	8.00	1.38
YW076	-2.62	7.90	1.39
YW077	-1.59	8.93	1.40
YW078	1.56	12.08	1.38
YW079	1.00	11.52	1.35
YW080	-1.13	9.40	1.40
YW081	3.15	13.67	1.44
YW082	3.66	14.18	1.34
YW083	3.38	13.90	1.37
YW084	0.94	11.46	1.38
YW085	0.26	10.78	1.34
YW086	3.88	14.41	1.65
YW088	-2.70	7.82	1.35
YW089	-1.92	8.60	1.39

YW040	-0.42	12.49	1.38
YW041	-3.53	9.38	1.38
YW042	-1.31	11.60	1.40
YW043	5.65	18.56	1.38
YW044	3.76	16.67	1.40
YW045	0.18	13.09	1.38
YW046	2.49	15.40	1.38
YW047	2.68	15.60	1.38
YW048	-2.02	10.89	1.45
YW049	0.60	13.51	1.38
YW050	-0.70	12.21	1.38
YW051	-2.37	10.55	1.38
YW052	1.69	14.60	1.38
YW053	-0.49	12.42	1.38
YW054	-3.83	9.08	1.38
YW055	-5.55	7.36	1.38
YW056	3.97	16.88	1.38
YW057	1.67	14.58	1.39
YW058	0.39	13.30	1.46
YW059	-0.86	12.05	1.38
YW060	-2.10	10.81	1.38
YW061	3.15	16.06	1.40
YW062	-0.39	12.52	1.38
YW063	0.56	13.47	1.38
YW064	-2.94	9.97	1.38
YW065	-2.90	10.01	1.38
YW066	-1.35	11.56	1.39
YW067	0.15	13.06	1.38
YW068	-0.50	12.42	1.38
YW069	-3.01	9.90	1.39
YW070	0.48	13.39	1.40
YW071	5.00	17.91	1.39
YW072	2.17	15.08	1.39
YW073	4.60	17.51	1.38
YW074	0.01	12.92	1.38
YW075	-4.49	8.42	1.44
YW076	-3.19	9.72	1.38
YW077	-1.42	11.49	1.38
YW078	2.20	15.11	1.40
YW079	1.63	14.55	1.38
YW080	-0.65	12.26	1.40
YW081	0.22	13.13	1.38
YW082	4.07	16.98	1.38
YW083	2.61	15.52	1.38
YW084	3.41	16.32	1.40
YW085	3.60	16.51	1.39
YW086	5.24	18.15	1.38

YW090	-1.55	8.97	1.40
YW091	-2.67	7.85	1.44
YW092	-0.05	10.48	1.35
YW093	-2.48	8.04	1.33
YW094	2.83	13.35	1.34
YW095	-2.86	7.66	1.38
YW096	1.73	12.25	1.39
YW097	-0.17	10.35	1.37
YW098	1.02	11.54	1.44
YW099	-0.43	10.09	1.40
YW100	1.00	11.52	1.39
YW101	2.03	12.55	1.39
YW102	0.77	11.29	1.43
YW103	-1.17	9.35	1.55
YW104	-2.03	8.49	1.65
YW105	1.48	12.00	1.42
YW106	0.93	11.45	1.36
YW107	-1.13	9.39	1.37
YW108	-2.91	7.61	1.66
YW109	-0.07	10.45	1.38
YW110	-1.07	9.45	1.38
YW111	-3.85	6.68	1.40
YW112	-3.64	6.88	1.34
YW113	3.92	14.44	1.64
YW114	2.18	12.70	1.54
YW115	0.08	10.60	1.61
YW116	0.87	11.39	1.35
YW117	-1.26	9.26	1.39
YW118	0.81	11.33	1.42
YW119	-2.53	7.99	1.39
YW120	-0.42	10.10	1.37
YW121	-2.46	8.06	1.61
YW122	0.11	10.63	1.65
YW123	2.23	12.75	1.36
YW124	2.23	12.75	1.39
YW125	-1.21	9.31	1.53
YW126	-2.29	8.23	1.42
YW127	-1.46	9.07	1.38
YW128	2.50	13.02	1.70
YW129	1.37	11.89	1.33
YW130	-0.57	9.95	1.36
YW131	-2.78	7.74	1.44
YW132	-1.46	9.06	1.36
YW133	-0.99	9.54	1.35
YW134	-1.18	9.34	1.62
YW135	0.59	11.11	1.70
YW136	-0.05	10.47	1.34

YW088	-1.00	11.91	1.40
YW089	-0.75	12.16	1.38
YW090	1.25	14.16	1.46
YW091	-0.85	12.06	1.38
YW092	2.45	15.36	1.38
YW093	-1.53	11.38	1.40
YW094	-2.06	10.86	1.38
YW095	-0.52	12.39	1.38
YW096	-1.13	11.79	1.38
YW097	-1.69	11.22	1.38
YW098	2.81	15.72	1.41
YW099	0.31	13.22	1.38
YW100	0.93	13.84	1.38
YW101	2.17	15.08	1.38
YW102	-1.86	11.05	1.38
YW103	-1.56	11.35	1.40
YW104	-3.29	9.62	1.39
YW105	2.13	15.04	1.40
YW106	1.21	14.12	1.40
YW107	-0.16	12.75	1.38
YW108	-2.40	10.51	1.39
YW109	-0.28	12.64	1.38
YW110	0.34	13.25	1.38
YW111	-1.19	11.72	1.38
YW112	-5.20	7.71	1.38
YW113	1.81	14.72	1.40
YW114	1.30	14.21	1.68
YW115	1.80	14.71	1.39
YW116	2.62	15.53	1.40
YW117	-1.19	11.72	1.38
YW118	-0.74	12.17	1.40
YW119	-2.95	9.96	1.39
YW120	2.34	15.25	1.38
YW121	-0.90	12.01	1.38
YW122	2.85	15.76	1.40
YW123	0.10	13.01	1.40
YW124	0.21	13.12	1.38
YW125	0.03	12.94	1.38
YW126	-3.77	9.14	1.38
YW127	0.86	13.77	1.40
YW128	4.26	17.18	1.38
YW129	-0.28	12.63	1.38
YW130	-0.91	12.00	1.38
YW131	-3.45	9.47	1.38
YW132	3.20	16.11	1.38
YW133	-1.33	11.59	1.40
YW134	0.15	13.06	1.38

YW137	2.13	12.65	1.36
YW138	0.48	11.00	1.34
YW139	1.30	11.82	1.37
YW140	-0.17	10.35	1.42
YW141	0.46	10.98	1.47
YW142	-3.36	7.16	1.37
YW143	-1.84	8.68	1.39
YW144	-2.88	7.64	1.39
YW145	-2.97	7.55	1.34
YW146	-2.76	7.76	1.39
YW147	-0.13	10.39	1.62
YW148	-2.55	7.97	1.69
YW149	-1.59	8.93	1.53
YW150	0.20	10.72	1.46
YW151	-0.93	9.59	1.40
YW152	-2.39	8.13	1.34
YW154	-0.75	9.77	1.71
YW155	-2.25	8.27	1.35
YW157	1.06	11.58	1.47
YW158	1.53	12.05	1.36
YW159	-1.34	9.18	1.79
YW160	-3.21	7.31	1.54
YW161	-2.62	7.90	1.65
YW162	1.96	12.48	1.40
YW163	0.66	11.18	1.39
YW164	0.91	11.43	1.53
YW165	0.65	11.17	1.39
YW166	1.90	12.42	1.33
YW167	1.52	12.04	1.43
YW168	1.39	11.91	1.43
YW169	-2.23	8.29	1.39
YW170	-2.64	7.88	1.39
YW171	2.68	13.20	1.47
YW172	1.07	11.59	1.35
YW173	-1.96	8.56	1.43
YW174	-1.08	9.44	1.47
YW175	-0.29	10.23	1.42
YW176	1.18	11.70	1.54
YW177	-0.49	10.03	1.65
YW178	0.93	11.45	1.44
YW179	-2.11	8.41	1.40
YW180	-0.74	9.78	1.39
YW181	6.20	16.72	1.45

YW135	-1.76	11.15	1.38
YW136	-1.83	11.08	1.40
YW137	3.60	16.51	1.38
YW138	-0.03	12.88	1.38
YW139	-2.35	10.56	1.40
YW140	0.04	12.96	1.39
YW141	1.51	14.42	1.38
YW142	-1.02	11.89	1.40
YW143	-1.48	11.43	1.38
YW144	-2.78	10.13	1.38
YW145	-1.94	10.97	1.40
YW146	-2.07	10.85	1.42
YW147	0.73	13.64	1.39
YW148	-3.76	9.15	1.38
YW149	-3.51	9.41	1.39
YW150	-0.01	12.90	1.39
YW151	-1.77	11.14	1.39
YW152	-0.89	12.02	1.42
YW154	-2.85	10.06	1.40
YW155	-1.98	10.93	1.46
YW157	-1.04	11.88	1.40
YW158	1.98	14.89	1.39
YW159	-2.34	10.57	1.38
YW160	-3.26	9.65	1.38
YW161	-4.37	8.54	1.39
YW162	0.59	13.50	1.38
YW163	4.11	17.02	1.46
YW164	-0.02	12.89	1.39
YW165	-0.64	12.27	1.39
YW166	2.74	15.66	1.38
YW167	1.34	14.25	1.38
YW168	0.22	13.13	1.40
YW169	-1.89	11.02	1.38
YW170	-3.12	9.80	1.39
YW171	4.66	17.57	1.40
YW172	1.84	14.75	1.40
YW173	-1.20	11.71	1.38
YW174	-2.97	9.94	1.40
YW175	0.97	13.88	1.52
YW176	1.65	14.56	1.39
YW177	0.30	13.21	1.35
YW178	2.88	15.79	1.38
YW179	-3.68	9.23	1.38

YW182	1.02	11.54	1.40
YW183	-0.60	9.93	1.38
YW184	-2.25	8.27	1.39
YW185	0.36	10.89	1.46
YW186	-6.09	4.43	1.38
YW187	4.49	15.01	1.40
YW 188 Whiteban		20.30	2.98
YW 189 Yellowcr oss		6.33	2.98

YW180	-1.71	11.20	1.38
YW181	8.03	20.94	1.38
YW182	-1.91	11.00	1.40
YW183	2.08	14.99	1.38
YW184	-3.72	9.19	1.38
YW185	-1.03	11.88	1.38
YW186	-8.77	4.14	1.42
YW187	5.43	18.34	1.38
YW 188 Whiteban		22.71	3.15
YW 189 Yellowcr oss		7.61	3.13

ts parental lines (Table S1b) grown across three
ılum test. EBLUP: empirical best linear unbiased

2023			
Accession	EBLUP	EBLUP with Trial Mean	EBLUP Standard Error
YW001	-3.65	9.68	1.27
YW002	-2.46	10.86	1.27
YW003	1.09	14.42	1.27
YW004	1.83	15.16	1.27
YW005	-3.98	9.35	1.28
YW007	4.82	18.15	1.27
YW009	6.25	19.58	1.27
YW010	1.74	15.07	1.27
YW012	1.61	14.94	1.27
YW013	1.82	15.15	1.27
YW016	-5.29	8.04	1.27
YW017	4.93	18.26	1.27
YW018	-2.24	11.09	1.27
YW019	3.03	16.36	1.27
YW020	-2.79	10.54	1.27
YW021	-2.90	10.42	1.27
YW022	-1.09	12.24	1.27
YW023	-1.88	11.44	1.27
YW024	-1.53	11.79	1.27
YW025	3.30	16.63	1.27
YW026	-1.45	11.88	1.27
YW027	0.86	14.18	1.27
YW028	0.98	14.31	1.27
YW029	-3.70	9.63	1.27
YW030	1.63	14.96	1.27
YW031	-0.39	12.94	1.27
YW032	2.52	15.85	1.27
YW033	-0.62	12.70	1.27
YW034	-2.08	11.25	1.27
YW035	-4.33	8.99	1.27
YW036	0.38	13.70	1.27
YW037	3.90	17.23	1.27
YW038	3.13	16.46	1.27
YW039	6.47	19.80	1.27
YW040	0.97	14.30	1.27
YW041	-2.86	10.47	1.27
YW042	4.16	17.49	1.27
YW043	-1.77	11.56	1.49
YW044	1.51	14.84	1.27

YW045	2.77	16.09	1.27
YW046	2.63	15.96	1.27
YW047	2.57	15.90	1.27
YW048	-0.49	12.84	1.27
YW049	-1.47	11.86	1.27
YW050	1.27	14.60	1.27
YW051	1.92	15.25	1.27
YW052	4.99	18.32	1.27
YW053	-0.39	12.93	1.28
YW054	-3.86	9.47	1.27
YW055	-3.04	10.29	1.27
YW056	2.94	16.26	1.27
YW057	6.29	19.62	1.27
YW059	4.22	17.55	1.27
YW060	-0.98	12.35	1.27
YW061	-1.69	11.64	1.27
YW062	-0.58	12.75	1.27
YW063	4.14	17.47	1.27
YW064	-5.12	8.20	1.27
YW065	-0.67	12.66	1.27
YW066	0.72	14.05	1.27
YW067	-0.73	12.59	1.27
YW068	1.10	14.43	1.27
YW069	-0.90	12.42	1.27
YW070	2.49	15.82	1.27
YW072	1.83	15.16	1.27
YW073	4.92	18.25	1.27
YW074	-1.31	12.02	1.27
YW075	0.78	14.11	1.27
YW076	0.28	13.61	1.27
YW077	-2.87	10.46	1.27
YW078	2.95	16.27	1.27
YW079	-1.21	12.12	1.27
YW080	-4.61	8.72	1.27
YW081	-0.36	12.97	1.27
YW082	3.36	16.69	1.28
YW083	-0.97	12.36	1.27
YW084	-2.32	11.01	1.27
YW085	2.24	15.57	1.27
YW088	-6.38	6.95	1.28
YW090	-2.55	10.77	1.27
YW091	-0.46	12.86	1.27
YW094	0.59	13.92	1.27
YW095	-2.26	11.06	1.27
YW097	1.24	14.57	1.27
YW099	-2.40	10.92	1.27
YW101	6.02	19.35	1.27

YW102	-0.98	12.35	1.27
YW103	2.53	15.86	1.27
YW104	-0.61	12.71	1.27
YW107	-0.57	12.75	1.27
YW111	-2.55	10.78	1.27
YW114	-1.44	11.89	1.27
YW115	-0.61	12.72	1.27
YW116	0.60	13.93	1.27
YW117	-5.79	7.54	1.50
YW120	-2.16	11.16	1.27
YW121	0.26	13.59	1.27
YW122	6.89	20.21	1.27
YW123	-0.45	12.88	1.27
YW125	-3.19	10.14	1.27
YW126	-4.63	8.70	1.27
YW128	1.96	15.29	1.27
YW129	1.14	14.47	1.27
YW130	-2.94	10.38	1.27
YW131	1.56	14.89	1.27
YW132	2.81	16.14	1.27
YW133	1.93	15.26	1.27
YW135	-1.71	11.62	1.27
YW136	0.27	13.59	1.27
YW137	2.95	16.28	1.27
YW138	-0.61	12.71	1.27
YW139	-1.17	12.16	1.27
YW140	-0.52	12.80	1.27
YW141	4.51	17.83	1.27
YW142	-3.12	10.21	1.27
YW143	-5.33	8.00	1.27
YW145	-5.55	7.78	1.27
YW147	4.74	18.07	1.27
YW148	-3.47	9.85	1.27
YW149	0.91	14.24	1.27
YW152	-5.05	8.28	1.27
YW154	-0.90	12.43	1.27
YW155	0.25	13.58	1.27
YW157	-1.13	12.20	1.27
YW158	5.39	18.72	1.27
YW159	0.24	13.57	1.27
YW160	-5.02	8.30	1.27
YW161	0.33	13.66	1.27
YW162	3.83	17.15	1.27
YW164	-2.95	10.37	1.27
YW165	1.63	14.95	1.27
YW166	1.32	14.65	1.27
YW167	-1.89	11.43	1.27

YW168	0.87	14.20	1.27
YW169	3.43	16.76	1.27
YW170	-3.74	9.58	1.27
YW171	3.63	16.96	1.27
YW172	0.95	14.28	1.27
YW173	-0.68	12.65	1.27
YW175	2.38	15.71	1.27
YW176	-1.58	11.75	1.27
YW177	1.81	15.14	1.28
YW178	-2.59	10.74	1.27
YW179	-5.30	8.03	1.27
YW180	-2.72	10.61	1.27
YW181	6.43	19.76	1.27
YW182	-2.00	11.32	1.27
YW183	-4.83	8.50	1.27
YW184	-0.43	12.89	1.27
YW185	-2.75	10.57	1.28
YW186	-9.32	4.01	1.27
YW187	3.65	16.98	1.27
YW188 Whiteban	6.97	20.30	1.27
YW189 Yellowcross	-2.43	10.90	1.27

Table S1b Predicted mean values for rupture energy (mJ) in the parental lines of the YWDH p
and check accessions (Table S1a). Rupture energy was measured by the pendulum test. EE
empiritcal best linear unbiased predictions. SE: standard error

	2013	
Accession	EBLUP with Trial Mean	EBLUP SE
ATC 93184	5.62	3.15
ATC 93883	4.22	3.18
ATC 94114	5.14	3.59
ATC 94126	16.00	3.08
ATC 94348	2.66	3.12
ATC 95379	6.19	3.17
BLN2762	2.82	3.05
BLN3614	1.57	3.05
Chon nam	3.81	3.38
OasisCL	3.35	3.06
Surpass400	3.73	3.14
Whiteban	20.30	2.98
Yellowcross	6.33	2.98

	2014
Accession	EBLUP with Trial Mean
ATC 93184	5.63
ATC 93883	4.29
ATC 94114	11.68
ATC 94126	19.33
ATC 94348	2.96
ATC 95379	8.80
BLN2762	3.43
BLN3614	5.75
Chon nam	3.17
OasisCL	2.04
Surpass400	2.43
Torch	3.22
YW 188 Whiteb	22.71
YW 189 Yellow	7.61

opulation
3LUP:

EBLUP SE
3.27
3.27
3.30
3.27
3.27
3.27
3.27
3.27
3.37
3.27
3.27
3.69
3.15
3.13

Table S2 Predicted mean for average pod length of the parental lines and YWDH lines along with chec

	2013				
	ID	Predicted. Value	EBLUP with trial mean	Std.er ror	Accuracy
1	ATC 93184-	0.00	55.55	5.32	NA
2	ATC 93883	0.00	55.55	5.32	NA
3	ATC 94114	0.00	55.55	5.32	NA
4	ATC 94126	0.00	55.55	5.32	NA
5	ATC 94348	0.00	55.55	5.32	NA
6	ATC 95379	0.00	55.55	5.32	NA
7	BLN2762	0.00	55.55	5.32	NA
8	BLN3614	0.00	55.55	5.32	NA
9	Chon name	0.00	55.55	5.32	NA
10	OasisCL	7.94E-15	55.55	5.32	NA
11	Surpass400	0.00	55.55	5.32	NA
12	Whiteban	2.15E-15	55.55	5.32	NA
13	Yellowcross	0.00	55.55	5.32	NA
14	YW001	0.11	55.66	2.21	0.91
15	YW002	-8.33	47.22	1.92	0.93
16	YW003	5.38	60.93	2.16	0.91
17	YW005	-0.29	55.26	2.03	0.92
18	YW006	-2.55	53.00	2.58	0.87
19	YW007	-5.49	50.06	2.02	0.93
20	YW008	-7.46	48.09	2.17	0.91
21	YW009	-6.52	49.03	2.58	0.87
22	YW010	-0.03	55.52	2.04	0.92
23	YW011	5.38	60.93	1.94	0.93
24	YW012	1.01	56.56	1.99	0.93
25	YW013	1.17	56.72	2.05	0.92
26	YW014	-4.32	51.23	1.98	0.93
27	YW015	-5.05	50.50	2.22	0.91
28	YW016	-1.21	54.34	2.32	0.90
29	YW017	0.07	55.62	2.23	0.91
30	YW018	-0.26	55.29	1.92	0.93
31	YW019	-0.09	55.46	2.35	0.90
32	YW020	-1.08	54.47	1.99	0.93
33	YW022	-5.65	49.90	2.11	0.92
34	YW023	0.19	55.74	2.11	0.92
35	YW024	-3.50	52.05	2.17	0.91
36	YW025	-0.09	55.46	2.02	0.93
37	YW026	-1.39	54.16	2.59	0.87
38	YW027	-6.90	48.65	2.42	0.89
39	YW028	0.22	55.77	2.02	0.92
40	YW029	-6.70	48.85	2.44	0.89
41	YW030	-0.26	55.29	2.11	0.92
42	YW031	4.87	60.42	2.23	0.91
43	YW032	4.03	59.58	2.05	0.92

	2014
	ID
1	ATC 93184-
2	ATC 93883
3	ATC 94114
4	ATC 94126
5	ATC 94348
6	ATC 95379
7	BLN2762
8	BLN3614
9	Chon nam
10	OasisCL
11	Surpass400
12	Torch
13	Whiteban
14	Yellowcross
15	YW001
16	YW002
17	YW003
18	YW004
19	YW005
20	YW006
21	YW007
22	YW008
23	YW009
24	YW010
25	YW011
26	YW012
27	YW013
28	YW014
29	YW015
30	YW016
31	YW017
32	YW018
33	YW019
34	YW020
35	YW021
36	YW022
37	YW023
38	YW024
39	YW025
40	YW026
41	YW027
42	YW028
43	YW029

44	YW033	3.02	58.57	2.17	0.91
45	YW034	0.17	55.72	2.06	0.92
46	YW035	1.26	56.81	2.02	0.93
47	YW036	10.99	66.54	2.37	0.90
48	YW037	12.75	68.30	2.11	0.92
49	YW038	0.61	56.16	2.05	0.92
50	YW039	10.64	66.19	2.03	0.92
51	YW040	-5.58	49.97	2.23	0.91
52	YW041	1.53	57.08	2.58	0.87
53	YW042	1.99	57.54	2.11	0.92
54	YW043	1.76	57.31	1.93	0.93
55	YW044	2.12	57.67	2.05	0.92
56	YW045	-1.86	53.69	2.05	0.92
57	YW046	-2.48	53.07	2.37	0.90
58	YW047	3.88	59.43	2.02	0.93
59	YW048	-1.19	54.36	2.33	0.90
60	YW049	1.32	56.87	2.45	0.89
61	YW050	14.91	70.46	1.94	0.93
62	YW051	1.51	57.06	2.59	0.87
63	YW052	-6.04	49.51	2.35	0.90
64	YW053	-5.36	50.19	2.06	0.92
65	YW054	4.22	59.77	1.92	0.93
66	YW055	-4.90	50.65	2.05	0.92
67	YW056	3.63	59.18	1.94	0.93
68	YW057	-5.44	50.11	1.97	0.93
69	YW058	7.21	62.76	2.06	0.92
70	YW059	11.54	67.09	2.12	0.92
71	YW060	-2.76	52.79	2.05	0.92
72	YW061	9.17	64.72	2.58	0.87
73	YW062	4.10	59.65	2.58	0.87
74	YW063	-4.82	50.73	2.16	0.91
75	YW064	-1.66	53.89	2.02	0.93
76	YW065	10.87	66.42	2.06	0.92
77	YW066	-5.42	50.13	2.03	0.92
78	YW067	-0.95	54.60	2.11	0.92
79	YW068	-5.25	50.30	1.94	0.93
80	YW069	2.84	58.39	1.94	0.93
81	YW070	0.13	55.68	1.95	0.93
82	YW071	1.54	57.09	1.98	0.93
83	YW072	1.59	57.14	2.05	0.92
84	YW073	5.55	61.10	2.03	0.92
85	YW074	-4.20	51.35	2.24	0.91
86	YW075	-1.70	53.85	1.99	0.93
87	YW076	1.63	57.18	2.05	0.92
88	YW077	4.94	60.49	2.06	0.92
89	YW078	-7.31	48.24	2.02	0.93
90	YW079	-1.98	53.57	1.94	0.93

44	YW030
45	YW031
46	YW032
47	YW033
48	YW034
49	YW035
50	YW036
51	YW037
52	YW038
53	YW039
54	YW040
55	YW041
56	YW042
57	YW043
58	YW044
59	YW045
60	YW046
61	YW047
62	YW048
63	YW049
64	YW050
65	YW051
66	YW052
67	YW053
68	YW054
69	YW055
70	YW056
71	YW057
72	YW058
73	YW059
74	YW060
75	YW061
76	YW062
77	YW063
78	YW064
79	YW065
80	YW066
81	YW067
82	YW068
83	YW069
84	YW070
85	YW071
86	YW072
87	YW073
88	YW074
89	YW075
90	YW076

91	YW080	3.31	58.86	2.05	0.92
92	YW081	6.72	62.27	2.17	0.91
93	YW082	9.98	65.53	1.94	0.93
94	YW083	0.68	56.23	2.02	0.93
95	YW084	-7.09	48.46	2.02	0.93
96	YW085	-5.67	49.88	1.92	0.93
97	YW086	5.28	60.83	2.45	0.89
98	YW088	-3.09	52.46	1.94	0.93
99	YW089	-2.23	53.32	2.05	0.92
100	YW090	-6.95	48.60	2.06	0.92
101	YW091	-0.53	55.02	2.17	0.91
102	YW092	-7.54	48.01	1.94	0.93
103	YW093	-3.93	51.62	1.92	0.93
104	YW094	5.16	60.71	1.94	0.93
105	YW095	-0.03	55.52	1.96	0.93
106	YW096	-2.51	53.04	2.05	0.92
107	YW097	-4.14	51.41	2.02	0.93
108	YW098	-0.73	54.82	2.17	0.91
109	YW099	-3.83	51.72	2.06	0.92
110	YW100	6.59	62.14	2.05	0.92
111	YW101	7.32	62.87	2.03	0.92
112	YW102	-1.10	54.45	2.11	0.92
113	YW103	5.22	60.77	2.42	0.89
114	YW104	3.92	59.47	2.44	0.89
115	YW105	-9.25	46.30	2.11	0.92
116	YW106	-2.96	52.59	1.98	0.93
117	YW107	0.00	55.55	1.98	0.93
118	YW108	-4.57	50.98	2.44	0.89
119	YW109	-4.38	51.17	2.02	0.93
120	YW110	-7.69	47.86	2.02	0.93
121	YW111	1.67	57.22	2.05	0.92
122	YW112	-3.30	52.25	1.92	0.93
123	YW113	-3.93	51.62	2.46	0.89
124	YW114	1.75	57.30	2.42	0.89
125	YW115	3.88	59.43	2.35	0.90
126	YW116	10.49	66.04	1.94	0.93
127	YW117	5.80	61.35	2.06	0.92
128	YW118	-5.15	50.40	2.11	0.92
129	YW119	-4.44	51.11	2.02	0.92
130	YW120	-3.66	51.89	1.98	0.93
131	YW121	10.19	65.74	2.35	0.90
132	YW122	-7.65	47.90	2.44	0.89
133	YW123	-0.85	54.70	1.98	0.93
134	YW124	-0.91	54.64	2.06	0.92
135	YW125	-3.15	52.40	2.35	0.90
136	YW126	7.91	63.46	2.11	0.92
137	YW127	-5.62	49.93	2.02	0.93

91	YW077
92	YW078
93	YW079
94	YW080
95	YW081
96	YW082
97	YW083
98	YW084
99	YW085
100	YW086
101	YW088
102	YW089
103	YW090
104	YW091
105	YW092
106	YW093
107	YW094
108	YW095
109	YW096
110	YW097
111	YW098
112	YW099
113	YW100
114	YW101
115	YW102
116	YW103
117	YW104
118	YW105
119	YW106
120	YW107
121	YW108
122	YW109
123	YW110
124	YW111
125	YW112
126	YW113
127	YW114
128	YW115
129	YW116
130	YW117
131	YW118
132	YW119
133	YW120
134	YW121
135	YW122
136	YW123
137	YW124

138	YW128	5.74	61.29	2.58	0.87
139	YW129	-1.67	53.88	1.92	0.93
140	YW130	-6.70	48.85	1.98	0.93
141	YW131	-3.72	51.83	2.17	0.91
142	YW132	0.90	56.45	1.98	0.93
143	YW133	3.22	58.77	1.94	0.93
144	YW134	1.90	57.45	2.36	0.90
145	YW135	6.29	61.84	2.58	0.87
146	YW136	-1.25	54.30	1.92	0.93
147	YW137	-0.17	55.38	1.98	0.93
148	YW138	-2.14	53.41	1.94	0.93
149	YW139	0.28	55.83	1.98	0.93
150	YW140	-4.41	51.14	2.11	0.92
151	YW141	-7.67	47.88	2.23	0.91
152	YW142	1.60	57.15	2.02	0.93
153	YW143	11.98	67.53	2.03	0.92
154	YW144	-1.23	54.32	2.05	0.92
155	YW145	-7.49	48.06	1.92	0.93
156	YW146	-0.81	54.74	2.03	0.92
157	YW147	5.68	61.23	2.35	0.90
158	YW148	3.05	58.60	2.58	0.87
159	YW149	-4.17	51.38	2.33	0.90
160	YW150	5.30	60.85	2.22	0.91
161	YW151	-1.64	53.91	2.05	0.92
162	YW152	-3.92	51.63	1.94	0.93
163	YW154	2.86	58.41	2.59	0.87
164	YW155	-4.55	51.00	1.96	0.93
165	YW157	-4.35	51.20	2.22	0.91
166	YW158	1.07	56.62	1.98	0.93
167	YW159	-2.37	53.18	2.82	0.85
168	YW160	4.86	60.41	2.42	0.89
169	YW161	2.46	58.01	2.44	0.89
170	YW162	2.31	57.86	2.04	0.92
171	YW163	2.07	57.62	2.05	0.92
172	YW164	-0.95	54.60	2.33	0.90
173	YW165	4.27	59.82	2.05	0.92
174	YW166	1.30	56.85	1.92	0.93
175	YW167	1.30	56.85	2.11	0.92
176	YW168	-4.23	51.32	2.12	0.92
177	YW169	2.48	58.03	2.05	0.92
178	YW170	-2.12	53.43	2.03	0.92
179	YW171	-4.24	51.31	2.22	0.91
180	YW172	-4.17	51.38	1.94	0.93
181	YW173	10.87	66.42	2.11	0.92
182	YW174	-0.75	54.80	2.23	0.91
183	YW175	-3.33	52.22	2.11	0.92
184	YW176	-0.17	55.38	2.41	0.89

138	YW125
139	YW126
140	YW127
141	YW128
142	YW129
143	YW130
144	YW131
145	YW132
146	YW133
147	YW134
148	YW135
149	YW136
150	YW137
151	YW138
152	YW139
153	YW140
154	YW141
155	YW142
156	YW143
157	YW144
158	YW145
159	YW146
160	YW147
161	YW148
162	YW149
163	YW150
164	YW151
165	YW152
166	YW154
167	YW155
168	YW157
169	YW158
170	YW159
171	YW160
172	YW161
173	YW162
174	YW163
175	YW164
176	YW165
177	YW166
178	YW167
179	YW168
180	YW169
181	YW170
182	YW171
183	YW172
184	YW173

185	YW177	3.75	59.30	2.44	0.89
186	YW178	-4.30	51.25	2.18	0.91
187	YW179	1.25	56.80	2.03	0.92
188	YW180	-4.49	51.06	2.06	0.92
189	YW181	-1.79	53.76	2.18	0.91
190	YW182	-3.48	52.07	2.06	0.92
191	YW183	-0.16	55.39	2.02	0.93
192	YW184	0.38	55.93	2.05	0.92
193	YW185	3.43	58.98	2.13	0.92
194	YW186	-6.56	48.99	2.02	0.93
195	YW187	5.62	61.17	2.03	0.92

185	YW174
186	YW175
187	YW176
188	YW177
189	YW178
190	YW179
191	YW180
192	YW181
193	YW182
194	YW183
195	YW184
196	YW185
197	YW186
198	YW187

ck accessions grown at Wagga Wagga Agricultural Institute during the 2013, 2014 and 2023 gro

2023.00

Predicted. Value	EBLUP with trial mean	Std.error	Accuracy		ID
0.00	57.39	6.10	NA	1	YW001
0.00	57.39	6.10	NA	2	YW002
0.00	57.39	6.10	NA	3	YW003
0.00	57.39	6.10	NA	4	YW004
0.00	57.39	6.10	NA	5	YW005
0.00	57.39	6.10	NA	6	YW007
0.00	57.39	6.10	NA	7	YW009
0.00	57.39	6.10	NA	8	YW010
0.00	57.39	6.10	NA	9	YW012
0.00	57.39	6.10	NA	10	YW013
0.00	57.39	6.10	NA	11	YW016
0.00	57.39	6.10	NA	12	YW017
0.00	57.39	6.10	NA	13	YW018
0.00	57.39	6.10	NA	14	YW019
0.96	58.35	1.92	0.95	15	YW020
-9.74	47.65	1.94	0.95	16	YW021
14.99	72.38	1.92	0.95	17	YW022
-5.40	51.99	1.86	0.95	18	YW023
-0.73	56.66	1.92	0.95	19	YW024
-5.63	51.76	1.91	0.95	20	YW025
-6.31	51.08	1.92	0.95	21	YW026
-5.05	52.34	1.92	0.95	22	YW027
-9.62	47.77	1.97	0.95	23	YW028
-2.13	55.26	1.96	0.95	24	YW029
3.08	60.47	1.89	0.95	25	YW030
-2.06	55.33	1.92	0.95	26	YW031
2.27	59.66	1.96	0.95	27	YW032
-8.37	49.02	1.91	0.95	28	YW033
-3.47	53.92	1.95	0.95	29	YW034
-0.56	56.83	1.92	0.95	30	YW035
-0.96	56.43	1.94	0.95	31	YW036
-2.04	55.35	1.92	0.95	32	YW037
-0.95	56.44	1.95	0.95	33	YW038
-0.70	56.69	1.91	0.95	34	YW039
-3.76	53.63	1.93	0.95	35	YW040
-5.54	51.85	1.92	0.95	36	YW041
3.13	60.52	1.94	0.95	37	YW042
-4.21	53.18	1.92	0.95	38	YW043
-1.20	56.19	2.02	0.94	39	YW044
-5.59	51.80	1.94	0.95	40	YW045
-1.96	55.43	1.93	0.95	41	YW046
-0.48	56.91	1.92	0.95	42	YW047
0.51	57.90	1.92	0.95	43	YW048

-1.10	56.29	1.92	0.95
3.40	60.79	1.92	0.95
2.96	60.35	1.92	0.95
5.10	62.49	1.92	0.95
1.95	59.34	1.92	0.95
4.84	62.23	1.92	0.95
9.26	66.65	1.91	0.95
15.03	72.42	1.91	0.95
-1.50	55.89	1.92	0.95
9.83	67.22	1.91	0.95
-6.62	50.77	1.91	0.95
0.69	58.08	1.91	0.95
3.70	61.09	1.95	0.95
0.92	58.31	1.91	0.95
-3.73	53.66	1.95	0.95
-3.99	53.40	1.92	0.95
-5.92	51.47	1.92	0.95
7.24	64.63	1.92	0.95
-1.36	56.03	2.03	0.94
1.72	59.11	1.89	0.95
17.11	74.50	1.90	0.95
-0.43	56.96	1.91	0.95
-3.98	53.41	1.92	0.95
-12.28	45.11	1.91	0.95
3.44	60.83	1.91	0.95
-3.86	53.53	1.92	0.95
4.26	61.65	1.92	0.95
-3.24	54.15	1.92	0.95
9.20	66.59	2.05	0.94
9.84	67.23	1.92	0.95
-3.40	53.99	1.92	0.95
11.32	68.71	1.94	0.95
1.19	58.58	1.91	0.95
-14.74	42.65	1.91	0.95
5.42	62.81	1.91	0.95
4.17	61.56	1.91	0.95
-3.52	53.87	1.92	0.95
-3.17	54.22	1.91	0.95
-3.87	53.52	1.90	0.95
5.34	62.73	1.94	0.95
-1.04	56.35	1.94	0.95
2.43	59.82	1.92	0.95
0.33	57.72	1.92	0.95
7.21	64.60	1.91	0.95
-2.88	54.51	1.91	0.95
-0.61	56.78	2.02	0.94
1.32	58.71	1.92	0.95

44	YW049
45	YW050
46	YW051
47	YW052
48	YW053
49	YW054
50	YW055
51	YW056
52	YW057
53	YW059
54	YW060
55	YW061
56	YW062
57	YW063
58	YW064
59	YW065
60	YW066
61	YW067
62	YW068
63	YW069
64	YW070
65	YW072
66	YW073
67	YW074
68	YW075
69	YW076
70	YW077
71	YW078
72	YW079
73	YW080
74	YW081
75	YW082
76	YW083
77	YW084
78	YW085
79	YW088
80	YW090
81	YW091
82	YW094
83	YW095
84	YW097
85	YW099
86	YW101
87	YW102
88	YW103
89	YW104
90	YW107

10.53	67.92	1.91	0.95
-8.33	49.06	1.95	0.95
-0.62	56.77	1.91	0.95
4.47	61.86	1.95	0.95
4.21	61.60	1.92	0.95
10.73	68.12	1.92	0.95
2.27	59.66	1.92	0.95
-9.39	48.00	1.94	0.95
-7.18	50.21	1.93	0.95
6.03	63.42	1.92	0.95
0.23	57.62	1.94	0.95
-3.65	53.74	1.91	0.95
-8.36	49.03	2.03	0.94
1.30	58.69	1.91	0.95
2.49	59.88	1.92	0.95
-8.22	49.17	1.96	0.95
4.57	61.96	1.91	0.95
-0.86	56.53	1.91	0.95
2.90	60.29	1.91	0.95
-3.76	53.63	1.91	0.95
-0.91	56.48	1.96	0.95
-6.98	50.41	1.92	0.95
8.95	66.34	1.91	0.95
4.96	62.35	1.92	0.95
-1.41	55.98	1.92	0.95
7.59	64.98	1.95	0.95
4.07	61.46	1.93	0.95
-7.86	49.53	1.95	0.95
0.31	57.70	1.95	0.95
2.54	59.93	1.92	0.95
-7.58	49.81	1.92	0.95
-2.97	54.42	1.91	0.95
-7.46	49.93	1.91	0.95
4.40	61.79	1.91	0.95
-6.19	51.20	1.91	0.95
-3.45	53.94	1.95	0.95
0.68	58.07	2.41	0.92
8.01	65.40	1.93	0.95
14.75	72.14	1.95	0.95
7.22	64.61	1.92	0.95
-4.70	52.69	1.95	0.95
-1.78	55.61	1.93	0.95
-7.37	50.02	1.92	0.95
2.56	59.95	1.92	0.95
-7.90	49.49	1.95	0.95
1.34	58.73	1.94	0.95
1.71	59.10	1.92	0.95

91	YW111
92	YW114
93	YW115
94	YW116
95	YW117
96	YW120
97	YW121
98	YW122
99	YW123
100	YW125
101	YW126
102	YW128
103	YW129
104	YW130
105	YW131
106	YW132
107	YW133
108	YW135
109	YW136
110	YW137
111	YW138
112	YW139
113	YW140
114	YW141
115	YW142
116	YW143
117	YW145
118	YW147
119	YW148
120	YW149
121	YW152
122	YW154
123	YW155
124	YW157
125	YW158
126	YW159
127	YW160
128	YW161
129	YW162
130	YW164
131	YW165
132	YW166
133	YW167
134	YW168
135	YW169
136	YW170
137	YW171

-3.14	54.25	1.91	0.95
10.09	67.48	1.92	0.95
-1.09	56.30	1.96	0.95
2.67	60.06	1.91	0.95
-0.75	56.64	1.93	0.95
-5.23	52.16	1.92	0.95
0.43	57.82	1.92	0.95
5.04	62.43	1.92	0.95
7.02	64.41	1.94	0.95
3.38	60.77	1.91	0.95
3.52	60.91	1.91	0.95
-5.50	51.89	1.95	0.95
-3.29	54.10	1.92	0.95
2.66	60.05	1.92	0.95
1.02	58.41	1.95	0.95
-5.62	51.77	1.92	0.95
-12.19	45.20	1.91	0.95
4.12	61.51	1.94	0.95
12.73	70.12	1.92	0.95
-4.77	52.62	1.91	0.95
-11.10	46.29	1.95	0.95
0.91	58.30	1.98	0.95
6.65	64.04	1.92	0.95
4.17	61.56	1.93	0.95
-3.81	53.58	1.93	0.95
8.70	66.09	1.93	0.95
4.95	62.34	1.92	0.95
-1.93	55.46	1.98	0.95
0.17	57.56	1.94	0.95
-9.41	47.98	2.03	0.94
-4.45	52.94	1.95	0.95
-3.64	53.75	1.93	0.95
-3.45	53.94	1.91	0.95
5.67	63.06	1.92	0.95
1.31	58.70	1.92	0.95
5.79	63.18	1.92	0.95
5.33	62.72	2.04	0.94
-3.81	53.58	1.92	0.95
6.01	63.40	1.93	0.95
0.61	58.00	1.92	0.95
-3.07	54.32	1.92	0.95
0.13	57.52	1.95	0.95
4.83	62.22	1.92	0.95
-1.48	55.91	1.92	0.95
-0.81	56.58	1.95	0.95
-6.24	51.15	1.95	0.95
10.37	67.76	1.91	0.95

138	YW172
139	YW173
140	YW175
141	YW176
142	YW177
143	YW178
144	YW179
145	YW180
146	YW181
147	YW182
148	YW183
149	YW184
150	YW185
151	YW186
152	YW187
153	YW188 Whi
154	YW189 Yell

-1.32	56.07	1.94	0.95
-1.98	55.41	2.14	0.94
-4.86	52.53	1.91	0.95
1.20	58.59	1.85	0.95
-9.03	48.36	1.92	0.95
-0.28	57.11	1.91	0.95
-2.04	55.35	1.92	0.95
0.07	57.46	1.92	0.95
-7.00	50.39	1.95	0.95
-3.48	53.91	1.92	0.95
-0.26	57.13	1.91	0.95
5.29	62.68	1.89	0.95
-11.34	46.05	1.97	0.95
3.15	60.54	1.90	0.95

ɔwing seasons. Std.error: Standard error

Predicted. Value	EBLUP with trial mean	Std.error	Accuracy
-1.18	53.26	1.74	0.96
-6.57	47.87	1.74	0.96
12.05	66.48	1.74	0.96
-3.85	50.59	1.74	0.96
-2.64	51.79	1.74	0.96
-3.68	50.75	1.74	0.96
-8.13	46.31	1.74	0.96
-1.55	52.88	1.74	0.96
-4.58	49.85	1.74	0.96
-0.92	53.51	1.74	0.96
2.48	56.91	1.74	0.96
-0.63	53.80	1.74	0.96
-2.52	51.91	1.74	0.96
-0.48	53.95	1.74	0.96
-6.51	47.93	1.74	0.96
0.94	55.38	1.74	0.96
-4.11	50.32	1.74	0.96
-1.30	53.14	1.74	0.96
-2.14	52.29	1.74	0.96
-5.70	48.74	1.74	0.96
-4.30	50.13	1.74	0.96
-2.40	52.03	1.74	0.96
1.44	55.88	1.74	0.96
3.86	58.29	1.74	0.96
-1.04	53.39	1.74	0.96
-0.01	54.42	1.74	0.96
5.55	59.99	1.74	0.96
2.27	56.71	1.74	0.96
-0.49	53.94	1.74	0.96
8.82	63.25	1.74	0.96
16.81	71.25	1.74	0.96
14.18	68.61	1.74	0.96
-5.40	49.04	1.74	0.96
6.81	61.25	1.74	0.96
-9.72	44.71	1.74	0.96
1.12	55.55	1.74	0.96
3.58	58.02	1.74	0.96
5.69	60.13	2.06	0.95
-5.33	49.10	1.74	0.96
-2.73	51.70	1.74	0.96
-0.66	53.77	1.74	0.96
3.46	57.89	1.74	0.96
-7.01	47.42	1.74	0.96

5.19	59.63	1.74	0.96
11.54	65.97	1.74	0.96
2.30	56.74	1.74	0.96
-5.06	49.37	1.74	0.96
-12.69	41.74	1.74	0.96
0.37	54.80	1.74	0.96
-0.06	54.37	1.74	0.96
7.43	61.86	1.74	0.96
-5.45	48.99	1.74	0.96
11.96	66.40	1.74	0.96
-5.76	48.68	1.74	0.96
8.54	62.97	1.74	0.96
4.04	58.48	1.74	0.96
-9.72	44.71	1.74	0.96
5.74	60.17	1.74	0.96
5.93	60.36	1.74	0.96
-3.86	50.58	1.74	0.96
-7.43	47.00	1.74	0.96
-5.96	48.47	1.74	0.96
5.01	59.44	1.74	0.96
1.20	55.64	1.74	0.96
-1.63	52.80	1.74	0.96
14.06	68.49	1.74	0.96
-2.76	51.67	1.74	0.96
-4.71	49.73	1.74	0.96
-1.18	53.25	1.74	0.96
11.33	65.76	1.74	0.96
-8.07	46.37	1.74	0.96
-0.50	53.93	1.74	0.96
5.05	59.48	1.74	0.96
4.09	58.52	1.74	0.96
9.28	63.72	1.74	0.96
-6.59	47.84	1.74	0.96
-1.29	53.14	1.74	0.96
-8.81	45.62	1.74	0.96
2.05	56.48	1.74	0.96
-9.55	44.88	1.74	0.96
1.29	55.72	1.74	0.96
6.59	61.03	1.74	0.96
-3.64	50.79	1.74	0.96
-3.00	51.43	1.74	0.96
-11.58	42.85	1.74	0.96
1.97	56.40	1.74	0.96
-1.75	52.68	1.74	0.96
3.48	57.91	1.74	0.96
8.02	62.45	1.74	0.96
0.26	54.69	1.74	0.96

5.85	60.28	1.74	0.96
-3.20	51.23	1.74	0.96
9.92	64.35	1.74	0.96
18.46	72.89	1.74	0.96
12.01	66.44	2.06	0.95
-4.77	49.66	1.74	0.96
8.08	62.52	1.74	0.96
-5.54	48.89	1.74	0.96
-0.44	53.99	1.74	0.96
-4.42	50.01	1.74	0.96
13.20	67.63	1.74	0.96
4.53	58.96	1.74	0.96
-7.49	46.94	1.74	0.96
-7.22	47.21	1.74	0.96
-0.35	54.09	1.74	0.96
2.67	57.10	1.74	0.96
-2.00	52.43	1.74	0.96
3.24	57.67	1.74	0.96
-4.82	49.61	1.74	0.96
-0.94	53.49	1.74	0.96
-6.63	47.80	1.74	0.96
3.92	58.36	1.74	0.96
-3.10	51.33	1.74	0.96
-9.87	44.56	1.74	0.96
0.58	55.01	1.74	0.96
15.41	69.85	1.74	0.96
-8.18	46.25	1.74	0.96
10.52	64.96	1.74	0.96
3.18	57.61	1.74	0.96
2.38	56.81	1.74	0.96
-2.08	52.35	1.74	0.96
2.95	57.38	1.74	0.96
-0.08	54.35	1.74	0.96
-4.31	50.12	1.74	0.96
-1.10	53.33	1.74	0.96
-3.77	50.66	1.74	0.96
-0.10	54.33	1.74	0.96
6.50	60.93	1.74	0.96
-1.38	53.05	1.74	0.96
-10.84	43.59	1.74	0.96
4.73	59.17	1.74	0.96
0.47	54.91	1.74	0.96
-3.65	50.78	1.74	0.96
-5.07	49.36	1.74	0.96
6.62	61.05	1.74	0.96
-6.68	47.75	1.74	0.96
-3.45	50.99	1.74	0.96

-4.89	49.54	1.74	0.96
6.86	61.29	1.74	0.96
-2.14	52.29	1.74	0.96
-4.42	50.01	1.74	0.96
4.76	59.19	1.74	0.96
-8.71	45.73	1.74	0.96
0.04	54.48	1.74	0.96
-3.28	51.15	1.74	0.96
-2.05	52.38	1.74	0.96
-12.43	42.00	1.74	0.96
-5.38	49.05	1.74	0.96
0.15	54.59	1.74	0.96
1.64	56.07	1.74	0.96
-4.76	49.67	1.74	0.96
1.06	55.49	1.74	0.96
-1.81	52.62	1.74	0.96
2.49	56.92	1.74	0.96

**Table S3 The nomenclature and orientation of the linkage groups of the YWDH genetic map of *B. carinata* updated according to the physical alignment with *B. nigra* Ni100 and *B. oleracea* TO1000.**		
**The previous nomenclature**	**Re-nomenclature**	**Orientation**
B1	B7	No change
B2	B5	No change
B3	B8	Invert
B4	B6	No change
B5	B1	Invert
B6	B4	No change
B7	B3	No change
B8	B2	No change
C1	C1	No change
C2	C2	No change
C3	C3	Invert
C4	C4	Invert
C5	C5	No change
C6	C6	Invert
C7	C7	Invert
C8	C8	No change
C9	C9	No change

Table S4a Genotypic marker data of the YWDH population (Zou et al 2014)

**Locus**	YW001	YW002	YW003	YW004	YW005	YW006	YW007	YW008
100050168	a	-	a	a	a	b	a	b
100008381	a	b	a	a	a	b	a	b
100015564	a	b	a	-	a	b	a	b
100022390	a	b	a	a	a	b	a	b
100008594	a	b	a	a	a	b	a	b
100020052	a	b	a	a	a	b	a	b
100017774	a	b	a	a	a	b	a	b
100055759	a	b	a	a	a	b	a	b
100021591	a	b	a	a	a	b	a	b
100011871	a	b	a	a	a	b	a	b
100060659	a	b	a	a	a	b	a	b
100075729	a	b	a	a	a	b	a	b
100053479	a	b	a	a	a	b	a	b
100048383	a	b	a	-	a	b	a	b
100022941	a	b	a	a	-	b	a	b
100007567	a	b	a	a	a	b	a	b
100027589	a	b	a	-	a	b	a	b
100023033	a	b	a	a	a	b	a	b
100052248	a	b	-	a	a	b	a	b
100041347	a	b	-	a	a	b	a	b
100033400	a	b	a	a	a	b	a	b
e6m3-280	a	b	a	a	a	b	a	b
100039563	-	b	a	a	a	b	a	b
100008824	a	b	-	a	a	b	a	b
100032112	-	b	a	a	b	b	a	b
100017597	a	-	a	a	a	b	a	b
100067851	-	b	a	a	a	b	a	b
100021600	a	b	a	a	a	b	a	b
100007740	a	b	a	a	b	b	a	b
100012258	a	b	a	a	b	b	a	b
100069283	a	b	a	a	b	b	a	b
cnu099	a	b	a	a	b	b	a	b
100048156	a	b	a	a	b	b	a	b
100033725	a	-	a	a	b	b	a	b
100061847	a	b	a	a	b	b	a	b
100055425	a	b	a	a	b	b	a	b
100029387	a	b	a	a	b	b	a	b
100007506	-	b	-	a	b	b	a	b
cnu098	a	b	a	a	b	b	a	b
100018914	a	b	-	a	b	b	a	b
100076398	b	b	a	-	b	b	a	b
100007757	b	b	a	a	b	b	a	b
100013191	b	b	a	a	b	b	a	b
100042453	b	b	a	a	b	b	a	b
100071901	b	b	a	a	b	b	a	b

100043465	b	b	a	a	b	b	-	b
100019987	b	b	a	a	b	b	a	b
100020128	b	b	a	a	b	b	a	b
100037660	b	b	a	a	b	b	a	b
100028301	b	b	a	a	b	b	a	b
100043226	b	b	a	-	b	b	a	b
100022141	b	b	a	a	b	b	a	b
100028838	b	b	a	a	b	b	a	b
100025963	b	b	a	a	b	b	a	b
100052876	b	b	a	-	b	b	a	b
100026124	a	b	a	a	b	b	a	b
100019213	b	b	b	a	b	b	a	b
100053770	b	b	b	a	b	b	a	b
100006973	b	b	b	a	b	b	a	b
100022658	b	b	b	a	b	b	a	b
100046735	b	b	b	a	b	b	a	b
100009190	b	b	b	a	b	b	a	b
100048863	a	b	b	a	b	b	a	b
Na14D07	b	b	b	a	b	b	a	b
100025864	b	b	b	-	b	b	a	b
100061514	b	b	b	-	b	b	a	b
100009526	b	b	b	a	b	b	a	b
100065580	b	b	a	-	-	b	a	b
100026799	b	b	b	-	b	b	a	b
100011772	b	b	b	a	b	b	a	b
100006925	b	b	b	a	b	b	a	b
100031225	a	b	a	a	b	b	a	b
100019404	a	b	b	a	b	b	a	b
100046092	a	b	b	a	b	b	a	b
100018779	a	b	b	a	b	b	a	b
100022946	a	b	-	a	b	b	a	b
100026981	b	b	b	a	b	b	a	b
100028921	a	b	b	a	b	b	a	b
100021852	a	b	b	a	-	b	a	b
100004375	a	b	b	a	b	b	a	b
100072931	a	b	b	a	b	b	a	b
100074843	a	b	b	a	b	b	a	b
100065271	a	-	b	a	b	b	a	b
100054851	a	b	b	-	a	b	a	b
100050773	b	b	a	b	a	b	a	b
100005245	b	b	b	-	a	b	a	b
100023735	a	b	b	b	b	-	a	b
100016421	b	b	b	-	b	b	a	b
100064697	b	b	b	-	b	b	a	b
100008226	b	b	b	b	b	b	a	b
100007307	b	b	b	b	b	b	a	b
100045111	b	b	b	b	b	b	a	b

100060363	b	b	b	b	b	b	a	b
100012220	b	b	b	b	b	b	a	a
100018012	b	b	b	b	b	b	a	a
100023940	-	-	b	b	b	b	a	a
100028569	a	-	b	b	b	b	a	a
100031847	-	b	b	b	b	b	a	a
100041563	b	b	b	b	-	b	a	a
100027247	b	b	-	-	b	b	a	a
100028171	b	b	b	b	b	b	a	a
100002981	-	b	b	b	b	b	a	a
100040381	a	b	b	b	-	b	a	a
100013734	-	b	a	b	b	b	a	a
100016187	a	b	a	b	b	b	a	a
100041395	a	-	a	b	b	b	a	a
100053387	a	b	a	b	b	b	a	a
100059009	-	b	a	b	b	b	a	a
100007386	a	b	-	b	b	b	a	a
100023391	a	b	-	b	b	b	a	a
100004789	a	b	a	b	b	b	a	a
100047433	b	b	a	b	b	b	a	a
100026480	b	b	a	b	b	b	a	a
100055106	-	b	a	b	a	b	a	a
100026976	b	b	a	b	a	b	a	a
100004548	b	-	a	b	a	b	a	a
100015527	b	b	a	b	a	b	a	a
100025898	b	b	a	b	a	b	a	a
100043823	b	b	a	b	a	b	a	a
100033806	-	b	a	b	a	b	a	a
100016777	b	b	a	b	-	b	a	a
100019799	a	b	a	b	a	b	a	a
100021480	b	b	a	b	a	b	a	a
100059161	b	b	a	b	a	b	a	a
100027954	a	a	a	a	a	-	a	a
100051437	-	b	a	a	a	b	a	a
100031574	b	b	a	a	a	b	a	a
100015179	b	b	-	-	a	b	a	a
100064401	b	b	a	a	a	b	a	a
100038131	b	b	a	a	a	b	a	a
100021699	b	b	a	a	a	b	a	a
Ni2F07	b	b	a	a	a	b	a	a
100038759	b	b	a	a	a	b	a	a
100062128	-	-	a	a	a	b	a	a
100045707	b	b	a	a	a	b	a	a
100059844	b	-	a	a	a	b	a	a
100012301	b	b	a	a	a	b	a	a
100043931	b	b	a	a	a	b	a	a
100036573	-	b	a	a	a	b	a	a

100033950	b	b	a	-	a	b	b	a
100060295	b	b	a	a	a	b	b	a
100030774	b	b	a	-	a	b	b	a
100019810	b	-	a	a	a	b	b	a
100032396	-	-	a	a	a	b	b	a
100024893	b	b	-	-	a	b	b	a
100027389	b	-	a	a	a	b	b	a
BRMS046	b	b	a	a	a	b	b	a
100012892	b	b	a	-	a	b	b	a
100016639	b	b	a	a	a	b	b	a
100001282	b	b	a	a	a	b	b	a
Ni2D10	b	b	a	a	a	b	b	a
100021083	b	b	a	a	a	b	b	a
100027670	b	b	a	a	a	b	b	a
100070363	b	-	a	a	a	b	b	a
100048879	b	b	a	a	a	b	b	a
100016457	b	b	a	a	a	b	b	a
100005674	b	b	a	a	a	b	b	a
100028204	b	b	a	a	a	b	b	a
100032373	a	b	a	a	a	b	b	a
100020956	a	-	a	a	a	b	b	a
100016893	a	a	a	a	a	b	b	a
100066710	a	a	a	a	a	b	b	a
100007664	a	a	a	a	a	b	b	a
100030861	a	a	a	a	a	b	b	a
100015095	a	a	a	a	a	b	b	a
100061527	a	a	a	a	a	b	b	b
BnGMS044	a	a	a	a	a	b	b	b
100016835	a	a	a	a	a	b	b	b
100015045	a	a	-	a	a	b	b	b
100048806	a	a	a	a	a	b	b	b
BRMS36	a	a	a	a	a	b	b	b
100037455	a	a	a	a	a	b	b	b
100015465	a	a	a	a	a	b	b	b
100014439	a	a	-	a	a	b	b	b
100001885	a	a	a	a	a	b	b	b
100021113	a	a	a	a	-	b	b	b
100032378	a	a	a	a	a	b	b	b
100026909	a	a	a	a	a	b	b	b
100014070	a	a	a	a	a	b	b	b
100046082	a	a	a	a	a	b	b	b
100038240	a	a	a	a	a	b	b	b
100057131	a	a	a	a	a	b	b	b
100019348	a	a	a	a	a	b	b	b
100067737	a	a	a	a	a	b	b	b
100070871	a	a	a	a	a	b	b	b
100069538	a	a	a	a	a	b	b	b

100054809	a	a	b	a	a	b	b	b
100016993	a	a	b	a	a	b	b	b
100075572	a	a	b	a	a	b	b	b
100034845	a	b	b	a	a	b	b	b
100030357	b	-	b	a	a	b	b	b
100004698	a	a	b	a	a	b	b	b
100071120	a	a	a	a	a	b	b	b
100031323	a	a	b	a	a	b	b	b
100012211	a	a	b	a	a	b	b	b
100024998	a	a	b	a	a	b	b	b
100078356	a	a	b	a	a	b	b	b
100058247	-	a	b	a	a	b	b	b
100066416	b	b	a	a	b	b	a	a
100025362	b	b	a	a	b	b	a	a
100013294	b	b	a	a	b	b	a	a
100071515	b	b	a	a	b	b	a	a
100032326	b	b	a	a	b	b	a	a
100065709	b	b	a	a	b	b	a	a
100007476	b	b	a	a	b	b	a	a
100023660	b	b	a	a	b	b	a	a
100059833	b	b	a	a	b	b	a	a
100040486	-	b	a	a	b	b	a	a
100071108	b	b	a	a	b	b	a	a
100024082	b	b	a	a	b	b	a	a
100042491	b	b	a	a	b	b	a	a
100005372	b	b	a	a	b	b	a	a
100049153	-	-	a	a	-	b	a	a
100002494	b	b	a	a	b	b	a	a
100024018	b	b	a	-	b	b	a	a
100024128	b	b	a	a	b	b	a	a
100017996	b	b	a	a	b	b	a	a
100017061	b	b	a	b	b	b	a	a
e5m5-50	b	b	a	b	b	b	a	a
100018028	b	b	a	b	b	b	a	a
100011719	-	b	a	b	b	b	a	a
100015971	-	b	a	b	b	b	a	a
100043417	a	b	-	b	b	b	a	a
100073700	a	b	a	b	b	b	a	a
100054104	a	b	a	b	b	b	a	a
100052494	-	b	a	b	b	b	a	a
100028386	a	b	a	b	b	b	a	a
100014239	a	-	a	b	b	b	a	a
100076152	a	b	b	b	b	b	a	a
100001440	a	b	b	b	b	a	a	a
100007589	a	b	b	b	b	a	a	a
100039529	a	-	b	b	b	a	a	a
100062881	a	b	b	b	b	a	a	a

100049688	a	b	b	b	b	a	a	a
100045878	a	b	-	b	b	a	a	a
100024846	a	a	b	b	b	a	a	a
100023630	-	-	b	b	b	a	a	a
100006430	a	-	b	b	b	a	a	a
100031529	a	a	b	b	b	a	a	a
100068880	-	a	b	b	b	b	a	a
100037800	b	a	b	b	b	b	a	a
100021262	b	a	b	b	b	b	a	a
100069575	-	a	b	b	b	b	a	a
100022376	b	a	b	b	b	b	a	a
100023615	a	a	b	b	a	b	a	a
100057003	b	a	b	-	b	b	a	a
100077122	b	a	b	b	a	b	a	a
100024207	b	a	-	b	a	b	a	a
100043978	b	a	b	b	-	b	a	a
100077309	b	a	b	-	a	b	a	a
100053505	b	a	b	b	a	b	a	a
100070293	b	a	a	b	a	b	a	a
100070432	b	a	b	-	a	b	a	a
100013735	b	a	b	-	a	b	a	a
100017540	b	a	b	b	a	b	a	a
100063747	b	a	b	-	a	b	a	a
100004378	b	a	b	b	a	b	a	a
100045154	b	a	b	b	a	b	a	a
100030257	b	a	b	b	a	b	a	a
100015259	b	a	b	b	a	b	a	a
100007413	b	a	b	b	a	b	a	a
100018229	-	a	b	b	a	b	a	a
100028818	b	a	-	b	a	b	a	a
100056430	b	a	b	b	a	b	a	-
100078699	b	a	-	b	a	b	a	a
100058267	a	a	b	b	a	a	a	a
em13me19-250	a	a	b	b	a	a	a	a
100006651	a	a	b	b	a	a	a	a
100013491	a	a	-	b	a	a	a	a
100025744	a	a	b	b	a	a	a	a
100064677	a	a	b	b	a	a	a	a
100024312	a	a	b	b	a	a	a	a
100016840	a	a	b	b	a	a	a	a
100012806	a	a	b	-	a	a	a	a
100045580	a	a	b	b	a	a	a	a
100033541	-	a	a	-	a	a	a	a
100066773	-	a	b	b	a	a	a	a
100023649	a	a	b	b	a	a	a	a
100016337	a	a	b	b	a	a	a	a
100044399	-	a	b	b	a	a	a	a

100073600	a	a	b	b	a	a	a	a
100018041	a	a	b	b	a	a	a	a
Na10D07	a	a	b	b	a	a	a	a
100034901	a	a	b	b	a	a	a	a
100035102	a	a	b	-	a	a	a	a
100043101	a	a	-	b	a	a	a	a
100017058	a	a	b	-	a	a	a	a
100024366	a	a	b	b	a	a	b	a
100021572	-	a	b	b	a	a	b	a
100059675	a	a	b	-	a	a	b	a
100022187	a	a	b	b	a	a	b	a
100017045	a	a	b	b	a	a	b	a
100011944	a	a	b	b	a	a	b	a
100056767	a	a	b	b	a	a	b	a
100071710	a	a	b	b	a	a	b	a
100028694	a	a	b	b	a	a	b	a
100010788	a	a	b	b	a	a	b	a
100020924	a	a	-	b	a	a	b	a
100029236	a	a	b	b	a	a	b	a
100050502	a	a	-	b	a	a	b	a
100031662	a	a	b	b	a	a	b	a
100015383	a	a	b	-	a	a	b	a
100005823	a	a	b	b	a	a	b	a
100059103	a	a	b	a	a	a	b	a
100042836	a	-	b	a	a	a	b	a
100052322	a	a	b	a	a	a	b	a
100014804	a	a	b	a	a	a	b	a
100049415	a	a	b	a	a	a	b	a
100017154	a	a	b	a	a	a	b	a
100048959	a	a	b	-	b	a	b	a
100062839	a	a	b	a	b	a	b	a
100026655	a	a	b	a	b	a	b	a
100035330	b	a	b	a	b	a	b	a
100024274	b	a	b	a	b	a	b	a
100025810	b	a	-	a	b	a	b	a
100006325	b	a	b	a	b	a	b	a
100007772	b	a	b	a	b	a	b	a
100040418	b	a	b	a	b	a	b	a
100026725	b	a	a	a	b	a	b	a
100002826	b	a	a	-	b	a	b	a
100036039	a	b	a	a	a	a	b	a
100025352	a	b	a	a	a	a	b	a
100076205	-	b	a	-	a	a	b	a
100078760	-	b	a	-	a	a	b	a
100023564	a	b	a	a	a	a	b	a
100022838	a	b	a	a	a	a	b	a
100016091	a	-	a	a	a	a	b	a

100009834	a	b	a	a	a	a	b	a
100028661	a	b	a	a	a	a	b	a
100017288	a	b	a	a	a	a	b	a
100041742	a	b	a	a	a	a	b	a
100013180	a	b	a	a	a	a	b	a
100062356	a	b	a	a	a	a	b	a
100013392	a	b	a	a	a	a	b	a
100009514	a	b	a	a	a	a	b	a
100063328	-	b	a	a	a	a	b	a
100048699	a	b	a	a	a	a	b	a
100076294	a	b	a	a	a	a	b	a
ks20640-B	a	b	a	a	a	a	b	a
100014228	a	b	a	-	a	a	b	a
100027451	a	b	a	a	a	a	b	a
100019182	a	b	a	a	a	a	b	a
100011380	a	-	a	a	a	a	b	a
100015166	a	b	a	a	a	a	b	a
100056005	a	b	a	a	a	a	b	a
100014914	-	b	a	a	a	a	b	a
100015546	a	b	a	a	a	a	b	a
100011988	a	b	a	a	a	a	b	a
100022182	a	b	a	a	a	a	b	a
100073479	a	b	a	a	a	a	b	a
100025951	a	b	-	a	a	a	b	a
100035292	a	b	a	a	a	a	b	a
100022670	a	b	a	a	a	a	b	a
100011299	a	b	a	a	a	a	b	a
100028364	a	b	a	a	a	a	b	a
100007539	-	b	a	a	a	a	b	a
100038090	a	b	a	a	a	a	b	a
100012880	-	b	a	a	a	a	b	a
100077635	a	b	a	a	a	a	b	a
100016097	a	b	-	a	a	a	b	a
100014992	a	b	a	a	-	a	b	a
100027960	b	b	a	a	a	a	b	a
100011009	a	b	a	a	-	a	b	a
100061647	b	a	a	a	a	b	b	a
100014469	-	a	a	a	a	b	b	a
100000155	b	a	-	a	a	b	b	a
Ni4A10	b	a	a	a	a	b	b	a
100016957	b	a	a	a	a	b	b	a
100022637	b	a	a	a	a	b	b	a
PBCESSRJU11	b	a	a	a	a	b	b	a
100021447	b	a	a	a	a	b	b	a
100019470	b	a	a	a	a	b	b	a
100077914	b	a	a	a	a	b	b	a
100011875	b	a	a	a	a	b	b	a

100077119	b	a	a	a	a	b	b	a
100024938	b	a	a	a	a	b	b	a
100014466	b	a	a	a	a	b	b	a
100052308	b	a	a	a	a	b	b	a
100030283	b	a	a	a	a	b	b	a
100017414	b	a	a	a	a	b	b	b
100029326	b	a	a	a	a	b	b	b
100042826	b	a	a	a	a	b	b	b
100005386	-	a	a	a	a	b	b	b
100020421	b	a	a	a	a	b	b	b
100041106	a	a	a	a	a	b	b	b
100061512	-	a	a	a	a	b	b	b
100037101	b	a	a	a	a	b	b	b
BRAS116	b	a	a	a	b	b	b	b
100013746	b	a	a	a	a	b	b	b
100001380	b	a	a	a	a	b	b	b
100025434	b	a	-	a	a	b	b	b
100075250	b	a	a	a	a	b	b	b
Ni2A09	b	a	a	a	a	b	b	b
100008486	b	b	a	a	a	b	b	b
BnGMS013	b	b	a	a	a	b	b	b
100069214	b	b	a	a	a	b	b	b
100040798	b	b	a	a	a	b	b	b
100031132	b	b	a	a	a	b	b	b
100004737	b	b	a	a	a	b	a	b
100042372	b	b	a	a	a	b	a	b
100022808	b	b	a	a	a	b	a	b
100026674	b	b	a	a	a	b	a	b
100008329	b	b	a	a	a	b	a	b
100017303	a	-	a	-	a	b	a	b
100006344	a	b	a	a	a	b	a	b
100078887	a	b	a	a	a	b	a	b
100023203	a	b	a	a	a	b	a	b
100023658	a	b	a	a	a	b	a	b
100017209	a	b	a	a	a	b	a	b
100033270	a	b	a	a	a	b	a	b
100037452	a	b	a	a	a	b	a	b
100029522	b	b	a	a	a	b	a	b
100011652	a	b	a	a	a	b	a	b
100047274	a	b	a	a	a	b	a	b
100015664	b	b	b	-	-	b	a	b
niab013	b	b	b	b	b	b	a	b
100022564	b	b	b	b	b	b	a	b
100011720	b	b	-	b	b	b	a	b
100029508	b	b	b	b	b	b	a	b
e6m3-220	b	b	b	b	a	b	a	b
100007094	b	b	-	b	a	b	a	b

100011937	b	b	b	b	a	b	a	b
100010320	b	b	b	b	a	b	a	b
100028246	b	b	b	b	a	b	a	b
100007647	b	b	b	b	a	b	a	b
100042745	b	b	b	b	a	b	a	b
100026299	b	b	b	b	a	b	a	b
100022847	b	b	b	b	a	b	a	b
100022959	b	b	b	-	a	b	a	b
100076031	b	b	b	-	a	b	a	b
100041944	b	b	b	b	a	b	a	b
100065423	b	b	b	b	-	b	a	b
100070200	b	b	b	-	a	b	a	b
100014341	b	b	b	-	a	b	a	b
100028912	b	b	b	a	a	b	a	b
100011531	b	b	b	a	a	b	a	b
100074174	b	b	b	a	a	b	a	b
100020829	b	b	b	a	a	b	a	b
100031276	b	b	b	a	a	b	a	b
100023650	b	b	b	a	a	b	a	b
100022091	b	b	b	a	a	b	a	b
100011424	b	b	b	a	a	b	a	b
100021449	b	b	b	-	-	a	a	b
100068765	b	b	b	a	a	a	a	b
100009934	b	b	b	a	a	a	a	b
100011722	b	b	b	a	a	a	a	b
BnGMS091	b	b	b	a	a	a	a	b
100033817	b	b	-	a	a	a	a	b
100038238	b	b	b	a	-	a	a	b
100057816	a	b	b	a	a	a	a	a
100074374	a	b	a	a	a	-	a	a
100045081	a	b	a	a	a	a	a	a
100011539	a	b	a	a	a	a	a	a
100023500	a	b	a	a	a	a	a	a
100052792	a	b	a	a	a	a	a	a
100065378	a	b	a	a	a	a	a	a
100066618	a	b	a	a	a	a	a	a
100029966	a	b	a	a	a	a	a	a
100056875	a	b	a	-	-	a	a	a
100030317	b	b	b	a	-	a	a	a
100053774	a	b	-	a	a	a	a	a
100007356	a	b	a	a	a	a	a	a
100021654	a	b	a	-	a	a	a	a
100068647	a	b	a	a	a	a	a	a
100068483	a	a	a	a	a	a	a	a
100002456	a	a	a	a	a	a	a	a
100001950	a	a	a	a	a	a	a	a
100062079	a	a	a	a	a	a	a	a

100007720	a	a	a	a	-	a	a	a
100045734	a	b	a	a	a	a	a	a
100075320	a	b	a	a	a	a	a	a
100055615	a	b	a	a	-	a	a	a
100027521	a	a	a	a	b	a	a	a
100037087	a	a	a	a	b	a	a	b
100023279	a	a	-	a	b	a	a	a
100064011	a	a	a	a	b	a	a	b
Ni2D08	a	a	a	a	b	a	a	b
100060279	a	-	a	a	b	a	a	b
100052894	a	a	b	a	b	a	a	b
100006932	a	a	a	a	b	a	a	b
100035548	b	a	a	a	b	a	a	b
100021620	a	a	a	-	b	a	a	b
100035017	a	a	a	a	b	a	a	b
100042788	a	a	a	a	b	a	a	b
100044445	a	a	a	a	b	a	a	b
100076763	a	a	a	a	b	a	a	-
100058331	a	a	a	a	b	a	a	b
100019767	a	a	a	a	b	a	a	b
100072282	a	a	a	a	-	a	a	b
100020968	a	a	a	a	b	a	a	b
100012485	a	-	-	a	b	a	a	b
100018102	a	a	a	a	b	a	a	b
100065962	-	a	a	a	b	a	a	b
100079097	a	a	a	a	b	a	a	b
100027560	a	a	a	-	b	a	a	b
100049168	a	a	a	a	b	a	a	b
100032315	a	a	a	a	b	a	a	b
100033169	-	b	a	a	b	a	a	b
100003585	a	a	a	a	b	a	a	b
100028078	a	a	a	a	b	a	a	b
100019234	a	a	-	a	b	a	a	b
100020285	a	a	a	-	b	a	a	b
100074919	a	a	-	a	b	-	a	b
100004668	a	a	a	a	b	a	a	b
100010967	a	a	a	a	b	a	a	b
100010952	a	-	a	a	b	a	a	b
e5m10-100	a	a	a	a	b	a	a	b
100074449	a	a	a	-	b	a	a	b
100032903	a	a	a	a	b	a	a	b
100038153	a	a	a	a	b	a	a	b
100056897	a	a	a	a	b	a	a	b
100015929	a	a	a	-	b	a	a	b
100005563	a	a	a	-	b	a	a	b
100059174	a	a	a	a	b	a	a	b
100057916	a	a	a	a	b	a	a	b

100022192	a	a	a	a	b	a	a	b
100020180	a	a	a	a	b	a	a	b
100043041	a	a	-	a	b	a	a	b
100017800	a	a	a	a	b	a	a	b
100037316	b	a	a	b	b	a	a	b
100070796	a	-	a	b	b	a	a	b
Ni3C08	a	a	a	b	b	a	a	b
100014913	a	a	-	b	b	a	a	b
100027568	a	a	a	b	a	b	a	b
100033200	a	a	a	b	b	b	a	b
KS20840	a	a	a	b	a	b	a	b
100025202	a	a	a	b	a	b	a	b
100073308	a	a	a	b	a	b	a	b
100026892	a	a	a	b	a	b	a	b
100007707	a	a	-	b	a	b	a	b
100020555	a	a	a	b	a	b	a	b
100020359	a	a	b	b	a	b	a	b
100022880	b	a	a	b	a	b	a	b
Na12E05	a	a	a	b	a	b	a	b
100017760	a	a	b	b	a	b	a	a
100036472	a	a	b	-	a	b	a	a
100008766	a	a	b	b	a	b	a	a
100020283	a	a	b	b	a	b	a	a
100015838	a	a	b	b	a	b	a	a
100039347	a	a	b	b	a	b	a	a
100015993	a	a	b	b	a	b	a	a
100067992	a	a	b	-	a	b	a	a
100067298	a	a	b	b	a	b	a	a
100025042	a	a	b	b	a	b	a	a
100009809	a	a	b	b	a	b	a	a
100018123	a	a	b	-	a	b	a	a
100079041	a	a	a	b	a	b	a	a
Pc	a	a	b	b	a	b	a	a
100022276	a	a	a	b	a	b	a	a
100054173	a	a	b	b	a	b	a	a
100054045	a	a	b	b	a	b	a	a
100031202	a	a	b	b	a	b	a	a
100075756	a	a	b	-	a	b	a	a
100075230	b	a	b	b	a	b	a	a
100001486	b	a	b	-	a	b	a	a
100018653	b	a	b	b	a	b	a	a
100047241	b	a	b	b	a	b	a	a
100004826	b	a	b	b	a	b	a	a
100007658	-	a	b	b	a	b	a	a
100018125	b	a	b	b	a	b	a	a
100029796	b	-	b	b	a	b	a	a
100063786	b	a	b	-	a	b	a	a

100002429	b	a	b	-	a	b	a	a
100019406	a	a	b	b	a	b	a	a
100019362	b	a	a	b	a	b	a	a
em13me19-550	b	a	b	b	a	b	a	a
100015803	b	a	b	b	a	b	a	a
100066297	b	a	b	-	a	b	a	a
100031644	b	a	a	b	a	b	a	a
100024244	b	a	b	b	a	b	a	a
100024197	-	a	-	b	a	b	a	a
100024396	b	-	b	b	a	b	a	a
100048523	b	a	b	b	a	b	a	a
BnGMS213	b	a	b	b	a	b	a	a
100024742	b	a	b	b	a	b	a	a
100045241	b	a	b	b	a	b	a	a
100006091	b	a	b	-	a	b	a	a
100043850	b	a	b	b	a	b	a	a
100078929	b	a	b	b	a	b	a	a
100067211	-	a	b	b	a	b	a	a
100021822	b	a	b	b	a	b	a	a
100070304	b	a	b	-	a	b	a	a
100018961	b	a	b	b	a	b	a	a
100013010	b	a	b	b	a	b	a	a
100027205	b	a	b	b	a	b	a	a
100055360	b	a	b	a	-	b	a	a
100005371	b	a	b	a	a	b	a	a
100012506	b	a	b	a	a	b	a	a
100027477	a	a	b	a	a	b	a	a
100066621	a	a	a	a	a	b	a	a
100016370	a	a	b	a	a	b	a	a
100019266	a	a	b	a	a	b	a	b
100030248	a	a	-	a	a	b	a	b
100030775	a	a	b	a	a	b	a	b
100025201	a	a	b	a	a	b	a	b
100025683	a	a	b	a	a	b	a	b
100014203	b	a	b	a	a	b	a	b
100012793	b	a	b	a	a	b	a	b
100048347	b	a	b	a	a	b	a	b
e6m9-80	b	a	b	a	a	b	a	b
100026006	b	a	b	a	a	b	a	b
100025622	b	a	b	a	a	b	a	b
100011867	b	a	b	a	a	b	a	b
100057157	a	a	b	a	a	b	a	b
100049162	b	b	b	a	a	b	a	b
100077435	b	b	b	a	a	b	a	b
100050354	b	b	b	a	b	b	a	b
100012372	-	b	-	a	b	b	a	b
100023256	a	b	b	a	a	b	a	b

100074521	a	b	b	a	a	b	a	b
100013001	a	b	a	a	a	b	a	b
100009827	a	b	b	a	a	b	a	b
100024947	b	b	b	a	a	b	a	b
100029954	a	-	b	a	a	b	a	b
100021199	a	b	b	a	a	b	a	b
100021508	a	b	b	a	a	b	a	b
Ni2E05	b	b	b	a	a	b	a	b
100017284	a	b	b	a	a	b	a	b
100037214	a	b	b	a	a	b	a	b
100022699	a	b	b	a	a	b	a	b
100007659	-	a	b	b	b	b	a	a
100016958	b	a	b	b	b	b	a	a
100038041	b	a	b	b	b	b	a	a
100059045	b	a	a	b	b	b	a	a
e5m10-150	b	a	b	b	b	b	a	a
100007671	b	a	a	b	b	b	a	a
100021203	b	a	b	b	b	b	a	a
100017886	b	a	b	-	b	b	a	a
100031742	b	a	b	b	b	b	a	a
100053349	b	a	b	b	b	b	a	a
100018506	a	a	b	-	b	b	a	a
100037601	a	a	b	b	b	b	a	a
100015346	a	a	b	b	b	b	a	a
100006506	a	a	b	b	b	b	a	a
100008476	a	a	b	b	b	b	a	a
100063129	-	a	b	b	b	b	a	a
100017676	a	a	a	b	b	b	a	a
100023051	a	a	b	b	b	b	a	a
100015321	a	a	b	b	b	b	a	a
100049231	a	a	b	-	b	b	a	a
100014480	a	a	b	-	b	b	a	a
100032507	a	a	b	b	b	b	a	a
100047463	a	a	b	-	b	b	a	a
100068721	-	a	b	b	b	b	a	a
100049395	a	a	-	b	b	b	a	a
100061126	a	a	a	b	b	b	a	a
100005111	a	a	a	b	b	b	a	a
100037967	a	a	a	b	b	b	a	a
100006764	-	a	a	b	b	b	a	a
100051178	a	a	a	b	b	b	a	a
100012163	a	-	a	b	b	b	a	a
100019422	a	a	-	b	b	b	a	a
100051571	a	a	a	b	b	b	a	a
em1me30-250	a	a	a	b	b	b	a	a
100071346	-	-	a	b	b	b	a	a
100066523	a	a	a	b	b	b	a	a

100015887	a	a	a	b	b	b	a	a
100006934	a	a	a	b	b	b	a	a
100058090	a	a	a	b	-	b	a	a
100055593	a	a	a	b	b	b	a	a
100032410	-	a	a	b	b	b	a	a
100059607	a	a	a	b	a	b	a	a
100049714	a	a	a	b	a	b	a	a
100010398	a	a	a	b	a	b	a	a
100012533	a	a	a	b	a	b	a	a
100053100	a	a	a	b	a	b	a	a
100069322	b	a	a	-	a	b	a	a
100023110	a	a	-	a	a	b	a	a
100020332	a	a	a	b	a	b	a	a
100019214	a	a	a	b	a	b	a	a
100007482	a	a	-	-	a	b	a	a
100057070	a	a	a	b	a	b	a	a
100036978	a	a	a	-	a	b	a	a
100033366	a	-	b	b	-	b	a	a
100020485	a	a	a	b	b	b	a	a
100010187	a	a	a	b	-	b	a	a
100049303	a	a	a	b	b	b	a	a
100037068	a	a	a	b	b	b	a	a
Ni4F09	a	a	a	b	b	b	a	a
100019505	a	a	a	-	b	b	a	a
100007683	a	a	a	b	b	b	a	a
100077998	a	a	a	b	b	b	a	a
100031234	a	a	a	b	b	b	a	a
100008064	a	a	a	b	b	b	a	a
100072161	a	a	a	b	-	b	a	a
100042030	a	-	a	b	b	b	a	a
100047311	a	a	a	-	b	b	a	a
100008060	a	a	a	b	b	b	a	a
100036414	a	a	a	b	b	b	a	a
100013867	a	a	a	b	b	b	b	b
100025623	a	a	a	b	b	b	b	b
100008558	a	a	a	b	b	b	b	b
100036541	a	a	a	b	b	b	b	b
100018762	a	a	-	b	b	a	b	b
100015060	a	-	a	b	b	a	b	b
e5m9-200	a	a	a	b	b	a	b	b
e3m3-250	a	a	a	b	b	a	b	b
Na12C01	a	a	a	b	b	a	b	b
100014967	a	a	a	b	b	a	b	b
100027006	b	a	-	b	b	a	b	b
100073744	a	a	a	-	b	a	b	b
e6m9-250	a	a	a	b	b	a	b	b
100005456	a	a	a	b	b	a	b	b

100062790	a	a	a	-	b	a	b	b
PBCESSRJU8-A	a	a	a	b	b	a	b	b
100045717	b	a	a	b	b	a	b	b
100020458	b	a	a	b	b	a	b	b
100018494	b	a	b	b	b	a	b	b
100028847	b	a	a	b	b	a	b	b
100030569	b	a	a	b	b	a	b	b
100027570	b	a	a	b	b	a	b	b
100052281	b	a	a	b	b	a	b	b
100022962	b	a	a	b	-	a	b	b
100007278	b	a	b	-	b	a	b	b
100046407	b	a	-	b	b	a	b	b
100015958	b	a	b	a	b	a	b	b
100030337	-	a	b	a	b	a	b	b
100027812	b	a	a	a	b	a	b	b
100068882	b	a	b	a	b	a	b	b
100050410	b	a	b	-	b	a	b	b
100015241	b	a	b	a	b	a	b	b
100052506	b	a	b	a	b	a	b	b
100025223	b	a	b	a	b	a	b	b
100039722	b	a	b	-	b	a	b	b
100023163	b	a	b	a	b	a	b	b
100079009	a	-	a	-	a	b	a	b
100028870	-	b	a	-	a	b	a	b
100016304	a	b	a	a	a	b	a	b
100073662	a	b	a	a	a	b	a	b
100058792	a	b	a	a	a	b	a	b
100067615	a	b	-	a	a	b	a	b
100009089	a	b	a	a	a	b	a	b
100046847	a	b	a	a	a	b	a	b
100065428	b	b	a	a	a	b	a	b
100004158	a	b	a	a	a	a	a	b
100021631	a	b	a	a	a	a	a	b
100036555	a	b	a	a	a	a	a	b
100025417	a	b	b	a	a	a	a	b
100062911	a	b	b	a	-	a	a	b
100004474	a	b	-	a	a	a	a	b
100010335	a	b	b	a	a	a	a	b
100005174	a	b	b	a	a	a	a	b
100029926	a	b	b	-	a	a	a	b
100021125	a	b	b	a	a	a	a	b
100011688	a	b	b	a	a	a	a	b
100017191	a	b	b	a	b	a	a	b
100078223	a	-	-	a	b	a	a	b
100024199	a	b	b	a	b	a	a	b
100015907	a	b	b	a	b	a	a	b
100064622	a	b	b	a	b	a	a	b

100033238	a	b	b	a	b	a	a	b
100033088	a	b	b	a	b	a	a	b
100019209	a	b	b	a	b	a	a	b
100043699	a	b	b	a	b	a	a	b
100028536	a	b	b	a	b	a	a	b
100033072	a	b	b	a	b	a	a	b
100014607	a	b	a	a	b	a	a	b
100034017	a	b	-	-	b	b	a	b
100033564	a	-	b	a	-	b	a	b
100007738	a	b	b	a	b	b	a	b
100030187	a	b	b	a	b	b	a	b
100014832	-	b	b	a	b	b	a	b
100020741	a	b	b	b	b	b	a	b
100017376	a	b	b	b	-	b	a	a
CB10278	a	b	b	b	b	b	a	a
e5m9-150	a	b	b	b	b	b	a	a
100005031	a	b	b	b	b	b	a	a
100030316	-	b	b	b	b	b	a	a
100039552	a	b	b	b	b	b	a	a
100018753	a	b	b	b	-	b	a	a
A8-BAC13	a	b	b	b	b	b	a	a
100035942	-	b	b	b	b	b	a	a
100015255	-	b	b	b	b	b	a	a
100021151	a	b	b	b	b	b	a	a
100059951	a	b	b	b	b	b	a	a
100011210	a	-	-	b	b	b	a	a
100053506	b	b	b	b	b	b	a	a
100061890	a	b	b	b	b	b	a	a
fito361	a	b	b	b	b	b	a	a
100002468	a	b	b	b	b	b	a	a
100055875	a	b	b	b	b	b	a	a
100052047	a	b	b	b	b	b	a	a
100023396	a	b	b	-	b	b	a	a
100019274	a	b	b	b	b	b	a	a
100025883	a	b	b	b	b	b	a	a
100018326	-	b	b	b	b	b	a	a
100013920	a	b	b	b	b	b	a	a
100024301	a	b	b	b	b	b	a	a
100067960	a	b	b	b	b	b	a	a
100031789	a	b	b	b	b	b	a	a
100019364	a	b	b	b	b	b	a	a
100019770	a	b	b	b	b	b	a	a
100076228	a	b	-	b	b	a	a	a
100028417	a	-	-	b	b	a	a	a
100030404	a	b	b	b	b	a	a	a
100071863	-	b	b	b	b	a	a	a
100031473	a	b	b	b	b	a	a	a

100056502	-	a	b	b	b	a	a	a
100034989	a	a	b	b	b	a	a	a
100015084	a	a	b	b	b	a	a	a
100006783	a	a	b	b	b	a	a	a
100030184	a	a	b	b	b	a	a	a
100033667	-	a	b	b	b	a	a	a
100035893	a	-	b	b	b	a	a	a
100056071	a	-	b	b	b	a	a	a
100055771	a	a	b	b	b	a	a	a
100037990	a	a	b	b	b	a	a	a
100013250	b	a	-	b	b	a	a	a
100012921	b	a	b	b	b	a	a	a
100053548	b	a	b	b	b	a	a	a
100066360	b	a	b	b	b	a	a	a
100060834	b	-	b	b	b	a	a	a
100061759	b	a	b	b	b	a	a	a
100007358	b	a	b	b	b	a	a	a
100036105	b	a	b	b	b	a	a	a
100062862	b	a	b	b	b	a	a	a
100008811	b	a	b	b	b	a	a	a
100033395	b	a	b	b	b	a	a	a
100070790	b	a	b	b	b	a	a	a
100033803	b	a	b	b	b	a	a	a
NA12H07	b	a	b	b	b	a	a	a
100047102	b	a	b	b	b	a	a	a
100031592	b	a	b	b	b	a	a	a
100020984	b	a	b	-	b	a	a	a
100017911	b	a	b	b	b	a	a	a
100005762	b	a	b	b	b	a	a	a
100022351	b	a	b	b	b	a	a	a
100015389	b	a	b	-	b	a	a	a
100039342	b	a	b	b	b	a	a	a
100044229	b	a	b	b	b	a	a	a
HAU61	b	a	b	b	b	a	a	a
100065908	b	a	b	b	b	a	a	a
100038821	b	a	b	a	b	a	a	a
100072429	b	a	b	-	b	a	a	a
100011123	b	a	b	-	b	a	a	a
100015262	b	a	b	a	b	a	a	a
100021279	b	a	b	a	b	a	a	a
100018709	b	a	b	a	b	a	b	a
100021088	b	a	-	a	b	a	b	a
100021683	b	a	-	a	b	a	a	a
100033412	b	-	b	-	b	a	b	a
100044738	b	-	b	a	b	a	b	a
100061546	b	a	b	-	b	a	b	a
100042137	b	a	-	a	b	a	b	a

100044338	-	a	b	a	b	a	b	a
100051017	a	a	b	a	b	a	b	a
Ni4H04	a	a	b	a	b	a	b	a
niab113	-	b	a	b	a	b	b	a
100019218	b	b	a	b	a	b	b	a
100011545	b	b	-	b	b	a	a	a
CB10587	b	b	a	b	b	a	a	a
100011069	b	-	a	b	b	a	a	a
100004218	b	b	a	b	b	a	a	a
100010016	b	b	a	b	b	a	a	a
100014201	b	b	a	b	b	a	a	a
100064031	-	b	a	b	b	a	a	a
100078611	b	b	a	b	b	a	a	a
100026214	b	-	a	b	b	a	a	a
100004960	b	b	a	b	b	a	a	a
100026062	b	b	a	b	b	a	a	a
BnGMS277-A	b	b	a	b	b	a	a	a
100018611	b	b	a	b	b	a	a	a
100044915	b	b	-	b	b	a	a	a
100063630	b	b	a	b	b	a	a	a
100015932	b	b	a	b	b	a	a	a
100063706	b	b	a	a	b	a	a	a
100052180	b	b	a	b	b	a	a	a
100018514	b	b	a	b	b	a	a	a
100060944	b	b	a	b	b	a	a	a
e2m10-380	b	b	a	b	b	a	a	a
100060450	b	b	a	b	b	a	a	a
100016520	a	b	a	b	b	a	a	a
100003472	a	b	a	b	b	a	a	a
e2m5-400	a	b	a	b	b	a	a	a
e5m10-290	a	b	a	b	b	a	a	a
100068682	a	a	a	b	b	a	a	a
SA39	a	a	a	b	b	a	a	a
100015028	a	a	a	b	b	a	a	a
SA54	a	a	a	b	b	a	a	a
100025702	-	a	-	b	b	a	a	a
100018249	a	a	a	-	b	a	a	a
Na10H06	a	a	a	b	b	a	a	b
100031795	a	a	a	a	b	b	-	b
100062611	a	a	a	a	b	b	a	b
100059879	-	-	a	a	a	b	a	b
100016232	a	a	a	a	a	b	a	b
100020118	a	-	a	a	a	b	a	b
100017613	a	a	b	a	a	b	a	b
100024486	a	a	b	a	a	b	a	b
100008983	a	a	b	a	a	b	a	b
100056536	a	a	b	a	a	b	a	b

100064206	a	a	b	a	a	b	a	b
100048162	a	a	b	a	a	b	a	b
100010271	-	a	b	a	a	b	a	b
Ni4B10	a	a	b	a	a	b	a	b
100018715	b	a	b	a	b	a	b	b
100025181	b	a	-	a	-	a	b	b
100055259	b	a	b	a	b	a	b	b
100027402	b	a	b	-	b	a	b	b
100046502	-	a	b	-	b	a	b	b
100072642	b	a	b	a	b	a	b	b
100064473	-	a	b	a	b	a	b	b
100020028	b	a	b	a	-	a	b	b
100071010	b	a	b	a	b	a	a	b
100068200	b	b	b	a	b	a	a	b
100007974	b	b	b	-	b	a	a	b
100008592	b	b	b	a	b	a	a	b
niab124	b	b	b	a	b	a	a	b
100054557	b	b	b	a	b	a	a	b
100049100	b	b	b	a	b	a	a	b
100014818	b	b	b	-	-	a	a	b
100057798	b	b	b	-	b	a	a	b
100037219	b	b	b	a	b	a	a	b
100028432	b	b	b	a	b	a	a	b
100013654	b	b	b	a	b	a	a	b
BnGMS345	b	b	b	a	b	a	a	b
100005075	b	b	b	b	b	a	a	b
100029810	b	b	b	b	b	a	a	b
100015422	b	b	b	b	b	a	a	b
100051900	a	b	b	b	-	a	a	b
100051280	a	b	b	b	b	a	a	b
e4m8-300	a	b	b	b	b	a	a	b
SA12	a	b	b	b	b	a	a	b
B027E01-2A	b	b	b	b	b	a	b	b
100063199	b	b	b	-	b	a	b	b
100040002	b	b	b	b	b	a	b	b
100011677	-	b	-	-	b	a	b	b
100024946	b	b	b	b	b	a	b	b
100015155	b	-	b	b	b	a	b	b
100047227	b	b	b	b	b	a	b	b
100061522	b	b	b	b	b	a	b	b
Ol13E08	b	b	b	b	b	a	b	b
100007874	b	b	b	b	b	a	b	b
fito019	b	b	b	b	b	b	b	b
100037636	b	b	-	-	b	a	b	b
100041321	b	b	b	b	b	a	b	a
100013753	b	b	b	b	b	b	a	b
100029139	b	b	b	b	b	b	a	b

100021460	b	b	b	b	b	b	a	b
100031168	-	b	b	b	b	b	a	b
100013435	b	b	b	b	b	b	a	b
BnGMS426	b	b	b	b	b	b	a	b
100020221	b	b	b	b	b	b	a	b
100032757	b	a	b	b	b	b	a	b
100017735	b	a	b	b	b	b	a	b
100050910	a	a	b	b	b	b	a	b
100014808	-	a	b	b	b	b	a	b
100015635	b	a	b	b	b	b	a	b
100062181	b	a	b	b	b	b	a	b
100020169	b	a	-	b	b	b	a	b
100029700	-	a	b	b	b	b	a	b
BRMS002	b	a	b	b	b	b	a	b
100002930	b	a	b	b	b	b	a	a
100037831	b	-	b	b	b	b	b	a
100026533	b	a	b	b	b	b	b	a
100036885	b	a	a	b	b	b	b	a
100039504	b	a	a	b	b	b	b	a
100028404	b	a	a	b	b	b	b	a
100053345	b	a	a	b	b	b	b	a
100027338	b	a	a	b	b	b	b	a
100038513	b	a	a	b	b	b	b	a
100023202	b	a	a	b	b	b	b	a
100024943	b	a	-	b	b	b	b	a
100023245	-	a	a	b	-	b	b	a
HAU23	b	a	a	b	b	b	b	a
cnu208	b	a	a	b	b	b	b	a
100011923	b	a	a	b	b	b	b	a
100012299	b	-	a	b	-	b	b	a
100008551	b	a	a	b	b	b	b	a
100061627	b	a	a	-	b	b	b	a
100026762	b	a	a	b	b	b	b	a
100055174	a	a	a	b	b	b	b	b
100042637	b	a	a	b	b	b	b	b
100014532	-	a	a	b	b	b	b	b
100043219	b	-	a	b	b	b	b	b
100025390	b	a	-	b	b	b	b	b
100010953	b	a	a	b	b	b	b	b
100025118	b	a	a	b	a	b	b	b
100018700	b	a	a	b	a	b	b	b
100050829	b	a	a	b	a	b	b	b
100079205	b	a	a	b	a	b	b	b
100074355	b	-	a	-	a	b	b	b
100065534	b	a	a	b	-	b	b	b
100068795	-	b	a	b	a	b	a	b
100049018	b	b	a	b	a	b	a	b

100012667	b	b	a	b	a	b	a	b
100034883	b	b	a	b	a	b	a	b
100042445	b	b	a	b	a	b	a	b
100049366	b	b	a	b	a	b	a	b
100012746	b	b	a	-	a	b	a	b
PBCGSSRBo38	b	b	a	b	a	b	a	b
100040377	b	b	a	b	a	b	a	b
100034569	b	b	a	b	a	b	a	b
100052320	-	b	a	b	a	b	a	b
100016849	b	b	a	b	a	b	a	b
100024449	b	b	a	b	a	b	a	b
CB10403	b	b	a	b	a	b	a	b
100069845	b	b	a	b	a	b	a	b
100031828	b	a	a	b	a	b	a	b
100014999	b	b	a	b	a	b	a	b
100022688	b	b	a	a	a	b	a	b
100022642	b	b	a	a	a	b	a	b
100006416	b	b	a	a	a	b	a	b
100056247	b	b	-	a	a	a	a	b
100052674	b	b	a	a	a	a	a	b
100055560	b	b	a	-	a	a	a	b
100015711	b	b	a	a	a	a	a	b
100014190	b	b	-	a	a	a	a	b
100010575	b	b	a	a	a	a	a	b
100070490	b	b	a	a	a	a	a	b
100022415	b	b	a	a	-	a	a	b
100042386	b	b	-	a	a	a	a	b
H015H17-1	b	b	a	a	a	a	a	b
100015854	a	-	a	a	a	a	a	b
100027868	b	b	a	a	a	a	a	b
100005398	b	b	a	a	a	a	a	a
100026334	-	b	a	a	a	a	a	a
100014090	b	b	a	a	a	a	a	a
100073972	b	b	b	a	a	a	a	a
100015636	-	b	b	-	a	a	a	a
Ol10B04	b	b	b	a	a	a	a	a
100076711	b	b	b	a	a	a	a	a
PBCGSSRBo14	b	b	b	a	a	a	a	a
100066748	b	b	b	a	a	a	a	a
100010996	b	b	b	a	a	a	a	a
100010157	b	b	b	a	a	a	a	a
100010185	b	b	b	a	a	a	a	a
100059493	b	b	b	a	b	a	a	a
100068873	a	b	a	a	b	a	a	a
100019306	b	b	b	a	b	a	a	a
100028512	b	b	b	a	b	a	a	a
100058609	b	b	b	a	b	a	a	a

100021092	b	b	b	a	b	a	a	a
100030726	-	-	-	a	b	a	a	a
100008367	-	b	b	a	b	a	a	a
100064710	b	-	b	a	b	b	a	a
100040264	b	b	b	-	b	b	a	a
100061792	b	b	b	-	b	b	a	a
100049050	a	b	b	a	b	b	a	a
100039080	b	b	-	a	b	b	a	a
100024089	a	a	b	b	b	a	b	a
100021627	a	a	b	b	b	a	b	a
OL11H02	a	a	b	b	b	a	b	a
Ol11D12	a	a	b	b	b	a	b	a
100008104	a	a	b	b	b	a	b	a
100029133	a	a	b	b	b	a	b	a
100021516	a	a	a	b	b	a	b	a
100026640	a	a	b	b	b	a	b	a
100019865	a	a	b	b	b	a	b	a
100058814	-	a	b	b	b	a	b	a
100027194	a	a	b	b	b	a	b	a
ks20640-A	a	a	b	b	a	a	b	a
100032039	a	a	b	a	a	a	b	a
100035805	a	a	-	b	a	a	b	a
100022173	a	a	a	b	b	a	b	a
100012629	a	b	b	-	a	a	b	b
100055466	a	b	b	b	a	a	b	b
100012771	a	b	a	a	a	a	b	b
100043087	a	b	a	a	a	a	b	b
100022579	a	b	a	a	a	a	b	b
niab021	a	b	a	a	a	a	b	b
100008940	a	b	a	a	a	a	b	b
Ni2E07	a	b	a	a	a	a	b	b
100007868	a	b	a	a	a	a	b	b
100013195	a	b	a	a	a	a	b	b
100044151	a	b	a	a	a	a	b	b
100022727	a	-	a	a	a	a	b	b
100040086	a	b	a	a	a	a	b	b
100029056	a	-	a	a	a	a	b	b
NA12E06A	a	b	a	a	a	a	b	b
100024625	a	b	a	a	a	a	b	b
100016523	a	b	a	a	a	a	b	b
100074863	a	b	a	a	a	a	b	b
100023611	a	b	a	a	a	a	b	b
100059630	a	b	a	a	a	a	b	b
100029356	a	b	a	-	a	a	b	b
100009621	a	b	a	b	a	a	b	b
100025551	-	b	a	b	a	a	b	b
100021474	-	b	a	b	a	a	b	b

100048982	a	b	a	b	a	a	b	b
100016930	a	b	a	-	a	a	b	b
100015148	a	b	b	-	a	a	b	b
100037486	a	b	-	-	a	a	b	b
BRAS063	a	b	b	b	a	a	b	b
100026342	a	b	b	b	a	a	b	b
100036778	a	b	b	-	a	a	b	b
100065508	a	b	b	b	a	a	b	b
100078970	a	b	b	b	a	a	b	b
100019506	a	b	b	b	a	a	b	b
100023632	a	b	b	b	a	a	b	b
100026061	a	b	b	b	a	a	b	b
100016245	a	b	a	b	b	b	a	b
100007983	a	b	-	b	b	b	a	b
BnGMS334	a	b	a	b	b	b	a	b
100069690	a	b	a	b	b	b	a	b
BnGMS027	a	b	a	b	b	b	a	b
100006256	a	b	a	b	b	b	a	b
100041974	a	b	a	b	b	b	a	b
100013309	a	b	a	b	b	b	a	b
100034032	a	b	a	b	b	b	a	b
100022700	b	b	a	b	b	b	a	b
100038782	a	b	a	b	b	b	a	b
BnGMS292	a	b	b	b	b	b	a	b
100013233	a	b	b	b	b	b	a	b
100018683	a	b	b	b	b	b	a	b
100047046	a	b	b	b	b	b	a	b
BnGMS256	a	a	b	b	a	b	a	b
100009644	a	a	b	b	a	b	a	b
CB10065	a	a	b	b	a	b	a	b
100078636	a	a	a	b	a	b	a	b
100065201	a	a	b	b	a	b	a	b
100035903	a	a	b	b	a	b	a	b
100018642	a	a	b	b	a	b	a	b
100012535	a	a	b	b	a	b	a	b
100037609	a	a	b	b	a	a	a	b
100016951	-	a	b	b	a	a	a	b
100057078	a	a	b	-	a	a	a	b
100034396	a	a	b	b	a	a	a	b
100006019	a	a	b	b	a	a	a	b
MR113	a	a	b	b	a	b	a	b
100044388	b	a	b	b	a	b	a	b
sS2131	b	a	b	b	a	b	a	b
Na10D11	b	a	b	b	a	b	a	b
Ol10A10	a	a	b	b	a	b	a	b
100030993	b	a	b	-	a	b	a	b
100017632	b	a	b	b	a	b	a	b

100021467	b	a	b	-	a	b	a	b
100059378	b	a	b	b	a	b	a	b
100016364	b	a	b	b	a	b	a	b
100026428	b	-	b	b	a	b	a	b
100067924	-	a	-	-	a	b	a	a
100013730	b	a	b	a	a	b	a	a
100028725	b	a	b	a	a	b	a	a
100008603	b	a	b	a	a	b	a	a
100057725	b	b	a	b	a	b	b	a
100034215	b	b	a	b	a	b	b	a
100007607	b	b	a	-	a	b	b	a
100032036	b	-	a	b	a	b	b	a
100029429	b	a	a	b	a	b	b	a
100030519	a	a	a	b	a	b	b	a
100027403	b	a	a	b	a	b	b	a
100024804	b	a	a	b	-	b	b	a
100037438	b	a	a	b	a	b	b	a
100062752	b	a	a	b	a	b	b	a
100013331	b	a	a	b	a	b	b	a
100078804	b	a	a	b	a	b	b	a
BnGMS323	b	a	a	b	a	b	b	a
100036823	b	a	a	b	a	b	b	a
100041764	b	a	a	b	a	b	b	a
100037463	b	a	a	b	a	b	b	a
100033606	a	a	a	b	a	b	b	a
100073913	-	a	a	b	a	b	b	a
100019630	a	a	a	b	a	b	b	a
100020122	a	a	a	b	b	b	b	a
100012727	a	a	a	b	b	b	b	a
100028996	a	a	b	b	a	b	a	a
100009082	a	a	-	b	a	b	a	a
100061440	a	a	a	b	a	b	a	a
100017755	a	a	a	b	a	b	a	a
HG-FT-C6a	a	a	a	b	b	a	a	a
100020694	a	a	a	b	a	b	a	a
100040545	a	a	a	b	a	b	a	a
100064230	a	a	-	b	a	b	a	a
100050944	a	a	a	b	a	b	a	a
100028305	a	-	a	b	a	b	a	a
100026430	a	a	a	b	a	b	a	a
100035118	a	a	a	b	a	b	a	a
100031328	a	a	a	b	a	b	a	a
100015500	a	a	a	b	a	a	a	a
100023693	-	a	a	b	a	a	a	a
100056481	a	a	a	b	a	a	a	a
100033089	a	a	a	b	a	a	a	a
100040008	a	a	a	b	b	a	a	a

100012448	a	a	a	b	b	a	a	a
BRAS004	a	a	a	b	b	a	a	a
e2m5-320	a	a	a	b	b	a	a	a
100008810	a	a	a	b	b	a	a	a
CB10439	a	a	a	b	b	a	a	a
100021971	a	a	a	b	b	a	a	a
Na12E01	a	a	a	b	b	a	a	a
100037880	a	a	a	-	b	a	a	a
CB10234	a	a	a	b	b	a	a	a
100035726	a	a	a	b	b	a	a	a
100028657	a	a	a	b	b	a	a	a
100020680	a	a	a	b	b	a	a	a
SA60	a	a	a	b	b	a	a	a
100023982	a	a	-	b	b	a	a	a
100023158	a	a	a	b	b	a	a	a
100008675	a	a	b	b	b	a	a	a
100035303	a	a	b	b	b	a	a	a
BnGMS567	a	a	b	b	b	a	a	a
100032948	a	a	-	a	a	a	a	a
100018304	a	a	b	a	a	a	a	a
100051548	b	a	a	b	b	a	b	a
100019616	b	a	a	b	b	a	b	a
100021982	b	a	a	b	b	a	b	a
em1me30-300	b	a	a	a	b	a	b	a
100026256	b	a	a	b	b	a	b	a
100028050	b	-	a	b	b	a	b	a
100027577	b	a	a	b	b	a	b	a
BnGMS392	b	a	a	b	b	a	b	a
100012656	b	-	b	b	b	a	b	a
B089G06-3	b	a	b	a	b	a	b	a
100015457	-	a	-	b	b	a	b	a
CB10413	b	a	b	b	b	a	b	a
100005592	b	-	b	b	b	a	b	a
BnGMS508	b	a	b	b	b	a	b	a
100013999	b	a	b	b	b	a	b	a
100051078	-	a	b	b	b	a	b	a
100065439	b	-	b	b	b	a	b	a
100004847	b	a	b	-	b	a	b	a
100011061	-	a	b	a	-	a	b	a
100038749	b	a	b	-	b	a	b	a
BnGMS277-B	b	a	b	a	b	a	b	a
100024563	a	a	b	a	b	a	b	a
100034961	a	a	b	a	b	a	b	a
100071984	b	a	a	a	b	a	b	a
BnGMS357	b	a	b	a	b	a	b	a
100034293	b	a	b	a	b	a	b	a
cnu371	b	a	b	a	b	a	b	a

100078147	b	a	b	a	b	a	b	a
100068546	b	a	-	a	b	a	b	a
100046712	b	a	-	a	b	a	b	a
100022249	b	a	-	a	b	a	b	a
100040390	b	a	b	a	b	a	b	a
100018746	-	a	-	a	b	a	b	a
100033605	b	a	b	-	b	a	b	a
100046587	b	a	b	a	b	a	b	b
PBCGSSRBo36	b	a	b	a	b	a	b	b
100054865	b	a	-	a	b	a	b	b
100048778	b	a	b	b	b	a	b	b
100028349	b	a	b	a	b	a	b	b
100053583	a	a	b	a	b	a	b	b
100028791	-	a	b	a	b	a	b	b
100072240	a	a	b	a	b	a	b	b
100006664	a	a	b	-	b	a	b	b
100009750	a	a	b	a	b	a	b	b
100030964	a	a	b	b	b	a	b	b
100061207	-	a	b	b	b	a	b	b
100037708	a	a	b	b	b	a	b	b
100065291	a	a	b	b	b	a	b	b
100062030	a	a	-	b	b	a	b	b
100019077	a	a	b	b	b	a	b	b
100034282	a	a	b	b	b	a	b	b
100024192	a	a	b	b	b	a	b	b
100020742	a	a	-	b	b	b	b	b
100003285	a	a	b	b	b	b	b	b
100038163	a	a	b	b	b	b	b	b
100013825	a	a	b	a	b	b	b	b
100025920	a	a	b	a	b	b	b	b
100013962	b	a	b	a	b	b	b	b
100040526	a	a	b	a	b	b	b	b
100048720	a	a	b	a	b	b	b	b
100007433	a	a	b	-	b	b	b	b
100037859	a	-	b	a	b	b	b	b
e6m3-180	a	b	b	a	a	b	a	b
100017146	-	b	b	a	a	b	a	b
100039765	a	b	-	a	a	b	a	b
100014017	a	b	a	a	a	b	a	b
100055365	a	b	b	a	a	b	a	b
100014401	a	b	b	a	a	b	a	b
SA69	a	b	b	a	a	b	a	b
100024334	a	b	b	a	a	b	a	b
100029984	a	b	b	a	a	b	a	b
SA06	a	a	b	a	a	b	a	b
100011973	a	a	b	a	a	b	a	b
100031848	a	a	b	a	a	b	a	b

100053216	a	a	a	a	a	b	a	b
100015981	a	a	b	a	a	b	a	b
100021875	a	a	b	a	a	b	a	b
100036359	a	a	-	a	a	b	a	b
100062727	a	a	b	a	a	b	a	b
100075300	a	a	b	a	a	b	a	b
100023350	a	a	-	a	a	b	a	b
100010968	a	a	b	a	a	b	a	b
100045208	a	a	-	a	a	b	a	b
100019441	a	a	b	a	a	b	a	b
100015313	a	-	b	a	a	b	a	b
100030360	a	a	b	a	a	b	a	b
100065251	a	a	b	a	-	b	a	b
100012490	a	a	b	a	a	b	a	b
100026947	a	a	b	a	a	b	a	b
em6me26-450	a	a	b	a	a	b	a	b
100075461	-	a	b	a	a	b	a	b
100022161	a	a	-	a	a	b	a	b
100027474	a	a	b	a	a	b	a	b
100032715	a	a	-	a	a	b	a	b
100011644	a	a	b	a	a	b	a	b
100029043	a	a	b	a	a	b	a	b
100006162	a	a	b	b	a	b	a	b
100028825	a	a	b	b	a	b	a	b
100009857	a	a	b	-	a	b	a	b
100029655	a	a	a	b	a	b	a	b
100009196	-	-	b	b	a	b	a	b
100040100	a	a	-	b	a	b	a	b
100001609	a	a	b	a	a	b	a	b
100008240	a	a	b	a	a	b	a	b
100042506	a	-	b	a	a	b	a	b
100041581	a	a	b	a	a	b	a	b
100043358	a	a	b	a	a	b	a	b
100038688	a	a	b	a	a	b	a	b
H010P01-2	a	a	b	a	a	b	a	b
100003965	a	a	b	a	a	b	a	b
CB10028	a	a	b	a	a	b	a	b
CB10092-B	a	a	b	a	a	b	a	b
100010513	a	-	b	a	a	b	a	b
100053806	a	-	b	a	a	b	a	b
100069841	a	a	b	a	a	b	a	b
100063523	a	a	b	a	a	b	a	b
100032920	a	a	b	a	a	b	a	b
e2m3-290	a	a	b	a	a	b	a	b
BnGMS148	a	a	b	a	a	b	a	b
100009976	a	a	b	a	a	b	a	b
100020225S	b	b	b	b	a	a	a	b

3078270S	b	b	b	b	a	a	a	b
BnGMS679	b	b	b	b	a	a	a	b
100034307	b	b	b	b	a	a	a	b
100015277	b	b	b	b	a	a	a	b
100005670	b	b	b	-	a	a	a	b
100054209	b	b	b	a	a	a	a	b
3077513S	b	b	b	a	a	a	a	b
H125N23-1	b	a	b	a	a	a	a	b
100012123	b	a	b	a	a	a	a	b
100015972	b	a	b	a	a	a	a	b
100060680	b	a	b	a	a	a	a	b
100028894	b	a	b	a	a	a	a	b
100017422	b	a	b	a	a	a	a	b
100005758	b	a	b	a	a	a	a	b
100050041	b	a	b	a	a	a	a	b
100079100	b	a	b	a	a	a	a	b
3135099S	b	a	b	a	a	a	a	b
Ol10D08	b	a	b	a	a	a	a	b
100040281S	a	a	b	a	a	a	a	b
100062459	a	a	-	a	a	a	a	b
100074931	a	a	a	a	a	a	a	b
100064959	a	a	a	a	a	a	a	b
100024455	a	a	a	a	a	a	a	b
100073939	a	a	a	a	a	a	a	b
100066781	a	a	a	a	a	a	a	b
100052899	a	a	a	a	a	a	a	b
3078850S	a	a	a	a	a	a	a	b
100013369	a	a	b	a	a	a	a	b
100008593	a	a	b	a	b	a	a	b
100006331	a	a	b	a	b	a	a	b
100030523	a	a	b	a	b	a	a	b
100074804	a	a	b	a	b	a	a	b
100010811	a	a	b	a	b	a	a	b
100040921	a	a	b	a	b	a	a	b
100064791	b	a	b	a	b	a	a	b
3079893S	a	a	b	a	b	a	a	b
100014743	a	a	b	a	b	a	a	b
3100861S	a	a	b	a	b	a	a	b
sN2016	a	a	b	a	b	a	a	b
A8-BAC22	a	a	b	a	b	a	a	b
3085906S	a	b	a	b	a	b	b	-
100026545	a	b	a	a	a	a	a	a
100024657	a	b	a	a	b	a	a	a
100013650	a	b	a	a	b	a	a	a
3082910S	a	b	a	a	b	a	a	a
100026791	a	b	a	a	b	a	a	a
BnGMS085	a	b	a	a	b	a	a	a

100053369	a	b	a	a	b	a	a	a
100013061	a	b	a	a	b	a	a	a
3074768S	a	b	a	a	b	a	a	a
100055717	a	b	a	a	b	a	a	a
100019001	a	b	a	a	b	a	a	a

YW009	YW010	YW011	YW012	YW013	YW014	YW015	YW016	YW017	YW018
a	a	b	a	b	a	a	a	a	a
-	b	b	b	b	b	a	b	a	a
a	b	b	a	b	b	a	b	a	a
a	b	b	a	b	b	a	b	a	a
a	b	b	a	b	b	a	b	a	a
a	b	-	a	b	b	a	b	a	a
a	b	b	a	b	b	a	-	a	a
a	b	b	a	b	b	a	b	a	a
a	b	b	a	b	b	a	b	a	a
-	b	b	a	b	b	a	b	a	a
a	b	b	a	b	b	a	b	a	a
-	b	b	a	b	b	a	b	a	a
a	b	-	a	b	b	a	b	a	a
a	b	b	a	b	b	-	b	a	a
a	b	b	a	b	b	a	b	-	a
a	b	-	a	b	b	a	b	a	a
b	b	b	a	b	b	a	b	a	-
a	b	b	a	b	b	a	b	a	a
a	b	b	a	b	b	b	b	a	a
a	b	b	a	b	b	a	b	a	a
a	b	b	a	b	b	a	b	a	a
a	b	b	a	b	b	a	b	a	a
a	b	b	a	b	b	a	b	a	a
a	b	b	a	b	b	a	b	a	a
a	b	b	a	b	b	-	b	-	a
b	b	b	a	b	b	a	b	a	a
a	b	b	a	b	b	a	b	a	a
a	b	b	a	b	b	a	b	a	a
a	b	b	a	b	a	a	b	a	a
a	b	b	a	b	a	a	b	a	a
a	b	b	a	b	a	a	b	a	a
a	b	b	a	b	a	a	b	a	a
a	b	b	a	b	a	b	b	a	a
a	b	a	a	b	a	a	b	a	a
a	b	a	a	b	a	-	b	a	a
a	b	a	a	b	a	a	b	a	a
-	b	a	a	-	a	a	b	a	a
a	b	a	a	b	a	a	b	a	a
a	b	a	a	b	a	a	b	a	a
a	b	a	-	b	a	a	b	a	a
a	b	a	a	b	a	a	b	a	a
b	b	a	a	b	a	a	-	a	a
b	b	a	a	b	a	a	b	a	a
b	b	a	-	b	a	-	b	a	a
b	b	a	a	b	a	a	b	a	a

b	b	a	a	b	a	a	b	-	a
-	b	a	a	b	a	a	b	a	a
b	b	a	a	a	a	a	b	a	a
b	b	a	a	a	a	a	b	a	a
b	b	-	a	a	a	a	b	a	a
-	b	a	a	a	a	a	b	a	a
b	b	a	a	a	-	a	b	a	a
b	b	a	a	a	a	a	b	a	a
b	b	a	a	a	a	a	b	a	a
-	b	a	a	a	a	a	b	a	a
b	b	a	a	a	a	a	b	a	a
b	b	a	b	a	a	a	b	a	a
b	b	a	a	a	a	a	b	a	a
b	b	a	b	a	a	a	b	a	a
b	b	a	b	a	a	a	b	a	a
b	b	a	b	a	-	a	b	a	a
b	b	a	b	a	a	a	b	a	a
b	b	a	b	a	a	a	b	a	a
b	b	a	b	a	a	a	b	a	a
b	b	a	b	a	a	a	b	a	a
b	b	a	b	a	a	-	b	a	a
b	b	a	b	a	a	a	b	a	a
b	b	b	b	a	a	a	b	a	a
b	b	b	b	a	a	a	b	a	a
b	b	b	b	a	a	a	b	a	a
b	b	b	b	a	a	a	b	a	a
b	b	b	-	a	a	a	b	a	a
b	b	b	b	a	a	a	b	a	a
b	b	b	b	a	a	a	b	a	a
b	b	b	b	a	a	a	b	a	a
b	b	b	b	a	a	a	b	a	a
b	b	b	b	a	a	a	b	a	a
b	b	b	b	a	a	a	b	a	a
b	b	-	b	a	a	a	b	a	a
b	b	b	b	a	a	a	b	a	a
b	b	b	b	a	a	a	b	a	a
-	b	b	b	a	a	a	b	a	a
-	b	b	-	a	a	a	b	a	a
b	b	b	b	a	a	a	b	a	a
b	a	a	a	a	a	b	a	a	b
-	a	a	a	-	a	b	a	a	b
b	a	a	a	a	a	b	a	a	b
b	a	a	a	a	a	b	a	a	b
b	a	a	a	a	a	b	a	a	b
b	a	a	-	a	a	b	a	a	b
-	a	a	a	a	a	b	a	a	b
b	a	a	a	a	a	b	a	a	b

b	a	a	-	-	a	b	a	a	b
b	a	a	a	a	a	b	a	a	b
b	a	a	a	a	a	b	a	a	b
b	a	a	a	a	a	b	a	a	b
b	a	a	a	a	a	b	a	a	b
b	a	a	a	a	a	b	a	a	b
b	a	a	a	a	a	-	a	a	b
b	a	a	a	a	a	b	a	-	b
b	a	a	a	a	a	b	a	a	b
b	a	a	a	a	a	a	a	a	b
b	a	a	a	a	-	a	a	a	b
b	a	a	-	a	a	a	-	a	b
b	a	a	-	a	a	a	a	a	b
b	a	a	a	a	a	a	a	a	b
b	a	a	-	a	a	a	a	-	b
b	a	a	a	a	a	a	a	a	b
-	a	a	-	a	a	a	a	a	b
b	a	a	a	a	a	a	a	a	b
b	a	a	a	a	a	a	a	a	b
b	a	b	a	a	a	a	a	a	b
b	a	b	a	a	-	a	a	a	b
b	a	b	a	a	a	a	a	a	b
a	a	b	a	a	a	a	a	a	b
b	a	b	a	a	a	a	a	a	b
a	a	b	b	a	a	a	a	a	b
b	a	b	-	a	a	a	a	a	b
b	a	b	a	a	a	a	a	a	b
b	a	b	b	a	a	a	a	a	b
b	a	b	a	a	b	a	a	a	b
-	a	b	a	a	b	a	a	a	b
b	a	-	a	a	b	a	a	a	b
b	a	b	a	a	b	a	a	a	b
-	a	-	a	a	b	a	a	a	b
-	a	-	a	a	b	a	a	a	b
-	a	b	a	a	b	a	a	a	b
b	a	b	a	-	b	-	a	a	b
b	a	b	a	a	b	a	a	a	b
b	a	b	a	a	b	-	-	a	b
b	a	b	-	a	b	a	a	a	b
b	a	b	a	a	b	a	a	a	b
b	a	b	a	a	-	a	a	a	b
b	a	b	a	a	b	a	a	a	b
b	a	b	a	a	b	a	a	a	b
-	a	b	a	a	b	a	a	a	-
b	a	b	a	a	b	a	a	a	b
b	a	-	a	a	b	a	a	a	b
b	a	b	a	a	b	a	a	a	b

a	a	b	a	-	b	a	a	a	b
b	b	-	a	a	b	a	a	a	a
b	b	b	a	a	b	a	a	a	a
b	b	b	a	a	b	a	a	a	a
b	b	b	a	a	b	a	a	a	a
b	b	b	a	a	b	a	a	a	a
b	-	b	a	a	b	a	a	a	a
b	b	a	a	a	b	a	a	a	a
b	b	a	a	a	b	a	a	a	a
b	b	a	-	a	b	a	a	a	a
b	b	a	a	a	b	a	a	a	a
b	b	a	a	a	b	a	a	a	a
b	b	-	a	a	b	a	-	a	a
b	b	a	a	b	b	a	a	a	a
b	b	a	a	b	b	a	a	a	a
b	b	a	-	b	b	a	a	a	a
b	b	a	a	b	b	a	a	a	a
b	b	a	a	b	b	a	a	a	a
b	b	a	a	b	b	a	a	a	a
b	b	-	-	b	b	b	a	a	a
b	a	b	a	b	b	a	a	a	a
b	a	b	a	b	b	a	a	a	a
a	a	b	a	b	b	a	a	a	a
a	a	b	a	b	b	-	a	a	a
a	a	b	a	-	b	b	a	a	a
a	a	b	a	b	b	b	a	a	a
a	a	b	a	b	b	b	a	a	a
a	a	b	a	b	b	b	a	a	a
a	a	b	a	b	b	b	a	a	a
a	a	b	a	b	b	b	a	a	a
a	a	b	a	b	b	b	a	a	a
a	a	b	a	b	b	-	a	a	a
a	a	b	a	b	-	b	a	a	a
a	a	b	a	b	b	a	a	a	a
a	a	b	a	b	b	a	a	a	a
a	a	b	a	b	b	a	a	a	a
a	a	b	a	b	b	b	a	a	a
-	a	b	-	b	b	b	a	a	a
a	a	b	-	b	b	b	-	a	a
a	a	b	a	b	b	b	a	a	a
a	a	b	a	b	b	-	a	a	a
a	a	b	a	b	b	b	a	a	a
a	a	b	a	b	a	b	a	a	a
a	a	b	a	b	a	b	a	a	a
a	a	b	a	b	a	b	a	a	a
a	a	b	a	b	a	b	a	a	a
a	a	b	a	b	a	b	a	a	a

a	a	b	a	b	a	-	a	a	a
a	a	b	a	b	a	b	-	a	a
a	a	b	a	b	a	b	a	a	a
a	a	b	a	b	a	b	a	a	a
a	a	b	-	b	-	b	a	a	a
a	a	b	a	b	a	b	a	a	a
a	a	b	a	-	a	b	a	a	a
-	a	b	a	b	a	b	a	b	a
a	b	b	a	b	-	b	a	b	a
a	b	b	a	b	b	b	a	b	a
a	a	-	a	b	a	a	a	b	a
a	b	b	a	b	b	b	a	b	a
b	a	a	b	b	b	a	b	b	b
b	a	a	b	b	b	a	b	b	b
b	a	-	b	b	b	a	b	b	b
b	a	a	b	b	b	a	b	b	b
b	a	a	b	b	b	a	b	b	b
b	a	a	b	b	b	a	b	b	b
b	a	a	b	b	b	a	b	b	b
b	a	b	b	b	b	-	-	b	b
b	a	a	b	b	b	a	b	b	b
b	a	a	b	b	b	a	b	b	b
b	a	a	b	b	b	a	b	b	b
b	a	a	b	b	b	a	b	b	b
b	a	a	b	b	b	-	b	b	b
-	a	a	b	b	b	a	b	b	b
-	a	a	b	b	-	a	b	b	b
b	a	a	b	b	b	-	b	b	b
-	a	a	b	b	b	a	b	b	b
b	b	a	b	b	b	a	b	b	b
b	b	a	-	b	b	a	b	b	b
b	b	a	b	b	b	a	-	b	b
b	b	a	b	b	b	a	b	b	b
b	b	a	b	b	b	a	b	b	b
b	b	a	b	b	b	a	b	b	b
b	b	a	b	b	b	a	b	b	b
b	b	a	b	b	b	a	a	b	b
b	b	a	b	b	b	-	a	b	b
b	b	a	b	b	b	a	a	b	b
a	b	a	b	b	b	a	a	b	b
a	b	a	b	b	a	a	a	b	b
a	b	a	b	b	a	a	a	b	b
a	b	a	b	b	a	a	a	b	b
-	b	a	b	b	a	a	a	b	b
a	b	a	b	b	a	a	a	b	b
a	b	a	b	b	a	a	a	b	b
a	a	b	b	b	a	b	a	a	b

a	a	b	b	b	a	b	a	a	b
a	a	b	-	b	a	b	a	a	b
a	a	b	b	b	a	b	a	a	b
a	a	b	b	b	a	b	a	a	b
a	a	b	b	b	a	b	a	a	b
a	a	b	b	b	a	b	a	a	b
a	a	b	b	b	a	b	a	a	b
a	a	b	b	b	a	b	a	a	b
a	a	b	b	b	-	b	a	a	b
a	a	b	b	b	a	b	a	a	b
a	a	b	-	b	a	b	a	a	b
a	a	b	b	b	a	b	a	a	b
a	a	b	-	b	a	b	a	a	b
-	a	b	b	b	a	b	a	a	b
a	a	b	-	b	a	-	a	a	b
a	a	b	b	b	a	b	a	a	b
a	a	b	b	b	a	b	a	a	b
a	a	b	b	b	a	b	a	a	b
a	a	b	b	b	a	b	a	a	b
a	a	b	b	b	a	b	a	a	b
a	a	b	b	b	a	b	a	a	b
a	a	b	-	b	a	b	a	a	b
a	a	b	b	b	a	b	a	a	b
a	a	b	b	b	a	b	a	a	b
a	a	b	b	b	a	b	a	a	b
a	a	b	b	b	a	b	a	a	a
a	a	b	b	b	a	b	a	a	a
a	a	b	b	b	a	b	a	a	a
a	a	-	b	b	a	-	a	a	a
a	a	b	b	b	a	b	a	a	a
a	a	b	b	b	a	b	a	a	a
a	a	b	b	b	a	b	a	a	a
a	a	b	b	b	a	b	a	a	a
a	a	b	b	b	a	b	a	a	a
a	a	b	b	b	a	b	a	a	a
a	a	b	b	b	a	b	a	a	a
a	a	a	b	b	a	b	a	a	a
a	a	b	b	b	a	b	a	a	a
a	a	b	b	b	a	b	a	a	a
a	a	b	b	b	a	b	a	a	a
a	a	b	b	b	a	b	a	a	a
a	a	b	b	b	a	b	a	a	a
a	a	b	b	b	a	b	a	a	a
a	a	b	b	b	a	b	a	a	a
a	a	b	-	b	a	b	a	a	a
a	a	b	b	b	a	b	a	a	a
a	a	-	b	b	a	b	a	a	a

a	a	b	b	b	a	b	a	a	a
-	a	b	b	b	a	b	a	a	a
a	a	b	b	b	a	b	a	a	a
a	a	b	b	b	a	b	a	a	a
a	a	b	b	b	a	b	a	a	a
a	a	b	b	b	a	b	a	a	a
a	a	b	b	b	a	b	a	b	a
a	a	b	b	b	a	b	a	b	a
a	a	a	b	b	a	b	a	b	a
a	a	b	b	b	a	b	a	b	a
a	a	b	b	-	a	b	a	b	a
a	a	b	b	b	a	b	a	b	a
a	a	b	b	b	a	b	a	b	a
b	a	b	b	b	a	b	a	b	a
b	a	b	b	b	a	b	a	b	a
b	a	b	b	b	a	b	a	b	a
b	a	b	b	b	a	b	a	b	a
b	a	b	b	b	a	b	a	b	a
b	a	b	b	b	a	b	a	b	a
b	a	b	b	b	a	b	a	b	a
b	a	b	b	b	a	b	a	b	a
b	a	b	b	b	a	b	a	b	a
b	a	b	b	b	b	b	a	b	a
b	a	b	b	b	b	b	a	b	a
b	b	b	b	b	b	b	a	b	a
b	a	-	b	b	b	b	a	b	a
b	a	b	a	b	b	b	a	b	a
b	a	b	a	b	b	b	a	b	a
b	a	b	a	b	b	b	a	b	a
b	a	b	a	b	b	b	a	b	a
b	a	b	a	b	b	b	a	b	a
b	a	b	a	b	b	b	a	b	a
b	a	b	a	b	b	-	a	b	a
b	a	b	a	b	b	b	a	b	a
b	a	b	a	b	b	-	a	b	a
b	a	b	a	b	b	b	a	b	a
b	a	b	a	b	b	b	a	b	a
a	a	b	a	b	b	b	-	b	a
a	b	b	b	a	a	a	b	b	a
-	b	b	b	a	a	b	b	b	a
-	b	-	b	a	a	b	b	b	a
a	-	b	-	a	a	b	b	b	a
-	b	b	b	a	a	b	b	b	a
a	b	b	b	a	a	b	b	b	a
a	b	b	b	a	a	b	-	b	a
a	b	b	b	a	a	b	b	b	a
a	b	b	b	a	a	b	b	b	a

a	b	b	b	a	a	b	b	b	a
a	b	b	b	a	a	b	b	b	a
a	b	b	b	a	a	b	b	b	a
a	b	b	b	a	a	b	b	b	a
a	b	b	b	a	a	b	b	b	a
a	b	b	b	a	a	b	b	b	a
-	b	b	b	a	a	b	b	b	a
a	b	b	b	a	a	b	a	b	a
a	b	b	b	a	a	b	a	b	a
a	b	b	-	a	a	b	a	-	a
a	b	b	b	a	a	b	a	b	a
a	b	b	b	a	a	b	a	b	a
a	b	b	b	a	a	b	a	b	a
-	b	b	b	a	a	b	a	b	a
a	b	b	b	a	-	b	a	b	a
a	b	b	-	a	a	-	a	b	a
a	b	b	b	a	a	b	a	b	a
a	b	b	b	a	a	-	a	b	a
a	b	b	b	a	a	b	a	b	b
b	b	b	b	a	b	b	a	b	b
a	b	b	b	a	b	b	a	b	b
a	b	b	b	a	-	b	a	b	b
a	b	b	b	a	b	b	a	b	b
a	b	b	b	a	b	b	a	b	b
a	b	b	b	a	b	b	a	b	b
b	b	b	b	a	b	b	a	b	b
a	b	b	b	a	b	-	a	b	b
a	b	b	b	a	b	a	a	b	b
a	b	a	b	a	b	b	a	b	b
a	b	a	b	a	b	b	a	b	b
a	b	a	b	a	b	b	a	b	b
a	b	a	b	a	b	b	a	b	b
a	b	a	b	b	b	b	a	b	b
a	b	a	b	b	b	b	a	b	b
a	b	a	b	b	b	b	a	b	b
a	b	a	b	b	b	b	a	b	b
a	-	a	b	b	b	a	a	b	b
-	b	a	b	b	b	a	a	b	b
-	b	a	b	b	b	a	a	b	b
a	b	a	b	b	b	a	a	b	b
b	b	a	b	b	b	a	a	b	b
a	b	a	b	b	b	a	a	b	-
a	b	a	b	b	b	a	b	b	b
a	b	-	b	b	b	a	b	b	b
a	b	a	b	b	b	a	b	b	b
a	b	a	b	b	b	a	b	b	b
a	b	a	b	b	b	a	b	b	b

a	b	a	b	b	b	a	b	b	b
a	b	a	a	b	-	a	b	b	b
a	b	a	a	b	b	a	b	b	a
a	b	a	a	b	b	a	b	b	a
-	b	a	a	b	-	a	-	b	a
a	b	a	a	b	b	a	b	b	a
a	b	a	a	b	b	a	b	b	a
-	b	a	-	b	b	a	b	b	a
a	b	a	a	a	b	a	b	b	a
a	b	a	a	a	b	a	b	b	a
a	b	a	a	a	b	a	b	b	a
a	b	a	a	a	b	a	b	b	a
a	b	b	a	a	b	a	b	b	a
a	b	a	a	a	b	a	b	b	a
a	b	a	a	a	b	a	b	b	a
a	b	a	a	a	b	a	b	b	a
a	b	a	a	a	b	a	b	b	a
a	b	a	a	a	b	a	b	b	a
a	b	a	a	a	b	a	b	b	a
-	b	a	a	a	b	a	b	b	a
a	b	a	a	a	b	b	b	b	a
a	b	a	a	a	b	b	b	b	a
a	b	a	a	a	b	b	b	b	a
-	b	a	a	a	b	b	b	b	a
a	b	a	a	b	b	b	b	b	a
a	b	a	a	b	b	b	b	b	a
b	b	a	a	b	b	b	b	b	a
a	b	a	a	-	b	b	-	b	a
a	b	a	a	b	b	-	b	b	a
a	b	a	a	b	b	b	b	b	a
a	b	a	a	b	b	-	b	b	a
a	b	a	a	b	b	b	b	a	a
a	b	a	a	b	b	b	b	a	a
a	b	a	a	b	b	b	b	a	a
a	b	a	a	b	b	b	-	a	a
-	b	a	a	b	b	b	b	a	a
a	b	a	a	b	b	b	b	a	a
a	b	-	a	b	b	b	b	a	a
a	b	a	a	b	b	b	b	a	a
a	b	a	a	-	b	b	-	a	a
a	b	a	-	b	b	b	b	b	a
a	b	b	b	b	b	b	b	b	a
a	b	b	b	b	b	b	b	b	a
a	b	b	b	b	b	a	b	b	a
a	b	b	b	b	b	b	b	b	a
a	b	a	b	b	a	b	a	b	a
a	b	b	b	b	b	b	a	b	a

-	b	-	b	b	b	b	a	b	a
-	b	b	b	b	b	a	a	b	a
b	b	b	b	b	b	-	a	b	a
b	b	b	b	b	b	b	a	b	a
b	b	b	b	b	b	b	a	b	a
b	b	b	b	b	b	b	a	b	a
b	b	b	b	b	b	b	a	b	a
b	b	b	b	b	b	b	a	b	a
b	b	b	b	b	b	b	a	b	a
b	b	b	a	b	b	a	a	b	a
b	b	b	a	b	b	b	a	b	a
b	b	b	a	b	b	b	a	b	a
b	b	b	a	b	b	b	a	b	a
b	b	b	a	b	b	b	a	b	a
b	b	b	a	b	a	a	a	-	a
b	b	b	a	b	a	b	a	b	a
b	b	b	a	b	a	b	a	b	a
b	b	b	-	b	a	b	a	b	a
b	b	b	a	b	a	b	a	b	a
b	b	b	a	b	a	b	b	b	a
b	b	b	a	b	a	b	b	b	a
b	a	b	a	b	a	b	b	b	a
b	a	b	a	b	a	b	b	b	a
b	a	b	a	b	a	-	b	b	a
b	a	b	a	b	a	b	b	b	a
b	a	b	a	b	a	b	b	b	a
b	a	b	a	b	a	b	b	b	a
b	a	b	a	b	a	b	b	b	a
b	a	b	a	b	a	-	b	b	a
b	a	b	a	b	a	b	b	b	a
b	a	b	a	b	a	b	b	b	a
b	a	b	a	b	a	b	b	b	a
b	a	b	a	b	a	b	-	b	a
b	a	b	a	-	a	-	b	b	a
b	a	-	a	b	a	a	-	b	a
b	a	b	a	b	a	-	b	b	a
b	a	b	a	b	a	b	b	b	a
b	a	b	a	b	a	b	b	b	a
b	a	b	a	b	a	b	b	b	a
b	a	b	a	b	a	b	b	b	a
b	a	b	a	b	a	b	b	b	a
b	a	b	-	b	a	b	b	b	a
b	a	b	a	b	a	-	b	b	a
b	a	b	a	b	a	b	b	b	a
b	a	b	a	b	a	b	b	b	a
b	a	b	a	b	a	b	b	b	a
b	a	b	a	b	a	b	b	b	a

a	a	b	a	b	a	b	b	b	a
b	a	b	b	b	a	-	b	b	a
b	a	b	b	b	a	b	b	b	a
b	b	b	b	b	a	-	b	b	a
b	-	-	b	b	a	b	b	-	a
b	b	b	b	b	a	b	b	b	a
b	b	b	b	b	a	b	b	b	a
b	b	b	b	b	a	b	b	b	a
b	b	b	b	b	a	b	b	b	a
b	b	b	b	b	a	b	b	b	a
b	b	b	b	b	a	b	b	b	a
b	b	b	b	b	a	b	b	b	a
b	b	b	b	b	a	b	b	b	a
b	b	b	b	b	a	-	b	b	a
b	b	b	b	b	a	b	b	b	a
-	b	b	b	b	a	b	b	b	a
b	b	b	b	b	a	b	b	b	a
b	b	b	b	b	a	b	b	b	a
b	b	b	b	b	a	b	b	b	a
b	b	b	b	b	a	b	b	b	a
b	b	b	b	b	a	b	b	b	a
b	b	b	b	b	a	b	b	b	a
b	b	b	b	b	a	b	b	b	a
b	b	b	b	b	a	b	b	b	-
b	b	b	b	b	a	b	b	b	a
b	b	b	b	b	a	b	b	b	b
b	b	b	b	b	a	b	b	b	b
b	b	b	b	b	a	b	b	b	b
-	b	-	b	b	a	b	b	b	b
b	b	b	b	b	a	b	b	b	b
b	b	-	b	b	a	b	b	b	b
b	b	b	b	b	a	b	b	b	b
b	b	b	b	b	a	b	b	b	b
b	b	b	b	b	a	b	b	b	b
b	b	b	b	b	a	b	b	b	b
b	b	b	b	b	a	b	b	b	b
b	b	b	b	b	a	b	b	b	b
b	b	b	b	b	a	b	b	b	b
b	b	b	b	b	a	b	b	b	b
b	b	b	b	b	a	b	b	b	b
b	b	b	b	b	a	-	-	b	b
b	b	b	b	b	a	b	b	b	b
b	b	b	b	b	a	b	b	b	b
b	b	b	b	b	a	b	b	b	b
b	b	b	b	b	a	b	b	b	b
b	b	b	b	b	a	b	b	b	b
b	b	b	b	b	a	b	b	b	b

b	b	b	b	b	a	b	b	b	b
b	b	b	b	b	a	b	b	b	b
b	b	b	b	b	a	b	b	b	b
b	b	b	b	b	a	b	b	b	b
b	b	b	b	b	b	b	b	b	b
b	b	b	b	b	-	b	b	b	b
b	b	a	b	b	a	b	b	b	b
-	a	-	b	b	b	b	b	b	b
a	a	a	b	b	b	b	b	b	b
a	a	a	b	b	b	b	b	b	b
a	a	a	b	b	b	b	b	b	b
a	a	a	a	b	b	b	b	b	b
a	a	a	a	b	b	b	b	b	b
a	a	a	a	b	b	b	b	b	b
a	a	a	a	b	b	b	b	b	b
a	a	a	a	b	b	b	b	b	b
a	a	a	a	b	b	b	b	b	b
-	a	a	a	b	b	b	b	b	b
a	a	a	b	b	b	b	b	b	b
-	a	-	b	a	a	a	b	a	b
b	a	b	b	a	a	a	b	a	b
b	a	b	-	a	a	a	b	a	b
-	a	b	b	a	a	a	b	a	b
-	a	b	b	a	a	a	b	a	b
b	a	b	b	a	a	a	b	a	b
b	a	b	b	a	a	a	b	a	b
b	a	b	b	a	a	a	b	a	b
b	a	b	b	a	a	a	b	a	b
b	a	b	b	a	a	a	b	a	b
b	a	b	b	a	a	a	b	a	b
b	a	-	b	a	a	a	a	a	b
-	a	b	b	a	a	a	a	a	b
b	a	b	b	a	a	a	a	a	b
b	a	b	b	a	a	b	a	a	b
b	a	b	b	a	a	b	a	a	b
b	a	b	-	a	a	a	a	a	b
b	a	b	b	a	a	a	a	a	b
b	a	b	b	a	a	a	a	a	b
b	a	b	b	a	a	a	a	a	b
b	a	b	b	a	a	a	a	a	b
b	a	b	b	a	a	a	a	a	b
b	a	b	b	a	a	a	a	a	b
b	a	b	b	a	a	a	a	a	b
b	a	b	b	-	a	a	a	a	b
b	a	b	b	a	a	a	a	a	b
b	a	b	b	b	a	a	a	-	b
b	a	b	b	b	a	b	a	a	b

-	a	b	b	b	a	a	-	a	b
b	a	b	b	b	a	a	a	a	b
b	a	b	b	b	a	a	a	a	b
b	a	b	b	b	a	a	a	a	b
b	a	b	b	-	a	a	a	a	b
a	a	b	b	b	a	a	a	a	b
-	a	a	-	b	a	a	a	a	b
a	a	b	b	b	a	a	a	a	b
a	a	b	b	-	a	a	a	a	b
a	a	b	b	b	a	a	a	-	b
a	-	b	b	b	a	-	-	a	b
a	a	b	b	b	a	a	a	a	b
a	a	b	b	b	a	a	a	a	b
a	a	b	b	b	a	a	a	a	b
a	a	b	b	b	a	a	a	a	a
a	a	b	b	b	a	a	a	a	a
a	a	b	b	b	a	a	a	a	a
a	a	b	b	b	a	a	a	a	a
a	a	b	b	b	a	a	a	a	a
a	a	b	b	a	a	a	a	a	a
a	a	b	b	a	a	a	a	a	a
a	a	b	b	a	a	a	a	a	a
a	a	b	b	-	a	a	a	a	a
a	a	b	b	a	-	a	-	a	a
a	a	b	b	a	a	a	a	a	a
a	a	b	b	a	a	a	a	a	a
a	a	b	b	a	a	a	a	a	a
a	a	b	b	a	a	a	a	a	a
a	a	b	b	a	a	a	a	a	a
a	a	b	b	a	a	a	a	a	a
a	a	b	b	a	a	a	a	a	a
a	a	b	b	a	a	a	a	a	a
a	a	b	b	a	a	a	a	a	a
a	a	b	b	a	a	a	a	a	a
a	a	b	b	a	a	a	a	a	a
a	a	b	b	a	a	-	a	a	a
a	a	b	b	a	a	a	a	a	a
a	a	b	b	a	a	a	a	a	a
a	a	b	b	a	a	a	a	a	a
a	a	b	b	a	a	a	a	a	a
a	a	b	b	a	a	a	a	a	a
a	a	b	b	a	a	a	a	a	a
a	a	b	-	a	a	a	-	a	a
a	a	b	b	a	a	a	b	a	a
a	a	-	b	a	a	a	b	a	b
a	a	b	b	a	a	a	b	a	b
-	b	b	b	a	a	a	b	a	b

a	b	b	b	a	a	a	b	a	b
a	b	b	b	a	a	a	b	a	b
a	b	b	b	-	a	a	b	a	b
a	b	b	b	a	a	a	b	a	b
a	b	b	b	a	a	a	b	a	b
a	b	b	b	a	a	a	b	a	b
a	b	b	b	a	a	a	b	a	b
a	b	b	a	a	a	a	b	a	b
a	b	b	a	a	a	a	b	a	b
a	b	-	a	a	a	a	-	a	b
a	b	a	b	a	a	a	b	b	b
a	a	a	b	b	b	a	a	b	b
-	a	a	b	b	b	a	a	b	b
a	a	a	b	b	b	a	-	b	b
a	a	a	b	b	b	a	a	b	b
a	a	a	b	b	b	a	a	b	b
a	a	a	b	b	b	a	a	b	b
a	a	a	b	b	b	a	a	b	b
a	a	a	-	b	b	a	a	-	b
a	a	a	b	b	b	a	a	-	b
a	a	a	b	b	b	-	a	b	b
a	a	a	b	b	b	a	a	b	b
a	a	a	b	b	b	a	a	b	b
a	a	a	b	b	-	a	a	b	b
a	a	a	b	b	b	a	a	b	b
a	a	a	b	b	b	a	a	b	b
a	a	a	b	b	b	a	a	b	b
a	a	a	b	b	b	a	a	b	b
a	a	a	b	b	a	a	a	a	b
a	a	a	b	b	a	a	a	a	b
a	a	a	b	b	a	a	a	a	b
a	a	a	b	b	a	a	a	a	b
a	b	b	b	b	a	a	a	a	b
a	b	b	b	b	a	a	a	a	b
a	b	b	b	b	a	a	a	a	b
a	b	b	b	b	a	a	a	a	b
a	b	b	b	b	a	a	a	a	b
-	b	b	b	b	a	a	a	a	b
a	b	b	b	b	a	a	a	a	b
a	b	b	b	b	a	a	a	a	b
a	b	b	b	b	a	a	-	a	b
a	b	b	b	b	a	a	a	a	b
-	b	b	b	b	a	a	a	a	b
a	b	b	b	b	a	a	a	a	b
a	b	b	b	b	a	a	a	a	b
-	b	b	b	b	a	a	a	a	b
-	b	b	b	b	a	a	a	a	b

a	b	b	b	b	a	a	-	a	-
a	b	b	b	b	a	a	a	a	a
a	b	-	b	b	a	a	a	a	a
a	b	b	b	b	a	a	a	a	a
a	b	b	b	b	a	a	a	a	a
b	b	b	b	b	a	a	a	a	a
b	b	b	b	b	a	a	a	a	a
b	b	b	b	b	a	a	a	a	a
b	b	b	b	b	a	b	a	a	a
b	b	b	b	b	a	-	a	a	a
b	b	b	b	b	a	b	a	a	a
b	b	b	b	b	a	b	a	-	a
b	b	b	b	b	a	b	a	a	a
b	b	-	-	b	a	b	a	a	a
b	b	b	b	b	a	b	a	a	a
-	b	b	b	b	a	b	a	a	a
b	b	b	b	b	a	b	a	a	a
b	b	b	b	b	a	b	a	a	a
b	b	b	-	b	a	-	a	a	a
b	b	b	b	b	a	b	a	a	a
b	b	b	b	b	a	b	a	a	a
b	b	b	b	b	a	b	a	a	a
b	b	b	b	b	a	b	a	a	a
-	b	-	b	b	a	b	a	a	a
b	b	b	b	b	a	b	a	a	a
b	b	b	b	b	a	b	a	a	a
b	b	b	b	b	a	b	a	a	a
b	b	b	b	b	a	b	a	a	a
b	b	b	b	b	a	b	a	a	a
b	b	b	b	b	a	b	a	a	a
b	b	b	b	b	a	b	a	a	a
-	b	b	b	b	a	b	a	a	a
b	b	-	b	b	a	-	a	a	a
a	b	b	b	b	a	b	a	a	a
a	b	b	b	b	a	a	a	a	a
a	b	b	b	b	a	b	a	a	a
a	b	b	b	b	a	b	a	-	a
a	b	b	b	b	a	b	a	a	a
a	b	b	b	b	a	b	a	a	a
a	b	b	b	b	a	b	a	a	a
a	b	b	b	b	a	b	a	a	a
a	b	b	a	b	a	b	a	a	a
a	b	b	a	b	a	b	a	a	a
a	b	b	a	b	a	b	a	a	a
a	b	b	a	b	a	b	a	a	a
a	b	b	a	b	a	b	a	a	a
a	b	b	a	b	a	b	a	a	a

a	b	b	a	b	a	b	a	a	a
a	b	b	a	b	a	b	a	a	a
a	b	b	a	b	a	b	a	a	a
a	b	b	a	b	a	b	a	a	a
a	b	b	a	b	a	b	a	a	a
b	b	b	a	b	a	b	a	a	a
a	b	b	a	b	a	b	a	a	a
a	b	b	a	b	a	b	a	b	a
a	b	b	a	b	a	-	a	b	a
a	b	b	a	b	a	b	a	b	a
a	b	b	a	b	a	b	a	b	a
a	b	b	a	b	a	b	a	b	a
-	b	b	a	b	a	b	a	b	a
a	b	b	a	b	a	b	a	b	a
a	b	b	a	b	a	b	a	b	a
a	b	b	a	b	a	b	a	b	a
-	b	b	a	b	a	b	a	b	a
a	b	b	a	b	a	b	a	b	a
a	b	b	a	b	a	b	-	b	a
a	b	b	a	b	a	b	a	b	a
a	b	b	a	b	a	b	a	b	a
a	b	b	a	b	-	b	b	b	a
b	b	a	b	-	b	a	b	a	b
a	b	a	b	a	b	b	b	a	b
a	b	a	b	a	b	b	b	a	b
a	b	a	b	a	b	b	b	a	b
-	b	a	b	a	b	b	b	a	b
a	b	a	b	a	b	b	b	a	b
-	b	a	b	a	-	b	-	a	b
a	b	a	b	a	b	b	b	a	b
a	b	-	b	a	b	b	b	a	b
-	b	a	b	a	-	b	b	a	b
a	b	a	b	a	b	b	b	a	b
a	b	a	-	b	b	b	b	a	b
a	b	a	b	b	b	b	b	a	b
a	b	a	b	b	b	b	b	a	b
a	b	a	b	b	-	b	b	a	b
-	b	a	b	b	b	b	b	a	b
a	a	a	b	b	b	b	b	a	b
a	a	a	b	b	b	b	b	a	b
a	a	a	b	b	b	b	b	a	b
a	a	a	b	b	b	b	b	a	b
a	a	a	b	b	b	b	b	a	b
a	a	a	b	b	b	-	b	a	b
a	a	a	b	b	b	b	b	a	b
a	a	a	b	b	b	b	b	a	b
-	a	a	b	b	b	b	b	a	b

a	a	a	b	b	b	b	b	a	b
a	a	a	b	b	b	b	b	a	b
a	a	-	b	b	b	b	-	a	b
a	a	a	b	b	b	b	-	a	a
a	a	b	b	b	b	b	b	a	a
a	a	b	b	b	b	b	b	a	a
a	a	b	b	b	b	b	b	a	a
a	a	b	b	-	b	b	b	a	a
a	a	b	b	b	b	b	b	a	a
a	a	b	b	b	b	b	b	a	a
a	a	b	b	b	b	b	b	a	a
a	a	b	b	b	b	b	b	a	a
a	a	b	b	b	b	b	b	a	a
a	a	b	-	b	b	b	b	a	a
a	a	b	b	b	b	b	b	a	a
a	a	b	b	b	b	b	b	a	a
a	a	b	-	b	b	b	b	a	a
a	a	b	b	b	b	b	b	a	a
a	a	b	b	b	b	b	b	a	a
a	a	b	b	b	b	-	b	a	a
a	a	b	b	b	b	b	b	a	a
a	a	b	b	b	b	b	b	a	a
a	a	b	b	b	b	b	b	a	a
a	a	b	b	b	b	b	b	a	a
a	a	b	b	b	b	b	b	a	a
a	a	b	b	b	b	b	b	a	a
a	a	b	b	b	b	b	b	-	a
a	-	b	b	b	a	b	b	a	b
a	a	b	b	b	a	b	b	a	b
a	a	b	b	b	a	-	b	a	b
a	a	b	b	b	a	b	b	a	b
a	a	b	b	b	a	b	b	a	b
a	a	b	b	b	a	b	b	a	b
a	a	b	b	b	a	b	b	a	b
a	a	b	b	b	a	b	b	a	b
a	a	b	b	b	a	b	b	a	b
a	a	b	b	b	-	b	b	a	b
a	a	b	b	b	a	b	b	a	b
a	a	b	b	b	a	b	b	a	b
a	a	b	b	b	a	b	b	a	b
a	a	b	b	b	a	b	b	a	b
a	a	b	b	b	a	b	b	a	b
a	a	b	b	b	a	b	b	a	b
a	a	b	b	b	a	b	b	a	b
-	a	b	b	b	a	b	b	a	b
a	a	b	b	b	a	b	b	a	b
a	a	b	b	b	a	b	b	a	b

a	a	b	b	b	a	b	b	a	b
a	a	b	b	b	a	b	b	a	b
a	a	b	b	b	a	b	b	a	b
a	a	b	b	b	a	-	b	a	b
a	a	b	b	b	a	b	b	a	b
a	a	b	b	b	a	b	b	a	b
a	a	b	b	b	a	b	b	a	b
a	a	b	b	b	a	b	b	a	b
-	a	b	b	b	a	b	b	a	b
-	a	b	b	b	a	b	b	a	b
a	a	b	-	b	a	b	b	a	b
a	a	b	b	b	a	b	b	a	b
a	a	b	b	b	a	b	b	a	b
a	a	b	b	b	-	b	b	a	b
-	a	b	b	b	a	b	b	a	b
a	a	b	b	b	a	b	b	a	b
a	a	a	-	b	a	b	b	a	b
a	a	-	b	b	a	b	b	a	a
-	a	a	b	b	a	b	b	a	a
a	a	a	b	b	a	b	b	a	a
a	a	a	b	b	a	b	b	a	a
a	a	a	b	b	a	b	b	a	a
a	a	a	a	b	a	a	b	a	a
a	a	a	b	b	a	a	b	a	a
a	a	a	b	b	a	a	b	a	a
a	a	a	b	b	a	a	b	a	a
a	a	a	b	b	a	a	b	a	a
a	a	a	b	b	a	a	b	a	a
-	a	a	b	b	a	a	b	a	a
b	a	a	b	b	-	a	b	a	-
b	a	a	b	b	a	a	b	a	a
-	a	a	b	b	a	a	b	a	a
b	a	a	b	b	a	a	b	-	a
b	a	a	b	b	a	a	b	a	a
b	a	a	-	b	a	a	b	a	a
b	a	a	b	b	a	a	b	a	a
-	a	a	b	b	a	a	b	a	a
b	a	a	b	b	a	a	b	a	a
b	a	a	b	b	a	b	b	a	a
b	a	a	b	b	a	a	b	a	a
b	a	a	b	b	a	a	b	a	a
b	a	a	b	b	a	a	b	a	a
b	a	a	b	b	a	a	b	a	a
b	a	-	b	b	a	a	-	a	a
b	a	a	b	b	a	a	a	a	a
b	a	a	b	b	a	a	a	a	a
b	a	a	b	b	a	a	a	a	a

b	a	a	b	b	a	a	a	a	a
b	a	a	b	b	a	a	a	a	a
b	a	a	b	a	a	a	b	a	a
a	b	a	b	b	a	b	b	a	a
a	a	a	b	b	a	b	b	a	a
a	a	a	b	b	a	b	b	a	a
a	a	a	b	b	a	b	b	a	a
a	a	a	b	b	a	-	b	a	a
a	a	a	b	b	a	b	b	b	a
a	a	a	b	b	a	b	b	b	b
a	a	a	b	b	b	b	b	b	b
-	b	a	b	b	b	b	b	b	b
a	b	a	b	b	b	b	b	b	b
a	b	a	b	b	b	b	b	b	b
a	b	a	b	b	b	b	b	b	b
a	b	a	b	b	b	b	b	b	b
b	b	a	b	b	b	b	b	b	b
a	b	a	b	b	b	b	-	b	b
a	b	-	b	b	b	b	b	b	b
a	b	a	b	b	b	b	a	b	b
a	b	a	b	b	b	b	a	b	b
a	b	a	b	b	b	a	a	b	b
a	b	a	b	b	b	b	a	b	b
b	b	a	b	b	b	b	a	-	b
-	b	a	b	b	b	b	a	b	b
b	b	a	b	a	b	b	a	b	b
b	b	a	b	a	b	b	a	b	b
b	b	a	b	a	b	b	a	b	b
-	b	a	b	a	b	b	a	b	b
a	b	a	b	a	b	b	a	b	b
a	b	a	b	a	b	b	a	b	b
a	b	-	b	a	b	b	-	b	b
a	b	a	b	a	b	b	a	b	b
a	b	a	-	-	b	b	a	a	b
a	b	a	a	a	b	b	a	a	b
a	b	a	a	a	b	b	a	a	b
a	b	a	a	a	b	b	a	a	b
a	b	a	a	a	b	b	a	a	b
a	b	a	a	-	-	b	a	a	b
a	b	a	a	a	a	b	a	a	b
a	b	b	-	a	a	b	a	a	b
a	b	b	a	a	a	b	a	a	b
a	b	b	a	a	a	b	a	-	b
a	b	b	a	a	a	b	a	a	b
a	b	b	a	a	a	b	a	a	b
a	b	b	a	a	a	b	a	a	b
a	b	b	a	a	a	b	a	a	b

a	b	b	b	a	a	b	a	a	b
a	b	b	b	a	a	b	a	a	b
a	b	b	b	a	a	b	a	a	a
a	a	b	b	a	a	a	a	a	a
b	b	b	b	b	b	b	b	b	a
b	b	b	b	b	b	b	b	b	a
b	b	b	b	b	b	b	b	b	a
b	b	b	b	b	b	b	b	b	a
b	b	b	b	b	b	b	b	b	a
b	b	b	b	b	b	b	b	b	a
b	b	b	b	b	b	b	b	b	b
b	b	b	-	b	b	b	-	b	b
b	a	b	b	b	b	b	b	b	b
b	-	b	b	b	b	b	b	b	b
b	a	-	b	b	b	b	b	b	b
-	a	b	b	b	b	b	b	b	b
b	a	b	b	b	b	b	b	b	b
b	a	b	b	b	b	b	b	b	b
b	-	b	b	b	b	b	a	b	b
-	a	b	b	b	b	a	a	b	b
b	a	b	b	b	b	b	a	b	b
b	a	b	b	b	b	b	a	b	b
b	a	b	b	b	b	b	a	b	b
b	a	b	b	b	b	b	a	b	b
b	a	b	b	a	b	b	a	b	b
-	a	b	b	a	b	a	a	b	b
b	a	b	b	a	b	a	a	b	b
b	a	b	b	a	b	a	a	b	b
b	a	-	-	a	b	a	a	b	b
b	a	b	b	a	b	a	a	b	b
b	a	b	b	a	b	a	a	b	b
b	a	b	b	a	b	a	a	b	b
b	a	b	b	a	a	a	a	b	b
b	a	b	b	a	a	a	a	b	b
b	a	b	b	a	a	-	a	b	b
b	a	b	b	a	a	a	a	b	b
b	a	b	-	a	a	a	b	b	b
b	a	a	b	a	a	a	b	-	b
b	a	b	b	a	a	a	b	b	b
b	a	b	b	a	a	a	b	b	b
a	b	b	b	a	a	a	b	b	b
a	b	b	a	a	a	a	b	a	b
a	b	b	a	a	a	a	b	a	b
a	b	b	a	a	a	a	b	a	b
a	b	b	a	a	a	a	b	a	b
b	b	a	b	a	b	a	b	b	b
b	b	a	b	-	b	a	b	b	b

b	b	a	b	a	b	a	b	b	b
b	b	a	b	a	b	a	b	b	b
b	b	a	b	a	b	a	b	b	b
b	b	a	b	a	b	a	b	b	b
b	b	-	b	a	b	a	-	b	b
b	b	a	b	a	b	a	b	b	b
b	b	-	b	a	b	a	b	b	b
b	-	a	b	a	b	a	b	-	b
b	b	a	b	a	-	a	b	b	b
b	b	a	b	a	b	a	b	b	b
b	b	a	a	a	b	a	b	b	b
b	b	a	a	a	b	a	b	b	b
-	b	a	a	a	b	a	b	b	b
b	b	a	a	a	b	a	b	b	b
b	b	a	a	a	b	a	b	b	b
b	b	-	a	a	b	a	b	b	b
b	b	a	a	a	b	a	b	b	b
b	b	a	a	a	b	a	b	b	b
b	b	a	a	a	b	-	b	b	b
-	b	a	a	a	b	a	-	-	b
b	b	a	a	a	b	b	b	b	b
b	b	-	a	a	b	b	b	b	b
b	b	a	a	a	-	a	b	b	b
b	b	a	a	a	b	a	b	b	b
b	b	a	-	a	b	a	b	b	b
b	-	a	a	a	b	a	b	-	b
b	b	a	a	a	b	b	b	a	b
b	b	a	a	a	b	b	b	a	b
b	b	a	a	a	b	b	b	a	b
b	b	a	-	a	b	b	b	-	b
b	b	a	a	a	b	b	b	a	b
b	b	a	a	a	b	b	b	a	b
b	b	b	a	a	b	b	b	a	b
a	b	b	a	a	b	b	a	a	b
a	b	b	a	a	b	b	a	a	b
a	b	b	a	a	b	b	a	a	b
-	b	b	-	a	b	b	a	a	b
-	b	b	a	a	b	b	a	a	b
a	b	b	a	a	b	b	a	a	b
a	b	b	a	a	b	b	a	a	b
a	b	b	a	a	b	b	a	-	b
a	b	b	-	a	b	b	a	a	b
a	b	b	a	a	-	b	a	a	b
a	b	b	a	a	b	b	a	a	b
a	b	b	a	a	b	b	a	a	b
a	b	b	a	a	b	b	a	a	b
a	b	b	a	a	-	-	a	a	b

-	b	b	a	a	b	b	a	a	b
a	b	b	a	a	a	b	a	a	b
-	b	b	a	a	a	b	a	a	b
a	b	b	a	a	a	-	a	a	b
a	b	b	a	a	a	-	a	a	b
a	b	b	a	a	a	b	b	a	b
a	b	b	-	a	a	b	b	b	b
a	b	-	a	a	a	b	b	b	b
-	b	b	a	a	a	b	b	b	b
a	b	b	a	a	a	b	b	b	b
a	b	b	a	a	a	-	b	b	b
a	b	b	a	a	a	b	b	b	b
-	b	b	a	a	a	b	b	b	b
a	b	b	a	a	a	b	b	b	b
a	b	b	a	a	a	b	b	b	b
a	b	b	a	a	-	b	b	b	b
a	b	b	a	a	a	b	b	b	b
a	b	b	a	a	a	b	b	b	b
a	b	b	a	a	a	b	b	b	b
a	b	b	a	a	a	b	b	b	b
a	b	b	a	-	a	b	b	b	b
a	b	b	-	a	a	b	b	b	b
a	b	b	a	a	a	b	b	b	b
a	b	b	a	a	a	-	b	b	b
a	b	b	a	a	a	a	b	b	b
a	b	b	a	a	a	a	b	b	b
a	b	b	-	a	a	a	-	b	b
a	b	b	a	a	a	a	b	b	b
a	b	b	a	a	a	a	b	-	b
a	b	b	a	a	a	a	b	b	b
a	b	b	-	a	a	-	b	b	b
a	b	-	a	a	a	a	b	b	b
a	b	b	a	a	-	a	b	b	b
a	b	b	a	a	a	a	b	b	b
a	b	b	a	a	a	a	b	b	b
a	a	b	a	a	a	a	b	b	b
a	a	b	a	a	a	a	b	b	b
a	b	b	a	a	a	a	b	b	b
a	b	b	a	a	a	a	b	b	b
a	b	b	a	a	a	a	b	b	b
a	b	b	a	a	a	a	b	b	b
a	b	b	a	a	a	-	b	b	b
a	a	b	a	a	a	a	b	b	a
a	a	b	a	a	a	a	b	b	a
-	a	b	a	a	a	a	b	b	a
a	a	b	a	a	a	-	b	b	a
a	a	b	a	a	a	a	b	b	a

a	a	b	a	a	a	a	b	b	a
a	a	b	a	a	a	a	b	b	a
a	a	b	a	a	a	a	b	b	a
a	a	b	a	a	a	a	b	-	a
a	a	b	a	a	a	a	b	b	a
-	a	b	a	a	a	-	b	b	a
a	a	b	a	a	a	a	b	b	a
a	a	-	a	a	a	a	b	b	a
a	a	b	b	b	b	a	b	b	a
a	a	b	b	b	b	a	b	b	a
a	a	b	b	b	b	a	b	b	a
a	a	b	b	b	b	a	b	b	a
a	a	b	b	b	b	a	b	b	a
a	a	a	b	b	b	a	b	b	a
a	a	a	b	b	b	a	b	b	a
a	a	a	b	b	b	a	b	b	a
a	a	a	b	b	b	a	b	b	a
a	a	a	b	b	b	-	b	b	a
b	a	a	b	b	b	a	b	b	a
b	a	a	b	a	a	a	b	b	a
b	a	a	b	a	a	a	b	b	a
b	a	a	b	a	a	a	b	b	a
b	a	a	b	a	a	a	b	b	a
b	b	-	b	a	b	a	-	b	a
b	b	a	b	a	b	a	a	b	a
b	b	a	b	a	b	a	a	b	a
b	b	a	-	a	-	a	a	b	a
b	b	a	a	a	a	a	a	b	a
b	b	a	a	a	a	a	a	b	a
b	b	a	a	a	a	a	a	b	-
b	b	a	a	a	a	b	a	a	a
b	b	a	a	a	a	b	a	a	a
b	b	a	a	a	a	b	a	a	a
b	b	a	a	a	a	b	a	-	a
-	-	a	a	a	a	b	a	a	a
b	b	a	a	a	a	-	a	b	a
b	b	a	a	a	a	-	a	b	a
b	b	a	a	a	a	b	a	b	a
b	b	a	a	a	a	b	a	b	a
b	b	a	a	a	a	b	a	b	a
b	b	a	a	a	a	b	a	b	a
b	b	a	a	a	a	b	a	a	a
b	b	a	a	a	a	b	a	a	a
b	b	b	a	a	a	b	a	a	a
b	b	b	a	a	a	b	a	a	a
b	b	b	a	a	a	b	a	a	a
b	b	b	a	a	a	b	a	a	a

b	b	b	a	a	a	b	a	a	a
-	b	b	a	a	b	-	a	a	a
-	a	b	a	a	-	b	a	a	a
b	a	b	a	a	b	b	a	a	a
b	a	b	a	a	b	b	a	a	a
-	a	b	a	a	b	b	a	a	a
-	a	b	b	a	b	b	a	-	a
b	a	b	-	a	-	b	a	a	a
b	b	b	b	a	b	b	a	a	a
b	b	-	b	a	b	b	a	a	a
b	b	b	b	a	b	b	a	a	a
b	b	b	b	a	b	b	-	a	a
a	b	b	a	a	a	a	a	a	b
a	b	b	a	a	a	a	a	a	b
a	b	b	a	a	a	a	a	a	b
-	a	b	a	a	a	a	a	a	a
a	b	b	a	a	b	a	a	a	a
a	b	b	a	a	b	a	a	a	a
a	a	a	a	a	b	a	a	a	a
a	a	a	a	a	b	a	a	-	a
a	a	a	a	a	b	a	a	a	a
a	a	a	a	a	b	a	a	a	a
a	a	a	a	-	b	a	a	-	a
a	a	a	b	a	b	a	a	a	a
a	a	a	b	a	b	a	b	a	a
a	a	a	b	a	b	a	-	a	a
a	a	a	b	a	b	a	b	a	a
a	a	a	b	b	a	a	b	b	a
a	a	a	b	a	b	a	b	a	a
a	a	a	b	a	b	a	b	a	a
a	a	a	-	a	b	a	b	a	a
a	a	a	b	a	b	a	b	a	a
-	a	a	b	a	b	a	b	a	a
a	a	a	b	a	b	a	b	a	a
a	a	a	b	a	b	a	b	a	a
b	a	b	b	a	b	a	b	a	a
b	a	b	b	a	b	a	b	a	a
b	a	b	b	a	b	a	-	a	a
b	a	b	b	a	b	a	b	a	a
b	a	b	-	a	b	a	b	a	a
b	a	b	b	a	b	a	a	b	a
b	a	b	b	a	b	a	a	b	a
b	a	b	b	a	b	a	a	b	a
b	a	b	b	a	b	a	a	b	a
b	a	b	b	a	b	a	a	b	a
a	a	a	b	a	b	a	a	b	a
a	b	a	b	a	b	a	a	a	a

a	b	a	b	a	b	a	a	a	a
-	b	a	b	a	b	a	b	a	a
a	b	a	-	a	b	a	b	a	a
a	b	a	b	a	a	a	b	a	a
a	b	a	b	a	a	a	b	a	a
a	b	a	b	a	a	a	b	a	a
a	b	-	b	a	a	a	-	a	a
a	b	-	b	a	a	a	b	a	a
b	b	a	a	-	a	a	b	a	b
b	b	a	a	b	a	a	b	a	b
b	b	a	a	b	a	b	b	a	b
b	b	a	a	b	a	b	b	a	b
b	b	a	a	b	a	b	b	a	b
-	b	a	a	b	a	b	b	a	b
b	b	-	a	b	a	b	b	a	b
b	b	a	a	b	a	b	b	a	b
b	b	a	a	b	a	b	b	a	b
b	b	a	a	b	a	b	b	a	b
b	b	a	a	b	a	b	b	a	b
b	b	a	a	b	a	b	b	a	b
b	b	a	a	b	a	b	b	a	b
b	b	a	a	b	a	b	b	a	b
b	b	a	a	b	a	-	b	a	b
b	b	a	a	b	a	b	b	a	b
b	b	a	a	b	a	b	b	a	b
a	b	a	a	b	a	b	b	a	b
b	b	a	a	b	a	b	b	a	b
a	b	a	a	-	a	b	b	a	a
a	b	a	a	b	a	-	b	a	a
-	b	a	a	b	b	b	b	a	a
a	b	a	a	b	a	b	b	a	a
a	b	a	a	b	a	b	b	a	a
a	b	a	a	b	a	b	b	a	a
a	b	a	a	b	a	b	b	a	a
a	b	a	a	b	a	b	b	a	a
a	b	a	a	b	a	b	b	a	a
-	b	a	a	b	a	b	b	a	a
a	b	a	a	b	a	a	b	a	a
a	b	a	a	b	a	b	b	a	a
a	b	a	a	b	a	b	-	a	a
a	b	a	a	b	a	b	b	a	a
a	b	a	a	b	a	b	b	a	a
a	b	-	-	b	a	b	b	a	a
a	b	a	-	b	a	b	b	a	a
a	b	a	a	-	a	b	b	a	a
a	b	a	a	b	a	b	-	a	a
-	b	a	a	-	a	b	a	a	a

a	b	a	a	b	a	-	a	a	a
a	b	a	a	b	a	b	a	a	a
a	b	a	a	b	a	b	a	a	a
b	b	a	a	b	a	-	a	a	a
b	b	a	a	b	a	b	a	a	a
b	b	a	a	b	a	b	a	a	a
b	b	b	a	b	a	b	a	a	a
b	b	b	a	b	a	b	a	a	a
b	b	b	a	b	a	b	a	a	a
b	b	a	a	b	a	-	a	a	b
b	b	b	a	b	a	b	a	-	b
b	b	b	a	b	a	b	a	a	b
b	b	b	a	b	a	b	a	a	b
b	b	b	b	b	a	b	a	a	b
b	b	b	a	-	a	b	a	a	b
b	b	b	a	b	a	b	a	a	b
b	b	b	a	b	a	b	a	a	b
b	b	b	a	b	a	b	a	a	b
a	b	b	-	b	a	b	a	a	a
a	b	b	a	b	a	b	a	a	a
a	a	b	-	a	b	b	-	a	a
a	a	b	a	a	b	b	a	a	a
a	a	b	a	a	b	b	a	a	a
a	a	b	a	a	b	b	a	a	a
-	a	b	a	a	b	b	a	a	a
a	a	b	a	a	b	b	a	a	a
a	a	b	a	-	b	b	a	a	a
a	a	b	a	a	b	b	a	a	a
a	a	b	a	a	-	b	a	a	a
a	a	b	a	a	b	b	a	a	a
a	a	b	a	a	b	b	a	a	a
a	a	b	b	a	b	b	a	a	a
a	a	b	a	a	b	b	a	a	a
a	a	b	a	a	b	b	a	a	a
a	a	b	a	a	b	b	a	a	a
a	a	b	a	a	b	b	a	a	a
a	a	b	a	a	b	b	a	a	a
a	a	b	a	a	b	b	a	a	a
a	a	b	a	a	b	-	a	a	a
a	a	b	a	a	b	b	a	a	a
a	a	b	a	a	b	b	a	a	a
a	a	b	a	a	b	b	a	a	a
a	a	b	a	a	b	b	a	a	a
a	a	-	a	a	-	b	a	a	a
a	a	b	a	a	b	b	a	a	a
a	a	b	a	a	b	-	a	a	a
a	a	b	a	a	b	b	a	a	a

-	a	b	-	a	b	b	a	a	a
a	a	b	a	a	b	-	a	a	a
a	a	-	a	a	b	a	a	a	a
a	a	b	a	a	b	-	a	a	a
a	a	-	a	a	b	b	b	a	a
a	a	b	a	a	b	b	b	a	a
a	a	b	a	-	b	b	b	a	a
a	a	b	a	a	b	b	b	a	a
a	a	b	a	a	b	b	b	a	a
a	a	b	a	a	b	b	b	a	a
a	-	b	a	a	b	b	b	a	a
a	a	b	a	a	b	-	b	a	a
a	a	b	a	a	b	b	b	a	a
a	a	b	a	a	b	b	b	a	a
a	a	b	a	a	b	b	b	a	a
a	b	b	a	a	b	-	b	a	a
a	b	b	a	a	b	b	b	a	a
a	b	b	a	a	b	-	b	a	a
-	b	b	a	a	b	b	b	a	a
a	b	b	a	a	b	b	b	a	a
a	b	b	a	a	-	b	b	a	a
a	b	b	a	a	b	b	b	a	a
a	b	b	a	a	b	-	b	a	a
a	b	b	a	a	b	-	b	a	a
-	b	b	a	a	b	-	b	a	a
a	-	b	a	a	b	b	b	a	a
a	b	b	a	a	b	b	b	a	a
-	b	b	a	a	b	b	b	a	a
a	b	b	a	a	b	b	b	a	a
a	b	b	a	a	b	b	b	a	a
-	b	b	a	a	b	b	b	a	a
-	b	b	a	a	b	b	b	a	a
a	-	b	a	a	b	b	b	a	a
a	b	b	a	a	b	b	b	a	a
a	b	-	a	a	b	b	b	a	a
b	b	b	b	b	b	a	b	b	b
b	b	b	b	b	b	a	b	b	b
b	b	b	b	b	b	a	b	b	b
b	b	b	b	b	b	a	b	b	b
b	b	b	b	b	b	a	b	b	b
b	b	b	b	b	b	a	b	b	b
b	b	b	b	b	b	b	b	b	b
b	b	b	b	b	b	b	b	b	b
b	b	b	b	b	b	b	b	b	b
b	b	b	b	b	b	b	b	b	a
b	b	b	b	b	b	b	b	b	a
a	b	-	b	a	b	a	b	b	a

b	b	b	-	a	b	a	b	b	a
b	b	b	-	a	b	b	b	b	a
b	b	b	b	a	b	b	b	b	a
b	b	b	b	a	b	b	b	b	a
-	b	b	b	a	b	-	b	b	a
b	b	b	-	a	b	b	b	b	a
b	b	b	b	a	b	-	b	b	a
b	b	b	b	a	b	b	b	b	a
b	b	b	b	a	b	b	b	b	a
b	b	b	b	a	b	b	-	b	a
b	b	b	b	-	b	b	b	a	a
b	b	b	b	a	b	b	b	-	a
b	b	b	b	a	b	b	b	b	a
b	b	b	b	a	b	b	b	b	a
-	b	b	-	a	b	b	b	b	a
b	b	b	b	a	b	b	b	b	a
b	b	b	b	a	b	b	b	a	a
b	b	b	-	a	b	b	b	a	a
b	b	b	b	a	b	b	b	a	a
b	b	b	b	a	b	-	b	a	a
b	b	b	b	a	a	b	b	a	a
b	b	b	b	a	a	b	b	b	a
-	b	b	b	a	a	b	b	b	a
b	b	b	-	a	a	b	b	b	a
b	b	-	b	a	a	b	b	b	a
b	b	b	b	a	a	b	b	b	a
a	b	b	b	a	a	b	b	b	a
a	b	-	b	a	a	b	b	b	a
-	b	b	b	a	a	-	b	b	a
-	b	b	b	a	a	b	b	b	a
a	b	b	b	a	a	b	b	b	a
a	b	b	b	a	a	b	b	b	a
a	b	b	b	a	a	b	b	b	a
a	b	b	b	a	a	b	b	-	a
a	b	b	b	b	a	b	b	b	a
a	b	b	b	b	a	b	b	b	a
a	b	b	b	b	a	b	b	b	b
a	b	b	b	b	a	b	b	a	b
a	b	b	b	b	a	b	b	b	b
a	b	b	b	b	a	b	b	b	b
a	b	b	-	b	a	-	b	b	b
a	b	-	-	b	a	b	b	b	b
a	b	b	b	b	a	b	b	b	b
a	b	b	b	b	a	b	b	b	b
a	b	b	b	b	a	b	b	b	b
a	b	b	b	b	a	b	b	b	b
a	b	a	b	b	a	a	a	b	a

a	b	a	b	b	a	a	a	b	a
a	a	a	b	b	a	a	a	b	a
a	a	a	b	b	a	a	a	b	a
a	a	a	b	b	a	a	a	b	a
a	a	a	b	b	a	a	a	b	a
a	a	a	b	b	a	a	a	b	a
a	a	a	b	b	a	a	a	b	a
a	a	a	b	b	a	a	a	b	a
a	a	a	b	b	a	a	a	b	a
a	a	a	b	b	a	a	a	b	a
a	a	a	b	b	a	a	a	b	a
a	a	a	b	b	a	a	a	b	a
a	a	a	b	b	a	a	a	b	a
a	a	a	b	b	a	a	a	b	a
a	a	a	b	b	a	a	a	b	a
a	a	a	b	b	a	a	a	b	a
a	a	a	b	b	a	a	a	b	a
a	a	a	b	b	a	a	b	b	a
a	a	a	b	b	a	a	a	b	a
a	a	a	b	b	a	a	a	b	a
a	a	a	b	b	a	a	a	b	a
a	a	a	b	b	a	a	a	b	a
a	b	a	b	b	a	a	a	b	a
a	b	a	b	b	a	a	a	b	a
a	b	a	b	b	a	a	a	b	a
a	b	a	b	b	a	a	a	b	a
a	b	a	b	b	a	a	b	b	a
a	b	a	b	a	a	a	b	b	a
a	b	b	b	a	a	a	b	b	a
a	b	b	b	a	a	a	b	b	a
a	b	b	b	a	a	a	b	b	a
a	b	b	b	a	a	a	b	b	a
a	b	b	b	a	a	a	b	b	a
a	b	b	b	a	a	a	b	b	a
a	b	b	b	a	a	b	b	b	a
a	b	b	b	a	a	b	b	b	a
a	b	b	b	a	a	b	b	b	a
a	b	b	b	a	a	b	b	b	a
a	b	b	b	a	a	b	b	a	a
a	a	b	b	a	a	b	b	a	a
a	a	a	b	b	b	b	a	a	b
a	b	a	a	b	a	a	b	-	a
a	b	a	a	b	a	a	b	b	a
a	b	a	a	b	a	a	b	b	a
a	b	a	a	b	a	a	b	b	a
a	b	a	a	b	a	a	b	b	a
a	b	a	a	b	a	a	b	b	a

a	b	a	a	b	a	a	b	b	a
a	b	a	a	b	a	a	b	b	a
a	b	a	a	b	a	b	a	b	a
a	b	a	a	b	a	b	a	b	a
a	b	a	a	b	a	b	a	b	a

YW019	YW020	YW021	YW022	YW023	YW024	YW025	YW026	YW027	YW028
a	b	a	b	a	-	a	b	a	-
a	b	a	b	a	a	a	b	a	b
a	b	a	b	a	a	a	b	-	b
a	b	a	b	a	a	a	b	a	b
a	b	a	b	a	a	a	b	a	-
a	b	a	b	a	a	a	b	a	b
a	b	a	b	a	a	a	b	a	b
a	b	a	b	a	a	a	b	a	b
a	b	a	b	a	a	a	b	a	b
a	b	a	b	a	a	a	b	a	b
a	b	a	b	a	a	a	b	a	b
a	b	a	b	a	a	a	b	a	b
a	b	a	b	a	a	a	b	a	b
a	b	a	b	a	a	a	b	a	b
a	b	-	b	a	a	a	b	-	b
a	b	a	b	a	a	a	b	a	b
a	b	a	b	a	a	a	b	a	b
a	b	a	b	a	a	a	b	a	b
a	b	a	b	a	a	-	b	a	b
a	b	a	b	a	a	a	a	-	b
a	-	a	b	a	a	a	a	a	b
a	b	a	b	a	a	a	a	a	b
a	b	a	b	a	a	a	a	a	a
a	b	a	b	a	a	a	a	a	a
a	b	a	b	a	-	a	a	a	a
a	b	a	b	a	a	a	a	a	a
a	b	-	b	a	a	a	a	a	a
a	-	a	b	a	a	a	a	a	a
a	b	a	b	a	a	a	a	a	a
a	b	a	b	-	a	a	a	a	a
a	b	a	b	a	a	a	a	a	a
a	b	a	b	a	a	a	a	a	a
a	b	a	b	a	a	a	a	a	a
a	b	a	b	a	a	a	a	a	a
a	b	a	b	-	a	a	a	-	a
a	b	a	b	a	a	a	a	a	a
a	b	a	b	a	a	a	a	a	a
a	b	a	b	a	a	a	a	a	a
a	b	a	b	a	a	a	a	a	a
a	b	a	b	a	a	a	a	a	a
a	b	a	b	a	a	a	a	a	a
a	b	a	b	a	a	a	a	a	a
a	b	a	b	a	a	a	a	a	a
a	b	a	b	a	a	a	a	-	-
a	b	a	b	a	a	a	a	a	-

a	b	a	b	a	a	a	a	a	-
a	b	a	b	a	a	a	a	a	a
a	b	a	b	a	a	a	a	a	a
a	b	a	b	a	a	a	a	a	a
a	b	a	b	a	a	a	a	-	a
a	b	a	b	a	a	a	a	a	a
a	b	a	b	a	b	a	a	a	a
a	b	a	b	a	b	a	-	-	a
a	b	a	b	a	b	a	a	a	a
a	b	a	b	a	b	a	a	a	a
a	b	a	b	a	b	a	a	a	a
a	b	a	b	a	-	a	a	a	a
a	b	a	b	a	-	a	a	a	a
a	b	a	b	a	b	-	a	a	a
a	b	a	b	a	-	a	a	-	a
a	b	a	b	a	b	a	a	a	a
a	b	a	b	a	b	a	a	a	a
a	b	a	b	a	b	a	a	a	a
a	b	a	b	a	b	a	a	a	a
a	b	a	b	a	b	a	a	a	a
a	b	a	b	a	b	a	a	a	a
a	b	a	b	a	b	a	a	a	a
a	b	a	b	a	b	a	a	a	a
a	b	a	b	a	b	a	a	a	a
a	b	a	b	a	b	a	a	a	a
a	b	a	b	a	b	a	a	a	b
a	b	a	b	a	b	a	a	a	b
a	b	a	b	a	-	a	a	a	b
a	b	a	b	a	-	a	a	a	b
a	b	-	b	a	b	a	a	a	b
a	b	a	b	a	b	a	a	a	b
a	b	a	b	a	b	a	a	-	b
a	b	a	b	a	b	a	a	a	b
a	b	a	b	a	b	a	a	a	b
a	b	a	b	a	b	a	a	a	b
a	b	a	b	a	b	a	a	a	b
a	b	a	b	a	b	a	a	a	b
a	b	a	b	a	b	a	a	a	b
a	-	a	b	a	b	a	a	a	b
b	b	b	a	a	b	a	a	a	b
b	b	b	a	a	-	a	a	a	b
b	b	b	a	a	b	a	a	a	b
b	b	-	a	a	b	a	a	a	b
b	b	b	a	a	b	a	a	a	b
b	b	b	a	a	b	a	a	a	b
b	b	b	a	a	b	a	a	a	b
b	b	b	a	a	b	a	a	a	b

b	b	a	a	a	b	a	a	a	b
b	b	-	a	a	b	b	a	a	b
b	b	a	a	a	b	b	a	a	b
b	b	a	a	a	b	b	a	a	b
b	b	a	a	a	b	b	a	a	b
b	b	a	a	a	b	b	a	a	b
b	b	a	a	a	b	b	a	a	b
b	b	a	a	a	b	b	a	a	b
b	b	a	b	a	b	b	a	a	b
b	b	a	b	a	b	b	a	a	b
b	b	a	b	a	b	b	a	a	b
b	b	a	b	a	b	b	a	-	b
b	b	a	b	a	b	b	-	-	b
b	b	a	b	a	b	b	a	a	b
b	b	a	b	a	b	b	a	a	b
b	b	a	b	a	-	b	a	a	b
b	b	a	b	a	b	b	a	a	b
b	b	a	b	a	b	b	a	a	b
b	b	-	b	a	b	b	a	a	b
b	b	a	b	a	b	b	a	a	b
b	b	a	b	a	b	b	a	a	b
b	b	a	b	a	b	b	a	a	b
b	b	a	b	b	b	b	a	a	b
b	b	a	b	b	b	b	a	a	b
b	b	a	b	b	b	b	a	-	b
b	b	a	b	b	b	b	a	a	b
b	b	a	b	b	b	-	a	a	b
b	b	a	b	b	b	a	a	a	b
b	b	-	b	b	b	a	a	-	b
b	b	a	b	b	b	a	a	a	b
b	b	a	b	b	b	a	a	a	b
b	b	a	b	b	b	a	a	a	b
b	b	a	b	b	b	a	a	a	b
b	b	a	b	b	b	a	a	a	b
b	b	a	b	b	-	a	a	a	-
b	b	a	b	b	b	-	-	-	b
b	b	a	b	b	b	a	-	a	b
b	b	a	b	b	b	a	-	-	b
b	b	a	b	b	b	a	a	a	b
b	b	a	b	b	b	a	a	a	b
b	b	a	b	b	b	a	a	a	b
b	b	a	b	b	b	a	a	a	b
b	b	-	b	b	b	a	a	a	b
b	b	a	b	b	b	a	a	a	b
b	b	a	b	b	b	a	a	a	b
b	b	a	b	b	b	a	a	a	b
b	b	a	b	b	b	a	a	a	b

b	b	a	b	b	b	a	a	-	b
b	b	a	b	b	b	a	b	a	b
b	b	a	b	b	a	a	b	a	b
b	b	a	b	b	b	a	b	a	b
b	b	a	b	b	b	a	b	a	b
b	b	-	b	b	b	a	b	-	b
b	b	a	b	b	b	a	b	a	b
b	b	a	b	b	b	a	b	a	b
b	b	a	b	b	b	a	b	a	b
b	b	a	b	b	b	a	b	-	b
b	b	a	b	b	b	a	b	a	b
b	b	a	b	b	b	a	b	a	b
b	b	a	b	b	b	a	b	a	b
b	b	a	b	b	b	a	b	a	b
b	b	a	b	b	b	a	b	a	b
b	b	a	b	b	b	a	b	a	b
b	b	a	b	b	b	a	b	a	b
b	b	a	b	b	b	a	b	a	b
b	b	a	b	b	-	a	b	a	b
b	b	a	b	b	b	a	b	a	b
b	b	a	b	b	b	a	b	a	b
b	b	a	b	b	b	a	b	a	b
b	b	b	b	b	b	a	b	a	b
b	b	b	b	b	-	a	b	-	b
b	b	b	b	b	b	a	b	a	b
b	b	b	b	b	b	a	b	a	b
b	b	b	b	b	b	a	b	a	b
b	b	b	b	b	b	a	b	a	b
b	b	b	b	b	b	a	b	a	b
b	b	b	b	b	b	b	b	a	b
b	b	b	b	b	-	b	b	a	b
-	b	b	b	b	b	b	b	a	b
b	b	b	b	b	b	b	b	a	b
b	b	b	b	b	b	b	b	a	b
b	b	b	b	b	b	b	b	a	b
b	b	b	b	b	b	b	b	a	b
b	b	b	b	b	b	b	b	a	b
b	b	b	b	b	b	b	b	a	b
b	b	b	b	b	b	b	b	a	b
b	b	b	b	b	b	b	b	a	b
b	b	b	b	b	b	b	b	a	b
b	b	b	b	b	b	b	b	a	b
b	b	b	b	b	b	b	b	a	b
b	b	b	b	b	b	b	b	a	b
b	b	b	b	b	b	-	b	a	b
b	b	b	b	b	b	b	b	b	b
b	b	b	b	b	b	b	b	b	b

b	b	-	b	b	b	b	b	b	b
b	b	b	b	b	b	b	b	b	b
b	b	b	b	b	b	b	b	b	b
b	b	b	b	b	b	b	b	b	b
b	b	b	b	b	b	b	b	b	b
b	b	b	b	b	b	b	b	b	b
b	b	b	b	b	b	b	b	b	b
b	b	b	b	b	b	b	b	b	b
b	b	b	b	b	b	b	b	b	b
b	b	b	b	b	b	b	b	b	b
b	b	-	b	b	b	b	b	b	b
b	b	b	b	b	b	b	b	b	b
a	b	a	a	a	b	a	b	a	b
a	b	a	a	a	b	a	b	a	b
a	b	a	a	a	b	a	b	a	b
a	b	a	a	a	b	a	b	a	b
a	b	a	a	a	b	a	b	a	b
a	b	a	a	a	b	a	b	a	b
a	b	a	a	a	b	a	b	a	b
a	b	a	a	a	b	a	b	a	b
a	b	a	a	a	b	a	b	a	b
a	b	a	a	a	b	-	b	a	b
a	b	a	a	a	b	a	b	a	b
a	b	a	a	a	-	a	b	a	b
a	b	b	a	a	b	-	b	a	b
a	b	a	a	a	b	a	b	a	b
a	b	a	a	a	b	a	b	a	-
a	b	a	a	a	b	a	b	a	b
a	b	a	a	a	b	a	b	a	b
a	b	-	b	a	b	b	b	a	b
a	b	a	b	a	b	b	b	a	b
a	b	a	a	a	b	b	b	a	b
a	b	a	a	a	b	b	b	a	b
a	b	a	a	a	b	b	b	a	b
a	b	a	a	a	b	b	b	a	b
a	b	a	a	a	b	b	b	a	b
a	b	a	a	a	b	b	b	-	b
a	b	a	a	a	b	b	b	a	b
a	b	a	a	a	b	b	b	a	b
a	b	a	a	a	b	b	b	a	b
a	b	a	a	a	b	b	b	a	b
a	b	a	a	a	b	b	b	a	b
a	b	a	a	a	b	b	b	a	b
a	b	a	a	a	b	b	b	a	b
a	b	a	a	a	b	b	b	a	b
a	b	a	a	a	b	b	b	a	b
a	b	a	a	a	b	b	b	a	b

a	b	a	a	a	b	b	b	a	b
a	b	a	a	a	b	b	b	a	b
a	b	a	a	a	b	-	a	a	b
a	b	a	a	a	b	b	a	a	b
a	b	a	a	a	b	b	a	a	b
a	b	a	a	a	b	b	a	a	-
a	b	a	a	a	b	b	a	a	a
a	b	a	a	a	b	b	a	a	a
a	b	-	a	a	b	b	a	a	a
a	b	a	a	a	b	b	a	a	a
a	b	a	a	a	b	b	a	a	a
a	b	a	a	a	b	b	a	a	a
a	b	a	a	a	b	b	a	a	a
a	b	a	a	a	b	b	a	a	a
a	b	a	a	a	b	b	a	a	a
a	b	a	a	a	b	b	a	a	a
a	b	a	a	a	b	b	a	a	a
a	b	-	a	a	b	b	a	a	a
a	b	a	a	a	b	b	a	a	a
a	b	a	a	a	b	b	a	-	a
a	b	a	a	a	b	b	a	a	a
a	b	a	a	a	b	b	a	a	a
a	b	a	a	a	b	b	a	-	a
a	b	a	a	a	b	b	a	a	-
a	b	a	a	a	b	b	a	a	a
a	b	a	a	a	b	b	a	a	a
a	b	a	a	a	b	-	a	a	a
a	b	a	a	a	b	b	a	a	a
a	b	a	a	a	b	b	a	a	a
a	b	a	a	a	b	b	a	a	a
a	b	a	a	a	b	b	a	a	a
a	b	a	a	a	-	b	a	a	a
a	b	a	a	a	b	b	a	a	a
a	b	a	a	a	b	b	a	b	a
a	b	a	a	a	b	b	a	a	a
a	b	a	a	a	b	b	a	a	a
a	b	a	a	a	b	b	a	a	a
a	b	a	a	a	b	b	a	a	a
a	b	a	a	a	b	b	a	a	a
a	b	a	a	a	b	b	a	a	a
a	b	a	a	a	b	b	b	a	a
a	b	a	a	a	b	b	b	a	a
a	b	a	a	a	b	b	b	a	a
a	b	a	a	a	b	b	b	a	a
a	b	a	a	a	b	b	b	a	a
a	b	a	a	a	b	a	b	a	a
a	b	a	a	a	a	a	b	a	a

a	b	a	a	a	b	a	b	a	a
a	b	-	a	b	b	a	b	a	a
a	b	a	a	b	b	a	b	a	a
a	b	a	a	b	b	-	b	a	a
a	b	a	a	b	b	a	b	a	a
a	b	a	a	b	b	a	b	a	a
a	b	a	a	b	b	a	b	a	a
a	b	a	a	b	b	a	b	a	-
a	-	a	a	b	b	a	b	a	a
a	b	a	a	b	b	a	b	a	a
a	b	a	a	b	b	a	b	a	a
a	b	a	a	b	b	a	b	a	a
a	b	a	a	b	b	a	b	a	-
a	b	a	a	b	b	a	b	a	a
a	b	a	a	b	b	a	b	a	a
a	b	a	a	b	b	a	b	a	a
a	b	a	a	b	b	a	b	a	a
a	b	a	a	b	b	a	b	a	a
a	b	a	a	b	b	-	b	a	a
a	b	a	a	b	b	a	b	a	a
a	b	a	a	b	b	a	b	-	a
b	b	-	a	a	b	a	b	a	a
b	b	a	a	a	b	a	b	a	a
b	b	a	a	a	b	a	b	a	a
b	b	-	a	a	b	a	b	a	a
b	b	a	a	a	b	a	b	a	a
b	b	a	a	a	b	a	b	a	a
b	b	a	a	a	b	a	b	b	a
b	b	a	a	a	b	a	b	b	a
b	b	a	a	a	b	a	b	b	a
b	b	a	a	a	b	a	b	b	a
b	b	a	a	a	b	a	b	-	a
b	b	a	a	a	b	a	b	b	b
b	b	a	a	a	b	a	b	b	b
b	b	a	a	a	b	a	b	b	b
b	b	a	a	a	b	a	b	b	b
b	b	a	a	a	-	a	b	b	b
b	b	a	a	a	-	-	b	b	b
b	b	a	a	b	b	b	b	b	b
b	b	a	a	b	b	b	b	b	b
b	b	a	a	b	b	b	b	b	b
b	b	a	a	b	b	b	-	b	b
b	b	a	a	b	b	b	b	b	b
b	b	a	a	b	b	b	b	b	b
b	b	a	a	b	b	b	b	b	b
b	b	a	a	b	b	b	b	b	b
b	b	a	a	b	b	b	b	b	b

b	b	a	a	b	b	a	b	b	b
b	b	a	a	b	b	a	b	b	b
b	b	a	a	b	b	a	b	b	b
b	b	b	a	b	b	a	b	b	b
b	b	a	a	b	b	a	b	b	b
b	b	-	a	b	b	a	b	b	b
a	b	a	a	b	b	a	b	b	b
a	b	a	a	b	b	a	a	b	b
a	b	a	a	b	b	a	a	b	b
a	b	a	a	b	b	a	a	b	b
a	b	a	a	b	b	a	a	b	b
a	b	a	a	b	b	a	a	b	b
a	b	a	a	b	b	a	a	b	b
a	b	a	a	b	b	a	a	b	b
a	b	-	a	b	b	a	a	b	b
a	b	-	a	b	b	a	a	-	b
a	b	b	a	b	b	a	a	b	b
a	b	-	a	b	b	a	a	b	b
a	b	b	a	b	b	a	a	b	b
a	b	b	a	b	b	a	a	b	b
a	b	b	a	b	b	a	a	b	b
a	b	b	a	b	b	a	a	b	b
a	b	b	a	b	b	a	a	b	b
a	b	b	a	b	b	a	a	b	b
a	b	b	a	b	b	b	a	b	-
a	b	b	a	b	b	b	a	b	b
a	b	b	a	b	b	b	a	b	b
a	b	b	a	b	b	b	a	b	b
a	b	b	a	b	b	b	a	b	b
a	b	b	a	b	b	b	a	b	b
a	b	b	a	b	b	b	a	b	b
a	b	b	a	b	b	b	a	-	b
a	b	b	a	b	b	b	a	b	b
a	b	b	a	b	b	b	a	b	b
a	b	b	a	b	b	b	a	b	b
a	b	b	a	-	-	b	a	b	b
a	b	a	a	b	b	b	a	b	b
a	b	b	a	b	b	b	a	b	b
a	b	b	a	b	b	b	a	b	b
a	b	b	a	b	b	b	a	b	b
a	b	b	a	b	b	b	a	b	b
a	b	b	a	b	b	b	a	-	b
a	b	b	a	b	a	b	a	b	b
a	b	b	a	b	a	b	-	b	b
a	b	b	a	b	a	b	a	b	b
a	b	b	a	b	a	b	a	b	b
a	b	b	a	b	-	b	a	b	b

a	b	b	a	b	a	b	a	b	b
a	b	-	a	b	a	b	a	b	b
a	b	-	a	b	a	b	a	b	b
a	b	b	a	b	a	b	a	b	b
a	b	b	a	b	a	b	a	-	b
a	a	b	a	b	a	a	a	b	b
a	a	b	a	b	a	a	a	b	b
a	a	b	a	b	a	a	a	b	b
a	a	b	a	b	a	a	a	b	b
a	a	b	a	b	a	a	a	b	b
a	a	b	a	-	a	a	a	b	-
a	a	b	a	b	a	a	a	b	b
a	a	b	a	b	a	a	a	b	b
a	a	b	a	b	a	a	a	b	b
a	a	b	a	b	a	a	a	b	b
a	a	b	a	b	a	a	a	b	b
a	a	b	a	b	a	a	b	a	b
a	a	b	a	b	a	a	b	a	b
a	a	b	a	b	a	a	b	a	b
a	a	b	a	b	a	a	b	a	b
a	a	b	a	b	a	a	b	a	b
a	a	b	a	b	a	a	b	a	b
a	a	b	a	a	a	a	b	a	-
a	a	b	a	a	a	a	b	a	b
a	a	b	a	a	a	-	b	a	b
a	a	b	a	a	a	a	b	a	b
a	a	b	a	a	a	a	b	a	a
a	a	b	a	a	a	a	b	a	a
a	a	b	a	a	a	a	b	a	a
a	a	b	a	a	a	a	b	a	a
a	a	b	a	a	a	a	b	a	a
a	a	b	a	a	a	a	b	a	a
a	a	b	a	a	a	-	b	a	a
a	a	b	a	a	a	a	b	a	a
a	a	b	a	a	a	a	b	a	a
a	a	b	a	a	a	b	b	a	a
a	a	b	a	a	a	b	b	a	a
a	a	b	a	a	-	b	b	-	a
a	a	b	a	a	a	b	b	a	a
a	a	-	a	a	a	b	b	a	a
b	a	-	b	a	b	a	a	b	a
b	a	b	b	a	b	a	a	b	a
b	a	b	b	a	b	a	a	b	a
b	a	b	b	a	b	a	a	b	a
b	a	b	b	a	b	a	a	b	a
b	a	b	b	a	b	a	b	b	a
b	a	b	b	a	b	a	b	b	a

b	a	b	b	a	b	a	b	b	a
b	a	b	b	a	b	a	b	b	a
b	a	b	b	a	-	a	b	b	a
b	a	b	b	a	b	a	b	b	a
b	a	b	b	a	b	a	b	b	a
b	a	b	b	a	b	b	b	b	a
b	a	b	b	a	b	b	b	b	a
b	a	b	b	a	-	b	b	b	a
b	a	b	b	a	b	b	b	b	a
b	a	b	b	a	b	b	b	b	a
b	a	b	b	a	b	b	b	b	a
b	a	b	b	a	b	b	b	b	a
b	a	b	b	a	b	b	b	b	a
b	a	b	b	a	b	b	b	b	a
b	a	b	b	a	b	b	b	b	a
b	a	b	b	a	b	b	b	b	a
b	a	b	b	a	b	b	b	b	a
b	a	b	b	a	b	b	b	b	a
b	-	b	b	a	b	b	b	b	a
b	a	b	b	a	b	b	b	b	a
b	a	b	b	a	-	b	b	b	a
b	a	b	a	a	b	b	b	b	a
b	a	b	a	a	b	b	b	b	a
b	a	b	a	a	-	b	b	b	a
b	a	a	a	a	b	b	b	b	a
b	a	b	a	a	b	b	b	b	a
b	a	b	a	b	b	b	b	-	a
b	a	b	a	b	b	b	b	b	a
b	a	b	a	b	b	b	b	b	a
b	a	b	a	b	b	b	b	b	a
b	a	b	a	b	b	b	b	b	a
b	a	b	a	b	b	b	b	b	a
b	a	b	a	b	b	b	b	b	a
b	a	b	a	b	-	-	b	-	a
b	a	b	a	b	b	b	b	-	a
b	a	b	a	b	b	b	b	b	a
b	a	b	a	b	b	b	b	b	-
b	a	b	a	b	b	b	b	b	a
b	a	b	a	b	b	b	b	b	a
b	a	b	a	b	b	b	b	b	a
b	a	b	a	b	b	b	b	-	a
b	a	b	a	b	-	b	b	b	a
b	a	b	a	b	b	b	b	b	a
b	a	b	a	b	-	b	b	b	a
b	a	b	a	b	b	b	b	b	a
b	a	b	a	b	b	b	b	b	a
b	a	b	a	b	b	b	b	b	a

b	a	b	a	b	b	b	b	b	a
b	a	b	a	b	b	b	b	b	a
b	a	b	a	b	b	b	b	b	a
b	a	b	a	b	b	b	b	b	a
b	a	b	a	b	b	b	b	b	a
b	a	a	a	b	b	b	b	-	a
b	a	b	a	b	b	b	b	b	a
-	a	b	a	b	b	b	b	b	a
b	a	b	a	b	b	b	b	b	a
b	a	b	a	b	-	b	b	b	a
b	-	b	a	b	b	b	b	b	a
b	a	b	a	b	b	b	b	b	a
b	a	b	a	b	-	b	b	b	a
b	a	b	a	b	b	b	b	b	a
b	a	b	a	b	b	b	b	b	a
b	a	b	a	b	b	b	b	b	a
b	a	b	a	b	b	b	b	b	a
b	a	b	a	b	b	b	b	b	a
b	a	b	a	b	b	b	b	b	a
b	a	-	a	b	b	b	b	-	a
b	a	b	a	b	b	b	b	b	a
b	a	b	a	b	b	b	b	b	a
b	a	b	a	b	b	b	b	b	a
b	a	b	a	b	b	b	b	b	a
b	a	b	a	a	b	b	b	b	b
b	a	b	a	a	b	b	b	b	b
b	b	b	a	a	b	b	b	b	b
b	a	b	a	a	b	b	b	b	b
b	a	b	a	a	b	b	b	b	b
b	a	b	a	a	b	b	b	b	b
b	a	b	a	a	b	b	b	b	b
b	a	b	a	a	b	b	a	b	b
b	a	b	a	a	b	b	a	b	b
b	a	-	a	a	b	b	a	b	b
b	a	b	a	a	b	b	a	b	b
b	a	b	a	a	b	b	a	b	b
b	a	b	a	a	b	b	a	b	b
b	a	b	a	a	b	b	a	b	b
b	a	b	a	a	b	b	a	b	b
b	a	b	a	a	b	b	a	b	b
b	a	b	a	a	-	b	a	b	b
b	b	-	a	a	b	b	a	b	b
b	b	-	a	a	b	b	a	b	b
b	-	b	a	a	b	b	a	b	b
b	b	b	a	a	b	b	a	b	b
b	b	b	a	a	b	b	a	b	b
b	b	-	a	a	b	b	a	b	b

b	b	b	a	a	b	b	a	b	b
b	b	b	a	a	b	b	a	b	b
b	b	b	a	a	b	b	a	b	b
b	b	b	a	a	b	b	a	b	b
b	b	b	a	a	b	b	a	b	b
b	b	b	b	a	b	b	a	b	b
b	b	b	b	a	b	b	a	b	b
b	b	b	b	a	b	b	a	b	b
b	b	b	b	a	b	b	a	b	b
b	b	b	b	a	b	b	a	b	b
b	b	b	b	a	b	b	a	b	b
b	b	b	b	a	b	b	a	b	b
b	b	b	b	a	b	b	a	b	b
b	b	b	b	a	b	b	a	b	b
b	b	b	b	a	b	b	a	b	b
b	b	b	b	a	b	b	a	b	b
b	b	b	b	a	b	b	a	b	b
b	b	b	b	a	b	b	a	b	b
b	b	b	b	a	b	b	a	b	b
b	b	b	a	a	b	b	b	a	a
b	b	b	a	-	-	b	b	a	a
b	b	b	a	a	b	b	b	a	a
b	b	b	a	a	b	b	b	a	a
b	b	b	a	a	b	b	b	a	a
b	b	b	a	a	b	b	b	a	a
b	b	b	a	a	b	b	b	a	a
b	b	b	a	a	b	b	b	a	a
b	b	b	a	a	b	b	b	a	a
b	b	b	a	a	b	b	b	a	a
b	b	b	a	a	b	b	b	a	a
b	b	b	a	b	b	b	b	a	a
b	b	b	a	b	-	b	b	a	a
b	b	b	a	b	b	b	b	a	a
b	b	b	a	b	b	b	b	a	a
b	b	b	a	b	b	b	b	a	a
b	b	b	a	b	b	b	b	a	a
b	b	b	a	a	-	b	b	a	a
b	b	b	a	a	b	b	b	a	a
b	b	b	a	a	b	b	b	b	a
b	b	b	a	a	b	b	b	b	a
b	b	b	a	a	b	-	b	b	a
b	b	b	a	a	-	b	b	b	a
b	b	b	a	a	b	b	b	b	a
b	b	b	a	a	b	b	b	b	a
b	b	b	a	a	b	b	b	b	a
b	b	b	a	a	b	b	b	b	a
b	b	b	a	a	b	b	b	b	a

b	b	b	a	a	b	b	b	b	a
b	b	b	a	a	b	b	b	b	a
b	b	b	a	a	-	b	b	b	a
b	b	b	a	a	b	b	b	a	b
b	b	b	a	a	b	b	b	b	a
b	b	b	a	a	b	b	b	b	a
b	b	b	a	a	b	b	b	b	a
b	b	b	a	a	b	b	b	b	a
b	b	b	a	a	b	b	b	b	a
b	b	b	a	a	b	b	b	b	a
b	b	b	a	a	b	b	b	b	a
b	b	b	a	a	b	b	b	b	a
b	b	b	a	a	b	b	-	b	a
b	b	b	a	a	b	b	b	b	a
b	b	b	a	a	b	b	b	b	a
b	b	b	a	a	b	b	b	b	a
b	b	b	a	a	b	b	b	b	a
b	b	b	a	a	b	b	b	b	a
b	b	b	a	a	-	b	b	b	a
b	b	b	a	a	b	b	b	b	a
b	b	b	a	a	b	b	b	b	a
b	b	b	a	a	b	b	b	b	a
b	b	b	a	a	b	b	b	b	a
b	b	b	a	a	b	b	b	b	a
b	b	b	a	a	b	b	b	b	a
b	b	b	a	a	-	b	b	b	a
b	b	b	a	a	b	b	b	b	b
b	b	b	a	a	b	b	b	b	b
b	b	b	a	a	b	b	b	b	b
b	b	b	a	a	b	b	b	b	b
b	b	b	a	a	-	b	b	b	b
b	b	b	a	a	b	b	b	b	b
b	b	b	a	a	b	b	b	b	b
b	b	b	a	a	b	b	b	b	b
b	b	b	a	a	b	b	b	b	b
b	b	b	a	a	b	b	b	b	b
a	b	a	a	a	a	b	b	b	b
a	b	a	a	a	a	b	b	b	b
a	b	a	a	a	a	b	b	b	b
a	b	a	a	a	a	b	b	-	b
a	b	a	a	a	a	b	b	b	b
a	b	a	a	a	a	-	b	b	b
a	b	a	a	a	a	b	-	b	b
a	b	a	a	a	a	b	b	b	b
a	b	a	a	a	a	b	b	b	b
a	b	a	a	a	a	-	b	b	b
a	b	-	a	a	a	b	b	b	b

a	b	a	a	a	a	b	b	b	b
a	b	a	a	a	a	b	b	b	b
a	b	a	a	a	a	b	b	b	b
a	b	a	a	a	a	b	b	b	b
a	b	a	a	a	a	b	b	b	b
a	b	a	a	a	a	b	b	b	b
a	b	a	a	a	a	b	-	b	b
a	b	a	a	a	a	b	b	b	b
a	b	a	a	a	a	-	b	b	b
a	b	a	a	a	a	b	b	b	b
a	-	a	a	a	a	b	b	b	b
a	b	a	b	a	b	b	a	b	b
a	b	-	b	a	b	b	-	b	b
a	b	a	b	a	b	b	a	b	b
a	b	a	b	a	b	b	a	-	b
a	b	a	b	a	b	b	a	b	b
a	b	a	b	a	b	b	a	b	b
a	b	a	b	a	b	b	a	b	b
a	b	a	b	a	b	-	a	b	b
a	b	a	b	a	b	-	a	b	b
a	b	a	b	a	b	b	a	b	b
a	b	a	b	a	b	b	a	b	b
a	b	a	b	a	b	b	a	b	b
a	b	a	b	a	b	b	a	b	b
a	b	a	b	a	b	b	a	b	b
a	b	a	b	a	b	b	a	b	b
a	b	a	b	a	b	b	a	b	b
a	b	a	b	a	b	b	a	b	b
a	b	a	b	a	b	b	a	b	b
a	b	a	b	a	b	b	a	b	b
a	b	a	b	a	b	b	a	b	b
a	b	a	b	a	b	b	a	b	b
a	b	a	b	a	b	b	a	b	b
a	b	-	b	a	b	b	a	b	b
a	b	a	b	a	b	b	a	b	b
a	b	a	b	a	b	a	a	-	b
a	b	a	b	a	b	a	a	b	b
a	b	a	b	a	b	a	a	b	b
a	b	a	b	a	b	b	a	b	b
a	b	a	b	a	b	a	a	b	b
a	b	a	b	a	b	b	a	b	b
a	b	a	b	a	b	b	a	b	b
a	b	a	b	a	b	b	a	b	b
a	b	-	b	a	b	a	a	b	b
a	b	a	b	a	b	a	a	b	b
a	b	a	b	a	b	a	a	b	b
a	b	a	b	a	-	a	a	b	b

a	b	b	b	a	b	a	a	b	b
a	b	a	b	a	b	a	a	b	b
a	b	a	b	a	b	a	a	b	b
a	b	a	b	a	b	a	a	b	b
a	b	a	b	a	b	a	a	b	b
a	b	a	a	a	b	b	a	b	b
a	b	a	a	a	b	b	a	b	b
a	b	a	a	a	b	b	a	b	b
a	b	a	a	a	b	b	a	b	b
a	b	a	a	a	b	b	a	b	b
b	b	a	a	a	b	b	a	b	b
b	b	a	a	a	b	b	a	b	b
b	b	a	a	a	b	b	a	b	b
b	b	a	a	a	b	-	a	b	b
b	b	a	a	a	b	b	a	b	b
b	b	a	a	a	b	b	a	b	b
b	b	a	a	a	b	b	a	-	b
b	b	a	a	a	b	b	a	b	b
b	b	a	a	a	b	b	a	b	-
b	b	a	a	a	b	-	a	b	b
b	b	a	a	a	b	b	a	b	b
b	b	a	a	a	b	b	a	b	b
b	b	a	a	a	b	b	a	b	b
b	b	a	a	a	b	b	a	b	b
b	b	a	a	a	b	b	a	b	b
b	b	a	a	a	b	b	a	b	b
b	b	a	a	a	b	-	a	-	-
b	b	a	a	a	b	b	a	b	b
b	b	a	a	a	-	b	a	b	b
b	b	a	a	a	b	b	a	b	b
b	b	a	a	a	b	b	a	-	b
b	b	a	a	a	b	b	a	b	b
b	b	a	a	a	b	b	a	b	b
b	b	a	a	a	b	b	a	b	b
b	b	a	a	a	b	b	a	b	b
b	a	a	a	a	b	b	a	b	b
b	a	a	a	a	b	b	a	b	b
b	a	a	a	a	b	b	a	b	b
b	a	a	a	a	-	b	a	b	b
b	a	a	a	a	b	b	a	b	b
b	a	a	a	a	b	b	a	b	b
b	a	a	a	a	b	b	a	b	b
b	a	a	a	a	b	b	a	b	b
b	a	a	b	a	b	b	a	b	b
b	a	a	b	a	b	b	a	b	b
b	a	a	b	a	b	b	a	b	b
b	a	a	b	a	b	b	a	b	b

b	a	a	b	a	b	b	a	b	b
b	a	a	b	a	b	b	a	b	b
b	a	-	b	a	b	b	a	b	b
b	a	a	b	a	b	b	a	b	b
b	a	a	b	a	b	b	a	b	b
b	a	a	b	a	b	b	a	b	b
b	a	a	b	a	-	b	a	b	b
b	a	a	b	a	-	b	a	b	b
b	a	a	b	a	b	b	a	b	b
b	a	a	b	a	-	b	a	b	b
b	a	a	b	a	b	b	a	b	b
b	a	a	b	a	b	b	a	b	b
b	a	a	b	a	b	b	a	b	b
b	a	a	b	a	b	b	a	b	b
b	a	a	b	a	b	b	a	b	b
b	a	a	b	a	b	b	a	b	b
b	a	a	b	a	b	b	a	b	b
b	a	a	b	a	b	b	a	b	b
b	a	-	b	a	b	b	-	b	b
b	a	-	b	a	b	b	a	b	b
b	a	a	b	a	b	b	a	b	-
b	a	a	b	a	b	b	a	b	b
a	a	a	b	a	b	b	a	a	a
a	a	a	b	a	b	b	a	b	a
a	a	a	b	a	b	b	a	a	a
a	a	a	b	a	b	b	a	a	a
a	a	a	b	a	-	b	a	a	a
a	a	a	b	a	b	b	a	a	a
a	a	a	b	a	b	b	a	a	a
a	a	a	b	a	b	b	a	a	a
b	b	b	b	a	b	b	a	a	a
b	b	-	b	a	b	b	a	a	a
b	b	b	b	a	b	b	a	a	a
b	b	b	b	a	b	b	a	a	a
b	b	b	b	a	b	b	a	-	a
b	b	b	b	a	b	b	a	b	a
b	b	b	b	a	b	b	a	b	a
b	b	b	b	a	b	b	a	b	a
b	b	b	b	a	b	b	a	b	a
b	b	b	b	a	b	b	a	b	a
b	b	b	b	a	b	b	a	b	a
b	b	b	b	a	b	b	a	b	a
b	b	b	b	a	b	-	a	b	a
b	b	b	b	a	b	b	a	b	a
b	b	b	b	a	b	b	a	b	a
b	b	b	b	a	b	b	a	b	a
b	b	b	b	a	b	b	a	b	a

b	b	b	b	a	b	a	a	b	a
b	b	b	b	a	b	a	a	b	a
b	b	b	b	a	b	a	a	b	a
b	b	a	b	b	-	a	a	b	a
b	b	a	b	b	b	a	a	b	a
b	b	a	b	b	-	a	a	-	a
b	b	a	b	b	b	a	a	b	a
b	b	a	b	b	b	a	a	b	a
b	b	a	b	b	b	a	a	b	a
b	b	a	b	b	b	a	a	b	a
b	b	a	b	b	b	a	a	b	-
b	b	a	b	b	b	a	a	b	a
b	b	a	b	b	b	a	a	-	a
b	b	a	b	b	b	a	a	b	a
b	b	a	b	b	b	a	a	b	a
b	b	a	b	a	b	a	a	b	a
b	b	a	b	b	b	a	a	b	a
b	b	a	b	a	b	a	a	b	a
b	b	a	b	a	b	a	a	b	a
b	b	a	b	b	b	a	a	b	a
b	b	a	b	a	b	a	a	b	a
b	b	a	b	a	b	a	a	b	a
b	b	a	b	a	b	a	a	b	b
b	b	a	b	a	b	a	a	b	b
b	b	a	b	a	b	a	a	b	b
b	b	a	b	b	b	a	a	b	b
b	b	-	b	a	b	a	a	b	b
b	b	a	b	a	b	b	a	-	b
b	b	a	b	a	b	b	a	a	b
b	b	a	b	b	b	b	a	a	b
b	b	-	b	a	b	b	a	a	b
b	b	a	b	b	b	b	a	a	b
b	b	-	b	a	b	b	a	a	b
b	b	a	b	a	b	b	a	a	b
b	b	a	b	a	b	b	a	a	b
b	b	a	b	a	b	b	a	a	b
b	b	a	b	a	b	b	a	a	b
b	b	-	b	a	b	b	a	a	b
b	b	a	b	a	b	b	a	a	b
b	b	-	b	a	b	b	a	a	b
b	b	a	b	b	b	b	a	a	b
b	b	a	b	b	b	b	a	a	b
b	b	a	b	b	b	b	a	a	b
b	b	a	b	b	b	b	a	-	b
b	b	a	b	a	b	b	a	a	b
b	b	a	b	b	b	b	a	a	b
b	b	a	b	a	b	b	a	a	b

b	b	a	b	a	b	b	a	a	b
b	b	a	b	a	b	b	a	a	b
b	b	a	b	b	b	-	-	a	b
b	b	a	b	b	b	b	b	a	b
b	b	b	b	a	b	b	b	a	b
b	b	a	b	a	b	b	b	a	b
b	b	a	b	a	b	b	b	a	b
b	b	a	b	a	b	b	b	-	b
b	b	-	b	a	b	b	b	a	b
b	b	a	b	a	b	b	b	a	b
b	b	a	b	b	b	b	b	a	b
b	b	a	b	a	b	b	b	a	b
b	b	a	b	b	b	b	b	a	b
b	b	a	b	a	b	b	b	a	b
b	b	a	b	a	b	b	b	a	b
b	b	a	b	b	b	b	-	a	b
-	b	a	b	b	b	b	b	a	b
b	b	a	b	a	b	b	b	a	b
b	b	a	b	a	b	b	b	a	b
b	b	a	b	b	b	a	b	b	b
b	b	a	b	a	b	b	b	b	b
b	b	a	b	b	b	b	b	b	b
b	b	a	b	a	b	b	b	b	b
b	b	b	a	a	b	b	b	b	b
b	b	a	a	a	b	b	b	b	b
b	b	a	a	a	b	b	b	b	b
b	b	a	a	a	b	b	b	b	b
b	b	a	a	a	b	-	b	b	-
b	b	a	a	a	b	b	b	b	a
b	b	a	a	a	b	b	b	b	a
b	b	a	a	a	b	b	b	b	a
b	b	a	a	a	b	b	b	b	a
b	b	-	a	a	b	b	b	b	a
b	b	a	a	a	b	b	b	b	a
b	b	a	a	a	b	b	b	b	a
b	b	a	a	a	b	b	b	b	a
b	b	a	a	a	b	b	b	b	a
b	b	a	a	a	b	b	b	b	a
b	b	a	a	a	b	b	b	b	a
b	b	a	a	a	b	-	b	b	a
b	b	a	a	a	b	b	b	b	a
b	b	a	a	a	b	b	b	b	a
b	b	a	a	a	b	b	b	b	a
b	b	a	a	a	b	b	b	b	a
b	b	a	a	a	b	-	b	b	a
b	b	a	a	a	b	b	b	b	a
b	b	a	a	a	b	b	b	-	a

b	b	a	a	a	b	b	b	b	a
b	b	a	a	a	b	-	b	b	a
b	a	a	a	a	a	a	a	a	a
a	b	b	a	b	b	a	b	a	b
a	b	b	a	b	b	-	b	a	b
a	b	b	a	b	b	a	b	b	b
a	b	a	a	b	b	a	b	b	b
a	b	a	a	b	b	a	b	b	b
a	b	a	a	b	b	a	b	b	b
a	b	a	a	b	b	a	b	-	b
a	a	a	a	b	b	a	b	b	b
a	a	a	a	b	b	a	b	b	b
a	a	a	a	b	b	a	b	b	b
a	a	a	a	b	b	a	b	b	b
a	a	a	a	b	b	a	b	b	b
a	a	a	a	b	b	a	b	b	b
a	a	a	a	b	b	a	b	b	b
a	a	a	b	b	b	a	b	b	b
a	a	a	b	b	b	a	b	b	b
a	a	a	a	b	b	a	b	b	b
a	a	a	a	b	b	a	b	b	b
a	a	a	a	b	b	a	b	-	b
a	a	a	a	b	b	-	b	b	b
a	a	a	a	b	b	a	b	b	-
a	a	a	a	b	b	a	b	b	b
a	a	a	a	b	b	a	b	b	b
a	a	a	a	b	b	a	b	b	b
a	a	a	a	b	b	a	b	b	b
a	a	a	a	a	b	b	b	b	b
a	a	a	a	a	b	b	b	b	b
a	a	a	a	a	b	b	b	b	b
a	a	b	a	a	b	b	b	b	b
a	a	a	a	a	b	b	b	b	b
a	a	a	a	a	b	b	b	b	b
a	a	a	a	a	b	b	b	b	b
a	a	a	a	a	b	b	b	b	b
a	a	a	a	a	b	b	-	b	b
a	b	a	a	a	b	b	b	b	b
a	b	-	a	a	a	-	b	b	b
a	b	a	a	a	-	-	b	b	b
a	b	a	a	a	a	-	b	b	b
a	b	a	a	a	a	a	b	b	b
a	b	a	a	a	-	a	b	b	b
a	b	a	a	a	a	a	b	b	b
a	b	a	a	a	a	a	b	b	b
a	b	a	a	a	a	a	b	b	b
a	b	a	a	a	a	a	b	b	b

a	b	a	a	a	a	a	b	b	b
a	b	a	a	a	a	a	-	b	b
a	b	a	b	b	a	a	b	b	b
b	b	a	b	b	a	a	b	b	b
a	a	a	a	b	b	b	a	a	a
a	a	a	a	b	b	b	a	a	a
a	a	-	a	b	b	b	a	a	a
a	a	a	a	b	b	b	a	a	a
a	a	a	a	b	b	b	a	a	a
a	a	a	a	b	b	b	a	a	a
a	b	a	a	b	b	b	a	a	a
a	-	a	a	b	b	b	a	a	a
a	b	b	a	b	a	b	a	a	a
a	b	b	a	b	a	b	a	a	a
a	b	-	a	b	a	b	a	a	a
a	b	b	a	b	a	b	a	a	a
a	b	b	a	b	a	b	a	a	a
a	b	b	a	b	a	b	a	a	a
a	b	b	a	b	a	b	a	-	-
a	b	b	a	b	a	b	a	a	a
a	b	b	a	b	a	b	a	a	a
a	b	b	a	b	a	b	a	a	a
a	b	b	a	b	a	b	a	a	a
a	b	b	a	b	a	b	a	a	a
a	b	b	a	b	a	b	a	b	a
a	b	a	a	a	a	b	a	a	a
a	b	a	a	a	a	b	a	a	a
a	b	a	a	a	a	b	a	b	b
a	b	a	a	a	a	b	a	b	b
a	b	a	a	a	b	b	a	b	b
a	b	a	a	a	b	b	a	b	b
a	b	a	a	a	b	b	a	b	b
a	b	a	a	a	b	b	a	b	b
a	b	a	a	a	b	b	b	b	b
a	b	a	a	a	b	b	b	b	b
a	b	a	a	a	b	b	b	b	b
a	b	a	a	a	b	b	b	b	b
a	b	a	a	a	b	b	b	b	b
-	b	b	a	a	b	b	b	b	b
a	b	b	a	a	b	b	b	b	b
a	b	b	a	a	a	b	b	b	b
a	b	b	a	a	a	b	b	b	b
a	a	b	a	a	a	b	b	b	b
a	b	b	a	a	a	b	b	b	b
a	-	-	a	a	a	b	b	b	b
b	b	a	b	b	b	a	b	a	b
b	b	a	b	b	b	a	b	a	b

b	b	a	b	b	b	a	b	a	b
b	b	a	b	b	-	a	-	a	b
b	b	a	b	b	-	a	b	a	b
b	b	a	b	b	b	a	b	a	b
b	b	a	b	b	b	a	b	a	b
b	b	a	b	b	b	a	b	a	b
b	b	a	b	b	b	a	b	a	b
b	b	-	a	b	b	a	-	a	b
b	b	b	a	b	b	a	b	a	b
b	b	b	a	b	b	a	b	a	b
b	b	b	a	b	b	a	b	b	b
b	b	b	a	b	b	a	b	b	b
b	b	b	a	b	b	a	b	-	b
b	b	b	a	b	b	a	b	b	b
b	a	b	a	b	b	a	b	b	b
b	a	b	a	b	b	a	b	b	b
b	a	b	a	b	b	a	a	b	b
b	a	b	a	b	b	a	a	b	b
b	a	b	a	b	-	a	a	b	b
b	a	b	a	b	b	a	a	b	b
b	a	b	a	b	b	a	a	b	b
b	a	b	a	b	-	a	-	b	b
b	a	b	a	b	b	a	a	b	b
b	a	b	a	b	b	a	-	b	b
b	a	b	a	b	-	a	a	b	b
b	a	-	a	b	b	a	a	b	-
b	a	b	a	b	a	a	a	b	b
b	a	b	a	b	a	a	a	b	b
b	a	b	a	b	a	a	a	b	b
b	a	b	a	b	a	-	a	b	b
b	a	b	a	b	a	a	a	b	b
b	a	b	a	b	a	a	a	b	b
b	a	b	a	b	a	-	a	b	b
b	a	b	a	b	a	b	a	-	b
b	a	b	a	b	a	b	a	b	b
b	a	b	a	b	b	b	a	b	b
b	a	b	a	b	b	b	a	b	b
b	a	b	a	b	b	b	a	b	b
b	a	b	a	b	b	b	a	b	b
b	a	b	a	b	b	-	a	-	b
b	a	-	a	b	b	b	a	b	b
b	a	-	a	b	b	b	a	b	b
-	a	a	a	b	b	b	a	b	b
b	a	a	a	b	b	b	a	b	b
b	a	a	a	b	b	b	a	b	b
b	a	a	a	b	b	b	a	b	b
b	a	a	a	b	b	b	a	b	b

b	a	a	a	b	b	b	a	b	b
b	a	a	a	b	b	b	a	b	b
b	a	a	a	b	b	-	a	b	b
b	a	a	a	b	b	b	a	b	-
b	a	a	a	b	b	b	a	b	b
b	a	a	a	b	b	b	a	b	b
b	a	a	a	b	b	b	a	b	b
b	a	a	a	b	b	b	a	a	b
b	a	a	a	b	b	b	a	b	b
b	a	a	a	b	b	b	a	b	b
b	a	a	a	b	b	-	a	b	-
b	a	a	a	b	b	b	a	b	b
b	a	a	a	b	b	b	a	b	b
b	a	a	a	b	b	b	a	b	-
b	a	a	a	b	b	-	a	b	b
b	a	a	a	b	b	b	a	b	b
b	a	-	a	b	b	b	a	b	b
b	a	a	a	b	b	b	a	b	b
b	a	a	a	b	b	b	a	b	b
b	a	a	a	b	b	b	a	b	b
b	a	a	a	b	-	b	a	-	b
b	a	a	a	b	b	b	a	-	b
b	a	a	a	b	b	b	a	a	b
b	a	a	a	b	b	b	a	a	b
b	a	a	a	b	b	b	a	a	b
b	a	a	a	b	b	b	a	a	a
b	a	a	a	b	-	b	a	a	a
b	a	a	a	b	b	b	a	a	a
b	a	a	a	b	b	b	a	a	a
b	a	a	a	b	b	-	a	a	a
b	a	a	a	b	b	b	a	a	a
b	a	a	a	b	b	b	a	a	a
b	a	-	a	b	b	b	a	a	a
b	a	a	a	b	b	b	a	a	a
b	a	a	a	b	b	b	a	-	a
b	a	a	a	b	b	b	a	a	a
b	a	a	a	b	b	b	a	a	a
b	a	a	a	b	b	b	a	a	a
b	a	a	a	b	b	b	a	a	a
b	a	a	a	b	a	b	a	a	a
b	a	a	a	b	a	b	a	a	a
b	a	a	a	b	a	b	a	a	a
b	a	a	a	b	b	b	a	a	a
b	a	a	a	b	b	b	a	a	a
b	a	a	b	b	b	a	a	a	a
b	a	a	a	b	b	a	a	a	a
a	a	a	a	b	b	a	-	-	a

a	a	a	a	b	b	a	a	a	a
a	a	a	a	b	b	a	a	a	a
a	a	a	a	a	b	a	a	a	a
a	a	a	a	a	b	a	a	-	a
a	a	a	a	a	b	a	a	a	a
a	a	a	a	a	b	-	a	-	a
a	a	-	a	a	b	a	a	a	a
a	a	a	a	a	b	a	a	a	a
b	a	b	a	b	b	-	a	b	b
b	a	b	a	b	b	a	a	b	b
b	a	b	a	b	b	a	a	b	b
b	a	b	a	b	b	a	a	b	b
b	a	b	a	b	b	a	a	b	b
a	a	b	b	b	b	a	a	-	b
a	a	-	b	b	b	a	a	b	b
a	a	b	b	b	b	a	a	-	b
a	a	b	b	b	b	a	a	b	b
a	a	b	b	b	b	a	a	b	b
a	a	b	b	b	b	a	a	b	b
a	a	b	b	b	b	a	a	b	a
a	a	b	b	b	b	a	a	b	a
a	a	b	b	b	b	a	a	b	a
a	a	b	b	b	b	a	a	b	a
a	a	b	b	b	b	a	a	b	a
a	a	b	b	b	b	b	a	-	a
a	a	b	b	b	b	b	a	b	a
a	a	b	b	b	b	b	a	b	a
a	a	b	b	b	a	b	a	b	a
a	a	b	b	b	a	b	a	b	a
a	a	b	b	b	a	b	a	b	a
a	a	b	a	b	a	b	a	b	a
a	a	b	a	b	a	b	a	b	a
a	a	b	a	b	b	b	a	b	a
a	a	b	a	b	b	b	a	b	a
a	a	b	a	b	b	b	a	b	a
a	a	b	a	b	b	b	a	b	a
a	a	b	a	b	b	b	a	b	a
a	a	b	a	b	b	b	a	b	a
a	a	b	a	b	b	b	a	b	a
a	a	b	a	b	b	b	a	b	a
a	a	b	a	b	b	b	a	b	a
a	a	b	a	b	b	b	a	b	a
a	a	a	a	b	b	b	a	b	a
a	b	a	a	b	b	b	a	a	a
a	b	a	a	b	b	b	a	a	a
a	b	a	a	b	b	b	a	a	a
a	b	-	a	b	b	b	a	a	a

a	b	a	a	b	b	b	a	a	a
a	b	a	a	b	b	b	a	a	a
a	b	a	a	a	b	b	a	a	a
a	b	a	a	a	b	b	a	a	a
a	b	a	a	a	b	b	a	a	a
a	b	a	a	a	a	b	b	a	a
a	b	a	a	a	a	b	b	a	a
a	b	a	a	a	a	b	b	a	a
a	b	-	a	a	a	b	b	a	a
a	b	a	a	a	a	b	b	a	a
a	b	a	a	a	a	b	b	a	a
a	b	-	a	a	a	b	b	a	a
b	b	a	b	b	b	a	-	b	a
b	b	a	b	b	b	a	a	b	a
b	b	a	b	b	b	a	a	b	a
b	b	a	b	b	b	a	a	b	a
b	b	a	b	b	b	a	a	b	a
b	b	a	b	b	b	a	a	b	a
b	a	a	b	b	b	a	a	b	a
b	a	a	b	b	b	a	a	b	a
b	a	a	b	b	b	a	a	b	a
b	a	a	b	b	b	-	a	b	a
b	a	a	b	b	b	a	a	b	a
b	a	b	b	b	b	a	a	b	a
b	a	b	b	b	b	a	a	b	a
b	a	b	b	b	b	-	a	b	a
b	a	b	b	b	-	-	a	b	a
b	a	b	b	b	a	a	a	b	a
b	a	b	b	b	b	a	a	b	a
b	a	b	b	b	b	a	a	b	a
b	a	b	b	b	b	a	a	b	a
b	a	b	b	b	b	a	a	b	a
b	a	b	b	b	b	a	a	b	a
b	a	b	b	b	b	a	a	b	a
b	a	b	b	b	b	a	a	-	a
b	a	b	b	b	b	a	a	a	a
b	a	b	b	b	b	a	a	a	a
b	a	a	b	b	b	a	a	a	a
b	a	b	b	b	a	a	a	a	a
b	a	b	b	b	a	a	a	a	a
b	a	b	b	b	a	a	a	a	a
b	a	b	b	b	a	a	a	a	a
b	a	b	b	b	a	a	a	a	a
b	a	b	b	b	a	a	a	a	a
b	a	-	b	b	a	a	a	a	a
b	a	b	b	b	a	a	b	a	a
b	a	b	b	b	-	a	b	a	a

b	a	-	b	b	a	a	b	a	a
b	a	b	b	b	a	a	b	a	a
b	a	b	b	b	-	a	b	a	a
b	a	b	b	b	a	a	b	-	a
b	a	b	b	a	-	a	b	a	a
b	a	a	a	a	b	a	b	a	a
b	a	b	a	a	b	a	b	a	a
b	a	b	a	a	b	a	b	a	a
a	a	b	b	b	a	a	b	a	a
a	b	b	b	b	a	a	b	a	a
a	b	b	b	b	a	a	b	a	a
a	b	b	b	b	a	a	a	a	a
a	b	b	b	b	b	a	a	a	a
a	-	b	b	b	a	a	a	a	a
a	b	b	b	b	a	a	a	a	a
a	b	b	b	b	a	a	a	a	a
a	b	b	b	b	-	a	a	a	a
a	b	b	b	b	a	a	a	a	a
a	b	b	b	b	a	a	a	a	a
a	b	b	b	b	a	a	a	a	a
a	b	b	b	b	a	a	a	a	a
a	b	b	b	b	a	a	a	a	a
a	b	b	b	b	a	a	a	a	a
a	b	b	b	b	a	a	a	a	a
a	b	-	b	b	a	a	a	a	a
a	b	b	b	b	a	b	a	a	a
a	b	b	b	b	-	b	a	a	a
a	b	b	b	b	a	b	a	a	a
a	b	b	b	a	a	a	a	a	a
a	b	b	b	b	a	a	a	a	a
a	b	b	b	b	a	a	a	a	a
a	b	-	b	b	a	a	a	a	a
a	b	b	b	b	a	a	a	a	a
a	b	b	b	b	a	a	a	a	-
a	b	b	b	b	a	a	a	a	a
a	b	b	b	b	a	a	a	a	a
a	b	b	b	b	a	a	a	a	a
a	b	b	b	b	a	a	a	a	a
a	b	b	b	b	a	a	a	a	a
a	b	b	b	b	a	a	a	a	a
a	b	b	a	b	a	a	a	a	a
a	b	b	a	b	a	a	a	a	a
a	b	b	a	b	a	a	a	a	a
a	b	b	a	b	a	a	a	a	a
a	b	b	a	b	a	a	a	a	a
a	b	b	a	b	a	a	a	b	a
a	b	a	a	b	-	a	a	b	a

a	b	a	a	b	b	a	a	b	a
a	b	a	a	b	b	a	a	b	a
a	b	a	a	b	b	a	a	b	a
a	b	a	a	b	b	-	a	b	a
a	b	a	a	b	b	a	a	b	a
a	b	a	a	b	b	a	a	b	a
a	a	a	-	b	b	a	b	b	a
a	a	a	a	b	b	a	-	b	b
a	a	a	a	b	b	a	b	b	b
a	a	a	a	b	b	a	b	-	b
a	a	-	a	b	b	a	b	b	b
a	a	a	a	b	b	a	b	b	b
a	a	-	a	b	b	a	b	b	b
a	a	a	a	b	b	a	b	b	b
a	a	a	a	b	b	a	b	b	b
a	a	a	a	b	a	a	b	b	b
a	a	a	a	b	a	-	b	b	b
a	a	a	a	b	a	-	b	b	b
a	a	a	a	a	-	a	a	b	b
a	a	a	a	a	b	-	a	b	b
b	b	b	a	b	b	a	a	a	a
b	b	b	a	b	a	a	a	a	a
b	b	b	a	b	a	a	a	a	a
b	b	b	a	b	a	a	a	a	a
b	b	b	a	b	a	a	a	b	a
b	b	a	a	b	a	a	a	-	a
b	b	a	a	b	a	a	a	a	a
b	b	a	a	b	a	a	a	a	a
b	b	a	a	b	a	a	-	a	a
b	b	a	a	b	a	a	a	a	a
b	b	a	a	b	a	a	a	a	a
b	b	a	a	b	a	a	a	a	a
b	b	a	a	b	a	a	a	a	a
b	b	a	a	b	a	a	a	a	a
b	b	-	a	b	a	a	-	a	a
b	b	a	a	b	a	a	a	a	a
b	b	a	a	b	a	-	a	a	a
b	b	a	a	b	a	a	a	a	a
b	b	a	a	b	a	a	a	a	a
b	b	a	a	b	a	a	a	a	a
b	b	a	a	b	a	a	a	a	a
b	b	a	a	b	a	a	a	a	a
b	b	a	a	b	a	a	a	a	a
b	b	a	a	b	a	a	a	a	a
b	b	a	a	b	a	a	a	a	a
b	b	a	a	b	a	a	a	a	a
b	b	a	a	b	a	a	a	a	a

b	b	-	a	b	-	a	-	b	a
b	b	a	a	b	a	a	a	a	a
b	b	a	a	b	a	a	a	a	a
a	b	a	b	b	a	a	a	a	a
a	b	a	b	a	a	a	a	a	a
a	b	a	b	a	a	a	a	a	a
a	b	b	b	a	a	a	a	a	a
a	b	-	b	a	b	a	a	a	a
a	b	a	b	a	b	a	a	a	a
a	a	a	b	a	b	a	a	a	a
a	a	a	b	a	b	a	a	-	a
b	a	a	b	a	-	a	a	a	a
b	a	a	b	a	-	a	a	a	a
b	a	-	a	a	b	a	a	a	a
b	a	a	a	a	b	a	a	a	a
b	a	a	a	a	b	a	b	a	a
b	a	a	a	a	b	a	b	a	a
b	a	a	a	a	b	a	b	a	b
b	a	a	a	a	b	a	b	a	b
b	a	a	a	a	b	b	b	a	b
b	a	a	a	b	b	b	b	a	b
b	a	a	a	b	b	b	b	a	b
b	a	a	a	b	b	b	b	a	b
b	a	a	a	b	b	b	b	a	b
b	b	a	a	b	b	b	b	a	b
b	b	a	a	b	-	b	b	a	b
b	b	a	a	b	b	b	b	a	b
b	b	a	a	b	b	b	b	a	b
b	a	a	b	b	b	b	b	a	b
b	b	a	b	b	b	b	b	a	b
b	b	a	b	b	b	b	b	a	b
b	b	a	b	b	b	b	b	b	b
b	b	a	b	b	a	-	b	a	b
a	b	b	b	b	a	b	b	a	b
a	b	b	b	b	b	b	b	a	b
b	b	b	a	b	b	b	b	b	a
b	b	b	a	b	b	b	b	b	a
b	b	b	a	b	b	-	b	b	a
b	b	-	a	b	-	b	b	b	a
b	b	a	a	b	a	b	b	b	a
b	b	a	a	b	a	b	b	b	a
b	b	a	-	b	a	b	b	b	a
b	b	a	a	b	a	b	b	b	a
b	b	a	a	b	a	b	b	b	a
b	a	a	a	b	a	b	a	b	a
b	a	a	a	b	a	b	a	b	a
b	a	a	a	b	b	b	a	b	b

b	a	a	a	b	a	b	a	-	b
b	a	a	a	b	a	b	a	b	b
b	a	-	a	b	a	b	a	b	b
b	a	a	a	b	b	b	a	b	b
b	a	a	a	b	b	b	a	b	b
b	a	a	a	a	-	b	a	b	b
b	a	a	a	a	-	b	a	b	b
b	a	a	a	a	b	b	a	b	b
b	a	a	a	a	b	b	a	b	b
b	a	a	a	a	b	b	a	b	b
b	a	b	a	a	b	b	a	b	b
b	a	a	a	a	-	b	a	b	b
b	a	a	a	a	b	b	a	b	b
b	a	a	a	a	-	b	a	b	b
b	a	a	a	a	b	b	a	b	b
b	a	a	a	a	b	b	a	b	b
b	a	a	a	a	b	b	a	b	b
b	a	a	a	a	-	b	a	b	b
b	a	a	a	a	b	b	a	b	b
b	a	a	a	a	b	b	a	-	b
b	a	a	a	a	b	b	a	b	b
b	a	a	a	a	b	b	b	b	b
b	a	a	a	a	b	b	b	b	b
b	a	a	a	a	b	b	b	b	b
b	a	a	a	a	b	b	b	-	b
b	a	a	a	a	b	b	b	b	b
b	a	a	a	a	b	b	b	b	b
b	a	a	a	a	b	b	a	b	b
b	a	a	a	a	-	b	a	b	b
b	a	a	a	a	b	b	a	b	b
b	a	a	a	a	b	b	a	b	b
b	a	a	a	a	b	b	a	b	b
b	a	a	a	a	b	b	a	b	b
b	a	a	a	a	b	b	a	-	b
b	a	a	a	a	b	b	a	b	b
b	a	a	a	a	b	b	a	b	-
b	a	a	a	a	b	b	a	b	b
b	a	a	a	a	b	b	a	b	b
b	a	a	a	a	b	b	a	b	b
b	a	b	a	a	b	b	-	b	b
b	a	b	a	a	b	b	a	b	b
b	a	b	a	a	b	b	a	b	b
b	b	b	a	a	b	b	a	b	b
b	b	b	a	a	b	b	a	b	b
b	b	b	a	a	b	b	a	b	b
b	b	b	a	a	b	-	a	b	b
a	a	b	b	b	a	a	b	a	a

a	a	b	b	b	a	a	b	a	a
a	a	b	b	b	a	a	b	a	a
a	a	b	b	b	a	a	b	a	a
a	a	b	b	b	a	a	b	a	a
a	a	b	b	b	a	a	b	a	a
a	a	b	b	b	a	a	b	a	a
a	a	b	b	a	a	a	b	a	a
a	a	b	b	a	a	a	b	a	a
a	a	b	b	a	a	a	b	a	a
a	a	b	b	a	a	a	b	a	a
a	a	b	b	a	a	a	b	a	a
a	a	b	b	a	a	a	b	a	a
a	a	b	b	a	a	a	b	a	a
a	a	b	b	a	a	a	b	a	a
a	a	b	b	a	a	a	b	a	a
a	a	b	b	a	a	a	b	a	a
a	a	b	b	a	a	a	b	a	a
a	a	b	b	a	a	a	b	a	a
a	a	b	b	a	a	a	b	b	a
a	a	b	b	a	a	a	b	b	a
a	a	b	b	a	a	a	b	b	a
a	a	b	b	a	a	a	b	b	a
a	a	b	a	a	a	a	b	b	a
a	a	b	a	a	a	a	b	b	a
a	a	b	a	a	a	a	b	b	a
a	a	b	a	a	a	a	b	b	a
a	a	b	a	a	a	a	b	b	a
a	a	b	a	a	a	a	b	b	a
a	a	b	a	a	a	a	b	b	a
a	a	b	a	a	a	a	b	b	a
a	a	b	a	a	a	a	b	b	a
a	a	b	a	a	a	b	a	b	a
a	a	b	a	a	a	b	a	b	a
a	a	b	a	a	a	b	a	b	a
a	a	b	a	a	a	b	a	b	a
a	a	b	a	a	a	b	a	b	a
a	a	b	a	a	a	b	a	b	a
a	a	b	a	a	a	b	a	b	a
a	a	b	a	a	a	b	a	b	a
a	a	b	a	b	a	a	a	b	a
b	b	a	b	b	-	a	a	a	a
a	a	-	a	a	b	a	b	a	b
a	a	b	a	a	b	a	b	a	b
a	a	b	a	a	b	a	b	a	b
a	a	b	a	a	b	a	b	a	b
a	a	b	a	a	b	a	b	a	b
a	a	b	a	a	b	a	b	a	b

a	a	b	a	a	b	a	b	a	b
a	a	b	a	a	b	a	b	a	b
a	a	b	a	a	b	a	b	a	b
a	a	b	a	a	b	a	b	a	b
a	a	b	a	a	b	a	b	a	b

YW029	YW030	YW031	YW032	YW033	YW034	YW035	YW036	YW037	YW038
a	b	a	b	a	a	a	a	b	a
a	b	a	b	a	a	b	a	b	a
a	b	a	b	a	a	b	a	b	a
a	b	a	b	a	a	b	a	b	a
a	b	a	b	a	a	b	a	b	a
a	b	a	b	a	a	b	a	b	a
a	b	a	b	a	a	b	a	b	a
a	b	a	b	a	a	b	a	b	a
a	b	a	b	-	a	b	a	b	a
a	b	a	b	a	a	b	a	b	a
a	b	a	b	a	a	b	a	b	a
a	b	a	b	a	a	b	a	b	a
a	b	a	b	a	a	b	a	b	a
a	b	a	b	a	a	b	a	b	a
a	b	a	b	a	a	b	a	b	a
a	b	a	b	a	a	b	a	b	a
-	b	a	b	-	-	b	a	b	a
a	b	a	b	a	a	b	a	b	a
a	b	a	b	a	a	b	-	b	a
a	b	a	b	a	a	b	a	b	a
a	b	a	b	a	a	b	a	b	a
a	b	a	b	a	a	b	a	b	a
a	b	a	a	a	b	a	a	a	a
a	b	a	a	a	b	a	a	a	a
a	b	a	a	a	b	a	-	a	a
a	b	a	a	a	b	a	a	a	a
a	b	a	a	a	b	a	a	a	a
a	b	-	a	a	b	a	a	a	a
a	b	a	a	a	b	a	a	a	a
-	b	a	a	a	b	a	a	a	a
a	b	a	a	a	b	a	a	a	a
a	b	a	a	a	b	a	a	a	a
a	b	a	a	a	b	a	a	a	a
a	b	a	a	a	b	a	a	a	a
a	b	a	a	a	b	a	a	a	a
a	b	a	a	a	b	a	a	a	-
a	b	a	a	a	b	a	a	a	a
a	b	a	a	a	b	a	a	a	a
a	b	a	a	a	b	a	a	a	a
-	b	a	a	a	b	a	a	a	a
a	b	a	a	a	b	a	a	a	a
a	b	b	a	a	b	a	a	a	a
a	b	b	a	a	b	a	a	a	a
a	b	b	a	a	b	a	a	a	a
-	b	b	a	a	b	a	a	a	-

a	b	b	a	a	b	a	a	a	a
a	b	b	a	a	b	a	a	a	a
a	b	b	a	a	b	a	a	a	a
-	b	b	a	a	b	a	a	a	a
b	b	b	a	b	b	a	a	a	a
b	b	b	a	a	b	a	a	a	a
b	a	b	a	-	b	a	a	a	a
b	a	b	a	a	b	a	a	a	a
b	a	b	a	a	b	a	a	a	a
b	a	b	a	a	b	a	a	a	a
b	a	b	a	a	b	a	a	a	a
b	a	b	a	a	b	a	a	a	a
b	a	b	a	a	b	a	a	a	a
b	a	b	a	a	b	a	a	a	a
b	a	b	a	a	b	a	a	a	a
b	a	b	a	a	b	a	a	a	a
b	a	b	a	a	b	a	a	a	a
b	a	b	a	a	b	a	a	a	a
b	a	b	a	b	b	a	a	a	a
b	a	b	a	b	b	a	a	a	a
b	a	b	a	b	b	a	a	a	a
b	a	b	a	b	b	a	a	a	a
b	a	b	a	b	b	a	a	a	a
b	a	b	a	b	b	a	a	a	a
b	a	b	a	b	b	a	a	a	a
b	a	b	a	b	b	a	a	a	a
b	a	b	a	b	b	a	a	a	a
b	a	b	a	b	b	a	a	a	a
b	a	b	a	b	b	a	a	-	b
b	a	b	a	b	b	a	a	a	b
b	a	b	b	b	b	a	a	a	b
b	a	b	b	b	b	a	a	a	b
b	a	a	b	b	b	a	a	a	b
b	a	b	b	b	b	a	a	a	b
b	a	b	b	b	b	a	a	a	b
b	a	b	b	b	b	a	a	a	b
b	a	b	b	b	b	a	a	a	b
b	a	b	b	-	b	a	a	a	b
b	a	b	b	b	b	a	a	a	b
b	a	b	a	b	b	b	a	b	a
b	a	b	a	b	b	b	a	b	a
b	a	-	a	b	b	b	a	b	a
b	a	b	a	b	b	b	a	-	a
b	a	b	b	b	b	b	a	b	a
b	a	a	b	b	b	b	a	b	a
b	a	a	b	b	b	b	a	b	a
b	a	a	b	b	b	b	a	-	a

b	a	a	b	b	b	b	a	b	a
b	a	a	b	b	b	b	a	b	a
b	a	a	b	-	b	b	a	b	a
b	a	a	b	b	b	b	a	b	a
b	a	a	b	b	b	b	a	b	a
b	a	a	b	b	b	b	a	b	a
b	a	a	b	b	b	b	a	b	a
b	a	a	b	b	b	b	a	b	a
b	a	a	b	b	b	b	a	b	a
a	a	a	b	b	b	b	a	b	a
a	a	a	b	b	b	b	a	b	a
a	a	a	b	b	b	b	a	b	a
a	a	a	b	b	b	b	a	b	a
a	a	a	b	b	a	b	a	b	a
a	a	a	b	b	a	b	a	b	a
a	a	a	b	b	a	b	a	b	a
a	a	a	b	b	a	b	a	b	a
a	a	a	b	b	a	b	a	b	a
a	a	a	b	b	a	b	a	b	a
a	a	a	b	b	a	b	a	b	a
-	a	-	b	b	a	b	a	b	a
a	a	a	b	b	a	b	a	b	a
a	a	a	b	b	b	b	a	b	a
a	a	a	b	b	b	b	a	-	a
a	a	a	b	b	b	b	a	a	a
a	a	a	b	b	b	b	a	a	a
a	a	a	b	b	b	b	a	a	a
a	a	a	b	b	b	b	a	a	a
a	a	a	b	b	b	b	a	a	a
a	a	a	b	b	b	b	a	a	a
a	a	a	b	b	b	b	a	a	a
a	a	a	b	b	b	b	a	a	a
a	a	a	b	-	b	b	a	a	-
a	a	a	b	-	b	b	a	a	-
a	a	a	b	b	b	b	a	a	b
a	a	a	b	b	b	b	a	a	b
a	a	a	b	b	b	b	a	a	b
-	a	a	b	b	b	b	a	-	b
a	b	a	b	b	b	b	a	a	b
a	b	a	b	b	b	b	a	a	b
a	b	a	b	b	b	b	a	a	b
a	b	a	b	b	b	b	a	a	b
-	b	a	b	b	b	b	a	a	b
a	b	a	b	b	b	b	a	a	b
a	b	a	b	b	b	b	a	a	b
a	b	a	b	b	b	b	a	a	b
a	b	a	b	b	b	b	a	a	b

-	b	a	b	b	b	b	a	a	b
a	b	a	b	b	b	b	a	a	b
a	b	a	b	b	b	b	-	-	b
a	b	a	b	b	b	b	a	a	b
a	b	a	b	-	b	b	a	a	b
a	b	a	b	b	b	b	a	a	b
a	b	a	b	b	b	b	a	a	b
a	b	a	b	b	b	b	a	a	b
a	b	a	b	b	b	b	a	a	b
-	b	a	b	b	b	b	a	a	b
a	b	a	b	b	b	b	a	a	b
a	b	a	b	b	b	b	a	a	b
a	b	a	b	b	b	b	a	a	b
a	b	a	b	b	b	b	a	a	b
a	b	a	b	b	b	b	a	a	b
-	b	a	b	b	b	b	a	-	b
a	b	a	b	b	b	b	a	a	b
a	b	a	b	b	b	b	a	a	b
a	b	a	b	b	b	b	a	a	b
a	b	a	b	b	b	b	a	a	b
a	b	a	b	b	b	b	a	a	b
-	b	a	b	b	b	b	a	a	b
a	b	a	b	b	b	b	a	a	b
a	b	a	b	b	b	b	a	a	b
a	b	a	b	b	b	b	a	a	b
a	b	a	b	b	b	b	a	a	b
a	b	a	b	b	b	b	a	a	b
a	b	a	b	b	b	b	a	a	b
a	b	a	b	b	b	b	a	a	b
a	b	a	b	b	b	b	a	b	b
b	b	a	b	b	b	b	a	b	b
b	b	a	b	b	b	b	a	b	b
b	b	a	b	b	b	b	a	b	b
b	b	a	b	b	b	b	a	b	b
b	b	-	b	b	b	b	a	b	b
b	b	a	b	b	b	b	a	b	b
b	b	a	b	b	b	b	a	b	b
b	b	a	b	b	b	b	a	b	b
b	b	a	b	b	b	b	a	b	b
-	b	a	b	a	b	b	a	b	b
b	b	a	b	-	b	b	a	b	b
b	b	a	b	b	b	b	a	b	b
b	b	a	b	b	b	a	a	b	b
b	b	a	b	b	b	a	a	b	b
b	b	a	b	-	b	a	a	b	b
b	b	b	b	b	a	a	a	b	a
b	b	b	b	b	a	a	a	b	a

b	b	b	b	-	a	a	a	b	a
b	b	b	b	b	a	a	a	b	a
b	b	b	b	b	a	a	a	b	a
b	b	b	b	b	a	a	a	b	a
b	b	b	b	b	a	a	a	b	-
b	b	b	b	b	a	a	a	b	a
b	b	b	b	b	a	a	a	b	a
b	b	b	b	b	a	a	a	b	a
-	b	-	b	b	a	a	a	b	a
b	b	b	b	b	a	a	a	b	a
b	b	b	b	b	a	a	a	b	a
b	b	b	b	b	a	a	a	b	a
a	b	a	a	b	b	a	a	b	b
a	b	a	a	b	b	a	a	b	b
b	b	a	a	b	b	a	a	b	b
b	b	a	a	b	b	a	a	b	b
-	b	a	a	b	b	a	a	b	b
b	b	a	a	b	b	a	a	b	b
b	b	a	a	b	b	a	a	b	b
b	b	a	a	b	b	a	a	b	b
b	b	a	a	b	b	a	a	b	b
b	b	a	a	b	b	a	a	b	b
b	b	-	a	b	b	a	a	b	b
b	b	a	a	b	b	a	a	b	b
b	b	a	a	b	b	a	a	b	b
b	b	a	a	b	b	a	a	a	b
b	b	a	a	b	b	a	a	a	b
b	b	a	a	b	b	a	a	a	b
b	b	a	a	b	b	a	-	-	b
b	b	a	a	b	b	a	a	a	b
b	b	a	a	b	b	a	a	a	b
b	b	a	a	b	b	a	b	a	b
b	b	a	a	b	b	a	b	a	b
b	b	a	a	b	b	a	b	a	b
b	b	a	a	b	b	a	b	a	a
b	b	a	a	b	b	a	b	a	a
b	b	-	a	b	b	a	b	a	a
b	b	a	a	b	b	a	b	a	a
b	b	a	b	b	b	a	b	a	a
b	b	a	b	b	b	a	b	a	a
b	b	a	b	a	b	a	b	a	a
b	b	a	b	a	b	a	b	a	a
b	b	a	b	a	b	b	b	a	a
b	b	a	b	a	b	b	b	a	a
b	b	a	b	a	b	b	b	a	a
b	b	a	b	a	b	b	b	a	a
b	b	a	b	a	b	b	b	a	a

b	b	-	b	a	b	b	b	a	-
b	b	a	b	a	b	b	b	a	b
b	-	a	b	a	b	b	b	a	b
b	b	a	b	-	b	b	b	a	a
b	b	a	b	a	b	b	b	a	a
-	b	a	b	a	b	b	b	a	a
a	b	a	b	b	b	b	b	a	a
-	b	a	b	b	b	b	b	a	a
a	b	a	b	b	b	b	b	a	a
b	b	a	b	b	b	b	b	a	a
b	b	a	b	b	b	b	b	a	a
b	b	a	b	a	b	b	b	a	a
b	b	a	b	b	b	b	b	a	a
b	b	a	b	b	b	b	b	a	a
b	b	a	b	b	b	b	b	a	a
b	b	a	b	b	b	b	b	a	a
b	b	a	b	b	b	b	b	a	a
b	b	a	b	b	b	b	b	a	a
b	b	a	b	b	b	b	b	a	a
b	b	a	b	b	b	b	-	a	a
b	b	a	b	b	b	b	b	a	a
b	b	a	b	b	b	b	b	a	a
b	b	a	b	b	b	b	b	a	a
b	b	a	b	b	b	b	b	a	a
b	b	a	b	b	b	b	b	b	a
b	b	a	b	b	b	b	-	b	a
b	b	a	b	b	b	b	b	b	a
b	b	a	b	b	b	b	b	b	a
b	b	a	b	b	b	b	b	b	a
b	b	a	b	b	b	b	b	b	a
b	b	a	b	b	b	b	b	b	a
b	b	a	b	b	b	b	b	b	a
b	b	a	b	b	b	b	b	b	a
b	b	a	b	b	b	b	b	b	a
b	b	a	b	b	b	b	b	-	a
b	b	a	b	b	b	b	b	b	b
b	b	a	b	b	-	b	b	b	b
b	b	a	b	b	b	b	b	b	b
b	b	a	b	b	b	b	b	b	b
b	b	a	b	b	b	b	b	b	b
b	b	a	b	b	b	b	b	-	b
b	b	a	b	b	b	b	b	b	b
b	b	a	b	b	b	b	b	b	-
b	b	a	b	b	b	b	b	b	b
b	b	a	b	b	b	b	b	b	b
b	b	a	b	b	b	b	b	b	b
-	b	a	b	b	b	b	-	b	b

-	b	a	b	b	b	b	b	-	b
b	b	a	b	b	b	b	b	b	b
b	b	a	b	b	b	b	b	b	b
b	b	a	b	a	b	b	b	b	-
b	b	a	b	b	b	b	b	b	b
b	b	a	b	b	b	b	b	b	b
b	b	a	b	b	b	b	b	b	b
b	a	a	b	b	b	b	b	b	b
b	a	a	b	b	b	b	b	b	b
b	a	a	b	b	b	b	b	b	b
b	a	a	b	b	b	b	b	b	b
b	a	a	b	b	b	b	b	b	b
b	a	a	b	b	b	b	b	b	b
b	a	a	b	b	b	b	b	b	b
b	a	a	b	b	b	b	a	b	b
b	a	a	a	b	b	b	a	b	b
b	a	a	a	b	b	b	a	b	b
-	a	a	a	b	b	b	a	b	b
b	a	a	a	b	b	b	a	b	b
-	a	a	a	b	b	b	a	b	b
b	a	a	a	b	b	b	a	b	b
b	a	a	a	b	b	b	a	b	b
b	a	a	a	b	a	b	a	b	b
b	a	-	a	b	a	b	a	b	b
b	a	a	a	b	a	b	a	b	b
b	a	a	a	b	a	b	a	b	b
b	a	a	a	b	a	b	a	b	b
b	a	a	a	b	a	b	a	b	b
b	a	a	a	b	a	b	a	b	b
b	a	a	a	b	a	b	a	b	b
b	a	a	a	b	a	b	a	-	b
b	a	a	a	b	a	b	a	b	b
b	a	a	a	-	a	b	a	b	b
b	a	a	a	b	a	b	a	b	b
b	a	a	a	b	a	b	a	b	b
b	a	a	a	b	a	b	a	b	b
b	a	a	a	b	a	b	a	b	b
b	a	a	-	b	a	b	a	b	b
b	a	b	a	a	a	b	b	-	a
b	a	b	a	a	a	b	b	b	a
b	a	b	a	a	a	b	b	b	b
b	a	b	a	a	a	b	b	b	b
b	a	b	a	a	a	b	b	b	b
b	a	b	a	a	a	b	b	b	b
b	a	b	a	a	a	b	b	b	-
b	a	b	a	a	a	b	b	b	b
b	a	b	a	a	a	b	b	b	b

b	a	b	a	a	a	b	b	b	b
-	a	b	a	a	a	b	b	b	b
b	a	b	a	a	a	b	b	b	b
b	a	b	a	-	a	b	b	b	b
b	a	b	a	a	a	b	b	b	b
b	a	b	a	a	a	b	b	b	b
b	a	b	a	a	a	b	a	b	b
b	a	b	a	a	a	b	a	b	b
b	a	b	a	a	a	b	a	b	b
b	a	b	a	a	a	b	a	b	b
b	b	b	a	a	a	b	a	b	b
b	b	b	a	a	a	b	b	b	b
b	b	b	a	a	a	b	b	b	b
b	b	b	a	a	a	b	b	b	b
b	b	b	a	a	a	b	b	b	b
b	b	b	a	a	a	b	b	b	b
b	b	b	a	a	a	b	b	b	b
b	b	b	a	a	a	b	b	b	b
b	b	b	a	a	a	b	b	b	b
b	b	b	a	a	a	b	b	b	b
b	b	b	a	a	a	b	b	b	-
b	b	b	a	a	a	b	b	b	b
b	b	b	a	a	a	b	b	b	b
b	b	b	a	a	a	b	b	b	b
b	b	b	a	a	a	b	b	b	b
b	b	b	a	a	a	b	b	b	b
b	b	b	a	a	a	b	b	b	b
b	b	b	a	a	a	b	b	b	b
b	b	b	a	a	a	b	b	b	b
b	b	b	a	a	a	b	b	b	b
b	b	b	a	a	a	b	b	b	b
b	b	b	a	a	a	b	b	b	b
b	b	b	a	a	a	b	-	b	b
b	b	b	a	a	a	b	b	b	b
b	b	b	a	a	a	b	b	b	b
b	b	b	a	a	a	b	b	-	a
b	b	b	a	a	b	a	b	-	b
b	-	b	a	a	b	a	b	b	b
b	b	b	a	a	b	a	b	b	b
b	b	b	a	a	b	a	b	b	b
b	b	b	a	a	b	a	b	b	b
b	b	b	a	a	b	a	b	b	b
b	b	b	a	b	b	a	b	a	b
b	b	b	a	b	b	a	b	a	b
b	b	b	a	b	b	a	b	a	b
b	b	b	a	b	b	a	-	a	b
b	b	b	a	b	b	a	b	a	b

b	b	b	a	b	b	a	b	a	b
b	b	b	a	b	b	a	b	a	-
b	b	b	a	b	b	a	b	a	b
b	b	b	a	b	b	a	b	a	a
b	b	-	a	b	b	a	b	a	a
b	b	b	a	b	b	b	b	a	a
-	b	b	a	b	b	b	b	a	a
b	b	b	a	b	b	b	b	a	a
b	b	b	a	b	b	b	b	a	a
b	b	b	a	b	b	b	b	a	a
b	b	b	a	b	b	b	b	a	a
b	b	b	a	b	b	b	b	a	a
b	b	b	a	b	b	b	b	a	a
b	b	b	a	b	b	b	b	a	a
b	b	b	a	-	b	b	b	a	a
b	b	b	a	b	b	b	b	a	a
b	b	b	b	b	b	b	b	a	b
b	b	b	b	b	b	b	a	a	b
b	b	b	b	b	b	b	a	a	b
b	b	b	b	b	b	b	a	a	b
b	b	b	b	b	b	b	a	a	b
b	b	b	b	b	b	b	a	a	b
b	b	b	b	b	b	b	a	a	b
b	a	b	b	b	b	b	a	a	b
a	a	b	b	b	b	b	a	a	b
a	a	b	b	b	a	b	a	a	b
a	a	b	a	b	a	b	a	a	b
a	a	b	a	b	a	b	a	a	b
a	a	b	a	b	a	b	a	a	b
a	a	b	a	b	a	b	a	a	b
a	a	b	a	b	a	b	a	a	b
a	a	b	a	-	a	b	a	a	b
a	a	b	a	b	a	b	a	a	b
a	a	b	a	b	a	b	a	a	b
a	a	b	a	b	a	b	a	a	b
a	a	b	a	b	a	b	a	a	b
a	a	b	a	a	a	b	a	a	b
a	a	b	a	b	a	b	a	a	b
a	a	b	a	b	a	b	a	a	b
a	a	b	a	b	a	b	a	a	b
a	a	b	a	b	a	b	a	a	a
a	a	b	a	b	a	b	a	a	a
a	a	b	a	b	a	b	-	-	a
a	a	b	a	b	a	b	a	a	a
-	a	b	a	b	a	b	a	a	-
a	a	b	a	b	a	b	a	a	b
-	a	b	a	b	a	b	a	a	b

a	a	b	a	b	a	b	a	a	b
a	a	b	a	b	a	b	a	a	b
a	a	b	a	b	a	b	a	a	b
a	a	b	a	b	a	b	a	a	b
-	a	b	a	b	a	b	a	a	b
a	a	b	a	b	a	b	a	a	b
a	a	b	a	b	a	b	a	-	b
a	a	b	a	b	a	b	a	a	b
a	a	b	a	b	a	b	a	a	-
a	a	b	a	b	a	b	a	a	b
a	a	b	a	b	-	b	a	a	b
a	a	b	a	b	a	b	a	a	b
a	a	b	a	b	a	b	a	a	b
-	a	b	a	b	a	b	a	a	b
a	a	b	a	b	a	b	a	a	b
a	a	b	a	b	a	b	a	a	b
a	a	b	a	b	a	b	a	a	b
a	a	b	a	b	a	b	a	a	b
-	-	b	a	b	a	b	a	a	b
a	a	b	a	b	a	b	a	-	b
a	a	b	a	b	a	b	a	a	b
a	a	b	a	b	a	b	a	a	b
a	a	b	a	b	a	b	a	a	b
a	a	b	a	b	a	b	a	a	b
a	a	b	a	b	a	b	a	a	b
a	a	b	a	b	a	b	a	a	b
a	a	a	a	b	a	b	a	a	b
a	a	a	a	b	a	b	a	a	b
a	a	a	a	b	a	b	a	a	b
a	a	a	a	b	a	b	a	a	b
a	a	a	a	b	a	b	a	a	b
a	a	a	a	b	a	b	a	a	b
a	a	a	a	-	a	b	a	a	b
a	a	a	a	a	a	b	a	a	b
a	a	a	a	b	a	b	a	a	b
a	a	a	a	b	a	b	a	a	b
a	a	a	a	b	a	b	a	a	b
a	a	a	a	b	a	b	a	a	b
a	a	a	a	b	a	b	a	a	b
a	a	a	a	b	a	b	a	b	b
a	a	a	a	b	a	b	a	a	b
a	b	a	a	b	a	b	a	a	b
a	b	a	a	b	a	b	a	a	b
a	b	a	a	b	a	b	a	a	b
a	b	a	a	b	a	b	a	a	b
a	b	a	a	b	a	b	a	a	b
a	b	a	a	a	a	b	a	a	b

a	b	a	a	a	a	b	a	a	b
a	b	a	a	a	a	b	a	-	b
a	b	a	a	a	a	b	a	a	b
a	b	a	a	a	a	b	a	b	b
-	-	a	a	a	a	b	a	b	b
b	b	a	a	a	a	b	a	b	b
b	b	a	a	a	a	b	-	b	b
-	b	a	a	a	a	b	a	b	b
b	b	a	a	a	a	b	a	b	b
b	b	a	a	a	a	b	a	b	b
b	b	a	-	a	a	b	a	b	b
b	b	a	a	a	a	b	a	a	b
b	b	a	a	a	a	b	a	a	b
b	b	a	a	a	a	b	a	a	b
b	b	a	a	a	a	b	a	a	b
b	b	a	a	a	a	b	a	a	b
b	b	a	a	a	a	b	a	a	b
b	b	a	a	a	a	b	a	a	b
b	b	a	a	a	a	b	a	a	b
b	b	a	a	a	a	b	a	a	b
b	b	a	a	a	a	b	a	-	b
b	b	a	a	a	a	b	a	a	b
b	b	a	a	a	a	b	a	a	b
b	b	a	a	a	a	b	a	a	b
b	b	a	a	a	a	b	a	a	b
b	b	b	a	a	a	b	a	a	b
b	b	b	a	a	a	b	-	a	b
b	b	b	a	a	a	b	-	a	b
b	b	b	a	a	a	b	a	a	b
b	b	b	-	a	a	b	a	a	b
b	b	b	a	a	a	b	a	a	b
b	b	b	a	a	a	b	a	a	b
b	b	b	a	a	a	b	a	a	b
b	b	b	a	a	a	b	a	a	b
b	b	b	a	a	a	b	a	a	b
b	b	b	a	a	a	b	a	a	b
b	b	b	a	a	a	b	a	a	b
b	b	b	a	a	a	b	a	a	b
b	b	b	a	a	a	b	a	a	b
b	b	b	a	a	a	b	-	a	b
b	b	b	a	a	a	b	a	a	b
b	b	b	a	a	a	b	a	a	b
b	b	b	a	a	a	b	a	a	b
-	b	b	a	a	a	b	a	a	b
b	b	b	a	a	a	b	a	a	b
b	b	b	a	a	a	b	a	a	b
b	b	b	a	a	a	b	a	a	b

b	a	b	a	a	a	b	a	-	b
b	a	b	a	a	a	b	a	a	b
b	a	b	a	a	a	b	a	a	b
b	a	b	a	a	a	b	-	-	b
b	a	b	a	-	a	b	a	-	b
b	a	b	a	a	a	b	a	a	b
b	a	b	a	a	a	b	a	a	b
b	a	b	a	a	a	b	a	a	b
b	a	b	a	a	a	b	a	a	b
b	a	b	a	a	a	b	a	a	b
b	a	b	a	a	a	b	a	a	b
b	a	b	a	a	a	b	a	a	b
b	a	b	a	a	a	b	-	a	b
b	a	b	a	a	a	b	a	a	b
b	a	b	a	a	a	b	a	a	b
b	a	b	a	a	a	b	a	a	b
b	a	b	a	a	a	b	a	a	b
b	a	b	a	-	a	b	-	a	b
b	a	b	a	a	a	b	a	a	b
b	b	b	a	b	b	b	a	a	a
b	b	b	a	b	b	b	-	a	a
b	b	b	a	b	b	b	a	a	a
b	b	b	a	b	b	b	a	a	a
b	b	b	a	b	b	b	a	a	a
b	b	b	a	b	b	b	a	a	a
b	b	b	a	b	b	b	a	a	a
b	b	b	a	b	b	b	a	a	a
b	b	b	a	b	b	b	a	a	a
b	b	b	a	b	b	b	a	a	a
b	b	b	a	b	b	b	a	a	a
b	b	b	a	b	b	b	a	a	a
-	b	b	a	b	b	b	a	a	a
b	b	b	a	b	b	b	a	a	a
-	b	b	a	b	b	b	a	a	a
b	b	b	-	b	b	b	a	a	a
b	b	b	a	b	b	b	a	a	a
b	b	b	a	b	b	b	a	a	a
b	b	b	a	b	b	b	a	a	a
b	b	b	a	b	b	b	a	a	a
b	b	b	a	b	b	b	a	a	a
b	b	b	a	b	b	b	a	a	a
b	b	b	a	b	b	b	a	a	a
b	b	b	a	b	b	b	a	a	-
a	b	b	a	b	b	b	a	a	a
a	b	b	a	b	b	b	a	a	a
a	a	b	a	b	b	b	a	a	a
a	a	b	a	b	b	b	a	-	a

a	a	b	a	b	b	b	a	a	a
a	a	b	a	b	b	b	a	a	a
a	a	b	a	b	b	b	a	a	a
a	a	b	a	b	b	b	a	a	a
a	a	b	a	b	b	b	a	a	a
a	a	b	a	a	b	b	a	a	a
a	a	b	a	b	b	b	a	a	a
a	a	b	a	b	b	b	a	a	a
a	a	b	a	b	b	b	a	a	a
a	a	b	a	b	b	b	a	a	a
a	a	b	a	b	b	b	a	a	a
a	a	b	a	b	b	b	a	a	a
a	a	b	a	b	b	b	a	a	a
a	a	b	a	b	b	b	a	a	a
a	a	b	a	b	b	b	-	a	a
a	a	b	a	b	b	b	a	a	a
a	a	b	a	b	b	b	-	a	a
a	a	b	a	b	b	b	a	a	a
a	a	b	a	b	b	b	a	a	-
a	a	b	a	b	b	b	a	a	a
a	a	b	a	b	b	b	a	a	a
a	a	b	a	b	b	b	a	a	a
a	a	b	a	b	b	b	a	a	a
a	a	b	a	b	b	b	a	a	a
a	a	b	a	b	b	b	a	a	a
a	a	b	a	b	b	b	a	a	a
a	a	b	a	b	b	b	a	a	a
a	a	b	a	b	b	b	a	a	a
a	a	b	a	b	b	b	a	a	a
a	a	b	a	b	b	b	a	a	a
a	a	b	a	b	b	b	a	a	a
a	a	b	b	b	b	b	a	a	a
a	a	b	b	b	b	b	a	a	a
-	a	b	b	b	b	b	a	a	a
a	a	b	b	b	b	b	a	a	a
a	a	b	b	b	b	b	a	a	-
a	a	a	b	b	b	b	a	a	a
a	a	a	b	b	b	b	a	a	a
a	a	a	b	b	b	b	a	a	a
a	a	a	b	b	b	b	a	a	a
a	a	a	b	b	b	b	a	a	a
a	a	a	b	b	b	b	a	a	a
a	a	a	b	b	b	b	a	a	a
a	a	a	b	-	b	-	a	a	a
a	a	b	b	a	b	a	a	a	a
a	a	b	b	a	b	a	a	a	a
a	b	b	b	a	b	a	a	-	a

a	b	b	b	a	b	a	a	a	a
a	b	b	b	a	b	a	a	a	a
a	b	b	b	a	b	a	a	a	a
a	b	b	b	a	b	a	a	a	a
a	b	b	b	a	b	a	a	a	a
a	b	b	b	a	b	a	a	a	a
a	b	b	b	a	b	a	a	a	a
a	b	b	b	a	b	a	a	a	a
a	b	b	b	a	b	a	a	a	a
a	b	b	b	a	b	a	a	a	a
a	b	b	b	-	b	a	a	a	a
b	b	a	b	b	a	b	b	b	b
b	b	a	b	b	b	b	b	b	b
b	b	a	b	b	b	b	b	b	b
b	b	a	b	b	b	b	b	b	-
b	b	a	b	b	b	b	b	b	b
b	b	a	b	b	b	b	b	b	b
b	b	a	b	b	b	b	b	b	b
-	b	a	b	b	b	b	b	b	b
b	b	a	b	b	b	b	b	b	b
b	b	a	b	b	b	b	b	b	b
b	b	a	b	b	b	b	b	b	b
-	b	a	b	b	b	b	b	b	b
b	b	a	b	b	b	b	b	b	b
b	b	a	b	b	b	b	b	b	b
b	b	a	b	b	b	b	b	b	b
b	b	a	b	b	b	b	b	b	b
b	b	a	b	b	b	b	-	b	b
b	b	a	b	b	b	b	b	b	b
b	b	a	b	b	b	b	b	b	b
a	b	a	b	b	b	b	b	b	b
a	b	a	b	b	b	b	b	b	b
a	b	a	b	b	b	b	b	b	b
a	b	a	b	b	b	b	b	b	b
a	b	a	b	b	b	b	b	b	b
a	b	a	b	b	b	b	b	b	b
a	b	a	b	b	b	b	b	b	b
a	b	a	b	b	b	b	b	b	b
a	b	a	b	b	b	a	b	b	b
a	b	a	b	b	b	a	b	b	b
a	b	a	b	b	b	a	b	b	b
a	b	a	b	b	b	a	b	b	b
a	b	a	b	b	b	a	b	b	b
a	b	a	b	b	b	a	b	a	a
a	b	a	b	b	b	a	b	a	a
a	b	a	b	b	b	a	b	a	a
a	b	a	b	b	b	a	b	a	a

a	b	a	b	b	b	a	b	a	-
a	b	a	b	b	b	a	b	a	a
a	b	a	b	b	b	a	b	a	a
a	b	a	b	b	b	a	b	a	a
a	b	b	b	b	b	a	b	-	a
a	b	b	b	b	b	a	b	a	b
a	b	b	b	b	b	a	b	a	b
a	b	b	b	b	b	a	b	a	b
a	b	b	b	b	b	a	b	a	b
a	b	b	b	b	b	a	b	a	b
a	b	b	b	b	b	a	b	a	b
a	b	b	b	b	b	a	b	a	b
a	b	b	b	b	b	a	b	-	b
a	b	b	b	b	b	a	b	a	b
a	b	b	b	b	b	a	b	a	b
a	b	b	b	b	b	a	b	a	b
a	b	b	a	b	b	a	b	a	b
a	b	b	a	b	b	a	b	a	b
a	b	b	a	a	b	a	b	a	-
a	b	b	a	a	b	a	b	a	b
a	b	b	a	a	b	a	a	-	b
a	b	b	a	a	b	a	a	a	b
a	b	b	a	a	b	a	a	a	b
a	b	b	a	a	b	a	a	a	b
a	b	b	a	a	b	a	a	a	b
a	b	b	a	a	b	a	a	b	b
a	b	-	a	a	b	a	a	b	b
a	b	b	a	a	b	a	a	b	b
-	b	b	a	a	b	a	a	b	b
a	b	-	a	a	b	a	a	b	b
a	b	b	a	a	b	a	a	b	b
a	b	b	a	a	b	a	a	b	b
a	b	b	a	a	b	a	a	b	b
a	b	b	a	a	b	a	a	b	b
a	b	b	a	a	b	a	a	-	b
a	b	b	a	a	b	a	a	b	b
a	b	b	a	a	b	a	a	b	b
a	b	b	a	a	b	a	a	b	b
a	b	b	a	a	b	a	a	b	b
a	b	b	a	a	b	a	a	b	b
a	b	b	a	a	b	a	a	b	b
a	b	b	a	a	b	a	a	b	b
a	b	b	a	a	b	a	a	b	b
a	b	a	a	a	b	a	a	b	b
a	b	a	a	a	b	a	a	b	b
a	b	a	a	a	b	a	a	b	b
a	b	a	a	a	b	a	a	b	b

a	b	a	a	a	b	a	a	b	b
a	b	a	a	a	b	a	a	b	b
a	b	a	a	a	b	a	a	b	b
a	b	a	a	a	b	a	a	b	b
a	b	a	a	a	b	a	a	b	b
a	b	a	a	a	b	a	a	b	b
-	b	a	a	a	b	a	a	b	b
-	b	a	a	a	b	a	a	b	b
a	b	a	a	a	b	a	a	b	b
a	b	a	a	a	b	a	a	b	b
a	b	a	a	a	b	a	a	b	b
a	b	a	a	a	b	a	a	b	b
a	b	a	a	a	b	a	a	b	b
a	b	a	a	a	b	a	a	b	b
a	b	a	a	a	b	a	a	a	b
-	b	a	b	a	b	a	a	b	b
a	b	a	b	a	b	a	a	b	b
a	b	a	b	a	b	a	a	b	b
a	b	a	b	a	b	a	a	b	b
a	b	a	b	a	b	a	a	b	b
a	b	a	b	a	b	a	a	b	b
a	a	a	b	a	b	a	a	b	b
b	a	b	a	a	b	a	a	a	b
a	a	b	a	a	b	a	a	a	b
a	a	b	a	a	b	a	a	a	b
b	a	b	a	a	b	a	a	a	b
b	a	b	a	a	b	a	a	a	b
b	a	b	a	a	b	a	a	a	b
b	a	b	a	a	b	a	a	a	b
b	a	b	a	a	b	a	-	a	b
b	a	b	a	a	b	a	-	a	b
b	a	b	a	a	b	b	a	a	b
b	a	b	a	a	b	b	a	a	b
b	a	b	b	a	b	b	a	a	b
b	a	b	b	a	b	b	-	a	b
b	a	b	b	a	b	b	b	a	b
b	a	b	b	a	b	b	b	a	b
b	a	b	b	a	b	b	b	a	b
b	a	b	b	a	b	b	b	a	b
b	a	b	b	a	b	b	b	a	b
b	a	b	b	a	b	b	b	a	b
b	-	b	b	a	b	b	b	a	b
b	a	b	b	a	b	b	b	a	b
b	a	b	b	a	b	b	b	a	b
b	a	b	b	a	b	b	b	a	b
b	a	b	b	a	b	b	b	a	b
b	a	b	b	b	b	b	b	a	b

b	a	b	b	b	b	b	b	a	b
b	a	b	b	b	b	b	b	a	b
b	a	b	b	b	b	b	b	a	b
b	b	b	b	b	b	b	b	b	b
b	b	b	-	a	b	b	b	b	b
b	b	b	b	b	b	b	b	b	-
-	b	b	b	b	b	b	b	-	b
-	b	b	b	b	b	b	a	b	b
-	b	b	b	b	b	b	b	b	b
b	b	b	b	b	b	b	b	b	b
b	b	b	b	b	b	b	b	b	b
b	b	b	b	b	b	b	b	b	b
b	b	b	b	b	b	b	b	b	b
b	b	b	b	b	b	b	b	b	b
b	b	b	b	b	b	b	b	b	b
b	b	b	b	b	b	b	b	b	b
b	b	b	b	b	b	b	b	b	b
b	b	b	b	b	b	b	b	b	b
b	b	b	b	b	b	b	b	b	b
b	b	b	b	-	b	b	b	b	b
b	b	b	b	b	b	b	b	b	b
b	b	b	b	b	b	b	b	b	b
b	b	b	b	b	b	b	b	b	b
b	b	b	b	b	b	b	b	b	b
b	b	b	b	b	b	b	b	b	b
b	b	b	b	b	b	b	b	-	b
b	b	b	b	b	b	b	b	b	b
b	b	b	b	b	b	b	b	b	b
b	b	b	b	b	b	b	b	b	b
b	b	b	b	b	b	b	b	b	b
b	b	b	b	b	b	b	b	b	b
b	b	b	b	b	b	b	b	b	b
b	b	b	b	b	b	b	b	b	b
b	b	b	b	b	b	b	b	b	b
b	b	b	b	b	b	b	b	b	b
b	b	b	b	b	b	b	b	b	b
b	b	b	b	b	b	b	b	b	b
b	b	b	b	b	b	b	b	b	b
b	b	b	b	b	b	b	b	b	b
b	b	b	b	b	b	b	b	b	b
b	b	b	b	b	b	b	b	b	b
b	b	b	b	b	b	b	b	b	b
b	b	b	b	b	b	b	b	b	b
b	b	b	b	b	b	b	b	b	b
b	b	b	b	b	b	b	b	b	b
b	b	b	b	b	b	b	b	b	b
b	b	b	b	b	b	b	b	b	b

b	b	b	b	b	b	b	b	b	b
b	b	b	b	b	b	b	b	b	b
b	b	b	b	b	b	b	b	-	-
b	b	b	b	b	b	b	b	b	b
b	b	b	b	b	b	b	b	b	b
b	b	b	b	b	b	b	b	b	b
b	b	b	b	b	b	b	b	b	b
b	b	b	b	b	a	b	b	b	b
b	b	b	b	b	a	b	b	b	b
b	b	b	b	b	a	b	b	b	b
b	b	b	b	b	a	b	b	b	b
b	b	b	b	b	a	b	b	b	b
b	b	b	b	b	a	b	b	b	a
b	b	b	b	b	a	b	b	b	a
b	b	b	b	b	a	b	b	b	a
b	b	b	b	b	a	b	b	b	a
b	b	b	b	b	a	b	b	b	a
b	b	b	b	b	a	b	b	b	a
b	b	b	b	b	a	b	b	b	a
b	b	b	b	b	a	b	b	b	a
b	b	b	b	b	a	b	b	b	a
b	b	b	b	b	a	b	b	b	a
b	b	b	b	b	a	b	b	b	a
b	b	b	b	b	a	b	b	b	a
b	b	b	b	b	a	b	b	b	a
b	b	b	b	b	a	b	b	b	a
b	b	b	b	b	a	b	b	b	a
b	b	b	b	b	a	b	-	b	a
b	b	b	b	b	a	b	b	b	a
b	b	b	b	b	a	b	b	b	a
b	b	b	b	b	a	b	b	b	-
b	b	b	b	b	a	b	b	b	a
b	b	b	b	b	a	b	b	b	a
b	b	-	b	b	a	b	b	b	a
b	b	b	b	b	a	b	b	b	a
b	b	a	b	b	a	b	b	b	a
b	b	a	b	b	a	b	b	b	a
b	b	a	b	b	a	b	b	b	a
-	b	a	-	b	a	b	b	b	a
-	b	a	b	b	a	b	b	b	a
b	b	a	b	b	a	b	b	b	a
b	b	a	b	-	a	b	b	-	a
b	b	a	b	b	a	b	-	b	a
b	b	-	b	b	a	b	b	b	a
b	b	a	b	b	a	b	b	b	a
b	b	a	b	b	a	b	b	b	a
b	b	a	b	b	a	b	-	b	a

b	b	a	b	b	a	b	b	b	a
b	b	a	b	b	a	b	b	b	a
b	a	a	b	b	a	b	b	a	a
a	b	a	a	a	b	b	a	b	b
a	b	a	a	a	b	b	a	b	b
a	b	a	b	a	b	b	a	-	b
a	b	a	b	a	b	b	a	a	b
a	b	b	b	a	b	b	a	a	b
a	b	b	b	a	b	b	a	a	b
a	b	b	b	a	b	a	a	a	b
a	b	b	b	a	b	a	a	a	b
a	b	b	b	a	b	a	a	a	b
a	b	b	b	a	b	a	a	a	b
a	b	b	b	a	b	a	a	a	b
a	b	b	b	a	b	a	a	a	b
a	b	b	b	a	b	a	a	b	b
a	b	b	b	a	b	a	-	b	b
a	b	b	b	a	b	a	a	b	b
a	b	b	b	a	b	b	-	b	b
a	b	b	b	a	b	b	-	b	b
a	b	b	b	a	b	b	b	b	a
a	b	b	b	a	b	b	b	-	a
a	b	b	b	a	b	b	b	b	-
a	b	b	b	a	b	b	b	b	a
a	b	b	b	a	b	b	b	b	a
a	b	b	b	a	b	b	b	b	a
a	b	b	b	a	b	b	b	b	a
a	a	b	b	a	b	b	b	-	a
a	a	b	b	a	b	b	b	b	a
a	a	b	b	a	b	b	b	b	a
a	a	b	b	a	b	b	b	b	a
a	a	b	b	a	b	b	b	b	a
a	a	b	b	a	b	b	b	b	a
a	a	b	b	-	b	b	b	b	a
a	a	b	b	a	b	b	b	b	a
a	a	b	b	a	b	b	b	b	a
a	a	b	b	a	b	b	-	b	a
a	a	b	b	a	b	b	b	b	a
a	a	a	b	a	b	b	b	b	b
a	a	a	b	a	b	b	b	b	b
a	a	a	b	a	b	b	b	b	b
a	a	a	b	a	b	b	b	b	b
a	a	a	b	a	a	b	b	b	b
a	a	a	b	a	a	b	b	b	b
a	a	a	b	a	a	b	b	b	b
a	a	a	b	a	a	b	b	b	b
-	a	a	b	a	a	b	b	b	b

a	a	a	b	a	a	b	b	b	b
a	a	a	b	a	a	b	b	b	b
a	a	a	b	a	a	b	b	b	b
a	b	a	b	a	a	b	b	b	b
-	b	a	a	b	a	a	-	a	a
b	b	a	a	-	a	b	b	a	a
b	b	a	a	b	a	b	b	-	a
b	b	a	a	b	a	b	b	a	a
b	b	a	a	b	a	b	b	a	a
b	b	a	a	b	a	b	b	a	a
b	b	a	a	b	a	b	b	a	a
-	b	a	a	b	a	b	b	a	a
b	b	-	a	b	a	b	b	-	a
b	b	b	a	-	a	b	b	b	b
b	b	b	a	b	a	b	b	b	b
a	b	b	a	b	a	b	b	b	b
a	b	b	a	b	a	b	b	b	b
a	b	b	a	b	a	b	b	b	b
a	b	b	a	a	a	b	b	b	b
a	b	b	a	a	a	b	b	b	b
a	b	b	a	a	a	b	b	b	b
a	b	b	a	a	a	b	b	b	b
a	b	b	a	a	a	b	b	b	b
a	b	b	a	a	a	b	b	b	b
a	b	b	a	a	a	b	b	b	b
a	b	b	a	b	a	b	b	b	b
a	b	b	a	b	a	b	b	b	b
a	b	b	a	b	a	b	b	b	b
a	b	a	a	b	a	b	b	b	b
a	b	b	a	b	a	a	b	b	b
a	b	b	a	b	a	a	b	b	b
a	b	b	a	b	a	a	b	b	b
a	b	b	a	b	a	a	b	b	b
a	b	b	a	a	a	a	b	b	b
a	b	b	a	b	a	a	b	b	b
a	b	b	a	b	a	a	b	b	b
a	b	b	a	b	a	a	b	b	b
a	b	b	a	-	a	a	b	b	b
a	b	b	a	b	a	a	b	b	b
a	b	b	a	b	a	a	b	b	b
a	b	a	a	a	a	a	b	b	b
a	b	a	a	a	a	a	b	b	b
a	b	a	a	a	a	a	b	b	b
a	b	a	a	a	a	a	-	b	b
a	a	a	a	a	a	a	b	b	b
b	b	b	b	a	b	b	a	a	b
b	b	b	b	a	b	b	a	a	b

b	b	b	b	a	a	b	a	a	b
b	b	b	b	a	a	b	a	a	-
b	b	b	b	a	a	b	a	a	b
b	b	b	b	a	a	b	a	a	b
b	b	b	b	a	a	b	a	a	b
b	b	b	b	a	a	b	a	a	b
b	b	b	b	a	a	b	a	a	b
a	b	b	b	a	a	b	a	a	b
a	b	b	b	a	a	b	a	a	b
a	b	b	b	a	a	b	a	a	b
a	b	b	b	a	a	b	a	-	b
a	b	b	b	a	a	b	a	a	b
a	b	b	b	a	a	b	a	a	b
a	b	b	b	a	a	b	a	a	b
a	b	b	b	a	a	b	a	a	b
a	b	b	b	a	a	b	a	a	b
a	b	b	a	a	a	b	a	a	b
a	b	b	a	a	a	b	a	a	b
a	b	b	a	a	a	b	a	a	b
a	b	-	a	a	a	b	a	a	a
a	b	b	a	a	a	b	a	a	a
a	b	b	a	a	a	b	-	a	a
a	b	b	a	a	a	b	a	a	a
a	b	b	a	-	a	b	a	a	a
a	a	b	a	b	a	b	a	a	a
a	a	b	a	b	a	b	a	a	a
a	a	b	a	a	a	b	a	a	a
a	a	b	a	a	a	b	a	a	a
a	a	b	a	a	a	b	a	a	a
a	a	b	a	a	a	b	a	a	a
a	a	b	a	a	a	b	a	a	a
a	a	b	a	a	a	b	a	a	a
-	a	b	a	a	a	b	a	a	a
a	a	b	a	a	a	b	a	a	a
a	a	b	a	a	a	b	a	a	a
a	a	b	a	a	a	b	a	a	a
-	a	b	a	a	a	b	a	-	a
a	a	b	a	a	a	b	-	a	a
a	a	b	a	a	a	b	a	a	a
a	a	b	a	a	a	b	a	a	-
a	a	b	a	a	a	b	a	a	a
a	a	b	a	a	a	b	a	a	-
a	a	b	a	a	a	b	a	a	a
a	a	b	a	a	a	b	a	a	-
a	a	b	a	a	a	b	a	a	a
a	a	b	a	a	a	b	a	a	a
a	a	b	a	a	a	b	a	a	a

a	a	b	a	a	a	b	a	a	a
a	a	-	a	a	a	b	a	a	a
a	a	b	a	a	a	b	a	a	a
a	a	b	a	a	a	b	a	a	a
a	a	b	a	a	a	b	a	a	a
a	a	a	a	a	a	b	a	a	a
a	a	-	a	a	a	b	a	a	a
a	a	a	a	a	a	b	a	a	a
a	a	a	a	a	b	b	a	a	a
a	a	a	a	a	b	b	a	-	a
a	a	a	a	a	b	b	a	a	a
a	a	a	a	a	b	b	a	a	a
a	a	a	a	a	b	b	a	a	a
a	a	a	a	a	b	b	a	a	a
a	a	a	a	a	b	b	a	a	a
-	a	a	a	a	b	b	a	a	a
a	a	-	a	a	b	b	a	a	a
a	a	a	a	a	b	b	a	a	a
a	a	a	a	a	b	b	a	a	a
a	a	a	a	a	b	b	a	a	a
a	a	a	a	b	b	b	a	b	a
a	a	a	a	b	b	b	a	-	a
a	a	a	a	b	b	b	a	b	a
a	a	a	a	b	b	b	a	b	a
a	a	a	a	b	b	b	a	b	a
a	a	a	a	b	b	b	a	b	a
a	a	a	a	b	b	b	a	b	a
a	a	a	a	b	b	b	a	b	a
a	a	a	a	b	b	b	a	b	a
-	a	a	a	b	b	b	a	b	a
a	a	a	a	b	b	b	a	b	a
a	a	a	a	b	b	b	a	b	a
a	a	a	a	b	b	b	a	b	a
a	a	a	a	b	b	b	a	-	a
a	b	a	a	b	a	b	a	b	a
a	b	a	a	b	b	b	a	b	a
a	b	a	a	b	b	b	a	b	a
a	b	a	a	b	b	b	a	b	a
a	b	a	a	b	b	b	a	b	a
a	b	a	a	b	b	b	a	b	b
a	b	a	a	b	b	b	a	b	b
a	b	a	a	b	b	b	b	b	b
a	b	a	a	b	b	b	b	b	b
a	b	a	a	-	b	b	b	b	b
a	b	-	a	b	b	a	b	b	b
a	b	a	a	b	b	a	b	b	b
b	a	a	a	b	b	a	b	b	b

b	a	a	a	b	b	a	b	b	b
b	a	a	a	b	b	a	b	b	b
b	a	a	a	b	b	a	b	b	-
b	a	a	a	b	b	a	b	b	b
b	a	a	a	b	b	a	b	b	b
b	a	a	a	b	b	a	b	b	b
b	a	a	a	b	b	a	b	b	b
b	a	a	a	b	b	a	b	b	b
a	a	a	b	a	a	b	a	a	b
a	a	a	b	a	a	b	a	a	b
a	a	a	b	a	a	b	a	a	b
a	a	a	b	a	a	b	a	a	b
a	a	a	b	a	a	b	a	a	b
a	a	a	b	a	a	b	a	a	b
a	a	a	b	a	a	b	a	a	b
a	a	a	b	a	a	b	a	a	b
-	a	a	b	-	a	b	a	a	b
a	a	a	b	a	a	b	-	a	b
a	a	a	b	a	a	b	a	a	b
a	a	a	b	a	a	b	a	a	b
a	a	a	b	a	a	b	a	a	b
a	a	a	b	a	a	b	a	-	b
a	a	a	b	a	a	b	a	a	b
b	a	a	b	a	a	b	b	a	b
b	a	a	b	a	a	b	-	a	b
b	a	a	b	a	a	b	b	a	a
b	a	a	b	a	a	b	b	a	a
b	a	a	b	a	b	b	b	a	a
b	a	a	b	a	b	b	b	a	a
b	a	a	b	a	b	b	b	a	b
b	a	a	b	a	b	b	b	a	b
b	a	a	b	a	b	b	b	-	b
b	a	a	b	a	b	b	b	a	b
b	a	a	b	a	b	b	b	a	b
b	a	a	b	a	b	b	b	a	b
b	a	a	b	b	b	b	b	a	b
b	a	a	b	b	b	b	b	a	b
b	a	a	b	b	b	b	b	a	b
b	a	a	b	b	b	b	b	a	b
b	a	a	b	b	b	b	b	a	b
b	a	a	b	b	b	b	b	a	b
b	a	a	b	b	b	b	b	a	b
b	a	a	b	b	b	b	b	a	b
b	b	a	b	b	a	b	b	a	b
b	b	a	b	b	a	b	b	a	b
b	b	a	b	b	a	b	b	a	b
b	b	-	b	b	a	b	b	a	b

-	b	a	b	b	b	b	b	a	b
b	b	a	b	b	a	b	b	a	b
b	b	a	b	-	a	b	b	a	b
b	b	a	b	b	a	b	-	a	b
b	b	a	b	b	a	b	b	a	b
b	b	a	a	b	a	b	b	a	b
b	b	a	a	b	a	b	b	a	a
b	b	a	a	b	a	b	b	a	a
b	b	a	a	b	a	b	b	a	a
b	b	a	a	b	a	-	b	a	a
b	b	a	a	b	a	b	b	a	a
b	b	a	a	b	a	b	b	a	a
b	b	b	b	-	a	a	b	b	a
b	b	a	b	a	a	a	b	b	a
b	b	a	b	a	a	a	b	b	a
-	b	b	b	a	a	a	b	b	a
b	b	b	b	a	a	a	b	b	a
b	b	b	b	a	a	a	b	b	a
a	b	b	b	a	b	a	b	b	a
a	b	b	b	a	b	a	b	b	a
a	b	b	b	a	b	a	b	b	a
a	b	b	b	a	b	a	b	b	-
a	b	b	b	a	b	a	b	b	a
a	b	b	b	a	b	a	b	a	a
-	b	b	b	a	b	a	b	a	a
-	b	b	b	a	b	a	b	a	a
a	b	b	b	a	b	a	b	a	a
a	b	b	b	a	b	a	b	a	a
a	b	b	b	a	b	a	b	a	a
a	a	b	b	a	b	a	b	a	a
a	b	b	b	a	b	a	b	a	a
a	b	b	b	a	b	a	b	a	a
a	b	b	b	a	b	a	b	-	a
a	b	b	b	a	b	a	b	a	a
a	b	b	b	a	b	a	b	a	a
-	b	b	b	a	b	a	b	-	a
a	b	b	b	a	b	a	b	a	a
a	b	b	b	a	b	a	b	a	a
a	b	b	b	a	b	a	b	-	-
a	b	b	b	a	b	a	b	a	a
a	b	b	b	a	b	a	b	a	a
a	b	b	b	a	b	a	b	a	-
a	b	b	b	a	b	a	b	a	a
a	b	b	b	a	b	a	b	a	a
a	b	b	b	-	b	a	b	a	a
a	b	b	b	a	b	a	b	a	a
a	b	b	b	a	b	a	b	a	a

a	b	b	b	a	b	a	b	a	a
a	b	b	b	a	b	a	b	-	-
a	b	b	a	a	b	a	b	a	a
-	b	b	b	b	b	a	b	a	-
a	b	-	b	a	a	a	a	a	a
a	b	b	b	a	a	b	a	b	a
a	b	b	b	a	a	b	a	b	a
a	b	b	b	a	a	b	a	b	a
a	a	a	a	a	a	a	b	b	a
a	a	a	a	a	a	a	b	b	a
a	a	a	a	a	a	a	b	b	a
a	a	a	a	a	a	a	b	b	a
a	a	a	a	a	a	a	b	a	a
a	a	a	a	a	a	a	b	a	a
a	a	a	a	a	a	a	b	a	a
-	a	a	a	a	a	a	b	a	a
a	a	a	a	a	a	a	b	a	a
b	a	a	a	a	a	a	b	a	a
b	a	a	a	a	a	a	b	a	a
b	a	a	a	a	a	a	b	a	a
b	b	a	a	a	a	a	b	a	a
b	b	a	a	a	a	a	b	a	a
b	b	a	a	a	a	b	b	a	a
b	b	a	a	a	b	b	b	a	a
b	b	a	a	a	b	b	b	a	a
b	b	a	a	a	b	b	b	a	a
b	b	a	a	a	b	b	b	a	a
b	b	b	a	a	b	b	b	a	a
b	b	b	a	a	b	b	b	a	a
b	b	b	a	a	b	b	b	a	a
b	b	b	a	a	b	b	b	a	a
b	b	b	a	a	b	b	b	a	a
b	b	b	a	a	b	b	b	a	a
b	b	b	a	a	b	b	b	a	a
b	b	b	a	a	b	b	b	a	a
b	b	b	a	a	b	b	b	a	a
b	b	b	a	a	b	b	b	a	a
-	b	b	a	a	b	b	b	a	a
b	b	b	a	a	b	b	b	a	a
b	b	b	a	a	b	b	b	a	a
b	b	b	a	a	b	b	b	a	a
b	b	b	a	a	b	b	b	a	a
b	b	b	a	a	b	b	b	-	a
b	b	b	a	a	b	b	b	a	a
-	b	b	a	a	b	b	b	a	a
b	b	b	a	a	b	b	b	a	a
b	b	b	a	a	b	b	b	a	a

b	b	b	a	a	b	b	b	a	a
b	b	b	a	a	b	b	b	a	a
b	b	b	a	a	b	b	b	a	a
a	b	b	a	a	b	b	b	a	a
a	b	b	a	a	b	b	b	a	a
a	b	b	a	a	b	b	b	a	a
a	b	b	a	a	b	b	b	a	b
a	b	b	a	a	b	b	b	a	b
a	b	b	a	a	b	b	b	a	b
a	b	b	a	a	b	-	b	a	b
a	b	b	a	a	a	b	b	a	b
a	b	b	a	a	a	b	b	a	b
a	b	b	a	a	a	b	b	a	b
a	b	b	a	a	a	b	b	a	b
a	b	b	a	a	a	b	b	a	b
a	b	b	a	a	a	b	b	a	b
a	b	b	a	a	a	b	b	a	b
b	b	b	-	a	a	b	b	a	b
a	b	a	a	a	a	b	a	a	b
-	b	a	a	a	a	b	a	a	b
a	b	a	a	b	b	a	a	b	b
a	b	a	a	b	b	a	a	b	b
-	b	a	a	b	b	a	a	b	b
a	b	a	a	b	b	a	a	b	b
a	b	-	a	b	b	a	-	b	b
a	b	a	a	b	b	a	a	b	b
a	b	a	a	b	b	a	a	b	b
a	b	a	a	b	b	a	a	b	b
a	b	a	a	b	b	a	a	b	b
a	b	a	a	b	b	a	a	b	b
a	b	a	a	b	b	a	a	b	b
a	b	a	a	b	b	a	a	b	b
a	b	a	a	b	b	a	a	b	b
a	b	a	a	b	b	a	a	b	b
a	b	a	a	b	b	a	a	b	b
a	b	a	a	b	-	a	a	b	b
a	b	a	a	b	b	a	a	b	b
a	b	a	a	b	b	a	a	b	b
a	b	a	a	b	-	a	a	b	b
a	b	a	a	b	b	a	a	b	b
a	b	a	a	a	b	a	-	b	b
a	b	a	a	a	b	a	a	b	b
a	b	a	a	a	b	a	a	b	b
a	b	a	a	a	b	a	a	-	b
a	b	a	a	a	b	a	a	b	b
a	b	a	a	a	b	a	a	b	b
a	b	a	a	a	b	a	a	b	b

a	b	a	a	a	b	a	a	b	b
a	b	a	a	a	b	a	a	b	b
a	b	a	a	a	b	a	a	-	b
a	b	a	a	a	b	a	a	b	b
a	b	a	a	a	b	a	a	-	-
a	b	a	a	a	b	a	a	b	b
-	b	a	a	a	b	a	a	b	b
a	b	b	a	a	b	a	a	b	b
a	b	b	a	a	b	a	a	b	b
a	b	b	a	a	b	b	a	b	b
a	b	b	a	a	b	b	a	b	b
a	b	b	a	a	b	b	a	b	b
a	b	b	a	a	b	b	a	b	b
a	a	b	-	a	b	b	a	b	b
a	a	b	a	a	b	b	-	b	b
a	a	b	b	a	b	b	b	b	b
a	a	b	b	a	b	b	b	b	b
a	a	b	b	a	b	b	b	b	b
a	a	b	b	a	b	b	b	b	b
a	a	b	b	a	b	b	b	b	b
a	a	b	b	a	b	b	b	b	b
a	a	b	b	a	b	b	b	b	b
a	a	b	b	a	b	b	b	b	b
a	a	b	b	a	b	b	b	b	b
a	a	b	b	a	b	-	b	b	b
a	a	b	b	a	b	b	b	b	b
a	a	b	b	a	b	b	b	b	b
a	a	b	b	a	b	b	b	b	b
a	a	b	b	a	b	b	b	b	b
a	a	b	b	a	b	b	-	b	b
a	a	b	b	a	b	b	b	b	b
b	a	b	b	a	b	b	b	b	b
a	a	b	b	a	b	b	b	b	b
a	a	b	b	a	b	a	b	b	b
a	a	b	b	a	b	a	b	b	-
a	a	b	a	a	a	a	b	b	a
a	a	b	a	-	b	a	b	b	-
a	a	b	a	a	b	a	b	b	a
a	a	b	a	a	b	a	b	b	a
a	a	b	a	-	b	a	b	b	-
-	a	b	a	a	b	a	b	b	a
-	a	b	a	a	b	a	b	b	a
a	a	b	a	a	b	a	b	a	a
a	a	b	a	a	b	a	b	a	a
a	a	b	a	a	b	a	b	a	a
a	a	a	a	a	b	a	b	a	a
a	a	a	b	a	b	a	b	a	a

a	a	a	b	a	b	a	-	a	a
a	a	a	b	a	b	a	b	a	a
a	a	a	b	a	b	a	b	a	a
a	a	a	b	a	b	a	b	a	a
a	a	a	b	a	b	a	b	a	a
a	a	a	b	a	b	a	a	a	a
a	a	a	b	a	b	a	a	a	a
a	a	a	b	a	b	a	a	a	a
a	a	a	b	a	b	a	a	a	a
a	a	a	a	b	b	a	a	a	b
a	a	a	a	b	b	a	a	a	b
-	a	a	a	b	b	a	-	a	b
a	a	a	a	b	b	a	-	a	b
a	a	a	a	b	b	a	a	a	b
a	a	a	a	b	b	a	a	a	b
a	a	a	a	b	b	a	a	a	b
a	a	a	a	b	b	a	a	a	b
a	a	a	a	b	b	a	a	a	b
a	a	a	a	b	b	a	a	a	b
a	a	a	a	-	b	b	a	a	b
a	b	a	a	b	b	b	a	a	b
a	b	a	a	b	b	b	a	a	b
a	b	a	a	b	a	b	a	b	b
b	b	a	a	b	a	b	a	b	b
b	b	a	a	b	a	b	a	b	b
b	b	a	a	b	a	b	a	b	b
b	b	a	a	b	a	b	a	b	b
b	b	a	a	b	a	b	a	b	b
b	b	a	a	b	a	b	a	-	b
b	b	a	a	b	a	b	a	a	b
b	b	a	a	b	a	b	a	a	b
b	b	a	a	-	a	b	-	a	b
b	b	a	a	b	a	b	a	a	b
-	b	a	a	b	a	b	a	a	b
b	b	a	a	b	a	b	a	a	b
b	b	a	a	b	a	b	a	a	-
b	b	a	a	b	a	b	a	a	b
b	b	a	a	b	a	b	a	a	b
b	b	a	a	b	a	b	a	a	b
b	b	a	a	b	a	b	a	a	b
-	b	a	a	b	a	b	a	a	-
b	b	a	a	b	a	b	a	a	b
b	b	a	a	b	a	b	a	a	b
b	b	a	a	b	a	b	a	a	b
b	b	a	a	b	a	b	a	a	b
b	b	a	a	b	a	b	a	a	b
b	b	b	a	b	b	a	a	b	a

b	b	b	a	b	b	a	a	b	a
b	b	b	a	b	b	a	a	b	a
b	b	b	a	b	b	a	a	b	a
b	b	b	a	b	b	a	a	b	a
b	b	b	a	b	b	a	a	b	a
b	b	b	a	b	b	a	a	b	a
b	b	b	a	b	b	a	a	b	a
b	b	b	a	b	b	a	a	b	a
b	b	b	a	b	b	a	a	b	a
b	b	b	a	b	b	a	a	b	a
b	b	b	a	b	b	a	a	b	a
b	b	b	a	b	b	a	a	b	a
b	b	b	a	b	b	a	a	b	a
b	b	b	a	b	b	a	a	b	a
b	b	b	a	b	b	a	a	b	a
b	b	b	a	a	b	a	a	b	a
b	b	b	a	a	b	a	a	b	a
b	b	b	a	b	b	b	b	b	a
b	b	b	a	a	b	a	a	b	a
b	b	b	a	a	b	a	a	b	a
b	b	b	a	a	b	a	a	b	a
b	b	b	a	a	b	a	a	b	a
b	b	b	a	a	b	a	a	b	a
b	b	b	a	a	b	a	a	b	a
b	b	b	a	a	b	a	a	b	a
b	b	b	a	a	b	a	a	b	a
b	b	b	a	a	b	a	a	b	a
b	b	b	a	a	b	a	a	b	a
b	b	b	a	a	a	a	a	b	a
b	b	b	a	a	a	a	a	b	a
b	b	b	a	a	a	a	a	b	a
-	b	b	a	a	a	a	a	b	a
a	b	b	a	a	a	a	a	b	a
a	b	b	a	a	a	a	a	b	a
a	a	b	a	a	a	a	a	b	a
a	a	b	a	a	a	a	a	b	a
a	a	b	a	a	a	a	a	b	a
a	a	b	a	a	a	a	a	b	a
a	b	b	a	a	a	a	a	b	a
a	a	b	a	a	a	a	a	b	a
a	b	a	b	b	b	b	b	a	b
b	b	a	a	a	a	b	a	b	b
b	b	a	a	a	a	b	a	b	b
b	b	a	a	a	a	b	a	b	b
b	b	a	a	a	a	b	a	b	b
b	b	a	a	a	a	b	a	b	b
b	b	a	a	b	a	b	a	b	b

b	b	a	a	b	a	b	a	b	b
b	b	a	a	b	a	b	a	b	b
a	b	a	a	b	a	b	a	b	b
a	b	a	a	b	a	b	a	b	b
a	b	a	a	b	a	b	a	a	b

YW039	YW040	YW041	YW042	YW043	YW044	YW045	YW046	YW047	YW048
b	b	a	a	b	b	a	a	b	b
b	b	a	a	b	b	a	a	b	b
b	b	a	a	b	b	a	a	b	b
b	b	a	a	b	b	a	a	b	b
b	b	a	a	b	b	a	a	b	b
b	b	a	a	b	b	a	a	b	b
b	b	a	a	b	b	a	a	b	b
b	b	a	a	b	b	a	a	b	b
b	b	a	a	b	b	a	a	b	b
b	b	a	a	b	b	a	a	b	b
b	b	a	a	b	b	a	a	b	b
b	b	a	a	b	b	a	a	b	b
b	b	a	a	b	b	a	a	b	b
b	b	a	a	b	b	a	a	b	b
b	b	a	a	b	b	a	a	b	b
b	b	a	a	b	b	a	a	b	b
b	b	a	-	b	b	a	a	b	b
b	b	a	a	b	b	a	a	b	-
b	b	a	a	b	b	a	a	b	b
b	b	a	b	b	b	a	a	b	b
b	b	a	a	b	b	a	a	b	b
b	b	a	a	b	b	a	a	b	b
b	b	a	a	b	b	a	a	b	b
b	b	a	-	b	b	a	a	b	b
b	b	a	a	b	b	a	a	b	b
b	b	a	a	b	b	a	a	b	b
b	b	a	a	b	b	b	a	b	b
b	b	a	a	b	b	b	a	b	b
b	b	b	a	b	b	b	a	b	b
b	b	b	a	b	b	b	a	b	b
-	b	b	a	b	b	b	a	b	b
b	b	b	a	b	b	b	a	b	b
b	b	b	a	b	b	b	a	b	b
b	b	b	a	b	a	b	a	b	b
b	b	b	a	b	a	b	a	b	b
b	b	b	-	b	a	b	a	b	b
b	b	b	a	b	a	b	a	b	b
b	b	b	a	b	a	b	a	b	b
b	b	b	a	b	a	b	a	b	b
b	b	b	a	b	a	b	a	b	b
b	b	b	a	b	a	b	a	b	b
a	b	b	a	a	a	b	a	a	b
a	b	b	a	a	a	b	a	a	b
a	b	b	a	a	a	b	a	a	b
a	b	b	a	a	a	b	a	a	b

a	b	-	a	a	a	b	a	a	b
a	b	b	a	a	a	b	a	a	b
a	b	b	a	a	a	b	a	a	b
-	b	b	a	a	a	b	a	a	b
a	b	b	a	a	a	a	a	a	b
a	b	b	a	a	a	a	a	a	b
a	b	b	a	a	a	a	a	a	b
a	b	b	a	a	a	a	a	a	b
a	b	b	a	a	a	a	a	a	b
a	b	b	a	a	a	a	a	a	b
a	b	b	a	a	a	a	a	a	b
-	b	b	a	a	a	a	a	a	b
a	b	b	a	a	a	a	a	a	b
a	b	b	a	a	a	a	a	a	b
a	b	b	a	b	a	a	a	a	b
a	b	b	a	b	a	a	a	a	b
a	b	b	a	b	a	a	a	a	b
a	b	b	a	b	a	a	a	a	b
a	b	b	a	b	a	a	a	a	b
a	b	b	a	b	a	a	a	a	b
-	b	b	a	b	a	a	a	a	b
a	b	a	a	b	a	a	a	a	b
a	b	a	a	b	a	a	a	a	b
a	b	a	a	b	a	a	a	a	b
a	b	a	a	b	a	a	a	a	b
a	b	a	a	b	a	a	a	a	b
a	b	a	a	b	a	a	a	a	b
a	b	a	a	b	a	a	a	a	b
-	b	-	a	b	a	a	a	a	b
a	b	a	a	b	a	a	a	a	b
a	b	a	a	b	a	a	a	a	b
a	b	a	a	b	a	a	a	a	b
a	b	a	a	b	a	a	a	a	b
a	b	a	a	b	a	a	a	a	b
a	b	a	a	b	a	a	a	a	b
a	b	a	a	b	a	a	b	a	b
a	b	a	a	b	a	a	b	a	b
a	b	a	a	b	a	a	a	a	b
a	b	a	a	b	a	a	a	a	b
a	b	b	a	a	a	b	a	b	a
a	b	b	a	a	a	b	a	b	a
a	-	b	a	a	a	b	a	b	a
a	b	b	a	a	a	b	a	b	a
a	b	b	a	a	a	b	a	b	a
a	b	b	a	a	a	b	a	b	a
a	b	b	a	a	a	a	a	b	a
a	-	b	a	a	a	a	a	b	a

-	b	b	a	a	a	a	a	b	a
a	b	b	a	a	a	a	a	b	a
a	b	b	a	a	a	a	a	b	a
a	b	b	a	a	a	a	a	b	a
a	b	b	a	a	a	a	a	b	a
a	b	b	a	a	a	a	a	b	a
a	b	b	a	a	a	a	a	b	a
a	b	b	a	a	a	a	a	b	a
a	b	b	a	a	a	a	a	b	a
a	b	b	a	a	a	a	a	b	a
a	b	b	a	a	a	a	a	b	a
a	b	b	a	a	a	a	a	b	a
a	b	b	a	a	a	a	a	b	a
a	b	b	a	a	a	a	a	b	a
a	b	b	a	a	a	a	a	b	a
a	b	b	a	a	a	a	a	b	a
a	b	b	a	a	a	a	a	b	a
-	b	a	a	a	a	a	a	b	a
a	b	a	a	a	a	a	a	b	a
a	b	a	a	a	a	a	a	b	a
a	b	a	a	a	a	a	a	b	a
a	-	a	a	a	a	a	a	b	a
a	b	a	-	a	a	a	a	b	a
a	b	a	a	a	a	a	a	b	a
-	b	a	a	-	a	a	a	b	a
a	b	a	a	a	a	a	a	b	a
a	b	a	-	a	a	a	a	b	a
a	b	a	a	a	a	a	a	b	a
a	b	a	a	a	a	a	a	b	a
a	b	a	a	a	a	a	a	b	a
a	-	a	a	a	a	a	a	b	a
a	b	a	a	a	a	a	a	b	a
a	b	a	a	a	a	a	a	b	a
a	b	a	a	a	a	a	a	b	a
a	b	a	a	a	a	a	a	b	a
a	b	a	a	a	a	a	a	b	a
a	b	a	a	a	a	a	a	b	a
a	b	a	a	a	a	a	a	b	a
-	b	a	b	a	a	a	a	b	a
a	b	a	b	a	a	a	a	b	a
a	b	a	b	a	a	a	a	b	a
a	b	a	b	a	a	a	a	b	a
a	b	-	b	a	a	a	a	b	a
a	b	a	b	a	a	a	a	b	a
a	b	a	b	a	b	a	a	b	a
a	b	a	b	a	b	a	a	b	a
a	b	a	b	a	b	a	a	b	a

a	b	a	b	a	b	a	a	b	a
a	b	a	b	a	b	a	a	b	a
-	b	a	b	a	b	a	a	b	b
a	b	a	b	a	b	a	a	a	b
a	b	a	b	a	-	a	a	a	b
-	b	a	b	a	b	a	a	a	b
a	b	a	a	a	b	a	a	a	b
a	b	a	b	a	b	a	a	a	b
a	b	a	b	a	b	a	a	a	b
a	b	a	b	a	b	a	a	a	b
a	b	a	b	a	b	a	a	a	b
a	b	a	b	a	b	a	a	a	b
a	b	-	b	a	b	a	a	a	b
a	b	a	b	a	b	a	a	a	-
a	b	a	b	a	b	a	a	a	b
a	b	a	b	a	b	a	a	a	b
a	b	a	b	a	b	a	a	a	b
a	b	a	b	a	b	a	a	a	b
a	b	a	b	a	b	a	a	a	b
a	b	a	b	a	b	a	a	a	b
a	b	a	b	a	b	a	a	a	-
a	b	a	b	a	b	a	a	a	b
a	b	a	b	a	b	a	a	a	b
a	b	a	b	a	b	a	a	a	b
a	a	a	b	a	b	a	a	a	a
a	a	a	b	a	b	a	a	a	a
a	a	a	b	a	b	a	a	a	a
a	a	a	b	a	b	a	a	a	a
a	a	a	b	a	b	a	a	a	a
a	a	a	b	a	b	a	a	a	a
a	a	a	b	a	b	a	a	a	a
a	a	a	b	a	b	a	a	a	a
a	a	a	b	a	b	a	a	a	a
a	a	a	b	a	b	a	a	a	a
-	a	a	b	a	b	a	a	a	a
a	a	a	b	a	b	a	a	a	a
-	a	a	b	a	b	a	a	a	a
a	a	a	b	a	b	a	a	a	a
a	a	a	b	a	b	a	a	a	a
a	a	a	b	a	b	a	a	a	a
a	a	a	b	a	b	a	a	a	a
a	a	a	b	a	b	a	a	a	a
a	a	a	b	a	b	a	a	a	a
a	a	a	b	a	b	a	a	a	b
a	a	a	a	a	b	a	a	a	b
a	a	a	-	b	a	a	a	a	b
a	a	a	b	b	a	a	a	a	b

a	a	a	b	b	a	a	a	a	b
a	a	a	b	b	a	a	a	a	b
a	a	a	b	b	a	a	a	a	b
a	a	a	b	b	a	b	a	a	b
a	a	a	b	b	a	b	a	a	b
a	a	a	b	b	a	b	a	a	b
a	a	a	b	b	a	b	a	a	b
a	a	a	b	b	a	b	a	a	b
a	a	a	-	b	a	b	a	a	b
a	a	a	b	b	a	b	a	a	b
a	a	a	b	b	a	b	a	a	b
a	a	a	b	b	a	b	a	b	b
a	b	-	a	a	a	b	a	a	b
a	b	b	a	a	a	b	a	a	-
-	b	b	a	a	a	b	a	a	b
a	b	b	a	a	a	b	a	b	b
a	b	b	a	a	a	b	a	b	-
a	b	b	a	a	a	b	a	b	b
a	b	-	a	a	a	b	b	b	b
a	b	b	a	b	a	b	b	b	b
a	b	b	a	b	a	b	b	b	b
a	b	b	a	b	a	b	b	b	b
a	b	b	a	b	a	b	b	b	b
a	b	b	a	b	a	b	b	b	b
a	b	b	a	b	a	b	b	b	b
a	b	b	a	b	a	b	b	b	b
a	b	b	a	b	a	b	b	b	b
a	b	b	a	b	a	b	b	b	b
a	b	b	a	b	a	b	b	b	b
a	b	b	a	b	b	b	b	b	b
a	b	b	a	b	b	b	b	b	b
a	b	b	a	b	b	b	b	b	b
a	b	b	a	b	b	b	b	b	b
a	b	b	a	b	b	b	b	b	b
a	a	b	a	b	b	b	b	b	b
a	a	b	a	b	b	b	b	b	b
b	-	b	a	b	b	b	b	b	b
a	a	b	a	b	b	b	b	b	b
-	a	b	a	b	b	b	b	b	b
-	a	b	a	b	b	-	b	b	b
a	a	b	a	b	b	a	b	b	b
a	a	b	a	b	b	a	b	b	b
a	a	b	a	b	b	a	b	b	b
b	a	b	a	b	b	a	b	b	b
b	a	b	a	b	b	a	b	b	b
b	a	b	a	b	b	a	b	b	-
b	a	b	a	b	b	a	b	b	b

b	a	b	a	b	b	a	b	b	b
b	a	b	a	b	b	a	b	b	-
b	a	b	a	b	b	a	b	b	b
b	a	b	a	b	b	a	b	b	b
b	a	b	a	b	a	a	b	b	b
b	a	b	a	b	a	a	b	b	b
b	a	b	a	b	a	a	b	a	a
b	a	b	a	b	a	a	b	a	a
b	a	b	-	b	a	a	b	a	a
b	a	b	a	b	a	a	b	a	a
b	a	b	a	b	a	a	b	a	a
b	a	b	a	b	a	a	b	a	a
b	a	b	a	b	a	a	b	a	a
b	a	b	a	b	a	a	b	a	a
b	a	b	a	b	a	a	b	a	a
b	a	a	a	b	a	a	b	a	a
b	a	a	a	b	a	a	b	a	a
b	a	a	a	b	a	a	b	a	-
b	a	a	a	b	a	a	b	a	a
b	a	a	a	b	a	a	b	a	a
b	a	a	a	b	a	a	b	a	a
b	a	a	a	b	a	a	b	a	a
b	a	a	a	b	a	a	b	a	-
b	a	a	a	b	a	a	b	a	a
b	a	a	a	b	a	a	b	b	a
b	a	a	a	b	a	a	b	b	a
b	a	a	a	b	a	a	b	b	a
b	a	a	a	b	a	a	b	b	a
b	a	a	a	b	a	a	b	b	a
b	a	a	a	b	a	a	b	b	a
b	a	a	b	b	a	a	b	b	a
b	a	a	a	b	a	a	b	b	a
b	a	a	a	b	a	a	b	b	a
b	a	a	a	b	a	a	b	b	a
b	a	a	a	b	a	a	b	b	a
b	a	a	a	b	a	a	b	b	a
a	a	a	a	b	a	a	b	b	a
b	a	a	a	b	a	a	b	b	a
b	a	a	a	b	a	a	b	b	a
-	a	a	a	b	a	a	b	b	a
b	a	a	a	b	a	a	b	b	a
b	a	a	a	b	a	a	b	b	a
b	b	a	a	b	a	a	b	b	a
b	b	a	a	b	a	a	b	b	a
b	b	a	a	b	a	a	b	b	a
b	b	a	a	b	a	a	b	b	a
b	b	a	a	b	a	a	b	b	a

b	b	a	a	b	a	a	b	b	a
b	b	a	a	b	a	a	b	b	a
b	b	a	a	b	b	a	b	b	a
b	b	a	a	b	a	a	b	b	a
b	b	a	a	b	a	a	b	b	a
b	b	a	a	b	a	a	b	b	a
b	b	a	-	b	a	a	b	b	a
a	b	a	a	b	a	a	b	b	a
a	b	a	a	b	a	a	b	b	a
a	b	a	a	b	a	a	b	b	a
a	b	a	a	b	a	a	b	b	a
a	b	a	a	b	a	a	b	b	a
a	b	a	a	b	a	a	b	b	a
a	b	a	a	b	a	a	b	b	a
a	b	a	a	b	a	a	b	b	a
a	b	a	a	b	b	a	b	b	a
a	b	a	a	b	b	a	b	b	a
a	b	a	a	b	b	a	b	b	a
a	b	a	b	b	b	a	b	b	a
a	b	a	a	b	b	a	b	b	a
a	b	a	a	b	b	a	b	b	a
a	b	a	a	b	b	a	b	b	a
a	b	a	a	b	b	a	b	b	a
a	b	-	b	b	b	a	b	b	a
a	b	a	a	b	b	a	b	b	a
a	b	a	a	b	b	a	b	b	a
a	b	a	a	b	b	a	b	b	a
-	b	a	a	b	b	a	b	b	a
a	b	a	a	b	b	a	b	b	a
a	b	a	a	a	b	a	a	b	a
a	b	a	a	a	b	a	a	b	a
a	b	a	a	a	b	a	a	b	a
a	b	a	a	a	b	a	a	b	a
a	b	a	a	a	b	a	a	b	a
a	b	a	a	a	b	a	a	b	a
a	b	a	a	a	b	a	a	b	a
a	b	a	a	a	b	a	a	b	a
a	b	a	a	a	b	a	a	b	a
a	a	b	b	b	b	a	b	b	b
a	a	b	b	b	b	a	b	b	b
a	a	b	b	b	b	b	b	b	b
a	a	b	b	b	b	b	b	b	b
a	a	b	b	b	b	b	b	b	b
a	a	b	b	b	b	b	b	b	b
a	a	b	b	b	b	b	b	b	b
a	a	b	b	b	b	b	b	b	b
a	a	b	b	b	b	b	b	b	b

a	a	b	b	b	b	b	b	b	b
a	a	b	b	b	b	b	b	b	b
a	a	b	b	b	b	b	b	b	b
a	a	b	b	b	b	b	b	b	b
a	a	b	b	b	b	b	b	b	b
-	a	b	b	b	b	b	b	b	-
a	a	b	b	b	b	b	b	b	b
a	a	b	b	a	b	b	b	b	-
a	a	b	b	a	b	b	b	b	b
a	a	-	b	a	b	b	b	b	b
-	a	b	b	a	b	b	b	b	b
a	a	b	b	a	b	b	b	b	b
a	a	b	b	a	b	b	b	b	b
a	a	b	b	a	b	b	b	b	b
a	a	b	b	a	b	b	b	b	b
a	a	b	b	a	b	b	b	b	b
a	a	b	b	a	b	b	b	b	b
a	a	b	b	b	b	b	b	b	b
a	a	b	b	b	b	b	b	b	b
a	a	b	b	b	b	b	b	b	b
a	a	b	b	b	b	b	b	b	b
a	a	-	b	b	b	b	b	b	-
a	a	b	b	b	b	b	b	b	b
-	a	b	b	b	b	b	b	b	b
-	a	b	b	b	b	b	b	b	b
b	a	b	b	b	b	b	b	b	b
b	a	b	b	b	b	b	b	b	b
b	a	b	b	b	b	b	b	b	b
b	a	b	b	b	b	b	b	b	b
b	a	b	b	b	b	b	b	b	b
b	a	b	b	b	b	b	b	b	b
b	a	b	b	b	b	b	b	b	b
b	a	b	b	b	b	b	b	b	b
b	a	b	b	b	b	b	b	b	b
b	a	b	b	b	b	b	b	b	b
b	a	b	b	a	b	b	b	b	b
-	a	b	b	b	b	b	b	b	b
b	a	b	b	b	b	b	b	b	b
b	a	b	b	b	b	b	b	b	b
b	a	b	b	b	b	b	b	b	b
b	a	b	b	b	b	b	b	b	b
b	a	b	b	b	b	b	-	b	b
b	a	b	b	b	b	a	b	b	b
b	a	b	b	b	b	a	b	b	b
b	a	b	b	b	b	a	b	b	b
b	a	b	b	b	b	a	b	b	-
b	a	b	b	b	b	a	b	b	b

b	a	b	b	b	b	a	b	b	b
b	a	b	b	b	b	a	b	b	b
b	a	b	b	b	b	a	b	b	b
b	a	b	b	b	b	a	b	b	b
b	a	b	b	b	b	a	b	b	b
b	a	b	b	b	-	a	b	b	b
b	a	b	b	b	b	a	b	b	b
b	a	b	b	b	b	a	a	b	b
b	a	b	b	b	b	a	a	b	b
b	a	b	b	b	b	a	a	b	b
b	a	b	b	b	a	a	a	b	b
b	a	b	b	b	a	a	a	b	b
b	a	b	b	b	a	a	a	b	b
b	a	b	b	b	a	a	a	b	b
b	a	b	b	b	a	a	a	b	b
a	a	b	b	b	a	a	a	b	b
a	a	b	b	b	a	a	a	b	b
a	a	b	b	b	a	a	a	a	b
a	a	b	b	b	a	a	a	a	b
a	a	b	b	b	a	a	a	a	b
a	a	b	b	b	a	a	a	a	b
a	a	b	b	b	a	a	a	a	b
a	a	b	b	b	a	a	a	a	b
a	a	b	b	b	a	a	a	a	b
a	a	a	b	b	a	a	a	a	b
a	a	a	a	b	a	a	a	a	b
a	a	a	a	b	a	a	a	a	b
a	a	a	a	b	a	a	a	a	b
a	a	a	a	b	a	a	a	a	b
a	a	a	a	b	a	a	a	a	b
a	a	a	a	b	a	a	a	a	b
a	a	a	a	b	a	a	a	a	-
a	a	a	a	b	a	a	a	a	b
a	a	-	a	b	a	a	a	a	b
a	a	a	a	b	a	a	a	a	b
a	a	a	a	b	a	a	a	a	b
a	a	a	a	b	a	a	a	a	b
-	a	a	a	b	a	a	a	a	b
a	a	a	a	b	a	a	a	a	b
a	a	a	a	b	a	a	a	a	-
a	a	a	a	a	a	a	b	a	b
a	a	a	a	b	a	a	b	a	b
a	a	a	a	b	a	a	b	a	b
a	a	a	a	b	a	a	b	a	b
a	a	a	a	b	a	a	b	a	b
a	a	b	a	b	a	a	b	a	b
a	a	b	a	b	a	a	b	a	b

a	a	b	a	b	a	a	b	a	b
a	a	b	a	b	a	a	b	a	b
a	a	b	a	b	a	a	b	a	b
a	a	b	a	b	a	a	b	a	b
a	a	b	a	b	a	a	b	a	b
a	-	b	a	b	a	a	b	b	b
a	a	b	a	b	a	a	b	b	b
a	a	b	a	b	a	a	b	b	b
a	a	b	a	b	a	a	b	b	b
a	a	b	a	b	a	a	b	b	b
a	a	b	a	b	a	a	b	b	b
a	a	b	a	b	a	a	b	b	b
a	a	b	a	b	a	a	b	b	b
a	a	b	a	b	a	a	b	b	b
a	a	b	a	b	a	a	b	b	b
a	a	b	a	b	a	a	b	b	b
a	a	b	a	b	a	a	b	b	b
a	a	b	a	b	a	a	b	b	b
a	a	b	-	b	a	a	b	b	b
a	a	b	a	b	a	a	b	b	b
a	a	b	a	b	a	a	b	b	b
a	a	b	a	b	a	a	b	b	b
a	a	b	-	b	a	a	b	b	b
a	a	b	a	b	a	a	b	b	b
a	a	b	a	b	a	a	b	b	b
a	a	b	a	b	a	a	b	b	b
a	a	b	a	b	a	a	b	b	b
a	a	b	a	b	a	a	b	b	b
a	a	b	a	b	a	a	b	b	b
a	a	b	a	b	a	a	b	b	b
a	a	-	a	b	a	a	b	b	b
a	a	-	a	b	a	a	b	b	b
a	a	b	a	b	a	a	b	-	b
a	a	b	a	b	a	a	b	b	b
a	a	b	a	b	a	a	b	b	b
a	a	b	a	b	a	a	b	b	b
a	a	b	a	b	a	a	b	b	b
a	a	b	-	b	a	a	b	b	b
a	a	b	a	b	a	a	b	b	b
a	a	b	a	b	-	a	b	b	b
a	b	b	a	b	a	a	b	b	b
a	b	b	a	b	a	a	b	a	b
a	b	b	a	b	a	a	b	a	b
a	b	b	a	b	a	a	b	a	b
a	b	b	a	b	a	a	b	a	b
a	b	b	a	b	a	a	b	a	b
a	b	b	a	b	a	a	a	a	b

a	b	b	a	b	a	a	a	a	b
a	b	b	a	b	a	b	a	a	b
a	b	b	a	b	a	b	a	a	b
b	b	b	a	b	a	b	a	a	b
b	b	-	b	b	a	b	a	a	b
b	-	b	b	b	a	b	a	a	b
b	b	b	b	b	a	b	a	a	b
-	b	b	b	b	a	b	a	a	-
b	b	b	b	b	a	b	a	a	b
b	b	b	b	b	a	b	a	a	b
b	b	b	b	b	a	b	a	a	b
-	b	b	b	b	a	b	a	b	b
b	b	b	b	b	a	b	a	b	b
b	b	b	b	b	a	b	a	b	b
b	b	b	b	b	a	b	a	b	b
b	b	b	b	b	a	b	a	b	b
b	b	b	b	b	b	b	a	b	b
b	b	b	b	-	b	b	a	b	b
b	b	b	b	b	b	b	a	b	b
-	b	b	b	b	b	b	a	b	-
b	b	b	b	b	b	b	a	b	b
b	b	b	b	b	b	b	a	b	b
b	b	b	b	b	b	b	a	b	b
b	b	b	b	b	b	b	a	b	b
b	b	b	b	b	b	b	a	b	b
b	b	b	b	a	b	b	a	b	b
b	b	b	b	a	b	b	a	b	b
b	b	b	b	a	b	b	a	b	b
b	b	b	b	a	b	b	a	b	b
b	b	b	b	a	b	b	a	b	b
b	b	b	b	a	b	b	a	b	b
b	b	b	b	a	b	b	a	b	b
b	b	b	b	a	b	b	a	b	b
b	b	b	b	a	b	b	a	b	-
b	b	b	b	a	b	b	a	b	b
b	b	b	b	a	b	b	a	b	b
b	b	b	b	a	b	b	a	b	b
b	b	b	b	a	b	b	b	b	b
b	b	b	b	a	b	b	b	b	b
b	b	b	b	a	b	b	b	b	b
b	b	b	b	a	b	b	b	b	-
b	b	b	b	a	b	b	b	b	b
b	b	b	-	a	b	b	b	b	b
b	b	b	b	a	b	b	b	b	b
b	b	b	b	a	b	b	b	b	b
b	b	b	b	a	b	b	b	b	b
-	b	b	b	a	b	b	b	b	-

a	b	b	b	a	b	b	b	b	b
a	b	b	b	a	b	b	b	b	b
a	b	b	b	a	b	b	b	b	b
a	b	b	b	a	b	b	b	b	b
a	b	b	b	a	b	b	b	b	b
a	b	b	b	a	b	a	b	b	b
a	b	b	b	a	b	a	b	b	b
a	b	b	b	a	b	a	b	b	b
a	b	b	b	a	b	a	b	b	b
a	b	b	b	a	b	a	b	b	b
a	b	-	b	a	b	a	b	b	b
-	b	b	b	a	b	a	b	b	b
a	b	b	b	a	b	a	b	b	b
a	b	b	b	a	b	a	b	b	-
-	b	b	b	a	b	a	b	b	b
a	b	b	-	a	b	a	b	b	b
a	b	b	b	a	b	a	b	b	b
a	b	-	b	a	b	a	b	b	b
a	b	b	b	a	b	a	b	b	b
a	a	b	a	a	a	a	a	a	-
-	a	b	a	a	a	a	a	a	a
a	a	b	a	a	a	a	a	a	b
a	a	b	a	a	a	a	a	a	b
a	a	b	a	a	a	a	a	a	b
a	a	b	a	a	a	a	a	a	-
a	a	b	a	a	a	a	a	a	b
a	a	b	a	a	a	a	a	a	b
a	a	b	a	a	a	a	a	a	b
a	a	b	a	a	a	a	a	a	-
a	a	b	a	a	a	a	a	a	b
a	a	b	a	a	a	a	a	a	b
a	a	b	a	a	a	a	a	a	b
a	a	b	a	a	a	a	a	a	b
a	a	b	a	a	a	a	a	a	b
a	a	b	a	-	a	a	a	a	b
a	a	b	a	a	a	a	a	a	-
a	a	b	a	a	a	a	a	a	b
a	a	b	a	a	a	a	a	a	b
a	a	b	a	a	a	a	a	a	b
a	a	b	a	a	a	a	a	a	a
a	a	b	a	a	a	a	a	a	a
a	a	b	b	a	a	a	a	a	-
a	a	b	a	a	a	a	a	a	a
a	a	b	a	a	a	a	b	a	a
a	a	b	a	a	a	a	b	a	a
a	a	b	a	a	a	a	b	a	a
a	a	b	a	a	a	a	b	a	a

a	a	-	a	a	a	a	b	a	a
a	a	b	a	a	a	a	b	a	a
a	a	b	a	a	a	a	b	a	a
a	a	b	a	a	a	a	b	a	a
a	a	b	a	a	a	a	b	a	a
a	a	b	a	a	a	a	b	a	a
a	a	b	a	a	a	a	b	a	a
a	a	b	a	a	a	a	b	a	a
a	a	b	a	a	a	a	b	a	a
-	a	b	a	a	a	a	b	a	a
a	a	b	a	a	a	a	b	a	a
a	a	b	a	a	a	a	b	a	a
a	a	b	a	a	a	a	b	a	a
a	a	b	a	a	a	a	b	a	a
a	a	b	a	a	a	a	b	a	a
a	a	b	a	a	a	a	b	a	a
a	a	b	a	a	a	a	b	a	a
a	a	b	a	a	a	a	b	a	a
a	a	-	a	a	a	a	b	a	a
a	a	a	a	a	a	a	b	a	a
a	a	a	a	a	a	a	b	a	a
a	a	a	a	a	a	a	b	a	a
a	a	a	a	a	a	a	b	a	a
a	a	a	a	a	a	a	b	a	b
a	a	a	a	a	a	a	b	a	b
a	a	a	a	a	a	a	b	a	b
a	a	a	a	a	a	a	b	a	b
a	a	a	a	a	a	a	b	a	b
a	a	a	a	a	a	a	b	a	b
a	a	a	a	a	a	a	b	a	b
a	a	a	a	a	a	a	b	a	b
a	a	a	a	a	a	a	b	a	b
a	a	-	a	a	a	a	b	a	b
a	-	a	a	a	b	a	b	a	b
a	a	a	a	a	b	a	b	a	b
a	a	a	a	a	b	a	b	a	b
a	a	a	a	a	b	a	b	a	b
a	a	a	a	a	b	a	b	a	b
a	a	a	a	a	b	a	b	a	b
a	a	a	a	a	b	a	b	a	b
a	a	a	a	a	b	a	b	a	b
a	a	a	a	a	b	a	b	a	b
a	a	a	a	a	a	a	b	a	b
a	a	a	a	a	b	a	b	a	-
a	a	a	a	b	b	a	b	a	b
a	a	a	a	b	b	a	b	a	b
a	a	a	a	b	b	a	b	a	b

a	a	a	a	b	b	a	b	a	b
a	a	a	a	b	b	a	b	a	b
a	a	a	a	b	b	a	b	a	b
a	a	a	a	b	b	a	a	a	b
b	a	a	a	b	b	a	a	b	-
b	a	a	a	b	a	a	a	b	b
b	a	a	a	b	b	a	a	b	b
b	a	a	a	b	b	a	a	b	b
b	a	a	a	b	b	a	a	b	b
b	a	a	a	b	b	a	a	b	-
b	a	b	a	b	b	a	a	b	b
-	a	a	b	a	a	b	a	b	a
b	a	a	b	a	a	b	a	b	a
b	a	a	b	a	a	b	a	b	a
b	a	a	b	a	a	b	a	b	a
b	a	a	b	a	a	b	a	b	a
b	a	a	b	a	a	b	a	b	a
b	a	a	b	a	a	b	a	b	a
b	a	a	b	a	a	b	a	b	a
b	a	a	b	a	a	b	a	b	a
b	a	a	b	a	a	b	a	b	a
b	a	a	b	a	a	b	a	b	a
b	a	a	b	a	a	b	a	b	a
b	a	a	b	a	a	b	a	b	a
b	a	a	b	a	a	b	a	b	a
b	a	a	b	a	a	b	a	b	a
b	a	a	b	a	a	b	a	b	a
-	a	a	b	a	a	b	a	-	a
b	a	a	b	a	a	b	b	b	a
b	a	a	-	a	a	b	b	b	a
b	a	a	b	a	a	b	b	b	a
b	a	a	b	a	a	b	b	b	a
b	a	a	b	a	a	b	b	b	a
b	a	a	b	a	a	b	b	b	a
b	a	a	b	a	a	b	b	b	a
b	a	a	b	a	a	b	b	b	a
b	a	a	b	b	a	b	b	b	a
b	a	a	b	b	a	a	b	-	a
b	a	a	b	b	a	a	b	b	a
b	a	a	b	b	a	a	b	b	a
b	a	a	b	b	a	a	b	b	a
b	a	a	b	b	a	a	b	b	-
b	-	a	b	b	a	a	b	b	a
b	a	a	b	b	a	a	b	b	a
b	a	a	b	b	a	a	b	b	a
b	a	a	b	b	a	a	b	b	a
b	a	a	b	-	a	a	b	b	a

b	a	a	b	b	a	a	b	b	-
b	a	a	b	b	a	a	b	b	a
b	a	a	b	b	a	a	b	-	a
b	a	a	b	b	a	a	b	b	a
b	a	a	b	b	a	a	b	b	a
b	a	a	b	b	a	a	b	a	a
-	a	a	b	b	a	a	b	a	a
b	a	a	b	b	a	a	b	a	a
b	a	a	b	b	a	a	b	a	a
b	a	a	b	b	a	a	b	a	a
b	a	a	b	b	a	a	b	a	a
b	a	a	b	b	a	a	b	a	a
b	a	a	b	b	a	a	b	a	a
-	a	a	b	b	a	a	b	a	a
b	a	a	b	b	a	a	b	-	-
b	a	a	b	b	a	a	b	a	a
b	a	a	b	b	a	a	b	a	a
b	a	a	b	b	a	a	b	a	a
b	a	a	b	b	a	a	b	a	a
b	a	a	b	b	a	a	b	a	b
b	a	a	b	b	a	a	b	a	b
b	a	a	b	b	a	a	a	a	b
b	a	a	b	b	a	a	a	a	b
b	a	a	b	b	a	a	a	a	-
b	a	a	b	b	a	a	a	a	b
b	a	a	b	b	a	a	a	a	b
b	a	a	b	b	a	a	a	a	b
b	a	a	b	b	a	a	a	a	b
b	a	a	b	b	a	a	a	a	b
b	a	a	b	b	a	a	a	a	b
b	a	a	b	b	a	a	a	a	b
b	a	a	b	b	a	a	a	a	b
b	a	a	b	b	a	a	a	a	-
b	a	a	b	b	a	a	a	a	b
b	a	a	b	b	a	a	a	a	b
b	a	a	b	b	b	a	a	a	b
b	a	b	b	b	b	a	a	a	b
b	a	b	b	b	b	a	a	a	b
b	a	-	b	b	b	a	a	a	-
b	a	b	b	b	b	a	a	a	b
b	a	b	b	b	b	a	a	a	b
b	a	b	b	b	b	a	a	a	b
b	a	b	b	b	b	a	a	a	b
b	a	b	b	b	b	a	a	a	b
b	a	b	b	b	b	a	a	a	b
b	a	b	b	b	b	a	a	a	b
b	a	b	b	b	b	a	a	a	b

b	a	b	b	b	b	a	a	a	b
b	a	b	b	b	b	a	a	a	b
b	a	b	b	b	b	a	a	a	b
b	a	b	b	b	-	a	a	a	b
b	a	b	b	b	b	a	a	a	b
b	a	a	b	b	b	a	a	a	b
b	a	a	b	b	b	a	a	a	b
b	a	-	b	b	b	a	a	a	b
-	-	a	b	b	b	a	a	a	b
b	a	a	b	b	b	a	a	a	b
b	a	a	b	b	b	a	b	a	b
b	a	a	b	b	b	a	b	a	-
b	a	a	b	b	b	a	b	a	b
b	a	a	b	b	-	a	b	a	b
-	a	a	b	-	b	a	b	a	b
b	a	a	b	b	b	a	b	a	b
b	a	a	b	b	b	a	b	a	b
b	a	a	b	b	b	a	b	a	b
b	a	a	b	b	b	a	b	a	b
b	a	a	b	b	b	a	b	a	b
b	a	a	b	b	b	a	b	a	a
b	a	a	b	b	b	a	b	a	-
-	b	b	b	b	a	a	a	a	b
b	b	b	b	b	a	a	a	a	b
-	b	b	b	b	a	a	a	a	b
b	b	b	b	b	a	a	a	a	b
b	b	b	b	b	a	a	a	a	b
b	b	b	b	a	a	a	a	a	b
b	b	b	b	a	a	a	a	a	b
b	b	b	b	-	a	a	a	a	b
b	b	b	b	a	a	a	a	a	b
b	b	b	b	a	a	a	a	a	b
b	b	b	-	a	a	a	a	a	b
b	b	b	b	a	a	a	a	a	b
b	b	b	b	a	a	a	a	a	b
b	b	b	b	a	a	a	a	a	b
b	b	b	b	a	a	a	a	a	b
b	b	b	b	a	a	a	a	a	b
b	b	b	b	a	a	a	a	a	b
b	b	b	b	a	b	a	a	a	b
b	b	b	b	a	b	b	a	a	b
b	b	b	b	a	b	b	a	a	b
b	b	b	b	a	b	b	a	a	b
b	b	b	b	a	b	b	a	a	b
b	b	b	b	a	b	b	a	a	b
b	b	b	b	a	b	b	b	a	b
b	b	b	b	a	b	b	b	a	b

b	b	b	b	a	b	b	b	a	b
b	b	b	b	a	b	b	b	a	b
b	b	b	b	a	b	b	b	a	b
b	a	b	a	a	b	b	b	a	a
b	a	-	b	a	b	b	b	a	a
b	a	b	b	a	b	b	b	a	a
b	a	b	-	a	b	b	b	a	a
b	a	b	-	a	b	b	b	a	a
b	a	b	b	a	b	b	b	a	a
b	a	b	b	a	b	b	b	a	a
b	a	b	b	a	b	b	b	a	a
b	a	b	b	a	b	b	b	a	a
b	a	b	b	a	b	b	b	a	a
b	a	b	b	a	b	b	b	a	a
b	a	b	b	a	b	b	b	-	a
b	a	b	b	a	b	b	b	a	a
b	a	b	b	a	b	a	b	a	a
b	a	b	b	a	b	a	b	a	-
b	a	b	b	a	b	a	b	a	a
b	a	b	b	a	b	a	b	a	a
b	a	b	b	a	b	a	b	a	a
b	a	b	b	a	b	a	b	a	a
b	a	b	b	a	b	a	b	a	a
b	a	b	b	a	b	a	b	a	a
b	a	b	b	a	b	a	b	a	a
b	a	b	b	a	b	a	b	a	a
b	a	b	b	a	b	a	b	a	a
b	a	b	b	a	b	b	b	a	a
b	a	b	b	a	b	b	b	a	a
-	a	b	b	a	b	b	b	a	a
b	a	b	b	a	b	b	b	a	a
a	a	b	b	a	b	b	b	a	a
b	a	b	b	a	b	b	b	a	a
a	a	b	b	a	b	b	b	a	-
a	a	b	b	a	b	b	b	b	a
a	a	b	b	a	b	b	b	b	a
a	a	b	b	a	b	b	b	b	a
a	a	b	b	a	b	b	b	b	a
a	a	b	b	a	b	b	b	b	-
a	a	b	b	a	b	b	b	b	a
a	a	b	b	a	b	a	b	b	a
a	a	b	b	a	b	a	b	b	a
a	a	b	b	a	b	a	b	b	a
a	a	b	b	a	b	a	b	b	a
a	a	b	b	a	b	a	b	b	a
a	a	b	b	a	b	a	b	b	a
a	a	b	b	a	b	a	b	b	a

a	a	b	b	a	b	a	b	b	a
a	-	b	b	a	b	a	b	b	b
a	a	b	b	a	b	a	b	b	b
a	a	b	b	a	b	a	b	b	b
a	-	b	b	a	b	a	b	b	b
a	a	b	b	a	b	a	b	b	b
a	a	b	b	a	b	a	b	b	b
b	a	b	b	a	b	a	b	b	b
b	a	b	b	a	b	a	b	b	b
b	a	b	b	a	b	a	b	b	b
b	a	b	-	a	b	a	b	b	b
b	a	a	b	a	b	a	b	b	b
b	a	a	b	a	b	a	b	b	b
b	a	a	b	a	b	a	b	b	b
b	a	a	b	a	b	a	b	b	b
b	a	a	b	a	b	a	b	b	b
b	a	b	b	a	b	a	b	b	b
b	a	b	b	a	b	a	b	b	b
b	a	b	b	a	b	a	b	b	b
b	a	b	b	a	b	a	b	b	b
b	a	b	b	a	b	a	b	b	b
b	a	b	b	a	b	a	b	b	b
-	a	b	b	a	b	a	b	b	-
b	a	b	b	a	b	a	b	b	b
b	a	b	b	a	b	a	b	b	b
b	a	b	b	a	b	a	b	b	-
b	a	b	b	a	b	a	b	b	b
b	a	b	b	a	b	a	b	b	-
b	b	b	b	a	b	a	b	b	b
b	b	b	b	a	b	a	b	b	b
b	b	b	b	a	b	a	b	b	b
b	b	b	b	a	b	a	b	b	b
b	b	b	b	a	b	a	b	b	b
b	b	b	b	a	b	a	b	b	b
b	b	b	b	a	b	a	b	b	b
-	b	-	b	a	b	a	b	b	b
b	b	a	b	a	b	a	b	b	b
b	b	a	b	a	b	a	b	b	b
b	b	a	b	a	b	a	b	b	b
-	b	a	b	a	b	a	b	b	b
b	b	a	b	a	b	a	b	b	b
b	b	a	b	a	b	a	b	b	b
b	b	a	b	a	b	a	b	-	b
b	b	a	b	a	b	a	b	b	b
b	b	a	b	a	b	a	b	b	b
-	-	a	b	a	b	a	b	b	b
b	b	a	b	a	b	a	b	b	b

b	b	a	b	a	b	a	b	b	b
b	b	a	b	a	b	a	b	b	b
b	a	a	a	a	b	a	b	b	b
b	a	a	b	a	a	b	b	b	a
b	a	a	b	a	a	b	b	b	a
-	a	a	b	a	a	b	b	b	a
a	a	a	b	a	a	a	b	a	a
a	a	a	b	a	b	b	b	b	a
a	a	a	-	a	b	b	b	b	a
a	a	a	b	a	b	b	b	b	a
a	a	a	b	a	b	b	b	b	a
a	a	a	b	a	b	a	b	b	a
a	a	a	b	a	b	a	b	b	a
a	a	a	b	a	b	a	a	b	a
a	a	a	b	a	b	a	a	b	a
a	a	a	b	a	b	a	a	b	a
a	a	a	b	a	b	a	a	b	a
a	a	a	b	a	b	a	a	b	a
-	a	a	b	a	b	a	a	b	a
a	a	a	b	a	b	a	a	b	a
a	-	a	b	a	b	a	a	b	a
a	a	a	b	a	b	a	a	b	a
a	a	a	b	a	b	a	a	b	a
a	a	a	b	a	b	a	a	b	a
a	a	a	b	a	b	a	a	b	a
a	a	a	b	a	b	a	b	b	a
a	a	a	b	a	b	a	a	b	a
a	a	b	b	a	b	a	a	b	a
a	a	b	b	a	b	a	a	b	a
a	a	b	b	a	b	a	-	b	a
a	a	b	b	a	b	a	a	b	a
a	a	b	b	a	b	a	a	b	a
a	a	b	b	a	b	a	a	b	a
a	a	b	b	a	b	a	a	b	-
a	a	b	b	a	b	a	a	b	a
a	a	b	b	a	b	a	a	b	a
a	a	b	b	a	b	a	a	b	a
a	a	b	b	a	a	a	a	b	a
-	a	b	a	a	a	a	a	b	a
a	a	b	a	a	a	a	a	b	a
a	a	a	a	a	a	a	a	b	a
-	a	a	a	a	a	a	a	b	a
a	a	a	a	a	a	a	a	b	a
a	b	a	a	a	a	a	a	b	a
a	b	a	a	a	a	a	a	b	a
a	b	a	a	a	a	a	a	b	a
a	b	a	a	a	a	a	a	b	a

a	b	a	a	a	a	a	a	b	a
a	b	a	a	a	a	a	a	b	a
a	b	a	a	a	a	a	a	a	a
a	b	a	a	a	a	a	a	a	a
a	a	a	b	b	b	b	b	a	b
a	a	a	b	b	b	b	b	a	b
a	a	a	b	b	b	b	b	a	b
a	a	a	b	b	b	b	b	a	b
a	a	a	b	b	b	b	b	a	b
a	a	a	b	b	b	b	b	a	b
a	a	a	b	b	b	b	b	a	b
a	a	a	b	b	b	b	b	a	b
a	a	a	b	b	b	b	b	a	b
a	b	a	b	b	b	b	b	a	b
a	b	a	b	b	b	b	b	a	b
b	b	a	b	b	b	b	b	a	b
b	b	a	-	b	b	b	b	a	b
b	b	a	a	b	b	b	b	a	b
b	b	a	a	b	b	b	b	a	b
b	b	a	a	b	b	b	b	a	b
-	b	a	a	b	b	b	b	a	b
b	b	a	a	b	b	b	b	a	b
b	b	a	a	b	b	b	b	a	b
b	b	a	a	b	b	b	b	a	b
b	b	a	a	b	b	b	b	a	b
b	b	a	a	b	a	b	b	b	b
b	b	a	a	b	a	b	a	b	b
b	b	a	a	b	a	b	a	b	b
b	b	a	a	b	a	b	a	b	b
a	b	a	a	b	a	b	a	b	b
a	b	a	-	b	a	b	a	b	b
a	b	a	a	b	a	b	a	b	b
a	b	a	a	a	a	b	a	a	b
a	b	a	a	a	a	b	a	a	b
a	b	a	a	a	a	b	a	a	b
a	b	a	a	a	a	b	a	a	b
a	b	a	a	a	a	b	a	a	b
-	b	a	a	a	a	b	a	a	b
a	b	a	a	a	a	b	a	a	b
a	b	a	a	a	a	b	a	a	b
a	b	a	a	a	a	b	a	a	b
a	b	a	a	a	a	b	b	a	b
a	b	a	a	a	a	b	b	a	b
a	b	a	a	a	a	b	b	a	a
a	b	a	a	a	a	b	b	a	b
a	b	b	a	a	b	a	b	a	a
a	b	b	a	a	b	a	b	a	a

a	b	b	a	a	b	a	b	a	a
a	-	b	a	a	b	a	b	a	a
a	b	b	a	a	-	a	b	a	a
a	b	a	a	a	b	a	b	a	a
a	b	-	a	a	b	a	b	a	a
a	b	b	a	a	b	a	b	a	a
a	b	b	a	a	b	a	b	a	a
a	b	b	a	a	b	a	b	a	a
a	-	b	a	a	b	a	b	a	a
a	b	b	a	a	b	a	b	a	a
-	b	b	a	a	b	a	b	a	a
a	b	b	a	a	b	a	b	a	a
a	b	b	a	a	b	a	b	a	a
a	b	b	a	a	b	a	b	a	a
a	b	b	a	a	b	a	b	a	a
a	b	b	a	a	b	a	b	a	a
a	b	b	a	a	b	a	b	a	a
-	b	b	a	a	b	a	b	a	a
a	b	b	a	a	b	a	b	a	a
a	-	b	a	a	b	a	b	a	a
a	b	b	a	a	b	a	b	a	a
a	b	b	-	a	b	a	b	a	a
a	-	a	a	a	b	a	b	a	a
a	b	a	a	a	b	a	b	a	a
-	b	a	a	a	b	a	b	a	b
a	b	a	a	a	b	a	b	a	b
a	b	a	a	a	b	a	b	a	b
a	b	a	a	a	b	a	b	a	b
a	b	a	a	a	b	a	b	a	-
a	b	a	a	a	b	a	b	a	b
a	b	a	a	a	b	a	b	a	b
a	b	a	a	a	b	a	b	a	b
-	b	-	a	a	b	a	b	a	b
a	a	a	a	a	b	a	b	a	b
a	a	a	a	a	b	a	b	a	b
a	a	a	a	a	b	a	b	a	b
a	a	a	a	a	b	a	b	a	b
a	a	a	a	a	b	a	b	a	b
-	a	a	a	a	b	a	b	a	b
a	a	a	a	a	b	a	b	a	-
a	a	-	a	a	b	a	b	a	b
a	a	a	a	a	a	a	b	a	b
a	a	a	a	a	a	a	b	a	b
a	a	a	a	a	a	a	b	a	b
a	a	a	a	a	a	a	b	a	b
a	a	a	a	a	a	a	b	a	b
a	a	a	a	a	a	a	b	a	b

a	a	a	a	a	a	a	b	a	b
a	a	a	a	a	a	a	b	a	b
a	a	a	a	b	a	a	b	a	b
a	a	a	a	b	a	a	b	a	-
a	a	a	a	b	a	a	b	a	b
a	a	a	a	b	a	a	b	a	b
a	a	-	a	b	a	a	b	a	b
a	a	a	a	b	a	a	b	a	b
a	a	a	a	b	a	a	b	a	b
a	a	a	a	b	a	a	b	a	b
a	a	a	a	b	a	a	b	a	b
a	a	a	a	b	a	a	b	a	b
a	a	a	a	b	a	a	b	a	b
a	a	a	a	b	a	a	b	a	b
a	a	a	a	b	a	a	b	a	b
a	a	b	a	a	a	a	b	a	b
a	a	b	a	a	a	a	b	a	b
a	a	b	a	a	a	a	b	a	b
b	a	b	a	a	a	a	b	a	b
b	a	b	a	a	a	a	b	a	b
b	a	b	a	a	a	a	a	a	b
b	a	b	a	b	a	a	a	a	b
b	a	b	a	b	a	a	a	a	b
-	a	b	a	b	a	a	a	a	b
b	a	b	a	b	a	a	a	a	b
b	a	b	a	b	a	a	a	a	b
b	a	b	a	b	a	a	a	a	b
b	a	b	a	b	a	a	a	a	b
b	a	b	a	b	a	a	a	a	-
b	a	b	a	b	a	a	a	a	b
b	a	b	a	b	a	a	a	a	b
b	a	b	a	b	a	a	a	a	b
b	a	b	a	b	a	a	a	a	b
b	a	b	a	b	a	a	a	a	b
b	a	b	a	b	a	a	a	-	b
b	a	b	a	b	b	b	a	b	b
b	a	b	a	b	b	b	a	b	b
b	a	b	a	b	b	b	a	b	b
b	a	b	a	b	b	b	a	b	b
b	a	b	a	b	b	b	a	b	b
b	a	b	b	b	b	b	a	b	b
b	a	b	b	b	b	b	a	b	b
b	a	-	b	b	b	b	a	b	b
b	a	b	b	b	b	b	a	b	b
b	a	b	b	b	a	b	a	b	b
b	a	b	b	b	b	b	a	b	b
b	a	b	b	b	a	b	a	a	b

b	a	b	b	b	a	b	a	a	b
b	a	-	b	b	a	b	a	a	b
b	a	a	b	b	a	b	a	a	b
b	a	a	b	b	a	b	a	a	b
b	a	a	b	b	a	b	a	a	b
b	a	a	b	b	a	b	a	a	b
b	a	a	b	b	a	b	a	a	b
b	a	a	b	b	a	b	a	a	b
a	a	-	a	a	b	b	a	b	b
a	a	a	a	a	b	b	a	b	b
a	a	a	a	a	b	b	a	b	b
a	a	a	a	a	a	b	a	b	b
-	a	a	a	a	a	b	a	b	b
a	a	a	a	a	a	b	a	b	b
a	a	a	a	a	a	b	a	b	b
a	a	a	a	a	a	b	a	b	b
a	a	a	a	a	a	b	a	b	b
a	a	a	a	a	a	b	a	b	b
-	a	a	a	a	a	b	a	b	b
b	a	a	b	a	a	b	a	b	b
b	a	-	b	a	a	b	a	b	b
b	a	a	b	a	a	b	a	b	b
b	a	-	b	a	a	b	a	b	b
b	a	a	b	a	a	b	b	a	b
b	a	a	b	a	a	b	b	a	b
b	a	a	b	a	a	b	b	a	b
b	a	a	-	a	a	b	b	a	b
b	b	a	b	a	a	b	b	b	b
b	b	a	b	a	a	b	b	b	b
b	b	a	b	a	a	a	b	b	b
b	b	a	b	a	a	a	b	b	b
b	b	a	b	a	a	a	b	a	b
b	b	a	b	a	a	a	b	a	b
b	b	a	b	a	a	a	b	a	b
b	b	a	b	a	a	a	b	a	b
b	-	a	b	a	a	a	b	b	b
b	b	a	b	a	a	a	b	b	b
b	b	a	b	a	a	a	b	b	b
-	b	a	b	a	a	a	b	b	b
b	b	a	b	a	a	a	b	b	-
b	b	a	b	a	a	a	b	b	b
-	b	a	b	a	b	a	b	b	b
b	b	a	b	a	b	a	b	b	b
b	a	a	a	a	-	a	b	b	b
b	a	a	a	a	b	a	b	b	b
b	a	a	a	a	b	a	b	b	b
b	a	a	b	a	b	a	b	b	b

b	a	a	a	a	b	a	b	b	b
b	a	a	a	a	b	a	b	b	b
b	a	a	a	a	b	a	b	b	b
b	a	a	a	a	b	a	b	b	b
b	a	a	a	a	b	a	b	b	b
b	a	a	a	b	b	a	b	a	b
b	a	a	b	b	b	a	a	a	b
b	a	a	b	b	b	a	a	a	b
b	a	a	b	b	b	a	b	a	b
b	a	a	b	b	b	a	b	a	b
b	a	a	b	b	b	a	b	a	-
b	a	a	b	b	b	a	b	a	b
b	a	b	b	b	a	b	b	b	b
b	a	b	b	b	a	b	b	b	b
b	a	b	b	b	a	b	b	b	a
a	a	b	b	b	a	b	b	b	a
a	a	b	b	b	a	b	b	b	a
a	a	b	b	b	a	b	b	b	a
a	a	b	b	b	b	b	b	b	a
a	a	b	b	b	b	b	b	b	a
a	a	b	b	b	b	b	b	b	a
-	a	b	b	b	b	b	b	b	a
a	a	b	b	b	b	b	b	b	a
a	a	b	b	b	b	b	a	b	a
a	a	b	b	b	b	b	a	b	a
a	a	b	b	b	b	b	a	b	a
a	a	b	b	b	b	b	a	b	a
a	a	b	b	b	b	a	a	b	a
a	a	b	b	b	b	a	a	b	a
b	a	b	b	b	b	a	a	a	a
a	a	b	b	b	b	a	a	a	a
a	a	b	b	b	b	a	a	a	a
-	a	b	b	b	b	a	a	a	a
a	-	b	b	b	b	a	a	a	a
a	a	b	b	b	b	a	a	a	a
-	a	b	b	b	a	a	a	a	-
a	a	b	b	b	a	a	a	a	a
a	a	b	b	b	a	a	a	a	a
a	a	b	b	b	a	a	a	a	a
a	a	b	b	b	a	a	a	a	a
a	a	b	b	b	a	b	a	a	a
a	a	b	b	b	a	b	a	a	a
a	a	b	b	b	a	b	a	a	a
a	a	b	b	b	a	b	a	a	a
a	a	b	b	b	a	b	a	a	a
a	a	b	a	b	a	b	a	a	a
a	a	b	a	b	a	b	a	a	a

a	a	b	a	b	a	b	a	a	a
a	a	b	a	b	a	b	a	a	-
a	a	b	a	b	a	b	a	a	a
a	a	b	a	b	a	b	a	a	a
a	a	b	a	b	a	b	a	a	a
-	a	b	a	b	a	b	a	a	a
a	a	b	a	b	b	b	a	a	a
a	a	b	a	b	b	b	a	a	a
b	-	a	a	a	a	b	b	a	a
b	b	a	a	a	a	b	b	a	a
b	b	a	a	a	a	b	b	a	a
b	b	a	a	a	a	b	b	a	a
b	b	a	a	a	a	b	b	a	a
b	b	a	a	a	a	b	b	a	a
b	b	a	a	a	a	b	b	a	a
b	b	a	a	a	b	b	b	a	a
b	b	a	a	a	b	b	b	a	a
a	b	a	a	a	b	b	b	a	a
a	b	a	a	a	b	b	b	a	a
a	b	a	a	a	b	a	b	a	a
a	b	a	a	a	b	a	b	a	a
a	b	a	a	a	b	a	b	a	a
a	b	a	a	a	-	a	b	a	a
a	b	a	a	a	b	a	b	a	a
a	b	a	a	a	b	a	b	a	a
-	b	a	a	a	b	a	b	a	a
a	b	a	a	a	b	a	b	a	a
a	b	a	a	a	b	a	b	a	a
a	b	a	a	a	b	a	b	a	a
a	b	a	a	b	b	a	b	a	a
a	b	a	a	b	b	a	b	a	a
a	b	a	a	b	b	a	b	a	a
a	b	a	a	b	b	a	b	a	a
a	b	a	a	b	b	a	b	a	a
a	b	a	a	b	b	a	b	a	a
a	b	a	a	b	b	a	b	a	a
a	b	a	a	b	b	a	b	a	a
a	b	a	a	b	b	a	b	a	a
a	b	a	a	b	b	a	b	a	a
a	b	a	a	b	b	a	b	a	a
a	b	-	a	b	b	a	b	a	a
a	-	a	a	b	b	a	b	a	a
a	b	a	a	b	b	a	b	b	a
a	b	a	a	b	b	a	b	b	a
a	b	a	a	b	b	a	b	b	a
a	b	a	a	b	b	a	b	b	a
a	-	a	a	b	-	a	b	b	a

a	b	a	a	b	b	a	b	b	a
a	b	a	a	b	b	a	b	b	a
a	b	a	a	b	b	a	-	b	a
a	b	a	a	b	b	a	b	b	a
a	b	a	a	b	b	a	b	b	a
a	b	a	a	b	b	a	b	b	a
a	b	a	a	b	a	b	b	b	a
a	b	a	a	b	a	b	b	b	a
a	b	a	a	b	a	b	b	b	a
a	b	a	a	b	a	b	b	b	a
b	b	a	a	b	-	b	b	b	a
a	b	a	a	b	a	b	b	b	a
a	b	a	a	b	a	b	b	b	a
a	b	a	a	b	a	b	b	b	a
a	b	a	a	b	a	b	b	a	a
a	b	a	a	b	a	b	b	a	a
a	b	a	a	b	a	b	b	a	a
a	b	a	a	b	a	b	b	a	a
a	b	a	a	b	a	b	a	a	a
a	b	a	a	b	a	b	a	a	a
-	a	b	a	b	a	b	a	b	b
-	a	b	a	b	a	b	a	b	b
a	a	b	a	b	a	b	a	b	b
a	a	b	a	b	a	b	a	b	b
a	a	b	a	b	a	b	a	b	b
-	a	b	a	b	a	b	a	b	b
a	a	b	a	b	a	b	a	b	b
a	a	b	a	b	a	b	a	b	b
a	a	-	a	b	a	b	a	b	b
a	a	a	a	b	a	b	a	b	b
a	a	a	a	b	a	b	a	b	b
a	a	b	a	b	a	b	a	b	b
a	a	a	a	b	a	b	a	b	b
a	a	a	a	b	a	b	a	b	b
a	a	-	a	b	a	b	a	b	b
a	a	a	a	b	a	b	a	b	b
a	a	a	a	b	a	b	a	b	b
b	a	a	a	b	a	b	a	b	b
b	a	a	a	b	a	b	a	b	b
b	a	a	a	b	a	b	a	b	b
b	a	a	a	b	a	a	a	b	a
b	a	a	a	b	a	a	a	b	a
b	a	a	a	b	a	a	a	b	a
b	b	a	a	-	a	a	a	b	a
b	b	a	a	b	a	a	a	b	a
b	-	a	a	b	a	a	a	b	a
b	b	a	a	b	a	a	a	b	a

b	b	a	a	b	a	a	a	b	a
b	b	a	a	b	a	a	a	b	a
b	-	a	a	b	a	a	a	b	a
b	b	a	a	b	b	a	a	b	a
b	b	a	a	b	b	a	a	b	a
b	b	a	a	b	b	a	a	b	a
b	b	a	a	b	b	a	a	b	a
b	b	a	a	b	b	a	a	b	a
b	b	a	a	b	b	a	a	b	a
b	b	a	a	b	b	a	a	a	a
b	b	a	-	b	b	a	a	a	a
b	b	a	a	-	b	a	a	a	a
b	b	a	a	b	b	a	a	a	a
b	b	a	-	b	b	a	a	a	a
b	b	a	a	b	b	a	a	a	a
b	b	a	a	b	b	a	a	a	a
b	b	-	a	b	b	a	a	a	a
b	b	a	a	-	b	a	a	a	a
-	b	a	a	b	b	a	a	a	a
b	b	a	b	b	b	a	a	a	a
b	b	a	b	b	b	a	a	a	a
b	b	a	b	b	b	a	a	a	a
b	b	a	-	b	b	a	a	a	a
b	b	a	b	b	b	a	a	b	a
b	b	a	b	b	b	a	a	b	a
-	a	a	b	b	b	a	a	b	a
b	a	a	b	b	b	a	a	b	a
b	a	a	b	b	b	a	a	b	a
b	a	a	b	b	b	a	a	b	a
-	a	a	b	b	b	a	a	b	a
b	a	a	b	b	b	a	a	b	a
b	a	a	b	b	b	a	a	b	a
b	a	a	b	b	b	a	a	b	a
b	a	a	b	b	b	a	a	b	a
b	a	a	-	b	b	a	a	b	b
a	a	a	b	a	a	a	a	b	b
a	a	a	b	a	a	a	a	b	b
a	a	a	b	a	a	a	a	b	b
a	a	a	b	a	a	a	a	b	b
a	a	a	b	a	a	a	a	b	b
a	a	a	b	a	a	a	a	b	b
a	a	a	b	a	b	a	a	b	b
a	a	a	b	a	b	a	a	b	b
a	a	a	b	a	b	a	a	b	b
a	-	b	b	a	b	a	a	a	b
a	b	b	b	a	b	a	a	a	b
a	b	b	b	a	-	b	a	a	b

a	-	b	b	a	b	b	a	a	a
a	b	b	b	a	b	b	a	a	b
a	b	b	b	a	b	b	a	a	b
a	b	b	b	a	b	b	a	a	b
a	b	b	b	a	b	b	a	a	b
a	b	b	b	a	b	b	a	a	b
a	b	b	b	a	b	b	a	a	b
a	b	b	b	a	b	b	a	a	b
a	b	b	b	a	b	b	a	a	b
a	b	b	b	a	b	b	a	a	b
a	b	b	b	a	b	b	a	a	b
a	b	b	b	a	b	b	a	a	b
a	b	b	b	a	b	b	a	a	b
a	b	b	b	a	b	b	a	a	b
a	b	b	b	a	b	b	a	a	b
a	b	b	b	a	b	b	a	a	b
a	b	b	b	a	b	b	a	b	b
a	b	b	b	a	b	b	a	b	b
a	b	b	b	a	b	b	a	b	b
a	b	-	-	a	b	b	a	a	b
a	b	-	b	a	b	b	a	a	b
a	b	b	b	a	b	b	a	a	b
a	b	-	b	a	a	b	a	a	b
a	b	b	b	a	a	b	a	a	b
a	b	b	b	a	a	b	a	a	b
a	b	b	b	a	a	b	a	a	b
a	b	b	b	a	a	b	a	a	b
a	b	b	-	a	a	b	a	a	b
a	b	a	b	a	a	b	a	a	b
a	b	a	b	a	a	b	a	a	b
a	b	a	b	a	a	b	a	a	b
a	b	a	b	a	a	b	a	a	b
a	b	a	b	a	a	b	a	a	b
a	b	a	b	a	a	b	a	a	b
a	b	a	b	b	a	b	a	a	b
a	b	a	b	b	a	b	a	a	b
a	b	a	b	b	a	b	a	a	b
a	b	b	b	b	a	b	a	a	b
a	b	a	b	b	a	b	a	a	b
a	b	a	b	b	a	b	a	b	b
a	b	a	b	b	a	b	a	b	b
a	b	a	b	b	a	b	a	b	b
a	b	-	b	b	a	b	a	b	b
a	b	a	b	b	a	b	a	b	b
a	b	a	b	b	a	b	a	b	a
a	b	a	b	b	a	b	a	b	a
b	a	b	a	b	b	b	b	a	a

b	a	b	a	b	b	b	b	a	a
b	a	b	a	b	b	b	b	a	a
b	a	b	a	b	b	b	b	a	a
b	a	b	a	b	b	b	b	a	a
b	a	b	a	b	b	b	b	a	a
b	a	b	a	b	b	b	b	a	a
b	a	b	b	b	b	b	a	a	a
b	a	b	b	b	b	b	a	a	a
b	a	b	b	b	b	b	a	a	a
b	a	b	b	b	b	b	a	a	a
b	a	b	b	b	b	b	a	a	a
b	a	b	b	b	b	b	a	a	a
b	a	b	b	b	b	b	a	a	a
b	a	b	b	b	b	b	a	a	a
b	a	b	b	b	b	b	a	a	a
b	a	b	b	b	b	b	a	a	a
b	a	b	b	b	b	b	a	a	a
b	a	a	a	a	b	b	a	a	a
b	a	b	b	b	b	b	a	a	a
b	a	b	b	b	b	b	a	a	a
b	a	b	b	b	b	b	a	a	a
b	a	b	b	b	b	b	a	a	a
b	a	b	b	b	b	b	a	a	a
b	a	b	b	b	b	b	a	a	a
b	a	b	b	b	b	b	a	a	a
b	a	b	b	b	b	b	a	a	a
b	a	b	b	b	b	b	a	a	a
b	a	b	b	b	b	b	a	a	a
b	a	b	b	b	b	b	a	a	a
b	a	b	b	b	b	b	a	a	a
b	a	b	b	b	b	b	a	a	a
b	a	b	b	b	b	b	a	a	a
b	a	b	b	b	b	b	a	a	a
b	a	b	b	b	b	b	a	a	a
b	a	b	b	b	b	a	a	a	a
b	a	b	b	b	b	a	a	a	a
b	a	b	b	b	b	a	a	a	a
b	a	b	b	b	b	a	a	a	a
b	a	b	b	b	b	b	a	a	a
b	a	b	b	a	b	a	a	a	a
-	b	a	a	a	b	b	b	b	b
b	a	b	b	b	a	a	a	a	a
b	a	b	b	b	a	a	a	a	a
b	a	b	b	b	a	a	a	a	a
b	a	b	b	b	a	a	a	a	a
b	a	b	b	b	a	a	a	a	a
b	a	b	b	b	a	a	a	a	a

b	a	b	b	b	a	a	a	a	a
b	a	b	b	b	a	a	a	a	a
b	a	b	b	b	a	a	a	a	a
b	a	b	b	b	a	a	a	a	a
b	a	b	b	b	a	a	a	a	a

YW049	YW050	YW051	YW052	YW053	YW054	YW055	YW056	YW057	YW058
b	b	-	b	a	b	a	b	b	b
b	b	b	b	a	b	a	b	b	b
b	b	b	b	a	b	a	b	b	b
b	b	b	b	a	b	a	b	b	-
b	b	b	b	a	b	a	b	b	-
b	a	b	-	a	b	a	b	b	b
b	-	b	b	a	b	a	b	b	b
b	b	b	b	a	b	a	b	b	b
b	b	b	b	a	b	a	b	b	b
b	-	b	b	a	b	a	b	b	b
b	a	-	a	a	b	a	b	b	b
b	b	b	b	a	b	a	b	b	b
b	-	b	b	a	b	a	b	b	b
b	b	b	b	a	b	a	b	b	b
b	b	b	b	a	b	a	b	b	b
b	b	-	b	a	b	a	b	b	b
b	b	b	b	a	b	a	b	b	b
b	b	b	b	a	b	a	b	b	b
b	b	b	b	a	b	a	b	b	b
b	b	b	b	a	b	a	b	b	b
-	b	b	b	a	b	a	b	b	b
b	b	b	b	a	-	a	b	b	b
b	b	b	b	a	b	a	b	b	b
b	b	b	b	a	b	a	b	b	b
b	b	b	b	a	b	a	b	b	b
b	-	b	b	a	b	a	b	b	b
b	b	b	b	a	b	a	b	b	b
b	b	b	b	a	b	a	b	b	b
b	a	-	b	a	b	a	a	b	b
b	a	b	b	a	b	a	a	b	b
-	a	b	b	a	b	a	a	-	b
b	a	b	b	a	b	a	a	b	b
b	a	b	b	a	b	a	a	b	b
b	a	b	b	a	-	a	a	b	b
b	a	b	b	a	b	b	a	b	b
b	-	b	b	a	b	b	a	b	b
b	a	b	b	a	b	-	a	b	b
b	-	b	b	a	b	b	a	b	b
b	a	b	b	a	b	b	a	b	b
b	a	b	b	-	b	b	a	b	b
b	a	b	b	a	b	b	a	b	b
b	a	b	b	a	b	b	a	b	a
b	a	b	b	a	b	b	a	b	a
b	a	b	b	a	b	b	a	b	-
b	a	b	b	a	b	b	a	b	a

a	a	b	b	a	b	b	a	b	-
a	a	b	a	a	b	b	a	b	a
a	a	b	b	a	b	b	a	b	a
a	a	b	b	a	b	b	a	b	a
a	a	b	b	a	b	b	a	b	-
a	a	b	b	a	b	b	a	b	a
a	a	b	b	a	b	b	a	b	-
a	-	b	b	a	b	b	a	b	a
a	a	b	-	a	b	b	a	b	a
a	a	b	-	a	b	b	a	b	a
a	a	b	b	a	b	b	a	b	a
a	a	b	b	-	b	b	a	b	a
a	a	b	b	a	b	-	a	b	a
a	a	b	b	a	a	b	a	b	a
b	a	b	b	a	b	b	a	b	a
b	a	b	b	a	b	b	a	b	a
b	a	b	b	a	b	b	a	b	a
b	a	-	a	a	b	b	a	b	a
b	a	b	b	a	b	b	a	b	a
b	a	b	b	a	b	b	a	b	a
b	a	b	b	a	b	b	a	b	a
b	a	b	b	a	b	b	a	b	a
b	a	b	b	a	b	-	a	b	a
b	a	b	-	a	b	b	a	b	a
b	-	b	b	a	b	b	a	b	a
b	-	b	b	a	b	b	a	b	a
-	a	b	b	a	b	b	b	b	a
b	a	b	b	a	b	b	b	b	a
b	b	-	b	a	b	b	b	b	a
b	a	a	b	a	b	b	b	b	a
-	a	a	b	a	b	-	b	b	a
b	b	a	b	a	b	b	b	b	a
b	a	a	b	a	b	b	b	b	a
b	a	a	b	a	b	b	b	b	a
b	a	a	b	a	b	b	b	b	a
b	a	a	b	a	b	b	b	b	a
b	a	a	b	a	b	b	b	b	a
b	a	a	-	a	b	b	b	b	a
b	a	a	b	a	b	a	b	b	a
b	a	a	b	b	a	a	a	a	a
b	a	a	b	b	a	a	a	a	a
b	a	a	b	b	a	a	a	a	a
b	a	a	b	b	a	a	a	a	a
b	a	a	b	b	a	a	a	a	a
b	a	a	b	b	a	a	a	a	a
b	a	a	b	b	a	a	a	a	a
b	a	a	b	b	a	a	a	a	a

b	-	-	a	b	a	a	a	a	a
b	-	a	-	b	a	a	a	a	a
b	a	a	a	b	a	a	a	a	a
-	a	a	a	b	a	a	a	a	a
b	a	a	a	b	a	a	a	a	a
b	a	a	a	b	a	a	a	a	a
b	a	a	a	b	a	a	a	a	a
b	a	a	a	b	a	a	a	a	a
b	a	a	a	b	a	a	a	a	a
b	a	a	a	b	a	a	a	a	a
b	a	a	a	b	a	a	a	a	a
b	a	-	a	b	a	a	a	a	a
b	-	a	-	b	a	a	b	a	a
b	-	a	a	b	a	b	b	a	a
b	b	a	a	b	a	b	b	a	b
b	b	a	-	b	a	b	b	a	a
b	b	-	-	b	a	b	b	a	a
b	b	a	-	b	a	b	b	b	a
b	b	a	a	b	a	b	b	b	a
b	-	a	a	b	a	b	b	b	a
b	b	a	a	b	a	b	b	b	-
-	b	a	a	b	a	b	b	b	a
b	b	a	b	b	a	b	b	b	a
b	b	a	a	b	a	b	b	b	a
b	b	a	b	b	a	a	b	b	a
b	b	a	a	b	a	a	b	b	a
b	b	a	a	b	a	a	b	b	a
b	b	a	a	b	a	a	b	b	a
b	b	-	b	b	a	a	b	b	a
b	b	a	a	b	a	a	b	-	a
b	a	a	a	b	a	a	b	b	a
b	b	a	a	b	a	a	b	b	a
b	b	a	a	b	a	a	b	b	a
b	b	a	a	b	a	a	b	b	a
b	b	a	a	b	a	a	b	b	a
b	b	a	a	b	a	a	b	b	a
b	b	a	a	b	a	a	b	b	a
b	b	a	a	b	a	a	b	b	a
b	b	a	-	b	a	a	b	b	a
b	b	a	a	b	a	a	b	b	a
b	b	a	a	b	a	a	b	b	a
a	a	a	a	b	a	a	b	b	a
b	b	a	a	b	a	a	b	b	a
b	b	a	a	b	a	a	b	b	a
b	b	a	a	-	a	a	a	b	a
b	b	a	a	-	a	a	a	b	a
b	b	a	a	b	a	a	a	b	a

b	b	a	a	b	a	a	a	b	a
b	b	a	a	b	a	a	a	b	a
b	b	a	a	-	a	a	a	b	a
b	b	a	a	b	a	a	a	b	a
b	b	a	a	b	a	a	a	b	a
b	b	a	a	b	a	a	a	b	a
b	a	a	a	b	a	a	a	b	a
b	b	a	a	b	a	a	a	b	a
b	b	a	a	b	a	-	a	b	a
b	b	a	a	b	a	a	a	b	a
b	b	a	a	b	a	a	a	b	a
b	b	a	a	b	b	a	a	b	-
b	b	a	a	b	b	a	a	b	a
a	b	a	a	b	b	a	a	b	a
a	b	a	a	b	b	a	a	b	a
a	b	a	a	b	b	a	a	b	a
a	b	a	a	b	b	a	a	b	a
a	b	a	a	b	b	a	a	b	a
a	b	a	a	b	b	a	a	b	-
a	b	a	a	b	b	b	a	b	a
a	a	a	a	b	b	b	a	b	a
a	b	a	a	b	b	b	a	b	a
a	b	a	a	b	b	b	a	b	a
a	b	a	a	-	b	b	a	b	a
a	b	a	a	b	b	b	a	b	a
a	-	a	b	b	b	b	a	b	a
a	b	b	b	b	b	b	a	b	a
a	b	b	b	b	b	b	a	b	a
a	a	b	b	-	b	b	a	b	a
a	b	b	b	b	b	b	a	b	a
a	b	b	b	b	b	b	a	b	a
a	b	b	b	b	b	b	a	b	a
a	b	b	b	b	b	b	a	b	a
a	b	b	b	-	b	b	a	b	a
a	b	b	b	b	b	b	a	b	a
a	b	b	b	b	b	b	b	b	a
a	b	b	b	b	b	b	b	b	a
-	b	b	b	b	b	b	b	b	a
a	b	b	b	b	b	b	b	b	a
a	b	b	-	b	b	b	b	b	a
a	b	b	b	b	b	b	b	b	a
a	a	b	b	-	b	b	b	b	a
a	b	-	b	b	b	b	b	b	a
a	b	b	b	b	b	b	b	b	a
a	b	b	b	b	b	-	b	b	a
a	b	a	b	b	a	b	b	b	b
a	b	a	b	b	a	b	b	b	b

a	b	a	b	b	a	b	b	b	b
a	b	a	b	b	a	b	b	b	b
a	b	a	b	b	a	b	b	b	b
a	b	a	b	b	a	b	b	b	b
a	b	a	b	b	a	b	b	b	b
a	b	a	-	b	a	b	b	b	b
a	a	a	-	b	a	b	b	b	b
a	b	a	b	b	a	b	b	a	b
a	a	a	b	b	a	b	b	a	b
a	b	a	b	b	a	b	b	a	b
a	a	a	b	b	a	b	b	a	b
a	b	a	b	b	a	b	b	a	b
b	a	a	b	b	b	b	a	a	-
b	a	a	b	b	b	b	a	a	a
b	a	a	b	b	b	b	a	a	a
b	a	a	b	b	b	b	a	a	a
b	a	a	b	b	b	b	a	a	a
b	a	a	a	b	b	b	a	a	a
b	a	a	a	b	b	b	a	a	b
b	a	a	a	b	b	b	a	a	b
b	-	a	a	b	b	b	a	a	b
b	a	a	-	b	b	b	a	a	b
b	-	a	-	b	b	b	a	a	b
b	a	a	a	b	b	b	a	a	b
b	-	a	a	b	b	b	a	a	b
b	a	a	a	b	b	b	a	a	b
-	a	a	a	b	b	b	a	a	-
b	a	a	a	b	b	b	a	a	b
b	a	a	-	b	b	b	a	a	b
b	-	a	a	b	b	b	a	a	b
b	a	a	a	b	b	b	a	a	b
b	a	a	-	b	b	b	b	a	b
b	a	b	a	b	b	b	b	a	b
b	-	a	a	b	b	b	b	a	b
b	a	a	a	b	b	b	b	a	b
b	a	a	a	b	b	b	b	a	b
b	a	a	a	b	b	b	b	a	b
b	-	a	a	b	b	b	b	a	b
b	-	a	a	b	b	b	b	a	b
b	a	a	a	b	b	b	b	-	b
b	a	a	a	b	b	b	b	a	b
b	a	a	a	b	b	b	b	a	b
b	a	b	a	b	b	b	b	a	b
b	a	b	a	b	b	b	b	a	b
b	a	b	a	-	b	b	b	a	b
-	a	b	a	b	b	b	b	a	b
b	a	b	a	b	b	b	b	a	b

b	-	b	a	b	b	b	b	a	b
b	a	b	a	b	b	b	b	a	b
-	a	b	a	b	b	b	b	a	b
b	-	b	a	b	b	b	b	a	b
b	a	b	a	b	b	b	a	a	b
b	a	-	a	-	b	b	a	a	b
b	a	a	a	b	b	b	a	a	b
b	a	a	a	b	b	b	a	a	b
b	a	a	a	b	b	b	a	a	b
b	a	a	a	b	b	a	a	a	b
b	a	a	a	b	b	a	a	a	b
b	a	a	a	b	b	a	a	a	b
b	a	a	a	b	b	a	a	a	b
b	-	a	-	b	b	a	a	a	b
b	a	a	a	b	b	a	a	a	b
b	a	a	a	b	a	a	a	a	b
b	a	a	a	b	a	a	a	a	b
b	-	a	a	b	a	a	a	a	b
b	a	a	a	b	a	a	a	a	b
b	a	a	a	b	a	a	a	a	b
b	a	a	a	b	a	a	a	a	b
b	a	a	a	b	a	a	a	a	b
b	a	a	a	b	a	a	b	a	b
b	-	a	a	b	a	a	b	a	b
b	a	a	a	b	a	b	b	a	b
b	a	a	a	b	a	b	b	a	b
b	a	a	a	b	a	b	b	a	b
b	b	a	a	b	a	b	b	a	b
b	-	a	a	b	a	b	b	a	b
b	b	a	a	b	a	b	b	a	b
b	b	a	a	b	a	b	b	a	b
b	b	a	a	b	a	b	b	a	b
b	b	a	a	b	a	b	b	a	b
b	b	a	a	b	a	b	b	a	b
b	b	a	a	b	a	b	b	a	b
b	a	a	a	b	a	b	b	a	b
b	a	a	a	b	a	b	b	a	b
b	a	a	a	b	a	b	b	a	b
b	a	a	a	a	a	b	b	a	-
b	a	a	a	-	a	b	b	a	b
b	a	a	a	a	a	b	b	a	b
b	a	a	-	a	a	b	b	a	b
b	a	a	a	a	a	b	b	a	b
b	a	a	a	a	a	b	b	a	b
b	a	a	a	a	a	b	b	a	b
b	a	a	a	a	a	b	b	a	b
b	a	a	a	a	a	b	b	a	b

b	a	a	a	a	a	b	b	a	b
b	a	a	-	a	a	b	b	a	b
b	a	b	a	a	a	b	b	a	b
b	a	b	a	a	a	b	b	a	b
b	a	b	a	a	a	b	b	a	b
b	a	b	a	a	a	b	b	a	b
b	a	b	a	a	a	b	b	a	b
b	-	b	-	-	a	b	b	a	b
b	a	b	a	a	a	b	b	a	b
b	a	b	a	a	a	b	b	a	b
-	a	b	a	a	a	b	b	a	b
b	a	b	a	a	a	b	b	a	b
b	a	b	a	-	a	b	b	a	b
a	a	b	a	a	a	b	b	a	b
a	a	b	a	a	a	b	b	a	b
a	a	b	a	a	a	b	b	a	b
a	a	b	a	a	a	b	b	a	b
a	a	b	a	a	a	b	b	a	b
a	a	b	a	a	a	b	b	a	b
a	a	b	a	a	a	b	b	a	b
a	a	b	a	a	a	b	b	a	b
a	a	b	a	-	a	b	b	a	b
a	a	b	-	a	a	b	b	a	b
-	-	b	a	a	a	b	b	a	b
-	a	b	-	a	a	b	b	a	b
a	a	b	a	a	a	a	b	a	b
a	a	b	a	a	a	b	b	a	b
a	a	b	a	a	a	b	b	a	b
a	a	b	a	a	a	b	b	a	b
a	a	b	a	a	a	b	b	a	b
a	a	b	a	a	a	b	b	a	b
a	a	-	a	a	a	b	b	a	b
a	a	-	a	a	a	b	b	a	b
a	a	b	a	a	a	b	b	a	b
a	a	-	a	a	a	b	b	a	b
b	a	b	a	a	a	b	b	a	b
b	-	b	a	a	a	b	b	a	b
b	a	b	a	a	a	b	b	a	b
b	a	b	a	a	b	b	b	a	b
b	a	b	a	a	b	b	b	a	b
b	a	-	a	a	b	b	b	a	b
b	a	b	a	a	b	b	b	a	b
b	a	b	a	a	b	b	b	a	b
b	a	b	b	a	b	b	b	a	b
-	a	-	a	a	b	b	b	a	b
b	a	b	a	a	b	b	b	a	b
b	a	b	a	a	b	b	b	a	b

b	a	b	a	a	b	b	b	a	b
b	a	b	a	a	b	b	b	a	b
b	a	b	a	a	b	b	b	a	b
b	a	b	a	a	b	b	b	a	b
b	a	b	a	a	b	b	b	a	b
b	-	b	a	a	b	b	b	-	b
b	a	b	a	a	b	b	b	a	b
b	a	b	a	a	b	b	b	a	b
b	a	b	a	a	b	b	b	a	b
b	a	-	a	a	b	-	b	a	b
b	a	b	a	a	b	b	b	a	b
b	a	b	a	a	b	a	b	a	b
b	-	b	a	a	b	a	b	a	b
b	a	b	a	a	b	a	b	a	b
b	a	b	a	a	b	a	b	a	b
b	a	b	a	a	b	a	b	a	b
b	a	b	b	a	b	a	b	a	b
b	a	b	a	a	b	a	b	a	b
b	a	b	a	a	b	a	b	a	b
-	a	b	a	a	b	a	b	a	b
b	a	b	a	a	b	a	b	a	b
b	a	b	a	a	b	a	b	a	b
b	a	b	a	a	b	a	b	a	b
b	a	b	a	a	b	a	b	a	b
b	a	a	b	a	b	a	b	a	b
b	b	a	b	a	b	a	b	a	b
b	a	a	b	a	b	a	b	a	b
b	a	a	-	a	b	a	b	a	b
b	a	-	b	a	b	a	b	a	b
b	a	a	b	a	b	a	b	a	b
b	a	a	b	a	b	-	b	a	b
b	a	a	b	a	b	a	b	a	b
b	a	a	b	a	b	a	b	a	b
b	a	a	b	a	b	a	b	a	b
b	b	a	b	a	b	a	b	a	b
-	a	a	b	a	b	a	b	a	b
b	a	a	b	b	a	a	-	a	b
b	a	a	b	b	a	a	b	a	b
b	a	a	b	b	a	a	b	a	b
b	a	a	b	b	a	a	b	a	b
b	a	a	b	b	a	a	b	a	b
-	a	a	-	b	a	a	b	a	b
b	a	a	b	b	a	a	b	a	b
b	-	a	b	b	a	a	b	a	b
b	-	a	b	b	a	a	b	a	b
b	a	a	b	b	a	a	b	a	b
b	a	a	-	b	a	a	b	a	b

b	a	a	-	b	a	a	b	a	b
b	a	a	b	b	a	a	b	a	b
b	a	a	b	b	b	a	b	a	b
b	a	a	b	b	b	a	b	a	b
b	a	a	b	b	b	a	b	a	b
b	a	a	a	b	b	a	b	a	b
b	a	a	b	b	b	a	b	a	a
b	a	a	b	b	b	a	b	a	a
b	a	a	b	b	b	a	b	a	a
b	a	a	b	b	b	a	b	a	a
-	a	a	b	b	b	a	b	a	a
b	a	a	-	b	b	a	b	a	a
b	b	a	b	b	b	a	b	a	a
b	a	a	b	b	b	a	b	a	a
b	a	a	b	b	b	a	b	a	a
b	a	a	b	b	b	a	b	a	a
b	a	a	b	b	a	a	b	a	-
b	a	a	b	a	a	a	b	a	-
b	a	a	b	a	a	a	b	a	a
b	a	a	b	a	a	a	b	a	a
b	a	a	b	a	a	a	b	a	a
b	a	a	b	a	a	a	b	a	a
b	a	a	b	a	a	a	b	a	a
b	a	a	b	a	a	a	b	a	a
b	a	a	b	a	a	a	b	a	a
b	a	a	-	a	a	a	b	a	a
-	a	a	-	a	a	-	b	a	a
b	a	a	b	a	a	b	b	a	a
b	a	a	b	a	a	b	b	a	a
b	a	a	b	a	a	b	b	a	a
b	a	a	b	a	a	b	b	a	a
-	a	a	b	a	a	b	b	a	a
b	-	a	b	a	a	b	b	a	a
b	a	a	b	-	a	b	b	a	a
b	a	a	b	a	a	b	b	a	a
b	a	a	b	a	a	b	b	a	a
b	a	a	b	a	a	b	b	a	a
b	a	a	b	a	a	b	b	a	a
b	a	a	b	a	a	b	b	b	a
b	a	a	b	a	a	b	b	b	a
b	b	a	b	b	b	a	a	a	a
b	b	a	b	b	b	a	a	a	a
b	b	a	b	b	b	a	a	a	a
b	b	a	b	b	b	a	a	a	a
b	b	a	b	b	b	a	a	a	a
a	b	a	b	a	-	a	a	a	a
a	b	a	b	a	b	a	a	a	a

a	b	a	b	a	b	a	a	a	a
a	b	-	b	a	b	a	a	a	a
a	b	a	b	a	b	a	a	a	a
a	b	a	b	a	b	a	a	a	a
a	b	a	b	a	b	a	a	a	a
a	b	a	b	a	b	a	a	a	a
a	b	a	b	a	b	a	a	a	a
a	b	a	b	-	b	a	a	a	a
a	-	a	-	a	b	a	a	a	a
a	-	a	b	a	b	a	a	a	a
a	b	-	b	a	b	a	a	a	b
a	b	a	-	a	b	a	a	a	a
a	b	a	b	a	b	a	a	a	a
a	b	-	b	a	b	a	a	a	a
a	b	a	b	a	b	a	a	a	a
a	b	a	b	a	b	a	a	a	a
a	b	a	b	a	b	a	a	a	a
a	b	-	b	a	b	a	a	b	a
a	b	a	b	a	b	a	a	a	a
a	b	a	b	a	b	a	a	a	a
a	b	-	b	a	b	a	a	a	a
a	b	a	b	a	b	a	a	a	a
a	b	a	b	a	b	a	b	a	a
a	b	a	b	a	b	a	b	a	a
a	b	a	b	a	b	a	b	a	a
a	b	a	b	a	b	a	b	b	a
a	-	a	b	a	b	a	b	b	a
a	b	-	b	-	b	a	b	b	-
a	a	a	b	a	b	a	b	b	a
a	b	a	b	a	b	a	b	b	a
a	b	-	b	a	b	a	b	b	a
a	-	a	-	a	b	a	b	b	a
a	b	a	b	a	b	a	a	-	a
a	b	a	b	a	b	a	a	b	b
a	b	a	b	a	b	a	a	b	b
a	b	a	-	a	b	a	a	b	b
-	b	a	b	a	b	a	a	b	b
a	b	a	b	a	b	a	a	b	b
a	b	a	b	a	b	a	a	b	b
-	b	a	b	a	b	-	b	b	b
a	b	a	b	a	b	a	a	b	b
a	b	a	b	a	b	a	-	b	b
a	b	a	b	a	b	a	a	b	b
a	b	a	b	a	b	a	a	-	-
a	b	a	b	a	b	a	a	b	b
a	b	b	b	a	b	a	a	b	b
a	a	b	b	a	b	a	a	b	b

a	b	b	b	a	b	a	a	b	b
a	b	b	b	b	b	a	a	b	b
a	b	b	b	b	b	a	a	b	b
a	b	b	b	b	b	a	a	b	b
a	-	b	b	b	b	a	a	b	b
a	b	-	b	b	b	a	a	b	b
-	b	b	b	b	b	a	a	b	b
a	b	-	b	b	b	a	a	b	b
a	b	b	b	b	b	a	a	b	b
a	b	b	b	b	b	a	a	b	b
a	b	b	b	b	b	a	a	b	b
a	-	b	b	b	b	a	a	b	b
a	b	b	b	b	b	a	a	b	b
a	b	b	b	b	b	a	a	b	b
a	b	b	b	b	b	a	a	b	b
a	-	b	-	b	b	a	a	a	b
a	b	b	b	b	b	a	a	a	b
a	-	b	b	b	b	a	a	a	b
-	b	b	b	b	b	a	a	a	b
a	a	b	b	b	b	a	a	a	b
a	b	b	b	b	b	a	a	b	b
a	b	b	b	b	b	a	a	b	b
a	b	b	b	b	b	a	a	b	b
a	b	b	b	b	b	a	-	b	b
a	b	b	b	b	b	a	a	b	b
-	b	b	b	b	b	a	a	b	b
a	b	b	b	b	b	a	b	b	b
a	b	b	b	b	b	a	b	b	b
a	b	-	b	b	b	a	b	b	b
-	b	b	b	b	b	a	b	b	b
a	b	b	b	b	-	a	b	b	b
a	b	b	b	b	b	a	b	b	b
a	b	b	b	b	b	a	b	b	b
a	b	-	b	b	b	a	b	b	b
a	b	b	b	b	b	a	b	b	b
a	b	b	b	b	b	a	b	b	b
a	-	b	b	-	b	a	b	b	b
a	b	b	b	b	b	a	b	b	b
a	b	b	b	b	b	a	b	b	b
a	-	b	b	b	b	a	b	b	b
a	b	b	b	b	b	a	b	b	b
a	b	b	b	a	b	a	b	b	b
a	b	a	b	a	-	a	b	b	b
a	-	a	b	a	b	a	b	b	b
a	b	a	b	a	b	a	b	b	b
a	b	a	b	a	b	a	b	a	b
a	-	a	b	a	b	a	b	a	-

a	b	-	b	a	b	a	b	a	b
a	b	a	b	a	b	a	b	a	b
a	b	a	b	a	b	a	b	-	b
a	b	a	b	a	b	a	b	a	b
a	b	a	b	a	b	a	b	-	b
a	b	a	b	a	b	a	b	a	b
a	b	a	b	a	b	a	b	a	b
a	b	a	b	a	b	a	b	a	b
a	b	a	b	a	b	a	b	a	b
a	b	a	b	a	b	a	b	a	b
a	b	a	b	a	b	a	b	a	b
a	b	a	b	a	b	a	b	a	b
a	b	a	b	a	b	a	b	a	b
a	b	a	b	a	b	a	b	a	b
a	a	a	b	a	b	a	b	a	b
a	-	a	-	a	b	a	b	a	b
a	b	a	b	a	b	a	b	a	b
a	b	a	b	a	b	-	b	a	b
a	b	a	b	a	b	a	b	a	b
a	a	b	b	a	b	a	a	b	a
a	b	b	b	a	b	a	a	b	-
a	b	b	b	a	b	a	a	b	a
a	b	b	b	a	b	a	a	b	a
a	b	b	b	a	b	a	a	b	a
a	b	b	-	a	b	a	a	b	a
a	b	-	b	a	b	a	a	b	a
a	b	b	a	a	b	a	a	b	a
a	b	b	b	a	b	a	a	a	a
a	b	b	a	a	b	a	a	a	a
a	b	b	a	a	b	a	a	a	a
a	b	b	a	a	b	a	a	a	a
a	b	b	a	a	b	a	a	a	a
a	b	b	a	a	b	a	a	a	a
a	b	b	a	a	b	a	a	a	a
a	b	b	a	a	b	a	a	a	b
a	b	b	a	a	b	a	a	a	a
a	-	b	a	a	b	a	a	a	a
a	b	b	a	a	b	-	a	a	a
a	b	b	a	a	b	a	a	a	a
a	b	b	a	a	b	a	a	a	a
a	b	b	a	a	b	a	a	a	a
a	b	b	a	-	b	a	a	a	a
a	b	b	a	a	b	a	a	a	a
a	b	b	a	a	b	a	a	a	a
a	-	b	a	a	b	a	a	a	a
a	b	b	a	a	b	a	a	a	b
a	b	b	a	a	b	a	a	a	a

a	b	b	a	a	b	a	a	a	a
a	b	b	a	a	b	a	a	a	a
a	b	b	a	a	b	a	a	a	a
a	b	b	a	a	b	a	a	a	a
a	-	b	a	a	b	a	a	a	a
a	b	b	a	a	b	a	a	a	a
a	a	b	a	a	b	a	a	a	a
a	-	b	a	a	b	a	a	a	a
a	b	b	a	a	b	a	a	a	a
a	b	b	a	-	b	-	a	a	a
a	b	b	a	a	b	a	a	a	a
a	b	b	a	a	b	a	a	a	a
a	b	b	a	a	b	a	a	a	a
a	b	b	a	a	b	a	a	a	a
a	b	b	a	a	b	a	a	a	a
a	b	b	a	a	b	a	a	a	a
a	b	b	a	a	b	a	a	a	a
a	b	b	a	a	b	a	a	a	a
a	b	b	a	a	b	a	a	a	a
a	b	b	-	a	b	a	b	a	a
a	b	b	a	a	b	a	a	a	a
a	a	b	a	a	b	a	a	a	a
a	a	b	a	a	b	a	b	a	a
-	b	b	a	a	b	-	a	a	a
a	b	b	a	a	b	a	a	a	a
a	b	b	a	a	b	a	a	a	a
a	-	b	a	a	b	a	a	a	a
a	-	b	a	a	b	a	a	a	a
a	b	b	a	a	b	a	a	a	a
a	b	b	a	a	b	a	a	a	a
a	b	b	a	a	b	a	a	a	a
a	b	a	a	a	b	a	a	a	a
a	b	a	a	a	b	a	a	a	a
a	b	a	a	b	b	a	a	a	a
a	b	a	a	b	b	a	a	a	a
a	b	a	a	b	b	a	a	a	a
a	-	a	a	b	b	a	a	a	a
a	b	a	b	b	-	a	a	a	a
a	b	a	b	b	b	a	a	a	a
a	b	a	b	b	b	a	a	a	a
a	b	a	b	b	b	a	a	a	a
a	b	a	b	b	b	a	a	a	a
a	b	a	b	b	b	a	a	a	a
a	b	a	b	b	b	a	a	a	a
a	-	a	b	a	b	a	b	a	a
a	b	a	b	a	b	a	b	a	a
a	b	a	b	a	b	a	b	a	-

a	-	a	b	a	b	a	b	a	a
a	b	a	b	a	b	a	b	a	a
a	b	a	b	a	b	a	b	a	a
a	b	a	b	a	b	a	b	a	a
a	b	a	b	a	b	a	b	a	a
a	b	a	b	a	b	a	b	a	a
a	b	a	b	a	b	a	b	a	a
a	b	a	b	a	a	a	b	a	a
a	b	a	b	a	a	a	b	a	a
a	-	a	b	a	a	a	b	a	a
a	b	a	b	a	a	a	b	a	a
b	a	b	b	b	b	a	b	a	b
b	-	b	b	b	b	a	b	a	b
b	b	b	b	b	b	a	b	a	b
-	a	b	b	b	b	a	b	a	b
b	a	b	b	b	b	a	b	a	b
b	a	b	b	b	b	a	b	a	b
-	a	b	b	b	b	a	b	a	b
b	a	b	b	b	b	a	b	a	b
b	a	b	b	b	b	a	b	a	b
b	a	b	b	b	b	a	b	a	b
b	a	b	b	b	b	-	b	a	b
b	a	b	b	b	b	a	b	a	b
b	a	b	b	b	b	a	b	a	b
b	b	b	b	b	b	a	a	a	b
b	b	a	b	b	b	a	a	a	b
b	b	a	b	b	b	a	a	a	b
b	b	a	-	b	b	a	a	a	b
b	b	a	b	b	b	a	a	a	b
b	b	a	b	b	b	a	a	a	b
b	b	a	b	b	b	a	a	a	b
b	b	a	b	b	b	a	a	a	b
b	b	a	b	b	b	a	a	a	b
b	b	a	b	b	b	a	a	a	b
b	b	a	b	b	b	a	a	a	b
b	b	a	b	b	b	a	a	a	b
b	-	b	b	b	b	a	a	a	-
b	b	b	b	b	b	a	a	a	b
b	b	b	b	b	b	a	a	a	b
b	b	b	b	b	b	a	a	a	b
b	b	b	b	b	b	a	a	a	b
b	b	b	b	b	b	a	a	a	b
b	b	b	b	b	a	a	a	a	b
b	b	b	b	b	a	a	a	a	b
b	b	b	b	a	a	a	a	a	b
b	b	b	b	a	a	a	a	a	b
b	b	b	b	a	a	a	a	a	b

b	b	b	b	a	a	a	a	a	b
b	b	b	b	b	a	a	a	a	b
b	a	b	-	b	a	a	a	a	-
b	b	-	b	-	a	a	a	a	b
b	b	b	b	b	a	a	a	a	b
b	a	b	b	b	a	a	a	a	b
b	b	b	b	b	a	a	a	a	b
b	b	b	b	b	a	a	a	a	b
b	-	b	-	b	a	a	a	a	b
a	b	b	-	b	a	a	a	a	b
b	b	b	b	b	a	a	a	a	b
b	b	b	b	b	a	a	a	a	b
b	b	b	b	b	a	a	a	a	b
b	b	-	b	b	a	a	a	a	b
b	b	b	b	b	a	a	a	b	b
b	-	b	b	b	a	a	a	b	b
b	b	-	-	b	a	a	a	b	b
b	b	b	b	b	a	a	a	b	b
b	b	b	b	b	a	a	a	b	b
b	b	b	b	b	a	a	a	b	b
b	b	b	b	b	a	a	a	b	b
b	-	b	b	-	a	a	a	b	b
b	b	b	b	b	-	a	a	b	b
b	b	b	-	b	a	a	a	b	a
b	b	b	b	b	a	a	a	b	b
b	b	b	b	b	a	a	a	b	b
b	b	b	b	b	a	a	a	b	b
b	b	b	b	b	a	a	a	b	b
a	b	b	b	b	a	a	a	b	b
a	b	b	b	b	a	a	a	b	b
a	b	b	b	b	a	a	a	b	b
a	b	b	b	b	a	a	a	b	b
a	b	b	b	b	a	a	a	b	b
a	-	b	b	b	a	a	a	b	b
a	b	b	b	b	a	a	a	b	b
a	b	b	b	b	a	a	a	b	b
a	b	b	b	b	a	a	a	b	b
a	b	b	b	b	a	a	a	b	b
a	-	b	b	b	a	a	a	b	b
a	b	b	b	b	a	a	a	b	b
a	b	b	b	b	a	b	a	b	b
a	b	b	b	a	a	b	a	b	b
a	b	b	b	a	a	b	a	b	b
a	a	b	b	a	a	b	a	b	b
a	a	-	a	a	a	b	a	b	-
a	a	b	b	a	-	b	a	b	b
a	a	b	b	a	a	b	a	b	b

a	a	b	b	a	a	b	a	b	b
a	a	b	b	a	a	b	a	b	b
-	-	b	b	a	a	b	a	b	b
a	a	b	b	a	a	b	a	b	b
a	-	b	b	a	a	b	a	b	b
a	a	b	b	a	a	b	a	a	b
a	a	b	b	a	a	b	a	a	b
a	a	b	b	a	a	b	a	a	b
a	a	b	b	a	a	b	a	a	b
a	a	b	b	a	a	b	a	a	b
a	a	b	-	a	a	b	a	a	b
a	a	b	b	a	a	b	a	a	b
a	-	b	b	a	a	b	a	a	b
a	a	b	b	a	a	b	a	a	b
a	a	b	-	a	a	b	a	a	b
a	b	b	a	a	a	b	a	a	b
a	a	b	a	a	a	b	a	a	b
a	a	-	a	a	a	b	a	a	b
a	a	b	a	a	a	b	a	a	b
a	-	b	b	a	a	b	a	a	b
a	a	b	a	a	a	b	a	a	b
a	-	b	-	a	a	b	a	a	b
-	a	a	b	b	b	-	a	b	a
b	a	a	b	b	b	b	a	b	a
b	a	a	b	b	b	b	a	b	a
b	a	a	b	b	b	b	a	b	a
b	-	a	b	b	b	b	a	b	a
b	a	a	b	b	b	b	-	b	a
b	a	a	b	b	b	b	a	b	a
b	a	a	b	b	b	b	a	b	a
b	a	a	b	b	b	b	a	b	a
b	a	a	b	b	b	b	a	b	a
b	a	a	b	b	b	b	a	b	a
b	a	a	-	b	b	b	a	b	a
b	a	a	b	b	b	b	a	b	a
b	a	a	b	b	b	b	a	b	a
-	a	a	b	b	b	b	a	b	a
b	-	-	b	b	b	b	a	b	a
b	b	a	b	b	b	b	a	b	a
b	b	-	b	b	b	b	a	b	-
b	b	a	b	b	b	b	a	b	a
b	b	a	b	b	b	b	a	b	a
b	b	a	b	b	b	b	a	b	a
b	-	a	b	b	b	-	a	b	a
b	b	a	b	b	b	b	a	b	a
b	b	a	b	b	b	b	a	b	a
b	b	b	b	b	b	b	a	b	a

b	b	b	b	a	b	b	a	b	a
b	b	b	b	a	b	b	a	b	-
b	b	b	b	a	b	b	a	b	a
b	b	b	b	a	b	b	a	b	a
b	-	b	b	a	b	b	a	b	a
b	b	b	b	a	b	b	a	b	a
-	b	b	b	a	b	b	a	b	a
b	-	b	b	a	b	b	a	b	a
b	b	b	a	a	b	b	a	b	a
b	b	b	b	a	b	b	a	b	-
b	b	b	b	a	b	b	a	b	a
b	b	-	b	a	b	b	a	b	a
b	-	b	a	a	b	b	a	b	a
b	b	b	a	a	b	b	a	b	a
b	b	b	a	a	b	b	a	b	a
b	b	b	a	a	b	b	a	b	a
b	b	b	a	a	b	b	a	b	a
b	b	b	b	a	b	b	a	b	a
b	b	b	a	a	b	b	a	b	-
-	a	b	a	a	b	b	a	a	a
b	b	b	a	a	b	b	a	a	a
b	b	b	-	a	b	b	a	a	a
b	b	b	-	a	b	b	a	a	a
b	b	b	a	a	b	b	a	a	-
b	b	b	a	a	b	b	a	a	a
b	b	b	a	a	b	b	a	a	a
b	b	b	a	a	b	b	a	a	a
b	b	b	-	a	b	b	a	a	a
b	b	b	a	a	b	b	a	a	a
b	b	b	a	a	b	b	a	a	a
b	b	b	a	a	b	b	a	a	a
b	b	b	a	a	b	b	a	a	a
b	b	b	-	a	b	b	a	a	a
b	b	b	-	a	b	b	a	a	a
b	b	b	a	a	b	b	a	a	a
b	b	b	-	-	b	b	a	a	a
b	b	b	a	a	b	b	a	a	b
b	b	b	a	a	b	b	a	a	b
b	b	b	a	a	b	b	a	a	b
b	b	b	-	a	b	b	a	a	b
b	b	b	a	a	b	b	a	a	b
b	b	b	a	a	b	b	a	a	b
b	b	b	a	a	b	b	a	a	b
b	b	b	a	a	b	b	a	a	b
b	b	b	a	a	b	b	a	a	b
b	-	b	a	a	a	b	a	a	b
b	b	b	a	a	a	b	a	a	b

b	b	b	a	a	a	b	-	a	b
b	b	b	-	a	a	b	a	a	b
b	b	b	a	a	a	b	a	a	b
b	-	b	a	a	a	b	a	a	b
b	b	b	-	a	a	b	a	a	b
b	b	b	a	a	a	b	a	a	b
b	b	b	a	a	a	b	a	a	b
b	b	b	b	a	a	b	a	a	b
b	b	b	a	a	a	b	a	a	b
b	b	b	a	a	a	b	a	a	b
b	-	b	a	a	a	b	a	a	b
b	b	b	a	-	a	b	a	a	b
b	b	b	a	a	a	b	a	a	b
b	b	b	a	a	a	b	a	a	b
b	b	b	a	a	a	b	a	a	b
b	b	b	a	a	a	b	a	a	b
b	b	b	a	a	a	b	a	a	b
b	b	b	a	a	a	b	a	a	b
b	b	b	a	a	a	b	a	a	b
b	-	b	a	a	a	b	a	a	b
b	b	b	a	-	a	b	a	a	b
b	b	b	a	a	a	b	a	a	b
-	-	b	a	a	a	b	a	a	b
b	b	b	a	a	a	b	a	a	b
b	b	b	a	a	a	b	-	a	b
b	b	b	a	a	a	b	a	a	b
b	b	b	a	a	a	b	a	a	b
b	-	b	a	a	a	b	a	a	b
a	b	b	a	a	a	b	a	a	b
a	b	-	a	a	a	b	a	a	b
a	b	b	a	a	a	b	a	a	b
a	a	b	a	a	a	b	a	a	b
a	a	b	a	a	a	b	a	a	b
a	a	b	a	a	a	b	a	a	b
a	a	b	a	a	a	b	a	a	b
a	a	b	a	a	a	b	a	a	b
a	a	b	a	a	a	b	a	a	b
a	a	b	a	a	a	b	a	a	b
a	a	b	a	a	a	b	a	a	b
a	a	b	a	a	a	b	a	a	b
a	a	b	a	-	a	b	a	a	b
a	a	-	a	a	a	b	a	a	b
a	a	b	a	a	a	b	a	a	b
a	a	b	a	a	a	b	a	-	b
a	a	b	a	a	a	b	a	a	b
a	a	b	a	a	a	b	a	a	b
a	a	-	a	a	a	b	a	a	b

a	a	b	a	a	a	b	a	a	-
a	a	b	a	a	a	b	a	a	-
a	a	b	a	a	a	b	a	a	a
a	b	a	a	a	a	b	a	b	a
b	a	a	a	a	a	b	b	b	a
b	-	a	a	-	a	b	b	b	a
b	a	a	a	b	a	b	b	b	a
b	a	a	a	b	a	a	b	b	a
b	a	a	a	b	a	a	b	b	b
b	a	a	a	b	b	a	b	b	b
b	a	a	a	b	b	a	b	b	b
b	a	a	a	b	b	a	b	b	b
b	a	a	a	b	b	a	b	b	b
-	a	a	a	b	b	a	b	-	b
b	-	a	a	b	b	a	b	b	b
b	a	a	a	b	b	a	b	b	-
b	a	a	a	b	b	a	b	b	b
b	b	a	a	b	b	a	b	b	b
b	b	a	-	b	b	a	b	b	b
b	b	a	a	b	b	a	b	b	b
b	b	a	a	b	b	a	b	b	b
b	a	a	a	b	b	a	b	b	b
b	b	a	-	b	b	a	b	b	b
-	b	a	a	b	b	a	b	b	b
b	a	a	a	b	b	a	b	b	b
b	b	a	a	b	b	a	b	b	b
b	b	a	a	b	b	a	b	b	b
b	b	a	a	b	b	a	b	b	-
b	b	a	a	b	b	a	b	b	b
b	b	a	a	b	b	a	b	b	b
b	b	a	a	b	b	a	b	b	b
b	b	a	a	b	b	a	b	b	b
b	b	a	a	b	b	a	b	b	b
b	b	a	a	b	b	a	b	b	b
b	b	a	a	b	b	a	b	b	b
b	b	a	a	b	b	a	b	b	b
b	b	a	a	b	b	a	b	b	b
a	b	a	a	b	b	a	b	b	a
-	b	a	a	b	a	b	b	a	b
a	b	-	a	b	a	b	b	a	a
a	b	b	a	b	b	b	b	a	a
a	b	b	a	b	b	b	b	a	a
a	b	b	a	b	b	b	b	a	a
a	b	b	a	b	b	b	b	a	a
a	-	b	a	b	b	b	b	a	a
a	a	b	a	b	b	b	b	a	a
b	a	b	a	b	b	b	b	a	a

b	a	b	a	b	b	b	b	a	a
b	a	b	a	b	b	b	b	a	a
b	a	b	a	b	b	b	b	a	b
b	a	b	a	b	b	b	b	a	b
b	b	b	a	b	a	a	a	a	a
b	b	b	a	b	a	a	a	a	a
b	b	b	-	b	a	a	a	a	a
b	-	b	a	b	a	a	a	a	a
b	b	b	a	b	a	a	a	a	a
b	b	b	-	b	a	a	a	a	a
b	b	b	a	b	a	a	a	a	a
b	-	b	b	b	a	a	b	a	a
b	b	b	b	b	b	a	b	a	a
b	b	b	b	b	b	a	b	a	b
b	-	-	b	b	b	a	b	a	b
b	-	b	b	b	b	a	b	a	b
b	b	b	b	b	b	a	b	a	b
b	a	b	b	b	b	a	b	a	b
b	-	b	b	b	b	a	b	a	b
b	a	b	b	b	b	a	b	a	b
b	a	b	b	-	b	a	b	a	b
b	a	b	b	b	b	b	b	a	b
b	a	b	b	b	b	b	b	-	b
b	a	b	b	b	b	b	b	a	b
b	a	b	b	b	b	b	b	a	b
b	-	b	a	b	b	b	b	a	b
b	-	b	a	b	b	b	b	a	b
b	a	b	-	b	b	b	b	a	b
b	a	b	a	b	b	b	b	a	b
b	a	b	a	b	b	b	b	a	b
b	a	b	a	b	-	b	b	a	b
a	a	b	a	b	b	b	b	a	b
a	a	b	a	b	b	b	b	a	b
b	a	b	a	b	b	a	b	a	b
b	a	b	a	b	b	b	b	a	b
b	a	b	a	b	b	b	b	a	-
b	a	b	a	b	b	b	b	a	b
-	a	b	a	b	b	b	b	a	b
b	-	b	a	b	b	b	b	a	b
a	a	b	a	b	b	b	b	a	b
a	a	b	a	b	b	b	b	a	b
-	a	b	a	b	b	b	b	a	b
a	a	b	b	b	b	b	b	a	b
a	a	-	a	b	b	b	b	a	b
a	a	b	a	a	b	b	b	a	b
a	b	b	-	b	a	a	-	b	a
a	a	b	a	b	a	a	a	b	a

a	a	b	a	b	a	a	a	b	a
a	a	b	a	b	a	a	a	-	a
a	a	b	a	-	a	a	a	b	a
a	a	b	a	b	a	a	a	b	a
a	a	b	a	b	a	-	a	b	a
a	a	b	-	b	a	a	a	b	a
a	a	b	a	b	a	a	a	b	a
a	a	-	a	b	a	a	a	b	a
a	a	b	a	b	a	a	a	b	a
a	-	b	a	b	a	-	a	b	a
b	a	b	-	b	a	a	a	b	a
b	a	b	a	b	a	a	a	b	a
b	a	-	a	b	a	a	a	a	a
b	a	b	a	b	a	a	a	b	a
b	a	b	a	b	a	a	a	b	a
b	a	b	a	b	a	a	b	b	a
b	a	b	-	b	a	a	b	a	a
b	-	b	a	b	a	a	b	a	a
b	a	b	a	b	a	a	b	a	a
b	a	b	a	b	a	a	b	a	a
b	a	b	a	b	a	a	b	a	a
b	b	b	a	b	a	a	b	a	a
b	a	b	a	-	a	a	b	a	a
b	a	b	a	b	a	a	b	a	-
b	a	b	a	b	a	b	b	a	a
b	a	b	a	b	a	b	b	a	a
b	a	b	a	b	a	b	b	b	a
b	a	b	a	b	a	b	b	b	a
b	a	b	a	b	a	b	b	b	a
b	-	b	-	b	a	b	b	b	a
b	a	b	a	-	a	b	b	b	a
b	a	b	a	b	a	b	b	b	a
b	a	b	a	b	a	b	b	b	a
b	a	b	a	a	b	b	b	b	a
b	a	b	a	a	b	b	b	b	a
b	a	b	a	a	b	b	b	b	a
b	b	b	a	a	b	b	b	b	a
b	a	b	a	a	b	b	b	b	a
b	-	b	a	a	b	b	b	b	a
b	a	b	a	a	b	b	-	b	a
b	-	b	-	a	b	b	b	b	a
b	a	b	a	a	b	b	a	b	a
b	a	-	a	a	b	b	a	b	a
b	a	b	-	a	b	b	a	b	a
b	a	b	a	a	b	b	a	b	a
b	a	-	a	a	b	b	a	b	a
b	a	b	a	a	b	b	a	b	a

b	a	b	a	-	b	b	a	b	a
-	a	b	a	a	b	b	a	b	a
b	a	b	a	a	b	b	b	b	a
b	a	b	a	a	b	b	b	b	a
b	a	b	a	a	b	b	b	b	a
b	a	b	a	a	b	b	b	b	a
b	a	b	a	a	b	b	b	b	a
b	a	b	a	a	b	b	-	b	a
b	a	b	a	a	b	b	b	b	a
b	a	b	-	a	b	b	b	b	a
b	a	b	a	a	b	b	b	b	a
b	a	b	a	a	b	b	b	b	a
b	a	b	a	a	b	b	b	b	a
b	a	b	a	a	b	b	b	b	a
b	a	b	a	a	b	b	b	b	a
b	-	b	a	a	b	b	b	b	a
b	a	b	-	a	b	b	b	b	a
b	a	b	a	a	b	b	b	b	a
b	a	b	a	a	b	b	b	b	b
b	a	b	a	a	b	b	a	b	b
a	b	b	a	a	b	b	b	b	b
b	b	b	a	a	b	b	b	b	b
a	b	b	a	a	b	b	b	b	b
a	b	b	a	a	b	b	b	b	b
b	b	b	a	a	b	b	b	b	b
b	b	b	-	a	b	b	b	b	b
b	b	b	a	a	b	b	b	b	b
b	b	b	a	a	b	b	b	b	b
b	-	b	a	a	b	b	b	b	b
b	b	b	a	a	b	b	b	b	b
b	b	b	a	a	b	b	b	b	b
b	b	b	a	a	b	b	b	b	b
b	b	b	a	a	b	b	b	b	b
b	b	b	a	a	b	b	b	b	b
b	b	b	a	a	b	b	b	a	b
b	b	b	a	a	b	b	b	b	b
b	b	b	-	a	b	b	b	b	b
b	b	b	a	a	b	b	b	b	b
b	b	b	a	a	b	b	b	b	b
b	-	b	a	a	b	b	b	b	b
b	-	b	a	a	b	b	b	b	b
b	b	b	a	a	b	b	b	b	b
b	-	b	b	a	b	b	b	b	b
b	b	b	b	a	b	b	b	b	b
b	b	b	b	a	b	b	b	b	b
b	b	b	b	a	b	b	b	b	b
b	b	b	b	b	b	b	b	a	b

b	b	b	b	b	b	b	b	a	b
b	-	b	b	-	b	b	b	a	b
b	b	b	b	b	b	-	b	a	b
b	b	b	b	b	b	b	b	a	b
b	b	b	b	b	b	b	b	a	b
b	b	b	b	b	b	b	b	a	b
b	b	b	b	b	b	b	b	a	b
b	a	b	b	b	b	a	b	a	b
a	b	b	b	-	a	b	b	a	b
a	b	b	b	a	a	b	b	a	b
a	b	b	b	a	a	b	b	a	b
a	b	b	b	a	a	b	b	a	b
a	b	b	b	a	a	b	b	a	b
a	-	b	b	a	a	b	b	a	b
a	b	b	b	a	a	b	b	a	b
a	-	b	b	a	a	b	b	a	b
a	b	b	b	-	a	b	b	a	b
a	b	b	b	a	a	b	b	a	b
-	b	b	b	-	a	b	b	a	b
b	b	b	b	a	a	b	b	a	b
b	-	b	b	a	a	b	b	a	b
b	a	b	b	a	a	b	b	a	b
b	a	b	b	a	a	b	b	a	-
b	a	b	b	a	a	b	b	-	-
b	a	b	b	a	a	b	b	a	a
b	a	b	b	a	a	b	b	a	a
b	a	b	b	a	a	b	b	a	a
b	a	b	b	a	a	b	b	a	b
b	a	b	b	a	a	b	b	a	b
b	a	b	b	a	a	b	b	a	b
a	a	b	b	a	a	b	b	a	b
b	a	b	b	a	a	b	b	a	b
b	a	b	b	a	a	b	b	a	b
b	a	b	b	a	a	b	b	a	b
b	a	b	b	a	a	b	b	a	b
b	a	b	b	b	a	a	b	b	b
-	a	b	b	b	a	a	b	b	-
b	a	b	b	b	a	a	b	b	b
b	a	b	-	-	a	a	b	b	b
-	a	b	b	b	a	a	b	b	b
b	a	b	b	b	a	a	b	b	b
b	a	b	b	b	a	a	b	b	b
b	a	b	b	b	a	a	b	b	b
b	a	a	-	b	b	a	b	b	b
b	-	a	b	b	b	a	b	b	b
b	a	a	b	b	b	a	b	b	b
b	a	a	b	b	b	a	b	b	b

b	a	a	b	b	b	a	b	b	b
-	a	a	b	b	b	a	b	b	b
b	a	a	b	b	a	a	b	b	b
b	a	a	b	b	a	a	b	b	b
b	a	a	b	b	a	a	b	b	b
b	a	a	-	b	a	a	b	b	b
b	a	a	b	b	a	a	b	b	b
b	a	a	b	-	a	a	b	b	b
b	a	a	b	b	a	a	b	b	b
a	a	a	-	b	a	a	-	b	-
b	a	a	b	b	a	a	b	b	-
b	a	a	b	b	a	a	b	b	b
b	b	a	a	b	a	b	a	a	a
b	b	a	a	b	a	b	a	a	a
b	b	a	a	b	a	b	a	a	a
b	a	-	a	-	a	a	a	a	a
b	a	b	a	b	a	a	a	a	a
b	a	b	b	a	a	a	a	a	a
b	a	b	-	a	a	a	a	a	b
b	a	b	b	a	a	a	a	a	b
b	a	b	a	a	a	a	a	a	b
b	b	b	b	-	a	a	a	a	b
b	a	b	b	a	a	a	a	a	b
b	a	b	b	a	a	a	a	a	b
b	-	b	b	a	b	-	a	a	b
b	b	b	b	a	b	a	a	a	b
b	a	b	b	a	b	a	a	b	b
b	a	b	b	a	b	a	b	b	b
b	a	b	b	a	b	a	b	b	b
b	a	b	b	a	b	a	b	b	b
a	a	b	b	a	b	a	b	b	b
b	a	-	b	a	b	a	b	b	b
b	a	b	b	-	b	a	b	b	b
b	a	b	b	b	b	a	b	b	b
b	a	b	b	b	b	a	b	b	b
b	-	b	b	b	b	a	b	b	b
b	a	b	b	b	b	a	b	b	b
-	a	b	-	b	b	a	b	b	-
b	a	b	b	b	b	-	b	b	b
b	a	b	b	b	b	a	b	b	b
b	b	b	b	b	b	a	b	b	b
b	b	b	b	b	b	a	b	b	b
b	b	b	b	b	b	a	b	b	b
b	b	b	b	b	b	a	b	b	b
b	b	b	b	b	b	a	b	b	b
b	b	b	b	b	b	b	a	b	b
b	-	a	b	b	b	b	a	b	b

b	-	a	b	b	b	b	a	b	b
b	b	a	b	b	b	b	a	b	b
b	b	a	b	b	b	b	a	b	b
a	b	-	a	b	b	b	a	a	b
a	b	a	a	b	b	b	a	a	-
a	b	a	a	b	b	b	a	a	a
a	b	a	a	b	b	b	a	a	a
a	b	a	a	b	b	b	a	a	a
a	a	b	a	-	b	a	a	a	b
a	-	b	a	b	b	a	a	a	b
a	a	b	a	b	b	a	a	a	b
a	a	b	a	-	b	a	a	a	b
a	b	b	a	b	b	-	a	a	b
a	a	-	a	b	b	a	a	a	b
a	a	b	a	b	b	a	a	a	b
a	a	b	-	b	b	a	a	a	b
a	a	-	a	b	b	a	a	a	b
a	-	b	a	b	b	a	a	a	b
a	a	b	a	b	b	a	a	a	b
a	a	b	a	b	b	a	a	a	-
b	a	b	a	b	b	a	a	a	b
b	a	b	a	b	b	a	a	a	b
b	a	b	a	b	b	a	a	a	b
b	a	b	a	b	b	a	a	a	b
-	a	b	a	b	b	a	a	a	b
b	a	b	a	b	b	a	a	a	b
b	a	b	a	b	b	a	a	a	b
b	a	b	a	b	b	a	a	a	-
b	a	b	a	b	b	a	a	a	b
b	a	b	a	b	a	a	a	-	b
b	-	b	a	b	a	a	a	a	b
b	a	b	a	b	a	a	a	a	b
b	a	-	a	b	a	a	a	a	b
b	a	b	a	b	a	a	a	a	b
b	a	b	b	b	a	a	a	a	b
-	a	b	b	b	a	a	a	a	b
b	a	b	b	b	a	a	a	a	b
b	a	b	b	b	a	a	a	b	-
b	a	b	b	b	a	a	a	b	b
b	a	b	b	b	a	a	a	b	b
b	a	-	b	b	a	a	a	b	-
-	a	b	b	-	a	a	a	b	b
b	a	b	b	b	a	a	a	b	b
b	a	b	b	b	a	a	a	b	b
b	a	b	b	b	a	a	a	b	b
b	a	-	a	b	a	a	a	b	-
b	a	b	-	b	a	a	b	b	b

b	a	-	b	b	a	a	b	b	b
b	a	b	b	b	a	a	b	b	b
b	a	b	b	b	a	a	b	b	b
b	a	b	b	b	a	b	b	b	b
b	a	b	b	b	a	b	b	b	b
-	a	b	b	b	a	b	b	b	b
b	a	b	b	b	a	a	b	b	b
b	a	b	a	b	a	a	b	b	b
b	-	b	b	b	a	a	b	b	b
b	a	b	b	b	a	a	b	b	b
b	a	-	b	b	a	a	b	b	b
b	a	a	b	b	a	a	b	b	b
b	a	a	b	b	a	a	b	b	b
b	-	a	b	b	a	a	b	b	b
b	a	a	b	b	a	a	b	b	b
b	a	a	b	b	a	a	b	b	b
b	a	a	b	b	a	a	b	b	b
b	a	a	b	b	a	a	b	b	b
a	a	-	b	-	a	a	b	b	b
a	a	b	b	-	a	a	b	b	b
b	a	b	b	b	a	a	b	b	b
b	-	b	b	b	a	a	b	b	b
b	a	b	b	b	a	a	b	b	b
b	a	b	b	b	b	a	b	b	b
b	a	b	b	b	b	a	b	b	b
b	-	a	b	b	b	a	b	b	b
b	a	a	b	b	b	a	b	b	b
b	a	a	b	b	b	a	b	b	b
a	a	a	-	b	b	a	b	b	b
a	a	a	b	b	b	a	b	b	b
a	a	a	b	b	b	a	b	b	b
a	a	a	b	b	b	a	b	b	b
a	a	a	b	b	b	a	b	b	b
a	a	a	b	b	b	a	b	b	b
a	a	a	b	b	b	a	b	b	b
a	a	a	b	-	b	a	b	b	b
a	a	a	b	b	b	a	b	b	b
a	a	a	b	b	b	a	b	b	b
a	a	a	b	b	b	a	b	b	b
a	b	a	b	b	b	a	b	b	b
a	b	a	a	b	b	a	b	b	b
a	b	a	a	b	b	a	-	b	b
a	-	a	a	b	b	a	b	b	a
a	b	a	a	b	b	a	b	-	-
a	b	a	a	b	b	a	b	b	b
a	-	a	a	b	b	a	b	b	b
a	b	a	a	b	b	a	b	b	b

a	b	-	a	b	b	a	a	b	b
a	b	a	a	b	b	a	a	-	b
a	b	a	a	b	b	a	a	b	b
a	b	a	a	b	b	a	a	b	b
a	-	a	a	b	b	a	a	b	b
a	b	a	a	b	b	a	a	b	b
a	b	a	a	b	b	a	a	b	b
-	b	a	a	b	b	a	a	b	b
a	b	a	a	b	b	a	a	b	b
a	b	a	a	b	b	b	a	b	b
a	b	a	a	b	b	b	a	b	b
a	-	a	a	b	b	b	a	b	b
a	a	a	a	b	b	-	a	b	b
a	a	a	a	b	b	b	a	b	b
a	a	a	a	b	b	b	a	b	b
a	a	a	a	b	b	b	a	b	b
a	a	a	a	b	b	b	a	b	b
a	b	a	a	b	b	b	a	b	b
a	b	a	-	b	b	b	a	b	b
a	b	-	a	b	b	b	a	b	b
a	b	a	a	b	b	b	a	b	b
a	b	a	a	b	b	b	a	b	b
a	-	a	a	b	b	b	a	b	b
a	b	a	a	-	b	b	a	b	b
a	b	a	a	-	b	b	a	b	b
a	-	a	a	b	b	b	a	b	b
a	b	a	a	b	b	b	a	b	b
a	b	a	-	b	b	b	a	b	b
a	b	a	a	-	b	b	a	b	b
a	b	a	a	b	b	b	a	b	b
a	b	a	a	b	b	b	b	b	b
-	b	a	b	b	b	b	a	b	b
a	b	a	a	b	b	a	a	b	b
a	b	a	a	-	b	a	a	b	-
a	b	a	a	b	b	a	a	b	b
a	b	b	b	b	-	a	b	a	a
a	b	b	b	b	a	a	b	a	a
a	b	b	-	b	a	a	b	a	a
a	b	b	b	b	a	a	b	a	a
a	b	b	b	b	a	a	b	a	-
a	b	b	b	b	a	a	b	a	a
a	b	b	b	b	a	a	b	a	a
a	b	b	b	b	a	a	b	a	a
a	b	b	b	b	a	a	b	a	a
a	b	b	b	b	b	a	b	a	a
a	b	b	b	b	b	a	b	a	a
a	b	b	b	b	b	a	b	a	a

a	a	b	b	-	b	a	b	a	a
a	b	b	b	b	-	a	b	a	a
a	b	b	b	b	b	a	b	a	a
a	b	b	b	-	b	a	b	a	a
a	b	b	b	b	b	a	b	a	a
b	b	b	b	b	b	a	b	a	a
b	b	b	b	b	b	a	b	a	a
b	b	b	b	b	b	a	b	a	a
b	b	b	b	b	b	a	b	b	a
b	a	b	b	-	b	a	b	b	a
b	b	b	b	b	a	a	b	b	a
b	-	b	b	b	a	a	b	b	a
b	a	b	b	b	a	b	b	b	a
b	a	b	b	b	b	a	b	b	a
b	a	b	-	b	b	a	b	b	a
b	a	b	b	b	b	a	b	b	a
b	a	b	b	b	a	a	b	b	a
-	a	b	b	b	a	a	b	b	a
b	a	b	b	b	a	a	b	b	a
b	a	b	b	b	a	a	b	b	a
a	a	b	b	b	b	a	b	b	a
a	a	b	b	b	b	a	b	b	a
a	a	b	b	-	b	a	b	b	a
a	a	b	b	a	b	a	b	b	a
a	a	b	b	a	b	a	b	b	a
a	a	b	b	a	b	a	b	b	a
a	a	b	b	a	b	a	b	b	a
a	a	b	b	a	b	a	b	b	a
a	a	b	b	a	b	a	b	b	b
a	a	b	b	a	b	a	b	b	b
a	a	b	b	a	b	a	b	b	b
a	a	b	b	a	b	a	b	b	b
a	a	b	b	a	b	a	b	b	b
a	a	b	b	a	b	a	b	b	b
a	a	b	b	a	b	a	b	b	b
a	a	b	b	a	b	a	b	b	-
a	a	b	b	a	b	a	a	b	b
a	a	b	b	a	b	a	a	b	b
a	a	b	b	a	b	a	a	b	b
a	a	b	b	a	b	a	a	b	b
a	a	b	b	a	b	a	a	b	b
a	a	-	a	a	b	a	a	b	b
a	a	b	b	a	b	a	a	b	b
a	a	b	b	a	b	a	a	b	b
a	a	b	b	a	b	a	a	b	b
a	a	b	b	a	b	a	a	b	b
b	a	b	b	a	b	b	a	b	a

b	a	b	b	a	b	b	a	b	a
b	a	b	b	a	b	b	a	b	b
b	a	b	b	a	b	b	a	b	b
b	a	b	b	a	b	b	a	b	b
b	a	b	b	a	b	b	a	b	b
b	a	b	b	a	b	b	a	b	b
b	a	b	b	a	b	b	a	b	b
b	a	b	b	a	b	b	a	b	b
b	a	b	b	a	b	b	a	b	b
b	a	b	b	a	b	b	a	b	b
b	a	b	b	a	b	b	a	b	a
b	a	b	b	a	b	b	a	b	a
b	a	b	b	a	b	b	a	b	a
b	a	b	b	a	b	b	a	b	a
b	a	b	b	a	b	b	a	b	a
b	a	b	b	a	b	b	a	b	a
b	a	b	b	a	b	b	a	b	a
b	a	b	b	a	b	b	a	b	a
b	a	b	b	a	b	a	a	a	b
b	a	b	b	a	b	a	a	a	b
b	a	b	b	a	b	a	a	a	b
b	a	b	b	a	b	a	a	a	b
b	a	b	b	a	b	a	a	a	b
b	a	b	b	a	b	a	a	a	b
b	a	a	b	a	b	a	a	a	b
b	a	a	b	a	b	a	a	a	b
b	a	a	b	a	b	a	a	a	b
b	a	a	b	a	b	a	a	a	b
b	a	a	b	a	b	a	a	a	b
b	a	a	b	a	b	a	a	a	b
b	a	a	b	a	b	a	a	a	b
b	a	a	b	a	b	a	b	a	b
b	a	a	b	a	b	a	b	a	b
b	a	a	b	a	b	a	b	a	b
b	a	a	b	a	b	b	b	a	b
b	a	a	b	a	b	b	b	a	b
b	a	a	b	a	b	b	b	a	b
a	-	a	b	a	b	b	b	a	b
b	a	a	b	a	b	a	b	a	b
b	b	a	b	b	b	b	b	a	b
a	-	b	a	b	b	a	a	b	a
b	b	b	a	b	a	a	b	a	b
b	b	b	b	b	a	a	b	a	b
b	b	b	b	b	a	a	b	a	b
b	b	b	b	b	a	a	b	a	b
b	b	b	b	b	a	a	b	a	b
b	b	b	b	b	a	a	b	a	b

b	b	b	b	b	a	a	b	a	b
b	b	b	b	b	a	a	b	a	b
-	b	b	b	b	a	a	b	a	b
a	b	b	b	b	a	a	b	a	b
a	b	b	b	b	a	a	b	a	b

YW059	YW060	YW061	YW062	YW063	YW064	YW065	YW066	YW067	YW068
b	a	a	b	a	b	b	a	a	a
b	a	b	b	a	b	b	a	a	a
b	-	b	b	a	b	b	a	a	a
b	a	b	b	a	-	b	a	a	a
b	a	b	b	a	b	b	a	a	a
b	a	b	b	a	b	b	a	a	a
b	a	b	b	a	b	b	a	a	a
b	a	b	b	a	b	b	a	a	a
b	-	b	b	a	b	b	a	a	a
b	a	b	b	a	-	b	a	a	a
b	a	b	b	a	b	b	a	a	a
b	a	b	b	a	b	b	a	a	a
b	a	b	b	a	b	b	a	a	-
b	b	b	b	a	b	b	a	a	a
b	b	b	b	a	b	b	a	a	a
b	a	b	b	a	b	b	a	a	b
b	a	b	b	a	b	b	a	a	b
b	a	b	b	a	b	b	a	a	b
b	b	b	b	a	b	b	a	a	b
b	-	b	b	a	b	b	a	a	b
b	a	b	-	a	b	b	a	a	b
b	a	b	b	a	b	b	a	a	b
b	a	b	b	a	b	b	a	a	b
b	a	b	b	a	b	b	a	a	b
b	a	b	b	a	b	b	a	a	b
b	a	b	b	a	b	-	a	a	b
b	b	b	b	a	b	b	b	a	b
b	b	-	b	a	b	b	b	a	b
b	b	b	b	a	-	b	b	a	b
b	b	b	b	a	b	b	b	a	b
b	b	b	b	a	b	b	b	a	b
b	b	b	b	a	b	b	b	a	b
b	b	b	b	-	b	b	b	a	b
b	b	-	b	a	b	b	b	a	b
b	b	b	b	a	b	b	b	b	b
b	b	b	b	a	b	b	b	b	b
b	b	b	b	a	b	b	b	b	b
b	b	b	b	-	b	b	b	b	b
b	b	b	b	a	b	b	b	b	b
b	b	b	a	a	b	b	b	b	b
b	b	b	a	-	b	b	b	b	b
b	b	a	a	a	b	b	b	b	b
b	b	a	a	a	b	b	b	b	b
b	b	a	a	a	b	b	b	b	b
b	b	-	a	a	b	b	b	b	b

b	b	a	a	a	b	b	b	b	b
b	b	a	a	a	b	b	b	b	b
b	a	a	a	a	b	b	b	b	b
b	b	a	-	a	b	b	b	b	b
b	b	a	a	a	b	b	b	b	b
b	b	a	a	-	b	b	b	b	b
b	b	a	a	a	b	b	b	b	b
b	b	a	a	a	b	b	b	b	b
b	b	a	a	a	b	b	b	b	b
b	b	a	a	a	b	b	b	b	b
b	b	a	a	a	b	b	b	b	b
b	b	a	a	a	b	b	b	b	b
b	a	a	a	a	b	b	b	b	b
b	-	a	a	a	b	b	b	b	b
b	a	a	a	a	b	-	b	b	b
b	a	a	a	a	b	b	b	b	b
b	a	a	a	a	b	b	b	b	b
-	a	a	a	a	b	b	b	b	b
b	a	a	b	a	b	b	b	b	b
b	a	a	b	-	b	b	b	b	b
b	a	a	b	a	b	b	b	b	b
b	a	a	b	a	b	b	b	b	b
b	a	a	b	-	b	b	b	b	b
b	a	a	b	a	b	b	b	b	b
b	a	a	b	a	b	b	b	b	b
b	a	a	b	a	b	b	b	b	b
b	a	a	b	-	b	b	b	b	b
b	a	a	b	a	b	b	b	b	b
b	a	a	b	a	b	b	b	b	b
b	a	a	b	a	b	-	b	b	b
b	a	a	b	a	b	b	b	b	b
b	a	a	b	a	b	b	b	b	b
b	a	a	-	a	b	b	b	b	b
b	a	a	b	a	b	-	b	b	b
b	a	a	b	a	b	b	b	b	b
b	a	a	b	a	b	b	b	b	b
b	a	a	b	a	b	b	b	b	b
b	a	a	b	a	b	b	b	b	b
b	a	a	b	a	b	b	b	b	b
b	b	a	b	a	b	b	b	a	a
b	b	-	b	a	b	b	b	a	a
a	b	a	b	a	b	b	-	a	a
b	b	a	b	a	b	b	b	a	a
b	b	a	b	a	b	b	b	a	a
b	b	a	b	a	b	b	b	a	a
a	b	a	b	a	b	b	b	a	a
a	b	a	b	a	b	-	b	a	a

a	b	a	b	a	b	b	b	a	a
-	b	a	b	-	b	b	b	a	a
a	b	a	b	a	-	b	b	a	a
a	b	a	b	a	b	b	b	a	a
a	-	a	b	a	b	b	b	a	a
a	-	a	b	a	b	b	b	a	a
a	b	a	b	a	b	b	b	a	a
a	b	a	b	-	b	b	b	a	a
a	b	a	b	a	b	b	b	a	a
a	b	a	b	a	b	b	b	a	a
a	b	a	b	a	-	b	b	a	b
a	b	a	b	a	b	b	b	a	b
a	b	a	b	a	b	b	b	a	b
a	-	a	a	-	b	b	b	a	b
-	b	a	a	b	b	b	b	a	b
a	b	a	a	b	b	b	b	a	b
a	b	a	a	b	b	b	b	a	b
a	b	a	a	b	b	b	b	a	b
a	b	a	a	b	b	b	b	a	b
a	a	a	a	b	b	b	-	a	b
a	b	a	a	b	b	b	b	a	b
a	b	a	a	b	b	b	b	a	b
a	b	a	a	b	b	b	b	a	b
a	-	a	a	-	b	b	b	a	b
a	b	a	a	b	b	a	b	a	b
a	b	a	a	-	b	a	b	a	b
a	b	a	a	b	b	-	b	a	b
a	b	a	a	b	-	a	b	a	b
-	b	a	a	b	b	a	b	a	b
a	b	a	a	b	b	a	b	a	b
a	b	a	a	b	b	a	b	a	b
a	-	a	a	b	b	a	b	a	b
a	b	a	a	b	b	a	b	a	b
a	b	a	a	b	b	a	b	a	b
a	b	a	a	b	b	a	b	a	b
a	b	a	a	b	b	a	b	a	b
a	b	a	a	b	b	a	b	a	b
a	b	a	a	b	b	a	b	a	b
a	b	a	a	b	b	a	b	a	b
a	b	a	a	b	b	a	b	a	b
a	b	a	a	b	b	a	b	a	b
a	-	a	a	-	b	a	b	a	b
a	b	a	-	b	b	a	b	a	b
a	b	a	a	b	b	a	b	a	b
a	b	a	a	b	b	a	b	a	b
a	b	a	a	b	b	a	b	a	b
a	b	a	a	-	-	a	b	a	b

a	b	a	a	b	b	a	b	a	b
a	b	a	a	b	b	a	b	a	b
a	b	-	a	b	b	a	b	a	b
a	b	a	a	b	b	a	b	a	b
a	b	a	a	b	b	a	b	a	b
b	b	a	a	b	b	a	b	a	b
b	b	a	a	b	b	a	b	a	b
b	b	a	b	b	b	a	b	a	b
b	b	a	b	b	b	a	b	a	b
b	b	a	b	b	b	a	b	a	a
b	b	a	b	b	b	a	b	a	a
b	b	a	b	b	b	a	b	a	a
b	b	a	b	b	b	a	b	a	a
b	a	a	b	b	b	a	b	a	a
b	a	a	b	b	b	a	b	b	a
b	a	a	b	b	b	a	b	b	a
b	a	a	b	b	b	a	b	b	a
b	a	a	b	b	b	a	b	b	a
b	a	a	b	b	b	a	b	b	b
b	b	a	b	b	b	a	b	b	a
b	a	a	b	b	b	a	b	b	a
b	a	a	b	b	b	a	b	b	a
b	a	a	b	b	a	a	b	b	a
b	a	a	b	b	a	a	b	b	a
b	a	a	b	b	a	a	b	b	a
b	a	a	b	b	a	a	b	b	a
b	a	a	b	b	a	a	b	b	a
b	a	a	b	b	a	a	b	b	b
b	a	a	b	-	a	a	b	b	b
b	a	a	b	b	a	a	b	b	b
b	a	a	b	b	a	a	b	b	b
b	a	a	b	b	a	a	b	b	b
b	a	a	b	b	a	a	b	b	b
-	a	a	b	b	a	a	b	b	b
b	a	a	b	b	a	a	b	b	b
b	a	a	b	b	a	a	b	b	b
b	a	a	b	b	-	a	b	b	b
b	a	a	b	b	a	a	b	b	b
b	a	a	a	b	a	a	b	b	b
b	a	a	a	b	a	a	b	b	b
b	a	a	a	b	a	a	b	b	b
b	a	a	a	-	a	a	b	b	b
b	a	a	a	b	a	a	b	b	b
b	a	a	a	b	a	a	b	b	b
b	a	a	a	b	a	a	b	b	b
b	a	a	a	b	a	a	b	a	b
b	a	a	a	b	a	a	b	a	b

b	a	a	a	b	a	a	b	a	b
b	a	a	a	b	a	-	b	a	b
b	a	a	a	b	a	-	b	a	b
b	a	a	a	b	a	a	b	a	b
b	a	a	a	b	a	a	b	a	b
b	a	a	a	b	a	-	b	a	b
b	a	a	a	b	a	a	b	a	b
b	a	a	a	b	a	a	b	a	b
b	a	a	a	b	a	a	b	a	b
b	a	a	a	b	a	a	b	a	b
b	a	a	a	b	a	a	b	a	b
b	b	a	a	b	a	a	b	a	b
a	-	b	a	b	a	b	b	a	b
a	a	b	a	b	a	b	b	a	a
a	a	b	a	b	a	b	b	a	a
a	a	b	a	b	a	b	b	a	a
a	a	b	a	b	a	b	b	a	a
a	a	b	a	b	a	b	b	a	a
a	a	b	a	b	a	b	b	a	a
a	a	b	a	b	a	b	b	a	a
a	a	b	-	b	a	b	b	a	a
a	a	a	a	b	a	b	b	a	a
a	b	a	a	b	a	b	b	a	a
a	a	a	a	b	a	b	b	a	a
a	a	a	a	b	a	b	b	a	a
a	a	a	a	a	a	b	b	a	a
a	a	a	a	a	a	b	b	a	a
a	a	a	a	a	a	b	b	a	a
a	a	a	a	a	-	b	b	a	a
b	a	a	a	a	a	a	b	a	a
b	a	a	a	a	a	a	b	a	a
b	b	a	b	a	a	a	a	a	b
b	b	a	b	a	a	a	a	a	b
b	b	a	b	a	a	a	a	a	b
a	b	a	b	a	a	a	a	a	b
a	-	a	b	a	a	a	a	a	b
a	b	a	b	a	a	a	a	b	b
a	b	a	b	a	a	a	a	b	b
a	b	a	b	a	a	a	a	b	b
a	b	a	b	a	a	a	a	b	b
a	b	a	b	a	-	a	a	b	b
a	b	a	b	-	a	a	a	b	b
a	b	a	b	-	a	a	a	b	b
a	b	a	b	a	a	a	a	b	b
a	b	a	b	a	a	a	a	b	b
a	b	a	b	a	a	a	a	b	b
a	b	a	b	a	a	a	a	a	b

a	b	a	b	a	a	a	a	a	b
a	b	a	a	a	a	a	a	a	b
a	b	a	b	a	a	a	a	a	b
a	b	a	b	-	b	a	a	a	b
a	b	a	b	a	b	a	a	a	b
a	b	a	-	a	-	a	a	a	b
a	b	a	b	a	b	a	a	a	b
a	b	a	b	a	b	a	a	a	b
a	b	a	b	a	b	a	a	a	b
a	a	a	b	a	b	a	a	a	b
a	a	a	b	a	b	a	a	a	b
a	a	a	b	a	b	a	a	a	b
a	a	a	b	a	b	a	a	a	b
-	a	a	b	a	b	a	a	a	b
b	a	a	b	a	b	a	a	a	b
-	a	a	b	-	b	a	a	a	b
b	a	a	b	a	b	a	a	a	b
b	a	a	b	a	b	a	a	a	b
b	a	a	b	a	b	a	a	a	b
b	a	a	b	a	b	a	a	a	b
b	a	a	b	a	b	a	a	a	b
b	a	a	b	a	b	b	a	a	b
b	a	a	b	a	b	b	a	a	b
b	a	a	b	-	b	b	a	a	b
b	a	a	b	a	b	b	a	a	b
b	a	a	b	a	b	b	a	a	b
b	a	a	-	a	b	b	a	a	b
b	a	a	a	a	b	b	a	a	a
b	a	a	a	a	b	b	a	a	a
b	a	a	a	-	b	b	a	a	a
b	a	-	a	a	b	b	a	a	-
b	a	a	a	a	b	b	a	a	a
b	a	a	a	a	-	b	a	a	a
b	a	a	a	a	b	b	a	a	a
b	a	a	a	a	b	b	a	a	a
b	a	a	a	-	b	b	a	a	a
b	a	a	a	a	b	b	a	a	a
b	a	a	a	a	b	b	a	a	a
b	a	a	a	a	b	b	a	a	a
b	a	a	a	a	b	-	a	a	a
b	a	a	a	a	b	b	a	a	a
b	b	a	a	-	b	b	a	a	a
a	a	a	a	a	b	b	a	a	a
b	a	a	a	a	b	b	a	a	a
b	a	a	a	a	b	b	a	a	a
-	a	a	a	a	b	b	a	a	a
b	a	b	a	a	b	b	a	a	a

b	a	b	a	a	b	b	a	a	a
b	-	b	a	a	b	b	a	a	a
b	a	b	a	a	b	b	a	a	a
b	a	b	a	a	b	b	a	a	a
b	a	b	a	a	b	b	a	a	a
b	a	b	a	a	b	b	a	a	a
b	-	b	a	a	b	b	a	a	a
b	a	b	a	a	b	b	a	a	a
b	a	b	a	a	b	b	a	a	a
b	a	b	a	a	b	b	a	a	a
b	a	b	a	a	-	b	a	a	a
b	a	b	a	a	b	-	a	a	a
b	a	b	a	a	b	b	a	a	a
b	a	b	a	a	b	b	a	a	a
b	a	b	a	a	b	b	a	a	a
b	a	b	a	a	b	b	a	a	a
b	a	b	a	a	b	b	a	a	a
b	a	b	a	a	b	b	a	a	a
b	a	b	a	a	b	b	a	a	a
b	a	b	a	a	b	b	a	a	a
b	a	b	a	a	b	b	a	a	a
b	a	b	a	a	b	b	a	a	a
b	a	b	a	a	b	b	a	a	a
b	b	b	-	b	b	b	a	a	-
b	b	b	a	b	b	b	a	a	a
b	a	b	a	b	-	b	a	a	a
b	a	b	a	b	b	b	a	a	a
b	a	b	a	b	b	b	a	a	a
b	a	b	a	b	b	b	a	a	a
b	a	b	a	b	b	b	a	a	a
b	a	b	a	b	b	b	a	a	a
b	a	b	a	b	b	b	a	a	a
b	a	b	a	b	b	b	a	a	a
b	a	b	a	b	b	b	a	a	a
b	a	b	a	b	b	-	a	a	a
b	a	b	a	b	b	b	a	a	a
b	a	-	a	b	b	b	a	a	a
b	a	b	a	b	b	b	a	a	a
a	b	b	b	b	b	a	a	b	a
a	b	b	b	b	b	a	a	b	a
a	-	b	b	b	b	a	a	b	a
a	b	b	b	b	b	a	a	b	a
a	b	b	b	b	b	a	a	b	a
a	b	b	b	b	b	a	a	b	-
a	b	b	b	-	b	a	a	b	a
a	b	b	b	b	b	a	a	b	a
a	b	b	b	b	b	a	a	a	a

a	a	b	b	b	b	a	a	a	a
a	a	b	b	b	b	a	a	a	a
a	a	b	b	b	b	-	a	a	a
a	a	b	b	b	b	a	a	a	a
a	a	b	b	b	b	a	a	a	a
a	a	b	b	b	b	a	a	a	a
a	a	b	b	b	b	a	a	a	a
a	a	b	b	b	-	a	a	a	a
a	a	b	b	b	b	a	a	a	a
a	a	b	b	b	b	a	a	a	a
b	-	b	b	b	b	a	a	a	a
b	a	b	b	b	b	a	a	a	a
b	a	b	b	b	b	a	a	a	a
b	a	b	-	-	b	a	a	a	a
b	a	b	b	b	b	a	a	a	a
b	a	b	b	b	b	a	a	a	a
b	a	b	b	b	b	a	a	a	a
b	a	b	b	b	b	a	a	a	a
b	-	b	b	b	b	a	a	a	a
b	a	b	b	b	b	a	a	a	a
b	a	b	b	b	b	a	a	a	a
b	a	b	b	b	b	a	a	a	a
b	a	b	b	b	b	a	a	a	a
b	a	b	b	b	b	a	a	a	a
b	a	b	b	b	b	a	a	a	a
b	a	b	b	a	b	a	a	a	a
b	a	b	b	a	b	a	a	a	a
b	a	b	-	a	b	a	a	a	a
b	a	b	a	a	b	a	a	a	a
b	a	b	a	a	b	a	a	b	a
b	a	b	a	a	b	-	a	b	a
b	a	b	a	a	b	a	a	b	a
b	a	b	a	a	b	a	a	b	a
b	a	b	a	a	b	a	b	b	a
b	a	a	a	a	b	a	b	b	a
b	b	b	a	a	b	a	a	b	a
b	a	b	a	a	b	b	-	b	a
b	a	b	a	-	b	b	b	b	a
b	a	b	a	a	b	b	b	b	a
b	a	b	a	a	b	b	b	b	a
-	a	b	a	a	b	b	b	b	a
b	a	b	a	a	b	b	b	b	a
b	a	b	a	a	b	b	b	b	a
b	a	b	-	a	b	b	b	b	a
b	a	b	a	a	b	b	b	b	-
b	a	b	a	a	b	b	b	b	a
b	a	b	a	a	b	b	b	b	a

b	a	b	a	-	b	b	b	b	a
b	a	b	a	a	-	b	b	b	a
b	a	b	a	a	b	b	b	b	a
b	a	b	a	a	b	b	b	b	a
b	a	-	a	a	b	b	b	b	a
b	a	-	a	a	b	b	b	b	a
b	a	-	a	a	b	b	b	b	a
b	a	b	a	a	b	b	b	b	a
b	a	b	a	a	b	b	b	b	a
b	a	b	a	a	b	b	b	b	a
b	a	b	a	a	b	b	b	b	a
b	a	b	a	a	b	b	b	b	a
b	a	b	-	a	b	b	b	b	a
b	a	b	a	a	b	b	b	b	a
b	a	b	a	a	b	b	b	b	a
b	a	b	a	a	b	-	b	b	a
b	a	b	a	a	b	b	b	a	a
b	a	b	a	a	b	b	b	a	a
b	a	b	a	a	b	b	b	a	a
b	a	b	a	a	b	b	b	a	a
b	a	b	a	a	b	b	b	a	a
b	a	b	a	a	b	b	b	a	a
b	a	b	a	a	b	b	a	a	a
b	-	b	a	a	b	b	a	a	a
b	a	b	a	a	b	b	a	a	a
b	a	b	a	a	b	-	a	a	a
b	a	b	a	a	b	b	a	a	a
b	a	-	a	a	-	-	a	a	a
b	a	b	a	a	b	b	a	a	a
b	a	b	a	a	b	b	a	a	a
b	a	b	a	a	b	b	a	a	a
b	a	b	a	a	-	b	a	a	a
b	a	b	a	-	b	b	a	a	a
b	a	b	a	-	b	b	a	a	a
b	a	b	a	a	b	b	a	a	a
b	-	b	a	a	b	-	a	a	a
-	b	b	a	a	b	b	a	a	a
b	b	b	a	a	b	b	a	a	a
b	b	b	a	a	b	b	a	a	a
b	b	b	a	a	b	b	a	a	a
a	a	a	a	b	b	a	a	a	a
a	a	a	a	b	b	a	a	a	a
a	a	a	a	b	b	a	a	a	a
a	a	a	a	b	b	a	a	a	a
a	a	a	a	b	b	a	a	a	a
a	a	a	a	b	b	a	a	a	a
a	a	a	a	b	b	a	a	a	a

a	a	a	a	-	b	a	a	a	a
a	a	a	a	b	b	a	a	a	a
a	a	a	a	b	b	a	a	a	a
a	a	a	a	b	b	a	a	a	a
a	a	a	a	b	b	a	a	a	a
a	-	a	a	b	b	a	a	-	a
a	a	a	a	b	b	a	a	a	a
a	a	a	a	b	b	-	a	a	a
a	a	a	a	b	b	a	a	a	a
a	a	a	a	b	b	a	a	a	a
a	a	a	a	b	b	a	a	a	a
a	a	a	a	b	b	a	a	a	a
a	-	a	a	b	b	a	a	a	a
-	a	a	-	b	b	a	a	a	a
a	a	a	a	-	b	a	a	a	a
a	a	a	a	b	b	a	a	a	a
a	a	a	a	b	b	a	a	a	a
a	a	a	a	b	b	a	a	a	a
a	a	a	a	b	b	-	a	a	a
a	a	a	a	b	b	a	a	a	a
a	a	a	a	b	b	a	a	a	a
a	a	a	a	b	b	a	a	a	a
a	a	a	a	b	b	a	a	a	a
a	a	a	a	b	b	a	a	a	b
a	a	a	a	b	b	a	a	a	b
a	a	a	a	b	b	a	a	b	b
a	a	a	a	b	b	a	a	b	b
a	a	a	a	b	b	a	a	b	b
a	a	a	a	b	b	a	a	b	b
a	-	a	a	b	b	a	a	b	b
a	a	a	a	b	b	a	a	b	b
a	a	a	a	-	b	a	a	b	b
a	a	a	a	b	b	a	a	b	b
a	a	a	a	b	b	a	a	b	b
a	a	a	a	b	b	a	a	b	-
a	a	a	-	b	b	a	a	b	b
a	a	a	a	b	b	a	a	b	b
b	-	a	a	b	b	a	a	b	b
a	-	a	a	b	b	a	a	b	b
-	b	a	a	b	b	a	a	b	b
a	a	a	a	b	b	-	a	b	b
a	a	a	a	b	b	a	a	b	b
a	a	a	a	b	b	a	a	b	b
a	a	a	a	b	b	a	a	b	b
a	a	a	a	b	b	a	a	b	b
a	a	a	a	b	b	a	a	b	b
a	a	a	a	b	b	a	a	b	b

a	a	a	a	b	b	a	a	b	b
a	a	-	a	b	b	a	a	b	b
a	a	a	a	b	b	a	a	b	b
a	a	a	a	b	a	a	a	b	-
b	a	a	a	b	a	a	a	b	b
b	a	a	a	b	b	-	a	b	a
b	a	a	a	b	a	-	a	b	b
b	a	a	a	b	b	a	a	b	b
b	a	a	a	b	b	a	a	b	b
b	b	a	a	b	b	a	a	b	b
b	b	a	a	b	b	a	a	b	b
b	a	a	a	b	b	a	a	b	b
b	a	a	a	b	b	a	a	b	b
b	a	a	a	b	b	a	a	b	b
b	a	a	a	b	b	a	b	b	b
b	a	a	a	b	b	a	b	b	b
b	a	a	a	b	b	a	b	b	b
b	a	a	a	b	b	a	b	b	b
a	-	a	a	b	b	a	b	b	b
a	a	a	a	b	b	a	b	b	b
a	a	a	a	b	b	a	b	b	b
a	-	a	a	b	b	a	b	b	b
a	a	a	a	b	b	a	b	b	b
a	a	a	a	b	b	a	b	b	b
a	a	a	a	b	b	a	b	b	b
a	a	a	a	b	b	-	b	b	b
a	a	a	a	b	b	a	b	b	a
a	a	a	a	b	b	a	b	b	a
a	a	a	a	-	b	a	b	b	a
a	a	a	a	b	b	a	b	b	a
a	a	a	a	b	b	a	b	b	a
a	b	a	a	b	b	a	b	b	a
a	b	a	a	b	b	a	b	b	a
a	b	a	a	b	b	a	b	b	a
a	b	a	a	b	b	a	b	b	a
a	b	a	a	b	b	a	b	b	a
a	a	a	a	b	b	a	b	b	a
a	a	a	a	b	b	a	b	b	a
a	a	a	a	b	b	a	b	b	a
a	a	a	a	b	b	a	b	b	a
a	a	a	a	b	b	a	b	b	a
a	a	a	a	b	b	a	b	b	a
a	a	a	a	-	b	-	b	b	a
a	a	a	a	b	-	b	b	b	a
a	a	a	a	b	b	b	b	b	a
a	a	a	a	b	b	b	b	b	a
a	a	a	a	b	b	b	b	b	a

a	a	a	a	b	b	b	a	b	a
a	a	a	a	b	b	b	a	b	a
a	a	a	a	b	b	b	a	b	a
a	a	a	a	b	b	b	a	b	a
a	a	a	a	b	b	b	a	b	a
a	a	a	-	b	b	b	a	b	a
a	a	a	a	b	b	b	a	b	a
a	a	a	a	b	b	b	a	b	a
a	a	a	-	-	b	b	a	b	a
a	a	a	a	b	b	b	a	b	a
a	a	a	a	b	b	b	a	b	a
a	a	a	a	b	b	b	a	b	a
a	a	a	a	b	b	b	a	b	a
a	a	a	a	b	-	-	a	b	a
a	a	a	a	b	b	b	a	b	a
a	a	a	a	b	-	b	a	b	a
a	a	a	a	b	b	b	a	b	a
a	a	a	a	b	b	b	a	b	a
a	a	a	a	b	b	b	a	b	a
a	a	a	a	b	a	b	a	b	a
a	a	a	a	b	a	b	a	b	a
a	a	a	a	b	a	b	a	b	a
a	a	a	a	b	a	b	a	b	a
a	a	a	a	b	a	b	a	b	a
a	a	a	a	b	a	b	a	b	a
a	a	a	a	b	a	b	a	b	a
a	a	a	a	b	a	-	a	b	a
a	a	a	a	b	a	b	a	b	a
a	a	a	a	a	a	b	a	b	a
a	a	a	a	a	a	b	a	b	a
a	a	a	a	a	a	b	a	b	a
a	a	a	a	a	a	b	a	b	a
a	a	a	a	a	a	b	a	a	a
a	a	a	a	a	a	b	a	a	a
a	b	a	a	-	a	b	a	a	a
a	-	a	a	a	a	b	a	a	a
a	b	a	a	a	a	b	a	a	a
a	b	-	a	a	a	b	a	a	a
a	b	a	a	a	a	b	a	a	a
a	b	a	a	a	a	b	a	a	a
a	b	a	a	a	a	b	a	a	a
a	b	a	a	a	a	b	a	a	a
a	b	-	a	a	a	b	a	a	a
a	b	a	a	a	a	b	a	a	a
a	b	a	a	a	a	b	a	a	a
a	b	a	a	a	a	b	a	a	a
a	b	a	a	a	a	b	a	a	a

a	b	a	a	a	a	b	a	a	a
a	-	a	a	a	a	b	a	a	a
a	b	a	a	a	a	b	a	a	a
a	a	a	a	a	a	b	a	a	a
a	b	a	a	a	a	-	a	a	a
a	b	a	a	a	a	b	a	a	a
a	b	a	a	a	a	a	a	a	a
a	-	a	a	a	a	b	a	a	a
a	b	a	a	a	a	b	a	a	a
a	b	a	-	a	a	b	a	a	a
a	b	a	a	a	a	b	a	a	a
a	a	a	a	a	a	b	a	a	a
a	a	a	a	a	a	b	a	a	a
a	a	a	a	a	a	b	a	a	a
a	-	a	a	a	a	b	a	a	a
a	-	a	a	a	a	b	a	a	a
a	a	a	a	a	-	b	a	a	a
a	a	a	a	a	a	b	a	a	a
a	a	a	a	a	a	b	a	a	a
a	a	a	a	a	a	b	a	a	a
a	a	a	a	a	a	b	a	a	a
a	a	a	a	a	a	b	a	a	a
a	a	a	a	a	a	b	a	a	a
b	-	a	a	-	a	a	a	a	a
b	a	b	a	a	a	b	a	a	a
b	a	a	a	a	a	a	a	a	a
-	b	a	a	a	a	a	a	a	a
b	b	a	a	a	a	a	a	a	a
b	b	a	a	a	a	a	a	a	a
b	-	a	a	a	a	a	a	a	a
b	-	a	a	a	b	a	a	a	a
b	b	a	a	a	b	a	a	a	a
b	b	a	a	a	b	a	a	a	a
b	b	a	a	-	b	a	a	a	a
b	b	a	a	a	b	a	a	a	a
b	b	a	a	a	b	a	a	a	a
a	b	a	a	a	b	a	a	a	a
a	b	a	a	a	b	a	a	a	a
a	b	a	a	a	b	a	a	a	a
a	b	a	a	a	b	a	a	a	a
a	-	a	a	a	b	a	a	a	a
a	b	a	a	a	b	a	a	a	a
a	a	a	a	a	b	a	a	a	a
a	b	a	a	a	b	a	a	a	a
a	a	a	a	a	b	a	a	a	a
a	a	a	a	a	b	a	a	a	a
-	a	a	a	a	b	a	a	a	a

a	a	a	a	a	b	a	a	a	a
a	a	a	a	a	b	a	a	a	a
a	a	a	a	a	b	a	a	a	a
a	a	a	b	a	b	a	a	a	a
a	a	a	b	a	b	a	a	a	a
a	a	a	b	a	b	a	a	a	a
a	a	a	b	a	b	a	a	a	a
b	a	a	b	a	b	a	a	a	b
b	a	a	b	a	b	a	a	a	b
b	a	a	-	a	b	a	a	a	b
b	a	a	b	a	b	a	a	a	-
b	a	b	b	a	a	b	b	a	b
b	a	b	b	-	a	b	b	a	b
b	a	b	b	a	a	b	b	a	b
b	a	b	b	a	a	b	b	a	b
b	a	b	b	a	a	b	b	a	b
b	a	b	b	a	a	b	b	a	b
b	a	b	b	a	a	b	b	a	b
b	a	-	b	a	a	b	b	a	b
b	a	b	b	a	a	b	b	a	b
b	a	b	b	a	-	b	b	a	b
b	a	b	b	a	a	b	b	a	b
b	a	b	b	a	a	b	b	a	b
b	a	b	b	a	a	-	b	a	b
b	a	b	b	a	a	b	b	a	b
b	-	b	b	a	a	b	b	a	b
b	a	b	b	a	a	b	b	a	b
b	a	b	b	a	a	b	b	a	-
b	a	b	b	a	a	b	b	a	b
b	a	b	b	a	a	b	b	a	b
b	a	b	b	a	a	b	b	a	b
b	a	b	b	a	a	b	b	a	b
b	a	b	b	a	a	b	b	a	b
b	a	b	b	a	a	b	b	a	b
b	a	b	b	a	a	b	b	a	b
b	a	b	-	a	a	b	b	a	b
-	a	b	b	a	a	b	-	a	b
b	a	b	b	a	a	b	b	a	b
b	a	b	b	a	-	b	b	a	b
b	a	b	b	a	a	b	b	a	b
b	a	-	b	a	a	b	b	a	b
b	b	b	b	a	a	b	b	a	b
b	a	b	b	a	a	b	b	a	b
b	b	b	b	a	a	b	b	a	b
b	a	b	b	a	a	-	b	a	b
b	-	b	b	a	a	b	b	a	b
b	a	b	-	a	a	b	b	a	b

b	a	b	b	a	a	b	b	a	b
b	a	b	b	a	a	b	b	a	b
b	a	b	b	a	a	b	b	a	b
b	a	b	b	a	a	b	b	a	b
b	-	b	b	a	a	b	b	a	b
b	a	b	b	b	a	b	b	a	b
b	a	b	b	b	a	b	b	a	b
b	a	b	b	b	a	b	b	a	-
b	-	b	b	b	a	b	b	a	b
b	a	b	b	b	a	b	b	a	-
b	a	b	b	b	a	b	b	a	b
b	b	b	b	b	a	-	b	a	b
b	a	b	b	b	a	b	b	a	b
b	a	b	b	b	a	b	b	a	b
b	a	b	b	b	a	b	b	a	b
b	a	b	b	b	a	b	b	a	b
b	a	-	b	b	a	b	b	a	a
b	a	b	b	b	-	b	b	a	a
b	a	b	b	b	a	b	b	a	a
b	a	b	b	b	a	b	b	a	a
b	a	b	b	b	a	b	b	a	a
b	a	b	b	b	a	b	b	a	a
b	a	b	b	b	a	b	b	a	a
b	a	b	b	b	a	b	b	a	a
b	a	b	b	b	a	b	b	a	a
b	a	b	b	b	a	b	b	a	a
b	a	b	b	b	a	b	b	a	a
b	a	b	b	b	a	b	b	a	a
b	a	b	b	b	a	b	b	a	a
-	a	b	b	b	a	b	b	a	a
b	a	b	b	b	a	b	b	a	a
b	a	b	b	b	a	b	b	a	a
b	a	a	b	b	a	b	-	a	a
b	-	a	b	b	a	b	b	a	a
b	b	a	b	-	a	b	b	a	a
b	b	a	b	b	a	b	b	a	a
b	b	a	b	b	a	b	b	a	a
b	b	a	b	b	a	b	b	a	a
b	b	a	b	b	a	b	b	a	a
b	b	a	b	b	a	b	b	a	a
b	b	a	b	b	a	b	b	a	a
b	b	a	a	b	a	b	b	a	a
b	-	a	a	b	a	b	b	a	a
b	b	a	a	b	a	b	a	a	-
b	b	a	a	b	a	b	a	a	a
b	b	a	a	b	a	b	a	a	a
b	b	a	a	b	a	b	a	a	a

b	b	a	a	b	a	b	a	a	a
b	b	a	a	b	a	b	a	a	a
b	b	a	a	b	a	b	a	a	a
b	-	a	a	b	a	b	a	a	a
b	b	a	a	b	a	b	a	a	a
b	b	a	a	b	-	b	a	a	a
b	b	a	a	b	-	b	a	a	a
b	b	a	a	b	-	b	a	a	a
b	b	a	a	b	a	b	a	a	a
b	a	a	a	b	a	b	a	a	a
b	a	a	a	b	a	b	a	a	a
b	a	a	a	b	a	b	a	a	a
b	-	a	a	b	a	b	a	a	a
b	a	a	a	b	a	b	a	a	a
b	a	a	a	b	a	b	a	a	a
b	a	a	a	b	a	b	a	a	a
b	a	a	a	b	a	b	a	a	a
b	a	a	a	b	a	b	a	a	a
b	a	a	a	b	-	b	a	a	a
b	a	a	a	b	-	b	a	a	a
b	a	a	a	b	a	b	a	a	a
a	b	a	a	b	a	b	a	a	a
-	-	a	b	b	a	a	a	a	b
b	b	-	b	b	a	a	a	a	b
b	b	a	b	b	a	-	a	a	b
b	b	a	b	b	a	a	a	a	b
b	b	a	b	b	a	a	a	a	b
b	b	a	b	b	a	a	a	a	b
b	b	-	b	b	a	a	a	a	b
b	b	b	b	b	a	a	a	a	b
b	b	b	b	b	a	a	a	a	b
b	b	b	b	b	a	b	a	a	b
b	b	b	b	b	a	b	a	a	b
b	b	b	b	b	a	b	a	a	b
b	b	b	b	b	a	b	-	a	b
b	b	b	b	b	a	b	a	a	b
b	-	b	-	b	a	b	a	a	b
b	b	b	b	b	a	b	a	a	b
b	b	b	b	b	a	b	a	a	b
b	b	b	b	b	a	b	a	a	b
b	b	b	b	a	a	-	a	a	b
b	b	b	b	a	b	b	a	a	b
a	b	b	b	a	b	b	a	a	b
a	b	b	b	a	-	b	a	a	-
a	b	b	b	a	b	b	a	a	b
a	-	b	b	a	b	b	a	a	b
a	b	b	b	a	b	b	a	a	b

b	b	b	a	a	b	b	a	a	a
b	b	b	a	a	b	b	a	a	-
b	b	b	a	a	b	b	b	a	a
b	b	-	a	a	b	-	a	-	a
b	b	b	a	a	b	b	a	b	a
b	b	b	a	a	b	b	a	b	a
b	b	b	a	-	b	b	a	b	a
b	b	b	a	a	b	b	a	b	a
b	b	b	a	a	b	b	a	b	a
b	b	b	a	a	b	b	a	b	a
b	b	b	-	-	b	b	a	b	a
b	b	b	a	a	b	b	a	b	a
b	b	b	a	a	b	b	a	b	a
b	b	-	a	a	b	b	a	b	a
b	b	b	a	a	b	b	a	b	a
b	b	b	a	a	b	b	a	b	a
b	b	b	a	a	b	b	a	b	a
b	b	b	a	-	b	b	a	b	a
b	b	b	a	a	b	b	a	b	a
b	b	b	a	a	b	b	a	b	a
b	b	b	a	a	b	b	a	b	a
b	b	b	a	a	b	b	a	b	a
b	b	b	a	-	b	b	a	b	a
b	b	b	a	-	b	b	a	b	-
b	b	b	a	a	b	b	a	b	a
b	-	b	a	a	b	b	a	b	a
b	b	b	a	a	b	b	a	b	a
b	b	b	a	a	b	b	a	b	a
b	b	b	a	a	b	b	a	b	a
b	b	b	a	a	b	b	a	b	a
b	b	b	a	a	b	b	a	b	b
b	b	b	a	a	b	-	a	b	b
b	b	b	-	a	b	b	a	b	b
b	b	b	a	a	b	b	a	b	b
b	b	b	a	a	b	b	a	b	b
b	b	b	a	a	b	b	a	b	b
b	b	b	a	a	b	b	a	b	b
b	b	b	a	a	b	b	a	b	b
b	b	b	a	a	b	b	a	b	b
b	b	b	a	a	b	b	a	b	b
b	-	b	a	a	b	b	a	b	b
b	b	b	a	a	b	b	a	b	b
b	b	b	a	a	b	b	a	b	b
b	b	b	a	a	b	b	a	b	b
b	b	b	b	a	b	b	a	b	b
b	b	b	b	a	b	b	a	b	b
b	b	b	b	a	b	b	a	b	b

b	b	b	b	a	b	b	a	b	b
b	b	b	b	-	b	b	a	b	b
b	a	b	b	a	b	b	a	b	b
b	a	b	b	a	b	-	a	b	b
b	a	b	b	a	b	b	a	b	b
b	a	b	b	-	b	b	a	b	b
b	a	b	b	a	b	b	a	b	b
b	-	b	b	a	b	b	a	b	b
b	a	b	b	a	b	b	a	b	b
b	a	b	b	a	b	b	a	b	b
b	a	b	b	a	b	b	b	b	b
b	-	b	b	a	b	b	b	b	b
b	a	b	b	a	b	b	b	b	b
b	a	b	b	a	b	b	b	b	b
b	-	b	b	a	b	b	b	b	b
b	a	b	b	a	b	b	b	b	b
b	a	b	b	a	b	b	b	b	b
b	a	b	b	a	b	b	b	b	b
b	a	b	b	a	b	b	b	b	b
a	a	b	b	a	b	b	b	b	b
a	a	b	b	a	b	b	b	b	b
a	a	b	b	a	b	b	b	b	b
a	a	b	b	a	-	-	b	b	b
a	a	b	b	a	b	b	b	b	b
a	a	b	b	a	b	b	b	b	b
a	a	b	b	a	b	b	b	b	b
a	a	b	b	a	b	b	b	b	b
a	a	b	b	a	b	b	b	b	b
a	a	b	b	a	-	-	b	b	b
a	a	b	b	a	b	b	b	b	b
a	a	b	b	a	b	b	b	b	b
a	a	b	b	a	b	b	b	b	b
a	a	b	b	-	b	b	b	b	b
a	a	b	b	a	b	b	b	b	b
a	a	b	b	a	b	b	b	b	b
a	a	b	b	a	b	b	b	b	b
a	a	b	b	a	b	b	b	b	b
a	a	b	b	a	b	b	b	b	-
a	a	b	b	a	b	b	b	b	b
a	a	b	b	a	b	b	b	b	b
a	a	b	b	a	b	b	b	b	b
a	a	b	b	a	b	b	b	b	b
a	a	b	b	a	b	b	b	b	b
a	a	b	b	a	b	b	b	b	b
a	a	b	b	a	b	b	b	b	b
a	a	b	b	a	b	b	b	b	b
a	a	b	b	a	b	b	b	-	b

a	a	b	b	a	b	b	b	b	-
a	a	b	-	a	b	b	b	b	b
a	a	a	a	a	a	a	b	a	b
b	b	a	a	b	b	b	b	a	b
b	b	a	a	b	b	b	b	a	b
b	b	a	a	b	a	b	b	a	b
b	b	a	a	b	a	b	b	a	b
b	b	a	a	b	a	b	b	b	b
b	b	a	a	-	a	b	b	b	b
b	-	a	a	b	a	b	b	b	b
b	b	a	a	b	a	b	b	b	b
b	b	a	a	b	a	b	b	b	a
b	b	a	a	b	a	b	b	b	-
b	-	a	a	b	a	-	b	b	a
b	b	a	a	b	a	b	b	b	a
b	b	a	b	b	a	b	a	b	a
b	b	a	b	b	a	b	a	b	a
b	-	a	b	b	a	b	a	b	a
b	b	a	b	b	a	b	a	b	-
b	b	b	b	b	a	b	a	b	a
b	b	b	b	b	a	b	a	b	a
b	b	b	b	b	a	b	a	b	a
b	b	b	b	b	a	b	a	b	-
b	b	b	b	-	a	b	a	b	a
-	b	b	b	b	a	b	a	b	a
b	b	b	b	b	a	b	a	b	a
b	b	b	b	b	a	b	a	b	a
b	b	b	b	b	a	b	a	b	a
b	b	b	b	b	a	b	a	b	a
b	b	b	b	b	a	b	a	b	a
b	b	b	b	b	a	b	a	b	a
b	b	b	b	b	a	b	a	b	a
b	b	b	b	b	a	b	a	b	a
b	b	b	b	b	a	b	a	a	a
b	b	b	b	b	a	b	a	a	a
b	b	b	b	b	a	-	a	a	a
b	b	b	b	b	a	b	a	a	a
b	b	b	b	b	a	b	a	a	a
b	b	b	b	b	a	b	a	a	a
b	a	b	b	b	a	b	a	a	a
b	a	b	b	b	b	b	a	a	a
b	a	b	b	b	b	b	a	a	a
b	-	b	b	b	b	b	a	a	a
b	a	b	b	-	b	b	a	a	a
b	a	b	b	b	b	b	a	a	a
b	a	b	b	b	b	b	a	b	a
b	a	b	b	b	b	b	a	b	a

b	a	b	b	b	b	b	a	b	a
b	a	b	b	b	b	b	a	b	a
b	a	b	b	b	b	b	a	b	a
a	b	b	a	b	b	b	a	b	a
a	a	a	a	b	a	b	b	a	a
a	a	a	a	b	a	b	b	a	a
a	a	a	a	b	a	b	b	a	a
a	a	a	a	b	a	-	b	a	a
a	a	a	a	b	a	b	b	a	a
a	b	a	a	b	a	b	b	a	b
a	a	a	a	b	a	b	b	a	b
a	a	a	a	b	a	b	b	a	b
a	a	a	a	b	a	b	b	a	b
-	a	a	a	-	b	b	a	b	b
a	a	a	a	a	b	-	a	b	b
a	a	a	a	a	b	b	a	b	b
a	a	a	a	a	b	b	a	b	b
a	a	a	a	a	b	b	a	b	b
a	a	a	a	-	b	b	a	b	b
a	a	a	a	a	b	b	a	b	b
a	a	a	a	a	b	b	a	b	b
-	b	b	a	a	b	a	a	b	b
a	a	b	a	a	b	a	a	b	b
-	a	b	a	a	b	a	a	b	b
a	a	b	a	a	b	a	a	b	b
a	a	b	a	a	b	a	a	b	b
a	a	b	a	a	b	a	a	b	b
a	a	b	a	a	b	a	a	b	b
a	a	-	a	a	b	a	a	b	b
a	a	a	a	a	b	a	a	b	b
a	a	a	a	a	b	a	a	b	b
a	a	a	a	a	b	a	a	b	b
a	a	a	a	a	b	a	a	b	b
a	a	a	a	a	b	a	a	a	b
a	a	a	a	a	b	-	a	a	b
a	a	a	a	a	b	a	a	a	b
a	a	a	a	a	b	a	a	a	b
a	a	a	a	a	b	a	a	a	b
-	a	a	a	a	b	a	a	a	b
a	a	a	a	a	b	a	a	a	b
a	a	a	b	a	b	b	a	a	a
a	a	a	b	a	b	b	a	a	a
a	a	a	b	a	b	b	a	a	a
a	a	a	b	a	b	b	a	a	a
b	a	a	b	a	b	b	a	a	a
b	b	b	b	b	-	a	a	b	a
b	b	b	b	b	a	a	a	b	a

b	b	b	b	b	-	a	a	b	a
b	b	b	b	b	a	a	a	b	a
b	a	b	b	b	a	b	a	b	a
b	b	b	b	b	a	b	a	b	a
b	b	b	b	b	a	b	a	b	a
b	b	b	b	b	a	b	a	b	a
b	b	b	b	b	a	b	a	b	a
b	b	a	b	b	a	b	a	b	a
b	b	a	b	b	a	b	a	b	a
b	b	a	b	b	a	b	a	b	a
b	b	a	b	b	a	b	a	b	a
b	b	a	b	b	a	b	a	b	a
-	b	a	b	b	a	-	a	b	a
b	b	a	b	b	a	b	b	b	a
b	b	a	b	b	a	b	b	b	a
b	b	a	b	b	a	b	b	b	a
b	b	a	b	b	a	b	b	b	a
b	b	a	b	b	a	b	b	b	a
b	b	a	b	b	a	b	b	b	a
b	b	a	b	b	a	b	b	b	a
b	b	a	b	b	-	b	b	b	a
b	b	a	b	b	a	b	b	b	a
b	b	a	b	b	a	b	b	b	a
b	b	-	b	b	a	b	b	b	a
b	a	a	b	b	a	b	b	b	a
b	a	a	b	b	a	b	b	b	a
b	a	a	b	b	a	b	b	b	a
b	a	a	b	b	a	b	b	b	a
b	a	a	b	b	a	b	b	-	a
b	a	a	b	b	a	b	b	b	a
b	a	a	b	b	a	b	b	b	a
b	a	a	b	b	a	b	b	b	a
b	b	a	b	b	-	b	b	b	a
b	a	a	b	b	b	b	b	b	a
b	a	a	b	b	b	b	b	b	a
b	a	a	b	b	b	b	b	b	a
b	b	a	b	b	b	b	b	b	a
b	a	a	b	b	b	b	b	b	a
b	a	a	b	b	b	b	b	b	a
b	a	a	b	a	b	b	b	b	a
b	a	a	b	a	b	-	b	b	a
b	-	a	b	a	b	b	b	b	a
b	a	a	b	a	b	b	b	b	a
b	a	a	b	a	b	b	b	b	a
b	a	a	b	a	b	b	b	b	a
b	a	a	b	a	b	b	b	b	a
b	a	a	b	a	b	b	b	b	a

b	a	a	b	a	b	b	b	b	a
b	a	a	b	a	b	b	b	b	a
b	a	a	b	a	b	b	b	b	a
b	a	a	b	a	b	b	-	b	a
b	a	a	b	a	b	b	b	b	a
b	a	a	b	a	b	b	b	b	a
b	a	a	b	a	b	b	b	b	a
b	a	a	b	a	b	b	b	b	a
b	a	a	b	a	b	b	b	b	a
b	a	a	b	a	b	b	b	b	a
b	a	a	b	a	b	b	b	b	a
b	a	a	b	a	b	b	b	b	a
b	a	a	b	a	b	b	b	b	a
b	a	a	b	a	b	b	b	b	a
b	a	a	b	a	b	b	b	b	a
b	a	a	b	-	b	b	b	b	b
b	a	a	b	a	b	b	b	b	b
b	a	a	b	a	b	b	b	b	b
b	a	a	b	a	b	b	b	b	b
b	a	a	-	a	b	b	b	b	b
b	a	a	b	a	b	b	a	b	b
b	a	a	b	a	b	b	a	b	b
b	a	a	b	-	b	b	a	b	b
b	-	a	b	a	b	b	a	b	b
b	a	a	b	a	b	b	a	b	b
b	a	a	b	a	b	b	a	b	b
b	a	a	b	b	b	b	b	b	b
b	a	a	b	b	b	b	b	b	b
-	a	a	b	b	b	b	b	b	b
b	a	a	b	b	b	b	b	b	b
b	a	a	b	b	b	b	b	b	b
b	a	a	b	b	b	b	b	b	b
b	-	a	b	b	b	b	b	b	b
b	a	a	b	b	b	a	b	b	b
b	a	a	a	b	b	a	b	b	b
b	a	b	a	b	b	a	b	b	b
b	a	b	a	b	b	a	b	b	b
b	a	b	a	b	b	a	b	b	b
b	a	b	a	b	b	a	b	b	b
b	a	b	a	b	b	a	b	b	b
b	a	b	a	b	b	a	b	b	b
b	a	b	a	b	b	a	b	b	b
b	a	-	a	b	b	a	b	b	b
-	a	b	a	-	b	a	b	b	-
b	a	b	a	b	b	-	b	b	b
b	a	b	a	b	b	a	b	b	b
b	a	a	a	b	a	a	b	b	b

b	a	a	a	b	a	-	b	b	b
b	a	a	a	b	a	a	b	b	b
b	a	a	a	b	a	a	a	b	b
b	a	a	a	b	a	a	a	b	b
b	-	a	a	b	a	a	a	b	b
b	a	a	a	b	a	a	a	b	b
b	a	a	a	b	a	a	a	b	b
b	a	a	a	-	a	-	a	b	-
-	b	a	a	b	b	a	b	a	b
a	b	a	a	b	b	a	b	a	b
a	b	a	a	b	b	a	b	a	b
a	b	a	a	b	b	a	b	a	b
a	b	a	a	b	b	a	b	a	b
a	b	a	a	b	b	a	b	a	b
a	b	a	a	-	-	a	b	a	b
a	b	a	a	b	b	a	b	a	-
a	b	a	a	b	b	a	b	a	b
a	b	a	a	b	b	a	b	a	b
a	b	a	a	b	b	a	b	a	b
a	b	a	a	b	b	a	b	a	b
a	b	-	a	b	b	a	b	a	b
a	b	a	a	b	b	a	b	a	b
a	b	a	a	b	-	a	b	a	b
a	a	a	a	b	b	a	b	a	a
a	a	a	a	b	b	a	a	a	a
a	a	a	a	a	b	a	a	a	a
a	a	a	a	b	b	a	a	a	a
a	a	a	a	a	b	a	a	a	-
a	a	a	a	a	b	a	a	a	a
a	a	a	a	a	b	a	a	a	a
a	a	a	a	a	b	a	a	a	a
a	a	a	a	a	b	a	a	a	a
a	a	a	a	a	b	-	a	a	a
a	a	-	a	a	b	a	a	a	a
a	a	a	a	a	b	a	a	a	a
a	a	a	a	b	b	a	a	a	a
b	a	a	a	b	b	a	a	a	a
b	a	a	a	b	b	a	a	b	a
b	a	a	a	b	-	a	a	a	a
b	a	a	a	b	-	a	a	a	a
b	-	a	a	b	b	a	a	a	a
b	a	a	a	b	b	a	a	a	a
b	a	a	a	b	b	a	a	a	a
b	a	a	a	b	b	a	b	a	a
b	-	a	a	b	b	a	b	a	a
b	a	a	a	b	b	a	b	a	a
b	b	a	-	b	b	a	b	a	a

b	a	a	a	b	b	a	b	a	a
a	a	a	a	b	b	a	b	a	a
a	a	a	a	-	b	a	b	a	a
a	a	a	a	b	b	a	b	a	a
a	a	a	a	b	b	a	b	a	a
a	a	a	a	b	b	a	b	a	a
a	a	a	a	b	b	a	b	a	a
a	a	a	a	b	b	a	b	a	a
a	a	a	b	b	b	a	b	a	a
a	a	a	b	b	b	a	b	a	a
a	a	a	b	b	b	a	b	a	a
a	-	a	b	b	b	-	b	a	a
a	a	b	a	a	a	b	a	b	b
a	a	b	a	a	-	b	a	b	b
a	a	b	a	a	a	b	a	b	b
a	a	b	b	a	a	-	a	a	b
a	a	b	b	a	a	b	b	a	b
a	a	b	b	a	a	b	b	a	b
a	a	b	b	b	a	b	b	a	b
a	a	b	b	b	a	b	b	a	b
a	a	b	b	b	a	b	b	a	b
-	a	b	b	b	a	b	b	a	b
a	a	b	b	b	b	b	b	a	b
a	a	b	b	b	b	b	b	a	b
a	b	b	b	b	b	-	b	b	b
a	b	b	b	b	b	b	b	b	b
a	b	b	b	b	b	-	b	b	b
a	b	b	b	b	b	b	b	b	b
a	b	b	b	b	b	b	b	b	b
a	b	b	b	b	b	b	b	b	b
a	-	b	b	b	b	b	b	b	b
a	b	b	b	b	b	b	b	b	b
a	b	b	b	b	b	b	b	b	b
a	b	b	b	b	b	-	a	b	b
a	b	b	b	b	-	b	a	b	-
a	b	b	b	b	b	b	a	b	b
a	b	b	b	b	b	b	a	b	b
a	b	b	b	b	b	-	a	b	b
a	b	b	b	b	b	b	a	b	b
a	b	b	b	b	b	b	a	b	b
a	b	b	b	b	b	b	a	b	b
a	b	b	b	b	b	b	a	b	b
a	b	b	b	b	b	b	a	b	b
a	b	b	b	b	b	b	a	b	b
a	b	b	b	b	b	b	a	b	b
b	b	b	b	b	b	b	a	b	b
a	b	b	b	b	b	b	-	b	b

a	b	b	b	b	-	b	b	b	b
a	b	b	b	b	b	b	b	b	b
a	b	b	b	-	b	b	b	b	b
a	b	b	b	b	b	b	b	b	b
a	a	b	b	b	b	b	b	b	-
a	b	b	b	b	b	b	b	b	b
a	b	-	b	b	b	b	b	b	b
a	b	b	b	b	b	b	b	b	b
a	-	a	b	b	b	a	a	b	b
a	b	a	b	b	b	a	a	b	b
a	b	a	b	b	b	a	a	b	b
a	-	a	b	a	-	a	a	b	b
a	b	a	b	-	b	a	a	b	b
a	b	a	b	a	b	a	a	b	b
a	b	a	b	a	b	a	a	b	b
a	b	a	b	a	b	-	a	b	b
a	b	a	b	a	b	a	a	b	b
a	b	a	b	a	b	a	a	b	b
a	b	a	b	a	b	a	b	b	b
a	b	a	b	a	b	a	b	-	b
a	b	a	b	a	b	a	b	b	b
a	b	a	b	a	b	a	b	b	b
a	b	a	b	a	-	a	b	b	b
a	b	a	b	a	b	a	b	b	b
a	-	a	b	a	b	a	b	b	a
a	b	a	b	a	b	a	b	b	a
a	b	a	b	a	b	a	b	b	a
a	b	a	b	a	b	a	b	b	a
a	b	a	b	a	b	a	b	b	a
a	b	a	b	-	b	-	b	b	a
a	b	a	b	a	b	a	b	b	a
a	b	a	-	a	b	a	b	b	a
a	b	a	b	a	b	a	b	b	a
a	b	a	b	a	b	a	b	b	a
a	b	a	b	a	b	a	b	b	a
a	b	a	b	a	a	a	b	b	a
a	b	b	b	a	b	a	b	b	a
a	b	a	b	a	-	a	b	b	a
a	b	a	b	a	b	a	b	b	a
a	b	a	-	a	b	a	b	b	a
a	b	a	b	a	b	a	b	b	a
a	b	a	b	a	b	a	b	b	a
a	b	a	b	a	b	a	b	b	a
a	b	-	b	a	b	a	b	b	b
a	b	a	b	a	b	a	b	b	b
a	b	a	b	a	b	a	b	b	-
a	b	a	b	a	-	a	b	b	-

a	b	a	b	a	b	a	b	b	b
a	b	a	b	a	b	a	b	b	b
a	b	a	b	a	b	a	b	b	b
a	b	a	b	a	b	-	b	b	b
a	b	a	b	a	b	a	b	b	b
a	-	a	b	a	b	a	b	b	b
a	a	a	b	a	b	a	b	b	b
b	a	a	b	a	b	a	b	b	b
b	a	a	b	a	b	a	b	b	b
b	a	a	b	a	b	a	-	b	b
b	a	a	b	a	b	a	b	b	b
b	a	a	b	a	b	a	b	b	b
b	a	a	b	a	b	a	b	b	b
b	a	a	b	a	b	a	b	b	b
b	a	a	b	a	-	a	b	b	b
b	-	a	b	a	b	a	b	b	b
b	a	a	b	a	b	a	b	b	b
b	a	a	b	a	b	a	b	b	b
a	a	a	a	a	a	a	a	a	a
a	-	a	a	a	a	a	a	a	a
b	b	a	b	a	a	a	b	a	b
b	b	a	-	a	a	a	b	a	b
b	b	a	b	-	a	-	b	a	b
b	b	a	b	a	b	-	b	a	b
b	b	a	b	a	a	a	b	a	b
b	b	a	b	a	a	a	b	a	b
b	b	a	b	-	-	a	b	a	b
b	b	a	b	a	a	a	b	a	b
b	b	a	b	a	a	a	b	a	b
b	b	a	b	a	a	a	b	a	b
b	b	a	b	a	a	a	b	a	b
b	b	a	b	a	a	a	b	a	b
b	b	-	b	-	a	a	b	a	b
b	b	a	b	a	a	a	b	a	b
b	b	a	b	a	a	a	b	a	b
-	b	a	b	a	b	a	b	a	b
b	b	a	b	a	b	a	b	a	b
b	b	a	b	a	b	a	b	a	b
b	b	a	b	a	b	a	b	a	b
b	b	a	b	a	b	a	b	a	b
b	b	a	b	a	b	a	b	a	b
a	b	a	b	a	b	a	b	a	b
b	b	a	b	a	b	a	b	a	b
b	-	a	-	a	b	a	b	a	b
b	b	a	b	a	b	a	b	a	b
b	b	a	b	a	b	a	b	a	b
b	b	a	b	a	b	a	b	a	b

b	b	a	b	a	b	a	b	a	b
b	b	a	b	a	b	a	b	a	-
b	b	a	b	a	b	a	b	a	b
b	-	a	b	a	b	-	b	a	b
b	-	a	b	a	b	a	a	a	b
b	b	a	b	a	b	a	a	a	b
b	b	a	b	a	b	a	a	a	b
b	b	a	b	a	b	a	a	a	b
b	b	a	b	a	b	a	a	a	b
b	b	b	b	a	b	a	a	a	b
b	b	b	b	a	b	a	a	a	b
b	b	-	b	a	b	a	a	a	b
b	b	b	b	a	b	a	a	a	a
b	b	b	b	a	b	a	a	a	b
b	b	b	b	a	b	a	a	a	a
b	b	b	b	a	b	a	a	a	a
b	b	b	b	a	b	a	a	a	a
b	b	b	b	a	b	a	a	a	a
b	b	b	b	a	b	a	a	a	a
b	b	b	b	a	b	a	a	a	a
b	b	b	b	a	b	a	a	a	a
b	b	b	b	a	b	a	a	a	a
b	b	b	b	a	b	a	a	a	a
b	b	b	b	a	b	a	a	a	a
b	b	b	b	a	-	a	a	a	a
b	b	b	b	a	b	a	a	a	a
b	b	b	b	a	b	a	a	a	a
b	b	b	b	a	b	a	a	a	a
a	b	-	b	a	b	a	b	a	a
a	b	b	b	a	-	a	b	a	a
a	b	b	b	a	b	a	b	a	a
a	b	b	b	a	b	a	b	a	a
a	b	b	b	a	b	a	b	a	a
a	b	b	b	-	-	a	b	a	a
a	-	b	b	a	b	a	b	a	a
b	b	a	a	b	a	a	a	a	a
b	b	a	a	b	a	a	a	a	a
b	b	a	a	b	a	a	a	a	a
b	b	a	a	-	a	a	a	a	a
b	b	a	a	b	a	a	a	a	a
b	b	a	a	b	a	a	a	a	a
b	b	a	a	b	a	a	a	a	a
b	b	a	a	b	a	a	a	a	a
b	b	a	a	b	a	a	a	-	a
b	b	b	a	b	a	a	a	a	a
b	b	b	a	b	a	a	a	a	a
-	b	b	a	b	a	a	a	a	a

b	b	b	a	-	a	a	a	a	a
b	b	b	a	b	a	a	a	a	a
b	b	b	a	b	a	a	a	a	a
b	b	-	a	b	a	a	a	a	a
b	b	b	a	b	a	a	a	a	a
a	b	b	a	b	a	-	a	a	a
a	b	b	a	b	a	-	a	a	a
a	b	b	a	b	a	b	a	a	a
a	b	b	a	b	a	b	a	a	a
a	b	b	a	b	a	-	a	a	a
a	b	b	a	b	a	b	a	a	a
a	b	b	a	b	a	b	a	a	a
a	b	b	a	b	a	b	a	a	a
a	b	b	a	b	a	b	a	a	a
a	b	b	a	b	a	b	a	a	a
a	b	b	a	b	a	b	a	a	a
a	b	b	a	-	a	b	a	a	a
a	b	b	a	b	a	b	a	a	a
a	b	b	a	b	a	b	a	a	a
a	b	b	a	b	a	b	a	a	a
a	b	b	a	b	a	b	a	a	a
a	b	b	a	b	a	b	a	a	a
a	b	b	a	b	a	b	a	a	a
a	b	b	a	b	a	b	a	a	a
a	-	b	a	b	a	b	a	a	a
a	-	b	a	b	a	b	a	a	a
a	b	b	a	b	a	b	a	a	a
a	b	b	a	b	a	b	a	a	a
a	b	a	a	b	a	b	a	a	a
a	b	a	a	b	a	b	a	a	a
a	b	a	a	b	a	b	a	a	a
a	b	a	a	b	a	b	a	a	a
a	b	a	a	b	a	b	a	a	a
a	b	a	a	b	a	b	a	a	a
a	b	a	a	b	a	b	a	a	a
a	b	a	a	b	a	b	a	a	a
a	b	a	a	b	a	b	a	a	a
a	b	a	a	b	a	b	a	a	a
a	b	a	a	b	a	b	a	a	a
a	b	a	a	b	a	b	a	a	a
a	a	a	a	-	a	b	a	a	a
a	a	a	a	-	a	b	a	a	a
a	a	a	a	b	a	b	a	a	a
a	a	a	a	b	a	b	a	a	a
a	a	a	a	b	a	b	a	a	a
a	a	a	a	b	a	b	a	a	a
b	b	a	a	b	b	a	b	a	b

b	b	a	a	b	b	b	b	a	b
b	b	a	a	b	b	b	b	a	b
b	b	a	a	b	b	b	b	a	b
b	b	a	a	b	b	b	b	a	b
b	b	a	a	b	b	b	b	a	b
b	b	a	a	b	b	b	b	a	b
b	a	a	a	b	b	b	b	a	b
b	a	a	a	b	b	b	b	a	b
b	a	a	a	b	b	b	b	a	b
b	a	a	a	b	b	b	b	a	b
b	a	a	a	b	b	b	b	a	b
b	a	a	a	b	b	b	b	a	b
b	a	a	a	b	b	b	b	a	b
b	a	a	a	b	b	b	b	a	b
b	a	a	a	b	b	b	b	a	b
b	a	a	a	b	b	b	b	a	b
b	a	a	a	b	b	b	b	a	b
b	a	a	a	b	b	b	b	a	b
b	a	b	a	b	b	b	b	a	b
b	a	b	a	b	b	b	b	a	b
b	a	b	a	b	b	b	b	a	b
b	a	b	a	b	b	b	b	a	b
b	a	b	a	b	a	b	a	a	b
b	a	b	a	b	a	b	a	a	b
b	a	b	a	b	a	b	a	a	b
b	a	b	a	b	a	b	a	a	b
b	a	b	a	a	a	b	a	a	b
b	a	b	a	a	a	b	a	a	b
b	a	b	a	a	a	b	a	a	b
b	a	b	a	a	a	b	a	b	b
a	a	b	a	a	a	b	a	b	b
b	a	a	a	a	a	b	a	a	b
b	a	a	a	a	a	b	a	a	b
b	a	a	a	a	a	b	a	a	b
b	a	a	a	a	a	b	a	a	a
b	a	a	a	a	a	b	a	a	a
b	a	a	a	a	a	b	a	a	a
b	a	a	a	a	a	b	a	a	b
b	a	a	a	a	a	b	a	a	b
b	a	a	a	a	a	b	a	a	b
a	a	b	b	b	b	a	a	b	a
a	b	a	a	a	b	b	a	a	a
b	b	a	a	a	b	b	a	a	a
b	b	a	a	a	b	b	a	a	a
b	b	a	a	a	b	b	a	a	a
b	b	a	a	a	b	b	a	a	a
b	b	a	a	a	b	b	a	a	a

b	b	a	a	a	b	b	a	a	a
b	b	a	a	a	b	b	a	a	a
b	b	a	a	a	b	b	a	a	a
b	b	a	a	a	b	b	a	a	a
b	b	a	a	a	b	b	a	a	a

YW069	YW070	YW071	YW072	YW073	YW074	YW075	YW076	YW077	YW078
b	a	b	b	b	a	b	b	b	b
b	a	b	b	b	a	b	b	a	b
b	b	b	b	b	b	b	b	a	b
b	b	b	b	b	a	-	b	a	-
b	b	b	b	b	a	b	b	a	b
b	b	b	b	-	a	b	-	a	-
b	b	b	b	b	a	b	b	a	b
b	b	b	b	b	a	b	b	a	b
b	b	b	b	b	b	b	b	a	b
b	b	b	b	b	a	b	-	a	b
b	b	b	-	b	a	b	b	a	b
b	b	b	b	b	a	b	b	b	b
b	b	b	b	b	a	b	b	-	b
b	b	b	b	b	a	b	b	b	b
b	b	b	b	b	a	b	b	b	b
b	b	b	-	b	a	b	b	b	b
b	b	-	b	b	a	b	b	b	b
b	a	b	b	b	a	b	b	b	b
b	a	b	b	b	a	-	b	b	b
b	a	b	b	b	a	b	b	b	b
b	a	-	b	b	a	b	b	b	b
b	a	b	b	b	a	b	b	b	b
b	a	a	b	b	a	b	b	b	b
b	a	a	b	b	a	b	b	b	b
b	a	a	b	b	a	b	b	b	b
b	a	a	-	b	a	-	b	b	b
b	a	a	b	b	a	b	b	b	b
b	a	a	b	b	a	b	b	b	a
b	a	a	-	b	a	b	b	b	b
b	a	a	b	b	a	b	b	b	b
b	a	a	b	b	a	b	b	-	-
b	a	a	b	b	a	b	b	b	b
b	a	a	b	b	a	b	b	b	b
b	a	a	b	a	a	b	b	b	a
b	a	a	b	a	a	b	b	b	-
b	a	a	b	a	a	b	b	b	a
b	a	a	a	a	a	b	b	b	a
b	a	a	b	a	-	-	b	b	a
b	a	a	b	a	a	b	b	b	a
b	a	a	b	a	a	b	b	b	a
b	a	a	b	a	a	b	b	b	a
b	a	a	b	a	a	b	b	b	a
b	a	a	b	a	a	b	b	b	a
b	a	a	b	a	a	b	b	b	b
b	a	a	b	a	a	b	b	b	a

b	a	a	b	-	a	b	b	b	a
b	a	a	a	a	a	b	b	b	a
b	a	a	b	a	a	b	b	b	a
b	a	a	b	a	a	b	b	b	a
b	a	a	b	a	a	b	b	b	a
b	a	a	b	a	a	b	b	b	a
b	a	a	b	a	a	b	b	b	-
b	a	a	b	a	a	b	b	b	a
b	a	a	b	a	a	-	b	b	a
b	a	-	b	a	a	b	b	b	a
b	a	-	b	a	a	b	b	b	a
b	a	a	b	a	a	b	b	b	-
b	a	-	b	a	a	b	b	-	a
b	a	a	b	a	a	b	b	a	a
b	a	a	a	a	b	b	b	a	a
b	a	a	a	a	b	b	b	a	a
b	a	a	a	a	b	b	b	a	a
b	a	a	a	a	b	b	b	a	a
b	a	a	-	a	b	b	b	b	a
b	a	-	a	a	b	b	b	b	a
b	a	a	a	-	b	b	b	b	a
b	a	a	a	a	b	b	b	b	a
b	a	a	a	a	-	b	b	b	a
b	a	a	a	a	b	b	b	b	a
b	a	a	a	a	b	b	b	b	a
b	a	a	-	a	b	b	b	b	a
b	a	a	a	a	b	b	b	b	-
b	a	a	a	a	b	b	b	b	b
b	a	a	a	a	b	b	b	b	b
b	a	a	a	a	b	b	b	b	b
-	a	a	a	b	b	b	b	b	b
b	a	a	a	b	b	b	b	b	b
b	a	a	a	b	-	b	b	b	b
b	a	a	a	b	b	b	b	b	b
b	a	-	a	b	-	b	b	b	-
b	a	a	a	b	b	b	b	b	a
b	a	-	a	b	b	b	b	b	b
b	a	-	a	b	b	b	b	-	b
b	a	a	a	-	-	b	b	b	b
a	a	a	-	b	b	a	b	a	a
a	a	a	b	b	b	a	b	a	a
a	a	a	b	b	b	a	b	b	a
a	a	a	b	b	b	a	-	b	a
a	a	a	b	b	b	a	b	b	a
a	a	a	b	b	b	a	b	b	a
a	a	a	b	b	b	a	b	b	a
a	a	a	-	b	-	a	b	b	a

a	a	a	b	b	b	a	b	b	a
a	a	a	b	b	b	a	b	b	a
a	a	a	b	b	b	a	b	b	a
a	a	a	b	b	-	a	b	b	a
a	a	a	b	b	a	a	-	b	a
a	a	a	b	b	b	a	b	b	a
a	a	a	b	b	a	a	b	b	a
a	a	a	b	b	b	a	b	b	a
a	a	a	b	b	a	a	b	-	a
a	a	a	a	b	a	-	b	b	a
a	a	a	a	b	a	a	b	b	a
-	a	a	a	b	a	a	b	b	a
a	a	a	a	b	a	a	b	a	a
b	a	a	a	b	a	b	-	a	a
b	a	a	-	b	a	b	b	a	b
b	a	a	a	b	-	b	b	a	a
b	a	a	a	-	a	b	b	a	a
b	a	a	a	b	a	b	b	a	-
b	a	a	a	b	a	b	b	a	a
b	a	a	a	b	a	b	b	a	a
b	a	a	b	b	b	b	b	a	a
b	a	a	a	b	a	b	b	a	a
b	a	a	a	b	a	a	b	a	b
b	a	a	a	b	a	a	b	a	b
b	a	a	b	b	-	a	b	a	b
b	a	a	a	b	a	a	b	a	b
b	a	a	a	b	a	a	b	-	b
b	a	a	a	b	a	a	b	a	b
b	a	a	a	b	a	a	b	a	b
b	a	a	a	b	a	a	b	a	-
b	a	a	a	b	a	a	-	a	b
b	a	a	a	b	a	-	b	a	-
b	a	a	a	b	a	-	-	a	b
b	a	a	a	-	a	a	b	a	b
b	a	a	a	b	a	a	b	a	-
b	a	a	a	-	a	a	b	a	b
b	a	a	a	b	a	a	b	a	b
b	a	a	a	b	-	a	b	a	b
b	a	a	a	b	a	-	b	a	b
b	a	a	a	b	a	a	b	a	b
b	a	a	a	-	a	a	b	a	-
b	a	a	a	b	a	a	b	a	b
b	a	a	a	b	a	a	b	a	b
b	a	a	a	b	a	a	b	a	a
b	a	a	-	b	a	a	b	a	b
b	a	a	a	b	a	a	-	a	b
b	a	a	a	b	a	a	b	a	a

b	a	a	-	b	a	a	b	a	b
b	a	a	a	b	a	a	-	a	-
b	a	a	a	b	a	a	b	a	b
b	a	a	a	b	a	a	b	a	b
b	a	a	a	b	a	a	b	a	b
b	a	a	a	b	-	a	b	a	b
b	a	a	a	b	a	a	b	a	b
b	a	a	a	b	a	a	b	a	b
b	a	a	a	b	b	-	b	a	b
b	a	a	a	b	a	-	b	a	b
b	a	a	a	b	a	a	b	a	b
b	a	a	a	b	a	a	b	a	b
b	a	a	a	b	a	a	b	a	b
b	a	a	a	-	a	a	b	a	a
b	a	a	a	b	a	a	b	a	b
b	a	a	a	b	b	a	b	a	b
b	a	a	a	b	-	a	b	a	b
b	a	a	a	b	b	a	b	a	b
b	a	a	a	b	b	b	b	a	b
b	a	-	a	b	b	b	b	a	b
b	a	a	-	b	b	b	b	b	b
b	a	a	a	b	b	b	b	b	b
b	a	a	a	b	b	b	b	b	b
b	a	a	a	b	b	b	b	b	b
b	a	a	a	b	b	a	-	b	-
b	a	a	a	b	-	a	b	b	b
b	a	a	a	b	b	a	b	b	b
b	a	a	a	b	b	a	b	b	b
b	a	a	a	b	b	a	-	b	-
b	a	a	a	a	b	a	a	b	b
b	a	a	a	-	b	a	a	b	b
b	a	a	a	a	-	a	a	b	b
b	a	a	a	a	b	a	a	b	b
b	a	a	a	a	b	a	a	b	b
b	a	a	a	a	b	a	a	b	b
b	-	a	-	a	b	a	a	b	b
b	a	a	a	a	b	a	-	b	b
b	a	a	-	a	b	a	a	b	b
b	a	a	a	a	b	a	a	b	b
b	a	a	a	a	-	a	a	b	b
b	a	-	a	a	b	a	a	b	b
b	a	-	a	a	-	a	a	b	b
b	a	a	a	a	-	a	a	b	b
b	a	a	-	a	b	a	a	b	-
b	a	a	b	a	a	a	a	a	-
b	a	a	b	a	a	a	a	b	b
b	a	-	b	a	b	a	a	b	b

b	a	a	b	a	b	a	a	b	a
b	a	a	b	a	b	a	a	b	b
b	a	a	b	a	b	a	a	b	b
b	a	a	b	a	b	a	a	b	b
b	a	a	b	a	b	a	a	b	b
b	a	-	b	a	b	a	a	b	b
b	a	a	b	a	b	a	a	b	b
b	a	a	b	a	b	a	a	a	b
a	a	a	a	a	b	a	a	a	b
a	a	a	b	a	b	a	a	a	b
b	a	a	b	a	b	a	a	a	b
a	a	a	b	a	b	-	a	a	b
a	b	a	a	a	b	b	a	a	b
a	b	a	a	a	b	b	a	a	b
a	b	a	a	a	b	b	a	a	b
a	b	a	-	a	b	b	a	a	b
a	b	-	a	a	b	b	a	a	b
a	b	a	-	a	b	b	a	a	b
a	b	a	b	a	b	b	a	a	-
a	b	a	b	a	b	b	a	a	b
a	b	a	b	a	b	b	a	a	b
a	b	a	b	a	b	b	a	-	b
a	b	a	b	a	b	b	a	a	b
a	b	a	b	a	b	b	-	a	b
a	b	a	b	a	b	b	a	a	b
a	b	a	b	a	b	b	a	a	b
a	b	a	b	a	b	b	a	a	a
a	b	a	b	a	b	b	a	a	b
a	b	a	b	a	b	b	a	a	b
a	b	a	b	b	b	b	a	a	b
a	b	a	b	-	-	b	a	a	b
a	b	a	b	b	b	b	a	a	b
a	b	a	b	b	b	b	a	a	b
a	b	a	b	b	b	b	a	a	b
a	b	a	b	b	b	b	a	a	b
a	b	a	b	-	b	b	a	a	b
a	b	a	b	b	b	b	a	a	b
a	b	a	b	b	b	b	a	a	b
a	b	a	b	b	b	b	a	a	b
a	b	a	b	b	b	b	a	a	b
a	b	a	b	b	b	b	a	b	a
a	b	a	-	b	b	b	a	b	a
a	b	a	-	b	b	-	a	b	a
a	b	-	-	b	b	b	a	b	a
a	b	a	b	b	b	b	a	b	a
a	b	a	b	b	b	b	a	b	a
a	b	a	b	b	b	b	a	b	-

a	b	a	b	b	b	b	a	b	a
a	b	a	b	b	b	a	a	b	a
a	b	a	b	b	b	a	a	-	a
a	b	a	b	b	a	a	a	b	a
a	b	a	b	a	-	a	a	b	a
a	b	a	b	a	a	a	a	b	a
a	b	-	b	b	a	a	a	b	a
a	b	a	b	b	a	a	a	b	a
a	b	a	b	b	a	a	-	b	a
a	b	a	b	b	a	b	a	b	a
a	b	a	b	b	a	b	a	b	a
a	b	a	b	b	a	b	a	-	a
a	b	a	b	b	a	b	a	b	a
a	b	a	b	b	a	b	a	b	-
a	b	a	b	b	a	b	a	b	a
a	b	a	b	b	a	b	a	b	a
a	b	a	b	b	a	b	a	b	a
a	b	a	b	b	b	b	a	b	b
a	b	a	-	b	a	b	a	b	a
a	b	a	b	b	a	b	a	b	a
a	b	a	b	b	a	b	a	b	a
a	b	a	b	b	a	b	a	b	a
a	b	a	b	b	-	b	a	b	-
a	b	a	b	b	a	b	a	b	a
b	b	a	b	b	a	b	a	b	a
b	b	a	b	b	a	b	a	b	a
b	b	a	a	b	a	b	a	b	a
b	a	a	b	b	a	b	a	b	a
b	a	a	b	b	a	b	a	b	a
b	a	a	b	b	a	b	a	a	a
b	a	a	a	b	a	b	a	a	a
b	a	a	a	b	a	b	a	a	a
b	a	-	a	b	a	b	a	a	a
b	a	a	a	b	a	b	a	a	a
b	a	a	a	b	a	b	a	a	a
b	a	a	a	b	a	b	a	a	a
b	a	a	a	b	a	-	a	a	a
b	a	a	a	b	a	b	a	a	a
b	a	-	a	-	a	b	a	a	a
b	a	a	a	a	a	b	a	a	a
b	a	a	a	a	a	b	a	a	a
b	a	a	a	-	a	b	a	a	a
b	a	a	a	a	a	b	a	a	a
b	a	a	a	-	a	b	a	a	a
b	a	a	a	a	a	b	a	a	a
b	a	a	a	a	a	b	a	a	a
b	a	a	a	a	a	b	a	a	a

-	a	a	a	a	a	b	a	a	a
b	a	a	a	a	a	b	a	a	-
b	a	a	a	a	a	b	a	a	a
b	a	a	a	a	a	b	a	a	a
b	a	a	a	a	a	b	a	a	a
b	a	a	a	a	a	b	a	a	a
b	a	a	a	a	a	b	-	a	a
b	a	a	a	a	-	b	a	a	a
b	a	a	a	a	a	b	a	a	a
b	a	a	a	a	a	b	a	a	a
b	a	a	a	a	a	b	a	a	a
b	a	-	a	a	a	b	a	a	a
b	a	a	a	a	a	-	a	a	a
b	a	a	a	a	a	b	a	a	a
b	a	-	a	a	a	b	a	a	a
b	a	a	a	a	a	b	a	a	a
b	a	-	a	a	a	b	a	a	a
b	a	a	a	a	a	b	a	a	a
b	a	a	a	a	b	b	a	a	a
b	a	a	a	a	a	b	a	a	a
b	a	-	a	a	-	b	a	-	a
b	a	a	a	a	a	b	a	a	a
b	a	a	a	a	a	b	a	a	a
b	a	a	a	a	b	b	-	a	a
b	a	a	a	a	b	b	a	a	a
b	a	-	a	a	a	b	a	a	a
b	a	a	a	a	-	b	a	a	a
b	a	a	a	a	a	b	a	a	a
b	a	a	a	a	a	b	a	a	a
b	a	a	a	a	a	b	a	a	a
b	a	a	a	a	a	b	a	a	a
b	a	-	a	a	a	b	a	a	a
b	a	a	a	a	a	b	a	a	a
b	a	-	b	a	a	b	a	a	a
b	a	a	b	a	a	b	a	a	a
b	a	a	b	a	a	b	a	a	a
b	a	a	b	a	a	b	a	a	a
b	a	a	b	a	a	b	a	a	a
b	b	a	a	a	b	b	a	b	a
b	b	-	a	a	b	b	a	b	a
b	b	a	a	a	a	b	a	b	a
b	b	a	a	a	a	b	a	b	a
b	b	a	a	a	b	b	a	b	a
b	b	a	-	a	-	b	a	b	a
b	b	a	a	a	a	b	a	b	a
b	b	a	a	a	a	b	a	b	a
b	b	a	a	a	a	b	a	b	a

b	b	a	a	a	a	b	-	b	a
b	b	a	a	a	a	b	a	b	a
b	b	-	a	a	a	b	a	b	a
b	b	a	a	a	a	b	a	b	a
b	b	a	a	a	a	b	a	b	a
b	b	a	a	a	a	b	a	b	a
b	a	-	a	a	a	b	a	b	a
b	a	a	a	a	a	b	b	b	a
b	a	a	b	a	a	b	b	b	b
b	a	a	a	a	a	b	b	b	a
b	a	a	-	a	-	b	b	b	-
b	a	a	a	a	a	b	b	b	a
b	a	a	a	a	a	b	b	b	-
b	a	a	a	a	a	b	b	b	a
b	a	a	a	a	a	b	b	b	a
b	a	b	a	a	a	b	b	b	a
b	a	b	a	a	a	b	b	b	a
b	a	b	a	a	a	b	b	b	a
b	a	b	a	a	a	b	b	b	a
b	a	b	a	a	a	b	b	b	a
b	a	b	a	a	a	-	b	b	a
b	a	b	a	a	a	b	b	b	a
b	a	b	-	-	a	b	b	b	a
b	a	b	a	a	a	b	b	b	a
b	a	b	a	a	a	-	b	b	a
b	a	-	b	a	a	b	b	b	b
b	a	b	a	a	a	b	b	b	a
b	a	b	a	a	a	b	b	b	a
b	a	b	a	a	a	b	b	b	b
b	a	b	-	a	a	b	b	b	a
b	a	b	a	a	a	b	b	b	a
b	a	b	a	a	a	b	b	b	a
b	a	b	a	a	a	b	b	b	a
b	a	b	a	a	a	b	b	b	a
b	a	b	a	a	a	b	b	b	a
b	a	b	a	a	a	b	-	b	a
b	a	b	a	b	a	b	b	a	a
b	a	b	a	b	a	b	b	a	a
b	a	b	a	b	a	b	b	-	a
b	a	b	a	b	a	b	b	a	a
b	a	b	a	b	a	b	b	a	a
b	a	b	a	b	a	b	b	a	a
b	a	b	a	b	a	b	b	a	a
b	a	-	a	b	b	b	b	a	a
b	a	b	a	b	-	b	b	a	a
b	a	b	a	b	a	b	b	a	a
b	a	b	a	b	a	b	b	a	a

b	a	b	a	b	a	-	b	a	a
b	a	b	a	b	a	b	b	a	a
b	a	b	a	a	a	b	b	a	a
b	a	b	a	a	a	b	b	a	a
b	a	b	a	a	a	b	b	a	a
b	a	b	a	a	a	b	b	a	a
b	a	b	a	a	b	b	b	a	a
b	a	b	a	a	b	b	b	a	a
b	a	b	a	a	b	b	b	a	a
b	a	b	a	a	b	b	b	a	a
b	a	b	a	a	b	b	b	a	a
b	a	b	a	a	b	b	b	a	a
b	a	b	-	a	b	b	b	a	a
b	a	b	a	a	b	b	b	a	a
b	a	b	a	a	b	b	b	a	a
b	a	-	a	a	b	b	b	a	a
b	a	b	a	a	b	b	a	a	a
b	a	-	a	a	b	b	a	a	a
b	a	b	a	a	b	b	a	a	a
b	a	b	a	a	b	b	a	a	a
b	a	b	a	a	b	b	a	a	a
b	a	-	-	a	b	-	a	a	a
b	a	b	a	a	b	b	a	-	a
b	a	b	a	a	b	-	a	a	-
a	b	b	a	a	b	b	a	a	a
a	b	b	a	a	-	b	a	a	a
a	b	b	a	a	b	b	a	a	a
a	b	b	a	a	b	b	a	a	a
a	b	b	a	-	b	b	a	a	a
a	b	b	a	a	a	b	a	a	a
a	b	b	a	a	b	b	a	a	a
a	b	b	a	a	b	b	a	a	a
a	b	b	a	a	b	b	-	a	a
a	b	b	a	a	b	b	a	a	a
a	b	b	a	a	b	b	a	a	a
a	b	b	a	a	b	a	a	a	a
a	b	b	a	a	-	a	a	a	a
a	b	b	a	a	b	a	a	a	a
a	b	b	a	a	b	a	a	a	a
a	b	b	a	a	b	a	a	a	a
b	a	b	b	b	b	b	b	a	b
b	a	b	b	b	b	b	b	a	b
b	a	-	b	b	b	b	b	a	b
b	a	b	-	b	b	b	b	a	b
b	b	b	b	b	b	b	b	a	b
b	b	b	b	a	b	b	b	a	b
b	b	b	b	a	-	b	b	a	b

b	b	b	b	a	a	b	-	a	b
b	b	b	b	-	b	b	-	a	-
b	b	b	b	a	b	b	b	a	b
b	b	-	b	a	b	b	b	a	b
b	b	b	a	-	b	b	b	a	b
b	b	b	b	a	b	b	b	-	b
b	b	b	a	a	b	b	b	a	b
b	b	b	a	a	b	b	b	a	b
a	b	b	a	a	b	b	b	a	b
a	b	b	a	a	a	b	b	a	b
a	b	b	a	-	b	b	b	a	b
a	b	b	a	a	b	b	b	a	b
a	b	b	a	a	b	b	b	a	b
a	b	b	a	a	b	b	b	a	b
a	b	b	a	a	-	b	b	a	-
a	b	b	a	a	b	b	b	a	b
a	b	b	a	-	b	b	b	a	b
a	b	b	a	a	b	b	b	a	b
a	b	b	a	a	b	b	b	a	b
a	b	-	a	a	b	b	b	a	-
a	b	b	a	a	b	b	b	a	-
a	b	b	-	a	b	b	b	a	a
a	b	b	a	a	b	b	b	a	a
a	b	b	a	a	b	b	b	a	a
a	b	b	a	a	-	b	b	a	a
a	b	b	a	a	b	b	b	a	a
a	b	b	a	a	b	b	b	a	a
a	b	b	a	a	b	b	b	a	a
a	b	b	a	a	b	b	b	a	a
a	b	b	a	a	b	b	b	a	a
a	b	b	a	a	-	b	b	a	a
a	b	b	a	a	b	-	b	a	a
a	b	b	a	a	a	b	b	a	a
a	a	b	a	a	b	b	b	a	a
a	b	b	a	a	b	b	b	a	a
a	b	b	a	a	b	b	b	a	a
a	b	b	a	a	b	b	b	a	a
a	b	b	a	a	b	b	b	a	a
a	b	b	a	a	b	b	b	-	a
a	b	b	a	a	b	b	b	a	a
a	b	b	a	a	b	b	b	a	a
a	b	b	-	a	b	b	b	a	a
a	b	b	a	a	b	b	b	a	a
a	b	b	a	a	a	b	-	a	a
a	b	-	a	a	b	b	b	a	a
a	b	b	a	a	b	b	b	a	a
a	b	b	a	a	-	b	b	a	a

a	b	b	a	a	b	b	b	a	a
a	b	b	a	a	-	b	b	a	a
a	b	b	-	a	b	b	b	a	a
a	b	b	a	a	b	b	b	a	a
a	-	b	a	a	-	b	b	a	a
a	b	b	a	a	b	b	b	a	a
a	b	b	-	a	b	b	b	a	a
a	b	b	a	a	a	b	b	a	a
a	b	b	a	a	b	b	b	a	a
a	b	b	a	a	b	b	b	a	a
a	b	b	a	a	b	b	b	a	a
a	b	b	a	a	b	b	b	a	a
a	b	b	a	a	b	b	b	a	a
a	b	b	a	a	b	b	b	a	a
a	a	b	a	a	b	b	b	-	-
a	a	b	a	a	b	b	b	a	a
a	a	b	a	a	b	b	b	a	a
a	a	b	a	a	b	b	b	a	a
a	a	b	a	a	b	b	b	a	a
a	a	b	a	a	-	b	b	a	a
-	a	b	a	a	b	b	b	a	a
a	a	-	a	a	b	b	b	a	a
a	a	b	a	a	b	b	b	a	a
a	a	b	a	-	b	b	b	a	a
a	a	b	a	a	b	b	b	a	a
a	a	b	a	a	b	b	b	a	a
a	a	b	-	a	b	b	b	a	a
a	a	b	a	a	b	b	b	a	a
a	a	b	a	a	b	b	b	a	a
a	a	b	a	a	b	b	b	-	a
a	a	b	a	a	b	b	b	a	a
a	a	b	a	a	b	b	b	a	a
a	a	b	a	a	b	b	b	a	a
a	a	b	a	a	b	b	-	a	a
a	a	b	a	a	b	b	b	a	a
a	a	b	a	a	b	b	b	a	a
a	a	b	a	a	-	b	b	a	a
a	a	b	a	a	b	b	b	a	a
a	a	b	a	a	b	b	b	a	a
a	a	b	a	a	b	b	b	a	a
a	a	b	a	a	b	b	b	a	a
a	a	b	a	a	-	b	b	a	a
a	a	b	a	a	b	b	b	a	a
a	a	b	a	a	-	b	b	a	a
a	a	b	a	a	b	b	b	a	a
a	a	b	a	a	b	b	b	a	a
a	a	b	a	a	a	b	b	a	a

a	a	b	a	a	b	b	b	a	b
a	a	-	a	a	b	b	b	a	b
a	a	b	a	a	b	b	b	a	b
a	a	b	-	a	b	b	b	a	b
a	a	b	b	a	b	b	b	a	b
a	a	b	-	a	b	b	b	-	b
a	a	b	a	a	b	b	b	a	b
a	a	b	-	a	b	b	b	a	b
a	a	b	a	a	b	b	b	a	b
a	a	-	a	a	b	b	b	a	b
a	a	b	a	a	b	b	b	a	b
a	a	b	a	a	b	b	b	a	b
a	a	b	a	a	b	b	b	a	-
a	a	b	a	a	b	b	b	a	b
a	a	b	a	a	a	b	b	a	b
a	a	b	a	a	a	b	b	a	-
a	a	b	-	a	a	b	b	a	b
a	a	b	a	a	a	b	b	a	b
a	a	b	a	a	b	b	b	a	b
a	a	a	b	a	a	a	b	b	a
a	a	a	b	a	a	a	b	b	a
a	a	a	b	a	a	a	b	b	a
a	a	a	b	a	a	a	b	b	a
a	a	a	b	a	a	a	b	b	a
a	a	a	b	a	a	a	b	b	a
a	a	a	b	a	a	a	b	b	a
a	a	a	b	a	a	a	b	b	a
a	a	a	b	a	a	a	b	b	a
a	a	a	b	a	a	a	b	b	a
a	a	a	b	a	a	a	b	b	a
a	a	a	b	a	a	a	b	b	a
a	a	a	b	a	a	a	b	b	a
a	a	a	b	a	a	a	b	b	a
a	a	a	-	a	a	a	b	-	a
a	a	a	b	-	a	a	b	b	b
a	a	a	b	a	a	a	b	b	a
a	a	a	b	a	a	a	b	b	a
a	a	a	b	a	a	a	b	b	a
a	a	a	b	a	a	a	b	b	a
a	a	a	b	a	-	a	b	b	-
a	a	a	b	a	a	a	b	b	a
a	a	a	b	a	b	a	b	b	b
a	a	a	b	a	a	a	b	b	a
a	a	a	b	a	a	a	b	b	a
a	a	a	b	a	a	a	b	b	a
a	a	a	b	a	a	a	b	b	a
a	a	a	a	a	a	a	b	b	a

a	a	a	a	a	a	a	b	b	a
a	a	a	a	a	a	a	b	-	a
a	a	a	a	a	a	a	b	b	a
a	a	a	a	a	a	a	b	b	a
a	a	a	a	a	a	a	b	b	a
a	a	a	a	a	a	a	b	b	a
a	a	a	a	a	a	a	b	-	a
a	a	a	a	a	a	a	b	a	a
a	a	a	a	a	a	a	b	a	a
a	a	a	a	a	a	a	b	a	a
a	b	a	a	a	a	a	b	a	a
a	b	a	a	a	a	a	b	a	a
a	b	a	a	a	a	a	b	a	a
a	b	a	a	a	a	a	b	a	a
a	b	a	-	a	-	a	b	a	a
a	b	a	a	a	a	a	b	-	a
a	b	a	-	a	a	a	b	a	a
a	b	a	a	a	a	a	b	a	a
a	b	a	a	a	a	a	b	a	a
a	b	a	a	a	a	a	b	a	a
a	b	a	a	a	a	a	b	a	a
a	b	a	a	a	a	a	b	a	a
a	b	a	a	a	a	a	b	a	a
a	b	a	a	a	-	a	a	a	-
a	b	a	a	a	a	a	a	a	a
a	b	a	a	a	a	a	a	a	a
a	b	a	a	a	a	a	a	a	a
a	b	a	a	a	a	a	a	a	a
a	b	a	a	a	a	a	a	a	a
a	b	a	a	a	a	a	a	a	a
a	b	a	a	a	a	a	a	a	a
a	b	a	a	a	a	a	a	a	a
a	b	a	a	a	a	a	a	a	a
a	b	a	a	a	a	-	a	a	a
a	b	a	a	a	a	a	a	a	a
a	b	a	a	-	-	a	a	a	a
a	b	a	a	a	a	a	a	a	a
a	b	a	a	a	a	a	a	a	a
a	b	a	a	a	a	a	a	a	a
a	b	a	a	a	a	a	a	a	a
a	b	a	a	a	a	a	a	a	a
a	b	a	a	a	a	a	a	a	a
a	b	a	a	a	a	a	a	a	a
a	b	a	a	a	a	a	a	a	a
a	b	a	a	a	a	a	a	a	b
a	b	a	a	a	a	a	a	a	b
a	b	a	a	-	a	a	a	a	b

a	b	a	a	a	a	a	a	a	b
a	a	a	a	a	a	a	a	a	b
a	a	a	a	a	a	a	a	a	b
a	a	a	a	a	a	a	a	a	b
a	a	a	a	a	a	-	a	a	b
a	a	a	a	a	a	b	a	a	b
a	a	a	a	a	a	b	a	a	b
a	a	a	a	b	a	b	a	a	b
a	a	a	a	b	a	b	a	a	b
a	a	a	a	b	a	b	a	a	b
a	a	a	a	b	a	b	a	a	a
b	a	a	b	a	b	a	a	b	a
b	a	a	b	a	b	a	a	b	a
b	a	b	b	a	b	-	a	-	a
b	a	a	b	a	b	a	a	a	a
b	a	a	b	a	b	a	a	a	a
b	a	a	b	a	b	a	a	a	a
b	a	a	b	a	b	a	a	a	a
b	a	a	b	a	-	a	a	a	a
b	a	b	b	a	b	a	a	a	a
b	a	b	b	a	b	a	a	-	a
b	a	b	b	a	b	a	a	a	a
b	a	b	b	a	b	a	a	a	a
b	a	b	b	a	b	a	a	a	a
b	a	b	b	-	b	a	a	a	a
b	a	b	b	a	b	a	a	a	a
b	a	b	b	a	b	a	a	-	a
b	a	b	b	a	b	a	a	a	a
b	a	-	b	a	b	a	a	a	a
b	a	b	b	a	b	a	a	a	a
b	a	-	b	a	b	a	a	a	a
b	a	-	b	a	b	a	a	a	a
b	a	b	b	a	b	a	a	a	a
b	a	b	b	a	b	a	a	a	-
b	a	b	b	-	b	a	a	a	a
b	a	b	b	a	-	a	a	a	a
b	b	b	b	a	a	a	a	a	a
b	b	b	b	-	b	a	a	a	a
b	b	b	b	a	b	a	a	a	a
b	b	b	b	a	b	a	a	a	a
b	b	b	b	a	b	a	b	a	a
b	b	b	b	a	b	a	b	a	a
b	b	-	b	a	b	a	b	-	a
b	b	b	b	a	b	a	b	a	a
b	b	b	b	a	b	a	b	a	a
b	b	-	b	a	b	a	b	a	a
b	b	b	b	a	a	a	b	a	a

b	b	b	b	a	b	a	b	a	-
b	b	b	-	a	b	a	b	a	a
b	b	b	b	a	-	a	b	a	a
b	b	b	b	a	b	a	b	a	a
b	b	b	b	a	b	a	b	a	a
b	b	b	b	a	-	b	-	a	a
b	b	b	b	a	a	b	b	a	a
b	b	b	b	a	b	-	b	a	a
b	b	b	b	a	-	b	b	a	a
b	b	-	b	a	b	b	b	a	a
b	b	-	b	a	b	b	b	a	a
b	b	b	b	a	b	b	b	a	a
b	b	b	b	a	b	b	b	a	-
b	b	b	b	a	b	b	b	a	a
b	b	b	b	a	b	b	b	a	a
b	b	b	b	a	-	b	b	a	a
b	b	b	b	a	b	b	b	a	a
b	b	b	b	a	b	b	b	a	a
b	b	b	b	a	-	-	b	a	a
b	b	b	b	a	-	b	b	a	a
b	b	b	b	-	b	b	b	a	-
b	b	b	b	a	b	b	b	a	a
b	b	b	a	a	b	b	b	a	a
b	b	b	a	a	a	b	b	a	a
b	b	b	a	a	a	b	b	a	a
b	b	-	a	a	-	b	b	a	a
b	b	b	a	a	a	-	-	a	a
b	b	b	a	a	a	b	-	a	a
b	b	b	a	a	a	b	-	a	a
b	b	b	a	a	a	b	b	a	a
b	b	b	a	a	a	b	b	a	a
b	b	b	a	a	a	b	b	a	a
b	b	b	a	a	a	b	b	a	a
b	b	b	a	a	a	b	b	a	a
b	b	b	a	a	a	b	b	a	a
b	b	b	a	a	-	b	b	a	a
b	a	b	a	a	-	b	b	a	a
b	a	-	a	a	a	b	b	a	a
b	a	b	a	a	a	b	b	a	a
b	a	b	a	a	a	b	b	a	a
b	a	b	a	a	a	b	b	a	a
b	a	a	a	a	a	b	b	a	a
b	a	-	a	-	a	b	b	a	a
a	b	a	b	b	-	b	a	b	b
a	b	a	a	b	a	b	a	a	b
a	b	a	a	b	a	b	a	a	b
a	b	a	a	b	a	b	a	a	b

a	b	a	a	b	a	b	a	a	b
a	b	a	a	b	a	b	a	a	b
a	b	a	a	b	a	b	a	a	b
a	b	a	a	b	a	b	a	a	a
a	b	a	a	b	a	b	a	a	b
a	b	a	a	b	a	b	a	a	b
a	b	a	a	b	b	b	a	a	b
a	b	a	a	b	a	b	a	a	b
a	b	a	a	b	a	b	a	a	b
a	b	a	a	b	a	b	a	a	b
a	b	a	a	b	a	b	a	a	b
a	b	a	a	b	a	b	a	a	b
a	b	a	a	b	a	b	a	a	b
a	b	a	a	b	a	b	a	a	b
a	-	a	a	b	a	b	a	a	b
a	b	a	a	b	b	b	a	a	b
a	b	a	a	b	a	b	a	a	b
a	b	a	a	b	-	b	a	a	b
a	b	a	a	b	a	b	-	a	b
a	b	a	a	b	a	b	a	-	b
a	b	-	a	b	a	b	a	a	b
a	b	a	a	b	a	b	a	b	b
a	a	b	b	b	a	a	a	a	b
a	a	b	b	b	a	a	-	a	b
a	a	b	b	b	a	a	a	a	b
a	a	b	b	b	a	a	a	a	b
a	a	b	b	b	a	a	a	a	b
a	a	b	b	b	a	a	a	a	b
a	a	b	b	-	a	a	a	b	b
a	a	b	b	b	-	a	a	b	b
a	a	b	b	b	b	a	a	b	b
a	a	b	a	b	a	a	a	b	a
a	a	b	a	b	a	a	a	b	a
a	a	b	a	b	a	a	a	-	a
a	a	b	a	b	a	a	a	b	a
a	a	b	a	b	a	a	-	b	b
a	a	b	a	-	a	a	a	b	a
a	a	b	a	b	a	a	-	b	a
a	a	b	a	b	a	a	a	b	a
a	a	b	-	b	b	a	a	b	a
a	a	b	a	b	a	a	a	b	a
a	b	b	a	b	a	-	a	b	a
a	b	b	a	b	a	a	a	b	a
a	b	b	a	-	a	a	a	b	a
a	b	b	a	b	a	a	a	b	a
a	b	b	a	b	a	a	-	b	a
a	b	b	-	b	-	b	a	b	a

a	b	b	a	b	b	b	a	b	a
a	b	b	-	b	b	b	a	b	b
a	b	b	a	b	b	b	-	b	a
b	b	b	a	b	-	a	a	b	b
b	b	b	a	b	b	a	a	-	b
b	b	b	a	b	-	a	a	b	b
b	b	b	b	b	b	-	a	b	b
b	b	b	a	b	a	a	a	b	a
b	b	b	a	-	-	a	a	b	a
b	b	b	b	b	b	a	a	b	b
b	b	b	b	b	b	a	a	b	b
b	b	b	b	b	b	a	a	b	b
b	b	b	b	b	b	a	a	b	b
b	b	b	b	b	a	a	a	b	b
b	b	b	b	b	b	a	a	-	b
b	b	b	b	b	b	a	a	b	b
b	b	b	-	b	b	a	a	b	b
b	b	b	b	b	b	a	a	b	b
b	b	b	b	b	b	a	a	b	b
b	b	b	b	-	b	a	a	b	b
b	b	b	b	b	b	a	a	b	b
b	b	b	b	b	b	a	a	b	b
b	b	b	b	b	b	a	a	b	b
b	b	b	b	b	b	a	a	b	b
b	b	b	b	b	b	a	a	b	b
b	b	b	b	b	b	a	a	b	b
b	b	b	b	b	b	a	a	b	b
b	b	b	a	b	b	a	a	b	-
b	b	b	a	b	b	a	a	b	a
b	b	b	a	b	b	a	a	b	a
b	b	b	a	b	b	a	a	b	-
b	b	b	a	b	b	a	a	b	a
b	b	b	a	b	b	a	a	b	a
b	b	b	a	b	b	a	a	b	a
b	b	b	a	b	b	a	a	b	-
b	b	b	a	b	b	a	a	b	a
b	b	b	a	b	b	a	a	b	a
b	b	b	a	b	b	a	a	b	a
b	b	b	a	b	b	a	a	b	a
b	b	b	-	b	b	a	a	b	a
b	b	b	a	-	b	a	a	b	a
b	b	b	a	b	b	a	a	b	a
b	b	b	a	b	b	-	a	b	a
-	b	b	a	b	b	a	a	b	a
b	b	b	-	b	b	a	a	b	a
b	b	b	a	b	b	a	a	b	a
b	b	b	a	b	b	a	a	b	a

b	b	b	a	b	b	a	a	b	a
b	b	b	a	b	b	a	a	b	a
b	b	a	a	b	b	a	a	b	a
b	b	a	a	b	b	a	a	b	a
b	b	a	-	b	b	a	a	b	-
b	b	a	a	b	b	a	a	b	a
b	b	a	a	b	b	a	a	b	a
b	b	a	-	b	b	a	a	b	-
b	b	a	b	b	b	a	-	b	b
b	b	a	b	b	b	a	a	b	a
b	b	a	a	b	b	a	a	b	a
b	b	a	a	b	b	a	a	b	a
b	b	a	a	b	b	a	a	b	a
b	b	a	a	b	b	a	a	b	a
b	b	a	a	b	b	a	a	b	-
b	b	a	a	b	b	a	a	b	a
b	b	a	a	b	b	a	a	-	a
b	b	-	a	b	b	a	a	b	b
b	b	a	a	b	b	a	a	b	a
b	b	a	a	b	b	a	a	b	a
b	b	a	a	b	b	a	a	b	a
b	b	-	a	b	b	a	a	b	a
b	b	a	a	b	b	a	a	b	a
b	b	a	b	b	b	a	a	b	a
b	b	a	a	b	b	a	a	b	a
b	b	a	a	b	b	a	a	b	a
b	b	a	a	b	b	-	a	b	a
b	b	a	a	b	b	a	a	b	a
b	b	a	a	b	b	a	a	b	a
b	b	a	a	b	b	a	a	b	b
b	b	a	a	b	b	a	a	b	a
b	b	a	a	b	b	a	-	b	a
b	b	a	-	b	b	a	-	b	a
b	b	a	a	b	b	a	a	b	a
b	b	a	b	b	b	a	a	b	a
b	b	a	b	b	b	a	a	b	a
b	b	a	b	b	b	a	a	b	b
b	b	a	b	b	b	a	a	b	a
b	b	a	b	b	b	a	a	b	a
b	b	a	-	b	b	a	b	b	a
b	b	a	b	b	b	a	b	b	a
b	b	a	b	b	b	a	b	b	a
b	b	a	b	b	a	a	b	b	a
b	b	a	b	b	b	a	b	b	b
b	b	a	b	b	b	a	b	b	b
b	b	a	b	b	b	a	b	b	b
b	b	a	b	b	b	a	b	b	b

b	b	a	b	b	b	a	b	b	b
b	b	a	b	b	a	a	b	b	b
b	b	b	b	a	a	a	b	b	b
a	b	b	a	a	b	a	b	a	b
a	b	b	a	a	b	a	b	a	b
a	b	b	a	a	b	a	b	a	b
a	b	b	a	a	-	a	b	a	b
a	b	a	a	a	b	a	b	a	b
a	b	a	a	a	b	a	b	a	b
a	b	a	a	a	b	a	-	a	b
a	b	a	a	a	-	a	b	a	b
a	b	a	a	a	b	a	b	a	b
a	b	a	-	a	b	a	b	a	b
a	b	a	a	a	b	a	b	a	b
a	b	a	a	a	b	a	b	a	b
a	a	a	a	a	a	a	b	a	-
a	a	a	a	a	b	a	b	a	b
a	a	a	a	b	b	a	b	a	b
a	a	a	-	b	b	a	b	a	b
a	a	a	a	b	-	a	b	a	b
a	a	a	a	b	b	a	b	-	b
a	a	a	a	-	b	a	b	a	b
a	a	a	-	b	b	a	b	a	b
a	a	a	a	b	b	a	b	a	b
a	a	a	a	b	b	a	b	a	a
a	a	a	a	b	b	a	b	a	b
a	a	a	a	b	b	a	b	a	b
a	a	a	a	b	b	a	b	a	b
a	a	a	a	b	b	a	b	b	b
a	a	a	a	b	b	a	b	b	b
a	a	a	a	b	b	a	b	b	b
a	a	a	a	b	b	a	b	b	b
a	a	a	a	b	b	a	b	b	b
a	a	a	-	b	b	b	b	-	b
a	a	a	a	b	b	b	b	b	b
a	a	a	a	b	b	b	b	b	b
a	a	a	a	b	-	b	b	b	b
a	a	a	a	b	b	b	b	b	b
a	a	a	b	b	b	b	b	b	-
-	a	a	a	b	b	b	b	b	a
a	a	a	-	b	b	b	a	b	a
a	a	-	a	b	-	b	a	b	a
a	a	a	a	b	b	b	a	b	a
a	a	-	a	b	b	b	a	b	a
a	a	a	a	-	b	b	a	b	a
a	a	a	a	b	b	b	a	b	a
a	a	a	a	b	b	b	a	b	a

a	a	-	a	b	b	b	a	b	a
a	a	-	a	-	b	b	a	b	a
a	a	a	a	b	b	b	a	b	a
a	a	a	a	b	b	b	a	b	a
b	a	a	b	b	b	a	a	a	b
b	a	a	b	b	b	a	a	a	b
b	a	a	b	b	b	a	a	-	b
b	a	a	b	b	b	a	a	a	b
b	a	a	b	b	-	a	a	a	b
b	a	a	b	b	b	a	-	a	b
b	a	a	-	b	-	a	a	a	b
b	a	a	b	b	b	b	a	a	-
b	a	a	b	b	b	b	a	a	b
b	a	a	b	b	b	b	a	a	b
b	a	a	b	b	b	b	a	a	b
b	a	a	-	b	a	b	a	a	b
b	a	a	a	b	a	b	a	a	b
b	a	a	a	b	a	b	a	a	b
b	a	a	a	b	a	b	a	b	b
b	a	a	a	b	a	b	a	a	b
b	a	a	a	b	a	b	a	a	b
b	a	a	a	b	a	b	a	-	b
b	a	a	a	b	a	b	a	a	b
b	a	a	a	b	a	b	a	a	b
b	a	a	a	b	a	b	a	a	b
b	b	a	a	a	a	b	a	a	a
b	b	a	a	a	a	b	a	a	a
b	b	a	a	a	-	b	a	a	a
b	b	a	a	a	a	-	a	a	a
b	a	a	a	a	a	b	a	a	a
b	a	a	a	a	a	b	a	a	a
b	a	a	a	a	a	b	a	a	a
b	a	a	a	a	a	a	a	a	a
b	a	a	a	a	a	a	a	a	a
b	a	-	a	a	a	a	a	a	a
b	a	a	a	a	a	a	a	a	a
b	a	-	a	a	a	a	a	a	a
b	a	a	a	a	a	a	a	a	a
b	a	a	a	a	b	a	a	a	b
b	a	a	a	a	a	a	a	a	a
b	b	a	a	a	a	a	a	a	a
b	b	a	a	b	a	-	a	a	a
a	b	b	a	b	a	b	a	a	a
-	b	a	a	b	a	-	a	a	a
b	b	-	a	b	a	b	b	a	a
b	a	b	a	a	b	b	b	b	a
b	a	b	-	a	b	b	b	b	a

b	a	b	a	a	b	b	b	b	a
b	a	b	a	a	b	b	b	b	a
b	a	b	a	a	b	b	b	b	a
b	a	b	a	a	b	b	b	b	a
b	a	b	a	a	b	b	a	b	a
b	a	b	a	a	b	b	a	b	a
b	a	b	a	a	b	b	a	b	b
b	a	b	a	a	b	b	a	b	a
b	a	b	a	a	b	b	a	b	a
b	a	b	a	a	b	b	a	b	a
b	-	-	b	a	b	b	a	b	a
-	a	b	a	a	b	b	a	b	a
b	a	b	a	a	a	b	a	b	a
b	a	b	a	a	b	b	a	b	a
b	a	b	a	-	b	b	a	-	a
a	a	b	a	b	b	b	a	b	a
a	a	b	a	b	b	b	a	b	-
b	a	b	-	b	b	b	a	b	-
a	a	b	a	b	b	b	a	b	a
a	a	b	a	b	b	b	a	b	a
a	a	b	a	b	b	b	a	b	a
a	a	b	a	b	b	b	a	b	a
a	a	b	a	b	b	b	a	b	a
a	a	b	a	b	b	b	a	b	a
a	a	b	a	b	b	b	a	b	a
a	a	b	a	b	b	b	a	b	a
a	a	b	b	b	b	a	a	b	a
a	a	b	b	b	b	a	a	b	a
a	a	b	b	b	b	a	a	b	a
a	a	b	b	b	b	a	a	b	a
a	a	b	b	b	b	a	a	b	a
a	a	b	b	b	b	a	a	b	a
a	a	b	b	b	b	a	a	b	-
a	a	b	-	b	b	a	a	b	a
a	a	b	b	b	b	a	a	b	a
a	a	b	b	b	b	a	a	b	a
a	a	b	b	b	b	a	a	b	a
a	a	b	b	b	b	a	a	b	-
a	a	b	b	b	b	a	a	b	-
a	a	b	-	b	-	a	a	b	a
a	a	b	b	b	b	a	a	b	a
a	a	b	b	b	b	-	a	b	b
a	a	b	b	b	b	a	a	b	b
a	a	b	b	b	b	a	a	b	b
a	a	b	b	b	b	a	a	b	b
a	a	b	b	b	b	a	a	b	b
a	a	b	b	b	-	a	a	b	b

a	a	b	b	b	b	a	a	b	b
a	a	b	b	b	-	a	a	b	-
a	a	b	b	b	b	a	a	b	b
a	a	b	b	b	b	a	a	b	b
a	a	b	b	b	b	a	a	b	b
a	a	b	b	b	b	a	a	b	b
a	a	b	b	b	b	a	a	b	b
a	a	b	b	b	-	a	a	-	-
a	a	b	-	b	b	a	a	b	b
a	a	b	b	a	b	a	a	b	b
a	a	b	b	a	b	a	a	b	b
a	a	b	b	a	b	a	a	b	b
a	a	b	b	a	a	a	a	b	b
a	a	b	b	a	a	a	a	b	b
a	a	b	b	a	a	a	a	b	b
a	a	b	b	a	-	a	-	b	b
a	a	b	b	a	a	a	a	b	b
a	a	b	b	a	a	a	a	b	b
a	a	b	b	a	a	a	a	b	b
a	a	b	-	a	a	a	a	b	a
a	a	b	b	a	a	a	a	b	b
a	a	b	b	a	a	a	a	b	b
a	a	b	b	a	a	a	a	b	b
a	a	b	b	a	a	a	a	b	b
a	a	b	b	a	a	a	a	b	b
a	a	b	b	b	a	a	a	b	b
a	a	b	b	b	-	a	a	b	b
b	a	b	b	b	a	a	a	b	b
b	a	b	b	-	a	a	a	b	-
b	a	b	b	b	a	a	a	b	b
b	a	b	b	b	a	a	a	b	b
b	a	b	b	b	a	a	a	b	a
b	a	b	b	b	a	a	a	b	b
b	a	b	b	b	a	a	a	a	b
b	a	b	b	b	a	a	a	a	b
b	a	b	a	b	a	b	a	a	b
b	a	b	a	b	a	b	a	a	b
b	a	b	a	b	a	b	a	a	b
b	a	b	a	b	a	b	a	a	b
b	a	b	a	b	a	b	a	a	b
b	a	b	a	b	a	b	a	a	b
b	a	b	a	b	a	b	a	a	b
b	a	b	a	b	a	b	a	b	b
b	a	b	a	b	a	b	a	b	b
b	a	b	a	b	a	b	a	b	b
b	a	b	a	b	a	b	a	b	b
b	a	b	-	b	a	b	b	b	b

b	a	b	a	b	-	b	b	b	b
b	a	b	a	b	a	b	b	b	b
b	a	b	a	b	a	b	b	b	b
b	a	b	a	b	a	b	b	b	b
b	a	b	-	b	a	b	b	b	b
b	a	b	b	b	a	b	b	b	b
b	a	b	a	b	a	b	b	b	b
b	a	b	a	b	a	b	b	b	b
b	a	a	a	a	a	b	b	b	b
b	a	a	a	a	a	b	b	b	b
b	a	a	a	a	a	b	b	b	b
b	a	a	a	a	a	b	b	a	b
b	a	a	a	a	a	b	b	a	b
b	a	a	a	a	a	b	b	a	a
b	a	a	a	a	a	-	b	a	a
b	a	-	a	a	a	b	b	a	a
b	a	a	a	a	a	b	b	a	a
b	a	a	-	a	a	b	b	a	-
b	a	a	-	a	-	b	b	a	-
b	b	a	a	a	a	b	b	a	a
b	b	a	a	a	-	b	b	a	a
b	b	a	a	a	a	b	b	a	a
b	b	a	a	a	a	b	b	a	a
a	b	a	a	a	b	b	b	a	a
a	b	a	a	a	b	a	b	a	a
a	b	b	a	a	-	a	b	a	a
a	b	b	a	a	-	a	b	a	a
a	b	b	a	b	b	a	b	a	a
a	b	b	a	b	b	a	b	a	a
a	b	b	a	b	b	a	b	a	a
a	b	b	b	b	b	a	b	a	a
a	b	b	b	b	b	a	b	b	a
a	b	b	b	b	b	a	b	b	a
a	b	b	b	b	b	a	b	b	a
a	b	b	b	b	b	a	-	b	a
a	b	b	b	b	b	a	b	b	a
a	b	b	-	b	b	a	b	b	b
a	b	b	b	b	b	a	b	b	b
a	b	b	b	b	b	a	b	b	b
a	b	b	b	b	b	a	b	-	-
a	b	b	b	b	b	a	b	b	b
a	-	b	b	b	b	a	b	-	b
a	b	b	b	b	-	a	b	b	b
b	b	b	a	b	b	b	b	b	a
b	b	b	b	b	b	b	b	b	a
b	b	b	b	b	b	b	b	b	a
b	b	b	b	b	b	b	b	b	-

b	b	b	b	b	b	b	b	b	a
b	b	a	b	b	b	b	b	b	a
b	b	a	b	b	b	b	b	a	a
b	b	a	b	b	a	b	b	a	a
b	b	a	b	b	b	b	b	a	-
b	b	a	b	b	a	b	a	a	a
b	b	b	b	b	a	b	a	a	a
b	b	b	b	b	a	b	a	a	a
b	b	b	b	b	-	b	a	a	a
b	b	b	b	-	a	b	a	a	a
b	b	b	b	b	a	b	a	a	a
b	b	b	b	b	-	b	a	-	b
a	a	b	b	a	a	b	a	a	b
a	a	b	b	a	a	b	a	a	b
a	a	b	b	a	a	b	a	a	b
b	a	b	a	a	a	-	a	a	b
b	a	-	a	a	a	b	a	a	b
b	a	b	a	a	a	b	a	a	-
b	a	b	a	a	a	b	a	a	b
b	a	-	a	a	a	b	a	a	b
b	a	b	a	a	-	b	a	a	b
b	a	b	a	a	b	b	a	a	b
b	a	b	a	a	b	b	-	a	b
b	a	b	a	a	b	b	a	a	b
b	a	b	a	a	b	b	a	a	b
b	a	b	a	a	b	b	a	a	b
b	a	-	a	a	b	b	a	a	b
b	a	b	a	a	a	a	a	a	b
b	a	b	a	a	b	b	a	a	b
b	a	a	a	b	b	a	a	a	b
b	a	b	a	a	-	b	a	a	b
b	a	b	a	a	b	b	-	a	b
b	a	b	a	a	b	b	b	a	b
b	a	b	a	a	b	b	b	a	b
b	a	b	a	a	b	b	b	a	b
b	a	b	a	a	b	b	b	a	b
b	a	b	a	a	b	b	b	-	b
b	a	-	a	a	b	b	b	a	b
b	a	b	a	a	b	b	b	a	b
b	a	b	a	a	b	b	-	b	b
b	b	b	a	a	b	b	b	b	b
b	b	b	a	a	b	b	b	b	b
b	b	b	a	a	b	b	b	b	b
b	b	b	a	a	b	b	b	b	b
b	b	b	a	a	b	b	b	b	b
b	b	b	a	a	b	b	b	b	b
b	b	b	a	a	b	b	b	b	b

b	b	b	a	a	b	b	b	b	b
b	b	b	a	a	b	b	b	b	b
a	b	a	b	a	b	b	a	b	b
a	b	a	b	a	b	b	a	b	b
a	b	a	b	b	b	b	a	b	b
a	b	a	b	b	b	b	a	b	b
a	b	a	b	b	b	b	a	b	b
a	b	a	b	b	b	b	a	b	b
a	a	b	a	b	a	-	b	b	a
a	a	-	a	b	a	b	b	b	a
a	a	-	a	b	a	a	b	b	a
a	a	b	a	b	a	a	b	b	a
a	a	b	a	b	a	a	b	b	a
a	a	b	a	b	a	a	b	b	a
a	a	-	-	b	a	a	b	b	a
a	a	b	a	b	a	a	b	b	a
a	a	b	a	b	a	a	b	b	a
a	a	b	a	b	a	a	b	b	a
a	a	b	a	b	a	a	b	b	a
a	a	b	a	b	a	a	b	b	a
a	a	b	a	b	a	a	b	b	a
a	a	b	a	b	a	a	b	b	a
a	a	b	a	b	a	a	b	-	a
a	a	b	a	b	a	a	b	b	a
a	a	b	a	b	a	a	b	b	a
a	a	b	a	-	-	a	b	-	-
a	a	b	a	b	a	a	b	a	a
a	a	b	a	b	a	-	b	a	a
a	a	b	a	-	a	a	b	a	a
a	a	b	a	b	a	a	a	a	-
a	a	b	a	b	a	a	a	a	a
a	a	b	a	b	a	a	a	a	a
a	a	b	a	b	a	a	a	a	a
a	a	b	a	b	a	a	a	a	a
a	a	b	a	b	a	a	a	a	a
a	a	b	a	b	a	a	a	a	a
a	a	b	a	b	a	a	a	a	b
a	a	b	a	-	a	a	a	a	a
a	a	b	a	b	a	a	a	a	a
a	a	b	a	b	a	a	a	a	a
a	a	b	a	b	a	a	a	a	a
a	a	b	a	b	a	a	a	a	a
a	a	b	a	b	a	a	a	a	b
a	a	b	a	b	-	a	a	a	a
a	a	b	a	b	a	a	a	a	a
a	a	b	a	-	a	a	a	a	a
b	a	b	a	b	a	a	a	a	a

b	a	b	a	b	a	a	a	a	a
b	a	b	a	b	a	a	a	a	a
b	a	b	a	b	a	a	a	a	a
b	a	b	a	b	a	a	a	a	a
b	a	b	a	b	a	a	a	a	a
b	a	b	a	b	a	a	a	a	a
b	b	b	a	b	a	a	a	a	a
b	b	b	a	b	a	a	a	a	a
b	b	b	a	b	a	a	a	a	a
b	b	b	a	b	a	a	a	a	a
b	b	b	-	b	a	a	a	a	a
b	b	b	a	b	a	a	a	a	a
b	b	b	a	b	a	a	a	a	a
b	b	b	a	b	-	a	-	a	a
b	b	b	a	b	a	a	a	a	a
b	b	-	a	b	a	a	a	a	a
b	b	b	-	b	a	a	a	a	a
b	b	b	a	b	a	a	a	a	a
a	a	b	a	b	a	a	a	-	a
a	a	b	a	b	a	a	a	a	a
a	a	-	b	a	-	b	b	a	b
a	a	a	b	a	a	b	b	a	b
a	a	a	b	a	a	b	b	a	b
a	a	a	-	a	a	b	b	a	b
a	a	a	b	-	-	b	b	a	b
a	a	a	b	a	-	b	b	a	b
a	a	a	b	a	a	b	b	a	b
a	a	a	b	a	a	b	b	a	b
a	a	a	b	a	a	b	b	a	b
a	a	a	b	a	a	b	b	a	b
a	a	a	b	a	a	b	b	a	b
a	a	a	b	a	a	b	b	a	a
a	a	a	b	a	a	b	b	a	a
a	a	a	b	a	a	b	b	a	a
a	a	a	b	a	a	b	b	a	a
a	a	a	b	a	a	b	b	a	a
a	a	a	b	a	a	a	b	a	a
a	a	a	b	a	a	a	b	a	a
a	a	a	b	a	a	a	b	a	a
a	a	a	b	a	a	a	b	a	a
a	a	a	b	a	a	a	b	a	a
a	a	a	b	a	a	a	b	a	a
a	a	a	b	a	a	a	b	a	a
a	a	a	-	a	a	a	-	a	a
a	a	b	b	a	a	a	b	a	a
a	a	b	b	a	a	a	b	a	a
b	a	b	b	a	a	a	b	a	a

b	a	b	b	-	a	a	b	a	-
b	a	b	b	b	a	a	b	a	a
b	a	b	b	b	a	a	b	a	a
b	a	b	b	b	a	a	b	a	a
b	a	b	a	b	a	a	b	a	a
b	a	b	b	b	a	a	b	a	a
b	a	b	b	b	a	a	b	a	a
b	a	b	b	b	a	a	b	a	a
b	a	b	b	b	a	a	b	a	a
b	a	b	b	b	a	a	b	a	b
b	a	b	b	b	a	a	b	-	b
b	a	b	b	b	a	a	b	a	a
b	a	b	b	b	a	a	b	a	a
b	a	b	b	b	-	a	b	a	-
b	a	b	b	b	a	a	b	a	a
b	a	b	-	-	a	a	b	a	a
b	a	b	b	b	a	b	b	a	a
b	a	b	b	b	a	b	b	a	a
b	a	b	b	b	-	b	b	a	a
b	a	b	b	b	a	b	b	a	a
b	a	b	b	b	a	b	b	a	a
b	a	b	b	b	a	b	b	a	a
b	a	b	b	b	a	b	b	a	a
b	a	b	b	b	a	b	b	a	a
b	a	b	b	b	a	b	b	a	a
b	a	b	b	b	a	-	b	a	a
b	a	b	b	a	a	b	b	a	a
b	a	b	b	a	a	b	b	-	a
b	a	b	-	a	a	-	b	a	a
b	a	b	b	a	a	b	-	a	a
b	a	b	b	a	a	b	b	a	a
b	a	b	b	a	a	b	b	a	a
b	a	b	b	a	a	b	a	a	a
b	a	b	b	a	a	b	a	a	a
b	a	b	b	a	a	b	a	a	a
a	b	b	a	b	b	a	b	a	a
a	b	b	a	b	b	a	b	a	a
a	b	b	a	b	b	a	b	a	a
a	b	b	a	b	b	a	b	a	a
a	b	b	a	b	b	a	b	a	a
a	b	b	a	b	b	a	b	a	a
a	b	b	a	b	b	a	b	a	a
a	b	b	a	b	b	a	b	a	a
a	b	b	b	b	b	a	b	a	a
a	b	b	a	b	b	a	b	a	a
a	b	b	a	b	-	a	b	a	a
a	b	b	a	b	a	a	b	a	a

a	b	b	a	b	a	a	b	a	a
a	b	b	a	b	-	a	b	a	a
a	b	-	a	b	b	a	b	a	a
a	b	b	a	b	b	a	b	a	a
a	b	b	a	b	-	a	b	a	a
a	b	b	b	b	b	a	b	a	a
a	b	b	b	b	b	a	b	a	a
a	b	-	b	b	b	a	b	a	a
a	b	-	b	b	a	a	-	a	a
a	b	b	b	b	b	a	-	b	a
a	b	b	b	b	b	a	b	b	-
a	b	b	b	b	b	a	b	b	a
a	b	b	b	b	b	a	b	b	a
a	b	b	b	b	b	a	b	-	a
a	b	b	b	b	b	a	b	b	a
a	b	b	b	b	b	a	b	b	a
a	b	b	b	b	b	a	b	b	a
a	b	b	b	b	b	a	-	b	a
a	a	b	b	b	b	a	b	b	-
a	a	b	b	b	b	a	b	-	a
a	a	b	b	b	b	a	b	b	a
a	a	-	b	b	b	a	-	b	a
a	a	b	b	b	b	a	b	b	a
a	a	b	-	b	-	a	b	b	a
a	a	b	b	b	b	a	b	b	a
a	a	b	b	b	b	a	b	b	a
a	a	b	b	b	b	a	b	b	a
a	a	b	a	b	b	a	b	b	a
a	a	b	b	b	b	a	b	b	a
a	a	b	b	b	b	a	b	b	a
a	a	b	b	b	b	a	b	b	a
a	a	b	b	b	b	a	-	b	a
a	a	b	b	b	b	a	b	b	a
a	a	b	b	b	b	a	b	b	a
a	a	b	b	b	b	a	b	b	a
a	a	b	b	b	-	a	b	b	a
a	a	b	b	b	b	a	b	b	a
a	a	b	b	b	a	a	b	b	a
a	a	b	b	b	b	a	b	b	a
b	a	-	b	b	b	a	b	b	-
b	a	b	b	-	b	a	b	b	a
b	a	b	b	b	-	a	b	b	a
b	a	b	b	b	b	a	b	b	a
b	a	b	b	b	b	a	b	b	a
b	a	b	b	b	b	a	b	b	a
b	a	b	b	b	b	a	b	b	a
a	a	b	a	b	b	a	a	b	a

a	a	b	a	b	b	a	a	b	a
a	a	b	a	b	b	a	a	b	a
a	a	b	a	b	b	a	a	b	a
a	a	b	a	b	b	a	a	b	a
a	a	b	a	b	b	a	a	b	a
a	a	b	a	b	b	a	a	b	a
a	a	b	a	b	b	a	a	a	a
a	a	b	a	b	b	a	a	a	b
a	a	b	a	b	b	a	a	a	b
a	a	b	a	b	b	a	a	a	b
a	a	b	a	b	b	a	a	a	b
a	a	b	a	b	b	a	a	a	b
a	a	b	a	b	b	a	a	a	b
a	a	b	a	b	b	a	a	a	b
a	a	b	a	b	b	a	a	a	b
a	a	b	a	b	b	a	a	a	b
a	a	b	a	b	b	a	a	a	b
a	a	b	a	b	b	a	a	a	b
a	a	b	a	a	b	a	a	a	b
a	a	b	a	a	b	a	a	a	b
a	a	b	a	a	b	a	a	a	b
a	a	b	a	a	a	a	a	a	b
a	b	b	a	a	-	a	a	a	b
a	b	b	a	a	b	a	a	a	b
a	b	b	a	a	b	a	a	a	b
a	b	b	a	a	b	a	a	a	b
a	b	b	a	a	b	a	a	a	b
a	b	b	a	a	b	a	a	a	b
a	b	b	a	a	b	a	a	a	b
a	b	b	a	a	b	a	a	a	b
a	b	b	a	a	b	a	a	a	b
a	b	b	a	a	b	a	a	a	b
a	b	b	a	a	b	a	a	a	b
a	b	b	a	a	b	a	a	a	b
a	b	b	a	a	b	a	a	a	b
a	b	b	a	a	b	a	a	a	b
a	b	b	a	a	b	a	a	a	b
a	b	b	a	a	b	a	a	a	b
a	b	b	a	a	b	a	a	b	b
b	a	a	a	a	a	b	a	a	a
a	a	a	b	b	b	b	a	b	b
a	a	a	b	b	b	b	a	b	b
a	a	a	b	b	b	b	a	b	b
a	a	a	b	b	b	b	a	b	b
a	a	a	b	b	b	b	a	b	b
a	a	a	b	b	b	b	a	b	b

a	a	a	b	b	b	b	a	b	b
a	a	a	b	b	b	b	a	b	b
a	a	a	b	b	b	b	a	a	b
a	a	a	b	b	b	b	a	a	b
a	a	a	b	b	a	b	a	a	b

YW079	YW080	YW081	YW082	YW083	YW084	YW085	YW086	YW087	YW088
b	b	a	b	b	a	a	b	-	b
b	b	a	b	b	a	a	-	b	b
b	b	a	b	b	a	a	b	b	b
b	-	a	b	b	a	a	b	b	b
b	-	a	b	b	a	a	b	b	b
b	b	a	b	b	a	a	b	b	b
b	b	a	b	b	a	a	b	b	b
b	b	a	b	b	a	a	b	b	b
b	b	a	b	b	a	a	b	b	b
b	b	a	b	b	a	a	b	b	b
b	-	a	b	b	a	a	b	b	b
b	b	a	b	b	a	a	b	b	b
a	b	a	b	-	a	a	b	b	b
b	b	a	b	b	a	a	b	b	b
b	b	a	b	b	a	a	b	b	b
b	b	a	b	b	a	a	b	b	b
b	b	a	b	b	a	-	b	b	b
b	b	a	b	b	a	a	b	b	b
b	b	a	b	b	b	-	b	b	b
b	b	a	b	b	a	b	b	b	b
b	b	a	b	b	a	a	b	b	-
b	b	a	b	b	a	a	b	b	b
b	b	a	b	b	-	a	b	b	b
b	b	a	b	b	a	a	b	b	b
b	b	a	b	b	a	a	b	b	b
b	b	a	b	b	a	a	b	b	b
b	b	a	b	b	a	a	b	b	b
b	b	a	b	b	a	a	b	b	b
b	b	a	b	b	a	a	b	b	b
b	b	a	b	b	a	a	b	a	b
b	b	a	b	b	a	a	b	a	b
b	b	a	b	b	a	a	b	a	b
b	b	a	b	b	a	a	b	a	b
a	b	a	b	b	a	a	b	a	a
-	b	a	b	b	a	b	b	a	-
a	b	a	b	b	a	b	b	-	a
a	b	a	b	b	a	b	b	a	a
a	b	a	b	b	-	b	b	a	a
a	b	a	b	b	a	b	b	a	a
a	b	a	b	b	a	b	b	a	a
a	b	a	b	b	a	b	b	a	a
a	-	a	b	b	a	b	a	a	a
a	b	a	b	b	a	b	a	a	a
-	b	a	b	b	a	b	a	a	-
a	b	a	a	b	-	b	-	a	a

a	b	a	a	b	-	b	a	a	a
a	-	a	a	b	a	b	a	a	a
a	b	a	a	b	a	b	a	a	a
a	b	a	a	b	a	b	a	a	a
a	b	a	a	b	a	b	a	a	a
a	b	a	a	b	a	b	a	a	a
a	b	a	a	b	b	b	a	-	a
a	b	a	a	b	-	b	a	b	a
a	b	a	a	b	a	b	a	b	a
a	b	a	a	b	a	b	a	b	a
a	b	a	a	b	a	b	a	b	a
a	b	a	a	b	a	b	a	b	a
a	a	a	a	b	a	-	a	b	a
-	b	a	a	a	a	b	a	b	-
-	b	a	a	a	-	a	a	b	a
a	b	a	a	a	-	a	a	b	a
a	b	a	a	a	a	a	a	b	a
a	a	a	a	a	a	a	a	b	a
a	b	a	a	a	a	a	a	b	a
a	b	a	a	a	a	a	a	b	a
-	b	a	a	a	a	a	a	b	a
a	b	a	a	a	a	a	a	b	a
a	b	a	a	a	a	a	a	b	a
a	b	a	a	a	a	a	a	b	a
a	b	a	a	a	a	a	a	b	a
a	b	a	a	a	a	a	a	b	a
a	b	a	a	a	a	a	a	b	a
a	b	a	a	a	a	a	a	b	a
a	b	a	a	-	a	a	-	b	a
a	b	a	a	a	a	a	a	b	a
a	b	a	a	a	a	a	a	b	a
a	b	a	a	-	b	a	a	b	a
a	b	a	a	a	a	a	a	b	a
a	-	a	a	a	a	a	a	b	a
a	b	a	a	a	a	a	a	b	a
a	b	a	a	a	a	a	a	b	a
a	b	a	a	a	a	a	a	b	a
a	b	a	a	a	a	a	a	b	a
a	b	a	a	a	a	a	a	-	a
a	a	a	a	b	b	b	b	b	b
a	a	a	a	b	b	b	b	b	b
a	a	a	a	b	b	b	a	b	b
a	a	a	a	b	b	b	a	b	b
a	a	a	a	a	b	b	a	b	b
a	-	a	a	a	b	b	a	b	b
a	a	a	a	a	b	b	a	b	b
a	a	a	a	a	a	b	a	b	b

a	a	a	a	a	b	b	a	b	b
a	a	a	a	a	b	b	a	b	b
a	a	a	a	a	a	b	a	b	b
a	a	a	a	a	a	b	a	b	b
a	a	a	a	a	b	b	a	b	b
a	a	a	a	a	b	b	a	b	a
a	a	a	a	a	a	b	a	b	a
a	a	a	a	a	b	b	a	b	a
a	a	a	a	a	b	-	a	b	a
-	a	a	a	a	b	b	a	b	-
a	a	a	a	a	b	b	a	b	a
a	a	a	b	a	b	b	a	b	-
a	a	a	b	a	b	b	a	b	a
a	a	a	b	a	b	b	a	b	a
a	a	a	b	a	b	b	a	b	a
a	a	a	b	a	b	b	a	b	a
a	a	a	b	a	b	b	a	b	a
a	a	a	b	a	b	b	a	b	a
a	a	a	b	a	b	b	a	b	a
a	a	a	b	a	a	b	a	b	a
-	a	a	b	a	b	b	a	b	a
a	a	a	b	a	b	-	-	b	a
a	a	a	b	a	b	b	-	b	a
a	a	a	b	a	b	b	a	b	a
-	b	a	b	a	b	b	a	b	a
a	a	a	b	a	b	b	a	b	a
a	a	a	b	a	b	b	a	b	a
a	a	a	b	a	b	b	a	b	a
b	a	a	b	a	b	b	a	b	a
b	a	a	b	a	b	b	a	b	a
b	a	a	b	a	b	b	a	b	a
b	a	a	b	a	b	b	a	b	a
b	a	a	b	a	a	b	a	b	a
b	a	a	b	a	a	b	a	b	a
b	a	a	b	a	b	b	a	b	a
b	a	a	b	a	b	b	a	b	a
b	a	a	b	a	b	b	a	b	a
b	a	a	b	a	b	b	a	b	a
b	a	a	b	a	b	b	a	b	a
b	a	a	b	a	b	b	a	b	a
b	a	a	b	a	b	b	a	b	a
b	a	a	b	a	b	b	a	b	a
b	a	a	b	a	b	b	a	b	a
b	a	a	b	a	b	b	a	b	a
b	b	a	b	a	b	b	a	b	a
b	a	a	b	a	b	b	a	b	a
b	a	a	b	a	b	b	a	b	-

b	a	a	b	-	b	b	a	b	a
-	a	a	b	a	-	b	a	b	a
b	-	a	b	b	b	b	a	b	-
b	a	a	b	b	b	b	a	b	a
b	a	a	b	b	-	b	a	b	a
b	a	a	b	b	b	b	a	b	a
b	a	a	b	b	b	-	a	b	a
b	a	a	b	b	b	b	a	a	a
b	a	a	b	b	b	b	a	a	a
b	a	a	b	b	b	b	a	a	a
b	a	a	b	b	b	b	a	a	a
b	a	a	b	b	b	b	a	a	a
b	a	a	b	b	b	b	a	a	a
b	a	a	b	b	a	b	a	a	a
b	a	a	b	b	b	b	a	a	a
b	a	a	b	b	b	b	a	a	a
b	a	a	b	b	b	b	a	a	a
b	a	a	b	b	b	b	a	a	a
b	a	a	b	b	b	b	a	a	a
b	a	a	b	b	b	b	-	a	a
b	-	a	b	b	b	b	b	a	a
b	a	a	b	b	b	b	b	a	-
-	a	a	b	-	b	b	b	a	a
b	a	a	b	b	a	b	b	a	a
b	a	a	b	b	b	a	a	a	a
b	a	a	b	b	b	a	a	a	a
-	a	a	b	b	b	a	b	a	a
b	a	a	b	b	b	a	b	a	a
a	a	a	b	b	b	a	b	a	a
b	a	a	b	b	b	a	b	a	a
b	-	a	b	b	b	a	b	a	a
b	a	a	b	b	b	a	b	a	a
b	a	a	b	b	b	a	b	a	a
-	a	a	b	-	b	a	b	a	a
b	a	b	b	b	b	a	b	a	a
b	a	b	b	b	b	a	b	a	a
b	a	b	b	b	b	a	b	a	a
b	a	b	b	b	b	a	b	a	a
b	-	b	b	b	b	a	b	a	a
b	a	b	b	b	a	a	b	a	a
b	a	b	b	b	a	a	b	a	a
b	a	-	b	b	a	a	b	a	a
b	a	b	b	-	b	a	b	a	a
b	a	b	b	b	-	a	b	a	a
b	a	b	b	b	b	a	b	a	a
b	a	b	-	b	b	a	b	a	b
b	a	b	b	b	b	a	b	a	b

b	a	b	b	b	b	a	b	a	b
b	a	b	b	b	b	a	b	a	b
b	a	b	b	a	b	a	b	a	b
b	a	b	b	a	b	a	b	a	b
b	a	b	b	a	b	a	b	a	b
b	a	b	b	a	b	a	b	a	b
b	a	b	b	a	b	a	b	a	b
b	a	b	b	a	b	a	b	a	b
b	a	b	b	a	b	a	b	a	b
b	a	b	b	a	b	a	b	a	b
b	a	b	b	a	b	a	-	a	b
b	a	b	b	a	b	a	b	a	b
b	a	b	a	a	b	b	b	a	b
b	a	b	a	a	a	b	b	a	b
b	a	b	a	a	a	b	b	a	b
b	a	b	a	a	a	b	b	a	b
b	a	b	a	a	a	b	b	a	b
b	a	b	a	a	a	b	b	a	b
b	a	b	a	a	a	b	-	a	b
b	a	b	a	a	a	b	b	a	b
b	a	b	a	a	a	b	b	a	b
b	a	b	a	a	-	b	b	a	b
b	-	b	a	a	b	b	b	a	b
b	a	b	a	a	a	b	b	a	b
b	a	b	-	-	a	b	b	a	b
b	a	b	a	a	a	b	b	a	b
-	a	b	a	a	a	b	b	a	b
b	a	b	a	a	a	b	b	a	b
b	a	b	a	a	a	b	b	a	b
b	a	b	a	a	a	b	b	a	b
b	a	b	a	a	a	b	-	a	-
b	b	b	b	a	a	b	b	a	b
b	b	a	b	a	a	a	b	a	b
b	b	a	b	a	a	b	b	a	b
b	b	a	b	a	a	b	b	a	b
-	a	a	b	a	a	b	b	a	b
b	b	a	b	a	a	b	b	a	b
b	b	a	b	a	-	b	b	a	b
a	b	a	b	a	-	b	b	a	b
a	b	a	b	-	b	b	b	-	b
a	b	a	b	a	a	b	b	a	b
a	b	a	b	a	a	b	b	a	-
a	b	a	b	a	a	b	b	a	a
a	b	a	b	a	a	b	b	a	a
a	b	a	b	a	a	b	b	a	a
a	b	a	b	a	a	b	b	a	a
a	b	a	b	a	a	b	b	a	a

a	b	a	b	a	a	b	b	a	a
a	b	a	b	a	a	b	b	a	a
a	-	a	b	a	a	b	a	a	a
a	b	a	b	a	-	b	a	a	a
a	b	a	b	a	a	b	a	a	a
a	b	a	b	a	a	b	a	a	a
a	b	a	b	a	a	b	a	a	a
a	b	a	b	a	a	b	a	a	a
a	b	a	b	a	a	b	a	-	a
a	b	a	b	a	a	b	b	a	a
a	b	a	b	a	a	b	b	a	a
a	-	a	b	a	a	b	b	b	a
a	b	a	b	a	a	b	b	b	a
a	b	a	b	a	a	b	b	b	a
a	-	a	b	a	a	b	-	b	b
a	b	a	b	a	a	b	b	b	b
a	b	a	b	a	a	b	b	b	b
a	b	a	b	a	a	b	b	b	b
a	b	a	b	a	a	b	b	-	-
a	b	a	b	a	a	b	b	b	b
a	b	a	b	a	a	b	b	b	b
a	b	a	b	a	a	b	b	b	b
a	b	a	b	a	a	b	b	b	b
a	b	a	b	a	a	b	b	b	b
a	b	a	b	a	a	b	b	b	a
a	b	a	b	a	a	b	b	b	a
a	-	a	b	a	a	b	b	b	a
a	b	a	b	a	a	b	b	b	a
a	a	a	b	a	a	b	b	-	a
a	b	a	b	a	a	b	b	b	a
a	b	a	b	a	a	b	b	b	a
a	b	a	-	a	a	b	b	b	a
a	b	a	b	a	a	b	b	b	a
a	b	a	b	a	a	b	b	b	a
a	b	a	b	a	a	b	b	b	a
a	b	a	b	a	a	b	b	b	a
a	b	a	b	a	a	b	b	b	a
a	b	a	-	a	a	b	b	b	a
a	b	a	b	a	a	b	b	b	a
a	-	a	b	a	a	b	b	b	a
a	b	a	b	a	a	b	b	b	a
a	b	a	b	a	a	b	b	b	a
a	-	a	b	a	a	b	b	b	a
a	b	a	b	a	a	b	b	b	a
a	b	a	b	a	a	b	-	b	a
a	b	a	b	a	a	b	-	-	a
a	b	a	b	a	a	b	b	-	a

a	b	a	a	a	a	b	b	b	a
a	b	-	a	a	b	b	b	b	a
a	b	a	a	a	b	b	b	b	a
a	b	a	a	a	b	b	b	b	a
a	b	a	a	a	b	b	b	b	a
a	b	a	a	a	b	b	b	b	a
a	b	a	a	a	b	b	b	b	a
a	b	a	a	a	b	b	b	b	-
a	b	a	a	a	b	b	b	-	a
-	b	a	a	a	b	b	b	b	a
a	b	a	a	a	a	b	-	b	a
a	b	a	a	a	a	b	b	b	a
a	b	a	a	a	b	b	b	b	a
a	b	a	a	a	b	b	b	b	a
a	b	a	a	a	-	b	b	b	a
a	b	a	a	a	b	b	b	b	a
a	b	a	a	a	b	b	b	b	a
a	b	a	a	a	b	b	b	b	a
-	b	a	a	a	b	b	b	b	a
a	b	a	a	a	-	b	b	b	a
a	b	a	a	a	b	b	b	b	a
a	b	a	a	a	b	b	b	b	a
a	b	a	a	a	b	b	b	b	a
-	b	a	a	a	b	b	b	b	a
a	b	a	a	a	b	b	b	b	a
a	b	a	a	a	a	b	b	-	a
a	b	a	a	a	b	b	b	b	a
a	b	a	a	a	b	b	b	b	a
a	b	a	a	a	a	b	b	b	a
a	b	a	a	a	b	b	b	b	a
a	b	a	a	a	a	b	b	b	a
a	b	a	a	a	b	b	b	b	a
a	b	a	a	a	b	b	b	b	a
a	b	a	a	a	b	b	b	b	a
a	b	a	a	a	a	b	-	b	a
a	-	a	a	a	b	b	b	b	a
a	b	a	a	a	b	b	b	b	a
a	b	a	a	a	b	b	b	b	a
a	a	a	a	a	a	-	a	a	a
a	a	a	a	a	a	b	a	a	a
a	a	a	a	a	a	b	a	a	a
a	a	a	a	a	a	b	a	a	a
-	-	a	-	a	b	b	a	a	a
a	a	a	a	a	b	b	a	a	a
a	a	a	a	a	b	b	a	a	a
a	a	a	a	a	b	b	a	a	a
a	a	a	a	a	-	b	a	a	a

a	a	a	b	a	b	b	a	a	a
a	b	a	b	a	b	-	a	a	a
a	b	a	b	a	b	b	a	a	a
a	b	a	b	a	b	b	a	a	a
a	b	a	b	a	b	b	a	a	a
a	b	a	b	a	b	b	a	a	a
a	b	a	b	a	b	-	a	a	a
a	b	a	b	a	b	b	a	a	a
a	b	a	b	a	b	b	a	a	a
a	b	a	b	a	b	b	a	a	a
a	b	a	b	a	b	b	a	a	a
a	b	a	b	a	b	b	a	a	a
a	b	a	b	a	b	b	a	a	a
a	-	a	b	a	b	b	a	a	a
a	b	a	b	a	b	b	a	a	a
a	-	a	b	a	b	b	a	a	a
-	b	a	b	a	b	b	a	a	a
a	a	a	b	b	a	b	a	a	a
a	b	a	b	b	b	b	a	a	a
a	b	a	b	b	b	b	a	a	a
a	b	a	b	b	b	-	b	a	a
a	a	a	b	b	-	b	b	a	a
a	a	a	b	b	b	b	b	a	a
a	a	a	b	b	b	b	b	a	a
a	a	a	b	b	b	b	b	a	a
-	a	a	b	b	b	b	b	a	a
a	a	a	b	b	b	b	b	a	a
a	a	a	b	b	b	b	b	a	a
a	a	a	b	b	b	b	b	a	a
a	a	a	b	b	b	b	b	a	a
a	a	a	b	b	b	b	b	a	a
a	a	a	b	b	b	b	b	a	a
a	-	a	b	b	b	b	b	a	a
a	a	a	b	-	b	b	b	a	a
a	a	a	b	b	b	b	b	a	a
a	a	a	-	b	a	-	b	a	a
a	a	a	-	b	b	-	a	a	b
a	a	a	b	b	a	b	a	a	b
a	a	a	b	b	b	b	a	a	b
a	a	a	b	b	b	b	a	a	b
a	a	a	-	b	-	b	a	a	b
a	a	a	b	b	b	-	a	a	b
a	a	a	b	b	b	b	a	a	b
a	a	a	b	b	b	b	a	a	b
a	a	a	b	b	b	b	a	-	b
a	a	a	b	-	-	b	a	a	b
a	a	a	b	b	a	b	a	a	b

a	a	a	b	b	a	b	a	a	b
a	a	a	b	b	a	b	a	a	b
a	a	a	b	b	a	a	a	a	b
a	a	a	b	b	a	a	a	a	b
a	a	a	b	b	a	a	a	a	b
a	a	a	b	a	a	a	a	a	b
a	a	a	b	a	a	a	a	a	b
a	a	a	b	a	a	a	a	a	b
a	a	a	b	a	a	a	a	a	b
a	a	a	b	a	a	a	a	a	b
a	a	a	b	a	a	a	a	a	-
a	a	a	b	a	a	a	a	a	b
a	a	a	b	a	b	a	a	a	b
a	a	a	b	a	a	a	a	a	b
a	a	a	b	a	a	a	a	a	b
a	a	a	b	a	a	a	a	a	b
a	a	a	b	a	a	a	a	a	b
a	a	a	b	a	-	a	a	a	b
a	a	a	b	a	a	a	a	a	b
a	a	a	b	a	a	a	a	a	b
a	a	a	b	a	a	a	a	a	b
a	a	a	b	a	a	a	a	a	-
a	a	a	b	a	a	a	a	a	b
a	a	a	b	a	a	a	-	a	b
a	b	a	b	a	a	a	a	a	b
a	b	a	b	a	a	a	a	a	b
a	b	a	b	a	a	a	a	a	b
a	b	a	b	a	a	a	a	a	b
a	b	a	b	a	a	a	a	a	b
a	b	a	b	a	a	a	a	a	b
a	b	a	b	a	a	a	a	a	b
a	-	a	b	a	a	a	a	a	b
a	b	a	b	a	-	a	a	a	b
a	b	a	b	a	b	a	a	a	b
a	b	a	b	a	a	a	a	a	b
a	b	a	b	a	a	a	a	a	b
a	b	a	b	a	a	a	a	a	b
a	b	a	b	-	a	a	-	a	b
a	b	a	b	a	-	a	a	a	b
a	b	a	b	a	b	a	a	a	b
a	a	a	a	b	a	b	b	a	b
a	a	a	a	b	a	b	b	a	b
a	a	a	a	b	a	b	b	a	b
a	a	a	a	b	a	b	b	a	b
a	-	a	a	b	a	b	b	a	b
a	a	a	a	b	a	b	a	a	b
a	a	a	a	b	a	b	b	a	b

a	a	a	a	b	a	b	b	a	b
a	a	a	a	b	a	-	-	a	b
a	a	a	a	b	a	b	b	a	b
a	a	a	a	b	a	b	b	a	b
a	a	a	a	b	a	b	b	a	b
a	b	a	a	b	a	b	b	a	b
a	b	a	a	b	a	b	b	a	b
a	b	a	a	b	a	b	b	a	b
a	b	a	a	b	a	b	b	a	b
a	-	a	a	b	a	b	b	a	b
a	b	a	a	b	b	b	b	a	b
a	b	a	a	b	a	b	b	a	b
a	b	a	a	b	a	b	b	a	b
a	b	a	-	b	a	b	b	a	b
a	b	a	a	b	a	b	b	a	b
a	b	a	a	b	a	b	b	a	b
b	b	a	a	b	a	b	b	a	b
a	b	a	a	b	b	b	b	a	b
a	b	a	a	b	b	b	b	-	b
-	b	a	a	b	b	b	b	a	b
a	b	a	a	b	b	b	b	a	b
-	b	a	a	b	b	b	b	a	b
a	b	a	a	b	b	b	b	a	b
a	b	a	a	b	b	b	b	a	b
a	b	a	a	b	b	b	b	a	b
a	b	a	a	a	b	b	b	a	b
a	b	a	a	a	b	b	b	a	b
a	b	a	a	a	b	b	b	a	b
a	b	a	a	a	b	b	b	a	b
a	b	a	a	a	b	b	b	a	b
a	a	a	a	a	b	b	b	a	b
a	a	a	a	a	b	b	b	a	-
a	a	a	a	a	a	b	a	a	b
a	a	a	a	a	b	b	b	a	b
a	a	a	a	a	b	b	b	a	b
a	a	a	a	a	a	b	b	a	b
-	a	a	a	a	b	b	b	a	b
a	a	a	a	a	b	b	a	a	b
a	-	a	a	a	b	b	a	a	b
a	a	b	-	a	b	b	a	a	b
a	a	b	a	a	b	b	a	a	b
a	a	b	a	a	b	b	a	a	b
a	a	b	a	a	b	b	a	a	b
a	a	b	a	a	b	b	a	a	b
a	a	b	a	a	b	b	a	a	b
a	a	b	a	a	b	b	a	a	b
a	a	b	a	a	b	b	a	a	b

a	a	b	a	a	b	b	a	-	b
a	a	b	a	a	b	b	a	a	b
a	a	b	a	a	b	b	a	a	b
a	b	b	a	a	b	b	a	a	b
a	b	b	a	a	b	a	a	a	b
a	b	b	a	a	a	a	a	a	-
a	b	b	a	a	b	a	-	-	b
a	b	b	a	a	b	a	a	a	b
a	b	b	a	a	b	a	a	a	b
a	b	b	a	a	b	a	a	a	b
a	b	b	a	a	b	a	a	a	b
a	b	b	a	a	a	a	a	a	b
a	b	b	a	a	b	a	a	a	b
a	b	b	a	a	b	a	a	a	-
a	b	b	a	a	b	a	a	a	b
a	b	b	a	a	b	a	a	a	b
a	b	b	a	a	b	a	a	a	b
a	b	b	a	a	b	a	a	a	b
a	b	b	a	a	b	a	a	a	b
a	b	b	a	a	b	a	a	a	b
a	b	b	a	a	b	a	-	a	b
a	b	b	a	a	b	a	a	a	b
a	b	b	a	a	b	a	a	a	b
a	b	b	a	a	b	a	a	a	b
a	b	b	a	a	b	a	a	-	b
a	b	b	a	a	b	a	a	a	b
a	a	b	a	a	b	a	a	a	b
a	a	b	a	a	b	a	-	a	b
a	a	b	a	a	b	a	a	a	b
b	a	b	a	a	b	a	a	-	b
a	a	b	a	a	b	a	a	a	b
a	-	b	a	a	b	a	-	a	a
a	a	b	a	a	b	a	a	a	a
a	a	b	a	a	b	a	a	a	a
a	a	b	a	a	b	a	-	a	a
a	a	b	a	a	b	a	a	a	a
a	a	b	a	a	b	a	a	a	a
a	a	b	a	a	b	a	a	a	a
a	a	b	a	a	b	a	a	a	a
a	a	b	a	a	b	a	a	a	a
a	a	b	a	a	b	a	a	a	a
a	b	b	a	a	b	a	a	a	a
a	b	b	a	a	-	a	a	b	a
a	b	b	a	a	b	a	a	b	a
a	b	b	a	a	b	a	a	b	a
a	b	b	a	a	b	a	a	b	a
a	b	b	a	a	a	a	a	b	a

a	b	b	a	a	b	a	a	b	a
a	b	b	a	a	b	b	a	b	a
a	b	b	a	a	b	b	-	b	a
-	b	b	a	a	b	a	a	b	a
-	b	b	a	a	b	a	a	b	a
a	b	b	a	a	b	b	a	b	-
a	b	b	a	a	b	b	a	b	a
a	b	b	a	a	b	b	a	b	a
b	b	b	a	a	b	b	a	b	a
a	b	b	a	a	b	b	a	b	a
a	b	b	a	a	b	b	a	b	a
-	a	b	a	a	b	b	a	b	a
a	a	b	a	a	b	b	a	b	a
a	a	b	a	a	a	b	a	b	a
a	a	b	a	a	b	b	-	b	a
a	a	-	a	a	b	b	a	b	a
a	a	b	a	a	b	b	a	b	a
a	b	b	a	a	b	b	a	b	a
a	b	b	a	a	b	b	a	b	a
b	b	b	b	a	a	b	b	a	b
b	b	b	b	a	b	b	b	a	b
b	b	b	b	a	b	b	b	a	b
b	-	b	b	a	b	b	b	a	b
b	b	b	b	a	b	b	b	a	b
b	-	b	b	a	b	b	b	a	b
b	-	b	b	a	b	b	b	a	b
b	b	b	b	a	-	b	b	a	b
b	b	b	b	a	b	b	b	a	b
b	b	b	b	a	b	b	b	a	b
b	b	b	b	a	b	b	b	a	b
b	b	b	b	a	b	a	b	a	b
b	-	b	b	a	a	a	b	a	b
b	b	b	b	a	b	a	b	a	b
b	a	b	b	a	b	a	b	a	b
b	b	a	b	a	b	a	b	a	b
b	b	a	b	a	b	a	b	a	b
b	b	a	a	a	a	a	b	a	-
b	b	a	a	a	b	a	b	a	b
b	-	a	a	a	b	a	b	a	b
b	b	a	a	a	b	a	b	a	b
b	b	a	a	a	b	a	b	a	b
b	b	a	a	a	b	a	b	a	b
b	b	a	a	a	b	a	b	a	b
b	b	a	a	a	b	a	b	a	b
b	b	a	a	a	-	a	b	a	b
b	b	a	a	a	b	a	b	a	b
b	b	a	a	a	b	a	b	a	b

b	b	a	a	a	b	a	b	a	b
-	-	a	a	a	b	a	b	a	b
b	b	a	a	a	b	a	b	a	b
b	b	a	a	a	b	a	b	a	b
b	b	a	a	a	b	a	b	a	-
b	-	a	a	a	a	a	b	a	b
b	b	a	a	a	b	a	b	a	b
a	b	a	a	a	a	a	b	a	b
a	b	a	a	a	b	a	b	a	b
-	b	a	a	a	b	a	b	-	b
a	b	a	a	a	b	a	b	a	b
a	b	a	a	a	b	a	b	a	b
a	b	a	a	a	b	a	b	a	b
a	b	a	a	a	b	a	b	a	b
a	b	a	a	a	b	-	b	a	b
a	b	a	a	a	b	a	b	a	b
a	b	a	a	a	b	a	b	a	b
a	-	a	a	a	a	a	b	a	b
a	b	a	a	a	b	a	b	a	b
a	b	a	a	a	b	a	b	a	b
a	b	a	a	a	b	a	b	a	b
a	b	a	a	a	b	a	b	a	b
a	b	a	a	a	b	a	-	a	b
a	-	a	a	a	b	a	a	a	b
a	a	a	a	a	b	a	a	a	b
a	a	a	a	a	b	a	a	a	b
a	a	a	a	a	b	a	a	a	b
a	a	a	a	a	b	a	a	a	b
a	a	a	a	a	b	a	a	a	b
a	a	a	a	a	b	a	a	a	b
a	a	a	a	a	b	a	a	a	b
a	a	a	a	a	b	a	a	a	b
a	a	a	a	a	b	a	a	-	b
a	a	a	a	a	b	a	a	a	b
a	a	a	a	a	b	a	a	a	-
a	a	a	a	b	b	a	a	a	b
a	b	a	a	a	b	a	a	a	b
a	b	a	a	a	b	a	a	a	b
a	b	a	a	a	-	a	a	a	b
a	b	a	a	a	a	a	a	a	b
a	b	a	a	a	b	a	a	a	b
a	b	a	a	a	b	a	a	a	b
a	b	a	a	a	b	a	a	a	b
a	b	a	a	a	b	a	a	a	b
a	b	a	a	a	b	a	a	a	-
a	b	a	a	a	b	a	a	a	b
a	b	a	a	a	b	a	a	-	b

a	b	a	a	a	-	a	a	a	b
a	b	a	a	a	b	a	a	a	b
a	b	a	a	a	b	a	a	a	b
a	b	a	a	a	b	a	a	a	b
a	b	a	a	a	b	a	a	a	-
a	-	a	a	a	b	a	a	a	b
a	b	a	a	a	b	a	a	a	b
a	b	a	a	a	b	a	a	a	b
a	b	a	a	a	b	a	a	a	b
a	-	a	a	a	a	a	a	a	b
a	-	a	a	a	b	a	a	a	b
a	a	b	a	b	b	a	a	b	a
a	-	b	a	b	b	a	a	b	a
a	a	b	a	b	b	a	a	b	a
a	a	b	a	b	b	a	a	b	a
a	a	b	a	b	b	a	a	b	a
a	a	b	a	b	b	a	a	b	a
a	a	b	a	b	-	a	a	b	a
a	a	b	a	b	b	a	a	b	a
a	a	b	a	b	b	a	a	b	a
a	a	b	a	b	b	a	a	b	a
a	a	b	a	b	b	a	a	b	a
a	a	b	a	b	-	a	a	b	a
a	a	b	a	b	a	a	a	b	a
a	-	b	a	b	a	a	a	b	a
a	a	b	a	b	-	a	a	b	a
a	a	b	a	b	-	a	a	b	a
a	a	b	a	b	a	a	a	b	a
a	a	b	a	b	a	a	a	b	-
a	a	b	a	b	a	a	a	b	a
a	a	b	a	b	a	a	a	b	a
a	a	b	a	b	a	a	-	b	a
a	a	b	a	b	-	a	a	b	a
a	-	b	a	b	a	-	a	a	-
a	a	b	a	b	a	a	a	a	a
a	a	b	a	b	a	a	a	a	a
a	a	b	a	b	a	a	a	a	a
a	a	b	a	b	a	a	a	-	a
a	a	b	a	b	a	a	a	a	a
a	a	b	a	b	a	a	a	a	a
a	a	b	a	b	a	a	a	a	a
a	a	b	a	b	a	-	a	a	a
a	a	b	a	b	a	a	a	a	a
-	a	b	a	b	a	a	a	a	a
a	a	b	a	b	a	-	a	a	a
a	a	b	a	b	a	a	a	a	a
a	a	b	a	b	a	a	a	a	a

a	a	b	a	b	a	a	a	a	a
a	a	b	a	b	a	a	a	a	a
a	a	b	a	b	a	a	a	a	a
a	a	b	a	b	a	a	a	a	a
a	-	b	a	b	-	a	a	a	a
a	a	b	a	b	a	a	a	a	a
a	a	b	a	a	a	a	a	a	a
a	a	b	a	a	a	a	a	a	a
a	a	b	a	a	a	a	a	a	a
a	a	b	a	a	a	a	a	a	a
a	a	b	a	a	a	a	a	a	a
a	a	b	a	a	a	a	-	a	a
a	a	b	a	a	a	a	a	a	a
a	a	b	a	a	a	a	a	a	a
a	a	b	a	a	a	a	a	a	b
a	a	b	a	a	a	a	a	a	b
a	a	b	a	a	a	a	a	a	b
a	-	b	a	a	a	-	a	a	b
a	a	b	a	a	a	a	a	a	b
a	a	b	a	a	a	a	a	a	b
a	a	b	-	a	-	a	a	a	b
a	a	b	a	a	a	a	a	a	b
a	a	b	a	a	a	a	a	a	b
a	a	b	a	a	a	a	a	a	b
a	a	b	a	a	b	a	a	a	b
a	a	b	-	a	-	a	a	a	b
a	a	b	a	a	a	a	a	a	b
a	b	b	a	a	a	a	a	a	b
a	b	b	a	a	-	a	a	a	b
a	b	b	a	a	a	a	a	a	b
a	-	-	a	a	a	a	a	a	b
a	-	b	a	a	a	a	a	a	b
a	b	b	a	a	a	a	a	a	b
a	b	b	a	a	a	a	a	a	b
a	b	b	a	a	a	a	a	a	b
a	b	b	a	a	a	a	a	a	b
a	b	b	a	a	a	a	a	a	b
a	b	b	a	a	a	a	a	a	b
a	b	b	a	a	a	a	a	b	b
a	b	b	a	a	a	a	a	b	b
a	b	b	a	a	a	a	a	b	b
a	b	b	a	a	a	a	a	b	b
a	b	b	a	a	b	a	-	b	b
-	b	b	a	a	b	a	a	b	a
a	-	b	a	a	b	a	a	-	a
a	b	b	a	a	b	a	a	b	a
a	b	b	a	a	b	a	a	b	a

a	-	b	a	a	b	a	a	b	a
a	b	a	a	a	b	a	a	b	a
a	b	a	a	a	b	a	a	b	a
a	b	a	a	a	b	a	a	b	a
a	b	a	a	a	b	a	a	b	a
a	b	a	a	a	b	a	a	b	a
a	b	a	a	-	b	a	a	b	a
b	b	a	a	a	b	a	a	b	a
a	b	a	a	a	b	a	a	b	a
a	b	a	a	a	b	a	a	b	a
a	-	a	a	b	b	a	a	b	a
a	b	a	a	b	b	a	a	b	a
a	b	a	a	b	b	a	a	b	a
a	b	a	a	b	b	a	a	-	a
a	b	a	a	b	-	a	a	b	a
a	b	a	a	b	b	a	a	b	-
a	b	a	a	b	b	a	a	b	a
a	b	a	a	b	b	a	a	b	a
b	b	a	a	b	b	a	a	b	a
-	b	a	a	b	b	a	-	b	a
a	b	a	a	b	b	a	a	b	a
a	a	a	a	b	b	a	a	b	a
-	a	a	a	a	b	b	b	-	b
b	a	a	a	a	b	b	b	b	b
b	a	a	a	a	b	b	-	b	b
b	a	a	a	a	b	b	b	b	b
b	a	a	a	a	b	b	b	b	b
b	a	a	a	a	b	b	b	b	b
b	a	a	a	a	b	b	b	b	b
b	a	a	a	a	b	b	b	b	b
b	-	a	a	a	b	b	b	b	b
b	a	a	a	a	b	b	-	b	b
b	a	a	a	a	a	b	-	b	b
a	a	a	a	a	a	b	b	b	b
b	a	a	a	-	b	b	b	b	b
b	a	a	a	a	b	b	b	b	b
b	a	a	a	a	b	b	b	b	b
b	a	a	-	a	b	b	b	b	b
b	-	a	a	a	b	b	b	b	b
b	a	a	a	a	b	b	b	b	b
b	a	a	a	a	b	b	b	b	b
b	a	a	a	a	b	b	b	b	b
b	a	a	a	a	b	b	b	b	b
b	a	a	a	a	a	b	b	b	b
b	a	a	a	a	b	b	b	b	b
b	a	a	b	a	b	b	b	b	b
a	a	a	b	a	b	b	b	b	b

a	a	b	b	a	b	b	b	b	b
a	a	b	b	a	b	b	b	b	b
a	-	b	b	a	b	b	b	b	b
a	a	b	b	a	a	b	a	b	b
a	a	b	b	a	a	b	a	b	-
a	a	b	b	a	a	b	a	b	b
a	a	b	b	a	a	b	a	b	b
a	a	b	b	a	a	a	a	-	b
a	a	b	b	a	a	a	a	b	b
a	a	b	b	a	-	a	a	b	b
a	b	b	b	a	a	a	a	b	b
a	a	b	b	a	a	a	a	b	b
b	a	b	b	a	a	b	a	b	-
b	a	b	b	a	a	b	a	b	-
b	a	b	b	a	a	b	a	b	b
b	a	b	b	a	a	b	a	b	b
b	a	b	b	a	a	a	a	b	b
b	a	b	b	b	b	b	a	b	b
b	a	b	b	a	a	a	a	b	b
b	a	b	b	a	a	a	a	-	b
b	a	b	b	a	a	b	b	b	b
b	a	b	b	a	a	a	a	b	b
b	-	b	b	a	a	a	a	b	b
b	a	b	b	a	a	a	a	b	b
b	-	b	b	a	-	a	a	b	b
b	a	b	b	a	a	a	-	b	b
b	a	b	b	a	a	a	a	b	b
b	a	b	b	a	a	a	b	b	b
b	a	b	b	a	a	a	b	b	b
b	a	b	b	a	a	a	b	b	b
b	a	b	b	a	b	a	b	b	b
b	a	b	b	a	a	a	b	b	b
b	a	b	b	a	a	a	b	b	b
b	a	b	b	a	a	a	b	b	b
b	-	b	b	a	b	a	b	b	b
b	a	b	b	a	a	a	b	b	b
b	a	b	b	a	a	a	b	b	b
b	a	b	b	b	-	a	b	b	b
b	a	b	b	b	a	-	b	b	b
b	-	b	b	b	a	a	b	b	b
b	-	b	b	b	a	a	b	b	b
b	b	b	b	b	a	a	b	b	b
b	b	b	b	b	a	a	b	b	b
b	b	b	b	b	a	a	b	b	b
b	b	b	b	b	a	a	b	b	b
b	b	b	b	b	a	a	b	b	b
b	b	b	b	b	a	a	b	b	b

b	b	b	b	b	a	a	b	b	b
b	b	b	b	b	-	a	b	b	b
b	b	b	b	b	a	a	b	b	b
b	b	b	b	b	a	a	b	b	-
b	b	b	b	b	a	a	b	b	b
b	b	b	b	b	-	-	b	b	b
b	b	b	b	b	a	a	b	b	b
b	b	b	b	b	a	a	b	b	b
b	b	b	b	b	a	a	b	b	b
b	b	b	b	b	a	-	b	b	b
b	b	b	b	b	a	a	b	b	b
b	b	b	b	b	-	a	b	b	b
b	b	b	b	b	a	a	b	b	b
b	b	b	b	b	b	b	b	b	b
b	b	b	b	b	a	b	b	b	b
b	-	b	b	b	a	b	b	b	b
b	b	b	b	b	a	b	b	b	b
b	b	b	b	b	-	b	b	b	b
b	b	b	b	b	a	b	b	b	b
b	b	b	b	b	a	b	b	b	b
b	b	b	b	b	a	b	b	a	b
b	b	b	b	b	a	b	b	a	b
b	b	b	b	b	a	b	b	a	b
b	b	b	b	b	a	b	b	a	b
b	b	b	b	b	a	b	b	a	b
b	b	b	b	b	b	b	b	a	b
b	b	b	b	b	a	b	b	a	b
b	-	b	b	b	a	b	b	a	b
-	b	b	b	b	-	b	b	a	-
b	b	b	b	b	b	b	b	-	b
b	-	b	b	b	a	b	b	a	b
b	b	b	b	b	b	b	b	a	b
b	b	b	b	b	b	b	b	a	b
b	b	b	b	b	b	b	b	a	b
b	b	b	b	b	b	b	b	a	b
b	b	b	b	b	b	b	b	a	b
b	b	b	b	b	b	b	b	a	b
b	b	b	b	b	b	b	b	a	-
b	b	b	b	-	b	b	b	a	b
b	b	b	b	b	b	b	b	a	b
b	b	b	b	b	b	b	b	a	b
b	b	b	b	b	b	b	b	a	b
-	-	b	b	-	b	b	b	a	b
b	b	b	b	b	b	b	b	a	b
b	b	b	b	b	b	b	b	a	b
b	b	b	b	b	a	-	b	a	b
b	b	b	b	b	b	b	b	a	b

b	a	b	b	b	a	-	b	a	b
b	b	b	b	b	b	b	b	a	b
b	b	b	b	b	b	b	b	a	b
b	b	a	b	b	a	a	a	a	b
b	b	a	b	b	a	b	a	a	b
b	b	a	b	b	a	b	a	a	b
b	b	a	a	b	a	b	a	a	b
b	b	a	a	b	a	b	a	a	b
b	b	a	a	b	a	b	a	a	b
b	b	a	a	b	a	b	a	a	b
b	b	a	a	b	a	b	a	a	b
b	b	a	a	b	a	b	b	a	b
b	b	a	a	b	a	b	b	a	b
b	b	a	a	b	a	b	b	a	b
b	b	a	a	b	a	b	b	a	b
b	b	a	a	b	a	b	b	a	b
b	b	a	a	b	a	b	b	a	b
b	b	a	a	b	a	b	b	a	b
b	b	a	a	b	b	b	b	a	b
b	b	a	a	b	a	b	b	a	-
b	b	a	a	b	a	b	b	a	b
-	b	a	a	b	a	b	b	a	b
b	b	a	a	b	a	b	b	a	b
b	b	a	a	b	a	b	b	a	b
-	b	a	a	b	a	b	a	a	b
b	b	a	a	b	a	b	a	a	b
b	b	a	a	b	a	b	a	a	b
b	a	b	a	b	a	b	a	a	b
-	a	b	a	b	a	b	a	a	b
b	a	b	a	b	a	b	a	a	b
b	a	b	b	b	a	b	a	a	b
b	a	b	b	b	a	b	a	a	b
b	a	b	b	b	a	b	a	a	b
b	a	b	b	b	a	b	a	a	b
b	a	b	b	b	a	b	a	a	b
b	a	b	b	b	a	b	a	a	b
b	a	b	b	b	a	-	a	a	b
b	b	b	b	a	a	b	a	a	b
b	b	b	b	a	a	b	a	-	b
b	b	b	b	a	a	b	a	a	b
b	b	b	b	a	a	b	a	a	b
b	b	b	b	a	a	b	a	a	b
b	b	b	b	a	a	b	a	a	b
b	b	b	b	a	a	b	a	a	b
b	a	b	b	a	a	b	a	a	-
b	a	b	b	a	a	b	a	a	b
b	a	b	b	a	a	b	a	a	b

b	a	b	b	a	a	b	a	a	b
b	a	b	b	a	a	b	a	a	b
b	a	b	b	a	-	b	a	a	b
b	a	b	b	a	a	b	a	a	b
b	a	b	-	b	b	a	b	a	a
b	a	b	b	b	b	a	b	a	a
b	-	b	b	b	b	a	b	a	a
b	a	b	b	b	b	a	a	a	a
b	b	b	b	b	b	a	a	a	a
b	b	b	b	b	b	a	a	a	a
b	a	b	b	b	b	a	a	b	a
b	b	b	b	b	b	a	a	b	a
b	b	b	b	b	b	a	a	b	-
b	b	b	b	b	b	b	a	b	a
b	b	b	b	b	b	b	a	b	a
b	b	b	b	b	b	b	a	b	a
b	b	b	b	b	b	b	a	b	a
b	b	b	b	a	b	b	a	b	a
b	b	b	b	a	b	b	a	b	a
b	b	b	-	a	b	b	a	b	a
b	b	b	b	a	b	b	a	b	a
b	b	b	b	a	b	b	a	b	-
b	b	b	b	b	b	b	a	b	a
b	b	b	b	a	b	b	a	b	a
b	b	b	b	a	b	b	a	b	a
b	b	b	b	a	b	b	a	b	a
b	b	b	b	a	b	b	a	b	a
b	b	a	b	a	b	b	a	b	a
b	b	a	b	a	b	b	a	b	a
b	b	a	a	a	-	a	a	b	b
b	b	a	a	a	b	a	a	b	b
b	b	a	a	a	b	a	a	b	b
b	a	a	a	a	b	a	a	b	b
b	a	a	a	a	b	a	a	b	b
b	a	a	a	a	b	a	a	b	b
a	a	a	a	a	-	a	a	b	b
b	a	a	a	a	-	a	a	b	b
b	a	a	a	a	b	a	a	b	b
b	a	a	a	a	b	a	a	b	b
b	a	a	a	a	b	a	a	b	b
b	a	a	a	a	b	a	a	b	b
b	b	a	a	b	b	a	a	b	b
b	b	a	a	b	b	a	a	b	b
b	b	a	a	b	b	a	a	b	b
b	b	a	a	b	b	a	a	b	b
a	a	a	a	a	b	b	b	a	a
a	a	a	a	a	b	b	b	a	a

-	a	a	a	a	b	b	b	a	a
a	a	a	a	a	b	a	b	a	a
a	a	a	a	a	b	a	-	a	a
a	a	a	a	a	b	a	b	a	a
a	a	a	a	a	b	a	b	a	a
a	a	a	a	a	b	a	b	a	a
a	a	a	a	a	b	a	b	a	a
a	a	a	a	a	b	a	-	a	a
a	a	a	a	a	b	a	b	a	a
a	a	a	a	a	b	a	b	a	-
a	a	a	a	a	b	a	b	a	a
a	a	a	a	a	b	a	b	a	a
a	a	a	a	a	b	a	b	a	a
a	a	a	a	a	b	a	b	a	a
a	a	a	a	a	-	a	b	a	a
b	-	a	a	a	b	a	b	a	a
a	a	a	a	a	b	a	b	a	a
a	a	a	a	a	b	a	b	a	a
a	a	a	a	a	b	a	b	a	a
a	a	a	a	a	a	a	-	b	a
a	a	a	a	a	a	a	b	b	a
a	a	a	a	a	a	a	b	b	a
a	a	a	a	a	a	a	-	b	a
a	a	a	a	a	a	a	b	b	a
a	b	a	a	a	a	a	b	b	a
a	a	a	a	a	a	a	-	b	a
a	a	a	a	a	a	a	a	b	a
a	a	a	a	a	a	a	a	b	a
a	a	a	a	a	a	a	a	b	a
a	a	a	a	a	a	a	a	b	a
a	a	a	a	a	a	a	a	b	a
a	a	a	a	a	a	a	a	b	a
-	-	a	a	a	b	a	a	b	a
a	b	a	a	a	a	a	-	a	a
a	b	a	a	a	a	a	-	a	a
a	b	a	a	a	a	a	b	a	a
a	b	a	a	a	a	a	b	a	a
a	b	a	a	a	b	a	b	a	a
a	b	a	a	a	a	a	b	a	a
a	b	a	a	a	a	a	b	a	a
a	b	a	a	a	a	-	b	a	a
a	b	a	a	-	b	a	b	a	a
a	-	a	a	a	a	a	-	a	a
a	b	a	a	a	a	a	b	a	a
a	b	a	a	a	a	a	b	a	a
a	b	a	a	a	a	a	b	a	a
a	b	a	a	a	a	a	b	a	a

a	b	a	a	a	a	a	b	a	a
a	b	a	a	a	a	a	b	a	a
a	b	a	a	a	a	a	b	a	a
a	-	a	a	a	a	a	b	a	a
a	b	a	a	a	a	a	-	a	a
a	b	a	a	a	a	a	b	a	a
a	b	b	a	a	b	a	b	a	a
a	b	b	a	a	a	a	b	a	a
a	b	b	a	a	a	a	b	a	a
a	b	a	a	a	-	a	b	a	a
a	b	a	a	a	a	a	b	a	a
a	b	a	a	a	a	a	b	a	a
a	b	a	a	a	a	a	b	a	a
a	b	a	a	a	a	a	b	a	a
a	b	a	a	a	a	a	b	a	a
a	b	a	a	a	a	a	b	a	a
a	b	a	a	a	b	a	b	-	a
a	b	a	a	a	a	a	b	a	a
a	b	a	a	a	a	a	b	a	a
a	b	a	a	a	a	a	b	a	a
a	b	a	a	a	b	a	b	a	a
b	b	a	a	a	b	a	b	-	a
a	b	a	a	a	b	a	b	a	a
b	b	a	a	a	b	a	b	a	a
b	b	a	a	a	b	a	b	a	a
b	b	a	a	a	b	a	b	a	a
b	b	a	a	a	b	a	b	a	a
b	b	a	a	a	b	a	b	a	a
b	b	a	a	a	b	a	b	a	a
b	b	a	a	a	b	a	b	a	a
b	b	a	a	a	b	a	b	a	a
b	b	a	a	a	b	a	-	a	a
b	b	a	a	a	b	a	b	a	a
b	b	a	a	a	b	a	b	a	a
b	b	a	a	a	b	a	b	a	a
b	b	a	a	a	b	a	b	a	a
b	b	a	a	a	b	a	b	a	a
b	b	b	a	a	b	a	b	a	a
b	b	a	a	a	b	a	b	a	a
b	b	a	a	b	b	a	b	a	b
b	b	a	a	b	-	a	b	a	b
b	b	a	a	b	b	a	b	a	b
b	b	a	a	b	b	a	b	a	b
a	b	a	a	b	a	a	b	a	b
b	b	a	a	b	b	a	b	a	b
b	b	a	a	b	b	a	b	-	b
b	b	a	a	b	b	a	a	a	b

b	b	a	a	b	b	-	a	a	b
b	b	a	a	b	-	a	a	a	b
b	b	a	a	b	b	a	a	a	-
b	b	a	a	b	b	a	a	a	b
b	b	a	-	b	b	a	-	a	b
b	b	a	b	b	b	a	-	a	b
b	b	a	a	b	b	a	a	a	b
b	b	a	a	a	a	a	a	a	b
a	a	b	a	b	b	a	a	-	b
a	a	b	a	b	b	a	a	a	b
a	a	b	a	b	b	a	a	a	b
a	a	b	a	b	b	a	a	a	b
a	a	b	a	b	-	a	a	a	b
a	a	b	a	b	b	a	b	a	b
a	a	b	a	b	a	a	b	a	b
a	a	b	a	b	b	a	b	a	b
-	a	b	a	b	b	a	b	-	b
a	a	b	a	b	b	a	b	a	b
a	-	b	a	b	b	a	b	a	b
a	a	b	a	b	b	a	b	b	b
a	a	b	a	b	b	a	b	b	b
a	a	b	a	b	b	a	b	b	b
a	a	b	a	b	b	a	-	b	-
a	b	b	a	b	b	a	b	b	b
a	b	b	a	b	b	a	b	b	b
a	b	b	a	b	b	a	b	b	b
a	b	b	a	b	a	a	b	b	b
-	b	b	b	b	b	a	b	b	a
a	b	b	a	b	b	a	b	b	a
a	a	b	a	b	b	a	b	b	a
a	a	a	a	b	b	b	b	b	a
a	a	a	b	b	b	b	b	b	a
a	a	a	a	b	b	b	b	b	a
a	a	a	a	b	b	b	b	b	a
a	a	a	a	b	-	b	b	-	a
a	b	b	a	b	b	b	b	b	a
a	a	-	a	b	b	b	b	-	a
a	a	b	a	b	b	b	b	b	a
a	a	b	a	b	b	b	b	b	a
a	a	b	a	b	b	b	b	b	a
a	a	b	a	b	b	b	b	b	a
a	a	b	a	b	-	b	b	b	a
a	a	b	a	b	a	b	-	b	a
a	-	b	a	b	-	b	b	b	a
a	b	b	a	b	b	b	b	b	a
a	b	b	a	b	b	b	b	b	a
a	b	b	a	b	b	b	b	b	a

a	b	b	a	b	b	b	b	b	a
a	b	b	a	a	-	b	b	b	a
a	b	b	a	a	b	b	-	-	a
a	b	b	a	a	b	b	b	-	a
a	b	b	a	a	b	b	b	b	a
a	-	b	a	a	b	b	b	b	b
a	b	b	a	a	a	b	b	b	b
a	b	-	a	a	a	b	a	b	b
a	b	b	a	a	a	b	b	a	b
a	b	b	a	a	a	b	b	a	b
a	b	b	a	a	a	b	b	a	b
-	b	b	a	b	a	b	b	-	b
-	-	b	b	a	b	-	b	a	-
a	a	b	b	a	b	a	a	a	a
a	a	b	b	a	b	a	a	a	a
a	a	b	b	a	b	a	a	a	a
a	a	b	b	a	b	a	b	a	a
a	a	b	b	a	b	a	b	a	a
a	a	b	b	a	b	a	b	a	b
a	a	b	b	a	b	a	b	a	b
a	a	b	b	a	b	a	-	a	-
-	a	b	b	-	b	a	b	a	b
a	a	b	b	a	b	a	b	a	b
a	a	b	b	a	b	a	b	a	b
a	a	b	b	a	b	a	b	a	b
a	a	b	b	a	b	a	b	a	b
a	a	b	b	a	b	a	b	a	b
a	b	b	b	b	b	a	b	a	b
a	b	b	b	b	-	a	b	a	b
a	b	b	b	b	b	a	b	a	b
a	b	b	b	b	b	a	b	a	b
a	b	b	b	b	b	a	b	a	b
a	b	b	b	b	b	-	b	a	b
a	b	b	b	b	b	a	b	a	b
a	b	b	b	b	b	a	b	a	b
a	b	b	b	b	b	a	b	a	b
a	b	b	b	b	b	a	b	a	b
a	a	b	b	b	a	a	b	a	b
a	a	b	b	b	b	a	b	a	b
a	a	b	a	b	b	a	b	a	b
b	b	b	a	b	b	a	b	a	b
b	b	b	a	b	b	a	b	a	b
b	b	b	a	b	b	a	b	a	b
b	b	b	a	b	b	a	b	a	b
b	b	b	a	b	b	a	b	a	b
b	a	b	a	b	a	a	b	b	b
b	a	b	a	b	a	a	b	b	b

a	a	b	a	b	a	a	b	b	b
b	a	b	a	b	a	a	b	b	a
b	a	b	a	b	a	a	b	b	a
b	a	b	a	b	a	a	b	b	a
b	a	b	a	-	a	a	b	b	a
b	a	b	a	b	b	a	a	b	a
a	a	b	a	b	a	a	a	b	a
a	a	b	a	a	a	a	a	b	a
b	b	a	b	b	a	b	b	a	b
b	b	a	b	b	-	b	b	-	b
b	b	a	b	b	a	b	b	a	b
b	b	a	a	-	a	-	b	a	b
b	b	a	a	b	a	b	b	a	b
-	b	a	a	b	a	-	b	a	b
b	b	a	a	b	a	b	b	a	b
b	b	a	a	b	a	b	b	a	b
b	b	a	a	b	a	b	b	a	b
b	b	a	a	b	a	b	b	a	b
-	b	a	a	b	a	b	b	a	b
b	b	a	a	-	b	b	b	a	b
b	b	a	a	-	b	b	b	a	b
b	b	a	a	b	b	b	b	b	b
b	b	a	a	b	-	b	b	b	b
b	b	a	a	b	b	b	b	b	b
b	b	a	a	b	b	b	b	b	b
b	b	b	a	b	b	b	b	b	b
b	b	b	a	b	b	b	-	b	b
b	b	b	a	b	a	b	b	b	b
b	b	b	a	b	b	a	b	b	b
b	b	b	a	b	b	a	b	b	b
b	b	b	a	b	b	a	b	b	b
b	b	b	a	-	b	a	b	b	b
b	b	b	a	b	b	a	b	b	b
b	b	b	b	-	b	a	a	-	b
b	b	b	a	b	-	a	a	b	b
b	a	b	a	b	a	a	a	b	b
b	b	b	a	b	b	a	a	b	b
b	b	b	a	b	b	a	a	b	b
b	-	b	a	b	b	a	a	b	b
b	b	b	a	b	b	a	a	b	b
b	-	b	a	b	-	a	a	b	-
b	b	b	a	b	b	a	a	b	b
b	b	b	a	b	b	a	a	b	b
b	b	b	a	b	b	a	a	b	b
a	-	b	a	-	a	a	a	b	b
b	b	b	a	b	a	a	a	b	b
b	b	b	a	-	a	a	a	b	b

b	b	b	a	b	b	a	a	b	b
b	b	b	a	b	b	a	a	b	b
b	b	a	a	b	b	a	a	b	b
b	b	b	a	b	b	a	a	b	b
b	b	b	a	b	b	a	a	b	a
b	b	b	a	b	b	a	a	b	b
b	b	b	a	b	b	a	a	b	a
a	b	b	a	b	-	a	a	b	a
a	b	b	a	b	b	a	a	b	a
a	b	-	a	b	a	a	a	b	a
b	b	b	a	b	b	-	a	b	a
a	b	b	a	b	b	a	a	b	a
a	b	b	a	b	b	a	a	b	a
a	b	b	a	b	b	a	a	b	a
a	b	b	a	b	b	a	a	b	a
a	b	b	a	a	b	a	a	b	a
a	b	b	a	a	b	a	a	b	a
a	b	b	a	a	b	a	a	a	a
a	a	a	a	a	a	a	a	b	a
a	a	a	a	a	a	a	a	b	a
b	b	a	a	b	a	a	b	a	b
b	b	a	a	b	a	a	b	a	b
b	b	a	a	b	-	a	a	a	b
b	b	a	a	b	a	-	a	a	b
b	b	a	a	b	-	a	a	a	b
b	b	a	a	b	a	-	a	a	a
b	b	a	a	b	a	a	a	a	a
b	b	a	a	b	a	a	a	a	a
b	b	a	a	b	a	a	a	a	a
b	b	a	a	b	a	a	a	a	a
b	b	a	a	b	a	a	a	a	a
b	b	a	a	a	a	a	a	a	a
b	b	a	a	b	a	a	a	a	-
b	b	a	a	b	a	a	a	a	a
b	b	a	a	b	-	a	-	-	a
b	b	a	a	b	a	a	a	a	a
b	b	a	a	b	b	a	-	a	a
b	b	a	a	b	a	a	b	b	a
b	b	a	a	b	a	a	b	b	a
b	b	a	a	b	a	a	b	b	-
b	b	b	a	b	a	a	b	b	a
b	-	b	a	b	a	a	b	b	a
b	b	b	a	b	a	a	b	b	a
b	b	b	a	b	a	a	b	a	a
b	b	b	a	b	a	a	b	a	a
b	b	b	a	b	a	a	b	a	a
b	b	b	a	b	a	a	b	a	a

b	b	b	a	b	-	a	b	a	a
b	b	b	a	b	a	a	b	a	a
-	b	b	a	a	a	a	b	a	a
b	b	b	a	b	a	a	b	a	a
b	b	b	a	b	a	a	b	a	a
b	a	b	a	b	a	a	b	a	a
b	a	b	a	b	b	a	b	a	a
b	-	b	a	b	-	a	b	a	a
b	a	b	a	b	a	a	b	a	a
-	a	b	a	b	a	a	b	a	a
b	-	b	a	a	a	a	b	a	a
b	a	b	a	a	a	a	-	a	a
b	a	b	a	a	a	a	b	a	a
b	a	b	a	a	a	a	b	a	a
b	a	b	a	a	-	a	b	a	a
b	a	b	a	a	a	a	b	b	a
b	a	b	a	a	a	a	b	b	a
b	a	b	a	a	a	a	b	b	a
b	a	b	a	a	-	a	b	b	a
b	a	b	a	a	a	a	b	b	a
b	a	b	a	a	a	a	b	b	a
b	a	b	a	a	a	a	b	b	a
b	a	b	a	a	a	a	b	b	a
b	a	b	a	a	a	a	-	b	a
b	a	b	a	a	a	a	b	b	a
b	a	b	a	a	a	a	a	b	a
b	a	b	a	a	a	a	a	b	a
b	a	b	a	a	a	a	a	b	a
b	a	b	a	a	a	a	a	-	a
b	a	b	a	a	a	a	a	b	a
b	a	b	a	a	a	a	a	b	a
b	a	b	a	a	a	a	a	b	-
b	a	b	a	a	a	a	a	b	-
b	a	b	a	a	b	a	a	b	a
b	a	b	a	a	b	a	a	b	a
a	a	a	b	b	a	b	a	a	a
a	b	a	b	b	b	b	b	a	a
a	b	a	b	b	a	b	b	a	a
a	b	a	b	b	a	b	b	a	a
a	b	a	a	b	a	b	b	a	-
a	b	a	a	b	b	b	b	a	a
a	a	a	a	b	a	b	a	a	a
a	a	a	a	b	-	b	-	a	a
a	a	a	a	b	a	b	a	a	-
a	a	a	a	b	a	b	a	a	a
a	a	a	a	b	a	-	a	a	a
a	a	a	a	b	a	b	a	a	a

a	a	a	a	b	a	b	a	a	a
a	a	a	a	b	a	b	a	a	a
a	a	a	a	b	a	b	a	a	a
a	a	a	a	b	a	b	a	a	a
a	a	a	a	b	a	b	a	a	a
a	a	a	a	b	a	b	a	a	a
a	a	a	a	b	a	b	a	a	a
a	a	a	a	b	b	b	a	a	a
a	a	a	a	b	a	b	a	a	a
a	a	a	a	b	a	b	b	a	a
a	-	a	a	b	a	b	b	a	a
a	a	a	a	b	-	b	b	a	a
-	-	a	a	b	a	b	b	a	a
a	a	a	a	b	a	b	b	a	a
a	a	a	a	b	a	b	b	a	a
a	a	a	a	b	a	b	b	a	a
a	a	a	b	b	-	b	b	a	a
a	a	a	b	b	a	b	b	a	a
a	a	a	b	b	a	b	b	a	a
a	a	a	b	b	a	b	b	a	a
a	a	a	b	b	a	b	b	a	a
a	a	a	b	b	a	b	b	a	a
a	a	a	b	b	a	b	-	a	a
a	a	a	b	b	a	b	-	a	a
a	a	a	b	b	a	b	b	b	a
a	a	a	b	b	a	b	b	b	a
a	a	a	b	b	a	b	b	b	a
a	a	a	b	b	a	-	b	b	a
a	a	a	b	b	a	b	b	b	a
-	a	a	b	b	a	b	b	b	a
a	a	a	b	b	a	a	b	b	a
a	a	a	b	b	a	a	b	b	a
a	a	a	b	b	a	a	b	b	a
a	a	a	b	b	a	a	b	b	a
a	a	a	b	a	a	a	b	b	a
a	a	a	b	a	a	a	b	b	a
a	a	a	b	a	a	a	b	b	a
a	a	a	b	a	a	a	b	b	a
a	a	a	b	a	a	a	b	b	-
b	-	a	b	a	a	a	b	b	a
a	a	a	b	a	a	a	b	-	a
a	a	a	b	a	a	a	b	b	-
a	a	a	b	a	a	a	b	b	-
a	a	a	b	a	a	a	b	b	a
a	a	a	b	a	a	a	b	b	a
a	a	a	b	a	a	a	-	b	a
b	b	a	b	b	a	b	a	a	a

b	b	a	b	b	a	b	a	a	a
b	a	a	b	b	a	b	a	a	a
b	-	a	b	b	a	b	a	a	a
b	b	a	b	b	a	b	a	a	a
b	b	a	b	b	a	b	a	a	a
b	b	a	b	b	a	b	a	a	a
b	b	a	a	b	-	b	a	a	a
b	b	a	a	b	b	b	a	a	a
b	b	a	a	b	b	b	a	a	a
b	b	a	a	b	b	b	a	a	a
b	b	a	a	b	b	b	a	a	a
b	b	a	a	b	b	b	a	a	a
b	b	a	a	b	b	b	a	a	a
b	b	a	a	b	b	b	a	a	a
b	b	a	a	b	b	b	a	a	a
b	b	a	a	b	b	b	a	a	a
b	b	a	a	b	b	b	a	a	a
b	b	a	a	b	b	b	a	a	a
b	b	a	a	b	b	b	a	a	a
b	b	a	a	b	b	b	a	a	a
b	b	a	a	b	b	b	a	a	a
b	b	a	a	b	b	b	a	a	a
b	b	a	a	b	a	b	a	a	a
b	b	a	a	b	b	b	a	a	a
b	b	a	a	b	b	b	a	a	a
b	b	a	a	b	b	b	a	a	a
b	b	a	a	b	b	b	a	a	a
b	b	a	a	b	b	b	a	a	a
b	b	a	a	b	b	b	a	a	a
b	b	a	a	b	b	b	a	b	a
b	b	a	a	b	a	b	a	b	a
a	b	a	a	a	b	b	a	b	a
a	b	a	a	a	b	b	a	b	a
a	b	a	a	a	b	b	a	b	a
a	b	a	a	a	b	b	a	b	a
a	b	a	a	a	b	b	a	b	a
a	b	a	a	a	b	b	a	b	a
a	b	a	a	a	b	b	a	b	a
a	b	b	a	a	b	b	a	b	a
a	b	a	a	a	b	b	a	b	a
b	b	b	b	a	b	b	a	a	a
a	a	a	a	a	a	a	-	b	b
a	a	a	a	a	a	a	b	b	b
a	a	a	a	a	a	a	b	b	b
a	a	a	a	a	a	a	b	b	b
a	a	a	a	a	a	a	b	b	b
a	a	a	a	a	a	a	b	b	b

a	a	a	a	a	a	a	b	b	b
a	a	a	a	a	b	a	b	b	b
a	a	a	a	a	a	a	b	b	b
a	a	a	a	a	a	a	b	b	b
a	a	a	a	a	a	a	b	b	b

YW089	YW090	YW091	YW092	YW093	YW094	YW095	YW097	YW098	YW099
a	a	b	a	a	a	a	b	b	b
a	b	b	a	a	b	a	b	b	a
a	b	b	a	a	b	a	b	b	a
a	b	b	a	a	b	a	b	b	a
a	b	b	a	a	b	a	b	b	a
a	b	b	a	a	b	a	b	b	a
a	b	b	a	a	b	a	b	b	a
a	b	b	a	a	b	a	b	-	a
b	b	b	a	a	b	a	b	b	b
b	b	b	a	a	b	a	b	b	b
b	b	b	a	a	b	a	b	-	b
b	b	b	a	a	-	a	b	b	b
-	b	b	a	a	b	a	b	b	b
b	b	b	a	a	b	a	b	b	b
b	b	b	a	a	b	a	b	b	b
b	b	b	a	a	b	a	b	b	b
b	b	b	a	-	b	a	b	b	b
b	b	b	a	a	b	a	b	a	b
b	b	b	a	a	b	a	b	a	b
b	b	b	a	-	b	a	b	a	b
b	b	b	a	a	-	a	b	a	b
b	b	b	a	a	a	b	b	a	b
b	b	b	a	a	b	a	b	a	b
b	b	b	a	a	b	a	b	a	b
b	b	b	a	a	b	a	b	a	b
-	b	b	a	a	b	a	b	a	b
b	b	b	a	a	b	a	b	-	b
b	b	b	a	a	b	a	b	a	b
b	b	b	a	a	-	a	b	a	b
b	b	b	a	a	b	a	b	a	b
b	b	b	a	a	b	a	b	a	b
b	b	b	a	a	a	b	b	a	b
b	b	b	a	a	b	b	b	a	b
b	b	b	a	a	b	a	b	a	b
b	b	b	a	a	b	a	b	a	b
b	b	b	a	a	b	a	b	a	b
b	b	b	a	a	b	a	b	a	-
b	b	b	a	a	b	a	b	a	b
b	b	b	a	a	a	b	b	a	b
b	b	b	a	a	b	a	b	a	b
b	b	b	a	a	b	a	b	a	b
b	b	b	a	a	b	a	b	a	-
b	b	b	a	a	b	a	b	b	b
b	b	b	a	a	b	a	b	b	b
b	b	b	a	a	b	a	b	b	b

b	b	b	a	a	b	a	b	b	b
b	b	b	a	a	b	a	b	-	b
b	b	b	a	a	b	a	b	b	b
b	b	b	a	a	b	a	b	b	b
b	b	b	a	a	b	a	b	b	b
b	b	b	a	a	b	a	b	b	b
b	a	b	a	a	b	a	b	b	b
b	a	b	a	a	b	a	b	b	b
b	a	b	a	a	b	a	b	b	-
b	a	b	a	a	b	a	b	b	b
b	b	b	a	a	b	a	b	b	b
b	b	b	a	a	b	a	b	b	b
b	b	b	a	a	b	a	b	b	b
b	b	b	a	-	b	a	b	b	b
b	b	b	a	a	b	a	b	b	b
b	b	b	a	a	b	a	b	b	b
b	b	b	a	a	-	a	b	b	b
b	b	b	a	a	b	a	b	b	b
b	b	b	a	a	a	b	b	b	a
b	b	b	a	a	b	a	b	b	a
b	b	b	a	a	b	a	b	b	a
b	b	b	a	a	b	a	b	b	a
b	b	b	a	a	b	a	-	b	a
b	b	b	a	a	b	a	b	b	a
b	b	b	a	-	b	a	b	b	a
b	b	b	a	a	b	a	b	b	a
b	b	b	a	a	b	a	b	b	a
b	b	b	a	a	b	a	b	b	a
b	b	b	a	a	b	a	b	b	a
b	b	b	a	a	b	a	b	b	a
b	b	b	a	a	b	a	b	a	a
b	b	b	a	a	b	b	b	b	a
b	b	b	a	a	b	b	b	-	a
b	b	b	a	a	b	b	b	-	a
b	b	b	a	a	b	b	b	b	a
b	b	b	a	a	b	b	b	b	a
b	b	b	a	a	b	b	b	b	a
b	b	b	a	a	b	b	b	b	a
b	b	b	a	a	a	b	b	-	a
b	b	a	b	b	a	a	b	b	a
b	b	a	b	b	a	a	b	b	a
b	b	a	b	b	a	a	-	b	a
b	b	a	b	b	a	a	b	b	a
b	b	a	b	b	-	a	b	b	a
b	b	a	b	b	a	a	b	b	a
a	b	a	b	b	a	a	b	b	a
a	b	a	b	b	a	a	b	b	a

a	b	a	b	b	a	a	b	b	a
a	b	b	b	b	a	a	b	b	a
a	b	a	b	b	a	a	b	b	a
a	b	a	b	b	a	a	-	b	a
a	b	a	b	b	a	a	b	-	a
a	b	a	b	b	a	a	b	b	a
a	b	a	b	b	a	a	b	b	a
a	b	a	b	b	a	a	b	b	a
a	b	a	b	b	a	a	b	b	a
a	b	a	b	b	a	a	b	b	-
a	b	a	b	b	a	a	b	b	a
a	b	a	b	b	a	a	b	b	a
a	b	a	b	b	a	a	b	b	a
a	b	a	b	b	b	a	b	b	a
a	b	a	b	b	b	a	b	b	a
a	b	-	b	b	b	a	b	b	a
a	b	a	b	b	b	a	b	-	a
a	b	a	b	b	b	a	b	b	a
a	b	a	b	b	b	a	b	b	a
a	b	a	b	b	-	a	b	a	a
a	b	a	b	b	b	a	b	a	a
a	b	a	b	-	b	a	b	a	a
a	b	-	b	b	b	a	b	a	a
a	b	a	b	b	b	a	b	a	a
a	a	a	b	b	b	a	b	a	a
a	a	a	b	b	-	a	b	a	a
a	a	a	b	b	b	a	b	a	a
a	a	a	b	b	b	a	b	-	a
a	a	a	b	b	b	a	b	a	a
a	a	a	b	b	b	a	b	a	a
a	a	a	b	b	b	a	b	a	a
a	a	a	b	b	b	a	b	a	a
a	a	a	b	b	b	a	b	a	a
a	a	a	b	b	b	a	b	a	a
a	a	a	b	b	b	a	b	a	a
-	a	a	b	b	b	a	b	a	a
a	a	-	b	b	b	a	b	a	a
a	a	a	b	b	b	a	b	a	a
a	a	a	b	b	b	a	b	a	a
a	a	a	b	b	a	b	b	a	a
a	a	a	b	b	b	a	b	a	a
a	a	a	b	b	b	a	b	a	a
a	a	a	b	b	b	a	b	-	a
a	a	a	b	b	b	a	b	a	a
a	b	a	b	b	b	a	b	a	a
a	b	a	b	b	-	a	b	a	a
a	b	a	b	b	b	a	b	a	a

a	b	a	b	b	b	a	b	a	a
-	b	a	b	b	b	a	b	a	b
b	b	a	b	b	b	a	b	a	b
b	b	a	b	b	b	a	b	a	b
b	b	a	b	b	b	a	b	a	b
b	b	a	b	b	b	a	b	a	b
b	b	a	b	b	b	a	b	a	b
b	b	a	b	b	a	b	b	a	b
b	b	a	b	b	b	a	b	a	b
b	b	a	b	b	b	a	b	a	b
b	b	a	b	b	b	a	b	a	b
b	b	a	b	b	a	b	b	a	b
b	b	a	b	b	b	a	b	a	b
-	b	a	b	b	a	a	-	b	-
b	b	a	b	b	a	a	b	b	b
b	b	a	b	b	a	a	b	b	b
b	b	a	b	b	a	a	b	b	b
b	b	a	b	b	a	a	b	b	b
b	b	a	b	b	a	a	b	b	b
b	b	a	b	b	-	a	b	b	b
b	b	a	b	b	a	a	b	b	b
b	b	a	b	b	a	a	b	b	b
b	b	a	b	b	a	-	b	b	b
b	b	a	b	b	a	b	b	b	b
b	b	a	b	b	a	b	b	b	b
b	b	a	b	b	a	b	b	b	b
b	b	a	b	b	a	b	b	b	b
b	b	a	b	b	b	a	b	b	b
b	b	a	b	b	a	b	b	b	b
b	b	a	b	b	a	b	b	b	b
b	b	a	b	b	a	b	b	b	b
b	a	-	b	b	b	a	b	b	b
b	b	a	b	b	a	-	b	-	-
b	b	a	b	b	a	b	b	b	b
b	b	a	b	b	a	b	b	b	b
b	a	a	b	b	a	b	b	b	b
b	a	a	b	b	a	b	b	b	b
b	a	-	b	b	a	b	b	b	b
b	a	-	b	b	a	b	b	b	b
b	a	a	b	b	a	b	b	b	b
b	a	a	b	a	a	b	b	b	b
b	a	a	b	a	a	a	b	b	b
b	a	a	b	a	a	a	b	b	b
b	a	a	b	a	a	a	b	b	b
b	a	a	b	a	a	a	b	b	-
a	a	a	b	a	a	a	b	-	b
a	a	a	b	a	a	a	b	b	b

a	a	a	b	a	a	a	-	-	b
a	a	a	b	a	a	a	b	b	b
a	a	a	b	a	a	a	b	b	b
-	a	a	b	a	a	-	b	b	b
a	a	a	b	a	a	a	b	b	b
a	a	a	b	a	a	a	b	b	b
a	a	a	b	a	a	a	b	b	b
a	a	a	b	a	a	a	b	b	b
a	a	a	b	a	a	a	b	b	b
a	a	a	b	a	a	-	b	b	b
a	a	a	b	a	a	a	-	b	b
a	a	a	b	a	a	a	b	b	b
b	a	-	b	b	a	a	b	a	a
b	a	a	b	b	a	a	b	a	a
b	a	a	b	b	a	a	b	a	a
b	a	a	b	b	a	a	b	a	a
b	a	a	b	b	a	a	b	a	a
b	a	a	b	b	a	a	b	a	a
b	a	a	b	b	a	a	b	a	a
b	a	a	b	b	a	a	b	-	a
b	a	a	b	b	a	a	b	a	a
b	a	a	b	b	a	a	b	-	a
b	a	a	b	b	a	a	b	a	a
b	a	a	b	b	a	a	b	a	a
b	-	a	b	b	-	a	b	a	a
b	a	a	b	b	-	a	b	a	a
b	b	a	b	b	a	a	a	a	a
b	b	a	b	b	a	a	a	a	-
b	b	a	b	b	-	a	a	a	a
b	b	a	b	b	b	a	-	a	a
b	b	a	b	b	b	a	a	a	a
b	b	a	b	b	b	a	a	a	a
b	b	a	b	b	a	b	a	a	a
a	b	a	b	b	b	a	a	a	a
a	b	a	b	b	b	a	a	a	a
a	b	b	b	b	b	a	a	a	a
a	b	b	b	b	b	a	a	a	a
a	b	b	b	b	b	a	a	a	a
a	b	b	b	b	b	a	a	a	a
a	b	b	b	b	b	a	a	a	a
a	b	b	b	b	b	a	a	a	-
a	b	b	b	b	b	a	a	a	a
a	b	b	b	b	b	a	a	a	a
a	b	b	b	b	b	a	a	a	a
a	b	b	b	b	b	a	a	a	a
a	b	b	b	b	-	a	a	a	a
a	b	b	b	b	b	a	a	b	a

a	b	b	b	b	b	a	a	b	a
a	b	b	b	b	b	a	a	b	a
a	b	b	b	b	b	a	a	b	a
a	b	b	b	b	b	a	a	a	a
a	b	b	b	b	b	a	a	a	a
a	b	b	b	b	b	a	a	a	a
a	b	b	b	b	b	b	a	a	a
a	b	b	b	b	b	b	a	a	a
a	b	b	b	b	b	b	a	-	a
a	b	b	b	b	b	b	a	b	a
a	b	b	b	b	-	b	a	b	a
a	b	b	b	b	b	b	a	-	a
a	b	b	b	b	b	b	a	b	a
a	b	b	b	b	b	b	a	b	a
a	b	b	b	b	b	b	a	b	a
a	b	b	b	b	b	b	a	b	a
a	b	b	b	b	b	b	a	b	a
a	b	b	b	b	b	b	a	b	a
a	b	-	b	b	-	a	a	b	a
a	b	b	b	b	b	b	a	b	a
a	b	b	b	b	b	b	a	b	-
a	b	b	b	b	b	b	a	b	a
a	b	b	b	a	b	b	a	b	a
a	b	b	b	a	b	b	a	b	a
a	b	b	b	a	b	b	a	b	a
a	b	b	-	a	b	b	a	b	a
a	b	a	b	a	b	b	a	b	a
a	b	b	b	a	b	b	a	b	a
a	b	b	b	a	b	b	a	b	a
a	b	b	b	a	b	b	a	b	a
a	b	b	b	-	b	b	-	b	a
a	b	b	b	a	b	b	a	b	a
a	b	b	b	a	b	b	a	b	a
a	b	b	b	a	b	a	a	b	a
a	b	b	b	a	b	b	a	b	a
a	b	b	b	a	b	b	a	b	a
a	b	b	b	a	b	b	a	b	a
a	b	b	b	a	b	b	a	b	a
a	b	b	b	a	b	b	a	b	a
a	b	b	b	a	b	b	a	b	a
a	b	b	b	a	b	b	a	b	a
-	b	b	b	a	b	b	a	b	a
a	b	b	b	a	b	b	a	b	a
a	b	b	b	a	b	b	a	b	a
a	b	b	b	a	b	b	a	b	a
a	b	b	b	a	b	b	a	b	a
a	b	b	b	a	b	b	a	b	a

a	b	b	b	a	b	b	a	-	a
a	b	b	b	a	b	b	a	b	a
a	b	b	b	a	b	b	a	b	a
a	b	b	b	a	b	b	a	b	a
a	b	b	b	a	b	b	a	b	a
a	b	b	b	a	b	b	a	b	a
a	b	b	a	a	b	b	a	b	a
a	b	b	a	a	b	b	a	b	a
a	b	b	a	a	b	b	a	-	a
a	b	b	a	a	b	b	a	b	a
a	b	b	a	a	b	b	a	b	a
a	b	b	a	a	b	b	a	b	a
a	b	b	a	a	b	b	a	b	a
a	b	b	a	a	b	b	a	-	a
a	b	b	a	a	b	b	a	b	a
a	b	b	a	a	b	b	a	b	a
a	b	b	a	a	b	b	a	b	a
a	b	a	a	a	b	a	a	b	a
a	b	a	a	a	b	a	a	b	a
a	b	a	a	a	b	a	a	b	a
a	b	a	a	a	b	a	a	b	a
a	b	a	a	a	b	a	a	-	a
a	b	a	a	a	b	a	a	b	a
a	b	a	a	a	b	a	a	b	a
a	b	a	a	a	b	a	a	b	a
a	b	a	a	a	-	a	a	b	a
a	b	a	a	a	b	a	a	b	a
a	b	a	a	a	b	a	-	b	a
a	b	a	a	a	b	a	a	b	a
a	b	a	a	a	b	a	a	b	a
a	b	a	a	a	b	a	a	b	a
a	b	a	a	a	b	a	a	b	a
a	b	a	a	a	b	a	a	b	a
a	a	a	a	a	b	a	a	b	a
a	a	a	a	a	b	a	a	b	a
a	a	a	a	a	b	a	a	b	a
a	a	a	a	a	b	a	a	b	a
a	a	a	a	a	-	a	-	b	-
a	a	b	b	-	a	a	b	a	b
a	a	b	b	b	a	a	b	a	b
a	a	b	b	b	a	a	b	b	b
a	a	b	b	b	a	a	b	b	a
a	a	b	b	b	a	a	b	b	a
a	a	b	b	b	a	a	b	b	a
a	a	b	b	b	a	a	b	b	a
a	a	b	b	b	a	a	b	b	a
a	a	b	b	b	a	a	b	b	a

a	a	b	b	b	a	a	a	b	a
a	a	b	b	b	a	a	a	b	a
a	a	a	b	b	a	a	a	b	a
a	a	a	b	b	-	a	a	b	b
a	a	b	b	b	a	a	a	b	b
-	a	b	a	b	a	a	a	b	b
a	a	b	a	b	a	a	a	b	b
a	a	b	a	b	a	a	a	b	b
a	a	b	a	b	a	a	a	b	b
a	a	b	a	b	a	a	a	b	-
a	a	b	a	b	a	b	a	b	b
a	a	b	a	b	b	a	a	b	b
a	a	b	a	b	-	b	a	b	b
a	a	b	a	b	a	b	a	b	b
a	a	b	a	b	a	b	a	b	b
b	a	b	a	b	a	b	a	b	b
b	a	b	a	b	a	b	a	b	b
b	a	b	a	b	a	b	a	b	b
b	a	b	a	b	-	b	-	b	b
b	a	b	a	b	a	b	a	b	b
b	a	b	a	b	a	b	a	b	b
b	a	b	a	b	a	b	a	b	b
b	a	b	a	b	a	b	a	b	b
b	a	b	a	b	a	b	a	b	b
b	a	b	a	b	a	b	a	b	-
b	a	b	a	b	a	b	a	b	b
b	a	b	a	b	a	b	a	b	b
b	a	b	a	b	a	b	a	-	b
b	a	b	a	b	a	b	a	b	b
b	a	b	a	b	a	b	a	b	b
b	a	b	a	b	a	b	a	b	b
b	b	b	a	b	a	b	a	b	b
b	b	b	a	b	a	b	a	b	b
b	b	b	a	b	a	b	a	b	b
b	b	b	a	b	a	b	a	b	b
b	b	b	a	b	a	b	a	a	b
b	b	b	b	b	a	b	a	b	a
b	b	b	b	b	a	b	a	b	a
b	b	b	b	b	a	b	a	b	a
b	b	b	b	b	b	a	a	b	a
b	b	-	b	b	a	b	a	b	a
b	b	a	b	b	a	b	a	b	a
b	b	b	b	b	b	a	a	b	b
b	b	b	b	b	a	b	a	b	b
b	b	b	b	b	a	b	a	b	b
b	b	b	b	-	a	b	a	b	b
b	b	b	b	b	a	b	a	b	b

b	b	b	b	b	a	b	a	b	b
b	b	b	-	b	a	b	a	b	b
b	b	b	b	b	a	b	-	b	b
b	b	b	b	b	a	b	a	b	b
b	b	b	b	b	a	b	a	-	b
b	b	-	b	b	a	b	a	b	b
b	b	b	b	-	a	b	a	b	b
b	b	b	b	b	a	b	a	b	b
b	b	b	b	b	a	b	a	b	b
b	b	b	b	b	a	b	a	b	b
b	b	-	b	b	a	b	a	b	b
b	b	-	b	b	a	b	a	b	b
b	b	b	b	b	-	b	a	b	b
b	b	b	b	b	b	a	a	b	b
-	b	b	b	b	a	b	a	b	b
b	b	b	b	b	a	b	a	b	b
a	b	b	b	b	a	b	a	a	b
a	b	b	b	b	a	b	a	a	-
a	b	b	b	b	b	a	a	a	b
a	b	b	b	b	a	b	a	a	b
a	b	b	b	b	b	a	a	a	b
a	b	b	b	b	a	b	a	a	b
a	b	b	b	b	a	b	a	a	-
a	b	b	b	b	a	b	a	a	b
a	b	b	b	b	a	b	a	a	b
a	b	b	b	b	a	b	a	a	b
a	b	b	b	b	a	b	a	a	b
a	b	b	b	b	a	b	a	a	b
a	b	b	b	b	a	b	a	a	b
a	b	b	b	b	b	b	a	a	b
a	b	b	b	b	b	b	a	a	b
a	b	b	b	b	b	b	a	a	a
-	b	b	b	b	b	b	a	-	a
a	b	b	b	b	b	b	a	-	a
a	b	b	b	b	b	b	a	a	a
a	b	b	b	b	-	b	a	a	a
a	b	b	b	b	b	b	a	a	a
a	b	b	b	b	b	b	a	-	a
a	b	b	b	b	b	b	a	a	a
a	b	b	b	b	b	b	a	a	a
a	b	a	b	a	a	a	a	a	b
a	b	a	b	a	a	a	a	a	b
a	b	a	b	a	a	a	a	a	b
a	b	a	b	a	a	a	a	a	b
a	b	a	b	-	a	a	a	-	b
b	b	a	b	a	a	a	a	a	b
b	b	a	-	a	a	a	a	a	-

b	b	a	b	a	a	a	a	a	b
b	b	a	b	a	a	a	a	a	b
b	b	a	b	a	a	a	a	a	b
b	b	a	b	a	a	a	a	a	b
b	b	a	b	a	-	a	a	a	b
b	b	a	b	a	a	a	a	a	b
b	b	a	b	a	-	a	a	a	b
b	b	a	b	a	a	a	a	a	b
b	b	a	b	a	a	a	a	a	b
b	b	a	b	b	a	a	a	a	b
b	b	a	b	b	a	a	a	a	b
b	b	a	b	b	a	a	a	a	b
b	b	a	b	b	a	a	a	a	b
b	b	a	b	b	a	a	a	b	b
b	b	a	b	b	a	a	a	b	b
b	b	a	b	b	a	a	a	b	b
b	b	a	b	b	a	a	a	b	b
b	b	a	b	b	a	a	a	b	b
b	b	a	b	b	a	a	-	b	b
b	b	a	b	b	a	a	a	b	b
b	b	a	b	b	a	a	a	b	b
b	b	a	a	b	-	a	b	b	b
b	b	a	a	b	a	a	b	-	b
b	b	a	a	b	a	a	b	b	b
b	b	a	a	b	a	a	b	-	b
b	b	a	a	b	a	a	b	b	b
b	b	a	a	b	a	a	b	b	b
b	b	a	a	b	a	a	b	b	b
b	b	a	a	b	a	a	b	b	b
b	b	a	a	b	a	a	b	b	b
b	b	a	a	b	a	a	b	-	b
b	b	a	a	b	a	a	b	b	b
b	b	a	a	b	a	a	b	b	b
b	-	a	a	-	a	a	-	a	-
b	b	a	a	b	a	a	b	-	b
b	b	a	a	b	a	a	b	b	b
b	b	a	a	b	a	a	b	b	b
b	b	a	a	b	-	a	b	b	b
b	b	a	a	b	-	a	b	b	b
b	b	a	a	b	a	-	b	b	b
b	b	a	a	b	a	a	b	b	b
b	b	a	a	b	-	a	b	b	b
b	b	a	a	b	a	a	-	b	b
b	b	a	a	b	a	a	-	b	-
b	b	a	a	b	a	a	b	b	b
b	b	a	a	b	a	a	b	b	b
b	b	a	a	b	a	a	b	b	b

b	b	-	a	b	-	a	b	b	b
b	b	a	a	b	b	a	b	b	b
b	b	a	a	b	b	a	-	b	b
b	b	a	a	b	b	a	b	b	b
b	b	a	a	b	b	a	b	b	b
-	b	a	a	b	a	a	b	b	b
b	b	a	a	b	b	a	b	b	b
b	b	a	a	b	b	a	b	b	b
b	b	a	a	b	a	b	b	b	b
b	b	a	a	b	b	a	b	b	b
b	b	a	a	b	b	a	b	b	b
b	b	a	a	b	b	a	b	b	b
b	b	a	a	b	b	a	b	b	b
b	b	a	a	b	b	a	b	b	b
b	b	a	a	b	b	a	b	-	b
b	b	a	a	b	b	a	b	b	b
b	b	a	a	b	b	a	b	b	b
b	b	a	a	b	b	a	b	b	b
b	b	a	a	b	b	a	b	b	b
b	b	a	a	b	b	a	b	b	b
b	b	a	a	b	b	a	b	b	b
b	b	a	a	b	b	a	b	b	b
b	b	a	a	b	b	a	b	b	b
b	b	a	a	b	b	a	b	b	b
b	b	a	a	b	b	a	b	b	b
b	b	a	a	b	b	a	b	b	b
b	b	a	a	b	b	a	b	b	b
b	b	a	a	b	b	a	b	b	b
b	b	a	a	b	b	a	b	b	b
b	b	a	a	b	b	a	b	b	b
b	b	a	a	b	b	a	-	b	b
b	b	-	a	b	b	a	b	b	b
b	b	a	a	b	b	a	b	b	b
b	b	a	a	b	-	a	b	b	b
b	b	a	a	b	b	a	b	b	b
b	b	a	a	b	b	a	b	b	b
b	b	a	a	b	b	a	b	b	b
b	b	a	a	b	b	a	b	b	b
b	b	a	a	b	a	b	b	b	b
b	b	a	a	b	b	a	b	b	b
b	b	a	a	b	b	a	b	-	b
b	b	a	a	b	b	a	b	b	b
b	b	a	a	b	b	a	b	-	b
b	b	a	a	b	-	a	b	-	-
b	b	a	a	b	b	a	b	b	b
b	b	a	a	b	b	a	b	b	b
b	b	a	a	b	b	a	b	b	b

b	b	a	a	b	b	a	b	b	b
b	b	a	a	b	b	a	b	b	b
b	b	a	a	b	b	a	b	b	b
b	b	a	a	b	b	a	b	b	b
b	b	a	a	b	b	a	b	b	b
b	b	a	a	b	b	a	b	b	b
b	b	a	a	b	a	b	b	b	b
b	b	a	a	b	b	a	b	b	b
b	b	a	a	b	b	a	b	b	b
b	b	a	a	b	b	a	b	b	b
b	b	a	a	b	a	b	b	b	b
b	b	a	a	a	b	-	b	b	b
b	b	a	a	a	b	a	b	b	b
b	b	a	a	a	-	a	b	b	b
b	b	a	a	a	b	a	b	b	b
b	b	a	a	a	b	a	b	b	b
b	b	a	a	a	b	a	b	b	b
b	b	a	a	a	b	a	b	b	b
b	b	a	a	b	a	b	b	b	b
a	a	a	a	a	b	b	b	b	a
a	a	a	a	a	b	b	b	b	a
a	a	a	a	a	b	b	b	b	a
a	a	a	a	a	b	b	b	b	a
a	a	a	a	a	b	b	b	b	a
a	a	a	a	a	-	b	b	b	a
a	a	a	a	a	b	b	b	b	-
a	a	a	a	a	b	b	b	b	a
a	a	a	a	a	b	b	b	b	a
a	a	a	a	a	-	b	b	b	a
a	a	a	a	a	b	b	b	b	a
a	a	a	a	a	b	b	b	b	a
a	a	a	a	a	-	b	b	b	a
a	a	a	a	a	a	b	b	b	a
a	a	a	a	a	b	b	b	b	a
a	a	b	a	a	b	a	b	b	a
a	a	a	a	a	b	a	b	b	a
a	a	a	a	a	b	a	b	b	a
a	a	a	a	a	b	a	b	b	-
b	a	a	a	a	b	a	b	b	a
b	a	a	a	a	b	a	b	b	a
b	a	a	a	a	-	a	b	b	a
b	a	a	a	a	b	a	b	b	a
b	a	a	a	a	-	a	-	b	-
b	a	a	a	a	b	a	b	b	a
b	a	a	a	a	b	a	b	b	a
b	b	a	a	a	b	a	b	b	a
b	b	a	a	a	b	a	b	b	a

b	b	a	a	a	b	a	b	b	-
b	b	a	a	a	b	a	b	b	a
b	b	a	a	a	b	a	b	b	a
b	b	a	a	a	a	b	b	b	a
b	b	a	a	a	b	a	b	b	a
b	b	a	a	a	b	a	b	b	a
b	b	a	a	a	b	a	b	b	a
a	b	a	a	a	b	a	b	b	a
a	b	a	a	a	b	a	b	b	a
b	b	b	a	a	b	a	b	b	a
a	b	a	a	a	b	a	b	b	-
a	b	a	a	a	a	b	b	b	a
a	b	a	a	a	b	a	b	-	a
a	b	a	a	a	b	a	b	b	a
a	b	a	a	a	b	a	b	b	a
a	b	a	a	a	b	a	b	b	a
a	b	a	a	a	b	a	b	b	a
a	b	a	a	a	b	a	b	b	a
a	b	a	a	a	b	a	b	b	a
a	b	a	a	a	b	a	b	b	-
a	b	a	a	a	b	a	a	b	a
a	b	a	a	a	b	a	a	b	a
a	b	a	a	a	b	a	b	b	a
a	b	a	a	b	b	a	a	b	a
a	b	a	a	b	b	a	a	b	a
a	b	a	a	b	b	a	a	b	a
a	b	a	a	b	b	a	a	-	a
a	b	a	a	b	b	a	a	b	a
a	b	a	a	b	a	a	a	b	a
a	b	a	a	-	a	a	a	b	a
a	b	a	a	b	a	a	a	b	a
a	b	a	a	-	a	a	-	b	a
a	b	a	a	b	a	a	a	b	a
a	b	b	a	b	a	a	a	b	a
a	b	a	a	b	a	a	a	b	a
a	b	a	a	b	a	a	a	b	a
a	b	a	a	b	a	a	a	b	a
a	b	a	a	b	b	a	a	b	a
a	b	a	a	b	a	b	a	b	a
a	b	a	a	b	a	b	a	b	a
a	b	a	a	b	a	b	a	b	a
a	b	a	a	b	a	b	a	-	a
a	b	a	a	b	a	b	a	b	a
a	b	a	a	b	a	b	a	-	a
a	b	a	a	b	b	b	a	b	a
a	b	a	a	b	b	b	a	b	a
a	b	a	a	b	b	b	a	b	a

a	b	a	a	b	b	b	a	b	a
a	b	a	a	b	b	b	a	b	-
a	b	a	a	b	b	b	a	-	b
a	b	a	a	b	b	b	a	a	b
a	b	a	a	b	-	b	a	a	b
a	b	a	a	b	b	b	a	a	-
a	b	a	a	b	b	b	a	a	b
a	b	a	a	b	b	b	a	a	b
a	b	a	a	b	b	b	a	a	b
a	b	a	a	b	b	b	a	a	b
a	b	a	a	b	b	b	b	a	b
b	b	b	b	b	b	b	a	a	a
b	b	b	b	b	b	b	a	-	a
b	b	b	b	b	b	b	a	a	a
b	b	b	b	b	b	b	a	a	a
b	b	b	b	b	b	b	a	a	a
b	b	b	b	b	b	b	a	a	a
b	b	b	b	b	b	b	a	a	a
b	b	b	b	-	b	b	a	a	a
b	b	b	b	b	-	b	a	a	a
b	b	b	b	b	b	b	a	a	a
b	b	b	b	b	b	b	a	a	a
b	b	b	b	b	b	b	a	a	a
b	b	b	b	b	b	b	a	a	a
b	b	b	b	b	b	b	a	a	a
b	b	b	b	b	b	b	a	a	a
b	b	b	b	b	a	b	a	a	a
b	b	b	b	b	a	b	a	a	a
b	b	b	b	b	a	b	a	a	a
b	b	b	b	b	a	b	a	a	a
b	b	b	b	b	a	b	a	a	a
b	b	b	b	b	a	b	a	a	a
b	b	b	b	b	a	b	a	a	a
b	b	b	b	b	a	b	a	a	a
b	b	b	b	b	a	b	a	a	a
b	b	b	b	b	a	b	a	a	a
b	b	b	b	b	a	b	a	a	a
b	b	b	b	b	a	b	a	a	a
b	b	b	b	b	a	b	a	a	a
b	a	b	b	b	a	b	a	a	a
b	a	b	b	b	a	b	a	a	a
b	a	b	b	b	a	b	a	a	a
b	a	b	b	b	a	b	a	a	a
b	a	b	b	b	a	a	a	a	a
b	a	b	b	b	a	a	a	a	a
b	a	b	b	b	a	a	a	a	a
b	a	b	b	-	a	a	a	a	a

b	a	b	b	b	a	a	a	-	a
b	a	b	a	b	a	a	a	a	a
b	a	b	a	b	a	a	a	a	a
b	a	b	a	b	a	a	a	a	a
b	-	b	a	b	a	a	a	a	a
-	a	a	a	b	b	a	a	a	a
b	a	a	a	b	-	a	a	a	a
b	a	a	a	b	b	a	a	a	a
b	a	a	a	b	b	a	a	a	a
b	a	a	a	b	-	a	a	a	a
b	a	a	a	b	b	a	a	a	a
b	a	a	a	b	b	a	a	a	a
b	a	a	a	b	b	a	a	a	a
b	a	a	a	b	b	a	a	a	a
b	a	a	a	b	b	a	a	a	a
b	a	a	a	b	b	a	a	a	a
b	a	a	a	b	b	a	a	a	a
b	a	a	a	b	b	a	a	a	a
b	a	a	a	b	b	a	a	a	a
b	a	a	a	b	b	a	a	a	a
b	a	-	a	b	b	a	a	a	a
b	a	a	a	b	b	a	a	b	a
b	a	a	a	b	a	b	a	b	a
b	a	a	a	b	b	a	a	b	a
b	a	a	a	b	b	a	a	b	a
b	a	a	a	b	b	a	a	b	a
b	a	a	a	b	b	a	a	b	a
b	a	a	a	b	b	a	a	b	a
b	a	a	a	b	b	a	a	b	a
b	a	a	a	b	b	a	a	b	a
b	a	a	a	b	-	a	a	b	a
b	a	a	a	b	b	a	a	b	b
b	a	a	a	b	b	a	a	b	b
b	a	a	a	b	b	a	a	b	b
-	a	a	a	b	-	a	a	b	b
b	a	a	a	b	b	a	a	b	b
b	a	a	a	b	b	a	a	b	b
b	a	a	a	b	b	a	a	b	b
b	a	a	a	a	b	a	a	b	b
b	a	a	a	a	a	b	a	b	b
b	a	a	a	a	a	b	a	b	b
b	b	a	a	a	a	b	a	b	a
b	b	a	a	a	b	a	a	b	a
a	b	-	a	a	b	a	b	b	a
a	b	a	a	a	b	a	b	b	a
a	b	a	a	a	a	b	b	b	a
a	b	a	a	a	b	a	b	b	a

a	b	a	a	a	b	a	b	b	a
a	b	a	a	a	a	b	b	b	a
a	b	-	a	a	b	a	b	b	a
a	b	a	a	a	b	a	b	b	a
a	b	a	a	a	b	a	b	b	a
a	b	b	a	a	b	a	b	b	a
a	b	-	a	a	b	a	b	b	b
a	b	a	a	a	b	a	b	b	a
a	b	a	a	a	b	a	b	b	a
a	b	a	a	a	-	a	b	-	a
a	b	a	a	a	b	a	b	b	a
a	b	a	a	a	-	a	b	b	a
a	b	a	a	a	b	a	b	b	a
a	b	a	a	a	b	a	b	b	a
a	b	a	a	a	b	a	b	b	a
a	b	a	a	a	b	a	b	b	a
a	b	a	a	a	b	a	-	b	a
a	b	a	a	a	b	a	b	b	a
a	b	a	a	a	b	b	b	b	-
b	b	-	a	a	b	a	b	b	a
a	b	a	a	a	b	b	b	-	a
a	b	a	a	a	b	b	b	b	a
a	a	a	a	b	b	a	a	a	a
a	a	a	a	b	b	a	a	a	a
a	a	a	a	b	b	a	a	a	a
a	a	a	a	b	b	a	a	a	a
a	a	a	a	b	b	a	a	a	a
a	a	-	a	b	b	a	a	a	a
a	a	a	a	b	b	a	a	a	a
-	a	a	a	b	b	a	a	a	-
a	a	a	a	b	b	a	-	a	a
a	a	a	a	b	b	a	a	a	a
a	a	a	a	b	-	a	a	a	a
a	a	a	a	b	b	a	a	a	a
a	a	a	a	b	b	a	a	a	a
a	a	a	a	b	b	a	-	b	a
a	a	a	a	b	b	a	a	b	a
a	a	a	a	b	b	a	a	b	a
a	a	-	a	b	b	a	a	b	a
a	a	a	a	b	b	a	b	b	a
-	a	a	a	b	b	a	b	b	a
a	a	a	a	b	b	a	b	b	a
a	a	a	a	b	b	a	b	-	a
a	a	a	a	b	b	a	b	-	a
a	a	a	a	b	b	a	b	b	a
a	a	a	a	b	b	a	b	b	a
a	a	a	a	b	b	a	b	b	a

a	a	a	a	b	b	a	b	b	a
a	a	a	a	b	b	a	b	b	a
a	a	a	a	b	b	b	b	b	-
a	a	a	b	b	b	a	b	b	a
a	a	a	b	b	b	a	b	b	a
a	a	a	b	b	b	a	b	b	a
a	a	a	b	b	b	a	b	-	a
a	a	a	a	b	b	a	b	b	b
a	a	a	a	b	b	a	b	b	b
a	a	a	a	b	b	a	b	b	b
a	a	a	a	b	b	a	b	b	b
a	a	a	a	b	b	a	b	b	b
a	a	a	a	b	-	a	b	b	b
a	a	a	a	b	b	a	b	b	b
a	a	a	a	b	a	b	b	b	b
a	a	a	a	b	a	b	b	b	b
a	a	b	a	b	b	a	b	b	b
a	a	b	a	b	b	a	b	b	b
a	a	b	a	b	b	a	b	b	b
a	a	b	a	b	b	b	-	b	b
a	a	b	a	b	b	b	b	b	b
a	a	b	a	b	b	b	b	b	b
a	a	b	a	b	b	b	b	b	b
a	a	b	a	b	b	b	b	b	b
b	a	b	a	b	b	b	b	-	b
b	a	b	a	b	b	b	b	b	b
b	a	b	a	b	b	b	b	b	b
b	a	b	a	b	b	b	b	b	b
b	a	b	a	b	b	b	b	b	b
b	a	b	a	b	b	b	b	b	b
b	a	b	a	b	b	b	b	b	b
b	a	b	a	b	b	b	b	-	b
b	a	b	a	b	b	b	b	b	b
b	a	b	a	b	b	b	b	b	b
b	a	b	a	b	b	b	b	b	b
b	a	b	a	b	b	b	b	b	b
b	a	b	a	b	b	b	b	b	a
b	a	b	a	b	b	b	b	b	a
b	a	b	a	b	b	b	b	b	a
b	a	b	a	b	b	b	b	b	a
b	a	b	a	b	b	b	b	b	a
b	a	b	a	b	b	b	b	b	a
b	a	b	a	b	b	b	b	b	a
b	a	b	a	b	b	b	-	b	a
b	-	b	a	b	b	b	b	b	a
b	a	b	b	b	b	b	b	b	a
b	a	b	b	b	b	b	b	b	a

b	a	b	b	b	b	b	b	b	a
b	a	b	b	b	b	b	b	b	a
b	a	b	b	b	b	b	b	b	a
b	a	b	b	a	b	b	b	b	a
b	a	b	b	a	b	b	b	b	a
b	a	b	b	a	b	b	b	b	a
b	a	b	b	a	b	b	b	b	a
b	a	b	b	a	b	b	b	b	a
b	a	b	b	a	b	b	b	b	a
b	a	b	b	a	b	b	b	b	a
b	a	b	b	a	-	b	b	b	a
b	a	b	b	-	b	b	b	b	a
b	a	b	b	a	b	b	b	b	a
b	a	b	b	a	b	b	b	b	a
b	a	b	b	a	b	b	b	b	a
b	a	b	b	a	b	b	b	b	a
b	a	b	b	a	b	b	b	b	a
b	a	b	b	a	b	b	b	b	a
b	a	b	b	a	b	b	b	b	a
b	a	b	b	a	b	b	b	b	a
b	a	b	b	a	b	b	b	b	a
b	a	b	b	a	b	b	b	b	a
b	a	b	b	a	b	b	b	-	a
b	b	b	b	a	b	b	b	b	a
b	a	b	b	a	b	b	b	b	a
b	a	b	b	a	b	b	b	b	a
b	a	b	b	a	b	b	b	b	a
b	a	b	b	a	b	b	b	b	a
b	a	b	b	a	-	b	b	b	a
b	a	b	b	a	b	b	b	b	-
b	a	b	b	a	b	b	b	b	a
b	a	b	b	a	b	b	b	b	a
b	a	b	b	-	b	b	b	b	a
b	a	b	b	a	b	b	b	b	a
b	a	b	b	a	b	b	-	b	a
b	a	b	b	a	b	-	b	b	a
b	a	b	b	a	b	b	-	b	a
b	a	b	b	a	b	b	b	b	a
b	a	b	b	a	b	b	b	-	a
b	a	b	b	a	-	b	b	b	a
b	a	b	b	a	b	b	b	b	a
b	a	b	b	a	b	b	b	b	a
b	a	b	b	a	b	b	b	b	a
b	b	b	b	a	b	b	b	b	a
b	b	b	b	a	b	b	b	b	a
b	b	b	b	a	b	b	b	b	a
b	b	b	b	a	b	b	b	b	a

b	b	-	b	a	b	b	b	b	a
b	b	b	b	a	b	b	b	b	a
b	b	b	b	a	b	b	b	b	a
a	b	a	b	a	a	b	b	a	a
a	b	b	b	a	b	a	b	-	a
a	b	b	b	a	b	a	b	a	b
a	b	b	b	a	a	b	b	a	b
a	b	b	b	a	b	a	b	a	b
a	a	b	b	b	b	a	b	a	b
a	a	b	b	b	b	a	b	a	b
a	a	b	b	b	b	a	b	a	b
a	a	b	b	b	b	a	b	a	b
a	a	b	b	b	b	a	b	a	b
a	a	b	b	b	b	a	-	a	b
a	a	b	b	b	b	a	b	a	b
a	a	b	b	b	b	a	b	a	b
a	a	b	b	b	a	b	b	a	b
a	a	b	b	b	b	a	b	a	b
a	a	b	b	b	b	a	b	a	b
a	a	b	b	b	b	a	b	a	-
a	a	b	b	b	b	a	b	a	b
a	a	a	b	-	b	a	b	a	b
a	a	b	b	b	b	a	b	a	b
a	a	b	b	b	b	a	b	a	b
a	a	b	a	b	b	a	b	a	b
a	a	b	a	b	a	b	b	a	b
a	a	b	a	b	b	a	b	a	b
a	a	b	a	b	b	a	b	b	b
a	a	b	a	b	b	a	b	b	b
a	a	b	a	b	a	b	b	b	b
a	a	b	a	b	a	b	b	b	b
a	a	b	a	b	b	a	b	b	b
a	a	b	a	b	a	b	b	b	b
a	a	b	a	b	b	a	b	b	b
a	a	b	a	b	a	b	b	b	b
a	a	b	a	b	b	a	b	b	b
a	a	b	a	b	b	a	-	b	b
a	a	b	a	b	a	b	b	a	b
a	a	b	a	b	b	a	b	a	b
a	a	b	a	b	b	a	b	-	-
a	a	b	a	b	b	a	b	a	a
a	a	b	a	b	b	a	b	a	a
a	a	b	a	b	b	a	b	a	a
a	a	b	a	b	b	a	b	a	a
a	a	b	a	-	b	a	-	a	a
a	a	b	b	b	-	a	b	a	a
a	a	b	b	b	b	a	b	a	a

a	a	b	b	b	b	a	b	a	a
a	a	b	b	b	-	a	-	a	a
a	a	b	b	b	b	a	b	-	a
b	a	b	b	b	a	b	b	a	a
b	a	-	b	a	-	a	b	a	a
b	a	b	b	a	b	b	b	a	a
b	a	b	b	a	b	b	b	a	a
b	a	b	b	a	b	b	b	a	a
b	a	b	b	a	b	b	b	a	a
b	a	b	b	a	b	b	b	a	a
b	a	b	b	a	b	b	b	a	a
b	a	b	b	b	b	b	b	a	a
b	a	b	b	b	b	b	b	a	a
b	a	b	b	b	b	b	b	a	a
b	a	b	b	b	b	b	b	a	a
b	a	b	b	b	b	b	b	-	a
b	a	b	b	b	b	b	b	a	a
b	a	b	b	b	b	b	b	-	a
b	a	b	b	b	b	b	b	a	-
b	a	b	b	b	b	b	b	a	a
b	a	b	b	b	b	-	b	a	a
b	a	b	b	b	b	b	b	a	a
b	a	b	b	b	b	b	b	a	-
b	a	b	b	b	b	b	b	a	a
b	a	b	b	b	b	b	b	a	a
a	a	b	a	b	a	b	b	a	a
a	a	b	a	b	a	b	b	a	a
a	a	b	a	b	a	b	b	a	a
a	a	-	a	b	a	b	-	a	a
a	a	b	a	b	a	b	b	a	a
a	a	b	a	b	b	a	b	a	a
a	a	b	a	b	b	a	b	a	a
a	a	b	a	b	b	a	b	a	a
a	a	b	a	-	b	-	b	a	a
a	a	b	a	b	b	b	b	a	a
b	a	b	a	b	b	b	b	a	a
b	a	b	a	b	b	b	b	a	a
b	a	b	a	b	b	b	b	a	-
b	a	b	a	b	b	b	b	a	a
b	a	b	a	b	b	b	b	b	a
b	a	b	a	b	b	b	b	b	a
b	a	b	a	b	b	b	b	b	a
b	a	b	a	b	b	b	b	b	a
b	a	b	a	b	a	b	a	b	a
b	a	b	a	b	b	b	b	b	a
b	a	b	b	b	b	b	b	-	a
b	a	b	b	b	b	b	b	b	a

b	a	b	b	b	b	b	b	b	a
b	a	b	b	b	b	b	b	b	a
b	a	b	b	b	b	b	b	b	a
b	a	b	b	b	b	b	b	b	a
b	a	b	b	b	b	b	b	b	a
b	a	b	b	b	b	b	b	b	-
b	a	b	b	b	b	b	-	b	-
b	a	a	b	b	b	b	b	b	a
b	a	a	b	b	b	b	b	b	a
b	a	a	b	b	b	b	b	b	a
b	a	a	b	b	b	b	b	b	-
b	a	a	b	-	b	b	b	b	a
a	a	a	b	b	-	b	b	b	a
a	a	a	b	b	b	b	b	b	a
a	a	a	b	b	b	b	b	b	a
-	b	a	b	b	b	b	b	b	a
-	b	a	b	b	b	b	b	b	a
a	b	a	b	b	b	b	b	b	a
-	b	a	b	b	b	b	b	b	a
a	b	a	b	b	b	b	b	b	a
a	b	a	b	b	b	b	b	b	a
a	b	a	b	b	b	b	b	b	a
a	b	a	b	b	b	b	b	b	a
a	b	a	b	b	b	b	b	b	a
a	b	a	b	b	b	b	b	b	a
a	b	a	b	b	b	b	b	b	a
a	b	a	b	b	b	a	b	b	a
a	b	a	b	b	b	a	b	b	a
a	b	a	b	b	a	b	b	b	a
a	b	a	b	b	a	b	b	b	a
a	b	a	b	b	a	b	-	b	a
a	b	a	b	b	a	b	b	b	a
-	b	a	b	b	a	b	b	b	-
b	b	a	b	b	a	b	a	a	a
b	b	a	b	b	a	b	a	a	a
b	b	a	b	b	b	b	a	a	a
b	b	a	b	b	b	b	a	-	a
b	b	a	b	b	b	b	a	a	a
b	b	a	b	b	b	b	a	a	a
b	b	a	b	b	b	b	a	a	a
b	b	a	b	b	b	b	a	a	a
b	b	a	b	b	b	b	a	-	a
b	b	a	b	b	b	b	a	a	a
b	b	-	b	b	b	b	a	-	a
b	b	a	b	b	b	b	a	a	a
b	b	a	b	b	b	b	a	a	a
b	b	a	b	b	b	b	a	a	a

b	b	a	b	b	b	b	a	a	a
b	b	a	b	b	-	b	a	a	a
b	b	a	b	b	b	b	-	a	b
b	b	a	b	b	b	b	a	a	b
b	b	a	b	b	b	b	a	a	b
b	b	a	b	b	b	b	a	a	b
b	b	a	b	b	b	b	a	b	b
b	-	a	b	b	b	b	a	b	b
b	b	a	b	b	b	b	a	a	b
b	b	b	b	b	b	b	a	a	a
b	b	-	a	b	b	b	a	a	a
b	b	b	a	b	b	b	a	a	a
b	b	b	a	b	b	b	a	a	-
-	b	b	a	b	-	b	a	a	a
b	b	b	a	b	a	b	a	a	a
b	b	b	a	b	a	b	a	a	-
b	b	b	a	b	a	b	a	a	-
b	b	b	a	b	a	b	a	a	a
b	b	b	-	b	-	b	a	a	a
b	b	-	a	b	a	b	a	a	a
b	b	b	a	b	-	b	b	a	a
b	b	b	b	b	a	b	b	a	a
b	b	b	b	b	a	b	b	a	a
b	b	b	b	b	a	b	b	a	a
b	b	b	b	b	-	b	b	a	a
b	b	b	b	b	-	b	b	a	a
b	b	b	b	b	a	b	b	a	a
-	b	b	b	b	b	a	b	a	a
-	b	b	b	b	a	b	-	a	a
b	b	b	b	b	-	b	b	a	a
b	b	b	b	b	a	b	b	a	a
b	b	-	b	b	a	b	b	a	a
b	b	b	b	b	a	b	b	a	a
b	a	b	b	b	a	b	b	a	a
b	a	b	b	b	a	b	b	a	a
a	a	b	b	b	b	a	b	a	a
a	a	b	b	b	a	b	b	a	a
a	a	a	b	a	b	a	b	a	a
a	a	a	b	a	a	b	b	a	a
a	a	a	b	a	a	b	b	a	a
a	a	a	b	a	a	b	b	a	a
a	a	a	b	a	a	b	b	a	a
a	a	b	b	a	a	b	b	a	a
a	a	b	b	a	a	b	b	a	a
a	a	b	b	a	a	b	b	a	a
a	a	b	b	a	a	b	b	-	a
a	a	a	b	-	a	a	a	a	a

a	a	a	b	a	a	a	a	a	a
a	a	a	b	a	a	a	a	a	a
a	a	a	b	a	a	a	a	b	a
a	a	a	b	a	a	a	a	b	a
a	a	a	b	a	a	a	a	b	a
a	a	-	b	a	a	a	a	b	b
a	a	a	b	a	-	a	a	b	b
a	a	a	b	a	a	a	a	b	b
a	b	a	a	b	-	b	b	a	a
a	b	a	a	b	a	b	b	a	a
a	b	a	a	b	b	a	b	a	a
a	b	a	a	b	b	a	b	a	a
a	b	a	a	b	a	b	b	a	a
a	b	a	a	b	a	b	b	-	a
a	b	a	a	b	a	b	b	a	a
a	b	a	a	b	a	b	b	a	a
a	b	a	a	b	-	b	b	a	a
a	b	a	a	b	a	b	b	a	a
a	b	a	a	b	a	b	b	a	a
a	b	a	a	a	b	a	b	a	a
a	b	a	a	a	a	b	b	a	a
a	b	a	a	a	a	b	b	a	a
a	b	a	a	a	a	b	b	a	a
b	b	a	a	a	a	b	a	b	a
b	b	a	a	a	a	b	a	b	a
b	b	a	a	a	a	-	a	b	a
b	b	a	a	a	a	b	a	b	a
b	a	a	a	a	-	b	a	-	-
b	a	a	a	a	b	a	a	b	a
b	a	a	b	-	a	b	b	b	a
b	a	a	b	a	a	a	b	b	a
b	a	a	b	a	a	-	b	b	a
b	a	a	b	a	a	a	b	b	a
b	a	a	b	a	a	a	b	b	-
b	a	a	b	a	a	a	b	b	a
b	a	a	b	a	a	a	b	b	a
b	a	a	b	a	a	a	b	b	a
a	b	a	b	a	a	a	b	b	a
b	a	a	b	a	a	a	b	b	a
b	a	a	b	a	a	a	b	b	a
b	a	a	b	a	a	a	b	b	a
-	a	a	b	a	a	a	-	b	a
b	a	a	-	a	a	a	b	b	a
b	b	a	b	b	a	a	a	b	a
b	b	b	b	b	a	a	a	b	a
b	b	a	b	b	a	a	a	b	a
b	b	a	b	b	a	a	a	b	a

-	b	a	b	b	a	a	a	b	a
b	b	a	b	b	a	a	a	b	a
b	b	b	b	b	a	a	a	b	a
b	b	b	b	b	a	a	a	b	a
b	b	b	b	b	a	a	a	b	a
b	b	b	b	b	a	a	a	-	a
b	b	b	b	b	a	a	a	b	a
b	b	b	b	b	a	a	a	b	a
b	b	b	b	b	b	a	-	b	a
b	b	b	b	b	b	a	a	b	a
b	b	b	b	b	b	a	a	-	a
b	b	b	b	b	b	a	a	b	a
b	a	b	b	b	a	a	a	a	b
b	a	b	b	b	-	a	a	a	b
b	a	b	b	b	a	a	a	a	b
b	a	b	b	b	a	a	a	a	b
b	a	b	b	b	a	a	a	a	b
b	a	b	b	b	a	a	a	a	b
b	a	b	a	b	a	a	a	a	b
b	a	b	a	b	-	a	a	-	b
-	a	b	a	b	a	a	a	a	b
b	a	b	a	b	a	a	a	a	b
b	a	b	a	b	a	a	-	a	b
b	a	b	a	b	a	a	a	a	b
b	a	b	a	b	a	a	a	a	b
b	a	b	a	b	a	a	-	a	b
b	a	b	a	b	a	a	a	a	b
a	a	b	a	b	a	a	a	a	b
a	a	b	a	b	a	a	a	a	b
a	a	b	a	b	a	a	a	a	b
a	a	b	a	b	a	a	a	a	b
a	a	b	a	b	a	a	a	a	b
a	a	b	a	b	a	a	a	a	b
a	a	b	a	b	a	a	a	a	a
a	a	b	a	b	a	a	a	a	a
a	b	b	b	b	a	a	a	-	a
a	b	b	b	b	a	a	a	a	a
a	b	b	b	b	a	a	a	a	a
a	b	b	b	b	a	a	a	-	a
a	b	b	b	b	a	a	a	a	a
a	b	b	b	b	a	a	a	a	b
a	b	b	b	b	a	a	a	a	a
a	b	b	b	b	a	a	a	a	a
a	b	b	b	b	a	a	a	a	a
a	b	b	b	b	a	a	a	a	a
a	b	b	b	b	a	a	a	b	b
a	b	b	b	b	a	a	a	b	b

a	b	b	b	b	a	a	a	b	b
a	b	b	b	b	a	a	a	b	b
a	b	b	b	b	a	a	a	-	b
-	b	b	b	b	a	a	a	b	b
a	b	-	b	b	a	a	a	b	b
a	b	b	b	b	a	a	a	b	b
a	b	b	b	b	a	a	-	b	b
a	b	b	b	a	a	a	a	b	b
a	b	a	a	b	a	a	b	a	a
a	b	a	a	b	a	a	b	a	-
a	b	b	a	b	a	a	b	a	a
a	b	b	a	b	a	a	b	a	a
a	b	b	a	b	a	a	b	a	a
a	b	b	a	b	a	a	b	a	a
a	b	b	a	b	a	a	b	a	a
a	b	b	a	b	a	a	b	-	a
a	b	b	a	b	a	a	b	a	a
a	b	b	a	b	b	a	b	a	a
a	b	b	a	b	b	a	b	a	a
a	b	b	a	b	b	a	b	a	a
a	b	b	a	b	a	b	b	a	a
a	b	b	a	b	b	a	b	a	a
a	b	b	a	-	b	a	b	a	a
a	b	b	a	b	b	a	b	a	a
a	b	b	a	b	b	a	b	a	a
a	b	b	a	b	b	a	b	a	a
a	b	b	a	b	b	a	b	a	a
a	b	b	a	b	-	a	b	a	b
a	b	b	a	b	-	a	b	a	b
a	b	b	a	b	b	a	b	a	b
-	b	b	a	b	b	a	b	a	b
a	b	b	a	b	b	a	b	a	b
a	b	b	a	b	b	a	b	a	b
a	b	b	a	b	a	b	b	a	b
a	b	b	a	b	b	a	b	a	b
a	b	b	a	b	b	a	b	a	b
a	b	b	a	b	b	a	b	a	b
b	b	b	a	b	b	a	a	a	b
b	b	b	a	b	-	a	a	a	b
b	b	b	a	b	b	a	a	a	b
b	b	b	a	b	b	a	a	a	-
b	b	b	a	b	b	a	a	a	b
a	b	b	a	b	b	a	a	a	b
a	b	b	a	b	b	a	a	a	b
a	b	-	a	b	b	a	a	a	b
a	b	b	a	b	b	a	a	a	b
a	b	b	a	b	a	a	a	a	b

a	b	b	a	b	-	a	a	a	-
a	b	b	a	b	a	b	a	a	b
a	b	b	a	a	a	b	a	a	b
a	b	b	a	b	b	a	a	a	b
a	b	b	a	b	a	b	a	a	b
a	b	b	a	b	b	a	a	a	b
a	a	b	a	b	a	b	a	a	b
a	a	b	a	b	b	a	a	a	b
a	a	b	a	b	a	b	a	a	b
a	a	b	a	b	b	a	a	b	b
a	a	b	a	b	b	a	a	b	b
a	a	b	a	b	b	a	a	b	b
a	a	b	a	b	a	b	a	b	b
a	a	b	a	b	b	a	a	-	b
a	a	b	a	b	b	a	a	b	-
a	a	b	a	b	b	a	a	b	b
a	a	b	a	b	b	a	a	b	b
a	a	b	a	b	a	b	a	b	b
a	a	a	a	b	a	a	a	b	-
a	a	a	a	b	a	a	a	b	a
b	a	b	b	a	-	a	b	a	-
b	a	b	b	a	a	a	b	a	a
b	a	b	b	a	a	a	b	-	a
b	a	b	b	a	a	a	b	a	a
b	a	b	b	a	a	a	b	a	a
b	a	b	b	a	b	a	b	a	a
b	a	b	b	a	b	a	b	a	a
b	a	b	b	a	a	b	a	a	a
b	a	b	b	a	b	a	b	a	-
b	a	b	b	a	a	b	b	a	a
b	a	b	b	a	b	a	b	a	a
b	a	b	b	a	a	b	b	a	a
b	a	b	b	a	b	a	b	a	a
b	a	b	b	a	a	b	b	a	a
b	a	b	b	a	b	a	b	a	a
b	a	b	b	a	b	a	b	a	a
b	a	b	b	a	b	a	b	a	a
b	a	b	b	a	b	a	b	a	a
b	a	b	b	a	b	a	b	a	a
b	a	b	b	a	b	a	b	a	a
b	a	b	b	a	a	b	a	a	a
b	a	b	b	a	-	a	a	a	a
-	a	b	b	a	b	a	a	a	a
b	a	a	b	a	b	a	a	a	a
b	a	a	b	a	a	b	a	a	a
b	a	a	b	a	b	a	a	a	a
a	a	a	b	a	a	b	a	a	a

a	a	a	b	a	b	a	a	a	-
a	a	a	b	a	b	a	a	a	a
a	a	a	b	a	b	a	a	a	a
a	a	a	b	a	-	a	a	a	a
a	a	a	b	a	b	a	a	a	a
a	a	a	b	a	b	a	a	a	a
a	a	a	b	a	b	a	a	a	a
a	a	a	b	a	b	a	a	a	a
a	a	a	b	a	a	b	a	a	a
a	a	a	b	a	b	a	a	a	a
a	a	a	b	a	b	a	a	a	a
a	a	a	b	a	b	a	a	a	a
a	a	a	b	a	b	a	a	a	a
a	a	a	b	a	b	a	a	a	a
a	a	a	b	a	b	a	a	a	a
a	a	a	b	a	b	a	a	-	a
a	a	a	b	a	b	a	a	a	a
a	a	a	b	a	b	a	a	-	a
a	a	a	b	a	b	a	a	a	a
a	a	a	b	a	b	a	-	-	a
a	a	a	b	a	b	a	a	a	b
a	a	a	b	a	b	a	a	a	b
a	a	a	b	a	b	a	a	a	b
a	a	a	b	a	-	a	a	a	b
a	a	a	b	a	b	a	a	a	b
a	a	a	b	a	b	a	a	a	b
a	a	a	b	a	b	a	a	a	b
a	a	a	b	a	b	a	a	-	b
a	a	a	a	a	b	a	a	a	-
a	a	a	a	a	b	a	a	a	b
a	a	a	a	a	b	a	a	a	b
a	a	a	a	a	b	a	-	a	b
a	a	a	a	a	a	a	-	a	b
a	a	a	a	a	a	a	a	a	-
a	a	a	a	a	a	a	a	a	b
b	b	b	b	b	a	b	b	b	b
b	b	b	b	b	b	a	b	b	b
b	b	b	b	b	b	a	b	b	-
b	b	-	b	b	b	a	b	b	b
b	b	b	b	b	b	a	b	b	b
b	b	b	b	b	a	a	b	b	b
b	b	b	b	b	a	a	b	a	b
b	b	b	b	b	a	a	b	a	a
b	b	b	b	b	a	a	b	a	a
a	a	b	b	b	a	a	b	a	a
a	a	b	b	b	a	a	b	a	a
a	a	a	a	b	a	a	b	a	a

a	a	b	b	b	a	a	b	a	a
a	a	b	b	b	a	a	b	a	a
a	a	b	b	b	a	a	b	a	a
a	a	b	b	b	a	a	b	a	a
a	a	b	a	b	a	a	b	a	a
a	a	b	a	b	a	a	b	a	a
a	a	b	a	b	a	a	b	a	a
a	a	b	a	b	a	a	b	a	a
a	a	b	a	b	a	a	-	a	a
a	a	a	a	b	a	a	b	a	a
a	a	a	a	b	a	a	a	a	a
a	a	a	a	b	a	a	a	a	a
a	a	a	a	b	a	a	a	a	a
a	a	a	a	b	-	a	a	a	a
a	a	a	a	b	a	a	a	a	a
a	a	a	a	b	a	a	a	a	a
a	a	a	a	b	a	b	a	b	a
a	a	a	b	b	a	b	a	b	a
a	a	a	b	b	a	b	a	b	a
a	a	a	b	b	a	b	a	b	a
b	a	a	b	b	-	b	a	b	a
b	a	a	b	b	a	b	a	b	a
b	a	a	b	b	a	b	a	-	a
b	a	a	b	b	a	b	a	b	a
b	a	a	b	b	a	b	a	b	a
b	a	a	b	-	a	b	a	-	a
b	a	a	b	b	a	b	a	b	-
-	a	a	b	b	a	b	a	b	a
b	a	a	b	b	a	b	a	b	a
b	a	a	b	b	a	b	a	b	a
b	a	a	b	b	a	b	a	b	-
b	a	a	b	b	a	b	a	-	a
b	a	a	b	b	a	b	a	b	a
b	a	a	b	b	-	b	a	b	a
b	a	a	b	b	b	a	a	b	a
b	a	a	b	b	a	b	a	b	a
b	a	a	b	b	b	a	a	a	a
b	a	a	b	b	b	a	a	a	a
b	a	a	b	b	a	b	a	a	a
b	a	a	b	b	-	b	a	a	a
b	a	a	b	b	a	b	a	a	a
a	a	a	b	b	a	b	a	a	a
b	a	a	b	b	a	b	a	a	a
b	a	a	b	b	b	a	a	a	a
b	a	a	b	b	b	a	a	a	a
b	a	a	b	b	a	b	a	a	a
a	a	b	b	a	a	b	b	b	a

a	a	b	b	a	a	b	b	b	a
a	a	b	b	a	b	a	b	b	a
a	a	b	b	a	a	b	b	b	a
a	a	b	b	a	a	b	b	b	a
a	a	b	b	a	a	b	b	b	a
a	a	b	b	a	a	b	b	b	a
a	a	a	b	a	a	b	b	b	a
a	a	a	b	a	b	a	b	b	a
a	a	a	b	a	a	b	b	b	a
a	a	a	b	a	a	b	b	b	a
a	a	a	b	a	a	b	b	b	a
a	a	a	b	a	a	b	a	b	a
a	a	a	b	a	a	b	a	b	a
a	a	a	b	a	a	b	a	b	a
a	a	a	b	a	a	b	a	b	a
a	a	a	b	a	a	b	a	b	a
a	a	a	b	a	a	b	a	b	a
a	a	a	b	a	b	a	a	b	a
a	a	a	b	a	b	b	a	b	a
a	a	a	b	a	b	b	a	b	a
a	a	a	b	a	b	b	a	b	a
a	a	a	b	a	b	b	a	b	a
a	a	a	b	a	b	b	a	b	a
a	a	a	b	a	b	b	a	b	a
a	a	a	b	a	b	b	a	b	a
a	a	a	b	a	b	b	a	b	a
a	a	a	b	a	b	b	a	b	a
a	a	a	b	a	b	b	a	b	a
a	a	a	b	a	b	b	a	b	a
a	b	a	b	a	b	b	a	b	a
a	b	a	b	a	b	b	a	b	a
a	b	a	b	a	b	b	a	b	a
a	b	a	b	a	b	b	a	b	a
a	b	a	b	a	b	b	a	b	a
a	b	a	b	a	b	b	a	b	a
a	b	a	b	a	b	b	a	b	a
a	b	a	b	a	b	b	a	b	a
a	b	a	b	a	b	b	a	b	a
a	b	a	b	a	b	b	a	b	a
a	-	a	b	a	b	b	a	b	b
b	b	a	a	a	a	a	a	a	a
a	a	a	b	a	b	b	-	a	a
a	a	a	b	a	b	b	b	a	a
a	a	a	b	a	b	b	b	a	a
a	a	a	b	a	b	b	b	a	a
a	a	a	b	a	b	b	b	a	a
a	a	a	b	a	b	b	b	a	a

a	a	a	b	a	b	b	b	a	a
a	a	a	b	a	b	b	b	a	a
a	a	a	b	a	b	b	b	a	a
a	a	a	b	a	b	b	b	a	a
a	a	a	b	a	b	b	b	a	a

YW100	YW101	YW103	YW104	YW105	YW106	YW107	YW108	YW109	YW110
a	a	a	a	b	b	a	a	a	a
a	a	b	-	b	b	a	a	a	a
a	a	b	a	b	b	a	a	a	b
a	a	-	a	b	b	a	a	a	a
a	a	b	a	b	b	a	a	a	a
a	a	b	a	b	b	a	a	a	a
b	a	b	a	-	b	a	a	a	a
b	a	b	a	-	b	a	a	a	a
b	a	b	a	b	b	a	a	b	a
b	a	b	a	b	b	a	a	a	a
-	a	b	a	b	b	a	a	a	a
b	a	b	a	b	b	a	a	a	a
b	a	b	-	b	b	a	a	a	a
b	a	b	a	b	b	a	a	a	a
b	a	b	a	b	b	a	a	a	b
b	a	b	a	-	b	a	a	a	a
b	a	b	a	b	b	a	a	a	a
b	a	b	-	b	b	a	a	a	a
b	a	b	a	b	b	a	-	a	a
b	a	b	a	b	b	a	-	a	-
b	a	b	a	b	b	a	a	a	a
b	a	b	a	b	b	a	a	a	a
b	-	b	a	b	b	a	a	a	a
b	a	b	a	b	b	a	a	a	-
b	a	b	a	b	b	a	a	a	-
b	a	b	-	b	b	a	a	a	a
b	a	b	-	b	b	a	a	-	-
-	a	b	a	b	b	a	a	a	a
b	a	b	a	b	b	a	a	a	a
b	a	b	a	b	b	a	a	a	a
b	a	b	a	b	b	a	a	a	a
b	a	b	a	b	b	a	a	a	a
-	a	b	a	b	b	a	a	a	a
b	a	b	a	b	b	b	a	a	a
b	a	b	a	b	b	b	a	a	a
b	a	b	a	b	b	b	a	-	-
b	a	-	a	b	b	b	a	a	a
b	a	b	a	b	b	b	a	a	a
b	a	b	a	b	b	b	a	a	a
b	-	b	a	b	b	b	a	a	a
b	a	b	a	b	b	b	a	a	a
b	a	b	a	b	b	b	a	b	a
b	a	b	a	b	b	b	a	b	b
b	a	b	a	b	b	b	-	b	b
-	a	b	a	b	b	b	a	b	b

b	a	b	a	b	b	b	a	b	b
-	a	b	a	b	b	b	a	b	b
b	a	b	a	b	b	b	a	b	-
b	a	b	a	b	b	b	b	b	b
b	-	b	a	b	b	b	-	b	b
b	a	b	a	b	b	b	b	b	b
b	a	b	-	b	b	b	b	b	b
b	a	b	b	b	b	b	b	b	b
b	a	b	b	-	b	b	-	b	b
b	a	b	b	b	b	b	b	b	b
b	a	b	-	b	-	b	b	-	b
b	a	b	b	b	-	b	b	b	b
b	a	b	-	b	a	b	b	-	-
b	a	b	-	b	a	b	b	b	b
b	a	b	b	a	a	b	b	b	b
b	a	b	b	a	a	b	b	b	b
b	a	b	b	a	a	b	b	-	b
-	a	b	b	a	a	b	b	b	b
b	a	b	b	a	a	b	b	b	b
b	a	b	b	a	a	b	b	b	b
-	b	b	b	a	a	b	b	b	b
b	a	b	b	a	a	b	b	b	b
-	a	-	b	a	a	b	b	b	a
b	a	b	b	a	a	b	b	b	b
b	a	b	b	a	a	b	b	b	b
b	a	b	b	a	a	b	b	b	b
b	a	b	b	a	a	b	b	b	-
-	a	b	b	a	a	b	b	b	b
b	a	b	b	a	a	b	b	b	b
b	a	b	b	-	a	b	b	b	b
b	a	a	b	a	a	b	-	b	a
b	a	a	b	a	a	b	b	b	b
b	a	a	b	a	a	b	-	b	b
-	a	a	b	a	a	b	-	b	b
b	a	a	-	a	a	b	-	b	-
b	a	a	b	a	a	b	b	b	-
b	a	a	b	a	a	b	b	b	b
b	a	a	b	a	a	b	b	b	a
b	a	a	b	a	a	b	b	-	-
a	a	a	b	a	a	b	b	b	b
a	a	a	b	a	a	b	b	b	b
a	a	a	b	a	a	b	b	b	a
-	a	a	b	a	a	b	-	b	-
a	a	-	b	a	a	b	b	b	b
a	a	a	b	a	a	b	b	b	b
a	a	a	b	a	a	b	b	b	b
a	a	a	b	a	a	b	-	b	b

a	a	a	b	a	a	b	b	b	b
a	a	a	b	a	a	b	b	b	b
a	a	a	b	a	a	b	-	b	b
a	a	a	b	a	a	b	b	b	b
a	a	a	b	a	a	b	b	b	b
a	a	a	b	a	a	a	-	b	a
a	a	a	b	a	a	a	b	b	a
a	a	a	b	a	a	a	b	b	a
a	a	a	b	a	a	a	b	b	a
a	a	a	b	a	a	a	b	b	-
a	a	a	-	a	a	a	b	b	a
a	-	a	b	a	a	a	b	b	a
a	a	a	a	a	a	a	b	b	-
a	a	a	a	a	a	a	b	b	a
a	a	-	-	a	a	a	b	b	-
a	a	a	a	a	a	a	b	b	a
a	a	a	a	a	a	a	b	b	a
a	a	a	a	-	a	a	b	b	a
a	a	a	a	a	a	a	b	b	a
a	a	a	a	a	a	a	b	-	a
a	a	a	a	a	a	a	b	b	b
a	a	a	a	a	b	a	-	b	a
a	a	a	a	a	b	a	b	b	a
a	a	a	a	a	b	a	b	b	a
a	-	b	a	a	b	a	b	b	-
a	a	a	a	a	b	a	b	b	a
a	a	a	a	a	b	a	b	b	b
a	a	a	a	a	b	a	b	b	a
a	b	a	a	a	b	a	b	b	-
a	a	a	a	a	b	a	b	b	a
a	a	a	a	a	b	a	-	b	a
a	a	a	a	a	b	b	b	b	a
a	a	a	a	a	b	b	b	b	a
a	a	a	a	a	-	b	b	b	a
a	a	a	a	a	b	b	b	b	a
a	a	a	a	a	b	b	b	b	a
a	a	a	a	a	b	b	b	b	a
a	a	a	a	a	b	b	b	b	a
a	-	a	a	a	b	b	b	b	a
a	a	a	a	a	b	b	a	b	a
a	a	a	-	a	b	b	b	b	a
a	a	a	a	a	b	b	-	b	a
a	a	a	a	a	b	b	b	b	a
a	a	a	a	a	b	b	b	b	a
a	a	a	-	a	b	b	b	b	-
a	a	a	a	b	b	b	b	b	a
a	a	a	a	b	b	b	-	b	a

-	a	a	a	b	b	a	b	b	-
a	a	a	a	b	a	a	b	b	a
a	a	a	-	b	-	a	b	b	a
a	a	a	a	b	a	a	b	b	-
a	a	a	a	b	a	a	-	b	a
a	a	a	a	b	a	a	b	b	b
a	a	a	a	b	a	a	b	b	a
a	a	a	a	b	a	a	b	b	a
a	-	a	a	b	a	a	b	b	a
a	a	a	a	b	a	a	b	b	a
a	a	a	a	b	a	a	b	b	a
a	a	a	a	b	a	a	b	b	a
a	a	a	a	b	a	a	b	b	a
a	a	a	a	b	a	a	-	b	a
a	a	a	a	b	a	a	b	b	a
a	b	-	a	b	a	a	b	b	a
a	a	a	a	b	a	a	b	b	a
a	a	a	a	b	a	a	b	b	a
-	a	a	a	b	a	a	b	b	a
b	a	a	a	b	a	a	b	b	-
b	a	a	a	b	a	a	b	b	a
b	-	a	a	b	a	a	b	b	a
b	a	a	a	b	a	a	b	b	a
b	a	a	a	b	a	a	b	b	-
b	a	a	a	b	a	a	b	b	a
b	a	a	a	b	a	a	b	b	a
b	a	a	a	b	a	a	b	b	a
b	a	a	a	b	a	a	b	b	a
-	a	a	a	b	a	a	b	b	a
a	-	a	a	b	a	a	b	b	a
a	a	a	a	b	a	a	b	b	a
a	-	a	a	b	a	a	b	b	a
a	a	a	a	b	a	a	b	b	a
a	a	a	a	b	a	a	b	b	a
-	a	a	a	b	a	a	b	b	a
a	a	a	a	b	a	a	b	b	a
-	a	a	a	b	a	a	b	b	b
a	a	a	a	b	a	a	b	b	b
a	a	a	a	b	a	a	b	b	b
a	a	a	a	b	a	a	b	b	b
a	a	a	a	b	a	a	b	b	b
a	a	a	a	b	a	a	b	-	a
a	a	a	a	b	a	a	-	b	a
a	a	a	a	b	a	a	b	-	a
a	a	a	a	b	a	a	-	b	a
a	a	a	a	b	a	a	b	b	a
a	a	a	a	b	a	a	b	b	a

a	a	a	a	b	a	a	b	b	a
a	-	a	a	b	a	a	b	b	a
a	a	a	a	b	a	a	b	a	a
a	a	a	a	b	a	-	b	a	a
a	a	a	a	b	a	a	b	a	-
a	a	a	a	b	a	a	b	a	a
a	a	a	a	b	a	a	b	a	a
a	a	b	a	b	a	a	b	a	a
a	a	b	a	b	a	a	-	a	a
a	a	b	a	b	a	a	b	a	a
a	a	-	a	b	a	a	b	a	a
-	a	b	a	b	a	a	b	a	-
a	a	a	b	b	a	b	b	b	b
a	a	a	b	b	a	b	-	b	b
a	a	a	b	b	a	b	b	b	b
a	a	a	b	b	a	b	b	b	b
a	a	a	b	-	a	b	b	b	-
a	a	a	b	b	a	b	b	b	-
a	a	a	b	b	a	b	b	b	a
a	a	a	b	b	a	b	b	b	b
a	a	a	b	b	a	b	b	b	a
a	a	a	b	b	a	b	b	b	a
a	-	a	b	b	a	b	b	b	a
-	a	a	b	b	a	b	b	b	a
a	b	a	b	b	a	b	b	b	a
a	a	a	b	b	a	b	b	b	a
a	a	a	b	b	a	b	b	a	a
a	a	a	b	b	a	b	b	b	a
a	a	a	b	b	a	b	b	b	-
a	a	a	b	b	a	b	b	b	-
a	a	a	b	b	a	b	b	b	a
a	a	a	a	b	a	b	b	b	a
a	a	a	a	b	a	b	b	b	a
a	a	a	a	b	a	b	b	b	a
a	a	a	a	b	a	b	b	b	a
a	a	a	a	b	a	b	b	b	a
-	-	a	a	b	a	b	b	b	b
a	a	a	a	b	a	b	b	b	b
a	-	b	a	b	a	b	b	b	-
a	a	b	a	b	a	b	b	b	a
a	-	b	a	b	a	b	b	a	b
a	a	b	a	b	a	b	b	a	a
a	a	b	-	b	a	b	b	a	a
a	a	b	a	b	a	b	b	a	a
a	-	b	a	b	a	b	-	a	a
a	a	b	a	b	a	b	b	a	a
a	b	b	a	b	a	b	a	a	a

a	a	b	a	b	a	b	a	a	a
a	a	b	-	b	a	b	a	a	a
a	a	b	a	b	a	b	a	a	a
a	-	b	a	b	a	b	a	a	a
a	a	b	a	b	a	b	a	a	b
a	a	b	a	-	a	b	a	a	a
a	a	b	-	b	a	b	a	a	a
a	a	b	a	b	a	b	a	a	a
a	a	b	-	b	a	b	a	a	b
a	a	b	-	b	a	b	a	a	a
a	a	b	a	b	a	b	a	a	b
a	a	b	a	b	a	b	a	a	a
a	a	b	-	b	a	b	a	a	b
a	a	b	a	b	a	b	a	-	b
a	a	b	a	b	a	b	a	a	b
a	a	b	a	b	a	b	a	a	a
a	a	b	a	b	a	b	a	a	b
a	-	b	a	b	a	b	a	a	b
a	a	b	a	b	a	b	a	a	b
a	a	b	a	b	a	b	a	a	b
a	-	b	a	b	a	b	a	a	b
a	a	b	-	b	a	b	a	a	b
a	a	b	a	b	a	b	a	a	b
a	a	b	a	b	a	b	a	a	b
a	a	b	a	b	a	b	a	a	b
a	a	b	a	b	a	b	a	a	b
a	a	b	a	b	a	b	a	a	b
a	a	b	-	b	a	b	a	b	b
a	a	b	a	b	a	b	a	b	b
a	a	b	-	-	a	b	a	b	-
a	a	b	a	b	a	b	a	b	b
a	a	b	a	b	a	b	a	b	-
a	a	b	-	b	a	b	a	b	b
a	a	b	a	b	a	b	a	b	b
a	a	b	-	b	a	b	a	b	b
a	a	b	a	b	a	a	a	b	b
a	a	b	a	b	a	a	a	b	b
a	a	b	-	b	a	a	a	b	b
a	a	b	a	b	a	a	a	b	a
a	a	b	-	b	a	a	a	b	b
a	a	b	a	b	a	a	a	b	b
-	-	b	a	b	a	a	a	b	b
a	a	b	a	b	a	a	a	-	a
a	a	b	-	b	a	a	a	b	b
a	a	b	a	b	b	a	a	b	-
a	a	b	a	b	b	a	a	b	b
a	a	b	a	b	b	a	a	b	b

a	a	b	-	b	b	a	a	b	b
a	-	b	a	b	b	a	-	b	b
a	a	b	a	b	b	a	a	b	b
a	a	-	a	-	b	a	a	b	b
a	a	b	a	b	b	a	-	b	b
a	a	b	-	b	b	a	a	b	b
a	a	b	a	b	b	a	a	b	b
a	a	b	b	b	b	a	-	b	b
a	a	b	-	b	b	a	a	b	a
a	a	b	b	b	b	a	a	b	b
a	a	-	b	b	b	a	a	b	a
a	a	b	b	b	b	a	a	b	b
a	a	b	b	b	b	a	a	b	b
a	a	b	b	b	b	a	a	b	b
a	a	b	b	b	b	a	a	b	-
a	a	b	b	b	b	a	a	b	b
a	a	b	b	b	b	a	b	b	b
a	a	b	b	b	b	a	b	b	b
a	-	b	b	b	b	a	b	b	b
a	a	b	b	b	b	a	b	b	a
-	a	b	b	b	b	a	b	b	b
a	a	b	b	b	b	a	b	b	b
a	a	a	b	b	b	a	b	b	b
a	-	b	b	b	b	a	b	b	b
a	a	-	b	b	b	a	b	b	b
a	a	a	b	-	b	a	b	b	b
a	a	a	b	b	b	a	b	b	b
a	a	a	b	b	b	a	b	b	b
a	a	a	b	-	b	a	b	b	a
a	a	a	b	b	b	a	-	b	b
a	a	a	b	b	b	a	b	b	-
a	a	a	b	b	b	a	b	-	a
a	a	a	b	b	b	a	b	-	a
a	a	a	b	b	b	a	b	b	b
a	a	a	b	b	b	a	b	-	b
a	a	a	b	b	b	a	b	b	b
a	a	a	b	b	b	a	b	b	b
a	a	a	b	b	b	a	b	b	b
a	a	b	b	a	b	b	b	a	a
a	a	b	b	a	b	b	b	a	a
a	a	b	b	a	a	b	b	b	a
a	a	b	b	a	b	b	-	b	a
a	a	b	b	a	b	b	b	b	a
-	a	b	b	a	b	b	b	b	a
a	a	b	b	a	b	b	-	b	a
a	a	b	b	a	b	b	b	b	a
a	a	b	b	a	b	b	b	b	a

a	-	b	b	a	b	b	b	b	a
a	a	b	b	a	b	b	b	b	a
a	a	b	b	a	b	b	b	b	a
a	a	b	b	a	b	b	b	b	a
a	a	b	b	a	b	b	b	b	a
a	a	b	b	a	b	b	b	b	b
a	a	b	b	a	b	b	b	b	b
a	a	b	-	a	b	b	-	b	a
a	a	b	b	a	b	b	b	b	b
a	a	b	b	a	b	b	b	b	-
a	a	b	b	a	b	b	b	b	b
a	a	b	b	a	b	b	b	b	b
a	a	b	b	-	b	b	b	b	b
a	a	b	b	a	b	b	b	b	b
a	a	b	b	a	b	b	b	b	b
a	a	b	b	a	b	b	b	b	-
a	a	b	b	a	b	b	b	b	b
a	a	b	b	a	b	b	-	-	a
a	a	b	b	a	b	b	b	b	b
a	a	b	b	a	b	b	b	b	a
a	a	b	b	a	b	b	b	b	-
a	a	b	b	a	b	b	-	b	b
a	a	b	b	a	b	b	b	b	b
a	a	b	b	a	b	b	b	b	b
a	a	b	b	a	b	b	b	b	b
a	a	b	b	b	b	b	b	b	b
a	a	b	b	b	b	b	b	b	b
a	a	b	b	b	b	b	b	-	-
a	a	b	b	b	b	b	b	b	b
a	a	b	b	b	b	b	b	b	b
a	a	b	b	b	b	b	b	b	b
a	a	b	b	b	b	b	b	b	b
a	a	b	b	b	b	b	b	b	b
a	a	b	b	b	b	b	b	b	b
a	a	b	b	b	b	b	b	b	b
a	a	b	b	b	b	b	a	-	b
a	a	b	b	b	a	b	b	b	a
a	a	b	b	b	a	b	-	b	b
a	a	b	b	b	a	b	b	b	b
a	a	b	b	b	a	b	b	b	b
a	a	b	b	b	a	b	-	b	b
a	a	-	b	b	a	b	a	b	a
a	a	b	b	a	a	b	b	b	b
a	a	b	b	a	a	b	b	b	b
a	a	b	b	a	a	b	b	b	b
a	a	b	b	a	a	b	b	b	a
a	a	b	b	a	a	b	b	b	b

a	a	b	b	a	a	b	b	b	b
a	a	b	b	a	a	b	b	b	a
a	a	b	b	a	a	b	b	b	b
a	a	b	b	a	a	b	b	b	-
a	a	b	b	a	a	b	b	b	a
a	a	b	b	a	a	b	-	b	a
a	a	b	a	a	a	b	-	b	b
a	a	b	a	a	a	b	b	b	b
a	a	b	a	a	a	b	b	b	-
a	a	b	-	a	a	b	b	b	b
a	a	b	a	a	a	b	b	b	b
a	a	b	a	a	a	b	b	b	b
a	-	b	a	a	a	b	b	b	b
a	a	b	a	a	a	b	b	b	b
a	a	b	-	a	a	b	b	b	b
a	a	b	a	a	a	b	b	b	b
a	-	b	a	a	a	b	-	b	b
a	a	b	a	a	a	b	-	b	b
a	a	b	a	a	a	b	a	b	b
a	a	b	-	a	a	b	a	b	b
a	a	b	a	a	a	b	a	b	b
a	a	b	a	a	a	b	a	b	b
a	a	b	a	a	a	b	a	b	b
a	-	b	a	a	a	b	a	b	b
-	b	b	-	a	a	b	a	b	-
a	b	b	a	a	a	b	a	a	b
a	b	b	a	a	a	b	a	a	b
a	-	b	a	a	a	b	a	a	b
a	b	b	a	a	a	b	a	a	b
a	b	b	-	a	a	b	-	a	b
-	b	b	-	a	a	b	b	a	b
a	-	b	-	a	a	b	b	a	a
a	b	b	-	-	a	b	b	a	b
a	b	b	a	a	a	b	b	a	b
a	b	b	-	a	a	b	b	a	b
-	b	b	a	a	a	b	b	a	b
a	-	b	a	a	a	b	-	a	b
b	b	b	a	-	-	b	b	a	b
a	b	b	a	a	a	b	-	a	b
a	-	b	a	a	a	b	b	a	b
a	a	b	b	a	b	b	a	b	a
a	a	b	b	a	b	b	a	b	a
-	a	b	b	a	b	b	a	b	a
a	a	b	b	a	b	b	a	b	a
a	a	b	b	-	b	b	a	b	a
a	a	b	b	b	b	b	a	b	a
a	a	-	b	b	b	b	a	b	a

a	a	b	b	-	b	b	a	b	a
a	a	b	b	b	b	b	a	b	a
a	a	b	b	b	b	b	a	b	a
a	a	b	-	b	b	b	-	b	a
a	a	b	b	b	b	b	a	b	a
a	a	b	b	b	b	b	a	b	b
a	a	b	b	b	b	b	a	b	b
a	a	b	b	b	b	b	-	b	b
a	a	b	b	b	b	b	a	b	b
a	a	b	b	-	b	b	a	b	b
a	a	b	b	b	b	b	a	b	b
a	a	b	b	b	b	b	a	b	b
a	a	b	b	b	b	b	a	b	b
a	a	b	b	b	b	b	a	b	b
a	a	b	b	b	b	b	a	b	-
a	a	b	b	b	b	b	-	b	b
-	a	b	b	b	b	b	-	b	b
a	a	b	b	b	b	b	a	b	b
a	a	b	b	b	b	b	a	b	b
a	a	b	-	b	b	b	a	b	b
a	-	b	b	b	b	b	-	b	b
a	-	b	b	b	b	b	a	b	b
a	a	b	b	b	b	b	a	b	b
a	a	b	b	b	b	b	a	b	b
a	a	b	b	b	b	b	a	b	b
a	a	b	b	b	b	b	a	b	b
a	a	b	-	b	b	b	a	b	b
a	a	b	b	b	b	b	a	b	b
a	a	b	b	b	b	b	b	b	b
-	a	b	b	b	b	b	b	b	b
a	a	b	b	b	b	b	b	b	-
a	a	b	-	b	b	b	-	b	b
a	a	b	b	b	b	b	-	a	a
a	a	b	b	b	b	b	b	a	b
a	a	-	-	b	b	b	b	a	a
a	a	b	b	b	b	b	-	a	b
a	a	b	b	b	b	b	b	a	b
a	a	b	b	b	b	b	b	-	b
a	a	b	b	b	b	b	b	a	b
a	a	b	b	b	b	b	b	a	b
a	a	b	b	b	b	b	b	a	b
a	a	a	b	b	b	a	b	a	b
a	a	a	-	b	b	a	b	a	b
a	a	a	b	b	b	a	b	a	a
a	a	a	b	b	b	a	b	a	b
a	a	a	b	b	b	a	b	a	b
a	a	a	b	b	b	a	b	a	b

a	a	a	b	b	b	a	b	a	b
a	a	a	b	b	b	a	b	a	b
a	a	a	b	b	b	a	b	a	b
a	a	a	b	b	b	a	b	a	a
a	a	a	-	-	b	a	b	a	a
a	a	a	b	b	b	a	b	a	b
a	a	a	b	b	b	a	b	-	b
a	a	a	b	b	b	a	b	a	a
a	a	a	b	b	b	a	b	a	b
a	a	a	b	b	b	a	b	a	b
a	a	a	b	b	b	a	b	a	b
a	a	a	b	b	b	a	b	a	-
a	a	a	-	b	b	a	b	a	b
a	a	a	b	b	b	a	b	a	-
a	a	a	a	b	b	a	b	-	b
a	a	a	a	b	b	a	b	a	b
-	-	a	-	b	b	a	b	a	b
a	a	a	a	b	b	a	-	a	a
a	-	a	a	b	b	a	b	a	b
a	a	a	a	b	b	a	b	a	b
a	a	a	a	b	b	a	b	a	b
a	a	a	a	b	b	a	b	a	b
a	-	a	a	b	b	a	b	a	b
a	a	a	a	b	b	a	b	a	b
a	a	a	-	b	b	a	b	a	b
a	a	a	-	b	b	a	b	a	b
a	a	a	-	b	b	a	b	-	b
a	-	a	a	b	b	a	b	-	b
a	a	a	a	b	b	a	-	a	-
a	a	a	a	b	b	a	b	a	b
a	a	a	a	b	b	a	b	a	-
a	a	a	a	b	b	a	b	a	b
a	a	a	-	b	b	a	b	a	b
a	a	a	-	b	a	a	b	a	b
a	a	a	a	b	b	a	b	a	b
a	a	a	a	b	b	a	b	a	b
a	a	a	a	b	b	a	b	a	b
a	-	a	a	-	b	a	b	a	b
a	a	b	a	b	b	a	b	a	a
a	a	a	-	b	b	a	b	a	a
a	a	a	a	-	b	a	b	a	a
a	a	a	a	-	b	a	b	a	a
a	a	a	-	b	b	a	-	a	a
a	a	a	-	b	b	a	b	a	a
a	a	a	-	b	b	a	-	b	a
a	a	a	a	b	b	a	b	b	a
-	a	a	-	b	b	a	b	b	a

a	-	a	a	b	b	a	b	b	a
a	a	a	a	b	b	a	b	b	a
a	a	a	a	b	b	a	b	b	a
a	a	a	a	b	b	a	b	b	a
-	a	a	b	b	b	a	b	b	a
-	a	a	-	b	b	a	b	b	a
a	a	a	a	b	b	a	b	b	a
a	a	a	-	b	b	a	b	b	-
a	a	a	a	b	b	a	b	b	a
a	a	a	a	b	b	a	b	b	a
a	a	a	a	b	b	a	b	b	a
a	a	a	a	b	b	a	b	b	a
a	a	a	-	b	b	a	b	b	a
a	a	a	a	b	b	a	b	-	a
a	a	b	a	-	b	a	b	b	a
a	a	b	a	a	b	a	b	b	a
a	-	b	a	a	b	a	b	b	a
a	b	b	a	a	b	a	b	b	b
a	a	b	b	b	b	a	a	a	a
a	a	a	b	a	b	b	a	a	a
-	-	a	b	a	b	b	a	a	a
a	a	a	b	a	b	b	a	a	a
a	a	a	b	a	b	b	a	a	a
a	-	a	b	a	b	b	a	a	a
a	a	a	b	a	b	b	a	a	a
a	a	a	b	a	b	b	b	a	a
a	a	a	b	a	b	b	b	a	a
a	a	a	b	a	b	b	b	a	a
b	a	a	b	a	b	b	b	a	a
b	a	a	b	a	b	b	b	a	a
-	a	a	b	a	b	a	b	a	a
b	a	a	b	a	b	a	b	a	a
b	a	a	b	a	b	a	b	a	a
b	a	a	-	a	b	a	-	a	a
b	a	a	b	a	b	a	b	a	a
b	a	a	b	a	b	a	b	a	a
b	a	a	b	a	b	a	b	a	a
-	a	a	b	a	b	a	b	a	-
b	a	a	b	a	a	a	b	a	a
b	-	a	b	a	a	a	b	a	a
-	a	a	b	a	a	a	b	a	a
b	a	a	b	a	a	a	b	a	-
b	a	a	-	a	a	a	b	a	a
b	a	a	b	a	a	a	b	a	a
-	a	a	b	a	a	a	b	a	a
b	a	-	b	a	a	a	b	a	a
b	a	a	b	a	-	a	b	a	a

-	a	a	-	a	a	a	b	a	a
b	a	a	a	a	a	a	-	a	a
a	a	a	b	a	a	a	a	a	a
a	a	a	b	a	a	a	a	a	a
a	a	a	b	a	a	a	a	a	a
a	a	a	b	a	a	a	a	a	a
a	a	a	a	a	a	a	a	a	a
a	a	a	b	a	a	a	a	a	a
-	a	a	b	a	a	a	a	a	a
a	-	b	b	a	a	a	a	-	b
a	-	a	b	a	a	a	a	a	-
a	a	a	b	a	a	a	a	a	a
a	a	a	b	a	a	a	a	a	a
a	a	a	b	a	a	a	a	a	a
a	a	a	b	a	a	a	a	a	a
a	a	a	-	a	a	a	-	a	a
a	a	a	a	a	a	a	a	a	b
a	a	a	a	a	a	a	a	a	a
a	a	a	a	a	a	a	a	a	a
a	a	a	a	a	a	a	a	a	a
a	a	a	a	a	a	a	a	a	a
a	a	a	a	a	a	a	a	a	a
a	a	a	a	a	a	a	a	a	a
a	a	a	-	a	a	a	a	b	a
a	a	a	a	a	a	a	a	b	a
a	a	a	a	a	a	a	a	b	a
a	a	a	a	a	a	a	a	b	a
a	a	a	a	a	a	a	a	b	a
a	a	a	a	a	a	a	a	b	a
a	a	a	a	a	a	a	a	b	a
a	a	a	a	a	a	a	a	b	a
a	a	a	-	a	a	a	a	b	a
a	a	a	-	a	a	a	a	b	-
-	-	a	b	a	a	a	a	b	-
a	a	a	b	a	a	a	a	b	a
a	a	a	b	a	a	a	a	b	a
a	a	a	-	a	a	a	a	b	a
a	a	a	b	a	a	a	a	b	a
-	a	a	b	a	a	a	a	b	a
a	a	a	b	a	a	a	a	b	a
a	a	a	b	a	a	a	a	b	a
a	a	a	b	a	a	a	a	b	a
a	a	a	b	a	a	a	a	b	a
a	a	a	b	a	a	a	a	b	a
a	a	a	b	a	a	a	a	b	a
a	a	a	b	a	a	a	a	b	a
a	b	a	b	a	a	a	a	b	-

-	-	a	b	a	a	a	a	b	a
a	b	a	b	a	a	a	a	b	a
a	b	a	-	a	a	a	a	b	-
a	b	a	-	a	a	a	a	b	a
a	b	a	b	a	a	a	a	b	a
a	-	a	b	a	a	a	a	b	a
-	a	a	b	a	a	a	a	b	a
a	b	a	b	a	a	a	a	b	a
a	b	a	b	a	a	a	a	b	a
a	b	a	b	a	a	a	a	b	a
a	a	a	b	a	-	a	a	b	a
-	-	a	-	b	b	b	b	b	b
a	b	a	a	b	b	b	b	b	b
a	b	a	a	b	b	b	b	b	b
a	b	a	a	b	b	b	-	b	a
a	b	a	a	b	b	b	b	b	b
-	b	a	a	b	b	b	b	b	-
a	b	a	a	b	b	b	b	b	-
a	b	a	a	b	b	b	-	b	a
a	b	a	a	b	b	b	-	b	b
a	b	a	b	b	b	b	b	b	b
a	b	a	-	b	b	b	b	b	b
a	b	a	b	b	b	b	b	b	b
-	-	a	b	b	b	b	b	b	b
a	b	a	-	b	b	b	b	b	b
a	b	a	b	b	b	b	b	b	b
a	b	a	b	b	b	b	b	b	b
a	b	a	b	-	b	b	b	b	-
-	b	a	b	b	b	b	b	b	b
a	b	a	b	a	b	b	b	b	b
a	b	a	-	a	b	a	b	b	b
a	b	a	b	a	b	a	b	b	b
b	b	a	-	a	b	a	b	b	b
b	b	a	-	a	b	a	b	b	b
b	b	a	-	a	b	a	b	b	b
b	a	a	b	a	b	a	b	b	b
b	a	a	b	a	b	a	b	-	b
b	b	a	b	a	b	a	b	b	-
b	b	a	b	a	b	a	b	b	b
b	b	a	b	a	b	a	b	b	-
b	b	a	b	a	b	a	b	b	b
b	b	a	b	a	b	a	b	b	b
b	b	a	-	a	b	a	b	b	b
b	b	a	-	a	b	b	b	b	b
b	b	a	b	b	b	b	b	b	b
b	b	a	-	a	b	b	b	b	b
b	b	a	b	a	b	b	b	b	b

b	b	a	b	a	b	b	b	b	b
b	b	a	b	a	b	b	b	b	b
b	b	a	b	a	b	b	b	b	-
b	b	a	b	a	b	b	-	b	-
b	b	a	b	a	b	b	b	b	b
-	b	a	b	a	a	b	b	b	b
b	b	a	b	a	a	b	-	b	-
b	-	a	b	a	a	b	b	b	b
-	b	a	b	a	a	b	b	b	b
b	-	a	b	a	a	b	b	-	b
b	b	a	-	a	a	b	b	b	b
b	b	a	b	a	a	b	b	b	b
b	b	a	-	a	a	b	b	b	b
b	b	a	-	a	a	b	b	b	a
b	b	a	b	a	a	b	b	b	b
b	b	a	b	a	b	b	b	b	b
b	b	a	b	a	b	b	b	b	b
b	b	a	b	a	b	b	b	b	b
b	b	a	b	a	b	b	b	b	-
b	b	a	b	a	b	b	b	b	b
b	b	-	b	a	b	b	b	b	b
b	b	a	b	a	b	b	b	b	b
b	b	a	b	a	b	b	b	b	b
b	b	a	b	a	b	b	b	b	b
b	b	a	-	a	b	b	b	b	b
b	b	a	-	a	b	b	b	b	b
b	b	a	b	a	b	b	b	b	b
b	b	a	b	a	b	b	b	b	b
b	b	a	b	a	b	b	b	b	b
b	b	a	b	a	b	b	b	-	b
b	b	a	b	a	b	b	b	b	b
b	b	a	-	a	b	b	b	b	b
b	b	a	b	a	b	b	b	b	-
b	b	a	-	a	b	b	b	b	a
b	b	a	b	a	b	b	b	-	a
b	b	a	-	a	b	b	b	b	b
b	b	a	b	a	a	b	b	b	b
b	b	a	b	a	a	b	b	b	b
b	b	a	b	a	a	b	b	a	-
b	b	a	b	a	a	b	b	a	b
b	b	a	b	a	a	b	b	a	b
b	b	a	b	a	a	b	b	a	b
b	b	a	b	a	b	b	b	a	b
b	b	a	-	b	b	b	b	a	b
b	b	a	b	b	b	b	b	a	a
b	b	a	b	b	b	b	b	a	b
b	b	a	b	b	b	b	b	a	b

b	b	a	b	b	b	b	b	a	b
b	b	a	b	b	b	b	b	a	b
b	b	a	-	b	b	b	b	a	b
b	b	a	-	b	b	b	-	a	b
b	b	a	b	b	b	b	b	a	b
b	b	-	b	b	b	b	b	a	b
b	b	a	b	b	b	b	b	a	b
b	b	a	b	b	b	b	b	a	b
b	b	a	b	b	b	b	b	a	b
-	b	a	b	-	b	b	b	a	a
b	a	a	b	b	b	b	b	a	b
b	a	a	b	b	b	b	b	a	-
b	a	a	b	b	b	b	b	-	b
b	a	a	b	b	b	b	-	a	b
b	a	a	-	b	b	b	b	a	b
b	a	a	b	b	b	b	b	a	b
-	a	a	b	b	b	b	-	a	a
b	a	a	b	b	b	b	b	a	b
b	-	a	b	b	b	b	b	a	b
b	a	a	b	b	b	b	b	b	b
b	a	a	b	b	b	b	b	a	b
b	a	a	b	b	b	b	b	a	b
a	a	a	b	b	a	a	b	a	a
a	a	a	b	b	a	a	b	a	b
a	-	a	a	b	a	a	b	a	-
a	-	a	-	b	a	a	b	a	b
a	a	a	-	b	a	a	b	b	b
a	-	a	b	b	a	a	b	b	b
a	a	a	b	-	a	a	b	-	-
a	a	a	b	b	a	a	b	b	b
-	a	a	b	b	a	a	b	b	b
a	a	a	b	b	b	a	b	b	b
a	a	a	b	b	b	a	b	-	b
a	a	a	b	b	b	a	-	b	b
-	a	a	b	b	b	a	b	b	b
a	a	b	b	b	b	a	a	b	b
a	a	a	b	b	b	b	a	b	b
a	a	a	b	b	b	b	a	b	b
-	-	a	b	b	b	b	a	b	b
a	a	a	b	b	b	b	a	b	b
a	-	a	-	b	b	b	a	b	b
a	a	a	b	b	b	b	a	b	b
a	a	a	-	b	b	b	a	b	b
a	a	a	b	b	b	b	a	b	a
a	-	a	b	b	b	b	a	b	b
a	-	a	-	b	b	b	-	b	b
a	a	b	-	b	b	b	a	b	b

b	a	b	b	b	b	b	-	b	b
b	a	b	-	b	b	b	-	b	b
b	a	b	b	b	b	b	a	b	b
-	a	-	b	b	b	b	a	b	a
-	a	b	b	b	b	b	a	b	a
b	a	b	b	b	b	b	a	b	a
b	a	b	b	b	b	b	a	b	a
b	a	-	b	b	b	b	a	b	a
-	a	b	b	b	b	b	a	b	a
b	a	b	b	b	b	b	a	b	a
-	a	b	b	b	b	b	-	b	b
b	a	b	b	b	b	b	a	b	a
b	a	b	-	a	b	b	a	b	a
b	a	b	-	a	b	b	a	b	a
b	a	b	b	a	b	b	a	b	a
b	a	b	b	a	b	b	a	b	a
b	a	b	b	a	b	b	a	b	a
b	a	b	b	a	b	b	a	b	a
b	a	b	b	a	b	b	a	b	-
b	a	-	b	a	b	b	-	b	a
b	a	b	b	b	b	b	b	b	a
a	a	b	b	b	b	b	b	b	a
b	a	b	b	b	b	b	b	b	a
b	a	b	b	b	b	b	b	b	b
b	a	b	b	b	b	b	b	b	a
b	a	b	b	b	b	b	-	b	a
b	-	b	b	b	b	b	b	b	b
b	a	b	b	b	b	b	b	b	a
b	a	b	b	b	b	b	b	b	a
b	a	b	b	b	b	b	b	b	a
b	a	b	b	b	b	b	b	b	a
b	a	b	b	b	b	b	b	b	a
b	a	b	b	b	b	b	b	b	a
b	a	b	b	b	b	b	b	b	a
b	a	b	b	b	b	b	b	b	-
b	a	b	b	b	b	b	b	b	-
-	a	b	b	b	b	b	b	b	a
-	a	b	b	b	b	b	b	b	a
b	a	b	b	b	b	a	b	b	a
-	a	b	b	a	b	a	b	b	a
-	a	b	-	a	b	a	b	b	a
b	a	b	b	a	b	a	-	b	a
b	a	b	b	a	b	a	b	b	a
b	b	b	-	a	b	a	-	-	a
b	b	b	b	a	b	a	b	b	a
b	b	b	b	a	b	a	b	b	a
b	b	b	b	a	b	a	b	b	a

b	b	b	b	a	b	a	b	b	a
b	b	b	b	b	b	a	b	b	b
b	b	b	b	b	b	a	b	-	a
b	-	b	b	b	b	a	b	b	a
b	b	b	b	b	b	a	b	b	a
b	b	b	b	b	b	a	b	b	-
b	b	b	b	b	b	a	b	b	a
b	b	b	b	b	b	a	b	b	-
b	b	b	b	b	b	a	b	b	a
b	b	b	b	b	b	a	b	b	a
b	b	b	b	b	b	a	b	b	a
b	b	b	b	b	b	a	b	b	a
b	b	b	b	b	b	a	b	b	a
b	b	b	b	b	b	a	b	b	a
b	b	b	b	b	b	a	a	b	a
b	b	b	b	b	b	a	a	b	a
b	b	b	b	b	b	a	a	b	a
b	b	b	b	b	b	a	a	b	b
b	b	b	b	b	b	a	a	b	a
b	b	b	b	-	b	a	a	b	a
b	b	b	b	b	b	a	a	b	a
b	b	b	b	b	b	a	a	b	a
b	b	b	b	b	b	a	a	b	a
b	b	b	b	b	b	a	a	b	a
b	b	b	b	b	b	a	a	b	-
b	b	b	b	b	b	a	a	b	a
b	b	b	b	b	b	a	a	b	a
b	-	b	-	b	b	a	a	b	a
-	b	b	b	b	b	a	a	a	a
b	b	b	b	b	b	a	a	a	a
-	b	b	-	b	b	a	a	a	a
b	b	b	b	b	b	a	a	a	-
b	b	b	b	b	b	a	a	a	a
b	b	b	b	b	b	a	a	a	a
-	b	b	b	b	-	a	a	a	-
b	b	b	b	b	b	a	a	a	a
b	b	b	b	b	b	a	a	a	a
b	b	b	b	-	b	a	a	a	a
b	b	b	b	b	b	a	a	a	a
b	b	b	-	b	b	a	a	a	a
b	b	b	b	b	b	a	a	a	-
-	b	b	-	b	b	a	a	a	a
b	-	b	b	b	b	a	a	a	a
b	b	b	b	b	b	a	a	a	a
b	b	b	b	b	b	a	a	a	a
b	b	b	-	b	b	a	a	a	a
b	-	b	b	b	b	a	a	a	a

b	b	b	b	b	b	a	a	a	a
b	b	b	b	b	b	a	a	a	a
b	b	b	b	b	b	a	a	a	a
a	a	b	a	b	b	a	b	a	a
a	a	b	a	b	b	a	b	a	-
a	a	b	-	b	b	a	b	a	a
a	a	b	a	b	b	a	b	a	a
a	b	b	b	b	b	a	-	a	a
a	-	b	b	b	b	b	b	a	a
a	b	b	-	b	b	b	b	a	a
a	b	b	b	b	b	b	b	a	a
a	b	b	b	b	-	b	b	a	a
-	b	b	b	b	b	b	b	a	a
a	b	b	b	-	b	b	-	a	a
a	b	b	b	b	b	b	b	a	-
b	b	b	b	b	b	b	b	a	a
b	b	b	b	b	b	b	b	a	a
b	b	b	b	b	b	b	b	a	a
b	b	b	b	b	b	b	b	a	a
b	b	b	b	b	b	b	b	a	a
b	b	b	b	b	b	b	b	a	a
b	b	b	b	b	b	b	-	a	a
b	b	b	b	b	b	b	b	a	a
b	b	b	b	b	b	b	b	a	a
b	b	b	b	b	b	b	b	a	a
b	b	b	b	b	b	b	b	a	a
b	b	b	b	b	b	b	b	a	a
b	-	b	b	b	b	b	b	a	a
a	b	b	b	a	b	b	b	a	a
a	b	b	b	a	b	b	b	a	a
a	b	b	b	a	b	b	b	a	a
a	b	b	b	a	b	b	b	a	a
a	b	b	b	a	b	b	b	a	a
a	b	b	b	a	b	b	b	a	a
a	b	b	b	a	b	b	-	a	a
a	b	b	b	a	a	b	b	a	-
a	-	b	b	a	a	b	b	a	a
a	b	b	b	a	a	b	b	a	a
-	b	b	b	a	a	a	b	a	b
a	b	b	b	a	-	a	b	a	b
a	b	b	b	a	a	a	b	a	b
a	-	b	b	a	a	a	b	a	a
a	b	b	b	a	a	a	b	a	b
a	b	b	b	a	a	a	b	a	b
a	b	b	b	a	a	a	-	a	b
b	b	b	-	a	a	a	b	a	b
b	b	b	b	a	a	a	b	a	b

b	b	b	b	a	a	a	b	a	b
b	b	b	b	a	a	a	b	a	b
b	b	b	b	a	a	a	b	a	b
b	b	b	b	a	a	a	b	a	b
a	-	b	a	a	a	a	b	a	-
a	b	-	a	a	b	a	b	a	-
a	b	b	a	a	b	a	b	a	b
a	-	b	a	a	b	a	b	a	b
a	a	-	-	a	b	a	b	a	b
a	b	b	a	a	b	a	b	a	b
a	b	b	-	a	b	a	b	a	b
a	a	b	-	a	a	a	b	a	a
a	a	b	a	a	b	a	b	a	a
b	a	b	b	a	b	a	b	a	a
b	a	b	b	a	b	a	b	a	a
-	a	b	b	a	b	a	b	a	a
b	a	b	b	a	b	a	b	a	a
b	a	b	b	a	b	a	b	a	a
b	a	b	b	a	b	a	b	-	a
-	a	b	b	a	b	a	-	a	a
b	a	b	b	a	b	a	b	a	a
b	a	b	b	a	b	a	-	a	a
b	a	b	b	-	b	a	b	a	a
b	a	b	b	a	b	a	b	a	a
b	a	b	b	a	b	a	b	a	a
b	a	b	b	b	b	a	b	a	a
b	a	b	b	b	b	a	b	a	a
b	a	b	b	b	a	b	b	a	b
a	a	b	b	b	a	b	b	a	a
b	a	b	b	b	a	b	b	a	a
b	a	b	b	b	a	b	b	a	a
b	a	b	b	b	a	b	b	a	a
b	a	b	b	b	a	b	b	a	a
b	a	b	b	b	-	b	b	a	a
-	-	b	b	b	b	b	b	a	a
b	a	b	b	b	b	b	b	a	a
-	a	b	b	b	b	b	b	a	a
b	a	-	-	b	b	b	b	a	a
b	b	b	b	b	b	b	b	a	a
-	a	b	b	b	b	b	b	a	a
b	a	b	a	b	b	b	b	a	a
b	b	a	a	a	b	b	b	a	a
b	b	a	a	a	b	b	b	a	a
b	b	a	-	a	b	b	b	a	a
b	b	b	-	a	b	b	b	a	a
b	b	b	b	b	b	a	a	a	b
b	a	b	b	b	b	a	a	a	b

b	-	b	b	b	b	a	a	a	b
b	a	b	b	b	b	a	a	a	a
b	a	b	b	b	b	a	a	a	b
b	b	b	b	b	a	a	a	a	b
-	a	b	b	b	b	a	-	a	b
-	a	b	b	b	b	a	a	a	b
b	a	b	b	b	b	a	-	-	b
b	a	b	b	b	b	a	a	a	a
b	a	b	b	b	b	a	a	a	b
-	-	b	b	b	b	a	-	a	b
b	a	b	b	b	b	a	a	a	b
-	a	b	b	-	b	a	a	a	b
b	a	b	b	b	-	a	a	a	b
b	a	b	b	b	b	a	a	a	b
b	a	b	b	-	b	a	-	a	b
b	a	b	b	b	b	a	b	a	b
b	a	b	b	b	b	a	b	-	b
b	a	b	b	b	b	a	b	a	b
-	a	b	b	b	b	a	b	a	b
b	a	b	b	b	b	a	b	a	a
-	a	b	b	b	b	a	b	a	b
b	a	b	b	b	b	a	b	a	b
-	a	b	b	b	b	a	b	a	-
b	a	b	b	b	b	a	b	a	b
-	a	b	b	a	b	a	b	a	b
b	a	b	b	a	b	a	b	a	a
b	a	a	b	a	b	a	b	a	b
b	a	a	b	a	b	a	b	a	b
b	a	a	-	a	b	a	b	a	b
b	a	a	b	a	b	a	b	a	b
b	a	a	b	a	b	a	-	a	b
b	a	a	b	a	b	a	b	a	b
b	-	a	b	a	b	a	b	a	b
b	a	a	b	a	-	a	b	b	a
-	a	a	b	a	b	a	b	b	b
b	a	a	-	a	b	a	a	b	b
b	a	a	b	a	b	a	b	b	b
b	-	a	b	a	b	a	b	b	b
b	-	a	b	a	b	a	b	b	b
b	a	a	b	a	b	a	b	b	b
b	-	a	b	a	b	a	b	b	b
b	a	a	b	a	b	a	b	b	b
-	a	a	b	a	b	a	b	b	b
b	a	a	b	a	b	a	b	b	b
b	a	a	b	a	b	a	b	b	b
-	a	a	b	a	b	a	b	b	b
b	a	a	b	a	b	a	b	b	-

b	a	a	-	a	b	a	b	b	b
b	a	a	b	a	b	a	b	b	b
b	a	a	b	a	b	a	b	b	b
b	a	a	b	a	b	a	-	b	b
b	a	a	b	a	-	a	b	b	a
b	a	a	b	a	b	a	b	b	b
b	-	a	b	a	b	-	b	b	-
b	a	a	b	a	b	a	-	b	a
-	b	a	-	a	b	a	b	b	a
b	b	a	-	a	b	a	b	b	a
b	b	a	b	a	b	a	b	-	a
b	b	a	b	a	b	a	b	b	a
b	b	a	b	a	b	a	b	b	a
b	b	a	-	a	b	a	b	b	a
b	-	a	b	a	b	a	b	b	a
b	b	a	-	a	b	a	b	b	a
b	b	a	b	a	b	a	b	b	a
b	b	a	-	a	b	a	b	b	a
b	b	a	b	a	b	a	b	b	-
b	b	a	b	a	b	a	b	b	a
b	a	a	-	a	b	a	b	a	a
b	a	a	b	a	b	a	-	-	-
-	a	a	b	a	b	a	b	a	a
b	a	a	b	a	b	a	b	a	a
b	-	a	b	b	b	a	-	a	a
b	a	a	b	b	b	a	-	a	-
b	a	a	b	b	b	a	a	a	-
b	a	a	b	b	b	a	a	a	a
b	a	a	b	b	b	a	a	a	a
b	a	a	b	b	b	a	-	a	a
b	a	a	-	b	b	a	a	a	-
b	a	a	b	b	b	a	a	a	a
b	a	a	b	b	b	a	a	a	a
b	a	a	-	b	b	a	-	a	a
-	a	a	b	b	b	a	a	a	-
b	a	a	b	b	b	a	a	a	a
b	a	a	b	b	b	a	-	a	a
b	a	a	b	b	b	a	a	a	a
b	a	a	-	b	b	a	a	a	a
b	a	a	-	b	b	a	a	a	a
b	a	a	b	b	b	a	a	a	a
-	a	a	b	b	b	a	a	a	a
b	a	a	-	b	b	a	a	a	a
b	a	a	b	b	b	a	a	a	a
b	a	b	b	b	b	a	a	-	-
-	a	b	b	b	b	a	-	a	a
-	a	b	b	b	b	a	a	-	b

b	a	b	b	b	b	a	-	a	b
b	a	b	b	b	b	a	a	a	b
b	a	b	b	b	b	a	a	b	b
b	a	b	b	b	b	a	a	b	b
b	a	b	b	b	b	a	a	b	b
b	a	b	b	b	b	a	a	b	b
b	-	b	b	b	b	a	-	b	b
b	a	b	b	b	b	a	a	b	b
b	b	b	b	a	a	b	-	b	b
b	a	b	b	a	a	b	a	b	a
b	a	b	b	a	a	b	a	b	a
b	a	b	b	a	a	b	a	b	a
b	a	b	b	a	a	b	a	b	a
b	a	b	b	a	a	b	a	b	a
b	b	b	b	a	a	b	a	-	a
b	b	b	b	a	a	b	a	b	a
b	b	b	b	a	a	b	a	b	b
b	b	b	b	a	a	b	a	b	a
b	b	b	-	a	a	b	a	b	a
b	b	a	b	a	a	b	a	b	a
b	b	a	b	a	a	b	a	b	-
b	b	a	-	a	a	b	a	b	a
b	b	a	b	a	a	b	a	-	a
b	b	a	b	a	a	b	b	b	b
b	-	a	b	b	a	a	b	b	a
b	b	a	b	b	b	a	b	b	a
b	b	a	b	b	a	a	-	b	a
b	b	a	b	b	b	a	b	b	a
b	b	a	b	a	b	a	b	b	a
b	b	a	b	b	b	a	b	b	-
b	b	a	b	b	b	a	b	b	b
b	b	a	-	b	b	a	b	b	b
b	b	a	b	b	b	a	-	b	b
b	b	a	b	b	b	a	b	b	b
-	b	a	b	b	b	a	-	b	a
b	b	a	b	b	b	a	b	b	a
-	b	a	-	b	b	a	-	b	a
b	b	a	b	b	b	a	b	b	a
b	b	a	b	-	b	b	b	-	a
b	b	a	-	b	b	b	b	b	a
b	-	a	a	b	b	b	b	b	a
-	b	a	a	-	b	b	b	b	a
b	b	a	a	b	b	b	-	b	a
a	b	a	a	b	-	b	-	b	a
a	b	a	a	b	b	b	b	b	b
a	b	a	a	b	b	b	b	b	a
a	b	-	a	b	b	b	b	b	b

a	b	a	-	b	-	b	b	-	a
a	a	a	a	b	b	b	b	-	a
a	a	a	a	b	b	b	b	b	b
a	a	a	-	b	b	b	b	b	-
a	a	a	a	b	b	b	b	b	b
a	a	a	a	b	b	b	b	b	a
a	a	a	a	b	b	b	b	b	b
a	a	a	a	b	b	b	b	b	b
a	a	a	a	b	b	b	b	b	b
a	a	a	-	b	b	b	b	b	b
a	a	a	a	b	b	b	b	b	b
a	a	a	-	b	b	b	b	b	b
a	b	b	b	a	b	a	a	b	b
a	b	b	-	a	b	a	a	b	b
a	b	b	b	a	b	a	a	b	b
a	b	b	-	a	b	a	a	b	-
a	b	b	a	a	a	b	b	b	a
a	b	b	b	b	b	a	a	b	-
a	b	b	b	b	b	a	a	b	a
-	b	b	b	b	b	a	a	b	b
a	b	b	b	b	b	a	a	b	b
a	b	b	b	b	b	-	-	b	b
-	b	b	b	b	b	a	-	b	b
b	b	a	b	b	b	b	a	b	b
b	b	a	b	b	a	b	a	b	b
b	b	a	b	b	a	b	a	b	b
b	b	a	-	b	a	b	a	b	b
b	b	a	b	b	a	b	a	b	b
b	b	a	b	b	a	b	a	b	b
b	b	b	b	b	a	b	a	b	b
b	a	b	b	b	a	b	a	-	-
b	b	b	b	b	a	b	a	b	b
b	b	b	b	b	a	b	a	b	b
b	b	b	b	b	a	b	a	b	b
b	b	b	b	b	a	b	a	-	a
b	b	b	b	b	a	b	a	b	b
b	b	b	b	b	-	b	a	b	b
b	b	b	b	b	a	b	a	b	-
b	b	b	b	b	a	b	a	b	b
b	-	b	b	b	a	b	a	b	b
b	b	b	b	b	a	-	a	b	-
b	b	b	b	b	b	b	-	-	b
b	b	b	b	b	a	b	a	b	b
b	b	b	b	b	a	b	a	b	b
b	b	b	b	b	a	b	a	b	b
b	b	a	b	a	b	b	-	a	b
b	b	a	b	a	b	b	b	a	b

b	b	a	b	a	b	b	b	a	b
b	b	a	b	a	b	b	b	a	a
b	b	a	b	a	b	b	b	a	a
b	b	a	a	a	b	b	b	a	a
-	-	b	a	a	b	b	b	a	a
b	b	b	a	a	b	b	b	a	b
b	b	b	a	a	b	b	b	a	a
b	b	b	a	a	b	b	b	a	a
b	a	a	-	a	b	-	-	b	b
b	a	a	-	a	b	b	b	b	b
-	a	a	a	a	b	b	b	b	b
b	a	a	a	a	b	b	b	b	b
b	b	a	a	a	b	b	b	b	b
b	b	a	a	a	b	b	b	b	b
b	b	a	a	a	b	b	b	b	b
b	b	a	a	a	b	b	b	b	b
b	b	a	a	a	b	b	b	b	-
b	-	a	-	a	b	b	-	b	b
b	b	a	a	a	b	b	-	b	-
b	-	a	a	a	b	b	-	b	b
b	b	a	a	a	b	b	b	b	b
b	b	a	a	a	b	b	-	b	b
b	b	a	-	a	b	b	-	b	b
b	b	a	a	a	b	b	b	b	b
b	a	a	a	a	b	-	b	b	a
b	b	a	a	a	b	b	b	b	b
b	b	a	a	a	b	b	b	b	b
b	-	a	-	a	-	b	-	b	b
b	-	a	a	a	b	b	b	b	-
b	b	a	b	a	b	b	b	a	b
b	b	a	b	-	b	b	-	b	-
b	b	a	b	a	b	b	b	a	a
b	b	a	b	a	b	b	-	a	a
b	b	a	b	a	-	b	b	a	a
b	b	a	b	a	b	b	b	a	a
b	b	a	-	a	b	b	b	a	a
b	b	a	b	a	b	b	b	a	a
b	b	a	b	a	b	b	b	a	a
-	b	a	b	a	b	b	-	a	a
b	b	a	b	a	b	b	b	a	a
b	b	a	b	a	b	b	b	a	a
b	-	a	b	a	b	b	b	a	a
b	b	a	b	a	b	b	b	a	a
b	b	a	b	a	b	b	b	a	a
b	b	a	b	a	b	b	b	a	a
-	b	a	-	a	b	b	b	a	a
b	b	a	a	b	a	b	b	a	a

-	b	a	a	-	a	b	b	a	a
b	b	a	a	b	a	b	b	a	a
b	b	a	a	b	a	b	b	a	a
b	b	a	a	b	a	b	b	a	a
b	b	a	a	b	a	b	b	a	a
b	b	a	a	b	a	b	b	a	a
b	-	-	a	-	b	b	b	a	a
b	b	b	a	a	b	b	b	a	a
b	b	b	a	a	b	b	b	a	a
b	b	b	a	a	b	b	b	a	a
b	b	b	-	a	b	b	b	a	a
b	b	b	-	a	b	b	b	a	a
b	b	b	a	a	b	b	b	a	a
b	b	b	a	a	b	b	b	a	a
-	b	b	a	a	b	a	b	a	a
b	b	b	a	a	b	a	b	a	a
b	b	b	a	a	b	a	b	a	a
a	a	b	a	a	b	a	b	-	-
a	b	b	a	a	a	b	b	a	a
a	b	b	a	a	a	b	b	a	b
b	b	b	a	a	a	b	b	b	b
b	b	b	-	a	a	b	b	b	b
b	b	b	a	a	a	b	b	b	b
b	b	b	a	a	a	b	b	b	b
b	b	b	a	a	a	b	b	b	b
b	b	b	a	a	a	b	b	b	b
b	b	b	a	a	a	b	b	b	b
b	b	b	a	a	a	b	b	b	b
b	b	b	a	a	a	b	b	b	b
b	b	b	a	a	a	b	b	b	b
b	b	b	a	a	a	b	b	b	b
b	b	b	a	a	a	b	b	b	b
b	b	b	a	b	a	b	b	b	b
b	b	b	a	b	a	b	b	b	b
b	b	b	a	b	a	b	b	b	b
b	b	b	a	b	a	b	-	b	b
b	b	b	a	b	a	b	b	b	a
b	b	b	a	b	a	b	b	b	a
b	b	b	a	b	a	b	b	b	a
b	b	b	-	b	a	b	b	b	a
b	b	b	a	b	a	b	b	b	a
b	b	b	a	b	a	b	-	b	a
b	b	b	a	b	a	b	b	b	a
b	b	b	a	b	a	a	b	-	a
b	b	b	a	b	a	a	b	b	a
-	b	b	a	b	a	a	b	b	a
b	b	b	a	b	a	a	b	b	a

b	b	b	a	b	a	a	b	b	b
b	b	-	a	b	a	a	b	b	a
b	-	b	a	b	a	a	b	b	a
b	-	b	a	b	a	a	b	b	a
b	-	b	a	b	a	a	b	b	a
b	b	a	a	b	a	a	b	b	a
b	b	a	a	b	a	a	b	b	-
b	b	a	a	b	a	a	b	b	a
b	b	a	a	b	a	a	b	b	a
b	b	a	a	b	a	a	b	a	a
b	b	a	a	b	a	a	b	a	a
b	b	a	a	b	a	a	b	a	a
b	b	a	a	b	a	a	-	a	a
b	b	a	a	b	a	a	b	a	b
b	b	a	-	b	a	a	b	a	-
b	b	a	a	b	a	a	b	a	a
b	b	a	a	b	a	a	b	a	b
b	b	a	a	b	a	a	b	a	a
b	b	a	a	b	a	a	b	a	a
b	b	a	a	b	a	a	b	a	b
b	b	a	a	b	a	a	b	a	a
b	b	a	a	b	a	a	b	a	a
b	b	a	a	b	a	a	b	a	a
b	b	a	a	b	a	a	b	a	a
b	b	a	a	b	a	a	b	a	a
b	b	a	a	b	a	a	b	a	a
b	b	a	a	b	a	a	b	a	a
b	b	a	-	b	a	a	b	-	b
b	b	a	a	a	a	a	a	a	b
b	b	a	a	a	a	a	a	a	b
b	b	a	a	a	a	a	a	-	b
b	b	a	a	a	a	a	a	a	b
b	b	a	a	a	a	a	a	a	b
b	b	a	a	a	a	a	a	a	b
b	b	a	a	a	a	a	a	a	a
b	b	a	a	a	a	b	b	b	a
b	b	a	a	a	a	b	b	b	-
b	b	a	a	a	a	b	b	b	a
b	b	a	a	a	a	b	b	b	a
b	b	a	a	a	a	b	b	b	a
b	b	a	a	a	a	b	b	b	a
-	b	a	a	a	a	b	b	b	a
b	b	a	a	a	a	b	b	b	a
b	b	a	a	a	-	b	b	b	a
b	b	a	a	a	a	b	b	b	a
b	b	a	a	a	a	b	-	b	a
-	b	a	b	a	b	b	b	-	a

b	b	a	b	a	b	b	-	b	a
b	b	a	-	a	b	b	b	b	a
b	b	a	b	a	b	b	b	b	b
b	b	a	-	a	b	b	b	b	a
b	b	a	b	a	b	b	b	b	a
b	b	a	-	a	b	b	b	b	a
-	b	b	-	a	b	b	b	b	a
b	b	b	b	a	b	b	b	b	a
b	b	b	b	a	b	b	b	b	a
b	b	b	-	a	b	b	a	b	a
b	b	b	b	a	b	b	a	b	b
b	b	b	-	a	b	b	a	b	a
b	b	b	b	a	b	b	a	b	a
b	b	b	b	a	b	b	a	b	a
b	b	b	b	a	b	b	a	b	a
b	b	b	b	a	b	b	a	b	a
b	b	b	b	a	b	b	a	b	a
-	-	b	b	a	b	b	a	b	a
b	b	b	-	a	b	b	a	b	a
b	b	b	b	a	-	b	a	-	a
b	b	b	b	a	b	b	a	b	a
b	b	b	-	a	b	b	a	b	b
b	b	b	b	a	b	b	a	b	a
b	b	b	-	a	b	b	a	-	a
b	b	b	b	a	b	b	a	b	a
b	b	b	b	a	b	b	a	b	a
b	b	b	b	a	b	b	a	b	-
b	b	b	b	a	b	b	a	b	a
-	b	b	b	a	a	b	a	b	a
a	b	b	b	a	a	b	a	b	a
a	b	b	b	a	a	b	a	b	a
-	b	b	b	a	a	b	a	b	a
-	b	b	b	a	a	b	a	b	a
a	-	b	b	a	a	b	a	b	a
a	b	b	b	a	a	b	b	b	a
a	b	-	b	a	a	b	b	b	a
a	b	b	b	a	b	b	b	b	a
a	b	b	a	a	a	b	b	b	a
a	b	b	a	a	a	b	b	b	-
a	b	b	a	a	a	b	b	b	b
a	b	b	a	a	a	b	b	b	-
a	b	b	a	a	a	b	b	-	b
a	b	b	a	a	a	a	b	b	b
a	b	b	a	a	a	a	b	b	b
a	b	b	a	a	a	a	b	b	b
a	b	b	a	a	a	a	b	b	-
a	a	b	a	a	a	a	a	b	b

a	a	b	b	a	a	a	a	b	b
a	a	b	b	a	a	a	a	b	b
a	a	b	b	a	a	a	a	b	b
a	a	b	b	a	a	a	a	b	b
a	a	b	b	a	a	a	a	b	b
a	a	b	b	a	a	a	a	b	b
a	a	b	b	a	a	a	a	b	b
a	a	b	b	a	a	a	a	b	b
a	a	b	b	a	a	a	a	b	b
a	a	b	b	a	a	a	a	b	b
a	a	b	b	a	a	a	a	b	b
a	a	b	b	a	a	a	a	b	b
a	a	b	b	a	a	a	a	b	b
a	a	b	b	a	a	a	a	b	b
a	a	b	b	a	a	a	a	b	b
a	a	b	b	a	a	a	a	b	b
a	a	b	b	a	a	a	a	b	b
a	-	b	a	a	-	a	a	-	b
a	a	b	b	a	a	a	a	b	b
a	a	b	b	a	a	a	a	b	b
a	a	b	b	a	a	a	a	b	b
a	a	b	b	a	a	a	a	b	b
a	a	b	b	a	a	a	b	b	b
a	a	b	b	a	a	a	b	b	b
a	a	b	b	a	b	a	b	b	b
a	a	b	b	a	b	a	b	b	b
a	a	b	b	a	b	a	b	b	b
a	a	b	b	a	b	a	b	b	b
a	a	b	b	a	b	a	b	b	b
a	a	b	b	a	b	a	b	a	-
a	a	b	b	a	b	a	b	a	a
a	a	b	b	b	b	a	b	a	b
a	a	b	b	b	b	a	b	a	-
a	a	b	b	b	a	a	b	a	a
a	a	b	b	b	b	a	b	a	b
a	a	b	b	b	b	a	b	a	b
a	a	b	b	b	b	a	b	a	b
a	a	b	b	b	b	a	b	a	b
a	a	b	b	b	b	a	b	a	b
a	a	b	b	a	b	a	b	a	b
b	b	a	a	b	a	b	a	b	a
a	b	b	a	a	b	a	b	a	b
a	b	b	a	a	b	a	b	a	b
a	b	b	a	a	b	a	b	a	b
a	b	b	a	a	b	a	b	a	b
a	b	b	a	a	b	a	b	a	b
a	b	b	a	a	b	a	b	a	b

a	b	b	a	a	b	a	b	a	b
a	b	b	a	a	b	a	b	a	b
a	b	b	a	a	b	a	b	a	b
a	b	b	a	a	b	a	b	a	b
a	b	b	-	b	b	a	b	a	b

YW111	YW112	YW113	YW114	YW115	YW116	YW117	YW118	YW119	YW120
b	b	b	b	a	b	a	a	b	-
b	b	b	b	a	b	a	a	b	a
b	b	b	b	a	b	a	a	b	b
b	b	b	b	a	b	a	a	b	b
b	b	b	b	a	b	a	a	b	b
b	b	b	b	a	b	a	a	b	b
b	b	b	b	a	b	a	a	b	b
-	b	-	b	a	b	a	a	b	b
b	b	b	b	a	b	a	a	b	b
-	b	b	b	a	b	a	a	b	b
b	-	-	b	a	b	a	a	b	b
b	b	b	b	a	b	a	a	b	b
-	b	a	a	a	b	a	a	b	b
b	b	b	b	a	b	a	a	b	b
b	b	a	b	a	b	a	a	b	b
b	b	a	b	a	b	a	a	b	b
b	b	a	b	a	b	a	a	b	b
b	b	a	b	a	b	a	a	b	b
b	b	a	b	a	b	a	a	b	b
b	b	-	b	a	b	a	a	b	b
b	b	a	b	a	b	a	a	b	-
b	b	a	b	a	b	a	a	b	b
b	b	a	b	a	b	a	a	b	b
b	b	a	b	a	b	a	a	b	b
b	b	a	b	a	b	a	a	b	b
b	b	a	b	a	b	a	a	b	-
b	b	a	b	a	b	a	a	b	b
b	-	a	a	a	b	a	a	b	b
b	b	a	b	a	b	a	a	b	a
b	b	a	b	a	b	a	a	b	a
-	b	a	b	a	b	a	a	b	a
b	b	a	b	a	b	a	a	b	a
b	b	a	b	a	b	a	a	b	a
b	b	a	b	a	a	a	a	b	a
b	b	a	b	a	a	a	a	b	a
b	b	a	b	a	a	a	a	b	a
b	b	a	b	a	a	a	a	b	a
b	b	a	b	a	a	a	a	b	a
b	b	a	b	a	a	a	a	b	a
b	b	a	b	a	a	a	a	b	-
b	b	a	b	a	a	a	a	b	a
b	b	a	-	a	a	a	a	b	a
b	b	a	b	a	a	-	a	b	a
b	b	a	b	a	a	a	a	b	a
b	b	a	b	a	a	a	a	b	a

b	b	-	b	a	a	a	a	b	a
-	b	a	b	a	a	a	a	b	a
b	-	a	b	a	a	a	a	b	a
b	b	a	b	a	a	a	a	b	a
b	b	a	b	a	a	a	a	b	a
b	b	a	b	a	a	a	a	b	a
b	b	a	a	a	a	a	a	b	a
b	b	-	a	a	a	a	a	b	a
b	b	a	a	a	a	a	a	b	a
b	b	a	a	a	a	a	a	b	a
b	b	a	a	a	a	a	a	b	a
b	b	a	a	a	-	a	a	b	a
b	b	a	a	a	a	a	a	b	a
b	b	a	a	a	a	a	a	b	a
b	b	a	b	a	a	a	b	b	a
b	b	a	a	a	a	a	b	b	a
b	b	a	a	a	a	a	b	b	a
b	b	a	a	a	a	a	b	b	a
b	b	a	a	a	a	a	b	b	a
-	b	a	a	a	a	a	b	b	a
b	b	-	a	a	a	a	b	b	a
-	b	a	a	a	a	a	b	b	a
-	b	a	a	a	a	a	b	b	-
b	b	a	a	a	a	a	b	b	a
b	b	a	-	a	a	a	b	b	a
b	b	a	a	a	a	a	b	b	a
a	b	a	a	a	a	a	b	b	a
a	b	a	a	a	a	a	b	b	a
a	b	a	a	a	b	a	b	b	a
a	b	a	a	a	b	-	b	b	a
a	b	a	a	a	b	a	b	b	a
-	b	a	a	a	b	-	b	b	a
a	b	a	a	a	b	a	b	b	a
a	b	a	a	a	b	a	b	b	a
a	b	a	a	a	b	a	b	b	a
a	b	a	a	a	b	a	b	b	a
a	b	a	a	a	b	a	b	b	-
a	-	a	a	a	b	a	b	b	a
a	-	a	a	a	b	a	b	b	a
a	-	a	b	b	b	b	a	a	a
-	b	a	b	b	b	b	a	a	a
a	b	a	b	b	b	b	a	a	a
a	b	a	b	b	b	b	a	a	a
a	b	a	b	b	b	b	a	a	a
a	b	a	b	b	b	b	a	a	a
a	b	a	b	b	b	b	a	a	a
a	-	a	b	b	b	b	a	a	a

a	b	a	b	b	b	b	a	a	-
a	b	a	b	b	b	b	a	a	a
a	b	a	b	b	b	b	a	a	a
a	a	a	a	b	b	b	a	a	a
a	a	a	b	b	b	b	a	a	a
a	a	a	b	b	b	b	a	a	a
a	a	a	b	-	b	b	a	a	a
-	-	a	b	b	b	b	a	a	a
a	a	a	b	b	b	b	a	a	a
a	a	a	b	b	b	b	a	a	a
a	a	a	b	b	b	b	a	a	a
a	a	a	b	b	b	b	a	a	a
a	a	a	b	b	b	b	a	a	a
a	a	b	a	b	b	b	a	a	b
a	a	b	a	b	b	b	a	a	b
-	a	b	-	b	b	b	a	a	b
a	a	b	-	b	b	b	a	a	b
-	a	b	-	b	b	b	a	a	b
a	a	b	a	b	b	b	a	a	b
-	a	b	a	b	a	b	a	a	b
a	-	b	b	b	a	b	a	a	b
a	a	b	a	b	a	b	a	a	b
a	a	b	a	b	a	b	a	a	b
a	a	b	a	b	a	b	a	a	b
a	a	b	-	b	a	b	a	a	b
a	a	b	a	b	a	b	a	a	b
a	a	b	a	b	a	b	a	a	-
a	a	b	a	b	a	b	a	a	b
a	a	b	b	b	a	b	a	a	b
a	a	b	a	b	a	b	a	a	b
a	a	b	a	b	a	b	a	a	b
a	a	b	a	b	a	b	a	a	b
a	a	b	a	a	a	b	a	a	b
a	a	b	a	a	a	b	a	a	b
a	a	b	a	a	a	b	a	a	b
a	a	b	a	a	a	b	a	a	-
-	a	b	b	a	a	b	a	a	b
a	a	b	-	a	a	b	a	a	b
a	-	b	a	a	a	b	a	a	b
a	a	b	a	a	a	b	a	a	b
a	a	b	a	a	a	b	a	a	b
a	a	b	a	a	a	b	a	a	b
a	a	b	a	a	a	b	a	a	b
a	a	b	a	a	a	b	a	a	b
a	-	b	a	a	a	b	a	a	b
a	a	b	a	a	a	b	a	a	b
a	a	b	a	a	a	b	a	a	b

-	a	b	a	a	a	b	a	a	b
a	a	b	a	a	a	b	a	a	b
a	a	b	a	a	b	b	a	a	b
a	a	b	a	a	a	b	a	a	b
a	a	b	a	a	a	b	a	a	b
a	-	b	a	a	a	b	a	a	b
a	a	b	a	a	a	b	b	a	b
a	a	b	a	a	a	b	b	a	b
-	a	b	-	a	a	b	b	a	b
-	a	b	-	a	a	b	b	a	b
a	a	b	a	a	a	b	b	a	b
a	a	b	a	a	a	b	b	a	b
a	a	b	-	a	a	b	b	a	b
a	a	b	a	a	a	b	b	a	-
a	a	b	a	a	a	b	b	a	b
a	a	b	-	b	a	b	b	a	b
a	a	b	a	a	a	b	b	a	-
a	a	b	a	a	a	b	b	a	b
a	a	b	-	a	a	b	b	a	b
a	a	b	a	a	a	b	b	a	b
a	a	b	a	a	a	b	b	b	b
a	a	b	b	a	a	b	b	b	b
a	a	b	a	a	a	a	b	-	a
a	a	b	a	a	a	a	b	b	a
a	a	b	a	a	a	a	b	b	a
a	a	b	a	a	a	a	b	b	a
a	a	-	a	a	a	a	b	b	a
a	a	b	a	a	a	a	b	b	a
a	a	b	a	a	a	a	b	b	a
b	a	b	b	a	a	a	b	b	a
b	a	b	b	a	a	-	b	b	a
b	a	b	b	a	a	a	b	b	a
b	a	-	b	a	a	a	b	b	-
b	a	b	b	a	a	a	b	b	a
b	a	b	b	a	a	a	b	b	a
b	a	b	b	a	a	a	b	b	a
b	a	b	b	b	a	a	b	b	-
b	a	b	b	b	a	a	b	b	-
b	a	b	b	b	a	a	b	b	a
b	a	b	-	b	a	a	b	b	a
b	a	b	b	b	a	a	b	b	a
b	a	b	b	b	a	a	b	b	a
b	a	a	b	b	a	a	b	b	a
-	a	-	-	b	a	a	b	b	a
b	a	b	b	b	a	a	b	b	a
b	a	b	b	b	a	a	b	b	b
b	a	b	b	b	a	a	b	b	b

b	a	-	b	b	a	a	b	b	b
b	-	b	b	b	a	a	b	b	b
b	a	b	b	b	a	a	b	b	b
b	a	b	b	b	a	a	b	b	b
b	a	b	b	b	a	a	b	b	b
b	a	b	b	b	a	a	b	b	b
b	a	b	b	b	a	a	b	b	-
b	a	b	b	b	a	a	b	b	b
-	a	b	b	b	a	a	b	b	b
b	a	b	b	b	a	a	b	b	b
b	a	b	b	b	a	a	b	b	b
b	a	b	b	b	a	a	b	b	b
b	b	a	a	a	b	a	a	b	-
b	-	a	a	a	b	a	a	b	b
b	b	a	a	a	b	a	a	b	b
b	b	a	a	a	b	a	a	b	b
b	b	a	a	a	b	a	a	b	b
b	b	a	a	a	b	a	a	b	b
b	b	a	a	a	b	a	a	b	b
b	b	a	a	a	b	a	a	b	b
b	b	a	a	-	b	a	a	b	-
b	b	a	-	a	b	a	a	b	b
b	b	a	a	a	b	a	a	b	-
b	b	a	a	a	b	a	a	b	-
b	b	a	a	a	b	a	a	b	b
b	b	a	a	a	b	a	a	b	b
a	b	-	a	a	b	a	a	b	b
a	b	b	a	a	b	a	a	b	b
a	b	b	-	a	b	a	a	b	b
-	b	b	a	a	b	a	a	b	a
a	b	b	a	a	b	a	a	b	a
a	b	b	a	a	b	a	a	b	a
a	b	b	a	a	b	a	a	b	a
a	b	b	a	a	b	a	a	b	a
a	a	b	a	a	b	a	a	b	a
a	a	b	a	a	b	a	a	b	a
-	a	b	-	a	b	a	a	b	-
a	a	b	a	a	b	a	-	b	a
a	a	b	a	a	b	a	a	b	a
a	a	b	-	-	b	a	a	b	a
a	a	b	a	a	b	a	a	b	a
a	a	b	a	a	b	a	b	b	a
a	a	b	a	a	b	a	b	b	a
a	a	b	a	a	b	a	b	b	a
a	a	b	a	a	b	a	b	b	a
a	a	-	a	a	b	a	b	b	a
a	a	b	-	a	b	a	b	a	a

a	-	b	a	a	b	a	b	a	a
a	a	-	a	a	b	a	b	a	a
a	a	-	a	a	b	a	b	a	a
a	a	b	a	a	b	a	b	a	a
a	a	b	b	a	b	a	b	a	a
a	a	b	a	a	b	a	b	a	a
a	a	b	a	a	b	a	b	a	a
a	a	b	a	a	b	a	b	a	a
a	a	b	a	a	b	a	b	a	a
a	a	b	a	a	b	a	b	a	a
a	a	b	a	a	b	a	b	a	a
a	a	a	a	a	b	a	b	a	a
a	a	b	a	a	b	a	b	a	a
a	a	b	a	a	b	a	b	a	a
a	a	b	a	a	b	a	b	a	a
a	a	b	a	a	b	a	b	a	a
a	a	b	-	a	b	a	b	a	a
a	a	b	b	a	b	a	b	a	a
a	a	b	a	a	b	a	b	a	-
a	a	b	-	a	b	a	b	a	a
a	-	b	a	a	b	a	b	a	-
a	a	-	a	a	b	a	b	a	a
a	a	b	a	a	b	a	b	a	a
a	a	b	a	a	b	a	b	a	a
a	a	b	a	a	b	a	b	a	a
a	a	b	a	a	b	a	b	a	a
a	a	b	a	a	b	a	b	a	a
a	a	b	-	a	b	a	b	a	a
a	a	-	b	a	b	a	b	a	a
a	a	b	b	a	b	a	b	a	a
-	a	a	b	a	b	a	b	a	a
a	a	a	b	a	b	a	b	a	a
a	a	a	-	a	b	a	b	a	a
a	a	a	b	a	b	a	b	a	a
a	a	a	-	a	b	a	b	a	a
a	a	a	b	a	b	a	b	a	a
a	a	a	-	a	b	a	-	a	a
a	a	a	-	a	b	a	b	a	a
a	a	a	b	a	b	a	b	a	a
a	a	a	b	a	b	a	b	a	a
a	a	a	b	a	b	a	b	a	a
a	a	-	b	a	b	a	b	a	a
a	a	a	b	a	b	a	b	a	a
a	a	a	b	a	b	a	b	a	a
a	a	a	-	a	b	a	b	a	a
a	a	a	-	a	b	a	b	a	a
a	a	a	-	a	b	a	-	a	a

a	a	a	-	a	b	a	b	a	a
a	a	a	b	a	b	a	b	a	a
a	a	a	b	a	b	b	b	a	a
a	a	a	b	a	b	b	b	a	a
a	a	a	b	a	b	b	b	a	a
a	a	b	b	a	b	b	b	a	a
a	a	b	b	a	b	b	b	a	a
a	a	b	b	a	b	b	b	a	a
a	a	b	b	a	b	b	b	a	a
a	a	b	b	a	b	b	b	a	a
a	a	b	b	a	b	b	b	a	a
a	b	b	b	a	b	b	b	a	a
a	b	b	b	a	b	b	b	a	a
a	-	b	b	a	b	b	-	a	a
a	b	b	a	a	b	b	b	a	a
a	b	b	-	a	b	b	b	a	-
a	b	b	b	a	b	b	b	a	b
a	b	b	-	a	b	b	b	a	b
a	b	b	b	a	b	b	b	a	b
a	-	b	-	a	b	b	b	a	-
a	b	b	b	a	b	b	b	a	b
a	b	b	b	a	b	b	b	a	b
a	b	b	b	a	b	b	b	a	b
a	b	b	b	a	b	b	b	a	b
-	b	b	b	-	b	b	b	a	b
a	b	b	b	a	b	b	b	a	b
a	b	b	b	a	b	b	b	a	b
a	b	b	b	a	b	b	b	a	b
a	b	b	b	a	b	b	b	a	b
a	b	b	b	a	b	b	b	a	b
a	b	b	b	a	b	b	b	a	b
a	b	b	b	a	b	b	b	a	b
a	b	b	-	a	b	b	b	a	b
a	b	b	b	a	b	b	b	a	b
a	b	b	b	a	b	b	b	a	b
a	b	b	b	a	b	b	b	a	-
a	b	b	b	a	b	b	b	a	a
a	b	b	b	a	b	b	b	a	a
a	a	b	a	b	a	a	a	a	a
a	a	b	a	b	a	a	a	a	a
a	a	b	a	b	a	a	a	a	a
a	a	b	a	b	a	a	a	a	-
-	a	b	a	b	a	a	a	a	a
a	a	b	a	b	b	b	a	-	a
a	a	b	a	b	b	b	a	a	a
a	a	b	-	b	b	b	a	a	a
a	a	b	a	b	b	b	a	a	a

a	a	b	a	b	b	b	a	a	a
a	a	b	a	b	b	b	a	a	a
a	a	-	a	b	b	b	a	a	a
a	a	b	a	b	b	b	a	a	a
a	a	b	a	b	b	b	a	a	a
a	-	b	-	b	b	b	a	a	-
a	a	b	a	b	b	b	a	a	a
a	a	b	a	b	b	b	a	a	a
a	a	b	a	b	b	b	a	a	a
a	a	b	a	a	b	b	a	a	a
-	a	b	a	b	b	b	a	a	a
a	a	b	a	a	b	b	a	a	a
a	a	b	a	a	b	b	a	a	a
a	a	b	a	a	b	b	a	a	a
a	a	b	-	a	b	b	a	a	a
-	a	b	a	a	b	b	a	a	a
b	a	b	-	a	b	b	a	a	a
b	a	b	a	a	b	b	a	a	a
b	a	b	b	a	b	b	a	a	a
b	a	b	b	a	b	b	b	a	a
-	a	b	-	a	b	b	b	a	-
-	a	b	-	a	b	b	b	a	a
b	a	b	b	a	b	b	b	a	a
b	a	b	b	a	b	b	b	a	-
b	a	b	-	a	b	b	b	a	a
b	a	b	b	a	b	b	b	a	a
b	a	b	b	a	b	b	b	a	a
b	a	b	b	a	b	b	b	a	a
b	a	b	b	a	b	b	b	a	a
b	a	b	b	a	b	b	b	a	a
b	a	b	b	a	b	b	b	a	a
b	a	b	b	a	b	b	b	a	a
b	a	b	b	a	b	b	b	a	a
b	a	b	b	a	b	b	b	a	a
b	a	b	a	-	b	b	b	b	a
b	a	b	b	a	b	b	b	a	a
b	a	b	a	a	b	a	b	b	b
b	a	b	a	a	b	a	b	b	b
b	a	b	-	a	b	a	b	b	b
b	a	b	a	a	b	a	b	b	b
b	a	b	a	a	b	a	b	b	-
b	a	b	a	a	b	a	b	b	b
b	a	b	a	a	b	b	b	b	b
b	a	b	a	a	b	b	b	b	b
b	a	b	b	a	b	b	b	b	b
b	a	b	a	a	b	b	b	b	b
b	a	b	a	a	b	b	b	b	b

b	a	b	a	a	b	b	b	b	b
b	a	b	a	a	b	b	b	b	b
b	a	b	a	a	b	b	b	b	b
b	a	b	a	a	b	b	b	b	b
b	a	b	a	a	b	b	b	b	b
b	a	b	a	a	b	b	b	a	b
b	a	b	a	a	b	b	b	a	b
b	a	b	b	a	b	b	b	a	b
b	a	a	a	b	b	b	b	a	b
b	a	a	a	b	b	b	b	a	b
b	a	a	a	-	b	b	b	a	b
-	a	a	a	b	b	b	b	a	b
b	a	a	a	b	b	b	b	a	b
b	a	a	a	b	b	b	b	a	b
b	a	a	a	b	b	b	b	a	-
b	a	a	a	b	b	b	b	a	-
b	a	a	b	b	b	b	b	a	b
b	a	a	-	b	b	b	b	a	b
b	a	a	a	b	b	b	b	a	b
b	a	a	a	b	b	b	b	a	b
b	a	a	a	b	b	b	b	a	b
b	a	a	-	b	b	b	b	a	b
b	a	a	a	b	b	b	b	a	-
b	a	a	a	b	b	b	b	a	b
b	a	a	a	b	b	a	b	a	b
b	a	a	a	b	b	a	b	a	b
-	a	a	a	a	b	a	b	a	b
b	a	a	b	a	b	a	b	a	b
b	a	a	b	b	b	a	b	a	b
b	a	a	b	b	b	a	b	a	b
b	a	a	b	b	b	a	b	a	b
b	a	a	b	b	b	a	b	a	b
b	a	a	b	b	b	a	b	a	b
b	a	-	b	b	b	a	b	a	b
b	a	a	b	b	b	a	b	a	b
b	a	a	b	b	b	a	b	a	b
a	a	a	b	b	b	a	b	a	b
b	a	a	b	b	b	a	b	a	b
b	a	a	a	a	b	a	b	a	b
b	a	a	-	a	b	a	b	a	b
b	b	b	b	a	a	b	b	b	b
b	b	b	b	a	a	b	b	b	b
b	b	b	b	a	a	b	b	b	b
-	b	b	b	a	a	b	b	b	-
a	b	b	b	a	a	b	b	b	b
a	b	b	b	a	a	b	b	b	b
a	b	b	b	a	a	b	b	b	b

a	b	b	-	a	a	b	b	b	b
a	b	b	-	a	a	b	b	b	b
a	b	b	a	a	a	b	b	b	-
a	b	b	b	a	a	b	b	b	b
a	b	b	b	a	a	b	b	b	b
-	b	b	b	a	a	b	b	b	b
a	b	b	b	a	a	b	b	b	b
a	b	b	b	a	a	b	b	b	b
a	b	-	b	a	a	b	b	b	b
a	b	b	a	a	a	b	b	b	b
-	b	b	b	a	a	b	b	b	b
a	b	b	b	a	a	b	b	b	b
a	b	b	b	a	a	b	b	b	b
a	b	b	b	a	a	b	b	b	b
a	b	b	-	a	a	b	b	b	b
a	b	b	b	a	a	b	b	b	b
-	b	b	b	a	a	b	b	b	b
a	b	b	b	a	a	b	b	b	b
a	b	b	b	a	a	b	b	b	b
a	b	b	b	a	a	b	b	b	b
a	b	b	b	a	a	b	b	b	b
a	b	b	b	a	a	a	b	b	b
a	b	b	b	a	a	a	b	b	b
a	b	b	-	a	a	a	b	b	-
a	b	b	b	a	a	a	b	b	b
a	b	b	b	a	a	a	b	b	b
a	b	-	-	a	a	a	b	b	-
a	b	b	b	a	a	a	b	b	b
a	b	-	-	a	a	a	a	b	b
a	b	b	b	a	a	a	a	b	b
a	b	b	-	a	a	a	a	b	b
a	b	-	b	a	a	a	a	b	b
a	b	b	a	a	a	a	a	b	b
a	b	b	b	a	a	a	a	b	b
a	-	b	b	a	a	a	a	b	b
a	b	b	b	a	a	a	a	b	b
a	b	b	b	-	a	a	a	b	b
a	b	b	b	a	a	a	a	b	b
a	b	b	b	a	a	a	a	b	b
a	b	b	b	a	a	a	a	b	b
a	b	b	b	a	a	a	a	a	b
a	b	b	b	b	a	a	a	a	b
a	b	-	b	b	a	a	a	a	b
a	b	b	b	b	a	a	a	a	b
a	b	b	b	b	a	a	a	a	b
a	b	b	b	b	a	a	a	a	b
a	b	b	b	b	a	a	a	a	b

a	b	b	b	b	a	a	a	a	b
a	b	b	b	b	a	a	a	a	b
a	b	b	b	b	a	a	a	a	b
a	b	b	b	b	a	a	a	-	b
a	b	b	-	b	a	a	a	a	b
a	b	b	b	b	a	a	a	a	b
a	b	b	b	b	a	a	a	a	b
a	b	b	b	b	a	a	a	a	b
a	b	b	b	b	a	a	a	a	b
a	b	b	b	b	a	a	a	a	b
a	b	b	b	b	a	a	-	a	b
a	b	b	b	b	a	a	a	a	b
a	b	b	b	b	a	a	a	a	b
a	b	b	-	b	a	a	a	a	b
a	b	b	b	b	a	a	a	a	b
a	b	b	b	b	a	a	a	a	b
a	b	b	b	b	a	a	a	a	b
a	b	b	b	b	a	a	a	a	b
a	b	b	b	b	a	a	a	a	b
a	b	b	b	b	a	a	a	a	-
a	b	b	b	b	a	a	a	a	b
a	b	b	b	b	a	a	a	a	b
a	b	b	b	b	a	a	b	a	b
a	b	b	b	b	a	a	b	a	b
a	-	b	b	b	a	a	b	a	b
a	b	b	b	b	a	a	b	a	b
a	b	b	b	b	a	a	b	a	b
-	b	b	b	b	a	a	b	a	b
a	b	b	b	b	a	a	b	a	b
a	b	b	b	b	a	a	b	a	b
a	b	b	b	b	a	a	b	a	b
a	b	b	b	b	b	a	b	a	b
a	b	b	b	b	b	a	b	a	a
a	b	b	b	b	b	a	b	a	a
a	b	b	b	b	b	a	b	a	-
a	b	b	b	b	b	a	b	a	a
a	b	b	b	b	b	a	b	a	a
a	b	b	b	b	b	a	b	a	a
a	b	b	b	b	b	a	b	a	a
a	b	b	b	b	b	a	b	a	a
a	b	b	b	b	b	a	b	a	a
a	b	b	-	b	b	a	b	a	a
a	-	b	b	b	b	a	b	a	a
a	-	b	b	b	b	a	b	a	a
a	b	b	b	b	b	a	b	a	a
a	b	b	b	b	b	a	b	a	a
a	b	b	b	b	b	a	b	a	a

a	b	b	b	b	b	a	b	a	a
a	b	b	b	b	b	-	b	a	a
a	b	b	b	b	b	a	b	a	a
a	b	b	b	b	b	a	b	a	a
a	b	b	b	b	b	a	b	a	a
a	b	b	b	b	b	a	b	a	a
a	b	b	b	b	b	a	b	a	a
a	b	b	b	b	b	a	b	a	a
a	b	b	b	b	b	a	b	a	a
a	b	b	b	b	b	a	b	a	a
a	b	b	b	b	b	a	b	a	a
a	b	b	b	b	b	a	b	a	a
a	b	b	b	b	b	a	b	b	a
a	-	-	-	b	b	a	b	b	a
a	b	b	b	b	b	a	b	b	a
a	b	b	b	b	b	a	b	b	a
a	b	b	b	b	b	a	b	b	a
a	b	b	b	b	b	a	b	b	a
a	b	b	b	b	b	a	b	a	a
a	b	b	a	b	b	b	a	b	a
a	b	b	a	b	b	b	a	b	a
a	b	b	a	b	b	b	a	b	a
a	b	b	a	b	b	b	a	b	a
a	b	b	-	b	b	b	a	b	a
a	b	-	a	b	b	b	a	b	a
a	b	a	a	b	b	b	a	b	a
a	b	a	a	b	b	b	a	b	a
-	b	a	a	b	b	b	a	b	a
a	b	a	a	b	b	b	a	b	-
a	b	a	a	b	b	b	a	b	a
a	b	a	a	b	b	b	a	b	a
a	-	a	a	b	b	b	a	b	a
a	b	a	a	b	b	b	a	b	a
a	b	a	a	b	b	-	a	b	a
a	b	a	a	b	b	b	a	b	a
a	b	a	a	b	b	b	a	b	a
a	-	a	a	b	a	b	a	b	a
a	b	a	a	b	a	b	a	b	a
a	b	a	a	b	a	b	a	b	a
a	b	a	a	b	a	b	a	b	a
a	-	a	a	b	a	b	a	b	a
a	b	a	a	b	a	b	a	b	a
a	b	a	-	b	a	b	a	b	a
a	b	a	a	b	a	b	a	b	a
a	b	a	a	b	a	b	a	b	a
-	b	a	a	b	a	b	a	b	a
a	b	a	-	b	a	b	a	b	a

a	b	a	a	b	a	b	a	b	a
a	b	a	a	b	a	b	a	b	a
a	b	a	a	b	a	b	a	b	-
a	b	a	a	b	a	b	a	b	a
a	b	a	a	b	a	b	a	b	a
a	b	a	a	b	a	b	a	b	-
a	b	a	a	b	a	b	a	b	a
a	b	a	a	b	a	b	a	b	-
a	b	a	a	b	a	b	a	b	a
a	b	a	a	b	a	b	a	b	-
a	b	a	a	b	a	b	a	b	a
a	b	a	a	b	a	b	a	b	a
a	b	a	a	b	a	b	a	b	a
a	b	a	a	b	a	b	a	b	-
a	b	a	a	b	a	b	a	b	a
a	b	a	a	b	a	b	a	b	a
a	b	-	a	b	a	b	a	b	-
a	-	a	a	b	a	b	a	b	a
a	-	a	a	b	a	b	a	b	a
a	b	a	a	b	a	b	a	b	a
a	b	a	a	b	a	b	a	b	a
a	b	a	a	b	a	b	a	b	a
a	b	a	a	b	a	b	a	b	a
a	b	a	a	b	a	b	a	b	a
a	b	a	a	b	a	b	a	b	-
a	b	a	a	b	a	b	a	b	a
a	b	a	a	b	a	b	a	b	a
a	b	a	a	b	a	b	a	b	a
a	b	a	a	b	a	b	a	b	-
a	b	a	a	b	a	b	a	b	-
a	b	a	a	b	a	b	a	b	a
a	b	a	a	b	a	b	a	b	a
a	b	a	-	b	a	b	a	b	-
a	b	a	b	a	a	b	a	b	a
a	b	a	a	a	a	b	a	b	a
a	b	a	a	a	a	b	a	b	a
a	b	a	a	a	a	b	a	b	a
a	b	a	a	a	a	b	a	b	a
a	b	a	a	a	a	b	a	b	a
a	b	a	a	a	a	b	a	b	a
a	b	a	a	a	a	b	a	b	a
a	-	a	a	a	a	b	a	b	a
a	b	a	a	a	a	b	a	b	a
a	b	a	a	a	a	b	a	b	a
b	-	a	a	a	a	a	a	b	a
b	-	a	a	a	a	a	a	b	a
b	b	b	a	a	a	-	a	b	a

b	-	b	a	a	a	a	a	b	a
b	b	b	a	a	a	a	a	b	a
b	b	b	a	a	a	a	a	b	a
b	b	b	a	a	a	a	a	b	a
b	b	b	-	a	a	a	a	b	a
b	-	b	b	a	a	a	a	b	a
b	b	b	b	a	a	a	a	b	a
b	b	b	b	a	b	a	a	b	a
b	b	b	-	a	b	a	a	b	a
b	b	b	b	a	b	a	a	b	a
b	b	-	b	a	a	a	a	b	a
a	b	b	a	a	a	b	a	b	b
a	b	b	a	a	a	b	a	b	b
a	b	b	-	a	a	b	a	b	b
a	b	b	a	a	a	b	a	b	b
a	b	b	a	a	a	b	a	b	b
a	b	b	a	a	a	b	a	b	b
a	b	b	a	a	a	b	a	b	b
a	b	b	a	a	a	b	a	b	b
a	b	b	a	a	a	b	a	b	-
a	b	b	a	a	a	b	a	b	b
a	b	a	a	a	a	b	a	b	b
a	b	a	a	a	a	b	a	b	b
a	b	a	a	a	a	b	a	b	b
a	b	a	a	a	a	b	a	b	-
a	b	a	a	a	a	b	a	b	b
a	b	a	-	a	a	b	a	b	b
a	b	a	a	a	a	b	a	b	b
a	b	a	a	a	a	b	a	b	b
a	b	a	a	a	a	b	a	b	b
a	b	a	a	a	a	b	a	b	-
a	b	a	-	a	a	b	a	b	b
a	b	a	a	a	a	b	a	b	b
a	b	a	a	a	a	b	a	b	b
a	b	a	a	a	a	b	a	b	b
a	b	a	a	a	a	-	a	b	b
a	b	a	a	a	a	b	a	b	b
a	b	a	a	a	a	b	a	b	b
-	b	a	a	a	a	b	a	b	b
a	-	a	a	a	a	b	a	b	b
a	b	a	a	a	a	b	a	b	b
a	b	a	a	a	a	b	a	b	b
-	b	-	a	a	a	b	a	b	b
a	b	-	a	a	a	a	b	b	b
a	b	a	a	a	a	a	b	b	b
a	b	a	a	a	a	a	b	a	b
a	b	a	a	a	a	a	b	a	-

a	b	a	a	a	a	a	b	a	b
a	b	a	a	a	a	a	b	a	b
a	b	a	a	a	a	a	b	a	b
a	b	a	a	a	a	a	b	a	-
a	b	-	-	a	a	a	b	a	b
a	b	a	a	a	a	a	b	a	b
a	b	a	a	a	a	a	b	a	b
a	b	a	a	a	a	a	b	a	-
a	b	a	a	a	a	a	b	a	b
a	b	a	a	a	a	a	b	a	b
-	b	a	a	a	a	a	b	a	-
a	b	a	a	a	a	a	b	a	b
a	b	a	-	-	a	a	b	a	b
a	b	a	a	a	a	a	b	a	b
a	b	-	a	a	a	a	b	a	b
a	b	a	a	a	a	a	b	a	b
a	b	a	a	a	a	a	b	a	b
a	b	a	a	a	a	a	b	a	b
a	b	a	a	a	a	a	b	a	-
a	b	a	a	a	a	a	b	a	b
a	b	b	a	a	a	a	b	a	b
a	b	a	a	a	a	a	b	a	a
a	b	a	a	a	a	a	b	a	a
a	-	a	a	a	a	a	b	a	a
a	b	a	a	a	a	a	b	a	-
a	b	a	a	a	a	a	b	a	a
a	b	a	a	a	a	a	b	a	a
a	b	a	a	a	a	a	b	a	a
a	b	a	b	a	a	a	b	a	a
a	b	a	a	a	a	a	b	a	a
a	b	a	a	a	a	a	b	a	a
a	b	a	a	a	a	a	b	a	a
a	-	a	a	a	a	a	b	a	a
a	b	a	a	a	a	a	b	a	a
a	-	a	a	a	a	a	b	a	a
-	b	a	a	a	a	a	b	a	a
a	b	a	a	a	a	a	b	a	a
a	b	a	b	a	a	a	b	a	a
a	-	a	b	a	a	a	b	a	a
a	b	a	b	a	a	a	b	a	a
a	b	a	b	a	a	a	b	a	a
b	b	a	a	a	a	a	b	a	a
b	b	a	a	a	a	a	b	a	a
b	b	a	a	a	a	a	b	a	-
b	b	a	a	a	a	a	b	a	a
b	b	a	a	a	a	a	b	a	a
b	b	a	-	a	a	a	b	a	a

-	b	a	a	a	a	a	b	a	a
b	b	a	a	a	a	a	b	a	a
b	b	a	a	a	a	a	b	a	a
b	b	a	a	a	b	a	b	a	b
b	b	a	a	a	b	a	b	a	a
b	b	-	a	a	b	a	b	a	a
b	b	a	-	a	b	a	b	a	a
b	b	a	b	a	b	a	b	a	a
b	b	a	a	a	b	a	b	a	a
b	b	a	a	a	b	a	b	a	a
b	b	a	a	a	b	a	b	a	a
a	b	a	a	a	b	a	b	a	a
-	b	a	a	a	b	a	b	a	a
a	b	a	a	a	b	a	b	a	a
a	b	a	a	a	b	a	b	a	a
-	b	a	a	a	b	a	b	a	a
a	-	a	a	a	b	a	b	a	a
a	b	a	-	a	b	a	b	a	a
a	b	a	a	a	b	a	b	a	a
a	b	a	a	a	b	a	b	a	a
a	b	a	a	a	b	a	b	a	a
a	b	a	a	a	b	b	b	a	a
-	a	a	b	a	a	a	b	b	b
b	a	a	b	a	a	a	b	b	b
-	a	a	b	a	a	a	b	b	b
b	a	a	b	a	a	a	b	b	b
-	a	a	b	a	a	a	b	b	b
-	a	a	b	a	a	a	b	b	b
a	a	a	b	a	a	b	b	b	b
a	a	a	b	a	a	b	b	-	b
a	a	a	b	a	a	b	b	b	-
a	a	b	-	a	a	b	b	b	b
a	a	b	-	a	a	b	b	b	b
a	a	b	b	a	a	b	b	b	b
-	a	b	b	a	a	b	b	b	b
a	a	b	b	a	a	b	a	b	b
a	a	b	b	a	a	b	a	b	b
a	a	b	b	a	a	b	a	b	b
a	a	b	b	a	a	b	a	b	b
a	a	b	b	a	a	b	a	b	a
a	a	b	b	a	a	b	a	b	a
a	b	b	b	a	a	b	a	b	a
a	b	b	b	a	a	b	a	b	a
a	b	b	b	a	a	b	a	b	a
a	b	b	b	a	a	b	a	b	a
a	b	b	b	a	a	b	a	b	a
-	b	b	b	a	a	b	a	b	a

a	b	b	b	a	a	b	a	b	a
a	b	b	b	a	a	b	a	b	a
-	b	b	b	a	a	b	a	b	a
b	b	a	-	a	b	b	a	b	a
b	b	a	b	a	-	b	a	b	a
-	b	a	b	a	b	b	a	b	-
b	b	a	b	a	b	b	b	b	a
-	b	a	b	a	b	b	b	b	a
b	-	a	-	a	b	b	b	b	a
b	b	a	b	a	b	b	b	b	a
b	b	a	b	a	b	b	b	b	a
b	b	a	b	b	b	b	b	b	a
b	b	a	-	b	b	b	b	b	a
b	b	a	b	b	b	b	b	b	a
b	b	a	b	b	b	b	b	b	a
b	b	a	b	b	b	b	b	b	a
b	b	a	b	b	b	b	b	b	a
b	b	a	b	b	b	b	b	b	a
b	b	a	b	b	b	b	b	b	a
-	b	a	b	b	b	b	a	b	a
b	b	a	b	b	b	b	a	b	a
b	b	a	b	b	b	b	a	b	-
b	b	a	b	b	b	b	a	b	a
b	b	a	b	b	b	b	a	b	a
b	b	a	b	b	b	b	a	b	a
b	b	a	b	b	b	b	a	b	a
b	b	a	b	b	b	b	a	b	a
b	b	a	b	b	b	b	a	b	a
b	b	a	b	b	b	b	a	b	a
-	b	a	b	b	b	b	a	b	-
b	b	a	b	b	b	b	b	b	a
-	-	a	-	b	b	b	b	-	a
b	b	a	b	b	b	b	b	b	a
b	b	a	b	b	b	b	b	b	a
b	b	a	b	b	b	b	b	b	a
b	b	a	b	b	b	b	b	b	a
b	b	a	b	b	b	b	b	b	-
b	b	a	b	b	b	b	b	b	a
b	b	a	b	b	b	b	b	b	a
b	b	a	b	b	b	b	b	b	a
b	b	a	a	b	b	b	b	b	a
b	b	a	b	b	b	b	b	b	a
b	b	a	b	b	b	b	b	b	a
-	b	a	-	b	b	b	b	b	a
b	b	a	b	b	b	b	b	b	a
b	b	a	b	b	b	b	b	b	a
b	b	a	b	b	b	b	b	b	a

b	b	a	b	b	b	b	b	b	a
b	b	a	b	b	b	b	b	b	a
-	b	a	b	b	b	b	b	b	a
b	b	a	b	b	b	b	b	b	a
b	b	a	b	b	b	b	b	b	a
b	b	a	b	b	b	b	b	b	a
b	b	a	b	b	b	b	b	b	b
b	b	a	b	b	b	b	b	b	b
b	b	a	b	b	b	b	b	b	b
b	b	a	b	b	b	b	b	b	b
b	-	a	b	b	b	b	b	b	b
b	b	a	b	b	b	b	b	b	b
b	b	a	b	b	b	b	b	b	b
b	b	a	b	b	b	b	b	b	b
b	b	a	b	b	b	b	b	b	b
-	-	a	b	b	b	b	b	b	b
b	b	a	b	b	b	b	b	b	b
b	b	a	b	b	b	b	b	b	b
b	b	a	b	b	b	b	b	b	b
b	b	a	b	b	b	b	b	b	b
b	b	a	b	b	b	b	b	b	b
b	b	a	b	b	b	b	b	b	b
b	b	a	b	b	b	b	b	b	b
b	b	a	b	b	b	b	b	b	b
b	b	a	b	b	b	b	b	b	b
b	b	a	b	b	b	b	b	b	b
b	b	a	b	b	b	b	b	b	b
b	b	a	-	b	b	b	b	b	b
-	b	a	b	b	b	-	b	b	b
b	b	a	b	b	b	b	b	b	b
b	b	a	b	b	b	b	b	b	b
b	b	a	b	b	b	b	b	b	b
b	b	a	b	b	b	b	b	b	b
b	b	a	b	b	b	b	b	b	b
b	b	a	b	b	b	b	b	b	b
b	b	a	b	b	b	b	b	b	-
b	b	b	b	b	b	b	b	b	b
b	-	b	b	b	b	b	b	b	b
b	-	b	-	b	b	b	b	b	-
b	b	b	b	b	b	b	b	b	b
b	b	b	b	b	b	b	b	b	b
-	b	b	b	b	b	b	b	b	b
b	b	b	-	b	b	-	b	b	b
b	a	b	b	b	b	b	b	b	b
b	a	b	b	b	b	b	b	b	b
-	a	b	b	b	b	b	b	b	b
-	a	-	b	b	b	b	b	b	b

b	a	b	-	b	b	b	b	b	b
b	a	-	b	b	b	b	b	b	b
b	a	b	b	b	b	b	b	b	b
a	b	b	b	a	a	b	b	a	a
a	b	b	b	a	a	b	b	a	a
a	b	b	b	a	a	b	b	b	a
a	b	b	b	a	a	b	b	b	a
a	b	b	b	a	a	b	b	b	a
a	a	b	b	a	a	b	b	b	b
a	a	b	b	b	a	b	b	b	-
a	a	b	-	b	a	b	b	b	b
a	a	b	b	b	a	b	b	b	-
a	-	b	b	b	a	b	b	b	b
a	a	b	a	b	a	-	a	b	b
a	a	b	a	b	a	b	a	b	b
a	a	b	a	b	b	b	a	b	b
a	a	b	a	b	b	b	a	b	b
a	a	b	a	b	b	b	a	b	b
a	a	b	a	b	b	b	a	b	b
a	a	b	a	b	b	b	a	b	b
a	a	b	-	b	b	b	a	b	b
a	a	b	a	a	b	b	a	b	b
a	a	b	a	a	b	b	a	b	b
a	a	-	a	a	b	b	a	-	-
a	a	b	a	a	b	-	a	b	b
a	a	b	a	a	b	b	a	b	b
a	a	b	a	a	b	b	a	b	b
a	a	b	a	a	b	b	a	b	a
a	a	b	a	a	b	b	a	b	a
a	a	b	a	a	b	b	a	b	a
a	a	b	a	a	b	b	a	b	a
-	a	b	a	a	b	b	a	b	a
a	a	b	a	a	b	b	a	b	a
a	b	b	a	a	b	b	a	b	b
a	b	b	a	a	b	b	a	b	b
a	b	b	a	a	b	b	a	b	b
a	b	-	a	a	b	b	a	b	b
a	b	b	a	a	b	b	a	b	b
a	b	b	a	a	b	a	a	b	b
a	b	b	-	a	b	a	a	b	b
a	b	b	-	a	b	a	a	a	b
a	b	b	a	a	b	a	a	a	b
a	b	b	-	a	b	a	a	a	b
a	b	b	a	a	b	a	a	a	b
a	b	b	a	a	b	a	a	a	b
a	b	b	a	a	b	a	a	a	b
-	b	b	a	a	b	a	a	a	a

a	b	b	a	a	b	a	a	a	a
a	b	b	a	a	b	a	a	a	a
-	b	b	b	-	b	a	a	a	a
a	b	b	b	a	b	a	a	a	a
b	a	b	a	a	b	b	a	b	a
b	a	b	a	a	b	b	a	b	a
b	a	b	a	a	b	b	a	b	a
b	a	b	a	a	b	b	a	b	a
b	a	b	a	a	b	b	a	b	a
b	a	b	a	a	b	b	a	b	a
-	a	b	a	a	b	b	a	b	a
b	b	b	a	a	b	b	a	b	a
b	b	b	a	a	b	b	b	b	-
b	b	b	a	a	b	b	b	b	a
b	b	b	a	a	b	b	b	b	-
b	b	b	a	a	b	b	b	b	a
b	b	b	a	a	b	b	b	b	a
b	b	b	a	a	b	b	b	b	a
b	b	b	a	a	b	b	b	b	a
b	b	b	a	a	b	b	b	b	a
b	-	b	a	a	b	b	b	b	a
b	b	b	b	a	b	b	b	b	a
b	b	b	a	a	b	b	b	b	a
b	b	b	a	a	b	b	b	b	a
b	b	b	a	a	b	b	b	b	a
b	b	b	a	b	b	a	b	a	a
b	b	b	a	b	b	a	b	a	a
b	b	b	a	b	b	a	b	a	a
b	a	-	a	b	b	a	b	a	a
b	b	b	a	b	b	a	b	a	a
b	b	b	a	b	b	a	b	a	a
b	b	b	a	b	b	a	b	a	a
b	b	b	a	a	b	a	b	a	a
b	b	b	a	a	-	a	b	a	a
b	b	b	a	a	b	a	b	a	a
b	b	b	a	a	b	a	b	a	a
b	b	b	a	a	b	a	b	a	a
b	b	b	a	a	b	a	b	a	a
b	b	b	a	-	b	a	b	a	-
b	b	b	-	a	b	a	b	a	a
b	b	a	a	a	b	a	b	a	a
b	b	-	a	a	b	b	b	a	a
b	b	a	a	a	b	b	b	a	a
b	b	a	a	a	b	b	b	a	a
b	b	a	a	a	b	b	b	a	a
a	a	a	a	a	b	b	a	b	a
-	a	-	-	a	b	b	a	b	-

a	a	a	a	a	b	b	a	b	a
a	a	a	a	a	b	b	a	b	a
a	a	a	a	a	b	b	a	b	a
a	a	a	a	a	b	b	a	b	a
a	a	a	a	a	b	b	a	b	a
b	a	a	a	a	b	b	a	b	a
a	a	a	a	a	b	b	a	b	a
a	a	a	a	a	b	b	a	b	a
a	a	a	a	a	b	b	a	b	a
a	a	a	a	a	b	b	a	b	a
a	a	a	a	b	b	b	a	b	a
a	a	a	a	b	b	b	a	b	a
a	a	a	a	b	b	b	a	b	a
a	a	a	a	b	b	b	a	b	a
a	a	a	a	b	b	b	a	b	a
a	a	a	-	b	b	b	a	b	-
-	a	a	a	b	b	-	a	b	a
a	a	-	a	b	b	a	a	b	-
-	a	a	a	b	b	a	a	b	a
b	a	a	a	b	b	a	b	b	a
b	a	a	a	b	b	a	b	b	a
b	-	a	a	b	b	b	b	b	a
b	a	a	a	b	b	a	b	b	a
b	a	a	a	b	b	a	b	b	a
b	a	-	a	b	b	a	b	b	a
-	a	a	a	b	b	a	b	b	a
b	a	a	b	b	b	b	b	b	a
b	a	a	b	b	b	b	b	b	a
-	a	a	-	b	b	b	b	b	a
b	a	a	b	b	b	b	b	b	a
b	a	a	b	b	b	b	b	b	a
b	a	a	b	a	b	b	b	b	a
b	a	-	b	b	b	b	b	b	a
b	b	a	-	a	b	b	b	b	a
b	b	a	-	b	-	b	b	b	a
-	b	a	-	b	b	-	b	b	a
b	b	a	b	b	b	b	b	b	a
b	b	a	b	b	b	b	b	b	a
b	b	a	b	b	b	b	b	b	a
b	b	a	-	b	b	b	b	b	a
b	b	a	b	b	a	b	b	b	a
b	b	a	b	b	a	b	b	b	a
b	-	a	-	b	a	b	b	b	a
b	b	a	b	b	a	b	b	b	a
b	b	a	b	b	a	b	b	a	b
b	b	a	b	b	a	b	b	a	b
b	b	a	b	b	a	b	b	a	b

b	b	a	b	b	a	b	b	a	b
b	b	a	b	b	a	b	a	a	b
b	b	a	a	b	a	b	a	a	-
b	-	a	b	b	a	-	a	a	b
b	b	a	b	b	a	b	a	a	-
b	b	a	b	b	a	b	a	a	b
b	b	a	b	b	a	b	a	a	b
b	-	a	b	b	a	b	a	a	b
-	b	a	-	b	a	b	a	a	b
b	b	a	b	b	a	b	a	a	b
b	b	a	b	b	a	b	a	a	b
b	b	a	b	b	a	b	a	a	b
b	b	a	b	b	a	b	a	a	b
b	-	a	b	b	a	-	a	a	b
b	-	a	b	b	a	-	a	a	b
b	b	a	b	a	a	b	a	a	b
b	b	a	b	a	a	b	a	a	b
b	b	a	b	a	a	b	a	a	b
b	b	a	b	a	a	b	a	a	b
-	-	a	b	a	a	b	a	a	b
b	b	a	a	b	a	b	a	a	b
b	b	-	a	b	a	b	a	a	b
b	b	a	-	b	a	b	a	a	b
b	b	a	a	b	a	b	a	a	b
b	b	a	a	b	a	b	a	a	b
b	b	a	a	b	a	b	a	a	b
b	b	a	a	b	a	b	a	a	b
b	b	a	a	b	a	b	a	a	b
b	b	a	a	b	a	b	b	a	b
b	b	a	a	b	a	b	b	a	b
b	b	a	a	b	a	b	b	a	b
b	b	a	a	b	a	b	b	a	b
b	a	a	a	b	a	b	b	a	b
b	a	a	a	b	a	b	b	a	a
b	a	a	a	b	a	b	b	a	a
b	a	a	a	b	a	b	b	a	a
b	a	a	-	b	a	b	b	a	a
b	a	a	a	b	a	b	b	a	a
b	-	a	a	b	a	b	b	a	a
b	a	a	a	b	b	b	b	a	a
b	a	a	a	b	b	b	b	a	a
b	a	a	a	b	b	b	b	a	a
-	a	b	a	b	b	a	a	a	a
b	a	b	a	b	b	a	a	a	a
b	a	b	b	b	b	-	a	a	a
b	a	b	-	b	b	a	a	a	a
b	a	b	b	b	b	a	a	a	a

b	-	b	b	b	b	a	a	a	a
b	a	b	a	b	b	a	a	a	a
b	a	b	b	b	b	a	a	a	b
-	a	b	b	b	b	a	a	a	b
b	a	b	b	b	b	a	a	a	b
b	a	b	b	b	b	a	a	a	b
b	a	b	b	b	b	a	a	a	b
b	a	-	b	b	b	a	a	a	b
b	a	a	a	a	a	a	b	b	b
b	a	-	a	a	a	a	b	b	b
b	a	a	a	a	a	a	b	b	b
b	a	a	a	b	a	a	b	b	b
b	a	a	a	b	a	a	b	b	b
b	a	a	a	b	a	a	b	b	b
b	a	a	a	b	a	a	b	b	b
b	a	a	a	b	a	a	b	b	b
b	-	a	a	b	a	a	b	b	b
b	a	a	a	b	a	a	b	b	-
b	-	-	a	b	a	a	b	b	b
b	a	a	a	b	a	a	b	b	a
b	a	a	a	b	a	a	b	b	-
b	a	a	a	b	a	a	b	b	-
b	a	a	a	b	a	a	b	b	b
a	a	a	a	b	a	a	b	b	a
a	a	a	a	b	a	a	b	b	a
a	a	a	a	b	a	a	b	b	a
a	a	a	a	b	a	a	b	b	a
a	a	-	a	b	a	a	b	b	a
a	a	a	a	b	a	a	b	b	a
a	b	a	b	b	a	a	b	a	a
a	b	a	b	b	a	a	a	a	a
a	b	a	b	b	a	a	a	a	a
a	-	a	a	-	a	a	a	a	a
a	b	a	b	b	a	a	a	a	-
a	b	a	b	b	a	a	a	a	a
b	b	a	b	b	a	a	a	a	a
b	b	b	b	b	a	a	a	a	a
b	b	b	b	b	a	a	a	a	a
b	b	b	b	b	a	a	a	a	a
b	b	b	a	b	a	a	a	a	a
b	b	b	b	b	a	a	a	a	a
b	b	-	b	b	a	a	a	a	a
b	b	b	b	b	a	a	a	a	a
-	a	b	b	b	a	b	a	a	a
b	a	b	b	b	a	b	a	a	a
b	-	b	b	b	a	b	a	a	a
b	-	b	b	b	a	b	a	a	a

b	a	b	a	b	a	b	a	a	a
b	a	b	b	b	a	a	a	a	a
b	a	b	b	b	a	a	a	a	a
b	a	b	-	b	a	a	a	a	a
b	a	b	b	b	a	a	a	a	a
a	a	b	a	b	a	a	a	a	a
-	a	b	-	b	a	b	a	a	a
a	a	b	a	b	a	b	a	a	a
a	a	b	-	b	a	b	b	a	a
a	a	-	a	b	a	b	b	a	a
a	a	b	a	-	a	b	b	a	a
a	a	b	a	b	a	b	b	a	a
b	a	b	b	b	-	a	a	b	b
b	a	b	b	b	a	a	a	b	b
b	a	b	b	b	a	a	a	b	b
a	a	b	b	-	a	a	a	b	b
b	a	b	b	b	a	a	a	b	b
a	a	b	-	b	a	a	a	b	b
a	a	b	b	b	a	a	a	a	b
a	a	b	b	b	a	a	a	a	b
a	a	b	a	a	a	a	a	a	b
a	a	b	b	a	a	a	a	a	b
a	-	b	a	a	a	a	a	a	-
a	a	b	a	a	a	a	a	a	b
a	a	b	a	-	a	a	a	a	b
a	a	b	a	a	a	a	a	a	b
a	a	b	a	a	a	a	a	a	b
a	a	b	a	a	b	a	a	a	b
a	a	b	a	a	b	a	a	a	b
a	a	b	a	a	b	a	a	a	b
a	a	b	a	a	b	a	a	a	b
a	a	b	a	a	b	a	a	a	b
a	a	b	a	a	b	a	a	a	b
a	a	b	a	a	b	a	a	a	b
a	a	b	a	a	b	a	a	a	b
a	a	b	a	a	b	b	a	a	b
a	a	b	a	a	b	b	a	a	b
a	a	b	a	a	b	b	a	a	b
a	a	b	a	a	b	b	a	a	b
a	a	b	a	a	b	b	a	a	b
a	-	b	a	a	-	b	b	a	b
a	a	b	a	a	a	b	b	a	b
a	a	b	a	a	a	b	b	a	b
a	a	b	a	a	a	b	b	a	b
a	a	b	a	a	a	b	b	a	b
a	a	b	a	a	a	b	b	a	b
a	a	b	a	a	a	b	b	a	-

a	a	b	a	a	a	b	b	a	b
a	a	b	a	a	a	b	b	a	b
a	a	a	a	b	b	b	a	a	b
a	a	a	-	b	b	b	a	a	b
a	a	a	a	b	b	b	a	b	b
-	a	a	a	b	b	b	a	b	b
a	a	a	a	b	b	b	a	b	b
a	a	a	-	b	b	b	a	b	b
b	-	b	-	a	b	b	a	b	a
b	b	b	a	a	b	b	a	b	a
b	b	b	a	a	b	b	a	b	a
b	b	-	a	a	b	b	a	b	a
b	b	b	a	a	b	b	a	b	a
b	-	a	a	a	b	b	a	b	a
b	b	a	a	a	b	b	a	b	-
b	b	-	a	a	b	b	a	b	a
b	b	a	a	a	b	b	a	b	b
-	b	a	a	a	b	b	a	b	b
b	b	a	a	a	b	b	a	b	b
b	b	a	a	a	b	b	a	b	b
b	b	a	a	a	b	b	a	b	b
b	-	a	a	a	b	b	a	b	b
b	b	a	a	a	b	b	a	b	b
b	b	a	-	a	b	b	a	b	b
a	b	a	a	a	b	-	a	b	b
-	b	a	a	a	b	b	-	b	-
a	b	a	a	a	b	b	a	b	-
a	b	a	a	a	b	b	a	b	b
a	b	a	a	a	b	b	a	b	b
a	a	a	a	-	b	b	-	b	b
a	a	a	a	a	b	b	a	b	b
a	a	a	a	a	b	b	a	b	b
a	a	a	a	a	b	b	a	b	b
a	a	a	a	a	b	b	a	b	b
a	a	a	a	a	b	b	a	b	b
a	a	a	a	a	b	b	a	b	b
-	a	a	a	a	b	b	a	b	b
a	a	a	a	a	b	b	a	b	b
a	a	a	a	a	b	b	a	a	b
a	a	a	a	a	b	b	a	a	-
a	a	a	a	a	b	b	a	a	b
a	a	a	a	a	b	b	a	a	b
a	a	a	a	a	b	b	a	a	b
a	a	-	a	a	b	b	b	a	b
a	a	a	a	a	b	b	b	a	b
a	a	a	a	a	b	b	b	a	b
a	a	a	a	a	b	a	b	a	b

a	a	a	a	a	b	a	b	a	b
a	a	a	a	a	a	a	b	a	b
a	a	a	a	a	b	a	b	a	b
a	a	a	a	a	a	a	b	a	b
a	a	a	a	a	a	a	b	a	b
a	a	b	a	a	b	a	b	a	-
a	a	b	a	a	b	a	b	a	b
a	a	-	a	a	b	a	b	a	b
a	a	b	a	a	b	a	b	a	b
a	a	b	a	a	b	a	b	a	b
-	a	b	-	a	b	-	b	a	b
a	a	b	a	a	b	a	b	a	b
a	a	b	a	a	b	a	b	a	b
a	a	b	a	a	b	a	b	a	b
a	a	b	a	a	b	a	b	a	b
a	a	b	a	a	b	a	b	a	a
a	a	b	a	a	b	a	b	a	a
a	a	b	a	a	b	a	b	a	a
a	a	b	a	a	a	a	a	a	a
a	a	b	a	a	a	a	a	a	a
b	b	b	b	b	a	b	b	b	b
b	b	b	b	b	a	b	b	b	b
b	b	b	b	b	a	b	b	b	b
b	b	b	b	b	a	b	b	b	b
b	b	b	b	b	a	b	b	b	b
b	b	b	b	b	a	b	b	b	b
b	b	b	b	b	a	b	b	b	-
b	b	b	b	b	a	b	b	b	b
b	b	-	b	b	a	b	b	b	b
b	b	b	b	b	a	b	b	b	b
b	b	-	b	b	a	b	b	b	b
b	b	b	b	b	a	b	b	b	b
b	b	b	b	b	a	b	b	b	-
b	b	b	b	b	a	b	b	b	a
b	b	b	b	b	a	b	b	b	a
b	b	b	-	b	a	b	b	b	a
b	b	b	b	b	a	b	b	b	a
b	b	b	b	b	a	b	b	b	a
b	b	b	b	b	a	b	b	b	a
b	b	a	b	b	a	b	a	b	a
b	b	a	b	b	a	b	a	b	a
b	b	a	b	b	a	b	a	b	a
-	b	a	b	b	a	b	a	b	a
b	b	a	b	b	a	b	a	b	a
a	b	a	b	b	a	b	a	b	a
a	b	a	b	b	a	b	a	b	a
a	b	a	b	b	a	b	a	b	a

a	b	a	b	b	a	b	a	b	a
a	b	a	b	b	a	b	a	b	a
a	b	a	b	b	a	b	a	b	a
a	b	a	b	a	a	b	a	b	a
a	b	a	b	a	a	b	a	b	a
a	b	a	b	a	a	b	a	b	a
-	b	a	b	a	a	b	a	b	a
a	b	a	b	a	a	b	a	b	a
a	b	a	b	a	a	b	a	b	a
a	b	a	b	a	a	b	a	b	a
a	a	a	b	a	a	b	a	b	a
a	a	a	b	a	a	b	a	b	a
a	a	a	b	a	a	b	a	b	a
a	-	a	b	a	a	b	a	b	a
a	a	a	b	a	a	b	a	b	a
a	a	a	b	b	a	b	a	b	a
-	a	a	b	b	a	b	a	b	a
a	a	a	b	b	a	b	a	b	a
a	a	a	b	b	a	b	a	b	a
b	a	a	b	b	a	b	a	b	a
-	a	a	b	b	a	b	a	b	a
b	a	a	b	b	a	b	a	b	a
b	a	a	b	b	a	b	a	b	a
b	a	a	b	b	a	b	a	b	a
b	a	a	b	b	a	b	a	b	a
b	a	-	b	b	a	b	b	b	a
b	a	b	b	b	a	b	b	b	a
b	a	b	b	b	a	b	b	b	a
-	a	b	b	b	a	b	a	b	a
b	a	b	b	b	a	b	a	b	a
b	a	b	b	b	a	b	a	b	a
b	a	b	b	b	a	b	a	b	a
b	a	b	-	b	a	b	a	b	a
b	a	b	b	b	a	b	a	b	a
b	a	b	b	-	a	b	a	b	a
a	a	a	b	b	a	a	a	b	a
a	a	-	b	b	a	a	a	b	a
a	a	a	b	b	a	a	a	b	-
a	a	a	b	-	a	a	a	b	a
a	a	a	b	b	a	a	a	b	a
a	a	a	b	b	a	a	a	b	a
a	b	a	b	b	a	a	a	b	a
a	b	a	b	b	a	a	b	b	a
a	b	a	b	b	a	a	b	b	a
a	b	a	b	b	a	a	b	a	a
a	b	a	b	b	a	a	b	a	a
a	a	a	b	b	a	a	b	a	a

a	b	a	-	b	a	a	b	a	a
a	b	a	b	b	a	a	b	a	a
a	b	-	b	b	a	a	b	a	a
a	b	a	-	b	a	a	b	a	a
a	b	a	b	b	a	a	b	a	a
a	b	b	b	b	a	a	b	a	a
a	b	b	b	b	a	a	b	a	a
a	b	b	b	b	a	a	b	a	-
a	b	b	b	b	a	a	b	a	a
a	b	b	b	b	a	a	b	a	a
-	b	b	a	b	a	a	b	a	-
a	b	b	b	b	a	a	b	a	-
a	b	b	b	b	a	a	b	a	a
a	b	-	a	b	a	a	b	a	-
a	b	b	a	b	a	a	b	a	-
a	b	b	a	b	a	a	b	a	a
a	b	b	a	b	a	a	b	a	a
a	-	b	a	b	a	a	b	a	a
-	b	b	a	b	a	a	b	a	a
a	b	b	a	b	a	a	b	a	a
a	b	b	b	b	a	a	b	a	a
a	b	b	a	a	a	a	b	a	a
a	b	b	a	b	a	a	b	a	-
a	b	-	a	b	a	a	b	a	a
a	b	-	a	b	a	a	b	a	a
a	b	b	a	b	a	a	b	a	a
a	b	b	a	b	a	a	b	a	a
a	-	b	a	b	a	a	b	a	a
a	b	b	a	b	a	a	b	a	-
a	b	b	a	b	a	a	b	a	-
a	b	b	a	b	a	a	b	a	a
a	-	b	a	b	a	a	b	a	a
a	b	b	a	b	a	a	a	a	a
a	b	b	a	b	a	a	a	a	a
a	a	b	a	b	a	a	a	a	a
a	a	b	a	b	a	a	a	a	a
a	a	b	a	b	a	a	a	a	a
a	a	b	a	b	a	a	a	a	a
a	a	b	-	b	a	a	a	a	a
a	a	b	a	b	a	a	a	a	a
a	a	b	a	b	a	a	a	a	a
a	a	b	a	-	a	a	a	a	-
a	a	b	-	b	a	a	a	a	a
a	a	b	a	b	a	a	a	a	a
a	a	b	a	b	a	a	a	a	a
a	a	b	a	b	a	a	a	a	-
a	b	b	a	b	b	b	a	a	b

a	b	b	a	b	b	b	a	a	b
a	b	b	a	b	b	b	a	a	b
a	b	b	a	b	b	b	a	a	b
a	b	b	a	b	b	b	a	a	b
a	b	b	a	b	a	b	a	a	b
a	b	b	a	b	a	b	a	a	b
a	b	b	a	b	a	b	a	a	b
a	b	b	a	b	a	b	a	a	b
a	b	b	a	b	a	b	a	a	b
a	b	b	a	b	a	b	a	a	b
a	b	b	a	b	a	b	a	a	b
a	b	b	a	b	a	b	a	a	b
a	b	b	a	b	a	b	a	a	b
a	b	b	a	b	a	b	a	a	b
a	b	b	a	b	a	b	a	a	b
a	b	b	a	b	a	b	a	a	b
a	b	b	a	b	a	b	a	a	b
a	b	b	a	b	a	b	a	a	b
a	a	b	a	b	a	b	a	a	b
a	a	b	a	b	a	b	a	a	b
a	a	b	a	b	a	b	a	a	b
a	a	b	a	b	a	b	a	a	b
a	a	b	a	b	a	b	a	a	b
a	a	b	a	b	a	b	a	a	b
a	a	b	a	b	a	b	b	a	b
a	a	b	a	b	a	b	b	a	b
a	a	b	a	b	a	b	b	a	b
a	a	b	a	b	a	b	b	a	b
a	a	b	a	b	a	b	b	a	b
a	a	b	a	b	a	b	b	a	b
a	a	b	a	b	a	b	b	a	b
b	a	b	a	b	a	a	b	a	b
b	a	b	a	b	a	a	b	a	b
b	a	b	a	b	a	a	b	a	b
b	a	b	a	b	a	a	b	a	b
b	a	b	a	b	a	a	b	a	b
b	a	b	a	b	a	a	b	a	b
b	b	b	a	b	a	a	b	b	b
b	a	b	a	b	a	a	b	a	b
b	b	b	a	b	a	a	b	b	b
a	a	a	-	a	b	b	a	b	-
a	b	a	a	b	b	b	b	a	a
b	b	a	a	b	b	b	b	a	a
a	b	a	a	b	b	b	b	a	a
a	b	a	a	b	b	b	b	a	a
a	b	a	a	b	b	b	b	a	a
a	b	a	a	b	b	b	b	a	a

a	b	a	b	b	b	b	b	a	a
a	b	a	b	b	b	b	b	a	a
a	b	a	b	b	b	b	b	a	a
a	b	a	b	b	b	b	b	a	a
a	b	a	b	b	b	b	b	a	a

YW121	YW122	YW123	YW124	YW125	YW126	YW127	YW128	YW129	YW130
a	-	b	a	b	b	a	a	a	a
b	b	b	a	b	b	a	b	a	a
b	b	-	a	b	b	a	b	a	a
b	b	a	a	-	b	a	b	a	a
b	b	b	a	b	b	a	b	a	a
b	b	-	a	b	b	a	b	a	a
b	b	b	a	b	b	a	b	a	a
b	b	b	a	b	b	a	b	a	a
b	b	b	-	b	b	a	b	a	a
a	b	b	a	b	b	a	b	a	a
a	b	b	a	b	b	a	b	a	a
a	b	b	a	b	b	a	b	a	a
a	b	a	a	b	b	a	b	a	a
a	b	b	a	b	b	a	b	a	a
a	b	b	a	b	b	a	b	a	a
a	b	b	a	b	b	a	b	a	a
a	b	b	a	b	b	-	b	a	a
a	b	b	a	b	b	a	b	a	a
a	b	b	a	b	b	a	b	a	a
a	b	b	a	b	b	a	b	a	a
a	b	-	a	b	b	a	b	a	a
a	b	b	a	b	b	a	b	a	a
a	b	b	a	b	b	a	b	b	a
a	b	b	-	b	b	a	b	b	a
a	b	b	a	b	b	a	b	b	a
a	b	b	a	-	b	a	b	b	a
a	b	b	a	b	b	a	b	b	a
a	b	b	a	b	b	a	b	b	a
a	b	b	a	b	b	a	b	b	a
-	b	b	a	b	b	a	b	b	a
a	b	b	a	b	b	a	b	b	a
a	b	b	a	b	b	a	b	b	a
a	b	b	a	b	b	a	b	b	a
a	b	-	a	b	b	a	a	b	b
a	b	b	-	b	b	a	a	b	b
a	b	b	a	b	b	a	a	b	b
a	b	b	a	b	b	a	a	b	b
a	b	b	a	b	b	a	a	b	b
a	b	b	a	b	b	a	a	b	b
a	b	b	a	b	b	a	a	b	b
a	b	b	a	b	b	a	a	b	b
a	b	b	a	b	b	a	a	b	b
a	b	b	a	b	b	a	a	b	b
a	b	b	a	b	b	a	a	b	b
a	b	b	a	b	b	a	a	b	b

a	b	b	a	b	b	a	a	b	b
a	b	a	a	b	b	a	a	b	b
a	b	-	a	b	b	a	a	b	b
a	b	b	a	b	b	a	a	b	b
a	b	b	-	b	b	a	a	b	b
a	b	b	a	b	b	a	a	b	b
-	b	b	a	b	b	a	a	b	b
a	b	b	a	b	b	a	a	b	b
a	b	b	a	b	b	a	a	b	b
a	b	b	a	b	b	a	a	b	b
a	b	b	a	b	b	a	a	a	b
a	b	b	a	b	b	a	a	a	b
a	b	b	a	-	-	a	a	a	b
a	b	b	a	b	b	a	a	a	b
a	b	b	-	b	b	b	a	a	b
a	b	b	a	b	b	b	a	a	b
a	b	b	a	b	b	b	a	a	b
a	b	b	a	-	b	-	a	a	b
a	b	b	a	b	b	b	a	a	b
a	b	b	a	b	b	b	a	a	b
-	b	b	a	b	b	b	a	a	b
a	b	b	a	b	b	b	a	a	b
a	b	a	a	b	b	b	a	a	b
a	b	a	a	-	b	b	a	a	b
a	b	a	-	b	b	b	a	a	b
a	b	a	a	b	b	b	a	a	b
a	b	a	a	a	b	b	a	a	b
a	b	-	a	a	b	b	a	a	b
a	b	b	a	a	b	b	a	a	b
a	b	-	a	a	b	b	a	a	b
a	-	a	a	a	b	b	a	a	b
a	b	b	a	a	b	b	a	a	b
a	b	a	a	a	b	b	a	a	b
a	-	a	a	a	b	b	a	a	b
b	b	a	a	a	b	b	a	a	b
b	b	a	a	a	b	b	a	a	b
b	b	a	a	a	b	b	a	a	b
a	b	a	a	a	-	b	a	a	b
a	b	a	a	a	b	b	a	a	b
b	b	a	b	a	a	b	b	a	a
b	b	a	b	a	b	b	b	a	a
b	b	a	-	a	b	-	b	a	a
b	b	a	b	a	b	b	b	a	a
b	b	a	b	a	b	b	b	a	a
b	b	a	b	a	b	b	b	a	a
b	b	a	b	a	b	b	b	a	a
b	b	a	-	a	b	b	b	a	a

b	b	a	b	a	b	b	b	a	a
b	b	a	b	a	b	b	b	a	a
-	b	a	b	a	-	b	b	a	a
b	b	a	b	a	b	b	b	a	a
-	b	a	b	a	b	b	b	a	a
b	b	a	b	a	b	-	b	a	a
b	b	a	b	a	b	b	b	a	a
b	b	a	b	a	a	b	b	a	a
b	-	a	b	a	a	b	b	a	a
b	-	a	b	a	a	b	b	a	a
b	a	a	b	a	a	b	b	a	a
b	a	a	b	-	a	b	b	a	a
b	-	-	b	a	a	b	b	a	a
a	a	a	b	a	a	a	b	a	a
a	a	a	b	a	a	a	b	a	a
a	a	a	b	a	a	a	b	a	a
a	a	a	b	a	a	a	b	a	a
b	a	-	b	a	a	a	b	a	a
a	a	a	b	a	a	a	b	a	a
a	a	a	b	a	a	a	b	a	a
a	a	a	b	-	-	a	b	a	a
a	a	a	b	a	a	a	b	a	a
b	a	b	b	a	a	a	b	a	a
b	a	a	b	a	a	a	b	a	a
b	a	b	b	a	b	a	b	a	a
b	a	a	b	a	b	a	b	a	a
b	a	a	b	a	b	a	b	a	a
b	a	a	b	a	b	a	b	a	a
b	b	b	b	a	b	a	b	a	a
b	a	a	b	a	b	a	b	a	a
-	a	a	b	a	a	a	b	a	a
-	a	a	b	a	a	a	b	a	b
b	a	a	b	a	a	a	b	a	b
b	a	a	b	a	a	a	b	a	b
b	a	a	-	a	a	a	b	a	b
b	a	a	b	a	a	a	b	a	b
b	a	a	b	-	a	a	b	a	b
b	a	-	b	a	a	a	b	a	b
b	a	a	b	a	a	a	b	a	b
b	a	a	b	a	a	a	b	a	b
b	a	a	b	a	a	a	b	a	b
b	a	a	b	a	a	a	b	a	b
b	a	a	b	a	a	a	a	a	b
b	a	a	b	a	a	a	a	a	b
b	a	a	b	a	a	a	a	a	b
b	a	a	b	a	a	a	a	a	b
b	a	a	b	a	a	a	a	a	b

b	a	a	b	a	a	a	a	a	b
b	a	a	b	a	a	a	a	a	b
b	b	a	b	a	a	a	a	a	b
b	b	a	b	a	a	a	a	a	b
b	b	a	-	a	a	a	a	a	b
b	b	a	b	a	a	a	a	a	b
b	b	a	b	a	a	a	a	a	b
b	b	a	b	a	a	a	a	a	b
b	b	a	b	a	a	a	a	a	b
b	b	a	b	a	a	-	a	a	b
b	b	a	b	a	a	a	a	a	b
a	b	a	b	a	a	a	a	a	b
a	b	a	b	a	a	a	a	a	b
a	-	a	-	a	a	a	a	a	b
a	b	a	b	a	a	a	a	a	b
a	b	a	b	a	a	a	a	a	b
a	b	a	b	a	a	a	a	a	b
a	b	a	b	a	a	a	a	a	b
a	b	a	b	a	a	a	a	a	b
a	b	a	b	a	a	a	a	a	b
b	b	a	b	a	a	a	b	a	b
b	b	a	b	a	a	a	b	a	b
-	b	b	b	a	a	a	b	b	a
b	b	b	b	a	a	a	b	b	a
a	a	b	a	a	a	a	a	a	a
a	a	b	a	a	a	a	a	a	a
a	a	b	a	a	a	a	a	a	a
a	a	b	a	a	a	a	a	a	a
a	a	b	a	a	a	a	a	a	a
b	a	b	a	a	a	a	a	a	a
b	a	b	-	a	a	a	a	a	a
b	a	b	a	a	a	a	a	a	a
b	a	-	a	a	a	a	a	a	a
b	a	b	a	a	a	a	a	a	a
b	a	-	a	a	a	a	a	a	a
b	a	a	a	a	a	a	a	a	a
b	a	a	a	a	a	a	a	a	a
b	a	a	a	a	a	a	a	a	a
b	a	a	a	-	a	a	a	a	a
b	a	a	a	a	a	a	a	a	a
b	a	a	a	a	a	a	a	a	a
b	a	a	a	a	a	a	a	a	a
-	a	a	a	b	a	b	a	a	a
b	a	a	a	b	a	b	a	a	a
-	a	a	a	b	a	a	a	a	a
b	-	a	a	b	a	b	a	a	a
b	b	a	a	b	a	b	a	a	a

b	b	a	a	b	a	b	a	a	a
b	b	a	a	b	a	b	a	a	-
b	b	a	a	b	a	b	a	a	a
b	b	a	a	b	a	b	-	a	a
b	b	-	a	b	-	b	a	a	a
b	b	a	a	b	a	b	a	a	a
b	b	a	a	b	a	b	a	a	a
b	b	a	a	b	a	b	a	a	a
b	-	a	a	b	a	b	a	a	a
b	b	b	a	b	a	b	a	a	a
a	b	a	a	b	a	b	a	a	a
b	b	a	a	b	a	b	a	a	a
a	a	b	b	b	b	b	a	a	b
a	a	-	b	b	b	b	a	a	b
a	a	b	b	b	b	b	a	a	b
a	a	b	b	b	b	b	a	a	b
a	a	b	b	b	b	b	a	b	b
a	a	b	b	b	b	b	a	b	b
a	a	b	b	b	b	a	a	b	b
a	a	b	b	b	b	a	a	b	b
a	-	b	b	b	b	a	a	b	b
-	a	b	b	b	b	a	-	b	b
-	a	b	b	b	b	-	a	b	b
a	a	b	b	b	b	a	a	b	b
a	a	b	b	b	b	a	a	b	b
a	a	b	b	b	b	a	a	b	b
a	a	b	a	b	b	a	a	b	b
a	a	b	a	b	b	a	a	b	b
-	a	b	a	b	b	a	a	b	b
a	a	b	a	b	b	a	a	b	b
a	a	b	a	b	b	a	a	b	b
a	a	b	-	b	b	a	b	b	b
a	a	b	a	b	b	a	b	b	b
a	a	b	a	b	b	a	b	b	b
a	a	b	a	b	b	a	-	b	b
a	a	b	a	b	b	a	b	b	a
a	a	b	-	a	b	a	b	b	a
a	a	b	a	a	b	a	b	b	a
a	a	b	a	a	b	a	b	b	a
a	a	b	a	a	b	-	b	b	a
a	a	b	a	a	b	a	b	b	a
a	a	b	a	a	b	a	b	b	a
a	a	b	a	a	b	a	b	b	a
a	a	b	a	a	b	a	b	b	a
a	a	b	a	a	b	a	b	b	a
a	a	a	a	a	b	a	b	b	a
a	-	b	a	a	b	a	b	b	a

a	a	b	a	a	b	a	b	b	a
a	a	b	a	a	-	a	b	b	a
a	a	b	a	a	b	a	b	b	a
-	a	b	a	a	b	b	b	b	a
a	b	b	a	a	b	b	b	b	a
a	b	-	a	a	b	b	b	b	a
a	b	a	a	a	b	b	b	b	a
a	b	a	a	a	b	b	b	b	a
a	b	a	a	a	b	b	b	b	-
a	b	a	a	a	b	b	b	b	a
a	b	a	a	a	b	b	b	b	a
a	b	a	a	a	b	b	b	b	a
a	b	a	a	a	b	b	b	b	a
-	b	a	a	a	b	b	b	b	a
a	b	a	a	a	b	b	b	b	a
b	b	a	a	a	b	b	b	b	a
b	b	a	a	a	b	b	b	b	a
b	b	-	a	-	b	b	b	b	a
b	b	a	a	a	b	-	-	b	a
b	b	a	a	a	b	a	b	b	a
b	b	a	a	a	b	a	b	b	a
b	b	a	a	a	b	a	b	b	a
b	b	a	a	a	b	a	a	b	a
b	b	a	a	a	b	a	a	b	a
b	b	b	a	a	b	a	a	b	a
b	b	b	a	a	b	a	a	b	a
b	b	b	a	a	b	a	a	b	a
b	b	b	a	a	b	a	a	b	a
b	-	a	a	a	b	a	a	b	a
b	-	b	a	a	b	a	a	b	a
a	b	b	a	a	b	b	a	b	a
a	b	a	a	a	b	a	a	b	a
a	b	b	a	a	b	a	a	b	a
a	b	b	a	a	b	a	a	b	a
a	b	b	a	a	b	a	a	b	a
a	b	b	a	a	b	a	a	b	a
a	b	b	a	a	b	a	a	b	a
a	b	b	a	a	b	a	a	b	a
a	b	b	a	a	b	a	a	b	a
a	b	b	a	a	b	a	a	b	a
a	b	b	a	a	b	a	a	b	a
a	b	b	a	a	b	a	a	b	a
a	b	-	a	a	b	a	a	b	a
a	b	b	a	a	b	a	a	b	a
a	b	b	a	a	b	a	a	b	a
a	b	b	a	a	b	a	a	b	a
a	b	b	a	a	b	a	a	b	a

a	b	b	a	a	b	a	a	b	a
a	b	b	a	a	b	a	a	b	a
a	b	b	a	b	a	a	a	b	a
a	b	b	a	a	b	a	a	b	a
a	b	b	a	a	b	a	a	b	a
a	b	b	a	a	b	a	a	b	a
-	b	b	a	a	b	a	a	b	a
a	b	b	a	a	b	a	a	b	a
a	b	b	a	a	b	a	a	b	a
a	b	b	a	a	b	a	a	b	a
a	b	b	a	a	b	a	a	b	a
a	b	b	a	a	b	a	a	a	a
a	b	b	a	a	b	a	a	a	a
a	b	b	a	a	b	a	a	a	a
a	b	b	a	a	b	a	a	a	a
a	b	b	a	a	b	a	a	a	a
a	b	b	a	a	b	a	a	a	a
a	b	b	a	a	b	a	a	a	a
a	b	b	a	a	b	a	a	a	a
a	b	b	a	a	-	a	a	a	a
-	b	b	a	a	b	a	a	a	a
a	b	b	-	-	b	a	a	a	a
a	b	b	a	a	b	a	a	a	a
a	b	b	a	-	b	a	a	a	a
a	b	b	a	a	b	a	a	a	a
a	b	a	a	a	b	a	a	a	a
a	b	b	a	a	b	a	a	a	a
a	b	b	-	a	b	a	a	a	a
a	-	b	a	a	b	a	a	a	a
a	b	b	a	a	b	a	a	a	a
a	-	b	a	a	b	a	a	a	a
a	b	b	a	a	-	a	a	a	a
a	-	b	a	a	b	a	a	a	a
a	b	b	a	a	b	a	a	a	a
a	b	-	a	a	b	a	a	a	a
a	b	-	a	a	b	a	a	a	a
a	b	b	-	a	b	a	a	a	a
-	b	b	a	-	b	a	a	a	a
a	a	a	b	b	b	b	a	a	a
a	a	a	b	b	b	b	a	a	a
a	a	a	b	b	b	b	a	a	a
a	a	a	b	b	b	b	a	a	a
-	-	-	b	b	b	b	a	a	a
a	a	a	b	b	b	b	a	a	-
a	a	a	b	b	b	b	a	a	a
a	-	a	b	b	b	b	-	a	a
a	a	a	b	-	b	b	a	a	a

-	a	-	b	b	b	b	a	a	a
a	a	a	b	-	b	b	a	b	a
a	a	a	b	b	b	b	a	b	a
-	a	b	b	b	b	b	a	b	a
a	a	a	b	-	-	b	a	b	a
a	a	-	b	b	b	b	a	b	a
a	a	a	b	b	a	b	a	b	a
a	a	a	b	-	a	b	a	b	a
-	a	a	b	b	a	b	a	b	a
a	a	a	b	b	a	b	a	b	a
a	a	a	b	b	a	b	a	b	a
a	a	a	b	b	a	b	a	b	a
a	a	a	b	b	-	b	a	b	a
a	a	a	b	b	a	b	a	b	a
a	-	a	b	b	a	b	a	b	a
a	a	a	b	b	a	b	a	b	a
a	-	b	b	b	a	b	a	b	a
a	a	a	b	b	a	b	a	b	a
a	a	-	b	b	a	b	a	b	a
a	a	a	-	b	a	b	a	b	a
a	a	a	b	b	a	b	a	b	a
a	a	a	-	-	a	b	a	b	a
a	a	a	b	b	a	b	a	b	a
a	a	a	b	b	a	b	a	b	a
a	a	a	b	b	a	b	a	b	a
a	a	a	b	b	a	b	a	b	a
a	a	a	-	b	a	b	a	b	a
a	a	a	b	b	a	a	a	b	a
a	a	a	b	b	a	b	a	b	a
a	a	a	b	b	a	a	a	b	a
a	a	a	b	b	a	a	a	b	a
-	a	-	b	b	a	a	a	b	a
a	a	-	b	b	a	a	a	b	a
a	a	a	b	b	a	a	a	b	a
-	a	a	b	b	a	a	a	b	a
a	a	a	b	b	a	a	a	-	a
a	a	a	b	b	a	a	a	b	a
a	a	a	b	b	a	a	a	b	a
a	a	-	b	b	a	a	a	b	a
a	a	a	b	b	a	a	a	b	a
a	a	a	b	b	a	a	a	b	a
a	a	a	b	b	a	a	a	b	a
a	a	a	b	b	a	a	a	b	a
a	a	a	b	b	a	a	a	b	a
-	a	a	b	b	a	a	a	b	a
a	a	a	b	b	a	a	a	b	a
a	a	a	b	b	a	a	a	b	a

a	a	a	b	b	a	a	a	b	a
a	a	a	b	b	a	a	a	b	a
a	a	a	b	b	a	a	a	b	a
a	a	a	b	b	a	a	a	b	a
a	a	a	b	b	a	a	a	b	a
a	a	b	b	b	a	a	a	b	a
a	a	b	b	b	a	a	a	b	a
a	a	b	b	b	a	a	a	b	a
a	a	b	-	-	a	a	a	b	a
a	a	-	b	-	a	a	a	b	a
a	a	-	b	b	a	a	a	b	a
a	a	b	b	b	a	a	a	b	a
-	-	b	b	b	a	a	a	b	a
a	a	b	b	b	a	a	a	b	a
a	a	b	b	b	a	a	a	b	a
a	a	b	b	b	a	a	a	b	a
a	a	b	b	a	a	b	a	b	b
a	a	b	b	-	a	b	a	b	b
a	a	b	b	a	a	b	a	b	b
a	a	b	b	a	a	b	a	b	b
a	a	b	b	a	a	b	a	b	a
a	a	b	b	a	a	b	a	b	a
a	a	b	b	a	a	b	a	b	a
a	a	b	b	-	a	b	a	b	a
a	a	b	b	a	a	b	a	b	a
a	a	b	b	a	a	b	a	b	a
a	a	b	-	a	a	b	a	b	a
a	a	b	b	a	a	b	a	b	a
a	a	b	b	a	a	b	a	b	a
a	a	b	b	a	a	b	a	b	a
a	a	-	b	a	a	b	a	b	a
a	a	b	b	a	a	b	a	b	a
a	a	b	b	a	a	b	a	b	a
a	a	b	b	a	a	b	a	b	b
a	b	b	b	a	a	b	a	b	b
a	b	-	b	a	a	b	a	b	b
a	-	b	b	a	a	b	a	b	b
-	b	b	b	-	a	b	a	b	b
a	-	b	b	a	a	b	a	b	b
a	b	a	b	a	a	b	a	b	b
b	a	b	b	a	a	a	a	b	b
b	a	b	b	a	a	a	a	b	b
b	a	a	b	a	a	a	a	b	b
b	a	a	-	a	a	a	a	b	b
b	a	a	b	a	a	a	a	b	b
b	a	a	b	a	a	a	a	b	b
-	a	a	b	a	a	a	a	b	b

b	a	a	-	a	a	a	a	b	b
b	a	a	b	a	a	a	a	b	b
b	a	a	b	a	a	a	a	b	b
b	a	a	b	-	a	a	a	b	b
b	a	a	b	a	a	a	a	b	b
b	a	b	b	a	a	a	a	b	b
b	a	-	b	a	a	a	a	b	b
b	a	a	b	a	-	a	a	b	b
-	a	a	b	a	a	a	a	b	b
b	a	a	b	a	a	a	a	b	b
b	a	a	b	a	a	-	a	b	b
b	a	-	b	a	a	a	a	b	b
b	a	-	b	a	a	a	a	b	b
b	a	a	b	a	a	-	a	b	b
b	b	a	b	a	a	a	a	b	b
b	b	a	b	a	a	a	a	b	b
b	b	a	b	a	a	a	a	b	b
b	b	a	b	a	a	a	-	b	b
b	b	a	b	a	a	a	a	b	b
b	b	a	b	a	a	a	a	b	b
b	b	a	b	a	a	a	a	b	b
b	b	a	b	a	a	a	-	b	b
b	b	a	b	a	a	a	a	b	b
b	b	a	b	a	a	a	a	b	b
b	b	a	b	a	a	a	a	b	b
b	b	a	b	a	a	a	a	b	b
b	b	a	b	a	a	a	a	b	b
b	b	a	b	a	a	a	a	b	b
b	b	a	b	a	a	a	a	b	b
b	b	a	b	a	a	a	a	b	b
b	b	a	b	a	a	a	a	b	b
-	b	a	b	a	a	a	a	b	b
a	b	a	-	-	a	a	a	b	b
b	b	a	b	b	a	a	a	b	b
b	b	a	b	-	a	a	a	b	b
-	b	a	b	b	a	a	a	b	b
b	b	a	b	b	a	a	a	b	b
b	b	a	b	b	a	a	a	b	b
b	b	a	b	b	a	a	a	b	b
b	b	-	b	b	-	a	a	b	b
b	b	a	b	b	a	a	a	b	b
b	b	a	b	b	a	a	a	b	b
b	b	a	b	b	a	a	a	b	b
a	b	a	b	b	a	a	a	b	b
b	b	a	b	b	a	a	a	b	b
b	b	a	a	b	a	a	a	b	b
b	b	a	a	b	a	a	a	b	b

b	b	a	-	b	a	a	a	b	b
b	b	a	b	b	a	a	a	b	b
b	b	a	b	b	a	a	a	b	b
a	b	a	b	b	a	a	a	a	b
b	b	a	b	b	a	a	a	a	b
b	b	a	b	b	a	a	a	a	b
b	b	-	b	b	a	a	a	a	b
-	-	a	b	b	a	a	a	a	b
b	b	a	b	b	a	a	a	a	b
b	b	a	b	b	a	a	a	a	b
b	b	b	b	b	a	a	a	a	b
b	b	a	b	b	a	a	a	a	b
b	b	a	b	b	-	a	a	a	b
b	-	a	b	b	a	a	a	a	b
b	b	a	b	b	a	a	a	a	b
b	-	a	b	-	a	a	a	a	b
b	b	a	b	b	a	a	a	b	b
b	b	a	b	b	a	a	a	b	b
b	b	-	b	b	a	a	a	b	b
-	b	a	-	b	a	a	a	b	b
b	b	a	b	b	a	a	b	b	b
b	b	a	b	b	a	a	b	b	b
b	b	a	b	b	a	a	b	b	b
b	b	a	b	b	a	a	b	b	b
b	b	a	b	b	a	a	b	b	b
b	b	a	b	b	a	a	b	b	b
b	b	b	-	b	a	b	b	b	b
b	b	-	a	b	a	b	b	b	b
b	b	a	a	b	a	b	b	b	b
b	b	a	a	b	a	b	b	b	b
-	-	a	a	b	a	b	b	b	b
b	b	a	-	b	a	b	b	b	b
b	b	a	a	b	a	b	b	b	b
b	b	a	a	b	a	b	b	b	b
a	b	a	a	b	a	b	b	b	b
a	b	a	a	b	a	b	b	b	b
a	b	a	a	b	a	b	b	b	b
a	b	a	a	b	a	b	b	b	b
a	b	a	a	b	a	b	b	b	b
a	b	a	a	b	a	b	b	b	b
a	-	a	a	b	a	b	b	b	b
a	-	a	a	b	a	b	b	b	b
a	b	a	a	-	a	b	b	b	b
a	b	a	a	b	a	b	b	b	b
a	b	a	a	b	a	b	b	b	b
a	b	-	a	b	a	b	b	b	b
a	b	a	a	b	a	b	b	b	b

a	b	a	a	b	b	b	b	b	b
a	b	-	a	b	b	b	b	b	b
a	b	a	-	b	b	b	b	b	b
a	b	-	a	b	b	b	b	b	b
a	b	a	a	b	b	b	b	b	b
a	b	a	a	b	b	b	b	b	b
a	b	a	a	b	b	b	b	b	b
a	b	a	a	b	b	b	b	b	b
-	b	a	a	b	b	b	b	b	b
a	b	a	-	b	b	b	b	b	b
a	b	a	a	b	b	b	b	b	b
a	b	a	a	b	b	b	b	b	b
a	b	a	a	b	-	b	b	b	b
a	-	a	a	b	-	b	b	b	b
a	b	a	a	b	b	b	b	b	b
a	b	a	a	b	b	b	b	b	b
a	b	-	-	b	b	b	b	b	b
-	b	-	a	b	b	b	b	b	b
a	b	a	a	b	b	b	b	b	b
b	b	a	b	b	a	b	b	a	a
b	b	b	b	b	a	b	b	a	a
b	b	b	b	b	a	b	b	a	a
a	b	-	b	b	a	b	b	a	a
b	b	b	b	b	a	b	b	a	a
b	a	-	b	-	a	b	b	a	a
b	b	-	b	b	a	b	b	a	a
b	b	-	b	b	a	b	b	a	a
b	b	b	b	b	a	b	b	a	a
b	b	a	b	-	a	b	b	a	a
b	b	b	b	b	a	b	b	a	a
b	a	b	b	b	a	b	b	a	a
b	a	b	b	b	a	b	b	a	a
b	a	b	b	b	a	b	b	a	a
b	a	b	-	b	a	a	b	a	a
b	-	b	b	b	a	b	b	a	a
-	a	b	b	b	a	-	b	a	a
b	a	-	b	b	a	b	b	a	a
b	a	b	b	b	a	b	b	a	a
b	a	b	b	b	a	b	b	a	a
b	a	b	b	b	a	b	b	a	a
b	a	-	b	b	a	b	b	a	a
b	a	b	b	b	a	b	b	a	a
b	a	b	b	b	a	b	b	a	a
b	a	-	b	b	a	b	b	a	a
b	a	b	b	-	a	b	b	a	a
b	-	b	b	b	a	b	b	a	a
b	a	b	b	b	a	b	b	a	a

b	a	b	b	b	a	b	b	a	a
-	a	b	b	b	a	b	b	a	a
-	a	b	b	b	a	b	a	a	a
b	a	b	b	b	a	b	a	a	a
b	a	-	-	b	a	b	a	a	a
-	a	-	b	b	a	b	a	a	a
-	a	a	b	-	a	b	a	a	a
b	a	b	b	b	a	b	a	a	a
b	a	b	b	b	a	b	a	a	a
b	a	b	b	b	a	b	a	a	a
b	a	b	b	b	a	b	a	a	a
b	a	b	b	b	a	b	a	a	a
b	a	b	b	b	a	b	a	b	a
b	a	b	b	b	a	b	a	b	a
b	a	b	b	b	a	b	a	a	a
b	a	b	b	b	b	b	a	a	a
b	a	b	b	b	b	b	a	a	a
b	a	b	b	b	b	-	a	a	a
-	a	-	b	b	b	b	a	a	a
b	a	b	b	b	b	b	a	a	a
b	a	b	b	b	b	b	a	a	a
b	a	-	b	b	b	b	a	a	a
b	a	a	b	b	b	b	a	a	a
b	-	b	b	b	b	b	a	a	a
b	a	a	b	b	b	b	a	a	a
b	a	a	b	b	b	b	a	a	a
a	a	a	b	b	b	b	a	a	a
a	a	a	b	b	b	b	a	a	a
a	a	a	b	b	a	b	a	a	a
a	a	a	b	b	a	b	a	a	a
a	a	a	b	b	a	b	a	a	a
a	a	a	b	b	a	b	a	a	a
-	a	a	b	b	a	b	a	a	a
a	a	a	b	b	a	b	a	a	a
a	a	a	b	b	a	b	a	a	a
-	a	a	b	b	a	b	-	a	a
a	a	a	b	a	b	b	a	a	a
a	b	a	b	a	b	b	b	a	a
a	b	a	b	a	b	b	b	a	a
a	b	a	-	a	b	b	b	a	a
a	b	a	b	a	b	b	b	a	a
a	b	a	b	a	-	b	b	a	a
a	b	a	b	a	b	b	b	a	a
a	b	a	b	a	b	b	b	a	a
a	-	a	b	a	b	-	b	a	a
a	-	a	b	a	b	b	b	a	a
a	b	-	b	a	b	b	b	a	a

a	b	a	b	a	b	b	b	a	a
a	b	a	b	a	b	b	b	a	a
a	b	a	b	-	b	b	b	a	a
-	b	-	b	a	b	b	b	a	a
a	b	a	b	a	b	b	b	a	a
a	-	a	b	a	b	b	b	a	a
a	b	a	b	a	b	b	b	a	a
b	b	a	a	a	a	b	b	b	a
b	b	a	a	a	a	b	b	b	a
-	-	a	a	a	a	b	b	b	a
a	b	b	a	a	a	b	b	b	a
a	b	-	b	a	a	a	a	b	a
a	b	b	b	a	a	a	a	b	a
a	b	b	b	-	a	a	a	b	a
a	b	b	b	a	a	a	a	b	a
a	b	b	b	a	a	a	a	b	a
a	b	b	b	a	a	a	a	b	a
a	b	b	b	a	a	a	a	b	a
a	-	b	b	a	a	a	a	b	a
a	b	b	-	a	a	a	a	b	a
a	b	b	b	a	a	a	a	b	a
a	b	b	b	a	a	a	a	b	a
a	b	b	b	a	a	a	a	b	a
a	b	b	b	a	a	a	a	b	a
a	b	b	b	a	a	a	a	b	a
a	b	b	b	a	a	a	a	b	a
a	b	b	b	a	a	a	a	b	a
a	b	a	-	a	a	a	a	b	a
a	b	b	b	a	a	a	a	b	a
a	b	b	b	a	a	a	a	b	a
a	b	b	b	a	a	a	a	b	a
a	b	b	b	a	a	a	a	b	a
a	b	b	b	a	a	a	a	b	a
b	b	b	b	a	a	a	a	a	a
a	b	b	b	a	a	a	a	a	a
a	b	b	b	a	a	a	a	a	a
a	b	b	b	a	a	a	a	a	a
a	b	b	b	a	a	a	a	a	a
a	b	b	b	a	a	a	a	a	a
a	b	b	b	a	a	a	a	a	a
a	-	b	b	a	a	a	a	a	a
a	b	b	b	-	a	a	a	a	a
a	b	b	b	a	a	a	a	a	a
-	b	b	b	-	a	a	a	a	a
a	b	b	b	a	a	a	a	a	a
a	b	b	b	a	a	a	a	a	a
a	b	-	b	a	a	a	a	a	a

a	b	b	b	-	a	a	a	a	a
a	b	b	b	a	a	a	a	a	a
a	b	b	b	a	a	a	a	a	a
-	b	b	b	a	a	a	a	a	a
a	b	b	b	a	a	a	a	a	a
a	b	b	a	a	a	a	a	a	b
a	-	b	a	a	a	a	a	a	b
a	b	-	a	a	a	a	a	a	b
a	b	b	a	a	a	a	a	a	b
a	b	b	a	a	a	a	a	a	b
a	b	b	a	a	a	a	a	a	b
a	b	b	a	a	a	a	a	a	b
a	b	b	-	a	a	a	a	a	b
a	b	b	a	a	a	a	a	a	b
a	b	b	a	a	a	a	a	a	b
a	b	b	a	a	a	a	a	a	b
a	b	b	a	a	a	a	a	a	b
a	b	b	-	-	-	a	a	a	b
a	b	b	a	a	a	a	a	a	b
a	b	b	a	a	a	a	a	a	b
a	b	b	a	-	a	a	a	a	b
a	b	b	a	a	a	a	a	a	b
a	b	b	a	a	a	a	a	a	b
a	b	a	a	a	a	a	a	a	b
a	b	b	a	a	b	a	b	a	b
-	b	b	a	a	b	a	b	a	b
a	b	b	a	a	b	a	b	a	b
a	b	b	a	a	b	b	b	a	b
a	b	b	a	a	b	b	b	a	b
a	b	a	b	a	b	b	b	a	b
a	b	a	b	a	b	b	b	a	b
a	b	a	b	a	b	b	b	a	b
a	b	a	b	a	b	b	b	a	b
a	b	a	b	a	b	b	b	a	b
a	b	a	b	a	b	b	b	a	b
a	b	a	b	a	b	b	b	a	b
-	b	a	b	a	b	b	b	a	b
a	b	a	b	a	b	b	b	a	b
a	b	a	b	a	b	b	b	a	b
a	b	a	b	a	b	b	b	a	b
a	b	a	b	a	b	b	b	a	b
a	b	a	b	a	b	b	b	a	b
a	b	a	b	a	b	b	b	a	b
b	b	b	b	a	b	b	b	a	b
b	-	b	b	a	b	b	b	a	b
b	b	b	b	a	b	b	b	a	b
b	b	b	b	a	b	b	b	a	b

b	b	-	b	a	b	b	b	a	b
b	b	b	b	a	b	b	b	a	b
b	b	b	b	a	b	a	b	a	b
b	-	-	b	a	b	a	b	a	b
b	b	b	b	a	b	a	b	a	b
b	b	b	b	a	b	a	b	a	b
b	b	b	b	a	b	a	b	a	b
b	b	b	b	a	b	a	b	a	b
b	b	b	b	a	b	a	b	a	b
b	b	b	-	a	b	a	b	a	b
a	b	b	-	a	b	a	b	a	b
b	b	b	b	a	-	a	b	a	b
b	b	b	b	a	b	a	b	a	b
b	b	b	b	a	b	a	b	a	b
b	b	b	-	a	b	a	b	a	b
b	b	b	b	a	b	a	b	a	b
b	b	b	b	a	b	a	b	a	b
b	b	b	b	a	b	a	b	a	b
b	b	b	b	a	b	a	b	a	b
b	b	b	b	a	b	a	b	a	b
b	b	b	b	a	b	a	b	a	b
b	b	b	b	a	b	a	b	a	b
a	b	a	b	a	a	b	a	a	b
-	b	a	b	a	a	b	a	a	b
a	b	a	b	a	a	b	a	a	b
a	b	a	b	a	a	b	a	a	b
a	b	a	b	a	a	b	a	a	b
-	b	a	b	a	a	b	a	a	b
a	b	a	b	a	a	b	a	a	b
a	b	-	b	a	a	b	a	a	b
-	b	a	b	a	a	b	a	a	b
a	b	a	b	b	a	b	a	a	b
a	b	a	-	b	a	b	a	a	b
a	-	a	b	b	a	b	a	a	b
a	b	a	b	b	a	b	a	a	b
a	b	a	b	b	b	b	a	a	b
b	b	a	b	b	b	-	a	a	b
b	b	a	b	b	b	b	a	a	b
b	b	a	b	b	b	b	a	a	b
b	b	-	b	b	b	b	a	a	b
b	b	a	b	b	b	b	a	a	b
b	b	a	b	b	b	b	a	a	b
b	b	a	b	b	b	b	a	a	b
b	b	a	b	b	b	b	a	a	b
b	b	-	b	b	b	b	a	a	b
b	a	-	b	b	b	b	a	a	b
b	a	a	b	b	b	b	a	a	b

b	a	-	a	b	b	b	a	a	b
b	a	a	a	b	b	b	a	a	b
b	a	a	a	b	b	b	a	a	b
b	a	a	a	b	b	b	a	a	b
b	a	a	a	b	b	b	a	a	b
b	a	a	a	b	b	b	a	a	b
b	a	a	a	-	b	b	a	a	b
b	a	a	a	b	b	b	a	a	b
b	a	a	a	b	b	b	a	a	b
b	-	a	a	b	b	b	a	a	b
b	a	a	a	b	b	b	a	a	b
b	a	a	a	b	b	b	a	a	b
-	a	a	a	b	b	b	a	a	b
b	a	a	a	b	b	a	a	a	b
b	a	a	a	b	b	a	a	a	b
b	a	a	a	b	b	a	a	a	b
b	a	a	a	b	b	a	a	a	b
b	a	b	a	b	b	a	a	a	b
b	a	a	a	b	b	a	a	a	b
a	a	a	a	b	b	a	a	a	b
a	a	a	a	b	b	a	a	a	b
a	a	a	a	b	b	a	a	a	b
a	a	a	a	b	b	a	a	a	b
a	a	a	a	b	b	a	a	a	b
a	a	-	a	b	b	a	a	a	b
a	a	a	b	b	b	a	a	a	b
a	a	a	b	b	b	a	a	a	b
a	a	-	b	b	b	a	a	a	b
a	a	a	b	b	b	a	a	a	b
a	a	a	b	b	b	a	a	a	b
a	a	b	b	b	b	a	a	a	b
a	a	a	b	b	b	a	a	a	b
a	a	a	b	b	b	a	a	a	b
a	a	a	b	b	b	a	a	a	b
-	a	a	b	b	b	a	a	a	b
-	-	-	b	b	b	a	a	a	b
a	-	a	b	b	b	a	a	a	b
-	-	a	b	b	b	a	a	a	b
-	a	b	b	b	b	a	a	a	b
-	a	-	b	b	b	a	a	a	b
-	a	a	b	-	b	a	a	a	b
b	a	a	b	b	b	a	a	a	b
b	a	a	b	b	b	a	a	a	b
-	a	a	b	b	b	a	a	a	b
b	a	-	b	b	b	a	a	a	b
b	a	a	b	b	b	a	a	a	b
b	-	a	b	b	b	a	a	a	b

b	a	a	b	b	b	a	a	a	b
b	a	b	b	b	b	a	a	a	b
-	a	a	b	-	b	a	a	a	b
b	a	a	b	b	b	a	a	a	b
b	-	a	b	b	b	a	a	a	b
b	a	a	b	b	b	a	a	a	b
b	a	-	b	b	b	a	a	a	b
b	a	a	b	b	b	a	a	a	b
b	a	a	b	b	b	a	a	a	b
b	a	b	b	b	b	a	a	a	b
b	a	a	a	b	b	a	a	a	b
b	a	a	a	b	b	a	a	a	b
-	a	a	a	b	b	a	a	a	b
b	a	a	a	b	b	a	a	a	b
b	a	a	a	b	b	a	a	a	b
-	a	a	a	-	b	a	a	a	b
-	a	a	a	b	b	a	a	a	b
b	a	a	a	b	b	a	a	a	b
b	a	a	-	b	b	a	a	a	b
b	a	a	a	b	b	a	a	a	b
b	a	a	a	b	b	a	a	a	b
b	a	a	a	b	b	a	a	a	b
b	a	a	a	b	b	a	a	a	b
b	a	a	a	b	b	a	a	a	b
b	a	-	a	b	b	a	a	a	b
b	a	a	a	b	b	a	a	a	b
b	a	a	a	b	b	a	a	a	b
b	a	a	a	b	b	a	a	a	b
b	a	a	a	b	b	a	a	a	b
b	a	a	a	b	b	a	a	a	b
b	a	a	a	b	-	a	a	a	b
b	a	a	-	b	b	a	a	a	b
b	a	a	a	b	b	a	a	a	b
b	a	a	a	b	b	a	a	a	b
b	a	a	a	-	b	a	a	a	b
b	a	a	a	b	b	a	a	a	b
b	a	a	a	b	b	a	a	a	b
b	a	a	a	b	b	a	a	a	b
b	a	a	a	b	b	a	a	a	b
b	a	a	a	b	b	a	a	a	b
b	-	-	a	b	b	a	a	a	b
b	a	a	a	b	b	a	a	a	b
b	a	a	a	-	b	a	a	a	b
b	b	a	a	b	b	a	a	a	b
b	a	-	a	b	b	a	a	a	b
b	a	a	a	b	b	a	a	a	b
b	a	a	a	b	b	a	a	a	b

b	a	a	a	b	b	a	a	a	b
b	a	a	a	b	b	a	a	a	b
b	a	a	a	b	b	a	a	a	b
b	a	b	b	b	b	b	a	b	a
b	a	b	b	b	b	b	a	b	a
b	a	b	a	b	b	b	a	b	a
b	a	b	a	b	b	b	a	b	a
b	a	b	a	-	b	b	a	b	a
b	a	b	a	b	b	b	a	b	a
b	a	b	a	b	b	b	a	b	a
b	a	b	a	b	b	b	a	b	a
-	a	b	a	b	b	b	a	b	a
b	a	b	a	b	b	b	a	b	a
b	a	a	a	b	b	b	a	b	a
b	a	b	a	b	b	b	a	b	a
b	a	-	a	-	b	b	a	b	a
b	a	b	a	b	b	b	a	b	a
b	a	-	a	b	b	b	a	b	a
b	a	b	a	b	b	b	a	b	a
b	a	b	a	b	b	b	a	b	a
b	a	b	a	b	b	b	a	b	a
b	a	-	a	b	b	-	a	b	a
b	a	b	a	b	b	b	a	b	a
b	a	-	a	b	b	b	a	b	a
b	a	b	a	b	b	b	a	b	a
b	a	b	a	b	b	b	a	b	a
b	a	b	a	b	b	b	a	b	a
b	a	b	a	b	b	b	a	b	a
b	b	b	a	b	b	b	a	b	a
b	b	b	a	b	b	b	a	b	a
b	b	b	a	b	b	b	a	b	a
b	b	b	a	b	b	b	a	b	a
b	b	b	a	b	b	b	a	b	a
a	b	b	a	b	b	b	a	b	a
a	b	b	a	b	b	b	a	b	a
a	b	b	a	b	b	b	a	b	a
a	b	b	a	b	b	b	a	b	a
a	b	b	a	b	b	b	a	b	a
a	b	b	a	a	b	b	a	b	a
a	b	b	a	a	b	b	a	b	a
-	a	b	a	a	b	a	a	b	a
a	a	b	a	a	b	a	a	b	a
a	a	b	a	a	b	a	a	b	a
a	a	b	a	a	b	a	a	b	b
a	a	b	a	a	b	a	a	b	b
a	a	b	a	a	b	a	a	b	b
a	a	b	a	a	b	a	a	b	b

a	a	b	a	a	b	a	a	b	b
a	a	b	a	a	b	a	a	b	b
a	a	b	a	a	b	a	a	b	b
a	a	b	a	a	b	a	a	b	b
a	a	b	a	-	a	a	a	a	b
a	a	a	a	b	a	a	a	a	b
a	a	b	a	b	a	a	a	a	b
a	a	b	a	b	a	a	a	a	b
a	a	b	a	b	a	a	a	a	b
-	a	b	a	b	a	a	a	a	b
a	a	-	a	b	a	a	a	a	b
-	a	b	a	b	a	a	a	a	b
b	-	b	a	b	a	b	a	a	b
b	b	b	b	b	b	b	a	a	b
b	b	b	b	b	b	b	a	a	b
b	b	b	b	b	b	b	a	a	b
b	b	b	b	b	b	b	a	a	b
b	b	b	b	b	b	b	a	a	b
b	b	b	b	b	b	b	a	a	b
b	b	-	b	b	b	b	a	a	b
-	b	b	b	b	b	b	a	a	b
b	b	b	b	b	b	b	a	a	b
b	b	b	b	b	b	b	a	a	b
b	b	b	b	b	b	b	a	a	b
b	b	b	b	b	b	b	b	a	b
a	b	b	b	b	b	b	a	b	b
a	b	b	b	b	b	b	a	b	b
a	b	b	b	b	b	b	b	b	b
a	b	a	b	-	b	a	a	b	b
-	b	b	a	b	b	a	b	b	b
b	b	b	a	b	b	a	b	b	b
b	b	b	a	b	b	a	b	b	b
b	b	b	a	b	b	a	b	b	b
-	b	a	a	-	b	a	b	b	b
b	b	b	-	b	b	a	b	b	b
b	b	b	a	b	b	a	b	b	b
b	b	b	a	b	b	a	b	b	b
b	b	b	a	-	b	a	b	b	b
b	b	b	a	b	b	a	b	b	b
b	b	b	a	a	b	a	b	b	b
b	b	b	a	a	b	a	b	b	b
b	-	a	a	a	a	a	b	b	b
b	b	a	a	a	a	a	b	b	b
b	-	a	a	a	a	a	b	b	b
b	b	a	a	a	a	a	b	b	b
-	-	b	b	b	a	b	a	a	b
a	-	b	b	b	a	b	a	a	b

a	a	b	b	b	a	b	a	a	b
a	b	b	b	b	a	b	a	a	b
a	b	b	b	b	a	b	a	a	b
a	b	b	b	b	a	b	a	a	b
-	b	b	b	b	a	b	a	a	a
a	b	b	b	b	a	b	a	b	-
a	b	b	b	b	a	b	a	b	a
a	b	b	b	b	a	b	a	b	a
a	b	b	b	b	a	b	a	b	a
a	b	b	b	b	a	b	a	b	a
a	b	b	b	b	a	b	a	b	-
a	b	-	-	b	a	b	a	b	a
a	b	b	-	b	a	b	a	b	a
a	b	b	b	b	b	b	a	b	a
a	b	-	b	-	b	b	a	b	a
b	b	b	b	b	b	b	-	b	-
b	b	b	b	b	b	b	a	b	a
b	b	b	b	b	b	b	a	b	a
b	b	b	b	b	b	b	a	b	a
b	b	a	b	b	b	a	a	b	a
b	b	b	b	b	b	a	a	b	a
b	b	b	b	b	b	a	a	b	a
a	b	b	b	b	b	a	a	b	a
b	b	b	b	b	b	a	a	b	a
b	b	b	b	b	b	a	a	b	a
-	a	b	b	b	-	a	a	b	a
b	b	b	b	b	b	a	a	b	a
b	b	b	b	b	b	a	a	b	a
b	b	b	b	b	b	a	a	b	a
b	b	b	b	b	b	a	a	b	a
b	b	b	b	b	b	a	a	b	a
-	-	-	b	b	b	a	a	b	a
b	b	b	b	b	b	a	a	b	a
b	-	-	b	b	a	a	a	b	a
b	b	b	b	-	a	a	a	b	a
b	b	b	b	b	a	a	a	b	a
b	b	b	b	b	a	a	a	b	a
b	b	b	b	b	a	a	a	b	a
b	b	b	b	b	a	a	a	b	a
b	b	b	b	b	a	a	a	b	a
b	b	b	b	b	a	a	a	a	a
b	b	b	b	b	a	a	a	a	a
b	-	a	b	b	a	a	a	a	a
b	b	b	b	b	a	a	a	a	a
b	b	b	b	b	a	a	a	a	a
b	b	b	b	b	a	a	a	a	a
b	b	b	b	b	a	a	a	a	a

b	b	b	b	b	a	a	a	a	a
b	-	b	b	b	a	a	a	a	a
b	b	b	b	b	a	a	a	a	a
b	b	b	b	b	a	a	a	a	a
b	b	b	-	b	a	a	a	a	a
b	b	b	b	b	a	a	b	a	a
b	-	b	b	b	a	a	b	a	a
-	a	b	b	b	a	a	b	a	a
b	a	-	b	b	a	a	b	a	a
b	a	b	b	b	a	a	b	a	a
-	b	-	b	-	a	a	b	a	a
b	b	b	b	b	a	a	b	a	a
b	b	-	b	b	a	a	b	a	a
b	b	b	b	b	a	a	b	a	a
b	b	b	a	b	b	a	b	a	a
b	b	b	a	b	b	a	b	a	a
b	b	b	a	b	b	a	b	-	a
b	b	b	a	b	b	a	b	a	a
b	b	b	a	b	b	a	b	a	a
b	-	b	a	b	b	a	b	a	a
b	b	b	a	b	b	a	b	a	a
b	b	b	a	b	b	a	b	a	a
b	b	b	-	b	b	a	b	a	a
b	b	-	a	b	b	a	b	a	a
b	b	b	-	b	b	a	b	a	a
b	b	b	a	b	b	a	b	a	a
b	b	b	b	b	b	a	b	a	a
b	b	b	b	b	b	a	b	a	a
b	b	b	b	b	b	a	b	a	a
b	b	b	b	b	b	a	b	a	a
b	b	b	b	b	b	a	b	a	a
b	b	-	-	b	b	a	b	a	a
b	b	b	b	b	b	a	b	a	a
b	b	b	b	b	b	a	b	a	a
b	b	b	b	-	b	b	a	a	a
b	b	b	b	b	b	b	b	a	a
b	b	b	b	b	b	b	b	a	a
b	b	b	b	b	b	b	b	a	a
b	b	b	b	b	b	b	b	a	a
b	b	b	b	b	b	b	b	b	a
b	b	b	b	b	b	b	-	b	a
b	b	b	b	b	b	b	b	b	a
-	b	b	-	b	b	b	b	a	a
-	b	b	b	b	b	-	b	a	a
a	b	b	b	b	b	b	b	a	a
b	b	b	b	b	b	b	b	a	a
a	b	b	b	b	b	b	b	a	a

a	b	b	b	b	b	b	b	a	a
a	-	b	b	-	b	b	b	a	a
a	b	b	b	b	b	b	b	a	a
a	b	b	b	b	b	b	b	a	a
-	b	b	b	b	b	b	b	a	a
a	b	b	b	b	b	b	b	a	a
b	a	b	b	b	b	b	b	a	a
a	b	b	b	b	b	b	b	a	a
b	b	b	b	-	b	b	a	a	b
b	b	a	b	a	b	b	a	a	b
b	a	a	b	a	b	b	a	a	b
b	a	a	b	a	b	b	a	a	b
b	a	a	b	a	b	b	a	a	b
b	a	a	b	a	b	b	a	a	b
b	a	a	-	a	b	b	a	a	b
b	a	a	b	a	b	b	a	a	b
b	a	b	b	a	b	b	a	a	b
b	-	-	b	a	b	b	a	a	b
b	a	a	b	a	b	b	a	a	b
b	a	b	b	a	b	b	a	b	b
b	a	a	b	a	b	b	a	b	b
b	a	a	b	a	b	b	a	b	b
b	a	a	b	a	b	b	a	b	b
a	a	b	b	a	b	b	a	b	b
a	a	b	b	a	b	b	a	b	b
a	a	b	b	a	b	b	a	b	b
a	a	b	b	a	b	b	a	b	b
a	-	b	b	-	b	b	a	b	a
a	a	b	b	a	b	b	a	b	a
a	a	b	b	b	b	b	a	b	a
a	a	b	b	b	a	b	a	b	a
a	a	b	b	b	a	b	a	b	a
a	a	-	b	b	a	b	a	b	a
-	a	b	b	b	a	b	a	b	a
a	a	b	-	a	a	b	a	b	a
a	-	b	b	a	a	b	a	b	a
a	a	a	b	a	a	-	a	b	a
a	a	b	b	a	a	b	a	b	a
b	a	b	b	a	a	b	a	b	a
b	a	b	-	a	a	b	a	b	a
b	a	b	b	a	a	b	a	b	a
-	a	b	b	a	a	b	a	b	a
b	a	b	b	a	a	b	a	b	a
a	a	b	-	a	a	b	a	b	a
a	a	b	b	a	a	b	a	b	a
a	a	b	b	a	a	b	a	b	a
a	a	b	b	a	a	b	a	b	a

a	a	b	-	a	a	-	a	b	a
a	a	b	-	a	a	b	a	b	a
a	a	b	b	a	a	b	a	b	a
a	a	b	b	a	a	b	a	b	a
a	a	b	b	a	a	b	a	b	a
a	a	b	-	a	a	b	a	b	b
a	a	b	b	b	a	b	a	b	b
a	a	b	b	b	a	b	a	b	b
a	a	-	b	b	a	b	a	b	b
a	a	a	b	b	a	b	a	b	b
a	a	a	b	b	a	b	a	b	b
b	-	a	b	b	a	b	a	b	b
b	a	b	a	b	a	b	a	b	b
b	a	b	a	b	a	b	a	b	b
b	a	b	a	b	a	b	a	b	-
b	a	b	a	b	a	b	a	b	b
b	a	b	a	b	a	b	a	a	b
b	a	b	a	b	a	b	a	a	b
-	a	b	a	b	a	b	a	a	b
b	a	b	-	b	a	b	a	a	b
b	a	b	a	b	a	b	a	a	b
b	a	b	a	b	a	b	a	a	b
b	a	b	a	b	a	b	a	a	b
b	a	b	b	b	a	b	b	a	b
b	a	b	b	b	b	b	b	a	b
b	a	b	b	b	b	b	b	a	b
b	a	b	b	b	b	b	b	a	b
b	a	b	b	b	b	b	b	a	b
-	a	b	b	b	b	b	b	a	b
b	a	b	b	b	-	b	b	a	b
b	a	b	b	-	b	b	b	a	b
b	a	b	b	b	b	b	b	a	b
b	b	a	b	b	b	b	b	a	b
b	a	b	b	b	b	b	b	b	a
b	a	b	b	b	b	b	b	b	a
b	a	b	b	b	b	b	b	b	a
b	a	b	b	b	b	b	b	b	a
b	a	b	b	b	b	b	b	b	a
b	b	b	b	b	b	b	b	b	a
b	a	b	-	b	b	b	b	b	a
b	a	b	b	b	b	b	b	b	a
b	-	b	b	b	a	b	b	b	a
b	a	b	b	b	a	b	b	b	a
b	a	b	b	b	a	b	b	b	a
b	a	b	b	b	a	b	b	b	a
b	a	b	b	b	a	b	b	a	b
b	a	b	-	b	a	b	b	a	b

b	a	b	b	b	a	b	b	a	b
b	a	b	b	a	-	b	b	a	b
b	a	a	b	a	a	b	b	a	b
b	-	a	b	a	a	b	b	a	b
-	a	a	b	a	a	b	b	a	b
b	a	a	b	-	a	b	b	a	b
b	a	a	b	a	a	b	b	a	b
b	a	a	b	a	a	b	b	a	b
a	a	a	b	a	b	a	b	a	b
a	-	a	b	a	b	a	b	a	b
a	a	a	b	a	b	a	b	a	b
a	a	a	b	a	b	a	b	a	-
a	a	-	b	a	b	a	b	a	b
a	a	a	b	a	b	a	b	a	b
a	a	a	b	-	b	a	b	a	b
a	a	a	b	a	b	a	b	a	b
a	a	a	b	a	b	a	b	a	b
a	a	a	b	a	b	a	b	a	b
a	a	a	b	a	-	a	b	a	b
a	a	a	b	a	b	a	b	a	b
a	a	a	b	a	b	a	b	a	b
a	a	a	b	a	b	a	b	a	b
a	a	a	b	-	b	a	b	a	b
a	-	a	b	a	b	a	b	a	b
a	a	a	b	a	b	a	b	a	b
a	a	a	a	a	b	a	b	a	b
a	a	a	a	a	b	a	b	a	b
a	a	a	a	a	b	a	b	a	b
a	a	a	a	a	b	a	b	a	-
a	a	a	a	a	b	a	b	a	b
a	a	-	a	a	b	a	b	a	b
a	-	a	a	a	b	a	b	a	b
a	a	a	a	a	b	a	b	a	b
a	a	a	a	a	b	a	b	a	b
a	a	a	a	a	b	a	b	a	b
a	a	a	a	a	a	a	b	a	b
a	-	-	a	a	a	a	b	a	b
a	a	a	a	a	a	a	b	a	b
a	a	a	a	a	a	a	b	a	b
a	a	a	a	a	a	a	b	a	b
a	a	a	a	a	a	a	b	a	b
a	a	a	a	a	a	a	b	b	b
a	a	-	-	-	a	a	b	b	b
a	a	a	a	a	a	a	b	b	b
a	a	a	a	a	a	a	b	b	b
a	a	a	a	a	a	a	b	b	b
a	a	a	a	a	a	a	b	b	b

a	a	a	a	a	a	a	b	b	b
a	a	a	a	a	a	a	b	b	b
a	a	a	a	a	a	a	b	b	b
a	a	a	a	a	b	a	b	b	b
a	a	a	a	a	b	a	b	b	b
a	a	a	a	a	b	b	b	b	b
a	a	a	b	a	b	b	b	b	b
a	a	a	b	a	b	b	b	b	a
a	a	a	b	a	b	b	b	b	a
a	a	a	b	a	b	b	b	b	a
a	a	-	b	b	b	b	b	b	a
a	a	a	b	a	b	b	-	b	a
a	a	a	b	a	b	b	b	b	a
a	a	a	b	a	b	b	b	b	a
a	a	a	b	a	b	b	b	b	a
b	a	a	b	a	b	b	b	b	a
b	a	a	b	a	b	b	b	b	a
b	a	a	b	a	b	b	b	b	a
a	a	a	b	-	b	a	b	a	a
a	a	-	b	a	b	a	b	a	a
b	b	a	b	b	b	b	b	a	a
b	b	a	b	b	b	b	a	a	a
b	b	a	b	a	b	b	a	a	a
b	b	a	b	a	b	a	a	a	a
b	b	a	b	a	b	b	a	a	a
b	b	a	b	-	b	b	a	a	a
b	b	a	b	a	b	b	a	a	a
b	b	a	b	a	b	a	a	a	a
b	b	a	b	a	b	a	a	a	a
b	b	a	b	a	b	a	a	a	a
b	b	a	b	a	b	a	a	a	a
b	b	a	b	a	b	a	a	a	a
b	b	a	b	a	b	a	a	a	a
b	b	a	b	a	b	a	a	a	a
b	b	a	b	a	b	a	a	a	a
b	b	a	b	a	b	a	a	a	a
b	b	a	b	a	b	a	a	a	a
b	b	a	b	a	b	a	a	a	a
-	b	a	b	a	b	a	a	a	a
b	a	a	b	-	b	a	a	b	a
b	a	a	b	a	b	a	b	a	a
b	a	a	b	a	b	a	b	b	a
-	a	a	-	a	b	a	b	b	a
a	a	a	b	a	b	a	b	b	a
a	a	a	b	a	b	a	b	b	a
a	a	a	b	a	a	a	-	b	a
a	a	a	b	a	b	a	b	b	a

a	a	a	b	a	b	a	b	b	a
a	a	a	b	a	b	a	b	-	a
a	a	a	b	a	b	a	b	b	a
a	a	a	b	a	b	a	b	b	a
a	a	a	b	a	b	a	b	b	a
a	a	a	b	a	b	a	b	b	a
a	a	a	b	-	b	a	b	b	a
a	a	-	b	a	b	-	b	b	a
a	a	a	b	a	b	a	b	b	a
a	a	a	b	a	b	a	b	b	a
a	a	a	b	a	b	a	b	b	a
a	a	a	b	a	b	a	b	b	a
b	a	a	b	a	b	a	b	b	a
b	a	a	b	a	b	a	b	b	a
b	a	a	b	a	b	a	b	b	a
b	a	a	b	a	b	a	b	b	a
b	-	a	b	a	b	a	b	b	a
-	a	a	b	a	b	a	b	b	a
b	a	a	-	a	b	a	b	b	a
b	-	a	-	a	b	a	b	b	a
b	a	a	a	a	b	a	b	b	b
b	a	a	a	a	b	a	b	b	b
b	a	a	a	a	b	a	b	b	b
b	a	a	a	a	b	a	b	b	b
b	a	a	a	a	b	a	b	b	b
a	a	a	a	a	b	b	b	b	b
a	a	a	a	a	b	b	b	b	b
a	b	a	a	a	b	b	b	b	b
a	a	a	a	b	b	b	b	b	b
a	a	a	a	-	-	b	b	b	b
a	a	a	a	b	b	b	b	b	b
-	a	a	a	b	b	b	b	b	b
b	a	a	a	-	b	b	b	b	b
b	a	a	a	b	b	b	b	b	b
b	a	a	a	b	b	b	b	b	b
a	b	a	b	a	b	a	b	b	b
a	b	b	b	a	b	a	b	b	b
a	b	a	b	a	b	a	b	b	b
a	b	a	b	a	b	a	b	b	b
a	b	a	b	a	b	-	b	b	a
a	b	-	b	a	b	a	b	b	a
a	b	a	b	a	b	a	b	b	a
-	b	a	b	a	b	a	b	b	a
a	b	a	b	-	b	a	b	b	a
a	b	a	b	-	b	a	b	b	a
a	b	a	b	a	b	a	b	b	a
a	b	a	b	a	b	a	b	b	a

a	b	a	b	a	b	a	a	b	a
a	b	a	b	a	b	a	a	b	a
a	b	a	b	a	b	a	a	b	a
a	b	a	b	a	b	a	b	b	a
a	b	a	b	a	-	a	b	b	a
a	b	-	b	a	b	a	b	b	a
a	-	a	b	a	b	a	b	b	a
a	b	a	b	a	b	a	b	b	a
a	b	a	b	a	b	a	b	b	a
a	b	a	-	a	b	a	b	b	a
a	b	a	b	a	b	a	b	b	a
a	b	a	b	a	b	a	b	b	a
a	b	a	b	a	b	a	b	b	a
a	b	a	-	a	b	a	b	b	a
a	b	a	b	a	b	a	b	b	a
a	b	a	b	a	a	a	b	b	a
-	b	b	b	a	b	a	b	b	a
a	b	b	b	a	b	a	b	b	a
a	b	b	b	a	b	a	b	b	a
a	-	b	b	b	b	a	b	b	a
a	b	b	b	b	b	a	b	b	a
a	b	b	b	b	b	a	b	b	a
a	b	b	b	b	b	a	b	b	a
a	b	-	b	b	b	a	b	b	a
a	b	b	b	b	b	a	b	b	a
a	b	b	b	-	b	a	b	b	a
a	b	b	b	b	b	a	b	b	a
a	b	-	-	b	b	a	b	b	a
a	a	b	b	b	b	a	b	b	b
-	a	b	b	b	b	a	b	b	b
a	a	b	b	b	b	a	b	b	b
a	a	b	b	b	b	a	b	a	b
a	a	b	b	b	b	a	b	a	b
a	a	-	b	b	b	a	-	a	b
a	a	b	b	b	b	a	b	a	b
a	a	b	b	b	-	a	b	a	b
a	a	b	b	b	b	a	b	a	b
a	a	b	b	b	b	a	b	a	b
a	a	b	b	b	b	a	b	a	b
a	a	b	b	b	b	a	b	a	b
a	a	b	-	b	b	a	b	a	b
a	a	a	b	b	b	a	b	a	b
a	-	b	b	b	b	b	b	a	b
a	a	b	b	b	b	b	b	a	b
a	a	b	b	b	b	b	b	a	b
a	a	b	b	b	b	b	b	a	b
b	b	a	a	b	a	b	a	a	a

b	b	a	a	b	a	b	a	a	a
b	b	a	a	b	a	b	a	a	a
b	b	a	a	b	a	b	a	a	a
b	b	a	a	b	a	b	a	a	a
b	b	a	a	b	a	b	a	a	a
b	b	a	a	b	a	b	a	a	a
b	b	a	a	b	a	a	a	a	a
b	b	a	a	b	b	a	a	a	a
b	b	a	a	b	b	a	a	a	a
b	b	a	a	b	b	a	a	a	a
b	b	a	a	b	b	a	a	a	a
b	b	a	a	b	b	b	a	a	a
b	b	a	a	b	b	b	a	a	a
b	b	a	a	b	b	b	a	a	a
b	b	a	a	b	b	b	a	a	a
b	b	a	a	b	b	b	a	a	a
b	b	a	a	b	b	b	a	a	b
b	b	a	a	-	b	b	a	a	-
b	b	a	a	b	b	b	a	a	b
b	b	a	a	b	b	b	a	a	b
b	b	a	a	b	b	b	a	a	b
b	b	a	a	b	b	b	a	a	b
b	b	a	a	b	b	b	a	a	b
b	b	a	a	b	b	b	a	a	b
b	b	a	a	b	b	b	a	a	b
b	b	a	a	b	b	b	b	a	b
b	b	a	a	b	b	b	b	a	b
b	b	a	a	b	b	b	b	a	b
b	b	a	a	b	b	b	b	a	b
b	b	a	a	a	b	b	b	a	b
b	b	a	a	a	b	b	b	a	b
b	b	a	a	a	b	b	b	a	b
b	b	a	a	a	b	b	b	a	b
a	b	a	a	a	b	b	b	a	b
b	b	a	a	a	b	b	b	a	b
b	b	a	a	a	b	b	b	a	b
b	b	a	a	a	b	b	b	a	b
b	b	a	a	a	b	b	b	a	b
b	b	a	a	a	b	b	b	a	a
b	b	a	a	a	b	b	b	b	b
a	b	a	b	b	a	a	a	a	a
a	a	a	a	b	b	a	b	b	b
b	a	b	a	b	b	a	b	b	b
b	a	b	a	b	b	a	b	b	b
b	a	b	a	b	b	a	b	b	b
b	a	b	a	b	b	a	b	b	b
b	a	b	a	b	b	a	b	b	b

b	a	b	a	b	b	b	b	b	b
b	a	b	a	b	b	b	b	b	b
b	a	b	a	b	b	b	b	b	b
b	a	b	a	b	b	b	b	b	a
b	b	b	a	b	b	b	b	a	a

YW131	YW132	YW133	YW134	YW135	YW136	YW137	YW138	YW139	YW140
b	b	b	b	-	b	b	a	a	a
b	b	b	b	b	b	b	a	a	a
b	b	b	b	b	b	b	a	a	a
b	b	b	b	b	b	b	a	a	a
a	b	b	b	-	b	b	a	a	a
b	-	b	b	b	b	b	a	a	a
a	b	b	b	b	b	b	a	a	a
b	b	b	b	-	b	b	a	a	a
b	b	b	b	b	b	b	a	a	a
b	b	b	-	-	-	b	a	a	a
b	b	-	-	b	b	b	a	a	a
b	b	b	b	b	b	b	a	a	a
-	-	-	b	b	b	b	a	a	a
b	b	b	b	b	b	b	a	a	-
b	b	b	b	b	b	b	a	a	a
b	b	b	b	b	b	b	a	a	a
b	b	b	b	b	b	b	a	a	a
b	b	b	b	b	b	b	a	a	a
b	b	b	b	b	b	b	a	a	a
b	b	b	b	b	b	b	a	a	a
b	b	-	b	b	-	b	a	a	a
b	b	b	b	b	b	b	a	a	a
b	b	b	b	b	b	b	a	a	a
b	b	b	b	b	b	b	a	a	a
b	b	b	b	b	b	b	a	a	a
b	b	b	-	b	b	-	a	a	a
b	b	b	b	b	b	b	a	a	a
b	b	b	-	b	b	b	a	a	a
b	b	b	b	b	b	b	a	a	a
b	b	b	b	b	b	b	a	a	a
b	b	-	b	b	b	b	a	a	a
b	b	b	b	b	b	b	a	a	a
b	b	b	b	b	b	b	a	a	a
b	b	b	b	b	-	a	a	b	a
b	b	b	b	b	b	a	a	b	a
b	b	b	-	b	b	a	a	b	a
a	b	b	b	b	b	a	a	b	a
b	b	b	b	b	b	a	a	b	a
b	b	b	b	b	b	a	a	b	a
b	b	b	b	b	b	a	a	b	a
b	b	b	b	b	b	a	a	b	a
b	b	b	b	b	b	a	a	b	a
b	b	b	b	b	b	a	a	b	a
b	b	b	b	b	b	a	a	b	a
b	b	b	b	b	b	a	a	b	a

b	b	b	b	b	b	a	a	b	a
b	-	a	b	-	b	a	a	b	a
a	b	a	b	-	b	a	a	b	a
b	b	a	b	b	b	a	a	b	a
b	b	a	b	b	b	a	a	b	a
b	b	a	b	b	b	a	a	b	a
b	b	-	b	b	b	a	a	b	a
b	b	-	b	b	b	a	a	b	a
b	b	a	b	-	b	a	a	b	a
b	b	a	b	b	b	a	a	b	a
b	a	a	b	b	b	a	b	b	a
b	b	a	b	a	a	a	b	b	a
b	b	a	b	a	a	a	b	b	a
b	b	a	b	a	a	a	b	b	a
b	b	a	b	a	a	a	b	b	a
b	b	a	b	a	a	a	b	b	a
b	b	a	b	a	-	a	b	b	a
b	-	a	-	a	a	a	b	b	a
b	b	a	b	a	a	a	b	b	a
b	b	a	b	a	a	a	b	b	a
b	b	-	b	b	a	a	b	b	a
b	-	a	b	a	a	a	-	b	a
-	b	a	-	a	a	a	b	b	a
b	-	a	b	a	a	a	b	b	a
b	b	a	b	a	a	a	b	b	a
b	b	-	b	a	a	a	b	b	a
b	a	a	b	a	a	a	b	b	a
b	a	a	b	a	a	a	b	b	a
b	b	a	b	-	a	-	b	b	a
b	b	a	b	a	a	a	b	b	a
b	b	a	-	a	a	a	b	b	a
b	b	a	b	a	a	a	a	b	a
b	b	a	-	a	a	a	a	b	a
b	b	a	b	a	a	a	a	b	a
-	b	a	b	a	a	a	a	b	a
b	b	a	b	a	a	a	a	b	a
b	b	a	b	a	a	a	a	b	a
a	b	a	-	a	a	a	a	b	a
b	b	a	-	a	a	a	a	b	a
b	a	a	a	b	a	b	b	-	a
b	a	-	a	b	a	b	b	b	a
-	a	a	a	b	a	b	b	b	a
-	a	a	a	-	a	b	b	b	a
b	a	-	-	b	a	b	b	b	a
b	a	a	a	b	a	b	b	b	a
b	a	a	a	b	a	b	b	b	a
-	a	a	a	-	a	b	b	b	a

b	-	a	a	b	a	b	b	b	a
b	-	-	a	b	a	b	b	b	a
b	a	a	a	a	a	b	b	b	a
b	a	a	a	b	a	b	b	b	a
b	a	a	a	b	a	-	b	b	a
a	a	a	a	b	a	b	b	b	a
b	a	a	a	b	a	b	b	b	a
b	a	-	a	b	a	b	b	b	a
b	a	a	a	b	a	b	b	b	a
b	a	a	a	a	a	b	b	b	a
b	a	a	a	a	a	b	b	b	a
b	a	a	a	a	a	b	b	b	a
b	a	a	a	a	a	b	b	b	a
b	a	a	a	a	a	b	b	b	a
b	a	a	a	a	a	b	b	b	a
b	a	a	-	a	a	b	b	b	a
b	-	a	a	a	a	b	b	b	a
b	a	a	-	-	a	b	b	b	a
b	a	a	a	a	a	b	b	b	a
b	a	a	a	a	a	a	b	b	a
b	a	a	-	a	a	a	b	b	a
b	a	a	a	a	a	a	b	b	a
b	a	a	-	a	a	-	b	b	a
b	a	a	a	a	a	a	b	b	a
b	a	a	-	a	a	a	b	b	a
b	a	a	a	a	a	a	b	b	a
b	-	a	-	-	a	a	b	b	a
a	a	a	a	a	a	a	b	b	a
b	a	a	a	-	a	a	b	b	a
-	a	a	a	a	a	a	b	b	a
a	a	a	a	a	a	a	b	b	a
-	a	a	a	a	a	a	b	b	a
-	a	a	a	a	a	a	b	b	a
a	a	b	a	a	a	a	b	b	a
b	a	a	a	a	a	a	-	b	a
b	a	b	a	b	a	a	b	b	a
b	a	b	a	-	a	a	b	b	a
b	a	-	a	a	b	a	b	b	a
b	a	b	-	a	a	-	a	b	a
b	a	b	a	a	b	a	a	b	a
b	a	b	a	a	b	a	a	b	a
b	a	b	a	a	-	a	a	b	a
b	a	b	a	a	b	a	-	b	a
b	a	b	a	a	-	a	a	b	a
b	a	b	a	a	b	a	a	b	a
b	a	b	a	a	b	a	a	b	a
-	a	b	a	a	b	a	a	-	a

b	a	b	a	-	b	a	a	b	a
b	a	b	a	a	b	a	a	b	a
b	a	b	a	a	b	a	a	b	a
b	a	b	a	a	b	a	a	b	a
b	a	b	a	a	b	a	a	b	a
b	-	b	a	a	b	a	a	b	a
-	a	b	a	a	b	a	a	b	a
b	a	b	a	a	b	a	a	b	a
b	a	b	a	a	b	a	a	b	a
b	b	b	a	a	b	a	a	b	a
b	b	-	a	a	b	a	a	b	a
b	b	b	a	a	b	a	a	b	a
b	b	b	a	-	b	a	a	b	a
a	b	b	a	a	-	a	a	b	a
a	b	b	a	a	b	a	a	b	a
-	b	b	a	a	b	a	a	b	a
a	-	b	a	a	b	a	a	b	a
a	b	b	a	a	b	a	a	b	a
a	b	b	a	a	b	a	a	b	a
a	b	b	a	a	b	a	a	b	a
a	b	-	a	a	b	a	a	b	a
a	b	b	a	-	b	a	a	b	a
a	b	b	a	a	b	b	a	b	a
-	b	b	a	a	b	b	a	b	a
a	b	b	a	a	b	b	a	b	a
a	b	-	a	a	b	b	a	b	a
a	b	b	a	a	b	b	a	b	a
a	b	b	a	a	b	b	a	b	a
a	b	-	a	a	b	b	a	b	a
b	b	b	a	a	b	b	a	b	a
a	b	b	a	a	b	b	a	b	a
b	b	b	a	a	b	b	a	b	a
a	-	b	a	a	-	b	a	b	a
a	-	-	a	a	a	b	a	b	a
a	b	b	-	a	a	b	a	b	a
a	b	b	a	a	a	b	a	b	a
a	b	b	a	-	a	b	a	b	b
b	b	b	-	a	a	b	a	b	b
a	b	b	a	b	a	b	a	b	b
a	b	b	a	a	a	b	a	b	b
a	b	a	a	a	a	b	a	b	b
a	-	b	a	a	a	b	a	a	b
-	a	b	b	a	a	a	a	a	b
b	a	b	b	a	a	a	a	a	b
b	a	b	b	a	a	a	a	a	b
b	a	b	-	a	a	a	a	a	b
b	a	b	b	a	a	a	a	a	b

b	a	b	b	a	a	a	a	a	b
b	a	b	b	-	a	a	a	a	b
b	a	b	b	a	a	a	a	a	b
b	a	b	b	-	a	a	b	a	b
b	a	b	b	-	a	a	b	a	b
b	a	b	-	a	a	a	b	a	b
a	a	b	b	a	a	a	b	a	b
b	a	b	b	a	a	a	b	a	b
-	a	b	b	a	a	a	b	a	b
b	a	b	b	-	a	a	b	a	b
a	a	b	b	a	a	a	b	a	b
b	a	b	b	-	a	-	b	a	b
a	a	a	a	a	b	b	a	a	b
a	a	a	a	a	b	b	a	a	b
a	a	a	a	a	b	b	a	a	b
a	a	a	a	a	b	b	a	a	b
a	a	a	a	a	b	b	a	a	b
a	a	a	a	a	b	b	a	a	b
a	a	a	a	a	b	b	a	a	a
a	a	a	a	a	b	b	a	b	a
a	a	a	a	a	b	b	a	b	a
a	a	b	a	a	b	b	a	b	a
a	a	b	a	a	b	b	a	b	a
a	-	b	a	-	b	b	a	b	a
a	a	b	a	-	b	b	a	b	a
a	a	b	a	a	b	a	a	b	a
a	a	a	a	a	b	a	a	b	a
a	a	b	a	b	b	a	a	b	a
a	a	b	a	b	b	a	a	b	a
b	a	b	a	b	b	a	b	b	a
a	a	b	a	b	b	a	b	b	a
b	a	b	a	b	a	b	b	b	a
b	a	b	a	b	a	b	b	b	a
b	a	b	a	b	a	b	b	b	a
b	a	b	a	b	a	b	b	b	a
a	a	-	a	b	a	b	b	b	b
b	a	b	a	b	-	b	b	b	b
b	a	b	a	b	a	b	b	b	b
b	a	b	a	b	a	b	b	b	b
b	a	b	a	b	a	b	b	b	b
b	a	b	a	b	a	b	b	b	b
-	a	b	a	b	a	b	b	b	b
b	a	b	a	-	a	b	b	b	b
b	a	b	a	b	a	b	b	b	b
b	a	b	a	b	a	b	b	b	b
-	a	b	a	b	a	b	b	b	b
b	a	b	a	b	a	b	b	b	b

a	a	b	a	b	a	b	b	b	b
a	a	b	a	b	a	b	b	b	b
a	a	b	a	-	a	b	b	b	b
-	a	b	a	-	a	b	b	b	b
a	a	b	a	a	a	b	b	b	b
a	a	-	a	a	a	b	b	b	b
a	a	b	b	a	a	b	b	b	b
a	a	b	b	a	-	b	b	b	b
a	-	b	b	a	-	b	b	b	b
a	a	-	b	a	a	b	b	b	b
a	a	b	b	a	a	b	b	b	b
a	a	b	b	a	a	b	b	b	b
a	a	b	b	a	a	b	b	b	b
a	a	b	b	a	a	b	b	b	b
a	a	b	b	a	a	b	b	b	b
a	a	b	-	a	a	b	b	b	b
a	a	b	b	a	a	b	b	b	b
a	-	b	b	-	a	b	b	b	b
a	a	b	b	a	b	b	b	b	b
a	a	b	b	a	b	b	b	b	b
a	a	b	b	a	b	b	b	b	b
a	a	b	b	a	b	b	b	b	b
a	a	b	b	a	b	b	a	b	b
a	a	b	b	a	b	b	a	b	b
a	a	b	b	a	-	-	a	b	b
a	a	b	b	a	b	b	a	b	b
a	a	b	b	a	b	b	a	b	b
a	a	b	b	b	b	b	a	b	b
a	a	b	b	b	-	b	a	b	b
a	a	-	b	b	b	b	a	b	b
-	a	b	b	b	b	b	a	b	b
a	a	b	b	b	b	b	a	b	b
a	a	b	b	b	b	b	a	b	b
a	a	b	a	a	b	b	a	b	b
a	a	b	-	-	b	b	a	b	b
a	a	b	b	b	b	b	a	b	b
a	a	b	b	b	b	b	a	b	b
a	a	b	-	b	b	b	a	b	b
a	a	b	-	b	b	b	a	b	b
a	a	-	b	-	b	b	a	b	b
a	a	-	-	b	b	b	a	b	b
-	b	b	b	b	b	b	a	b	b
a	b	b	b	-	b	b	a	b	b
a	b	b	b	b	b	b	a	b	b
a	b	b	b	b	b	b	a	b	b
a	b	-	b	-	b	b	a	b	b
a	b	-	-	b	b	b	a	b	b

a	b	b	b	b	b	b	a	b	b
b	b	b	b	b	b	b	a	b	b
a	b	b	b	b	b	b	a	b	b
a	b	b	b	-	b	b	a	b	b
a	b	b	b	b	b	b	a	b	b
a	b	b	a	b	b	b	a	b	b
a	b	b	-	b	b	b	a	b	b
a	b	b	a	b	b	b	a	b	b
a	b	b	a	b	-	b	a	b	b
a	b	b	a	b	b	b	a	b	b
a	b	b	a	b	b	b	a	b	b
a	b	-	a	b	b	b	a	b	b
a	b	b	a	b	b	b	a	b	b
b	-	b	a	b	b	b	a	b	-
b	b	b	a	b	b	b	a	b	b
b	b	b	a	b	b	b	a	b	a
b	b	b	a	b	b	b	a	b	a
-	b	b	a	b	b	b	a	b	a
b	b	b	a	b	b	b	a	b	a
b	b	b	a	b	b	b	a	b	a
b	b	b	a	b	b	b	a	b	a
b	b	b	a	b	b	b	a	b	a
b	b	b	a	b	b	b	a	b	a
b	b	b	-	b	b	b	a	b	a
b	b	b	a	b	b	b	a	b	a
b	b	b	a	b	-	b	a	b	a
b	b	b	a	b	b	b	a	b	a
b	b	b	a	b	b	b	a	b	a
-	b	b	a	a	b	b	a	b	a
b	b	b	a	b	b	b	a	b	a
b	b	b	a	b	b	b	a	b	a
b	-	b	a	b	b	b	a	b	a
b	b	b	a	b	b	-	a	b	a
b	b	b	a	b	b	-	a	b	a
-	-	b	a	-	b	b	a	b	a
b	b	b	a	b	b	b	a	b	a
b	b	b	a	b	b	b	a	b	a
b	b	b	-	b	b	b	a	b	-
a	a	a	b	a	a	-	a	a	a
a	a	a	b	a	a	b	a	a	a
a	a	a	b	a	a	b	a	a	a
a	a	a	b	a	a	b	a	a	a
a	a	a	b	-	a	b	a	a	a
a	a	a	b	a	a	b	a	a	a
a	a	a	b	a	a	b	a	a	a
a	a	a	b	a	a	b	a	a	a
a	a	a	b	a	a	b	a	a	a

a	-	a	b	a	a	b	a	a	a
a	a	a	b	a	a	b	a	a	a
b	a	a	b	a	a	b	a	a	a
b	a	a	b	a	a	b	a	a	b
b	a	a	b	a	a	b	a	a	b
b	a	a	b	a	a	b	a	a	b
b	a	a	b	a	a	b	a	a	b
b	a	a	b	a	a	b	a	a	b
b	a	a	b	a	a	b	a	a	b
b	a	a	b	a	a	b	a	a	b
b	b	a	b	a	b	b	a	a	b
b	b	a	b	a	b	b	a	a	b
b	b	a	b	a	b	b	a	a	b
b	b	a	b	a	b	b	a	a	b
b	b	a	b	-	b	b	-	a	b
b	b	a	b	a	b	b	a	a	b
b	b	-	b	a	b	b	a	a	b
-	b	a	b	a	b	b	a	a	b
b	b	-	b	a	b	b	a	a	b
b	b	a	a	a	b	b	a	a	b
-	b	a	a	a	b	b	b	a	b
b	b	a	a	a	b	b	b	a	b
b	b	-	a	a	b	b	b	a	b
b	b	a	a	a	b	b	b	a	b
b	b	a	a	a	b	b	b	a	b
b	b	b	a	a	a	b	b	a	b
-	b	a	a	a	a	b	-	a	b
-	b	a	a	a	a	b	b	a	b
b	b	-	a	a	a	b	b	a	a
b	b	a	a	a	a	b	b	a	a
b	b	a	a	a	a	b	b	a	a
b	b	-	a	a	a	b	b	a	a
b	b	a	a	a	a	b	b	a	a
b	b	a	a	a	a	b	b	a	a
b	b	a	-	a	a	b	b	a	a
b	b	a	a	a	a	a	b	a	a
b	b	a	a	a	a	b	b	a	a
b	b	a	a	-	a	b	b	a	a
b	b	a	a	b	a	b	b	a	a
b	b	a	a	b	a	b	b	a	a
a	b	a	a	b	a	b	b	a	a
a	b	a	a	b	a	-	b	a	a
b	a	a	a	b	a	b	b	a	a
b	b	a	a	b	a	b	b	a	a
b	b	a	a	b	a	b	b	a	a
-	b	a	a	b	a	b	b	a	a
b	a	a	a	b	a	b	b	a	a

b	a	a	a	b	a	b	b	a	a
a	a	a	a	-	a	b	b	a	a
a	a	a	a	b	a	b	b	a	a
a	a	a	a	b	-	b	b	a	a
a	a	a	a	b	a	b	b	a	a
a	a	a	a	b	a	b	b	a	a
a	a	a	a	b	a	b	b	a	b
a	a	a	a	a	a	b	b	a	b
a	a	a	a	a	a	-	b	a	b
a	a	a	a	a	a	b	a	a	b
a	a	a	a	a	a	b	a	a	b
a	a	a	a	a	a	b	a	a	b
a	a	a	a	a	a	b	a	a	b
a	a	a	a	a	a	b	a	a	b
a	a	a	a	a	a	b	a	a	b
a	a	a	a	-	a	b	a	a	b
a	a	a	a	-	-	b	a	a	b
-	a	-	a	a	a	b	a	a	b
a	a	a	a	a	a	b	a	a	b
a	a	a	a	a	a	b	a	a	b
a	a	a	a	a	a	b	a	a	b
a	a	a	a	a	a	b	a	a	b
a	a	a	a	a	a	b	a	a	b
a	a	-	a	b	a	b	a	a	b
a	a	a	a	b	a	b	a	a	b
a	a	a	a	b	a	b	a	a	b
a	a	a	a	b	a	b	a	a	b
a	a	a	a	-	a	b	a	a	b
a	a	a	a	b	a	b	a	a	b
a	a	a	a	b	a	-	a	a	b
a	a	a	a	b	a	b	a	a	b
a	a	a	b	-	a	b	a	a	b
-	a	a	b	b	a	b	a	a	b
-	a	a	b	b	a	b	a	a	b
a	a	a	b	b	a	b	a	a	b
a	a	a	b	b	a	b	a	a	b
a	a	a	b	b	a	-	a	a	b
b	a	-	b	b	a	b	a	a	b
a	a	a	b	b	a	b	a	a	b
a	a	a	b	-	a	-	a	a	b
-	a	a	-	a	b	b	b	a	b
b	a	a	a	a	b	b	b	a	b
b	a	a	a	a	b	b	b	a	b
b	a	a	a	a	b	b	b	a	b
b	b	a	a	a	b	b	b	a	b
b	b	a	a	a	b	b	b	a	b
b	b	a	a	a	-	b	b	a	b

b	b	a	a	a	-	b	b	a	b
a	b	a	a	a	b	b	b	a	b
b	b	a	a	a	b	b	b	a	b
b	b	a	a	a	b	b	b	a	b
b	b	a	-	a	b	b	b	a	b
b	b	-	a	a	b	b	b	a	b
b	b	a	a	a	b	b	b	a	b
b	b	a	a	a	b	b	b	a	b
-	b	a	a	a	b	b	b	a	b
b	b	a	a	a	b	-	b	a	b
b	b	a	a	-	b	b	b	a	b
b	b	-	a	a	b	b	b	a	b
b	b	-	a	a	b	b	b	a	b
b	b	a	-	-	b	b	b	a	b
-	b	a	a	a	b	b	b	a	b
b	b	-	a	a	b	b	b	a	b
b	b	-	a	-	b	b	b	a	b
b	b	a	a	a	b	b	b	a	b
b	b	a	a	a	b	b	b	a	b
b	b	a	a	a	b	b	b	a	b
b	b	a	a	a	b	b	b	a	b
b	b	a	a	a	b	b	b	a	b
b	b	a	a	a	b	b	b	a	b
b	b	a	a	a	b	b	b	a	b
b	b	a	a	a	b	b	b	a	b
b	b	a	a	a	b	b	b	a	b
b	-	a	a	a	b	b	-	a	b
b	b	a	a	a	b	b	b	a	b
b	b	a	a	a	-	-	a	a	b
b	b	a	a	a	b	b	a	a	b
b	b	a	a	a	b	b	a	a	b
b	b	a	a	a	b	-	a	a	b
a	b	a	a	a	b	b	a	a	b
a	b	a	a	a	b	b	a	a	b
-	b	a	a	a	-	b	a	a	b
a	b	a	a	a	b	b	a	a	b
b	b	-	a	a	b	b	a	a	b
b	b	a	a	a	b	b	a	a	b
b	b	a	a	a	b	b	a	a	b
b	b	a	a	a	b	b	a	a	b
b	b	a	a	a	b	b	a	a	b
b	b	a	a	a	b	b	a	a	b
b	b	a	a	a	b	b	a	a	b
a	b	a	a	a	b	b	a	a	b
b	b	a	a	a	b	b	a	a	b
b	b	a	a	a	b	b	a	a	b
b	b	a	a	a	b	-	a	a	b

b	b	-	a	a	b	b	a	a	b
b	b	a	a	a	b	b	a	a	a
b	b	a	a	a	b	b	a	a	a
-	b	a	a	a	-	b	a	a	b
b	b	a	a	a	b	b	a	a	b
a	b	a	a	a	b	-	a	a	b
b	b	a	-	-	b	b	a	a	b
b	b	a	a	a	b	b	a	a	b
b	b	a	a	a	b	b	a	a	b
b	b	a	a	a	b	b	a	a	b
b	b	a	a	a	b	b	a	a	b
b	b	a	a	a	b	-	a	a	b
b	b	a	a	a	b	b	a	a	b
b	b	a	a	a	b	b	a	a	b
b	b	a	a	a	b	b	a	a	b
b	b	b	a	a	b	b	a	a	b
b	b	b	a	a	b	b	a	a	b
b	b	b	a	a	b	b	a	a	b
b	b	b	a	a	b	b	a	a	b
b	b	b	a	a	b	b	a	a	b
b	b	b	a	-	b	b	a	a	b
b	b	b	a	a	b	b	a	a	b
b	b	b	-	a	b	b	a	a	b
b	b	b	a	a	b	b	a	a	b
b	b	b	a	a	b	b	a	a	b
b	b	b	a	a	b	b	a	a	b
b	b	b	b	a	b	b	a	a	b
b	b	b	a	a	b	b	a	a	b
-	b	b	a	a	b	b	a	a	b
b	b	b	a	a	b	b	a	a	b
b	b	-	a	a	-	b	a	a	b
b	b	a	a	a	b	b	a	a	b
b	b	a	a	a	b	b	a	a	a
a	-	a	a	a	b	b	a	a	a
b	b	a	b	a	b	b	a	a	a
b	a	a	-	a	b	b	-	a	a
b	a	a	a	a	b	b	a	a	a
a	a	a	a	-	b	b	a	a	a
a	a	a	a	a	b	b	a	a	a
a	a	a	a	a	b	b	a	a	a
a	a	a	a	a	b	b	a	a	a
a	a	a	a	a	-	b	a	a	a
a	a	a	a	a	b	b	a	a	a
a	a	a	a	a	b	-	a	a	a
a	a	a	a	a	b	b	a	a	a
a	a	a	a	-	b	b	a	a	a
a	a	a	a	a	b	b	a	a	a

a	a	a	-	a	b	b	a	b	a
a	a	a	a	a	b	b	a	b	a
a	a	a	a	a	b	b	a	b	a
a	a	a	a	a	b	b	a	b	a
-	a	a	a	-	b	b	a	b	a
a	a	-	a	a	b	b	b	b	a
a	a	a	a	a	b	b	b	b	a
-	-	-	a	a	b	b	b	b	a
-	-	a	a	a	b	b	b	b	a
a	a	a	a	-	b	b	b	b	a
a	a	a	a	a	b	b	b	b	a
a	a	a	-	a	b	b	b	b	a
a	a	a	a	a	b	b	b	b	a
a	a	a	a	a	b	b	b	b	a
a	a	a	a	a	b	b	b	b	a
a	a	a	a	a	b	b	b	b	a
a	a	a	a	a	b	b	b	b	a
a	a	a	a	a	b	b	b	b	a
a	a	a	a	a	b	b	b	b	a
b	-	b	b	a	b	b	a	b	b
b	b	b	b	-	b	b	a	b	b
b	-	b	b	a	b	b	a	b	b
-	b	a	b	a	b	-	a	b	b
b	b	b	b	a	b	b	a	b	b
b	b	b	b	a	b	-	a	b	b
b	b	b	b	a	b	b	a	b	b
b	b	-	b	a	b	b	a	b	b
b	b	b	b	a	b	b	a	b	b
-	b	b	b	a	b	b	a	b	b
b	b	b	b	a	b	b	a	b	b
b	b	-	b	a	b	-	a	b	b
-	b	b	-	a	b	b	a	b	b
b	b	b	b	a	b	b	a	b	b
b	-	b	b	a	b	b	a	b	b
b	b	b	b	-	b	b	a	b	b
-	b	-	b	a	b	-	a	b	b
-	b	-	b	a	b	b	a	b	b
b	b	b	b	a	b	b	a	b	b
b	b	-	b	a	a	b	a	b	b
b	b	b	b	a	a	b	a	b	b
b	b	b	b	a	a	b	a	b	b
b	b	b	b	a	a	b	a	b	b
b	b	b	b	a	a	b	a	b	b
b	-	b	b	a	a	b	a	b	b
b	b	b	b	a	a	b	a	b	b
b	b	b	b	a	a	b	a	b	b
b	b	b	b	-	a	b	a	b	b

b	b	b	b	a	a	b	a	b	b
-	b	b	b	a	a	b	a	b	b
-	b	b	b	a	a	-	a	b	b
b	b	b	b	a	a	b	a	b	b
b	b	a	b	a	a	b	a	b	b
b	b	-	b	a	a	b	a	b	b
a	b	b	b	a	a	b	a	b	b
b	-	b	b	a	a	b	a	b	b
b	b	b	b	a	a	b	a	b	b
b	b	b	b	a	a	b	a	b	b
b	b	b	b	a	a	b	a	b	b
b	b	b	b	a	a	b	a	b	b
b	b	b	b	b	a	b	a	b	b
b	b	b	b	b	a	b	a	a	-
b	b	b	b	b	a	-	a	a	b
b	b	b	b	b	a	b	a	a	b
b	b	b	b	b	-	b	a	a	b
b	b	b	b	b	a	b	a	a	b
b	b	b	-	b	a	b	a	a	b
b	b	b	b	b	a	b	a	a	b
b	b	b	b	b	a	b	a	a	b
b	b	-	b	b	a	b	a	a	b
a	b	a	b	b	a	b	a	a	b
b	b	b	b	b	a	b	a	a	b
b	b	b	b	b	a	b	a	a	b
b	b	b	b	b	a	b	a	a	b
b	b	b	b	b	a	a	a	a	b
b	b	-	b	-	a	a	a	a	b
b	b	b	b	b	a	a	a	a	b
a	-	b	b	b	a	a	a	a	b
b	b	b	b	b	a	a	a	a	b
b	b	a	b	b	a	a	a	a	b
b	b	a	b	b	a	a	a	a	b
b	b	-	b	b	-	a	a	a	b
b	b	a	b	-	a	a	a	a	b
b	b	a	b	b	a	a	a	a	b
b	a	a	a	b	a	a	a	a	b
b	a	a	a	b	a	a	a	a	b
b	a	a	a	b	a	a	a	a	b
a	a	a	a	b	a	a	a	a	b
b	a	a	a	b	a	a	a	a	b
-	a	a	a	-	a	a	a	a	b
b	a	a	a	b	a	a	a	a	b
b	a	a	a	-	a	a	a	a	b
-	a	a	a	b	-	a	a	a	b
b	a	a	a	b	b	a	a	a	b
b	a	a	a	b	b	a	a	a	b

b	a	a	a	b	b	a	a	a	b
a	a	a	a	b	b	a	a	a	b
a	a	a	a	b	b	a	a	a	b
a	a	a	a	b	b	a	a	a	b
a	a	a	a	b	b	a	a	a	b
a	a	a	a	b	b	a	a	a	b
a	a	a	a	-	b	a	a	a	b
a	a	a	a	a	b	a	a	a	b
a	a	a	a	a	b	a	a	a	b
a	a	a	a	a	b	a	a	a	b
b	a	a	a	a	b	a	a	a	b
b	b	b	b	b	a	a	b	a	a
b	b	b	b	b	-	a	b	a	a
b	a	b	b	b	-	-	b	a	a
b	a	b	b	-	a	a	b	a	a
b	a	b	b	b	a	a	b	a	a
b	a	b	b	b	a	a	b	a	a
b	a	b	b	b	a	a	b	a	a
b	a	b	b	-	a	a	b	a	a
-	a	b	-	b	a	a	b	a	a
b	a	b	b	b	a	a	b	a	a
b	a	b	b	b	a	a	b	a	a
a	a	b	b	-	a	a	b	a	a
b	a	b	b	b	a	a	b	a	a
b	a	b	b	b	a	a	b	a	a
b	a	b	b	b	a	a	b	a	a
b	a	b	b	b	a	a	b	a	a
-	a	b	b	b	a	a	b	a	a
b	a	b	b	b	a	a	b	a	a
b	a	b	b	b	a	a	b	a	b
b	a	b	b	a	a	a	b	b	b
b	a	b	b	-	a	a	b	b	b
b	a	b	b	a	a	a	b	b	b
b	a	b	b	a	a	a	b	b	b
b	a	b	b	a	a	a	b	b	b
b	a	b	b	a	a	a	b	b	b
b	a	b	-	a	a	a	b	b	b
b	a	b	b	a	a	a	b	b	b
b	a	b	b	a	a	a	b	b	b
b	a	b	b	a	a	a	b	b	b
b	a	-	b	a	a	a	b	b	b
b	a	b	b	a	a	a	b	b	b
b	a	b	b	a	a	a	b	b	b
b	a	b	b	a	a	a	b	b	b
-	a	b	b	a	a	a	b	b	b
b	a	b	b	a	a	a	b	b	b
b	a	b	b	a	a	a	b	b	b

b	a	b	b	a	-	a	b	b	b
b	a	b	b	a	a	a	b	b	b
-	a	b	b	a	a	a	b	b	b
-	a	b	b	a	a	a	b	b	b
b	a	b	b	a	a	a	b	b	b
b	a	b	b	a	a	b	b	b	a
a	a	b	b	a	a	b	b	b	a
b	a	-	b	a	a	-	b	b	a
b	a	b	b	-	a	b	b	b	a
b	a	b	b	a	a	b	b	b	a
b	a	b	b	a	a	-	b	b	a
b	a	b	b	a	a	b	b	b	a
b	a	b	b	a	a	b	b	b	a
a	a	-	-	a	a	b	b	b	a
b	a	b	b	a	a	b	b	b	a
b	a	b	-	a	a	b	b	b	a
b	a	b	b	a	a	b	b	b	a
b	a	b	b	a	a	b	b	b	a
b	a	a	b	a	a	b	b	b	a
b	a	b	b	a	a	b	b	b	a
b	a	b	b	a	a	b	b	b	a
-	a	b	b	a	a	b	b	b	a
b	a	b	b	a	a	b	b	b	a
b	a	-	b	a	a	b	b	b	a
b	a	b	b	a	a	b	b	b	a
b	a	b	b	a	a	b	b	b	a
-	a	b	b	a	a	b	b	b	a
b	a	b	b	a	a	b	b	b	a
b	a	b	b	a	a	b	b	b	a
b	a	b	b	a	b	b	b	b	a
a	a	-	b	a	b	b	b	b	a
b	a	b	b	a	b	b	b	b	a
b	a	b	b	a	b	b	b	b	a
-	a	b	b	a	-	-	b	b	a
b	a	b	b	a	b	b	b	b	a
b	a	b	b	-	b	b	b	b	a
b	a	b	b	a	b	b	b	b	a
b	a	b	b	a	b	b	b	b	a
b	a	b	b	a	b	b	b	b	a
b	a	b	b	a	b	b	b	b	a
b	a	b	b	a	b	b	b	b	a
b	a	b	b	a	b	b	b	b	a
b	a	b	b	a	b	b	b	b	a
b	a	a	b	a	b	b	b	b	a
b	a	a	b	a	b	a	b	b	a
b	a	a	b	a	b	b	b	b	a
b	a	a	b	a	b	b	b	b	a

b	a	a	b	a	b	-	b	b	a
b	a	a	b	a	b	b	b	b	a
b	a	-	b	a	b	b	b	b	a
-	a	a	b	a	b	-	b	b	a
b	a	a	b	-	b	b	b	b	a
b	a	a	b	-	b	b	b	b	a
b	a	-	b	a	b	b	b	b	a
b	a	a	b	a	b	b	b	b	a
b	a	a	b	a	b	b	b	b	a
b	a	a	b	a	b	b	b	b	a
b	a	a	b	a	b	b	b	b	a
-	a	a	b	a	b	-	b	b	a
b	a	a	b	a	b	b	b	b	a
b	a	a	b	a	b	b	b	b	a
b	a	a	b	a	b	b	b	b	a
b	a	a	b	a	b	b	b	b	a
b	a	a	b	a	b	b	b	b	a
a	a	a	b	-	b	b	b	b	a
a	-	-	b	a	b	b	b	b	a
b	a	b	b	a	b	b	b	b	a
a	a	a	b	a	b	b	b	b	a
a	a	a	b	-	b	b	b	b	a
a	a	b	b	b	b	a	a	a	b
-	a	b	b	b	b	a	a	a	b
a	a	b	b	b	b	a	a	a	b
a	a	b	b	b	b	a	a	a	b
a	a	b	b	b	b	a	a	a	b
a	a	b	b	b	b	a	b	a	b
a	a	b	b	b	-	a	b	a	b
a	a	b	b	b	b	a	b	a	b
-	-	b	b	b	b	a	b	a	b
a	a	b	-	b	b	a	b	a	b
a	a	b	b	b	b	a	b	a	b
a	a	-	b	-	b	a	b	a	b
-	a	b	b	b	b	a	b	a	b
a	b	b	b	b	a	a	b	a	b
a	b	-	b	b	a	a	b	a	b
a	b	b	b	b	-	a	b	a	b
a	b	b	b	b	a	a	b	a	b
a	b	b	b	b	a	a	b	a	b
a	b	b	b	b	-	a	b	a	b
a	b	b	b	b	a	a	b	a	b
a	b	b	b	b	a	a	b	a	b
a	b	b	b	-	a	a	b	a	b
a	b	b	b	b	a	a	b	a	b
a	b	b	b	b	a	a	b	a	b
a	b	b	b	b	a	a	b	a	b

-	b	b	b	b	a	a	b	a	a
b	b	b	b	b	a	a	b	a	a
b	b	b	b	b	a	-	b	a	a
b	-	-	b	b	a	a	b	a	a
b	b	b	b	b	a	a	b	a	a
b	b	b	b	b	a	a	b	a	a
b	-	-	b	b	a	a	b	a	a
-	b	b	b	b	a	a	b	a	a
b	b	b	b	b	a	a	b	a	a
b	b	b	b	b	a	a	b	a	a
b	b	a	b	b	a	a	b	a	a
b	b	a	b	b	a	a	b	a	a
b	b	a	b	b	a	a	b	a	a
b	b	a	-	b	a	a	b	a	a
b	b	a	b	b	a	a	b	a	a
b	b	a	b	b	a	a	b	a	a
b	b	a	b	b	a	a	b	a	a
b	b	a	-	b	a	-	b	a	a
b	b	a	b	b	a	a	b	a	a
b	b	a	b	b	a	a	b	a	a
b	b	a	b	b	a	a	b	a	a
b	b	-	b	b	a	a	b	a	a
b	b	a	b	b	a	a	b	a	a
b	b	a	b	b	a	a	b	a	a
b	b	a	b	b	a	a	b	a	a
b	b	b	b	b	a	a	b	a	a
b	b	b	b	b	a	a	b	a	a
b	b	b	b	b	a	a	b	a	a
b	b	b	b	b	a	a	b	a	a
b	b	b	b	b	a	a	b	a	a
b	b	b	b	b	-	a	b	a	a
b	-	b	-	b	a	a	b	a	a
b	b	b	b	b	a	a	b	a	a
b	b	b	b	b	a	a	b	a	a
b	b	b	b	b	a	a	b	a	a
b	b	b	b	b	a	a	b	a	a
b	b	b	b	b	a	a	b	a	a
b	b	b	b	b	a	a	b	a	a
b	b	b	b	b	a	a	b	a	a
b	b	b	b	b	a	a	b	a	a
-	b	-	b	b	a	a	b	a	a
b	b	b	b	-	a	a	b	a	a
b	b	-	b	b	a	a	b	a	a
b	b	b	b	b	a	a	b	a	a
b	b	b	b	b	a	a	b	a	a
b	b	b	b	b	a	a	b	a	a
b	b	b	b	b	a	a	b	a	a

b	b	b	b	b	a	a	b	a	a
b	b	b	b	b	a	a	b	a	a
b	b	b	b	b	a	a	b	a	a
b	b	-	b	-	a	a	b	a	a
b	b	b	b	b	a	a	b	a	a
b	b	b	b	b	a	a	b	a	a
b	b	b	b	b	-	a	b	a	a
b	b	b	b	b	a	a	b	a	a
b	b	b	b	b	a	a	b	a	a
b	b	b	b	b	-	a	b	a	a
-	b	b	b	b	a	a	b	a	a
b	b	b	b	b	a	a	b	a	b
b	b	-	b	b	a	a	b	a	b
b	b	b	b	b	a	a	b	a	b
b	b	b	b	b	a	a	b	a	b
b	b	b	b	b	a	a	b	a	b
b	b	b	b	b	a	a	b	a	b
b	b	b	b	b	a	a	b	a	b
b	b	b	b	b	a	a	b	a	b
b	b	b	b	b	a	a	b	a	b
b	b	b	b	b	a	a	b	a	b
b	b	b	b	-	a	a	b	a	b
b	b	b	-	-	a	a	b	a	b
b	b	b	b	b	a	a	b	a	b
b	b	b	b	b	a	-	b	b	b
b	b	b	b	b	a	a	b	b	b
b	b	b	b	b	a	a	b	b	b
b	-	b	b	b	a	a	b	a	b
b	b	b	b	b	a	a	b	b	b
b	b	b	b	b	a	a	b	b	b
b	b	b	b	b	a	a	b	b	b
b	b	b	b	b	a	a	b	b	b
b	b	b	b	b	a	a	b	b	b
b	b	b	b	b	a	a	b	b	b
a	-	b	b	-	a	a	b	b	b
b	b	b	b	b	a	a	b	b	b
b	b	b	b	b	a	a	b	b	b
b	b	b	b	-	a	a	b	b	b
b	b	b	b	-	a	a	b	b	b
-	b	b	b	b	a	a	b	b	b
b	b	b	b	b	a	a	b	b	b
a	b	b	b	b	a	a	b	b	b
a	b	b	b	b	a	a	b	b	b
b	b	b	b	b	a	a	b	b	b
b	b	b	b	b	a	a	b	b	b
b	b	b	b	b	a	a	b	b	b
a	b	-	b	-	a	-	b	b	b

b	b	b	b	b	a	a	b	b	b
-	-	b	-	b	a	a	b	b	b
b	b	b	b	b	a	a	b	b	b
b	a	b	b	b	b	a	a	a	b
b	a	b	b	b	b	a	a	a	b
b	b	b	b	b	b	a	a	a	b
b	b	a	a	b	b	a	a	a	a
b	a	b	b	b	b	a	b	a	a
b	a	b	b	b	b	a	b	a	a
b	a	b	b	b	-	a	b	a	a
b	a	b	b	b	b	a	b	a	a
b	a	b	b	b	b	a	b	a	a
b	a	b	b	b	b	a	b	a	a
b	a	b	b	-	b	a	b	a	a
b	a	b	b	b	b	a	b	a	a
b	a	b	b	-	b	b	b	a	a
b	a	b	b	b	b	b	b	a	a
b	a	b	b	b	b	b	b	a	a
b	a	b	b	b	b	b	b	a	a
b	a	b	b	b	b	b	b	a	a
b	-	b	b	b	b	b	b	a	a
-	a	b	-	b	b	b	b	a	a
b	a	b	b	b	b	b	b	a	a
b	a	b	b	b	b	b	b	a	a
b	a	b	-	b	b	b	b	a	a
b	a	b	b	b	b	b	b	a	a
b	a	b	b	-	b	b	b	a	a
b	a	b	-	b	b	b	b	a	a
b	a	b	b	b	b	b	b	a	a
b	a	b	b	b	b	b	b	a	a
b	a	b	b	b	b	b	b	a	a
b	a	b	b	b	b	b	b	a	a
b	a	b	b	b	b	b	b	a	a
b	-	b	b	b	b	b	b	a	a
b	a	b	b	b	b	b	b	a	a
b	a	b	b	b	b	b	b	a	b
b	a	b	b	b	b	b	b	a	b
b	a	b	a	b	b	b	b	a	b
b	-	b	-	b	b	b	a	a	b
b	-	b	a	b	b	b	a	a	b
b	a	b	b	b	b	b	a	a	b
a	a	-	b	b	b	b	a	a	b
b	a	b	b	b	b	b	a	a	b
b	a	b	b	b	a	b	a	a	b
b	a	b	b	b	a	b	a	a	b
b	a	b	b	b	a	b	a	a	b
b	a	b	b	b	a	b	a	a	b

b	a	b	b	b	a	b	a	-	b
b	a	b	-	b	a	-	a	a	b
b	b	b	b	b	a	b	a	a	b
b	b	b	b	b	a	b	a	a	b
b	a	-	a	a	a	a	a	a	a
-	a	b	b	a	a	a	a	a	a
b	a	b	b	a	a	a	a	a	a
b	a	-	b	a	a	a	a	a	a
b	a	b	b	a	a	a	a	a	a
b	a	b	b	a	a	a	a	a	a
b	a	b	b	a	a	a	a	a	a
b	a	-	b	a	a	a	a	a	a
b	b	b	b	-	a	b	a	a	b
b	b	b	b	b	b	b	a	a	b
b	b	a	b	b	b	-	a	a	b
b	b	a	b	b	b	b	a	a	b
b	b	a	b	b	b	b	a	a	b
b	b	a	b	b	b	b	a	a	b
b	b	a	b	b	b	b	a	a	b
b	b	a	b	b	-	b	a	a	b
b	b	a	b	b	b	b	a	a	b
b	b	-	b	b	b	b	a	b	b
b	b	a	b	b	b	b	a	b	b
b	b	a	b	b	b	b	a	b	b
b	b	a	b	b	b	b	a	b	b
b	b	a	b	b	b	a	a	a	b
b	b	a	b	b	b	a	a	a	b
b	b	a	b	b	b	a	a	a	b
b	b	a	-	b	b	a	a	a	b
b	a	a	b	b	b	a	a	a	b
b	a	a	b	b	b	a	a	a	b
b	a	a	b	b	b	a	a	a	b
b	a	a	b	b	b	a	a	a	b
-	a	a	-	-	b	a	a	a	b
b	a	a	b	b	b	a	a	a	b
b	a	a	b	b	b	a	a	a	b
b	a	a	b	b	b	a	a	a	b
b	a	a	b	b	b	a	a	a	b
b	a	a	b	b	b	a	a	a	b
b	a	a	b	b	b	-	a	a	b
b	a	a	b	b	b	a	a	a	b
b	a	b	b	a	b	a	a	a	b
b	a	b	b	a	b	a	a	a	b
b	a	b	b	a	b	a	a	a	b
b	a	b	a	a	a	a	a	a	b
a	a	-	a	b	b	b	a	a	a
-	-	a	a	b	b	b	a	a	a

a	a	a	a	b	b	b	a	a	a
a	a	a	a	-	-	b	a	a	a
a	a	a	a	-	b	b	a	a	a
a	a	a	a	b	b	b	a	a	a
a	a	a	a	b	b	b	a	a	a
a	a	a	a	b	b	b	-	a	a
a	a	a	a	b	b	b	a	a	a
a	a	a	a	b	b	-	a	a	a
a	a	a	a	b	-	b	a	a	a
b	a	a	a	b	b	b	a	a	a
b	a	a	a	b	b	b	a	a	a
b	a	a	a	-	b	b	a	a	a
b	a	a	a	b	b	b	a	a	a
b	a	a	a	b	a	b	a	a	a
b	a	a	a	b	a	b	a	a	a
b	a	a	b	b	a	b	a	a	a
b	a	a	b	b	a	b	a	a	a
b	a	a	b	b	a	b	a	a	a
b	a	a	b	b	a	b	a	a	a
b	a	a	b	b	a	b	a	a	a
b	a	-	b	b	a	b	a	a	a
b	-	a	b	b	a	b	a	a	a
b	a	a	b	b	a	b	a	a	a
b	a	a	b	b	a	b	a	a	a
b	b	a	b	b	a	b	a	a	a
b	b	a	a	b	a	b	a	a	a
b	b	a	b	b	a	b	a	a	a
b	b	a	b	b	a	b	a	a	a
b	-	a	b	b	a	-	a	a	a
b	b	a	b	b	a	b	a	a	a
b	b	a	b	b	a	-	a	a	a
b	b	a	b	b	a	b	a	a	a
b	b	a	b	b	a	b	a	a	a
b	b	a	b	b	b	b	a	a	a
b	b	a	b	b	b	b	a	a	a
b	b	a	b	b	b	b	a	a	a
b	b	a	b	b	b	b	a	a	a
b	b	a	b	b	b	b	a	a	a
b	b	a	b	b	b	-	a	a	a
b	b	a	b	b	b	b	a	a	a
b	b	a	b	b	b	b	a	a	a
b	b	a	b	b	b	b	a	a	a
b	b	a	b	b	b	b	a	a	a
b	b	-	b	b	b	b	a	a	a
b	b	a	b	b	b	b	a	a	a
b	b	a	b	b	b	b	a	a	a
b	b	a	b	b	b	b	a	a	a

b	b	a	b	b	b	b	a	a	a
-	b	a	b	b	b	b	a	a	a
b	b	a	b	b	b	b	a	a	a
-	b	a	-	b	-	b	a	a	a
b	b	a	b	b	b	b	a	a	a
b	b	a	b	b	b	b	a	a	a
b	b	a	b	b	b	b	a	a	a
-	-	a	-	b	b	b	a	a	a
b	b	a	b	b	b	b	a	a	a
b	b	a	b	b	b	b	-	a	a
b	b	a	b	b	b	b	a	a	a
b	b	a	b	b	b	b	b	a	a
b	b	a	b	b	b	b	a	a	a
-	-	a	-	a	b	b	a	a	a
b	b	a	b	b	b	b	a	a	a
b	b	-	b	b	b	b	a	a	b
b	b	a	b	b	b	b	a	a	b
a	b	a	b	b	b	b	a	a	b
a	b	a	b	b	b	b	a	a	b
a	b	a	b	b	b	b	a	a	b
a	b	a	b	b	b	a	a	a	b
a	b	a	b	b	b	a	a	a	b
a	b	-	b	b	b	-	a	a	b
a	b	a	b	b	b	a	a	a	b
a	b	a	b	b	b	a	a	a	b
a	b	a	b	b	b	a	a	-	b
a	b	a	a	b	b	a	a	a	b
a	b	a	a	b	b	a	a	a	a
a	b	a	a	-	b	a	a	a	a
a	b	a	a	b	b	a	a	a	a
a	b	a	a	b	b	a	a	a	a
a	-	a	a	b	b	a	a	a	a
-	b	a	a	b	b	a	a	a	a
a	b	-	a	b	b	a	a	a	a
a	-	a	a	b	b	a	a	a	a
a	b	a	a	b	b	a	a	a	a
a	b	-	a	b	b	a	a	a	a
a	b	a	a	b	b	a	a	a	a
a	b	a	a	b	b	a	a	a	a
b	b	a	a	b	-	a	a	b	a
b	b	a	a	b	b	a	a	b	a
b	b	a	a	b	b	a	a	b	a
b	b	a	a	a	b	a	a	b	a
b	b	a	a	a	b	a	a	b	a
b	b	a	a	a	b	-	a	b	a
b	b	a	a	a	b	a	a	b	a
b	b	a	a	a	b	a	a	b	a

b	b	a	a	b	b	a	a	b	a
b	b	a	a	b	b	a	a	b	a
b	b	a	a	b	-	a	a	b	a
b	b	a	a	b	b	a	a	b	a
b	b	a	a	b	b	a	a	b	a
b	b	a	a	b	b	a	a	b	a
b	b	-	a	b	b	-	a	a	a
a	b	a	a	b	b	a	a	b	a
b	a	b	-	b	b	a	b	b	b
b	a	b	-	b	b	a	b	b	b
b	a	b	a	b	b	a	b	b	b
b	a	b	a	b	b	a	b	b	b
b	a	b	a	b	b	a	b	b	b
b	a	b	a	b	b	a	b	b	b
b	a	b	a	b	b	a	b	b	-
b	a	b	a	b	b	a	b	b	b
b	-	b	a	b	b	a	b	b	b
b	-	b	a	b	b	-	b	b	b
b	a	b	a	b	b	-	b	b	b
a	a	b	a	b	b	a	b	b	b
a	a	b	a	b	b	a	b	b	b
a	a	b	a	b	b	a	b	b	b
a	a	b	a	b	b	a	b	b	b
b	a	b	a	b	a	-	b	b	b
a	a	b	a	b	a	a	b	b	b
a	a	b	a	b	a	a	b	b	b
a	a	b	a	b	a	a	b	b	b
a	a	b	-	b	a	a	b	b	-
a	a	b	a	b	a	a	b	b	a
a	a	b	a	b	b	a	b	b	a
b	a	b	a	b	b	a	a	b	b
b	a	b	a	b	b	b	a	b	b
b	a	b	a	b	b	b	a	b	b
b	a	b	-	b	b	b	a	b	b
-	a	b	a	b	b	b	a	b	b
b	a	b	b	b	b	b	a	a	b
-	a	b	b	a	b	b	a	a	b
b	a	b	b	b	b	b	a	a	b
b	a	-	b	b	b	b	a	a	b
-	a	b	-	b	b	b	a	a	b
b	a	b	b	b	b	b	a	a	b
b	a	b	b	b	b	b	a	a	b
b	a	-	b	b	b	b	a	a	b
a	a	b	a	a	a	b	a	a	b
a	a	b	a	a	a	b	a	a	b
a	a	b	a	a	a	b	a	a	b
b	a	b	a	a	a	b	a	a	b

a	a	b	a	a	a	b	a	a	b
a	a	-	a	a	a	b	a	a	b
a	a	b	a	a	a	b	a	a	b
a	a	b	a	a	a	-	a	a	b
a	a	b	a	a	a	b	a	a	b
a	a	b	a	a	a	b	a	a	b
a	a	b	b	a	a	b	a	a	b
a	a	b	-	a	a	b	a	a	b
a	b	b	b	a	a	a	a	a	b
a	b	b	b	a	a	a	a	a	b
a	b	-	b	a	a	a	a	a	b
a	b	b	b	a	a	a	a	a	b
a	b	b	b	-	b	a	b	a	a
a	b	b	b	a	b	a	b	a	a
a	b	b	b	a	b	a	b	a	a
b	b	b	b	a	b	a	b	a	a
b	b	b	b	a	b	a	b	a	a
b	b	b	-	a	b	a	b	a	a
b	b	b	-	-	b	a	b	a	a
b	b	b	b	b	b	a	b	a	a
a	a	b	b	-	b	a	b	a	a
b	-	b	b	b	b	-	b	a	a
b	a	b	b	b	b	a	b	a	a
b	a	b	b	b	b	a	b	a	a
b	a	b	b	b	b	a	b	a	a
b	a	b	b	b	b	a	b	a	a
b	a	b	b	b	b	a	b	a	a
b	b	a	b	b	a	a	b	a	a
b	b	a	b	b	a	a	b	a	a
b	b	a	a	b	a	a	b	a	a
b	b	a	a	b	a	a	b	a	a
b	b	a	a	b	a	a	b	a	a
b	b	b	a	b	a	a	b	a	a
b	b	a	a	b	a	a	b	a	a
a	b	a	a	b	a	a	b	a	a
b	b	a	a	b	a	a	b	a	a
b	b	a	a	b	a	a	b	a	a
b	b	a	a	b	a	a	b	a	a
b	b	-	a	b	a	a	b	a	a
b	b	a	a	b	a	a	a	a	a
b	b	b	a	b	-	a	a	a	a
b	b	b	a	b	a	a	a	a	a
b	b	b	a	b	a	a	a	a	a
b	b	b	a	b	a	a	a	a	a
b	b	b	a	b	a	b	a	a	b
b	b	b	b	a	a	a	a	a	a
b	b	b	b	a	a	a	a	a	b

b	b	b	b	a	a	a	a	a	b
b	b	b	b	a	-	a	a	a	b
a	-	b	b	a	a	a	a	a	b
b	b	a	b	a	-	a	b	b	b
b	b	a	b	a	a	a	b	b	b
b	b	a	b	-	b	a	b	b	b
b	b	-	b	b	b	a	b	b	b
b	b	a	b	b	b	a	b	b	b
a	a	b	b	b	b	b	b	a	b
a	a	b	b	b	b	b	b	a	b
a	a	b	b	b	b	b	b	a	b
a	a	b	b	b	b	b	-	a	b
a	a	b	b	b	b	b	b	a	b
a	a	b	b	b	b	b	b	a	b
b	a	b	b	b	b	b	b	a	b
a	a	b	b	b	b	b	b	a	b
a	a	b	b	b	b	b	b	a	b
a	a	b	b	b	b	b	b	a	b
a	a	b	b	b	b	b	-	a	b
a	a	-	b	b	b	b	b	a	b
a	a	b	a	b	b	b	b	a	b
a	a	b	a	b	b	b	b	a	b
a	a	b	a	b	b	b	b	a	b
-	a	b	a	b	b	b	b	a	b
a	a	b	a	b	b	-	b	a	b
-	a	b	-	b	b	b	b	a	b
a	a	b	a	b	b	b	b	a	b
a	a	-	a	b	b	b	b	a	b
a	a	b	a	a	b	b	b	a	b
a	a	b	a	a	a	b	b	a	b
a	a	b	a	a	a	b	b	a	b
a	a	b	a	a	a	b	b	a	b
a	a	b	a	a	a	a	b	a	b
a	a	b	a	a	a	a	b	a	b
a	a	b	a	a	a	a	b	a	b
a	a	b	a	a	a	a	b	a	b
a	a	b	-	-	a	a	b	a	b
a	a	-	a	a	a	a	a	a	b
a	a	b	a	a	a	a	a	a	b
a	a	b	a	a	a	a	a	a	b
a	a	b	a	a	a	a	a	a	b
a	a	b	a	a	a	a	a	a	b
-	a	b	a	-	a	a	a	a	b
a	a	b	a	a	a	a	a	a	b
a	a	-	a	a	a	a	a	a	b
a	a	-	a	a	a	a	a	a	b
a	a	b	a	a	a	a	a	a	b

a	a	b	a	a	a	a	a	a	b
a	a	b	a	a	a	a	a	a	b
a	a	b	a	a	a	a	a	a	b
a	a	b	a	a	a	a	a	a	b
a	a	b	a	a	a	a	a	a	b
a	a	-	a	a	a	a	a	a	b
a	a	b	a	a	a	a	a	a	b
a	a	-	a	a	a	a	a	a	b
a	a	b	a	a	a	a	a	a	b
a	a	b	a	a	a	a	a	a	b
b	a	b	b	b	a	a	a	a	b
a	a	b	b	b	a	a	a	a	b
a	a	b	b	b	a	a	a	a	b
-	a	b	b	b	a	a	a	a	b
a	a	b	b	b	a	a	a	a	b
a	a	b	b	b	a	a	a	a	b
a	a	b	b	b	a	a	a	a	b
a	a	b	b	b	a	b	a	a	b
a	a	a	b	a	a	a	a	a	b
a	-	a	b	-	-	a	a	a	b
b	a	a	a	a	a	a	b	b	b
b	a	a	a	-	a	-	b	b	b
b	a	a	-	a	a	a	b	b	b
-	a	a	a	b	b	a	b	b	b
b	a	-	a	a	b	a	b	b	b
b	-	a	a	a	b	a	b	b	b
b	a	a	a	-	b	a	b	b	b
b	a	a	a	a	b	a	b	b	b
b	a	a	a	a	b	a	b	b	b
b	a	a	a	a	b	a	b	b	b
b	a	a	a	a	b	a	b	b	b
b	a	a	a	a	b	a	b	b	b
b	a	a	-	a	b	a	b	b	b
b	a	a	a	a	b	a	b	b	b
b	-	a	a	a	b	a	b	b	b
b	a	a	a	a	b	a	b	b	b
b	a	-	-	-	b	a	b	b	b
b	a	a	a	a	b	a	b	b	b
-	a	a	a	a	b	a	b	b	a
b	a	a	a	a	b	a	b	b	a
b	a	a	a	a	b	a	b	b	a
b	a	a	a	a	-	a	b	b	a
b	a	a	a	a	b	a	b	b	a
-	a	a	a	b	b	a	b	b	a
b	a	a	a	b	b	a	b	b	a
-	a	a	a	b	b	a	a	b	a
b	a	a	a	b	b	a	a	b	a

b	a	a	a	b	b	a	a	b	a
b	a	a	a	b	b	a	a	b	a
a	a	a	a	b	b	a	a	b	a
b	a	a	a	b	b	a	a	b	a
a	a	a	a	b	b	a	a	b	a
-	a	a	a	b	b	a	a	b	a
b	a	-	a	b	b	a	a	b	a
b	a	a	a	b	b	a	a	b	a
b	a	a	a	b	b	a	a	b	a
b	a	a	a	b	b	a	a	b	a
b	a	a	-	b	b	a	a	b	a
-	a	a	a	b	b	a	a	b	a
b	a	a	a	b	b	a	a	b	b
b	a	a	-	b	b	-	a	b	b
b	a	a	a	b	b	a	a	b	b
b	a	a	a	-	b	a	a	b	b
b	a	a	-	b	b	a	a	b	b
b	a	a	a	b	-	a	a	b	b
b	a	a	a	b	b	a	a	b	b
a	a	a	a	b	b	a	-	b	b
a	a	a	a	b	b	a	a	b	b
a	a	a	a	b	b	a	a	a	b
a	a	a	a	b	b	a	a	a	b
a	a	a	a	b	b	a	a	a	b
a	a	a	a	-	-	a	a	a	b
a	a	a	a	b	b	a	a	a	b
a	a	a	a	b	b	a	a	a	b
a	a	a	a	b	b	a	a	a	b
a	a	a	a	b	b	a	a	a	b
a	a	a	b	b	b	a	a	a	b
b	a	-	a	b	b	a	a	a	b
a	a	a	a	b	b	a	a	a	b
a	a	a	b	b	b	a	a	a	b
a	a	a	b	b	b	a	a	a	b
a	a	a	b	-	b	a	a	a	b
b	b	a	b	a	b	a	b	a	a
b	b	-	b	a	b	b	b	a	a
b	b	a	b	a	b	a	b	a	a
b	b	a	b	a	b	b	b	a	a
b	b	a	b	-	b	b	b	a	a
b	b	a	b	a	b	b	b	a	a
b	b	a	b	a	b	a	b	a	a
b	b	a	b	a	b	b	b	a	a
b	b	a	b	a	b	b	b	a	a
b	b	a	a	a	b	b	b	a	b
-	b	a	a	a	b	b	b	a	b
-	b	a	a	a	b	b	b	a	b

b	b	a	a	a	b	b	b	a	b
b	b	a	a	a	b	b	b	a	b
b	b	-	a	a	b	b	b	a	b
b	b	a	a	a	b	b	b	a	b
b	b	a	a	a	b	b	b	a	b
b	b	a	a	a	b	b	b	a	b
b	b	a	a	a	b	b	b	a	b
b	b	a	a	a	b	b	b	a	b
-	b	a	a	a	b	b	-	a	b
b	b	a	a	a	b	b	b	a	b
b	b	a	a	a	b	b	b	a	b
b	b	a	a	a	b	b	b	a	b
b	b	-	a	-	b	b	b	a	b
-	b	a	a	a	b	b	b	a	b
b	b	a	a	a	b	b	b	a	b
b	b	a	a	a	b	b	b	a	b
b	b	a	a	-	b	b	b	a	b
b	-	a	a	a	b	b	b	a	b
b	b	-	a	-	b	b	b	a	b
b	b	a	a	a	b	b	b	a	b
b	b	a	a	a	b	b	b	a	b
b	b	a	-	a	b	a	b	a	b
-	b	a	a	a	b	a	b	a	b
b	-	a	a	a	-	a	b	a	b
b	b	a	a	a	b	a	b	a	b
b	b	a	a	a	b	a	b	a	b
b	b	a	a	a	b	a	b	a	b
a	b	a	a	a	-	a	b	a	b
b	b	a	a	a	b	a	b	a	b
b	b	a	a	a	b	a	b	a	b
b	b	a	a	a	b	a	b	a	b
b	b	a	a	a	b	a	b	a	b
b	b	a	b	a	b	a	b	a	b
b	b	a	b	a	b	a	b	a	b
b	b	a	b	a	b	a	b	b	b
b	b	a	b	a	b	a	b	b	b
b	b	a	b	a	b	a	b	b	b
b	b	a	b	a	b	a	b	b	b
b	b	a	b	-	b	a	b	b	b
b	b	a	b	a	b	a	b	b	b
b	b	a	b	a	b	a	b	b	b
-	b	a	b	a	b	a	b	b	b
b	b	a	b	a	b	-	b	b	b
b	b	a	b	a	b	a	b	b	b
b	b	a	b	a	b	a	b	b	b
b	b	a	b	a	b	a	b	b	b
a	b	b	b	a	b	b	a	b	b

a	b	b	b	a	b	b	a	b	b
a	b	b	b	a	b	b	a	b	b
a	b	b	b	a	b	b	a	b	b
a	b	b	b	a	b	b	a	b	b
a	b	b	b	a	b	b	a	b	b
a	b	b	b	a	b	b	a	b	b
b	b	b	b	a	b	b	a	b	b
b	b	b	b	a	b	b	a	b	b
b	b	b	b	a	b	b	a	b	b
b	b	b	b	a	b	b	a	b	b
b	b	b	b	a	b	b	a	b	b
b	b	b	b	a	b	b	a	b	b
b	b	b	b	a	b	b	a	b	b
b	b	b	b	a	b	b	a	b	b
b	b	b	b	a	b	b	a	b	b
b	b	b	b	a	b	b	a	b	b
b	b	b	b	a	b	b	a	b	b
b	b	b	b	a	b	b	a	b	b
b	b	b	b	a	b	b	a	b	b
b	b	b	b	a	b	b	a	b	b
b	b	b	b	a	b	b	a	b	b
b	b	b	b	a	b	b	a	b	b
b	b	b	b	a	b	b	a	b	b
b	b	b	b	a	b	b	a	b	b
b	b	b	b	a	b	b	a	b	b
b	b	b	b	a	b	b	a	b	b
b	b	b	b	a	b	b	a	b	b
b	b	b	b	a	b	b	a	b	b
b	b	b	b	a	b	b	a	b	b
b	b	b	b	a	b	b	a	b	b
a	b	b	b	a	b	b	a	b	b
b	b	b	b	a	b	b	a	b	b
b	b	b	b	a	b	b	a	b	b
b	b	b	b	a	b	b	a	b	b
b	b	b	b	a	b	b	a	b	b
b	b	b	b	a	b	b	a	b	b
b	b	b	b	a	b	b	a	b	b
b	b	b	b	a	b	b	a	b	b
a	a	a	a	a	b	b	a	b	b
b	b	b	b	a	b	b	a	b	b
a	a	a	a	b	a	b	a	a	a
a	a	b	b	a	b	a	a	a	b
b	a	b	b	a	b	a	a	a	b
b	a	b	b	a	b	a	a	a	b
b	a	b	b	a	b	a	a	a	b
b	a	b	b	a	b	a	a	a	b
b	a	b	b	a	b	a	a	a	b

b	a	b	b	a	b	a	a	a	b
b	a	b	b	a	b	a	a	a	b
b	a	b	b	a	b	a	a	a	b
b	a	b	b	a	b	a	a	a	b
b	a	b	b	a	b	a	a	a	b

YW141	YW142	YW143	YW144	YW145	YW146	YW147	YW148	YW149	YW150
a	b	b	a	a	a	b	a	a	a
a	b	b	a	a	a	b	-	a	a
a	b	b	a	-	a	b	a	a	a
a	b	b	a	a	a	b	a	a	a
a	b	b	a	a	a	b	a	a	a
a	b	b	a	a	a	b	a	a	a
a	b	a	a	a	a	b	a	a	a
a	-	b	a	a	a	b	a	a	a
a	b	b	a	a	a	b	a	a	a
a	a	b	a	a	a	b	a	a	a
a	b	b	a	a	a	-	a	a	a
a	b	b	a	a	a	b	a	a	a
a	b	b	a	a	a	b	a	a	a
a	b	b	a	-	a	b	a	a	a
a	b	b	a	a	a	b	-	a	a
a	b	b	a	a	a	-	a	a	a
a	b	b	a	b	a	b	-	a	a
a	b	b	a	a	a	b	a	a	a
a	b	b	a	-	a	b	a	a	a
a	b	b	a	a	a	b	a	a	a
a	b	b	a	a	a	b	a	a	a
a	b	b	a	a	a	b	a	a	a
a	b	a	a	a	a	b	a	a	a
a	b	a	a	a	a	b	a	a	a
-	b	a	a	a	a	b	a	a	a
a	-	a	a	a	a	b	a	a	a
a	b	a	a	a	a	b	a	b	a
a	a	a	a	a	a	b	a	b	a
a	-	a	a	a	a	a	b	b	a
a	b	a	-	a	a	a	b	b	a
a	b	a	a	b	a	a	b	b	a
a	b	a	a	b	a	a	b	b	a
a	b	a	a	a	a	a	b	b	-
a	b	a	a	a	a	a	b	b	a
a	b	a	a	b	a	-	b	b	a
a	b	a	a	a	a	a	b	b	a
a	b	a	a	a	a	a	b	b	a
a	b	a	a	a	a	a	b	b	a
a	b	a	a	b	a	a	b	b	a
a	b	a	a	b	a	a	b	b	a
a	b	a	a	-	a	a	b	b	a
a	b	a	a	b	b	a	b	b	a
a	b	a	a	b	b	a	b	b	a
a	b	a	a	b	b	a	b	b	a
a	b	-	-	b	b	a	b	b	a

a	b	a	a	b	b	a	b	b	a
a	b	a	a	-	-	a	b	b	a
a	b	a	a	b	b	a	b	b	a
a	b	a	a	b	b	a	b	b	a
a	b	a	a	b	b	a	b	b	a
a	b	a	a	b	b	a	b	b	a
a	b	a	a	a	b	a	b	b	a
a	b	a	a	b	b	a	b	b	a
a	b	a	a	b	b	a	b	b	a
a	b	a	a	a	b	a	b	b	a
a	a	a	a	a	b	a	b	b	a
a	b	a	a	b	b	a	b	b	a
a	b	a	a	b	b	a	b	b	a
a	b	a	b	a	b	a	b	b	a
a	b	a	b	b	b	-	b	b	a
a	b	a	b	b	b	a	b	b	a
a	b	a	b	a	b	a	b	b	a
a	b	a	b	-	b	a	b	b	a
a	b	a	b	a	b	a	b	b	a
a	b	a	b	a	b	a	b	b	a
a	b	a	b	a	b	a	b	b	a
a	b	a	b	b	-	a	b	b	a
a	b	a	b	b	b	a	b	b	a
a	-	a	-	b	b	a	b	b	a
a	b	a	b	b	b	a	b	b	a
a	b	a	b	b	b	a	b	b	a
a	-	a	b	a	b	a	-	b	a
a	b	a	b	b	b	a	b	b	a
a	b	a	b	a	b	a	b	b	a
a	b	a	b	a	b	a	b	b	a
a	b	a	-	a	b	a	b	b	a
a	b	a	b	a	b	a	b	b	a
a	b	a	b	a	b	a	b	b	a
a	a	a	b	b	b	a	b	b	a
a	b	a	b	b	b	a	b	b	a
a	b	a	b	b	b	a	b	b	a
a	b	a	b	b	b	a	b	b	a
a	b	a	b	a	b	a	b	b	a
a	-	a	b	a	b	a	b	b	a
a	a	b	b	a	b	a	b	-	a
a	a	b	b	a	b	a	b	b	a
a	a	b	b	a	b	a	b	b	a
a	a	b	b	a	b	a	b	b	a
a	a	b	b	a	b	a	b	b	a
a	a	b	b	a	b	-	b	b	a
a	a	b	b	a	b	a	b	b	a
a	a	b	b	a	b	a	b	-	a

a	a	b	b	a	b	a	b	b	a
a	a	b	b	a	b	a	b	b	a
a	a	b	b	a	b	a	b	-	a
a	a	b	b	a	b	a	b	-	a
a	a	-	b	a	b	a	b	b	a
a	a	b	b	a	b	a	b	b	a
a	a	b	b	a	b	a	-	b	a
a	a	b	b	a	b	a	b	b	a
a	a	b	b	a	b	a	-	a	a
a	a	b	b	a	b	a	b	a	a
a	a	b	b	a	b	a	b	a	a
a	a	b	b	a	b	a	b	a	a
a	a	b	b	a	b	a	b	a	a
a	a	b	b	a	b	a	b	-	b
a	a	b	b	a	b	a	b	b	b
a	a	b	b	a	b	a	b	b	b
a	-	b	b	-	b	a	b	b	b
a	a	b	b	a	b	-	b	b	b
a	a	b	b	-	b	a	b	b	b
a	a	b	b	a	b	a	b	b	b
a	a	b	b	a	b	a	b	b	b
a	a	b	b	a	b	a	b	b	b
a	a	b	b	-	b	a	b	b	b
a	a	b	b	a	b	a	b	b	b
a	a	b	b	a	b	a	a	b	b
a	a	b	b	a	b	a	a	b	b
a	b	b	b	a	b	a	a	b	b
a	a	b	b	a	b	a	a	b	b
a	a	b	b	b	b	a	a	b	b
a	a	-	b	a	b	a	a	b	b
a	a	b	b	a	b	a	a	b	b
a	a	b	b	b	b	a	-	b	b
a	a	b	b	a	b	a	a	b	b
a	a	b	b	a	b	a	a	b	b
a	a	b	b	a	b	a	a	b	b
a	a	b	b	b	b	a	a	b	b
a	a	b	b	b	b	a	a	b	b
a	a	b	b	a	b	a	a	b	b
a	a	b	b	a	b	a	a	b	b
a	a	b	b	a	b	a	a	b	b
a	a	b	b	a	b	a	a	b	b
a	a	b	b	a	b	a	a	b	b
b	-	b	b	-	b	a	a	b	b
b	a	a	b	a	b	a	a	b	b
b	-	b	b	a	b	b	-	b	b
b	a	-	b	a	b	b	a	b	b
b	a	b	b	a	b	b	a	b	b

b	a	b	b	a	b	b	a	b	b
b	a	b	-	a	b	b	a	b	b
b	a	b	b	a	b	b	a	b	b
b	a	b	b	a	b	b	a	b	b
b	a	b	b	a	b	b	a	b	b
b	a	b	b	a	b	b	a	b	b
b	a	b	b	a	b	b	a	b	-
b	a	b	b	a	b	b	a	b	b
b	a	b	b	a	b	b	a	b	b
b	a	b	b	a	b	b	a	b	b
b	a	b	b	a	b	b	a	b	b
b	a	b	b	a	b	b	a	b	b
b	a	b	b	a	b	b	a	-	b
b	a	b	b	a	b	b	a	b	b
b	a	b	b	a	b	b	a	b	b
b	a	b	b	a	b	b	a	b	b
b	a	b	a	a	b	b	a	b	b
b	a	b	a	a	b	b	a	b	b
b	-	b	a	a	b	b	a	b	b
b	b	b	a	a	b	b	a	b	b
b	a	b	a	a	b	b	a	b	b
b	b	b	a	-	b	b	a	b	b
b	a	b	a	a	b	-	a	-	b
b	-	b	a	a	b	b	a	b	b
b	a	b	a	a	-	b	a	b	b
b	a	b	a	a	b	b	a	-	b
b	a	b	a	a	b	b	a	b	b
b	a	b	a	a	b	b	a	b	b
b	a	b	a	a	b	b	a	b	b
b	a	b	a	a	b	b	a	b	b
b	a	b	a	a	b	b	a	b	b
b	a	b	a	a	b	b	a	b	b
b	a	b	a	a	b	b	a	b	b
b	a	b	a	a	b	b	a	b	b
b	b	b	a	a	b	b	a	b	b
b	b	b	a	a	b	b	a	b	b
b	b	b	a	a	b	b	a	b	b
b	b	b	a	a	b	b	a	b	b
b	b	b	a	a	b	b	a	b	b
b	b	b	a	a	b	-	a	b	b
b	b	b	a	a	b	b	a	b	b
b	-	b	a	a	b	b	a	b	b
b	b	b	b	a	b	b	a	b	b
b	b	b	b	a	b	-	a	b	b
b	b	b	b	a	b	b	a	b	b
b	b	b	-	a	b	b	a	b	b
b	b	b	b	a	b	b	a	b	b

b	b	b	b	a	b	-	a	b	b
b	b	b	b	a	b	b	a	b	b
b	b	b	b	a	b	b	a	b	b
b	b	b	-	a	b	b	a	b	b
b	b	b	a	a	b	b	a	b	b
b	b	b	a	a	b	b	a	b	b
b	-	b	a	a	b	-	a	b	b
b	b	b	a	a	b	b	b	b	b
b	b	b	a	a	b	b	-	b	b
b	b	b	a	a	b	b	b	b	b
b	b	-	a	a	b	b	b	b	b
b	b	b	a	a	b	b	b	b	b
a	a	b	b	a	a	a	b	a	a
a	a	b	-	a	a	a	-	a	a
a	a	b	b	a	a	a	b	a	a
a	a	b	b	a	a	a	b	a	a
a	a	b	b	a	a	a	b	a	a
a	a	b	b	b	a	a	b	a	a
a	a	b	b	a	a	a	b	a	a
a	a	b	b	a	a	a	b	a	a
a	a	b	b	a	a	a	b	a	a
a	a	b	b	a	a	-	b	a	a
a	a	b	b	b	a	a	b	a	a
a	a	b	b	b	a	a	b	-	a
a	-	b	b	b	a	a	b	a	a
a	a	b	b	b	a	a	b	a	a
a	a	b	b	a	a	a	b	a	a
a	a	b	b	b	a	a	b	a	a
a	a	b	b	a	a	a	b	a	a
a	a	b	b	-	a	a	b	a	a
a	a	b	b	a	a	a	b	a	a
a	-	b	b	a	a	a	b	a	a
a	a	b	b	b	a	a	a	a	a
a	-	b	b	b	-	a	a	a	a
a	a	b	b	a	a	a	a	a	a
a	a	b	b	b	-	a	a	a	a
a	a	b	b	b	-	a	a	a	a
a	a	b	b	b	a	a	a	a	a
a	a	b	b	b	a	a	a	a	a
a	a	b	b	a	a	a	a	a	a
a	a	b	b	a	a	a	b	a	a
a	a	b	b	a	a	a	-	a	a
a	a	b	b	a	a	a	b	a	a
a	a	b	b	a	a	a	b	a	a
a	a	b	b	a	a	a	b	a	a
a	a	b	b	a	a	b	-	a	a
a	-	b	b	a	a	b	b	a	a

a	a	b	b	-	a	b	b	a	a
a	a	b	-	a	a	b	b	a	a
a	a	b	b	a	a	b	b	a	a
a	b	b	b	a	a	b	b	a	a
a	b	b	b	-	a	b	b	a	a
a	b	b	b	a	a	b	b	a	a
a	b	b	b	a	a	b	b	b	a
a	b	b	b	a	a	b	b	b	b
a	b	b	b	a	a	b	b	b	b
a	b	b	b	a	a	b	b	b	b
a	-	b	b	a	a	b	b	b	b
a	b	-	b	a	a	b	b	b	b
a	b	b	b	a	a	b	b	b	b
a	b	b	b	a	-	b	b	b	b
a	b	b	b	a	a	b	b	b	b
a	b	b	b	a	a	b	b	b	b
a	b	b	-	a	a	b	b	b	b
a	b	b	b	a	-	b	b	b	b
a	-	b	b	a	a	-	b	b	b
a	b	b	b	a	a	b	b	b	b
a	b	b	b	a	a	b	b	b	b
a	b	b	b	a	a	b	b	b	b
a	b	b	b	a	a	b	b	b	b
a	b	b	b	a	a	b	b	b	b
a	-	b	b	a	b	b	b	b	b
a	-	b	b	a	b	b	b	b	b
a	b	b	b	a	b	b	b	b	b
a	b	b	b	a	b	b	b	b	b
a	b	b	-	a	b	b	b	b	b
a	b	b	b	a	b	b	b	b	b
a	b	b	-	a	b	b	b	b	b
a	-	b	a	a	b	b	b	b	b
a	b	b	a	a	b	b	b	b	b
a	b	b	a	a	b	b	b	b	b
a	b	b	a	a	b	b	b	b	b
a	b	b	a	a	b	b	b	b	b
a	b	a	a	a	b	b	b	b	b
a	b	a	a	a	b	b	b	b	b
a	b	a	a	a	b	b	b	b	b
a	b	a	a	a	b	b	b	b	b
a	b	a	a	a	b	b	b	b	b
a	a	-	a	a	b	b	b	b	b
a	a	a	a	a	b	b	b	b	b
a	a	a	a	a	b	b	b	b	b
a	a	a	a	a	b	b	b	b	b
a	a	a	a	a	b	b	b	b	b
a	a	a	a	a	b	-	b	b	b

a	a	a	a	a	-	-	b	b	b
a	a	a	a	-	b	b	b	b	b
a	a	a	a	a	b	a	b	b	b
a	a	a	a	a	b	a	-	b	b
a	a	a	a	a	b	a	b	b	b
a	a	a	a	a	b	a	b	b	b
a	a	a	a	a	b	a	b	b	b
a	a	a	a	a	b	a	b	b	b
a	a	a	a	a	b	a	b	-	b
a	a	a	a	a	b	a	b	b	b
a	a	a	a	a	b	a	b	b	b
a	a	a	a	a	b	a	-	-	b
a	a	a	a	a	b	a	b	b	b
a	a	a	a	a	b	a	b	b	b
a	a	a	a	-	b	a	b	b	b
a	a	a	a	a	b	a	b	b	b
a	a	a	a	a	b	a	b	b	b
a	a	a	a	a	b	a	b	b	b
a	b	a	a	a	b	-	b	b	b
a	a	a	a	a	b	a	b	b	b
a	a	a	a	a	b	a	b	b	b
b	a	a	a	a	b	a	b	b	b
b	a	a	-	a	b	a	b	b	b
b	a	a	a	a	b	a	b	b	b
b	-	a	a	b	b	-	b	b	b
b	a	a	a	a	b	a	b	b	b
b	a	a	a	a	b	a	b	b	b
b	a	a	a	a	b	a	b	b	b
b	a	a	a	a	b	a	b	b	b
b	a	a	a	a	b	a	b	b	b
b	a	a	a	a	b	a	b	b	b
b	a	a	a	a	b	a	-	b	b
b	a	a	a	a	b	a	b	b	b
b	a	a	a	a	b	a	-	b	b
b	a	a	a	a	b	a	-	b	b
b	a	a	a	a	-	a	b	b	b
b	a	a	a	a	-	a	b	b	b
b	a	a	a	a	b	a	b	b	b
a	b	b	a	b	a	a	a	b	a
a	b	b	a	a	a	a	a	b	a
a	b	b	a	b	a	a	a	b	a
a	b	b	a	b	a	a	a	b	a
a	b	b	a	b	a	a	a	b	a
a	b	b	a	a	a	a	a	b	a
a	b	b	a	a	a	a	a	b	a
a	b	b	a	a	-	-	a	b	a
a	b	b	a	a	a	a	a	b	a

a	b	b	a	b	a	a	a	b	a
a	a	b	a	a	a	a	a	b	a
a	a	b	a	b	-	a	a	b	a
a	a	b	a	a	a	a	a	b	a
a	a	b	a	a	a	a	a	b	a
a	a	b	a	a	a	a	a	b	a
b	a	b	a	a	a	a	a	b	a
b	a	b	a	a	a	a	a	b	a
b	a	b	a	a	a	a	a	b	a
b	a	b	a	a	a	a	a	b	a
b	a	b	a	a	-	a	a	b	a
b	a	b	a	a	a	a	a	b	a
b	a	b	a	a	a	-	a	b	a
b	a	b	a	b	a	a	a	b	a
b	a	b	a	b	a	-	a	b	a
b	a	b	a	b	a	a	a	b	a
b	a	b	a	b	a	a	a	b	a
b	a	b	a	b	a	a	a	b	a
b	a	b	a	b	a	a	a	b	a
b	a	b	a	b	a	a	a	b	b
b	a	b	a	b	a	a	a	b	b
b	a	b	a	a	a	a	a	b	-
b	a	b	a	b	a	a	a	b	b
b	-	b	a	a	a	a	a	b	b
b	a	b	a	a	a	a	a	-	b
b	a	b	a	a	a	a	a	b	b
b	a	b	a	a	a	a	a	-	b
b	a	b	a	a	a	a	a	b	b
b	-	b	a	a	a	a	a	b	b
b	a	b	a	b	a	a	a	b	b
b	a	b	a	b	a	-	a	b	b
b	a	b	a	a	a	a	b	b	b
b	-	b	a	b	a	a	b	b	b
b	a	b	a	a	a	a	b	b	b
b	a	b	a	b	a	a	b	b	b
b	a	b	a	a	a	a	b	b	b
b	a	b	a	b	b	a	b	b	b
b	a	b	a	b	b	a	b	-	b
b	a	b	a	a	b	a	b	b	b
b	a	b	a	a	b	a	b	b	b
b	a	b	a	a	b	a	b	b	b
b	a	b	a	b	b	a	b	b	b
b	a	b	a	a	b	a	b	a	b
b	a	b	a	b	b	a	b	a	b
b	-	b	a	b	b	a	b	a	b
b	a	b	a	a	-	a	b	a	b
b	a	b	a	b	b	a	b	a	b

b	a	b	a	b	b	a	b	a	b
b	a	b	a	b	b	a	b	a	b
b	a	b	a	a	b	a	b	a	b
b	a	b	a	b	b	a	b	a	b
b	a	b	a	a	b	a	b	a	-
b	a	b	a	a	b	a	b	a	b
b	a	b	a	-	b	a	b	a	b
b	b	b	a	b	b	a	b	a	b
b	b	b	a	a	b	a	b	a	b
b	b	b	a	b	b	a	b	a	b
b	b	b	a	b	b	a	b	a	b
b	b	b	a	b	b	a	b	a	b
b	b	b	a	b	b	a	b	a	b
b	b	b	a	b	b	a	b	a	b
b	b	-	a	a	b	a	-	a	b
b	b	b	a	b	b	a	b	a	b
b	b	b	a	a	b	a	b	a	b
b	b	b	a	b	b	a	b	a	b
b	b	b	a	b	b	a	b	a	b
b	b	b	b	a	b	a	b	a	b
b	b	a	b	a	b	a	b	a	b
b	b	b	b	a	b	a	b	a	b
b	b	b	b	a	b	a	b	b	b
b	b	b	b	a	b	a	b	b	b
b	b	b	b	a	b	a	b	b	a
b	b	b	b	a	b	a	b	b	a
b	b	b	b	a	-	a	b	b	a
b	a	b	b	a	b	a	b	b	a
b	-	b	b	a	b	a	b	b	a
b	a	b	b	a	-	a	b	b	a
b	a	b	b	a	b	a	b	b	a
b	a	a	b	a	b	a	b	b	a
b	a	a	b	a	b	a	b	b	a
b	a	a	b	a	b	a	b	b	a
b	a	a	b	a	b	a	b	b	a
b	a	a	b	a	b	a	b	b	a
b	a	a	b	a	b	a	b	b	a
b	a	a	b	a	b	a	b	b	a
b	a	a	b	a	b	a	b	b	a
b	a	a	b	a	b	a	b	b	a
b	b	a	b	b	-	a	a	b	a
b	b	a	b	b	a	a	a	b	a
b	b	a	b	b	a	a	a	b	a
b	b	a	b	b	a	a	a	b	a
b	b	a	b	b	a	a	a	b	a
b	b	a	b	a	a	a	a	b	a
b	b	a	b	a	a	a	a	b	a

b	b	a	b	a	a	a	a	b	a
b	b	a	-	b	a	a	a	b	a
b	b	a	b	b	a	a	a	b	a
b	b	a	b	b	a	a	a	b	a
b	b	a	b	b	a	a	a	b	a
b	b	a	b	b	-	a	a	b	a
b	b	a	b	b	-	a	a	b	a
b	b	a	b	b	-	a	a	b	a
b	b	a	b	a	b	a	a	b	a
b	b	a	b	b	b	a	a	b	a
b	b	a	b	b	b	a	a	b	a
b	b	a	b	b	b	a	a	b	a
b	b	a	b	b	b	a	a	b	a
b	b	a	b	b	b	a	a	b	a
b	b	a	b	b	b	a	a	b	a
b	b	a	b	a	b	a	a	b	a
b	b	a	b	-	b	-	a	b	b
b	b	a	b	a	b	a	a	b	b
b	b	a	b	b	b	a	a	b	b
b	b	a	b	b	b	a	a	b	b
b	b	a	b	a	b	a	a	b	b
b	b	a	b	a	b	a	a	b	b
a	b	a	b	b	b	a	a	b	b
a	-	a	b	a	b	a	a	b	b
a	b	a	b	b	b	a	a	-	b
a	b	a	b	a	b	a	a	b	b
a	a	a	b	a	b	a	a	b	b
a	b	a	b	b	b	a	a	b	b
a	-	a	b	a	b	a	a	b	b
a	b	a	b	a	b	a	a	b	b
a	b	a	b	b	b	a	a	b	b
a	b	a	b	b	b	a	a	b	b
a	b	a	b	b	b	a	a	b	b
a	b	a	b	b	b	a	a	b	-
a	b	a	b	b	b	a	a	b	b
a	b	a	b	b	b	a	a	b	b
a	b	b	b	b	b	a	a	b	b
a	b	a	b	b	b	a	a	b	b
a	b	a	b	b	b	a	a	b	b
a	b	a	b	b	b	a	a	b	b
a	-	a	b	b	b	a	-	b	b
a	b	a	b	a	b	-	a	b	b
a	b	a	b	b	b	a	a	a	b
a	-	a	b	b	b	a	a	a	b
a	b	a	b	b	b	a	a	a	b
a	b	a	b	b	b	a	a	a	b
a	b	a	b	b	b	a	a	a	b

a	b	a	b	b	b	a	a	-	b
a	-	a	b	a	b	a	a	a	b
a	b	a	b	b	b	a	-	a	b
a	b	a	-	b	b	a	a	a	b
a	b	a	b	a	b	a	a	a	b
a	b	a	b	a	b	a	a	a	b
a	b	a	b	a	b	a	a	a	b
a	b	a	b	b	b	a	a	a	b
a	b	a	b	b	b	a	a	a	b
a	b	a	b	a	b	a	a	a	b
a	b	a	b	a	b	a	a	a	b
a	b	a	b	b	b	a	a	a	b
a	b	a	b	b	b	-	-	a	b
a	-	a	b	b	b	a	a	a	b
a	b	a	b	b	b	a	a	a	b
a	b	a	b	b	-	a	b	a	b
a	b	a	b	b	b	a	b	a	b
a	b	a	b	-	b	a	b	a	b
a	b	a	b	b	b	a	b	a	b
a	-	a	b	b	b	a	b	a	b
a	b	a	b	b	b	a	b	a	b
a	b	a	b	a	b	-	b	a	b
b	b	a	b	b	b	a	b	-	b
b	b	a	b	a	b	a	b	a	b
b	b	a	b	b	b	a	b	a	b
b	b	a	b	b	b	a	b	a	b
b	b	a	b	b	b	a	b	a	b
b	b	a	b	b	b	a	b	a	b
b	b	a	b	-	b	a	b	a	b
b	b	a	b	b	b	a	b	a	b
b	b	a	b	b	b	a	b	a	b
b	b	a	b	b	b	a	b	a	b
b	b	a	b	b	b	a	b	a	b
b	b	a	b	b	b	a	-	a	b
b	b	a	b	b	b	a	b	a	b
b	b	a	b	b	b	a	b	-	b
b	b	a	b	a	b	a	b	a	b
b	b	a	b	b	b	a	b	a	b
b	b	a	b	b	b	a	b	a	b
b	b	a	b	b	b	a	b	a	b
b	-	a	b	b	-	a	b	a	b
b	b	a	b	a	b	a	b	a	b
b	b	a	b	b	b	a	b	a	b
b	b	a	b	a	b	a	b	a	b
b	-	a	b	a	b	a	b	a	b
b	b	a	b	a	b	a	b	a	b
b	b	a	b	a	b	a	b	a	b

b	b	a	b	a	b	a	b	a	b
b	b	a	b	b	b	a	b	a	b
b	b	a	b	b	b	a	b	a	b
b	b	a	b	b	b	-	b	a	b
b	b	a	b	b	b	a	b	a	b
b	b	a	-	b	b	a	b	a	b
b	b	a	a	b	b	a	b	a	b
b	b	a	a	a	b	a	b	a	b
b	b	a	-	b	b	a	b	a	b
b	b	a	a	a	b	a	b	a	b
b	b	a	a	b	b	a	b	a	b
b	b	a	a	a	b	a	b	a	b
b	b	a	a	a	b	a	b	a	b
b	b	a	a	a	b	a	-	a	-
b	b	a	a	b	b	a	b	a	b
b	a	a	a	a	b	a	b	a	b
b	b	a	a	a	b	a	b	a	b
b	b	a	a	b	b	-	b	a	b
b	b	a	a	b	b	a	b	a	b
b	b	b	b	b	-	a	b	b	a
b	b	b	b	a	b	a	b	b	a
b	b	b	b	b	b	a	b	b	a
b	-	b	b	a	b	a	b	b	a
b	b	b	b	a	b	a	b	b	a
b	-	b	b	b	b	a	-	b	a
b	b	b	b	b	b	a	b	b	a
b	a	b	b	-	b	a	b	b	a
b	b	b	b	b	b	a	b	b	a
b	b	b	b	a	b	a	-	b	a
b	b	b	b	b	b	a	b	b	a
b	-	b	b	b	-	a	b	b	a
b	-	b	b	a	b	a	b	b	a
b	b	b	b	b	b	a	b	b	a
b	b	b	b	b	b	a	b	b	a
b	b	b	b	b	b	a	b	b	a
b	b	b	b	b	b	a	b	b	a
b	b	b	b	a	b	a	b	b	a
b	b	b	b	a	b	a	b	b	a
b	b	b	b	-	b	a	b	b	a
b	b	b	b	b	b	a	b	b	a
b	b	b	-	a	b	a	b	b	a
b	b	b	b	a	b	b	b	b	a
b	b	b	b	a	b	b	b	b	a
b	b	b	b	b	b	b	b	b	a
b	b	b	b	a	b	b	b	b	a
b	b	b	b	a	b	b	b	b	a
b	b	b	b	a	b	b	b	b	a

b	b	b	b	a	b	b	b	b	a
b	a	b	b	a	-	b	-	b	a
b	a	b	b	b	-	b	b	b	a
b	b	b	b	a	b	b	b	b	a
b	b	b	b	a	b	b	b	b	a
b	b	-	b	b	b	b	b	b	a
b	-	b	b	-	b	b	b	-	a
b	b	b	b	a	b	b	b	b	a
a	b	b	b	b	b	b	b	b	a
a	b	b	b	a	b	b	b	b	-
a	b	b	b	a	b	b	b	b	a
a	b	-	b	b	b	b	b	b	a
a	b	a	b	b	b	b	b	b	a
a	-	a	b	a	b	-	b	b	a
a	b	a	b	a	b	b	b	b	a
a	b	a	b	b	b	b	-	b	a
a	b	a	b	b	b	b	b	b	a
a	b	a	b	a	b	b	b	b	a
a	b	a	b	b	b	b	b	b	a
a	b	a	b	a	b	b	b	b	a
a	b	a	b	a	b	b	b	b	a
a	b	a	b	a	b	b	-	b	a
a	b	a	-	a	b	b	b	b	a
a	b	a	b	a	b	b	b	b	b
a	b	a	b	a	b	b	b	b	b
a	b	a	b	a	b	b	b	b	b
a	b	a	b	a	b	b	b	b	b
a	b	a	b	a	b	b	b	b	b
a	b	a	b	a	b	-	b	b	b
a	b	a	b	a	-	b	b	-	b
a	b	a	b	a	a	b	b	b	b
a	b	a	b	a	a	b	b	b	b
a	b	a	b	a	a	b	b	b	b
a	b	a	b	a	a	b	b	b	b
a	b	a	b	a	a	b	b	b	b
a	b	a	b	a	a	b	b	b	b
a	a	a	b	a	a	b	b	b	b
a	b	a	b	a	a	b	b	b	a
a	b	a	b	a	a	b	b	b	b
a	b	a	b	a	a	b	b	b	b
a	b	a	b	a	a	b	b	-	b
a	a	a	b	a	a	b	b	b	b
a	b	a	b	a	a	b	b	b	b
a	b	a	-	a	a	b	b	b	b
b	a	b	b	a	a	a	b	b	b
b	a	b	b	a	a	a	b	b	b
b	a	b	b	-	a	-	b	b	b

b	a	b	b	a	a	a	b	b	b
b	a	b	b	a	a	a	b	b	b
b	a	b	b	a	a	a	b	b	b
b	a	b	b	a	a	a	b	b	b
b	a	b	b	a	a	a	b	b	b
b	a	b	b	a	a	a	b	b	b
b	a	b	b	a	a	a	b	b	b
b	a	b	b	a	a	a	b	b	b
b	b	b	b	a	a	a	b	b	b
b	b	b	b	a	a	a	b	b	b
b	b	-	b	a	a	a	-	b	b
a	b	b	a	a	a	a	b	b	a
a	b	b	a	a	-	a	b	b	a
a	b	b	-	a	b	a	b	b	a
a	b	b	a	a	b	a	b	b	a
a	b	b	a	a	b	a	b	b	a
a	a	b	a	a	b	a	b	b	a
a	a	b	a	a	b	a	b	b	a
a	a	b	a	a	b	a	-	b	a
a	a	b	a	a	b	a	b	b	a
a	a	b	-	a	b	a	b	b	a
a	a	b	a	a	b	a	b	b	a
a	a	b	a	a	b	a	b	b	a
a	a	b	a	a	b	a	b	b	a
a	a	b	a	a	b	a	b	b	a
a	a	a	a	a	b	a	b	b	a
a	a	a	a	a	b	a	b	b	a
a	a	a	a	a	b	a	b	b	a
a	a	a	a	a	b	a	b	b	a
a	a	a	a	a	b	a	b	b	a
a	a	a	a	a	b	a	b	b	a
a	a	a	a	a	b	a	b	b	a
a	a	a	a	a	b	a	b	b	a
a	a	a	a	a	b	a	b	b	a
a	a	a	a	b	b	a	b	b	a
a	a	a	a	a	b	a	b	b	a
a	a	a	a	a	b	a	b	b	a
a	a	a	a	a	b	a	b	b	a
a	a	a	a	b	b	a	b	a	a
a	a	a	a	a	b	a	b	a	a
a	a	a	a	a	b	a	b	a	a
a	a	a	a	-	b	a	b	a	a
a	b	a	a	a	b	a	b	a	a
a	a	a	b	a	b	b	b	a	a
b	a	a	b	a	b	b	b	a	a
a	a	a	b	a	b	b	b	a	a
a	a	a	b	a	b	b	b	a	a

a	a	a	b	b	b	b	b	a	a
a	a	a	b	-	b	b	b	a	a
a	a	a	b	a	b	b	b	a	a
a	a	a	b	a	b	b	-	a	b
a	a	a	b	b	b	b	b	a	b
a	a	a	-	b	b	-	b	a	b
a	a	a	b	b	b	-	b	a	b
a	a	a	b	b	b	b	b	a	b
a	a	a	b	b	b	b	b	a	b
a	a	a	b	a	b	b	b	a	b
a	a	a	b	b	b	b	b	a	b
a	a	a	b	a	b	b	b	a	b
a	a	a	b	b	b	b	b	a	b
a	a	a	b	a	b	b	b	a	b
a	a	a	b	a	b	b	b	a	b
a	a	a	b	a	b	b	b	a	b
a	a	a	b	a	b	b	-	a	b
a	a	a	b	a	b	b	b	a	b
a	a	a	b	a	b	b	b	a	b
a	a	a	b	a	b	a	b	a	b
a	a	a	b	a	b	a	b	a	b
a	a	a	b	a	b	a	b	a	b
a	a	a	b	a	b	a	b	a	b
a	a	a	b	a	b	a	b	a	b
a	a	a	b	a	b	a	b	a	b
a	a	a	b	a	b	a	b	a	b
a	a	a	-	a	b	a	b	a	b
a	a	a	b	a	b	a	b	a	b
a	a	a	b	a	b	a	b	a	b
a	a	a	b	a	b	a	b	a	b
a	a	a	b	a	b	a	-	a	b
a	a	a	b	a	b	a	b	a	b
a	a	a	b	a	b	a	b	a	b
a	a	a	b	a	-	a	b	a	b
a	a	a	b	a	b	a	b	a	b
a	a	a	b	a	b	-	b	a	b
a	a	a	b	a	b	a	b	a	b
a	-	a	b	a	b	a	b	a	b
a	a	a	b	a	b	a	b	a	b
a	a	a	b	a	b	a	b	a	b
a	a	a	b	a	b	a	b	a	b
a	a	a	b	a	b	a	b	a	b
a	a	a	b	a	b	a	b	a	b
b	a	a	b	a	b	a	b	a	b
b	a	a	b	a	b	a	b	a	b
b	a	a	b	a	b	a	b	a	b
b	a	a	b	a	b	a	b	a	b

b	a	a	b	a	a	a	b	a	b
b	a	a	b	a	a	a	b	a	b
b	b	a	b	a	a	a	b	a	b
b	b	a	b	a	a	a	b	a	b
b	b	a	b	a	a	a	b	a	b
b	b	a	b	a	a	a	b	a	b
b	b	a	b	a	a	a	b	a	b
b	b	-	b	a	a	a	b	a	b
b	b	a	b	a	a	a	b	a	b
b	-	a	b	a	a	a	b	a	b
b	b	a	b	a	a	a	-	a	b
b	-	a	a	a	a	a	b	a	b
b	b	b	a	-	a	a	b	a	b
b	b	a	a	a	a	a	-	a	b
b	a	a	a	a	a	a	b	a	b
b	b	a	-	-	a	a	b	a	b
b	b	a	a	a	a	a	b	a	b
b	b	a	a	a	a	-	b	a	b
b	b	a	a	-	a	a	b	a	b
b	b	a	a	a	-	a	b	a	b
b	b	a	a	a	a	a	b	b	-
b	b	a	a	a	a	a	b	b	a
b	a	b	b	a	b	b	b	b	a
b	-	b	b	a	b	b	b	b	-
b	a	b	b	a	b	b	b	b	a
b	a	b	b	a	b	b	b	b	a
b	-	b	b	b	b	b	b	b	a
b	a	b	b	b	b	b	b	b	a
b	a	b	-	b	b	b	b	b	a
b	-	b	b	b	b	b	b	b	a
b	a	b	b	b	b	b	b	b	a
b	a	b	b	b	b	b	b	b	a
b	a	b	b	b	b	b	b	b	a
b	a	b	-	b	b	-	a	b	a
b	a	b	b	b	b	b	-	b	a
b	a	b	b	b	a	b	a	b	a
b	a	b	b	b	a	b	a	b	a
b	a	b	b	b	a	b	a	b	a
b	a	b	b	a	a	b	a	b	a
b	a	b	b	a	a	b	a	b	a
b	a	b	b	b	a	b	a	b	a
b	-	b	b	b	a	b	a	b	a
b	a	b	b	b	a	b	a	b	a
b	a	b	b	b	a	b	a	b	a
b	-	b	b	b	a	b	a	b	a
b	b	b	b	a	a	b	a	b	a
a	b	b	b	b	a	b	a	b	a

a	b	b	b	a	a	b	a	b	a
a	b	b	b	a	a	b	a	b	a
a	b	b	b	a	a	b	a	b	a
a	b	b	b	a	a	-	a	b	b
a	b	b	b	b	a	b	a	-	b
a	b	b	b	-	a	b	a	b	b
a	b	b	b	a	a	b	a	b	b
a	b	b	b	b	a	b	a	b	b
a	-	b	b	b	a	b	a	b	b
a	b	b	b	b	a	b	a	b	b
a	b	b	b	b	-	b	-	b	b
a	b	b	b	a	a	b	a	b	b
a	b	b	b	b	a	b	a	-	b
a	b	b	b	a	a	b	a	b	b
a	b	b	b	a	a	b	a	b	b
a	b	b	b	a	a	b	a	b	b
a	b	b	b	b	a	b	a	b	b
a	b	b	b	b	a	b	a	b	b
a	b	b	b	b	a	b	a	b	b
a	-	b	-	a	a	b	a	b	b
a	b	b	b	b	a	b	a	b	b
a	b	b	b	b	a	b	-	b	b
a	b	b	b	b	a	b	a	b	b
a	b	b	b	b	a	b	a	b	b
a	b	b	b	b	a	b	a	b	b
a	b	b	b	-	a	b	a	b	b
a	b	b	b	a	a	b	a	b	b
a	b	b	b	a	a	b	a	b	b
a	b	b	b	b	a	b	a	b	b
a	b	b	b	a	a	b	a	b	b
a	b	b	b	b	a	b	a	b	b
a	b	b	b	b	a	b	a	b	b
a	b	b	b	b	a	b	a	b	b
a	b	b	b	b	a	b	a	b	b
a	b	b	b	a	a	b	a	b	b
a	b	b	b	a	a	b	a	b	b
a	b	b	b	b	a	b	a	b	b
a	b	b	b	a	a	b	a	b	b
a	b	b	b	b	a	b	a	b	b
a	b	b	b	a	a	b	a	b	b
a	b	b	b	b	a	b	a	b	b
a	b	b	b	-	a	b	a	-	b
a	b	b	b	a	a	b	a	b	b
a	a	b	b	a	a	b	a	b	b
a	b	b	b	b	a	b	-	b	b
a	b	b	b	a	a	b	a	b	b
a	b	b	b	a	a	b	-	b	b

a	b	b	b	b	a	b	a	b	b
a	b	b	b	b	a	b	a	b	b
a	b	b	b	a	a	a	a	b	b
a	b	b	-	a	a	a	a	b	b
a	b	b	b	a	a	a	a	b	b
a	b	b	b	a	-	a	a	b	b
a	b	b	b	a	a	a	a	b	b
a	b	b	b	a	a	a	a	b	b
a	b	b	b	a	a	a	a	b	b
a	b	b	b	a	a	a	-	b	b
a	-	b	b	a	a	a	a	b	b
a	b	b	b	-	a	-	a	b	b
a	b	b	b	a	a	a	a	-	b
a	b	b	b	-	a	a	a	b	b
a	b	b	b	a	a	a	a	b	b
a	b	b	b	a	a	a	a	-	b
a	b	b	b	a	a	a	a	-	b
a	b	b	b	a	a	a	a	b	b
a	b	b	b	a	a	a	b	b	b
a	b	b	b	a	a	a	b	-	b
a	b	b	b	a	a	a	b	-	b
a	b	b	b	b	b	a	b	a	b
a	-	b	b	a	b	a	-	a	b
a	b	b	b	a	b	a	b	a	b
a	b	b	b	a	b	a	b	a	b
a	b	b	b	b	b	a	b	a	b
a	b	b	b	a	b	a	b	a	b
a	-	b	b	b	b	a	b	a	b
a	b	b	b	a	b	a	b	a	b
a	b	b	b	b	b	-	b	-	b
a	b	b	b	b	b	a	b	a	b
a	b	b	b	-	b	a	b	a	b
a	b	b	b	b	b	-	b	a	b
a	b	b	b	b	b	a	b	a	b
a	b	b	b	b	b	a	-	a	b
a	b	b	b	a	b	a	b	a	b
a	b	b	b	b	b	a	b	a	b
a	b	b	b	a	b	a	b	a	b
a	b	b	b	a	b	a	b	a	b
a	-	b	b	a	b	a	b	a	b
a	b	b	b	a	b	-	b	a	b
a	b	b	b	a	-	a	b	a	a
a	b	b	-	a	b	a	b	a	b
a	b	b	b	a	b	a	b	b	b
a	b	b	b	b	b	a	b	b	b
a	b	b	b	b	b	a	b	b	b
a	b	b	b	-	b	a	b	b	b

a	b	b	-	b	b	a	b	b	b
a	-	b	b	b	b	a	b	b	b
a	b	b	b	a	b	a	b	b	b
b	b	a	b	b	b	a	b	a	a
b	b	-	b	a	b	a	b	a	a
b	b	a	a	a	b	a	b	a	a
b	b	a	a	a	b	a	b	a	a
b	b	a	a	a	b	a	b	a	b
b	-	a	a	a	b	a	b	a	b
b	-	a	a	a	b	a	b	a	b
b	b	a	a	b	b	a	b	a	b
b	b	a	a	b	b	a	b	a	b
b	b	a	a	a	b	a	b	a	b
b	b	a	a	b	b	a	b	a	b
b	b	a	a	b	b	a	b	a	b
b	-	a	a	a	b	a	-	a	b
b	b	a	a	a	b	a	b	a	b
b	b	a	a	a	b	a	b	a	b
b	b	b	a	a	b	a	b	-	b
b	b	b	a	a	b	a	b	a	b
b	b	b	a	a	b	a	b	-	b
b	b	b	a	b	b	a	b	a	b
b	b	b	a	b	b	a	b	a	b
b	b	b	a	b	b	a	b	a	b
b	b	b	a	b	b	a	b	a	b
b	b	b	a	b	b	a	b	a	b
b	b	b	a	b	b	a	b	a	b
b	-	b	a	-	b	a	b	a	b
b	b	b	a	a	b	a	b	a	b
b	b	b	a	b	b	a	b	a	b
b	b	b	a	b	b	a	b	a	b
b	b	b	a	-	b	a	b	a	b
a	b	b	a	b	b	a	b	a	b
a	b	b	a	-	b	a	b	a	b
a	b	b	a	b	b	a	b	a	b
a	b	b	a	a	b	a	b	a	b
a	b	b	a	b	b	a	b	a	b
a	b	b	a	a	b	a	b	a	b
a	b	b	a	a	b	a	b	a	b
b	b	b	a	-	b	a	-	a	b
b	-	b	a	-	b	b	a	a	b
b	a	b	a	a	b	b	a	a	b
b	a	b	a	-	b	b	a	a	b
b	a	b	a	a	b	b	a	a	b
-	a	b	a	a	-	b	a	a	b
b	a	b	a	a	b	b	a	a	b
b	a	b	a	a	b	b	a	a	b

b	a	b	a	a	b	b	a	a	b
b	a	b	a	a	-	b	a	a	b
a	a	b	a	a	a	b	a	a	b
a	a	b	a	a	a	b	a	a	b
a	a	b	a	a	b	b	b	b	a
a	a	b	a	a	b	b	b	b	a
a	-	b	a	a	b	b	b	b	a
a	a	b	a	a	b	b	b	b	a
a	a	-	a	a	b	b	b	b	a
a	a	b	a	a	b	b	b	b	a
a	a	b	a	a	b	b	b	b	a
a	a	b	a	a	b	-	b	b	a
b	a	b	a	a	b	b	b	b	b
-	a	b	a	a	b	a	a	b	b
a	a	b	a	a	b	a	a	b	b
a	a	b	a	-	a	a	a	b	b
a	a	b	a	a	b	a	a	b	b
a	a	b	a	b	a	a	a	b	b
a	a	b	a	b	a	a	a	b	b
a	a	b	a	a	a	a	a	b	b
a	a	b	a	b	a	a	a	b	b
a	a	b	a	b	-	-	a	b	b
-	a	b	a	a	a	a	a	b	b
a	a	b	a	a	a	a	a	b	b
a	a	b	b	a	b	a	a	b	b
a	-	b	b	a	a	a	a	b	b
a	a	b	b	a	a	-	a	b	b
a	a	b	b	a	a	-	-	b	b
a	a	b	b	a	a	a	a	b	b
a	a	b	b	a	a	a	a	a	b
a	a	b	b	a	a	a	a	a	b
a	a	b	b	a	a	a	a	a	b
a	a	b	b	a	a	a	a	a	b
a	a	b	b	a	a	a	a	a	b
a	a	b	b	a	a	a	a	a	b
a	a	a	b	a	a	a	a	a	b
a	a	a	b	a	a	a	a	a	b
a	a	a	-	a	a	a	a	a	b
a	-	a	b	a	a	a	a	a	b
b	a	a	b	a	a	a	a	a	b
b	a	a	b	a	a	b	a	a	b
b	a	a	b	a	b	b	-	b	b
b	a	a	b	a	b	b	b	b	b
b	a	a	b	a	b	b	a	b	b
b	a	a	b	a	-	b	a	b	b
b	b	a	b	a	b	b	b	b	b
b	b	a	b	a	b	b	b	b	b

b	b	a	b	a	b	b	b	b	b
b	b	a	b	a	b	b	b	b	b
b	b	a	b	a	b	b	b	b	b
b	b	a	b	a	b	b	b	b	b
b	b	b	b	a	b	b	b	b	b
b	b	b	b	a	b	b	b	b	b
b	b	b	b	a	b	b	b	b	b
b	b	b	-	a	b	b	-	-	b
b	b	b	b	a	b	b	b	b	b
b	b	b	b	a	b	b	b	b	b
b	b	b	b	a	b	b	b	b	b
b	b	b	b	a	b	b	b	b	b
b	b	b	b	a	b	b	b	b	b
b	b	b	b	a	b	b	b	b	b
b	-	b	b	a	b	b	b	b	b
b	b	b	b	a	b	b	b	b	b
b	b	b	b	a	b	b	b	b	b
b	b	b	b	b	b	b	b	b	b
b	b	b	b	a	b	b	b	b	b
b	-	b	-	a	-	b	b	b	b
b	b	b	b	a	b	b	b	b	b
b	b	b	b	-	b	b	b	b	b
b	b	b	b	a	b	b	b	-	b
b	b	b	b	a	b	b	a	b	b
b	b	b	b	a	b	b	a	b	b
b	b	b	b	a	b	b	a	b	b
b	b	b	b	a	a	a	a	b	b
b	b	b	b	a	a	a	a	b	b
b	a	b	b	a	a	a	a	b	b
b	b	b	b	a	a	a	-	b	b
b	b	b	b	a	a	a	a	b	b
b	b	b	b	a	-	a	a	b	b
b	b	b	b	a	a	a	a	b	b
b	-	b	b	a	a	a	a	b	b
b	-	b	b	a	a	a	a	b	b
b	b	b	b	a	a	a	a	b	b
b	b	b	b	a	a	a	a	b	b
b	b	b	b	a	a	a	-	b	b
b	b	b	b	a	a	-	a	b	b
b	-	b	b	a	a	a	a	b	b
b	b	b	b	a	a	a	a	b	b
b	b	b	b	a	-	a	a	b	b
b	b	-	b	a	a	a	a	b	-
b	b	b	b	a	a	a	a	b	b
b	b	b	b	a	a	a	a	b	b
b	b	b	a	a	a	a	a	b	b
b	-	b	a	a	a	a	a	b	b

b	b	b	a	a	-	a	a	b	b
b	-	b	a	a	a	b	a	b	b
b	b	b	a	a	a	b	a	b	b
b	-	b	a	a	a	b	a	b	b
b	b	b	a	a	a	b	a	-	b
b	b	b	a	b	b	b	a	b	b
b	b	b	a	a	b	b	a	b	b
b	b	b	a	a	b	b	a	b	b
b	b	b	a	a	b	b	a	b	b
b	b	b	a	a	b	b	a	b	b
b	b	b	a	a	b	b	a	-	b
b	b	b	b	b	b	b	a	b	b
b	b	b	b	b	b	b	a	b	b
b	b	b	b	b	b	b	a	b	b
b	b	b	b	-	b	b	a	b	b
b	a	b	b	b	b	b	a	b	b
b	a	b	b	b	b	b	-	b	b
b	-	b	b	b	b	b	a	b	b
b	a	b	b	b	b	b	a	b	b
b	a	b	b	b	b	b	a	b	b
a	-	b	b	a	b	b	b	b	b
a	b	b	b	b	b	b	b	b	b
a	a	b	b	a	b	b	b	b	b
a	a	b	b	a	b	b	b	b	b
a	-	b	b	b	b	b	b	b	b
a	a	b	b	a	b	b	b	b	b
a	a	b	b	b	b	b	b	b	b
a	a	b	b	b	b	b	b	b	b
a	a	b	b	-	b	b	b	b	b
a	a	b	b	a	b	b	b	b	b
a	a	b	b	a	b	b	b	b	b
a	a	b	b	b	b	b	b	b	b
a	a	b	b	b	b	b	b	b	b
a	-	b	b	b	b	b	b	b	b
a	a	b	b	b	b	b	-	b	b
a	a	b	b	b	b	b	b	b	b
a	a	b	b	a	b	b	b	b	b
a	a	b	b	a	b	b	b	b	b
a	a	b	b	a	b	b	b	b	b
a	b	b	b	b	b	b	b	b	b
a	b	-	b	b	b	b	b	b	b
a	b	b	b	b	b	b	b	b	b
a	-	b	-	a	a	b	b	b	b
a	b	b	b	a	a	-	b	b	b
a	b	b	b	a	-	b	b	b	b
a	b	b	b	a	a	b	b	b	b
a	b	b	a	a	a	b	b	b	b

a	b	b	a	-	-	b	b	b	b
a	a	b	a	a	a	b	b	b	b
a	b	b	a	a	a	a	b	b	b
a	b	b	a	a	a	a	b	b	a
a	b	b	a	a	a	a	b	b	a
a	b	b	a	a	a	-	b	b	a
a	b	b	-	a	a	a	b	b	a
a	b	b	a	a	a	a	b	b	a
a	b	a	a	a	b	b	-	b	a
a	b	a	a	a	b	b	b	b	a
a	b	a	a	a	b	b	b	b	a
a	b	a	a	a	b	b	b	b	a
a	b	a	-	a	b	b	b	b	a
a	-	a	a	a	b	b	b	b	a
a	b	a	a	a	b	b	b	-	a
a	b	a	a	-	b	b	b	b	a
a	b	a	a	a	b	b	b	b	a
a	b	a	a	a	b	b	b	b	a
a	b	a	a	a	b	b	b	b	a
a	b	b	a	a	b	b	b	b	a
a	b	b	-	a	b	b	b	b	a
a	b	b	a	a	b	b	b	b	a
a	b	b	a	a	b	b	b	b	a
a	b	b	b	a	b	b	b	b	a
a	-	b	b	a	b	b	b	b	a
a	b	b	b	a	b	b	b	b	a
a	b	b	b	a	b	b	b	b	a
a	b	b	b	a	a	b	b	b	a
a	b	b	b	a	a	b	b	b	a
a	a	b	a	a	a	b	b	b	a
a	a	b	a	b	a	b	b	a	a
a	a	b	a	b	a	b	a	a	a
a	a	b	a	b	a	b	a	a	a
a	a	b	a	b	a	b	a	a	a
a	a	b	a	b	a	b	a	a	a
a	b	b	a	a	a	a	a	a	a
a	b	b	a	a	a	a	a	a	a
a	b	b	a	b	a	b	a	a	a
a	b	b	a	b	a	a	a	a	a
a	a	b	a	b	a	a	a	a	a
a	b	b	a	a	a	a	a	a	a
a	b	b	a	b	a	a	a	a	a
a	-	b	a	b	a	a	a	a	a
b	b	b	b	a	a	a	a	a	a
b	b	b	b	b	a	a	a	a	a
b	b	b	b	a	a	a	a	a	-
b	b	b	b	a	a	a	a	a	a

b	b	b	b	a	a	a	a	a	a
b	b	b	b	b	a	a	a	a	a
b	b	a	b	a	a	a	a	a	a
b	b	a	b	a	a	a	a	a	a
b	b	a	b	a	a	a	a	a	a
b	-	a	b	a	a	a	a	a	a
b	b	a	b	a	-	a	a	a	a
b	b	a	b	a	a	a	a	a	a
b	b	a	b	a	a	a	a	b	a
b	b	a	-	a	a	a	a	b	a
b	a	a	b	a	a	a	a	-	a
b	b	a	b	a	a	a	a	b	a
a	a	a	a	-	b	a	b	a	a
a	a	a	a	a	b	a	b	a	a
a	a	a	a	b	b	a	b	-	a
a	a	a	a	a	-	a	b	b	a
a	a	a	a	a	b	a	b	b	a
a	a	a	a	b	b	a	b	b	a
a	a	a	a	b	b	a	b	b	a
a	a	a	a	b	b	a	b	b	a
a	a	a	a	b	b	a	b	b	a
a	a	a	-	b	b	a	b	b	a
a	a	a	a	b	b	a	b	b	a
a	a	a	a	a	b	a	b	b	a
a	a	a	a	b	-	a	b	b	a
a	-	a	a	a	b	a	b	b	a
a	a	a	a	a	b	a	b	b	a
a	b	a	a	a	b	a	b	b	a
a	b	a	a	b	b	a	b	b	a
a	b	a	a	a	b	a	b	b	a
a	b	a	a	a	b	a	b	-	a
a	b	a	a	a	b	a	b	b	a
a	b	a	a	a	b	a	b	b	a
a	b	a	a	b	b	a	b	b	a
a	b	a	a	b	b	a	b	b	a
a	b	a	a	a	b	a	b	b	a
a	b	a	a	a	b	a	b	b	a
a	b	a	a	a	b	a	-	b	a
a	b	a	a	b	b	a	b	b	a
a	b	a	a	a	b	a	b	-	a
a	b	a	a	a	b	a	b	b	a
a	b	a	a	a	b	a	b	b	a
a	b	a	a	b	b	a	b	b	a
b	b	a	a	b	b	a	b	b	a
b	b	a	a	b	b	a	b	b	a
a	b	a	a	a	b	a	b	b	a
a	-	a	a	b	b	a	b	b	a

a	b	a	a	b	b	a	b	b	a
a	b	a	a	b	b	a	b	b	b
a	b	a	a	b	b	a	b	b	b
a	b	a	a	b	b	a	b	b	b
a	b	a	b	a	b	a	b	b	b
a	b	a	b	a	b	-	b	b	b
a	b	a	b	a	b	a	b	b	b
a	b	b	b	a	b	a	b	b	b
b	b	b	b	a	a	a	a	a	a
b	b	b	b	b	a	a	a	a	a
b	b	b	b	a	a	a	a	a	a
b	b	b	b	a	a	a	a	a	a
b	b	b	b	b	a	a	a	a	a
b	b	b	b	a	a	a	a	a	a
b	b	b	b	b	a	a	a	a	a
b	b	b	b	b	a	a	a	a	a
b	b	b	b	a	a	a	a	a	a
b	b	b	b	b	a	a	a	a	a
b	b	b	b	b	a	a	a	a	a
b	b	b	b	b	a	a	a	a	a
b	b	b	b	b	a	a	a	a	a
b	b	b	b	b	a	a	a	a	a
b	a	b	b	b	a	a	a	a	a
b	b	b	b	a	a	a	-	a	a
b	b	b	b	b	a	a	a	a	a
b	b	b	b	b	a	a	a	a	a
b	-	b	b	b	a	a	a	a	a
b	b	b	b	b	a	a	a	a	a
b	b	b	b	b	a	a	a	a	a
b	a	b	b	b	a	-	a	a	a
b	a	b	b	b	a	a	a	a	a
b	a	b	b	a	a	a	a	a	a
b	a	b	b	a	a	a	a	a	a
b	a	b	b	a	a	a	a	b	a
b	a	b	b	a	a	a	a	b	a
b	a	b	b	a	a	a	a	-	a
b	a	b	b	a	-	a	a	b	a
b	a	b	b	a	a	a	a	b	a
b	a	b	-	b	a	a	a	b	a
b	a	b	b	a	a	a	a	b	a
b	a	b	b	b	a	a	a	b	a
b	a	b	b	a	a	a	a	b	a
b	-	b	b	a	a	-	a	b	a
b	a	b	b	b	-	a	a	b	a
b	a	b	b	b	a	a	a	b	a
b	a	b	-	b	a	a	a	b	a
b	a	-	b	b	a	a	a	-	a

b	a	b	b	a	a	a	a	b	a
b	a	b	b	b	a	a	a	b	a
b	a	b	a	a	a	a	a	b	a
b	a	b	a	a	a	a	a	b	a
b	a	b	a	a	a	a	a	b	a
b	a	b	a	a	a	a	a	b	a
b	a	b	a	a	a	a	a	b	b
b	a	b	a	a	a	a	a	b	a
b	a	b	a	a	a	a	a	b	a
b	a	b	a	a	a	a	a	b	a
b	a	b	b	a	a	a	a	b	a
b	a	b	b	a	a	a	a	b	a
b	a	b	b	a	a	a	a	b	a
b	b	b	b	a	a	a	a	b	a
b	a	b	b	a	a	a	a	b	a
b	-	b	b	a	a	a	a	b	a
b	a	b	b	b	a	a	-	b	a
b	a	a	b	a	a	a	a	b	a
b	a	b	a	a	-	a	a	a	a
b	a	b	a	a	-	a	-	a	a
b	b	b	b	a	b	b	a	-	b
b	b	b	b	a	b	b	a	a	b
b	b	b	b	a	b	b	b	a	b
a	b	b	b	a	b	b	b	a	a
b	b	b	b	-	b	b	b	a	b
b	b	b	b	a	b	b	b	a	b
b	b	b	b	a	b	b	b	-	b
b	b	b	b	a	b	b	b	a	b
b	b	b	b	a	b	b	b	a	b
b	b	b	b	a	b	b	b	a	b
b	b	b	b	a	b	-	b	a	b
b	b	b	b	a	b	b	b	a	b
b	b	b	b	a	b	b	b	a	b
b	b	b	b	a	b	b	b	a	b
b	b	b	b	a	b	b	b	a	b
a	b	b	b	a	b	b	b	a	b
a	b	b	b	-	b	b	-	a	b
a	b	b	b	a	b	b	a	a	b
a	b	b	b	a	b	b	a	a	b
a	b	b	b	a	b	b	a	a	b
a	b	b	b	a	b	a	a	a	b
a	b	b	b	a	b	b	a	a	b
a	b	b	b	a	b	-	a	a	b
a	b	b	a	a	b	b	a	a	b
a	b	b	a	a	b	b	a	a	b
a	b	b	a	a	b	b	a	a	b
a	a	b	a	a	b	b	a	a	b

a	a	b	a	a	b	b	a	a	b
a	a	b	a	a	b	b	a	a	b
a	a	b	a	a	b	b	a	a	b
a	a	b	b	a	-	b	a	a	b
a	a	b	b	a	b	b	a	a	b
a	a	b	b	a	-	b	a	a	b
a	a	b	b	a	b	b	a	a	b
a	a	b	b	a	b	b	a	a	b
a	a	b	b	a	b	b	a	a	b
a	a	b	b	a	b	b	a	a	b
a	a	b	b	a	b	b	a	a	b
a	a	b	b	a	b	b	a	a	b
a	a	b	b	a	b	b	a	a	b
a	-	b	b	a	b	b	a	a	b
a	a	b	b	a	b	b	a	a	b
a	a	b	b	a	b	-	b	a	b
a	b	b	b	a	b	b	b	a	b
a	a	b	b	a	b	b	b	a	b
a	-	b	b	a	b	b	b	a	b
a	-	b	b	a	a	b	b	a	b
a	a	b	b	a	a	b	b	a	b
a	a	b	b	a	a	b	b	a	b
a	a	b	b	a	a	b	b	a	b
a	a	b	b	a	a	b	b	a	b
a	a	b	b	a	a	b	b	a	b
a	a	b	b	a	a	b	b	a	b
a	a	b	b	a	a	b	b	a	b
a	-	b	b	-	a	b	b	a	b
b	a	b	b	a	a	b	b	a	b
b	a	b	b	a	a	b	b	a	b
b	a	b	b	a	a	b	b	a	b
b	a	b	b	a	a	b	b	a	b
b	a	b	b	b	a	b	-	a	b
b	b	b	b	a	a	b	b	a	b
b	b	b	b	a	a	b	b	a	b
b	a	a	a	b	b	b	a	a	a
b	-	a	a	b	b	b	a	a	a
b	a	a	a	b	b	b	a	a	a
b	a	a	a	b	b	b	a	a	a
b	a	a	a	a	b	b	a	a	a
b	a	a	-	a	b	b	a	a	a
b	a	a	a	a	b	b	a	a	a
b	a	a	a	a	b	b	a	a	a
b	a	a	a	a	b	b	-	a	a
b	a	a	a	a	-	a	a	b	a
b	a	a	a	b	-	a	a	b	a
b	a	a	a	a	b	a	b	b	a

b	a	a	a	b	b	a	a	b	a
b	a	a	a	a	b	a	a	b	a
b	a	a	a	a	-	a	a	b	a
b	a	a	a	a	b	a	a	b	a
b	a	a	a	a	b	a	b	b	a
b	a	a	a	a	b	a	b	a	a
b	a	a	a	a	b	a	b	a	a
b	a	a	a	a	b	a	b	a	a
b	a	a	a	a	b	a	b	a	a
b	a	a	a	a	b	a	b	a	a
b	a	a	a	b	b	a	b	a	a
b	a	a	a	b	b	a	b	a	a
b	a	a	a	a	b	a	b	a	a
b	a	a	a	a	b	a	b	a	a
b	a	a	a	b	b	a	b	a	a
b	a	a	a	b	b	a	b	a	a
b	a	b	a	b	b	a	b	a	a
b	a	b	a	a	-	a	-	a	a
b	a	b	a	b	b	a	b	a	a
b	a	b	a	a	b	a	b	a	a
b	a	b	a	a	b	a	b	a	b
b	a	b	a	a	b	a	b	a	b
b	a	b	a	b	b	a	b	a	b
b	a	b	a	a	b	a	b	a	b
b	a	b	a	-	b	a	b	a	b
b	a	b	a	b	-	a	b	a	b
b	a	b	a	b	b	a	b	a	b
b	a	b	a	b	b	a	b	a	b
b	a	b	a	b	b	a	b	a	b
b	-	b	a	a	b	a	b	a	b
b	a	b	a	b	b	a	b	a	b
b	a	b	a	b	b	a	b	a	b
b	b	b	a	b	b	a	b	a	b
b	a	b	a	a	b	a	b	a	b
b	a	b	a	b	b	a	b	a	b
b	a	b	a	a	-	a	b	a	b
b	a	b	a	b	b	a	a	b	b
b	a	a	a	b	b	a	b	b	b
b	a	b	a	a	b	a	b	b	b
b	a	b	a	a	b	a	b	b	b
a	a	b	a	a	b	a	-	b	b
a	a	b	a	a	-	a	b	b	b
a	a	b	a	b	b	a	b	b	b
a	a	b	a	b	b	a	b	b	b
a	a	b	a	a	b	a	b	b	b
a	a	b	a	a	b	a	b	b	b
a	a	a	a	b	a	a	a	b	a

a	a	a	a	b	a	a	a	b	a
a	a	a	a	b	a	a	a	a	a
a	a	a	a	a	a	a	a	a	a
a	a	a	a	a	a	a	a	a	a
a	a	a	a	a	a	a	a	a	a
a	a	a	a	a	a	a	a	a	a
a	a	a	a	b	a	a	a	a	b
a	a	a	a	b	a	a	a	a	b
a	a	a	a	b	a	a	a	a	b
a	b	a	a	a	a	a	a	a	b
a	b	a	a	a	a	a	a	a	b
a	b	a	a	b	a	a	a	a	b
a	b	a	a	b	a	a	a	a	b
a	b	a	a	b	a	a	a	a	b
a	b	a	a	b	a	a	a	a	b
a	b	a	a	a	a	a	a	a	b
a	b	a	a	a	a	a	a	a	b
a	b	a	a	b	a	a	a	a	b
a	b	a	a	b	a	a	a	a	b
a	b	a	a	b	a	a	a	a	b
a	b	a	a	b	a	a	a	a	b
a	b	a	a	b	a	a	a	a	b
a	b	a	a	b	a	a	a	a	b
a	b	a	a	b	a	a	a	a	b
a	b	a	a	b	a	a	a	a	b
a	b	a	a	b	a	a	a	a	b
a	b	a	a	b	a	a	a	a	b
a	b	a	a	b	a	a	a	a	b
a	b	a	a	b	a	a	a	a	b
a	b	a	a	b	a	a	a	a	b
a	b	a	a	b	a	a	a	a	b
b	b	a	a	b	b	a	a	a	b
b	b	a	a	a	b	a	a	a	b
b	b	a	a	a	b	a	a	a	b
b	b	a	a	a	-	a	a	a	b
b	b	a	a	a	a	a	a	a	b
b	b	a	a	a	a	a	a	a	b
b	b	a	a	a	a	a	a	a	b
b	b	a	a	a	b	a	a	a	b
b	a	a	a	a	a	b	a	a	b
a	b	b	b	a	b	b	b	b	a
b	a	a	a	-	a	a	a	a	a
b	a	a	a	b	a	a	a	a	a
b	a	a	a	b	a	a	a	a	a
b	a	a	a	a	a	a	a	a	a
b	a	a	a	a	a	a	a	a	a
b	a	a	a	a	a	a	a	a	a

b	a	a	a	a	a	a	a	a	a
b	a	a	a	a	a	a	a	a	a
b	a	b	a	b	a	a	a	a	a
b	a	b	a	b	a	a	a	a	a
b	a	b	a	a	a	a	a	a	a

YW151	YW152	YW153	YW154	YW155	YW156	YW157	YW158	YW159	YW160
a	a	b	a	b	a	a	a	-	b
a	b	b	a	b	a	a	a	b	b
a	b	b	a	b	a	a	a	b	b
a	b	b	a	b	a	a	a	b	b
a	b	b	a	b	a	a	a	b	b
a	b	b	a	-	a	a	a	b	b
a	b	b	a	b	a	a	a	b	-
a	b	b	a	b	a	a	a	b	b
a	b	b	-	b	a	a	-	b	b
a	b	b	a	b	a	a	a	b	b
a	b	b	a	b	a	a	a	b	b
a	b	b	a	b	a	a	a	b	b
a	b	-	a	b	a	a	a	b	b
a	b	b	a	b	a	-	a	b	b
a	b	b	a	b	a	-	a	b	b
a	b	b	a	b	a	a	a	b	b
a	b	b	a	b	a	a	-	b	b
a	b	b	a	b	a	a	a	b	b
a	b	b	-	b	a	a	a	b	b
a	b	b	a	b	a	a	a	b	b
a	b	b	a	a	a	a	a	b	b
a	b	b	a	b	a	a	a	b	b
a	b	b	a	b	a	a	a	b	b
a	b	b	a	b	a	a	a	b	b
a	b	b	a	b	a	a	a	b	b
a	b	b	a	-	a	a	a	b	b
a	b	b	a	b	a	a	-	b	b
a	b	b	a	b	a	a	a	-	b
a	b	b	a	a	a	a	a	b	-
a	b	b	a	a	a	a	-	b	b
a	b	b	a	a	a	a	a	b	b
a	b	b	a	a	a	a	a	b	b
a	b	b	-	a	b	a	a	b	b
a	b	b	a	a	a	a	a	b	b
a	b	b	a	a	a	a	a	b	b
a	b	b	a	a	a	a	a	b	b
a	b	b	a	a	a	a	a	b	b
a	b	b	a	a	a	a	a	b	b
a	b	b	a	b	a	a	a	b	b
a	b	b	a	a	a	a	a	b	b
a	b	a	a	a	a	a	a	b	b
a	b	a	a	a	a	a	a	b	b
a	b	a	a	a	a	a	a	b	b
-	b	a	a	a	a	a	a	b	b
b	b	a	a	a	-	a	-	b	b

b	b	a	a	a	a	-	a	b	b
b	b	a	a	a	a	a	a	b	b
b	b	a	a	a	a	a	a	b	b
b	b	a	a	a	a	a	a	b	b
b	b	a	a	a	a	a	a	b	b
b	b	a	a	a	a	a	a	b	b
b	b	a	a	-	a	a	a	a	b
b	b	a	a	a	a	b	a	a	b
b	b	a	a	a	a	a	a	a	b
b	b	a	a	a	a	b	a	a	b
b	b	a	a	a	a	b	a	a	b
b	b	a	a	a	a	b	a	a	b
b	b	a	a	a	a	b	a	a	b
b	b	a	a	a	-	b	a	a	b
b	b	a	a	a	a	b	a	a	a
a	b	a	a	a	a	b	a	a	a
a	b	a	a	a	a	b	a	a	a
a	b	a	a	a	a	b	a	a	a
a	a	a	a	a	a	b	a	a	a
a	a	a	a	a	a	b	a	a	a
a	a	a	a	a	a	b	a	a	a
a	a	a	a	a	a	b	a	a	a
a	a	a	a	a	a	-	a	a	a
a	a	a	a	a	a	b	a	a	a
a	a	a	a	a	a	b	a	a	a
a	a	a	a	a	a	b	a	a	a
a	a	a	a	a	a	b	a	a	a
a	a	a	a	a	a	b	a	a	a
a	a	a	a	-	a	b	a	a	a
a	a	a	a	a	a	b	a	a	a
a	a	a	a	a	a	b	a	a	a
a	a	a	a	a	a	b	-	a	a
a	a	a	a	a	a	b	a	a	a
a	a	a	a	a	a	b	-	a	a
a	a	a	a	a	a	b	a	a	a
a	a	a	a	a	a	-	a	a	a
a	a	a	a	a	a	b	a	a	a
a	a	a	a	a	a	b	a	a	a
a	a	a	a	a	a	a	a	a	a
b	b	a	b	b	-	b	b	a	a
b	b	a	b	b	b	b	b	a	-
-	b	a	b	a	b	b	b	a	a
b	b	a	b	b	b	b	b	a	a
b	b	a	b	b	b	b	b	a	a
b	b	a	b	b	b	b	b	a	a
b	b	a	b	b	b	b	b	a	a
b	b	a	b	b	b	b	b	a	a

b	b	a	b	b	b	b	b	a	a
b	b	a	b	b	b	b	b	a	a
b	b	a	-	b	b	b	b	a	a
-	b	a	b	b	b	b	b	a	a
b	b	a	b	b	b	b	b	a	a
b	b	a	b	b	b	b	b	a	a
b	b	a	-	-	b	b	b	a	a
b	b	a	b	b	b	b	b	a	b
b	b	a	b	b	b	-	b	a	b
b	b	a	b	b	b	b	b	a	b
b	b	a	-	b	b	b	b	a	b
b	b	a	b	b	b	b	b	a	b
b	b	a	b	b	b	b	b	a	b
b	b	a	b	b	b	a	b	a	b
b	b	a	b	b	b	a	b	a	b
b	b	-	b	b	b	a	b	a	b
b	b	a	b	b	b	a	b	a	b
b	b	a	b	b	b	a	b	a	b
b	b	a	b	b	b	a	b	a	b
b	b	a	b	b	b	a	b	a	b
b	b	a	b	b	b	a	b	a	b
b	b	a	b	b	b	a	b	a	b
b	b	a	b	b	b	-	b	a	b
b	b	a	b	b	b	a	-	a	b
b	b	a	b	b	b	a	a	a	b
b	b	a	b	b	b	a	a	a	b
b	b	a	b	b	b	a	a	a	b
-	b	a	b	b	-	a	a	a	b
b	b	a	b	b	b	-	a	a	b
b	b	a	-	-	b	a	a	a	b
b	b	a	b	b	-	a	a	a	b
b	b	a	b	b	-	a	a	a	-
b	b	a	b	b	b	a	a	a	b
b	b	a	b	b	b	a	a	a	-
-	-	a	b	b	b	a	a	a	b
b	b	a	b	b	b	a	a	a	b
b	b	a	b	b	b	a	a	a	b
b	b	a	b	b	b	a	a	a	b
b	b	a	b	b	b	a	a	a	b
b	b	a	b	b	b	a	a	a	b
b	b	a	b	b	b	a	a	a	b
b	b	a	-	b	b	a	a	a	b
b	b	a	b	b	b	a	a	a	b
b	-	a	b	b	b	a	a	a	b
b	b	a	a	b	b	b	a	b	b
b	b	a	a	b	b	b	a	b	b
b	b	a	a	b	-	b	a	b	b

b	b	a	a	b	b	b	-	b	b
b	b	a	a	b	-	b	a	b	b
b	b	a	a	b	b	b	a	b	b
b	b	a	a	b	b	b	a	b	b
b	b	a	a	b	b	b	a	b	b
b	b	a	a	b	b	b	-	b	b
b	b	a	a	b	b	b	a	b	-
b	b	a	a	b	b	b	a	b	b
b	b	a	a	b	b	b	a	b	b
b	a	a	a	b	b	b	a	b	b
b	a	a	a	b	b	b	a	b	b
b	a	a	a	b	b	b	a	b	b
b	a	a	a	b	b	b	a	b	b
b	a	a	a	b	b	b	a	b	b
b	a	a	a	b	b	b	a	b	b
b	a	a	-	b	b	b	a	b	b
b	a	a	a	b	b	b	a	b	b
b	a	a	a	b	b	b	a	b	b
b	a	a	a	b	b	b	a	b	b
b	a	a	a	b	b	b	a	b	b
b	a	a	a	b	b	b	a	-	b
b	a	-	a	b	b	b	a	b	b
b	a	a	a	b	b	b	a	b	b
b	a	a	a	b	b	b	a	b	b
b	a	a	a	b	b	b	a	b	-
b	a	a	a	b	b	b	a	b	b
b	a	a	a	b	b	b	a	b	b
b	a	a	a	b	b	b	a	b	b
b	a	a	a	b	-	b	a	b	b
b	a	b	a	b	b	b	-	b	b
b	a	b	a	b	b	b	a	b	b
b	a	b	a	b	b	b	a	b	b
b	a	b	a	b	b	-	a	b	b
b	a	b	a	b	b	b	a	b	b
b	a	b	a	b	b	b	-	b	b
b	a	b	a	b	b	b	a	b	b
b	a	b	a	b	b	b	a	b	a
b	a	b	a	b	b	b	a	b	a
b	a	b	a	b	b	b	a	b	a
b	a	b	a	b	-	b	a	b	a
b	a	b	a	b	b	b	a	b	a
b	a	b	a	-	-	b	a	b	a
b	a	b	a	b	a	b	a	b	a
b	a	b	a	b	a	-	a	b	a
b	a	b	a	b	a	b	a	b	a
b	a	b	a	b	a	-	a	b	a
b	a	b	a	b	a	b	a	b	a

b	a	b	a	b	a	b	a	b	a
b	a	b	a	b	a	b	a	b	a
b	a	b	a	b	-	b	a	b	a
b	a	b	a	b	-	b	a	b	a
b	a	b	a	b	a	b	a	b	a
b	a	b	a	b	a	b	a	b	a
b	a	b	a	b	a	b	a	b	a
b	a	b	a	b	a	b	a	b	a
b	a	b	a	-	a	-	a	b	a
b	a	b	a	b	-	b	a	b	-
b	a	b	a	a	a	-	a	b	a
b	a	b	a	b	a	b	a	b	a
a	a	b	a	b	b	b	b	b	b
a	a	b	a	b	b	-	b	b	b
a	a	b	a	b	b	b	a	b	b
a	a	b	a	b	b	b	a	b	b
a	a	b	a	b	b	b	a	b	b
a	a	b	a	b	b	-	a	b	b
a	a	b	a	b	b	b	a	b	b
a	a	b	a	b	b	b	a	b	b
a	a	b	a	b	b	b	a	b	b
a	a	b	a	b	b	b	a	b	b
a	a	b	a	b	b	b	a	b	b
a	a	b	a	b	b	b	a	b	b
a	a	b	a	b	b	b	a	b	b
a	a	b	a	b	b	b	a	b	b
a	a	b	a	b	b	b	a	b	b
a	a	b	a	b	b	b	a	b	b
a	a	b	a	b	b	b	-	b	b
a	a	b	a	b	b	b	-	b	a
a	a	b	a	b	b	b	a	b	a
a	a	b	a	b	b	b	a	b	a
a	a	b	a	b	b	b	a	b	a
a	a	b	a	b	b	b	a	b	a
a	a	b	a	b	b	b	a	b	a
a	a	b	a	b	b	b	a	b	a
a	a	b	-	b	b	b	a	b	a
a	a	b	a	b	b	b	a	b	a
a	a	b	a	b	b	b	a	b	a
a	a	b	a	b	b	b	a	b	a
a	a	b	a	b	b	b	a	b	a
a	a	b	a	b	b	b	a	b	a
a	a	b	a	b	b	b	a	b	a
a	a	b	a	b	b	b	a	b	a
a	a	b	a	b	b	b	a	b	a
a	a	b	a	b	b	-	a	b	a
a	a	b	a	b	b	b	a	b	a

a	a	b	a	b	b	b	a	a	a
a	a	b	a	b	-	b	a	a	a
a	a	b	a	b	b	b	a	a	a
a	a	b	a	b	b	b	a	a	a
a	a	b	a	b	b	b	a	a	a
a	a	b	a	b	b	b	a	a	a
a	a	b	a	b	b	b	a	a	a
a	a	b	a	b	b	b	a	a	a
a	a	b	a	b	b	b	a	-	a
a	a	b	a	b	b	b	a	b	a
a	a	b	a	b	b	b	a	b	a
a	a	b	a	b	b	b	a	-	a
a	a	b	a	b	b	b	a	b	a
a	a	b	a	b	b	b	a	b	a
a	a	b	a	b	b	b	a	b	a
a	a	b	a	b	b	b	a	b	a
a	a	b	a	b	b	b	a	b	a
a	a	b	a	b	b	b	a	b	a
a	a	b	a	b	-	b	a	b	a
a	a	b	a	b	b	b	a	b	a
a	-	b	a	b	b	b	a	b	a
a	a	b	a	b	b	b	a	b	a
a	a	b	a	b	b	b	a	b	a
a	a	b	a	b	b	b	a	b	a
a	a	b	a	b	-	b	a	b	a
a	a	b	a	b	b	b	a	b	a
a	a	b	a	b	b	b	a	b	a
a	a	b	a	b	b	b	a	b	a
a	a	b	a	b	b	b	a	b	a
a	a	b	a	b	b	b	a	b	a
a	a	b	b	b	b	b	a	b	a
a	a	b	-	b	b	b	a	b	a
a	a	b	b	b	b	-	a	b	a
a	a	b	b	b	a	b	a	b	a
a	a	b	b	b	a	b	a	b	a
a	a	b	b	b	a	b	a	b	a
a	a	b	b	b	a	b	a	b	a
a	a	b	b	b	a	b	a	b	a
a	a	b	b	b	a	b	a	b	a
a	a	b	b	b	a	b	a	b	a
a	a	b	b	b	a	b	a	b	a
a	a	b	b	b	a	b	a	b	a
a	a	b	b	a	a	b	a	b	a
a	a	b	b	b	a	b	a	b	a
a	a	b	b	b	a	b	a	b	a
a	a	b	b	b	a	b	a	b	a
a	a	b	b	-	a	b	a	b	a

a	a	b	b	b	a	-	a	b	a
a	a	b	b	b	a	b	-	b	a
a	a	b	b	b	a	b	a	b	a
a	a	b	b	-	a	b	a	b	a
a	a	b	b	b	a	b	a	b	a
a	a	b	b	b	a	b	a	b	a
a	a	b	b	b	a	b	a	b	a
a	a	b	b	b	-	b	a	b	a
a	a	b	b	b	a	b	a	b	a
a	a	b	b	b	a	b	a	b	a
a	a	b	b	b	a	b	a	b	a
a	a	b	b	b	a	b	a	b	a
a	-	b	b	b	a	b	a	b	a
a	a	b	b	b	a	b	a	-	a
a	a	b	b	b	a	b	a	b	a
a	a	b	b	b	a	b	a	b	a
a	a	b	b	b	-	b	a	b	a
a	a	b	b	b	a	b	a	b	a
a	a	b	b	b	a	b	a	b	-
a	a	b	b	b	a	b	a	b	a
a	a	b	b	b	-	b	-	b	a
a	a	b	b	b	-	b	a	b	a
a	a	b	b	b	-	b	a	b	a
a	a	b	b	b	a	b	a	b	-
a	a	b	b	b	a	b	a	b	a
a	a	b	b	-	a	b	a	b	a
a	a	b	b	b	a	b	a	b	a
a	a	b	b	b	a	b	a	b	a
a	a	b	b	b	a	b	a	b	a
a	a	b	b	b	a	b	a	b	a
a	a	b	b	b	a	b	a	b	a
a	a	b	b	b	a	b	a	b	a
a	a	b	b	b	a	b	a	b	a
a	a	b	b	b	a	b	a	b	a
a	a	b	b	b	a	-	a	b	a
a	a	b	b	b	a	b	a	b	b
a	a	b	b	b	a	b	a	b	b
a	a	b	b	b	-	b	a	b	b
b	a	a	a	a	b	a	a	a	a
b	a	a	a	a	b	a	a	a	a
b	a	a	a	a	b	b	a	a	a
b	a	a	a	a	b	b	a	a	a
b	a	a	a	a	-	b	a	a	a
b	a	a	a	-	b	b	a	a	a
b	a	a	a	a	b	b	a	a	a
b	a	a	a	a	b	b	a	a	b
b	a	a	a	a	b	b	a	a	b

b	a	a	a	a	b	b	-	a	b
b	a	a	a	a	b	b	a	a	b
b	a	a	a	a	-	b	a	a	b
b	a	a	a	a	b	b	a	a	b
b	a	a	a	a	b	b	a	a	b
b	a	a	a	-	b	b	a	a	b
b	a	a	a	a	b	b	a	a	b
b	a	a	a	a	b	b	a	a	b
b	a	-	a	a	b	b	a	a	b
b	a	a	a	a	-	b	a	a	b
b	a	a	a	a	b	b	a	a	b
b	a	a	a	a	b	b	a	a	b
b	b	a	a	a	b	b	-	a	b
b	a	a	a	a	b	b	a	a	b
b	-	a	a	a	b	b	a	a	b
b	a	a	a	a	a	-	a	a	b
b	a	a	a	-	a	b	a	a	b
b	a	a	a	a	a	b	a	a	b
b	a	a	a	a	a	b	a	a	b
b	a	a	a	b	a	-	a	a	b
b	a	a	a	b	a	b	a	a	b
b	a	a	a	b	a	b	a	a	b
b	a	a	a	b	a	b	a	a	b
b	-	a	a	b	a	b	-	a	b
b	a	a	a	b	a	b	a	a	b
b	a	a	a	b	a	b	a	a	b
b	a	a	a	b	a	b	a	a	b
b	a	a	a	-	a	b	a	a	b
b	a	a	a	b	a	b	a	a	b
b	b	a	a	b	a	b	a	a	b
b	b	a	-	b	a	b	-	a	b
b	b	a	a	b	a	b	a	a	b
b	b	a	a	b	a	b	-	a	b
b	b	a	a	b	a	-	a	a	b
b	b	a	a	b	a	b	a	-	b
b	b	a	a	b	a	b	a	a	b
b	b	a	a	b	a	b	a	a	b
b	b	a	a	b	a	b	a	a	b
b	b	a	a	b	a	b	a	a	b
b	b	a	a	b	a	b	a	b	a
b	b	a	a	b	a	b	a	a	b
b	b	a	a	b	a	b	a	a	b
b	b	a	a	b	a	b	a	a	b
b	b	a	a	b	a	b	a	a	b
b	b	a	a	b	a	b	-	a	b
b	b	a	a	b	a	-	a	a	b
b	b	a	a	b	a	-	a	a	b

b	b	a	a	b	a	b	a	a	b
b	b	a	a	b	a	b	a	a	b
b	-	a	a	b	a	b	a	a	b
b	b	a	a	b	-	b	a	a	b
b	b	a	a	b	a	b	a	a	b
b	b	a	a	-	a	-	a	a	b
b	b	a	a	b	a	b	a	a	-
b	b	a	a	b	a	b	b	a	b
b	b	a	a	b	a	b	-	a	b
b	b	a	a	b	a	b	-	a	b
b	b	a	a	b	a	b	b	a	b
-	b	a	b	b	a	b	-	a	b
b	b	a	b	b	a	b	b	a	b
b	b	a	a	b	a	b	b	a	b
b	b	a	b	b	a	b	b	a	b
b	b	a	b	b	a	b	b	a	b
b	b	a	b	b	a	b	b	a	b
b	b	a	b	b	a	b	b	a	b
b	b	a	b	b	a	b	b	a	b
b	b	a	b	b	a	b	b	a	b
b	b	a	b	b	a	b	b	a	b
b	b	a	b	b	a	b	b	a	b
b	-	a	b	b	a	b	b	a	b
b	b	a	b	b	a	b	b	a	b
b	b	a	b	b	a	b	b	a	b
b	b	a	b	b	a	b	b	a	-
b	b	a	b	b	a	b	-	a	b
b	b	a	-	b	a	b	b	a	b
b	b	a	b	b	a	b	b	a	b
b	b	a	b	-	a	b	b	a	b
b	b	a	b	b	a	b	b	a	b
b	b	a	b	b	a	b	-	a	b
b	b	a	b	b	a	b	b	a	b
b	b	a	b	b	-	b	b	a	b
b	b	a	b	b	a	b	b	a	b
b	b	a	b	b	a	b	b	a	b
b	b	a	b	b	a	-	b	a	b
b	b	a	b	b	a	b	b	a	b
b	b	a	b	b	a	b	b	a	-
b	b	a	b	b	a	b	b	a	b
b	b	b	-	b	b	b	a	b	b
b	b	b	b	b	b	b	a	b	b
b	b	b	b	b	b	b	a	b	b
b	b	b	b	b	b	b	a	b	b
b	b	b	b	b	b	b	a	b	b
b	b	b	b	b	b	b	a	b	b
b	b	b	b	b	b	b	a	b	b

b	b	b	b	b	b	b	b	b	b
b	-	b	b	b	b	b	b	b	b
b	b	b	b	b	b	b	b	b	b
b	b	b	b	b	b	b	b	b	b
b	b	b	b	b	b	b	b	b	b
b	b	b	b	b	b	b	b	b	b
b	b	b	b	b	b	b	b	b	b
b	b	b	b	b	b	b	b	b	b
b	b	b	b	b	b	b	b	b	b
b	b	b	b	b	-	b	-	b	b
b	b	b	b	b	b	b	b	b	b
b	b	b	b	b	b	b	b	b	b
b	b	b	b	b	b	b	b	b	b
b	b	b	b	b	b	b	b	b	b
b	b	b	b	b	-	b	-	b	b
b	b	b	b	b	b	b	b	b	b
b	b	b	b	b	b	a	b	b	b
b	b	b	b	b	b	a	b	b	b
b	b	b	b	b	b	a	b	b	b
b	b	b	b	b	b	-	b	b	b
b	b	b	b	b	b	a	b	b	b
b	b	b	b	b	b	a	b	b	b
b	b	b	b	b	b	a	b	b	b
b	b	b	b	b	b	a	b	b	b
b	b	b	b	b	b	a	-	b	b
b	b	b	b	b	b	a	b	b	b
b	b	b	b	b	b	a	b	b	b
b	b	b	b	b	b	a	b	b	b
b	b	b	b	b	b	a	b	b	b
b	b	b	b	b	b	a	b	b	b
b	b	b	b	b	b	a	b	b	b
b	b	b	b	b	b	a	b	b	b
b	b	b	b	b	b	a	b	b	-
b	b	b	b	b	b	a	b	b	b
b	b	b	b	b	b	a	b	b	b
b	b	b	-	b	b	a	b	b	b
b	b	b	b	b	b	a	b	b	b
b	b	b	b	b	b	-	b	b	b
b	b	b	b	b	b	a	b	b	b
b	b	b	b	b	b	-	b	b	b
b	b	b	b	b	b	a	b	b	b
b	b	b	b	b	b	a	b	b	b
b	b	b	b	b	b	a	b	b	b
b	b	b	b	b	b	a	b	b	b
b	b	b	b	b	b	a	b	b	b
b	b	b	b	b	b	a	b	b	b
b	b	b	b	b	b	a	-	b	b

b	b	b	b	b	b	a	b	b	b
b	b	b	b	b	a	a	b	b	b
b	b	b	b	b	a	-	b	b	b
b	b	b	b	b	b	a	b	b	b
b	b	b	b	b	b	a	-	b	-
b	b	b	b	b	b	a	-	b	b
b	b	b	b	b	b	a	b	b	b
b	b	b	b	b	b	a	b	b	b
b	b	b	b	b	b	a	b	b	b
b	b	b	b	b	b	a	b	b	b
b	b	b	b	b	b	a	b	b	b
b	b	b	b	b	b	a	b	b	b
b	b	b	b	b	b	a	b	b	b
a	b	b	b	b	b	a	b	b	b
a	b	b	b	b	b	a	b	b	b
a	b	b	b	b	-	a	b	b	-
a	b	b	b	b	b	a	b	b	b
a	b	b	b	b	b	a	-	b	b
a	b	b	b	b	b	a	b	b	b
a	b	b	-	-	b	a	-	b	b
a	b	b	b	b	b	a	b	b	b
a	b	b	b	b	b	a	b	b	b
a	b	b	b	b	b	a	b	b	b
a	b	b	b	b	b	a	b	b	b
a	b	b	b	b	b	a	b	b	b
a	b	b	b	b	b	a	b	b	b
a	b	b	b	b	b	a	b	b	b
a	b	b	b	b	b	a	b	b	b
a	b	b	b	b	b	a	-	b	b
a	b	b	b	b	b	a	b	b	b
a	b	b	b	b	b	a	b	b	b
a	b	b	b	b	b	a	b	b	b
a	b	b	b	b	b	a	b	b	b
a	b	b	b	b	b	a	b	b	b
a	a	b	b	b	b	a	b	b	b
a	a	b	b	b	b	-	b	b	b
a	a	b	b	b	b	a	b	b	-
a	a	b	b	b	b	a	b	b	b
a	a	b	b	b	b	a	b	b	b
a	a	b	b	b	b	a	b	b	b
a	a	b	b	b	b	a	b	-	b
a	a	b	b	b	b	a	-	b	b
a	a	b	b	b	b	a	b	b	b
a	a	b	b	b	b	a	b	b	b
a	a	b	b	b	-	a	b	b	b
a	a	b	b	b	b	a	b	b	b
a	a	b	b	b	-	a	b	b	b

-	a	b	b	b	b	a	a	b	b
a	a	b	b	b	b	a	-	b	b
a	a	b	b	b	b	a	a	b	b
a	a	b	b	b	b	a	a	b	b
a	a	b	b	b	b	-	a	b	b
a	a	b	b	b	b	a	a	b	b
a	a	b	b	b	b	a	a	b	b
a	a	b	b	b	b	-	a	b	b
a	a	a	b	a	a	a	-	b	b
a	a	a	b	a	a	a	a	b	b
a	a	a	b	a	a	a	a	b	b
a	a	a	b	a	-	a	a	b	b
a	a	a	b	a	a	a	a	b	b
a	a	a	-	a	a	a	a	b	b
a	a	a	b	a	a	a	a	b	b
a	a	a	b	a	a	a	a	b	b
a	a	a	b	a	a	a	a	b	b
a	a	a	b	a	a	a	a	b	b
a	a	a	b	a	a	a	a	b	b
b	a	a	b	b	a	b	a	a	b
b	a	a	b	b	a	b	a	a	b
b	a	a	b	b	a	b	a	a	b
b	a	a	a	b	a	b	a	a	b
b	a	a	b	b	a	b	a	a	b
b	a	a	b	b	a	b	a	a	b
b	a	a	b	b	a	-	a	a	b
b	a	a	b	b	a	-	a	a	b
b	a	a	b	b	a	b	a	a	b
b	a	a	b	b	a	b	a	a	b
b	a	a	b	b	-	b	a	a	b
b	a	a	b	b	a	b	a	a	b
b	a	a	b	b	a	b	a	a	b
b	a	a	b	b	a	b	a	a	b
b	a	a	b	b	a	b	a	a	b
b	a	a	b	b	a	b	a	a	b
b	a	a	b	-	a	-	a	a	b
b	a	a	b	b	a	b	a	a	b
b	a	a	b	b	a	b	a	a	b
b	a	a	b	-	a	b	a	a	b
b	-	a	b	b	a	b	a	a	b
b	a	a	b	b	a	b	a	a	b
b	-	a	b	b	a	b	a	a	b
b	-	a	b	b	a	b	a	a	b
b	a	a	b	b	a	b	a	a	b
b	a	a	b	b	a	b	a	a	b
b	a	a	b	b	a	b	a	a	b
b	-	a	b	b	a	b	a	a	b

b	a	a	b	b	a	b	a	a	b
b	a	a	b	-	a	b	a	a	b
b	a	a	b	b	a	b	a	a	b
b	a	a	b	b	a	b	a	a	b
b	a	a	b	b	a	b	a	a	-
b	a	a	b	b	a	b	a	a	b
b	a	a	b	b	a	b	a	a	b
b	a	a	b	b	a	b	a	b	b
b	a	a	b	b	a	b	a	b	b
b	b	a	b	b	a	b	a	b	b
b	a	a	b	b	a	b	a	b	b
b	a	a	b	b	a	b	a	b	b
b	a	a	b	a	a	b	a	b	b
b	a	a	b	a	a	b	a	b	b
b	a	a	b	a	a	a	a	b	b
b	-	a	b	a	a	a	a	b	b
b	b	a	b	a	a	a	a	b	b
b	a	a	b	a	a	a	a	b	b
b	a	a	b	a	a	a	a	b	b
b	a	a	b	a	-	a	a	b	b
b	a	a	b	a	a	a	a	a	b
b	a	a	b	a	a	a	a	a	b
b	a	a	-	a	a	a	a	b	b
b	a	a	b	a	a	a	a	a	b
b	a	a	b	a	a	a	a	a	b
b	-	a	b	a	a	a	a	a	b
b	a	a	b	a	a	a	a	a	b
b	a	a	b	a	a	a	a	a	-
a	a	a	b	a	a	a	a	a	b
a	-	a	b	a	a	a	a	a	b
a	b	a	b	a	a	a	a	a	b
a	a	a	b	a	a	a	a	a	b
a	b	a	b	a	-	a	-	a	b
a	b	a	b	a	-	a	a	b	b
a	a	a	b	a	a	a	a	b	b
a	b	a	b	-	a	a	a	b	b
a	-	b	b	a	a	a	a	b	a
b	b	b	b	a	a	a	a	b	a
a	-	b	b	a	a	a	a	b	a
a	-	b	b	a	a	a	a	b	a
a	-	b	b	a	a	a	a	b	a
a	b	b	b	a	a	a	a	b	a
a	b	b	-	a	a	a	a	b	a
a	-	-	-	a	a	-	a	b	a
a	a	b	a	a	a	a	a	b	a
a	b	b	b	a	a	a	a	b	a
a	b	b	b	a	a	a	a	b	a

a	b	b	-	a	a	a	a	b	a
a	-	b	b	a	a	a	a	b	a
a	b	b	b	a	a	a	a	b	a
a	b	b	b	a	a	a	a	b	a
a	b	b	b	a	a	a	a	b	a
a	b	b	b	a	a	a	a	b	a
a	b	b	b	a	a	a	a	b	a
a	b	b	b	a	a	a	a	b	a
a	-	b	b	a	a	a	a	b	a
a	a	b	-	a	a	a	a	b	a
a	a	-	b	a	a	a	b	-	a
a	b	-	b	a	a	a	a	a	a
a	b	b	b	a	a	a	a	a	a
a	b	b	b	b	-	a	a	a	a
a	b	-	b	a	a	a	a	a	a
a	b	b	b	a	a	a	a	a	a
a	b	b	b	a	a	a	a	a	a
a	b	b	b	a	a	a	a	a	a
a	b	b	b	b	a	a	a	a	a
a	b	b	b	-	a	a	a	a	a
a	b	b	b	b	a	a	a	a	a
a	b	b	b	b	a	a	a	a	a
a	b	b	b	b	a	a	a	a	a
a	b	b	b	b	a	a	a	a	a
a	b	b	b	b	a	a	a	a	a
a	b	b	b	b	a	a	a	a	a
a	b	b	b	b	a	a	a	a	a
a	b	b	-	b	a	a	a	a	a
a	b	b	b	b	a	a	a	a	a
a	b	-	b	b	a	a	b	a	a
a	b	b	b	b	a	a	b	a	a
a	b	b	b	b	a	a	b	a	a
a	b	b	b	b	a	a	b	a	a
a	b	b	b	b	a	a	b	a	a
a	b	b	b	b	a	a	b	a	a
a	b	b	b	b	a	a	b	a	a
a	b	b	b	b	a	a	b	a	a
a	b	b	b	b	a	a	b	a	a
a	b	b	b	b	-	a	b	a	a
a	b	b	b	b	a	a	b	a	a
a	b	b	b	b	a	a	b	a	a
a	b	b	b	b	a	a	b	a	a
a	b	b	b	b	-	a	b	a	a
a	a	a	b	b	a	a	b	a	a
a	a	a	b	b	b	a	b	a	a
a	a	a	b	b	a	a	b	a	a
a	a	a	b	b	a	a	b	a	a

a	a	a	b	b	-	a	b	a	a
a	a	a	b	b	a	a	b	a	a
a	a	a	b	b	a	a	b	a	a
a	a	a	b	b	a	a	-	a	a
a	a	a	b	b	a	a	b	a	a
a	a	a	b	b	a	a	b	a	a
a	a	a	b	b	a	a	b	a	a
a	a	a	b	b	a	a	b	a	a
a	a	a	b	b	a	a	b	a	a
a	a	a	b	b	a	a	b	a	a
a	a	a	b	b	a	a	b	a	a
a	a	-	b	b	a	a	b	a	a
a	a	-	b	b	a	a	b	a	a
a	a	a	b	b	a	a	b	a	a
a	a	a	b	b	a	a	b	a	a
a	a	a	b	b	a	a	b	a	a
b	a	a	b	b	a	a	b	a	a
b	a	a	b	b	a	a	b	a	a
b	a	a	b	b	a	a	-	a	a
b	a	a	b	b	-	a	b	a	a
b	a	a	b	b	a	b	b	a	a
b	a	a	b	b	a	a	b	a	a
b	a	a	b	b	a	a	b	a	a
b	a	a	b	b	a	a	b	a	a
b	a	-	b	b	a	a	b	a	a
b	a	a	b	b	a	a	b	a	a
b	a	a	b	b	a	a	-	a	a
b	a	a	b	b	-	a	b	a	a
b	a	a	b	b	a	a	b	a	a
b	a	a	b	b	a	a	b	a	a
b	a	a	b	b	a	a	b	a	a
b	a	a	b	b	a	a	b	a	a
b	a	a	b	b	a	a	b	a	a
b	a	a	b	b	a	a	b	a	a
b	a	a	b	b	a	a	b	-	a
b	a	a	b	b	a	a	b	a	a
b	a	a	b	b	a	-	b	a	a
b	a	-	b	b	a	a	b	a	a
b	a	a	b	b	a	a	b	a	a
b	a	a	b	b	a	a	b	a	a
b	a	a	b	b	a	a	b	a	a
b	a	a	b	a	a	a	b	a	a
b	a	a	b	a	a	a	b	a	a
b	-	a	b	a	a	a	b	a	a
b	a	a	b	a	a	a	b	a	a
b	a	a	b	a	a	a	b	a	a
b	a	-	b	a	a	a	b	a	a

b	a	a	b	a	a	a	b	a	a
b	a	a	b	a	a	a	b	a	a
b	a	a	b	a	a	a	b	a	a
b	a	a	b	a	a	a	b	a	a
b	a	a	b	a	a	-	b	-	a
b	a	a	b	a	a	a	b	a	a
b	a	a	b	a	a	a	b	a	a
b	a	a	b	a	a	-	b	a	a
b	a	a	b	a	a	a	b	a	a
b	a	a	b	a	a	a	b	a	a
b	a	a	b	a	a	a	b	a	a
b	a	a	b	a	a	a	b	a	a
b	a	a	b	a	a	a	b	a	a
b	a	a	b	a	a	a	b	a	a
b	a	a	b	a	a	a	b	a	a
b	a	a	b	a	a	a	b	a	a
b	a	a	b	a	a	a	b	a	a
b	a	a	b	a	a	a	b	a	a
b	a	a	b	a	-	a	b	a	a
b	-	a	b	a	-	a	b	a	-
b	a	a	b	a	a	a	b	a	a
b	a	a	b	b	a	a	b	a	a
b	a	a	b	b	a	b	b	a	a
b	-	a	b	b	a	b	b	a	a
b	a	a	b	b	a	b	b	a	a
b	a	a	b	b	a	b	b	a	a
b	-	a	b	b	a	b	b	a	a
b	a	a	b	b	-	b	b	a	a
b	a	a	b	b	a	b	-	a	a
b	a	a	b	b	-	b	b	a	a
b	a	a	b	b	a	b	b	a	a
b	a	a	b	b	a	b	b	-	a
b	a	a	b	b	a	b	b	a	a
b	a	a	b	b	a	-	b	a	a
b	a	a	b	b	a	b	b	a	-
b	a	b	b	a	a	b	b	a	a
b	a	a	b	a	a	b	-	a	a
b	a	a	b	a	a	b	b	a	a
b	a	a	b	a	a	b	b	a	a
b	-	a	a	a	a	b	b	a	a
b	a	a	a	a	a	b	b	a	a
b	-	a	a	a	-	b	b	a	a
b	a	a	a	a	a	b	b	a	a
b	a	a	a	a	a	a	-	a	a
b	a	a	a	a	a	a	b	a	a
b	-	a	a	a	a	a	b	a	a
b	a	a	a	a	a	a	b	a	a

b	a	a	a	a	a	a	b	a	b
b	a	a	a	a	-	a	b	-	b
b	a	a	a	b	a	-	b	a	b
b	a	a	a	b	b	a	b	a	a
b	a	a	a	b	b	a	b	a	a
b	a	a	a	b	b	a	-	a	a
b	a	a	a	b	a	a	b	a	a
b	a	a	a	b	-	a	b	a	b
b	a	a	a	b	b	a	b	a	b
b	a	a	a	b	b	-	b	a	b
b	a	a	a	b	b	a	b	a	b
b	a	a	a	b	a	a	b	a	b
b	a	a	a	b	a	a	a	a	b
b	a	a	a	b	a	a	a	a	-
b	a	a	a	b	a	a	a	a	b
b	a	a	a	b	a	a	a	a	b
b	a	a	a	b	-	a	a	a	b
b	a	a	a	b	a	a	a	-	b
b	a	a	a	b	-	-	a	a	b
b	a	a	b	b	a	a	a	a	b
b	a	a	b	b	a	a	a	a	b
b	a	a	b	b	a	a	-	a	b
b	a	a	b	b	-	-	a	a	b
b	a	a	b	b	a	a	-	a	b
b	a	a	b	b	a	a	-	a	b
b	a	a	b	b	a	a	a	a	b
b	-	a	b	b	a	a	a	a	b
b	b	a	b	b	a	a	a	a	b
b	b	a	b	b	a	a	a	a	b
b	b	a	b	b	a	a	a	a	b
b	b	a	b	b	a	a	a	a	b
b	b	a	b	b	a	a	a	a	b
b	b	a	b	b	a	a	a	a	b
b	b	a	b	b	a	a	-	a	b
b	b	a	b	b	a	a	-	a	b
b	b	a	b	b	a	a	a	a	b
b	b	a	b	b	a	a	a	a	b
b	b	a	b	b	a	a	-	a	b
b	b	a	b	b	a	-	-	a	b
b	b	a	b	b	a	a	b	a	b
b	-	a	b	b	a	a	a	a	b
b	b	a	b	b	a	a	a	a	b
b	b	a	b	b	a	a	a	a	b
b	b	a	b	b	a	a	a	a	b
b	b	a	b	b	-	a	a	a	b
b	b	a	b	b	a	a	a	a	b
b	b	a	b	b	a	a	b	a	b

b	b	a	b	b	-	a	b	a	b
b	b	a	b	b	a	b	b	a	b
b	b	a	b	b	a	a	b	a	b
b	b	a	b	b	a	a	b	a	b
b	b	a	b	b	a	-	b	a	b
b	b	a	b	b	a	b	b	a	b
b	b	a	b	b	a	a	b	a	b
b	b	a	b	b	-	-	b	a	b
b	b	a	b	b	a	a	b	a	b
b	b	a	b	b	a	a	b	a	b
b	b	a	b	b	a	a	b	a	-
b	b	a	b	b	-	a	b	-	b
b	b	a	b	b	a	a	-	a	b
b	b	a	b	b	a	a	b	a	b
b	b	a	b	b	a	a	b	a	b
b	b	a	b	b	a	a	b	a	b
b	b	a	b	b	a	a	b	a	b
b	b	a	b	b	a	a	b	a	b
b	b	a	b	b	-	a	b	a	b
b	b	a	b	b	a	a	-	a	b
b	b	a	b	b	a	a	b	-	b
b	b	a	b	b	a	a	b	b	b
b	b	a	-	b	a	a	-	b	b
b	b	a	b	b	a	a	b	b	b
b	b	a	b	b	a	a	b	b	b
b	b	a	b	b	a	a	b	b	b
b	b	a	b	b	a	a	b	b	b
b	b	a	b	b	a	a	b	b	b
b	-	a	b	b	a	a	b	b	b
b	b	a	b	b	a	a	b	b	b
b	b	a	b	b	a	a	-	b	b
b	b	a	b	b	a	a	b	b	b
b	b	a	b	b	a	-	b	b	b
b	b	a	b	b	a	a	b	b	b
b	b	a	b	b	a	a	b	b	b
b	b	a	b	b	a	a	b	b	b
b	b	-	b	b	a	a	b	b	b
b	b	a	b	b	a	a	b	b	b
b	b	a	b	b	a	a	b	b	b
b	b	a	b	b	a	a	b	b	b
b	b	a	b	b	a	a	b	b	b
b	b	a	b	b	a	a	b	b	b
b	b	a	b	b	a	a	b	b	b
b	b	b	b	b	a	a	b	b	b
b	b	a	b	b	a	a	b	b	b
b	b	a	b	b	a	a	b	b	b
b	b	a	b	b	a	a	b	b	b

b	b	a	b	-	a	a	b	b	b
b	b	a	b	b	a	a	b	b	b
b	b	a	b	b	b	a	b	b	b
a	b	a	b	a	b	a	b	b	b
a	b	a	b	a	b	a	b	b	b
a	a	a	b	a	b	a	b	b	b
a	a	a	b	a	b	a	b	b	b
a	a	a	b	a	b	a	b	-	b
a	a	a	b	a	b	a	b	b	b
a	a	a	b	a	b	a	b	b	b
a	a	a	b	a	b	a	-	b	b
a	a	a	b	a	b	a	b	b	b
a	a	a	b	a	b	-	b	b	b
a	a	a	b	a	b	a	b	b	-
a	a	a	b	a	b	a	b	b	b
a	a	a	b	a	b	a	-	b	-
a	a	a	b	a	b	a	b	b	b
a	a	a	b	a	b	b	b	b	b
a	a	a	b	a	b	b	b	b	b
a	a	a	b	a	-	b	b	b	b
a	a	a	b	a	b	b	b	b	-
a	a	a	b	a	b	b	b	b	a
a	-	a	b	a	b	b	b	b	a
a	a	a	b	a	b	b	b	b	a
a	a	a	b	a	-	b	b	b	a
a	a	a	b	a	b	b	b	b	a
a	a	a	b	a	b	b	b	b	a
a	a	a	-	a	b	b	b	b	a
a	a	a	b	a	b	b	b	b	a
a	a	a	b	a	b	b	b	b	a
a	a	a	b	a	b	b	b	b	a
b	-	a	b	a	b	b	b	b	a
b	a	a	b	a	b	b	b	b	a
b	a	a	b	a	b	b	b	b	a
b	a	a	b	a	b	b	b	b	a
b	a	a	b	a	a	b	b	b	a
b	a	a	b	a	a	b	b	b	a
b	a	a	b	a	a	b	b	b	b
b	a	a	b	a	a	b	b	b	b
b	a	a	b	a	a	b	b	b	b
b	a	a	b	a	b	b	b	b	b
b	a	a	b	a	b	b	b	b	b
b	a	b	b	a	b	b	b	b	b
b	a	b	b	a	b	b	b	b	b
b	a	b	b	a	b	b	b	b	b
-	a	b	b	a	b	b	b	b	b
b	a	b	b	a	b	b	b	b	b

b	a	b	b	a	b	b	b	b	b
b	a	b	-	a	b	b	b	b	b
b	a	b	b	a	b	b	b	b	b
b	b	b	b	a	b	b	b	b	b
a	a	b	a	-	-	a	a	a	-
b	a	b	a	b	-	a	a	a	-
b	a	b	a	b	b	a	a	a	b
b	a	b	a	b	b	-	a	a	b
b	a	b	a	b	-	a	a	a	b
b	a	b	a	b	b	a	a	a	b
b	a	b	a	b	-	a	a	a	b
b	a	b	a	b	b	a	a	a	-
b	b	b	a	b	b	a	b	a	b
b	b	b	b	b	b	b	b	a	b
b	b	b	b	b	-	b	b	a	b
b	b	b	b	b	b	b	b	a	b
b	b	b	b	b	b	b	b	a	b
b	b	b	b	b	b	b	b	a	b
b	b	b	b	b	b	b	b	-	b
b	b	b	b	b	-	b	b	a	b
b	b	b	b	b	-	b	b	a	-
a	b	b	b	b	b	b	b	a	b
a	b	b	b	b	b	b	b	a	b
a	b	b	b	b	b	b	b	a	b
a	b	b	b	b	b	b	b	a	b
a	b	b	b	b	b	b	b	a	a
a	b	b	b	b	b	b	b	-	a
a	b	b	b	b	b	b	b	a	a
a	b	b	b	a	b	b	-	a	a
b	b	b	b	b	b	b	b	a	a
b	b	b	b	b	b	b	b	a	a
b	b	b	b	b	b	b	b	a	a
b	b	b	b	b	b	b	b	a	a
b	b	-	b	b	b	b	a	-	a
b	b	b	b	b	b	b	b	a	a
b	b	b	b	b	b	b	-	a	a
b	b	b	b	b	b	b	b	a	a
b	b	b	b	-	b	b	b	a	a
b	b	b	b	b	b	b	b	a	a
b	b	b	b	b	b	b	b	a	a
b	b	b	b	b	b	b	b	a	a
b	b	a	b	-	b	b	b	a	a
b	b	a	b	a	b	b	b	a	a
b	b	a	b	a	b	b	-	a	a
b	b	a	b	a	-	b	b	a	a
b	b	b	b	a	b	b	b	b	b
b	b	b	b	a	b	b	b	b	b

b	b	b	b	a	b	b	b	b	b
b	b	b	b	a	b	b	-	b	b
b	b	b	a	a	b	-	b	b	b
b	b	b	b	b	b	b	b	b	b
b	b	b	b	b	b	b	b	b	b
b	b	b	b	b	b	b	b	b	b
b	b	b	b	b	b	b	b	b	b
b	b	b	b	b	-	b	b	b	b
b	b	b	b	b	b	b	b	b	b
b	b	b	b	b	b	b	b	b	b
b	b	b	b	b	b	b	b	b	b
b	b	b	b	b	b	b	-	b	b
b	b	b	b	a	b	b	b	b	b
b	b	b	b	b	b	b	b	b	b
b	b	b	b	b	-	b	b	b	b
b	b	b	b	b	b	b	b	b	b
b	b	b	b	b	b	b	b	b	b
b	b	b	b	b	b	b	b	b	b
b	b	b	b	b	b	b	b	b	b
b	b	b	-	b	b	b	b	a	b
b	b	b	b	b	b	b	b	a	b
b	b	b	b	b	b	b	b	a	b
b	-	b	b	b	b	b	b	a	b
b	b	b	b	b	b	b	b	-	b
b	b	b	b	b	b	b	b	a	b
b	b	b	b	b	b	b	b	a	b
b	b	b	b	a	b	b	a	a	b
b	b	b	a	a	b	a	a	a	b
b	b	b	b	a	b	b	a	a	b
b	b	b	b	a	b	b	a	-	b
b	b	b	b	a	-	b	a	a	b
b	b	b	a	a	-	a	a	a	b
b	b	b	b	a	b	b	-	a	b
b	b	b	a	a	b	a	a	a	b
b	b	b	a	a	-	a	a	a	b
b	b	b	a	a	b	a	a	a	-
b	b	b	a	a	b	-	-	a	b
b	b	b	a	a	b	a	a	a	b
b	b	b	a	a	b	a	-	a	b
b	-	b	a	a	b	a	a	a	b
b	b	b	a	a	b	a	a	a	b
b	b	b	a	a	b	a	-	a	b
b	b	b	a	a	-	a	a	a	b
b	b	b	a	a	b	a	-	a	b
b	b	b	a	a	b	a	a	a	b
b	b	b	a	a	b	a	a	a	b
b	b	b	a	a	b	a	a	a	b

b	b	a	a	a	b	a	a	a	b
b	b	a	a	a	b	a	a	a	b
b	b	a	a	a	b	a	a	a	b
b	b	a	a	a	b	a	a	a	b
b	b	a	a	a	b	a	a	a	b
b	b	a	a	a	b	a	a	a	b
b	b	a	a	a	b	a	a	a	b
b	b	a	a	a	b	a	a	a	-
b	b	a	a	a	b	a	a	a	b
b	a	a	a	a	b	a	a	a	b
b	a	a	a	a	-	a	a	a	b
b	a	a	a	a	b	a	a	a	b
b	a	a	a	a	b	a	a	-	b
b	a	a	a	a	b	a	a	a	b
b	a	a	a	a	b	a	a	a	b
b	a	a	a	a	b	a	a	b	b
b	-	a	-	a	b	a	a	b	b
b	a	a	a	a	-	a	-	b	b
b	a	a	b	a	b	a	a	b	b
b	a	a	b	a	-	a	a	b	-
a	a	a	b	a	b	a	a	b	b
a	a	a	b	a	b	a	-	b	b
a	a	a	b	a	b	a	a	b	b
a	a	a	b	a	b	a	a	b	b
a	a	a	b	a	b	a	a	b	b
a	a	a	b	a	b	a	a	b	b
a	a	a	b	a	b	a	a	b	b
a	a	a	b	a	b	a	a	b	b
a	a	a	b	a	b	a	a	-	-
a	a	a	b	a	b	a	-	b	b
a	a	-	b	a	b	a	-	b	b
a	a	a	b	a	b	a	a	b	b
a	a	a	b	a	b	a	a	b	b
a	a	a	b	a	b	a	a	b	b
a	a	a	b	a	b	a	a	b	-
a	a	a	b	a	b	a	a	b	b
a	a	a	b	a	b	a	a	b	b
a	a	a	b	a	b	a	a	b	b
a	a	a	b	a	b	-	-	b	b
a	a	a	b	a	b	a	-	b	b
a	a	a	b	a	b	a	b	b	b
a	a	a	b	a	b	a	b	b	b
a	a	a	b	a	b	a	b	-	b
a	a	a	b	a	b	a	b	-	b
b	a	a	b	a	b	a	b	b	b
b	-	a	b	-	b	-	b	b	b
b	b	a	b	a	b	a	a	b	b

b	b	a	b	a	b	a	a	b	b
b	b	a	b	a	b	a	a	b	b
b	-	a	b	a	b	a	a	a	b
b	b	a	b	a	-	a	a	a	b
b	b	a	b	a	b	a	a	a	b
b	b	a	b	a	b	-	-	a	b
b	b	a	a	a	b	a	a	a	b
b	b	a	b	a	b	a	a	a	b
b	b	-	a	a	a	a	b	b	b
b	b	a	-	a	-	a	b	b	b
b	b	a	a	a	a	a	b	b	b
b	b	a	a	a	a	a	b	b	b
b	b	a	a	a	-	a	b	b	b
b	b	a	a	a	a	a	b	b	b
b	a	a	a	a	a	a	b	b	b
b	b	a	a	a	a	a	b	b	b
b	b	a	a	a	a	a	b	b	b
b	b	a	a	a	b	a	b	b	b
b	b	a	b	a	b	a	b	b	b
b	b	a	b	b	b	a	b	a	b
b	b	a	b	a	b	a	b	a	b
b	-	a	b	a	-	a	b	a	b
b	b	a	b	a	b	a	b	a	b
b	b	a	b	b	b	a	b	a	b
b	a	a	b	b	b	a	b	a	b
b	a	a	b	b	b	a	b	a	-
b	a	a	b	b	b	a	b	a	b
b	a	a	b	b	b	a	b	-	b
b	a	a	b	b	b	a	b	a	a
b	a	a	b	b	b	a	b	a	a
b	b	a	a	b	b	a	a	a	a
b	b	-	b	b	b	a	-	a	a
b	b	a	a	b	b	a	a	a	a
b	b	a	a	b	-	a	a	a	-
b	b	a	a	b	b	a	a	a	a
b	b	a	a	b	b	a	-	a	a
b	b	a	a	b	b	a	a	a	a
b	b	a	a	b	b	a	b	a	a
b	b	a	a	b	b	a	a	a	a
b	-	a	a	b	-	a	a	a	a
b	b	a	a	b	b	a	a	a	a
b	b	a	a	b	-	a	a	a	a
b	b	a	a	b	b	a	a	a	a
a	a	a	a	b	b	a	b	a	a
a	a	a	a	b	b	a	b	a	a
a	a	a	a	b	b	a	b	a	a
a	a	a	-	b	b	-	b	a	a

a	b	a	a	a	b	a	b	a	a
a	a	a	a	b	b	a	b	a	a
a	a	a	a	b	b	a	b	a	a
a	a	a	a	b	b	a	b	a	a
a	a	a	a	b	b	a	b	a	a
a	a	a	a	b	b	a	b	a	a
b	a	a	a	b	b	a	b	a	a
b	a	a	a	b	b	a	b	b	a
b	a	a	a	b	b	a	b	b	a
b	a	a	a	b	b	a	b	b	a
b	a	a	a	b	b	a	b	b	a
b	a	a	-	b	b	a	b	b	a
b	a	a	a	b	a	b	b	a	b
b	a	a	a	b	a	b	b	a	b
b	a	a	a	b	a	b	b	a	b
b	a	a	a	b	a	b	b	a	b
b	a	a	a	b	a	b	b	a	b
b	a	a	a	b	a	b	b	a	b
b	a	a	a	b	a	b	b	a	b
b	a	-	a	b	a	b	b	a	b
b	a	a	a	b	a	-	b	a	b
b	a	a	a	b	a	b	b	a	b
b	a	a	a	b	a	b	b	-	b
b	a	a	a	b	b	b	b	a	b
b	a	a	a	b	b	b	b	a	b
b	a	a	a	b	b	b	b	a	b
b	a	a	a	b	b	b	b	a	b
b	b	b	a	b	b	b	b	a	a
b	b	b	a	b	b	b	b	a	-
b	b	b	a	b	b	b	b	a	b
b	b	-	a	b	b	b	-	a	b
b	b	b	a	b	-	b	-	a	b
b	b	b	a	b	b	b	b	a	b
b	b	b	a	b	b	b	b	a	b
b	b	b	a	b	b	b	b	a	b
b	b	b	a	b	b	b	b	a	b
b	b	b	a	b	b	b	b	a	b
b	b	b	a	b	b	-	-	a	b
b	b	b	a	b	b	b	b	a	b
b	-	b	a	b	b	a	b	a	-
b	b	b	b	b	b	a	b	a	b
b	b	b	b	b	b	a	b	a	b
b	b	b	b	b	b	a	b	a	b
b	b	b	b	b	b	a	b	a	b
b	b	b	b	b	b	a	b	a	b
b	b	b	b	b	b	a	b	a	b
b	b	b	b	b	b	a	b	a	a

b	b	b	b	b	-	a	b	a	a
b	b	b	b	a	b	a	b	a	a
b	b	b	b	a	a	a	b	a	a
b	b	b	b	a	a	a	b	a	a
b	b	b	b	a	a	a	a	a	a
a	b	b	b	a	a	a	-	a	a
a	b	b	b	a	a	a	a	a	b
a	b	b	b	a	a	a	-	a	b
b	a	a	a	a	a	a	a	a	a
b	a	a	a	a	-	a	a	a	a
b	a	a	a	a	a	a	a	a	b
b	a	a	a	a	a	a	a	a	-
b	a	a	-	a	a	a	a	a	b
b	a	a	a	a	a	a	a	a	b
b	a	a	a	a	a	a	a	a	b
b	b	a	a	a	a	-	a	a	b
b	b	a	a	a	a	a	a	a	b
-	b	a	a	a	a	a	a	a	b
b	b	a	a	a	a	a	a	a	b
b	b	a	a	a	a	a	a	a	b
b	b	a	a	a	a	a	a	a	b
b	b	a	a	a	a	a	a	a	b
b	b	a	a	a	a	a	a	a	b
b	b	a	a	a	a	a	a	a	b
b	b	a	a	a	a	a	a	a	a
b	b	a	-	a	a	a	a	a	a
b	b	a	a	a	a	a	a	a	a
b	b	a	a	a	a	a	a	a	a
b	b	a	a	a	a	a	a	b	a
b	b	a	a	a	-	a	a	b	-
b	b	a	a	a	-	-	a	b	a
b	b	a	a	a	a	a	a	b	a
b	b	a	a	a	a	a	a	b	a
b	b	-	a	a	a	a	a	b	a
b	b	a	a	a	a	a	a	b	a
b	b	a	a	a	a	a	a	b	a
b	b	a	a	a	a	-	a	b	a
b	b	a	a	a	a	a	a	-	a
b	b	a	a	a	a	a	a	b	a
b	b	a	a	a	a	a	a	b	a
-	b	a	a	a	a	a	a	b	a
b	b	a	a	a	a	a	a	b	a
b	b	a	-	a	a	a	a	b	a
b	b	a	a	a	a	a	a	b	a
b	b	a	a	a	a	a	a	b	a
b	a	a	a	a	a	a	a	-	a
b	a	a	a	a	a	a	a	-	a

b	a	a	a	a	a	a	a	b	a
b	a	a	a	a	a	a	a	b	a
b	a	a	a	a	a	a	a	b	a
b	a	a	a	a	a	a	a	b	a
b	a	a	a	a	a	a	a	b	a
b	a	a	a	a	a	a	a	b	a
b	a	a	a	a	a	a	a	b	a
b	a	a	a	a	a	a	a	-	a
b	a	a	a	a	a	a	a	b	a
b	a	a	a	a	a	a	a	-	a
b	a	a	a	a	a	-	a	b	a
b	a	a	a	a	a	a	a	b	a
a	a	a	a	a	a	a	a	b	a
a	a	a	a	a	a	a	a	b	a
a	a	a	a	a	a	a	a	b	a
a	a	a	a	a	a	a	a	b	a
a	a	a	a	a	a	a	a	b	a
a	a	a	a	a	a	a	a	b	a
b	a	a	a	a	a	-	a	b	a
b	a	a	-	a	a	a	a	b	a
a	a	a	b	b	-	a	b	a	a
a	a	a	b	b	a	a	b	a	a
a	-	a	b	b	a	a	b	a	a
a	a	a	b	b	a	a	b	a	a
a	a	a	b	b	a	a	b	a	a
a	a	a	b	b	a	a	b	a	a
a	a	a	b	b	a	a	b	a	a
a	a	a	b	b	a	a	b	a	a
a	a	a	b	b	a	a	b	a	a
a	a	a	b	b	a	a	b	a	a
a	a	a	b	b	a	a	b	a	a
a	a	a	b	b	a	a	b	a	a
a	a	a	b	b	-	a	b	a	a
a	a	a	b	b	a	a	b	a	a
a	a	a	b	b	-	a	b	a	a
a	a	a	b	b	a	a	b	a	a
a	a	a	b	b	-	a	b	a	a
a	a	a	b	b	a	a	b	a	a
a	a	a	b	b	a	a	b	a	a
a	a	a	b	b	a	a	b	a	a
a	a	a	b	b	a	a	b	a	a
a	a	a	b	b	a	a	b	a	a
a	a	a	b	b	a	a	b	a	a
a	a	a	b	a	a	a	a	a	a
a	a	a	b	a	a	a	b	a	a
a	a	a	b	a	a	a	b	a	a
a	a	a	b	a	a	a	b	a	a

a	a	a	b	a	a	a	b	-	a
a	a	a	b	a	a	a	b	a	a
a	a	a	b	a	a	a	b	a	a
a	a	a	b	a	a	a	b	a	a
a	a	a	a	a	a	a	b	a	a
a	a	a	a	a	a	a	b	a	a
a	a	a	a	a	a	a	b	a	a
a	a	a	a	a	-	a	b	a	a
a	a	a	a	a	a	a	b	a	a
a	a	a	a	a	-	a	a	a	a
a	b	b	a	b	a	-	a	a	a
a	a	b	a	a	a	a	a	a	a
a	a	b	a	a	a	a	a	a	a
-	a	b	a	a	a	a	a	a	a
a	a	b	a	a	a	a	a	a	a
a	a	b	a	a	a	a	a	b	a
a	a	b	a	a	a	a	a	b	a
a	a	b	a	a	a	a	a	b	a
a	a	b	a	a	a	a	-	b	a
a	a	b	a	b	a	b	a	b	a
a	b	b	a	b	a	b	a	b	a
a	b	b	a	b	a	b	a	b	a
a	b	b	a	b	a	-	a	b	a
a	b	b	a	b	a	b	a	b	a
a	-	b	a	b	a	b	a	b	a
a	b	b	a	b	a	a	a	b	a
a	b	b	a	b	a	a	a	a	a
a	b	b	a	b	a	a	a	b	a
a	b	b	a	b	a	a	a	a	a
a	-	b	a	b	a	a	a	a	a
a	b	b	a	b	a	-	a	a	-
a	b	b	a	b	-	-	-	a	a
a	b	b	a	b	a	a	a	a	a
a	b	b	a	b	a	a	a	a	a
a	b	b	a	b	a	a	a	a	a
a	a	a	a	a	b	a	a	b	a
a	a	a	a	a	b	a	-	b	-
a	a	a	a	a	b	a	a	b	a
a	a	a	a	a	b	a	a	-	a
a	a	a	b	a	b	a	a	b	-
a	a	a	b	a	b	a	a	b	a
a	a	a	b	a	b	a	a	b	a
a	a	a	b	a	b	a	a	b	a
a	a	a	b	a	b	a	a	b	a
a	a	a	b	a	b	a	a	b	a
a	a	a	b	a	b	a	a	b	a
a	a	a	b	a	b	a	a	b	a

a	a	a	b	a	b	a	a	b	a
a	a	a	b	a	b	a	a	b	a
a	a	a	b	a	b	a	a	b	a
a	a	a	b	a	b	a	a	b	a
a	a	a	b	a	b	a	a	b	a
a	a	a	b	a	b	a	a	b	a
a	a	a	b	a	b	a	a	b	a
a	a	a	b	a	b	a	a	b	a
a	a	a	b	a	b	a	a	b	-
a	a	a	b	a	-	a	a	b	a
a	-	a	b	a	b	a	a	b	-
a	a	a	b	a	b	a	-	b	-
a	a	a	b	a	b	-	a	b	a
a	a	a	b	a	-	a	a	b	a
a	a	a	b	a	b	a	a	b	a
a	a	a	b	a	b	a	a	b	a
b	-	a	b	a	b	b	a	b	a
b	a	a	b	a	b	b	a	b	a
b	a	a	b	a	b	b	a	b	-
a	a	a	b	a	b	b	a	b	a
a	a	a	b	a	b	b	a	b	a
b	a	a	b	b	b	b	a	b	a
b	a	a	b	b	-	b	a	b	a
b	a	a	b	b	b	b	a	b	a
b	a	a	b	b	b	b	a	b	a
b	a	a	b	b	b	b	a	b	a
b	a	a	b	b	b	b	-	b	a
b	a	a	b	b	b	b	a	b	a
b	a	b	b	b	b	b	a	b	a
b	a	b	b	b	b	b	a	b	a
b	a	b	b	b	b	b	a	b	a
b	a	b	b	b	-	b	a	b	a
b	a	b	b	b	b	b	a	b	a
b	a	b	b	b	b	b	a	b	a
b	a	b	b	b	b	b	a	b	a
b	a	b	b	b	b	b	a	b	a
b	a	b	b	b	b	b	a	b	a
b	a	b	b	b	b	b	a	b	a
b	a	b	b	b	b	b	a	b	a
b	a	b	b	b	a	b	a	b	a
b	a	b	b	b	a	b	a	b	a
b	a	b	b	b	a	b	a	b	a
b	a	b	b	b	-	b	a	b	a
b	a	b	b	b	a	b	a	b	a
b	a	b	b	b	a	b	a	b	a
b	a	b	b	b	a	b	a	b	a
b	b	a	b	b	a	a	b	b	b

b	b	a	b	b	a	a	b	b	b
b	b	b	b	b	a	a	b	b	b
b	b	b	b	b	a	a	b	b	b
b	b	b	b	b	a	a	b	b	b
b	b	b	b	b	a	a	b	b	b
b	b	b	b	b	a	a	b	b	b
b	b	b	a	b	a	a	b	b	b
b	b	b	a	b	b	a	b	b	b
b	b	b	a	b	b	a	b	b	b
b	b	b	a	b	b	a	b	b	b
b	b	b	a	b	b	a	b	b	b
b	b	b	a	b	b	a	b	b	b
b	b	b	a	b	b	a	-	b	b
b	b	b	a	b	b	a	a	b	b
b	b	b	a	b	b	a	a	b	b
b	b	b	a	b	b	a	a	b	b
b	b	b	a	b	b	a	a	b	b
b	b	b	a	b	b	a	b	b	b
b	b	b	a	b	b	a	a	b	b
b	b	b	a	-	b	a	a	b	b
b	b	b	a	a	b	a	a	b	b
b	b	b	a	a	b	a	a	b	b
b	b	b	a	a	b	a	a	b	b
b	b	b	a	a	b	a	a	b	b
b	b	b	a	a	b	a	a	b	b
b	b	b	a	a	b	a	a	b	b
a	b	b	a	a	b	a	a	b	b
a	b	b	a	a	b	a	a	b	b
a	b	b	a	a	b	a	a	b	b
a	b	b	b	a	b	a	a	b	b
a	b	b	b	a	b	a	a	b	b
a	b	b	b	a	b	a	a	b	b
a	b	b	b	a	b	a	a	b	b
a	b	b	b	a	b	a	a	b	b
a	b	b	b	a	b	a	a	b	b
a	b	b	b	a	b	a	a	b	b
a	b	b	b	a	b	a	a	b	b
a	b	b	b	a	b	a	a	b	b
a	b	b	b	b	b	a	a	b	b
a	b	b	b	a	b	a	a	b	b
a	a	a	a	b	a	b	b	a	a
b	a	b	a	a	a	a	a	a	b
b	a	b	a	a	a	a	a	a	b
b	a	b	a	a	a	a	a	a	b
b	a	b	a	a	a	a	a	a	b
b	a	b	a	a	a	a	a	a	b
b	a	b	a	a	a	a	a	a	b

b	a	b	a	a	a	a	a	a	b
b	a	b	a	a	a	a	a	a	b
b	a	b	a	a	a	a	a	b	b
b	a	b	a	b	a	a	a	b	b
b	a	b	a	b	a	a	a	b	b

YW161	YW162	YW163	YW164	YW165	YW166	YW167	YW168	YW169	YW170
-	b	a	a	a	a	a	a	b	a
b	b	a	a	a	a	a	a	b	b
b	b	a	a	a	a	a	a	b	a
b	b	a	a	a	a	a	a	b	a
b	b	a	a	a	a	a	a	b	a
b	b	a	a	a	a	a	a	b	a
b	b	a	a	a	a	a	a	b	a
b	b	a	a	a	a	a	a	b	a
b	b	a	a	a	-	a	a	b	a
b	b	a	a	a	a	a	a	b	a
b	b	a	a	a	a	a	a	b	a
b	b	a	a	a	a	a	a	b	a
b	b	a	a	a	a	a	a	b	a
b	b	a	a	a	a	a	a	b	a
b	b	a	a	a	b	a	a	b	a
b	b	a	a	a	a	a	a	b	a
b	b	a	a	a	a	a	a	b	a
b	b	a	a	a	a	a	a	b	a
b	b	a	a	a	a	a	a	b	a
b	b	a	-	a	a	a	a	b	a
b	b	a	a	a	a	a	a	b	a
b	b	a	a	a	a	a	a	b	a
b	b	a	a	b	a	a	a	b	a
b	b	-	a	b	a	a	a	b	a
b	b	a	a	b	a	a	a	b	a
b	-	a	a	b	a	a	a	-	a
b	b	a	a	b	a	a	a	b	a
b	b	a	a	b	a	a	a	b	a
b	b	a	a	a	a	a	a	b	a
b	b	a	a	a	-	a	a	b	a
b	b	a	a	a	a	a	a	-	a
b	b	a	a	a	a	a	a	b	a
b	b	a	a	a	a	a	a	b	a
b	b	a	a	a	a	a	a	-	a
b	b	a	a	-	a	a	a	b	a
b	b	a	a	a	a	a	-	b	a
b	b	a	a	a	a	a	a	b	a
b	b	a	a	a	a	a	a	b	a
b	b	a	a	a	a	a	a	b	a
b	b	a	a	a	a	a	-	b	a
b	b	a	a	a	a	a	a	b	a
b	b	a	a	a	a	a	a	b	a
b	b	a	a	a	a	a	a	b	a
b	b	a	a	a	a	-	-	b	a
b	b	a	a	a	a	a	a	b	a

b	b	a	a	a	a	a	a	b	a
b	b	a	a	a	a	a	a	b	a
-	b	a	a	a	a	a	a	b	a
b	b	a	a	a	-	a	a	b	a
b	b	a	a	a	a	a	a	-	a
b	b	a	a	a	a	a	a	b	a
b	b	a	a	a	a	a	a	b	a
b	b	a	a	a	-	a	a	b	a
b	b	a	a	a	a	a	a	b	a
b	b	a	a	a	a	a	a	-	a
b	b	a	a	a	a	a	a	b	a
b	b	a	a	a	a	a	b	b	a
b	b	a	a	a	a	a	b	b	a
b	b	a	a	a	a	a	b	b	a
b	b	a	a	a	a	a	b	b	a
b	b	a	a	a	-	a	b	b	a
b	b	a	a	a	a	b	-	b	a
b	b	a	a	a	a	b	b	b	a
b	b	a	a	a	a	b	b	b	a
b	b	a	a	a	a	b	b	b	a
b	b	a	a	a	a	b	b	b	a
-	b	a	a	a	a	b	b	-	a
b	b	a	a	a	a	b	b	b	a
b	b	a	a	a	a	b	b	b	a
b	b	a	a	a	a	b	b	b	a
b	b	a	a	a	a	b	b	-	a
b	b	a	a	a	a	b	b	b	a
b	b	a	a	a	a	b	b	b	a
a	b	a	a	-	a	b	b	b	-
a	b	a	a	a	a	b	b	b	a
a	b	a	a	b	a	b	b	b	a
a	b	a	a	b	a	b	b	b	a
a	b	a	a	b	a	b	b	b	a
a	b	a	a	b	a	b	b	-	a
a	b	a	a	b	a	b	b	b	a
a	b	a	a	b	a	b	b	b	a
a	b	a	a	b	a	b	b	b	-
a	b	a	a	b	a	b	b	b	a
a	b	a	a	b	a	b	b	b	a
b	a	a	a	a	a	a	a	a	a
b	a	a	a	a	a	a	a	a	a
b	a	a	a	a	a	a	a	a	a
b	a	a	a	a	a	a	a	a	a
b	a	a	a	a	-	a	-	a	-
b	a	a	a	a	a	a	a	a	a
b	a	a	a	a	-	a	-	a	a
b	a	a	a	a	a	a	a	a	a

b	a	a	a	a	a	a	a	-	a
b	a	a	a	a	a	a	a	a	a
b	a	a	a	a	a	a	a	a	a
b	a	a	a	a	a	a	a	a	a
b	a	a	a	a	a	a	a	a	a
b	a	a	a	a	a	a	a	a	a
b	a	a	a	a	a	a	a	a	a
b	a	a	a	a	-	a	a	a	a
b	a	a	a	a	a	a	a	a	a
b	a	a	a	a	a	a	a	a	a
b	a	a	a	a	a	a	a	a	a
b	a	a	a	a	a	a	a	a	a
b	a	a	a	a	a	a	a	b	a
b	a	a	a	b	a	a	a	b	a
b	a	a	a	b	a	a	a	b	a
b	a	a	a	b	a	a	-	b	-
b	a	a	a	b	a	a	a	b	a
b	a	a	a	b	a	a	a	b	a
b	a	a	a	b	a	a	a	b	-
b	a	a	b	b	a	a	a	b	a
b	a	a	b	b	a	a	a	b	a
b	a	a	b	b	a	a	a	b	a
b	a	a	b	b	a	a	a	b	b
b	a	a	b	b	a	a	a	b	b
b	a	a	b	b	a	a	a	b	b
b	a	a	b	b	a	a	a	b	b
b	a	a	b	b	a	a	-	b	b
b	a	a	b	b	a	a	a	b	b
b	a	b	b	b	a	a	-	b	b
b	a	b	b	b	a	a	a	b	b
b	a	b	b	b	a	a	a	b	b
b	b	b	b	b	a	a	a	b	-
b	b	b	b	b	a	a	a	-	-
b	b	b	b	b	a	a	a	-	-
b	b	b	b	b	a	a	a	b	b
b	b	b	b	b	b	a	a	b	b
b	b	b	b	b	-	a	a	b	b
b	b	b	b	b	a	a	-	b	b
b	b	b	b	b	-	a	a	b	b
b	b	b	b	b	a	a	a	b	b
b	b	b	b	b	a	a	a	b	b
b	b	b	b	b	a	a	a	b	-
b	b	b	b	b	-	a	a	b	b
b	b	b	b	b	a	a	a	b	b
b	b	b	b	b	a	a	a	b	b
b	b	b	b	b	a	a	a	b	b
b	b	b	b	b	a	a	a	b	b

b	b	b	b	b	-	a	a	b	b
b	b	b	b	b	a	a	a	b	b
b	b	b	b	b	a	a	a	b	b
b	b	b	b	b	a	a	a	b	b
b	b	b	b	b	a	a	a	b	b
b	b	b	b	b	a	a	-	b	b
b	b	b	-	b	a	a	a	b	b
b	b	b	b	b	a	a	a	b	b
b	b	b	b	b	a	a	a	b	b
b	b	b	b	b	a	a	a	b	b
b	b	b	b	b	a	a	a	b	b
b	b	b	b	b	a	a	a	b	b
b	b	b	b	b	a	a	a	a	b
b	b	b	b	b	a	a	a	a	b
b	b	b	b	b	a	a	a	a	-
b	b	b	b	b	a	a	a	a	b
b	b	b	b	b	a	a	a	a	b
b	b	b	b	b	a	a	a	a	-
b	b	b	b	b	a	a	a	b	b
b	b	b	b	b	-	a	a	b	b
b	b	b	b	b	a	a	a	b	b
b	b	b	b	b	a	a	a	b	b
b	b	b	b	b	a	a	a	b	b
b	b	b	b	b	-	a	a	b	a
a	b	b	b	b	a	a	a	b	a
a	b	b	b	b	a	a	a	b	a
a	b	a	b	a	a	a	a	b	a
a	b	a	b	a	a	a	a	b	a
a	b	a	b	a	a	a	a	b	a
a	b	a	b	a	a	a	a	b	a
a	b	a	b	a	a	a	a	b	b
a	b	a	b	a	a	a	a	b	b
a	b	a	b	a	a	a	a	b	-
a	b	a	b	a	a	a	a	b	b
a	b	a	b	a	a	a	a	b	b
a	b	a	b	a	a	a	a	b	b
a	b	a	b	a	a	a	a	b	b
a	b	a	b	a	-	a	-	b	b
a	b	a	b	a	a	a	b	b	b
a	b	a	b	a	a	a	b	b	b
a	b	a	b	a	a	a	b	b	b
a	b	a	b	a	a	a	b	b	a
a	-	a	b	a	a	a	b	b	b
a	b	a	b	a	a	a	b	b	b
a	b	a	b	a	a	a	-	-	b
a	b	a	a	a	a	a	b	b	-
a	b	a	a	a	a	b	b	b	b

a	b	a	a	a	a	b	b	b	a
a	b	a	a	a	a	b	b	b	b
a	b	a	a	a	a	b	b	b	b
a	b	a	a	a	b	b	b	b	b
a	b	a	a	a	-	b	b	b	b
a	b	a	a	a	a	b	b	b	b
a	b	a	a	a	a	b	b	b	b
a	b	a	a	a	a	b	b	b	-
a	b	a	a	a	a	b	b	b	-
a	b	a	a	a	-	b	b	b	b
a	b	a	a	a	a	b	b	b	-
a	b	a	a	-	b	b	b	b	b
b	a	a	a	a	-	a	a	a	b
b	a	a	a	a	a	a	a	a	b
b	a	a	a	a	a	a	-	a	b
b	a	a	a	a	-	a	a	a	b
b	a	a	a	a	a	b	a	a	b
b	a	a	a	a	a	b	a	b	-
b	a	a	a	a	a	b	a	b	b
b	a	a	a	a	a	b	a	b	b
b	a	a	a	a	a	b	a	b	b
b	a	a	a	a	-	b	a	b	b
b	a	a	a	a	a	b	a	b	b
b	a	a	a	a	a	b	a	b	b
b	a	a	a	a	a	b	-	b	b
b	a	a	a	a	a	b	a	b	b
a	a	a	b	a	a	b	a	b	a
a	a	a	a	a	a	b	a	b	b
a	a	a	b	a	a	b	a	b	b
a	a	a	b	a	b	b	-	b	b
a	a	a	b	a	b	b	a	b	b
a	a	a	b	b	b	b	a	b	b
a	a	a	b	b	b	b	a	b	b
a	a	a	b	b	b	b	a	b	b
a	a	a	b	b	a	b	a	b	-
a	a	a	b	b	a	b	a	b	-
a	a	a	b	b	a	b	a	b	b
a	a	a	b	b	a	b	a	b	b
a	a	a	b	b	-	b	a	b	b
a	a	a	b	b	a	b	a	b	b
-	-	a	b	b	a	b	-	b	b
a	a	a	b	b	a	b	a	b	b
a	a	a	b	b	a	b	a	b	b
a	a	a	b	b	a	b	a	b	b
a	a	a	b	b	a	b	a	b	b
a	a	a	b	b	a	b	a	b	b
a	a	a	a	b	a	b	a	b	b

a	a	a	a	b	a	b	a	b	b
a	a	a	a	b	a	b	a	b	b
a	a	a	a	b	a	b	a	-	b
a	a	a	a	b	a	b	a	b	a
a	a	a	a	b	a	b	a	b	a
a	a	a	a	b	a	b	a	b	a
a	a	a	a	b	a	b	a	b	a
a	a	b	a	b	-	b	-	b	a
a	a	b	a	b	-	b	a	b	a
a	a	a	a	b	a	b	a	b	a
a	a	a	a	b	a	b	a	b	a
a	a	a	a	b	a	b	a	b	a
a	a	a	a	b	a	b	a	-	a
a	a	a	a	b	a	b	a	b	a
a	a	b	a	b	a	b	a	b	a
a	a	b	a	b	a	b	a	b	a
a	a	b	a	b	a	b	a	b	a
a	a	b	a	b	-	b	a	b	a
a	a	b	a	b	a	b	a	b	a
a	a	b	a	b	a	b	a	b	a
a	a	b	a	b	-	b	a	b	a
a	a	b	a	b	a	b	a	b	a
a	a	b	a	b	a	b	a	b	a
a	a	b	b	b	a	b	-	b	a
a	a	b	b	b	-	b	a	-	a
a	a	b	b	b	-	b	a	b	a
a	a	b	b	b	b	b	a	b	a
a	b	b	b	b	b	b	a	b	a
a	b	b	b	-	-	b	a	b	a
a	b	b	b	b	b	b	a	b	a
a	b	b	b	b	b	b	a	b	a
a	b	b	b	b	b	b	a	b	a
a	b	b	b	b	b	b	a	b	a
a	b	b	b	b	b	b	a	b	a
a	b	b	b	b	b	b	a	b	a
a	b	b	b	b	-	b	b	b	a
a	b	b	b	b	-	a	b	-	a
a	b	b	b	b	b	a	b	b	a
a	b	b	b	b	b	a	b	b	a
a	b	b	b	b	b	a	b	b	a
a	b	b	b	b	-	a	b	b	a
a	b	b	b	b	b	a	b	b	a
a	b	b	b	b	b	a	b	b	a
a	b	b	b	b	b	a	b	b	a
a	b	b	b	b	-	a	b	b	a
a	b	b	b	b	-	a	b	b	a
a	b	b	b	b	-	a	b	b	a

a	b	b	b	b	b	a	b	b	a
a	b	b	b	b	b	a	b	b	a
a	b	b	b	b	a	b	a	b	a
a	b	b	b	a	a	a	b	b	a
a	b	b	b	a	b	a	b	b	a
a	b	b	b	a	-	a	b	b	a
a	b	b	b	a	b	a	b	b	a
-	b	b	b	a	b	a	b	b	a
a	b	b	b	a	b	a	b	b	b
a	b	b	b	a	b	a	b	b	b
a	b	b	b	a	b	a	b	b	b
a	b	b	b	a	b	a	b	-	b
a	b	b	b	a	b	a	b	b	b
a	b	b	b	a	-	a	-	b	b
a	b	b	b	a	b	a	b	b	b
a	b	b	b	a	b	a	b	b	b
a	b	b	b	a	b	a	b	b	b
a	b	b	b	a	-	a	b	b	b
a	b	b	b	a	b	a	b	b	b
a	b	b	b	a	b	a	b	b	b
a	b	b	b	a	b	a	b	b	b
-	b	b	b	a	b	a	b	b	b
a	b	b	b	a	b	a	b	b	b
a	b	b	b	-	b	a	b	b	b
a	b	b	b	a	b	a	b	b	b
a	-	b	b	a	b	a	b	b	b
a	b	b	b	a	b	a	b	b	b
a	b	b	b	a	b	a	b	b	b
a	b	b	b	a	b	a	b	b	b
a	b	b	b	a	b	a	b	b	b
a	b	b	b	a	b	a	b	b	b
a	b	b	b	a	b	a	b	b	a
a	b	b	b	a	-	a	b	b	a
a	b	b	b	a	b	a	-	b	b
a	b	b	b	a	-	a	b	b	b
a	b	b	b	a	-	a	b	b	b
a	b	b	b	a	b	a	b	b	b
-	b	b	b	a	b	a	b	b	b
a	a	b	a	b	a	a	a	a	a
a	a	b	a	b	a	a	a	a	a
a	a	b	a	b	a	a	a	a	a
a	a	b	a	b	a	a	a	a	a
a	a	b	a	b	a	a	a	a	a
a	a	b	a	b	-	a	-	a	-
a	a	b	a	b	a	a	a	a	a
a	a	b	a	b	-	a	-	a	a
a	a	b	a	b	a	a	a	a	a

a	a	b	a	b	a	a	a	a	-
a	a	b	a	b	a	a	a	a	a
a	a	b	a	b	a	a	a	a	a
a	a	b	a	b	a	a	-	a	a
a	a	b	a	b	a	a	a	a	a
a	a	b	a	b	-	a	a	a	a
a	a	b	a	b	a	a	a	a	b
a	a	b	a	b	a	a	a	a	b
a	a	b	a	b	a	a	a	a	b
a	a	b	a	b	a	a	a	a	b
a	a	b	a	b	a	a	a	b	b
a	a	b	a	b	a	a	a	b	b
a	a	b	a	b	a	a	a	b	b
a	a	b	a	b	a	a	a	b	b
-	a	b	a	b	a	a	-	b	b
a	a	b	a	b	a	a	a	-	b
a	a	b	a	b	a	a	a	b	b
a	a	b	a	b	a	a	a	-	b
-	a	b	a	b	a	a	a	b	b
a	a	b	a	b	a	b	a	-	b
a	a	b	a	b	a	b	a	b	b
a	a	b	a	b	a	b	a	b	b
a	a	b	a	b	a	b	a	b	b
a	a	b	a	b	a	b	a	b	b
a	b	b	a	b	a	b	a	-	b
a	b	b	a	b	a	b	a	b	b
a	b	b	a	b	a	b	a	b	b
a	b	b	a	b	a	b	a	b	b
a	b	b	a	b	a	b	-	b	b
a	b	b	a	b	a	b	a	b	b
a	b	b	a	b	a	b	a	b	b
a	b	b	a	b	a	b	a	b	b
a	b	b	a	b	-	b	a	b	b
a	b	b	a	b	a	b	a	b	b
a	b	b	a	b	a	b	a	b	b
a	b	b	a	b	a	b	a	b	b
a	b	b	a	b	a	b	b	b	a
a	b	b	a	b	a	b	b	b	a
b	b	b	a	b	a	b	b	b	-
b	b	b	a	b	a	a	b	b	a
b	b	b	a	b	a	a	b	b	a
b	b	b	a	-	a	a	b	b	a
b	a	b	a	b	a	a	a	a	a
b	a	b	a	b	b	a	-	a	a
b	a	b	a	b	a	a	a	a	a
-	a	b	a	b	a	a	a	a	a
b	a	b	a	b	a	a	a	a	a

b	a	b	a	a	a	a	a	a	b
b	a	b	a	a	a	a	a	a	b
b	a	b	a	a	a	a	a	a	b
b	a	b	a	a	a	a	a	a	b
b	a	b	a	a	a	a	a	a	b
b	a	b	a	a	a	a	a	a	b
b	a	b	a	a	a	a	a	a	b
b	a	b	a	a	a	a	a	-	b
b	a	b	a	a	a	a	a	a	-
b	a	b	a	a	a	a	a	a	b
b	a	b	a	a	a	a	a	a	-
b	a	b	a	a	a	a	a	a	b
b	a	b	a	a	a	a	-	a	b
b	a	b	a	a	a	a	a	a	b
b	a	a	a	a	a	a	a	a	b
b	a	a	a	a	a	a	a	a	b
b	a	a	a	a	a	a	a	a	b
b	a	a	a	a	-	a	a	a	b
b	a	a	a	a	a	a	a	a	b
b	a	a	a	a	a	a	a	a	b
b	a	a	b	a	a	a	a	a	a
b	a	a	b	a	a	a	a	a	a
b	a	a	b	a	a	a	a	a	a
b	a	a	b	a	a	a	a	a	a
b	a	a	b	a	a	a	a	a	a
b	a	a	b	a	a	a	a	a	a
b	a	a	b	a	a	a	b	a	a
b	a	a	b	a	a	a	-	a	a
b	a	a	b	a	a	a	b	a	a
b	a	a	b	a	a	a	b	a	a
b	a	a	b	a	a	a	b	a	a
-	a	a	b	a	a	a	b	a	a
b	-	a	b	a	a	a	b	a	-
b	a	a	b	a	a	b	b	-	a
b	a	a	b	a	a	b	b	a	a
b	a	a	b	a	a	b	b	a	a
b	a	a	b	a	a	b	b	a	a
b	a	a	b	a	-	b	b	a	-
b	a	a	b	a	a	b	-	a	a
b	a	a	b	a	a	b	b	a	a
b	b	b	b	b	a	b	a	a	b
b	b	b	b	b	a	b	a	a	b
b	b	b	b	b	a	b	b	a	b
b	b	b	b	b	a	b	b	a	b
a	b	b	b	b	-	b	b	a	b
a	b	b	b	b	a	b	b	b	a
a	b	b	b	b	a	b	b	b	a

a	b	b	b	b	a	b	b	-	a
a	b	b	b	b	a	b	b	b	a
a	b	b	b	b	a	b	-	b	a
a	b	b	b	b	a	b	b	b	a
a	a	b	b	b	a	b	b	b	a
a	a	b	a	b	a	b	b	b	a
a	a	b	a	b	a	b	b	b	a
a	a	b	a	b	-	b	b	b	a
a	a	b	a	b	a	b	b	b	a
a	a	b	a	b	a	b	b	b	a
a	a	b	a	b	a	b	b	b	-
a	a	b	a	b	a	b	b	b	-
a	a	b	a	b	-	b	b	b	a
a	a	b	a	b	b	b	b	b	a
a	a	b	a	b	b	b	b	b	a
a	a	b	a	b	b	b	b	b	a
a	a	b	a	b	b	b	b	b	a
b	a	b	a	b	b	b	b	b	a
a	a	b	a	b	b	b	b	b	b
a	a	b	a	b	b	b	b	b	a
a	a	b	a	b	b	b	b	b	a
a	a	b	a	b	b	b	b	b	a
a	a	b	a	b	b	b	b	b	a
a	a	b	a	b	-	b	b	b	a
a	a	b	a	b	b	b	b	b	a
a	a	b	a	b	b	b	b	b	a
a	a	b	a	b	b	b	b	b	a
a	a	b	a	b	b	b	b	b	-
a	a	b	a	b	b	b	b	b	a
a	a	b	a	b	b	b	b	b	a
a	a	b	a	b	b	b	b	b	a
a	a	b	a	b	b	a	b	b	a
a	a	b	a	b	b	a	b	b	a
a	a	a	a	a	b	a	b	b	a
a	a	-	a	b	b	a	b	b	a
a	a	b	a	b	b	a	b	b	a
a	a	b	a	b	b	a	b	b	a
a	a	b	a	b	b	a	b	b	a
a	a	b	a	b	b	a	b	b	a
a	a	b	a	b	b	a	b	b	a
a	a	b	a	b	-	a	b	b	a
a	a	b	a	b	b	a	b	b	a
a	a	-	a	b	b	a	b	b	a
a	a	-	a	b	b	a	b	b	a
a	a	b	a	b	b	a	b	b	a
a	a	b	a	b	b	a	b	b	a
a	a	b	a	b	b	a	b	b	a

a	a	b	a	b	b	a	b	b	-
b	a	b	a	b	b	a	b	b	a
b	a	b	a	b	b	a	b	b	-
a	a	b	a	b	b	a	-	b	a
a	a	b	a	b	b	a	b	b	a
b	a	b	a	b	b	a	b	b	b
a	a	b	a	b	b	a	b	b	-
a	a	b	a	b	b	a	b	b	-
a	a	b	a	b	b	a	b	b	b
b	a	b	a	b	b	a	b	b	b
b	a	b	a	b	b	a	b	b	b
b	a	b	a	b	b	a	b	b	b
b	a	b	a	b	b	a	b	b	b
b	a	b	a	b	b	a	-	b	b
b	a	b	-	b	b	a	b	b	b
b	a	b	a	b	b	a	b	-	-
b	b	b	a	b	b	a	b	b	b
b	b	b	a	b	b	a	b	b	b
b	b	b	a	b	b	a	b	b	b
b	b	b	a	b	b	a	b	b	a
b	b	b	a	b	b	a	b	b	b
b	b	b	a	b	b	a	b	b	b
b	b	b	a	b	b	a	b	b	b
b	b	b	a	b	b	a	b	b	b
b	b	b	a	b	b	a	b	b	b
b	b	b	a	b	b	a	b	b	b
b	b	b	a	-	b	a	b	b	b
b	b	b	a	a	b	a	b	b	b
b	b	b	a	a	b	a	b	b	b
b	b	b	a	a	b	b	b	b	b
-	b	b	a	a	b	b	b	b	b
b	b	b	b	a	b	b	b	b	b
b	b	b	b	a	b	b	b	b	b
b	b	b	b	a	b	b	b	b	b
b	b	b	b	a	b	b	b	b	b
b	b	b	b	a	b	b	b	b	b
b	b	b	b	a	b	b	b	b	b
b	b	b	b	a	b	b	b	b	b
b	b	b	b	a	b	b	b	b	b
b	b	b	b	a	b	b	b	b	b
b	b	a	b	a	b	b	b	b	b
b	b	b	b	a	b	b	b	b	b
b	b	b	b	a	-	b	b	b	-
-	b	b	b	a	b	b	b	b	b
b	b	b	b	a	b	b	-	b	b
b	b	b	b	a	b	b	b	b	b
b	b	b	b	a	-	b	b	b	b

b	b	b	b	a	b	b	b	b	b
b	b	b	b	a	b	b	b	b	b
b	b	b	b	a	b	b	b	b	b
b	b	b	b	a	b	b	b	b	b
b	b	b	b	a	b	b	b	b	b
b	b	b	b	a	b	b	b	b	b
b	b	b	b	a	b	b	b	b	b
b	b	b	b	a	b	b	b	b	b
b	b	b	b	-	b	b	b	b	b
b	b	b	b	a	b	b	b	b	b
b	b	b	b	a	b	b	a	b	b
b	b	b	b	a	b	b	b	b	b
b	b	b	b	a	b	b	b	b	b
b	b	b	b	a	b	b	b	b	b
b	b	b	b	a	b	b	b	b	b
b	b	b	b	a	-	b	b	b	-
b	b	b	b	a	b	b	b	b	b
b	b	b	b	a	b	b	b	b	b
b	b	b	b	a	b	b	b	b	b
a	b	a	a	a	a	a	a	b	a
a	b	a	a	a	b	a	a	b	a
a	b	a	a	a	b	a	a	b	a
a	-	a	a	a	b	a	a	b	a
a	b	a	a	a	b	a	a	b	a
a	b	a	a	a	a	a	a	b	a
a	b	a	a	a	b	a	a	b	a
a	b	a	a	a	-	a	a	b	a
a	b	a	a	a	b	a	a	b	a
a	b	a	a	a	b	a	a	b	a
a	b	a	a	a	-	a	a	b	a
a	b	a	a	a	a	a	a	b	a
a	b	a	a	a	b	a	a	b	a
a	b	a	a	a	b	a	a	b	a
a	b	a	a	a	-	a	a	b	a
a	b	a	a	a	b	b	a	b	a
a	b	a	a	a	b	b	a	b	a
a	-	a	a	a	b	b	a	-	a
a	b	a	a	a	b	b	a	b	a
a	b	a	a	a	b	b	a	b	a
a	b	a	a	a	b	b	a	b	-
a	b	a	a	a	b	b	a	b	a
a	b	a	a	a	b	b	a	b	a
a	b	a	a	a	b	b	a	b	a
a	b	a	a	a	b	b	a	b	a
a	b	a	a	a	b	b	a	b	a
a	b	a	a	a	b	b	a	b	-
a	b	a	a	a	b	b	a	b	a

a	b	a	a	a	b	b	a	b	a
a	b	a	a	a	b	b	a	b	a
a	b	a	a	a	b	b	a	b	a
a	b	a	a	b	b	b	a	b	a
a	b	a	a	b	b	b	a	b	a
a	b	a	a	b	b	b	a	b	a
a	b	a	a	b	b	b	a	b	a
a	b	a	a	b	b	b	a	b	a
a	b	a	a	b	b	b	a	b	a
a	b	a	a	b	b	b	a	b	b
a	b	a	a	b	b	b	a	b	b
a	b	a	a	b	b	b	a	b	a
a	b	a	a	b	b	b	a	b	b
a	b	a	a	b	-	b	a	b	b
a	b	a	a	b	b	b	a	b	b
a	b	a	a	b	b	b	a	b	b
a	b	a	-	b	b	b	a	b	b
a	b	a	a	b	b	b	a	b	b
a	b	a	a	b	a	b	a	b	-
a	b	a	a	b	b	b	a	b	a
a	b	a	a	b	b	b	a	b	a
a	b	a	a	b	a	b	a	b	a
a	b	a	a	b	b	b	a	b	a
a	b	a	a	b	b	b	a	b	-
a	b	a	a	b	-	b	a	b	a
a	b	a	a	b	b	b	a	b	a
a	a	a	a	b	b	b	a	b	a
a	a	a	a	b	b	b	a	b	a
a	a	a	a	b	b	b	a	b	a
a	a	a	a	b	b	b	a	b	a
a	a	a	a	b	b	b	a	b	a
a	a	a	a	b	b	b	a	b	a
a	-	a	a	b	b	b	a	b	a
a	a	a	a	b	b	b	a	b	a
a	a	a	a	b	-	b	a	b	a
a	a	a	a	b	b	b	a	b	a
a	a	a	b	b	b	b	a	b	a
a	a	a	b	a	b	b	a	b	a
a	a	a	b	a	-	b	a	b	a
a	a	a	b	a	b	b	a	-	a
a	a	a	b	a	-	b	a	a	a
a	a	a	b	a	b	b	a	a	a
a	a	a	b	a	b	b	a	a	a
a	a	a	b	a	a	b	-	a	a
a	a	a	a	a	b	b	a	a	a
a	a	a	a	a	-	b	a	a	a
a	a	a	a	a	b	b	a	a	a

a	a	a	a	a	b	b	a	a	a
a	a	a	a	a	b	b	a	a	a
a	a	a	a	-	b	b	a	a	-
a	a	a	a	a	b	b	a	a	a
a	a	a	a	a	b	b	a	a	a
a	a	a	a	a	-	b	a	a	a
a	a	a	a	a	b	b	a	a	a
b	a	a	b	a	b	b	b	a	a
b	a	a	b	a	b	b	b	a	a
b	a	a	-	a	b	b	b	a	a
b	a	a	b	a	b	b	b	a	a
a	a	b	b	b	a	a	b	a	a
a	a	b	b	b	-	a	b	a	a
a	-	b	b	b	a	a	b	a	b
a	a	b	-	b	a	a	b	a	a
a	a	b	b	b	a	a	b	a	a
a	a	b	b	b	a	a	b	a	a
a	a	b	b	b	a	a	b	a	a
a	a	b	b	b	a	a	b	a	a
a	a	b	b	b	a	a	b	a	a
a	a	b	b	b	a	-	a	a	a
a	a	b	b	b	a	a	a	a	a
a	a	b	b	b	a	a	a	a	a
a	a	b	b	b	a	a	a	a	a
a	a	b	b	b	a	a	a	a	a
a	a	b	b	b	a	a	a	a	a
a	a	b	b	b	a	a	-	a	-
a	a	b	b	b	a	a	a	a	a
a	a	b	b	b	a	a	a	a	a
a	a	b	b	b	a	a	a	a	a
a	a	b	b	b	a	a	a	a	a
b	a	b	b	b	-	a	a	a	a
b	a	b	b	b	a	a	a	a	a
b	a	b	b	b	a	-	a	a	a
b	a	b	b	b	a	a	a	a	a
b	a	b	b	-	a	a	a	a	a
b	a	b	b	b	a	a	a	b	a
b	a	b	b	b	a	a	a	b	a
b	a	b	b	b	a	a	a	b	-
b	a	b	b	b	a	a	a	b	a
-	a	b	b	b	a	a	a	-	a
b	a	b	b	b	a	a	a	b	a
b	a	b	b	b	a	a	a	b	a
b	a	b	b	a	a	a	a	b	a
b	a	b	b	a	a	a	a	b	a
b	a	b	b	a	a	a	a	b	a
b	a	b	b	a	a	a	a	b	a

b	a	b	b	a	-	a	a	b	a
b	a	b	b	a	a	a	a	-	a
-	a	b	b	a	a	a	a	b	a
b	a	b	b	a	a	a	a	b	a
b	a	b	b	a	a	a	-	b	-
-	b	b	b	a	b	a	b	b	b
b	b	b	b	a	-	a	-	b	b
b	b	b	b	a	b	a	-	b	b
b	b	b	b	a	b	a	b	b	b
b	b	b	b	a	b	a	b	b	-
b	b	b	b	a	b	a	b	b	b
b	b	b	b	a	b	a	b	b	b
b	b	b	b	a	b	a	b	b	b
b	b	b	b	a	b	a	b	b	b
b	b	a	b	a	b	a	b	b	b
b	b	a	b	a	b	a	b	b	b
-	b	a	b	a	-	a	-	b	b
b	b	a	b	a	b	a	b	b	b
b	b	a	b	a	b	a	b	-	b
b	b	a	b	a	b	a	b	b	b
b	b	a	b	a	b	a	b	b	b
b	b	a	b	a	-	a	b	b	b
b	b	a	b	a	b	a	b	b	b
b	a	a	b	a	b	a	b	b	b
b	b	a	b	a	b	a	b	b	b
b	b	a	b	a	b	a	b	b	b
b	b	a	b	a	b	a	b	b	-
b	b	a	b	a	b	a	-	b	-
b	b	a	b	a	b	a	b	b	b
b	b	a	b	a	b	a	b	b	b
-	b	a	b	a	b	a	b	b	b
b	b	a	b	a	b	a	-	b	b
b	b	a	b	a	b	a	b	b	b
b	b	a	b	a	-	a	b	-	b
-	b	a	b	a	a	a	b	b	b
b	b	a	b	a	b	a	b	b	b
b	b	a	b	a	b	a	b	b	b
b	b	a	b	a	b	a	b	b	b
a	b	a	b	a	b	a	-	b	a
a	b	a	b	a	b	a	b	b	a
a	b	a	b	a	b	a	b	b	a
a	b	a	b	a	b	a	b	b	a
a	b	a	b	a	b	a	b	b	a
a	b	a	a	a	b	a	b	b	a
a	b	a	a	a	b	a	b	b	a
a	b	a	a	a	b	a	b	b	a
a	b	a	a	a	b	a	b	-	a

a	b	a	a	a	-	a	-	b	a
a	b	a	a	a	b	a	b	b	a
a	b	a	a	a	b	a	b	b	a
a	b	a	a	a	b	a	b	b	a
a	b	a	a	a	b	a	b	b	a
a	b	a	a	a	b	a	b	b	a
a	b	a	a	a	b	-	b	b	a
a	b	a	a	a	b	a	b	b	a
a	b	a	a	a	b	a	b	b	a
a	b	a	a	a	b	a	b	b	a
a	b	a	a	a	-	a	b	b	a
a	b	a	a	a	b	a	b	b	a
a	b	a	a	-	b	a	b	b	a
a	b	a	a	a	b	a	b	b	a
a	b	a	a	a	-	a	b	b	a
a	b	a	b	a	b	a	b	b	a
a	b	a	b	a	b	a	b	b	a
a	b	a	b	a	b	a	b	b	a
a	b	a	b	a	b	-	b	b	b
a	b	a	b	a	b	a	b	b	a
a	b	a	b	a	b	a	b	b	a
a	b	a	a	b	b	a	b	b	a
b	b	b	a	b	a	a	b	-	b
b	b	b	a	b	a	a	b	b	b
b	b	b	a	b	a	a	b	b	b
b	b	b	a	b	a	a	b	b	b
b	b	b	a	b	-	a	b	b	b
b	b	b	a	b	a	a	b	b	b
b	b	b	a	b	a	a	b	b	b
b	b	b	a	b	a	a	b	b	b
b	b	b	a	b	a	a	b	b	b
b	b	b	a	b	a	b	b	b	b
b	b	b	a	b	a	b	b	b	b
-	b	b	a	b	a	b	b	b	b
b	b	b	a	b	a	b	b	b	b
b	b	b	a	b	a	b	b	b	b
b	b	b	a	b	a	b	b	b	b
b	b	b	a	b	-	b	b	b	b
b	b	b	a	b	a	b	b	b	b
b	b	b	a	b	-	b	b	b	b
b	b	b	a	b	a	b	b	b	b
b	b	b	a	b	a	b	b	b	b
b	b	b	a	b	-	b	b	b	b
b	b	b	a	b	a	b	-	b	-
b	b	b	a	b	a	b	b	b	b
b	b	b	a	b	-	b	b	b	b
b	b	b	a	b	-	b	b	b	b

b	b	b	a	b	a	b	b	b	b
b	b	b	a	b	a	b	b	b	b
b	b	b	a	b	a	b	b	b	b
b	b	b	a	b	a	a	b	b	b
b	b	b	a	b	a	a	b	b	b
b	b	b	a	b	a	a	b	b	b
b	b	b	a	b	a	a	-	b	b
-	b	b	a	b	a	a	-	b	a
b	b	b	a	b	a	a	-	b	-
b	b	b	a	b	a	a	b	b	b
b	b	b	a	b	a	a	b	b	b
b	b	b	a	b	-	a	b	b	b
b	b	b	a	b	a	a	b	b	b
b	b	b	a	b	a	a	b	b	b
b	b	b	a	b	a	a	b	b	b
b	b	b	a	b	a	a	b	b	b
b	b	b	a	-	a	a	b	b	-
b	b	b	a	b	a	a	b	b	b
b	b	b	a	b	a	a	b	b	b
b	b	b	a	b	a	a	b	b	b
b	b	b	a	b	a	a	b	b	b
b	b	b	a	b	a	a	b	b	b
b	b	b	a	b	a	a	b	b	b
b	b	b	a	b	a	a	b	b	b
b	b	b	a	b	-	a	b	b	b
b	b	b	a	b	a	a	b	b	b
b	b	b	a	b	a	a	b	b	b
b	b	a	a	b	-	a	b	b	b
b	b	a	a	b	a	a	b	b	b
b	b	a	a	b	a	a	b	b	-
b	b	a	a	b	a	a	b	b	b
b	b	a	a	b	a	a	b	b	b
b	b	a	a	b	a	b	b	b	b
b	b	a	a	b	-	b	b	b	b
b	b	a	a	b	-	b	b	b	b
b	b	a	a	b	a	b	b	b	b
b	b	a	a	b	a	b	b	b	a
b	b	a	a	b	a	b	b	b	a
b	b	a	a	b	a	b	b	b	a
b	b	a	a	b	b	b	b	b	b
b	b	a	a	b	a	b	-	b	b
b	b	a	a	b	a	b	b	-	b
b	b	a	a	b	a	b	b	b	b
b	b	b	a	b	a	b	b	b	b
b	b	b	a	b	a	b	b	b	b
b	b	b	a	b	a	b	b	b	b
b	b	b	a	b	a	b	b	b	b

b	b	b	a	b	b	b	b	b	b
b	b	b	a	b	a	b	b	b	b
-	-	b	a	b	a	b	-	b	b
b	b	b	a	b	a	b	-	b	b
b	b	b	a	b	a	b	b	b	b
b	b	b	a	b	-	b	b	b	b
b	b	b	a	b	a	b	b	b	b
b	b	b	a	b	-	a	b	b	b
b	b	b	a	b	a	a	b	b	b
b	b	b	a	b	a	a	b	b	b
b	b	b	a	b	a	a	b	b	b
b	b	b	a	b	-	a	b	b	b
b	b	b	a	b	a	a	b	b	-
b	b	b	a	b	-	a	b	b	b
a	b	b	a	b	a	a	b	b	b
a	b	b	a	b	a	a	b	b	b
a	b	b	a	b	a	a	b	b	b
a	b	b	a	b	a	a	b	-	b
a	b	b	a	b	a	a	b	b	b
a	b	b	a	b	a	a	-	-	b
-	b	b	a	b	a	a	b	b	b
a	b	b	a	b	a	a	b	b	b
a	b	b	a	b	a	a	b	b	b
a	b	b	a	b	a	a	b	b	b
a	b	b	a	b	b	a	b	b	b
a	b	b	-	b	-	a	b	b	b
a	b	b	a	b	a	a	b	b	a
a	b	b	a	b	a	a	b	b	-
a	b	b	a	b	a	a	b	b	a
a	b	b	a	b	a	a	b	b	-
a	b	b	a	b	a	a	-	b	a
a	b	b	a	b	a	a	b	b	a
a	b	b	a	b	b	a	b	b	a
a	b	b	a	b	a	a	b	b	a
a	b	b	a	b	a	a	b	b	a
a	b	b	a	b	a	a	b	b	a
a	b	b	a	b	a	a	b	b	a
a	b	b	a	b	a	a	b	b	a
a	b	b	a	b	a	a	b	-	a
a	b	b	a	b	a	a	b	b	a
a	b	b	a	b	a	a	b	b	a
a	b	b	a	b	a	a	b	-	a
a	b	b	a	b	a	a	b	b	a
a	b	b	a	b	a	a	b	b	a
a	b	b	a	b	-	a	b	b	a
a	b	b	b	b	a	a	b	b	a
a	b	b	b	-	a	a	b	b	a

a	b	b	a	b	a	a	b	b	a
a	b	b	b	b	a	a	b	b	a
a	b	b	a	b	a	a	b	b	a
a	a	a	b	a	a	a	a	b	a
-	a	a	a	b	a	a	a	b	b
b	a	a	a	b	a	a	a	b	b
b	a	b	a	b	a	a	a	a	b
b	a	b	a	b	a	a	a	a	b
b	a	b	a	b	a	b	a	a	-
b	a	b	a	b	a	b	a	a	b
b	a	b	a	b	a	a	a	a	b
b	a	b	a	b	a	a	a	a	b
b	a	b	a	b	-	a	a	a	b
b	a	b	a	b	a	a	a	a	b
b	a	b	a	b	a	a	a	a	b
b	a	b	a	b	a	a	a	a	b
b	a	b	a	b	a	a	a	a	b
b	a	b	a	b	a	a	a	a	b
b	a	b	-	b	a	a	a	-	b
b	a	b	a	b	a	a	a	a	a
b	a	b	a	b	-	a	a	-	a
-	a	b	a	b	a	a	a	a	a
b	a	b	a	b	a	a	a	a	a
b	a	b	a	b	a	a	a	a	a
b	a	b	a	b	a	a	a	a	a
b	a	b	a	b	a	a	a	a	a
b	a	b	a	b	a	a	a	a	a
b	a	b	a	b	a	a	a	a	a
b	a	b	a	b	a	a	a	a	a
b	a	b	a	b	a	a	a	a	a
b	a	b	a	b	a	a	a	a	a
b	a	b	a	b	a	a	-	a	-
b	a	b	a	b	a	a	a	a	a
b	a	b	a	b	a	a	a	a	a
b	a	b	a	b	a	a	a	a	a
b	a	b	a	b	a	a	a	a	a
b	a	b	a	b	a	a	a	a	a
b	a	b	a	b	a	a	a	a	a
b	a	b	a	b	b	a	a	a	b
b	a	b	a	b	b	a	a	a	b
b	a	b	a	b	b	b	a	a	b
b	a	b	a	b	b	b	a	a	b
b	a	b	-	b	b	b	a	-	b
b	a	b	a	b	-	b	a	a	b
b	a	b	a	b	b	b	a	a	b
b	a	b	a	b	b	b	a	a	b
b	a	b	-	b	b	b	a	a	b

b	a	b	a	b	b	b	a	a	b
b	a	b	a	b	b	b	a	a	b
b	a	b	a	b	b	b	a	a	b
b	a	b	a	b	b	b	a	a	b
a	a	b	a	b	a	b	a	b	a
a	a	-	a	b	a	b	a	b	a
a	a	b	a	b	a	b	a	b	a
a	a	b	a	b	a	b	a	b	a
a	a	b	a	b	a	b	a	b	a
a	a	b	a	b	a	b	a	b	a
a	a	b	a	b	a	b	a	b	a
a	a	b	a	b	a	b	a	b	a
a	a	b	a	b	-	b	a	b	a
a	b	b	a	b	b	b	a	b	b
a	b	b	a	b	b	b	a	b	b
a	b	b	a	b	b	b	a	b	b
a	b	b	a	b	b	b	a	b	b
-	b	b	a	b	b	b	-	b	b
a	b	b	a	b	b	b	a	b	b
a	b	a	a	b	b	b	a	b	b
a	a	a	a	b	-	b	a	b	b
-	b	a	a	b	b	b	a	b	b
a	b	a	-	b	b	b	a	a	b
a	b	a	a	b	b	b	a	a	b
a	b	b	a	b	b	b	a	a	b
a	b	a	a	b	a	b	a	a	b
a	b	a	a	b	-	b	a	a	b
a	b	a	a	b	a	b	-	-	b
a	b	a	a	b	a	b	a	a	b
b	b	a	b	b	a	b	b	a	b
b	b	a	b	b	a	b	a	a	b
b	b	a	b	b	a	b	a	a	b
b	a	a	a	b	a	b	a	a	b
a	a	a	a	b	a	b	b	a	a
a	a	a	a	b	a	b	b	a	a
a	a	a	a	b	a	b	b	a	a
a	a	a	a	b	a	b	b	a	a
a	a	a	a	-	a	b	b	a	a
a	a	a	-	b	a	b	b	a	a
a	a	a	a	b	a	b	b	a	a
a	a	a	a	b	a	b	b	a	a
a	a	a	a	b	a	a	b	a	a
a	a	a	a	b	a	a	b	a	a
a	a	a	a	b	a	a	b	a	a
a	a	a	a	b	b	a	b	a	a
b	b	b	b	b	-	b	a	b	b
b	b	b	b	b	a	b	a	b	b

b	b	b	b	b	a	b	a	b	b
b	b	b	b	b	a	b	a	b	-
b	a	b	b	b	a	b	a	b	-
b	a	b	b	b	a	b	a	b	b
b	a	a	b	b	b	b	b	b	b
b	a	a	b	b	-	b	b	b	b
b	a	a	b	b	a	b	b	b	b
b	a	a	b	b	a	b	b	-	b
b	a	a	b	b	a	b	b	b	b
b	a	a	b	b	a	b	b	b	b
b	a	a	b	b	a	b	b	b	b
-	a	a	b	b	a	b	b	b	b
b	a	a	b	b	a	b	b	b	b
b	a	a	b	b	b	b	b	b	b
-	a	a	b	b	-	b	b	b	b
b	a	a	b	b	b	b	b	b	b
b	a	a	b	b	b	b	b	b	b
b	a	a	b	b	b	b	b	b	b
b	a	a	b	b	b	b	b	b	b
b	a	a	b	b	a	b	-	b	b
b	a	a	b	b	b	b	b	b	b
b	-	a	b	b	b	b	b	b	b
b	a	a	b	b	b	b	b	b	b
b	a	a	b	b	b	b	b	b	b
b	a	a	b	b	b	b	b	b	b
b	a	a	b	b	b	b	b	b	b
b	a	a	a	b	b	b	b	b	a
b	a	a	a	b	b	b	b	b	a
b	a	a	a	b	-	b	b	b	a
b	a	a	a	b	b	b	b	b	a
b	a	a	a	b	-	b	b	b	a
b	a	a	a	b	-	b	b	b	a
b	a	a	a	b	b	b	b	b	-
b	a	a	a	b	b	b	b	b	a
a	a	a	a	b	-	b	b	b	b
b	a	a	b	a	b	b	b	-	b
b	a	a	b	a	b	b	b	b	b
b	a	a	b	a	b	b	b	b	b
b	a	a	b	a	b	b	b	b	b
b	a	a	b	a	-	b	b	b	b
b	a	a	b	a	b	b	b	b	b
b	a	a	b	a	b	b	b	b	b
b	a	a	b	a	b	b	b	b	b
b	a	a	b	a	b	b	b	b	b
b	a	a	b	a	b	b	b	b	b
b	a	a	b	a	b	b	b	b	b
b	a	a	b	a	-	b	b	b	b

b	a	a	b	a	b	b	b	b	b
b	a	a	b	a	-	b	b	b	b
b	a	a	b	a	b	b	b	b	b
-	a	a	b	a	-	b	b	b	b
b	a	a	b	a	-	b	b	b	b
b	a	a	b	a	b	b	b	b	a
b	a	a	b	a	b	b	b	b	a
b	a	a	b	a	-	b	b	b	a
b	a	a	b	a	b	b	b	b	a
b	a	a	b	a	b	b	b	b	a
b	a	a	b	a	-	b	b	-	a
b	a	a	b	a	b	b	b	b	a
b	a	a	b	a	-	b	b	b	a
b	a	a	b	a	b	b	b	b	a
b	a	a	b	a	-	b	b	-	a
b	a	a	b	a	b	b	b	b	a
b	a	a	b	a	b	b	b	b	b
b	a	a	b	a	b	b	b	b	a
b	a	a	b	a	b	b	b	b	a
b	a	a	b	a	b	b	b	b	a
b	a	b	b	a	b	b	b	b	-
b	a	b	b	a	b	b	b	b	a
b	a	b	b	a	b	b	b	b	-
b	a	b	b	a	b	b	b	b	a
b	a	b	b	a	a	b	b	b	a
b	a	b	b	-	a	b	b	-	a
b	a	b	b	a	a	b	b	b	a
b	a	b	b	a	a	b	b	b	a
b	a	b	b	a	a	b	b	b	a
b	a	b	b	a	a	b	b	b	a
b	a	b	b	a	a	b	b	b	-
b	a	b	b	a	a	b	b	b	a
b	a	b	b	a	a	b	b	b	a
b	a	b	b	a	a	b	b	b	b
b	a	b	b	a	a	b	-	b	b
b	a	b	b	a	a	b	b	b	b
b	a	b	b	a	a	b	b	b	b
b	a	b	b	a	a	a	b	b	b
b	a	b	b	a	a	a	b	b	b
b	a	b	b	a	a	a	b	-	b
b	a	b	b	a	a	a	b	b	b
b	a	b	b	-	a	a	b	b	b
b	a	b	b	a	a	a	b	a	a
b	a	b	b	a	a	a	b	a	a
b	a	-	b	a	a	a	b	a	-
b	a	a	b	a	-	a	b	a	a
a	a	a	b	a	a	a	b	a	a

a	a	a	b	a	a	a	b	a	a
a	a	a	b	a	a	a	b	a	a
a	b	a	b	a	a	a	b	a	a
a	b	a	b	a	a	a	b	a	a
-	b	a	b	-	-	a	b	a	a
a	b	a	b	a	a	a	b	a	-
a	b	a	b	a	a	a	b	a	a
a	b	a	b	a	a	a	b	a	a
a	b	b	a	a	-	a	a	b	a
a	b	b	a	a	a	a	a	b	a
a	b	b	a	a	a	a	a	b	a
b	b	b	a	a	a	a	a	b	a
b	b	b	a	a	a	a	a	b	a
b	b	b	a	a	a	a	a	b	a
-	b	b	a	a	a	a	a	b	b
b	b	b	a	a	a	a	a	b	b
b	b	b	a	a	-	a	a	b	b
b	b	a	a	b	a	a	a	b	b
b	b	a	a	b	a	a	a	b	b
b	b	a	a	b	a	a	a	a	b
b	b	a	a	b	a	a	a	a	b
b	b	a	a	b	a	a	a	a	b
b	b	a	a	b	a	a	a	b	b
b	a	a	a	b	a	a	a	a	b
b	a	a	a	b	a	a	a	a	b
b	a	a	a	b	a	a	a	a	b
b	a	a	a	b	a	a	a	a	b
b	a	a	a	b	a	a	a	a	b
-	a	a	a	b	a	a	a	a	b
b	a	a	b	b	a	a	a	-	b
a	a	a	b	a	b	a	a	a	b
a	a	a	b	b	a	a	a	a	b
a	a	a	b	b	a	a	a	a	b
a	a	a	b	b	a	a	a	a	b
a	a	a	b	b	a	a	a	a	b
a	a	a	a	b	a	a	b	a	b
a	a	a	a	b	a	a	b	a	b
a	a	a	a	b	a	a	b	a	b
b	a	a	a	b	a	a	b	a	a
b	a	a	a	b	a	a	b	a	a
b	a	a	a	b	a	a	b	a	a
b	a	a	a	b	a	a	b	a	a
-	a	a	a	b	a	a	b	a	a
b	a	b	a	b	a	a	b	b	-
b	a	b	a	b	a	a	b	b	a
b	a	b	a	b	-	a	b	b	b
b	a	b	a	b	-	a	b	b	b

b	a	b	a	b	a	a	b	b	a
b	a	b	a	a	a	a	-	b	b
b	a	b	a	a	a	a	b	b	b
b	a	b	a	a	a	a	b	b	-
b	a	b	b	b	a	a	b	a	a
a	a	b	a	a	a	a	b	a	b
a	-	b	b	a	a	a	b	a	b
a	a	b	b	a	a	a	-	a	b
-	a	b	a	a	a	a	b	a	b
a	a	b	a	a	a	a	b	a	b
a	a	b	a	a	a	a	b	a	-
a	b	b	a	a	a	a	b	a	b
b	b	a	b	a	b	b	b	b	b
b	b	a	b	a	b	b	b	b	b
b	b	a	b	a	b	b	b	b	b
b	b	a	b	a	b	b	b	b	b
a	b	a	a	a	a	b	b	a	b
a	b	a	a	a	a	b	b	a	-
a	b	a	a	a	a	b	b	a	b
a	b	a	a	a	a	b	b	a	b
a	b	a	a	a	a	b	b	a	b
a	b	a	a	a	a	b	b	a	b
a	b	a	a	a	a	b	b	a	b
a	b	b	a	a	a	b	b	a	b
-	b	b	a	b	-	b	b	a	-
a	b	b	a	b	a	b	b	a	a
a	b	b	a	b	a	b	b	a	a
a	a	b	a	b	a	a	b	a	a
a	a	b	a	b	a	a	b	a	a
a	a	b	a	b	a	a	b	a	a
a	a	b	a	-	a	a	b	a	a
a	a	b	a	b	a	a	b	a	a
a	a	b	a	b	a	a	b	a	a
a	a	b	a	b	-	a	b	a	b
a	a	b	a	b	a	a	b	a	b
a	a	b	a	b	a	a	b	-	a
a	a	b	b	b	a	a	b	-	a
a	a	b	b	b	a	a	b	a	a
a	a	b	b	b	-	a	b	a	-
a	a	b	b	b	a	a	-	a	a
a	a	b	b	b	a	a	b	a	b
a	a	b	b	b	-	a	b	a	b
a	a	b	b	b	a	a	b	a	b
a	a	b	b	b	a	a	b	a	b
a	a	b	b	b	a	a	b	a	b
a	a	b	b	b	a	a	b	a	b
a	a	b	b	b	a	a	b	a	b

a	a	b	b	b	a	a	b	a	b
-	a	b	b	b	a	a	b	a	b
a	a	b	b	a	-	a	b	a	b
a	a	b	b	-	b	a	b	b	b
a	a	b	b	a	a	a	b	b	b
a	a	b	b	a	b	a	b	b	b
a	a	b	b	a	b	a	b	b	b
a	a	b	b	a	b	a	b	b	b
a	a	a	a	a	a	b	a	b	b
a	a	a	a	a	-	b	a	b	a
a	a	a	a	a	a	b	a	b	-
a	a	a	a	a	a	b	b	b	a
a	a	-	a	a	-	b	b	a	a
a	a	a	a	a	a	b	b	a	a
a	a	a	a	a	-	b	b	a	a
-	a	a	a	a	a	b	b	a	-
a	a	a	a	a	a	b	b	a	a
a	a	a	a	a	a	b	b	a	a
a	a	a	a	a	a	b	b	a	a
a	a	a	a	a	a	b	b	a	a
a	a	a	a	a	a	b	b	a	a
a	a	a	a	a	a	b	-	a	a
a	a	a	a	a	a	b	b	a	a
a	a	a	a	a	a	b	b	a	a
a	a	a	a	a	a	b	b	a	a
a	a	-	a	a	-	b	b	a	a
a	a	a	a	a	a	b	b	a	a
a	a	a	a	a	a	b	b	a	a
a	a	a	a	a	a	b	b	a	a
a	-	a	a	-	-	b	b	a	a
a	a	a	a	a	a	b	b	a	a
a	a	a	a	a	a	b	b	a	a
a	a	a	a	a	a	b	b	a	a
a	a	a	b	a	a	b	b	a	a
a	a	a	a	a	a	b	b	a	a
a	a	a	a	a	a	b	b	a	a
a	a	a	a	a	a	b	b	-	a
-	a	a	a	a	a	b	b	a	a
-	a	a	a	a	a	b	b	a	a
b	a	a	a	a	a	b	b	a	a
b	a	a	a	a	a	b	b	a	a
b	a	a	a	a	a	b	b	a	a
b	a	a	a	a	a	b	b	a	a
b	a	a	a	a	-	b	b	a	b
b	a	a	a	a	a	b	b	a	b
b	a	a	a	a	a	b	b	a	b
b	a	a	a	a	a	b	b	a	b

b	a	a	a	a	a	b	b	a	-
b	a	a	a	a	a	b	b	a	b
b	a	a	a	a	a	b	b	a	b
b	a	a	a	a	a	b	b	a	b
b	a	a	a	a	a	b	b	a	b
b	a	a	a	a	a	b	b	a	b
b	a	a	a	a	a	b	b	a	b
-	a	a	a	a	a	b	b	a	b
b	a	a	a	a	a	b	b	a	b
b	a	a	a	a	a	b	b	a	a
b	a	-	a	a	a	b	b	a	a
b	a	a	a	a	a	b	b	a	a
b	a	a	a	a	a	b	b	a	a
b	a	a	a	a	-	b	b	a	a
-	a	a	a	a	a	a	b	a	a
b	a	a	a	a	-	a	b	a	a
b	a	a	a	a	a	a	b	a	a
b	a	a	a	a	a	a	b	a	b
b	a	a	a	a	a	a	a	a	a
b	-	a	a	a	-	a	a	a	a
-	b	a	b	a	b	b	a	a	a
a	b	a	b	a	b	b	a	a	a
a	b	a	b	a	b	b	a	a	-
a	b	a	b	a	a	b	a	a	a
-	b	a	b	a	a	b	-	a	a
a	b	a	b	a	-	b	a	-	a
a	b	a	b	a	-	b	a	a	a
a	b	a	b	a	a	b	a	a	a
a	b	a	b	a	a	b	a	a	a
a	b	a	b	a	a	b	a	a	b
a	b	a	b	a	a	b	a	a	b
a	b	a	b	a	a	b	a	a	b
a	b	a	b	a	-	b	a	-	b
a	b	a	b	a	a	b	b	a	b
-	b	a	b	a	a	b	b	a	b
a	b	a	b	a	a	b	b	a	b
b	b	a	b	a	-	b	b	a	b
b	b	a	b	a	a	b	-	a	b
b	b	a	b	a	a	b	b	a	b
b	b	a	b	b	-	b	b	a	b
b	b	a	b	a	a	b	b	a	b
-	b	a	b	b	a	b	b	a	b
b	b	a	b	b	a	b	b	a	a
b	b	a	b	b	a	b	b	a	b
b	a	a	b	b	a	b	b	a	b
b	a	a	b	b	a	b	b	a	b
b	a	a	b	b	a	b	b	a	b

b	a	a	b	b	a	b	b	a	a
b	a	a	b	b	a	b	b	a	a
b	a	a	b	b	a	b	b	a	a
b	a	a	a	b	a	b	b	a	a
b	a	a	a	-	a	b	b	a	b
b	a	a	a	b	a	b	b	a	b
b	a	a	a	b	a	b	b	a	b
b	a	a	a	b	a	b	b	a	b
b	a	a	a	b	a	b	b	a	b
b	a	a	a	b	a	b	b	a	b
b	a	a	a	b	a	b	b	a	b
b	a	a	a	b	a	b	b	a	b
b	a	a	a	-	a	b	b	-	a
b	a	a	a	b	a	b	b	a	b
b	a	a	a	b	a	b	b	a	b
b	a	a	a	b	a	b	b	a	b
b	a	a	a	b	a	b	b	a	b
b	a	a	a	b	a	b	b	a	b
b	a	a	a	b	-	b	b	a	b
b	a	a	a	b	a	a	b	a	b
b	a	a	a	b	a	a	b	a	a
b	a	a	a	-	a	a	b	a	a
b	a	a	a	b	a	a	b	a	a
b	a	a	a	b	a	a	b	a	a
-	a	a	a	b	a	a	b	a	a
a	a	a	a	b	b	a	b	a	a
a	a	a	a	b	b	a	b	a	a
a	a	a	a	b	b	a	b	a	a
a	a	a	a	b	-	a	b	a	a
a	a	a	a	b	b	a	b	a	a
a	-	a	a	b	b	a	b	a	-
a	a	-	a	b	b	a	b	a	a
a	a	a	a	b	b	a	b	a	a
a	a	a	a	b	b	a	b	a	a
a	a	a	a	b	b	a	b	a	a
a	b	b	b	a	b	b	a	b	b
a	b	b	b	a	b	b	a	b	b
a	b	b	b	a	b	b	a	b	-
a	b	b	b	a	b	b	a	b	b
a	b	b	b	a	b	b	-	b	a
a	b	b	b	a	b	b	a	b	a
a	b	b	b	a	-	-	-	b	a
a	b	b	b	a	b	b	a	b	a
a	b	b	b	a	b	b	a	b	-
a	b	b	b	a	b	b	a	b	b
a	b	b	b	a	b	b	a	b	a
a	b	b	a	a	b	b	a	-	b

a	b	b	b	a	b	b	a	b	b
a	b	b	b	a	b	b	a	b	b
a	b	b	b	a	b	b	a	b	b
a	b	b	b	a	b	-	a	b	b
a	b	b	b	a	b	b	a	b	b
a	b	b	b	a	b	b	a	b	b
a	b	b	b	a	b	b	a	b	b
a	b	b	b	a	b	b	a	b	b
a	b	b	b	a	b	b	a	b	b
a	b	b	b	a	-	a	b	b	b
a	b	b	b	a	b	a	a	b	b
a	b	b	b	a	b	a	a	b	b
a	b	b	b	a	b	a	-	b	b
a	b	b	b	a	b	a	b	b	b
a	b	b	b	a	b	a	-	b	b
a	b	b	b	a	b	a	b	b	b
a	b	b	b	a	b	a	a	b	b
a	b	b	b	a	b	a	a	b	-
a	b	b	b	a	b	-	a	b	b
a	b	b	a	a	b	a	a	b	b
a	b	b	a	a	b	a	a	b	b
a	b	b	a	a	b	a	a	b	b
a	b	b	a	a	b	a	a	b	b
a	b	b	a	b	b	a	a	b	b
a	b	b	a	a	b	a	a	b	b
a	b	b	a	b	-	a	a	b	b
a	b	b	a	b	b	a	a	b	b
a	b	b	a	b	b	a	a	b	b
a	b	b	a	b	b	a	a	b	b
a	b	b	a	b	b	a	a	b	b
b	b	b	a	b	b	a	a	b	b
b	b	b	a	b	b	a	a	b	b
b	b	b	a	b	b	a	a	b	b
b	b	b	a	b	-	a	a	b	b
b	b	b	a	b	b	a	a	b	b
b	b	b	a	a	b	a	a	b	b
b	b	b	a	a	b	a	a	b	b
b	b	b	a	a	b	a	a	b	b
b	b	b	a	a	b	a	a	b	b
b	b	b	a	a	b	b	a	b	b
b	b	b	a	a	b	b	a	b	b
b	b	b	a	a	b	b	a	b	-
b	b	b	a	a	b	b	a	b	b
b	b	b	a	a	b	b	a	b	b
b	b	b	a	a	b	b	a	b	b
b	-	b	a	a	b	b	a	-	b
b	a	b	b	b	a	a	b	b	-

b	a	b	b	b	a	a	b	b	-
b	a	b	b	b	a	a	b	b	a
b	a	b	b	b	a	a	b	b	b
b	a	b	b	b	a	a	b	b	b
b	a	b	b	b	a	a	b	b	b
b	a	b	b	b	a	a	b	b	a
b	a	b	b	b	a	a	b	b	a
b	a	b	b	b	a	a	b	b	a
b	a	b	b	b	a	a	b	b	a
b	a	b	b	b	a	a	b	b	b
b	a	b	b	b	a	a	b	b	b
b	a	b	b	b	a	a	b	b	b
b	a	b	b	b	a	a	b	b	b
b	a	b	b	b	a	a	b	b	b
b	a	b	b	b	a	a	b	b	b
b	a	b	b	b	a	b	b	b	b
b	a	b	b	b	a	b	b	b	b
b	a	b	b	b	a	a	b	b	b
b	b	b	a	b	a	b	b	b	b
b	b	b	a	b	a	b	b	b	b
b	b	b	a	b	a	b	a	b	b
b	b	b	a	b	a	b	a	b	b
b	b	b	a	b	a	b	a	b	b
b	b	b	a	b	a	b	a	b	a
b	b	b	a	b	a	b	a	b	a
b	b	b	a	b	a	b	a	b	a
b	b	b	a	b	a	b	a	b	a
a	b	a	a	b	a	b	b	b	a
a	b	a	a	b	a	b	b	b	a
a	a	a	a	b	a	b	b	b	b
a	a	a	a	b	a	b	b	b	b
a	a	a	a	b	a	b	b	b	a
a	a	a	a	b	a	b	b	b	b
a	a	a	a	b	a	b	b	b	b
a	a	a	a	b	a	b	b	b	a
a	a	a	a	b	a	b	b	b	a
a	a	a	a	b	b	b	b	b	a
a	b	a	a	b	b	b	b	b	a
b	a	a	b	b	b	b	b	b	b
a	b	a	b	b	b	b	b	b	b
b	a	b	a	a	b	a	a	a	a
b	b	a	b	b	a	a	a	b	a
b	b	a	b	b	a	a	a	b	a
b	b	a	b	b	a	a	a	b	a
b	b	a	b	b	a	a	a	b	a
b	b	a	b	b	a	a	a	b	a
b	b	a	b	b	a	a	a	b	a

b	b	a	b	b	a	a	a	b	a
b	b	a	b	b	a	a	a	b	a
b	b	a	b	b	a	a	a	b	a
b	b	a	b	b	a	a	a	b	a
b	b	a	b	b	a	a	a	b	a

YW171	YW172	YW173	YW174	YW175	YW176	YW177	YW178	YW179	YW180
a	a	-	a	b	b	b	a	a	a
a	a	b	a	b	b	b	a	b	a
a	a	b	a	b	b	b	a	b	b
a	a	b	a	b	b	b	a	b	a
a	a	b	a	b	b	b	a	b	a
a	a	b	a	b	b	b	a	b	a
a	a	b	a	b	b	b	a	b	a
a	a	b	a	b	b	b	a	b	a
a	a	b	a	b	b	b	a	b	b
a	a	b	a	b	b	b	a	b	a
a	a	b	a	b	b	b	a	b	a
a	a	b	a	b	b	b	a	b	a
a	a	-	a	b	b	b	a	b	a
a	a	b	a	b	b	b	a	b	a
a	a	b	a	b	b	b	a	b	a
a	a	b	a	b	b	b	a	b	a
a	a	b	-	b	b	b	a	b	a
a	a	b	a	b	b	b	a	b	a
a	a	b	a	b	b	b	-	b	b
a	a	b	a	b	b	b	a	b	a
a	a	-	a	b	b	b	a	b	a
a	a	b	a	b	b	b	a	b	a
a	a	b	a	b	b	b	a	b	a
a	a	b	a	b	b	b	a	b	b
a	a	b	a	b	b	b	a	b	a
a	a	b	a	b	b	b	a	b	a
a	-	b	a	b	b	b	a	b	a
a	a	b	a	b	b	b	a	b	a
a	a	b	a	-	b	b	a	b	a
a	a	b	a	b	b	b	a	b	a
a	a	b	a	b	b	b	a	b	a
a	a	b	a	b	b	b	a	b	a
-	a	b	a	b	b	b	a	b	a
a	a	b	a	b	b	b	a	b	a
a	a	b	a	b	b	b	a	b	a
a	a	b	a	b	b	b	a	b	a
a	a	b	a	b	a	b	a	b	a
a	a	b	a	b	a	b	a	b	a
a	a	b	a	b	a	b	a	b	a
a	a	b	a	b	a	b	a	b	b
a	a	b	a	b	a	b	a	a	a
a	a	b	a	b	a	b	a	b	a
a	a	b	a	b	a	b	a	b	a
a	a	b	a	b	a	b	a	b	a
a	a	b	a	b	a	b	a	b	b

a	a	b	a	b	b	b	a	b	-
a	a	-	a	b	a	b	a	b	a
a	a	b	a	b	a	b	a	b	a
a	a	b	a	b	a	b	a	b	a
-	a	b	a	b	a	b	a	b	-
a	a	b	a	b	a	b	a	b	a
a	a	b	a	b	a	b	a	b	a
a	a	b	a	b	a	b	a	b	a
a	a	b	a	b	a	b	a	b	a
a	a	b	a	b	a	b	a	b	a
a	a	b	a	b	a	b	b	b	a
a	a	b	a	b	a	b	b	b	a
a	a	b	a	b	a	b	b	b	a
a	a	b	a	b	a	b	b	b	a
a	a	b	a	b	a	b	b	b	a
a	a	b	a	b	a	b	b	b	a
a	a	b	a	b	a	b	b	b	a
a	a	b	a	b	a	b	b	b	a
a	a	b	a	b	a	b	-	b	a
a	a	b	a	b	a	b	b	b	a
a	-	b	a	b	a	b	b	b	-
a	a	b	a	b	a	b	b	b	a
a	a	b	a	b	a	b	b	b	a
a	b	b	a	b	a	b	b	b	a
a	b	b	a	b	a	b	b	b	b
a	b	b	a	b	a	b	b	b	b
a	b	b	a	b	a	b	b	b	a
a	b	b	a	b	a	b	b	b	b
a	b	b	a	b	a	b	b	b	b
a	b	b	a	b	a	b	b	b	b
a	b	b	a	b	a	b	b	b	a
a	b	a	a	b	a	b	b	b	b
a	b	a	a	b	a	b	b	b	b
a	b	a	a	b	a	b	b	b	b
a	b	a	a	b	a	b	b	b	b
a	b	a	a	b	a	b	b	b	b
a	b	a	a	b	a	-	b	b	b
a	b	a	a	b	a	b	b	b	b
a	b	a	a	b	a	b	b	b	b
a	a	b	a	a	a	a	a	a	b
a	a	b	a	a	a	a	a	a	b
a	a	b	a	a	a	a	a	a	a
a	a	-	a	a	a	a	a	a	b
a	a	b	a	a	a	a	a	a	b
a	a	b	a	a	a	a	a	a	b
a	a	b	a	a	a	a	a	a	b
a	a	b	a	a	a	a	a	a	a

a	a	b	a	a	a	a	a	a	b
a	a	b	a	a	a	a	a	a	b
a	a	b	a	a	a	a	a	a	b
a	a	b	a	a	a	a	a	a	b
a	a	-	a	a	a	a	a	a	-
a	a	b	a	a	a	a	a	a	-
a	a	-	a	a	a	a	a	a	b
a	a	b	a	a	a	a	a	a	b
a	a	b	a	a	a	a	a	a	b
a	a	b	a	a	a	a	a	a	b
a	a	b	a	a	a	a	a	a	b
a	a	a	a	a	a	a	a	a	b
a	a	a	a	a	a	a	a	a	b
a	a	a	a	a	a	a	a	a	a
a	a	-	a	a	a	a	a	a	b
a	a	a	a	a	a	a	a	a	b
a	a	a	a	a	a	a	a	a	b
a	a	a	a	a	a	a	a	a	b
a	a	a	a	a	a	a	a	a	b
a	a	a	a	a	a	a	a	a	b
-	a	a	a	a	a	a	a	a	b
a	a	a	a	a	a	a	a	a	-
a	a	a	a	a	a	a	a	a	b
a	a	a	a	a	a	a	a	a	-
-	a	-	a	a	a	a	a	a	b
a	a	a	a	a	a	a	a	a	b
a	a	a	a	a	a	a	a	a	b
a	a	a	a	a	a	a	a	a	b
a	a	a	a	a	a	a	a	a	b
a	a	a	a	a	a	a	a	a	b
a	a	a	a	a	a	a	a	a	a
a	a	a	a	a	a	a	a	b	b
a	a	a	a	a	a	a	a	b	b
a	a	a	a	a	a	a	a	b	a
a	a	a	a	a	a	a	a	b	b
a	a	a	a	a	a	a	a	b	b
a	a	a	a	a	a	a	a	b	b
a	a	a	a	a	a	a	a	b	b
a	a	a	b	a	a	a	-	b	b
a	a	a	b	a	a	a	a	b	b
a	a	a	b	a	a	a	a	b	b
a	a	a	b	a	a	a	a	a	b
a	a	a	b	a	a	a	a	a	b
a	a	a	b	a	a	a	a	a	-
b	a	a	b	a	a	a	a	a	b
b	a	a	b	a	a	a	a	a	b
b	a	a	b	a	a	a	a	a	a

b	a	-	b	a	a	a	b	a	b
b	a	a	b	a	a	a	b	a	a
b	a	a	b	a	a	a	b	a	a
b	a	-	b	a	a	a	b	a	-
b	a	a	b	a	a	a	b	a	a
b	a	a	b	a	b	a	b	a	a
b	a	a	b	a	b	a	b	a	a
b	a	a	b	a	b	a	b	a	a
b	a	a	b	a	b	a	b	a	a
b	a	a	b	a	b	a	b	a	a
b	a	a	b	a	b	a	a	a	a
b	a	-	b	a	b	a	a	a	a
b	a	a	b	a	b	a	a	a	a
b	a	a	b	a	b	a	a	a	a
b	a	a	b	a	b	a	a	a	a
b	a	a	b	b	b	a	a	a	a
b	a	a	b	b	b	a	a	a	a
b	a	a	b	b	b	a	a	a	a
b	a	a	b	b	b	a	a	a	a
b	a	a	b	b	b	a	a	a	a
b	a	-	b	b	b	-	a	a	a
b	a	a	b	b	b	b	a	a	b
b	a	a	-	b	b	b	a	a	a
b	a	a	b	b	b	b	a	a	a
b	a	a	b	b	a	b	a	a	a
b	a	a	b	b	a	b	a	a	a
b	b	a	b	b	a	b	a	a	a
b	b	a	b	b	a	b	a	a	a
b	b	a	b	b	a	b	a	a	a
b	b	a	b	b	a	b	a	a	-
b	b	a	b	b	a	b	a	a	a
b	b	a	b	b	a	b	a	a	a
b	b	a	b	b	a	b	a	a	a
b	b	a	b	b	a	b	a	a	a
b	b	a	b	b	a	b	a	a	a
b	b	a	b	b	a	b	a	a	a
b	b	a	b	b	a	b	a	a	a
b	b	a	b	b	a	b	a	a	a
b	b	a	b	b	a	b	a	a	a
b	b	a	b	b	a	b	a	a	a
b	b	a	b	b	a	b	a	a	a
b	-	a	b	b	a	b	a	a	a
b	b	a	a	b	a	b	a	a	a
b	b	a	a	b	a	b	a	a	a
b	b	a	a	b	a	b	a	a	a
b	b	a	a	b	a	b	b	a	a
b	b	a	a	b	a	b	b	a	a

-	b	a	a	b	a	b	b	a	a
b	b	a	a	b	a	b	b	a	b
b	b	a	a	b	a	b	b	a	a
b	b	a	a	b	a	b	b	a	a
b	b	a	a	b	a	b	b	a	a
b	b	a	a	a	a	b	b	a	a
b	b	a	a	a	a	b	b	a	a
b	b	a	a	a	a	b	b	a	a
-	-	a	a	a	a	b	b	a	a
b	b	a	a	a	a	b	b	a	a
b	b	a	a	a	a	b	b	a	a
b	b	a	a	a	a	b	b	a	-
a	b	a	b	a	b	a	a	b	b
a	b	a	b	a	b	a	a	b	a
a	b	a	b	a	b	a	a	b	a
a	b	a	b	a	b	a	a	b	a
a	b	a	b	a	b	a	a	b	a
a	b	a	a	a	b	a	a	b	a
a	b	a	a	a	b	a	a	b	a
a	b	a	a	a	b	a	a	b	a
a	b	a	a	a	b	a	a	b	a
a	b	a	a	a	b	a	a	b	a
a	b	-	a	a	b	a	a	b	a
a	b	a	a	a	b	a	a	b	a
a	b	a	a	a	b	a	a	b	-
a	b	a	a	a	b	a	a	b	a
a	-	a	a	a	-	a	a	b	a
a	b	a	a	a	b	a	a	b	a
a	b	-	a	a	b	a	a	b	a
a	b	a	a	a	a	a	a	b	a
a	b	b	a	a	a	a	a	b	a
-	b	b	a	a	a	a	a	b	-
a	b	b	a	a	a	a	a	a	a
a	b	b	a	a	a	a	-	a	a
a	a	b	a	a	a	a	a	a	a
a	a	b	a	a	a	a	a	a	a
a	a	b	a	a	a	a	a	a	a
a	a	b	a	a	a	a	a	a	a
a	a	b	a	a	a	a	a	a	a
a	-	b	a	a	a	a	a	a	a
a	a	b	a	a	a	a	a	a	a
a	a	b	a	a	a	a	a	a	a
a	a	b	a	a	a	a	a	a	a
a	a	b	a	a	a	a	a	a	a
a	a	-	a	a	a	a	a	a	a
a	a	b	a	a	a	a	a	a	a
a	a	b	a	a	a	a	-	a	a

a	a	b	a	a	a	a	a	a	a
a	a	b	a	a	a	a	a	a	a
a	a	b	a	a	a	a	a	a	a
a	a	a	a	a	a	a	a	a	a
a	a	a	a	a	a	a	a	a	a
a	a	a	a	a	a	a	a	a	a
a	a	a	a	a	a	a	a	a	a
a	a	a	a	a	a	a	a	a	-
a	a	a	a	a	a	a	a	a	a
a	a	a	b	a	a	a	a	a	a
a	a	a	b	a	a	a	a	a	a
a	a	a	b	a	a	a	a	a	a
a	a	a	b	a	a	a	a	a	a
a	a	a	b	a	a	a	a	a	-
a	a	a	b	a	a	a	a	b	a
a	a	a	b	a	a	a	a	b	a
a	a	a	b	a	a	a	a	b	a
a	a	a	b	a	a	a	a	b	b
a	a	a	b	a	a	a	a	b	a
-	a	a	b	a	a	a	a	b	a
a	a	a	b	a	b	a	a	b	a
a	a	a	b	a	b	a	a	b	a
a	a	a	b	a	b	a	a	b	-
a	a	a	b	a	b	a	a	b	a
a	a	a	b	a	b	a	a	b	a
a	a	a	b	a	b	a	a	b	a
a	a	a	b	a	b	a	a	b	a
a	a	a	b	a	b	a	a	b	a
a	a	a	b	a	b	a	a	b	a
a	a	a	b	a	b	a	a	b	a
a	a	a	b	a	b	a	a	b	a
a	a	a	b	a	-	a	a	b	a
a	a	a	b	a	b	a	a	b	a
a	a	a	b	a	b	a	a	b	a
a	a	a	b	a	b	b	a	b	a
a	a	a	a	a	b	b	a	b	a
a	a	a	a	a	b	b	a	b	a
a	a	a	a	a	b	b	a	b	a
a	a	a	a	a	b	b	a	b	a
a	a	a	a	a	b	b	a	a	a
a	a	a	a	a	b	b	a	a	a
a	a	-	a	a	b	b	a	a	-
a	a	a	a	a	b	b	a	a	a
a	a	a	a	a	b	b	a	a	a
a	a	a	a	a	b	b	a	a	a
a	a	a	a	a	b	b	a	a	a
a	a	a	a	a	b	b	a	a	a

a	a	a	a	a	b	b	a	a	a
a	a	a	a	a	b	b	a	a	b
a	a	a	a	a	b	b	a	a	a
b	a	a	a	a	b	b	a	a	a
b	a	a	a	-	b	b	a	a	a
b	a	a	a	a	b	b	a	a	a
b	a	a	a	a	b	b	a	a	a
b	a	a	a	a	b	b	a	a	a
b	a	a	a	a	b	b	a	a	a
b	a	a	a	a	b	b	a	a	a
b	a	a	a	a	b	b	a	a	a
b	a	a	a	a	b	b	a	a	a
b	a	a	a	a	b	b	a	a	a
b	a	a	a	a	b	b	a	a	a
b	a	a	a	a	b	b	a	a	a
b	a	a	a	a	b	b	a	a	a
b	a	a	a	a	b	b	a	a	a
b	a	a	a	a	b	b	a	a	a
b	a	a	a	a	b	b	a	a	-
b	b	a	a	a	b	b	a	a	a
b	b	a	a	a	b	b	a	a	-
b	b	a	a	a	b	b	a	a	b
b	b	a	a	a	b	b	a	a	a
b	b	a	a	a	b	b	b	a	b
b	b	a	a	a	b	b	b	a	-
-	b	a	a	a	b	b	b	a	a
b	b	a	a	a	b	b	b	a	-
b	b	a	a	a	b	b	b	a	a
b	b	a	a	a	b	b	b	a	a
b	b	a	a	a	b	a	b	a	a
b	b	a	a	a	b	a	b	a	a
-	b	a	a	a	b	a	b	a	a
b	b	a	a	a	b	a	b	a	a
b	b	a	a	a	b	a	b	a	a
b	b	a	a	a	b	a	b	a	a
b	b	b	a	a	b	a	b	a	a
b	b	a	a	a	b	a	b	a	a
b	b	a	a	a	b	a	b	a	a
a	b	b	b	a	b	b	a	a	a
a	b	b	b	a	b	b	a	a	a
a	b	b	b	a	b	b	a	a	a
a	b	b	b	a	b	b	a	a	a
a	b	b	b	a	b	b	a	a	-
a	b	b	b	a	b	b	a	a	a
a	b	b	b	a	b	b	a	a	a
a	b	b	b	a	b	b	a	a	a
a	b	b	b	a	b	b	a	a	a

-	b	b	b	a	b	b	a	a	a
a	b	b	b	a	b	b	a	a	a
a	b	-	b	a	b	b	a	a	a
a	b	b	b	a	b	b	a	a	a
a	b	b	-	a	b	b	a	a	a
a	b	b	b	a	b	b	a	a	a
a	b	b	b	a	b	b	a	a	a
a	b	b	b	a	b	b	a	a	a
a	b	b	b	a	b	b	a	a	a
a	b	b	b	a	b	b	a	a	a
a	b	b	b	a	b	b	a	a	a
a	b	b	b	a	b	b	a	a	a
a	b	b	b	a	b	b	a	a	a
a	b	b	b	a	b	b	a	a	a
a	b	b	b	a	b	b	a	a	-
a	b	b	b	a	b	b	a	a	a
a	b	b	b	a	b	b	a	a	a
a	b	-	-	a	b	b	a	a	a
a	b	b	b	b	a	b	a	a	-
a	b	-	b	b	a	b	a	a	a
a	b	b	b	b	a	b	a	a	a
a	b	b	b	b	a	b	a	a	a
a	b	b	b	b	a	b	a	b	-
a	b	b	b	b	a	b	a	b	a
a	b	b	b	b	a	b	a	b	a
a	b	-	b	b	a	b	a	b	b
a	b	a	b	b	a	b	a	b	a
a	-	a	b	b	a	b	a	b	a
a	b	a	b	b	a	b	a	b	a
b	b	a	b	b	a	b	a	b	a
b	b	a	b	b	a	b	a	b	b
b	b	a	b	b	a	b	a	b	a
b	b	a	b	b	a	b	a	b	-
b	b	a	b	b	a	b	a	b	a
b	b	a	b	b	-	b	a	b	a
-	b	a	b	b	a	b	a	b	b
a	b	a	b	b	a	b	a	b	a
a	b	a	b	b	a	b	a	b	a
a	b	a	b	b	a	b	a	b	a
a	b	a	b	b	a	b	a	b	a
a	b	a	b	b	a	b	a	b	a
a	b	a	b	b	a	b	a	b	a
a	b	a	b	a	a	b	a	b	a
-	b	a	b	a	a	b	a	b	a
a	b	-	b	a	a	b	a	b	-
a	-	a	b	a	a	b	a	b	a
a	b	a	b	a	a	b	a	b	a

a	b	a	b	a	a	b	a	b	a
a	b	a	b	a	a	b	a	b	a
a	b	a	b	a	a	b	a	b	a
a	b	a	b	a	a	b	a	b	a
a	b	a	b	a	a	b	a	b	a
a	b	a	b	a	a	b	a	b	a
b	b	a	b	a	a	b	a	b	a
b	b	a	b	a	a	b	a	b	a
b	b	a	a	a	a	b	a	b	a
b	b	a	a	a	a	b	a	b	a
b	a	a	a	a	a	b	a	b	a
b	a	a	a	a	a	b	a	b	a
b	a	a	a	a	a	b	a	b	a
b	a	a	a	a	a	b	a	b	a
b	a	a	a	a	a	b	a	b	a
b	a	a	a	a	a	b	a	b	a
b	-	a	a	a	a	b	a	b	a
b	a	-	a	a	a	b	a	b	a
b	a	a	a	a	a	b	a	b	a
b	a	a	a	a	a	b	a	b	a
b	a	a	a	a	a	b	a	b	a
b	a	a	a	a	a	b	a	b	a
b	a	a	a	a	a	b	a	b	a
b	a	a	a	a	a	b	-	b	b
b	a	a	a	a	a	b	a	a	a
b	a	a	a	a	a	b	a	a	a
b	a	a	a	a	a	b	a	a	a
-	a	a	a	a	a	b	a	a	a
b	a	a	a	a	a	b	a	a	a
b	a	a	a	a	a	b	a	a	a
b	a	a	a	a	a	b	a	a	a
b	a	a	a	a	a	b	a	a	a
b	a	a	a	a	a	b	a	a	a
b	-	a	a	a	a	b	a	a	-
b	a	a	a	a	a	b	a	a	a
-	a	a	a	a	a	b	a	a	a
b	a	a	a	a	a	b	a	a	a
b	a	-	a	a	a	b	a	a	b
b	a	a	a	a	a	a	a	a	a
b	a	a	a	a	a	a	a	a	a
a	b	a	b	a	a	b	a	a	a
a	b	a	b	a	a	b	a	a	a
a	b	-	b	a	a	b	a	a	-
a	b	a	b	a	a	b	a	a	a
a	b	a	b	a	a	a	a	a	a
a	b	a	b	a	a	a	b	a	b
a	b	a	b	a	a	a	b	a	-

a	b	a	b	a	a	a	b	a	b
a	b	a	b	a	a	a	b	a	b
a	b	a	b	a	a	a	b	a	b
a	b	a	b	a	a	a	b	a	b
-	b	a	b	a	a	a	b	a	b
a	b	a	b	a	b	a	b	a	b
a	b	a	b	a	b	a	b	a	b
a	b	a	b	a	b	a	b	a	b
a	b	a	b	b	b	a	b	a	-
a	b	a	b	b	b	a	b	a	a
a	b	a	b	b	b	a	b	a	b
a	b	a	b	b	b	a	b	a	b
a	b	a	b	b	b	a	b	a	b
-	b	a	b	b	b	a	b	a	b
a	b	a	b	b	b	a	b	a	b
a	b	a	b	b	b	a	b	a	b
-	b	a	b	b	b	a	b	b	b
a	b	-	b	b	b	a	b	b	b
a	b	-	b	b	b	a	b	b	b
a	b	-	b	b	b	a	b	b	b
a	b	a	b	b	b	a	b	b	b
a	b	-	b	b	b	a	b	b	b
a	b	-	b	b	b	a	b	b	b
a	b	a	b	b	b	a	b	b	b
a	b	a	b	b	b	a	b	b	a
a	b	a	b	b	b	a	b	b	b
a	b	a	b	b	b	a	b	b	-
-	b	a	b	b	b	a	b	b	b
a	b	a	b	b	b	a	b	b	b
a	b	-	b	b	b	a	b	b	b
a	b	a	b	b	b	a	b	b	b
a	-	a	b	-	b	a	b	b	-
a	b	a	b	b	b	a	-	b	b
a	b	a	b	b	b	a	b	b	b
a	b	a	b	b	b	a	b	b	a
a	b	a	b	b	b	a	b	b	b
a	b	a	b	b	b	-	b	b	b
a	b	b	b	b	b	a	b	b	b
a	b	a	b	b	b	a	b	b	b
a	b	a	b	b	b	a	b	b	b
a	b	a	b	b	b	a	b	b	b
a	b	a	b	b	b	a	b	b	b
b	b	a	b	b	b	a	b	b	b
b	b	a	b	b	b	a	b	b	a
b	b	a	b	b	b	a	b	b	b
b	b	a	b	b	b	a	b	b	b
b	-	a	b	b	b	a	b	b	b

b	b	a	b	b	b	a	b	b	b
b	b	a	b	b	b	a	b	b	b
b	b	a	b	b	b	a	b	a	b
-	b	a	b	b	b	a	b	a	a
b	b	a	b	b	b	a	b	b	b
b	b	a	b	b	b	a	b	b	b
b	b	a	b	b	b	a	b	b	b
b	b	a	b	b	b	a	b	b	a
b	b	a	b	b	b	a	b	b	b
b	b	a	b	b	b	a	b	b	b
b	b	a	b	b	b	a	b	b	b
b	b	a	b	b	b	a	b	b	b
b	b	-	b	b	b	a	b	b	b
b	b	a	b	b	b	a	b	b	b
b	b	a	b	b	b	a	b	b	b
b	b	a	b	b	b	a	b	b	b
b	b	-	b	b	b	a	b	b	b
b	-	a	b	b	b	a	b	b	a
b	b	a	b	b	b	a	b	b	b
b	-	a	b	b	b	a	-	b	-
b	b	a	b	b	b	a	a	b	b
b	b	a	b	b	b	a	a	b	b
b	b	a	b	b	b	a	a	b	b
b	b	a	b	b	b	a	a	b	b
b	b	-	b	b	b	a	a	b	b
b	b	a	b	b	b	a	a	b	b
b	b	a	b	b	b	a	a	b	b
b	b	a	b	b	b	a	a	b	b
b	b	a	b	b	b	a	a	b	a
b	b	a	b	b	b	a	a	b	b
b	b	a	b	b	b	a	a	b	b
b	b	a	b	b	b	b	a	b	b
b	b	a	b	b	b	b	a	b	b
b	b	a	b	b	b	b	a	b	b
b	b	a	b	b	b	b	a	b	b
b	b	-	b	b	b	b	a	b	b
b	-	a	b	b	b	b	a	b	b
b	b	a	b	b	b	b	a	b	b
b	b	a	b	b	b	b	a	b	b
b	b	a	b	b	b	b	a	b	b
b	b	a	b	b	b	b	a	b	b
b	b	a	b	b	b	b	a	b	a
b	-	a	b	b	b	b	a	b	b
b	b	a	b	b	b	b	a	b	b
b	b	a	b	b	b	b	a	b	b
b	b	a	b	b	b	b	a	b	b
b	b	a	b	b	b	b	a	b	-

b	a	a	b	b	b	b	a	b	b
b	-	a	b	b	b	b	a	b	b
b	a	-	b	b	b	b	a	b	b
b	a	-	b	b	b	b	a	b	b
b	a	b	b	b	b	b	a	b	b
b	a	b	b	b	b	b	a	b	b
b	a	b	b	b	b	b	a	b	b
b	-	b	b	b	b	b	a	b	b
b	-	b	b	b	b	b	a	b	b
b	a	b	b	b	b	b	a	b	b
b	a	b	b	b	b	b	a	b	b
b	a	b	b	b	b	b	a	b	b
b	a	b	b	b	b	b	a	b	-
b	a	b	b	b	b	b	a	b	b
b	a	b	b	b	b	b	a	b	b
b	a	b	b	b	b	b	a	b	b
b	a	b	b	b	b	b	a	b	b
b	a	b	b	b	b	b	a	b	b
b	a	b	b	b	b	b	a	b	b
b	a	a	b	a	a	b	a	a	a
b	a	a	b	a	a	b	a	a	a
b	a	a	b	a	a	b	a	a	a
b	a	a	a	a	a	b	a	a	a
b	a	a	a	a	a	b	a	a	a
b	a	a	b	a	a	b	a	a	a
b	a	a	a	a	a	b	a	a	a
b	a	a	a	a	a	b	a	a	a
b	a	a	a	a	a	b	a	a	a
-	a	a	a	a	a	b	a	b	a
b	a	a	a	a	a	b	a	b	a
b	a	a	a	a	a	b	a	b	a
b	a	a	a	a	a	b	a	b	a
b	a	a	a	a	a	b	a	b	a
b	a	a	a	a	a	b	a	b	a
b	a	a	a	a	a	b	a	b	a
b	a	a	a	a	a	b	a	b	a
-	a	a	a	a	a	b	a	b	a
b	a	a	a	a	a	b	a	b	-
b	a	a	a	a	a	b	a	b	a
b	a	a	a	a	a	b	a	b	a
b	a	a	a	a	a	b	a	b	a
b	a	a	a	a	a	b	a	b	a
b	a	a	a	a	a	b	a	b	a
-	b	a	a	a	a	b	a	b	a
b	b	a	a	a	a	b	a	b	a
b	b	-	a	a	a	b	a	b	b
b	b	a	a	a	a	b	a	b	-

b	b	a	a	a	a	b	a	b	a
b	b	a	a	a	a	b	a	b	a
b	b	a	a	a	a	b	a	b	a
b	b	a	a	a	a	b	a	b	a
b	b	a	a	a	a	b	a	b	a
b	b	a	a	a	a	b	a	b	a
b	b	a	a	a	a	-	a	b	a
b	b	a	a	a	a	a	a	b	a
b	b	a	a	a	a	a	a	b	a
b	b	a	a	a	a	a	a	b	b
b	b	a	a	a	a	a	a	b	b
b	b	a	a	a	a	a	a	b	a
b	b	b	a	a	a	a	a	b	a
b	b	b	a	a	a	a	a	b	a
b	b	b	a	a	a	a	a	b	a
b	b	b	a	a	a	a	a	b	b
b	b	b	a	a	a	a	a	b	a
b	b	-	a	a	a	a	a	b	a
b	-	b	a	a	a	a	a	b	a
b	b	a	a	a	a	a	a	b	a
b	b	a	a	a	a	a	a	b	a
b	-	a	a	a	a	a	a	b	a
b	b	a	a	a	a	a	a	b	a
b	b	-	a	a	a	a	a	b	a
b	b	a	a	a	a	a	a	b	a
b	b	a	a	a	a	a	a	b	a
b	b	a	a	a	a	a	a	b	a
b	b	a	a	a	a	a	a	b	a
b	b	a	a	a	a	a	a	b	a
b	b	a	a	a	a	a	a	b	a
b	b	a	a	a	a	a	a	b	a
b	-	a	a	a	a	a	a	b	a
b	b	a	a	a	a	a	a	b	a
b	b	a	a	a	a	a	a	b	a
b	b	a	a	a	a	a	b	-	a
b	b	-	a	a	a	a	b	b	a
b	b	a	a	a	a	a	b	b	a
b	b	a	a	a	b	a	b	b	a
b	b	a	a	a	b	a	b	b	a
b	b	a	a	a	b	a	b	b	a
b	b	a	a	a	b	a	b	b	a
b	b	a	a	a	b	a	b	b	a
b	b	a	a	a	b	a	b	b	a
b	b	a	a	a	b	a	b	b	a
b	-	a	a	a	-	b	b	b	a
b	b	a	a	a	b	b	b	b	a
b	b	-	a	a	b	b	b	b	a

-	b	a	a	a	b	b	b	b	a
a	b	a	a	a	b	b	b	b	a
a	b	a	a	a	b	b	b	b	a
-	b	-	a	a	b	b	b	b	a
a	b	a	a	a	b	b	b	b	a
a	b	a	a	a	b	b	b	b	a
a	b	a	a	a	b	b	b	b	a
a	b	a	a	a	b	b	b	b	a
a	b	a	a	a	b	b	b	b	a
a	b	a	a	a	b	b	b	b	a
b	b	a	a	a	b	b	b	a	a
b	b	a	b	a	b	a	a	a	a
b	b	a	b	a	b	a	a	a	b
b	b	a	b	a	b	a	a	a	b
b	b	a	b	a	b	a	a	a	a
b	b	a	b	a	b	a	a	a	a
b	b	a	b	a	b	a	a	a	a
b	b	a	b	a	b	a	a	a	a
b	b	a	b	a	b	a	a	a	a
b	b	a	b	a	b	a	a	a	a
b	b	a	b	a	b	a	a	a	a
b	b	a	b	a	b	a	a	a	a
b	b	a	b	a	b	a	a	a	a
b	b	a	b	a	b	a	a	a	a
b	b	a	b	a	b	a	a	a	a
b	b	a	b	a	b	a	a	a	a
b	b	a	b	a	b	a	a	a	a
b	b	a	b	a	b	a	a	a	a
b	b	a	b	a	b	a	a	a	-
b	b	a	b	a	b	a	a	a	a
b	b	a	b	a	b	a	a	a	a
b	b	a	b	a	b	a	a	a	-
b	b	a	b	a	b	a	a	a	-
b	b	a	b	a	b	a	a	a	-
b	b	a	b	a	b	a	a	a	a
b	b	a	b	a	b	a	a	a	a
b	b	a	b	a	b	a	a	a	a
b	b	a	b	a	b	a	a	a	a
-	b	a	b	a	b	a	a	a	a
a	b	a	b	a	b	a	a	a	a
a	-	a	b	a	b	a	a	a	a
a	b	a	b	a	b	a	a	a	a
a	b	a	b	a	b	a	a	a	-
a	b	a	b	a	b	a	a	a	a
a	b	a	a	a	b	a	a	a	a
a	b	a	a	a	b	a	a	a	a
a	b	a	a	a	b	a	a	a	a

a	b	a	a	a	b	a	a	a	a
a	b	a	a	a	b	a	a	a	a
a	b	a	a	a	b	a	a	a	a
a	b	a	a	a	b	a	a	a	b
a	b	a	a	a	b	a	a	a	b
a	b	a	a	a	b	a	a	b	a
a	b	a	a	a	b	a	a	b	a
a	b	a	a	a	b	a	a	b	a
a	b	a	a	a	b	a	a	b	a
a	b	a	a	a	b	b	a	b	a
a	b	a	a	a	b	b	a	b	a
a	b	a	a	a	b	b	a	b	a
a	b	a	a	a	b	b	a	b	b
a	b	a	a	a	b	b	a	b	a
a	b	a	a	a	b	b	a	b	a
a	b	a	a	a	b	b	a	b	a
a	b	a	a	a	b	b	a	b	a
a	b	a	a	a	b	b	a	b	a
a	b	a	a	a	b	b	a	a	a
a	b	a	a	a	b	b	a	a	a
a	b	a	a	a	b	b	a	a	a
a	b	a	a	a	b	b	a	a	a
a	b	a	a	a	b	b	a	a	a
a	b	a	a	a	b	b	a	a	a
a	a	a	a	a	b	b	a	a	-
a	a	-	a	a	b	b	a	a	b
a	a	a	a	a	b	b	a	a	a
a	a	a	a	a	b	b	a	a	a
a	a	a	a	a	b	b	a	a	a
a	a	a	a	a	b	b	a	a	a
a	a	a	a	a	b	b	a	a	a
a	a	a	a	a	b	b	a	a	a
a	a	a	a	a	b	b	a	a	a
a	a	a	a	a	b	b	a	a	a
a	a	a	a	a	b	b	a	a	a
-	a	a	a	a	b	b	a	a	a
a	a	a	a	a	b	b	a	a	a
a	a	a	a	a	b	b	a	a	a
a	a	a	a	a	b	b	a	a	a
a	a	a	a	a	b	b	a	a	a
b	a	a	a	a	b	b	a	a	a
b	a	a	a	a	b	b	a	a	a
b	a	a	a	a	b	b	a	a	a
b	-	a	a	a	b	b	a	a	a
-	a	a	a	a	-	b	a	a	a
b	a	a	a	a	b	b	a	a	a
b	a	a	a	a	b	b	a	a	a

b	a	a	a	a	b	b	a	a	a
b	a	a	a	a	b	b	a	a	a
b	a	b	a	a	b	b	a	a	a
b	a	-	a	a	b	b	a	a	a
b	a	b	a	a	b	b	a	a	a
b	a	b	a	a	b	b	a	a	b
b	a	b	a	a	b	b	a	a	a
b	a	b	a	a	b	b	a	a	a
b	a	b	a	a	b	b	a	a	a
b	a	b	a	a	b	b	a	a	a
b	a	-	a	a	b	b	a	a	a
b	a	b	a	a	b	b	a	a	a
b	a	b	a	a	b	b	a	a	a
b	a	b	a	a	b	b	a	a	a
b	a	b	a	a	b	b	a	a	a
b	a	b	a	a	b	b	a	a	a
-	a	b	a	a	b	b	a	a	a
b	a	b	a	a	b	b	a	a	a
b	a	b	a	a	b	b	a	a	a
b	a	b	a	a	b	b	a	a	a
b	a	b	a	a	b	b	a	a	a
b	a	b	b	a	a	b	a	a	a
b	a	a	a	a	a	b	b	b	a
b	a	a	a	a	a	b	b	b	b
b	a	a	a	a	a	b	b	b	a
b	a	a	a	a	a	b	b	b	a
b	a	a	a	a	a	b	b	b	a
b	a	a	a	a	-	b	b	b	a
b	a	a	a	a	a	b	b	b	a
b	a	-	a	a	a	b	b	b	b
b	a	a	a	a	a	b	b	b	a
b	a	a	a	a	a	b	b	b	a
b	a	a	a	a	a	b	b	b	a
b	a	a	a	a	a	b	-	b	a
b	a	-	a	a	a	b	b	b	a
b	a	a	a	a	a	b	b	b	b
b	a	a	a	a	a	b	b	b	-
b	-	a	a	a	a	b	b	b	b
b	a	a	a	a	a	b	b	b	b
b	a	a	a	a	b	b	b	b	b
b	a	a	a	a	b	b	b	b	b
b	a	a	a	a	b	b	b	b	b
b	a	a	a	a	b	b	b	b	b
b	a	a	a	a	b	b	b	b	b
b	a	a	a	a	b	b	b	b	b
b	a	a	a	a	b	a	b	b	b
b	a	a	b	a	b	a	b	b	b

b	a	b	b	a	b	a	b	a	b
b	a	b	b	a	b	a	b	a	b
b	-	b	b	a	b	a	b	a	b
a	a	a	b	a	-	a	b	a	b
a	a	b	b	a	b	a	b	a	b
a	a	b	b	a	b	a	b	a	b
a	a	-	b	a	b	a	b	a	b
a	a	-	b	a	b	a	b	a	b
a	a	b	b	a	b	a	b	a	b
a	a	b	b	a	b	a	b	a	b
a	a	b	b	a	b	a	b	a	b
a	a	b	b	a	b	a	b	a	b
a	a	b	b	a	b	a	b	a	b
a	a	b	b	a	b	a	b	a	-
a	a	b	b	a	b	a	b	a	b
a	a	b	b	a	b	a	b	a	b
a	a	b	b	a	b	a	b	a	b
a	-	b	b	a	b	a	b	a	b
a	a	b	b	a	b	a	b	a	b
a	a	b	b	b	b	a	b	a	b
a	a	b	b	b	b	a	b	a	b
a	a	b	b	b	b	a	b	a	b
a	a	b	b	b	b	a	b	a	b
a	a	b	b	b	b	a	b	a	b
a	a	b	b	b	b	a	b	a	b
a	a	b	b	b	b	a	b	a	b
a	a	b	b	b	b	a	b	a	b
a	a	b	b	b	b	a	b	b	b
a	a	b	b	b	b	a	b	b	b
a	a	b	b	b	b	a	b	b	b
a	a	b	b	b	b	a	b	b	b
a	a	-	b	b	b	a	b	b	b
a	a	b	b	b	b	a	b	b	b
a	a	b	b	b	b	a	a	b	b
a	a	b	b	b	b	a	a	b	b
a	a	b	b	b	b	b	a	b	b
a	a	b	b	b	b	b	a	b	b
a	a	b	b	b	b	b	a	b	b
a	a	b	b	b	b	b	a	b	b
a	a	b	b	b	b	b	a	b	b
a	a	b	b	b	b	b	a	b	b
a	a	b	b	b	b	b	a	b	b
a	a	b	b	b	b	b	a	b	b
a	a	-	b	b	b	b	a	b	b
-	a	b	b	b	b	b	a	b	b
a	a	b	b	b	b	b	a	b	b
a	a	b	b	b	b	b	a	b	b

a	a	b	b	b	b	b	a	b	b
a	a	b	b	b	b	b	a	b	b
a	a	b	b	b	b	b	a	b	b
a	a	b	b	b	b	b	a	b	b
a	a	b	b	b	b	b	a	b	b
a	a	b	b	b	b	b	a	b	b
a	a	b	b	b	b	b	a	b	b
b	a	b	b	b	b	b	a	b	b
b	a	b	b	b	b	b	a	b	b
b	a	b	b	b	b	b	a	b	b
b	a	b	b	b	b	b	a	b	b
b	a	b	b	b	b	b	a	b	b
-	a	b	b	b	b	b	a	b	b
b	a	b	b	b	b	b	a	b	b
b	a	b	b	b	b	b	a	b	b
b	a	b	b	b	b	b	a	b	b
b	a	b	b	b	b	b	a	b	b
b	a	b	b	b	b	b	a	b	b
b	a	b	b	b	b	b	a	b	b
b	a	-	b	b	b	b	b	b	b
b	a	b	b	b	b	b	b	b	b
b	a	b	b	b	b	b	b	b	-
b	a	b	b	b	b	b	b	b	b
a	a	a	b	b	b	b	b	b	b
b	a	b	b	b	b	b	b	b	b
b	a	b	b	b	b	b	b	b	b
b	a	b	b	b	b	b	b	b	b
b	a	b	b	b	b	b	b	b	b
b	a	b	b	b	b	b	b	b	b
b	a	b	b	b	b	b	b	b	b
-	a	b	b	b	b	b	b	b	b
b	a	b	b	b	b	b	b	b	b
b	a	b	b	b	b	b	b	b	b
b	a	b	b	b	b	b	b	b	b
b	a	b	b	b	b	b	b	b	b
b	a	b	b	b	b	b	b	b	b
b	a	b	b	b	b	b	b	b	b
b	a	b	b	b	b	b	b	b	b
b	a	b	b	b	b	b	b	b	b
-	a	b	b	b	b	b	b	b	b
b	a	b	b	b	b	b	b	b	b
-	a	b	b	b	b	b	b	b	b
b	a	b	b	b	b	b	b	b	a
b	-	b	b	b	b	b	b	b	b
b	a	b	b	b	b	b	b	b	b
b	a	b	b	b	b	b	b	b	b
b	a	b	b	b	b	b	b	b	a

b	a	b	b	b	b	b	b	b	b
b	a	b	b	b	b	b	b	-	a
b	a	a	b	b	b	b	b	b	b
b	a	b	b	b	b	b	b	a	b
b	a	b	b	b	b	b	b	a	b
b	a	b	b	b	b	b	b	a	b
b	a	b	b	b	b	b	b	a	b
b	a	b	b	b	b	b	b	a	b
b	a	b	b	b	b	b	b	a	-
b	a	b	b	b	b	b	b	a	-
b	a	b	b	b	b	b	b	a	b
b	a	-	b	b	b	b	b	a	b
b	a	b	b	b	b	b	b	a	b
b	a	b	b	b	b	b	b	a	b
b	a	b	b	b	b	b	b	a	b
b	a	-	b	b	b	b	b	a	b
b	a	b	b	b	b	b	b	a	b
b	a	b	b	b	b	b	b	a	b
b	a	b	b	b	b	b	b	a	b
b	b	b	b	b	b	b	b	a	-
b	b	b	b	b	b	b	b	a	b
b	b	b	b	b	a	b	b	a	b
b	b	b	b	b	b	b	b	a	b
b	b	b	b	b	b	b	b	a	b
a	b	b	b	b	b	b	b	b	b
a	b	b	b	b	b	b	b	b	b
a	b	b	b	b	b	b	b	b	-
a	b	b	b	b	b	b	b	b	-
a	b	b	b	b	b	b	b	b	b
a	b	b	b	b	b	b	b	b	b
a	b	b	b	b	b	b	b	b	b
a	b	b	b	b	b	b	b	b	b
a	b	b	b	b	b	b	b	b	b
a	b	b	b	b	b	b	b	b	b
a	b	b	b	b	b	b	b	b	b
a	b	b	b	b	b	b	b	a	b
a	b	b	a	b	-	b	b	a	b
a	b	b	b	a	b	b	b	b	b
b	b	b	b	b	a	b	b	b	a
b	b	b	b	b	a	b	b	b	a
b	b	b	b	b	b	b	b	b	b
b	b	b	b	b	b	b	b	b	a
b	b	b	b	b	b	b	b	b	a
b	b	b	b	b	b	b	b	b	a
-	b	b	b	b	b	b	b	b	a
b	b	b	b	b	b	b	a	b	a
b	b	b	b	b	b	b	a	b	b

b	b	b	b	b	b	b	a	b	a
b	b	b	b	b	b	b	a	b	a
b	b	b	b	b	b	b	a	b	a
b	b	b	b	b	b	b	a	b	a
b	a	a	b	a	a	b	b	b	a
-	a	a	b	a	a	b	b	b	b
b	a	a	b	a	a	b	b	b	b
-	a	a	b	a	a	b	b	b	b
b	a	a	b	a	a	b	b	b	b
b	a	a	b	a	a	b	a	b	b
b	a	a	b	a	a	b	a	b	b
b	a	a	b	a	a	b	a	b	-
b	a	a	b	b	a	b	a	b	b
b	a	a	b	b	a	b	a	b	b
b	a	a	b	b	a	b	a	b	b
b	a	a	b	b	a	b	a	b	b
b	a	a	b	b	a	b	a	b	b
b	a	a	b	b	a	b	a	b	b
b	a	-	b	b	a	b	a	b	b
b	a	a	b	b	a	b	a	b	b
b	a	a	b	b	a	b	a	b	b
b	a	-	b	b	b	b	a	b	b
b	a	a	b	b	b	b	a	b	b
b	a	a	b	b	b	b	a	b	b
a	a	a	b	b	b	b	a	b	b
a	a	a	a	a	b	b	b	b	b
a	a	a	a	a	b	b	b	b	b
-	a	a	a	a	b	b	b	b	b
a	a	a	a	a	-	b	b	b	b
a	b	a	a	a	b	b	b	b	b
a	b	a	a	a	b	b	b	b	b
a	b	a	a	a	b	b	b	b	b
a	b	a	a	a	b	b	b	b	b
a	b	a	a	a	b	b	b	b	b
a	b	a	a	a	b	b	b	b	b
a	b	a	a	a	b	b	b	b	-
a	b	a	a	a	b	b	b	b	b
a	b	a	a	a	b	b	b	b	-
a	b	a	a	a	b	b	b	b	b
a	b	-	a	a	b	b	b	b	a
a	b	a	a	a	b	b	b	b	a
a	b	a	b	b	b	b	b	a	a
a	b	a	b	b	b	b	b	a	a
a	b	a	b	b	b	b	b	a	a
a	-	a	b	b	b	b	b	a	a
a	b	a	b	a	b	a	a	b	b
a	b	a	b	a	b	a	a	b	b

a	b	-	b	a	b	a	-	b	b
a	b	a	b	a	b	a	a	b	b
a	b	a	b	a	b	a	a	b	b
a	b	a	b	a	b	a	a	b	b
a	b	a	b	a	b	a	a	b	b
a	b	a	b	a	b	a	a	b	b
a	b	a	b	a	b	a	a	b	b
a	b	a	b	a	b	a	b	b	b
a	b	a	b	a	b	a	b	b	b
a	b	-	b	a	b	a	b	b	b
a	b	a	b	a	b	a	b	b	b
a	b	a	b	a	b	a	b	b	b
a	b	a	b	a	b	a	b	b	b
b	b	a	b	a	b	a	b	b	b
b	b	a	b	b	b	a	b	b	b
b	b	a	b	b	b	a	b	b	b
b	b	a	b	b	b	a	b	b	b
b	b	a	b	b	b	a	b	b	b
b	b	a	b	b	b	a	b	b	b
-	b	a	b	b	a	a	b	b	b
b	b	a	b	b	b	a	b	b	b
b	b	a	b	b	b	a	b	b	b
b	b	a	b	b	b	a	b	b	b
b	b	a	b	b	b	a	b	b	b
b	b	a	b	b	b	a	b	b	b
b	b	a	b	b	-	a	b	b	-
a	b	a	b	b	b	a	b	b	b
a	b	a	b	b	b	a	b	b	b
a	b	a	b	b	b	a	b	b	-
a	b	a	b	b	b	a	b	b	b
a	b	a	b	b	b	a	b	b	b
a	b	a	b	b	b	a	b	b	-
a	b	a	b	b	b	a	b	b	b
a	b	a	b	b	a	a	b	b	b
a	b	a	b	b	a	a	b	b	b
a	b	a	b	b	a	a	b	b	b
a	b	a	b	b	-	a	b	b	b
-	b	a	b	b	a	a	b	b	b
a	b	a	b	b	a	a	b	b	b
a	b	a	b	b	a	a	b	b	b
a	b	a	b	b	a	a	b	b	b
a	b	a	b	b	a	a	b	b	b
a	-	a	b	b	a	a	b	b	b
a	b	a	b	b	a	a	b	b	b
a	b	a	b	b	a	a	b	b	b
a	b	a	b	b	a	a	b	b	b
a	b	a	b	b	a	a	b	b	b

a	b	a	b	b	a	a	b	b	b
a	-	a	b	b	a	a	b	b	b
a	b	a	b	b	a	a	b	b	b
a	b	a	b	b	a	a	b	b	b
a	b	a	-	b	a	a	b	b	b
a	b	a	b	b	a	a	b	b	b
a	b	a	b	b	a	a	b	b	b
a	b	a	b	b	a	a	b	b	a
a	b	a	b	b	a	a	b	b	b
a	-	a	b	b	a	a	b	b	b
a	b	a	b	-	a	a	b	b	b
a	b	a	b	b	a	a	b	b	b
a	b	-	b	b	a	a	b	b	-
a	b	a	b	b	a	a	b	b	a
a	b	a	b	b	a	a	b	b	b
a	b	a	b	b	a	a	b	b	-
a	b	a	b	b	a	a	b	b	a
a	b	a	b	b	a	a	b	b	a
a	b	b	b	b	a	a	b	b	a
a	b	b	b	b	a	a	b	b	a
a	b	b	b	b	a	b	b	b	a
b	b	b	b	b	a	b	b	b	-
b	b	b	b	b	a	b	b	b	a
b	b	b	b	b	a	b	b	b	a
b	b	b	b	b	a	b	b	b	a
b	b	b	b	b	a	b	b	b	-
b	b	b	b	b	a	b	b	b	a
b	b	b	b	b	a	b	b	b	a
b	b	-	b	b	a	b	b	b	a
b	b	b	b	b	a	b	b	b	a
b	b	b	b	b	a	b	b	b	a
b	b	-	b	b	a	b	b	b	a
b	b	b	b	b	a	b	b	b	a
b	b	b	b	b	a	b	b	b	a
b	b	b	b	b	-	b	b	b	a
b	b	b	b	b	b	b	b	b	a
b	b	b	b	b	b	b	b	b	a
b	b	b	b	b	b	b	b	b	a
b	b	b	b	b	b	b	b	b	a
b	b	b	b	b	b	b	b	b	a
b	b	b	b	b	b	b	b	b	a
b	b	b	b	b	b	b	b	b	a
a	b	b	b	a	a	b	a	b	a
a	b	-	b	a	a	b	a	b	a
-	b	b	b	a	-	b	-	b	a
a	b	b	b	a	a	b	a	b	a
a	b	b	b	a	a	b	a	b	b

a	b	b	b	a	a	b	a	b	a
a	b	-	b	a	a	b	a	b	a
a	b	b	b	a	a	b	a	b	a
a	b	b	a	a	a	b	a	b	a
a	b	a	a	a	a	b	a	b	a
a	b	a	a	a	a	b	b	b	b
-	b	a	a	a	a	b	a	b	a
a	b	a	a	a	a	b	a	b	a
b	b	b	b	b	a	a	a	b	a
b	b	b	b	b	a	a	a	b	a
b	b	b	b	b	a	a	a	b	a
b	b	b	b	b	a	a	a	b	a
b	b	b	b	b	a	a	a	b	a
b	b	b	b	b	a	a	a	b	a
b	b	b	b	b	a	a	b	b	a
-	b	b	b	b	a	a	b	b	a
b	b	b	b	b	a	a	b	b	a
b	b	b	b	b	a	a	b	b	a
b	b	b	b	b	a	a	b	b	a
b	b	b	b	b	a	a	b	b	a
b	b	b	b	b	a	a	b	b	a
b	b	-	b	b	a	a	b	b	a
b	b	-	b	b	a	a	b	b	a
b	a	a	b	b	a	a	b	b	-
b	a	a	b	b	a	a	b	b	a
b	a	a	b	b	a	a	b	b	a
b	a	a	b	b	a	a	b	b	a
b	a	a	b	b	a	a	b	b	a
b	a	a	b	b	a	a	b	b	a
a	a	a	b	b	a	a	b	b	a
a	b	a	a	a	a	a	b	a	a
a	b	a	a	-	a	a	b	a	a
a	b	a	a	a	a	a	b	a	a
a	b	a	a	a	a	a	b	a	-
a	b	a	a	a	a	a	b	a	a
a	b	b	a	a	a	a	b	a	a
a	b	-	a	a	a	a	b	a	a
a	b	b	a	a	a	a	b	a	a
a	b	b	a	b	a	a	b	a	a
a	-	b	a	b	a	a	b	a	a
a	b	b	a	b	a	a	b	a	a
a	b	b	a	b	a	a	b	a	a
a	b	b	a	b	a	a	b	a	a
-	a	a	a	b	a	b	b	a	a
b	b	a	a	b	a	b	b	a	a
b	a	a	a	b	a	b	b	a	a
b	a	a	a	b	a	b	b	a	a

b	b	a	a	b	a	b	b	a	a
b	a	a	a	b	a	b	b	a	a
b	a	a	a	b	a	b	b	a	a
b	a	a	a	b	a	b	b	a	a
a	a	a	a	b	a	b	b	a	a
b	a	a	a	b	a	b	a	a	a
b	a	-	a	b	a	b	a	a	a
b	a	a	a	b	a	b	a	a	a
b	a	a	a	b	a	b	a	a	a
b	a	a	a	b	a	b	a	a	a
b	a	a	a	b	a	b	a	a	a
b	a	b	a	b	-	b	a	a	a
a	a	b	a	b	a	b	a	b	b
a	a	b	a	b	a	b	a	b	a
a	a	b	a	b	a	b	a	b	a
a	a	b	a	b	a	b	b	b	a
a	a	b	a	b	b	b	b	b	a
a	a	b	a	b	b	b	b	b	a
a	a	b	a	b	b	b	b	b	-
a	a	b	a	b	b	b	b	b	b
a	a	b	a	b	b	b	b	b	b
a	a	b	a	b	b	b	b	b	b
b	b	b	a	a	b	b	b	b	b
a	b	b	a	a	b	b	b	b	b
a	b	b	a	a	b	b	b	b	b
a	b	b	a	a	b	b	b	b	b
a	b	b	a	a	b	b	b	b	b
a	b	b	a	b	b	b	b	b	b
a	b	b	a	b	b	b	b	b	b
a	b	b	b	b	b	b	b	b	b
a	b	b	b	b	b	b	b	b	b
a	b	b	b	b	b	b	b	b	b
a	b	b	b	b	b	b	b	b	b
a	b	a	b	b	b	b	b	b	b
a	b	a	b	b	b	b	b	b	-
b	b	a	b	b	b	b	b	b	b
b	b	a	b	b	b	b	b	b	b
b	b	a	-	b	b	b	b	b	b
b	b	a	b	b	a	b	b	b	b
b	b	a	b	b	a	b	b	b	b
b	b	a	b	b	b	b	b	b	b
b	b	-	b	b	b	b	b	b	b
b	b	a	b	-	b	b	b	b	b
b	b	a	b	b	b	b	b	b	b
b	b	a	b	b	b	b	b	b	-
a	b	a	b	b	b	b	b	a	b
a	b	a	b	b	b	b	b	a	b

a	b	a	b	b	b	b	-	a	b
-	b	a	b	b	b	b	b	a	b
a	b	a	b	b	b	b	b	a	b
a	b	a	b	b	b	b	b	a	b
a	b	a	b	b	b	b	b	a	b
a	a	a	b	b	b	b	b	a	b
a	a	a	b	b	b	a	b	a	b
a	a	a	b	b	b	a	b	a	b
a	a	a	a	a	b	b	b	a	a
a	a	a	a	a	b	b	b	a	a
a	a	a	a	a	b	b	b	a	a
a	a	a	a	a	b	b	b	a	a
a	a	a	a	a	b	b	b	a	-
a	a	a	a	a	b	b	b	a	a
a	a	a	a	a	b	b	b	a	-
a	a	a	b	a	b	b	b	a	a
a	a	a	b	a	b	b	b	a	a
a	a	a	b	a	b	b	b	a	a
a	a	a	b	a	b	b	b	a	a
a	a	a	b	a	b	b	b	a	a
a	a	a	b	a	b	b	b	a	a
a	a	a	b	a	b	b	b	a	a
a	a	a	b	a	b	b	b	a	a
a	a	-	b	a	b	b	b	a	-
a	a	a	b	a	b	b	b	a	a
a	a	a	b	a	b	b	b	a	a
a	a	a	b	a	b	b	b	a	a
a	a	a	b	a	b	b	b	a	a
a	a	a	b	a	b	b	b	a	a
-	a	a	b	a	b	b	b	b	a
a	a	a	b	a	b	b	b	b	a
a	a	a	b	a	-	b	b	b	a
a	a	a	b	a	b	b	b	b	a
a	a	a	b	a	b	b	b	b	a
a	b	a	b	a	b	b	b	b	a
a	b	a	b	a	b	b	b	b	a
a	b	a	b	a	b	b	b	b	b
a	b	a	b	a	b	b	b	b	a
a	b	a	b	a	b	b	b	b	a
a	b	a	b	a	b	b	b	b	a
a	b	b	b	a	b	b	b	b	a
b	b	b	b	a	b	a	b	b	a
b	b	b	b	a	b	a	b	b	b
b	b	b	b	a	b	a	b	b	a
b	b	b	b	a	b	a	b	b	a
b	-	b	b	a	b	a	b	b	a
b	b	b	b	a	-	a	b	b	a

b	b	b	b	a	b	a	b	b	a
b	b	b	b	a	b	a	b	b	a
b	b	b	b	a	b	a	b	b	a
b	b	b	b	a	b	a	b	b	a
b	b	b	b	a	b	a	b	b	a
b	b	b	b	a	b	a	b	b	a
a	b	b	b	a	b	b	b	b	a
a	b	b	b	a	b	b	b	b	a
a	b	b	b	a	b	b	b	b	a
a	b	b	b	a	b	b	b	b	a
-	b	b	b	a	b	b	b	b	a
a	b	b	b	a	b	b	b	b	a
a	b	b	b	a	b	b	b	b	a
a	b	b	b	a	b	b	b	b	a
a	b	b	b	a	b	-	b	b	a
a	b	b	b	a	b	b	b	b	a
a	b	b	b	a	b	b	b	b	-
a	b	b	b	a	b	b	b	b	a
-	b	b	b	a	a	b	b	b	a
a	b	b	b	a	-	b	b	b	a
b	-	-	a	b	a	a	b	a	b
b	a	a	a	b	a	a	b	a	b
b	a	a	a	b	a	a	b	a	b
b	a	a	a	b	a	a	b	a	b
b	a	a	a	b	a	a	b	a	b
b	a	a	a	b	a	a	b	a	b
b	a	a	a	b	a	a	b	a	b
b	a	a	a	b	a	a	b	a	b
b	a	a	a	b	a	a	b	a	b
b	a	a	a	b	a	b	b	a	b
b	a	a	a	b	a	b	b	a	b
b	a	a	a	b	a	b	b	a	b
b	a	a	-	b	a	b	b	a	b
b	b	a	a	b	a	b	b	a	b
b	a	a	a	b	a	b	b	a	b
b	a	a	a	b	a	b	b	a	b
a	a	a	b	b	a	b	b	a	b
a	a	a	b	b	a	b	b	a	b
a	a	a	b	b	a	b	b	a	a
a	a	a	b	b	a	b	b	a	b
a	a	a	b	b	a	b	b	a	b
a	a	a	b	b	a	b	b	a	b
a	a	-	b	b	a	b	b	a	b
b	a	b	b	a	a	b	b	a	b
b	a	b	b	a	a	b	b	a	b
b	a	b	b	a	a	b	b	a	b
b	a	b	b	a	a	b	a	a	b

b	a	b	b	a	a	b	a	a	b
b	a	b	b	a	a	b	a	a	b
-	a	b	b	a	a	b	a	a	a
b	a	b	b	a	b	b	a	a	b
b	a	b	b	a	b	b	a	a	a
b	a	-	b	b	b	b	a	a	a
b	a	b	b	b	b	b	a	a	a
b	a	b	b	b	b	b	a	a	-
b	a	b	b	b	b	b	a	a	a
b	a	-	b	b	b	a	a	a	a
b	-	b	b	b	b	a	a	a	b
b	a	b	b	b	b	a	a	a	a
b	a	b	b	b	b	a	a	a	a
b	a	b	b	b	b	a	a	a	a
b	a	b	b	b	b	a	a	a	a
b	a	b	b	a	a	a	a	a	a
b	a	b	b	a	b	a	a	a	-
-	a	b	b	a	b	a	a	a	a
b	-	b	b	a	b	a	a	a	a
b	b	b	b	a	b	a	a	a	a
a	b	b	b	a	b	a	a	a	a
a	b	b	b	a	b	a	a	a	a
a	b	b	b	a	b	a	a	b	a
a	b	-	b	a	-	a	a	-	a
a	b	b	b	a	b	a	a	b	a
a	b	b	b	a	b	a	a	b	a
a	b	b	b	a	b	a	a	b	a
a	b	b	b	a	b	a	-	b	-
a	b	-	b	a	b	a	a	b	a
a	b	b	b	a	-	a	a	b	a
a	b	b	b	a	b	a	a	b	a
a	b	b	b	a	b	a	a	b	a
a	b	b	b	a	b	a	a	b	a
a	b	b	b	a	b	a	a	a	a
a	b	b	b	a	b	a	a	a	b
a	b	a	b	a	b	b	b	a	a
-	b	-	b	a	b	b	b	a	a
a	b	a	b	a	b	b	b	a	a
a	b	a	b	a	b	b	-	a	a
-	b	a	b	a	b	b	b	a	a
a	b	a	b	a	b	b	a	a	-
a	b	a	b	a	b	b	a	a	a
a	b	a	b	a	b	b	a	a	-
-	b	a	b	a	b	b	a	a	-
a	b	a	b	a	b	b	a	a	a
a	-	-	b	a	b	b	a	a	a
a	b	b	a	b	b	b	a	a	a

a	b	b	b	a	-	b	a	a	b
a	b	b	b	a	b	b	a	a	b
a	b	b	b	a	b	b	a	a	b
a	b	b	b	a	b	b	a	a	b
a	b	b	b	b	b	b	a	a	-
a	b	b	b	b	b	b	a	a	b
a	b	b	b	b	b	b	a	a	-
a	b	b	b	b	b	b	a	a	b
a	-	b	b	b	b	b	a	a	b
a	b	b	b	b	b	b	a	a	b
-	a	b	b	b	b	b	a	a	b
a	a	b	b	b	b	b	a	a	b
a	-	b	b	b	b	b	a	a	b
a	a	b	b	b	b	b	a	a	b
a	b	b	b	b	b	b	a	a	-
a	b	b	b	b	b	b	a	a	b
a	b	b	b	b	b	b	a	a	b
a	b	b	b	b	b	b	a	a	-
a	b	b	b	b	b	b	a	a	b
a	b	-	b	b	b	b	a	a	b
a	b	b	b	b	b	b	b	b	b
a	b	b	b	b	b	b	b	b	b
b	b	b	b	b	b	b	b	b	b
b	b	b	b	b	b	b	b	b	-
-	b	b	b	b	b	b	b	b	b
b	b	b	b	b	b	b	b	b	b
b	b	b	b	b	b	b	b	b	b
b	b	b	b	b	b	b	b	b	b
b	b	b	b	b	b	b	b	b	b
b	b	b	b	b	b	b	b	b	b
b	b	b	b	b	b	b	b	b	b
b	b	b	b	b	b	b	b	b	b
b	b	b	b	b	a	b	b	b	b
b	b	b	b	b	a	b	b	b	b
b	b	b	b	b	a	b	b	b	b
b	b	b	b	b	a	b	b	b	b
b	b	b	b	b	a	b	b	b	b
b	b	b	b	b	a	b	b	b	b
b	b	b	b	b	a	b	b	b	b
b	b	b	b	b	a	a	b	b	b
b	b	b	b	b	a	a	b	b	b
b	b	b	b	b	a	a	b	b	b
b	b	b	b	b	a	a	b	b	b
b	b	b	b	b	a	a	b	b	b
b	b	b	b	b	a	a	b	b	b
b	-	b	b	b	a	a	b	b	a
a	a	b	b	b	b	b	a	a	a

a	a	b	b	b	b	b	a	a	a
a	a	b	b	b	b	b	a	a	a
a	a	b	b	b	b	b	a	a	a
a	a	b	b	b	b	b	a	a	a
a	a	b	b	b	b	b	a	a	a
a	a	b	a	b	b	b	a	a	a
a	a	b	a	b	b	a	a	a	a
a	a	b	a	b	b	a	a	a	a
a	a	b	a	b	b	a	a	a	a
a	a	b	a	b	b	a	a	a	a
a	a	b	a	b	b	a	a	a	b
a	a	b	a	b	b	a	a	a	b
a	a	b	a	b	b	a	a	a	b
a	a	b	a	b	b	b	a	a	b
a	a	b	a	b	b	b	a	a	b
a	a	b	a	b	b	b	a	a	b
a	a	b	a	b	b	b	a	a	b
a	a	b	a	b	b	a	a	a	b
a	a	b	a	b	b	b	a	a	b
a	a	b	a	b	b	b	a	a	b
a	a	b	a	b	b	b	a	a	b
a	a	b	a	b	b	b	a	a	b
a	a	b	a	b	b	b	a	a	b
a	a	b	a	b	b	b	a	a	b
a	a	b	a	b	b	b	a	a	b
a	a	b	a	b	b	b	a	a	b
a	a	b	a	b	b	b	a	a	b
a	a	b	a	b	b	b	a	a	b
a	a	b	a	b	b	b	a	a	b
a	a	b	a	b	b	b	a	a	b
a	a	b	a	b	b	b	a	a	b
a	a	b	a	b	b	b	a	a	b
a	a	b	a	b	b	b	a	a	b
a	a	b	a	b	b	b	a	a	a
a	a	b	a	b	a	b	a	a	b
a	a	b	a	b	a	b	a	a	b
a	a	b	a	b	a	b	a	a	b
a	a	b	a	b	a	b	a	a	b
a	a	b	a	b	a	b	a	a	a
a	a	b	a	b	a	b	a	a	b
b	b	a	b	a	b	a	b	b	a
a	a	a	b	b	a	a	b	a	a
a	a	a	b	b	a	a	b	a	b
a	a	a	b	b	a	a	b	a	b
a	a	a	b	b	a	a	b	a	b
a	a	a	b	b	a	a	b	a	b
a	a	a	b	b	a	a	b	a	b

a	a	a	b	b	a	a	b	a	b
a	a	a	b	b	a	a	b	a	b
a	a	a	b	b	a	a	b	b	b
a	a	a	b	b	a	a	b	b	b
a	a	a	b	b	a	a	b	b	b

YW181	YW182	YW183	YW184	YW185	YW186	YW187
a	b	a	a	b	b	b
a	b	a	a	b	b	b
a	b	-	a	b	b	b
a	b	a	a	b	b	b
a	b	a	a	b	b	b
a	b	a	a	b	b	b
a	b	a	a	b	b	b
a	b	a	a	b	b	b
a	b	a	a	b	b	b
a	-	a	a	b	b	b
a	b	a	a	b	b	b
a	b	a	a	b	b	b
b	b	a	a	b	b	b
b	b	a	a	b	b	b
b	b	a	-	b	b	b
b	b	a	a	b	b	b
b	b	a	a	b	b	b
-	a	a	a	b	b	b
b	b	a	a	b	b	b
b	a	a	a	b	b	b
b	a	a	a	b	b	b
b	a	a	-	-	-	-
b	a	a	a	b	a	b
b	a	a	a	b	a	b
b	a	a	a	b	a	b
b	a	a	a	b	a	b
b	a	-	a	b	a	b
b	a	a	a	b	a	b
b	a	a	a	b	a	b
b	a	a	a	b	a	b
b	a	a	a	b	a	b
b	a	a	-	-	-	-
b	a	-	a	b	a	b
b	a	a	b	b	a	b
b	a	a	b	b	-	b
b	a	-	b	b	a	b
b	a	a	b	b	a	b
b	a	a	b	b	a	b
b	a	a	-	-	-	-
b	a	a	b	b	-	b
b	a	a	b	b	a	b
-	a	a	b	b	a	a
b	a	a	b	b	a	a
b	a	a	b	b	a	a
b	-	a	b	b	a	a

b	a	a	b	b	a	a
b	a	a	b	b	a	a
b	a	a	-	b	a	a
b	-	a	b	b	a	a
b	a	a	b	b	a	-
b	a	a	b	b	a	a
b	a	b	b	b	a	a
b	a	b	b	b	a	a
b	a	b	b	b	a	a
a	a	b	b	b	a	a
a	a	b	b	b	a	a
a	a	-	b	b	b	a
a	a	-	-	b	b	a
a	a	b	b	b	b	a
-	a	b	b	b	b	a
a	a	b	b	b	b	a
a	a	b	a	b	b	a
a	a	b	a	b	b	a
a	a	a	-	-	-	-
a	a	b	a	b	b	a
a	a	b	a	b	b	a
a	a	b	a	a	b	a
a	a	b	a	a	b	a
a	a	b	a	a	b	a
a	a	-	a	a	b	a
a	a	b	a	a	b	a
a	a	b	a	b	b	a
a	a	b	a	a	b	a
-	a	b	a	b	b	a
a	a	b	a	b	b	a
a	a	b	a	b	b	a
a	a	b	a	b	b	a
a	a	b	a	b	b	a
a	a	b	a	b	b	a
a	a	b	a	b	b	a
a	a	b	a	b	b	a
a	a	b	a	b	b	a
a	a	b	a	b	b	a
a	a	b	a	b	b	a
a	a	b	b	b	a	a
a	a	b	b	b	a	a
a	a	b	a	b	a	a
a	a	b	b	b	a	a
-	a	b	b	b	a	a
a	a	b	b	b	a	a
a	a	b	b	b	a	a
a	a	b	b	b	a	a

-	a	b	b	b	a	a
a	a	b	a	b	a	a
a	a	b	a	b	a	a
a	a	b	a	b	a	a
a	a	b	a	b	a	a
a	a	b	a	b	a	a
a	a	b	a	b	a	a
-	a	b	a	b	a	a
a	a	b	a	b	a	a
-	a	b	a	b	a	a
a	a	b	a	b	a	a
a	a	b	a	b	a	a
a	a	b	b	b	a	a
a	a	b	-	b	a	a
a	a	b	b	b	a	a
a	a	b	b	b	a	a
-	a	b	b	b	a	a
b	a	b	b	b	a	a
b	a	b	b	b	a	a
b	a	b	b	b	a	a
b	a	b	b	b	-	a
b	a	b	-	b	a	a
b	a	b	b	b	a	a
b	a	b	-	a	a	a
b	a	b	b	a	a	a
-	a	b	b	a	a	a
b	a	b	b	a	a	a
b	a	b	-	a	a	a
b	a	b	b	a	a	-
b	a	b	b	a	a	a
b	a	b	-	a	a	a
b	a	b	b	a	a	a
b	a	b	b	a	a	a
b	a	b	b	a	a	a
b	a	b	b	a	a	a
b	a	b	b	a	a	a
b	-	b	b	a	a	a
b	a	b	b	a	a	a
b	-	b	b	a	a	a
b	a	a	-	-	-	-
b	a	-	b	a	a	a
b	a	b	b	a	a	a
b	a	b	b	a	a	a
b	a	b	b	a	a	a
b	a	b	b	a	a	a
-	a	b	b	a	a	a
b	a	b	b	a	a	a

b	a	b	b	a	a	a
b	a	a	b	a	a	a
b	a	a	b	a	a	a
b	a	a	b	a	a	a
b	a	a	b	a	a	b
b	a	a	b	a	a	b
b	a	a	b	a	a	b
b	a	a	-	-	-	-
b	a	a	b	a	a	b
b	a	a	b	a	a	b
-	a	a	b	a	a	b
b	a	a	-	-	-	-
b	-	a	b	a	a	b
b	a	a	b	a	a	b
b	a	a	b	a	a	b
b	a	a	b	a	a	b
b	a	a	-	a	a	b
b	a	a	-	a	a	b
b	a	a	b	a	a	b
b	a	a	b	a	a	b
b	-	a	b	a	a	b
b	b	a	b	a	a	b
a	b	a	b	a	a	b
a	b	a	b	a	a	b
-	b	a	a	a	a	b
b	b	a	b	a	a	b
b	-	a	b	a	a	b
b	b	a	-	-	-	-
b	b	a	b	a	a	b
b	b	a	b	a	-	b
b	b	a	b	a	a	b
b	b	a	-	-	-	-
b	b	a	-	a	a	b
b	b	a	-	a	a	b
b	b	a	b	a	a	b
b	b	a	b	a	a	b
b	b	a	b	a	a	b
b	b	a	b	a	a	b
b	b	a	b	a	a	b
b	b	a	b	a	a	b
b	b	a	b	a	a	b
b	b	a	b	a	a	b
b	b	a	b	a	a	b
b	b	a	b	a	a	b
-	-	a	b	a	a	b
a	b	a	b	a	b	b
a	b	a	b	a	b	b

a	b	a	-	a	b	b
-	b	-	b	a	b	b
a	b	b	b	a	b	b
a	b	b	b	a	b	b
a	b	b	b	a	b	b
a	b	b	b	a	b	b
a	b	b	b	a	b	b
a	b	b	-	a	b	b
a	b	b	b	a	b	b
a	b	b	b	a	b	b
a	b	b	b	a	-	b
a	b	b	b	a	b	b
a	b	a	b	a	a	a
a	b	a	b	a	a	a
a	b	a	b	b	a	a
a	b	a	b	b	a	a
a	b	a	b	b	a	a
a	b	a	b	b	a	a
a	b	a	-	b	a	a
a	b	-	b	b	a	a
a	b	a	b	b	a	a
a	b	a	b	b	a	a
a	b	a	b	b	a	a
-	b	a	b	b	a	a
a	b	a	b	b	a	a
a	b	a	b	b	a	a
a	b	a	a	b	a	a
a	b	a	b	b	a	a
a	b	a	b	b	a	a
a	b	-	a	b	a	a
a	b	a	a	b	a	a
a	b	a	a	b	b	a
a	b	a	-	-	-	-
a	b	a	a	b	b	a
a	b	a	a	b	b	a
a	b	a	a	b	b	a
-	b	a	a	b	b	a
a	b	a	a	b	b	a
-	b	a	a	b	b	a
-	b	a	-	b	b	a
a	b	a	a	b	b	a
a	b	a	a	b	b	a
a	a	a	a	b	b	b
a	a	a	a	b	b	b
a	a	a	a	b	b	b
a	a	a	a	b	b	b
a	a	a	a	b	b	b

a	a	a	-	b	b	b
a	a	a	a	b	b	b
a	a	a	a	b	b	b
a	a	-	-	b	b	b
a	a	a	a	b	b	b
a	a	a	a	b	b	b
a	a	a	a	b	b	b
a	a	a	a	b	b	b
a	a	a	a	b	b	b
a	a	a	a	a	b	b
a	a	a	a	a	b	-
a	a	a	a	a	b	b
a	a	a	a	a	b	b
a	a	a	a	a	b	b
a	a	a	a	a	b	b
a	a	a	a	a	b	b
a	a	a	a	a	b	b
a	a	a	a	a	b	b
a	a	a	a	a	b	b
a	a	a	a	a	b	b
a	a	a	a	a	b	b
a	a	a	a	a	b	b
a	a	a	a	a	b	b
a	a	a	-	a	b	b
a	a	a	a	a	b	b
a	a	a	a	a	b	b
a	a	a	a	a	b	b
a	a	a	a	a	b	b
a	a	a	a	a	b	b
a	a	a	a	a	b	b
a	-	a	a	a	b	b
a	a	a	a	a	b	b
b	a	a	a	a	b	b
b	a	a	-	-	-	-
b	a	a	a	a	a	b
b	a	a	a	a	a	b
-	a	a	a	a	a	b
-	a	a	a	a	a	b
b	a	a	a	a	a	b
b	a	a	a	a	a	b
b	a	a	a	a	a	b
b	a	a	-	a	a	b
b	a	a	a	a	a	b
b	-	a	a	a	a	b
b	a	a	a	a	a	b
b	a	a	a	a	a	b
-	a	a	a	a	a	b

b	a	a	a	a	a	b
b	a	a	a	a	a	b
b	b	b	-	-	-	-
b	b	a	a	a	a	b
b	b	a	a	a	a	b
b	b	a	a	a	a	b
b	b	a	a	a	a	b
b	b	a	a	a	a	b
b	b	a	a	a	a	b
b	b	a	a	a	a	b
-	b	a	a	a	a	a
b	-	a	a	a	a	b
b	b	a	a	a	a	b
b	b	-	a	a	a	b
b	b	a	a	a	a	b
b	b	a	a	a	a	a
b	b	a	a	a	a	a
b	b	a	a	a	a	a
b	b	a	a	a	a	a
b	b	a	a	a	a	a
b	b	a	a	a	a	a
b	b	a	a	a	a	a
b	b	a	-	a	a	a
b	b	a	a	a	a	a
b	b	a	a	a	-	a
b	b	a	a	a	a	a
b	b	a	a	a	a	a
b	b	a	a	a	a	a
b	b	a	a	a	a	a
-	b	a	a	a	a	a
b	b	a	a	a	a	a
b	b	a	a	a	a	a
-	b	a	a	a	a	a
b	b	a	a	a	a	a
b	b	a	a	a	a	a
-	b	a	a	a	a	a
b	b	a	a	a	a	a
b	b	a	a	a	a	a
a	a	a	a	b	a	b
a	a	a	a	b	a	b
a	a	a	a	a	a	b
a	a	a	a	a	a	b
a	a	a	a	a	a	b
a	a	a	a	a	a	b
a	a	a	a	a	a	b
a	a	a	a	a	a	b
a	a	a	a	a	a	b

-	a	a	a	a	a	b
a	a	a	a	a	b	b
a	a	a	a	a	b	b
a	a	a	-	a	b	b
a	a	a	a	a	b	b
a	a	a	a	a	b	b
a	a	a	a	a	b	b
a	a	a	a	a	b	b
a	a	a	a	a	b	b
a	a	a	a	a	b	b
a	a	a	a	a	b	b
a	a	a	-	-	-	-
-	a	a	-	a	b	b
a	a	a	a	a	b	b
-	a	a	a	a	b	b
a	a	a	a	a	b	b
a	a	a	b	a	b	b
a	a	a	a	a	b	b
a	a	a	a	a	b	b
a	a	a	a	a	b	b
a	a	a	a	a	b	b
a	b	a	a	a	b	b
a	b	a	-	a	b	b
a	b	a	a	a	b	b
a	b	a	a	a	b	b
a	b	a	a	a	b	b
a	b	a	a	a	b	b
a	b	a	a	a	b	b
a	b	a	a	-	b	b
a	b	a	a	a	b	b
a	b	a	a	a	b	b
a	b	a	a	a	b	b
a	b	a	a	a	b	b
a	b	a	a	a	b	b
a	b	a	a	a	b	b
a	b	a	a	a	b	b
a	-	b	a	a	a	b
a	b	b	a	a	a	b
a	-	b	-	a	a	b
a	a	b	-	-	-	-
a	a	b	a	a	a	b
a	a	b	a	a	a	b
a	a	a	-	-	-	-
a	a	a	a	a	a	b
a	a	a	a	a	a	b
a	a	a	a	a	a	b
a	a	a	b	a	a	b

a	a	a	b	a	a	b
a	a	a	b	a	a	b
a	a	a	b	a	a	b
a	a	a	b	a	a	b
a	a	a	b	a	a	b
a	a	a	b	a	a	b
a	a	a	b	a	a	b
a	a	a	b	a	a	b
a	a	a	-	a	a	b
a	a	a	b	a	a	b
a	a	b	b	a	a	b
a	a	a	b	a	a	b
a	a	a	b	a	a	b
a	a	a	-	-	-	-
a	a	b	b	a	b	b
a	a	b	b	a	b	b
a	a	b	b	a	b	b
a	a	b	b	a	b	b
a	a	b	-	-	-	-
a	a	b	b	a	b	b
a	a	b	-	-	-	-
a	a	b	b	a	b	b
a	a	b	b	a	b	b
a	a	b	b	a	b	b
a	b	b	b	a	b	b
a	b	b	b	a	b	b
a	b	b	b	a	b	b
a	b	-	b	a	b	b
a	b	b	-	a	b	b
a	b	b	-	a	b	b
a	b	b	b	a	b	b
a	b	b	b	a	b	b
a	b	b	b	a	b	b
a	b	b	b	a	b	b
a	b	b	-	a	b	b
a	b	b	b	a	b	b
a	-	b	b	a	b	b
a	b	b	b	a	b	b
a	b	b	b	a	b	a
a	b	b	b	a	b	a
-	a	b	b	a	b	b
b	a	b	-	-	-	-
b	a	b	b	a	b	b
b	a	b	b	a	b	b
b	a	b	b	a	b	b
b	a	b	-	-	-	-
b	a	-	b	a	b	b

-	a	b	b	a	b	b
b	a	b	b	a	b	b
b	a	b	b	a	b	b
b	a	-	b	a	b	b
b	a	b	-	a	b	b
b	a	b	b	a	b	b
b	a	b	b	a	b	b
b	a	b	b	a	b	b
b	a	b	b	a	-	b
b	a	-	b	a	b	b
b	a	b	b	a	b	b
b	a	b	b	a	b	b
b	a	b	b	a	b	b
b	a	b	b	a	b	b
b	a	-	-	a	b	b
b	a	b	b	a	b	b
b	a	b	b	a	b	b
b	a	b	b	a	b	b
b	a	b	b	a	b	b
b	a	b	b	a	b	b
b	a	b	b	a	b	b
b	a	b	b	a	b	b
b	a	b	b	a	b	b
b	a	b	b	a	b	b
b	a	b	b	a	b	b
b	a	b	-	-	-	-
b	a	b	b	a	b	b
b	a	b	b	a	b	b
b	a	-	b	a	b	b
b	a	b	b	a	b	b
b	a	b	-	a	b	b
b	a	b	-	a	b	b
a	a	b	b	a	b	b
b	a	b	-	a	b	b
b	a	b	a	a	-	b
b	a	b	-	a	b	b
b	a	b	b	a	b	b
b	a	b	b	a	b	b
a	a	b	b	a	b	a
a	-	b	b	a	b	a
a	a	b	b	a	b	a
-	a	b	b	a	b	a
a	a	b	b	a	b	a
a	a	b	b	a	b	a
a	a	b	b	a	b	a
a	a	b	b	a	b	a
a	a	b	-	a	b	a

-	-	b	b	a	b	a
a	a	b	b	a	b	a
a	a	b	b	a	b	a
a	b	b	b	a	b	a
a	b	b	b	a	b	a
a	b	b	-	a	b	a
a	b	b	b	a	b	a
a	b	b	-	a	b	a
a	b	b	-	-	-	-
a	b	b	b	a	b	a
a	b	b	b	a	b	a
a	b	a	b	a	b	a
a	b	a	b	a	b	a
a	b	a	b	a	b	a
a	b	a	b	a	b	a
a	b	a	b	a	b	a
-	b	a	b	a	b	a
a	b	a	b	a	b	a
a	b	a	b	a	b	a
a	b	a	b	a	b	a
a	b	a	b	a	b	a
a	b	a	b	a	b	a
-	b	a	b	a	b	a
a	b	-	b	a	b	a
a	b	-	b	a	b	a
a	b	a	b	a	b	a
a	b	a	b	a	b	a
a	b	a	b	a	b	a
a	b	a	b	a	b	a
a	b	a	b	a	b	a
a	b	a	-	a	b	a
a	b	-	b	a	b	a
a	b	a	b	a	b	a
a	b	a	b	a	b	a
-	b	a	b	a	b	a
a	b	-	b	a	b	a
a	b	a	b	a	b	a
a	b	a	b	a	b	b
a	b	a	-	-	-	-
a	b	a	b	a	b	b
a	b	a	b	a	b	b
a	b	a	b	a	b	b
a	b	a	b	a	b	b
a	b	a	b	a	b	b
a	-	a	b	a	b	b
a	b	a	b	a	b	b
a	b	-	b	a	b	b

a	b	a	b	a	b	b
a	b	a	b	a	b	b
-	b	a	b	a	b	b
a	b	a	b	a	b	b
-	b	a	b	a	b	b
a	b	a	b	a	b	b
a	b	a	-	-	-	-
a	b	a	b	a	b	b
a	b	a	b	a	b	b
a	b	a	b	a	b	b
a	b	a	-	-	-	-
a	b	a	b	-	b	b
a	b	a	b	a	b	b
a	b	a	b	a	b	b
a	b	a	b	a	b	b
a	b	a	b	a	b	b
-	b	a	b	a	b	b
a	b	a	b	a	b	b
a	b	a	-	-	-	-
b	b	a	-	a	b	b
b	b	a	b	a	b	b
b	b	a	-	a	b	b
b	b	a	b	a	b	b
b	b	a	b	a	b	b
b	b	a	b	a	b	b
b	b	a	b	a	b	b
b	b	a	b	a	a	b
b	b	a	b	a	b	b
-	b	a	b	a	-	b
b	b	a	b	a	b	b
b	b	a	b	a	b	b
b	b	b	b	a	b	b
b	b	b	-	-	-	-
b	b	b	b	a	b	b
b	b	b	b	a	b	b
b	b	b	b	a	b	b
b	b	b	b	a	b	b
b	b	b	b	a	b	b
b	b	b	b	a	b	b
b	b	b	b	a	b	b
b	b	b	b	a	b	b
b	b	b	b	a	b	b
b	b	b	b	a	b	b
b	b	b	b	a	b	b
b	b	b	b	a	b	b
b	b	b	b	a	b	b
b	b	b	b	a	b	b

b	b	b	b	a	b	b
b	b	b	b	a	b	-
-	b	b	-	a	b	a
b	b	b	-	-	-	-
b	b	b	b	a	b	a
b	b	b	b	a	b	a
b	b	b	b	a	b	a
b	b	b	b	a	b	a
-	b	b	-	a	b	a
b	b	b	b	a	b	a
b	b	-	b	a	b	a
b	b	b	-	-	-	-
-	b	b	b	a	b	a
b	-	b	-	a	b	a
b	b	b	b	a	b	a
b	b	b	b	a	b	a
b	b	b	b	a	b	a
b	b	b	b	a	b	a
b	b	b	-	a	b	a
b	b	b	b	a	b	a
b	b	b	b	-	b	a
b	b	b	b	a	b	a
b	b	b	b	a	b	a
b	b	b	b	a	b	-
b	b	b	b	a	b	a
b	b	b	b	a	b	a
b	b	b	a	a	a	a
b	b	b	a	a	a	a
b	b	-	a	a	a	a
b	b	b	a	a	a	a
b	b	b	a	a	a	a
-	b	b	a	a	a	a
b	b	b	a	a	a	a
b	b	b	a	a	a	a
-	b	b	a	a	a	a
b	b	b	a	a	a	a
b	b	b	a	a	a	a
b	b	b	-	-	-	-
b	-	b	a	a	a	a
-	b	b	a	a	a	a
b	b	-	a	a	a	a
b	b	b	a	a	a	a
b	b	b	a	a	a	a
b	-	b	a	a	a	a
-	b	b	a	a	a	a
b	b	b	a	a	a	a
b	b	b	a	a	a	a

b	b	b	a	a	a	a
b	b	b	a	a	a	a
b	b	b	a	a	a	a
b	b	b	a	a	a	a
b	b	b	a	a	a	a
-	-	b	a	a	a	a
-	b	b	a	a	a	a
b	b	b	-	-	-	-
b	-	b	a	a	b	a
-	-	b	a	a	b	a
b	b	b	a	a	b	a
-	b	b	b	b	b	b
b	b	b	b	b	b	b
b	b	b	b	b	b	b
b	b	b	b	b	b	b
b	b	b	-	-	-	-
-	b	-	b	b	b	b
-	b	-	b	b	b	b
-	b	b	b	b	b	b
-	b	b	-	b	b	b
b	b	b	b	b	b	b
b	b	b	b	b	b	b
b	b	b	b	b	-	b
b	b	-	b	b	b	b
b	b	b	b	b	b	b
b	b	b	b	b	b	b
b	b	b	b	b	b	b
-	b	b	b	b	b	b
b	b	b	b	b	b	b
b	b	b	b	b	b	b
b	b	b	b	b	b	b
b	b	b	b	b	b	b
b	b	b	b	b	b	b
b	b	b	b	b	b	b
b	b	b	b	b	b	b
b	b	-	-	b	b	b
b	b	b	-	b	b	b
b	b	b	b	b	b	b
b	b	b	b	b	b	b
b	b	b	b	b	b	b
b	b	b	b	b	b	b
b	b	b	b	b	b	b
b	b	b	b	b	b	b
b	b	b	b	b	b	b
b	b	b	-	-	-	-
b	b	b	b	b	b	b
b	b	-	b	b	b	b

b	b	b	-	b	b	b
b	b	b	a	b	b	b
b	b	b	a	b	b	b
b	b	b	a	b	b	b
b	b	b	a	b	b	b
b	-	b	a	b	b	b
b	b	b	a	b	b	b
b	b	b	a	b	b	b
b	b	b	a	b	b	b
b	b	b	a	b	b	b
b	b	b	a	b	b	b
b	b	b	a	b	b	b
b	b	a	a	b	b	b
b	b	a	a	b	b	b
b	b	-	a	b	b	b
b	b	a	a	b	b	b
-	b	a	b	b	b	b
b	b	a	b	b	b	b
b	-	a	b	b	b	b
b	b	a	b	b	b	b
b	b	a	b	b	b	b
b	b	a	b	b	b	b
b	b	a	-	-	-	-
b	b	a	-	b	b	b
b	b	a	b	b	b	b
b	b	a	b	b	b	b
b	-	a	-	b	b	b
b	b	a	b	b	b	b
b	a	a	b	b	b	b
b	a	a	-	b	b	b
b	a	a	-	b	a	b
b	a	a	b	b	a	b
b	a	a	-	b	a	b
b	a	a	b	b	a	b
b	a	a	b	b	a	b
b	a	a	b	b	-	b
b	a	a	b	b	a	b
b	a	a	b	b	a	b
b	a	a	b	b	a	b
b	a	a	-	-	-	-
b	a	a	-	-	-	-
b	a	a	-	-	-	-
b	a	a	b	b	a	b
b	-	a	b	b	b	b
b	a	a	a	b	a	b
b	a	a	-	-	-	-
b	a	a	b	b	a	b

-	a	a	b	b	a	b
b	a	a	-	-	-	-
b	a	a	b	a	a	b
-	a	a	-	a	a	b
b	a	-	b	a	a	b
b	a	a	b	a	a	b
b	a	a	b	a	a	b
b	a	a	b	a	a	b
b	a	a	b	a	a	b
b	a	a	b	a	a	b
b	a	a	-	a	a	b
b	a	a	b	a	a	b
b	a	a	b	a	a	b
b	a	a	a	a	a	b
b	a	a	b	a	a	b
b	a	a	b	a	a	b
b	a	a	b	a	a	b
b	a	a	b	a	a	b
b	a	a	b	a	a	b
b	a	a	b	a	b	b
b	a	a	b	a	a	b
b	a	a	b	a	a	b
b	b	b	a	a	a	a
b	b	b	a	a	a	a
b	b	b	a	a	a	a
b	b	b	a	a	a	a
b	b	b	a	a	a	a
b	b	b	a	a	a	a
b	b	b	a	a	a	a
b	b	b	a	a	a	a
b	b	b	a	a	a	a
b	b	b	a	a	a	a
b	b	b	a	a	a	a
b	b	b	a	a	a	a
b	b	b	a	a	a	a
b	b	b	a	a	a	a
b	b	b	-	a	a	a
b	b	b	b	a	a	a
b	a	b	b	a	a	a
b	-	b	b	a	a	a
b	a	b	b	a	a	a
b	a	b	b	a	a	a
b	a	b	b	a	a	a
b	a	b	-	a	a	b
b	a	b	b	a	a	b
b	a	b	b	a	a	b
b	a	b	b	a	a	b

b	a	b	b	b	a	b
b	a	b	b	b	a	b
b	a	b	b	b	a	b
b	a	a	b	b	a	b
b	a	a	b	b	a	b
b	a	a	-	b	a	b
-	a	-	b	b	a	b
b	a	a	b	b	a	b
b	a	a	b	b	a	b
b	a	a	b	b	a	b
b	a	a	b	b	a	b
b	a	a	b	b	a	b
b	a	a	b	b	a	b
b	a	a	b	b	a	b
b	b	b	-	-	-	-
b	a	a	-	-	-	-
b	a	a	b	b	a	b
b	a	a	b	b	a	b
b	a	a	b	b	a	b
b	a	a	b	-	a	b
b	a	a	-	-	-	-
b	-	a	b	b	b	b
b	a	-	b	b	b	b
b	a	a	b	b	b	b
b	a	a	b	b	b	b
b	a	a	b	b	b	b
b	a	-	b	b	b	b
b	a	a	b	b	b	b
b	a	a	-	-	-	-
-	a	a	b	b	b	b
b	a	a	b	b	b	b
-	-	a	b	b	b	b
b	b	a	b	b	b	b
b	b	-	b	b	b	b
b	b	a	b	b	b	b
b	b	a	b	b	b	b
b	b	a	b	b	b	b
b	b	a	b	b	b	b
b	b	a	b	b	b	b
b	b	a	b	b	b	b
b	b	a	b	b	b	b
b	b	a	b	b	b	b
b	b	a	b	b	b	b
b	b	a	b	b	b	b
b	b	a	b	b	b	b
b	b	a	b	b	b	b
b	b	a	b	b	b	b

b	b	a	b	b	b	b
b	b	a	b	b	b	b
b	b	a	-	b	b	b
b	b	a	b	b	b	b
b	b	a	b	b	b	b
b	b	a	b	b	b	b
b	b	b	b	a	b	b
b	b	b	b	a	b	b
b	b	b	b	a	b	b
b	b	b	b	a	b	b
b	b	b	b	a	b	b
b	b	b	b	a	b	b
b	a	b	-	a	b	b
b	a	b	b	a	b	b
b	a	b	b	a	b	b
-	a	b	b	a	b	b
b	a	b	b	a	b	b
b	a	b	b	a	b	b
b	a	b	b	a	b	b
b	a	b	b	a	b	b
a	a	b	b	a	b	b
a	a	b	b	a	b	b
a	a	b	b	a	b	b
a	a	b	-	-	-	-
-	a	b	b	a	b	b
a	a	b	b	a	b	b
a	a	b	b	a	b	b
a	a	b	b	a	b	b
a	a	b	b	a	b	b
a	a	b	b	a	b	b
a	a	-	b	a	b	b
a	a	b	b	a	b	b
a	a	b	b	a	b	b
a	a	b	-	-	-	-
a	a	b	b	b	b	b
a	-	b	b	b	b	b
a	a	-	b	b	b	b
a	a	b	-	b	b	b
a	a	b	b	b	b	b
a	a	b	b	b	b	b
-	a	b	b	b	b	b
a	a	b	-	b	b	b
a	a	-	b	b	b	b
a	a	b	b	b	b	b
-	a	b	b	b	b	b
a	a	b	b	b	b	b
a	a	b	b	b	b	b

a	a	b	-	b	b	-
a	a	b	b	b	b	b
a	a	b	-	-	-	-
a	a	b	-	-	-	-
b	a	b	-	a	a	b
b	a	-	a	a	a	b
b	a	a	-	-	-	-
b	a	a	a	a	a	b
b	a	-	a	a	a	b
-	a	a	a	a	a	b
b	a	a	a	a	a	b
b	a	a	a	a	a	b
b	a	-	a	a	a	b
-	a	a	a	a	a	b
b	a	a	a	a	a	b
b	a	a	b	a	a	b
b	a	a	-	-	-	-
b	a	a	b	a	a	b
b	a	-	b	a	a	b
b	a	-	b	a	b	b
b	a	-	b	a	b	b
b	a	a	a	a	b	b
b	-	-	b	a	b	b
b	a	a	-	a	b	b
-	a	a	b	a	b	a
b	a	a	-	-	-	-
b	a	-	b	a	b	a
b	a	a	b	a	b	a
b	a	a	b	a	b	a
b	a	a	-	-	-	-
b	a	a	-	-	-	-
b	a	a	b	a	b	a
b	a	a	-	-	-	-
b	a	a	b	a	b	a
b	a	a	-	-	-	-
b	a	a	b	a	b	a
b	a	a	b	a	b	a
b	a	a	-	-	-	-
a	a	a	b	b	b	a
-	a	a	b	b	b	a
a	a	a	b	b	b	-
a	a	a	b	b	b	a
-	a	a	b	b	b	a
a	a	a	-	b	b	a
a	a	a	b	b	b	a
a	a	a	b	b	a	a
a	a	a	b	b	a	a

a	a	a	b	b	a	a
a	a	a	a	b	a	a
a	a	a	a	b	a	a
a	a	b	-	-	-	-
b	b	b	b	a	b	a
b	b	b	b	a	b	a
b	b	b	b	a	b	a
b	b	b	b	a	b	a
b	b	b	b	a	b	a
b	b	a	b	a	b	a
b	b	a	b	a	b	a
b	b	a	b	b	b	a
b	b	a	b	b	b	a
b	b	b	b	b	b	a
b	b	-	b	b	b	a
b	b	b	b	b	b	a
b	b	b	-	-	-	-
b	b	b	b	b	b	a
b	b	b	b	b	b	-
b	b	b	b	b	b	a
b	b	-	b	b	b	a
b	b	b	b	b	b	-
b	b	b	-	b	b	a
b	b	b	a	b	b	a
b	b	b	-	-	-	-
b	a	-	b	b	a	a
b	a	b	b	b	a	a
b	a	b	b	b	a	a
b	a	b	b	b	a	a
-	a	b	b	b	a	a
b	a	b	-	-	-	-
b	a	b	-	-	-	-
b	a	b	-	-	-	-
b	a	b	a	b	a	a
b	-	b	a	b	a	a
b	a	b	a	b	a	a
b	a	b	a	b	a	a
b	a	b	a	b	a	a
b	a	b	a	b	-	a
b	a	b	-	b	a	a
b	a	b	-	-	-	-
a	b	b	a	a	a	a
a	b	b	-	-	-	-
a	b	-	a	a	a	a
a	b	b	a	a	a	a
a	a	a	a	b	b	b
a	a	a	-	b	b	b

a	a	a	a	b	b	b
a	a	a	a	b	b	b
a	a	a	a	b	b	b
a	a	a	-	-	-	-
a	a	-	a	b	b	b
a	a	a	a	b	b	b
a	a	a	a	b	b	b
a	a	a	a	b	b	b
a	a	a	a	b	b	b
a	a	a	a	b	b	b
a	a	a	a	b	b	b
a	a	a	a	b	b	b
a	a	a	a	b	b	b
a	a	a	-	-	-	-
a	a	a	a	b	b	b
-	a	a	a	b	b	b
-	a	a	a	b	b	b
a	-	a	-	b	b	b
a	a	a	a	b	b	b
a	a	a	a	b	-	b
a	a	a	a	b	b	b
a	a	a	-	b	b	b
a	a	a	a	b	b	b
a	a	a	a	b	b	b
a	a	a	b	b	b	b
a	a	a	-	b	b	b
a	a	a	-	-	-	-
a	a	a	-	-	-	-
a	a	a	-	b	b	a
a	a	a	b	b	b	a
a	a	a	b	b	b	a
a	a	a	b	b	b	a
a	a	a	b	b	b	a
a	b	a	b	b	b	a
a	b	a	b	b	b	a
a	b	a	-	b	b	a
-	b	a	b	b	b	a
a	b	a	b	b	b	a
a	b	a	b	b	b	a
a	b	a	b	b	b	a
a	b	a	b	b	b	a
a	b	-	b	b	b	a
a	b	a	b	b	b	a
a	b	-	b	b	b	a
a	b	a	b	b	b	a
a	b	a	b	b	b	a
a	b	a	b	b	b	a

a	-	a	b	b	b	a
a	b	a	b	b	b	a
a	b	a	b	b	a	a
a	b	a	-	b	a	a
a	b	a	a	b	a	a
a	b	a	-	-	-	-
a	b	a	b	b	a	b
a	b	a	b	b	a	b
a	b	a	b	b	a	b
a	b	a	a	b	a	b
a	b	a	a	b	a	b
a	a	-	-	-	-	-
a	b	a	a	b	a	b
a	-	a	a	b	a	b
b	b	a	a	b	a	b
b	b	a	-	b	a	a
b	b	a	a	b	a	a
b	b	a	-	b	a	a
b	b	a	a	b	-	a
b	-	a	a	b	a	a
b	b	a	a	a	b	a
b	b	a	a	a	a	a
b	b	a	a	a	a	a
b	b	a	a	a	a	a
b	b	-	a	a	a	a
b	b	a	a	a	a	a
b	b	a	a	a	a	a
b	b	a	-	-	-	-
b	b	a	a	a	a	a
b	a	a	a	a	a	a
b	a	a	a	a	a	-
b	a	b	a	a	a	a
b	a	b	a	a	a	a
b	a	b	a	b	a	a
b	a	b	b	b	a	a
b	a	b	-	-	-	-
b	a	b	a	b	a	a
b	a	b	-	-	-	-
b	a	b	a	b	a	a
b	a	-	a	b	a	a
b	a	b	a	b	a	a
b	a	b	a	b	a	a
b	a	b	a	b	a	a
b	a	b	a	b	a	a
b	a	b	a	b	a	a
b	-	b	-	b	a	a
a	b	b	a	b	a	a

a	b	b	a	b	a	a
a	b	b	a	b	a	a
a	b	b	a	b	a	a
a	-	b	a	b	a	a
-	b	b	a	b	a	a
a	b	b	-	b	a	a
a	b	b	a	b	a	-
a	b	b	a	b	a	a
a	b	b	a	a	b	a
-	b	b	a	a	b	a
a	b	b	-	-	-	-
a	b	b	-	-	-	-
a	b	b	a	a	b	a
a	-	b	a	a	b	a
a	b	-	a	b	b	a
a	b	b	a	b	b	a
-	b	b	a	b	b	a
a	b	b	a	b	b	a
a	a	b	a	b	b	a
a	a	a	-	-	-	-
a	-	a	a	b	b	a
a	a	a	a	b	b	a
a	a	a	a	b	b	a
-	a	a	a	b	b	a
a	a	a	a	b	b	a
a	a	a	a	b	b	a
a	a	a	a	b	b	a
a	a	a	a	b	b	a
a	a	a	-	-	-	-
-	a	a	-	b	b	a
a	a	a	-	-	-	-
a	a	a	-	b	b	a
a	a	a	a	b	b	a
a	a	-	a	b	b	a
a	a	a	a	b	-	a
b	a	a	-	b	b	a
-	a	a	a	b	b	a
b	a	a	-	-	-	-
b	a	a	a	b	b	a
b	a	a	a	b	b	a
b	a	a	a	b	a	a
-	a	a	a	b	a	a
b	a	a	a	b	a	a
b	a	a	a	b	a	a
b	a	a	b	b	a	a
b	a	-	b	b	a	a
b	a	a	b	b	a	a

b	a	a	b	b	a	a
b	a	a	-	b	a	a
b	a	a	b	b	a	a
b	a	a	b	b	a	a
b	a	a	-	-	-	-
b	a	a	b	b	a	a
b	a	a	b	b	a	a
-	a	a	b	b	a	a
a	b	a	b	a	a	a
a	b	a	b	a	a	a
a	b	a	b	a	a	a
a	b	a	b	a	a	-
b	a	b	a	a	-	b
b	-	b	a	a	a	b
b	a	b	-	-	-	-
b	a	b	a	a	a	b
b	a	b	-	-	-	-
b	a	b	a	a	a	b
b	a	b	a	a	a	a
b	a	b	a	a	a	a
b	a	-	a	a	a	a
b	a	b	a	a	a	-
b	a	b	a	a	a	a
b	a	b	-	-	-	-
b	a	b	b	a	a	a
b	a	b	b	a	a	a
b	a	b	b	a	a	a
b	a	b	-	-	-	-
b	a	b	b	a	a	a
b	a	b	-	-	-	-
b	a	-	-	a	b	a
b	a	b	b	a	b	a
b	a	b	b	a	b	a
b	a	b	b	a	b	a
b	a	b	b	a	b	a
b	a	b	b	a	b	a
b	a	b	b	a	b	a
b	a	-	-	a	b	a
b	a	b	b	a	b	a
b	a	b	b	a	b	a
a	a	b	-	-	-	-
a	a	b	b	a	b	a
a	a	b	-	-	-	-
a	a	b	-	-	-	-
a	a	b	-	-	-	-
a	b	b	b	b	b	a
a	b	b	b	b	b	a

a	b	b	b	b	b	a
-	b	b	b	b	b	a
a	b	b	b	b	b	a
a	b	b	b	b	b	a
a	b	b	b	b	b	a
a	b	b	b	b	b	a
a	b	b	b	b	b	a
a	b	b	b	b	b	a
b	b	b	b	a	b	b
b	b	b	b	a	b	b
b	b	b	b	a	b	b
-	b	b	b	a	b	b
b	b	b	b	a	b	b
b	b	b	b	a	-	b
b	b	b	b	a	b	b
b	b	b	b	a	b	b
b	b	b	b	a	b	b
b	b	b	b	a	b	b
b	b	b	b	a	b	b
b	b	b	-	a	b	b
b	b	b	-	-	-	-
-	b	b	-	a	b	b
-	b	b	a	a	b	b
b	b	b	-	a	b	b
b	b	b	a	a	b	b
b	b	b	a	b	b	b
b	b	b	a	b	b	b
b	b	-	a	b	b	b
-	b	b	a	b	b	b
b	b	b	b	b	b	b
b	b	b	a	b	b	b
b	b	b	a	b	b	b
b	b	b	a	b	b	b
-	b	b	a	b	b	b
b	b	-	a	b	a	b
-	b	b	a	b	a	b
b	b	b	a	b	a	b
b	b	b	a	b	a	b
b	b	b	a	b	a	b
-	b	b	a	b	a	b
b	b	-	a	b	a	b
b	b	b	a	b	a	b
b	b	b	a	b	a	b
b	b	b	a	b	a	b
-	-	b	a	b	a	b
b	b	b	a	b	a	b
b	-	b	a	b	a	-

b	b	b	a	b	a	b
b	b	b	-	-	-	-
b	b	b	-	-	-	-
b	b	b	a	b	a	b
b	b	b	-	-	-	-
b	-	b	a	-	a	b
b	b	b	-	-	-	-
b	b	b	a	b	a	b
b	b	b	-	-	-	-
b	-	-	b	b	a	b
a	b	b	b	b	a	b
a	b	b	b	b	a	b
a	b	b	-	-	-	-
a	b	b	b	-	a	b
a	a	b	b	b	a	b
a	a	b	b	b	a	b
-	a	b	b	b	a	b
a	a	b	-	-	-	-
a	a	a	b	b	a	a
a	a	a	b	b	a	a
b	a	b	b	b	a	b
b	a	b	b	b	a	b
b	a	b	b	b	-	b
b	a	b	-	-	-	-
b	a	b	b	b	a	b
b	a	b	b	b	b	b
b	a	b	b	b	a	b
b	a	b	-	-	-	-
b	a	b	b	b	a	b
b	a	b	-	-	-	-
b	a	b	b	b	a	b
b	a	b	-	-	-	-
b	a	b	b	b	a	b
b	a	b	-	-	-	-
b	a	b	b	b	b	b
b	a	b	b	b	b	b
b	-	b	b	b	b	b
b	a	b	-	b	b	b
b	a	b	b	b	b	b
b	a	b	a	b	b	b
b	a	b	-	-	-	-
b	a	b	a	b	b	b
b	a	b	a	b	b	b
b	a	b	b	b	b	b
b	a	b	-	-	-	-
b	a	b	b	b	b	b
b	a	b	-	-	-	-

b	a	b	b	b	b	b
b	a	b	-	b	b	b
b	a	b	b	b	b	b
b	b	b	b	b	b	b
b	b	-	a	b	b	b
b	b	b	a	b	b	b
b	b	b	a	b	b	b
b	b	b	a	b	b	b
b	b	b	-	-	-	-
b	b	b	a	b	b	a
b	b	b	a	b	b	a
b	b	b	a	b	b	a
b	b	a	a	b	b	a
b	b	a	-	b	b	a
b	b	a	a	b	b	a
b	b	a	a	a	b	a
b	b	a	a	a	b	a
-	b	a	a	a	b	a
b	b	a	b	a	b	a
b	b	a	b	a	b	a
b	b	-	-	a	b	a
-	b	a	b	a	b	a
b	b	a	b	a	b	a
b	b	a	b	a	b	a
-	b	a	-	a	b	a
b	b	a	b	a	b	a
b	b	a	b	a	b	a
b	b	a	b	a	b	-
-	b	a	b	a	b	a
b	b	a	b	a	b	a
b	b	a	b	a	b	a
b	b	a	b	a	b	a
-	b	a	b	b	b	a
b	b	a	b	b	b	a
-	b	a	-	b	b	a
b	a	b	-	-	-	-
b	a	b	a	b	a	a
b	a	b	a	b	a	a
b	a	b	a	b	a	a
b	a	b	a	b	b	a
b	a	b	-	b	b	a
b	a	b	-	-	-	-
b	a	b	a	b	b	a
b	a	b	a	b	b	a
b	b	a	-	-	-	-
b	b	a	a	b	b	a
b	a	a	a	b	b	a

-	a	a	a	b	a	a
b	a	a	a	b	b	a
b	a	a	a	b	b	a
b	a	a	a	b	b	a
b	a	a	a	b	b	a
b	a	a	a	b	b	a
b	a	a	a	b	b	a
b	a	a	a	b	b	a
b	a	a	a	b	b	a
b	a	a	a	b	b	a
b	a	a	a	b	b	a
b	a	a	a	b	b	a
b	a	a	a	b	b	a
b	a	a	a	b	b	a
b	a	a	a	b	b	a
b	a	a	-	-	-	-
b	a	a	-	b	a	a
b	a	a	a	b	a	a
b	a	a	a	b	a	a
b	b	a	a	b	a	a
b	b	a	a	b	a	a
a	b	a	a	b	a	a
a	b	a	a	b	a	a
a	a	a	a	b	a	a
a	a	a	a	b	a	a
a	a	a	a	b	a	a
a	a	a	a	b	a	a
a	a	a	a	b	a	a
a	a	a	b	b	a	a
a	a	a	b	b	a	a
a	a	a	b	b	a	a
a	a	a	b	b	a	a
a	a	a	b	b	a	a
a	a	a	b	b	a	a
a	a	a	-	-	-	-
a	a	a	-	b	a	a
a	b	a	-	-	-	-
a	b	a	-	-	-	-
a	b	a	b	b	a	a
a	b	a	b	b	a	a
a	b	a	b	b	a	a
a	b	a	b	b	a	a
a	b	a	b	b	a	a
a	b	a	-	-	-	-
a	b	a	-	-	-	-
a	b	a	b	b	a	a
a	a	b	a	b	a	b

a	a	b	a	b	a	b
a	a	b	a	b	a	b
a	a	b	a	b	a	b
a	a	b	a	b	a	b
a	a	b	a	b	a	b
a	a	b	a	b	a	b
a	a	b	a	b	a	b
a	a	b	b	b	a	b
a	a	b	a	b	a	b
a	a	b	a	b	a	a
a	a	b	a	b	a	a
a	a	b	a	b	a	a
a	a	b	a	b	a	a
a	a	b	a	b	a	a
b	a	b	a	b	a	a
b	a	b	a	b	a	a
b	a	b	a	b	a	a
a	a	b	-	-	-	-
b	a	b	a	b	a	a
b	a	b	a	b	a	a
b	a	b	a	b	a	a
b	a	b	a	b	a	a
b	a	b	a	b	a	a
b	a	b	a	b	a	a
b	a	b	a	b	a	a
b	a	b	a	b	a	a
b	a	b	a	b	b	a
b	a	b	a	b	b	a
b	a	b	a	b	b	a
b	a	b	a	b	b	a
b	a	b	a	b	b	a
b	a	b	a	b	b	a
b	a	b	a	b	b	a
b	a	b	a	b	b	a
b	a	b	a	b	b	a
b	a	b	a	b	b	a
b	a	b	a	b	b	a
b	a	b	a	b	b	a
b	b	b	-	-	-	-
b	a	b	a	-	b	a
a	b	a	a	b	b	b
b	a	b	a	a	a	a
b	a	b	a	a	a	a
b	a	b	a	a	a	a
b	a	b	a	a	a	a
b	a	b	b	a	a	a
b	a	b	b	a	a	a

b	a	b	b	a	a	a
b	a	b	b	a	a	a
b	a	a	b	a	a	a
b	a	a	b	a	a	a
b	a	a	b	a	a	a

Table S4b The map infomation between new and old genetic map of the YWDH population

New_geneticmap

Locus	Linkage group	
100015664	B01	111.618
niab013	B01	110.746
100022564	B01	109.451
100011720	B01	109.146
100029508	B01	105.379
e6m3-220	B01	99.874
100007094	B01	98.966
100011937	B01	98.511
100010320	B01	97.793
100028246	B01	97.593
100007647	B01	97.215
100042745	B01	96.216
100026299	B01	93.073
100022847	B01	92.26
100022959	B01	91.723
100076031	B01	90.73
100041944	B01	90.217
100065423	B01	89.891
100070200	B01	89.485
100014341	B01	89.149
100028912	B01	88.297
100011531	B01	87.147
100074174	B01	86.725
100020829	B01	85.041
100031276	B01	84.584
100023650	B01	84.227
100022091	B01	83.858
100011424	B01	83.494
100021449	B01	79.908
100068765	B01	78.92
100009934	B01	78.48
100011722	B01	78.271
BnGMS091	B01	76.544
100033817	B01	75.06
100038238	B01	74.697
100057816	B01	72.846
100074374	B01	72.364
100045081	B01	71.353
100011539	B01	71.033
100023500	B01	70.339

Revised linkage map -Niu et al (2023)

Locus	Linkage group		Bin code and marker included
100015664	B01	0	
niab013	B01	0.872	bin232
100022564	B01	2.167	bin233
100011720	B01	2.472	bin234
100029508	B01	6.239	bin235
e6m3-220	B01	11.744	
100007094	B01	12.652	bin236
100011937	B01	13.107	
100010320	B01	13.825	
100028246	B01	14.025	
100007647	B01	14.403	bin237
100042745	B01	15.402	
100026299	B01	18.545	
100022847	B01	19.358	
100022959	B01	19.895	bin238
100076031	B01	20.888	
100041944	B01	21.401	
100065423	B01	21.727	
100070200	B01	22.133	bin239
100014341	B01	22.469	bin240
100028912	B01	23.321	
100011531	B01	24.471	
100074174	B01	24.893	bin241
100020829	B01	26.577	bin242
100031276	B01	27.034	bin243
100023650	B01	27.391	bin244
100022091	B01	27.76	bin245
100011424	B01	28.124	
100021449	B01	31.71	
100068765	B01	32.698	bin246
100009934	B01	33.138	
100011722	B01	33.347	bin247
BnGMS091	B01	35.074	bin248
100033817	B01	36.558	
100038238	B01	36.921	bin249
100057816	B01	38.772	
100074374	B01	39.254	bin250
100045081	B01	40.265	bin251
100011539	B01	40.585	bin252
100023500	B01	41.279	

100052792	B01	69.82
100065378	B01	69.191
100066618	B01	68.656
100029966	B01	68.095
100056875	B01	67.724
100030317	B01	66.453
100053774	B01	65.97
100007356	B01	64.767
100021654	B01	61.934
100068647	B01	60.863
100068483	B01	60.564
100002456	B01	60.2
100001950	B01	59.341
100062079	B01	58.267
100007720	B01	57.554
100045734	B01	55.427
100075320	B01	55.192
100055615	B01	53.495
100027521	B01	51.038
100037087	B01	50.698
100023279	B01	50.15
100064011	B01	49.898
Ni2D08	B01	49.103
100060279	B01	48.651
100052894	B01	48.312
100006932	B01	47.282
100035548	B01	46.765
100021620	B01	46.482
100035017	B01	44.921
100042788	B01	43.914
100044445	B01	42.583
100076763	B01	42.369
100058331	B01	42.01
100019767	B01	41.588
100072282	B01	40.936
100020968	B01	40.563
100012485	B01	39.686
100018102	B01	39.284
100065962	B01	38.606
100079097	B01	36.696
100027560	B01	33.856
100049168	B01	33.314
100032315	B01	32.982
100033169	B01	32.698
100003585	B01	32.291
100028078	B01	29.54
100019234	B01	28.236

100052792	B01	41.798	
100065378	B01	42.427	
100066618	B01	42.962	bin253
100029966	B01	43.523	bin254
100056875	B01	43.894	bin255
100030317	B01	45.165	bin256
100053774	B01	45.648	bin257
100007356	B01	46.851	bin258
100021654	B01	49.684	
100068647	B01	50.755	bin259
100068483	B01	51.054	bin260
100002456	B01	51.418	bin261
100001950	B01	52.277	bin262
100062079	B01	53.351	bin263
100007720	B01	54.064	bin264
100045734	B01	56.191	
100075320	B01	56.426	bin265
100055615	B01	58.123	
100027521	B01	60.58	
100037087	B01	60.92	
100023279	B01	61.468	bin266
100064011	B01	61.72	bin267
Ni2D08	B01	62.515	bin268
100060279	B01	62.967	bin269
100052894	B01	63.306	bin270
100006932	B01	64.336	bin271
100035548	B01	64.853	bin272
100021620	B01	65.136	
100035017	B01	66.697	
100042788	B01	67.704	
100044445	B01	69.035	bin273
100076763	B01	69.249	
100058331	B01	69.608	bin274
100019767	B01	70.03	
100072282	B01	70.682	bin275
100020968	B01	71.055	bin276
100012485	B01	71.932	
100018102	B01	72.334	
100065962	B01	73.012	
100079097	B01	74.922	bin277
100027560	B01	77.762	
100049168	B01	78.304	bin278
100032315	B01	78.636	
100033169	B01	78.92	bin279
100003585	B01	79.327	
100028078	B01	82.078	bin280
100019234	B01	83.382	bin281

100020285	B01	27.558
100074919	B01	27.151
100004668	B01	26.398
100010967	B01	25.657
100010952	B01	24.391
e5m10-100	B01	24.154
100074449	B01	23.849
100032903	B01	23.452
100038153	B01	21.83
100056897	B01	20.746
100015929	B01	20.429
100005563	B01	19.615
100059174	B01	19.268
100057916	B01	18.696
100022192	B01	15.114
100020180	B01	14.892
100043041	B01	14.417
100017800	B01	14.033
100037316	B01	12.744
100070796	B01	11
Ni3C08	B01	10.39
100014913	B01	9.445
100027568	B01	6.41
100033200	B01	6.188
KS20840	B01	5.747
100025202	B01	4.433
100073308	B01	3.805
100026892	B01	3.392
100007707	B01	2.13
100020555	B01	1.776
100020359	B01	1.282
100022880	B01	0.467
Na12E05	B01	0
Ni4H04	B02	140.959
100051017	B02	130.912
100044338	B02	129.707
100042137	B02	129.262
100061546	B02	128.95
100044738	B02	128.259
100033412	B02	127.522
100021683	B02	126.34
100021088	B02	125.937
100018709	B02	125.57
100021279	B02	124.936
100015262	B02	124.651
100011123	B02	124.391
100072429	B02	124.034

100020285	B01	84.06	
100074919	B01	84.467	bin282
100004668	B01	85.22	bin283
100010967	B01	85.961	bin284
100010952	B01	87.227	bin285
e5m10-100	B01	87.464	
100074449	B01	87.769	bin286
100032903	B01	88.166	bin287
100038153	B01	89.788	
100056897	B01	90.872	bin288
100015929	B01	91.189	bin289
100005563	B01	92.003	
100059174	B01	92.35	bin290
100057916	B01	92.922	
100022192	B01	96.504	
100020180	B01	96.726	bin291
100043041	B01	97.201	
100017800	B01	97.585	bin292
100037316	B01	98.874	
100070796	B01	100.618	
Ni3C08	B01	101.228	
100014913	B01	102.173	
100027568	B01	105.208	
100033200	B01	105.43	bin293
KS20840	B01	105.871	bin294
100025202	B01	107.185	bin295
100073308	B01	107.813	
100026892	B01	108.226	
100007707	B01	109.488	bin296
100020555	B01	109.842	bin297
100020359	B01	110.336	
100022880	B01	111.151	
Na12E05	B01	111.618	
Ni4H04	B02	0	
100051017	B02	10.047	
100044338	B02	11.252	
100042137	B02	11.697	
100061546	B02	12.009	
100044738	B02	12.7	bin468
100033412	B02	13.437	
100021683	B02	14.619	bin467
100021088	B02	15.022	
100018709	B02	15.389	bin466
100021279	B02	16.023	
100015262	B02	16.308	
100011123	B02	16.568	bin465
100072429	B02	16.925	bin464

100038821	B02	123.493
100065908	B02	122.29
HAU61	B02	121.849
100044229	B02	121.497
100039342	B02	121.07
100015389	B02	120.757
100022351	B02	120.353
100005762	B02	120.086
100017911	B02	117.523
100020984	B02	117.293
100031592	B02	116.725
100047102	B02	116.428
NA12H07	B02	116.066
100033803	B02	115.561
100070790	B02	114.439
100033395	B02	113.325
100008811	B02	112.068
100062862	B02	110.262
100036105	B02	109.472
100007358	B02	109.229
100061759	B02	108.648
100060834	B02	108.392
100066360	B02	107.347
100053548	B02	106.665
100012921	B02	105.588
100013250	B02	104.987
100037990	B02	103.653
100055771	B02	103.336
100056071	B02	103.047
100035893	B02	101.068
100033667	B02	99.97
100030184	B02	99.589
100006783	B02	99.381
100015084	B02	98.822
100034989	B02	96.532
100056502	B02	95.845
100031473	B02	95.395
100071863	B02	94.822
100030404	B02	93.656
100028417	B02	93.108
100076228	B02	92.115
100019770	B02	91.596
100019364	B02	91.18
100031789	B02	90.482
100067960	B02	90.027
100024301	B02	89.443
100013920	B02	88.583

100038821	B02	17.466	bin463
100065908	B02	18.669	
HAU61	B02	19.11	
100044229	B02	19.462	
100039342	B02	19.889	bin462
100015389	B02	20.202	bin461
100022351	B02	20.606	bin460
100005762	B02	20.873	
100017911	B02	23.436	bin459
100020984	B02	23.666	bin458
100031592	B02	24.234	bin457
100047102	B02	24.531	bin456
NA12H07	B02	24.893	bin455
100033803	B02	25.398	
100070790	B02	26.52	bin454
100033395	B02	27.634	bin453
100008811	B02	28.891	bin452
100062862	B02	30.697	bin451
100036105	B02	31.487	
100007358	B02	31.73	bin450
100061759	B02	32.311	bin449
100060834	B02	32.567	bin448
100066360	B02	33.612	bin447
100053548	B02	34.294	bin446
100012921	B02	35.371	bin445
100013250	B02	35.972	bin444
100037990	B02	37.306	bin443
100055771	B02	37.623	
100056071	B02	37.912	bin442
100035893	B02	39.891	bin441
100033667	B02	40.989	
100030184	B02	41.37	bin440
100006783	B02	41.578	bin439
100015084	B02	42.137	
100034989	B02	44.427	
100056502	B02	45.114	
100031473	B02	45.564	bin438
100071863	B02	46.137	bin437
100030404	B02	47.303	bin436
100028417	B02	47.851	
100076228	B02	48.844	bin435
100019770	B02	49.363	bin434
100019364	B02	49.779	
100031789	B02	50.477	bin433
100067960	B02	50.932	bin432
100024301	B02	51.516	bin431
100013920	B02	52.376	bin430

100018326	B02	87.647
100025883	B02	87.232
100019274	B02	86.427
100023396	B02	85.401
100052047	B02	85.043
100055875	B02	84.293
100002468	B02	83.651
fito361	B02	83.325
100061890	B02	82.914
100053506	B02	78.648
100011210	B02	78.235
100059951	B02	77.27
100021151	B02	76.868
100015255	B02	76.648
100035942	B02	76.218
A8-BAC13	B02	75.753
100018753	B02	74.651
100039552	B02	71.963
100030316	B02	71.748
100005031	B02	71.525
e5m9-150	B02	70.812
CB10278	B02	70.528
100017376	B02	69.82
100020741	B02	69.058
100014832	B02	66.65
100030187	B02	65.654
100007738	B02	65.134
100033564	B02	64.364
100034017	B02	64.009
100014607	B02	62.199
100033072	B02	61.824
100028536	B02	61.249
100043699	B02	60.58
100019209	B02	50.366
100033088	B02	49.58
100033238	B02	49.116
100064622	B02	40.85
100015907	B02	36.814
100024199	B02	33.657
100078223	B02	33.129
100017191	B02	32.34
100011688	B02	31.523
100021125	B02	29.45
100029926	B02	28.107
100005174	B02	25.466
100010335	B02	23.736
100004474	B02	23.081

100018326	B02	53.312	bin429
100025883	B02	53.727	bin428
100019274	B02	54.532	bin427
100023396	B02	55.558	bin426
100052047	B02	55.916	
100055875	B02	56.666	bin425
100002468	B02	57.308	bin424
fito361	B02	57.634	bin423
100061890	B02	58.045	bin422
100053506	B02	62.311	bin421
100011210	B02	62.724	bin420
100059951	B02	63.689	
100021151	B02	64.091	bin419
100015255	B02	64.311	
100035942	B02	64.741	
A8-BAC13	B02	65.206	bin418
100018753	B02	66.308	
100039552	B02	68.996	bin417
100030316	B02	69.211	
100005031	B02	69.434	bin416
e5m9-150	B02	70.147	
CB10278	B02	70.431	
100017376	B02	71.139	bin415
100020741	B02	71.901	bin414
100014832	B02	74.309	bin413
100030187	B02	75.305	
100007738	B02	75.825	bin412
100033564	B02	76.595	
100034017	B02	76.95	
100014607	B02	78.76	
100033072	B02	79.135	bin411
100028536	B02	79.71	bin410
100043699	B02	80.379	
100019209	B02	90.593	
100033088	B02	91.379	bin409
100033238	B02	91.843	bin408
100064622	B02	100.109	bin407
100015907	B02	104.145	
100024199	B02	107.302	bin406
100078223	B02	107.83	
100017191	B02	108.619	
100011688	B02	109.436	
100021125	B02	111.509	
100029926	B02	112.852	bin405
100005174	B02	115.493	bin404
100010335	B02	117.223	bin403
100004474	B02	117.878	

100062911	B02	22.208
100025417	B02	15.808
100036555	B02	15.173
100021631	B02	13.327
100004158	B02	12.997
100065428	B02	8.199
100046847	B02	5.935
100009089	B02	5.656
100067615	B02	4.472
100058792	B02	3.123
100073662	B02	1.945
100016304	B02	1.478
100028870	B02	1.027
100079009	B02	0
100023163	B03	114.403
100039722	B03	108.697
100025223	B03	108.024
100052506	B03	107.519
100015241	B03	106.933
100050410	B03	106.43
100068882	B03	106.149
100027812	B03	105.033
100030337	B03	104.519
100015958	B03	104.205
100046407	B03	103.881
100007278	B03	103.225
100022962	B03	100.635
100052281	B03	100.256
100027570	B03	99.926
100030569	B03	99.707
100028847	B03	99.139
100018494	B03	98.255
100020458	B03	97.924
100045717	B03	97.558
PBCESSRJU8	B03	94.567
100062790	B03	94.033
100005456	B03	93.398
e6m9-250	B03	92.823
100073744	B03	92.57
100027006	B03	91.912
100014967	B03	82.893
Na12C01	B03	81.776
e3m3-250	B03	78.049
e5m9-200	B03	77.054
100015060	B03	76.308
100018762	B03	73.991
100036541	B03	72.914

100062911	B02	118.751	bin402
100025417	B02	125.151	
100036555	B02	125.786	
100021631	B02	127.632	bin401
100004158	B02	127.962	bin400
100065428	B02	132.76	
100046847	B02	135.024	
100009089	B02	135.303	
100067615	B02	136.487	
100058792	B02	137.836	bin399
100073662	B02	139.014	bin398
100016304	B02	139.481	bin397
100028870	B02	139.932	
100079009	B02	140.959	
100023163	B03	0	bin396
100039722	B03	5.706	
100025223	B03	6.379	
100052506	B03	6.884	
100015241	B03	7.47	bin395
100050410	B03	7.973	bin394
100068882	B03	8.254	bin393
100027812	B03	9.37	bin392
100030337	B03	9.884	bin391
100015958	B03	10.198	
100046407	B03	10.522	bin390
100007278	B03	11.178	
100022962	B03	13.768	
100052281	B03	14.147	bin389
100027570	B03	14.477	
100030569	B03	14.696	
100028847	B03	15.264	bin388
100018494	B03	16.148	bin387
100020458	B03	16.479	
100045717	B03	16.845	bin386
PBCESSRJU	B03	19.836	bin385
100062790	B03	20.37	bin384
100005456	B03	21.005	bin383
e6m9-250	B03	21.58	
100073744	B03	21.833	
100027006	B03	22.491	
100014967	B03	31.51	bin382
Na12C01	B03	32.627	
e3m3-250	B03	36.354	
e5m9-200	B03	37.349	
100015060	B03	38.095	bin381
100018762	B03	40.412	bin380
100036541	B03	41.489	bin379

100008558	B03	71.435
100025623	B03	70.612
100013867	B03	69.99
100036414	B03	67.977
100008060	B03	67.398
100047311	B03	66.854
100042030	B03	66.481
100072161	B03	64.847
100008064	B03	63.719
100031234	B03	62.743
100077998	B03	62.453
100007683	B03	61.813
100019505	B03	60.327
Ni4F09	B03	59.561
100037068	B03	58.82
100049303	B03	57.237
100010187	B03	56.474
100020485	B03	55.74
100033366	B03	54.443
100036978	B03	53.92
100057070	B03	51.904
100007482	B03	51.341
100019214	B03	49.579
100020332	B03	49.226
100023110	B03	48.973
100069322	B03	48.695
100053100	B03	48.264
100012533	B03	47.466
100010398	B03	46.964
100049714	B03	46.37
100059607	B03	46.022
100032410	B03	36.639
100055593	B03	35.769
100058090	B03	35.187
100006934	B03	34.901
100015887	B03	34.051
100066523	B03	33.62
100071346	B03	33.296
em1me30-250	B03	32.848
100051571	B03	31.748
100019422	B03	25.203
100012163	B03	25.001
100051178	B03	24.365
100006764	B03	24.151
100037967	B03	23.158
100005111	B03	21.487
100061126	B03	21.089

100008558	B03	42.968	bin378
100025623	B03	43.791	bin377
100013867	B03	44.413	bin376
100036414	B03	46.426	bin375
100008060	B03	47.005	
100047311	B03	47.549	bin374
100042030	B03	47.922	
100072161	B03	49.556	
100008064	B03	50.684	
100031234	B03	51.66	
100077998	B03	51.95	
100007683	B03	52.59	bin373
100019505	B03	54.076	
Ni4F09	B03	54.842	
100037068	B03	55.583	bin372
100049303	B03	57.166	bin371
100010187	B03	57.929	bin370
100020485	B03	58.663	
100033366	B03	59.96	
100036978	B03	60.483	bin369
100057070	B03	62.499	bin368
100007482	B03	63.062	bin367
100019214	B03	64.824	
100020332	B03	65.177	
100023110	B03	65.43	bin366
100069322	B03	65.708	bin365
100053100	B03	66.139	
100012533	B03	66.937	bin364
100010398	B03	67.439	bin363
100049714	B03	68.033	bin362
100059607	B03	68.381	
100032410	B03	77.764	
100055593	B03	78.634	
100058090	B03	79.216	
100006934	B03	79.502	bin361
100015887	B03	80.352	
100066523	B03	80.783	
100071346	B03	81.107	bin360
em1me30-2:	B03	81.555	
100051571	B03	82.655	
100019422	B03	89.2	
100012163	B03	89.402	bin359
100051178	B03	90.038	bin358
100006764	B03	90.252	bin357
100037967	B03	91.245	bin356
100005111	B03	92.916	bin355
100061126	B03	93.314	

100049395	B03	18.736
100068721	B03	18.11
100047463	B03	17.878
100032507	B03	16.653
100014480	B03	14.821
100049231	B03	14.445
100015321	B03	12.443
100023051	B03	10.801
100017676	B03	9.079
100063129	B03	8.531
100008476	B03	8.329
100006506	B03	7.296
100015346	B03	6.661
100037601	B03	6.46
100018506	B03	5.744
100053349	B03	4.725
100031742	B03	3.941
100017886	B03	3.525
100021203	B03	2.971
100007671	B03	2.553
e5m10-150	B03	2.133
100059045	B03	1.862
100038041	B03	1.424
100016958	B03	0.273
100007659	B03	0
100017760	B04	0
100036472	B04	1.684
100008766	B04	1.938
100020283	B04	2.271
100015838	B04	2.692
100039347	B04	3.488
100015993	B04	3.833
100067992	B04	4.198
100067298	B04	4.603
100025042	B04	6.389
100009809	B04	6.643
100018123	B04	8.614
100079041	B04	9.664
Pc	B04	10.244
100022276	B04	10.498
100054173	B04	12.26
100054045	B04	12.714
100031202	B04	14.261
100075756	B04	14.536
100075230	B04	17.156
100001486	B04	17.37
100018653	B04	17.665

100049395	B03	95.667	bin354
100068721	B03	96.293	bin353
100047463	B03	96.525	
100032507	B03	97.75	bin352
100014480	B03	99.582	bin351
100049231	B03	99.958	bin350
100015321	B03	101.96	
100023051	B03	103.602	bin349
100017676	B03	105.324	
100063129	B03	105.872	
100008476	B03	106.074	bin348
100006506	B03	107.107	bin347
100015346	B03	107.742	
100037601	B03	107.943	bin346
100018506	B03	108.659	bin345
100053349	B03	109.678	bin344
100031742	B03	110.462	bin343
100017886	B03	110.878	
100021203	B03	111.432	bin342
100007671	B03	111.85	
e5m10-150	B03	112.27	bin341
100059045	B03	112.541	
100038041	B03	112.979	
100016958	B03	114.13	
100007659	B03	114.403	
100017760	B04	0	
100036472	B04	1.684	bin298
100008766	B04	1.938	bin299
100020283	B04	2.271	
100015838	B04	2.692	bin300
100039347	B04	3.488	
100015993	B04	3.833	bin301
100067992	B04	4.198	
100067298	B04	4.603	bin302
100025042	B04	6.389	
100009809	B04	6.643	bin303
100018123	B04	8.614	bin304
100079041	B04	9.664	
Pc	B04	10.244	bin305
100022276	B04	10.498	
100054173	B04	12.26	
100054045	B04	12.714	bin306
100031202	B04	14.261	
100075756	B04	14.536	bin307
100075230	B04	17.156	
100001486	B04	17.37	
100018653	B04	17.665	

100047241	B04	18.017
100004826	B04	18.232
100007658	B04	19.811
100018125	B04	20.063
100029796	B04	21.466
100063786	B04	22.066
100002429	B04	22.619
100019406	B04	23.351
100019362	B04	24.177
em13me19-55(	B04	24.494
100015803	B04	24.944
100066297	B04	25.711
100031644	B04	26.52
100024244	B04	28.191
100024197	B04	28.525
100024396	B04	29.096
100048523	B04	29.625
BnGMS213	B04	30.206
100024742	B04	31.851
100045241	B04	32.509
100006091	B04	33.616
100043850	B04	33.951
100078929	B04	34.383
100067211	B04	34.727
100021822	B04	35.086
100070304	B04	35.962
100018961	B04	36.517
100013010	B04	36.953
100027205	B04	37.552
100055360	B04	41.839
100005371	B04	42.361
100012506	B04	42.863
100027477	B04	46.497
100066621	B04	46.814
100016370	B04	48.106
100019266	B04	48.936
100030248	B04	50.158
100030775	B04	51.425
100025201	B04	51.735
100025683	B04	54.284
100014203	B04	55.103
100012793	B04	55.349
100048347	B04	62.187
e6m9-80	B04	65.206
100026006	B04	65.922
100025622	B04	66.294
100011867	B04	66.727

100047241	B04	18.017	
100004826	B04	18.232	
100007658	B04	19.811	bin308
100018125	B04	20.063	bin309
100029796	B04	21.466	
100063786	B04	22.066	bin310
100002429	B04	22.619	bin311
100019406	B04	23.351	
100019362	B04	24.177	
em13me19-5	B04	24.494	bin312
100015803	B04	24.944	bin313
100066297	B04	25.711	
100031644	B04	26.52	
100024244	B04	28.191	bin314
100024197	B04	28.525	bin315
100024396	B04	29.096	
100048523	B04	29.625	bin316
BnGMS213	B04	30.206	bin317
100024742	B04	31.851	bin318
100045241	B04	32.509	bin319
100006091	B04	33.616	
100043850	B04	33.951	
100078929	B04	34.383	
100067211	B04	34.727	bin320
100021822	B04	35.086	bin321
100070304	B04	35.962	bin322
100018961	B04	36.517	bin323
100013010	B04	36.953	bin324
100027205	B04	37.552	
100055360	B04	41.839	
100005371	B04	42.361	bin325
100012506	B04	42.863	bin326
100027477	B04	46.497	bin327
100066621	B04	46.814	
100016370	B04	48.106	bin328
100019266	B04	48.936	bin329
100030248	B04	50.158	bin330
100030775	B04	51.425	
100025201	B04	51.735	
100025683	B04	54.284	bin331
100014203	B04	55.103	
100012793	B04	55.349	
100048347	B04	62.187	bin332
e6m9-80	B04	65.206	
100026006	B04	65.922	bin333
100025622	B04	66.294	
100011867	B04	66.727	bin334

100057157	B04	66.94
100049162	B04	67.842
100077435	B04	68.384
100050354	B04	77.707
100012372	B04	78.614
100023256	B04	81.167
100074521	B04	81.394
100013001	B04	83.086
100009827	B04	83.693
100024947	B04	85.072
100029954	B04	86.641
100021199	B04	87.145
100021508	B04	87.509
Ni2E05	B04	95.064
100017284	B04	95.764
100037214	B04	96.475
100022699	B04	102.047
100050773	B05	0
100005245	B05	0.536
100023735	B05	2.144
100016421	B05	2.656
100064697	B05	3.483
100008226	B05	4.115
100007307	B05	5.823
100045111	B05	6.33
100060363	B05	6.95
100012220	B05	7.967
100018012	B05	8.792
100023940	B05	9.293
100028569	B05	9.534
100031847	B05	10.754
100041563	B05	11.524
100027247	B05	11.811
100028171	B05	13.477
100002981	B05	15.25
100040381	B05	16.856
100013734	B05	18.073
100016187	B05	20.582
100041395	B05	28.491
100053387	B05	28.82
100059009	B05	29.178
100007386	B05	29.458
100023391	B05	30.745
100004789	B05	30.954
100047433	B05	33.868
100026480	B05	34.188
100055106	B05	34.883

100057157	B04	66.94	
100049162	B04	67.842	
100077435	B04	68.384	
100050354	B04	77.707	
100012372	B04	78.614	
100023256	B04	81.167	
100074521	B04	81.394	bin335
100013001	B04	83.086	
100009827	B04	83.693	bin336
100024947	B04	85.072	bin337
100029954	B04	86.641	bin338
100021199	B04	87.145	
100021508	B04	87.509	
Ni2E05	B04	95.064	bin339
100017284	B04	95.764	bin340
100037214	B04	96.475	
100022699	B04	102.047	
100050773	B05	0	
100005245	B05	0.536	bin047
100023735	B05	2.144	
100016421	B05	2.656	bin048
100064697	B05	3.483	
100008226	B05	4.115	bin049
100007307	B05	5.823	bin050
100045111	B05	6.33	
100060363	B05	6.95	bin051
100012220	B05	7.967	
100018012	B05	8.792	bin052
100023940	B05	9.293	
100028569	B05	9.534	
100031847	B05	10.754	bin053
100041563	B05	11.524	
100027247	B05	11.811	bin054
100028171	B05	13.477	bin055
100002981	B05	15.25	
100040381	B05	16.856	bin056
100013734	B05	18.073	
100016187	B05	20.582	
100041395	B05	28.491	
100053387	B05	28.82	
100059009	B05	29.178	bin057
100007386	B05	29.458	
100023391	B05	30.745	
100004789	B05	30.954	bin058
100047433	B05	33.868	bin059
100026480	B05	34.188	
100055106	B05	34.883	

100026976	B05	37.888
100004548	B05	39.599
100015527	B05	40.715
100025898	B05	42.391
100043823	B05	42.669
100033806	B05	42.95
100016777	B05	44.348
100019799	B05	45.077
100021480	B05	45.358
100059161	B05	47.078
100027954	B05	48.665
100051437	B05	49.113
100031574	B05	49.593
100015179	B05	49.924
100064401	B05	50.15
100038131	B05	50.381
100021699	B05	52.478
Ni2F07	B05	52.993
100038759	B05	53.227
100062128	B05	53.533
100045707	B05	54.358
100059844	B05	54.85
100012301	B05	59.355
100043931	B05	60.113
100036573	B05	60.587
100033950	B05	61.755
100060295	B05	66.068
100030774	B05	67.535
100019810	B05	68.391
100032396	B05	69.068
100024893	B05	70.518
100027389	B05	70.927
BRMS046	B05	72.274
100012892	B05	72.519
100016639	B05	74.199
100001282	B05	74.518
Ni2D10	B05	75.529
100021083	B05	76.06
100027670	B05	79.384
100070363	B05	79.991
100048879	B05	80.877
100016457	B05	81.254
100005674	B05	81.569
100028204	B05	82.417
100032373	B05	84.161
100020956	B05	87.623
100016893	B05	88.602

100026976	B05	37.888	
100004548	B05	39.599	bin060
100015527	B05	40.715	
100025898	B05	42.391	bin061
100043823	B05	42.669	
100033806	B05	42.95	
100016777	B05	44.348	
100019799	B05	45.077	bin062
100021480	B05	45.358	bin063
100059161	B05	47.078	
100027954	B05	48.665	
100051437	B05	49.113	
100031574	B05	49.593	bin064
100015179	B05	49.924	
100064401	B05	50.15	bin065
100038131	B05	50.381	
100021699	B05	52.478	bin066
Ni2F07	B05	52.993	bin067
100038759	B05	53.227	bin068
100062128	B05	53.533	
100045707	B05	54.358	bin069
100059844	B05	54.85	
100012301	B05	59.355	bin070
100043931	B05	60.113	bin071
100036573	B05	60.587	
100033950	B05	61.755	
100060295	B05	66.068	bin072
100030774	B05	67.535	bin073
100019810	B05	68.391	bin074
100032396	B05	69.068	bin075
100024893	B05	70.518	
100027389	B05	70.927	bin076
BRMS046	B05	72.274	
100012892	B05	72.519	bin077
100016639	B05	74.199	bin078
100001282	B05	74.518	bin079
Ni2D10	B05	75.529	
100021083	B05	76.06	bin080
100027670	B05	79.384	
100070363	B05	79.991	bin081
100048879	B05	80.877	bin082
100016457	B05	81.254	bin083
100005674	B05	81.569	bin084
100028204	B05	82.417	bin085
100032373	B05	84.161	
100020956	B05	87.623	
100016893	B05	88.602	bin086

100066710	B05	93.045
100007664	B05	93.838
100030861	B05	98.838
100015095	B05	99.19
100061527	B05	102.542
BnGMS044	B05	103.147
100016835	B05	103.618
100015045	B05	107.891
100048806	B05	108.88
BRMS36	B05	109.28
100037455	B05	109.533
100015465	B05	109.812
100014439	B05	111.109
100001885	B05	112.486
100021113	B05	114.717
100032378	B05	115.193
100026909	B05	116.024
100014070	B05	116.54
100046082	B05	116.86
100038240	B05	118.086
100057131	B05	124.349
100019348	B05	124.992
100067737	B05	125.902
100070871	B05	135.891
100069538	B05	136.921
100054809	B05	137.435
100016993	B05	137.841
100075572	B05	139.212
100034845	B05	140.552
100030357	B05	140.835
100004698	B05	141.39
100071120	B05	141.778
100031323	B05	144.322
100012211	B05	145.691
100024998	B05	145.929
100078356	B05	146.744
100058247	B05	148.237
100026725	B06	0
100002826	B06	0.914
100036039	B06	6.316
100025352	B06	6.772
100076205	B06	7.169
100078760	B06	8.67
100023564	B06	8.887
100022838	B06	9.381
100016091	B06	9.891
100009834	B06	12.683

100066710	B05	93.045	
100007664	B05	93.838	bin087
100030861	B05	98.838	bin088
100015095	B05	99.19	bin089
100061527	B05	102.542	bin090
BnGMS044	B05	103.147	bin091
100016835	B05	103.618	bin092
100015045	B05	107.891	
100048806	B05	108.88	bin093
BRMS36	B05	109.28	
100037455	B05	109.533	bin094
100015465	B05	109.812	bin095
100014439	B05	111.109	bin096
100001885	B05	112.486	bin097
100021113	B05	114.717	bin098
100032378	B05	115.193	
100026909	B05	116.024	
100014070	B05	116.54	
100046082	B05	116.86	
100038240	B05	118.086	bin099
100057131	B05	124.349	
100019348	B05	124.992	bin100
100067737	B05	125.902	
100070871	B05	135.891	
100069538	B05	136.921	bin101
100054809	B05	137.435	
100016993	B05	137.841	bin102
100075572	B05	139.212	
100034845	B05	140.552	
100030357	B05	140.835	
100004698	B05	141.39	bin103
100071120	B05	141.778	
100031323	B05	144.322	bin104
100012211	B05	145.691	
100024998	B05	145.929	bin105
100078356	B05	146.744	
100058247	B05	148.237	
100026725	B06	0	
100002826	B06	0.914	bin180
100036039	B06	6.316	bin181
100025352	B06	6.772	bin182
100076205	B06	7.169	
100078760	B06	8.67	
100023564	B06	8.887	
100022838	B06	9.381	bin183
100016091	B06	9.891	bin184
100009834	B06	12.683	bin185

100028661	B06	14.489
100017288	B06	15.153
100041742	B06	16.031
100013180	B06	16.655
100062356	B06	17.622
100013392	B06	20.637
100009514	B06	22.24
100063328	B06	22.606
100048699	B06	23.308
100076294	B06	26.053
ks20640-B	B06	26.733
100014228	B06	27.314
100027451	B06	27.991
100019182	B06	28.369
100011380	B06	29.925
100015166	B06	30.736
100056005	B06	31.718
100014914	B06	33.759
100015546	B06	37.032
100011988	B06	38.025
100022182	B06	38.842
100073479	B06	40.011
100025951	B06	40.49
100035292	B06	42.507
100022670	B06	44.542
100011299	B06	44.837
100028364	B06	45.327
100007539	B06	46.52
100038090	B06	48.886
100012880	B06	49.087
100077635	B06	50.065
100016097	B06	50.727
100014992	B06	51.183
100027960	B06	53.198
100011009	B06	54.602
100061647	B06	63.302
100014469	B06	63.609
100000155	B06	64.575
Ni4A10	B06	65.227
100016957	B06	65.6
100022637	B06	65.996
PBCESSRJU1	B06	72.176
100021447	B06	72.463
100019470	B06	72.695
100077914	B06	72.998
100011875	B06	74.005
100077119	B06	75.095

100028661	B06	14.489	bin186
100017288	B06	15.153	bin187
100041742	B06	16.031	
100013180	B06	16.655	bin188
100062356	B06	17.622	
100013392	B06	20.637	bin189
100009514	B06	22.24	
100063328	B06	22.606	bin190
100048699	B06	23.308	
100076294	B06	26.053	bin191
ks20640-B	B06	26.733	
100014228	B06	27.314	
100027451	B06	27.991	bin192
100019182	B06	28.369	
100011380	B06	29.925	bin193
100015166	B06	30.736	bin194
100056005	B06	31.718	
100014914	B06	33.759	
100015546	B06	37.032	bin195
100011988	B06	38.025	bin196
100022182	B06	38.842	
100073479	B06	40.011	bin197
100025951	B06	40.49	
100035292	B06	42.507	bin198
100022670	B06	44.542	
100011299	B06	44.837	bin199
100028364	B06	45.327	
100007539	B06	46.52	bin200
100038090	B06	48.886	bin201
100012880	B06	49.087	
100077635	B06	50.065	bin202
100016097	B06	50.727	bin203
100014992	B06	51.183	
100027960	B06	53.198	
100011009	B06	54.602	
100061647	B06	63.302	
100014469	B06	63.609	
100000155	B06	64.575	bin204
Ni4A10	B06	65.227	bin205
100016957	B06	65.6	
100022637	B06	65.996	
PBCESSRJU	B06	72.176	
100021447	B06	72.463	bin206
100019470	B06	72.695	
100077914	B06	72.998	bin207
100011875	B06	74.005	bin208
100077119	B06	75.095	bin209

100024938	B06	76.364
100014466	B06	78.292
100052308	B06	78.819
100030283	B06	79.144
100017414	B06	82.933
100029326	B06	85.667
100042826	B06	87.579
100005386	B06	89.779
100020421	B06	90.242
100041106	B06	91.583
100061512	B06	92.2
100037101	B06	92.453
BRAS116	B06	92.88
100013746	B06	94.528
100001380	B06	94.799
100025434	B06	101.811
100075250	B06	103.279
Ni2A09	B06	103.897
100008486	B06	105.434
BnGMS013	B06	106.893
100069214	B06	107.616
100040798	B06	109.478
100031132	B06	110.564
100004737	B06	117.501
100042372	B06	119.321
100022808	B06	121.311
100026674	B06	122.073
100008329	B06	122.413
100017303	B06	123.996
100006344	B06	124.25
100078887	B06	126.516
100023203	B06	126.928
100023658	B06	127.87
100017209	B06	128.334
100033270	B06	129.563
100037452	B06	129.804
100029522	B06	130.268
100011652	B06	132.124
100047274	B06	133.295
100054851	B07	92.393
100065271	B07	91.048
100074843	B07	90.44
100072931	B07	90.234
100004375	B07	89.885
100021852	B07	89.591
100028921	B07	89.054
100026981	B07	87.979

100024938	B06	76.364	bin210
100014466	B06	78.292	bin211
100052308	B06	78.819	bin212
100030283	B06	79.144	bin213
100017414	B06	82.933	
100029326	B06	85.667	bin214
100042826	B06	87.579	bin215
100005386	B06	89.779	bin216
100020421	B06	90.242	bin217
100041106	B06	91.583	
100061512	B06	92.2	bin218
100037101	B06	92.453	bin219
BRAS116	B06	92.88	
100013746	B06	94.528	bin220
100001380	B06	94.799	bin221
100025434	B06	101.811	bin222
100075250	B06	103.279	bin223
Ni2A09	B06	103.897	
100008486	B06	105.434	bin224
BnGMS013	B06	106.893	
100069214	B06	107.616	
100040798	B06	109.478	bin225
100031132	B06	110.564	
100004737	B06	117.501	
100042372	B06	119.321	bin226
100022808	B06	121.311	
100026674	B06	122.073	
100008329	B06	122.413	bin227
100017303	B06	123.996	
100006344	B06	124.25	bin228
100078887	B06	126.516	
100023203	B06	126.928	
100023658	B06	127.87	bin229
100017209	B06	128.334	bin230
100033270	B06	129.563	bin231
100037452	B06	129.804	
100029522	B06	130.268	
100011652	B06	132.124	
100047274	B06	133.295	
100054851	B07	0	
100065271	B07	1.345	bin046
100074843	B07	1.953	bin045
100072931	B07	2.159	
100004375	B07	2.508	bin044
100021852	B07	2.802	
100028921	B07	3.339	bin043
100026981	B07	4.414	

100022946	B07	86.997
100018779	B07	84.643
100046092	B07	84.335
100019404	B07	81.982
100031225	B07	81.584
100006925	B07	79.341
100011772	B07	78.76
100026799	B07	78.514
100065580	B07	78.09
100009526	B07	76.873
100061514	B07	76.207
100025864	B07	75.853
Na14D07	B07	75.155
100048863	B07	72.934
100009190	B07	72.651
100046735	B07	71.574
100022658	B07	70.834
100006973	B07	65.812
100053770	B07	64.298
100019213	B07	63.117
100026124	B07	60.487
100052876	B07	58.171
100025963	B07	57.716
100028838	B07	57.317
100022141	B07	56.283
100043226	B07	52.676
100028301	B07	52.362
100037660	B07	51.702
100020128	B07	51.057
100019987	B07	50.204
100043465	B07	49.497
100071901	B07	49.084
100042453	B07	48.271
100013191	B07	48.07
100007757	B07	47.314
100076398	B07	40.911
100018914	B07	39.876
cnu098	B07	38.9
100007506	B07	38.515
100029387	B07	38.189
100055425	B07	37.945
100061847	B07	37.135
100033725	B07	35.984
100048156	B07	28.774
cnu099	B07	28.526
100069283	B07	28.265
100012258	B07	27.728

100022946	B07	5.396	
100018779	B07	7.75	bin042
100046092	B07	8.058	
100019404	B07	10.411	bin041
100031225	B07	10.809	
100006925	B07	13.052	bin040
100011772	B07	13.633	bin039
100026799	B07	13.879	
100065580	B07	14.303	
100009526	B07	15.52	bin038
100061514	B07	16.186	
100025864	B07	16.54	bin037
Na14D07	B07	17.238	bin036
100048863	B07	19.459	
100009190	B07	19.742	bin035
100046735	B07	20.819	bin034
100022658	B07	21.559	
100006973	B07	26.581	bin033
100053770	B07	28.095	bin032
100019213	B07	29.276	
100026124	B07	31.906	bin031
100052876	B07	34.222	bin030
100025963	B07	34.677	bin029
100028838	B07	35.076	bin028
100022141	B07	36.11	bin027
100043226	B07	39.717	bin026
100028301	B07	40.031	
100037660	B07	40.691	bin025
100020128	B07	41.336	
100019987	B07	42.189	
100043465	B07	42.896	
100071901	B07	43.309	
100042453	B07	44.122	
100013191	B07	44.323	bin024
100007757	B07	45.079	bin023
100076398	B07	51.482	bin022
100018914	B07	52.517	
cnu098	B07	53.493	bin021
100007506	B07	53.878	bin020
100029387	B07	54.204	
100055425	B07	54.448	bin019
100061847	B07	55.258	
100033725	B07	56.409	bin018
100048156	B07	63.619	
cnu099	B07	63.867	
100069283	B07	64.128	bin017
100012258	B07	64.665	bin016

100007740	B07	27.14
100021600	B07	22.622
100067851	B07	21.71
100017597	B07	20.15
100032112	B07	19.929
100008824	B07	19.362
100039563	B07	18.961
e6m3-280	B07	14.413
100033400	B07	14.211
100041347	B07	13.479
100052248	B07	13.252
100023033	B07	12.763
100027589	B07	11.843
100007567	B07	11.256
100022941	B07	10.829
100048383	B07	10.198
100053479	B07	9.855
100075729	B07	9.324
100060659	B07	8.929
100011871	B07	8.649
100021591	B07	7.452
100055759	B07	7.223
100017774	B07	6.38
100020052	B07	6.162
100008594	B07	5.915
100022390	B07	5.641
100015564	B07	4.877
100008381	B07	4.237
100050168	B07	0
100066416	B08	148.694
100025362	B08	147.207
100013294	B08	145.417
100071515	B08	144.587
100032326	B08	143.587
100065709	B08	141.477
100007476	B08	138.876
100023660	B08	138.074
100059833	B08	137.624
100040486	B08	136.575
100071108	B08	136.227
100024082	B08	135.915
100042491	B08	135.709
100005372	B08	134.367
100049153	B08	130.763
100002494	B08	130.284
100024018	B08	129.684
100024128	B08	122.359

100007740	B07	65.253	
100021600	B07	69.771	
100067851	B07	70.683	bin015
100017597	B07	72.243	
100032112	B07	72.464	bin014
100008824	B07	73.031	bin013
100039563	B07	73.432	bin012
e6m3-280	B07	77.98	bin011
100033400	B07	78.182	
100041347	B07	78.914	bin010
100052248	B07	79.141	
100023033	B07	79.63	bin009
100027589	B07	80.55	
100007567	B07	81.137	bin008
100022941	B07	81.564	bin007
100048383	B07	82.195	bin006
100053479	B07	82.538	
100075729	B07	83.069	bin005
100060659	B07	83.464	
100011871	B07	83.744	bin004
100021591	B07	84.941	
100055759	B07	85.17	bin003
100017774	B07	86.013	
100020052	B07	86.231	
100008594	B07	86.478	bin002
100022390	B07	86.752	bin001
100015564	B07	87.516	
100008381	B07	88.156	
100050168	B07	92.393	
100066416	B08	0	
100025362	B08	1.487	bin106
100013294	B08	3.277	bin107
100071515	B08	4.107	bin108
100032326	B08	5.107	bin109
100065709	B08	7.217	bin110
100007476	B08	9.818	bin111
100023660	B08	10.62	
100059833	B08	11.07	bin112
100040486	B08	12.119	
100071108	B08	12.467	
100024082	B08	12.779	bin113
100042491	B08	12.985	
100005372	B08	14.327	bin114
100049153	B08	17.931	
100002494	B08	18.41	bin115
100024018	B08	19.01	
100024128	B08	26.335	bin116

100017996	B08	121.984
100017061	B08	112.618
e5m5-50	B08	111.055
100018028	B08	110.331
100011719	B08	106.942
100015971	B08	104.877
100043417	B08	103.403
100073700	B08	102.971
100054104	B08	101.379
100052494	B08	99.946
100028386	B08	96.374
100014239	B08	95.454
100076152	B08	92.334
100001440	B08	91.587
100007589	B08	91.073
100039529	B08	90.259
100062881	B08	85.347
100049688	B08	84.945
100045878	B08	84.451
100024846	B08	83.077
100023630	B08	79.533
100006430	B08	77.993
100031529	B08	77.197
100068880	B08	72.251
100037800	B08	70.988
100021262	B08	70.61
100069575	B08	67.165
100022376	B08	66.711
100023615	B08	66.073
100057003	B08	65.386
100077122	B08	64.792
100024207	B08	63.486
100043978	B08	62.257
100077309	B08	61.813
100053505	B08	61.458
100070293	B08	61.109
100070432	B08	60.801
100013735	B08	60.159
100017540	B08	59.67
100063747	B08	57.552
100004378	B08	56.905
100045154	B08	53.398
100030257	B08	52.712
100015259	B08	52.295
100007413	B08	48.691
100018229	B08	48.226
100028818	B08	47.946

100017996	B08	26.71	
100017061	B08	36.076	bin117
e5m5-50	B08	37.639	
100018028	B08	38.363	
100011719	B08	41.752	bin118
100015971	B08	43.817	
100043417	B08	45.291	
100073700	B08	45.723	
100054104	B08	47.315	bin119
100052494	B08	48.748	
100028386	B08	52.32	bin120
100014239	B08	53.24	bin121
100076152	B08	56.36	bin122
100001440	B08	57.107	bin123
100007589	B08	57.621	bin124
100039529	B08	58.435	bin125
100062881	B08	63.347	bin126
100049688	B08	63.749	bin127
100045878	B08	64.243	
100024846	B08	65.617	bin128
100023630	B08	69.161	
100006430	B08	70.701	
100031529	B08	71.497	
100068880	B08	76.443	bin130
100037800	B08	77.706	bin131
100021262	B08	78.084	
100069575	B08	81.529	bin132
100022376	B08	81.983	
100023615	B08	82.621	bin134
100057003	B08	83.308	bin135
100077122	B08	83.902	
100024207	B08	85.208	
100043978	B08	86.437	bin136
100077309	B08	86.881	bin137
100053505	B08	87.236	
100070293	B08	87.585	
100070432	B08	87.893	bin138
100013735	B08	88.535	bin139
100017540	B08	89.024	bin140
100063747	B08	91.142	bin141
100004378	B08	91.789	bin142
100045154	B08	95.296	bin143
100030257	B08	95.982	bin144
100015259	B08	96.399	bin145
100007413	B08	100.003	bin146
100018229	B08	100.468	
100028818	B08	100.748	bin147

100056430	B08	45.806
100078699	B08	45.603
100058267	B08	43.939
em13me19-25(	B08	43.018
100006651	B08	42.299
100013491	B08	39.415
100025744	B08	38.447
100064677	B08	38.147
100024312	B08	37.313
100016840	B08	36.766
100012806	B08	35.969
100045580	B08	35.078
100033541	B08	34.602
100066773	B08	34.173
100023649	B08	33.667
100016337	B08	33.146
100044399	B08	32.2
100073600	B08	31.613
100018041	B08	30.715
Na10D07	B08	28.655
100034901	B08	27.607
100035102	B08	27.147
100043101	B08	26.07
100017058	B08	25.242
100024366	B08	22.732
100021572	B08	22.428
100059675	B08	22.196
100022187	B08	21.649
100017045	B08	21.26
100011944	B08	20.936
100056767	B08	19.434
100071710	B08	18.721
100028694	B08	16.221
100010788	B08	15.428
100020924	B08	14.46
100029236	B08	14.244
100050502	B08	13.912
100031662	B08	13.388
100015383	B08	11.583
100005823	B08	10.213
100059103	B08	8.955
100042836	B08	8.674
100052322	B08	8.087
100014804	B08	7.694
100049415	B08	7.069
100017154	B08	6.528
100048959	B08	4.819

100056430	B08	102.888	
100078699	B08	103.091	bin148
100058267	B08	104.755	bin149
em13me19-2	B08	105.676	
100006651	B08	106.395	bin150
100013491	B08	109.279	bin151
100025744	B08	110.247	
100064677	B08	110.547	bin152
100024312	B08	111.381	bin153
100016840	B08	111.928	bin154
100012806	B08	112.725	
100045580	B08	113.616	
100033541	B08	114.092	
100066773	B08	114.521	bin155
100023649	B08	115.027	bin156
100016337	B08	115.548	bin157
100044399	B08	116.494	
100073600	B08	117.081	bin158
100018041	B08	117.979	
Na10D07	B08	120.039	
100034901	B08	121.087	bin159
100035102	B08	121.547	bin160
100043101	B08	122.624	bin161
100017058	B08	123.452	bin162
100024366	B08	125.962	bin163
100021572	B08	126.266	
100059675	B08	126.498	bin164
100022187	B08	127.045	
100017045	B08	127.434	
100011944	B08	127.758	
100056767	B08	129.26	bin165
100071710	B08	129.973	bin166
100028694	B08	132.473	bin167
100010788	B08	133.266	bin168
100020924	B08	134.234	bin169
100029236	B08	134.45	
100050502	B08	134.782	
100031662	B08	135.306	bin170
100015383	B08	137.111	bin171
100005823	B08	138.481	bin172
100059103	B08	139.739	
100042836	B08	140.02	
100052322	B08	140.607	
100014804	B08	141	bin173
100049415	B08	141.625	bin174
100017154	B08	142.166	
100048959	B08	143.875	bin175

100062839	B08	4.409
100026655	B08	4.133
100035330	B08	2.812
100024274	B08	2.039
100025810	B08	1.647
100006325	B08	0.85
100007772	B08	0.551
100040418	B08	0
niab113	C01	0
100019218	C01	7.818
100011545	C01	16.456
CB10587	C01	19.393
100011069	C01	26.279
100004218	C01	30.205
100010016	C01	31.956
100014201	C01	34.367
100064031	C01	36.165
100078611	C01	36.372
100026214	C01	37.881
100004960	C01	38.216
100026062	C01	42.377
BnGMS277-A	C01	43.049
100018611	C01	44.654
100044915	C01	45.639
100063630	C01	48.72
100015932	C01	49.715
100063706	C01	50.325
100052180	C01	50.911
100018514	C01	51.405
100060944	C01	53.854
e2m10-380	C01	54.342
100060450	C01	54.739
100016520	C01	58.398
100003472	C01	61.702
e2m5-400	C01	62.348
e5m10-290	C01	62.901
100068682	C01	63.861
SA39	C01	64.734
100015028	C01	67.745
SA54	C01	68.35
100025702	C01	70.498
100018249	C01	70.814
Na10H06	C01	76.105
100031795	C01	87.704
100062611	C01	89.799
100059879	C01	98.736
100016232	C01	99.205

100062839	B08	144.285	bin176
100026655	B08	144.561	
100035330	B08	145.882	
100024274	B08	146.655	bin177
100025810	B08	147.047	
100006325	B08	147.844	bin178
100007772	B08	148.143	bin179
100040418	B08	148.694	
niab113	C01	0	
100019218	C01	7.818	bin469
100011545	C01	16.456	bin470
CB10587	C01	19.393	
100011069	C01	26.279	bin471
100004218	C01	30.205	bin472
100010016	C01	31.956	bin473
100014201	C01	34.367	bin474
100064031	C01	36.165	bin475
100078611	C01	36.372	bin476
100026214	C01	37.881	
100004960	C01	38.216	bin477
100026062	C01	42.377	
BnGMS277-	C01	43.049	bin478
100018611	C01	44.654	bin479
100044915	C01	45.639	bin480
100063630	C01	48.72	bin481
100015932	C01	49.715	bin482
100063706	C01	50.325	
100052180	C01	50.911	bin483
100018514	C01	51.405	bin484
100060944	C01	53.854	
e2m10-380	C01	54.342	
100060450	C01	54.739	bin485
100016520	C01	58.398	
100003472	C01	61.702	
e2m5-400	C01	62.348	
e5m10-290	C01	62.901	bin487
100068682	C01	63.861	
SA39	C01	64.734	
100015028	C01	67.745	
SA54	C01	68.35	
100025702	C01	70.498	bin488
100018249	C01	70.814	
Na10H06	C01	76.105	
100031795	C01	87.704	
100062611	C01	89.799	
100059879	C01	98.736	
100016232	C01	99.205	

100020118	C01	100.574
100017613	C01	103.132
100024486	C01	103.947
100008983	C01	106.982
100056536	C01	108.165
100064206	C01	108.756
100048162	C01	109.467
100010271	C01	114.255
Ni4B10	C01	120.717
100018715	C02	0
100025181	C02	3.499
100055259	C02	3.774
100027402	C02	4.204
100046502	C02	4.636
100072642	C02	6.433
100064473	C02	7.955
100020028	C02	12.436
100071010	C02	23.022
100068200	C02	37.177
100007974	C02	37.913
100008592	C02	40.556
niab124	C02	40.894
100054557	C02	42.012
100049100	C02	42.932
100014818	C02	43.594
100057798	C02	43.854
100037219	C02	46.858
100028432	C02	48.282
100013654	C02	48.434
BnGMS345	C02	50.645
100005075	C02	65.511
100029810	C02	66.001
100015422	C02	69.244
100051900	C02	70.629
100051280	C02	79.072
e4m8-300	C02	80.144
SA12	C02	80.531
B027E01-2A	C02	85.35
100063199	C02	88.559
100040002	C02	89.139
100011677	C02	90.269
100024946	C02	90.944
100015155	C02	91.356
100047227	C02	91.807
100061522	C02	94.12
Ol13E08	C02	101.024
100007874	C02	115.53

100020118	C01	100.574	bin489
100017613	C01	103.132	bin490
100024486	C01	103.947	
100008983	C01	106.982	bin491
100056536	C01	108.165	bin492
100064206	C01	108.756	bin493
100048162	C01	109.467	
100010271	C01	114.255	
Ni4B10	C01	120.717	
100018715	C02	0	
100025181	C02	3.499	
100055259	C02	3.774	bin494
100027402	C02	4.204	bin495
100046502	C02	4.636	
100072642	C02	6.433	bin496
100064473	C02	7.955	
100020028	C02	12.436	
100071010	C02	23.022	bin497
100068200	C02	37.177	
100007974	C02	37.913	bin498
100008592	C02	40.556	
niab124	C02	40.894	
100054557	C02	42.012	bin499
100049100	C02	42.932	
100014818	C02	43.594	bin500
100057798	C02	43.854	
100037219	C02	46.858	
100028432	C02	48.282	bin501
100013654	C02	48.434	bin502
BnGMS345	C02	50.645	
100005075	C02	65.511	bin503
100029810	C02	66.001	bin504
100015422	C02	69.244	bin505
100051900	C02	70.629	
100051280	C02	79.072	bin506
e4m8-300	C02	80.144	
SA12	C02	80.531	bin507
B027E01-2A	C02	85.35	
100063199	C02	88.559	
100040002	C02	89.139	
100011677	C02	90.269	bin508
100024946	C02	90.944	bin509
100015155	C02	91.356	
100047227	C02	91.807	
100061522	C02	94.12	bin510
Ol13E08	C02	101.024	
100007874	C02	115.53	bin511

fito019	C02	116.266
100037636	C02	117.215
100041321	C02	121.683
100039080	C03	0
100049050	C03	2.257
100061792	C03	3.289
100040264	C03	3.697
100064710	C03	4.27
100008367	C03	6.028
100030726	C03	10.948
100021092	C03	11.359
100058609	C03	11.574
100028512	C03	22.993
100019306	C03	24.032
100068873	C03	26.717
100059493	C03	27.124
100010185	C03	36.168
100010157	C03	36.665
100010996	C03	37.364
100066748	C03	42.512
PBCGSSRBo1	C03	42.87
100076711	C03	44.215
Ol10B04	C03	44.845
100015636	C03	48.801
100073972	C03	51.205
100014090	C03	54.913
100026334	C03	55.482
100005398	C03	56.251
100027868	C03	56.943
100015854	C03	57.68
H015H17-1	C03	58.073
100042386	C03	59.21
100022415	C03	61.512
100070490	C03	62.389
100010575	C03	64.511
100014190	C03	64.963
100015711	C03	65.41
100055560	C03	67.234
100052674	C03	76.295
100056247	C03	76.916
100006416	C03	79.622
100022642	C03	79.937
100022688	C03	80.312
100014999	C03	84.553
100031828	C03	85.459
100069845	C03	86.258
CB10403	C03	87.287

fito019	C02	116.266	
100037636	C02	117.215	
100041321	C02	121.683	
100039080	C03	0	
100049050	C03	2.257	
100061792	C03	3.289	
100040264	C03	3.697	
100064710	C03	4.27	
100008367	C03	6.028	bin560
100030726	C03	10.948	
100021092	C03	11.359	bin559
100058609	C03	11.574	bin558
100028512	C03	22.993	bin557
100019306	C03	24.032	
100068873	C03	26.717	
100059493	C03	27.124	bin556
100010185	C03	36.168	
100010157	C03	36.665	
100010996	C03	37.364	bin555
100066748	C03	42.512	bin554
PBCGSSRB	C03	42.87	
100076711	C03	44.215	bin553
Ol10B04	C03	44.845	
100015636	C03	48.801	
100073972	C03	51.205	
100014090	C03	54.913	bin552
100026334	C03	55.482	bin551
100005398	C03	56.251	bin550
100027868	C03	56.943	bin549
100015854	C03	57.68	
H015H17-1	C03	58.073	
100042386	C03	59.21	bin548
100022415	C03	61.512	bin547
100070490	C03	62.389	bin546
100010575	C03	64.511	bin545
100014190	C03	64.963	bin544
100015711	C03	65.41	
100055560	C03	67.234	
100052674	C03	76.295	
100056247	C03	76.916	bin543
100006416	C03	79.622	
100022642	C03	79.937	
100022688	C03	80.312	
100014999	C03	84.553	
100031828	C03	85.459	
100069845	C03	86.258	bin542
CB10403	C03	87.287	

100024449	C03	87.977
100016849	C03	88.398
100052320	C03	90.597
100034569	C03	91.333
100040377	C03	91.996
PBCGSSRBo3	C03	93.294
100012746	C03	95.718
100049366	C03	96.281
100042445	C03	96.703
100034883	C03	97.989
100012667	C03	99.802
100049018	C03	100.04
100068795	C03	100.446
100065534	C03	102.13
100074355	C03	102.901
100079205	C03	103.13
100050829	C03	103.376
100018700	C03	104.617
100025118	C03	106.145
100010953	C03	106.974
100025390	C03	107.194
100043219	C03	107.544
100014532	C03	107.785
100042637	C03	109.741
100055174	C03	110.425
100026762	C03	120.848
100061627	C03	121.424
100008551	C03	121.843
100012299	C03	122.063
100011923	C03	122.449
cnu208	C03	122.654
HAU23	C03	123.109
100023245	C03	131.189
100024943	C03	131.506
100023202	C03	135.698
100038513	C03	135.938
100027338	C03	136.296
100053345	C03	136.55
100028404	C03	136.83
100039504	C03	140.59
100036885	C03	141.211
100026533	C03	141.563
100037831	C03	143.633
100002930	C03	147.66
BRMS002	C03	149.84
100029700	C03	152.778
100020169	C03	153.095

100024449	C03	87.977	
100016849	C03	88.398	bin541
100052320	C03	90.597	
100034569	C03	91.333	
100040377	C03	91.996	
PBCGSSRB	C03	93.294	bin540
100012746	C03	95.718	
100049366	C03	96.281	
100042445	C03	96.703	bin539
100034883	C03	97.989	bin538
100012667	C03	99.802	bin537
100049018	C03	100.04	bin536
100068795	C03	100.446	bin535
100065534	C03	102.13	
100074355	C03	102.901	
100079205	C03	103.13	
100050829	C03	103.376	
100018700	C03	104.617	
100025118	C03	106.145	bin534
100010953	C03	106.974	bin533
100025390	C03	107.194	bin532
100043219	C03	107.544	
100014532	C03	107.785	bin531
100042637	C03	109.741	bin530
100055174	C03	110.425	
100026762	C03	120.848	
100061627	C03	121.424	
100008551	C03	121.843	
100012299	C03	122.063	bin529
100011923	C03	122.449	
cnu208	C03	122.654	bin528
HAU23	C03	123.109	
100023245	C03	131.189	
100024943	C03	131.506	bin527
100023202	C03	135.698	bin526
100038513	C03	135.938	bin525
100027338	C03	136.296	
100053345	C03	136.55	bin524
100028404	C03	136.83	
100039504	C03	140.59	
100036885	C03	141.211	
100026533	C03	141.563	bin523
100037831	C03	143.633	
100002930	C03	147.66	bin522
BRMS002	C03	149.84	bin521
100029700	C03	152.778	
100020169	C03	153.095	bin520

100062181	C03	153.446
100015635	C03	155.641
100014808	C03	156.14
100050910	C03	156.447
100017735	C03	159.69
100032757	C03	159.902
100020221	C03	160.848
BnGMS426	C03	163.861
100013435	C03	164.701
100031168	C03	165.754
100021460	C03	166.68
100029139	C03	167.003
100013753	C03	168.045
100026061	C04	0
100023632	C04	2.883
100019506	C04	3.087
100078970	C04	3.521
100065508	C04	10.431
100036778	C04	11.135
100026342	C04	16.42
BRAS063	C04	23.268
100037486	C04	24.519
100015148	C04	24.85
100016930	C04	28.912
100048982	C04	32.374
100021474	C04	33.105
100025551	C04	33.367
100009621	C04	34.259
100029356	C04	34.849
100059630	C04	54.266
100023611	C04	54.696
100074863	C04	55.7
100016523	C04	56.579
100024625	C04	56.832
NA12E06A	C04	59.483
100029056	C04	60.459
100040086	C04	61.546
100022727	C04	70.261
100044151	C04	71.026
100013195	C04	71.522
100007868	C04	72.245
Ni2E07	C04	73.941
100008940	C04	84.191
niab021	C04	90.964
100022579	C04	92.097
100043087	C04	99.511
100012771	C04	99.861

100062181	C03	153.446	bin519
100015635	C03	155.641	bin518
100014808	C03	156.14	bin517
100050910	C03	156.447	
100017735	C03	159.69	bin516
100032757	C03	159.902	
100020221	C03	160.848	bin515
BnGMS426	C03	163.861	bin514
100013435	C03	164.701	
100031168	C03	165.754	
100021460	C03	166.68	bin513
100029139	C03	167.003	bin512
100013753	C03	168.045	
100026061	C04	0	
100023632	C04	2.883	bin578
100019506	C04	3.087	
100078970	C04	3.521	
100065508	C04	10.431	
100036778	C04	11.135	bin577
100026342	C04	16.42	
BRAS063	C04	23.268	
100037486	C04	24.519	
100015148	C04	24.85	bin576
100016930	C04	28.912	
100048982	C04	32.374	
100021474	C04	33.105	bin575
100025551	C04	33.367	bin574
100009621	C04	34.259	bin573
100029356	C04	34.849	
100059630	C04	54.266	
100023611	C04	54.696	
100074863	C04	55.7	bin572
100016523	C04	56.579	
100024625	C04	56.832	bin571
NA12E06A	C04	59.483	bin570
100029056	C04	60.459	
100040086	C04	61.546	
100022727	C04	70.261	
100044151	C04	71.026	bin569
100013195	C04	71.522	
100007868	C04	72.245	
Ni2E07	C04	73.941	
100008940	C04	84.191	
niab021	C04	90.964	
100022579	C04	92.097	
100043087	C04	99.511	
100012771	C04	99.861	

100055466	C04	101.041
100012629	C04	105.171
100022173	C04	120.909
100035805	C04	121.33
100032039	C04	121.698
ks20640-A	C04	122.487
100027194	C04	131.982
100058814	C04	133.966
100019865	C04	135.306
100026640	C04	135.526
100021516	C04	135.73
100029133	C04	137.752
100008104	C04	140.451
Ol11D12	C04	141.224
OL11H02	C04	143.368
100021627	C04	143.807
100024089	C04	145.33
100008603	C05	123.88
100028725	C05	122.277
100013730	C05	119.728
100067924	C05	114.826
100026428	C05	108.145
100016364	C05	102.455
100059378	C05	95.525
100021467	C05	92.05
100017632	C05	91.604
100030993	C05	88.235
Ol10A10	C05	79.445
Na10D11	C05	76.745
sS2131	C05	76.309
100044388	C05	75.948
MR113	C05	75.028
100006019	C05	68.588
100034396	C05	66.839
100057078	C05	65.949
100016951	C05	65.346
100037609	C05	64.692
100012535	C05	60.141
100018642	C05	59.537
100035903	C05	56.035
100065201	C05	55.338
100078636	C05	54.854
CB10065	C05	54.617
100009644	C05	52.208
BnGMS256	C05	50.484
100047046	C05	42.265
100018683	C05	41.534

100055466	C04	101.041	bin568
100012629	C04	105.171	
100022173	C04	120.909	
100035805	C04	121.33	bin567
100032039	C04	121.698	
ks20640-A	C04	122.487	bin566
100027194	C04	131.982	bin565
100058814	C04	133.966	
100019865	C04	135.306	
100026640	C04	135.526	bin564
100021516	C04	135.73	
100029133	C04	137.752	bin563
100008104	C04	140.451	bin562
Ol11D12	C04	141.224	
OL11H02	C04	143.368	
100021627	C04	143.807	bin561
100024089	C04	145.33	
100008603	C05	0	bin593
100028725	C05	1.603	bin592
100013730	C05	4.152	
100067924	C05	9.054	
100026428	C05	15.735	
100016364	C05	21.425	bin591
100059378	C05	28.355	
100021467	C05	31.83	
100017632	C05	32.276	bin590
100030993	C05	35.645	bin589
Ol10A10	C05	44.435	
Na10D11	C05	47.135	
sS2131	C05	47.571	bin588
100044388	C05	47.932	bin587
MR113	C05	48.852	
100006019	C05	55.292	
100034396	C05	57.041	bin586
100057078	C05	57.931	
100016951	C05	58.534	bin585
100037609	C05	59.188	bin584
100012535	C05	63.739	
100018642	C05	64.343	bin583
100035903	C05	67.845	
100065201	C05	68.542	bin582
100078636	C05	69.026	
CB10065	C05	69.263	
100009644	C05	71.672	bin581
BnGMS256	C05	73.396	
100047046	C05	81.615	
100018683	C05	82.346	

100013233	C05	41.076
BnGMS292	C05	37
100038782	C05	29.615
100022700	C05	27.607
100034032	C05	26.864
100013309	C05	25.695
100041974	C05	24.764
100006256	C05	17.863
BnGMS027	C05	14.422
100069690	C05	10.029
BnGMS334	C05	2.015
100007983	C05	0.979
100016245	C05	0
100018304	C06	0
100032948	C06	1.201
BnGMS567	C06	17.482
100035303	C06	21.853
100008675	C06	22.277
100023158	C06	24.847
100023982	C06	26.844
SA60	C06	27.662
100020680	C06	28.267
100028657	C06	29.36
100035726	C06	32.083
CB10234	C06	33.861
100037880	C06	34.691
Na12E01	C06	36.739
100021971	C06	44.216
CB10439	C06	45.229
100008810	C06	45.766
e2m5-320	C06	47.498
BRAS004	C06	47.924
100012448	C06	48.894
100040008	C06	49.302
100033089	C06	54.227
100056481	C06	55.199
100023693	C06	55.792
100015500	C06	57.46
100031328	C06	59.073
100035118	C06	60.597
100026430	C06	61.728
100028305	C06	62.07
100050944	C06	62.862
100064230	C06	65.2
100040545	C06	65.677
100020694	C06	66.037
HG-FT-C6a	C06	67.916

100013233	C05	82.804	
BnGMS292	C05	86.88	
100038782	C05	94.265	
100022700	C05	96.273	
100034032	C05	97.016	
100013309	C05	98.185	
100041974	C05	99.116	
100006256	C05	106.017	bin580
BnGMS027	C05	109.458	
100069690	C05	113.851	
BnGMS334	C05	121.865	
100007983	C05	122.901	bin579
100016245	C05	123.88	
100018304	C06	0	
100032948	C06	1.201	
BnGMS567	C06	17.482	
100035303	C06	21.853	bin617
100008675	C06	22.277	bin616
100023158	C06	24.847	bin615
100023982	C06	26.844	bin614
SA60	C06	27.662	bin613
100020680	C06	28.267	bin612
100028657	C06	29.36	
100035726	C06	32.083	bin611
CB10234	C06	33.861	
100037880	C06	34.691	
Na12E01	C06	36.739	
100021971	C06	44.216	bin610
CB10439	C06	45.229	
100008810	C06	45.766	bin609
e2m5-320	C06	47.498	
BRAS004	C06	47.924	
100012448	C06	48.894	bin608
100040008	C06	49.302	
100033089	C06	54.227	
100056481	C06	55.199	
100023693	C06	55.792	
100015500	C06	57.46	bin607
100031328	C06	59.073	bin606
100035118	C06	60.597	bin605
100026430	C06	61.728	
100028305	C06	62.07	
100050944	C06	62.862	bin604
100064230	C06	65.2	
100040545	C06	65.677	
100020694	C06	66.037	bin603
HG-FT-C6a	C06	67.916	

100017755	C06	69.321
100061440	C06	69.888
100009082	C06	70.993
100028996	C06	71.731
100012727	C06	76.878
100020122	C06	78.99
100019630	C06	81.206
100073913	C06	82.092
100033606	C06	83.851
100037463	C06	86.425
100041764	C06	86.797
100036823	C06	87.808
BnGMS323	C06	88.662
100078804	C06	90.375
100013331	C06	91.478
100062752	C06	92.105
100037438	C06	93.943
100024804	C06	94.209
100027403	C06	96.012
100030519	C06	96.534
100029429	C06	97.637
100032036	C06	98.923
100007607	C06	101.876
100034215	C06	103.79
100057725	C06	104.931
100037859	C07	0
100007433	C07	1.642
100048720	C07	4.721
100040526	C07	8.186
100013962	C07	8.506
100025920	C07	8.859
100013825	C07	9.289
100038163	C07	14.206
100003285	C07	14.67
100020742	C07	15.861
100024192	C07	20.428
100034282	C07	20.997
100019077	C07	21.688
100062030	C07	21.976
100065291	C07	22.83
100037708	C07	26.011
100061207	C07	30.784
100030964	C07	31.45
100009750	C07	32.916
100006664	C07	33.631
100072240	C07	38.953
100028791	C07	39.231

100017755	C06	69.321	bin602
100061440	C06	69.888	
100009082	C06	70.993	
100028996	C06	71.731	
100012727	C06	76.878	bin601
100020122	C06	78.99	bin600
100019630	C06	81.206	
100073913	C06	82.092	bin599
100033606	C06	83.851	
100037463	C06	86.425	bin598
100041764	C06	86.797	
100036823	C06	87.808	
BnGMS323	C06	88.662	
100078804	C06	90.375	bin597
100013331	C06	91.478	
100062752	C06	92.105	
100037438	C06	93.943	
100024804	C06	94.209	
100027403	C06	96.012	bin596
100030519	C06	96.534	
100029429	C06	97.637	
100032036	C06	98.923	
100007607	C06	101.876	
100034215	C06	103.79	bin595
100057725	C06	104.931	bin594
100037859	C07	0	
100007433	C07	1.642	bin648
100048720	C07	4.721	
100040526	C07	8.186	
100013962	C07	8.506	
100025920	C07	8.859	bin647
100013825	C07	9.289	
100038163	C07	14.206	
100003285	C07	14.67	bin646
100020742	C07	15.861	
100024192	C07	20.428	
100034282	C07	20.997	bin645
100019077	C07	21.688	bin644
100062030	C07	21.976	bin643
100065291	C07	22.83	bin642
100037708	C07	26.011	
100061207	C07	30.784	
100030964	C07	31.45	bin641
100009750	C07	32.916	bin640
100006664	C07	33.631	
100072240	C07	38.953	bin639
100028791	C07	39.231	

100053583	C07	40.441
100028349	C07	43.465
100048778	C07	43.847
100054865	C07	45.63
PBCGSSRBo3	C07	50.584
100046587	C07	51.136
100033605	C07	52.268
100018746	C07	52.609
100040390	C07	54.204
100022249	C07	57.732
100046712	C07	61.189
100068546	C07	61.939
100078147	C07	62.258
cnu371	C07	63.721
100034293	C07	66.414
BnGMS357	C07	67.131
100071984	C07	68.627
100034961	C07	73.534
100024563	C07	74.54
BnGMS277-B	C07	76.112
100038749	C07	80.026
100011061	C07	82.476
100004847	C07	83.874
100065439	C07	85.437
100051078	C07	88.579
100013999	C07	89.664
BnGMS508	C07	90.149
100005592	C07	91.091
CB10413	C07	92.171
100015457	C07	92.74
B089G06-3	C07	93.311
100012656	C07	94.051
BnGMS392	C07	95.569
100027577	C07	95.919
100028050	C07	96.192
100026256	C07	97.965
em1me30-300	C07	98.338
100021982	C07	99.536
100019616	C07	101.171
100051548	C07	102.277
e6m3-180	C08	0
100017146	C08	1.495
100039765	C08	2.052
100014017	C08	2.745
100055365	C08	6.069
100014401	C08	7.247
SA69	C08	10.013

100053583	C07	40.441	bin638
100028349	C07	43.465	bin637
100048778	C07	43.847	
100054865	C07	45.63	bin636
PBCGSSRB	C07	50.584	
100046587	C07	51.136	bin635
100033605	C07	52.268	
100018746	C07	52.609	bin634
100040390	C07	54.204	bin633
100022249	C07	57.732	bin632
100046712	C07	61.189	
100068546	C07	61.939	
100078147	C07	62.258	
cnu371	C07	63.721	bin631
100034293	C07	66.414	
BnGMS357	C07	67.131	
100071984	C07	68.627	bin630
100034961	C07	73.534	
100024563	C07	74.54	bin629
BnGMS277-	C07	76.112	
100038749	C07	80.026	bin628
100011061	C07	82.476	bin627
100004847	C07	83.874	bin626
100065439	C07	85.437	
100051078	C07	88.579	bin625
100013999	C07	89.664	bin624
BnGMS508	C07	90.149	
100005592	C07	91.091	bin623
CB10413	C07	92.171	
100015457	C07	92.74	bin622
B089G06-3	C07	93.311	
100012656	C07	94.051	bin621
BnGMS392	C07	95.569	
100027577	C07	95.919	bin620
100028050	C07	96.192	bin619
100026256	C07	97.965	
em1me30-3(	C07	98.338	
100021982	C07	99.536	
100019616	C07	101.171	bin618
100051548	C07	102.277	
e6m3-180	C08	0	
100017146	C08	1.495	
100039765	C08	2.052	
100014017	C08	2.745	
100055365	C08	6.069	
100014401	C08	7.247	
SA69	C08	10.013	

100024334	C08	12.133
100029984	C08	12.605
SA06	C08	22.058
100011973	C08	22.922
100031848	C08	26.896
100053216	C08	27.45
100015981	C08	27.97
100021875	C08	28.278
100036359	C08	28.912
100062727	C08	30.146
100075300	C08	34.278
100023350	C08	34.684
100010968	C08	35.15
100045208	C08	35.928
100019441	C08	40.736
100015313	C08	41.318
100030360	C08	41.693
100065251	C08	42.099
100012490	C08	42.425
100026947	C08	43.058
em6me26-450	C08	43.519
100075461	C08	48.13
100022161	C08	48.688
100027474	C08	49.132
100032715	C08	51.31
100011644	C08	55.774
100029043	C08	58.734
100006162	C08	61.64
100028825	C08	62.528
100009857	C08	62.809
100029655	C08	63.49
100009196	C08	63.999
100040100	C08	64.561
100001609	C08	69.283
100008240	C08	69.676
100042506	C08	71.017
100041581	C08	71.741
100043358	C08	73.139
100038688	C08	73.493
H010P01-2	C08	77.073
100003965	C08	77.495
CB10028	C08	80.419
CB10092-B	C08	80.975
100010513	C08	81.703
100053806	C08	85.51
100069841	C08	86.515
100063523	C08	87.332

100024334	C08	12.133	bin649
100029984	C08	12.605	
SA06	C08	22.058	
100011973	C08	22.922	bin650
100031848	C08	26.896	
100053216	C08	27.45	
100015981	C08	27.97	bin651
100021875	C08	28.278	
100036359	C08	28.912	bin652
100062727	C08	30.146	bin653
100075300	C08	34.278	bin654
100023350	C08	34.684	bin655
100010968	C08	35.15	bin656
100045208	C08	35.928	bin657
100019441	C08	40.736	
100015313	C08	41.318	
100030360	C08	41.693	
100065251	C08	42.099	
100012490	C08	42.425	bin658
100026947	C08	43.058	
em6me26-4	C08	43.519	
100075461	C08	48.13	bin659
100022161	C08	48.688	bin660
100027474	C08	49.132	bin661
100032715	C08	51.31	bin662
100011644	C08	55.774	bin663
100029043	C08	58.734	
100006162	C08	61.64	bin664
100028825	C08	62.528	
100009857	C08	62.809	
100029655	C08	63.49	bin665
100009196	C08	63.999	bin666
100040100	C08	64.561	
100001609	C08	69.283	
100008240	C08	69.676	bin667
100042506	C08	71.017	
100041581	C08	71.741	
100043358	C08	73.139	
100038688	C08	73.493	bin668
H010P01-2	C08	77.073	
100003965	C08	77.495	bin669
CB10028	C08	80.419	
CB10092-B	C08	80.975	bin670
100010513	C08	81.703	bin671
100053806	C08	85.51	bin672
100069841	C08	86.515	bin673
100063523	C08	87.332	

100032920	C08	88.455
e2m3-290	C08	89.212
BnGMS148	C08	89.933
100009976	C08	90.345
100020225S	C09	0
3078270S	C09	1.126
BnGMS679	C09	2.242
100034307	C09	3.582
100015277	C09	3.649
100005670	C09	4.435
100054209	C09	5.322
3077513S	C09	11.6
H125N23-1	C09	13.449
100012123	C09	14.115
100015972	C09	15.509
100060680	C09	16.325
100028894	C09	17.481
100017422	C09	17.719
100005758	C09	18.328
100050041	C09	18.858
100079100	C09	20.005
3135099S	C09	20.542
Ol10D08	C09	21.588
100040281S	C09	26.998
100062459	C09	27.461
100074931	C09	28.312
100064959	C09	28.52
100024455	C09	31.718
100073939	C09	32.236
100066781	C09	33.885
100052899	C09	34.442
3078850S	C09	36.621
100013369	C09	38.906
100008593	C09	40.633
100006331	C09	44.215
100030523	C09	44.979
100074804	C09	50.682
100010811	C09	51.457
100040921	C09	51.913
100064791	C09	54.979
3079893S	C09	55.141
100014743	C09	55.669
3100861S	C09	57.546
sN2016	C09	62.03
A8-BAC22	C09	65.37
3085906S	C09	77.319
100026545	C09	100.13

100032920	C08	88.455	bin674
e2m3-290	C08	89.212	
BnGMS148	C08	89.933	
100009976	C08	90.345	
100020225S	C09	0	
3078270S	C09	1.126	
BnGMS679	C09	2.242	
100034307	C09	3.582	
100015277	C09	3.649	bin675
100005670	C09	4.435	
100054209	C09	5.322	bin676
3077513S	C09	11.6	
H125N23-1	C09	13.449	
100012123	C09	14.115	bin677
100015972	C09	15.509	
100060680	C09	16.325	bin678
100028894	C09	17.481	
100017422	C09	17.719	
100005758	C09	18.328	bin679
100050041	C09	18.858	bin680
100079100	C09	20.005	bin681
3135099S	C09	20.542	
Ol10D08	C09	21.588	
100040281S	C09	26.998	
100062459	C09	27.461	
100074931	C09	28.312	
100064959	C09	28.52	
100024455	C09	31.718	
100073939	C09	32.236	bin682
100066781	C09	33.885	bin683
100052899	C09	34.442	
3078850S	C09	36.621	bin684
100013369	C09	38.906	bin685
100008593	C09	40.633	bin686
100006331	C09	44.215	bin687
100030523	C09	44.979	
100074804	C09	50.682	bin688
100010811	C09	51.457	bin689
100040921	C09	51.913	
100064791	C09	54.979	bin690
3079893S	C09	55.141	bin691
100014743	C09	55.669	bin692
3100861S	C09	57.546	
sN2016	C09	62.03	
A8-BAC22	C09	65.37	bin693
3085906S	C09	77.319	
100026545	C09	100.13	

100024657	C09	103.457
100013650	C09	103.9
3082910S	C09	104.289
100026791	C09	104.831
BnGMS085	C09	105.376
100053369	C09	106.454
100013061	C09	106.806
3074768S	C09	111.332
100055717	C09	112.73
100019001	C09	115.651

100024657	C09	103.457	
100013650	C09	103.9	bin694
3082910S	C09	104.289	
100026791	C09	104.831	bin695
BnGMS085	C09	105.376	bin696
100053369	C09	106.454	
100013061	C09	106.806	
3074768S	C09	111.332	bin697
100055717	C09	112.73	bin698
100019001	C09	115.651	

.

	OLD linkage map-	
Marker type	Original linkage group	Position
silico DArTs	B05	0
SSR marker	B05	0.872
silico DArTs	B05	2.167
silico DArTs	B05	2.472
silico DArTs	B05	6.239
SSR marker	B05	11.744
silico DArTs	B05	12.652
silico DArTs	B05	13.107
silico DArTs	B05	13.825
silico DArTs	B05	14.025
silico DArTs	B05	14.403
silico DArTs	B05	15.402
silico DArTs	B05	18.545
silico DArTs	B05	19.358
silico DArTs	B05	19.895
silico DArTs	B05	20.888
silico DArTs	B05	21.401
silico DArTs	B05	21.727
silico DArTs	B05	22.133
silico DArTs	B05	22.469
silico DArTs	B05	23.321
silico DArTs	B05	24.471
silico DArTs	B05	24.893
silico DArTs	B05	26.577
silico DArTs	B05	27.034
silico DArTs	B05	27.391
silico DArTs	B05	27.76
silico DArTs	B05	28.124
silico DArTs	B05	31.71
silico DArTs	B05	32.698
silico DArTs	B05	33.138
silico DArTs	B05	33.347
SSR marker	B05	35.074
silico DArTs	B05	36.558
silico DArTs	B05	36.921
silico DArTs	B05	38.772
silico DArTs	B05	39.254
silico DArTs	B05	40.265
silico DArTs	B05	40.585
silico DArTs	B05	41.279

silico DArTs	B05	41.798
silico DArTs	B05	42.427
silico DArTs	B05	42.962
silico DArTs	B05	43.523
silico DArTs	B05	43.894
silico DArTs	B05	45.165
silico DArTs	B05	45.648
silico DArTs	B05	46.851
silico DArTs	B05	49.684
silico DArTs	B05	50.755
silico DArTs	B05	51.054
silico DArTs	B05	51.418
silico DArTs	B05	52.277
silico DArTs	B05	53.351
silico DArTs	B05	54.064
silico DArTs	B05	56.191
silico DArTs	B05	56.426
silico DArTs	B05	58.123
silico DArTs	B05	60.58
silico DArTs	B05	60.92
silico DArTs	B05	61.468
silico DArTs	B05	61.72
SSR marker	B05	62.515
silico DArTs	B05	62.967
silico DArTs	B05	63.306
silico DArTs	B05	64.336
silico DArTs	B05	64.853
silico DArTs	B05	65.136
silico DArTs	B05	66.697
silico DArTs	B05	67.704
silico DArTs	B05	69.035
silico DArTs	B05	69.249
silico DArTs	B05	69.608
silico DArTs	B05	70.03
silico DArTs	B05	70.682
silico DArTs	B05	71.055
silico DArTs	B05	71.932
silico DArTs	B05	72.334
silico DArTs	B05	73.012
silico DArTs	B05	74.922
silico DArTs	B05	77.762
silico DArTs	B05	78.304
silico DArTs	B05	78.636
silico DArTs	B05	78.92
silico DArTs	B05	79.327
silico DArTs	B05	82.078
silico DArTs	B05	83.382

silico DArTs	B05	84.06
silico DArTs	B05	84.467
silico DArTs	B05	85.22
silico DArTs	B05	85.961
silico DArTs	B05	87.227
SSR marker	B05	87.464
silico DArTs	B05	87.769
silico DArTs	B05	88.166
silico DArTs	B05	89.788
silico DArTs	B05	90.872
silico DArTs	B05	91.189
silico DArTs	B05	92.003
silico DArTs	B05	92.35
silico DArTs	B05	92.922
silico DArTs	B05	96.504
silico DArTs	B05	96.726
silico DArTs	B05	97.201
silico DArTs	B05	97.585
silico DArTs	B05	98.874
silico DArTs	B05	100.618
SSR marker	B05	101.228
silico DArTs	B05	102.173
silico DArTs	B05	105.208
silico DArTs	B05	105.43
SSR marker	B05	105.871
silico DArTs	B05	107.185
silico DArTs	B05	107.813
silico DArTs	B05	108.226
silico DArTs	B05	109.488
silico DArTs	B05	109.842
silico DArTs	B05	110.336
silico DArTs	B05	111.151
SSR marker	B05	111.618
SSR marker	B08	140.959
silico DArTs	B08	130.912
silico DArTs	B08	129.707
silico DArTs	B08	129.262
silico DArTs	B08	128.95
silico DArTs	B08	128.259
silico DArTs	B08	127.522
silico DArTs	B08	126.34
silico DArTs	B08	125.937
silico DArTs	B08	125.57
silico DArTs	B08	124.936
silico DArTs	B08	124.651
silico DArTs	B08	124.391
silico DArTs	B08	124.034

silico DArTs	B08	123.493
silico DArTs	B08	122.29
SSR marker	B08	121.849
silico DArTs	B08	121.497
silico DArTs	B08	121.07
silico DArTs	B08	120.757
silico DArTs	B08	120.353
silico DArTs	B08	120.086
silico DArTs	B08	117.523
silico DArTs	B08	117.293
silico DArTs	B08	116.725
silico DArTs	B08	116.428
SSR marker	B08	116.066
silico DArTs	B08	115.561
silico DArTs	B08	114.439
silico DArTs	B08	113.325
silico DArTs	B08	112.068
silico DArTs	B08	110.262
silico DArTs	B08	109.472
silico DArTs	B08	109.229
silico DArTs	B08	108.648
silico DArTs	B08	108.392
silico DArTs	B08	107.347
silico DArTs	B08	106.665
silico DArTs	B08	105.588
silico DArTs	B08	104.987
silico DArTs	B08	103.653
silico DArTs	B08	103.336
silico DArTs	B08	103.047
silico DArTs	B08	101.068
silico DArTs	B08	99.97
silico DArTs	B08	99.589
silico DArTs	B08	99.381
silico DArTs	B08	98.822
silico DArTs	B08	96.532
silico DArTs	B08	95.845
silico DArTs	B08	95.395
silico DArTs	B08	94.822
silico DArTs	B08	93.656
silico DArTs	B08	93.108
silico DArTs	B08	92.115
silico DArTs	B08	91.596
silico DArTs	B08	91.18
silico DArTs	B08	90.482
silico DArTs	B08	90.027
silico DArTs	B08	89.443
silico DArTs	B08	88.583

silico DArTs	B08	87.647
silico DArTs	B08	87.232
silico DArTs	B08	86.427
silico DArTs	B08	85.401
silico DArTs	B08	85.043
silico DArTs	B08	84.293
silico DArTs	B08	83.651
SSR marker	B08	83.325
silico DArTs	B08	82.914
silico DArTs	B08	78.648
silico DArTs	B08	78.235
silico DArTs	B08	77.27
silico DArTs	B08	76.868
silico DArTs	B08	76.648
silico DArTs	B08	76.218
SSR marker	B08	75.753
silico DArTs	B08	74.651
silico DArTs	B08	71.963
silico DArTs	B08	71.748
silico DArTs	B08	71.525
SSR marker	B08	70.812
SSR marker	B08	70.528
silico DArTs	B08	69.82
silico DArTs	B08	69.058
silico DArTs	B08	66.65
silico DArTs	B08	65.654
silico DArTs	B08	65.134
silico DArTs	B08	64.364
silico DArTs	B08	64.009
silico DArTs	B08	62.199
silico DArTs	B08	61.824
silico DArTs	B08	61.249
silico DArTs	B08	60.58
silico DArTs	B08	50.366
silico DArTs	B08	49.58
silico DArTs	B08	49.116
silico DArTs	B08	40.85
silico DArTs	B08	36.814
silico DArTs	B08	33.657
silico DArTs	B08	33.129
silico DArTs	B08	32.34
silico DArTs	B08	31.523
silico DArTs	B08	29.45
silico DArTs	B08	28.107
silico DArTs	B08	25.466
silico DArTs	B08	23.736
silico DArTs	B08	23.081

silico DArTs	B08	22.208
silico DArTs	B08	15.808
silico DArTs	B08	15.173
silico DArTs	B08	13.327
silico DArTs	B08	12.997
silico DArTs	B08	8.199
silico DArTs	B08	5.935
silico DArTs	B08	5.656
silico DArTs	B08	4.472
silico DArTs	B08	3.123
silico DArTs	B08	1.945
silico DArTs	B08	1.478
silico DArTs	B08	1.027
silico DArTs	B08	0
silico DArTs	B07	114.403
silico DArTs	B07	108.697
silico DArTs	B07	108.024
silico DArTs	B07	107.519
silico DArTs	B07	106.933
silico DArTs	B07	106.43
silico DArTs	B07	106.149
silico DArTs	B07	105.033
silico DArTs	B07	104.519
silico DArTs	B07	104.205
silico DArTs	B07	103.881
silico DArTs	B07	103.225
silico DArTs	B07	100.635
silico DArTs	B07	100.256
silico DArTs	B07	99.926
silico DArTs	B07	99.707
silico DArTs	B07	99.139
silico DArTs	B07	98.255
silico DArTs	B07	97.924
silico DArTs	B07	97.558
SSR marker	B07	94.567
silico DArTs	B07	94.033
silico DArTs	B07	93.398
SSR marker	B07	92.823
silico DArTs	B07	92.57
silico DArTs	B07	91.912
silico DArTs	B07	82.893
SSR marker	B07	81.776
SSR marker	B07	78.049
SSR marker	B07	77.054
silico DArTs	B07	76.308
silico DArTs	B07	73.991
silico DArTs	B07	72.914

silico DArTs	B07	71.435
silico DArTs	B07	70.612
silico DArTs	B07	69.99
silico DArTs	B07	67.977
silico DArTs	B07	67.398
silico DArTs	B07	66.854
silico DArTs	B07	66.481
silico DArTs	B07	64.847
silico DArTs	B07	63.719
silico DArTs	B07	62.743
silico DArTs	B07	62.453
silico DArTs	B07	61.813
silico DArTs	B07	60.327
SSR marker	B07	59.561
silico DArTs	B07	58.82
silico DArTs	B07	57.237
silico DArTs	B07	56.474
silico DArTs	B07	55.74
silico DArTs	B07	54.443
silico DArTs	B07	53.92
silico DArTs	B07	51.904
silico DArTs	B07	51.341
silico DArTs	B07	49.579
silico DArTs	B07	49.226
silico DArTs	B07	48.973
silico DArTs	B07	48.695
silico DArTs	B07	48.264
silico DArTs	B07	47.466
silico DArTs	B07	46.964
silico DArTs	B07	46.37
silico DArTs	B07	46.022
silico DArTs	B07	36.639
silico DArTs	B07	35.769
silico DArTs	B07	35.187
silico DArTs	B07	34.901
silico DArTs	B07	34.051
silico DArTs	B07	33.62
silico DArTs	B07	33.296
SSR marker	B07	32.848
silico DArTs	B07	31.748
silico DArTs	B07	25.203
silico DArTs	B07	25.001
silico DArTs	B07	24.365
silico DArTs	B07	24.151
silico DArTs	B07	23.158
silico DArTs	B07	21.487
silico DArTs	B07	21.089

silico DArTs	B07	18.736
silico DArTs	B07	18.11
silico DArTs	B07	17.878
silico DArTs	B07	16.653
silico DArTs	B07	14.821
silico DArTs	B07	14.445
silico DArTs	B07	12.443
silico DArTs	B07	10.801
silico DArTs	B07	9.079
silico DArTs	B07	8.531
silico DArTs	B07	8.329
silico DArTs	B07	7.296
silico DArTs	B07	6.661
silico DArTs	B07	6.46
silico DArTs	B07	5.744
silico DArTs	B07	4.725
silico DArTs	B07	3.941
silico DArTs	B07	3.525
silico DArTs	B07	2.971
silico DArTs	B07	2.553
SSR marker	B07	2.133
silico DArTs	B07	1.862
silico DArTs	B07	1.424
silico DArTs	B07	0.273
silico DArTs	B07	0
silico DArTs	B06	0
silico DArTs	B06	1.684
silico DArTs	B06	1.938
silico DArTs	B06	2.271
silico DArTs	B06	2.692
silico DArTs	B06	3.488
silico DArTs	B06	3.833
silico DArTs	B06	4.198
silico DArTs	B06	4.603
silico DArTs	B06	6.389
silico DArTs	B06	6.643
silico DArTs	B06	8.614
silico DArTs	B06	9.664
SSR marker	B06	10.244
silico DArTs	B06	10.498
silico DArTs	B06	12.26
silico DArTs	B06	12.714
silico DArTs	B06	14.261
silico DArTs	B06	14.536
silico DArTs	B06	17.156
silico DArTs	B06	17.37
silico DArTs	B06	17.665

silico DArTs	B06	18.017
silico DArTs	B06	18.232
silico DArTs	B06	19.811
silico DArTs	B06	20.063
silico DArTs	B06	21.466
silico DArTs	B06	22.066
silico DArTs	B06	22.619
silico DArTs	B06	23.351
silico DArTs	B06	24.177
SSR marker	B06	24.494
silico DArTs	B06	24.944
silico DArTs	B06	25.711
silico DArTs	B06	26.52
silico DArTs	B06	28.191
silico DArTs	B06	28.525
silico DArTs	B06	29.096
silico DArTs	B06	29.625
SSR marker	B06	30.206
silico DArTs	B06	31.851
silico DArTs	B06	32.509
silico DArTs	B06	33.616
silico DArTs	B06	33.951
silico DArTs	B06	34.383
silico DArTs	B06	34.727
silico DArTs	B06	35.086
silico DArTs	B06	35.962
silico DArTs	B06	36.517
silico DArTs	B06	36.953
silico DArTs	B06	37.552
silico DArTs	B06	41.839
silico DArTs	B06	42.361
silico DArTs	B06	42.863
silico DArTs	B06	46.497
silico DArTs	B06	46.814
silico DArTs	B06	48.106
silico DArTs	B06	48.936
silico DArTs	B06	50.158
silico DArTs	B06	51.425
silico DArTs	B06	51.735
silico DArTs	B06	54.284
silico DArTs	B06	55.103
silico DArTs	B06	55.349
silico DArTs	B06	62.187
SSR marker	B06	65.206
silico DArTs	B06	65.922
silico DArTs	B06	66.294
silico DArTs	B06	66.727

silico DArTs	B06	66.94
silico DArTs	B06	67.842
silico DArTs	B06	68.384
silico DArTs	B06	77.707
silico DArTs	B06	78.614
silico DArTs	B06	81.167
silico DArTs	B06	81.394
silico DArTs	B06	83.086
silico DArTs	B06	83.693
silico DArTs	B06	85.072
silico DArTs	B06	86.641
silico DArTs	B06	87.145
silico DArTs	B06	87.509
SSR marker	B06	95.064
silico DArTs	B06	95.764
silico DArTs	B06	96.475
silico DArTs	B06	102.047
silico DArTs	B02	0
silico DArTs	B02	0.536
silico DArTs	B02	2.144
silico DArTs	B02	2.656
silico DArTs	B02	3.483
silico DArTs	B02	4.115
silico DArTs	B02	5.823
silico DArTs	B02	6.33
silico DArTs	B02	6.95
silico DArTs	B02	7.967
silico DArTs	B02	8.792
silico DArTs	B02	9.293
silico DArTs	B02	9.534
silico DArTs	B02	10.754
silico DArTs	B02	11.524
silico DArTs	B02	11.811
silico DArTs	B02	13.477
silico DArTs	B02	15.25
silico DArTs	B02	16.856
silico DArTs	B02	18.073
silico DArTs	B02	20.582
silico DArTs	B02	28.491
silico DArTs	B02	28.82
silico DArTs	B02	29.178
silico DArTs	B02	29.458
silico DArTs	B02	30.745
silico DArTs	B02	30.954
silico DArTs	B02	33.868
silico DArTs	B02	34.188
silico DArTs	B02	34.883

silico DArTs	B02	37.888
silico DArTs	B02	39.599
silico DArTs	B02	40.715
silico DArTs	B02	42.391
silico DArTs	B02	42.669
silico DArTs	B02	42.95
silico DArTs	B02	44.348
silico DArTs	B02	45.077
silico DArTs	B02	45.358
silico DArTs	B02	47.078
silico DArTs	B02	48.665
silico DArTs	B02	49.113
silico DArTs	B02	49.593
silico DArTs	B02	49.924
silico DArTs	B02	50.15
silico DArTs	B02	50.381
silico DArTs	B02	52.478
SSR marker	B02	52.993
silico DArTs	B02	53.227
silico DArTs	B02	53.533
silico DArTs	B02	54.358
silico DArTs	B02	54.85
silico DArTs	B02	59.355
silico DArTs	B02	60.113
silico DArTs	B02	60.587
silico DArTs	B02	61.755
silico DArTs	B02	66.068
silico DArTs	B02	67.535
silico DArTs	B02	68.391
silico DArTs	B02	69.068
silico DArTs	B02	70.518
silico DArTs	B02	70.927
SSR marker	B02	72.274
silico DArTs	B02	72.519
silico DArTs	B02	74.199
silico DArTs	B02	74.518
SSR marker	B02	75.529
silico DArTs	B02	76.06
silico DArTs	B02	79.384
silico DArTs	B02	79.991
silico DArTs	B02	80.877
silico DArTs	B02	81.254
silico DArTs	B02	81.569
silico DArTs	B02	82.417
silico DArTs	B02	84.161
silico DArTs	B02	87.623
silico DArTs	B02	88.602

silico DArTs	B02	93.045
silico DArTs	B02	93.838
silico DArTs	B02	98.838
silico DArTs	B02	99.19
silico DArTs	B02	102.542
SSR marker	B02	103.147
silico DArTs	B02	103.618
silico DArTs	B02	107.891
silico DArTs	B02	108.88
SSR marker	B02	109.28
silico DArTs	B02	109.533
silico DArTs	B02	109.812
silico DArTs	B02	111.109
silico DArTs	B02	112.486
silico DArTs	B02	114.717
silico DArTs	B02	115.193
silico DArTs	B02	116.024
silico DArTs	B02	116.54
silico DArTs	B02	116.86
silico DArTs	B02	118.086
silico DArTs	B02	124.349
silico DArTs	B02	124.992
silico DArTs	B02	125.902
silico DArTs	B02	135.891
silico DArTs	B02	136.921
silico DArTs	B02	137.435
silico DArTs	B02	137.841
silico DArTs	B02	139.212
silico DArTs	B02	140.552
silico DArTs	B02	140.835
silico DArTs	B02	141.39
silico DArTs	B02	141.778
silico DArTs	B02	144.322
silico DArTs	B02	145.691
silico DArTs	B02	145.929
silico DArTs	B02	146.744
silico DArTs	B02	148.237
silico DArTs	B04	0
silico DArTs	B04	0.914
silico DArTs	B04	6.316
silico DArTs	B04	6.772
silico DArTs	B04	7.169
silico DArTs	B04	8.67
silico DArTs	B04	8.887
silico DArTs	B04	9.381
silico DArTs	B04	9.891
silico DArTs	B04	12.683

silico DArTs	B04	14.489
silico DArTs	B04	15.153
silico DArTs	B04	16.031
silico DArTs	B04	16.655
silico DArTs	B04	17.622
silico DArTs	B04	20.637
silico DArTs	B04	22.24
silico DArTs	B04	22.606
silico DArTs	B04	23.308
silico DArTs	B04	26.053
SSR marker	B04	26.733
silico DArTs	B04	27.314
silico DArTs	B04	27.991
silico DArTs	B04	28.369
silico DArTs	B04	29.925
silico DArTs	B04	30.736
silico DArTs	B04	31.718
silico DArTs	B04	33.759
silico DArTs	B04	37.032
silico DArTs	B04	38.025
silico DArTs	B04	38.842
silico DArTs	B04	40.011
silico DArTs	B04	40.49
silico DArTs	B04	42.507
silico DArTs	B04	44.542
silico DArTs	B04	44.837
silico DArTs	B04	45.327
silico DArTs	B04	46.52
silico DArTs	B04	48.886
silico DArTs	B04	49.087
silico DArTs	B04	50.065
silico DArTs	B04	50.727
silico DArTs	B04	51.183
silico DArTs	B04	53.198
silico DArTs	B04	54.602
silico DArTs	B04	63.302
silico DArTs	B04	63.609
silico DArTs	B04	64.575
SSR marker	B04	65.227
silico DArTs	B04	65.6
silico DArTs	B04	65.996
SSR marker	B04	72.176
silico DArTs	B04	72.463
silico DArTs	B04	72.695
silico DArTs	B04	72.998
silico DArTs	B04	74.005
silico DArTs	B04	75.095

silico DArTs	B04	76.364
silico DArTs	B04	78.292
silico DArTs	B04	78.819
silico DArTs	B04	79.144
silico DArTs	B04	82.933
silico DArTs	B04	85.667
silico DArTs	B04	87.579
silico DArTs	B04	89.779
silico DArTs	B04	90.242
silico DArTs	B04	91.583
silico DArTs	B04	92.2
silico DArTs	B04	92.453
SSR marker	B04	92.88
silico DArTs	B04	94.528
silico DArTs	B04	94.799
silico DArTs	B04	101.811
silico DArTs	B04	103.279
SSR marker	B04	103.897
silico DArTs	B04	105.434
SSR marker	B04	106.893
silico DArTs	B04	107.616
silico DArTs	B04	109.478
silico DArTs	B04	110.564
silico DArTs	B04	117.501
silico DArTs	B04	119.321
silico DArTs	B04	121.311
silico DArTs	B04	122.073
silico DArTs	B04	122.413
silico DArTs	B04	123.996
silico DArTs	B04	124.25
silico DArTs	B04	126.516
silico DArTs	B04	126.928
silico DArTs	B04	127.87
silico DArTs	B04	128.334
silico DArTs	B04	129.563
silico DArTs	B04	129.804
silico DArTs	B04	130.268
silico DArTs	B04	132.124
silico DArTs	B04	133.295
silico DArTs	B01	92.393
silico DArTs	B01	91.048
silico DArTs	B01	90.44
silico DArTs	B01	90.234
silico DArTs	B01	89.885
silico DArTs	B01	89.591
silico DArTs	B01	89.054
silico DArTs	B01	87.979

silico DArTs	B01	86.997
silico DArTs	B01	84.643
silico DArTs	B01	84.335
silico DArTs	B01	81.982
silico DArTs	B01	81.584
silico DArTs	B01	79.341
silico DArTs	B01	78.76
silico DArTs	B01	78.514
silico DArTs	B01	78.09
silico DArTs	B01	76.873
silico DArTs	B01	76.207
silico DArTs	B01	75.853
SSR marker	B01	75.155
silico DArTs	B01	72.934
silico DArTs	B01	72.651
silico DArTs	B01	71.574
silico DArTs	B01	70.834
silico DArTs	B01	65.812
silico DArTs	B01	64.298
silico DArTs	B01	63.117
silico DArTs	B01	60.487
silico DArTs	B01	58.171
silico DArTs	B01	57.716
silico DArTs	B01	57.317
silico DArTs	B01	56.283
silico DArTs	B01	52.676
silico DArTs	B01	52.362
silico DArTs	B01	51.702
silico DArTs	B01	51.057
silico DArTs	B01	50.204
silico DArTs	B01	49.497
silico DArTs	B01	49.084
silico DArTs	B01	48.271
silico DArTs	B01	48.07
silico DArTs	B01	47.314
silico DArTs	B01	40.911
silico DArTs	B01	39.876
SSR marker	B01	38.9
silico DArTs	B01	38.515
silico DArTs	B01	38.189
silico DArTs	B01	37.945
silico DArTs	B01	37.135
silico DArTs	B01	35.984
silico DArTs	B01	28.774
SSR marker	B01	28.526
silico DArTs	B01	28.265
silico DArTs	B01	27.728

silico DArTs	B01	27.14
silico DArTs	B01	22.622
silico DArTs	B01	21.71
silico DArTs	B01	20.15
silico DArTs	B01	19.929
silico DArTs	B01	19.362
silico DArTs	B01	18.961
SSR marker	B01	14.413
silico DArTs	B01	14.211
silico DArTs	B01	13.479
silico DArTs	B01	13.252
silico DArTs	B01	12.763
silico DArTs	B01	11.843
silico DArTs	B01	11.256
silico DArTs	B01	10.829
silico DArTs	B01	10.198
silico DArTs	B01	9.855
silico DArTs	B01	9.324
silico DArTs	B01	8.929
silico DArTs	B01	8.649
silico DArTs	B01	7.452
silico DArTs	B01	7.223
silico DArTs	B01	6.38
silico DArTs	B01	6.162
silico DArTs	B01	5.915
silico DArTs	B01	5.641
silico DArTs	B01	4.877
silico DArTs	B01	4.237
silico DArTs	B01	0
silico DArTs	B03	0
silico DArTs	B03	1.487
silico DArTs	B03	3.277
silico DArTs	B03	4.107
silico DArTs	B03	5.107
silico DArTs	B03	7.217
silico DArTs	B03	9.818
silico DArTs	B03	10.62
silico DArTs	B03	11.07
silico DArTs	B03	12.119
silico DArTs	B03	12.467
silico DArTs	B03	12.779
silico DArTs	B03	12.985
silico DArTs	B03	14.327
silico DArTs	B03	17.931
silico DArTs	B03	18.41
silico DArTs	B03	19.01
silico DArTs	B03	26.335

silico DArTs	B03	26.71
silico DArTs	B03	36.076
SSR marker	B03	37.639
silico DArTs	B03	38.363
silico DArTs	B03	41.752
silico DArTs	B03	43.817
silico DArTs	B03	45.291
silico DArTs	B03	45.723
silico DArTs	B03	47.315
silico DArTs	B03	48.748
silico DArTs	B03	52.32
silico DArTs	B03	53.24
silico DArTs	B03	56.36
silico DArTs	B03	57.107
silico DArTs	B03	57.621
silico DArTs	B03	58.435
silico DArTs	B03	63.347
silico DArTs	B03	63.749
silico DArTs	B03	64.243
silico DArTs	B03	65.617
silico DArTs	B03	69.161
silico DArTs	B03	70.701
silico DArTs	B03	71.497
silico DArTs	B03	76.443
silico DArTs	B03	77.706
silico DArTs	B03	78.084
silico DArTs	B03	81.529
silico DArTs	B03	81.983
silico DArTs	B03	82.621
silico DArTs	B03	83.308
silico DArTs	B03	83.902
silico DArTs	B03	85.208
silico DArTs	B03	86.437
silico DArTs	B03	86.881
silico DArTs	B03	87.236
silico DArTs	B03	87.585
silico DArTs	B03	87.893
silico DArTs	B03	88.535
silico DArTs	B03	89.024
silico DArTs	B03	91.142
silico DArTs	B03	91.789
silico DArTs	B03	95.296
silico DArTs	B03	95.982
silico DArTs	B03	96.399
silico DArTs	B03	100.003
silico DArTs	B03	100.468
silico DArTs	B03	100.748

silico DArTs	B03	102.888
silico DArTs	B03	103.091
silico DArTs	B03	104.755
SSR marker	B03	105.676
silico DArTs	B03	106.395
silico DArTs	B03	109.279
silico DArTs	B03	110.247
silico DArTs	B03	110.547
silico DArTs	B03	111.381
silico DArTs	B03	111.928
silico DArTs	B03	112.725
silico DArTs	B03	113.616
silico DArTs	B03	114.092
silico DArTs	B03	114.521
silico DArTs	B03	115.027
silico DArTs	B03	115.548
silico DArTs	B03	116.494
silico DArTs	B03	117.081
silico DArTs	B03	117.979
SSR marker	B03	120.039
silico DArTs	B03	121.087
silico DArTs	B03	121.547
silico DArTs	B03	122.624
silico DArTs	B03	123.452
silico DArTs	B03	125.962
silico DArTs	B03	126.266
silico DArTs	B03	126.498
silico DArTs	B03	127.045
silico DArTs	B03	127.434
silico DArTs	B03	127.758
silico DArTs	B03	129.26
silico DArTs	B03	129.973
silico DArTs	B03	132.473
silico DArTs	B03	133.266
silico DArTs	B03	134.234
silico DArTs	B03	134.45
silico DArTs	B03	134.782
silico DArTs	B03	135.306
silico DArTs	B03	137.111
silico DArTs	B03	138.481
silico DArTs	B03	139.739
silico DArTs	B03	140.02
silico DArTs	B03	140.607
silico DArTs	B03	141
silico DArTs	B03	141.625
silico DArTs	B03	142.166
silico DArTs	B03	143.875

silico DArTs	B03	144.285
silico DArTs	B03	144.561
silico DArTs	B03	145.882
silico DArTs	B03	146.655
silico DArTs	B03	147.047
silico DArTs	B03	147.844
silico DArTs	B03	148.143
silico DArTs	B03	148.694
SSR marker	C01	0
silico DArTs	C01	7.818
silico DArTs	C01	16.456
SSR marker	C01	19.393
silico DArTs	C01	26.279
silico DArTs	C01	30.205
silico DArTs	C01	31.956
silico DArTs	C01	34.367
silico DArTs	C01	36.165
silico DArTs	C01	36.372
silico DArTs	C01	37.881
silico DArTs	C01	38.216
silico DArTs	C01	42.377
SSR marker	C01	43.049
silico DArTs	C01	44.654
silico DArTs	C01	45.639
silico DArTs	C01	48.72
silico DArTs	C01	49.715
silico DArTs	C01	50.325
silico DArTs	C01	50.911
silico DArTs	C01	51.405
silico DArTs	C01	53.854
SSR marker	C01	54.342
silico DArTs	C01	54.739
silico DArTs	C01	58.398
silico DArTs	C01	61.702
SSR marker	C01	62.348
SSR marker	C01	62.901
silico DArTs	C01	63.861
SSR marker	C01	64.734
silico DArTs	C01	67.745
SSR marker	C01	68.35
silico DArTs	C01	70.498
silico DArTs	C01	70.814
SSR marker	C01	76.105
silico DArTs	C01	87.704
silico DArTs	C01	89.799
silico DArTs	C01	98.736
silico DArTs	C01	99.205

silico DArTs	C01	100.574
silico DArTs	C01	103.132
silico DArTs	C01	103.947
silico DArTs	C01	106.982
silico DArTs	C01	108.165
silico DArTs	C01	108.756
silico DArTs	C01	109.467
silico DArTs	C01	114.255
SSR marker	C01	120.717
silico DArTs	C02	0
silico DArTs	C02	3.499
silico DArTs	C02	3.774
silico DArTs	C02	4.204
silico DArTs	C02	4.636
silico DArTs	C02	6.433
silico DArTs	C02	7.955
silico DArTs	C02	12.436
silico DArTs	C02	23.022
silico DArTs	C02	37.177
silico DArTs	C02	37.913
silico DArTs	C02	40.556
SSR marker	C02	40.894
silico DArTs	C02	42.012
silico DArTs	C02	42.932
silico DArTs	C02	43.594
silico DArTs	C02	43.854
silico DArTs	C02	46.858
silico DArTs	C02	48.282
silico DArTs	C02	48.434
SSR marker	C02	50.645
silico DArTs	C02	65.511
silico DArTs	C02	66.001
silico DArTs	C02	69.244
silico DArTs	C02	70.629
silico DArTs	C02	79.072
SSR marker	C02	80.144
SSR marker	C02	80.531
SSR marker	C02	85.35
silico DArTs	C02	88.559
silico DArTs	C02	89.139
silico DArTs	C02	90.269
silico DArTs	C02	90.944
silico DArTs	C02	91.356
silico DArTs	C02	91.807
silico DArTs	C02	94.12
SSR marker	C02	101.024
silico DArTs	C02	115.53

SSR marker	C02	116.266
silico DArTs	C02	117.215
silico DArTs	C02	121.683
silico DArTs	C03	168.045
silico DArTs	C03	165.788
silico DArTs	C03	164.756
silico DArTs	C03	164.348
silico DArTs	C03	163.775
silico DArTs	C03	162.017
silico DArTs	C03	157.097
silico DArTs	C03	156.686
silico DArTs	C03	156.471
silico DArTs	C03	145.052
silico DArTs	C03	144.013
silico DArTs	C03	141.328
silico DArTs	C03	140.921
silico DArTs	C03	131.877
silico DArTs	C03	131.38
silico DArTs	C03	130.681
silico DArTs	C03	125.533
SSR marker	C03	125.175
silico DArTs	C03	123.83
SSR marker	C03	123.2
silico DArTs	C03	119.244
silico DArTs	C03	116.84
silico DArTs	C03	113.132
silico DArTs	C03	112.563
silico DArTs	C03	111.794
silico DArTs	C03	111.102
silico DArTs	C03	110.365
SSR marker	C03	109.972
silico DArTs	C03	108.835
silico DArTs	C03	106.533
silico DArTs	C03	105.656
silico DArTs	C03	103.534
silico DArTs	C03	103.082
silico DArTs	C03	102.635
silico DArTs	C03	100.811
silico DArTs	C03	91.75
silico DArTs	C03	91.129
silico DArTs	C03	88.423
silico DArTs	C03	88.108
silico DArTs	C03	87.733
silico DArTs	C03	83.492
silico DArTs	C03	82.586
silico DArTs	C03	81.787
SSR marker	C03	80.758

silico DArTs	C03	80.068
silico DArTs	C03	79.647
silico DArTs	C03	77.448
silico DArTs	C03	76.712
silico DArTs	C03	76.049
SSR marker	C03	74.751
silico DArTs	C03	72.327
silico DArTs	C03	71.764
silico DArTs	C03	71.342
silico DArTs	C03	70.056
silico DArTs	C03	68.243
silico DArTs	C03	68.005
silico DArTs	C03	67.599
silico DArTs	C03	65.915
silico DArTs	C03	65.144
silico DArTs	C03	64.915
silico DArTs	C03	64.669
silico DArTs	C03	63.428
silico DArTs	C03	61.9
silico DArTs	C03	61.071
silico DArTs	C03	60.851
silico DArTs	C03	60.501
silico DArTs	C03	60.26
silico DArTs	C03	58.304
silico DArTs	C03	57.62
silico DArTs	C03	47.197
silico DArTs	C03	46.621
silico DArTs	C03	46.202
silico DArTs	C03	45.982
silico DArTs	C03	45.596
SSR marker	C03	45.391
SSR marker	C03	44.936
silico DArTs	C03	36.856
silico DArTs	C03	36.539
silico DArTs	C03	32.347
silico DArTs	C03	32.107
silico DArTs	C03	31.749
silico DArTs	C03	31.495
silico DArTs	C03	31.215
silico DArTs	C03	27.455
silico DArTs	C03	26.834
silico DArTs	C03	26.482
silico DArTs	C03	24.412
silico DArTs	C03	20.385
SSR marker	C03	18.205
silico DArTs	C03	15.267
silico DArTs	C03	14.95

silico DArTs	C03	14.599
silico DArTs	C03	12.404
silico DArTs	C03	11.905
silico DArTs	C03	11.598
silico DArTs	C03	8.355
silico DArTs	C03	8.143
silico DArTs	C03	7.197
SSR marker	C03	4.184
silico DArTs	C03	3.344
silico DArTs	C03	2.291
silico DArTs	C03	1.365
silico DArTs	C03	1.042
silico DArTs	C03	0
silico DArTs	C04	145.33
silico DArTs	C04	142.447
silico DArTs	C04	142.243
silico DArTs	C04	141.809
silico DArTs	C04	134.899
silico DArTs	C04	134.195
silico DArTs	C04	128.91
SSR marker	C04	122.062
silico DArTs	C04	120.811
silico DArTs	C04	120.48
silico DArTs	C04	116.418
silico DArTs	C04	112.956
silico DArTs	C04	112.225
silico DArTs	C04	111.963
silico DArTs	C04	111.071
silico DArTs	C04	110.481
silico DArTs	C04	91.064
silico DArTs	C04	90.634
silico DArTs	C04	89.63
silico DArTs	C04	88.751
silico DArTs	C04	88.498
SSR marker	C04	85.847
silico DArTs	C04	84.871
silico DArTs	C04	83.784
silico DArTs	C04	75.069
silico DArTs	C04	74.304
silico DArTs	C04	73.808
silico DArTs	C04	73.085
SSR marker	C04	71.389
silico DArTs	C04	61.139
SSR marker	C04	54.366
silico DArTs	C04	53.233
silico DArTs	C04	45.819
silico DArTs	C04	45.469

silico DArTs	C04	44.289
silico DArTs	C04	40.159
silico DArTs	C04	24.421
silico DArTs	C04	24
silico DArTs	C04	23.632
SSR marker	C04	22.843
silico DArTs	C04	13.348
silico DArTs	C04	11.364
silico DArTs	C04	10.024
silico DArTs	C04	9.804
silico DArTs	C04	9.6
silico DArTs	C04	7.578
silico DArTs	C04	4.879
SSR marker	C04	4.106
SSR marker	C04	1.962
silico DArTs	C04	1.523
silico DArTs	C04	0
silico DArTs	C05	123.88
silico DArTs	C05	122.277
silico DArTs	C05	119.728
silico DArTs	C05	114.826
silico DArTs	C05	108.145
silico DArTs	C05	102.455
silico DArTs	C05	95.525
silico DArTs	C05	92.05
silico DArTs	C05	91.604
silico DArTs	C05	88.235
SSR marker	C05	79.445
SSR marker	C05	76.745
SSR marker	C05	76.309
silico DArTs	C05	75.948
SSR marker	C05	75.028
silico DArTs	C05	68.588
silico DArTs	C05	66.839
silico DArTs	C05	65.949
silico DArTs	C05	65.346
silico DArTs	C05	64.692
silico DArTs	C05	60.141
silico DArTs	C05	59.537
silico DArTs	C05	56.035
silico DArTs	C05	55.338
silico DArTs	C05	54.854
SSR marker	C05	54.617
silico DArTs	C05	52.208
SSR marker	C05	50.484
silico DArTs	C05	42.265
silico DArTs	C05	41.534

silico DArTs	C05	41.076
SSR marker	C05	37
silico DArTs	C05	29.615
silico DArTs	C05	27.607
silico DArTs	C05	26.864
silico DArTs	C05	25.695
silico DArTs	C05	24.764
silico DArTs	C05	17.863
SSR marker	C05	14.422
silico DArTs	C05	10.029
SSR marker	C05	2.015
silico DArTs	C05	0.979
silico DArTs	C05	0
silico DArTs	C06	104.931
silico DArTs	C06	103.73
SSR marker	C06	87.449
silico DArTs	C06	83.078
silico DArTs	C06	82.654
silico DArTs	C06	80.084
silico DArTs	C06	78.087
SSR marker	C06	77.269
silico DArTs	C06	76.664
silico DArTs	C06	75.571
silico DArTs	C06	72.848
SSR marker	C06	71.07
silico DArTs	C06	70.24
SSR marker	C06	68.192
silico DArTs	C06	60.715
SSR marker	C06	59.702
silico DArTs	C06	59.165
SSR marker	C06	57.433
SSR marker	C06	57.007
silico DArTs	C06	56.037
silico DArTs	C06	55.629
silico DArTs	C06	50.704
silico DArTs	C06	49.732
silico DArTs	C06	49.139
silico DArTs	C06	47.471
silico DArTs	C06	45.858
silico DArTs	C06	44.334
silico DArTs	C06	43.203
silico DArTs	C06	42.861
silico DArTs	C06	42.069
silico DArTs	C06	39.731
silico DArTs	C06	39.254
silico DArTs	C06	38.894
SSR marker	C06	37.015

silico DArTs	C06	35.61
silico DArTs	C06	35.043
silico DArTs	C06	33.938
silico DArTs	C06	33.2
silico DArTs	C06	28.053
silico DArTs	C06	25.941
silico DArTs	C06	23.725
silico DArTs	C06	22.839
silico DArTs	C06	21.08
silico DArTs	C06	18.506
silico DArTs	C06	18.134
silico DArTs	C06	17.123
SSR marker	C06	16.269
silico DArTs	C06	14.556
silico DArTs	C06	13.453
silico DArTs	C06	12.826
silico DArTs	C06	10.988
silico DArTs	C06	10.722
silico DArTs	C06	8.919
silico DArTs	C06	8.397
silico DArTs	C06	7.294
silico DArTs	C06	6.008
silico DArTs	C06	3.055
silico DArTs	C06	1.141
silico DArTs	C06	0
silico DArTs	C07	102.277
silico DArTs	C07	100.635
silico DArTs	C07	97.556
silico DArTs	C07	94.091
silico DArTs	C07	93.771
silico DArTs	C07	93.418
silico DArTs	C07	92.988
silico DArTs	C07	88.071
silico DArTs	C07	87.607
silico DArTs	C07	86.416
silico DArTs	C07	81.849
silico DArTs	C07	81.28
silico DArTs	C07	80.589
silico DArTs	C07	80.301
silico DArTs	C07	79.447
silico DArTs	C07	76.266
silico DArTs	C07	71.493
silico DArTs	C07	70.827
silico DArTs	C07	69.361
silico DArTs	C07	68.646
silico DArTs	C07	63.324
silico DArTs	C07	63.046

silico DArTs	C07	61.836
silico DArTs	C07	58.812
silico DArTs	C07	58.43
silico DArTs	C07	56.647
SSR marker	C07	51.693
silico DArTs	C07	51.141
silico DArTs	C07	50.009
silico DArTs	C07	49.668
silico DArTs	C07	48.073
silico DArTs	C07	44.545
silico DArTs	C07	41.088
silico DArTs	C07	40.338
silico DArTs	C07	40.019
SSR marker	C07	38.556
silico DArTs	C07	35.863
SSR marker	C07	35.146
silico DArTs	C07	33.65
silico DArTs	C07	28.743
silico DArTs	C07	27.737
SSR marker	C07	26.165
silico DArTs	C07	22.251
silico DArTs	C07	19.801
silico DArTs	C07	18.403
silico DArTs	C07	16.84
silico DArTs	C07	13.698
silico DArTs	C07	12.613
SSR marker	C07	12.128
silico DArTs	C07	11.186
SSR marker	C07	10.106
silico DArTs	C07	9.537
SSR marker	C07	8.966
silico DArTs	C07	8.226
SSR marker	C07	6.708
silico DArTs	C07	6.358
silico DArTs	C07	6.085
silico DArTs	C07	4.312
SSR marker	C07	3.939
silico DArTs	C07	2.741
silico DArTs	C07	1.106
silico DArTs	C07	0
SSR marker	C08	0
silico DArTs	C08	1.495
silico DArTs	C08	2.052
silico DArTs	C08	2.745
silico DArTs	C08	6.069
silico DArTs	C08	7.247
SSR marker	C08	10.013

silico DArTs	C08	12.133
silico DArTs	C08	12.605
SSR marker	C08	22.058
silico DArTs	C08	22.922
silico DArTs	C08	26.896
silico DArTs	C08	27.45
silico DArTs	C08	27.97
silico DArTs	C08	28.278
silico DArTs	C08	28.912
silico DArTs	C08	30.146
silico DArTs	C08	34.278
silico DArTs	C08	34.684
silico DArTs	C08	35.15
silico DArTs	C08	35.928
silico DArTs	C08	40.736
silico DArTs	C08	41.318
silico DArTs	C08	41.693
silico DArTs	C08	42.099
silico DArTs	C08	42.425
silico DArTs	C08	43.058
SSR marker	C08	43.519
silico DArTs	C08	48.13
silico DArTs	C08	48.688
silico DArTs	C08	49.132
silico DArTs	C08	51.31
silico DArTs	C08	55.774
silico DArTs	C08	58.734
silico DArTs	C08	61.64
silico DArTs	C08	62.528
silico DArTs	C08	62.809
silico DArTs	C08	63.49
silico DArTs	C08	63.999
silico DArTs	C08	64.561
silico DArTs	C08	69.283
silico DArTs	C08	69.676
silico DArTs	C08	71.017
silico DArTs	C08	71.741
silico DArTs	C08	73.139
silico DArTs	C08	73.493
SSR marker	C08	77.073
silico DArTs	C08	77.495
SSR marker	C08	80.419
SSR marker	C08	80.975
silico DArTs	C08	81.703
silico DArTs	C08	85.51
silico DArTs	C08	86.515
silico DArTs	C08	87.332

silico DArTs	C08	88.455
SSR marker	C08	89.212
SSR marker	C08	89.933
silico DArTs	C08	90.345
SNP marker	C09	0
SNP marker	C09	1.126
SSR marker	C09	2.242
silico DArTs	C09	3.582
silico DArTs	C09	3.649
silico DArTs	C09	4.435
silico DArTs	C09	5.322
SNP marker	C09	11.6
SSR marker	C09	13.449
silico DArTs	C09	14.115
silico DArTs	C09	15.509
silico DArTs	C09	16.325
silico DArTs	C09	17.481
silico DArTs	C09	17.719
silico DArTs	C09	18.328
silico DArTs	C09	18.858
silico DArTs	C09	20.005
SNP marker	C09	20.542
SSR marker	C09	21.588
SNP marker	C09	26.998
silico DArTs	C09	27.461
silico DArTs	C09	28.312
silico DArTs	C09	28.52
silico DArTs	C09	31.718
silico DArTs	C09	32.236
silico DArTs	C09	33.885
silico DArTs	C09	34.442
SNP marker	C09	36.621
silico DArTs	C09	38.906
silico DArTs	C09	40.633
silico DArTs	C09	44.215
silico DArTs	C09	44.979
silico DArTs	C09	50.682
silico DArTs	C09	51.457
silico DArTs	C09	51.913
silico DArTs	C09	54.979
SNP marker	C09	55.141
silico DArTs	C09	55.669
SNP marker	C09	57.546
SSR marker	C09	62.03
SSR marker	C09	65.37
SNP marker	C09	77.319
silico DArTs	C09	100.13

silico DArTs	C09	103.457
silico DArTs	C09	103.9
SNP marker	C09	104.289
silico DArTs	C09	104.831
SSR marker	C09	105.376
silico DArTs	C09	106.454
silico DArTs	C09	106.806
SNP marker	C09	111.332
silico DArTs	C09	112.73
silico DArTs	C09	115.651

Table S4C. Collinearity between the genetic linkage map map of the YWDH populatio			
Marker	Chromosome Genetic map	Position of marker on the linkage map	Chromosome of *B. carinata* pan-genome
100022880	B01	0.467	B01
100020359	B01	1.282	B01
100020555	B01	1.776	B01
100026892	B01	3.392	B01
100027568	B01	6.41	B01
100070796	B01	11	B01
100037316	B01	12.744	B01
100017800	B01	14.033	B01
100043041	B01	14.417	B01
100022192	B01	15.114	B01
100057916	B01	18.696	B01
100059174	B01	19.268	B01
100005563	B01	19.615	B01
100015929	B01	20.429	B01
100056897	B01	20.746	B01
100038153	B01	21.83	B01
100032903	B01	23.452	B01
100074449	B01	23.849	B01
100010952	B01	24.391	B01
100010967	B01	25.657	B01
100074919	B01	27.151	B01
100020285	B01	27.558	B01
100019234	B01	28.236	B01
100028078	B01	29.54	B01
100003585	B01	32.291	B01
100033169	B01	32.698	B01
100032315	B01	32.982	B01
100049168	B01	33.314	B01
100079097	B01	36.696	B01
100065962	B01	38.606	B01
100018102	B01	39.284	B01
100012485	B01	39.686	B01
100020968	B01	40.563	B01
100072282	B01	40.936	B01
100019767	B01	41.588	B01
100058331	B01	42.01	B01

100076763	B01	42.369	B01
100044445	B01	42.583	B01
100042788	B01	43.914	B01
100035017	B01	44.921	B01
100021620	B01	46.482	B01
100006932	B01	47.282	B01
100060279	B01	48.651	B01
100064011	B01	49.898	B01
100023279	B01	50.15	B01
100037087	B01	50.698	B01
100027521	B01	51.038	B01
100055615	B01	53.495	B01
100075320	B01	55.192	B01
100045734	B01	55.427	B01
100007720	B01	57.554	B01
100062079	B01	58.267	B01
100001950	B01	59.341	B01
100068483	B01	60.564	B01
100068647	B01	60.863	B01
100021654	B01	61.934	B01
100007356	B01	64.767	B01
100053774	B01	65.97	B01
100030317	B01	66.453	B01
100056875	B01	67.724	B01
100029966	B01	68.095	B01
100066618	B01	68.656	B01
100065378	B01	69.191	B01
100052792	B01	69.82	B01
100023500	B01	70.339	B01
100011539	B01	71.033	B01
100045081	B01	71.353	B01
100074374	B01	72.364	B01
100057816	B01	72.846	B01
100038238	B01	74.697	B01
100033817	B01	75.06	B01
100011722	B01	78.271	B01
100009934	B01	78.48	B01
100068765	B01	78.92	B01
100021449	B01	79.908	B01
100011424	B01	83.494	B01

100022091	B01	83.858	B01
100023650	B01	84.227	B01
100031276	B01	84.584	B01
100020829	B01	85.041	B01
100074174	B01	86.725	B01
100011531	B01	87.147	B01
100028912	B01	88.297	B01
100065423	B01	89.891	B01
100041944	B01	90.217	B01
100076031	B01	90.73	B01
100022847	B01	92.26	B01
100026299	B01	93.073	B01
100042745	B01	96.216	B01
100007647	B01	97.215	B01
100028246	B01	97.593	B01
100011937	B01	98.511	B01
100007094	B01	98.966	B01
100029508	B01	105.379	B01
100011720	B01	109.146	B01
100022564	B01	109.451	B01
100015664	B01	111.618	B01
100079009	B02	0	B02
100028870	B02	1.027	B02
100016304	B02	1.478	B02
100073662	B02	1.945	B02
100058792	B02	3.123	B02
100009089	B02	5.656	B02
100046847	B02	5.935	B02
100065428	B02	8.199	B02
100004158	B02	12.997	B02
100021631	B02	13.327	B02
100036555	B02	15.173	B02
100025417	B02	15.808	B02
100062911	B02	22.208	B02
100004474	B02	23.081	B02
100010335	B02	23.736	B02
100021125	B02	29.45	B02
100011688	B02	31.523	B02
100017191	B02	32.34	B02
100078223	B02	33.129	B02

100024199	B02	33.657	B02
100015907	B02	36.814	B02
100033238	B02	49.116	B02
100033088	B02	49.58	B02
100019209	B02	50.366	B02
100043699	B02	60.58	B02
100028536	B02	61.249	B02
100033072	B02	61.824	B02
100014607	B02	62.199	B02
100034017	B02	64.009	B02
100033564	B02	64.364	B02
100007738	B02	65.134	B02
100030187	B02	65.654	B02
100014832	B02	66.65	B02
100020741	B02	69.058	B02
100017376	B02	69.82	B02
100030316	B02	71.748	B02
100039552	B02	71.963	B02
100018753	B02	74.651	B02
100035942	B02	76.218	B02
100015255	B02	76.648	B02
100059951	B02	77.27	B02
100011210	B02	78.235	B02
100053506	B02	78.648	B02
100061890	B02	82.914	B02
100002468	B02	83.651	B02
100055875	B02	84.293	B02
100052047	B02	85.043	B02
100023396	B02	85.401	B02
100019274	B02	86.427	B02
100025883	B02	87.232	B02
100018326	B02	87.647	B02
100013920	B02	88.583	B02
100067960	B02	90.027	B02
100031789	B02	90.482	B02
100019364	B02	91.18	B02
100019770	B02	91.596	B02
100076228	B02	92.115	B02
100028417	B02	93.108	B02
100030404	B02	93.656	B02

100071863	B02	94.822	B02
100031473	B02	95.395	B02
100034989	B02	96.532	B02
100015084	B02	98.822	B02
100006783	B02	99.381	B02
100030184	B02	99.589	B02
100033667	B02	99.97	B02
100035893	B02	101.068	B02
100055771	B02	103.336	B02
100037990	B02	103.653	B02
100012921	B02	105.588	B02
100053548	B02	106.665	B02
100066360	B02	107.347	B02
100060834	B02	108.392	B02
100061759	B02	108.648	B02
100007358	B02	109.229	B02
100062862	B02	110.262	B02
100008811	B02	112.068	B02
100033395	B02	113.325	B02
100070790	B02	114.439	B02
100033803	B02	115.561	B02
100047102	B02	116.428	B02
100031592	B02	116.725	B02
100020984	B02	117.293	B02
100017911	B02	117.523	B02
100005762	B02	120.086	B02
100015389	B02	120.757	B02
100039342	B02	121.07	B02
100065908	B02	122.29	B02
100038821	B02	123.493	B02
100015262	B02	124.651	B02
100021279	B02	124.936	B02
100018709	B02	125.57	B02
100021088	B02	125.937	B02
100021683	B02	126.34	B02
100033412	B02	127.522	B02
100044738	B02	128.259	B02
100061546	B02	128.95	B02
100042137	B02	129.262	B02
100044338	B02	129.707	B02

100051017	B02	130.912	B02
100007659	B03	0	B03
100038041	B03	1.424	B03
100059045	B03	1.862	B03
100007671	B03	2.553	B03
100021203	B03	2.971	B03
100017886	B03	3.525	B03
100031742	B03	3.941	B03
100053349	B03	4.725	B03
100018506	B03	5.744	B03
100037601	B03	6.46	B03
100015346	B03	6.661	B03
100006506	B03	7.296	B03
100008476	B03	8.329	B03
100063129	B03	8.531	B03
100017676	B03	9.079	B03
100023051	B03	10.801	B03
100015321	B03	12.443	B03
100049231	B03	14.445	B03
100014480	B03	14.821	B03
100032507	B03	16.653	B03
100068721	B03	18.11	B03
100061126	B03	21.089	B03
100005111	B03	21.487	B03
100037967	B03	23.158	B03
100051178	B03	24.365	B03
100019422	B03	25.203	B03
100051571	B03	31.748	B03
100066523	B03	33.62	B03
100015887	B03	34.051	B03
100006934	B03	34.901	B03
100058090	B03	35.187	B03
100055593	B03	35.769	B03
100032410	B03	36.639	B03
100059607	B03	46.022	B03
100049714	B03	46.37	B03
100010398	B03	46.964	B03
100012533	B03	47.466	B03
100053100	B03	48.264	B03
100069322	B03	48.695	B03

100023110	B03	48.973	B03
100020332	B03	49.226	B03
100019214	B03	49.579	B03
100007482	B03	51.341	B03
100057070	B03	51.904	B03
100036978	B03	53.92	B03
100033366	B03	54.443	B03
100020485	B03	55.74	B03
100010187	B03	56.474	B03
100049303	B03	57.237	B03
100037068	B03	58.82	B03
100019505	B03	60.327	B03
100007683	B03	61.813	B03
100077998	B03	62.453	B03
100031234	B03	62.743	B03
100008064	B03	63.719	B03
100042030	B03	66.481	B03
100047311	B03	66.854	B03
100008060	B03	67.398	B03
100036414	B03	67.977	B03
100013867	B03	69.99	B03
100025623	B03	70.612	B03
100008558	B03	71.435	B03
100036541	B03	72.914	B03
100018762	B03	73.991	B03
100015060	B03	76.308	B03
100014967	B03	82.893	B03
100005456	B03	93.398	B03
100062790	B03	94.033	B03
100045717	B03	97.558	B03
100020458	B03	97.924	B03
100018494	B03	98.255	B03
100028847	B03	99.139	B03
100030569	B03	99.707	B03
100027570	B03	99.926	B03
100052281	B03	100.256	B03
100022962	B03	100.635	B03
100046407	B03	103.881	B03
100015958	B03	104.205	B03
100030337	B03	104.519	B03

100027812	B03	105.033	B03
100068882	B03	106.149	B03
100050410	B03	106.43	B03
100015241	B03	106.933	B03
100052506	B03	107.519	B03
100025223	B03	108.024	B03
100039722	B03	108.697	B03
100023163	B03	114.403	B03
100017760	B04	0	B04
100036472	B04	1.684	B04
100008766	B04	1.938	B04
100020283	B04	2.271	B04
100015838	B04	2.692	B04
100039347	B04	3.488	B04
100015993	B04	3.833	B04
100067992	B04	4.198	B04
100067298	B04	4.603	B04
100025042	B04	6.389	B04
100009809	B04	6.643	B04
100018123	B04	8.614	B04
100079041	B04	9.664	B04
100022276	B04	10.498	B04
100054173	B04	12.26	B04
100054045	B04	12.714	B04
100031202	B04	14.261	B04
100075756	B04	14.536	B04
100075230	B04	17.156	B04
100001486	B04	17.37	B04
100047241	B04	18.017	B04
100004826	B04	18.232	B04
100007658	B04	19.811	B04
100029796	B04	21.466	B04
100063786	B04	22.066	B04
100019406	B04	23.351	B04
100019362	B04	24.177	B04
100066297	B04	25.711	B04
100031644	B04	26.52	B04
100024244	B04	28.191	B04
100024197	B04	28.525	B04
100024396	B04	29.096	B04

100048523	B04	29.625	B04
100024742	B04	31.851	B04
100045241	B04	32.509	B04
100006091	B04	33.616	B04
100078929	B04	34.383	B04
100067211	B04	34.727	B04
100021822	B04	35.086	B04
100070304	B04	35.962	B04
100018961	B04	36.517	B04
100013010	B04	36.953	B04
100027205	B04	37.552	B04
100055360	B04	41.839	B04
100005371	B04	42.361	B04
100012506	B04	42.863	B04
100027477	B04	46.497	B04
100066621	B04	46.814	B04
100016370	B04	48.106	B04
100019266	B04	48.936	B04
100030248	B04	50.158	B04
100030775	B04	51.425	B04
100025201	B04	51.735	B04
100025683	B04	54.284	B04
100014203	B04	55.103	B04
100012793	B04	55.349	B04
100048347	B04	62.187	B04
100026006	B04	65.922	B04
100025622	B04	66.294	B04
100011867	B04	66.727	B04
100057157	B04	66.94	B04
100049162	B04	67.842	B04
100050354	B04	77.707	B04
100012372	B04	78.614	B04
100023256	B04	81.167	B04
100074521	B04	81.394	B04
100013001	B04	83.086	B04
100009827	B04	83.693	B04
100024947	B04	85.072	B04
100029954	B04	86.641	B04
100021199	B04	87.145	B04
100021508	B04	87.509	B04

100017284	B04	95.764	B04
100037214	B04	96.475	B04
100022699	B04	102.047	B04
100050773	B05	0	B05
100005245	B05	0.536	B05
100023735	B05	2.144	B05
100016421	B05	2.656	B05
100064697	B05	3.483	B05
100008226	B05	4.115	B05
100007307	B05	5.823	B05
100045111	B05	6.33	B05
100060363	B05	6.95	B05
100012220	B05	7.967	B05
100018012	B05	8.792	B05
100023940	B05	9.293	B05
100028569	B05	9.534	B05
100031847	B05	10.754	B05
100041563	B05	11.524	B05
100027247	B05	11.811	B05
100028171	B05	13.477	B05
100002981	B05	15.25	B05
100040381	B05	16.856	B05
100013734	B05	18.073	B05
100016187	B05	20.582	B05
100053387	B05	28.82	B05
100059009	B05	29.178	B05
100007386	B05	29.458	B05
100023391	B05	30.745	B05
100004789	B05	30.954	B05
100047433	B05	33.868	B05
100026480	B05	34.188	B05
100055106	B05	34.883	B05
100026976	B05	37.888	B05
100004548	B05	39.599	B05
100015527	B05	40.715	B05
100025898	B05	42.391	B05
100043823	B05	42.669	B05
100016777	B05	44.348	B05
100019799	B05	45.077	B05
100021480	B05	45.358	B05

100059161	B05	47.078	B05
100027954	B05	48.665	B05
100031574	B05	49.593	B05
100015179	B05	49.924	B05
100064401	B05	50.15	B05
100038131	B05	50.381	B05
100021699	B05	52.478	B05
100038759	B05	53.227	B05
100062128	B05	53.533	B05
100045707	B05	54.358	B05
100059844	B05	54.85	B05
100012301	B05	59.355	B05
100043931	B05	60.113	B05
100036573	B05	60.587	B05
100033950	B05	61.755	B05
100060295	B05	66.068	B05
100032396	B05	69.068	B05
100024893	B05	70.518	B05
100027389	B05	70.927	B05
100012892	B05	72.519	B05
100016639	B05	74.199	B05
100001282	B05	74.518	B05
100021083	B05	76.06	B05
100027670	B05	79.384	B05
100070363	B05	79.991	B05
100048879	B05	80.877	B05
100016457	B05	81.254	B05
100005674	B05	81.569	B05
100028204	B05	82.417	B05
100032373	B05	84.161	B05
100020956	B05	87.623	B05
100016893	B05	88.602	B05
100066710	B05	93.045	B05
100007664	B05	93.838	B05
100030861	B05	98.838	B05
100015095	B05	99.19	B05
100061527	B05	102.542	B05
100016835	B05	103.618	B05
100015045	B05	107.891	B05
100048806	B05	108.88	B05

100037455	B05	109.533	B05
100015465	B05	109.812	B05
100014439	B05	111.109	B05
100001885	B05	112.486	B05
100021113	B05	114.717	B05
100032378	B05	115.193	B05
100026909	B05	116.024	B05
100014070	B05	116.54	B05
100046082	B05	116.86	B05
100038240	B05	118.086	B05
100057131	B05	124.349	B05
100019348	B05	124.992	B05
100067737	B05	125.902	B05
100069538	B05	136.921	B05
100054809	B05	137.435	B05
100075572	B05	139.212	B05
100030357	B05	140.835	B05
100004698	B05	141.39	B05
100071120	B05	141.778	B05
100031323	B05	144.322	B05
100012211	B05	145.691	B05
100024998	B05	145.929	B05
100058247	B05	148.237	B05
100026725	B06	0	B06
100002826	B06	0.914	B06
100036039	B06	6.316	B06
100025352	B06	6.772	B06
100076205	B06	7.169	B06
100078760	B06	8.67	B06
100023564	B06	8.887	B06
100022838	B06	9.381	B06
100016091	B06	9.891	B06
100009834	B06	12.683	B06
100028661	B06	14.489	B06
100017288	B06	15.153	B06
100041742	B06	16.031	B06
100013180	B06	16.655	B06
100062356	B06	17.622	B06
100013392	B06	20.637	B06
100063328	B06	22.606	B06

100076294	B06	26.053	B06
100014228	B06	27.314	B06
100027451	B06	27.991	B06
100019182	B06	28.369	B06
100011380	B06	29.925	B06
100015166	B06	30.736	B06
100056005	B06	31.718	B06
100014914	B06	33.759	B06
100015546	B06	37.032	B06
100011988	B06	38.025	B06
100022182	B06	38.842	B06
100025951	B06	40.49	B06
100035292	B06	42.507	B06
100022670	B06	44.542	B06
100011299	B06	44.837	B06
100028364	B06	45.327	B06
100007539	B06	46.52	B06
100012880	B06	49.087	B06
100077635	B06	50.065	B06
100016097	B06	50.727	B06
100014992	B06	51.183	B06
100027960	B06	53.198	B06
100011009	B06	54.602	B06
100061647	B06	63.302	B06
100014469	B06	63.609	B06
100000155	B06	64.575	B06
100016957	B06	65.6	B06
100022637	B06	65.996	B06
100021447	B06	72.463	B06
100019470	B06	72.695	B06
100077914	B06	72.998	B06
100011875	B06	74.005	B06
100077119	B06	75.095	B06
100024938	B06	76.364	B06
100014466	B06	78.292	B06
100052308	B06	78.819	B06
100030283	B06	79.144	B06
100029326	B06	85.667	B06
100042826	B06	87.579	B06
100005386	B06	89.779	B06

100020421	B06	90.242	B06
100041106	B06	91.583	B06
100061512	B06	92.2	B06
100037101	B06	92.453	B06
100013746	B06	94.528	B06
100025434	B06	101.811	B06
100075250	B06	103.279	B06
100008486	B06	105.434	B06
100069214	B06	107.616	B06
100040798	B06	109.478	B06
100031132	B06	110.564	B06
100004737	B06	117.501	B06
100042372	B06	119.321	B06
100022808	B06	121.311	B06
100026674	B06	122.073	B06
100008329	B06	122.413	B06
100017303	B06	123.996	B06
100006344	B06	124.25	B06
100078887	B06	126.516	B06
100023658	B06	127.87	B06
100017209	B06	128.334	B06
100033270	B06	129.563	B06
100037452	B06	129.804	B06
100029522	B06	130.268	B06
100011652	B06	132.124	B06
100047274	B06	133.295	B06
100050168	B07	0	B07
100008381	B07	4.237	B07
100015564	B07	4.877	B07
100022390	B07	5.641	B07
100008594	B07	5.915	B07
100020052	B07	6.162	B07
100017774	B07	6.38	B07
100055759	B07	7.223	B07
100021591	B07	7.452	B07
100011871	B07	8.649	B07
100075729	B07	9.324	B07
100053479	B07	9.855	B07
100048383	B07	10.198	B07
100022941	B07	10.829	B07

100007567	B07	11.256	B07
100027589	B07	11.843	B07
100023033	B07	12.763	B07
100052248	B07	13.252	B07
100041347	B07	13.479	B07
100033400	B07	14.211	B07
100039563	B07	18.961	B07
100008824	B07	19.362	B07
100032112	B07	19.929	B07
100017597	B07	20.15	B07
100067851	B07	21.71	B07
100021600	B07	22.622	B07
100007740	B07	27.14	B07
100012258	B07	27.728	B07
100069283	B07	28.265	B07
100048156	B07	28.774	B07
100033725	B07	35.984	B07
100061847	B07	37.135	B07
100055425	B07	37.945	B07
100029387	B07	38.189	B07
100007506	B07	38.515	B07
100018914	B07	39.876	B07
100007757	B07	47.314	B07
100013191	B07	48.07	B07
100042453	B07	48.271	B07
100071901	B07	49.084	B07
100043465	B07	49.497	B07
100019987	B07	50.204	B07
100020128	B07	51.057	B07
100043226	B07	52.676	B07
100022141	B07	56.283	B07
100028838	B07	57.317	B07
100025963	B07	57.716	B07
100052876	B07	58.171	B07
100026124	B07	60.487	B07
100019213	B07	63.117	B07
100053770	B07	64.298	B07
100006973	B07	65.812	B07
100022658	B07	70.834	B07
100046735	B07	71.574	B07

100048863	B07	72.934	B07
100025864	B07	75.853	B07
100061514	B07	76.207	B07
100009526	B07	76.873	B07
100065580	B07	78.09	B07
100026799	B07	78.514	B07
100006925	B07	79.341	B07
100031225	B07	81.584	B07
100019404	B07	81.982	B07
100046092	B07	84.335	B07
100018779	B07	84.643	B07
100022946	B07	86.997	B07
100026981	B07	87.979	B07
100028921	B07	89.054	B07
100021852	B07	89.591	B07
100004375	B07	89.885	B07
100074843	B07	90.44	B07
100065271	B07	91.048	B07
100054851	B07	92.393	B07
100007772	B08	0.551	B08
100006325	B08	0.85	B08
100025810	B08	1.647	B08
100024274	B08	2.039	B08
100026655	B08	4.133	B08
100062839	B08	4.409	B08
100048959	B08	4.819	B08
100017154	B08	6.528	B08
100049415	B08	7.069	B08
100014804	B08	7.694	B08
100052322	B08	8.087	B08
100042836	B08	8.674	B08
100059103	B08	8.955	B08
100005823	B08	10.213	B08
100015383	B08	11.583	B08
100031662	B08	13.388	B08
100050502	B08	13.912	B08
100029236	B08	14.244	B08
100020924	B08	14.46	B08
100010788	B08	15.428	B08
100028694	B08	16.221	B08

100071710	B08	18.721	B08
100056767	B08	19.434	B08
100011944	B08	20.936	B08
100017045	B08	21.26	B08
100022187	B08	21.649	B08
100059675	B08	22.196	B08
100021572	B08	22.428	B08
100024366	B08	22.732	B08
100017058	B08	25.242	B08
100043101	B08	26.07	B08
100035102	B08	27.147	B08
100034901	B08	27.607	B08
100018041	B08	30.715	B08
100073600	B08	31.613	B08
100044399	B08	32.2	B08
100016337	B08	33.146	B08
100023649	B08	33.667	B08
100066773	B08	34.173	B08
100033541	B08	34.602	B08
100012806	B08	35.969	B08
100016840	B08	36.766	B08
100024312	B08	37.313	B08
100064677	B08	38.147	B08
100025744	B08	38.447	B08
100013491	B08	39.415	B08
100006651	B08	42.299	B08
100058267	B08	43.939	B08
100056430	B08	45.806	B08
100028818	B08	47.946	B08
100018229	B08	48.226	B08
100007413	B08	48.691	B08
100015259	B08	52.295	B08
100030257	B08	52.712	B08
100004378	B08	56.905	B08
100063747	B08	57.552	B08
100017540	B08	59.67	B08
100013735	B08	60.159	B08
100070432	B08	60.801	B08
100070293	B08	61.109	B08
100077309	B08	61.813	B08

100043978	B08	62.257	B08
100024207	B08	63.486	B08
100077122	B08	64.792	B08
100057003	B08	65.386	B08
100022376	B08	66.711	B08
100069575	B08	67.165	B08
100021262	B08	70.61	B08
100037800	B08	70.988	B08
100068880	B08	72.251	B08
100006430	B08	77.993	B08
100024846	B08	83.077	B08
100045878	B08	84.451	B08
100049688	B08	84.945	B08
100062881	B08	85.347	B08
100039529	B08	90.259	B08
100076152	B08	92.334	B08
100014239	B08	95.454	B08
100028386	B08	96.374	B08
100054104	B08	101.379	B08
100073700	B08	102.971	B08
100043417	B08	103.403	B08
100015971	B08	104.877	B08
100018028	B08	110.331	B08
100017061	B08	112.618	B08
100017996	B08	121.984	B08
100024128	B08	122.359	B08
100024018	B08	129.684	B08
100002494	B08	130.284	B08
100049153	B08	130.763	B08
100042491	B08	135.709	B08
100024082	B08	135.915	B08
100071108	B08	136.227	B08
100040486	B08	136.575	B08
100059833	B08	137.624	B08
100023660	B08	138.074	B08
100007476	B08	138.876	B08
100065709	B08	141.477	B08
100032326	B08	143.587	B08
100071515	B08	144.587	B08
100013294	B08	145.417	B08

100025362	B08	147.207	B08
100066416	B08	148.694	B08
100019218	C01	7.818	C01
100011545	C01	16.456	C01
100011069	C01	26.279	C01
100004218	C01	30.205	C01
100010016	C01	31.956	C01
100014201	C01	34.367	C01
100064031	C01	36.165	C01
100078611	C01	36.372	C01
100004960	C01	38.216	C01
100026062	C01	42.377	C01
100018611	C01	44.654	C01
100044915	C01	45.639	C01
100063630	C01	48.72	C01
100015932	C01	49.715	C01
100063706	C01	50.325	C01
100052180	C01	50.911	C01
100018514	C01	51.405	C01
100060944	C01	53.854	C01
100060450	C01	54.739	C01
100016520	C01	58.398	C01
100003472	C01	61.702	C01
100015028	C01	67.745	C01
100025702	C01	70.498	C01
100018249	C01	70.814	C01
100031795	C01	87.704	C01
100062611	C01	89.799	C01
100059879	C01	98.736	C01
100016232	C01	99.205	C01
100020118	C01	100.574	C01
100017613	C01	103.132	C01
100024486	C01	103.947	C01
100008983	C01	106.982	C01
100056536	C01	108.165	C01
100010271	C01	114.255	C01
100018715	C02	0	C02
100025181	C02	3.499	C02
100055259	C02	3.774	C02
100027402	C02	4.204	C02

100046502	C02	4.636	C02
100072642	C02	6.433	C02
100064473	C02	7.955	C02
100020028	C02	12.436	C02
100071010	C02	23.022	C02
100068200	C02	37.177	C02
100007974	C02	37.913	C02
100008592	C02	40.556	C02
100054557	C02	42.012	C02
100049100	C02	42.932	C02
100014818	C02	43.594	C02
100057798	C02	43.854	C02
100037219	C02	46.858	C02
100028432	C02	48.282	C02
100013654	C02	48.434	C02
100005075	C02	65.511	C02
100029810	C02	66.001	C02
100015422	C02	69.244	C02
100051900	C02	70.629	C02
100051280	C02	79.072	C02
100063199	C02	88.559	C02
100040002	C02	89.139	C02
100011677	C02	90.269	C02
100024946	C02	90.944	C02
100015155	C02	91.356	C02
100047227	C02	91.807	C02
100061522	C02	94.12	C02
100007874	C02	115.53	C02
100037636	C02	117.215	C02
100041321	C02	121.683	C02
100049050	C03	2.257	C03
100061792	C03	3.289	C03
100040264	C03	3.697	C03
100064710	C03	4.27	C03
100008367	C03	6.028	C03
100030726	C03	10.948	C03
100021092	C03	11.359	C03
100058609	C03	11.574	C03
100028512	C03	22.993	C03
100019306	C03	24.032	C03

100068873	C03	26.717	C03
100059493	C03	27.124	C03
100010185	C03	36.168	C03
100010157	C03	36.665	C03
100010996	C03	37.364	C03
100066748	C03	42.512	C03
100076711	C03	44.215	C03
100015636	C03	48.801	C03
100014090	C03	54.913	C03
100026334	C03	55.482	C03
100015854	C03	57.68	C03
100022415	C03	61.512	C03
100070490	C03	62.389	C03
100010575	C03	64.511	C03
100014190	C03	64.963	C03
100015711	C03	65.41	C03
100052674	C03	76.295	C03
100056247	C03	76.916	C03
100006416	C03	79.622	C03
100022642	C03	79.937	C03
100022688	C03	80.312	C03
100014999	C03	84.553	C03
100031828	C03	85.459	C03
100069845	C03	86.258	C03
100016849	C03	88.398	C03
100052320	C03	90.597	C03
100034569	C03	91.333	C03
100040377	C03	91.996	C03
100012746	C03	95.718	C03
100049366	C03	96.281	C03
100042445	C03	96.703	C03
100034883	C03	97.989	C03
100012667	C03	99.802	C03
100049018	C03	100.04	C03
100068795	C03	100.446	C03
100074355	C03	102.901	C03
100079205	C03	103.13	C03
100050829	C03	103.376	C03
100018700	C03	104.617	C03
100025118	C03	106.145	C03

100010953	C03	106.974	C03
100025390	C03	107.194	C03
100043219	C03	107.544	C03
100014532	C03	107.785	C03
100042637	C03	109.741	C03
100055174	C03	110.425	C03
100026762	C03	120.848	C03
100061627	C03	121.424	C03
100008551	C03	121.843	C03
100012299	C03	122.063	C03
100011923	C03	122.449	C03
100023245	C03	131.189	C03
100024943	C03	131.506	C03
100023202	C03	135.698	C03
100038513	C03	135.938	C03
100027338	C03	136.296	C03
100053345	C03	136.55	C03
100039504	C03	140.59	C03
100036885	C03	141.211	C03
100026533	C03	141.563	C03
100002930	C03	147.66	C03
100029700	C03	152.778	C03
100062181	C03	153.446	C03
100015635	C03	155.641	C03
100050910	C03	156.447	C03
100017735	C03	159.69	C03
100032757	C03	159.902	C03
100020221	C03	160.848	C03
100013435	C03	164.701	C03
100031168	C03	165.754	C03
100021460	C03	166.68	C03
100029139	C03	167.003	C03
100023632	C04	2.883	C04
100019506	C04	3.087	C04
100065508	C04	10.431	C04
100036778	C04	11.135	C04
100037486	C04	24.519	C04
100015148	C04	24.85	C04
100016930	C04	28.912	C04
100048982	C04	32.374	C04

100021474	C04	33.105	C04
100025551	C04	33.367	C04
100009621	C04	34.259	C04
100029356	C04	34.849	C04
100059630	C04	54.266	C04
100023611	C04	54.696	C04
100074863	C04	55.7	C04
100016523	C04	56.579	C04
100024625	C04	56.832	C04
100029056	C04	60.459	C04
100040086	C04	61.546	C04
100022727	C04	70.261	C04
100044151	C04	71.026	C04
100013195	C04	71.522	C04
100007868	C04	72.245	C04
100008940	C04	84.191	C04
100022579	C04	92.097	C04
100043087	C04	99.511	C04
100012771	C04	99.861	C04
100012629	C04	105.171	C04
100022173	C04	120.909	C04
100035805	C04	121.33	C04
100032039	C04	121.698	C04
100027194	C04	131.982	C04
100058814	C04	133.966	C04
100019865	C04	135.306	C04
100026640	C04	135.526	C04
100021516	C04	135.73	C04
100029133	C04	137.752	C04
100008104	C04	140.451	C04
100021627	C04	143.807	C04
100024089	C04	145.33	C04
100016245	C05	0	C05
100007983	C05	0.979	C05
100069690	C05	10.029	C05
100006256	C05	17.863	C05
100041974	C05	24.764	C05
100013309	C05	25.695	C05
100034032	C05	26.864	C05
100022700	C05	27.607	C05

100038782	C05	29.615	C05
100013233	C05	41.076	C05
100018683	C05	41.534	C05
100047046	C05	42.265	C05
100009644	C05	52.208	C05
100078636	C05	54.854	C05
100065201	C05	55.338	C05
100035903	C05	56.035	C05
100018642	C05	59.537	C05
100012535	C05	60.141	C05
100037609	C05	64.692	C05
100016951	C05	65.346	C05
100057078	C05	65.949	C05
100034396	C05	66.839	C05
100006019	C05	68.588	C05
100030993	C05	88.235	C05
100021467	C05	92.05	C05
100059378	C05	95.525	C05
100016364	C05	102.455	C05
100026428	C05	108.145	C05
100067924	C05	114.826	C05
100013730	C05	119.728	C05
100028725	C05	122.277	C05
100008603	C05	123.88	C05
100018304	C06	0	C06
100032948	C06	1.201	C06
100008675	C06	22.277	C06
100023158	C06	24.847	C06
100023982	C06	26.844	C06
100020680	C06	28.267	C06
100028657	C06	29.36	C06
100035726	C06	32.083	C06
100037880	C06	34.691	C06
100021971	C06	44.216	C06
100008810	C06	45.766	C06
100012448	C06	48.894	C06
100040008	C06	49.302	C06
100033089	C06	54.227	C06
100056481	C06	55.199	C06
100023693	C06	55.792	C06

100015500	C06	57.46	C06
100031328	C06	59.073	C06
100035118	C06	60.597	C06
100026430	C06	61.728	C06
100028305	C06	62.07	C06
100050944	C06	62.862	C06
100040545	C06	65.677	C06
100020694	C06	66.037	C06
100017755	C06	69.321	C06
100061440	C06	69.888	C06
100009082	C06	70.993	C06
100028996	C06	71.731	C06
100012727	C06	76.878	C06
100020122	C06	78.99	C06
100019630	C06	81.206	C06
100073913	C06	82.092	C06
100033606	C06	83.851	C06
100037463	C06	86.425	C06
100041764	C06	86.797	C06
100036823	C06	87.808	C06
100078804	C06	90.375	C06
100013331	C06	91.478	C06
100062752	C06	92.105	C06
100037438	C06	93.943	C06
100024804	C06	94.209	C06
100027403	C06	96.012	C06
100029429	C06	97.637	C06
100032036	C06	98.923	C06
100057725	C06	104.931	C06
100037859	C07	0	C07
100007433	C07	1.642	C07
100048720	C07	4.721	C07
100040526	C07	8.186	C07
100013962	C07	8.506	C07
100025920	C07	8.859	C07
100013825	C07	9.289	C07
100038163	C07	14.206	C07
100003285	C07	14.67	C07
100020742	C07	15.861	C07
100024192	C07	20.428	C07

100019077	C07	21.688	C07
100062030	C07	21.976	C07
100065291	C07	22.83	C07
100037708	C07	26.011	C07
100061207	C07	30.784	C07
100030964	C07	31.45	C07
100009750	C07	32.916	C07
100006664	C07	33.631	C07
100072240	C07	38.953	C07
100028791	C07	39.231	C07
100053583	C07	40.441	C07
100028349	C07	43.465	C07
100048778	C07	43.847	C07
100054865	C07	45.63	C07
100033605	C07	52.268	C07
100018746	C07	52.609	C07
100040390	C07	54.204	C07
100022249	C07	57.732	C07
100046712	C07	61.189	C07
100078147	C07	62.258	C07
100034293	C07	66.414	C07
100071984	C07	68.627	C07
100034961	C07	73.534	C07
100024563	C07	74.54	C07
100038749	C07	80.026	C07
100011061	C07	82.476	C07
100004847	C07	83.874	C07
100065439	C07	85.437	C07
100051078	C07	88.579	C07
100013999	C07	89.664	C07
100005592	C07	91.091	C07
100015457	C07	92.74	C07
100012656	C07	94.051	C07
100026256	C07	97.965	C07
100021982	C07	99.536	C07
100019616	C07	101.171	C07
100017146	C08	1.495	C08
100039765	C08	2.052	C08
100014017	C08	2.745	C08
100055365	C08	6.069	C08

100014401	C08	7.247	C08
100024334	C08	12.133	C08
100029984	C08	12.605	C08
100011973	C08	22.922	C08
100031848	C08	26.896	C08
100053216	C08	27.45	C08
100015981	C08	27.97	C08
100021875	C08	28.278	C08
100036359	C08	28.912	C08
100062727	C08	30.146	C08
100075300	C08	34.278	C08
100023350	C08	34.684	C08
100045208	C08	35.928	C08
100019441	C08	40.736	C08
100015313	C08	41.318	C08
100030360	C08	41.693	C08
100012490	C08	42.425	C08
100026947	C08	43.058	C08
100075461	C08	48.13	C08
100022161	C08	48.688	C08
100032715	C08	51.31	C08
100011644	C08	55.774	C08
100029043	C08	58.734	C08
100028825	C08	62.528	C08
100009857	C08	62.809	C08
100029655	C08	63.49	C08
100040100	C08	64.561	C08
100001609	C08	69.283	C08
100008240	C08	69.676	C08
100042506	C08	71.017	C08
100041581	C08	71.741	C08
100043358	C08	73.139	C08
100038688	C08	73.493	C08
100010513	C08	81.703	C08
100053806	C08	85.51	C08
100069841	C08	86.515	C08
100063523	C08	87.332	C08
100032920	C08	88.455	C08
100009976	C08	90.345	C08
100034307	C09	3.582	C09

100005670	C09	4.435	C09
100054209	C09	5.322	C09
100012123	C09	14.115	C09
100015972	C09	15.509	C09
100060680	C09	16.325	C09
100028894	C09	17.481	C09
100017422	C09	17.719	C09
100005758	C09	18.328	C09
100079100	C09	20.005	C09
100062459	C09	27.461	C09
100074931	C09	28.312	C09
100064959	C09	28.52	C09
100024455	C09	31.718	C09
100052899	C09	34.442	C09
100013369	C09	38.906	C09
100008593	C09	40.633	C09
100006331	C09	44.215	C09
100030523	C09	44.979	C09
100074804	C09	50.682	C09
100010811	C09	51.457	C09
100040921	C09	51.913	C09
100064791	C09	54.979	C09
100026545	C09	100.13	C09
100024657	C09	103.457	C09
100013650	C09	103.9	C09
100026791	C09	104.831	C09
100053369	C09	106.454	C09
100013061	C09	106.806	C09
100055717	C09	112.73	C09

ɪn and B. carinata pangenome	
Alignment Position _Start_pangenome (bp)	Alignment Position_end_pangenome
1423489	1423556
1454764	1454830
1454764	1454830
402892	402943
2410414	2410363
2869966	2869900
3985165	3985109
3945448	3945513
3275045	3274979
3464651	3464587
4389352	4389299
4389352	4389299
4501345	4501410
4876636	4876702
4525908	4525974
5263216	5263282
5476711	5476774
5463212	5463150
5533409	5533469
5753493	5753559
6546983	6547046
7172368	7172312
6930655	6930592
7191913	7191847
7867090	7867031
7860627	7860563
7952546	7952612
7952562	7952604
9673725	9673791
9906449	9906514
10073065	10072996
10011334	10011395
10795314	10795248
10528821	10528755
10952265	10952331
11025465	11025401

11235709	11235755
11118065	11118130
11646545	11646611
12048695	12048760
12152374	12152440
12220423	12220357
12824777	12824743
14233358	14233397
15349025	15348959
12673630	12673696
14728991	14728925
16537166	16537203
31668509	31668443
35303037	35302971
42746133	42746087
42746133	42746083
43289138	43289072
43802368	43802302
45360003	45360037
46282295	46282361
46629519	46629453
11399540	11399587
46758677	46758610
47211307	47211369
47564953	47565019
47571003	47570949
48147244	48147205
49462002	49461948
47078811	47078759
49671382	49671448
49817900	49817966
50351407	50351360
50475632	50475566
50727484	50727520
50727494	50727520
51303981	51304041
51202754	51202688
51356456	51356412
51545112	51545046
52000062	52000125

51963686	51963754
52141978	52141912
52194707	52194773
52262614	52262548
52486919	52486881
52358255	52358321
52774315	52774249
52910759	52910732
52916179	52916125
53032342	53032408
53369121	53369095
53369183	53369224
53424934	53424998
53619346	53619278
53619346	53619280
53773682	53773748
53961784	53961844
54807209	54807275
55104014	55103962
55124221	55124277
55051565	55051618
821785	821812
754991	755057
37136	37199
821764	821814
977409	977475
1201869	1201810
1399573	1399507
1476700	1476659
1797804	1797866
1866971	1866905
2001086	2001130
2001086	2001141
2414644	2414682
2627576	2627510
2627576	2627525
3665703	3665768
3841062	3840996
36537419	36537480
4315472	4315424

4325381	4325447
4601991	4602057
7591473	7591539
7572287	7572353
7466942	7467000
33244381	33244447
17464899	17464833
25843861	25843798
37240501	37240567
42465071	42465006
15363567	15363614
42451440	42451382
42519493	42519559
42967680	42967746
45239683	45239749
46215763	46215827
46699391	46699426
47335508	47335442
48679410	48679361
48943727	48943792
49472456	49472522
49751041	49750995
39958406	39958442
50500290	50500356
51784812	51784875
51778022	51777956
52135991	52135945
52238870	52238804
52376713	52376649
52583900	52583834
52636000	52636066
52684464	52684398
53858747	53858681
54340619	54340578
54603309	54603243
16251003	16251067
55375273	55375209
56029831	56029880
56280031	56280097
56553389	56553455

56760099	56760165
57165433	57165499
900294	900264
58486113	58486050
58666603	58666542
58555868	58555802
58903409	58903343
60134772	60134836
60785638	60785692
56702566	56702518
61801435	61801498
61812221	61812272
62472668	62472605
62181627	62181693
62181627	62181693
62754715	62754649
62975390	62975324
63518843	63518909
64170731	64170665
64869778	64869844
65084371	65084305
66286518	66286452
66629145	66629079
67151386	67151320
65960110	65960044
67863576	67863634
67614925	67614859
63559861	63559903
68663278	68663212
68996094	68996160
69262036	69262102
69599436	69599502
69674951	69675017
69747994	69748060
69564736	69564670
70303958	70303891
70542396	70542462
70954800	70954857
71059217	71059274
70435009	70434961

70986340	70986274
180246	180180
1081992	1082045
605363	605429
849788	849841
750559	750516
951409	951450
1029019	1028953
1132540	1132474
1238005	1238071
1269485	1269551
1316694	1316628
1622410	1622349
1879674	1879745
1894714	1894663
1894711	1894777
2006018	2006084
2492956	2492890
2643056	2642994
2970860	2970926
3142244	3142296
3736325	3736370
4230246	4230282
4291554	4291489
4471281	4471346
5243441	5243406
5408191	5408257
6562828	6562894
6689599	6689533
6718334	6718268
6972507	6972565
6832426	6832464
7217573	7217507
20223164	20223230
9039627	9039561
9150429	9150371
9150269	9150335
9841129	9841063
10310571	10310632
10463906	10463941

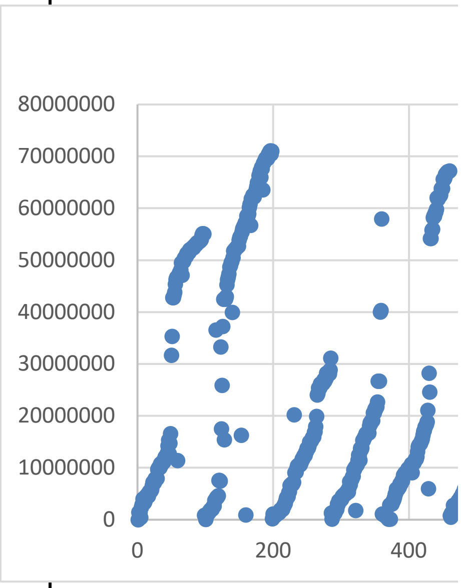

10422488	10422422
10598900	10598966
10598900	10598966
10897125	10897059
11245187	11245225
11721694	11721628
11721694	11721628
12124387	12124453
12355439	12355379
12357151	12357088
13005248	13005182
13137245	13137306
13719701	13719642
13797283	13797331
13820320	13820259
14173474	14173439
14948287	14948230
15011508	15011572
15150423	15150358
15384821	15384887
15839460	15839398
15842146	15842203
15954155	15954215
16797344	16797281
16938399	16938465
17895893	17895827
19893042	19892985
24055072	24055138
24330667	24330601
25367384	25367340
25395658	25395593
25395658	25395592
26166606	26166671
26166132	26166198
26213317	26213271
26189205	26189271
26379636	26379702
27001739	27001805
26676656	26676598
26985163	26985229

27181801	27181867
28232944	28232995
28069207	28069162
28270078	28270144
28280879	28280817
28075757	28075691
28822835	28822901
31099813	31099748
1256432	1256498
106448	106514
1108628	1108694
1442623	1442689
1371877	1371811
1261135	1261069
1828415	1828481
1827967	1827901
2000348	2000282
2985494	2985429
2944135	2944187
3618464	3618530
3659102	3659036
3697723	3697789
3904869	3904916
3904869	3904914
4477503	4477557
4511551	4511499
4731339	4731372
4874627	4874654
4916602	4916668
4958984	4959050
5271460	5271525
5443418	5443484
5503570	5503636
5325162	5325223
6140521	6140587
7338880	7338914
6738910	6738960
7707405	7707340
7839731	7839797
8284285	8284219

8337788	8337722
9579560	9579626
9675154	9675220
10000900	10000955
1765418	1765470
10444965	10444930
10619241	10619306
11448532	11448573
12427387	12427335
12396399	12396455
11454164	11454226
13745184	13745226
13472895	13472961
13781551	13781492
15297935	15298001
15299564	15299515
15615392	15615326
15960430	15960496
16078753	16078687
16587705	16587771
16587705	16587768
16682217	16682166
16699498	16699432
16703339	16703407
18051882	18051948
18676321	18676255
18525410	18525476
19020350	19020290
19020064	19020128
19072404	19072348
20602196	20602262
20602199	20602134
21331111	21331045
21260535	21260485
21735709	21735666
21747793	21747859
22678251	22678305
26659606	26659540
26669692	26669758
26659906	26659840

39975617	39975551
40288136	40288192
57912001	57912067
1108873	1108807
1085990	1085925
945148	945091
971931	971993
756053	756099
711248	711314
536186	536120
550521	550583
317078	317012
118250	118191
118250	118191
2817082	2817144
82350	82416
3012496	3012430
2999220	2999168
3399509	3399575
3588178	3588227
3837604	3837545
4297513	4297579
4823335	4823269
5128511	5128577
5896363	5896429
5896366	5896329
5896542	5896476
6259184	6259125
6249890	6249943
6854602	6854638
6904351	6904415
7009310	7009368
7478746	7478694
7519714	7519775
8566682	8566654
8566024	8565958
8940464	8940398
9025257	9025193
9025257	9025192
9122556	9122494

9346540	9346475
9785449	9785391
9743477	9743411
10078436	10078502
10078439	10078373
9778685	9778751
10714560	10714494
9036416	9036450
10825536	10825577
10948023	10948089
10948023	10948089
11456651	11456600
11693129	11693063
11676480	11676414
12154309	12154375
13129911	13129977
14060840	14060896
14342838	14342902
14465970	14465904
14626300	14626232
15011635	15011701
15273271	15273330
15655261	15655327
16295164	16295098
16973151	16973116
17179429	17179363
17795721	17795786
17965154	17965088
18392865	18392931
18849387	18849453
21094703	21094769
5942835	5942901
28216502	28216566
24560139	24560201
54159869	54159803
54162319	54162253
55782724	55782684
55981383	55981333
58153418	58153357
58525392	58525355

58515867	58515933
59095730	59095795
59284983	59285050
59813943	59813882
62002486	62002420
61758961	61759027
62224880	62224817
62225603	62225659
62370412	62370454
62531081	62531015
63815481	63815547
63675332	63675266
63800546	63800604
65555702	65555658
65589078	65589025
65832150	65832101
66517798	66517864
66673512	66673572
66667248	66667182
67016843	67016909
67081530	67081596
67082151	67082216
67158869	67158922
803070	803004
488000	487942
1255205	1255139
1229716	1229650
1230396	1230330
2724417	2724351
2724391	2724365
1725415	1725350
2948335	2948368
2625269	2625217
2205049	2204983
3434115	3434049
3641382	3641448
3745058	3745021
3820510	3820574
4182500	4182440
4395268	4395303

4587536	4587504
4966898	4966832
5107388	5107322
5077529	5077463
5807840	5807902
5806896	5806830
5980956	5980890
6159004	6158946
6567378	6567345
6848577	6848638
6917092	6917158
7007434	7007500
7578296	7578362
7751716	7751650
7806275	7806341
7790229	7790295
8493893	8493959
8602237	8602188
8691524	8691573
8845339	8845405
8868877	8868815
9551631	9551686
8821119	8821057
19293215	19293253
19293218	19293152
22992553	22992486
43493430	43493383
23406189	23406255
52744908	52744856
52744905	52744971
52784110	52784176
53207838	53207778
53387176	53387221
53421099	53421033
53645151	53645216
54215050	54215091
54074573	54074509
55943119	55943185
56070165	56070225
56632625	56632561

56694241	56694307
56986703	56986759
56927286	56927242
56924661	56924595
57347102	57347167
58953878	58953851
59184845	59184802
59456792	59456845
60013886	60013840
60816645	60816710
60825972	60826029
61782559	61782601
61959694	61959760
62195018	62195084
62197969	62198035
62202347	62202413
62948506	62948458
62930440	62930506
63232821	63232887
63711870	63711814
64243330	64243264
63841301	63841367
63935435	63935501
64138864	64138930
65232525	65232591
65248525	65248591
5609022	5608960
1876	1810
1388458	1388396
1390891	1390838
1683794	1683728
1390888	1390954
2290346	2290280
2344566	2344632
4702916	4702977
4624381	4624447
5528871	5528828
30557469	30557535
7719181	7719247
21294193	21294133

30123855	30123921
12348182	12348242
33673335	33673269
32777813	32777850
33972625	33972687
34110769	34110703
37084782	37084717
837242	837298
36420024	36419957
36450571	36450505
37881210	37881262
38143811	38143877
39686137	39686203
40258313	40258247
40071344	40071290
40136043	40136109
41916549	41916493
42025652	42025718
42093699	42093765
42115116	42115182
42185769	42185829
42351666	42351731
45041153	45041219
45077665	45077726
45117586	45117651
45238081	45238124
45711486	45711523
45805435	45805501
45943771	45943705
46243864	46243921
46681365	46681431
46948960	46949022
46903327	46903261
47146641	47146575
47703667	47703733
49105660	49105726
48651965	48651925
49803836	49803778
51266036	51265982
51537022	51536973

52061907	52061973
53389952	53389924
53401726	53401775
54030633	54030567
54206504	54206539
54291709	54291643
54380034	54380085
55236567	55236508
55365781	55365847
54700532	54700598
54687745	54687811
55574799	55574865
56551765	56551699
56718936	56718872
57220270	57220334
57252549	57252483
57944665	57944599
56766811	56766745
56580191	56580242
21966	21900
575418	575352
1883988	1883922
1885010	1885076
2501086	2501034
2499402	2499438
2521908	2521968
3196375	3196319
3313718	3313652
3372335	3372401
3562693	3562638
3577433	3577489
3562693	3562638
3661676	3661616
3860317	3860374
3922642	3922708
3922642	3922708
3932794	3932855
4047289	4047356
4112164	4112229
4265580	4265514

4480438	4480394
4647008	4647074
4690944	4691010
4690944	4691010
4980558	4980615
4979593	4979648
4877468	4877402
5076994	5076928
5202801	5202748
5307007	5307068
5963898	5963959
5959905	5959971
6548688	6548622
6643095	6643029
6652983	6653049
6683844	6683781
6766618	6766684
6826176	6826131
6823917	6823851
6890563	6890500
6949251	6949316
7231458	7231515
7317965	7317913
7288063	7288008
7404666	7404732
8567987	8567932
9573534	9573472
10313122	10313174
10998159	10998093
11038560	11038626
11038563	11038503
12858371	12858424
12644068	12644002
13456393	13456434
13571476	13571525
13734710	13734776
13787083	13787149
13874197	13874263
13973178	13973118
14214344	14214277

14194805	14194739
14258277	14258211
14695227	14695161
15293445	15293380
16013993	16013927
15574383	15574343
19445254	19445317
19437697	19437763
18892562	18892628
25354589	25354528
29639813	29639879
7128949	7129010
35370462	35370528
50092908	50092842
58302693	58302759
59369354	59369288
60654566	60654620
60668550	60668484
61571354	61571420
61626102	61626146
61622540	61622474
61764996	61765052
63174380	63174350
63488814	63488880
64415866	64415800
64428888	64428833
65198644	65198702
11820745	11820684
65198647	65198613
65967365	65967431
65794249	65794191
65967368	65967320
65940451	65940512
66786415	66786383
66791479	66791425
66686646	66686709
66426679	66426718
66042356	66042422
67194242	67194202
67587943	67588009

68427880	68427946
68448886	68448914
1672321	1672387
2837047	2836981
3832925	3832871
4247038	4247104
5612519	5612453
6226821	6226755
6493916	6493982
6498865	6498799
6764850	6764911
8202544	8202478
8800848	8800914
9044976	9045042
10103992	10104058
10703084	10703021
11777703	11777665
11772083	11772022
11738904	11738970
13330984	13331047
13372402	13372346
14862925	14862975
16103270	16103338
28776884	28776824
38016163	38016095
37780849	37780783
47122533	47122467
4094825	4094762
48459628	48459687
48463588	48463654
48738262	48738196
49056911	49056977
49056914	49056848
50129996	50130062
50236775	50236709
50730296	50730233
349183	349117
52925	52991
276206	276149
558911	558845

589015	589081
1289743	1289775
1413264	1413328
2317208	2317274
3306719	3306653
4978099	4978060
4976067	4976133
5435459	5435393
6045358	6045414
6209781	6209715
6292892	6292954
6223911	6223977
7442236	7442274
7545500	7545440
7552668	7552611
19880533	19880467
23068585	23068531
26493518	26493468
26272358	26272298
33608394	33608328
50655217	50655280
50669413	50669479
51593931	51593865
51692382	51692316
51717073	51717139
51770408	51770474
54288029	54287995
59870402	59870338
59868678	59868612
60555463	60555529
233880	233847
1663395	1663361
1884001	1883935
1977075	1977141
2079007	2078941
3541405	3541471
3541405	3541471
3761559	3761625
6797188	6797122
6375631	6375574

7319827	7319860
7330554	7330488
10638655	10638710
10720137	10720074
10725573	10725507
12547078	12547042
13256038	13255972
14310753	14310805
16616876	16616942
16708252	16708200
16956881	16956815
18546808	18546742
18580674	18580628
64449756	64449823
19933746	19933811
19004183	19004117
25361200	25361134
25361200	25361134
25925776	25925727
25951329	25951396
25942434	25942483
27391836	27391898
28145861	28145795
28147464	28147528
29504924	29504858
32124285	32124228
32984774	32984708
32984774	32984712
37643560	37643626
37657342	37657276
37644149	37644083
38079985	38080051
38734985	38735051
40526839	40526905
39475111	39475063
40899965	40900011
40903572	40903638
40903572	40903638
41037254	41037189
41703079	41703144

42246869	42246935
42283501	42283567
42289729	42289663
42246869	42246935
54448166	54448100
54329782	54329848
61135579	61135522
60489193	60489152
61149909	61149840
61123714	61123780
61123714	61123779
65545756	65545822
22872101	22872159
66394494	66394428
66608184	66608118
49225660	49225719
67595157	67595223
69797282	69797341
69985893	69985959
69860005	69859939
71859379	71859313
73662480	73662413
72624271	72624337
73227007	73226941
73680454	73680520
73742585	73742523
74113728	74113662
74189241	74189308
75151106	75151162
74791842	74791776
74707952	74708016
74616597	74616531
1556406	1556350
1544364	1544416
3937981	3938047
4139156	4139222
6782962	6782896
6777316	6777380
8082446	8082512
9634432	9634366

8720240	8720198
8754646	8754711
9164354	9164294
8986194	8986251
15155332	15155390
15155335	15155269
15490540	15490485
17941541	17941475
17941538	17941599
25086722	25086788
30578015	30577949
41721131	41721197
41720767	41720820
41720767	41720833
41948174	41948240
49127617	49127551
52477938	52478002
55766200	55766138
55595575	55595641
57391746	57391791
62015226	62015278
61879408	61879342
61879408	61879342
63782425	63782491
64018213	64018279
64440374	64440440
64434124	64434189
64440374	64440438
64553456	64553390
65361925	65361859
512457	512400
1639675	1639614
49090539	49090473
175502	175436
1255520	1255454
1966104	1966168
2796234	2796195
2796234	2796168
3632675	3632609
3632675	3632609

3973482	3973545
7632595	7632653
7613910	7613844
7880774	7880840
10185410	10185344
10650738	10650695
10874233	10874167
6265887	6265928
14050347	14050414
13985924	13985858
16605627	16605693
16655987	16655921
16983547	16983498
17325515	17325449
18423882	18423941
47980885	47980826
49058422	49058473
50828684	50828730
52020546	52020612
52827041	52826978
53619543	53619596
54182253	54182187
54614742	54614808
54884932	54884998
20726427	20726475
20726999	20727048
6640229	6640295
10314173	10314239
16039530	16039596
16495900	16495834
16634986	16634921
18604569	18604635
19978212	19978278
27326788	27326854
28186293	28186359
29849298	29849237
30227143	30227077
31837110	31837164
32512027	32512064
32512171	32512232

33566373	33566439
34442004	34442064
35366693	35366759
35536574	35536640
35489258	35489192
35605949	35605913
37984616	37984558
38462153	38462194
39999125	39999190
40171151	40171112
40342560	40342626
17856955	17857014
42054484	42054550
42316792	42316854
43672071	43672005
43638882	43638816
43991343	43991287
44720071	44720005
44753195	44753261
44808281	44808347
45139140	45139206
45328341	45328407
45598135	45598072
45824687	45824753
45832786	45832720
46011901	46011966
46550196	46550130
46732313	46732250
48346891	48346957
2423808	2423874
8015155	8015213
13368696	13368762
15435928	15435994
15727236	15727299
15299473	15299407
15681550	15681484
23425304	23425370
23412202	23412136
23571474	23571407
27728420	27728486

27859178	27859112
27981641	27981575
28013992	28014043
29754111	29754165
33326819	33326885
33431921	33431868
34076619	34076678
34394498	34394555
36428815	36428771
36428818	36428870
37178123	37178057
37732594	37732658
39114460	39114394
39886369	39886415
42382172	42382238
42382172	42382238
43405122	43405060
44718933	44718999
46047721	46047786
46040632	46040566
46859640	46859574
47106418	47106465
48640321	48640283
48781070	48781004
44703700	44703655
51473915	51473981
51514752	51514698
51885627	51885684
52425282	52425348
52779458	52779524
53781290	53781224
54329160	54329226
40142244	40142178
55169881	55169940
56101953	56102019
56443222	56443157
9922413	9922479
7855411	7855477
9922413	9922479
17227109	17227175

18556706	18556772
23197352	23197286
23299196	23299132
27821035	27820970
30429217	30429279
28444437	28444502
28755828	28755762
28820834	28820888
29359445	29359511
30428514	30428448
32678344	32678404
33325305	33325371
33375580	33375514
35548402	35548468
37230606	37230543
37206593	37206529
36605184	36605118
36136621	36136678
38790836	38790886
38873300	38873366
39463946	39464012
40851907	40851849
41472614	41472681
43458390	43458456
43513255	43513194
43532961	43532895
43624302	43624236
44502145	44502085
44566539	44566605
44627285	44627220
44679106	44679172
45017344	45017410
45017344	45017409
46948279	46948344
48159751	48159692
48262413	48262479
48271947	48271884
49423656	49423704
49731044	49730977
1959185	1959247

2383672	2383724
2787488	2787422
3810675	3810611
4080957	4081023
4487222	4487156
4864637	4864701
4864637	4864703
5131363	5131303
5390571	5390637
6955438	6955504
7556369	7556417
7550617	7550573
8626607	8626673
8795348	8795414
9838149	9838215
9997317	9997251
11078485	11078551
10889474	10889537
19202269	19202203
18911469	18911413
18921711	18921645
22799450	22799516
61563712	61563772
61412581	61412635
61446900	61446940
61668560	61668494
61875781	61875836
61875781	61875836
63180607	63180644

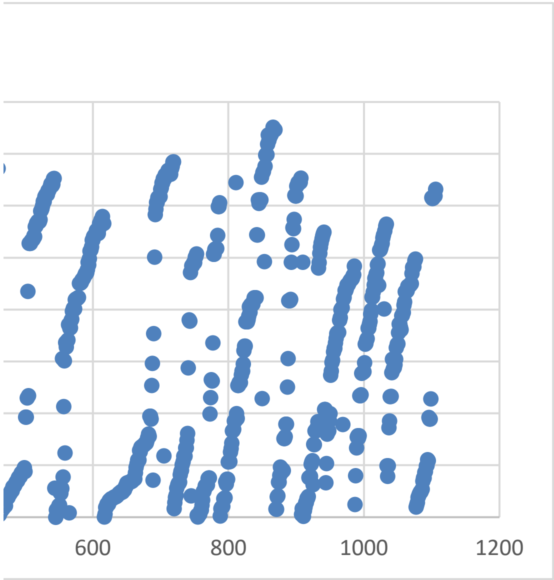

600
800
1000
1200

Table S5. QTL associated with pod shatter resistance, measured using rupture energy with three p[illegible] WGAIM, WinQTL and IciMapping. R2 values represent to genotypic variance analysed by WG[illegible] phenotypic variance analysed by WinQTL and IcIMapping. QTL detected with multiple package[illegible] bold.

Method	Trait	Chromosome	Left Marker	Left Marker Position (cM)	Right Marker	Right Marker Position (cM)	Allelic Effect Size	R2 (%)
WAGIM	RE 2013	B02	100031592	24.23	100047102	24.53	0.65	6.10
ICIM	RE 2014	B02	100034989	44.43	100056502	45.11	0.65	6.01
WinQTL	RE 2014	B02	100071863	46.14	100028417	47.85	0.65	5.59
**WAGIM**	**RE 2014**	**B02**	**100019770**	**49.36**	**100019364**	**49.78**	**0.70**	**6.00**
**ICIM**	**RE 2023**	**B02**	**100019770**	**49.36**	**100019364**	**49.78**	**0.84**	**6.63**
WinQTL	RE 2014	B02	100025883	53.73	100052047	55.92	0.67	6.26
WAGIM	RE 2023	B03	100022962	13.77	100052281	14.15	-1.00	9.00
WinQTL	Re 2023	B03	100045717	16.85	e6m9-250	21.58	-1.10	12.28
ICIM	RE 2023	B03	100062790	20.37	100005456	21.01	-0.88	7.88

WinQTL	RE 2023	B03	100027006	22.49	100014967	31.51	-1.07	11.66
WAGIM	RE 2023	B04	100043850	33.95	100078929	34.38	0.85	6.50
ICIM	RE 2013	B06	100037101	92.45	0RAS116	92.88	0.70	11.30
**WAGIM**	**RE 2013**	**B06**	**100013746**	**94.53**	**100001380**	**94.80**	**0.78**	**8.50**
**WAGIM**	**RE 2023**	**B06**	**100001380**	**94.80**	**100025434**	**101.81**	**0.92**	**7.60**
**WinQTL**	**RE 2013**	**B06**	**100001380**	**94.80**	**100025434**	**101.81**	**0.57**	**6.59**
WinQTL	RE 2013	B06	100025434	101.81	Ni2A09	103.90	0.59	7.09
**WinQTL**	**Re 2023**	**B07**	**100009190**	**19.74**	**100006973**	**26.58**	**0.78**	**6.18**
**WinQTL**	**RE 2013**	**B07**	**100009190**	**19.74**	**100022658**	**21.56**	**0.84**	**13.89**
**WAGIM**	**RE 2013**	**B07**	**100046735**	**20.82**	**100022658**	**21.56**	**1.43**	**27.60**
**WinQTL**	**RE 2014**	**B07**	**100022658**	**21.56**	**100006973**	**26.58**	**1.06**	**16.09**

**WinQTL**	**Re 2023**	**B07**	**100006973**	**26.58**	**100026124**	**31.91**	**0.79**	**6.48**
**WinQTL**	**RE 2013**	**B07**	**100006973**	**26.58**	**100026124**	**31.91**	**0.88**	**15.77**
**WAGIM**	**RE 2023**	**B07**	**100053770**	**28.09**	**100019213**	**29.28**	**0.72**	**4.60**
**WinQTL**	**RE 2014**	**B07**	**100019213**	**29.28**	**100026124**	**31.91**	**1.14**	**18.96**
**ICIM**	**RE 2013**	**B07**	**100020128**	**41.34**	**100019987**	**42.19**	**1.06**	**25.49**
**WinQTL**	**RE 2014**	**B07**	**100020128**	**41.34**	**100013191**	**44.32**	**1.00**	**14.67**
**WinQTL**	**RE 2013**	**B07**	**100019987**	**42.19**	**100042453**	**44.12**	**0.57**	**4.30**
**ICIM**	**RE 2014**	**B07**	**100019987**	**42.19**	**100043465**	**42.90**	**1.22**	**24.38**
**WAGIM**	**RE 2014**	**B07**	**100043465**	**42.90**	**100071901**	**43.31**	**1.22**	**17.30**
ICIM	RE 2014	B08	100013294	3.28	100071515	4.11	0.65	6.96
WAGIM	RE 2014	B08	100024082	12.78	100042491	12.98	0.55	3.80

**WinQTL**	**Re 2023**	**B08**	**100049153**	**17.93**	**100024018**	**19.01**	**0.76**	**5.91**
**ICIM**	**RE 2023**	**B08**	**100049153**	**17.93**	**100002494**	**18.41**	**0.87**	**7.78**
**WAGIM**	**RE 2023**	**B08**	**100002494**	**18.41**	**100024018**	**19.01**	**0.90**	**7.30**
WAGIM	RE 2013	B08	100064677	110.55	100024312	111.38	-0.58	4.90
**WinQTL**	**RE 2013**	**C01**	**100018249**	**70.81**	**100031795**	**87.70**	**0.50**	**5.08**
**WAGIM**	**RE 2013**	**C01**	**Na10H06**	**76.11**	**100031795**	**87.70**	**0.75**	**6.80**
WAGIM	RE 2014	C01	100062611	89.80	100059879	98.74	0.75	6.10
WAGIM	RE 2013	C03	100055560	67.23	100052674	76.30	0.65	5.20
**WinQTL**	**RE 2013**	**C03**	**100034883**	**97.99**	**100012667**	**99.80**	**0.52**	**5.50**
**WinQTL**	**RE 2013**	**C03**	**100012667**	**99.80**	**100018700**	**104.62**	**0.55**	**6.35**
WAGIM	RE 2023	C04	100023632	2.88	100019506	3.09	-0.75	5.00

WinQTL	RE 2014	C04	100029133	137.75	100021627	143.81	0.68	7.02
WAGIM	RE 2014	C04	100008104	140.45	Ol11D12	141.22	0.84	8.30
WinQTL	Re 2023	C06	100028657	29.36	C010234	33.10	-0.84	5.93
**WinQTL**	**Re 2023**	**C06**	**HG-FT-C6**	**67.92**	**100061440**	**69.89**	**1.31**	**13.95**
**ICIM**	**RE 2023**	**C06**	**HG-FT-C6**	**67.92**	**100017755**	**69.32**	**1.05**	**11.25**
**WAGIM**	**RE 2023**	**C06**	**HG-FT-C6**	**67.92**	**100017755**	**69.32**	**1.12**	**11.20**
WinQTL	RE 2014	C07	100020742	15.86	100030964	31.45	0.58	5.09
WAGIM	RE 2014	C07	100065291	22.83	100037708	26.01	0.67	5.40

ackages, AIM and s are in
LOD
2.96
4.07
3.03
**3.04**
**4.01**
3.42
4.63
5.88
4.72

4.60
3.17
6.02
**4.79**
**3.62**
**3.56**
3.96
**3.09**
**7.26**
**15.63**
**7.53**

2.96

8.33

2.42

9.00

12.22

7.32

2.62

12.94

10.21

4.67

2.29

2.96

4.43

3.76

2.66

2.67

3.90

3.71

3.01

3.04

3.52

2.52

3.78

4.93

2.97

6.41

6.51

5.67

2.81

3.09

Table S6. Marker haplotypes generated from the SNP markers using LD block detection method.

Markers	Chromosome	Position	Block #
M294311	B01	294311	1
M323926	B01	323926	1
M337281	B01	337281	1
M345324	B01	345324	1
M378298	B01	378298	2
M415256	B01	415256	2
M416231	B01	416231	2
M422308	B01	422308	2
M425753	B01	425753	2
M428298	B01	428298	2
M432529	B01	432529	2
M438308	B01	438308	2
M443398	B01	443398	2
M447190	B01	447190	2
M448375	B01	448375	2
M454621	B01	454621	2
M459213	B01	459213	2
M463331	B01	463331	2
M468721	B01	468721	2
M498285	B01	498285	2
M535608	B01	535608	2
M537375	B01	537375	2
M544310	B01	544310	3
M547273	B01	547273	3
M549816	B01	549816	3
M557321	B01	557321	3
M571872	B01	571872	3
M583745	B01	583745	3
M586483	B01	586483	3
M588495	B01	588495	3
M596339	B01	596339	3
M598344	B01	598344	4
M599814	B01	599814	4
M602057	B01	602057	4
M756462	B01	756462	4
M771716	B01	771716	5
M786925	B01	786925	5
M794326	B01	794326	5
M804145	B01	804145	5
M806085	B01	806085	5
M818105	B01	818105	5
M821670	B01	821670	5
M824990	B01	824990	5
M834396	B01	834396	5

M847145	B01	847145	5
M857081	B01	857081	5
M868969	B01	868969	5
M874112	B01	874112	5
M885077	B01	885077	5
M895092	B01	895092	5
M926678	B01	926678	6
M976868	B01	976868	6
M988918	B01	988918	6
M992122	B01	992122	6
M100540	B01	1005400	7
M102427	B01	1024278	7
M116385	B01	1163858	7
M118729	B01	1187294	8
M118974	B01	1189746	8
M126124	B01	1261244	9
M130602	B01	1306026	9
M130811	B01	1308113	9
M131358	B01	1313583	9
M131785	B01	1317853	9
M131907	B01	1319074	9
M135397	B01	1353979	9
M135509	B01	1355099	9
M137517	B01	1375179	9
M138048	B01	1380485	9
M140087	B01	1400878	9
M160534	B01	1605347	10
M160847	B01	1608470	10
M166520	B01	1665208	10
M171635	B01	1716356	10
M171641	B01	1716410	10
M171761	B01	1717612	10
M172398	B01	1723981	10
M172848	B01	1728488	10
M173215	B01	1732154	10
M174992	B01	1749924	11
M175226	B01	1752264	11
M175475	B01	1754751	11
M176191	B01	1761913	11
M176255	B01	1762559	11
M176742	B01	1767425	11
M177428	B01	1774281	11
M178407	B01	1784074	11
M181470	B01	1814708	11
M181946	B01	1819466	11
M182572	B01	1825729	11
M183105	B01	1831054	11

M183735	B01	1837352	11
M185744	B01	1857440	11
M186193	B01	1861937	11
M187954	B01	1879542	11
M188751	B01	1887519	11
M189298	B01	1892981	11
M189700	B01	1897005	11
M190026	B01	1900262	11
M190873	B01	1908734	11
M191507	B01	1915073	12
M191837	B01	1918371	12
M192051	B01	1920519	12
M192584	B01	1925848	12
M193093	B01	1930933	12
M193605	B01	1936054	12
M194952	B01	1949525	12
M195998	B01	1959984	12
M196117	B01	1961171	12
M196529	B01	1965299	12
M197236	B01	1972364	12
M198285	B01	1982858	12
M199794	B01	1997941	12
M201347	B01	2013479	12
M204073	B01	2040732	12
M222945	B01	2229453	13
M223526	B01	2235264	13
M223950	B01	2239506	13
M224198	B01	2241983	13
M224505	B01	2245057	13
M228383	B01	2283837	13
M231264	B01	2312642	13
M233298	B01	2332983	14
M233538	B01	2335389	14
M234023	B01	2340233	14
M234167	B01	2341678	14
M243876	B01	2438766	14
M244139	B01	2441399	14
M244618	B01	2446185	14
M244677	B01	2446774	14
M247317	B01	2473176	14
M249163	B01	2491639	14
M272938	B01	2729384	15
M273483	B01	2734837	15
M273680	B01	2736805	15
M273881	B01	2738817	15
M274090	B01	2740900	15
M275202	B01	2752024	15

M275773	B01	2757732	15
M276418	B01	2764188	15
M277442	B01	2774427	15
M300663	B01	3006630	16
M301100	B01	3011000	16
M301119	B01	3011195	16
M301613	B01	3016137	16
M302580	B01	3025808	16
M303439	B01	3034392	16
M303672	B01	3036720	16
M304052	B01	3040523	16
M304786	B01	3047868	16
M304984	B01	3049842	16
M305349	B01	3053496	16
M305608	B01	3056080	16
M309295	B01	3092957	16
M309900	B01	3099001	16
M311159	B01	3111599	17
M311331	B01	3113310	17
M311789	B01	3117897	17
M312784	B01	3127847	18
M315288	B01	3152886	18
M320516	B01	3205168	18
M321216	B01	3212165	18
M326484	B01	3264849	18
M328784	B01	3287842	18
M355521	B01	3555215	19
M355878	B01	3558787	19
M356267	B01	3562675	19
M356776	B01	3567764	19
M358010	B01	3580104	19
M359391	B01	3593917	19
M359770	B01	3597707	19
M360882	B01	3608822	19
M361827	B01	3618270	19
M362154	B01	3621548	19
M362484	B01	3624842	19
M362748	B01	3627489	19
M363701	B01	3637016	19
M364482	B01	3644829	19
M364610	B01	3646105	19
M364910	B01	3649108	19
M365055	B01	3650550	19
M367584	B01	3675844	19
M368025	B01	3680259	19
M368326	B01	3683262	19
M368509	B01	3685098	19

M370556	B01	3705563	19
M372956	B01	3729563	20
M381245	B01	3812451	20
M383440	B01	3834407	21
M392651	B01	3926517	21
M393093	B01	3930936	21
M393349	B01	3933493	21
M393991	B01	3939918	21
M395409	B01	3954094	21
M397597	B01	3975978	21
M399511	B01	3995116	22
M400239	B01	4002394	22
M401002	B01	4010025	22
M401471	B01	4014715	22
M402428	B01	4024283	22
M403405	B01	4034056	22
M404176	B01	4041767	22
M404435	B01	4044352	22
M434555	B01	4345550	23
M434888	B01	4348881	23
M435133	B01	4351333	23
M437331	B01	4373314	24
M438803	B01	4388034	24
M447269	B01	4472694	24
M449283	B01	4492833	24
M449704	B01	4497048	24
M451720	B01	4517200	24
M452014	B01	4520149	24
M453021	B01	4530210	24
M453550	B01	4535506	25
M453737	B01	4537374	25
M454103	B01	4541030	25
M455279	B01	4552799	25
M455450	B01	4554509	25
M456052	B01	4560521	25
M456481	B01	4564810	25
M456683	B01	4566835	25
M457384	B01	4573849	25
M459057	B01	4590577	25
M459707	B01	4597070	25
M460995	B01	4609957	26
M461075	B01	4610755	26
M461779	B01	4617798	26
M462702	B01	4627028	26
M462808	B01	4628088	26
M463011	B01	4630111	26
M469130	B01	4691300	26

M474683	B01	4746833	26
M475306	B01	4753067	26
M476395	B01	4763954	26
M476838	B01	4768380	26
M485437	B01	4854378	27
M486771	B01	4867711	27
M500054	B01	5000542	27
M525929	B01	5259292	28
M526690	B01	5266905	28
M526781	B01	5267812	28
M533065	B01	5330650	28
M533686	B01	5336863	28
M534685	B01	5346859	28
M537826	B01	5378269	28
M537982	B01	5379824	28
M539479	B01	5394790	28
M541013	B01	5410132	28
M542935	B01	5429357	29
M543223	B01	5432233	29
M545716	B01	5457167	29
M546249	B01	5462493	29
M546430	B01	5464307	29
M546736	B01	5467360	29
M547315	B01	5473150	29
M549742	B01	5497423	30
M561469	B01	5614699	30
M591266	B01	5912668	31
M591698	B01	5916981	31
M595680	B01	5956808	31
M595994	B01	5959948	31
M598388	B01	5983887	31
M598996	B01	5989966	31
M600212	B01	6002124	31
M600458	B01	6004584	31
M602240	B01	6022400	31
M603174	B01	6031741	31
M604097	B01	6040976	31
M606205	B01	6062056	31
M606811	B01	6068113	31
M606944	B01	6069440	31
M608331	B01	6083318	32
M618780	B01	6187805	32
M619806	B01	6198068	32
M620233	B01	6202330	32
M620558	B01	6205581	32
M620803	B01	6208031	32
M624156	B01	6241569	32

M624447	B01	6244476	33
M625464	B01	6254648	33
M625923	B01	6259234	33
M626771	B01	6267711	33
M627769	B01	6277698	33
M627868	B01	6278688	33
M628343	B01	6283439	33
M628608	B01	6286085	33
M629058	B01	6290582	33
M629227	B01	6292278	33
M629393	B01	6293933	33
M629946	B01	6299461	33
M630640	B01	6306408	33
M681023	B01	6810238	34
M681441	B01	6814413	34
M682321	B01	6823219	35
M682524	B01	6825243	35
M685796	B01	6857968	35
M686444	B01	6864442	35
M687832	B01	6878325	35
M689145	B01	6891454	35
M690145	B01	6901450	35
M690779	B01	6907797	35
M693602	B01	6936026	36
M694256	B01	6942560	36
M694823	B01	6948231	36
M695074	B01	6950742	36
M695723	B01	6957233	36
M696696	B01	6966965	36
M697481	B01	6974814	36
M698555	B01	6985555	36
M702415	B01	7024152	36
M703531	B01	7035319	37
M705533	B01	7055336	37
M715121	B01	7151210	37
M829356	B01	8293560	38
M837908	B01	8379088	38
M852961	B01	8529616	39
M854021	B01	8540214	39
M856802	B01	8568025	39
M857603	B01	8576031	39
M859452	B01	8594526	39
M861040	B01	8610402	39
M862784	B01	8627849	39
M868505	B01	8685059	39
M868804	B01	8688047	39
M870635	B01	8706359	40

M871094	B01	8710948	40
M871429	B01	8714298	40
M877905	B01	8779050	40
M882575	B01	8825754	41
M883022	B01	8830222	41
M883542	B01	8835420	41
M886644	B01	8866442	41
M887319	B01	8873191	41
M888457	B01	8884576	41
M888837	B01	8888373	41
M889144	B01	8891443	42
M889353	B01	8893533	42
M889789	B01	8897899	42
M891066	B01	8910663	42
M892037	B01	8920372	42
M892836	B01	8928364	42
M896288	B01	8962881	42
M897690	B01	8976904	42
M897839	B01	8978398	42
M898693	B01	8986935	42
M899211	B01	8992111	42
M899768	B01	8997689	42
M901298	B01	9012986	42
M905041	B01	9050417	42
M928773	B01	9287736	43
M929564	B01	9295649	43
M930273	B01	9302733	43
M931150	B01	9311508	43
M931572	B01	9315727	44
M932934	B01	9329347	44
M933206	B01	9332063	44
M934879	B01	9348792	44
M936426	B01	9364263	44
M936796	B01	9367966	44
M937853	B01	9378534	44
M941076	B01	9410762	44
M941907	B01	9419077	44
M944653	B01	9446533	44
M945409	B01	9454099	44
M945760	B01	9457602	44
M946491	B01	9464918	44
M946712	B01	9467121	44
M947473	B01	9474733	44
M950718	B01	9507189	45
M955571	B01	9555717	45
M958450	B01	9584508	45
M962980	B01	9629808	45

M965968	B01	9659686	45
M966428	B01	9664284	45
M969299	B01	9692990	46
M972170	B01	9721704	46
M972586	B01	9725863	46
M986335	B01	9863352	47
M987536	B01	9875365	47
M988278	B01	9882789	47
M989472	B01	9894726	47
M991790	B01	9917908	47
M992125	B01	9921255	47
M994998	B01	9949989	47
M995604	B01	9956040	47
M996725	B01	9967252	47
M997557	B01	9975573	47
M998549	B01	9985498	47
M999168	B01	9991687	47
M100053	B01	10005386	47
M100189	B01	10018966	48
M100230	B01	10023001	48
M100285	B01	10028538	48
M100386	B01	10038670	48
M100420	B01	10042046	48
M100444	B01	10044497	48
M100492	B01	10049241	48
M101181	B01	10118117	48
M101232	B01	10123217	48
M101445	B01	10144568	48
M101565	B01	10156542	48
M101590	B01	10159062	48
M101669	B01	10166930	48
M101786	B01	10178675	48
M104697	B01	10469789	49
M104843	B01	10484375	49
M107740	B01	10774068	50
M108068	B01	10806829	50
M108102	B01	10810228	50
M108131	B01	10813123	50
M108183	B01	10818376	50
M108245	B01	10824568	50
M108308	B01	10830876	50
M108480	B01	10848019	50
M108728	B01	10872885	50
M108970	B01	10897069	50
M109066	B01	10906636	50
M109118	B01	10911883	50
M109154	B01	10915429	50

M109190	B01	10919017	51
M109299	B01	10929954	51
M109430	B01	10943025	51
M110046	B01	11004663	51
M110132	B01	11013273	51
M110227	B01	11022759	51
M110347	B01	11034760	51
M110784	B01	11078491	51
M111039	B01	11103905	52
M112525	B01	11252576	52
M112685	B01	11268568	53
M112990	B01	11299016	53
M113000	B01	11300015	53
M113368	B01	11336829	53
M113410	B01	11341036	53
M113516	B01	11351634	53
M113540	B01	11354045	53
M113740	B01	11374035	53
M113826	B01	11382677	53
M114086	B01	11408692	54
M114105	B01	11410552	54
M114726	B01	11472605	54
M114947	B01	11494708	54
M115310	B01	11531032	54
M115593	B01	11559366	54
M115686	B01	11568658	54
M115848	B01	11584824	55
M115967	B01	11596720	55
M116292	B01	11629259	55
M116591	B01	11659175	56
M116875	B01	11687549	56
M118000	B01	11800009	56
M118146	B01	11814600	56
M118367	B01	11836713	57
M118710	B01	11871006	57
M119331	B01	11933197	58
M119339	B01	11933958	58
M119592	B01	11959296	58
M120096	B01	12009600	58
M120328	B01	12032858	58
M120431	B01	12043151	58
M120596	B01	12059671	58
M120709	B01	12070976	58
M120768	B01	12076845	58
M120915	B01	12091578	59
M121118	B01	12111833	59
M121359	B01	12135970	59

M121735	B01	12173588	59
M121828	B01	12182878	59
M122044	B01	12204443	59
M122280	B01	12228030	59
M122514	B01	12251456	59
M122761	B01	12276175	60
M122922	B01	12292214	60
M123073	B01	12307337	60
M123117	B01	12311773	60
M123452	B01	12345264	61
M123485	B01	12348503	61
M123607	B01	12360747	61
M123691	B01	12369147	61
M123778	B01	12377827	62
M123861	B01	12386188	62
M124011	B01	12401111	62
M124057	B01	12405791	62
M124222	B01	12422242	62
M124376	B01	12437644	62
M124556	B01	12455622	62
M125099	B01	12509957	62
M125192	B01	12519283	62
M125300	B01	12530041	62
M125321	B01	12532160	62
M125365	B01	12536528	62
M125549	B01	12554954	63
M126021	B01	12602111	63
M126113	B01	12611382	63
M126367	B01	12636756	63
M126445	B01	12644588	63
M126489	B01	12648974	63
M126754	B01	12675498	64
M127221	B01	12722132	64
M127506	B01	12750623	64
M127607	B01	12760740	64
M127609	B01	12760907	64
M127834	B01	12783455	64
M128102	B01	12810207	64
M128233	B01	12823386	64
M128274	B01	12827492	64
M128348	B01	12834886	64
M128459	B01	12845988	65
M128545	B01	12854502	65
M128985	B01	12898556	66
M129042	B01	12904284	66
M129281	B01	12928102	66
M129403	B01	12940343	66

M129490	B01	12949014	66
M129562	B01	12956236	66
M129654	B01	12965405	66
M129705	B01	12970592	66
M129840	B01	12984004	66
M130158	B01	13015856	66
M130185	B01	13018538	66
M130212	B01	13021209	66
M130531	B01	13053157	66
M130584	B01	13058485	66
M130682	B01	13068207	67
M130703	B01	13070310	67
M130772	B01	13077298	67
M130938	B01	13093852	67
M130977	B01	13097781	67
M131031	B01	13103152	67
M131245	B01	13124586	67
M131933	B01	13193323	67
M133286	B01	13328693	68
M134815	B01	13481574	68
M135361	B01	13536171	69
M135554	B01	13555440	69
M135888	B01	13588890	69
M138299	B01	13829993	70
M138448	B01	13844802	70
M138518	B01	13851891	70
M138609	B01	13860976	70
M139146	B01	13914643	70
M139455	B01	13945549	70
M139747	B01	13974781	70
M140373	B01	14037344	71
M140873	B01	14087360	71
M140934	B01	14093438	71
M141248	B01	14124831	72
M141660	B01	14166073	72
M142216	B01	14221657	72
M142713	B01	14271332	72
M143045	B01	14304563	73
M143127	B01	14312729	73
M143651	B01	14365130	73
M143784	B01	14378429	73
M143790	B01	14379065	73
M143842	B01	14384247	73
M143993	B01	14399364	73
M144088	B01	14408864	73
M144416	B01	14441612	73
M144507	B01	14450726	73

M144640	B01	14464007	73
M148050	B01	14805048	74
M148063	B01	14806351	74
M165489	B01	16548981	75
M166259	B01	16625959	75
M167013	B01	16701331	76
M167569	B01	16756956	76
M168703	B01	16870367	77
M169098	B01	16909881	77
M175154	B01	17515464	78
M175722	B01	17572249	78
M191143	B01	19114301	79
M192215	B01	19221569	79
M204143	B01	20414396	80
M205132	B01	20513268	80
M205492	B01	20549285	80
M211801	B01	21180152	81
M213066	B01	21306699	81
M256814	B01	25681481	82
M258004	B01	25800447	82
M259922	B01	25992204	83
M261418	B01	26141875	83
M262266	B01	26226600	84
M262355	B01	26235572	84
M266646	B01	26664675	85
M266790	B01	26679087	85
M267801	B01	26780196	86
M268512	B01	26851285	86
M270930	B01	27093034	87
M271005	B01	27100569	87
M271098	B01	27109856	88
M271234	B01	27123445	88
M271328	B01	27132868	88
M271560	B01	27156097	88
M271733	B01	27173322	88
M272675	B01	27267595	88
M273695	B01	27369538	89
M273784	B01	27378471	89
M273975	B01	27397518	89
M274102	B01	27410234	89
M274238	B01	27423832	89
M274729	B01	27472990	89
M277228	B01	27722847	90
M277465	B01	27746557	90
M277619	B01	27761931	90
M278322	B01	27832215	91
M278546	B01	27854645	91

M279212	B01	27921218	92
M279368	B01	27936807	92
M279520	B01	27952085	93
M279559	B01	27955992	93
M279700	B01	27970076	93
M280110	B01	28011049	94
M280631	B01	28063131	94
M281431	B01	28143185	95
M281941	B01	28194102	95
M282217	B01	28221795	95
M282260	B01	28226017	95
M282647	B01	28264722	95
M283060	B01	28306086	96
M283347	B01	28334713	96
M283725	B01	28372568	96
M283925	B01	28392532	96
M283978	B01	28397811	96
M284072	B01	28407265	96
M284282	B01	28428240	96
M284381	B01	28438183	96
M284434	B01	28443428	96
M284620	B01	28462062	96
M284936	B01	28493608	97
M284985	B01	28498559	97
M285350	B01	28535035	98
M285480	B01	28548026	98
M285653	B01	28565330	98
M285780	B01	28578076	98
M286161	B01	28616127	98
M286245	B01	28624569	99
M286282	B01	28628291	99
M286653	B01	28665339	100
M286957	B01	28695749	100
M286985	B01	28698579	100
M287062	B01	28706240	100
M287134	B01	28713448	101
M287432	B01	28743275	101
M287625	B01	28762598	101
M287731	B01	28773199	102
M287801	B01	28780174	102
M288109	B01	28810928	103
M288141	B01	28814118	103
M288249	B01	28824910	104
M288397	B01	28839707	104
M288433	B01	28843351	105
M288466	B01	28846676	105
M288565	B01	28856566	105

M288593	B01	28859313	105
M289213	B01	28921380	105
M289231	B01	28923119	106
M289255	B01	28925560	106
M289315	B01	28931594	106
M289486	B01	28948686	107
M289552	B01	28955283	107
M289761	B01	28976132	108
M289901	B01	28990110	108
M291158	B01	29115812	108
M291258	B01	29125866	108
M291521	B01	29152117	109
M291849	B01	29184992	109
M294798	B01	29479873	110
M295191	B01	29519178	110
M295502	B01	29550265	110
M295943	B01	29594374	110
M295972	B01	29597230	110
M296107	B01	29610731	110
M296291	B01	29629136	111
M296626	B01	29662615	111
M297717	B01	29771720	111
M297949	B01	29794992	112
M298044	B01	29804410	112
M298111	B01	29811163	113
M298161	B01	29816126	113
M298287	B01	29828701	113
M298357	B01	29835777	113
M301783	B01	30178342	114
M301918	B01	30191885	114
M302276	B01	30227633	114
M302591	B01	30259168	115
M302619	B01	30261946	115
M302652	B01	30265263	115
M302749	B01	30274920	116
M302961	B01	30296135	116
M303000	B01	30300028	116
M303241	B01	30324106	116
M303450	B01	30345015	117
M303745	B01	30374506	117
M303820	B01	30382054	117
M304016	B01	30401674	117
M304227	B01	30422755	117
M304500	B01	30450037	117
M304716	B01	30471682	118
M304828	B01	30482873	118
M305036	B01	30503603	118

M305181	B01	30518172	118
M305295	B01	30529558	119
M305369	B01	30536994	119
M305429	B01	30542916	120
M305465	B01	30546567	120
M305542	B01	30554220	120
M305620	B01	30562014	121
M305691	B01	30569146	121
M305989	B01	30598996	121
M306085	B01	30608577	121
M306203	B01	30620374	121
M306537	B01	30653718	121
M314429	B01	31442978	122
M315899	B01	31589995	122
M315968	B01	31596831	122
M316049	B01	31604900	123
M316078	B01	31607833	123
M316265	B01	31626592	123
M316717	B01	31671778	124
M316755	B01	31675588	124
M316810	B01	31681091	124
M316854	B01	31685431	124
M316938	B01	31693816	124
M317034	B01	31703415	124
M317235	B01	31723571	125
M317255	B01	31725578	125
M317313	B01	31731398	125
M317413	B01	31741346	125
M317620	B01	31762067	125
M317693	B01	31769341	126
M317936	B01	31793644	126
M318525	B01	31852562	127
M318608	B01	31860860	127
M318687	B01	31868762	127
M318835	B01	31883561	127
M318940	B01	31894099	127
M318969	B01	31896954	127
M319060	B01	31906070	127
M319279	B01	31927919	127
M319584	B01	31958443	127
M319798	B01	31979889	128
M319878	B01	31987824	128
M319901	B01	31990187	129
M320009	B01	32000968	129
M320979	B01	32097985	130
M321196	B01	32119641	130
M321372	B01	32137267	131

M321574	B01	32157442	131
M321648	B01	32164852	132
M321681	B01	32168177	132
M321697	B01	32169701	133
M321748	B01	32174862	133
M322109	B01	32210995	133
M322247	B01	32224785	134
M322947	B01	32294777	134
M323054	B01	32305451	134
M323125	B01	32312583	134
M323336	B01	32333672	135
M323755	B01	32375537	135
M323874	B01	32387409	135
M323924	B01	32392498	135
M323973	B01	32397351	136
M324058	B01	32405842	136
M324409	B01	32440996	137
M324440	B01	32444047	137
M324472	B01	32447202	137
M324487	B01	32448723	138
M324548	B01	32454832	138
M324561	B01	32456119	139
M324571	B01	32457145	139
M324586	B01	32458614	139
M324973	B01	32497363	139
M325027	B01	32502722	139
M325050	B01	32505021	140
M325077	B01	32507729	140
M325108	B01	32510802	140
M325374	B01	32537494	141
M325420	B01	32542047	141
M325440	B01	32544087	141
M325624	B01	32562473	142
M325720	B01	32572038	142
M325758	B01	32575820	143
M325833	B01	32583369	143
M325920	B01	32592031	144
M325954	B01	32595414	144
M326334	B01	32633487	145
M326383	B01	32638341	145
M326400	B01	32640025	145
M326651	B01	32665160	145
M326719	B01	32671938	145
M326954	B01	32695415	145
M327132	B01	32713294	145
M327221	B01	32722142	146
M327239	B01	32723994	146

M327267	B01	32726709	147
M327403	B01	32740329	147
M327560	B01	32756071	147
M327685	B01	32768591	148
M327809	B01	32780971	148
M327929	B01	32792940	149
M328071	B01	32807104	149
M328118	B01	32811897	149
M328165	B01	32816565	149
M328384	B01	32838461	149
M328385	B01	32838592	149
M328617	B01	32861786	150
M328657	B01	32865736	150
M328663	B01	32866343	150
M328760	B01	32876052	151
M328835	B01	32883515	151
M328847	B01	32884789	151
M328911	B01	32891100	152
M328961	B01	32896158	152
M328996	B01	32899663	152
M329224	B01	32922489	153
M329261	B01	32926180	153
M329355	B01	32935532	154
M329384	B01	32938476	154
M329433	B01	32943306	154
M329760	B01	32976056	154
M330022	B01	33002238	155
M330049	B01	33004959	155
M330192	B01	33019297	155
M330293	B01	33029358	155
M332012	B01	33201216	156
M332034	B01	33203454	156
M332049	B01	33204939	156
M332101	B01	33210106	156
M332792	B01	33279274	157
M332872	B01	33287238	157
M333046	B01	33304694	158
M333266	B01	33326696	158
M333302	B01	33330252	159
M333327	B01	33332738	159
M333623	B01	33362389	160
M333650	B01	33365029	160
M335094	B01	33509463	160
M335474	B01	33547438	161
M336725	B01	33672569	161
M336727	B01	33672757	161
M336795	B01	33679585	162

M336860	B01	33686013	162
M336943	B01	33694312	162
M337162	B01	33716294	163
M337265	B01	33726559	163
M337313	B01	33731302	164
M337445	B01	33744518	164
M337651	B01	33765192	165
M337692	B01	33769205	165
M337717	B01	33771700	166
M337722	B01	33772253	166
M337858	B01	33785837	167
M337921	B01	33792120	167
M338048	B01	33804879	167
M338395	B01	33839589	167
M338759	B01	33875912	168
M340327	B01	34032714	168
M341608	B01	34160819	169
M341710	B01	34171081	169
M341733	B01	34173344	169
M341742	B01	34174208	169
M341808	B01	34180877	170
M341832	B01	34183285	170
M341870	B01	34187072	171
M341972	B01	34197253	171
M342053	B01	34205328	172
M342230	B01	34223076	172
M342424	B01	34242451	172
M342498	B01	34249862	173
M342524	B01	34252401	173
M342536	B01	34253635	173
M342596	B01	34259646	173
M342934	B01	34293499	174
M342991	B01	34299195	174
M343068	B01	34306807	175
M343082	B01	34308227	175
M343260	B01	34326095	175
M343288	B01	34328880	175
M343575	B01	34357559	175
M343701	B01	34370126	176
M343815	B01	34381557	176
M345472	B01	34547273	177
M345501	B01	34550178	177
M345557	B01	34555734	178
M345580	B01	34558034	178
M345622	B01	34562231	178
M345659	B01	34565983	178
M345708	B01	34570818	178

M345744	B01	34574437	179
M345776	B01	34577671	179
M345815	B01	34581501	179
M345840	B01	34584003	179
M345846	B01	34584629	180
M345884	B01	34588455	180
M346387	B01	34638796	181
M346464	B01	34646498	181
M346501	B01	34650124	182
M346534	B01	34653410	182
M346689	B01	34668941	183
M346795	B01	34679523	183
M347095	B01	34709556	184
M347147	B01	34714774	184
M347227	B01	34722783	184
M347251	B01	34725168	184
M347287	B01	34728718	184
M347354	B01	34735450	184
M347574	B01	34757499	185
M347587	B01	34758714	185
M347896	B01	34789671	186
M347998	B01	34799822	186
M351798	B01	35179888	187
M351922	B01	35192286	187
M351964	B01	35196497	187
M351973	B01	35197395	187
M353434	B01	35343479	188
M353580	B01	35358021	188
M354064	B01	35406417	189
M354102	B01	35410211	189
M354125	B01	35412567	189
M356247	B01	35624786	190
M356301	B01	35630156	190
M356371	B01	35637102	190
M356420	B01	35642001	191
M356544	B01	35654448	191
M356673	B01	35667316	191
M356718	B01	35671897	191
M357069	B01	35706916	191
M357151	B01	35715130	191
M357195	B01	35719572	191
M357248	B01	35724839	191
M357264	B01	35726499	192
M357324	B01	35732445	192
M357379	B01	35737902	192
M357455	B01	35745521	193
M357507	B01	35750793	193

M357559	B01	35755972	193
M357580	B01	35758032	193
M359063	B01	35906351	194
M359333	B01	35933312	194
M359350	B01	35935009	195
M359396	B01	35939634	195
M359452	B01	35945256	195
M359504	B01	35950495	196
M359564	B01	35956459	196
M359591	B01	35959145	197
M359650	B01	35965001	197
M359684	B01	35968452	197
M359775	B01	35977515	198
M360905	B01	36090538	198
M360911	B01	36091162	198
M361467	B01	36146749	199
M361698	B01	36169881	199
M361764	B01	36176492	200
M361806	B01	36180628	200
M361833	B01	36183376	201
M361867	B01	36186754	201
M361880	B01	36188039	202
M362399	B01	36239932	202
M362460	B01	36246070	203
M362646	B01	36264668	203
M362876	B01	36287640	203
M362914	B01	36291467	203
M363262	B01	36326232	203
M363406	B01	36340695	203
M363419	B01	36341967	203
M363537	B01	36353701	204
M363570	B01	36357062	204
M363717	B01	36371765	205
M363834	B01	36383481	205
M365228	B01	36522807	206
M365913	B01	36591331	206
M366085	B01	36608573	207
M366184	B01	36618412	207
M366278	B01	36627839	207
M366283	B01	36628308	208
M366302	B01	36630284	208
M366329	B01	36632911	208
M366407	B01	36640724	209
M366639	B01	36663955	209
M366670	B01	36667050	209
M366763	B01	36676358	209
M366780	B01	36678043	209

M366822	B01	36682280	209
M366871	B01	36687100	209
M366969	B01	36696991	210
M367007	B01	36700769	210
M367367	B01	36736760	211
M367385	B01	36738540	211
M367528	B01	36752804	212
M367548	B01	36754852	212
M367659	B01	36765989	213
M367694	B01	36769420	213
M367710	B01	36771096	214
M367744	B01	36774437	214
M367832	B01	36783235	214
M367900	B01	36790090	214
M367987	B01	36798718	214
M368072	B01	36807272	214
M368185	B01	36818502	215
M368316	B01	36831690	215
M368343	B01	36834378	215
M369017	B01	36901789	215
M369063	B01	36906340	216
M369157	B01	36915767	216
M369191	B01	36919110	216
M369304	B01	36930480	217
M369332	B01	36933256	217
M369362	B01	36936253	217
M369968	B01	36996898	218
M370031	B01	37003156	218
M370057	B01	37005771	218
M370137	B01	37013740	218
M370198	B01	37019838	218
M370322	B01	37032296	218
M370898	B01	37089890	218
M370974	B01	37097413	219
M370990	B01	37099059	219
M371980	B01	37198066	220
M372143	B01	37214303	220
M372260	B01	37226034	220
M372280	B01	37228039	220
M372388	B01	37238870	221
M372582	B01	37258289	221
M373805	B01	37380548	221
M373828	B01	37382865	221
M373838	B01	37383827	221
M373861	B01	37386102	221
M373923	B01	37392355	221
M373999	B01	37399988	222

M374034	B01	37403499	222
M374602	B01	37460285	222
M374617	B01	37461740	222
M374836	B01	37483619	223
M374873	B01	37487345	223
M374910	B01	37491082	223
M374951	B01	37495178	223
M375089	B01	37508952	223
M376180	B01	37618073	224
M376198	B01	37619898	224
M376227	B01	37622741	224
M376265	B01	37626580	224
M376301	B01	37630120	224
M376527	B01	37652733	225
M376560	B01	37656012	225
M377207	B01	37720754	226
M377241	B01	37724145	226
M377299	B01	37729909	226
M377311	B01	37731181	226
M377770	B01	37777096	227
M378082	B01	37808274	227
M380150	B01	38015049	228
M380221	B01	38022187	228
M380302	B01	38030213	228
M380500	B01	38050026	228
M382643	B01	38264314	229
M382669	B01	38266991	229
M382939	B01	38293904	229
M383058	B01	38305889	230
M383517	B01	38351774	230
M384186	B01	38418619	231
M384212	B01	38421258	231
M384228	B01	38422890	231
M384347	B01	38434755	232
M384375	B01	38437565	232
M384396	B01	38439669	232
M384570	B01	38457015	233
M384590	B01	38459007	233
M385374	B01	38537497	234
M385424	B01	38542446	234
M385453	B01	38545380	234
M385515	B01	38551576	235
M385570	B01	38557001	235
M390778	B01	39077847	236
M390827	B01	39082799	236
M390857	B01	39085711	236
M390901	B01	39090171	236

M390997	B01	39099730	236
M391870	B01	39187070	236
M394711	B01	39471130	237
M394743	B01	39474397	237
M394889	B01	39488945	237
M395126	B01	39512649	238
M395152	B01	39515249	238
M395190	B01	39519086	238
M395227	B01	39522746	238
M395428	B01	39542891	238
M395443	B01	39544333	238
M395711	B01	39571161	238
M395745	B01	39574536	239
M395767	B01	39576742	239
M395944	B01	39594419	240
M396119	B01	39611974	240
M396579	B01	39657964	240
M396580	B01	39658021	240
M396635	B01	39663584	240
M396743	B01	39674312	240
M396836	B01	39683676	240
M396874	B01	39687424	240
M396889	B01	39688950	240
M396911	B01	39691163	240
M396926	B01	39692693	240
M397286	B01	39728691	241
M397401	B01	39740149	241
M397460	B01	39746004	241
M397504	B01	39750428	241
M397564	B01	39756414	241
M94318	B02	94318	242
M97250	B02	97250	242
M98613	B02	98613	242
M102007	B02	102007	242
M103374	B02	103374	242
M107397	B02	107397	242
M183082	B02	183082	243
M341431	B02	341431	243
M350390	B02	350390	244
M354922	B02	354922	244
M641409	B02	641409	245
M642914	B02	642914	245
M649687	B02	649687	245
M657251	B02	657251	245
M661521	B02	661521	245
M666133	B02	666133	245
M680834	B02	680834	245

M683688	B02	683688	245
M687874	B02	687874	245
M697194	B02	697194	245
M699550	B02	699550	245
M701575	B02	701575	245
M705094	B02	705094	245
M709352	B02	709352	245
M716102	B02	716102	245
M725722	B02	725722	246
M730257	B02	730257	246
M735824	B02	735824	246
M790443	B02	790443	246
M802040	B02	802040	246
M804918	B02	804918	246
M831087	B02	831087	247
M832331	B02	832331	247
M838432	B02	838432	248
M848754	B02	848754	248
M857538	B02	857538	248
M861981	B02	861981	248
M884545	B02	884545	248
M898920	B02	898920	248
M905276	B02	905276	248
M915912	B02	915912	248
M917014	B02	917014	248
M920550	B02	920550	248
M925744	B02	925744	248
M936792	B02	936792	248
M945909	B02	945909	248
M948248	B02	948248	248
M960684	B02	960684	248
M102028	B02	1020283	249
M106106	B02	1061062	249
M107127	B02	1071272	250
M107401	B02	1074011	250
M108422	B02	1084227	251
M108718	B02	1087184	251
M111495	B02	1114958	252
M112198	B02	1121983	252
M114236	B02	1142364	252
M114586	B02	1145860	252
M115163	B02	1151635	252
M131631	B02	1316317	253
M132434	B02	1324348	253
M132892	B02	1328920	253
M133148	B02	1331482	254
M133972	B02	1339725	254

M134282	B02	1342826	254
M136360	B02	1363606	255
M143062	B02	1430622	255
M144588	B02	1445883	256
M145177	B02	1451777	256
M146168	B02	1461683	257
M146952	B02	1469523	257
M147807	B02	1478076	258
M148282	B02	1482824	258
M154194	B02	1541940	259
M159201	B02	1592019	259
M159247	B02	1592470	259
M169004	B02	1690041	259
M169457	B02	1694576	260
M174468	B02	1744682	260
M180106	B02	1801063	261
M180951	B02	1809516	261
M182133	B02	1821331	261
M182898	B02	1828987	261
M183744	B02	1837449	261
M184042	B02	1840421	261
M193696	B02	1936963	261
M194335	B02	1943351	261
M237705	B02	2377055	262
M238711	B02	2387115	262
M239754	B02	2397547	262
M242848	B02	2428485	263
M242973	B02	2429738	263
M243908	B02	2439083	263
M255798	B02	2557987	263
M260995	B02	2609959	264
M262626	B02	2626260	264
M272443	B02	2724435	265
M272682	B02	2726823	265
M273509	B02	2735091	265
M276146	B02	2761466	265
M352899	B02	3528992	266
M363057	B02	3630576	266
M364246	B02	3642460	266
M364761	B02	3647610	266
M368622	B02	3686228	266
M368899	B02	3688993	267
M371561	B02	3715612	267
M373167	B02	3731670	267
M373518	B02	3735183	267
M374356	B02	3743563	267
M378034	B02	3780344	267

M379309	B02	3793095	267
M380283	B02	3802837	268
M385588	B02	3855881	268
M385590	B02	3855902	268
M386159	B02	3861591	269
M387870	B02	3878700	269
M395011	B02	3950115	269
M400596	B02	4005960	269
M401261	B02	4012616	269
M402073	B02	4020736	269
M402612	B02	4026129	270
M403298	B02	4032981	270
M417268	B02	4172685	271
M431591	B02	4315918	271
M433138	B02	4331386	271
M441733	B02	4417336	272
M441852	B02	4418527	272
M442475	B02	4424758	272
M445276	B02	4452765	272
M445450	B02	4454504	272
M449971	B02	4499714	273
M450777	B02	4507776	273
M450883	B02	4508830	273
M452335	B02	4523353	273
M452827	B02	4528272	273
M453294	B02	4532942	274
M453543	B02	4535431	274
M464462	B02	4644621	275
M466302	B02	4663027	275
M544804	B02	5448049	276
M545543	B02	5455434	276
M547543	B02	5475435	276
M547782	B02	5477823	276
M554772	B02	5547725	277
M555588	B02	5555889	277
M556805	B02	5568053	278
M557980	B02	5579801	278
M560381	B02	5603816	279
M561367	B02	5613671	279
M561695	B02	5616951	279
M562286	B02	5622869	279
M653082	B02	6530828	280
M656609	B02	6566093	280
M746941	B02	7469412	281
M748897	B02	7488979	281
M751239	B02	7512393	282
M752544	B02	7525440	282

M752900	B02	7529002	282
M754872	B02	7548720	282
M756286	B02	7562860	282
M758632	B02	7586321	283
M759449	B02	7594495	283
M761973	B02	7619739	283
M762456	B02	7624568	283
M763192	B02	7631920	283
M795043	B02	7950439	284
M796169	B02	7961694	284
M800414	B02	8004147	285
M812278	B02	8122789	285
M899748	B02	8997488	286
M900553	B02	9005534	286
M900944	B02	9009442	286
M100714	B02	10071430	287
M100841	B02	10084187	287
M100988	B02	10098822	287
M101002	B02	10100216	287
M101137	B02	10113790	287
M101452	B02	10145239	288
M101471	B02	10147194	288
M167128	B02	16712831	289
M167709	B02	16770933	289
M186996	B02	18699676	290
M187021	B02	18702167	290
M194268	B02	19426842	291
M194887	B02	19488794	291
M195185	B02	19518529	291
M195382	B02	19538205	291
M200784	B02	20078425	292
M202026	B02	20202619	292
M213884	B02	21388466	293
M214608	B02	21460828	293
M218461	B02	21846102	294
M219671	B02	21967140	294
M222480	B02	22248097	295
M223995	B02	22399538	295
M225912	B02	22591257	296
M226281	B02	22628129	296
M229633	B02	22963374	297
M229767	B02	22976781	297
M236712	B02	23671249	298
M237820	B02	23782049	298
M270820	B02	27082091	299
M271631	B02	27163161	299
M276954	B02	27695402	300

M277962	B02	27796295	300
M278486	B02	27848624	300
M278727	B02	27872780	301
M278898	B02	27889878	301
M278979	B02	27897961	301
M279017	B02	27901723	301
M279107	B02	27910769	301
M279331	B02	27933193	301
M279468	B02	27946860	302
M279523	B02	27952328	302
M279829	B02	27982966	303
M279966	B02	27996600	303
M280056	B02	28005620	303
M280072	B02	28007285	303
M280130	B02	28013047	303
M280202	B02	28020213	303
M280463	B02	28046308	303
M280499	B02	28049953	303
M280654	B02	28065497	303
M280860	B02	28086027	303
M280894	B02	28089440	303
M280977	B02	28097793	303
M281138	B02	28113829	303
M281195	B02	28119501	303
M281229	B02	28122923	303
M281345	B02	28134561	303
M281778	B02	28177817	304
M281972	B02	28197291	304
M282306	B02	28230618	304
M282429	B02	28242914	305
M282511	B02	28251135	305
M282589	B02	28258953	306
M282623	B02	28262365	306
M282656	B02	28265694	306
M282724	B02	28272407	306
M282751	B02	28275196	306
M282835	B02	28283517	306
M283259	B02	28325919	307
M283382	B02	28338237	307
M283409	B02	28340986	307
M283442	B02	28344266	307
M283578	B02	28357848	307
M283661	B02	28366190	307
M284169	B02	28416991	308
M284302	B02	28430214	308
M284323	B02	28432308	308
M284448	B02	28444841	308

M284613	B02	28461317	309
M284648	B02	28464816	309
M284663	B02	28466385	309
M284964	B02	28496471	310
M285087	B02	28508780	310
M285312	B02	28531226	310
M285323	B02	28532390	310
M285339	B02	28533988	310
M285451	B02	28545134	310
M285824	B02	28582412	311
M285855	B02	28585520	311
M307183	B02	30718368	312
M307189	B02	30718912	312
M307224	B02	30722457	312
M307251	B02	30725132	312
M307301	B02	30730199	312
M307536	B02	30753677	313
M307539	B02	30753940	313
M307587	B02	30758797	313
M307832	B02	30783203	313
M307895	B02	30789564	313
M307947	B02	30794771	314
M308018	B02	30801848	314
M308096	B02	30809618	314
M308220	B02	30822051	314
M308246	B02	30824672	314
M315537	B02	31553797	315
M315618	B02	31561834	315
M318880	B02	31888028	316
M319045	B02	31904559	316
M319233	B02	31923371	317
M319382	B02	31938222	317
M319947	B02	31994708	317
M320059	B02	32005932	317
M320693	B02	32069397	317
M321109	B02	32110984	318
M321129	B02	32112908	318
M321202	B02	32120214	318
M321489	B02	32148919	318
M321505	B02	32150503	318
M322029	B02	32202932	318
M322218	B02	32221811	318
M322401	B02	32240174	318
M322443	B02	32244315	319
M322861	B02	32286167	319
M323105	B02	32310561	319
M325399	B02	32539978	320

M326117	B02	32611722	320
M328955	B02	32895502	321
M329153	B02	32915349	321
M329308	B02	32930891	321
M329395	B02	32939517	321
M329455	B02	32945549	321
M329734	B02	32973415	322
M329805	B02	32980511	322
M329841	B02	32984185	322
M330030	B02	33003011	322
M330047	B02	33004751	322
M330157	B02	33015753	323
M330196	B02	33019681	323
M330539	B02	33053990	324
M330586	B02	33058628	324
M330719	B02	33071949	324
M330818	B02	33081850	324
M330831	B02	33083101	324
M330901	B02	33090194	325
M330951	B02	33095187	325
M330987	B02	33098779	326
M331120	B02	33112018	326
M331134	B02	33113434	327
M331160	B02	33116028	327
M331394	B02	33139432	327
M331726	B02	33172672	328
M331784	B02	33178427	328
M331918	B02	33191867	328
M331942	B02	33194242	328
M332187	B02	33218797	329
M332920	B02	33292048	329
M333697	B02	33369727	329
M333750	B02	33375022	329
M333786	B02	33378654	329
M335802	B02	33580241	330
M335905	B02	33590527	330
M336180	B02	33618024	331
M336271	B02	33627153	331
M336303	B02	33630387	331
M336440	B02	33644014	331
M336475	B02	33647533	331
M336526	B02	33652641	331
M336672	B02	33667203	331
M336977	B02	33697781	331
M337156	B02	33715665	331
M338324	B02	33832487	332
M338587	B02	33858754	332

M339051	B02	33905109	332
M339835	B02	33983595	332
M347492	B02	34749255	333
M347556	B02	34755677	333
M347791	B02	34779137	333
M347830	B02	34783032	333
M347911	B02	34791150	333
M347954	B02	34795407	333
M347976	B02	34797682	333
M348095	B02	34809547	334
M348197	B02	34819756	334
M348297	B02	34829747	334
M348390	B02	34839097	334
M348404	B02	34840415	334
M348846	B02	34884628	334
M349305	B02	34930574	334
M349636	B02	34963630	334
M349664	B02	34966457	334
M349703	B02	34970384	335
M349731	B02	34973169	335
M349754	B02	34975464	335
M349833	B02	34983344	335
M349853	B02	34985303	335
M349912	B02	34991297	336
M349980	B02	34998015	336
M349989	B02	34998977	336
M350065	B02	35006514	336
M350085	B02	35008538	337
M350154	B02	35015420	337
M350337	B02	35033769	338
M350361	B02	35036138	338
M351836	B02	35183644	338
M351840	B02	35184081	338
M351895	B02	35189523	338
M352167	B02	35216714	339
M352431	B02	35243123	339
M353760	B02	35376032	339
M354028	B02	35402866	340
M354359	B02	35435984	340
M354389	B02	35438932	340
M354444	B02	35444479	340
M354506	B02	35450661	340
M355481	B02	35548190	340
M355795	B02	35579543	341
M356134	B02	35613453	341
M356394	B02	35639444	341
M356463	B02	35646336	341

M356695	B02	35669512	341
M357228	B02	35722842	341
M357277	B02	35727794	341
M357323	B02	35732372	341
M357371	B02	35737182	341
M357373	B02	35737330	341
M358076	B02	35807668	342
M358198	B02	35819855	342
M358201	B02	35820191	342
M360485	B02	36048510	343
M361072	B02	36107287	343
M362627	B02	36262742	344
M364096	B02	36409682	344
M364219	B02	36421902	344
M364397	B02	36439744	345
M364449	B02	36444967	345
M364506	B02	36450605	345
M365086	B02	36508684	345
M366742	B02	36674201	346
M366745	B02	36674530	346
M367302	B02	36730256	346
M367325	B02	36732531	346
M367346	B02	36734693	346
M367383	B02	36738371	346
M367415	B02	36741524	346
M367443	B02	36744384	346
M367460	B02	36746055	347
M367485	B02	36748556	347
M367550	B02	36755071	347
M367576	B02	36757680	348
M367606	B02	36760637	348
M372647	B02	37264761	349
M372690	B02	37269018	349
M372800	B02	37280043	350
M372862	B02	37286296	350
M372958	B02	37295822	350
M372986	B02	37298641	350
M373001	B02	37300155	350
M373004	B02	37300407	350
M373122	B02	37312224	351
M373156	B02	37315617	351
M373201	B02	37320144	351
M373272	B02	37327266	351
M373320	B02	37332091	352
M373365	B02	37336598	352
M373406	B02	37340639	352
M376449	B02	37644910	353

M376502	B02	37650208	353
M376560	B02	37656022	353
M376577	B02	37657741	353
M376590	B02	37659098	353
M376769	B02	37676974	353
M376825	B02	37682553	354
M376962	B02	37696296	354
M377322	B02	37732265	355
M377367	B02	37736721	355
M377852	B02	37785238	356
M377948	B02	37794859	356
M379071	B02	37907192	356
M380319	B02	38031980	357
M380452	B02	38045289	357
M381330	B02	38133095	358
M381359	B02	38135989	358
M381380	B02	38138065	358
M381409	B02	38140974	358
M381643	B02	38164363	358
M382817	B02	38281726	359
M382867	B02	38286730	359
M384187	B02	38418771	359
M384247	B02	38424772	359
M384600	B02	38460057	360
M384642	B02	38464247	360
M384648	B02	38464861	360
M384661	B02	38466196	360
M384690	B02	38469050	361
M384733	B02	38473304	361
M384754	B02	38475495	361
M384777	B02	38477768	362
M384797	B02	38479773	362
M384815	B02	38481552	363
M384841	B02	38484119	363
M384864	B02	38486434	363
M384926	B02	38492693	363
M384949	B02	38494908	363
M384961	B02	38496187	363
M385191	B02	38519197	363
M385214	B02	38521462	363
M385277	B02	38527738	364
M385330	B02	38533036	364
M385480	B02	38548053	365
M385594	B02	38559460	365
M385627	B02	38562787	365
M385644	B02	38564406	365
M385719	B02	38571948	366

M386890	B02	38689011	366
M386911	B02	38691130	366
M389498	B02	38949825	367
M389584	B02	38958463	367
M389619	B02	38961989	367
M389664	B02	38966440	367
M389690	B02	38969033	367
M389741	B02	38974179	367
M390000	B02	39000074	367
M390125	B02	39012506	367
M390156	B02	39015692	367
M390206	B02	39020645	367
M391049	B02	39104904	368
M391218	B02	39121873	368
M391303	B02	39130380	368
M391328	B02	39132880	369
M391909	B02	39190939	369
M395431	B02	39543120	370
M396290	B02	39629065	370
M396325	B02	39632521	370
M396392	B02	39639263	371
M396413	B02	39641354	371
M396633	B02	39663398	372
M396641	B02	39664140	372
M396667	B02	39666764	372
M396888	B02	39688822	373
M396935	B02	39693572	373
M396982	B02	39698297	373
M397033	B02	39703381	374
M397106	B02	39710622	374
M397151	B02	39715134	374
M397348	B02	39734874	375
M397642	B02	39764237	375
M397832	B02	39783251	375
M397850	B02	39785092	375
M398026	B02	39802625	376
M398065	B02	39806574	376
M398096	B02	39809638	376
M398153	B02	39815364	376
M398231	B02	39823189	376
M398286	B02	39828605	376
M398327	B02	39832741	376
M398343	B02	39834361	376
M398645	B02	39864528	376
M398971	B02	39897168	376
M398992	B02	39899260	376
M399027	B02	39902789	377

M399082	B02	39908231	377
M399099	B02	39909939	378
M399191	B02	39919193	378
M399249	B02	39924944	378
M399304	B02	39930425	379
M399338	B02	39933857	379
M399433	B02	39943319	379
M399460	B02	39946087	379
M399477	B02	39947714	379
M399677	B02	39967771	379
M399774	B02	39977474	380
M399795	B02	39979547	380
M399908	B02	39990837	381
M399937	B02	39993766	381
M399967	B02	39996778	382
M399991	B02	39999119	382
M400016	B02	40001603	382
M400080	B02	40008041	382
M400166	B02	40016628	382
M400199	B02	40019900	382
M400234	B02	40023493	382
M400249	B02	40024909	382
M400319	B02	40031997	382
M400406	B02	40040699	382
M402406	B02	40240630	383
M402432	B02	40243208	383
M402453	B02	40245333	383
M402466	B02	40246659	383
M402525	B02	40252539	384
M403487	B02	40348727	384
M403488	B02	40348883	384
M403528	B02	40352849	384
M403565	B02	40356535	384
M403705	B02	40370581	384
M403751	B02	40375129	384
M403820	B02	40382031	384
M403912	B02	40391218	385
M403960	B02	40396037	385
M404143	B02	40414301	386
M404173	B02	40417333	386
M404263	B02	40426310	386
M404626	B02	40462621	386
M404679	B02	40467952	386
M404742	B02	40474270	386
M405601	B02	40560124	386
M409788	B02	40978865	387
M409891	B02	40989113	387

M410002	B02	41000245	387
M410072	B02	41007274	388
M410296	B02	41029664	388
M410395	B02	41039501	389
M410593	B02	41059389	389
M410626	B02	41062620	389
M410688	B02	41068840	389
M410797	B02	41079702	389
M410880	B02	41088028	390
M410894	B02	41089476	390
M410962	B02	41096283	391
M411017	B02	41101743	391
M411191	B02	41119193	391
M411268	B02	41126812	391
M411358	B02	41135893	392
M411582	B02	41158268	392
M411674	B02	41167431	392
M412621	B02	41262102	392
M412881	B02	41288139	392
M412926	B02	41292616	392
M413004	B02	41300441	393
M413148	B02	41314853	393
M413290	B02	41329037	393
M413340	B02	41334003	394
M413368	B02	41336814	394
M413398	B02	41339833	395
M413457	B02	41345779	395
M413520	B02	41352022	395
M413671	B02	41367194	395
M413729	B02	41372976	395
M416680	B02	41668019	396
M417535	B02	41753581	396
M417584	B02	41758434	396
M417734	B02	41773472	396
M418032	B02	41803232	397
M418154	B02	41815449	397
M418375	B02	41837530	397
M419039	B02	41903911	398
M420121	B02	42012109	398
M420344	B02	42034434	399
M420674	B02	42067401	399
M421582	B02	42158231	399
M421656	B02	42165677	400
M421777	B02	42177752	400
M421964	B02	42196431	401
M422015	B02	42201533	401
M422285	B02	42228584	402

M422304	B02	42230478	402
M422375	B02	42237548	402
M422427	B02	42242719	402
M422489	B02	42248982	402
M422630	B02	42263076	403
M422658	B02	42265855	403
M422735	B02	42273508	403
M422841	B02	42284189	403
M422907	B02	42290787	404
M422964	B02	42296460	404
M423021	B02	42302123	404
M423117	B02	42311775	404
M423163	B02	42316330	404
M423217	B02	42321784	404
M423336	B02	42333610	404
M423390	B02	42339005	404
M425625	B02	42562558	405
M426745	B02	42674521	405
M426815	B02	42681507	405
M427632	B02	42763252	406
M427667	B02	42766782	406
M427876	B02	42787608	407
M427937	B02	42793769	407
M428170	B02	42817059	407
M428457	B02	42845762	407
M428557	B02	42855735	408
M428611	B02	42861157	408
M428664	B02	42866444	408
M428703	B02	42870370	408
M428815	B02	42881544	409
M428940	B02	42894011	409
M428973	B02	42897390	409
M429044	B02	42904405	409
M429082	B02	42908288	409
M429230	B02	42923011	410
M429304	B02	42930483	410
M429364	B02	42936421	410
M429374	B02	42937432	411
M429439	B02	42943951	411
M429498	B02	42949803	412
M430092	B02	43009217	412
M430210	B02	43021003	412
M430273	B02	43027355	412
M430813	B02	43081350	412
M433175	B02	43317599	413
M433202	B02	43320291	413
M435888	B02	43588846	414

M435911	B02	43591184	414
M436072	B02	43607298	414
M436220	B02	43622081	415
M436254	B02	43625499	415
M436421	B02	43642181	415
M436465	B02	43646589	415
M436479	B02	43647916	415
M436522	B02	43652252	415
M441752	B02	44175233	416
M441790	B02	44179012	416
M442538	B02	44253886	416
M442716	B02	44271625	416
M442737	B02	44273713	416
M443058	B02	44305813	417
M443411	B02	44341122	417
M443476	B02	44347664	417
M443653	B02	44365369	418
M443681	B02	44368138	418
M443759	B02	44375915	418
M443833	B02	44383347	419
M443886	B02	44388605	419
M443908	B02	44390803	420
M443933	B02	44393373	420
M443952	B02	44395213	420
M443977	B02	44397744	420
M444015	B02	44401517	420
M444063	B02	44406389	420
M444087	B02	44408749	420
M444123	B02	44412301	420
M444317	B02	44431720	421
M444343	B02	44434393	421
M444496	B02	44449681	422
M444518	B02	44451822	422
M444755	B02	44475587	423
M444793	B02	44479393	423
M444850	B02	44485076	423
M445027	B02	44502782	424
M445043	B02	44504372	424
M445070	B02	44507049	424
M445100	B02	44510024	424
M445186	B02	44518614	425
M445295	B02	44529589	425
M446143	B02	44614341	425
M446354	B02	44635451	425
M446417	B02	44641713	426
M446702	B02	44670285	426
M446790	B02	44679019	426

M446859	B02	44685904	426
M446964	B02	44696424	426
M447027	B02	44702782	426
M447073	B02	44707358	426
M447084	B02	44708428	426
M447175	B02	44717539	426
M447198	B02	44719826	426
M447469	B02	44746991	427
M447597	B02	44759732	427
M447641	B02	44764181	427
M448087	B02	44808775	427
M448100	B02	44810094	427
M448141	B02	44814120	427
M448178	B02	44817833	427
M448328	B02	44832847	427
M455004	B02	45500480	428
M456559	B02	45655909	428
M457188	B02	45718833	429
M457214	B02	45721449	429
M457348	B02	45734897	429
M457354	B02	45735401	430
M457884	B02	45788455	430
M460344	B02	46034416	431
M461177	B02	46117715	431
M461179	B02	46117913	431
M461582	B02	46158218	432
M462503	B02	46250349	432
M462796	B02	46279629	432
M462811	B02	46281125	432
M462848	B02	46284871	432
M462880	B02	46288027	432
M463064	B02	46306445	432
M463088	B02	46308856	432
M463120	B02	46312066	432
M463287	B02	46328735	433
M463296	B02	46329673	433
M463672	B02	46367246	433
M463769	B02	46376927	433
M463860	B02	46386003	433
M463899	B02	46389911	433
M464107	B02	46410775	434
M464151	B02	46415170	434
M464229	B02	46422912	435
M464257	B02	46425734	435
M464282	B02	46428275	435
M464370	B02	46437067	435
M464500	B02	46450075	435

M464594	B02	46459441	435
M464612	B02	46461270	435
M464743	B02	46474393	435
M464874	B02	46487453	435
M465022	B02	46502201	435
M465142	B02	46514281	436
M465173	B02	46517348	436
M465211	B02	46521174	436
M465267	B02	46526787	437
M465327	B02	46532745	437
M465413	B02	46541321	437
M465475	B02	46547522	437
M466355	B02	46635502	437
M466542	B02	46654284	437
M466562	B02	46656250	437
M466617	B02	46661734	437
M466781	B02	46678117	438
M466813	B02	46681365	438
M466921	B02	46692118	439
M466985	B02	46698502	439
M467057	B02	46705778	439
M467165	B02	46716569	439
M467754	B02	46775424	440
M468821	B02	46882109	440
M468859	B02	46885944	440
M468877	B02	46887763	440
M468913	B02	46891397	441
M468938	B02	46893878	441
M468998	B02	46899859	442
M469019	B02	46901986	442
M469049	B02	46904946	442
M469252	B02	46925287	442
M469276	B02	46927694	442
M469310	B02	46931087	442
M469372	B02	46937263	442
M469540	B02	46954002	442
M469564	B02	46956493	442
M473765	B02	47376530	443
M473778	B02	47377865	443
M473786	B02	47378632	443
M473836	B02	47383655	443
M473875	B02	47387575	443
M474050	B02	47405030	444
M474178	B02	47417801	444
M474271	B02	47427103	444
M474778	B02	47477830	445
M474806	B02	47480602	445

M474914	B02	47491450	446
M474946	B02	47494682	446
M475401	B02	47540167	447
M475481	B02	47548181	447
M475502	B02	47550222	447
M475565	B02	47556543	447
M475622	B02	47562205	447
M475715	B02	47571598	447
M475830	B02	47583001	447
M476055	B02	47605500	448
M476092	B02	47609200	448
M476131	B02	47613129	448
M476206	B02	47620627	448
M476314	B02	47631436	449
M476374	B02	47637470	449
M477146	B02	47714663	450
M477276	B02	47727615	450
M477386	B02	47738651	451
M477459	B02	47745931	451
M477689	B02	47768998	452
M477718	B02	47771864	452
M477748	B02	47774859	453
M477845	B02	47784503	453
M477873	B02	47787301	453
M478027	B02	47802706	453
M478146	B02	47814616	453
M478651	B02	47865129	453
M478721	B02	47872121	453
M478772	B02	47877283	453
M478818	B02	47881885	453
M482969	B02	48296979	454
M483025	B02	48302564	454
M483102	B02	48310214	454
M483159	B02	48315967	454
M483356	B02	48335613	455
M483411	B02	48341193	455
M483443	B02	48344371	455
M483599	B02	48359945	455
M483635	B02	48363568	455
M483788	B02	48378806	455
M483803	B02	48380313	455
M483976	B02	48397621	455
M484010	B02	48401096	455
M484040	B02	48404011	455
M484068	B02	48406803	455
M484101	B02	48410136	455
M484150	B02	48415009	455

M484280	B02	48428052	456
M484409	B02	48440915	456
M484421	B02	48442113	457
M484581	B02	48458180	457
M484625	B02	48462534	457
M484646	B02	48464678	457
M484687	B02	48468753	457
M484716	B02	48471673	457
M484786	B02	48478632	457
M484930	B02	48493034	457
M484959	B02	48495903	457
M484974	B02	48497488	457
M485025	B02	48502560	457
M485056	B02	48505620	457
M485093	B02	48509347	457
M485172	B02	48517212	457
M485212	B02	48521261	457
M485273	B02	48527303	457
M485331	B02	48533123	457
M485568	B02	48556820	457
M485704	B02	48570484	458
M485714	B02	48571482	458
M485752	B02	48575202	458
M485813	B02	48581326	458
M485868	B02	48586812	458
M485987	B02	48598768	458
M486038	B02	48603882	458
M486163	B02	48616368	458
M486232	B02	48623257	459
M486276	B02	48627630	459
M486305	B02	48630596	459
M486443	B02	48644326	459
M489277	B02	48927792	460
M489790	B02	48979050	460
M490655	B02	49065531	460
M490691	B02	49069126	460
M490742	B02	49074279	460
M490828	B02	49082885	461
M490936	B02	49093668	461
M491152	B02	49115290	461
M491168	B02	49116863	461
M491192	B02	49119293	461
M491216	B02	49121640	461
M491297	B02	49129741	461
M493356	B02	49335673	462
M493385	B02	49338567	462
M493401	B02	49340182	462

M493778	B02	49377832	463
M493782	B02	49378288	463
M493862	B02	49386217	463
M493899	B02	49389944	463
M493937	B02	49393748	463
M494211	B02	49421167	464
M494273	B02	49427300	464
M494307	B02	49430713	465
M494352	B02	49435213	465
M494450	B02	49445037	465
M494479	B02	49447990	465
M494484	B02	49448410	465
M494945	B02	49494533	466
M494990	B02	49499077	466
M495995	B02	49599594	466
M496527	B02	49652769	466
M496838	B02	49683876	467
M496874	B02	49687416	467
M496920	B02	49692022	468
M496944	B02	49694453	468
M496989	B02	49698927	469
M497144	B02	49714490	469
M497197	B02	49719710	469
M497286	B02	49728644	469
M497350	B02	49735044	469
M497475	B02	49747575	469
M497495	B02	49749590	469
M497515	B02	49751569	469
M497543	B02	49754377	469
M497637	B02	49763708	469
M497672	B02	49767258	469
M497756	B02	49775628	469
M497783	B02	49778379	469
M497813	B02	49781380	469
M497884	B02	49788428	470
M497933	B02	49793341	470
M498077	B02	49807771	471
M498314	B02	49831409	471
M503484	B02	50348460	472
M503564	B02	50356482	472
M504080	B02	50408005	472
M504169	B02	50416901	473
M504197	B02	50419770	473
M504277	B02	50427751	474
M504315	B02	50431532	474
M504351	B02	50435153	475
M504385	B02	50438514	475

M504445	B02	50444534	475
M504557	B02	50455785	475
M504620	B02	50462004	475
M504657	B02	50465786	475
M504686	B02	50468683	475
M504693	B02	50469378	475
M504706	B02	50470691	475
M505242	B02	50524287	475
M505252	B02	50525290	475
M505290	B02	50529021	475
M505543	B02	50554395	476
M505609	B02	50560963	476
M505655	B02	50565510	476
M505763	B02	50576364	476
M505927	B02	50592787	476
M506032	B02	50603248	476
M506121	B02	50612156	476
M506208	B02	50620862	476
M506240	B02	50624084	476
M506343	B02	50634367	477
M506774	B02	50677446	477
M506790	B02	50679082	477
M506866	B02	50686689	477
M507114	B02	50711422	478
M508485	B02	50848560	478
M508879	B02	50887956	479
M509624	B02	50962442	479
M509673	B02	50967375	479
M509731	B02	50973170	479
M509821	B02	50982125	479
M511710	B02	51171070	480
M513239	B02	51323912	480
M513408	B02	51340881	481
M513434	B02	51343422	481
M514304	B02	51430496	482
M514346	B02	51434650	482
M514361	B02	51436184	482
M514437	B02	51443768	482
M515286	B02	51528644	482
M515410	B02	51541075	482
M515438	B02	51543814	482
M515473	B02	51547337	482
M515719	B02	51571943	483
M515751	B02	51575137	483
M515772	B02	51577276	483
M515797	B02	51579739	484
M516577	B02	51657744	484

M516840	B02	51684015	485
M516953	B02	51695380	485
M516998	B02	51699817	485
M517104	B02	51710426	485
M517169	B02	51716949	486
M517184	B02	51718401	486
M517243	B02	51724391	486
M517329	B02	51732977	486
M517566	B02	51756636	487
M517691	B02	51769113	487
M517842	B02	51784255	488
M517933	B02	51793304	488
M517958	B02	51795803	488
M517985	B02	51798542	488
M517996	B02	51799699	488
M518028	B02	51802836	488
M518083	B02	51808377	488
M518168	B02	51816835	489
M518198	B02	51819897	489
M518215	B02	51821557	489
M518240	B02	51824025	489
M518282	B02	51828203	489
M518303	B02	51830396	489
M518381	B02	51838152	490
M518445	B02	51844556	490
M518536	B02	51853635	490
M518634	B02	51863469	491
M518638	B02	51863852	491
M518729	B02	51872971	491
M518809	B02	51880910	491
M521872	B02	52187273	492
M521941	B02	52194153	492
M527123	B02	52712349	493
M527160	B02	52716059	493
M527197	B02	52719774	493
M527224	B02	52722446	494
M527333	B02	52733342	494
M527480	B02	52748025	494
M527607	B02	52760767	495
M527674	B02	52767405	495
M527683	B02	52768308	495
M527724	B02	52772420	496
M527743	B02	52774316	496
M527798	B02	52779833	497
M527836	B02	52783675	497
M527916	B02	52791665	498
M527974	B02	52797454	498

M528008	B02	52800855	498
M528127	B02	52812734	498
M528172	B02	52817291	498
M528258	B02	52825831	498
M528689	B02	52868901	498
M528707	B02	52870797	498
M528737	B02	52873765	498
M528793	B02	52879380	498
M529089	B02	52908937	498
M530741	B02	53074118	499
M531652	B02	53165263	499
M531682	B02	53168240	500
M531737	B02	53173708	500
M531778	B02	53177866	500
M531824	B02	53182491	500
M531857	B02	53185757	500
M532253	B02	53225307	501
M533783	B02	53378306	501
M533868	B02	53386833	502
M533974	B02	53397435	502
M534181	B02	53418114	502
M534211	B02	53421100	502
M534292	B02	53429223	502
M534331	B02	53433106	503
M534352	B02	53435253	503
M534388	B02	53438813	503
M534414	B02	53441475	503
M534451	B02	53445141	503
M534469	B02	53446972	503
M535215	B02	53521501	503
M535270	B02	53527079	503
M535281	B02	53528124	504
M535295	B02	53529537	504
M535309	B02	53530975	504
M535337	B02	53533715	504
M537019	B02	53701981	505
M537166	B02	53716651	505
M537231	B02	53723122	505
M537660	B02	53766033	505
M537696	B02	53769699	505
M537716	B02	53771612	505
M538030	B02	53803009	506
M538048	B02	53804844	506
M538095	B02	53809543	506
M538136	B02	53813658	506
M541951	B02	54195117	507
M541987	B02	54198718	507

M542026	B02	54202691	507
M542111	B02	54211104	508
M542305	B02	54230555	508
M545446	B02	54544680	509
M545495	B02	54549560	509
M545537	B02	54553711	509
M545570	B02	54557050	509
M545698	B02	54569844	509
M545717	B02	54571709	509
M546123	B02	54612389	510
M546474	B02	54647448	510
M546516	B02	54651645	511
M546590	B02	54659008	511
M547916	B02	54791691	512
M548052	B02	54805243	512
M548081	B02	54808113	512
M548431	B02	54843116	512
M548460	B02	54846056	513
M548537	B02	54853759	513
M548622	B02	54862298	514
M548672	B02	54867200	514
M548765	B02	54876551	514
M548782	B02	54878280	514
M548822	B02	54882266	514
M548841	B02	54884157	514
M548926	B02	54892634	515
M549005	B02	54900547	515
M549012	B02	54901299	515
M549081	B02	54908112	515
M549120	B02	54912009	515
M549332	B02	54933260	516
M549404	B02	54940408	516
M549599	B02	54959931	516
M549624	B02	54962426	516
M549655	B02	54965546	516
M549757	B02	54975778	516
M549813	B02	54981329	516
M549923	B02	54992304	516
M549981	B02	54998172	516
M550019	B02	55001905	516
M550102	B02	55010280	516
M550161	B02	55016157	516
M550195	B02	55019569	516
M550235	B02	55023550	516
M550386	B02	55038629	516
M550416	B02	55041601	516
M550520	B02	55052044	516

M550572	B02	55057212	516
M550605	B02	55060593	516
M550629	B02	55062983	516
M550774	B02	55077479	516
M550812	B02	55081270	516
M550848	B02	55084898	516
M550914	B02	55091493	516
M550926	B02	55092611	516
M550983	B02	55098383	517
M551088	B02	55108858	517
M551163	B02	55116373	518
M551218	B02	55121843	518
M551301	B02	55130161	519
M551349	B02	55134924	519
M551361	B02	55136101	519
M551427	B02	55142771	519
M551452	B02	55145248	519
M551516	B02	55151665	519
M551528	B02	55152884	519
M551565	B02	55156581	519
M551609	B02	55160991	519
M551637	B02	55163799	519
M551664	B02	55166443	519
M551695	B02	55169546	519
M551755	B02	55175588	519
M551772	B02	55177216	519
M551789	B02	55178931	519
M551827	B02	55182775	519
M551931	B02	55193123	519
M552041	B02	55204156	520
M552783	B02	55278332	520
M553318	B02	55331817	520
M553591	B02	55359190	520
M553630	B02	55363027	520
M553657	B02	55365765	521
M553699	B02	55369936	521
M553821	B02	55382199	521
M553879	B02	55387914	521
M554052	B02	55405210	521
M554111	B02	55411195	521
M555778	B02	55577827	522
M555810	B02	55581020	522
M555900	B02	55590030	522
M555949	B02	55594970	522
M555985	B02	55598531	522
M556403	B02	55640303	522
M556516	B02	55651646	522

M556526	B02	55652672	522
M556598	B02	55659870	522
M556628	B02	55662862	522
M556666	B02	55666606	522
M556718	B02	55671857	522
M556788	B02	55678854	522
M557270	B02	55727004	522
M557348	B02	55734816	522
M557617	B02	55761741	523
M557648	B02	55764873	523
M557897	B02	55789796	523
M557903	B02	55790323	523
M558011	B02	55801163	523
M558032	B02	55803239	523
M558061	B02	55806175	523
M558273	B02	55827310	523
M558333	B02	55833399	523
M558401	B02	55840161	523
M558491	B02	55849137	523
M558564	B02	55856427	523
M559146	B02	55914692	523
M559174	B02	55917434	523
M559192	B02	55919263	523
M559205	B02	55920578	523
M559636	B02	55963616	524
M559710	B02	55971003	524
M559768	B02	55976847	524
M559808	B02	55980854	524
M559848	B02	55984812	524
M559969	B02	55996924	524
M559988	B02	55998879	524
M560022	B02	56002220	524
M560051	B02	56005133	524
M560098	B02	56009850	524
M560143	B02	56014346	524
M560229	B02	56022977	524
M560232	B02	56023259	524
M560709	B02	56070965	525
M560731	B02	56073117	525
M560749	B02	56074983	525
M560820	B02	56082056	525
M560979	B02	56097990	525
M560996	B02	56099602	525
M561039	B02	56103920	525
M561163	B02	56116344	525
M561368	B02	56136836	525
M561389	B02	56138902	525

M561485	B02	56148528	525
M563361	B02	56336123	526
M563382	B02	56338248	526
M563479	B02	56347910	526
M563623	B02	56362382	526
M563882	B02	56388290	526
M564823	B02	56482390	526
M564865	B02	56486526	526
M565129	B02	56512940	527
M565179	B02	56517919	527
M565201	B02	56520116	527
M565691	B02	56569112	527
M565720	B02	56572096	527
M565768	B02	56576814	527
M566152	B02	56615235	527
M566713	B02	56671301	527
M566908	B02	56690885	528
M567094	B02	56709498	528
M397981	B03	397981	529
M403153	B03	403153	529
M406930	B03	406930	530
M534039	B03	534039	530
M535783	B03	535783	530
M538768	B03	538768	530
M552186	B03	552186	531
M553668	B03	553668	531
M561798	B03	561798	531
M572986	B03	572986	532
M574059	B03	574059	532
M575190	B03	575190	532
M578392	B03	578392	532
M588163	B03	588163	533
M590233	B03	590233	533
M592207	B03	592207	533
M601786	B03	601786	534
M609482	B03	609482	534
M691263	B03	691263	535
M704344	B03	704344	535
M706183	B03	706183	535
M712102	B03	712102	535
M721650	B03	721650	535
M723530	B03	723530	535
M728978	B03	728978	535
M747473	B03	747473	535
M812826	B03	812826	536
M817940	B03	817940	536
M821825	B03	821825	536

M841059	B03	841059	537
M841882	B03	841882	537
M847953	B03	847953	538
M853513	B03	853513	538
M893990	B03	893990	539
M897284	B03	897284	539
M899802	B03	899802	539
M903291	B03	903291	539
M914773	B03	914773	539
M915900	B03	915900	540
M919670	B03	919670	540
M922459	B03	922459	541
M100026	B03	1000269	541
M100357	B03	1003573	541
M100851	B03	1008519	541
M101299	B03	1012993	541
M102249	B03	1022493	541
M103244	B03	1032441	541
M103640	B03	1036408	541
M104834	B03	1048340	541
M105537	B03	1055370	541
M110240	B03	1102409	542
M110549	B03	1105491	542
M111122	B03	1111223	542
M111996	B03	1119961	542
M112764	B03	1127648	542
M115695	B03	1156952	543
M118350	B03	1183506	543
M118641	B03	1186413	543
M119595	B03	1195950	543
M119671	B03	1196710	544
M119915	B03	1199151	544
M120185	B03	1201855	545
M120411	B03	1204113	545
M122877	B03	1228775	545
M123236	B03	1232362	545
M123442	B03	1234428	545
M125533	B03	1255330	546
M126772	B03	1267729	546
M126886	B03	1268863	546
M127402	B03	1274023	546
M127715	B03	1277151	546
M128429	B03	1284296	546
M129140	B03	1291400	547
M129385	B03	1293855	547
M131909	B03	1319095	547
M132560	B03	1325604	548

M133092	B03	1330921	548
M133758	B03	1337580	549
M134811	B03	1348119	549
M138081	B03	1380812	550
M138488	B03	1384889	550
M139140	B03	1391408	550
M139544	B03	1395447	550
M140286	B03	1402869	551
M140635	B03	1406357	551
M146920	B03	1469208	552
M147827	B03	1478270	552
M157408	B03	1574084	553
M157530	B03	1575300	553
M158425	B03	1584251	553
M159223	B03	1592238	554
M163686	B03	1636864	554
M163932	B03	1639320	555
M164171	B03	1641715	555
M164552	B03	1645525	556
M165106	B03	1651064	556
M165208	B03	1652086	556
M181548	B03	1815484	557
M182001	B03	1820016	557
M182231	B03	1822313	557
M182514	B03	1825147	557
M182878	B03	1828787	557
M183067	B03	1830670	558
M183497	B03	1834978	558
M183651	B03	1836519	559
M184291	B03	1842916	559
M191173	B03	1911735	559
M191215	B03	1912158	559
M191454	B03	1914549	559
M191818	B03	1918185	559
M192822	B03	1928222	559
M194207	B03	1942074	559
M194401	B03	1944015	559
M194610	B03	1946100	559
M195014	B03	1950140	559
M195794	B03	1957942	559
M196347	B03	1963475	559
M196958	B03	1969581	559
M197381	B03	1973810	559
M198007	B03	1980070	559
M198327	B03	1983279	559
M198842	B03	1988426	559
M199249	B03	1992493	559

M200553	B03	2005534	560
M200967	B03	2009672	560
M201431	B03	2014310	560
M203905	B03	2039054	560
M204086	B03	2040864	560
M204499	B03	2044994	560
M204965	B03	2049652	560
M205565	B03	2055650	561
M205941	B03	2059412	561
M208140	B03	2081402	561
M235298	B03	2352984	562
M240315	B03	2403157	562
M240805	B03	2408058	562
M241024	B03	2410241	562
M241932	B03	2419326	563
M252398	B03	2523980	563
M257229	B03	2572296	563
M257547	B03	2575475	564
M257901	B03	2579010	564
M258426	B03	2584267	564
M261592	B03	2615928	565
M262036	B03	2620362	565
M263979	B03	2639798	565
M271005	B03	2710059	566
M271739	B03	2717393	566
M284223	B03	2842230	567
M284581	B03	2845816	567
M284865	B03	2848655	568
M285473	B03	2854738	568
M287073	B03	2870739	568
M287353	B03	2873535	568
M287431	B03	2874319	568
M287913	B03	2879133	568
M288718	B03	2887187	568
M288943	B03	2889431	568
M289012	B03	2890122	568
M289393	B03	2893934	568
M289873	B03	2898737	568
M290386	B03	2903861	568
M290825	B03	2908257	568
M291367	B03	2913679	568
M291677	B03	2916777	569
M292077	B03	2920779	569
M292268	B03	2922689	570
M292840	B03	2928400	570
M300429	B03	3004299	571
M301381	B03	3013819	571

M301397	B03	3013972	572
M301793	B03	3017930	572
M302128	B03	3021281	572
M304224	B03	3042242	572
M309679	B03	3096790	573
M310125	B03	3101250	573
M313088	B03	3130880	573
M321786	B03	3217865	573
M324466	B03	3244665	573
M325441	B03	3254418	573
M367229	B03	3672299	574
M367647	B03	3676477	574
M387163	B03	3871630	575
M387592	B03	3875921	575
M387872	B03	3878721	575
M393758	B03	3937587	575
M394084	B03	3940842	576
M394387	B03	3943875	576
M394860	B03	3948605	577
M395222	B03	3952221	577
M396554	B03	3965544	577
M416799	B03	4167994	578
M418047	B03	4180474	578
M418314	B03	4183142	578
M418635	B03	4186357	578
M419000	B03	4190006	578
M419615	B03	4196158	578
M421477	B03	4214778	579
M422876	B03	4228762	579
M423186	B03	4231861	580
M423813	B03	4238133	580
M434301	B03	4343019	580
M434709	B03	4347095	580
M435887	B03	4358879	580
M436041	B03	4360415	580
M436581	B03	4365816	580
M436975	B03	4369756	580
M437940	B03	4379407	580
M438788	B03	4387881	581
M440975	B03	4409752	581
M441202	B03	4412026	581
M441935	B03	4419352	581
M443210	B03	4432107	581
M443440	B03	4434405	581
M454078	B03	4540787	582
M455691	B03	4556914	582
M456255	B03	4562557	582

M456266	B03	4562668	582
M456483	B03	4564834	582
M457739	B03	4577393	582
M458655	B03	4586552	582
M459256	B03	4592569	582
M459530	B03	4595303	582
M459712	B03	4597123	582
M460694	B03	4606945	582
M461073	B03	4610735	583
M461332	B03	4613326	583
M461650	B03	4616502	583
M465015	B03	4650150	583
M465541	B03	4655410	583
M467385	B03	4673850	583
M469488	B03	4694889	583
M472942	B03	4729427	584
M473923	B03	4739230	584
M474670	B03	4746700	584
M475113	B03	4751133	584
M492421	B03	4924210	585
M494409	B03	4944097	585
M495114	B03	4951140	585
M495575	B03	4955755	585
M495742	B03	4957422	585
M496183	B03	4961832	585
M496490	B03	4964906	585
M497347	B03	4973471	585
M497546	B03	4975464	585
M497933	B03	4979330	585
M498365	B03	4983656	585
M498855	B03	4988550	585
M500132	B03	5001323	586
M500716	B03	5007166	586
M500882	B03	5008825	586
M502512	B03	5025127	586
M503780	B03	5037808	586
M503988	B03	5039883	586
M507815	B03	5078158	586
M507899	B03	5078999	586
M507980	B03	5079804	586
M508170	B03	5081705	586
M508386	B03	5083864	586
M508626	B03	5086260	586
M509661	B03	5096614	586
M509907	B03	5099078	586
M510139	B03	5101396	586
M511684	B03	5116848	586

M511905	B03	5119058	586
M512470	B03	5124702	586
M512971	B03	5129718	587
M513355	B03	5133552	587
M513815	B03	5138159	587
M514295	B03	5142956	587
M515408	B03	5154088	587
M516074	B03	5160744	587
M516625	B03	5166251	587
M516751	B03	5167517	587
M516866	B03	5168669	587
M517084	B03	5170848	587
M517521	B03	5175217	587
M518645	B03	5186454	587
M519375	B03	5193758	588
M519753	B03	5197532	588
M520081	B03	5200814	588
M520339	B03	5203399	588
M521788	B03	5217883	588
M522673	B03	5226735	588
M528679	B03	5286798	588
M528865	B03	5288658	588
M530107	B03	5301077	588
M530734	B03	5307345	588
M531376	B03	5313769	588
M532287	B03	5322878	588
M533109	B03	5331096	588
M533688	B03	5336880	588
M534165	B03	5341656	588
M535076	B03	5350760	588
M535370	B03	5353703	588
M535997	B03	5359978	589
M536982	B03	5369822	589
M543561	B03	5435619	589
M543578	B03	5435786	589
M547200	B03	5472002	589
M547359	B03	5473596	589
M547689	B03	5476895	589
M650307	B03	6503076	590
M650629	B03	6506295	590
M651255	B03	6512553	590
M651940	B03	6519408	591
M660614	B03	6606147	591
M661585	B03	6615856	591
M661715	B03	6617155	591
M662048	B03	6620483	591
M662233	B03	6622333	591

M662972	B03	6629721	591
M663165	B03	6631654	591
M664154	B03	6641541	591
M664524	B03	6645245	591
M664902	B03	6649028	591
M665223	B03	6652235	591
M665619	B03	6656193	591
M666106	B03	6661063	591
M666368	B03	6663680	591
M667924	B03	6679240	591
M669501	B03	6695012	592
M670109	B03	6701096	592
M670801	B03	6708011	592
M675613	B03	6756131	592
M676657	B03	6766576	592
M677114	B03	6771148	592
M679469	B03	6794694	592
M679691	B03	6796913	593
M680244	B03	6802442	593
M682980	B03	6829800	593
M683428	B03	6834283	593
M685114	B03	6851144	593
M687664	B03	6876646	593
M687934	B03	6879345	593
M690467	B03	6904675	593
M691881	B03	6918816	593
M694885	B03	6948855	593
M695055	B03	6950550	593
M695502	B03	6955023	593
M695813	B03	6958133	594
M696540	B03	6965407	594
M696892	B03	6968929	594
M697319	B03	6973197	594
M697527	B03	6975276	594
M699589	B03	6995896	594
M702202	B03	7022021	594
M703967	B03	7039675	594
M704051	B03	7040513	594
M704630	B03	7046306	594
M705601	B03	7056017	594
M705961	B03	7059614	594
M706150	B03	7061508	594
M706559	B03	7065593	594
M707111	B03	7071114	594
M707639	B03	7076395	594
M713398	B03	7133986	595
M714632	B03	7146325	595

M728801	B03	7288012	595
M728921	B03	7289219	595
M729354	B03	7293546	595
M732268	B03	7322689	596
M733323	B03	7333237	596
M733582	B03	7335828	596
M809044	B03	8090442	597
M809321	B03	8093211	597
M839347	B03	8393475	598
M843374	B03	8433740	598
M871766	B03	8717667	599
M872623	B03	8726232	599
M873227	B03	8732271	599
M873859	B03	8738599	599
M874293	B03	8742930	599
M875364	B03	8753649	599
M876873	B03	8768738	599
M878910	B03	8789103	599
M884667	B03	8846677	599
M884832	B03	8848327	599
M885749	B03	8857493	599
M886615	B03	8866156	599
M890362	B03	8903629	600
M891765	B03	8917650	600
M892530	B03	8925305	600
M899048	B03	8990484	600
M899515	B03	8995152	600
M901820	B03	9018206	600
M902986	B03	9029863	600
M903479	B03	9034795	600
M904708	B03	9047088	600
M905037	B03	9050373	600
M905278	B03	9052783	600
M101069	B03	10106990	601
M101148	B03	10114801	601
M101174	B03	10117434	601
M101294	B03	10129490	602
M101863	B03	10186344	602
M101873	B03	10187359	602
M101937	B03	10193797	603
M101962	B03	10196209	603
M102043	B03	10204301	603
M102056	B03	10205637	603
M102139	B03	10213941	603
M102515	B03	10251597	604
M102601	B03	10260131	604
M102750	B03	10275027	604

M102858	B03	10285867	604
M102930	B03	10293081	604
M102987	B03	10298727	604
M103056	B03	10305659	604
M103123	B03	10312395	604
M104027	B03	10402781	604
M104076	B03	10407678	604
M104123	B03	10412345	605
M105257	B03	10525710	605
M105463	B03	10546315	606
M105870	B03	10587076	606
M106333	B03	10633331	606
M106359	B03	10635952	606
M106481	B03	10648112	607
M106529	B03	10652922	607
M106568	B03	10656822	607
M106663	B03	10666379	607
M106688	B03	10668806	607
M107352	B03	10735258	608
M108143	B03	10814303	608
M108308	B03	10830830	608
M108855	B03	10885507	609
M108865	B03	10886506	609
M113002	B03	11300293	610
M113125	B03	11312539	610
M113325	B03	11332541	610
M113390	B03	11339048	611
M113665	B03	11366521	611
M113684	B03	11368418	611
M113940	B03	11394022	612
M114133	B03	11413354	612
M114411	B03	11441141	612
M114620	B03	11462064	613
M114653	B03	11465377	613
M114788	B03	11478899	614
M114882	B03	11488247	614
M118383	B03	11838384	615
M118419	B03	11841936	615
M118466	B03	11846606	615
M118501	B03	11850103	615
M118539	B03	11853985	615
M118621	B03	11862186	615
M118730	B03	11873035	615
M119078	B03	11907888	616
M119092	B03	11909298	616
M119130	B03	11913076	616
M119194	B03	11919486	616

M119252	B03	11925243	616
M119306	B03	11930636	616
M119324	B03	11932462	616
M119344	B03	11934421	616
M119365	B03	11936551	616
M119372	B03	11937298	616
M119428	B03	11942800	616
M119509	B03	11950983	616
M119812	B03	11981208	616
M120676	B03	12067648	616
M120709	B03	12070979	617
M120799	B03	12079902	617
M120829	B03	12082919	617
M120848	B03	12084840	617
M120934	B03	12093441	618
M120998	B03	12099806	618
M121139	B03	12113971	618
M121216	B03	12121671	619
M121261	B03	12126156	619
M121274	B03	12127423	619
M121299	B03	12129995	620
M121414	B03	12141463	620
M121987	B03	12198707	620
M122002	B03	12200264	621
M122045	B03	12204593	621
M122291	B03	12229180	621
M122482	B03	12248205	622
M122659	B03	12265940	622
M122836	B03	12283623	622
M122844	B03	12284470	622
M126731	B03	12673150	623
M126889	B03	12688959	623
M126911	B03	12691116	623
M126948	B03	12694874	623
M127004	B03	12700488	623
M127044	B03	12704433	623
M127082	B03	12708276	623
M127121	B03	12712166	623
M127183	B03	12718380	623
M127260	B03	12726071	623
M127316	B03	12731643	623
M127396	B03	12739641	623
M127424	B03	12742428	623
M127445	B03	12744566	623
M127480	B03	12748030	623
M127548	B03	12754836	623
M127669	B03	12766997	624

M127709	B03	12770932	624
M127773	B03	12777393	624
M127831	B03	12783121	624
M127848	B03	12784832	624
M127870	B03	12787094	624
M127891	B03	12789156	624
M128013	B03	12801362	624
M128158	B03	12815824	624
M128196	B03	12819687	624
M128318	B03	12831830	624
M128347	B03	12834786	625
M128772	B03	12877285	625
M128918	B03	12891879	625
M128958	B03	12895809	625
M128972	B03	12897278	625
M129014	B03	12901409	625
M129042	B03	12904202	625
M129232	B03	12923236	625
M129317	B03	12931721	625
M129391	B03	12939134	625
M129419	B03	12941933	625
M129508	B03	12950879	625
M129563	B03	12956315	625
M129572	B03	12957238	625
M129598	B03	12959887	625
M129638	B03	12963886	625
M129675	B03	12967531	625
M129713	B03	12971320	625
M129769	B03	12976941	625
M129810	B03	12981020	625
M129868	B03	12986860	625
M129936	B03	12993638	625
M129957	B03	12995754	626
M130013	B03	13001329	626
M130330	B03	13033074	626
M130780	B03	13078010	627
M130993	B03	13099310	627
M131030	B03	13103016	627
M131115	B03	13111526	627
M131211	B03	13121127	627
M131430	B03	13143013	627
M131646	B03	13164610	627
M131861	B03	13186135	627
M132045	B03	13204598	627
M132312	B03	13231258	627
M132559	B03	13255932	628
M132602	B03	13260224	628

M132666	B03	13266608	629
M132833	B03	13283345	629
M132865	B03	13286544	629
M132909	B03	13290991	629
M132928	B03	13292890	629
M132957	B03	13295703	629
M132987	B03	13298701	629
M133122	B03	13312275	629
M133153	B03	13315343	629
M133209	B03	13320999	629
M133334	B03	13333415	629
M133472	B03	13347289	629
M133570	B03	13357058	629
M133627	B03	13362781	629
M133659	B03	13365913	629
M133832	B03	13383287	629
M134206	B03	13420677	629
M134254	B03	13425450	629
M134317	B03	13431735	630
M134360	B03	13436085	630
M134400	B03	13440038	630
M134515	B03	13451515	630
M136573	B03	13657337	631
M137762	B03	13776234	631
M137785	B03	13778538	631
M137835	B03	13783553	631
M137854	B03	13785450	631
M137898	B03	13789836	631
M137999	B03	13799922	631
M142241	B03	14224110	632
M142272	B03	14227260	632
M142368	B03	14236877	632
M142446	B03	14244658	632
M142459	B03	14245973	632
M142592	B03	14259213	632
M142719	B03	14271915	632
M142865	B03	14286599	632
M142924	B03	14292481	632
M143066	B03	14306655	633
M143160	B03	14316056	633
M144655	B03	14465533	633
M144703	B03	14470374	634
M144718	B03	14471853	634
M144854	B03	14485422	634
M144977	B03	14497712	634
M145054	B03	14505429	634
M145121	B03	14512114	634

M145160	B03	14516097	634
M145209	B03	14520966	634
M145228	B03	14522835	634
M145389	B03	14538906	634
M148174	B03	14817476	635
M148197	B03	14819764	635
M148260	B03	14826078	636
M148374	B03	14837470	636
M148392	B03	14839210	636
M148469	B03	14846945	636
M148507	B03	14850750	636
M149355	B03	14935532	636
M149599	B03	14959991	636
M149723	B03	14972346	636
M149754	B03	14975447	636
M149852	B03	14985280	636
M152280	B03	15228080	637
M152852	B03	15285234	637
M153074	B03	15307483	637
M153143	B03	15314381	638
M153173	B03	15317327	638
M153239	B03	15323998	638
M153308	B03	15330869	638
M153396	B03	15339693	638
M153559	B03	15355915	638
M153660	B03	15366060	638
M153927	B03	15392776	638
M153980	B03	15398024	638
M153992	B03	15399228	639
M154022	B03	15402267	639
M154080	B03	15408010	640
M154091	B03	15409162	640
M154120	B03	15412007	640
M159003	B03	15900320	641
M160000	B03	16000027	641
M162143	B03	16214338	642
M162157	B03	16215748	642
M162205	B03	16220539	642
M162224	B03	16222471	642
M162344	B03	16234428	642
M164142	B03	16414275	643
M164328	B03	16432882	643
M164549	B03	16454960	643
M164713	B03	16471322	643
M164756	B03	16475648	643
M164772	B03	16477203	643
M164967	B03	16496720	643

M164992	B03	16499238	643
M170980	B03	17098083	644
M171013	B03	17101391	644
M171081	B03	17108189	644
M171088	B03	17108843	644
M171152	B03	17115292	644
M171201	B03	17120106	644
M171486	B03	17148676	644
M171522	B03	17152294	644
M171824	B03	17182489	644
M172099	B03	17209917	645
M172363	B03	17236397	645
M172400	B03	17240057	646
M172424	B03	17242429	646
M172556	B03	17255681	646
M172665	B03	17266552	646
M172692	B03	17269298	646
M172909	B03	17290974	646
M172980	B03	17298081	646
M173126	B03	17312651	646
M173142	B03	17314232	646
M173461	B03	17346128	647
M173475	B03	17347550	647
M173519	B03	17351903	647
M177630	B03	17763064	648
M177824	B03	17782494	648
M178216	B03	17821620	648
M178558	B03	17855855	649
M178570	B03	17857068	649
M178628	B03	17862841	649
M178660	B03	17866030	649
M178813	B03	17881332	650
M178873	B03	17887328	650
M183459	B03	18345958	651
M184932	B03	18493242	651
M186740	B03	18674047	652
M186778	B03	18677861	652
M186825	B03	18682507	652
M191209	B03	19120941	653
M191272	B03	19127216	653
M191485	B03	19148512	654
M191531	B03	19153138	654
M191748	B03	19174831	655
M191852	B03	19185281	655
M192068	B03	19206884	655
M192095	B03	19209567	655
M192099	B03	19209938	655

M192114	B03	19211422	655
M192186	B03	19218603	655
M192249	B03	19224916	655
M192337	B03	19233762	655
M192384	B03	19238414	655
M192420	B03	19242046	655
M192440	B03	19244053	655
M192567	B03	19256727	655
M192704	B03	19270459	655
M192807	B03	19280782	655
M192919	B03	19291952	656
M193095	B03	19309541	656
M193119	B03	19311964	656
M193131	B03	19313160	656
M193269	B03	19326993	657
M193532	B03	19353238	657
M193563	B03	19356307	657
M193582	B03	19358264	657
M193706	B03	19370606	658
M193728	B03	19372876	658
M193983	B03	19398366	659
M194064	B03	19406433	659
M194094	B03	19409401	659
M194185	B03	19418545	659
M194356	B03	19435613	659
M194579	B03	19457986	659
M194716	B03	19471682	660
M194741	B03	19474103	660
M194838	B03	19483864	660
M194877	B03	19487777	660
M194993	B03	19499364	660
M195097	B03	19509788	660
M195112	B03	19511207	660
M195136	B03	19513654	660
M195186	B03	19518688	661
M195199	B03	19519942	661
M195348	B03	19534862	661
M195714	B03	19571416	662
M195761	B03	19576177	662
M195844	B03	19584457	662
M195921	B03	19592196	662
M196648	B03	19664852	663
M196685	B03	19668583	663
M196741	B03	19674183	663
M196902	B03	19690203	663
M207529	B03	20752953	664
M207530	B03	20753073	664

M207619	B03	20761911	665
M207733	B03	20773384	665
M207818	B03	20781866	665
M208126	B03	20812694	666
M208171	B03	20817146	666
M208248	B03	20824871	666
M208316	B03	20831605	666
M208322	B03	20832260	666
M208377	B03	20837749	666
M208461	B03	20846124	667
M208565	B03	20856537	667
M208669	B03	20866997	667
M208698	B03	20869809	667
M208755	B03	20875588	668
M208801	B03	20880187	668
M208990	B03	20899047	669
M209249	B03	20924948	669
M209338	B03	20933863	669
M209492	B03	20949250	669
M209585	B03	20958522	669
M209656	B03	20965658	669
M209739	B03	20973961	669
M209769	B03	20976986	669
M209977	B03	20997768	669
M210529	B03	21052904	669
M225055	B03	22505504	670
M225077	B03	22507765	670
M225106	B03	22510629	670
M225344	B03	22534490	671
M225675	B03	22567508	671
M225723	B03	22572348	671
M225723	B03	22572361	671
M225878	B03	22587842	671
M226017	B03	22601796	671
M226072	B03	22607242	671
M226188	B03	22618837	672
M226219	B03	22621912	672
M226625	B03	22662502	672
M226641	B03	22664109	672
M226852	B03	22685203	672
M226929	B03	22692963	672
M227010	B03	22701016	672
M227024	B03	22702454	672
M227091	B03	22709145	673
M227119	B03	22711993	673
M227228	B03	22722878	673
M227376	B03	22737640	673

M227473	B03	22747370	673
M229346	B03	22934619	674
M229362	B03	22936253	674
M231268	B03	23126820	675
M231310	B03	23131025	675
M231429	B03	23142907	675
M231591	B03	23159197	675
M231642	B03	23164233	675
M231651	B03	23165197	675
M231700	B03	23170006	675
M231843	B03	23184385	675
M232035	B03	23203534	675
M232162	B03	23216289	675
M232181	B03	23218190	675
M232514	B03	23251445	676
M232561	B03	23256175	676
M232649	B03	23264916	676
M233192	B03	23319257	676
M233407	B03	23340729	677
M233466	B03	23346664	677
M233478	B03	23347889	677
M233763	B03	23376307	677
M233827	B03	23382768	677
M233983	B03	23398300	677
M234140	B03	23414075	678
M234229	B03	23422926	678
M234341	B03	23434191	678
M234410	B03	23441001	678
M234450	B03	23445057	678
M234990	B03	23499037	678
M235075	B03	23507592	678
M235095	B03	23509511	678
M235160	B03	23516003	678
M235253	B03	23525333	678
M235348	B03	23534890	678
M235448	B03	23544835	678
M235665	B03	23566521	678
M235789	B03	23578962	679
M235829	B03	23582936	679
M235856	B03	23585666	679
M235881	B03	23588113	679
M235923	B03	23592324	679
M235956	B03	23595698	679
M236011	B03	23601194	679
M236426	B03	23642638	679
M236486	B03	23648617	679
M236494	B03	23649459	679

M236514	B03	23651485	679
M236519	B03	23651928	679
M236570	B03	23657062	679
M236643	B03	23664379	679
M236952	B03	23695279	680
M237012	B03	23701201	680
M237256	B03	23725650	680
M237304	B03	23730458	680
M237361	B03	23736144	680
M237473	B03	23747371	680
M237600	B03	23760049	681
M238085	B03	23808596	681
M238118	B03	23811871	682
M238411	B03	23841109	682
M238639	B03	23863946	682
M238695	B03	23869545	683
M238735	B03	23873564	683
M238888	B03	23888823	684
M238929	B03	23892909	684
M239079	B03	23907971	685
M239100	B03	23910025	685
M239104	B03	23910476	685
M239127	B03	23912768	685
M239185	B03	23918548	685
M239274	B03	23927413	685
M239316	B03	23931655	685
M239349	B03	23934976	685
M239370	B03	23937033	685
M239413	B03	23941356	685
M239528	B03	23952805	685
M239751	B03	23975139	685
M239762	B03	23976250	685
M239830	B03	23983049	685
M239849	B03	23984944	685
M239926	B03	23992679	685
M240079	B03	24007948	686
M240162	B03	24016200	686
M240188	B03	24018875	686
M240249	B03	24024946	687
M240251	B03	24025128	687
M240309	B03	24030918	687
M241463	B03	24146322	687
M246099	B03	24609957	688
M246121	B03	24612149	688
M246285	B03	24628537	689
M246553	B03	24655373	689
M246579	B03	24657908	689

M246733	B03	24673354	689
M246817	B03	24681718	689
M246898	B03	24689835	689
M247077	B03	24707798	689
M252618	B03	25261866	690
M253511	B03	25351138	690
M253620	B03	25362065	690
M253762	B03	25376258	690
M253879	B03	25387901	690
M253964	B03	25396489	690
M254118	B03	25411890	690
M254154	B03	25415477	690
M254243	B03	25424399	691
M254279	B03	25427950	691
M254344	B03	25434403	691
M254392	B03	25439218	691
M254425	B03	25442517	691
M254454	B03	25445400	691
M254593	B03	25459308	691
M254636	B03	25463679	691
M254736	B03	25473631	692
M254805	B03	25480535	692
M254872	B03	25487258	692
M254957	B03	25495754	692
M255030	B03	25503001	692
M255102	B03	25510206	692
M255191	B03	25519191	692
M255295	B03	25529506	692
M256060	B03	25606069	692
M256181	B03	25618128	692
M256246	B03	25624657	692
M256271	B03	25627141	692
M256357	B03	25635761	693
M256413	B03	25641315	693
M256478	B03	25647813	694
M256565	B03	25656516	694
M256646	B03	25664606	694
M256689	B03	25668921	694
M257046	B03	25704624	694
M257199	B03	25719920	694
M257637	B03	25763797	695
M258044	B03	25804401	695
M258532	B03	25853289	695
M258668	B03	25866868	695
M258895	B03	25889569	695
M258983	B03	25898371	695
M259000	B03	25900084	695

M259077	B03	25907733	695
M259133	B03	25913382	695
M259190	B03	25919016	695
M259279	B03	25927955	696
M259515	B03	25951577	696
M259756	B03	25975699	697
M261273	B03	26127388	697
M261348	B03	26134899	697
M261368	B03	26136857	698
M261484	B03	26148479	698
M261540	B03	26154076	698
M261642	B03	26164278	698
M261673	B03	26167382	698
M261909	B03	26190938	698
M261961	B03	26196157	698
M262077	B03	26207716	698
M262398	B03	26239875	698
M262416	B03	26241683	698
M262462	B03	26246266	698
M262484	B03	26248438	698
M262605	B03	26260573	698
M262814	B03	26281453	698
M262898	B03	26289813	698
M262951	B03	26295140	698
M263012	B03	26301267	699
M263276	B03	26327649	699
M263296	B03	26329641	699
M263320	B03	26332067	700
M263346	B03	26334690	700
M263385	B03	26338552	700
M263418	B03	26341834	700
M263489	B03	26348999	700
M263521	B03	26352145	700
M263697	B03	26369702	700
M263865	B03	26386527	700
M263920	B03	26392030	700
M263959	B03	26395983	700
M264079	B03	26407930	700
M264091	B03	26409161	700
M264161	B03	26416136	700
M264797	B03	26479769	700
M264819	B03	26481989	700
M264863	B03	26486358	700
M264905	B03	26490568	700
M264919	B03	26491938	700
M265048	B03	26504813	701
M265060	B03	26506034	701

M265070	B03	26507040	701
M265131	B03	26513163	701
M265207	B03	26520731	702
M266742	B03	26674225	702
M266772	B03	26677224	702
M266840	B03	26684089	703
M266885	B03	26688576	703
M267049	B03	26704956	703
M267086	B03	26708644	703
M267138	B03	26713889	703
M267203	B03	26720308	703
M267218	B03	26721845	703
M268823	B03	26882315	704
M269361	B03	26936189	704
M270271	B03	27027143	704
M270471	B03	27047106	705
M270551	B03	27055199	705
M270605	B03	27060505	705
M270662	B03	27066259	706
M270666	B03	27066699	706
M271140	B03	27114038	706
M271306	B03	27130663	706
M271596	B03	27159682	706
M271954	B03	27195440	706
M271980	B03	27198010	706
M272049	B03	27204937	706
M272120	B03	27212059	706
M272180	B03	27218003	706
M272258	B03	27225844	706
M272437	B03	27243765	707
M272678	B03	27267828	707
M272794	B03	27279473	707
M272814	B03	27281497	707
M272933	B03	27293336	707
M272955	B03	27295573	707
M272971	B03	27297120	707
M272997	B03	27299723	707
M273093	B03	27309399	707
M273133	B03	27313338	707
M273239	B03	27323977	707
M273356	B03	27335670	707
M273407	B03	27340744	707
M273412	B03	27341240	707
M273448	B03	27344819	707
M273484	B03	27348487	707
M273587	B03	27358753	707
M273660	B03	27366090	707

M273757	B03	27375733	707
M273804	B03	27380492	707
M273859	B03	27385995	708
M274514	B03	27451427	708
M274623	B03	27462373	708
M274899	B03	27489977	708
M275088	B03	27508802	708
M275215	B03	27521547	708
M275279	B03	27527965	708
M275447	B03	27544720	708
M275556	B03	27555639	709
M275591	B03	27559123	709
M275630	B03	27563060	710
M275698	B03	27569839	710
M275741	B03	27574169	710
M275750	B03	27575053	710
M275793	B03	27579317	710
M275876	B03	27587602	710
M275895	B03	27589547	710
M275946	B03	27594639	710
M276039	B03	27603993	710
M276065	B03	27606579	710
M276089	B03	27608948	710
M276618	B03	27661837	710
M277080	B03	27708064	710
M277223	B03	27722393	710
M277321	B03	27732195	711
M277721	B03	27772107	711
M277823	B03	27782362	711
M277858	B03	27785895	711
M277885	B03	27788560	711
M278145	B03	27814526	711
M278195	B03	27819594	711
M281369	B03	28136916	712
M281588	B03	28158865	712
M281805	B03	28180520	712
M287363	B03	28736339	713
M287998	B03	28799806	713
M288783	B03	28878315	713
M291192	B03	29119288	714
M292385	B03	29238500	714
M292707	B03	29270775	714
M294636	B03	29463684	715
M294837	B03	29483736	715
M297461	B03	29746104	716
M297533	B03	29753345	716
M297645	B03	29764529	716

M297713	B03	29771340	716
M297855	B03	29785501	717
M299313	B03	29931395	717
M300838	B03	30083891	718
M300874	B03	30087480	718
M300963	B03	30096388	718
M301073	B03	30107350	718
M301118	B03	30111816	719
M301258	B03	30125890	719
M301288	B03	30128893	720
M301416	B03	30141654	720
M301443	B03	30144343	720
M301633	B03	30163370	720
M302101	B03	30210130	721
M302213	B03	30221394	721
M302369	B03	30236989	721
M302406	B03	30240614	721
M302478	B03	30247851	722
M303550	B03	30355055	722
M313803	B03	31380347	723
M314143	B03	31414319	723
M314205	B03	31420530	723
M317220	B03	31722006	724
M318705	B03	31870570	724
M320311	B03	32031117	725
M321830	B03	32183069	725
M326099	B03	32609950	726
M327158	B03	32715855	726
M335258	B03	33525870	727
M335642	B03	33564260	727
M336789	B03	33678952	727
M337742	B03	33774256	728
M338872	B03	33887245	728
M340858	B03	34085824	729
M342274	B03	34227435	729
M348809	B03	34880918	730
M349732	B03	34973210	730
M351196	B03	35119643	731
M351419	B03	35141915	731
M608375	B04	608375	732
M630579	B04	630579	732
M638062	B04	638062	732
M648755	B04	648755	732
M674488	B04	674488	732
M681257	B04	681257	732
M692904	B04	692904	732
M699050	B04	699050	733

M702100	B04	702100	733
M850691	B04	850691	733
M860950	B04	860950	734
M872961	B04	872961	734
M875438	B04	875438	734
M878672	B04	878672	734
M882410	B04	882410	734
M886924	B04	886924	734
M888532	B04	888532	734
M896420	B04	896420	735
M924181	B04	924181	735
M101971	B04	1019711	736
M102883	B04	1028832	736
M103467	B04	1034678	736
M103748	B04	1037487	736
M107271	B04	1072714	736
M107279	B04	1072792	736
M107748	B04	1077489	736
M107877	B04	1078772	736
M109181	B04	1091814	736
M109501	B04	1095011	736
M109697	B04	1096975	736
M112383	B04	1123836	737
M112988	B04	1129880	737
M113552	B04	1135521	737
M116001	B04	1160013	737
M120394	B04	1203940	738
M121193	B04	1211937	738
M121469	B04	1214690	738
M121813	B04	1218137	738
M123604	B04	1236043	739
M127380	B04	1273808	739
M128900	B04	1289000	740
M129493	B04	1294935	740
M130367	B04	1303670	741
M132154	B04	1321547	741
M133319	B04	1333193	741
M134940	B04	1349402	742
M135242	B04	1352422	742
M136731	B04	1367319	743
M136792	B04	1367922	743
M138053	B04	1380530	744
M140051	B04	1400514	744
M141066	B04	1410661	745
M141483	B04	1414830	745
M178713	B04	1787135	746
M179312	B04	1793128	746

M180198	B04	1801988	746
M180853	B04	1808534	746
M181274	B04	1812741	746
M181465	B04	1814657	746
M181669	B04	1816697	747
M181867	B04	1818677	747
M182172	B04	1821729	748
M182748	B04	1827480	748
M183497	B04	1834972	748
M184276	B04	1842764	749
M187578	B04	1875789	749
M187953	B04	1879533	750
M188645	B04	1886457	750
M189114	B04	1891142	751
M189283	B04	1892837	751
M193646	B04	1936466	752
M194222	B04	1942220	752
M195032	B04	1950329	752
M195947	B04	1959472	752
M196232	B04	1962320	752
M197998	B04	1979988	752
M198410	B04	1984107	752
M205623	B04	2056233	753
M206501	B04	2065012	753
M207428	B04	2074284	753
M210127	B04	2101276	754
M210303	B04	2103030	754
M212405	B04	2124052	754
M213252	B04	2132524	755
M213601	B04	2136018	755
M217249	B04	2172497	755
M217655	B04	2176556	755
M218427	B04	2184274	755
M218982	B04	2189827	755
M219524	B04	2195245	755
M219791	B04	2197911	755
M220410	B04	2204102	755
M222367	B04	2223679	755
M223334	B04	2233344	755
M224810	B04	2248102	755
M293498	B04	2934989	756
M295041	B04	2950418	756
M297037	B04	2970373	756
M297753	B04	2977537	756
M297865	B04	2978653	756
M320900	B04	3209003	757
M321075	B04	3210752	757

M321168	B04	3211687	757
M324696	B04	3246967	758
M324891	B04	3248911	758
M325223	B04	3252232	758
M325871	B04	3258713	758
M326348	B04	3263485	758
M326431	B04	3264317	758
M327533	B04	3275334	758
M329478	B04	3294782	758
M329764	B04	3297645	758
M330652	B04	3306520	758
M338320	B04	3383200	759
M346672	B04	3466724	759
M346954	B04	3469548	759
M347416	B04	3474161	760
M347684	B04	3476840	760
M348097	B04	3480976	761
M350613	B04	3506131	761
M350860	B04	3508600	761
M350906	B04	3509068	761
M351099	B04	3510991	761
M351545	B04	3515454	761
M352113	B04	3521133	762
M352988	B04	3529889	762
M353691	B04	3536914	763
M353788	B04	3537887	763
M354596	B04	3545960	763
M355330	B04	3553309	764
M355540	B04	3555402	764
M356253	B04	3562531	765
M356399	B04	3563991	765
M358680	B04	3586808	766
M358786	B04	3587862	766
M358877	B04	3588772	766
M359421	B04	3594212	766
M360027	B04	3600273	766
M367261	B04	3672616	767
M367316	B04	3673162	767
M378661	B04	3786612	767
M379758	B04	3797581	767
M436374	B04	4363749	768
M437246	B04	4372466	768
M437494	B04	4374944	768
M438019	B04	4380197	768
M438965	B04	4389654	768
M439473	B04	4394737	768
M440281	B04	4402814	769

M440532	B04	4405324	769
M454073	B04	4540735	769
M461531	B04	4615318	770
M461932	B04	4619325	770
M462298	B04	4622989	770
M463486	B04	4634867	771
M463891	B04	4638915	771
M464552	B04	4645527	771
M465134	B04	4651342	771
M466808	B04	4668089	771
M473129	B04	4731293	771
M476475	B04	4764758	771
M477137	B04	4771375	771
M477189	B04	4771899	771
M477786	B04	4777865	771
M478170	B04	4781709	771
M478570	B04	4785703	771
M486142	B04	4861421	772
M488568	B04	4885687	772
M488740	B04	4887407	772
M489038	B04	4890386	772
M491032	B04	4910329	772
M494513	B04	4945138	772
M495429	B04	4954291	772
M495813	B04	4958134	772
M497173	B04	4971735	772
M498053	B04	4980537	773
M498647	B04	4986478	773
M498835	B04	4988357	773
M499115	B04	4991153	773
M500402	B04	5004021	774
M501428	B04	5014283	774
M501840	B04	5018404	774
M504186	B04	5041869	775
M504228	B04	5042288	775
M508965	B04	5089659	775
M509166	B04	5091664	775
M509720	B04	5097208	776
M511182	B04	5111825	776
M515756	B04	5157568	777
M516041	B04	5160415	777
M516827	B04	5168273	777
M518041	B04	5180410	777
M519090	B04	5190902	777
M520280	B04	5202807	778
M522339	B04	5223397	778
M523170	B04	5231705	778

M525506	B04	5255065	778
M526941	B04	5269416	778
M528674	B04	5286748	778
M528996	B04	5289963	778
M529429	B04	5294290	778
M529985	B04	5299851	778
M530196	B04	5301969	779
M531155	B04	5311550	779
M531355	B04	5313559	780
M531868	B04	5318684	780
M536336	B04	5363361	781
M537700	B04	5377004	781
M538809	B04	5388097	781
M539860	B04	5398609	781
M539976	B04	5399765	781
M546187	B04	5461874	781
M574426	B04	5744260	782
M575072	B04	5750722	782
M575530	B04	5755308	782
M577332	B04	5773327	783
M578205	B04	5782051	783
M578293	B04	5782938	783
M578530	B04	5785301	783
M580986	B04	5809862	784
M581233	B04	5812337	784
M581735	B04	5817354	784
M583257	B04	5832572	784
M584095	B04	5840952	785
M584468	B04	5844680	785
M584860	B04	5848600	785
M586893	B04	5868938	786
M587693	B04	5876934	786
M588064	B04	5880640	786
M605917	B04	6059173	787
M607111	B04	6071117	787
M608115	B04	6081159	787
M609773	B04	6097734	788
M609868	B04	6098685	788
M610229	B04	6102296	788
M610509	B04	6105092	788
M610914	B04	6109149	789
M611696	B04	6116965	789
M642800	B04	6428008	790
M642871	B04	6428717	790
M643391	B04	6433911	790
M643693	B04	6436936	790
M645948	B04	6459485	791

M646100	B04	6461003	791
M646522	B04	6465224	791
M646602	B04	6466028	791
M647253	B04	6472536	791
M647511	B04	6475117	791
M650572	B04	6505726	792
M653762	B04	6537628	792
M655857	B04	6558570	793
M656184	B04	6561845	793
M656752	B04	6567527	793
M700441	B04	7004417	794
M701005	B04	7010058	794
M701779	B04	7017798	794
M702764	B04	7027641	794
M710311	B04	7103112	794
M711066	B04	7110667	794
M714030	B04	7140307	795
M715384	B04	7153841	795
M716894	B04	7168948	795
M717543	B04	7175431	795
M718860	B04	7188605	796
M719118	B04	7191186	796
M739105	B04	7391059	797
M739358	B04	7393585	797
M745430	B04	7454308	798
M746104	B04	7461046	798
M746979	B04	7469794	798
M748402	B04	7484020	798
M748798	B04	7487986	798
M749828	B04	7498280	798
M786752	B04	7867523	799
M787136	B04	7871367	799
M789890	B04	7898905	800
M792718	B04	7927187	800
M793295	B04	7932952	800
M793493	B04	7934936	800
M794750	B04	7947502	800
M795582	B04	7955828	800
M795809	B04	7958090	800
M796724	B04	7967244	800
M798353	B04	7983538	801
M799228	B04	7992286	801
M800023	B04	8000230	801
M801072	B04	8010724	801
M807570	B04	8075709	802
M807697	B04	8076970	802
M807939	B04	8079391	802

M809871	B04	8098716	803
M811321	B04	8113219	803
M811896	B04	8118961	803
M816071	B04	8160710	804
M816240	B04	8162404	804
M816481	B04	8164815	805
M817946	B04	8179461	805
M818855	B04	8188555	805
M819234	B04	8192340	805
M831041	B04	8310416	805
M838449	B04	8384495	806
M840056	B04	8400568	806
M888153	B04	8881536	807
M889516	B04	8895163	807
M890403	B04	8904038	807
M890914	B04	8909148	808
M891622	B04	8916223	808
M892034	B04	8920344	808
M892456	B04	8924564	808
M892754	B04	8927545	809
M893756	B04	8937564	809
M914437	B04	9144375	810
M916320	B04	9163204	810
M926256	B04	9262562	811
M927365	B04	9273651	811
M928951	B04	9289513	811
M931955	B04	9319555	812
M932829	B04	9328291	812
M933201	B04	9332014	812
M933554	B04	9335540	812
M933969	B04	9339695	812
M935137	B04	9351376	813
M935708	B04	9357080	813
M936849	B04	9368498	814
M936984	B04	9369846	814
M937256	B04	9372567	814
M937585	B04	9375856	815
M945581	B04	9455817	815
M945974	B04	9459749	816
M946201	B04	9462015	816
M947542	B04	9475421	816
M947907	B04	9479072	816
M949999	B04	9499999	817
M950428	B04	9504288	817
M964165	B04	9641655	818
M964364	B04	9643643	818
M966744	B04	9667444	818

M100319	B04	10031972	819
M100356	B04	10035646	819
M100361	B04	10036188	819
M100488	B04	10048869	820
M100517	B04	10051770	820
M101460	B04	10146008	821
M101638	B04	10163829	821
M101851	B04	10185164	821
M102129	B04	10212939	822
M102373	B04	10237383	822
M102438	B04	10243883	822
M108337	B04	10833730	823
M108363	B04	10836371	823
M109097	B04	10909797	823
M109156	B04	10915625	823
M109256	B04	10925614	823
M109423	B04	10942343	823
M109462	B04	10946215	823
M109505	B04	10950530	823
M109754	B04	10975452	824
M109920	B04	10992050	824
M109981	B04	10998186	824
M110072	B04	11007222	824
M110545	B04	11054517	825
M111487	B04	11148718	825
M111632	B04	11163256	825
M112119	B04	11211952	825
M112451	B04	11245124	826
M112511	B04	11251117	826
M112616	B04	11261642	827
M112723	B04	11272360	827
M112868	B04	11286808	827
M113546	B04	11354612	827
M113768	B04	11376804	827
M113795	B04	11379528	827
M113889	B04	11388912	827
M114206	B04	11420612	827
M114332	B04	11433216	828
M114433	B04	11443348	828
M114455	B04	11445545	828
M114510	B04	11451097	828
M114620	B04	11462016	828
M114734	B04	11473475	828
M114756	B04	11475607	828
M114810	B04	11481087	828
M115074	B04	11507409	829
M115133	B04	11513364	829

M115275	B04	11527520	830
M115282	B04	11528291	830
M115365	B04	11536509	830
M115819	B04	11581996	831
M117170	B04	11717087	831
M117255	B04	11725574	831
M117415	B04	11741522	831
M117642	B04	11764219	832
M117660	B04	11766013	832
M117686	B04	11768608	832
M117932	B04	11793280	833
M118020	B04	11802029	833
M118604	B04	11860441	834
M118778	B04	11877827	834
M119013	B04	11901349	835
M119499	B04	11949945	835
M119521	B04	11952188	835
M119644	B04	11964467	836
M119705	B04	11970533	836
M119897	B04	11989714	837
M120442	B04	12044289	837
M121610	B04	12161014	838
M121625	B04	12162530	838
M121690	B04	12169025	839
M121768	B04	12176853	839
M121996	B04	12199635	839
M122144	B04	12214415	839
M122424	B04	12242478	839
M122616	B04	12261619	839
M122631	B04	12263197	839
M126315	B04	12631588	840
M126331	B04	12633146	840
M127432	B04	12743296	840
M129380	B04	12938020	841
M129390	B04	12939009	841
M129509	B04	12950929	841
M130111	B04	13011147	842
M130134	B04	13013437	842
M130147	B04	13014788	842
M131661	B04	13166162	842
M131944	B04	13194433	843
M132056	B04	13205690	843
M132280	B04	13228033	843
M132337	B04	13233732	843
M146600	B04	14660005	844
M146646	B04	14664605	844
M146821	B04	14682171	844

M146841	B04	14684118	844
M150207	B04	15020795	845
M150216	B04	15021680	845
M150561	B04	15056147	845
M150690	B04	15069064	845
M150749	B04	15074997	845
M151095	B04	15109512	846
M151116	B04	15111670	846
M151227	B04	15122797	846
M151342	B04	15134210	847
M151539	B04	15153927	847
M152705	B04	15270547	847
M152725	B04	15272541	847
M152812	B04	15281226	847
M152876	B04	15287633	847
M153739	B04	15373933	848
M153832	B04	15383208	848
M154163	B04	15416354	849
M154326	B04	15432698	849
M154339	B04	15433984	849
M154385	B04	15438555	849
M154936	B04	15493670	849
M154955	B04	15495552	849
M155067	B04	15506769	850
M155115	B04	15511538	850
M155168	B04	15516839	850
M155191	B04	15519100	850
M155383	B04	15538378	851
M155523	B04	15552345	851
M155641	B04	15564182	851
M155759	B04	15575958	852
M155792	B04	15579274	852
M155808	B04	15580824	852
M155951	B04	15595140	853
M156002	B04	15600258	853
M156062	B04	15606236	853
M158069	B04	15806903	854
M158226	B04	15822663	854
M158459	B04	15845930	855
M158491	B04	15849100	855
M158585	B04	15858528	855
M158612	B04	15861248	855
M158787	B04	15878722	856
M160138	B04	16013828	856
M160297	B04	16029721	856
M160911	B04	16091173	857
M160978	B04	16097890	857

M161045	B04	16104577	857
M161120	B04	16112077	857
M161143	B04	16114313	857
M161424	B04	16142484	857
M161472	B04	16147273	857
M161522	B04	16152250	857
M164082	B04	16408233	858
M164163	B04	16416391	858
M164289	B04	16428988	859
M165400	B04	16540095	859
M170107	B04	17010722	860
M170186	B04	17018645	860
M170215	B04	17021571	860
M170714	B04	17071437	860
M170745	B04	17074580	860
M170763	B04	17076386	860
M170834	B04	17083490	860
M170867	B04	17086773	860
M170944	B04	17094424	861
M171006	B04	17100613	861
M171027	B04	17102776	861
M171102	B04	17110200	861
M171226	B04	17122647	861
M175998	B04	17599873	862
M176051	B04	17605137	862
M176233	B04	17623323	862
M176380	B04	17638026	862
M176455	B04	17645530	862
M176517	B04	17651732	863
M176534	B04	17653434	863
M176630	B04	17663073	863
M176923	B04	17692339	864
M177052	B04	17705299	864
M177265	B04	17726585	865
M177296	B04	17729678	865
M177336	B04	17733634	865
M177430	B04	17743073	865
M177446	B04	17744645	865
M177507	B04	17750748	865
M177812	B04	17781244	865
M177938	B04	17793805	865
M178236	B04	17823624	866
M178386	B04	17838654	866
M178445	B04	17844525	866
M178492	B04	17849282	866
M178550	B04	17855076	866
M178763	B04	17876324	866

M178863	B04	17886392	866
M178906	B04	17890699	866
M178931	B04	17893179	866
M178953	B04	17895328	866
M178996	B04	17899678	866
M179135	B04	17913504	866
M179765	B04	17976593	866
M179774	B04	17977463	866
M180125	B04	18012583	867
M180190	B04	18019022	867
M180377	B04	18037709	867
M180424	B04	18042470	867
M180457	B04	18045750	867
M180469	B04	18046976	867
M180501	B04	18050105	868
M180585	B04	18058514	868
M180642	B04	18064233	868
M180713	B04	18071332	868
M180803	B04	18080392	868
M180982	B04	18098275	868
M202308	B04	20230862	869
M203198	B04	20319848	869
M203277	B04	20327741	869
M203553	B04	20355357	870
M203672	B04	20367283	870
M203801	B04	20380180	871
M203879	B04	20387970	871
M203967	B04	20396761	871
M204135	B04	20413575	872
M204256	B04	20425632	872
M204416	B04	20441649	872
M204458	B04	20445876	872
M204496	B04	20449666	872
M204506	B04	20450698	872
M204727	B04	20472732	872
M204763	B04	20476300	872
M204963	B04	20496364	873
M205130	B04	20513042	873
M205315	B04	20531562	874
M205378	B04	20537861	874
M206704	B04	20670448	874
M208505	B04	20850512	875
M208676	B04	20867612	875
M208800	B04	20880002	875
M208832	B04	20883231	876
M208880	B04	20888016	876
M216230	B04	21623043	877

M216424	B04	21642479	877
M216452	B04	21645217	877
M216486	B04	21648643	878
M216525	B04	21652516	878
M217014	B04	21701497	879
M217087	B04	21708783	879
M217202	B04	21720268	879
M217757	B04	21775779	879
M219924	B04	21992408	880
M220730	B04	22073017	880
M221025	B04	22102589	881
M221737	B04	22173786	881
M225941	B04	22594162	882
M225985	B04	22598588	882
M265633	B04	26563383	883
M266962	B04	26696258	883
M268402	B04	26840242	884
M268935	B04	26893531	884
M304902	B04	30490292	885
M306146	B04	30614649	885
M324602	B04	32460239	886
M325603	B04	32560369	886
M333113	B04	33311330	887
M334231	B04	33423127	887
M359242	B04	35924293	888
M359968	B04	35996879	888
M362116	B04	36211610	889
M362863	B04	36286312	889
M366503	B04	36650397	890
M366797	B04	36679704	890
M392692	B04	39269299	891
M393753	B04	39375377	891
M398103	B04	39810326	892
M398368	B04	39836890	892
M399185	B04	39918505	892
M403857	B04	40385787	893
M404319	B04	40431942	893
M406293	B04	40629327	894
M407398	B04	40739879	894
M432103	B04	43210346	895
M433239	B04	43323927	895
M7947	B05	7947	896
M12456	B05	12456	896
M16937	B05	16937	897
M22280	B05	22280	897
M35961	B05	35961	898
M41996	B05	41996	898

M50221	B05	50221	898
M77852	B05	77852	898
M496956	B05	496956	899
M504336	B05	504336	899
M512733	B05	512733	899
M519645	B05	519645	899
M523594	B05	523594	899
M957920	B05	957920	900
M106992	B05	1069921	900
M111239	B05	1112391	900
M113821	B05	1138219	901
M116767	B05	1167673	901
M117345	B05	1173459	901
M117715	B05	1177154	901
M119815	B05	1198158	902
M120739	B05	1207399	902
M121667	B05	1216671	902
M122112	B05	1221125	902
M124043	B05	1240432	902
M135354	B05	1353541	902
M136022	B05	1360227	903
M136758	B05	1367582	903
M136954	B05	1369545	903
M140773	B05	1407738	904
M140865	B05	1408651	904
M141072	B05	1410725	904
M141228	B05	1412282	904
M142528	B05	1425285	904
M142989	B05	1429893	904
M147041	B05	1470417	905
M149525	B05	1495251	905
M149597	B05	1495978	905
M150348	B05	1503486	906
M151146	B05	1511469	906
M151428	B05	1514285	907
M151693	B05	1516939	907
M153911	B05	1539110	907
M155378	B05	1553784	908
M155924	B05	1559241	908
M157045	B05	1570457	908
M157270	B05	1572704	908
M158084	B05	1580844	908
M158125	B05	1581252	908
M158470	B05	1584709	908
M158985	B05	1589859	908
M159800	B05	1598001	908
M159900	B05	1599001	908

M164639	B05	1646391	908
M165325	B05	1653254	908
M165900	B05	1659007	908
M170526	B05	1705261	909
M171028	B05	1710289	909
M174269	B05	1742698	909
M182230	B05	1822303	909
M182696	B05	1826963	909
M183219	B05	1832197	909
M183530	B05	1835305	909
M183908	B05	1839083	909
M184206	B05	1842066	909
M184684	B05	1846846	909
M185182	B05	1851827	909
M185891	B05	1858916	909
M186261	B05	1862618	909
M212595	B05	2125959	910
M212674	B05	2126744	910
M222830	B05	2228300	911
M232062	B05	2320627	911
M232331	B05	2323312	911
M233251	B05	2332510	912
M233597	B05	2335970	912
M233867	B05	2338678	913
M234313	B05	2343133	913
M235308	B05	2353087	914
M235756	B05	2357567	914
M240353	B05	2403532	914
M241003	B05	2410039	914
M241143	B05	2411435	914
M242712	B05	2427127	914
M242991	B05	2429917	914
M244033	B05	2440331	914
M251239	B05	2512396	914
M265093	B05	2650930	915
M265795	B05	2657954	915
M268086	B05	2680864	915
M268526	B05	2685267	916
M268880	B05	2688809	916
M273089	B05	2730897	917
M280684	B05	2806845	917
M281499	B05	2814995	917
M282128	B05	2821280	917
M286510	B05	2865109	918
M286778	B05	2867789	918
M299056	B05	2990565	918
M299348	B05	2993480	918

M300793	B05	3007937	918
M304545	B05	3045455	919
M304690	B05	3046901	919
M305156	B05	3051568	919
M305998	B05	3059989	919
M306164	B05	3061644	919
M306348	B05	3063484	919
M306878	B05	3068787	919
M338527	B05	3385274	920
M339355	B05	3393550	920
M339672	B05	3396724	920
M340664	B05	3406646	920
M341200	B05	3412007	920
M341927	B05	3419276	920
M342363	B05	3423634	920
M344141	B05	3441411	920
M345446	B05	3454462	920
M346862	B05	3468620	920
M348662	B05	3486629	920
M349574	B05	3495747	920
M350217	B05	3502172	920
M397006	B05	3970060	921
M397132	B05	3971324	921
M397471	B05	3974712	921
M398127	B05	3981273	921
M417207	B05	4172072	922
M417515	B05	4175152	922
M417866	B05	4178668	922
M418196	B05	4181965	922
M419306	B05	4193061	922
M420057	B05	4200576	922
M420419	B05	4204193	922
M420635	B05	4206356	922
M420896	B05	4208964	922
M421060	B05	4210600	922
M421298	B05	4212987	922
M421322	B05	4213228	922
M421543	B05	4215439	922
M422947	B05	4229475	922
M424086	B05	4240860	922
M424212	B05	4242123	922
M424258	B05	4242585	922
M430117	B05	4301177	922
M488848	B05	4888488	923
M493736	B05	4937369	923
M493939	B05	4939392	923
M494705	B05	4947054	923

M494955	B05	4949559	923
M495802	B05	4958029	923
M497874	B05	4978744	923
M498015	B05	4980153	923
M499523	B05	4995237	923
M499619	B05	4996198	923
M500787	B05	5007876	923
M500889	B05	5008897	923
M501156	B05	5011561	923
M501576	B05	5015768	923
M501894	B05	5018942	923
M502157	B05	5021576	923
M502434	B05	5024348	923
M503299	B05	5032999	923
M503510	B05	5035105	923
M503697	B05	5036972	923
M519896	B05	5198965	924
M520289	B05	5202895	924
M521010	B05	5210102	924
M521992	B05	5219924	924
M523871	B05	5238713	924
M523963	B05	5239636	924
M524692	B05	5246929	925
M525044	B05	5250445	925
M525585	B05	5255854	925
M525978	B05	5259785	925
M526335	B05	5263352	925
M526824	B05	5268246	925
M527645	B05	5276451	925
M528081	B05	5280819	925
M528156	B05	5281568	925
M528562	B05	5285626	925
M528894	B05	5288941	925
M529095	B05	5290958	925
M529562	B05	5295629	925
M530082	B05	5300828	925
M530518	B05	5305185	925
M531115	B05	5311151	925
M531213	B05	5312133	925
M531644	B05	5316449	925
M532085	B05	5320851	925
M532311	B05	5323116	925
M532646	B05	5326462	925
M533056	B05	5330568	925
M533958	B05	5339581	925
M534364	B05	5343648	925
M534731	B05	5347317	925

M540363	B05	5403631	925
M577895	B05	5778958	926
M586074	B05	5860740	926
M587148	B05	5871481	927
M592148	B05	5921485	927
M592337	B05	5923374	927
M593184	B05	5931844	927
M594749	B05	5947493	927
M599044	B05	5990444	927
M599393	B05	5993933	927
M600430	B05	6004305	927
M600890	B05	6008909	927
M601292	B05	6012920	927
M601867	B05	6018677	927
M603098	B05	6030981	927
M603945	B05	6039450	928
M605700	B05	6057001	928
M607165	B05	6071658	928
M610955	B05	6109557	928
M615679	B05	6156795	928
M627279	B05	6272798	929
M637034	B05	6370341	929
M646979	B05	6469793	930
M647255	B05	6472556	930
M647657	B05	6476574	930
M647804	B05	6478048	930
M649041	B05	6490416	930
M649792	B05	6497920	930
M650550	B05	6505503	930
M653326	B05	6533268	930
M654865	B05	6548655	930
M655558	B05	6555580	930
M656088	B05	6560883	930
M656755	B05	6567552	930
M656990	B05	6569905	930
M694275	B05	6942753	931
M700387	B05	7003877	931
M700850	B05	7008503	931
M705150	B05	7051507	931
M738252	B05	7382523	932
M738670	B05	7386700	932
M741501	B05	7415017	932
M742057	B05	7420572	932
M743641	B05	7436418	932
M744652	B05	7446520	932
M747128	B05	7471287	932
M747704	B05	7477041	933

M748348	B05	7483487	933
M748851	B05	7488515	933
M749110	B05	7491100	933
M749733	B05	7497333	933
M750410	B05	7504104	933
M750980	B05	7509808	933
M751583	B05	7515836	933
M751821	B05	7518217	933
M752418	B05	7524180	933
M761380	B05	7613804	933
M763616	B05	7636163	933
M767303	B05	7673034	934
M767876	B05	7678763	934
M769698	B05	7696981	934
M771385	B05	7713857	934
M771916	B05	7719166	934
M772430	B05	7724307	934
M772736	B05	7727368	934
M773228	B05	7732281	934
M773963	B05	7739636	934
M774514	B05	7745145	934
M775071	B05	7750710	934
M775823	B05	7758230	934
M776639	B05	7766394	934
M777038	B05	7770380	934
M778794	B05	7787949	934
M781598	B05	7815984	934
M782410	B05	7824100	935
M783735	B05	7837353	935
M784704	B05	7847047	935
M786227	B05	7862278	935
M786972	B05	7869729	935
M787529	B05	7875295	935
M788013	B05	7880133	935
M788945	B05	7889450	935
M789988	B05	7899881	935
M791798	B05	7917984	935
M791904	B05	7919043	935
M792247	B05	7922476	935
M792603	B05	7926030	935
M792723	B05	7927238	935
M792954	B05	7929540	935
M793313	B05	7933130	935
M793868	B05	7938685	935
M794192	B05	7941920	935
M798248	B05	7982487	935
M798393	B05	7983937	935

M798624	B05	7986242	936
M799141	B05	7991410	936
M799930	B05	7999306	936
M800878	B05	8008786	936
M801446	B05	8014467	936
M801933	B05	8019337	936
M804249	B05	8042493	936
M804847	B05	8048473	936
M805087	B05	8050878	936
M805391	B05	8053917	936
M806675	B05	8066750	936
M807288	B05	8072880	936
M807640	B05	8076408	936
M808133	B05	8081331	936
M808270	B05	8082702	936
M808498	B05	8084984	936
M808777	B05	8087778	936
M810558	B05	8105585	936
M811589	B05	8115893	936
M811982	B05	8119822	936
M812460	B05	8124607	936
M813569	B05	8135695	936
M814531	B05	8145311	936
M814658	B05	8146587	937
M816391	B05	8163918	937
M820119	B05	8201197	937
M826743	B05	8267432	937
M828549	B05	8285493	937
M828738	B05	8287388	937
M829719	B05	8297191	937
M830580	B05	8305800	937
M857770	B05	8577703	938
M860379	B05	8603791	938
M861123	B05	8611235	938
M862558	B05	8625588	938
M862924	B05	8629247	938
M863499	B05	8634992	938
M863930	B05	8639307	938
M864521	B05	8645210	938
M865767	B05	8657671	938
M866147	B05	8661476	938
M866834	B05	8668340	938
M867591	B05	8675914	938
M868215	B05	8682156	938
M869403	B05	8694036	938
M869806	B05	8698065	938
M869956	B05	8699568	938

M870581	B05	8705818	938
M870723	B05	8707231	939
M870847	B05	8708471	939
M871247	B05	8712473	939
M873414	B05	8734148	939
M886368	B05	8863686	939
M886653	B05	8866534	939
M887864	B05	8878643	940
M888504	B05	8885041	940
M888803	B05	8888037	940
M889143	B05	8891430	940
M889244	B05	8892441	940
M889445	B05	8894454	940
M890276	B05	8902762	940
M890784	B05	8907842	940
M891236	B05	8912362	940
M892447	B05	8924472	940
M892961	B05	8929618	940
M893247	B05	8932477	940
M894177	B05	8941770	940
M894486	B05	8944862	940
M894814	B05	8948148	940
M895208	B05	8952083	940
M896221	B05	8962210	940
M898092	B05	8980929	940
M900815	B05	9008154	940
M902532	B05	9025320	940
M903604	B05	9036041	940
M904172	B05	9041720	941
M906854	B05	9068543	941
M907811	B05	9078119	941
M909352	B05	9093523	941
M957644	B05	9576448	942
M958925	B05	9589256	942
M963404	B05	9634042	942
M963413	B05	9634134	942
M964126	B05	9641267	942
M964549	B05	9645495	942
M964974	B05	9649745	942
M965566	B05	9655665	942
M965999	B05	9659992	942
M966200	B05	9662005	942
M966591	B05	9665915	942
M966873	B05	9668735	942
M967534	B05	9675343	942
M969670	B05	9696701	943
M969693	B05	9696938	943

M971650	B05	9716503	943
M972958	B05	9729586	943
M974510	B05	9745105	943
M974980	B05	9749803	943
M980630	B05	9806309	943
M985560	B05	9855601	943
M986273	B05	9862731	944
M986883	B05	9868834	944
M989949	B05	9899490	944
M103819	B05	10381975	945
M103824	B05	10382459	945
M103945	B05	10394589	946
M103953	B05	10395337	946
M104133	B05	10413340	946
M104161	B05	10416185	946
M104279	B05	10427902	946
M104343	B05	10434311	946
M104916	B05	10491678	946
M105492	B05	10549288	946
M106164	B05	10616447	947
M106229	B05	10622912	947
M106260	B05	10626016	947
M106380	B05	10638095	947
M106473	B05	10647355	947
M106737	B05	10673746	947
M106788	B05	10678898	947
M106986	B05	10698673	947
M110853	B05	11085340	948
M110892	B05	11089210	948
M111011	B05	11101134	949
M111163	B05	11116327	949
M111201	B05	11120135	949
M111284	B05	11128401	949
M111514	B05	11151481	949
M111535	B05	11153534	949
M119978	B05	11997810	950
M119989	B05	11998921	950
M120111	B05	12011123	950
M120211	B05	12021147	950
M122373	B05	12237331	951
M122517	B05	12251796	951
M122587	B05	12258784	951
M122930	B05	12293026	952
M122995	B05	12299557	952
M123096	B05	12309627	952
M123292	B05	12329236	952
M123508	B05	12350835	953

M123529	B05	12352978	953
M123762	B05	12376217	953
M126388	B05	12638826	954
M126429	B05	12642978	954
M126450	B05	12645041	954
M126574	B05	12657412	955
M126625	B05	12662544	955
M126692	B05	12669225	955
M126718	B05	12671822	955
M126748	B05	12674810	955
M126787	B05	12678735	956
M126823	B05	12682366	956
M127133	B05	12713330	957
M127180	B05	12718028	957
M127383	B05	12738308	958
M127430	B05	12743083	958
M127493	B05	12749373	958
M127639	B05	12763918	959
M127749	B05	12774900	959
M127761	B05	12776171	959
M128617	B05	12861707	960
M128625	B05	12862527	960
M128663	B05	12866341	961
M128673	B05	12867354	961
M128740	B05	12874060	961
M128932	B05	12893254	962
M129291	B05	12929146	962
M129489	B05	12948945	963
M129526	B05	12952602	963
M130180	B05	13018018	963
M130312	B05	13031239	963
M130445	B05	13044537	963
M130498	B05	13049855	964
M130602	B05	13060295	964
M130818	B05	13081824	965
M132068	B05	13206823	965
M132070	B05	13207017	966
M132121	B05	13212113	966
M132276	B05	13227677	966
M132308	B05	13230822	966
M132430	B05	13243059	967
M132486	B05	13248690	967
M132670	B05	13267095	968
M132712	B05	13271208	968
M133110	B05	13311096	969
M133126	B05	13312615	969
M133191	B05	13319169	969

M133407	B05	13340711	970
M133435	B05	13343553	970
M133506	B05	13350626	970
M133530	B05	13353099	970
M133543	B05	13354384	971
M133681	B05	13368172	971
M133816	B05	13381631	971
M133862	B05	13386209	971
M133891	B05	13389170	971
M133923	B05	13392309	971
M134117	B05	13411712	972
M134176	B05	13417640	972
M134273	B05	13427355	972
M134586	B05	13458668	972
M134769	B05	13476963	973
M134776	B05	13477664	973
M134798	B05	13479887	973
M134819	B05	13481994	973
M134879	B05	13487962	974
M134908	B05	13490807	974
M135023	B05	13502307	975
M135093	B05	13509352	975
M135127	B05	13512754	976
M135216	B05	13521661	976
M135276	B05	13527638	977
M136015	B05	13601570	977
M136119	B05	13611977	978
M136216	B05	13621650	978
M136415	B05	13641589	978
M136982	B05	13698264	979
M136997	B05	13699783	979
M137020	B05	13702025	979
M137055	B05	13705563	980
M137166	B05	13716613	980
M137231	B05	13723159	981
M137232	B05	13723279	981
M137339	B05	13733941	982
M137345	B05	13734514	982
M137443	B05	13744368	983
M137509	B05	13750962	983
M137702	B05	13770244	984
M137755	B05	13775510	984
M137904	B05	13790489	985
M137926	B05	13792698	985
M138333	B05	13833377	985
M138392	B05	13839229	985
M138459	B05	13845901	985

M138638	B05	13863868	985
M138762	B05	13876203	986
M138893	B05	13889361	986
M139226	B05	13922652	987
M139286	B05	13928688	987
M139393	B05	13939315	987
M139487	B05	13948702	987
M139654	B05	13965490	988
M139712	B05	13971229	988
M139736	B05	13973645	989
M139827	B05	13982732	989
M142296	B05	14229676	990
M142540	B05	14254069	990
M142751	B05	14275175	990
M142771	B05	14277189	990
M142782	B05	14278273	990
M142834	B05	14283446	990
M144532	B05	14453268	991
M144649	B05	14464902	991
M144803	B05	14480364	991
M144884	B05	14488471	992
M145129	B05	14512910	992
M149923	B05	14992384	993
M149998	B05	14999894	993
M150061	B05	15006174	993
M150197	B05	15019725	993
M150240	B05	15024053	993
M150252	B05	15025260	993
M150316	B05	15031662	993
M150327	B05	15032730	993
M150398	B05	15039892	993
M150611	B05	15061112	993
M150630	B05	15063070	993
M150665	B05	15066581	993
M150695	B05	15069570	993
M150837	B05	15083773	994
M150929	B05	15092938	994
M151206	B05	15120649	995
M151255	B05	15125507	995
M151278	B05	15127872	995
M151322	B05	15132257	995
M151487	B05	15148793	995
M152109	B05	15210952	995
M152113	B05	15211363	996
M152488	B05	15248847	996
M152506	B05	15250614	997
M152534	B05	15253491	997

M152615	B05	15261535	997
M152707	B05	15270744	997
M152716	B05	15271623	997
M152814	B05	15281417	997
M152907	B05	15290784	997
M152996	B05	15299642	997
M153482	B05	15348284	998
M153579	B05	15357997	998
M153668	B05	15366825	998
M153755	B05	15375529	999
M153779	B05	15377906	999
M154562	B05	15456213	1000
M154920	B05	15492024	1000
M155288	B05	15528832	1000
M155324	B05	15532402	1001
M155463	B05	15546302	1001
M155585	B05	15558557	1002
M155670	B05	15567010	1002
M155690	B05	15569035	1002
M155729	B05	15572994	1002
M155769	B05	15576962	1002
M155837	B05	15583795	1003
M155897	B05	15589712	1003
M156027	B05	15602748	1003
M156337	B05	15633727	1004
M156482	B05	15648225	1004
M156618	B05	15661857	1004
M156808	B05	15680861	1004
M157197	B05	15719744	1005
M157212	B05	15721217	1005
M157280	B05	15728071	1005
M157398	B05	15739885	1005
M157737	B05	15773747	1005
M159990	B05	15999026	1006
M160074	B05	16007412	1006
M160271	B05	16027193	1006
M160396	B05	16039652	1006
M160494	B05	16049409	1007
M160629	B05	16062998	1007
M160900	B05	16090016	1008
M161442	B05	16144291	1008
M163193	B05	16319388	1009
M163328	B05	16332824	1009
M163979	B05	16397910	1010
M164031	B05	16403126	1010
M164250	B05	16425040	1011
M164408	B05	16440875	1011

M164675	B05	16467587	1012
M164744	B05	16474432	1012
M164808	B05	16480802	1012
M164861	B05	16486113	1012
M165091	B05	16509153	1012
M165129	B05	16512939	1012
M165189	B05	16518969	1012
M165274	B05	16527444	1013
M165770	B05	16577011	1013
M168194	B05	16819476	1014
M168283	B05	16828398	1014
M168974	B05	16897481	1015
M169086	B05	16908612	1015
M169099	B05	16909919	1015
M169296	B05	16929647	1016
M169330	B05	16933089	1016
M169389	B05	16938973	1016
M169484	B05	16948447	1017
M169784	B05	16978455	1017
M170370	B05	17037047	1018
M170785	B05	17078559	1018
M170868	B05	17086859	1018
M170900	B05	17090060	1018
M199064	B05	19906491	1019
M199696	B05	19969677	1019
M199868	B05	19986879	1019
M199932	B05	19993268	1019
M200112	B05	20011242	1019
M200413	B05	20041396	1019
M200473	B05	20047351	1020
M200509	B05	20050959	1020
M200589	B05	20058967	1021
M200671	B05	20067129	1021
M200709	B05	20070930	1021
M200800	B05	20080034	1021
M200934	B05	20093497	1021
M209428	B05	20942811	1022
M209493	B05	20949356	1022
M209545	B05	20954558	1022
M210359	B05	21035998	1023
M210650	B05	21065092	1023
M211664	B05	21166454	1023
M222266	B05	22226670	1024
M222471	B05	22247160	1024
M223670	B05	22367027	1024
M250161	B05	25016114	1025
M250495	B05	25049539	1025

M262811	B05	26281150	1026
M263840	B05	26384038	1026
M279019	B05	27901946	1027
M279229	B05	27922935	1027
M305940	B05	30594057	1028
M306046	B05	30604697	1028
M324894	B05	32489455	1029
M325900	B05	32590009	1029
M330714	B05	33071469	1030
M331133	B05	33113310	1030
M331582	B05	33158204	1030
M331591	B05	33159102	1030
M331741	B05	33174177	1030
M331786	B05	33178612	1030
M331950	B05	33195009	1030
M332048	B05	33204814	1030
M337290	B05	33729003	1031
M337415	B05	33741567	1031
M338482	B05	33848289	1031
M344454	B05	34445482	1032
M344786	B05	34478636	1032
M346430	B05	34643015	1033
M347247	B05	34724799	1033
M377942	B05	37794271	1034
M378005	B05	37800511	1034
M379675	B05	37967557	1035
M379729	B05	37972912	1035
M379925	B05	37992579	1035
M380057	B05	38005736	1035
M380088	B05	38008853	1035
M380158	B05	38015842	1035
M380202	B05	38020200	1035
M380331	B05	38033173	1035
M380339	B05	38033958	1035
M380420	B05	38042081	1036
M380665	B05	38066509	1036
M380741	B05	38074193	1037
M380854	B05	38085471	1037
M380909	B05	38090918	1037
M380958	B05	38095854	1037
M381361	B05	38136172	1037
M381658	B05	38165864	1037
M381792	B05	38179204	1037
M382082	B05	38208206	1038
M382182	B05	38218253	1038
M382482	B05	38248204	1038
M390846	B05	39084687	1039

M391362	B05	39136200	1039
M391722	B05	39172208	1040
M391960	B05	39196045	1040
M397880	B05	39788091	1041
M398123	B05	39812300	1041
M398380	B05	39838054	1041
M398427	B05	39842765	1041
M398527	B05	39852769	1041
M398580	B05	39858046	1041
M398686	B05	39868667	1041
M398857	B05	39885730	1041
M398884	B05	39888431	1041
M399035	B05	39903588	1041
M402617	B05	40261735	1042
M402639	B05	40263918	1042
M402787	B05	40278718	1042
M402834	B05	40283433	1042
M402986	B05	40298633	1042
M403834	B05	40383498	1042
M403898	B05	40389866	1042
M404023	B05	40402356	1042
M404267	B05	40426761	1043
M404420	B05	40442054	1043
M404524	B05	40452409	1044
M404685	B05	40468554	1044
M404763	B05	40476330	1044
M404882	B05	40488279	1044
M404916	B05	40491689	1044
M405187	B05	40518758	1044
M406916	B05	40691614	1045
M407030	B05	40703002	1045
M407508	B05	40750882	1046
M407594	B05	40759425	1046
M408507	B05	40850723	1047
M408829	B05	40882935	1047
M408880	B05	40888083	1048
M409209	B05	40920905	1048
M413822	B05	41382273	1049
M415275	B05	41527536	1049
M428139	B05	42813967	1050
M428354	B05	42835419	1050
M428398	B05	42839836	1051
M428443	B05	42844359	1051
M428744	B05	42874435	1051
M428849	B05	42884936	1051
M429004	B05	42900478	1051
M429046	B05	42904665	1052

M429333	B05	42933346	1052
M429464	B05	42946457	1052
M429508	B05	42950804	1052
M429556	B05	42955647	1052
M429672	B05	42967286	1053
M429800	B05	42980049	1053
M429877	B05	42987770	1053
M430033	B05	43003359	1053
M430186	B05	43018695	1054
M430238	B05	43023898	1054
M430325	B05	43032575	1054
M430976	B05	43097696	1055
M430978	B05	43097859	1055
M431368	B05	43136869	1056
M431474	B05	43147467	1056
M431588	B05	43158893	1056
M431839	B05	43183985	1056
M431909	B05	43190990	1056
M431939	B05	43193942	1057
M431996	B05	43199644	1057
M432135	B05	43213555	1057
M432142	B05	43214260	1057
M432757	B05	43275703	1058
M432860	B05	43286052	1058
M432962	B05	43296274	1058
M434177	B05	43417777	1059
M434585	B05	43458522	1059
M434954	B05	43495429	1060
M435012	B05	43501227	1060
M435021	B05	43502180	1060
M435072	B05	43507229	1060
M435151	B05	43515185	1060
M435285	B05	43528581	1061
M435287	B05	43528790	1061
M435425	B05	43542566	1062
M435469	B05	43546934	1062
M435637	B05	43563771	1062
M435673	B05	43567323	1062
M435719	B05	43571969	1062
M435822	B05	43582230	1062
M435843	B05	43584391	1062
M435913	B05	43591308	1062
M436286	B05	43628610	1063
M436553	B05	43655350	1063
M436768	B05	43676804	1064
M436844	B05	43684444	1064
M436867	B05	43686784	1064

M436916	B05	43691642	1064
M437031	B05	43703147	1065
M437042	B05	43704266	1065
M437170	B05	43717017	1065
M437905	B05	43790584	1066
M438224	B05	43822455	1066
M438571	B05	43857162	1067
M438902	B05	43890207	1067
M439276	B05	43927610	1067
M439282	B05	43928299	1068
M439494	B05	43949430	1068
M440099	B05	44009981	1069
M440186	B05	44018662	1069
M440647	B05	44064726	1069
M441283	B05	44128345	1070
M441308	B05	44130834	1070
M444714	B05	44471474	1071
M444851	B05	44485145	1071
M445185	B05	44518509	1072
M445538	B05	44553867	1072
M459027	B05	45902777	1073
M460105	B05	46010583	1073
M460198	B05	46019860	1073
M460243	B05	46024327	1074
M461262	B05	46126280	1074
M461307	B05	46130722	1074
M461599	B05	46159914	1075
M461723	B05	46172381	1075
M461969	B05	46196932	1075
M462352	B05	46235291	1076
M462679	B05	46267909	1076
M462706	B05	46270624	1076
M462800	B05	46280061	1076
M464128	B05	46412883	1077
M464244	B05	46424407	1077
M464793	B05	46479334	1078
M464964	B05	46496454	1078
M465420	B05	46542013	1079
M465830	B05	46583033	1079
M466664	B05	46666403	1080
M466687	B05	46668761	1080
M466688	B05	46668824	1080
M466723	B05	46672303	1080
M466779	B05	46677919	1080
M466863	B05	46686385	1081
M466874	B05	46687495	1081
M467521	B05	46752178	1082

M467759	B05	46775985	1082
M467865	B05	46786535	1082
M468124	B05	46812417	1082
M468364	B05	46836480	1083
M468418	B05	46841832	1083
M468466	B05	46846661	1083
M469209	B05	46920959	1083
M469329	B05	46932972	1083
M469877	B05	46987739	1083
M475529	B05	47552968	1084
M475565	B05	47556561	1084
M475595	B05	47559576	1084
M475630	B05	47563057	1084
M475802	B05	47580255	1084
M477409	B05	47740948	1085
M478719	B05	47871959	1085
M478797	B05	47879747	1085
M478886	B05	47888687	1085
M478926	B05	47892673	1085
M478991	B05	47899199	1085
M479016	B05	47901649	1086
M479032	B05	47903264	1086
M479449	B05	47944909	1086
M479608	B05	47960845	1086
M479731	B05	47973112	1087
M479915	B05	47991570	1087
M479949	B05	47994969	1088
M480006	B05	48000614	1088
M480057	B05	48005776	1088
M480313	B05	48031343	1089
M480359	B05	48035952	1089
M481943	B05	48194370	1090
M482091	B05	48209158	1090
M488862	B05	48886234	1091
M488899	B05	48889985	1091
M489092	B05	48909217	1092
M489103	B05	48910344	1092
M489180	B05	48918040	1092
M489533	B05	48953332	1092
M490010	B05	49001056	1093
M490159	B05	49015939	1093
M490230	B05	49023095	1093
M490397	B05	49039726	1093
M493113	B05	49311335	1094
M493506	B05	49350631	1094
M493519	B05	49351963	1094
M493673	B05	49367382	1094

M493696	B05	49369600	1094
M497345	B05	49734576	1095
M497415	B05	49741538	1095
M497479	B05	49747943	1096
M497540	B05	49754071	1096
M498035	B05	49803582	1097
M498075	B05	49807519	1097
M498401	B05	49840193	1098
M498490	B05	49849066	1098
M498731	B05	49873198	1099
M499134	B05	49913462	1099
M499562	B05	49956270	1100
M500113	B05	50011366	1100
M500192	B05	50019216	1100
M500328	B05	50032892	1100
M500399	B05	50039905	1100
M500581	B05	50058170	1100
M506423	B05	50642337	1101
M506454	B05	50645477	1101
M508054	B05	50805497	1102
M508685	B05	50868598	1102
M510409	B05	51040974	1103
M511264	B05	51126461	1103
M511355	B05	51135527	1104
M511510	B05	51151071	1104
M511519	B05	51151964	1104
M511831	B05	51183142	1104
M511845	B05	51184575	1104
M511875	B05	51187586	1104
M511882	B05	51188230	1104
M511927	B05	51192776	1104
M511969	B05	51196936	1104
M511998	B05	51199893	1104
M512011	B05	51201121	1104
M512052	B05	51205229	1104
M512203	B05	51220359	1104
M512268	B05	51226823	1105
M512371	B05	51237160	1105
M512491	B05	51249119	1105
M512532	B05	51253280	1105
M627148	B06	627148	1106
M636755	B06	636755	1106
M705838	B06	705838	1106
M712580	B06	712580	1106
M720197	B06	720197	1106
M725211	B06	725211	1106
M727739	B06	727739	1106

M729251	B06	729251	1106
M748491	B06	748491	1107
M766433	B06	766433	1107
M776350	B06	776350	1107
M784283	B06	784283	1107
M791865	B06	791865	1108
M799031	B06	799031	1108
M806168	B06	806168	1108
M810501	B06	810501	1108
M813245	B06	813245	1108
M833456	B06	833456	1108
M842937	B06	842937	1108
M861222	B06	861222	1108
M944673	B06	944673	1108
M986855	B06	986855	1109
M999867	B06	999867	1109
M100385	B06	1003859	1109
M102779	B06	1027797	1109
M105907	B06	1059077	1109
M167053	B06	1670537	1110
M167149	B06	1671493	1110
M172686	B06	1726869	1111
M172719	B06	1727197	1111
M178588	B06	1785889	1111
M179090	B06	1790904	1111
M185686	B06	1856864	1111
M185738	B06	1857380	1111
M186071	B06	1860710	1111
M187298	B06	1872989	1111
M188658	B06	1886585	1111
M189471	B06	1894712	1112
M190347	B06	1903477	1112
M190830	B06	1908305	1112
M191038	B06	1910386	1113
M192938	B06	1929385	1113
M193159	B06	1931591	1113
M197193	B06	1971930	1114
M197968	B06	1979684	1114
M199756	B06	1997563	1114
M199982	B06	1999822	1114
M201303	B06	2013032	1114
M204705	B06	2047056	1115
M205358	B06	2053589	1115
M206968	B06	2069683	1115
M208025	B06	2080259	1115
M212574	B06	2125743	1115
M212887	B06	2128872	1115

M213627	B06	2136271	1116
M214012	B06	2140125	1116
M214328	B06	2143283	1117
M216637	B06	2166370	1117
M221260	B06	2212608	1117
M222653	B06	2226531	1117
M223145	B06	2231453	1117
M223229	B06	2232297	1117
M223635	B06	2236353	1117
M223871	B06	2238713	1117
M228890	B06	2288905	1118
M229066	B06	2290661	1118
M234781	B06	2347810	1119
M236262	B06	2362626	1119
M240417	B06	2404175	1120
M242348	B06	2423489	1120
M243607	B06	2436074	1121
M244122	B06	2441227	1121
M244426	B06	2444260	1121
M245259	B06	2452593	1121
M245445	B06	2454455	1121
M257570	B06	2575701	1121
M262240	B06	2622402	1122
M274464	B06	2744644	1122
M274489	B06	2744890	1122
M276859	B06	2768591	1122
M280389	B06	2803896	1123
M281863	B06	2818639	1123
M291468	B06	2914689	1123
M294272	B06	2942723	1124
M295478	B06	2954780	1124
M296143	B06	2961430	1125
M297122	B06	2971220	1125
M297447	B06	2974479	1125
M297872	B06	2978723	1125
M298185	B06	2981855	1125
M298282	B06	2982828	1125
M299089	B06	2990891	1126
M299982	B06	2999823	1126
M300608	B06	3006080	1127
M301302	B06	3013027	1127
M301569	B06	3015696	1127
M302304	B06	3023041	1128
M303636	B06	3036367	1128
M304197	B06	3041976	1128
M304345	B06	3043453	1128
M307697	B06	3076978	1128

M307798	B06	3077983	1128
M308064	B06	3080647	1128
M309138	B06	3091386	1128
M309555	B06	3095553	1128
M310654	B06	3106547	1128
M310940	B06	3109405	1128
M311442	B06	3114421	1128
M311663	B06	3116639	1128
M312277	B06	3122771	1128
M312385	B06	3123855	1128
M312581	B06	3125816	1128
M312874	B06	3128749	1128
M313688	B06	3136885	1128
M313967	B06	3139672	1128
M318291	B06	3182912	1128
M318702	B06	3187029	1129
M319002	B06	3190021	1129
M320066	B06	3200668	1129
M321430	B06	3214306	1129
M322806	B06	3228062	1129
M329303	B06	3293033	1129
M329506	B06	3295066	1129
M329938	B06	3299389	1129
M330419	B06	3304194	1129
M330780	B06	3307802	1129
M331189	B06	3311895	1130
M332497	B06	3324971	1130
M332917	B06	3329179	1131
M333796	B06	3337962	1131
M335238	B06	3352385	1131
M336465	B06	3364652	1132
M336612	B06	3366128	1132
M337320	B06	3373205	1132
M337507	B06	3375079	1132
M337711	B06	3377116	1133
M339212	B06	3392126	1133
M341012	B06	3410128	1133
M341700	B06	3417002	1133
M344899	B06	3448995	1133
M346700	B06	3467009	1133
M346826	B06	3468264	1133
M353552	B06	3535529	1133
M358175	B06	3581751	1134
M359903	B06	3599036	1134
M360612	B06	3606120	1134
M361362	B06	3613620	1134
M361889	B06	3618892	1134

M362053	B06	3620539	1134
M362254	B06	3622549	1134
M362635	B06	3626359	1134
M363071	B06	3630712	1134
M365080	B06	3650805	1134
M365884	B06	3658842	1134
M366075	B06	3660751	1134
M367141	B06	3671412	1135
M367419	B06	3674198	1135
M368403	B06	3684033	1136
M370420	B06	3704208	1136
M370894	B06	3708949	1136
M371066	B06	3710666	1136
M373030	B06	3730304	1136
M373989	B06	3739890	1136
M378362	B06	3783620	1137
M378537	B06	3785374	1137
M380354	B06	3803547	1137
M385816	B06	3858161	1138
M385837	B06	3858374	1138
M386700	B06	3867003	1138
M387071	B06	3870713	1139
M387607	B06	3876079	1139
M387758	B06	3877583	1139
M388191	B06	3881911	1139
M388736	B06	3887367	1139
M390823	B06	3908232	1140
M391382	B06	3913828	1140
M391624	B06	3916242	1141
M391923	B06	3919230	1141
M394012	B06	3940121	1141
M394281	B06	3942812	1141
M394430	B06	3944300	1141
M394767	B06	3947671	1141
M401705	B06	4017058	1141
M401854	B06	4018541	1141
M402437	B06	4024372	1141
M406167	B06	4061676	1141
M407447	B06	4074478	1141
M408553	B06	4085530	1142
M409161	B06	4091616	1142
M409436	B06	4094362	1143
M409700	B06	4097001	1143
M410883	B06	4108839	1144
M411273	B06	4112730	1144
M411564	B06	4115646	1144
M419654	B06	4196541	1144

M426080	B06	4260800	1144
M426530	B06	4265302	1144
M426544	B06	4265448	1144
M426789	B06	4267895	1144
M436566	B06	4365668	1145
M444232	B06	4442323	1145
M444829	B06	4448291	1145
M445228	B06	4452281	1145
M445372	B06	4453727	1145
M445979	B06	4459794	1145
M446665	B06	4466654	1145
M455940	B06	4559405	1146
M456042	B06	4560427	1146
M458349	B06	4583491	1146
M460151	B06	4601516	1147
M460958	B06	4609582	1147
M465015	B06	4650155	1148
M465693	B06	4656930	1148
M466227	B06	4662272	1148
M467023	B06	4670233	1149
M468142	B06	4681423	1149
M468948	B06	4689484	1149
M469795	B06	4697950	1149
M470352	B06	4703528	1149
M473282	B06	4732823	1150
M474053	B06	4740531	1150
M474406	B06	4744061	1150
M474797	B06	4747976	1150
M483684	B06	4836842	1150
M483979	B06	4839796	1150
M513169	B06	5131694	1151
M519520	B06	5195200	1151
M519664	B06	5196644	1151
M519894	B06	5198947	1151
M520233	B06	5202332	1151
M520347	B06	5203471	1151
M520692	B06	5206929	1151
M521520	B06	5215205	1151
M521644	B06	5216446	1151
M522745	B06	5227456	1151
M523168	B06	5231683	1151
M523554	B06	5235541	1151
M524189	B06	5241897	1151
M526826	B06	5268261	1152
M528163	B06	5281637	1152
M534508	B06	5345085	1152
M535055	B06	5350550	1152

M535617	B06	5356179	1152
M536017	B06	5360171	1153
M536403	B06	5364035	1153
M536445	B06	5364452	1154
M536871	B06	5368716	1154
M537015	B06	5370151	1154
M538302	B06	5383026	1155
M546619	B06	5466195	1155
M549889	B06	5498894	1155
M551317	B06	5513175	1155
M553906	B06	5539062	1155
M554274	B06	5542742	1155
M555092	B06	5550926	1156
M555413	B06	5554135	1156
M555461	B06	5554616	1156
M556654	B06	5566549	1156
M557017	B06	5570173	1157
M558106	B06	5581063	1157
M558255	B06	5582555	1158
M558628	B06	5586284	1158
M559362	B06	5593623	1158
M561358	B06	5613584	1158
M597656	B06	5976561	1159
M602218	B06	6022189	1159
M602531	B06	6025319	1159
M604777	B06	6047775	1160
M605844	B06	6058443	1160
M608294	B06	6082948	1160
M614037	B06	6140370	1161
M628600	B06	6286009	1161
M628643	B06	6286434	1161
M629128	B06	6291287	1161
M629436	B06	6294368	1161
M631761	B06	6317612	1162
M636144	B06	6361448	1162
M642829	B06	6428298	1163
M645720	B06	6457201	1163
M646575	B06	6465755	1163
M648370	B06	6483703	1163
M650892	B06	6508927	1163
M651527	B06	6515278	1163
M651867	B06	6518676	1163
M652328	B06	6523289	1163
M654458	B06	6544581	1163
M654921	B06	6549213	1163
M655992	B06	6559921	1163
M656545	B06	6565456	1163

M657510	B06	6575108	1164
M658493	B06	6584936	1164
M658739	B06	6587397	1164
M658846	B06	6588460	1164
M659740	B06	6597408	1164
M693351	B06	6933512	1165
M693681	B06	6936819	1165
M694582	B06	6945829	1165
M696101	B06	6961017	1166
M697018	B06	6970183	1166
M697615	B06	6976156	1166
M698204	B06	6982042	1166
M698787	B06	6987872	1166
M699110	B06	6991108	1166
M699726	B06	6997269	1166
M700734	B06	7007342	1166
M701286	B06	7012861	1166
M701589	B06	7015896	1166
M702068	B06	7020687	1166
M704038	B06	7040382	1167
M704849	B06	7048494	1167
M707810	B06	7078104	1168
M708376	B06	7083765	1168
M709850	B06	7098500	1168
M709984	B06	7099842	1168
M713607	B06	7136070	1168
M717358	B06	7173589	1169
M719781	B06	7197812	1169
M719982	B06	7199823	1169
M720343	B06	7203433	1169
M720557	B06	7205578	1169
M722273	B06	7222736	1169
M723078	B06	7230788	1169
M747961	B06	7479611	1170
M754421	B06	7544212	1170
M780089	B06	7800896	1171
M780404	B06	7804047	1171
M782667	B06	7826673	1171
M784269	B06	7842699	1171
M785278	B06	7852785	1171
M785703	B06	7857031	1171
M786089	B06	7860893	1172
M787377	B06	7873772	1172
M787971	B06	7879716	1172
M788508	B06	7885085	1172
M798244	B06	7982443	1173
M799255	B06	7992559	1173

M799475	B06	7994758	1173
M800978	B06	8009782	1174
M802668	B06	8026683	1174
M803419	B06	8034193	1174
M804468	B06	8044689	1174
M807466	B06	8074669	1174
M807710	B06	8077102	1174
M808092	B06	8080923	1174
M808582	B06	8085828	1174
M808628	B06	8086283	1174
M808921	B06	8089210	1174
M809279	B06	8092796	1174
M809899	B06	8098999	1174
M810140	B06	8101403	1174
M810390	B06	8103903	1174
M810776	B06	8107768	1174
M811173	B06	8111737	1174
M811391	B06	8113918	1174
M811631	B06	8116319	1174
M811797	B06	8117970	1174
M812040	B06	8120403	1174
M818295	B06	8182956	1175
M819148	B06	8191485	1175
M834079	B06	8340798	1175
M873651	B06	8736510	1176
M875455	B06	8754558	1176
M875770	B06	8757706	1176
M876292	B06	8762923	1176
M877293	B06	8772931	1177
M878123	B06	8781235	1177
M879176	B06	8791767	1177
M887610	B06	8876108	1177
M892697	B06	8926970	1177
M121911	B06	12191107	1178
M122205	B06	12220597	1178
M145154	B06	14515405	1179
M145360	B06	14536098	1179
M145640	B06	14564080	1180
M146966	B06	14696636	1180
M156569	B06	15656982	1181
M157687	B06	15768733	1181
M203093	B06	20309392	1182
M203151	B06	20315123	1182
M203233	B06	20323391	1182
M203858	B06	20385801	1182
M203897	B06	20389702	1182
M203924	B06	20392441	1182

M203963	B06	20396340	1183
M203989	B06	20398999	1183
M204778	B06	20477887	1183
M214583	B06	21458383	1184
M215125	B06	21512561	1184
M215341	B06	21534123	1184
M215620	B06	21562035	1184
M216020	B06	21602054	1185
M216147	B06	21614775	1185
M221316	B06	22131626	1186
M221756	B06	22175633	1186
M222375	B06	22237512	1187
M222475	B06	22247561	1187
M226446	B06	22644690	1188
M226638	B06	22663820	1188
M226747	B06	22674724	1188
M226783	B06	22678354	1188
M226937	B06	22693726	1188
M226946	B06	22694645	1188
M228364	B06	22836408	1189
M229233	B06	22923329	1189
M229553	B06	22955397	1189
M230853	B06	23085300	1190
M231193	B06	23119316	1190
M231606	B06	23160665	1190
M232127	B06	23212781	1191
M232520	B06	23252078	1191
M232743	B06	23274313	1191
M232828	B06	23282884	1191
M232922	B06	23292258	1192
M232983	B06	23298360	1192
M233156	B06	23315655	1192
M256090	B06	25609010	1193
M256802	B06	25680246	1193
M281418	B06	28141809	1194
M281425	B06	28142536	1194
M281952	B06	28195235	1195
M281957	B06	28195709	1195
M282057	B06	28205703	1196
M282111	B06	28211169	1196
M282196	B06	28219626	1196
M282245	B06	28224500	1196
M282644	B06	28264485	1196
M286189	B06	28618982	1197
M286311	B06	28631142	1197
M286330	B06	28633057	1197
M286346	B06	28634649	1198

M286459	B06	28645988	1198
M286565	B06	28656599	1198
M286611	B06	28661136	1198
M286674	B06	28667424	1198
M286730	B06	28673062	1198
M287015	B06	28701516	1199
M287046	B06	28704633	1199
M287159	B06	28715943	1199
M287561	B06	28756145	1200
M287604	B06	28760416	1200
M287668	B06	28766854	1201
M287774	B06	28777455	1201
M287815	B06	28781548	1202
M287865	B06	28786525	1202
M287920	B06	28792020	1202
M287937	B06	28793764	1202
M287974	B06	28797416	1202
M288006	B06	28800616	1202
M288123	B06	28812376	1202
M288225	B06	28822578	1202
M288431	B06	28843110	1202
M288468	B06	28846859	1202
M288492	B06	28849284	1203
M288596	B06	28859668	1203
M288658	B06	28865883	1203
M288838	B06	28883874	1204
M288961	B06	28896140	1204
M288998	B06	28899879	1204
M289044	B06	28904442	1204
M289090	B06	28909079	1204
M289253	B06	28925351	1204
M289416	B06	28941696	1204
M290133	B06	29013309	1205
M290144	B06	29014451	1205
M290265	B06	29026522	1206
M290339	B06	29033953	1206
M290352	B06	29035248	1206
M290379	B06	29037964	1207
M290395	B06	29039501	1207
M290449	B06	29044934	1207
M290544	B06	29054440	1207
M290575	B06	29057516	1207
M290960	B06	29096066	1207
M291417	B06	29141710	1207
M291450	B06	29145071	1207
M291478	B06	29147822	1207
M291510	B06	29151039	1207

M293038	B06	29303857	1208
M293045	B06	29304580	1208
M293218	B06	29321805	1208
M293344	B06	29334482	1208
M296031	B06	29603192	1209
M296083	B06	29608315	1209
M296112	B06	29611237	1210
M296145	B06	29614549	1210
M296201	B06	29620199	1210
M296249	B06	29624900	1210
M296543	B06	29654346	1211
M296568	B06	29656888	1211
M297563	B06	29756374	1211
M311836	B06	31183688	1212
M312229	B06	31222982	1212
M312253	B06	31225349	1212
M313816	B06	31381657	1213
M313837	B06	31383732	1213
M313888	B06	31388836	1213
M313913	B06	31391397	1213
M314043	B06	31404318	1213
M314059	B06	31405972	1213
M314494	B06	31449412	1213
M314680	B06	31468005	1214
M314754	B06	31475424	1214
M314895	B06	31489563	1215
M314987	B06	31498795	1215
M315003	B06	31500381	1215
M315139	B06	31513931	1216
M315148	B06	31514843	1216
M315192	B06	31519247	1217
M315296	B06	31529606	1217
M315886	B06	31588600	1217
M316100	B06	31610005	1217
M316267	B06	31626789	1218
M316346	B06	31634655	1218
M318588	B06	31858864	1219
M318651	B06	31865157	1219
M318673	B06	31867383	1219
M321871	B06	32187108	1220
M321986	B06	32198652	1220
M322121	B06	32212183	1220
M322151	B06	32215145	1220
M322230	B06	32223004	1220
M322238	B06	32223811	1220
M322273	B06	32227307	1220
M322315	B06	32231518	1221

M322379	B06	32237928	1221
M322461	B06	32246160	1222
M322498	B06	32249802	1222
M322566	B06	32256667	1223
M322597	B06	32259732	1223
M322680	B06	32268062	1223
M322841	B06	32284173	1223
M323046	B06	32304699	1223
M323254	B06	32325401	1223
M323304	B06	32330430	1223
M323346	B06	32334678	1223
M323380	B06	32338089	1223
M323442	B06	32344238	1223
M323454	B06	32345428	1224
M323482	B06	32348230	1224
M323508	B06	32350847	1224
M324894	B06	32489472	1225
M324954	B06	32495410	1225
M324986	B06	32498658	1225
M325068	B06	32506805	1225
M325131	B06	32513164	1225
M325549	B06	32554954	1225
M325610	B06	32561098	1225
M328277	B06	32827754	1226
M328428	B06	32842826	1226
M328456	B06	32845663	1226
M328528	B06	32852834	1227
M328547	B06	32854701	1227
M329302	B06	32930222	1227
M332878	B06	33287828	1228
M332951	B06	33295187	1228
M337533	B06	33753316	1229
M337786	B06	33778678	1229
M338834	B06	33883467	1230
M338969	B06	33896929	1230
M339055	B06	33905590	1230
M339185	B06	33918504	1231
M339405	B06	33940590	1231
M339470	B06	33947026	1231
M341802	B06	34180286	1232
M341809	B06	34180945	1232
M341837	B06	34183723	1233
M342988	B06	34298866	1233
M343057	B06	34305703	1234
M343132	B06	34313245	1234
M343435	B06	34343528	1234
M343684	B06	34368458	1235

M343708	B06	34370845	1235
M343762	B06	34376247	1235
M346498	B06	34649849	1236
M347545	B06	34754574	1236
M347608	B06	34760882	1236
M347657	B06	34765721	1237
M347692	B06	34769224	1237
M348295	B06	34829560	1238
M348380	B06	34838020	1238
M348521	B06	34852143	1239
M348548	B06	34854826	1239
M348579	B06	34857991	1239
M348627	B06	34862702	1239
M348949	B06	34894969	1240
M348995	B06	34899582	1240
M349056	B06	34905698	1240
M349237	B06	34923700	1240
M349247	B06	34924710	1240
M349298	B06	34929868	1240
M349526	B06	34952666	1241
M349641	B06	34964161	1241
M349779	B06	34977923	1241
M349899	B06	34989952	1241
M354401	B06	35440174	1242
M354437	B06	35443778	1242
M357132	B06	35713245	1243
M357898	B06	35789815	1243
M358323	B06	35832364	1243
M360847	B06	36084763	1244
M360876	B06	36087628	1244
M360911	B06	36091190	1244
M360951	B06	36095161	1244
M361188	B06	36118845	1244
M361200	B06	36120054	1245
M361245	B06	36124534	1245
M361281	B06	36128172	1246
M361316	B06	36131619	1246
M362574	B06	36257472	1246
M363143	B06	36314323	1247
M364710	B06	36471047	1247
M364784	B06	36478463	1248
M364812	B06	36481292	1248
M364836	B06	36483670	1248
M364849	B06	36484942	1248
M364896	B06	36489681	1248
M364938	B06	36493840	1248
M365050	B06	36505028	1248

M365398	B06	36539805	1248
M365428	B06	36542845	1248
M365529	B06	36552939	1248
M365603	B06	36560367	1248
M365652	B06	36565219	1248
M365710	B06	36571003	1248
M367563	B06	36756334	1249
M367605	B06	36760566	1249
M368602	B06	36860251	1249
M368605	B06	36860561	1249
M368657	B06	36865721	1249
M368763	B06	36876367	1249
M368881	B06	36888188	1250
M368905	B06	36890588	1250
M368943	B06	36894310	1250
M370067	B06	37006770	1250
M370438	B06	37043803	1250
M370464	B06	37046444	1250
M370499	B06	37049970	1251
M370602	B06	37060252	1251
M370612	B06	37061248	1251
M370630	B06	37063019	1251
M370799	B06	37079911	1251
M371031	B06	37103180	1251
M371071	B06	37107157	1251
M371523	B06	37152363	1251
M371553	B06	37155395	1251
M371588	B06	37158863	1251
M371646	B06	37164646	1251
M371682	B06	37168286	1251
M371789	B06	37178941	1251
M371858	B06	37185878	1251
M371915	B06	37191500	1251
M371950	B06	37195003	1251
M371978	B06	37197857	1251
M372028	B06	37202825	1251
M372049	B06	37204944	1251
M372095	B06	37209592	1251
M372198	B06	37219821	1252
M372353	B06	37235365	1252
M372643	B06	37264382	1252
M372656	B06	37265649	1252
M373337	B06	37333759	1252
M373413	B06	37341359	1252
M373813	B06	37381335	1253
M373930	B06	37393009	1253
M374193	B06	37419305	1253

M374228	B06	37422898	1253
M374270	B06	37427006	1253
M374318	B06	37431857	1253
M374364	B06	37436488	1253
M374438	B06	37443869	1253
M374558	B06	37455856	1253
M374577	B06	37457754	1253
M374626	B06	37462690	1253
M374684	B06	37468459	1253
M374750	B06	37475001	1253
M374802	B06	37480229	1253
M374829	B06	37482914	1253
M374876	B06	37487603	1253
M375248	B06	37524865	1253
M375402	B06	37540260	1253
M375474	B06	37547406	1254
M375479	B06	37547996	1254
M375557	B06	37555771	1254
M375988	B06	37598813	1254
M378901	B06	37890194	1255
M378953	B06	37895398	1255
M379508	B06	37950852	1255
M379666	B06	37966600	1256
M380844	B06	38084492	1256
M381250	B06	38125095	1256
M381277	B06	38127795	1257
M381335	B06	38133512	1257
M381382	B06	38138298	1257
M381580	B06	38158059	1257
M381656	B06	38165622	1257
M381737	B06	38173752	1257
M381761	B06	38176164	1257
M381787	B06	38178776	1257
M381847	B06	38184755	1257
M381858	B06	38185807	1257
M381951	B06	38195111	1257
M384391	B06	38439147	1258
M384517	B06	38451743	1258
M384566	B06	38456612	1259
M384610	B06	38461081	1259
M384673	B06	38467306	1259
M385020	B06	38502018	1259
M385033	B06	38503353	1259
M385050	B06	38505053	1259
M385127	B06	38512758	1259
M385212	B06	38521215	1259
M385256	B06	38525601	1259

M385286	B06	38528667	1259
M385361	B06	38536182	1259
M385486	B06	38548601	1259
M385525	B06	38552501	1259
M385693	B06	38569361	1259
M386053	B06	38605394	1259
M386083	B06	38608385	1259
M386153	B06	38615325	1259
M386165	B06	38616539	1259
M386187	B06	38618720	1260
M386202	B06	38620203	1260
M387149	B06	38714905	1260
M387252	B06	38725278	1260
M387302	B06	38730205	1261
M387385	B06	38738548	1261
M387416	B06	38741639	1261
M387454	B06	38745459	1261
M387734	B06	38773416	1261
M387775	B06	38777591	1261
M387916	B06	38791649	1262
M387983	B06	38798304	1262
M388105	B06	38810535	1262
M388196	B06	38819693	1262
M388338	B06	38833822	1262
M389491	B06	38949108	1262
M390333	B06	39033319	1263
M390416	B06	39041607	1263
M390497	B06	39049713	1263
M390560	B06	39056057	1263
M390698	B06	39069888	1263
M391396	B06	39139656	1263
M391459	B06	39145980	1263
M391504	B06	39150494	1263
M391553	B06	39155385	1263
M391697	B06	39169792	1263
M391932	B06	39193256	1263
M391966	B06	39196651	1264
M391998	B06	39199846	1264
M392056	B06	39205616	1264
M392491	B06	39249135	1264
M392503	B06	39250307	1264
M392581	B06	39258158	1264
M392670	B06	39267019	1264
M392747	B06	39274719	1264
M392789	B06	39278925	1264
M392907	B06	39290775	1264
M392964	B06	39296466	1264

M393149	B06	39314942	1265
M393154	B06	39315431	1265
M393184	B06	39318498	1266
M393226	B06	39322663	1266
M393371	B06	39337105	1266
M393398	B06	39339868	1266
M393745	B06	39374580	1267
M393847	B06	39384782	1267
M394038	B06	39403858	1268
M394100	B06	39410037	1268
M394249	B06	39424942	1268
M394395	B06	39439514	1268
M394444	B06	39444416	1268
M394510	B06	39451079	1268
M394650	B06	39465063	1268
M394685	B06	39468579	1268
M394735	B06	39473502	1268
M394798	B06	39479839	1268
M394909	B06	39490969	1268
M394943	B06	39494322	1268
M394952	B06	39495280	1268
M394989	B06	39498942	1268
M395017	B06	39501790	1268
M395042	B06	39504274	1268
M395079	B06	39507969	1268
M395138	B06	39513804	1268
M395241	B06	39524141	1268
M395270	B06	39527058	1269
M395293	B06	39529304	1269
M396664	B06	39666424	1269
M396709	B06	39670996	1269
M396742	B06	39674264	1269
M396765	B06	39676512	1269
M396784	B06	39678420	1269
M396822	B06	39682279	1269
M396857	B06	39685724	1269
M396877	B06	39687787	1270
M396895	B06	39689568	1270
M396926	B06	39692632	1270
M396941	B06	39694147	1270
M396968	B06	39696843	1270
M397010	B06	39701050	1270
M397029	B06	39702912	1270
M397063	B06	39706352	1270
M397108	B06	39710825	1270
M397166	B06	39716678	1270
M397244	B06	39724491	1270

M397335	B06	39733579	1270
M397352	B06	39735216	1270
M397408	B06	39740881	1270
M397490	B06	39749078	1270
M397851	B06	39785148	1270
M28465	B07	28465	1271
M32839	B07	32839	1271
M69473	B07	69473	1272
M78569	B07	78569	1272
M80394	B07	80394	1272
M82669	B07	82669	1272
M90572	B07	90572	1273
M113410	B07	113410	1273
M121245	B07	121245	1273
M149538	B07	149538	1273
M149912	B07	149912	1273
M153395	B07	153395	1273
M161315	B07	161315	1274
M166812	B07	166812	1274
M236551	B07	236551	1275
M245328	B07	245328	1275
M329155	B07	329155	1276
M332544	B07	332544	1276
M347358	B07	347358	1277
M405104	B07	405104	1277
M408904	B07	408904	1278
M418435	B07	418435	1278
M490036	B07	490036	1279
M499864	B07	499864	1279
M514152	B07	514152	1279
M550983	B07	550983	1279
M612253	B07	612253	1279
M619876	B07	619876	1279
M635743	B07	635743	1279
M668992	B07	668992	1280
M684011	B07	684011	1280
M692437	B07	692437	1281
M717362	B07	717362	1281
M739480	B07	739480	1282
M747281	B07	747281	1282
M776657	B07	776657	1283
M777497	B07	777497	1283
M796106	B07	796106	1283
M823752	B07	823752	1283
M826702	B07	826702	1283
M834153	B07	834153	1283
M835499	B07	835499	1283

M839482	B07	839482	1283
M846906	B07	846906	1283
M887289	B07	887289	1284
M896074	B07	896074	1284
M921648	B07	921648	1285
M924431	B07	924431	1285
M927151	B07	927151	1285
M944660	B07	944660	1286
M951722	B07	951722	1286
M954285	B07	954285	1287
M957900	B07	957900	1287
M960857	B07	960857	1287
M964664	B07	964664	1287
M969573	B07	969573	1287
M975294	B07	975294	1287
M984485	B07	984485	1287
M138967	B07	1389679	1288
M139430	B07	1394306	1288
M139557	B07	1395576	1288
M164411	B07	1644118	1289
M165131	B07	1651312	1289
M184850	B07	1848505	1290
M185117	B07	1851174	1290
M200725	B07	2007254	1290
M201794	B07	2017947	1291
M202001	B07	2020011	1291
M202431	B07	2024313	1291
M218517	B07	2185175	1292
M218994	B07	2189942	1292
M219254	B07	2192549	1292
M228357	B07	2283575	1293
M228709	B07	2287097	1293
M229378	B07	2293789	1294
M245288	B07	2452880	1294
M245610	B07	2456105	1295
M245871	B07	2458716	1295
M246185	B07	2461857	1295
M250395	B07	2503950	1295
M252299	B07	2522992	1295
M253349	B07	2533499	1295
M255150	B07	2551508	1295
M256819	B07	2568196	1296
M257355	B07	2573551	1296
M257740	B07	2577402	1297
M257995	B07	2579953	1297
M258766	B07	2587660	1297
M261382	B07	2613822	1298

M262042	B07	2620423	1298
M262525	B07	2625251	1298
M274418	B07	2744186	1299
M274773	B07	2747731	1299
M275708	B07	2757087	1300
M276738	B07	2767383	1300
M279001	B07	2790013	1301
M279328	B07	2793286	1301
M280938	B07	2809385	1301
M285772	B07	2857721	1302
M287564	B07	2875647	1302
M287618	B07	2876182	1303
M288222	B07	2882226	1303
M289032	B07	2890321	1304
M289260	B07	2892609	1304
M289736	B07	2897369	1304
M290730	B07	2907300	1305
M291283	B07	2912838	1305
M291425	B07	2914255	1305
M292166	B07	2921662	1305
M292541	B07	2925419	1305
M298993	B07	2989939	1306
M299232	B07	2992321	1306
M299772	B07	2997727	1306
M300636	B07	3006361	1306
M301340	B07	3013409	1306
M304139	B07	3041391	1307
M305717	B07	3057173	1307
M306186	B07	3061861	1307
M306698	B07	3066981	1307
M307028	B07	3070284	1307
M307353	B07	3073536	1307
M309569	B07	3095695	1307
M310543	B07	3105431	1308
M311553	B07	3115534	1308
M313446	B07	3134469	1308
M313856	B07	3138560	1308
M315527	B07	3155275	1308
M316127	B07	3161270	1308
M317779	B07	3177797	1309
M318607	B07	3186072	1309
M320918	B07	3209185	1310
M321141	B07	3211415	1310
M321770	B07	3217705	1311
M321820	B07	3218205	1311
M345840	B07	3458408	1312
M346431	B07	3464312	1312

M347865	B07	3478653	1312
M352116	B07	3521161	1312
M352403	B07	3524033	1312
M353633	B07	3536333	1312
M354127	B07	3541273	1312
M354197	B07	3541979	1312
M354472	B07	3544729	1313
M355507	B07	3555072	1313
M356384	B07	3563846	1313
M357471	B07	3574719	1314
M357983	B07	3579830	1314
M358412	B07	3584128	1314
M360424	B07	3604244	1314
M361079	B07	3610790	1314
M361519	B07	3615196	1314
M362078	B07	3620788	1314
M364867	B07	3648675	1315
M365151	B07	3651510	1315
M366137	B07	3661374	1315
M368492	B07	3684925	1316
M368847	B07	3688477	1316
M368860	B07	3688602	1316
M369138	B07	3691385	1317
M370343	B07	3703432	1317
M372329	B07	3723297	1318
M372669	B07	3726699	1318
M373262	B07	3732620	1319
M373360	B07	3733606	1319
M373519	B07	3735196	1319
M374622	B07	3746223	1319
M385861	B07	3858613	1320
M387528	B07	3875285	1320
M387684	B07	3876847	1320
M389607	B07	3896075	1321
M389925	B07	3899258	1321
M390776	B07	3907760	1321
M396125	B07	3961259	1321
M399073	B07	3990739	1321
M414272	B07	4142720	1322
M416507	B07	4165071	1322
M416898	B07	4168981	1322
M419059	B07	4190599	1323
M424226	B07	4242261	1323
M424554	B07	4245547	1324
M424969	B07	4249695	1324
M426046	B07	4260462	1325
M426346	B07	4263463	1325

M426825	B07	4268250	1325
M427946	B07	4279463	1325
M430658	B07	4306582	1325
M431433	B07	4314335	1325
M433016	B07	4330165	1326
M433362	B07	4333627	1326
M436428	B07	4364280	1326
M437536	B07	4375360	1326
M438435	B07	4384358	1326
M440027	B07	4400270	1326
M441052	B07	4410527	1327
M441792	B07	4417927	1327
M442598	B07	4425980	1328
M444676	B07	4446769	1328
M452458	B07	4524587	1329
M453474	B07	4534748	1329
M455800	B07	4558002	1329
M456502	B07	4565029	1329
M457369	B07	4573699	1329
M458460	B07	4584605	1329
M458873	B07	4588731	1329
M485842	B07	4858426	1330
M487289	B07	4872894	1330
M488037	B07	4880373	1330
M488171	B07	4881713	1330
M488616	B07	4886169	1331
M488801	B07	4888013	1331
M488990	B07	4889900	1331
M490357	B07	4903573	1332
M490786	B07	4907864	1332
M492934	B07	4929349	1332
M493099	B07	4930994	1332
M495355	B07	4953550	1332
M495565	B07	4955654	1332
M497018	B07	4970189	1332
M497448	B07	4974482	1332
M497714	B07	4977141	1333
M509842	B07	5098427	1333
M512011	B07	5120118	1334
M515061	B07	5150614	1334
M515902	B07	5159029	1334
M522024	B07	5220242	1335
M523687	B07	5236878	1335
M524304	B07	5243049	1335
M524869	B07	5248694	1336
M525355	B07	5253550	1336
M525517	B07	5255173	1336

M526701	B07	5267010	1336
M528523	B07	5285231	1337
M529013	B07	5290135	1337
M533693	B07	5336932	1338
M534422	B07	5344225	1338
M534622	B07	5346221	1338
M537502	B07	5375029	1339
M538502	B07	5385024	1339
M541991	B07	5419919	1340
M542983	B07	5429834	1340
M543297	B07	5432975	1340
M546045	B07	5460458	1340
M548947	B07	5489473	1340
M550396	B07	5503966	1340
M558791	B07	5587919	1341
M559364	B07	5593641	1341
M559710	B07	5597100	1341
M559929	B07	5599294	1341
M560727	B07	5607279	1341
M562999	B07	5629997	1341
M564590	B07	5645901	1341
M567793	B07	5677933	1341
M581661	B07	5816614	1342
M581831	B07	5818319	1342
M586954	B07	5869541	1342
M587305	B07	5873056	1342
M589627	B07	5896271	1342
M593858	B07	5938587	1342
M598675	B07	5986754	1343
M604007	B07	6040076	1343
M613521	B07	6135212	1343
M616090	B07	6160904	1344
M616804	B07	6168049	1344
M618221	B07	6182213	1344
M618964	B07	6189644	1344
M619564	B07	6195646	1344
M619943	B07	6199437	1344
M620964	B07	6209642	1344
M621343	B07	6213437	1344
M622000	B07	6220009	1344
M625029	B07	6250296	1345
M625573	B07	6255733	1345
M627050	B07	6270507	1346
M627496	B07	6274968	1346
M635091	B07	6350914	1347
M635353	B07	6353539	1347
M644754	B07	6447544	1347

M645349	B07	6453499	1347
M645943	B07	6459432	1347
M647533	B07	6475334	1347
M717394	B07	7173948	1348
M718215	B07	7182157	1348
M719304	B07	7193048	1348
M719811	B07	7198114	1348
M720141	B07	7201411	1348
M773851	B07	7738513	1349
M773979	B07	7739795	1349
M776418	B07	7764181	1350
M785174	B07	7851747	1350
M795467	B07	7954677	1351
M796036	B07	7960368	1351
M797912	B07	7979125	1351
M798389	B07	7983899	1351
M799021	B07	7990215	1351
M799197	B07	7991972	1351
M799443	B07	7994434	1351
M799792	B07	7997921	1351
M800411	B07	8004111	1351
M800944	B07	8009440	1351
M803478	B07	8034787	1352
M804145	B07	8041457	1352
M815726	B07	8157260	1352
M833494	B07	8334946	1353
M834645	B07	8346459	1353
M834876	B07	8348762	1353
M880451	B07	8804511	1354
M881748	B07	8817488	1354
M882542	B07	8825429	1354
M883180	B07	8831804	1354
M883471	B07	8834714	1354
M886530	B07	8865302	1354
M888177	B07	8881778	1354
M888775	B07	8887756	1354
M890283	B07	8902837	1354
M891033	B07	8910338	1354
M894008	B07	8940081	1354
M894462	B07	8944627	1354
M896040	B07	8960409	1355
M900901	B07	9009012	1355
M904617	B07	9046170	1355
M905666	B07	9056660	1355
M907614	B07	9076141	1355
M909004	B07	9090041	1356
M909366	B07	9093663	1356

M910851	B07	9108511	1357
M911388	B07	9113881	1357
M912052	B07	9120526	1357
M912916	B07	9129160	1357
M913033	B07	9130337	1357
M924982	B07	9249826	1357
M925120	B07	9251209	1357
M962064	B07	9620644	1358
M962666	B07	9626667	1358
M963392	B07	9633923	1358
M963533	B07	9635330	1358
M963594	B07	9635946	1358
M966367	B07	9663678	1358
M966735	B07	9667355	1358
M967371	B07	9673714	1359
M982815	B07	9828155	1359
M983518	B07	9835186	1360
M984716	B07	9847165	1360
M986908	B07	9869086	1360
M987395	B07	9873951	1360
M987490	B07	9874908	1360
M987856	B07	9878563	1360
M989178	B07	9891787	1360
M998950	B07	9989508	1360
M100907	B07	10090715	1361
M100969	B07	10096933	1361
M101117	B07	10111765	1361
M101999	B07	10199983	1362
M102022	B07	10202247	1362
M102161	B07	10216164	1362
M103157	B07	10315737	1362
M103466	B07	10346664	1362
M103522	B07	10352268	1363
M103754	B07	10375422	1363
M103808	B07	10380820	1363
M103824	B07	10382457	1363
M103849	B07	10384901	1363
M103906	B07	10390648	1363
M103951	B07	10395153	1363
M103996	B07	10399691	1363
M104047	B07	10404754	1363
M104076	B07	10407663	1363
M104106	B07	10410658	1363
M104251	B07	10425151	1363
M104256	B07	10425668	1363
M104408	B07	10440870	1363
M104567	B07	10456762	1364

M104582	B07	10458261	1364
M104679	B07	10467973	1364
M105688	B07	10568831	1364
M105742	B07	10574212	1364
M105808	B07	10580897	1364
M105907	B07	10590717	1364
M105951	B07	10595132	1364
M106057	B07	10605701	1364
M106161	B07	10616163	1364
M106505	B07	10650500	1365
M106521	B07	10652124	1365
M109028	B07	10902883	1366
M109045	B07	10904507	1366
M109073	B07	10907333	1366
M109290	B07	10929033	1366
M109316	B07	10931651	1366
M109529	B07	10952963	1366
M109543	B07	10954303	1366
M109607	B07	10960752	1366
M109688	B07	10968894	1366
M109735	B07	10973556	1366
M109825	B07	10982534	1366
M110146	B07	11014660	1366
M110303	B07	11030326	1366
M110334	B07	11033441	1366
M110405	B07	11040571	1366
M110553	B07	11055329	1366
M110572	B07	11057229	1366
M110683	B07	11068381	1367
M110700	B07	11070086	1367
M110737	B07	11073709	1367
M110860	B07	11086004	1367
M110932	B07	11093266	1367
M110957	B07	11095740	1367
M110988	B07	11098834	1367
M111137	B07	11113733	1367
M111177	B07	11117727	1367
M111487	B07	11148714	1367
M111592	B07	11159269	1367
M111682	B07	11168206	1367
M111823	B07	11182326	1367
M112074	B07	11207412	1368
M112657	B07	11265736	1368
M112725	B07	11272585	1368
M112739	B07	11273996	1368
M112816	B07	11281634	1368
M112866	B07	11286688	1368

M112964	B07	11296423	1368
M113231	B07	11323186	1368
M116073	B07	11607341	1369
M116077	B07	11607718	1369
M116142	B07	11614228	1369
M116787	B07	11678738	1369
M116792	B07	11679296	1369
M116930	B07	11693008	1369
M117186	B07	11718692	1370
M117323	B07	11732392	1370
M117460	B07	11746071	1370
M117494	B07	11749470	1370
M117644	B07	11764435	1370
M118069	B07	11806929	1371
M118090	B07	11809097	1371
M119074	B07	11907439	1372
M120564	B07	12056431	1372
M123828	B07	12382897	1373
M123878	B07	12387854	1373
M123910	B07	12391072	1373
M123971	B07	12397149	1373
M124014	B07	12401457	1373
M124106	B07	12410651	1374
M124119	B07	12411928	1374
M124492	B07	12449285	1375
M124747	B07	12474743	1375
M124885	B07	12488519	1376
M124924	B07	12492401	1376
M125010	B07	12501087	1376
M125059	B07	12505936	1376
M125224	B07	12522458	1376
M125603	B07	12560324	1376
M125689	B07	12568910	1377
M125701	B07	12570149	1377
M125815	B07	12581586	1377
M125854	B07	12585456	1377
M125937	B07	12593782	1377
M130313	B07	13031360	1378
M130321	B07	13032117	1378
M131173	B07	13117339	1378
M131733	B07	13173392	1378
M131882	B07	13188246	1378
M132080	B07	13208012	1379
M132552	B07	13255297	1379
M132752	B07	13275269	1380
M132795	B07	13279502	1380
M132804	B07	13280473	1380

M132987	B07	13298784	1381
M133195	B07	13319570	1381
M133462	B07	13346230	1382
M133615	B07	13361598	1382
M133933	B07	13393388	1383
M134057	B07	13405718	1383
M134605	B07	13460542	1384
M134777	B07	13477779	1384
M135266	B07	13526624	1385
M135334	B07	13533440	1385
M135369	B07	13536932	1385
M135521	B07	13552189	1385
M135748	B07	13574887	1386
M135749	B07	13574967	1386
M136202	B07	13620201	1387
M136354	B07	13635456	1387
M136782	B07	13678201	1387
M137536	B07	13753632	1388
M137646	B07	13764641	1388
M137732	B07	13773252	1388
M137826	B07	13782626	1389
M137907	B07	13790772	1389
M138140	B07	13814008	1389
M138201	B07	13820139	1390
M138447	B07	13844766	1390
M138838	B07	13883806	1391
M138900	B07	13890042	1391
M139025	B07	13902514	1391
M139067	B07	13906702	1392
M139184	B07	13918465	1392
M139297	B07	13929715	1392
M139436	B07	13943671	1392
M140434	B07	14043418	1393
M140558	B07	14055815	1393
M140643	B07	14064382	1393
M140843	B07	14084392	1393
M140960	B07	14096078	1394
M141034	B07	14103469	1394
M141152	B07	14115264	1394
M141216	B07	14121635	1395
M141280	B07	14128042	1395
M141299	B07	14129952	1395
M141340	B07	14134089	1396
M141366	B07	14136614	1396
M141581	B07	14158134	1397
M141633	B07	14163354	1397
M141651	B07	14165179	1398

M141675	B07	14167588	1398
M141907	B07	14190734	1399
M141916	B07	14191683	1399
M141956	B07	14195629	1399
M142704	B07	14270427	1400
M142895	B07	14289565	1400
M143208	B07	14320800	1401
M143257	B07	14325719	1401
M143560	B07	14356051	1402
M144016	B07	14401683	1402
M144072	B07	14407236	1402
M144099	B07	14409976	1403
M144132	B07	14413289	1403
M144171	B07	14417128	1403
M144293	B07	14429375	1404
M144296	B07	14429651	1404
M144348	B07	14434855	1405
M144382	B07	14438226	1405
M144396	B07	14439677	1405
M144439	B07	14443910	1405
M144452	B07	14445209	1405
M144997	B07	14499710	1406
M145089	B07	14508930	1406
M145113	B07	14511360	1406
M145431	B07	14543176	1407
M145599	B07	14559957	1407
M145652	B07	14565234	1408
M145698	B07	14569860	1408
M146050	B07	14605048	1408
M146102	B07	14610245	1408
M146209	B07	14620942	1408
M146303	B07	14630339	1408
M146412	B07	14641264	1408
M146643	B07	14664349	1409
M146768	B07	14676891	1409
M146782	B07	14678291	1409
M148079	B07	14807931	1410
M148081	B07	14808170	1410
M162592	B07	16259212	1411
M163802	B07	16380265	1411
M163854	B07	16385485	1411
M163876	B07	16387674	1411
M163893	B07	16389355	1411
M163936	B07	16393601	1411
M163960	B07	16396023	1411
M164031	B07	16403191	1411
M164160	B07	16416064	1411

M164171	B07	16417199	1411
M164515	B07	16451555	1412
M164551	B07	16455135	1412
M165113	B07	16511341	1413
M165371	B07	16537145	1413
M165666	B07	16566661	1414
M166009	B07	16600932	1414
M166328	B07	16632877	1414
M166330	B07	16633083	1414
M166452	B07	16645210	1414
M166743	B07	16674335	1414
M166916	B07	16691656	1415
M167157	B07	16715708	1415
M167207	B07	16720709	1415
M167309	B07	16730973	1415
M167388	B07	16738820	1415
M167699	B07	16769955	1416
M168030	B07	16803017	1416
M168292	B07	16829242	1416
M168329	B07	16832920	1416
M168510	B07	16851087	1416
M168558	B07	16855851	1416
M168655	B07	16865555	1416
M169455	B07	16945549	1417
M169467	B07	16946771	1417
M176196	B07	17619688	1418
M177209	B07	17720902	1418
M177489	B07	17748998	1418
M177524	B07	17752431	1418
M177737	B07	17773728	1418
M177797	B07	17779767	1419
M177930	B07	17793047	1419
M178125	B07	17812561	1419
M185722	B07	18572234	1420
M185955	B07	18595514	1420
M186025	B07	18602509	1420
M186055	B07	18605546	1421
M186146	B07	18614673	1421
M186227	B07	18622731	1421
M186255	B07	18625582	1421
M186289	B07	18628978	1421
M186462	B07	18646207	1421
M187409	B07	18740956	1421
M187521	B07	18752106	1421
M187599	B07	18759951	1421
M187648	B07	18764812	1421
M188172	B07	18817226	1422

M188194	B07	18819418	1422
M188530	B07	18853064	1422
M188575	B07	18857516	1422
M188657	B07	18865754	1423
M188692	B07	18869261	1423
M188780	B07	18878013	1424
M188844	B07	18884442	1424
M188893	B07	18889349	1424
M189390	B07	18939019	1424
M194052	B07	19405247	1425
M194073	B07	19407382	1425
M194342	B07	19434263	1425
M194370	B07	19437093	1425
M194609	B07	19460986	1426
M194989	B07	19498943	1426
M195352	B07	19535283	1427
M195458	B07	19545848	1427
M199268	B07	19926846	1428
M199281	B07	19928147	1428
M199308	B07	19930835	1428
M199347	B07	19934771	1428
M199399	B07	19939992	1429
M199546	B07	19954637	1429
M199651	B07	19965171	1430
M199731	B07	19973111	1430
M199772	B07	19977205	1431
M199822	B07	19982234	1431
M200012	B07	20001204	1431
M200135	B07	20013599	1431
M220136	B07	22013632	1432
M220194	B07	22019498	1432
M220285	B07	22028505	1432
M220409	B07	22040928	1433
M220482	B07	22048268	1433
M221058	B07	22105894	1434
M221341	B07	22134136	1434
M221533	B07	22153301	1435
M221560	B07	22156087	1435
M221741	B07	22174119	1435
M221898	B07	22189848	1435
M222082	B07	22208256	1435
M222179	B07	22217940	1435
M222242	B07	22224231	1435
M222409	B07	22240902	1435
M222455	B07	22245528	1435
M222505	B07	22250538	1435
M222726	B07	22272661	1435

M222903	B07	22290305	1435
M223086	B07	22308679	1435
M224171	B07	22417108	1436
M224226	B07	22422614	1436
M224472	B07	22447228	1437
M224709	B07	22470923	1437
M224958	B07	22495815	1437
M226234	B07	22623449	1438
M226327	B07	22632702	1438
M226843	B07	22684390	1439
M226991	B07	22699159	1439
M227217	B07	22721772	1439
M227557	B07	22755774	1439
M228023	B07	22802362	1440
M228366	B07	22836628	1440
M228393	B07	22839381	1440
M228555	B07	22855580	1440
M228970	B07	22897089	1440
M229853	B07	22985367	1441
M229970	B07	22997056	1441
M231345	B07	23134557	1441
M233648	B07	23364813	1442
M233951	B07	23395155	1442
M234185	B07	23418558	1442
M234743	B07	23474307	1442
M235244	B07	23524495	1442
M236106	B07	23610656	1443
M236133	B07	23613342	1443
M236348	B07	23634892	1443
M236365	B07	23636568	1443
M236393	B07	23639395	1443
M236717	B07	23671740	1443
M236890	B07	23689085	1443
M237021	B07	23702191	1443
M238771	B07	23877164	1444
M240135	B07	24013512	1444
M240411	B07	24041170	1445
M241287	B07	24128771	1445
M243555	B07	24355519	1446
M244020	B07	24402049	1446
M244161	B07	24416151	1447
M244267	B07	24426748	1447
M244345	B07	24434503	1448
M244524	B07	24452406	1448
M244661	B07	24466185	1448
M245077	B07	24507778	1448
M245453	B07	24545305	1448

M245634	B07	24563473	1448
M245814	B07	24581421	1448
M245830	B07	24583069	1448
M245924	B07	24592451	1448
M246065	B07	24606538	1449
M246152	B07	24615253	1449
M247431	B07	24743180	1449
M247635	B07	24763505	1449
M248074	B07	24807426	1450
M248113	B07	24811307	1450
M248185	B07	24818501	1450
M248212	B07	24821260	1450
M248281	B07	24828107	1450
M248353	B07	24835388	1450
M248502	B07	24850267	1450
M248577	B07	24857700	1451
M248848	B07	24884893	1451
M248910	B07	24891069	1451
M249159	B07	24915947	1451
M249362	B07	24936287	1451
M249528	B07	24952804	1451
M249638	B07	24963812	1451
M249762	B07	24976212	1451
M249854	B07	24985409	1451
M249877	B07	24987714	1451
M250079	B07	25007980	1451
M250173	B07	25017377	1451
M250596	B07	25059617	1452
M250637	B07	25063793	1452
M251000	B07	25100098	1452
M251098	B07	25109886	1453
M251114	B07	25111463	1453
M251344	B07	25134447	1453
M251401	B07	25140154	1453
M251481	B07	25148185	1453
M252275	B07	25227598	1454
M252392	B07	25239205	1454
M253727	B07	25372764	1455
M254383	B07	25438374	1455
M254566	B07	25456641	1455
M255265	B07	25526524	1455
M255426	B07	25542671	1456
M255648	B07	25564895	1456
M256504	B07	25650458	1457
M256721	B07	25672161	1457
M256797	B07	25679789	1458
M256958	B07	25695860	1458

M257376	B07	25737621	1458
M257866	B07	25786640	1458
M258168	B07	25816855	1458
M258365	B07	25836597	1458
M258385	B07	25838519	1458
M258658	B07	25865828	1459
M258716	B07	25871615	1459
M258919	B07	25891999	1460
M259070	B07	25907031	1460
M259388	B07	25938889	1460
M259822	B07	25982214	1460
M259859	B07	25985993	1460
M260013	B07	26001317	1460
M260382	B07	26038232	1460
M260608	B07	26060839	1461
M260915	B07	26091584	1461
M261025	B07	26102527	1461
M261079	B07	26107973	1462
M261228	B07	26122853	1462
M261576	B07	26157699	1462
M261739	B07	26173949	1462
M261876	B07	26187655	1462
M261938	B07	26193813	1462
M262007	B07	26200703	1462
M262219	B07	26221949	1462
M262648	B07	26264812	1462
M262956	B07	26295602	1463
M263236	B07	26323600	1463
M263583	B07	26358342	1464
M263676	B07	26367620	1464
M263718	B07	26371853	1464
M263843	B07	26384379	1464
M264727	B07	26472722	1465
M264904	B07	26490411	1465
M265517	B07	26551713	1465
M266284	B07	26628405	1466
M266532	B07	26653240	1466
M266829	B07	26682963	1466
M266891	B07	26689194	1466
M266946	B07	26694681	1466
M267488	B07	26748836	1466
M267681	B07	26768184	1466
M267846	B07	26784699	1466
M267968	B07	26796805	1467
M268079	B07	26807908	1467
M268176	B07	26817656	1467
M268337	B07	26833712	1467

M269766	B07	26976672	1468
M269999	B07	26999919	1468
M270254	B07	27025466	1468
M270258	B07	27025887	1468
M270339	B07	27033912	1468
M270364	B07	27036469	1468
M270914	B07	27091434	1468
M274210	B07	27421040	1469
M274641	B07	27464119	1469
M274768	B07	27476862	1469
M274946	B07	27494614	1469
M275101	B07	27510148	1469
M275224	B07	27522473	1469
M276364	B07	27636453	1470
M276753	B07	27675377	1470
M276867	B07	27686794	1470
M276963	B07	27696325	1470
M277374	B07	27737413	1470
M277412	B07	27741278	1470
M277944	B07	27794412	1470
M277983	B07	27798399	1471
M278066	B07	27806664	1471
M278436	B07	27843689	1472
M278998	B07	27899870	1472
M279744	B07	27974412	1473
M280640	B07	28064024	1473
M281142	B07	28114210	1473
M283720	B07	28372098	1474
M284642	B07	28464265	1474
M284896	B07	28489658	1474
M285025	B07	28502515	1474
M285102	B07	28510205	1474
M285170	B07	28517059	1474
M286887	B07	28688749	1475
M286935	B07	28693540	1475
M286991	B07	28699182	1475
M287516	B07	28751672	1475
M288454	B07	28845439	1475
M288894	B07	28889443	1476
M289309	B07	28930917	1476
M289719	B07	28971966	1476
M290548	B07	29054822	1477
M291835	B07	29183587	1477
M292017	B07	29201718	1477
M294111	B07	29411190	1478
M294228	B07	29422837	1478
M294270	B07	29427064	1478

M294829	B07	29482905	1478
M295302	B07	29530246	1479
M296037	B07	29603748	1479
M296285	B07	29628570	1479
M296756	B07	29675610	1479
M296811	B07	29681103	1479
M297515	B07	29751536	1480
M297913	B07	29791338	1480
M298299	B07	29829989	1480
M299043	B07	29904301	1480
M300228	B07	30022816	1481
M300433	B07	30043328	1481
M300929	B07	30092920	1481
M301085	B07	30108594	1481
M301263	B07	30126354	1481
M301527	B07	30152701	1481
M301537	B07	30153767	1481
M303617	B07	30361755	1482
M304869	B07	30486924	1482
M305104	B07	30510485	1482
M305179	B07	30517906	1482
M305206	B07	30520630	1482
M305255	B07	30525555	1483
M305348	B07	30534860	1483
M305469	B07	30546920	1483
M305573	B07	30557343	1484
M305579	B07	30557989	1484
M305917	B07	30591759	1484
M306261	B07	30626135	1484
M306509	B07	30650941	1484
M306724	B07	30672411	1484
M307119	B07	30711919	1484
M308891	B07	30889160	1485
M310008	B07	31000819	1485
M311282	B07	31128252	1486
M312469	B07	31246995	1486
M312673	B07	31267384	1486
M312693	B07	31269352	1486
M313258	B07	31325852	1487
M313431	B07	31343113	1487
M313512	B07	31351274	1488
M313586	B07	31358635	1488
M313882	B07	31388258	1488
M314170	B07	31417079	1488
M314719	B07	31471931	1488
M315055	B07	31505537	1488
M315319	B07	31531980	1489

M316117	B07	31611702	1489
M319520	B07	31952043	1490
M320254	B07	32025496	1490
M320905	B07	32090504	1490
M321763	B07	32176325	1491
M322923	B07	32292314	1491
M323775	B07	32377523	1492
M325265	B07	32526585	1492
M325576	B07	32557616	1493
M326027	B07	32602721	1493
M327496	B07	32749694	1494
M328876	B07	32887688	1494
M329973	B07	32997333	1495
M331533	B07	33153329	1495
M334326	B07	33432684	1496
M335057	B07	33505791	1496
M335401	B07	33540194	1497
M335632	B07	33563219	1497
M335979	B07	33597959	1497
M336195	B07	33619544	1497
M336852	B07	33685264	1497
M337833	B07	33783360	1498
M338253	B07	33825318	1498
M339356	B07	33935619	1498
M340648	B07	34064890	1499
M341360	B07	34136017	1499
M342112	B07	34211281	1499
M342233	B07	34223395	1499
M342330	B07	34233026	1500
M342536	B07	34253632	1500
M342686	B07	34268602	1500
M342933	B07	34293399	1500
M343394	B07	34339424	1500
M343800	B07	34380073	1500
M343881	B07	34388162	1500
M344182	B07	34418225	1501
M344285	B07	34428500	1501
M346480	B07	34648028	1502
M346966	B07	34696604	1502
M347211	B07	34721163	1502
M347606	B07	34760652	1502
M347875	B07	34787570	1502
M347970	B07	34797004	1502
M348063	B07	34806397	1502
M349035	B07	34903569	1503
M349446	B07	34944669	1503
M349524	B07	34952462	1503

M349992	B07	34999235	1504
M350059	B07	35005985	1504
M350375	B07	35037530	1504
M350588	B07	35058860	1505
M351494	B07	35149435	1505
M351800	B07	35180085	1505
M352026	B07	35202629	1505
M352105	B07	35210521	1505
M358460	B07	35846019	1506
M358686	B07	35868600	1506
M358868	B07	35886800	1506
M358946	B07	35894652	1507
M358959	B07	35895908	1507
M359193	B07	35919366	1507
M359607	B07	35960799	1507
M359904	B07	35990485	1508
M360421	B07	36042144	1508
M360912	B07	36091214	1509
M361365	B07	36136513	1509
M361422	B07	36142212	1510
M361765	B07	36176587	1510
M362119	B07	36211916	1511
M362190	B07	36219061	1511
M362476	B07	36247671	1511
M362741	B07	36274189	1512
M363092	B07	36309200	1512
M363102	B07	36310266	1512
M363229	B07	36322946	1512
M363402	B07	36340286	1512
M363496	B07	36349641	1512
M363682	B07	36368215	1512
M363781	B07	36378117	1512
M363965	B07	36396582	1512
M364032	B07	36403229	1512
M364291	B07	36429100	1512
M364335	B07	36433512	1512
M364395	B07	36439544	1513
M364566	B07	36456649	1513
M364858	B07	36485875	1513
M365219	B07	36521972	1513
M365334	B07	36533475	1513
M365466	B07	36546681	1513
M365700	B07	36570018	1513
M365772	B07	36577292	1514
M366068	B07	36606896	1514
M366141	B07	36614197	1514
M366279	B07	36627946	1514

M366571	B07	36657162	1514
M366904	B07	36690458	1514
M367174	B07	36717494	1514
M367316	B07	36731647	1514
M367368	B07	36736899	1514
M367670	B07	36767097	1515
M367752	B07	36775283	1515
M368109	B07	36810946	1516
M368269	B07	36826988	1516
M368573	B07	36857397	1516
M368971	B07	36897122	1516
M369503	B07	36950300	1516
M369826	B07	36982693	1517
M370329	B07	37032943	1517
M371228	B07	37122804	1518
M371410	B07	37141015	1518
M371491	B07	37149131	1518
M372511	B07	37251190	1518
M372732	B07	37273288	1518
M373158	B07	37315865	1519
M373196	B07	37319621	1519
M373225	B07	37322564	1519
M373484	B07	37348456	1519
M374317	B07	37431703	1520
M374370	B07	37437010	1520
M374855	B07	37485572	1520
M375406	B07	37540680	1520
M375601	B07	37560165	1520
M375866	B07	37586614	1520
M375991	B07	37599152	1521
M376081	B07	37608115	1521
M376252	B07	37625244	1521
M376549	B07	37654974	1522
M376828	B07	37682863	1522
M376901	B07	37690121	1522
M377032	B07	37703271	1522
M377103	B07	37710315	1522
M377148	B07	37714850	1523
M377167	B07	37716741	1523
M377211	B07	37721143	1523
M377387	B07	37738747	1523
M377481	B07	37748143	1523
M377578	B07	37757823	1523
M377600	B07	37760023	1523
M377726	B07	37772688	1523
M377933	B07	37793329	1523
M378098	B07	37809830	1523

M378423	B07	37842319	1523
M378526	B07	37852607	1523
M378743	B07	37874373	1523
M379063	B07	37906384	1524
M379301	B07	37930199	1524
M379436	B07	37943642	1524
M379531	B07	37953127	1525
M379633	B07	37963331	1525
M379940	B07	37994032	1525
M380363	B07	38036328	1525
M380737	B07	38073768	1525
M381036	B07	38103645	1525
M381038	B07	38103884	1525
M381891	B07	38189169	1526
M381984	B07	38198414	1526
M382047	B07	38204710	1526
M382202	B07	38220204	1527
M382376	B07	38237601	1527
M382538	B07	38253879	1527
M382608	B07	38260848	1527
M382665	B07	38266585	1527
M382818	B07	38281822	1527
M382976	B07	38297674	1527
M383018	B07	38301866	1527
M383118	B07	38311873	1527
M383295	B07	38329584	1527
M383665	B07	38366584	1527
M383671	B07	38367113	1527
M383765	B07	38376514	1527
M384081	B07	38408159	1528
M384373	B07	38437375	1528
M384495	B07	38449589	1528
M384759	B07	38475936	1528
M384915	B07	38491516	1528
M384960	B07	38496011	1528
M386097	B07	38609726	1529
M386647	B07	38664784	1529
M386850	B07	38685085	1529
M387070	B07	38707046	1529
M387502	B07	38750201	1529
M387801	B07	38780127	1530
M388180	B07	38818094	1530
M388283	B07	38828300	1530
M388664	B07	38866415	1531
M388832	B07	38883275	1531
M388945	B07	38894581	1531
M389287	B07	38928725	1532

M389421	B07	38942161	1532
M389852	B07	38985286	1533
M389899	B07	38989918	1533
M389965	B07	38996533	1533
M390020	B07	39002033	1533
M390101	B07	39010106	1533
M390501	B07	39050115	1534
M391146	B07	39114685	1534
M391561	B07	39156169	1534
M391623	B07	39162344	1535
M391787	B07	39178737	1535
M391925	B07	39192548	1535
M391999	B07	39199935	1535
M392062	B07	39206241	1535
M392242	B07	39224208	1535
M392992	B07	39299217	1536
M393025	B07	39302593	1536
M393081	B07	39308177	1536
M398337	B07	39833731	1537
M398537	B07	39853776	1537
M420718	B07	42071854	1538
M420957	B07	42095776	1538
M421303	B07	42130323	1538
M421497	B07	42149765	1538
M421638	B07	42163883	1539
M421898	B07	42189846	1539
M422041	B07	42204113	1540
M422397	B07	42239793	1540
M422485	B07	42248580	1540
M422529	B07	42252962	1540
M422626	B07	42262610	1540
M422956	B07	42295647	1540
M423286	B07	42328690	1541
M423513	B07	42351367	1541
M423725	B07	42372522	1542
M424154	B07	42415403	1542
M424608	B07	42460842	1542
M425180	B07	42518037	1543
M425210	B07	42521032	1543
M425223	B07	42522350	1543
M425236	B07	42523614	1543
M425270	B07	42527085	1543
M425303	B07	42530384	1543
M425356	B07	42535629	1543
M425474	B07	42547432	1543
M425608	B07	42560894	1544
M425627	B07	42562724	1544

M425831	B07	42583170	1544
M426269	B07	42626907	1544
M426399	B07	42639995	1544
M433702	B07	43370206	1545
M434310	B07	43431034	1545
M94994	B08	94994	1546
M97645	B08	97645	1546
M114290	B08	114290	1547
M258208	B08	258208	1547
M259553	B08	259553	1547
M262234	B08	262234	1547
M266832	B08	266832	1548
M269361	B08	269361	1548
M270765	B08	270765	1548
M273211	B08	273211	1548
M302688	B08	302688	1548
M306257	B08	306257	1548
M319093	B08	319093	1548
M378999	B08	378999	1548
M384542	B08	384542	1548
M394136	B08	394136	1548
M397755	B08	397755	1548
M398832	B08	398832	1548
M412707	B08	412707	1548
M414618	B08	414618	1548
M420444	B08	420444	1548
M426142	B08	426142	1548
M427814	B08	427814	1549
M436263	B08	436263	1549
M442260	B08	442260	1549
M446469	B08	446469	1549
M463601	B08	463601	1550
M466297	B08	466297	1550
M478308	B08	478308	1550
M481752	B08	481752	1550
M486868	B08	486868	1550
M493023	B08	493023	1550
M497735	B08	497735	1550
M501459	B08	501459	1550
M505244	B08	505244	1550
M507838	B08	507838	1550
M686267	B08	686267	1551
M743751	B08	743751	1551
M746359	B08	746359	1551
M750732	B08	750732	1551
M752274	B08	752274	1551
M754260	B08	754260	1552

M768255	B08	768255	1552
M770964	B08	770964	1552
M913709	B08	913709	1552
M916338	B08	916338	1553
M919744	B08	919744	1553
M101433	B08	1014332	1553
M101920	B08	1019205	1553
M102173	B08	1021738	1554
M102666	B08	1026661	1554
M103044	B08	1030446	1554
M103414	B08	1034149	1554
M103800	B08	1038003	1554
M104272	B08	1042722	1554
M104421	B08	1044212	1554
M105366	B08	1053662	1554
M105573	B08	1055734	1554
M106038	B08	1060388	1554
M106647	B08	1066476	1554
M106894	B08	1068943	1554
M111338	B08	1113387	1554
M111645	B08	1116450	1554
M116667	B08	1166672	1554
M117672	B08	1176720	1554
M118012	B08	1180128	1554
M120000	B08	1200001	1555
M123675	B08	1236759	1555
M125179	B08	1251793	1556
M125444	B08	1254440	1556
M128297	B08	1282974	1556
M130886	B08	1308868	1556
M130992	B08	1309924	1556
M131305	B08	1313050	1556
M139324	B08	1393240	1556
M139426	B08	1394263	1556
M139567	B08	1395670	1556
M140551	B08	1405516	1556
M141078	B08	1410781	1556
M141499	B08	1414999	1557
M156233	B08	1562334	1557
M156363	B08	1563635	1557
M156751	B08	1567515	1557
M157162	B08	1571626	1557
M157466	B08	1574662	1557
M157836	B08	1578367	1558
M158255	B08	1582553	1558
M167291	B08	1672913	1559
M178870	B08	1788704	1559

M180967	B08	1809670	1559
M187487	B08	1874870	1560
M188595	B08	1885957	1560
M188980	B08	1889808	1560
M189888	B08	1898882	1560
M190127	B08	1901279	1560
M190832	B08	1908321	1560
M191496	B08	1914965	1560
M191649	B08	1916495	1560
M192088	B08	1920880	1560
M192500	B08	1925005	1560
M193937	B08	1939371	1561
M194102	B08	1941023	1561
M194616	B08	1946165	1561
M197905	B08	1979050	1561
M198056	B08	1980563	1561
M198434	B08	1984342	1561
M199995	B08	1999953	1561
M200555	B08	2005554	1561
M204117	B08	2041176	1561
M209794	B08	2097948	1561
M211595	B08	2115952	1562
M212179	B08	2121797	1562
M220913	B08	2209132	1563
M220995	B08	2209958	1563
M221397	B08	2213970	1563
M225746	B08	2257460	1563
M227790	B08	2277905	1563
M229242	B08	2292429	1563
M229503	B08	2295031	1563
M231015	B08	2310155	1564
M231742	B08	2317429	1564
M233562	B08	2335626	1565
M233752	B08	2337528	1565
M235729	B08	2357295	1565
M239091	B08	2390911	1565
M239675	B08	2396759	1565
M239882	B08	2398827	1565
M240995	B08	2409957	1565
M242498	B08	2424987	1565
M243425	B08	2434259	1565
M250829	B08	2508295	1566
M252338	B08	2523382	1566
M253020	B08	2530207	1566
M253081	B08	2530811	1566
M254353	B08	2543533	1566
M254501	B08	2545017	1566

M254845	B08	2548458	1567
M255003	B08	2550034	1567
M255386	B08	2553867	1567
M255518	B08	2555181	1567
M255865	B08	2558658	1567
M256260	B08	2562607	1567
M261656	B08	2616562	1567
M264521	B08	2645219	1567
M266016	B08	2660165	1567
M266542	B08	2665423	1567
M267279	B08	2672796	1567
M270714	B08	2707145	1567
M272347	B08	2723479	1568
M273957	B08	2739573	1568
M274740	B08	2747406	1568
M275332	B08	2753326	1568
M276169	B08	2761697	1568
M277060	B08	2770604	1568
M284474	B08	2844746	1568
M288232	B08	2882327	1568
M288532	B08	2885324	1569
M291469	B08	2914691	1569
M292623	B08	2926238	1570
M292695	B08	2926959	1570
M292900	B08	2929004	1570
M293818	B08	2938189	1570
M294183	B08	2941837	1570
M294372	B08	2943726	1570
M294597	B08	2945970	1570
M294887	B08	2948870	1570
M295756	B08	2957566	1570
M296020	B08	2960207	1570
M296343	B08	2963438	1570
M298992	B08	2989927	1570
M299242	B08	2992424	1570
M299704	B08	2997041	1570
M300155	B08	3001559	1570
M301489	B08	3014898	1570
M304594	B08	3045941	1570
M304960	B08	3049605	1570
M307413	B08	3074139	1570
M308828	B08	3088282	1571
M309186	B08	3091862	1571
M309444	B08	3094442	1571
M310669	B08	3106691	1571
M311056	B08	3110569	1571
M311299	B08	3112992	1571

M311719	B08	3117199	1571
M312401	B08	3124011	1571
M312663	B08	3126631	1571
M312989	B08	3129892	1571
M313438	B08	3134389	1571
M313514	B08	3135143	1571
M314219	B08	3142193	1571
M314683	B08	3146839	1571
M314834	B08	3148340	1571
M315101	B08	3151017	1571
M315718	B08	3157189	1571
M315869	B08	3158693	1571
M316033	B08	3160331	1571
M317396	B08	3173968	1571
M318073	B08	3180736	1571
M318631	B08	3186311	1571
M319651	B08	3196514	1572
M327225	B08	3272250	1572
M329340	B08	3293400	1572
M329557	B08	3295576	1572
M330090	B08	3300900	1572
M331131	B08	3311317	1572
M331476	B08	3314765	1572
M331750	B08	3317506	1572
M332359	B08	3323590	1572
M332828	B08	3328282	1572
M333251	B08	3332515	1572
M333517	B08	3335171	1572
M333817	B08	3338175	1572
M334926	B08	3349268	1572
M334999	B08	3349992	1572
M335618	B08	3356189	1572
M336921	B08	3369219	1573
M337365	B08	3373650	1573
M337571	B08	3375712	1573
M338061	B08	3380619	1573
M340818	B08	3408180	1574
M341126	B08	3411267	1574
M341163	B08	3411630	1574
M341629	B08	3416295	1575
M342341	B08	3423417	1575
M342531	B08	3425313	1575
M344928	B08	3449284	1575
M345662	B08	3456625	1575
M347565	B08	3475658	1575
M350679	B08	3506797	1575
M353384	B08	3533844	1575

M356823	B08	3568230	1575
M357377	B08	3573779	1575
M358004	B08	3580046	1576
M358209	B08	3582096	1576
M358477	B08	3584772	1576
M359103	B08	3591032	1576
M359958	B08	3599583	1576
M362714	B08	3627146	1576
M371875	B08	3718757	1576
M372406	B08	3724063	1576
M373018	B08	3730188	1576
M373983	B08	3739834	1576
M375419	B08	3754196	1577
M377034	B08	3770340	1577
M377276	B08	3772766	1577
M378028	B08	3780282	1577
M378516	B08	3785165	1577
M378637	B08	3786372	1577
M378748	B08	3787480	1577
M379924	B08	3799240	1578
M380139	B08	3801394	1578
M380482	B08	3804821	1578
M381850	B08	3818507	1578
M382043	B08	3820437	1578
M382213	B08	3822134	1578
M382424	B08	3824240	1578
M383340	B08	3833400	1578
M383909	B08	3839093	1578
M384482	B08	3844827	1578
M387860	B08	3878601	1578
M388189	B08	3881894	1578
M389331	B08	3893314	1578
M409318	B08	4093186	1579
M410102	B08	4101023	1579
M410383	B08	4103836	1579
M410517	B08	4105171	1580
M414713	B08	4147134	1580
M415048	B08	4150485	1580
M421604	B08	4216042	1580
M421979	B08	4219796	1580
M425997	B08	4259970	1580
M426159	B08	4261591	1580
M426453	B08	4264537	1580
M427101	B08	4271015	1581
M431687	B08	4316874	1581
M431705	B08	4317053	1581
M431904	B08	4319044	1581

M432354	B08	4323541	1581
M432490	B08	4324906	1581
M432876	B08	4328762	1581
M434824	B08	4348242	1581
M436612	B08	4366121	1581
M437547	B08	4375475	1581
M438429	B08	4384290	1581
M439701	B08	4397017	1582
M441189	B08	4411898	1582
M441406	B08	4414063	1582
M442987	B08	4429877	1582
M444141	B08	4441413	1582
M444554	B08	4445549	1582
M445179	B08	4451797	1582
M448550	B08	4485502	1582
M449789	B08	4497891	1583
M450038	B08	4500384	1583
M450954	B08	4509546	1584
M451288	B08	4512880	1584
M451729	B08	4517292	1584
M452755	B08	4527550	1584
M452927	B08	4529275	1584
M454535	B08	4545351	1584
M455677	B08	4556771	1584
M456043	B08	4560430	1584
M458162	B08	4581624	1585
M458353	B08	4583539	1585
M459242	B08	4592424	1586
M459981	B08	4599812	1586
M460855	B08	4608554	1586
M461271	B08	4612711	1586
M461534	B08	4615345	1586
M461892	B08	4618923	1586
M462540	B08	4625401	1586
M466582	B08	4665827	1587
M466976	B08	4669761	1587
M468174	B08	4681747	1587
M468766	B08	4687666	1587
M473542	B08	4735424	1587
M490888	B08	4908887	1588
M491901	B08	4919017	1588
M500013	B08	5000130	1589
M500267	B08	5002670	1589
M500593	B08	5005932	1590
M501855	B08	5018558	1590
M501997	B08	5019973	1591
M502429	B08	5024297	1591

M503367	B08	5033676	1591
M503495	B08	5034950	1591
M504089	B08	5040898	1592
M504271	B08	5042716	1592
M504727	B08	5047277	1593
M506281	B08	5062816	1593
M506362	B08	5063629	1593
M507813	B08	5078136	1594
M508859	B08	5088597	1594
M516275	B08	5162758	1595
M516736	B08	5167369	1595
M517020	B08	5170208	1596
M518132	B08	5181326	1596
M521107	B08	5211073	1597
M522734	B08	5227342	1597
M525259	B08	5252591	1597
M528523	B08	5285239	1597
M532762	B08	5327627	1597
M554051	B08	5540515	1598
M554616	B08	5546167	1598
M555154	B08	5551543	1598
M556296	B08	5562965	1598
M556600	B08	5566004	1598
M556944	B08	5569445	1599
M557090	B08	5570907	1599
M559782	B08	5597822	1600
M560467	B08	5604678	1600
M564971	B08	5649715	1600
M565144	B08	5651441	1600
M567764	B08	5677643	1600
M567913	B08	5679138	1600
M568414	B08	5684147	1600
M569124	B08	5691244	1600
M569750	B08	5697501	1600
M570372	B08	5703726	1600
M572048	B08	5720487	1600
M572873	B08	5728731	1601
M579974	B08	5799742	1601
M580721	B08	5807213	1601
M581029	B08	5810294	1601
M582449	B08	5824491	1602
M582996	B08	5829963	1602
M585480	B08	5854802	1602
M586225	B08	5862251	1602
M586972	B08	5869726	1602
M587454	B08	5874541	1603
M588618	B08	5886184	1603

M589016	B08	5890169	1604
M589317	B08	5893171	1604
M590465	B08	5904656	1604
M590709	B08	5907096	1604
M591546	B08	5915468	1605
M592082	B08	5920827	1605
M592433	B08	5924334	1605
M597176	B08	5971766	1606
M598628	B08	5986282	1606
M598741	B08	5987412	1606
M598848	B08	5988480	1606
M599877	B08	5998778	1606
M607751	B08	6077510	1606
M608280	B08	6082808	1606
M609053	B08	6090536	1606
M609390	B08	6093903	1606
M609866	B08	6098663	1606
M612347	B08	6123470	1606
M612673	B08	6126734	1606
M613035	B08	6130358	1606
M616256	B08	6162569	1607
M616554	B08	6165541	1607
M617364	B08	6173641	1607
M617629	B08	6176296	1607
M617968	B08	6179688	1607
M618271	B08	6182714	1608
M618771	B08	6187719	1608
M619016	B08	6190169	1608
M619214	B08	6192149	1608
M622035	B08	6220356	1609
M622239	B08	6222394	1609
M622407	B08	6224075	1609
M624017	B08	6240178	1610
M624918	B08	6249187	1610
M626670	B08	6266702	1611
M627063	B08	6270631	1611
M627957	B08	6279571	1612
M629947	B08	6299473	1612
M640448	B08	6404489	1613
M640813	B08	6408137	1613
M641422	B08	6414226	1613
M641638	B08	6416380	1613
M642218	B08	6422182	1614
M643096	B08	6430967	1614
M643242	B08	6432422	1614
M644771	B08	6447712	1615
M644930	B08	6449304	1615

M646930	B08	6469309	1616
M647470	B08	6474707	1616
M647678	B08	6476783	1616
M647957	B08	6479575	1616
M648329	B08	6483292	1616
M648601	B08	6486013	1616
M649478	B08	6494785	1616
M649707	B08	6497074	1616
M653947	B08	6539471	1617
M654135	B08	6541350	1617
M654579	B08	6545795	1617
M740303	B08	7403032	1618
M743268	B08	7432688	1618
M767706	B08	7677067	1619
M768183	B08	7681832	1619
M768786	B08	7687869	1619
M770790	B08	7707902	1620
M773192	B08	7731925	1620
M777215	B08	7772154	1620
M778390	B08	7783900	1620
M778996	B08	7789960	1620
M799340	B08	7993400	1621
M799360	B08	7993601	1621
M800790	B08	8007905	1621
M806544	B08	8065449	1621
M807945	B08	8079452	1621
M809171	B08	8091713	1621
M857373	B08	8573734	1622
M858994	B08	8589946	1622
M860439	B08	8604394	1622
M861220	B08	8612202	1622
M862144	B08	8621444	1622
M863035	B08	8630356	1622
M864989	B08	8649899	1623
M865285	B08	8652857	1623
M892409	B08	8924093	1624
M892877	B08	8928779	1624
M893625	B08	8936254	1624
M893917	B08	8939178	1624
M894463	B08	8944631	1624
M897156	B08	8971560	1624
M898521	B08	8985218	1624
M900102	B08	9001027	1624
M900149	B08	9001494	1624
M901420	B08	9014200	1624
M901805	B08	9018059	1624
M902265	B08	9022651	1624

M902811	B08	9028115	1624
M905407	B08	9054072	1624
M905592	B08	9055925	1624
M924611	B08	9246118	1625
M929391	B08	9293914	1625
M930999	B08	9309991	1626
M932001	B08	9320016	1626
M932568	B08	9325685	1626
M933320	B08	9333204	1626
M934044	B08	9340447	1626
M934431	B08	9344310	1626
M936798	B08	9367984	1626
M938486	B08	9384865	1626
M939826	B08	9398269	1626
M943505	B08	9435054	1626
M944994	B08	9449941	1626
M983665	B08	9836652	1627
M984397	B08	9843972	1627
M985544	B08	9855441	1627
M986146	B08	9861460	1627
M986733	B08	9867330	1627
M986917	B08	9869178	1627
M991158	B08	9911584	1628
M991391	B08	9913911	1628
M992177	B08	9921776	1628
M993036	B08	9930364	1628
M997487	B08	9974876	1628
M998908	B08	9989081	1629
M100163	B08	10016355	1629
M100335	B08	10033546	1629
M102666	B08	10266620	1630
M102683	B08	10268358	1630
M102741	B08	10274107	1630
M102811	B08	10281100	1630
M103278	B08	10327849	1630
M103302	B08	10330276	1630
M103334	B08	10333459	1630
M103425	B08	10342551	1630
M103474	B08	10347423	1630
M103556	B08	10355606	1630
M111627	B08	11162711	1631
M112503	B08	11250332	1631
M112727	B08	11272740	1632
M112782	B08	11278283	1632
M112811	B08	11281167	1632
M112835	B08	11283596	1632
M112999	B08	11299982	1632

M113040	B08	11304050	1632
M113060	B08	11306091	1632
M113096	B08	11309690	1632
M113145	B08	11314525	1632
M113868	B08	11386808	1632
M114037	B08	11403756	1632
M114101	B08	11410166	1632
M114396	B08	11439685	1633
M114458	B08	11445852	1633
M114527	B08	11452740	1634
M114614	B08	11461417	1634
M114665	B08	11466571	1635
M114755	B08	11475547	1635
M115383	B08	11538327	1636
M115514	B08	11551434	1636
M115536	B08	11553673	1636
M115621	B08	11562107	1636
M115647	B08	11564725	1636
M115700	B08	11570046	1636
M115733	B08	11573373	1636
M115760	B08	11576035	1636
M115964	B08	11596466	1636
M115980	B08	11598007	1636
M116059	B08	11605914	1637
M116230	B08	11623076	1637
M116740	B08	11674080	1638
M116841	B08	11684140	1638
M116919	B08	11691955	1638
M117666	B08	11766677	1639
M118148	B08	11814801	1639
M118165	B08	11816509	1639
M118347	B08	11834798	1639
M118500	B08	11850007	1639
M118731	B08	11873148	1639
M118843	B08	11884367	1639
M118951	B08	11895185	1639
M119015	B08	11901568	1639
M119160	B08	11916095	1639
M119198	B08	11919883	1639
M119274	B08	11927408	1640
M119367	B08	11936799	1640
M119412	B08	11941206	1641
M119449	B08	11944982	1641
M122303	B08	12230352	1642
M122532	B08	12253275	1642
M122540	B08	12254021	1642
M122597	B08	12259701	1642

M122704	B08	12270492	1642
M122859	B08	12285973	1642
M122923	B08	12292377	1642
M123117	B08	12311793	1642
M123358	B08	12335802	1642
M123578	B08	12357810	1642
M123708	B08	12370815	1642
M123852	B08	12385256	1642
M123901	B08	12390100	1642
M124155	B08	12415594	1643
M124210	B08	12421038	1643
M124541	B08	12454133	1643
M125902	B08	12590264	1644
M125957	B08	12595738	1644
M126022	B08	12602275	1645
M126088	B08	12608880	1645
M126138	B08	12613833	1645
M126151	B08	12615135	1645
M126166	B08	12616668	1645
M126364	B08	12636409	1645
M126845	B08	12684597	1646
M127037	B08	12703771	1646
M127214	B08	12721439	1646
M127321	B08	12732174	1646
M127348	B08	12734830	1646
M127355	B08	12735548	1646
M127469	B08	12746927	1646
M127497	B08	12749729	1646
M127559	B08	12755927	1646
M127651	B08	12765103	1646
M127789	B08	12778967	1646
M127864	B08	12786497	1646
M127941	B08	12794154	1647
M128082	B08	12808298	1647
M128134	B08	12813465	1648
M128242	B08	12824260	1648
M128473	B08	12847388	1648
M128664	B08	12866438	1648
M128978	B08	12897811	1648
M129154	B08	12915463	1648
M129522	B08	12952229	1648
M129598	B08	12959842	1648
M129653	B08	12965386	1648
M130530	B08	13053020	1649
M130551	B08	13055134	1649
M130644	B08	13064451	1650
M130687	B08	13068730	1650

M130701	B08	13070196	1650
M130755	B08	13075552	1650
M130806	B08	13080676	1650
M130982	B08	13098220	1650
M131092	B08	13109275	1650
M131334	B08	13133454	1651
M131359	B08	13135922	1651
M132059	B08	13205920	1652
M132194	B08	13219458	1652
M132465	B08	13246508	1652
M134855	B08	13485527	1653
M134874	B08	13487427	1653
M134928	B08	13492807	1653
M134977	B08	13497756	1654
M135285	B08	13528582	1654
M135544	B08	13554488	1655
M135555	B08	13555591	1655
M135596	B08	13559676	1655
M135623	B08	13562307	1655
M136063	B08	13606305	1656
M136098	B08	13609807	1656
M136228	B08	13622850	1657
M136238	B08	13623805	1657
M136277	B08	13627714	1658
M136361	B08	13636140	1658
M136436	B08	13643658	1659
M136518	B08	13651845	1659
M136543	B08	13654356	1659
M136822	B08	13682201	1660
M136884	B08	13688402	1660
M136966	B08	13696681	1660
M138975	B08	13897578	1661
M138995	B08	13899583	1661
M139116	B08	13911630	1661
M139359	B08	13935931	1662
M139497	B08	13949747	1662
M139757	B08	13975759	1663
M139804	B08	13980405	1663
M140135	B08	14013593	1663
M140160	B08	14016083	1663
M140212	B08	14021237	1663
M140231	B08	14023139	1663
M140404	B08	14040450	1663
M140822	B08	14082294	1664
M140884	B08	14088413	1664
M141013	B08	14101375	1664
M141224	B08	14122422	1665

M141288	B08	14128841	1665
M141340	B08	14134097	1666
M141378	B08	14137805	1666
M141610	B08	14161040	1666
M141614	B08	14161415	1666
M141650	B08	14165086	1667
M141777	B08	14177709	1667
M142022	B08	14202291	1667
M142191	B08	14219190	1668
M142464	B08	14246462	1668
M142555	B08	14255590	1668
M142600	B08	14260000	1668
M142648	B08	14264846	1668
M143119	B08	14311948	1669
M143152	B08	14315273	1669
M143197	B08	14319796	1669
M143396	B08	14339686	1670
M143666	B08	14366621	1670
M144168	B08	14416804	1670
M144185	B08	14418519	1670
M144245	B08	14424505	1670
M144425	B08	14442585	1670
M144899	B08	14489918	1670
M147194	B08	14719474	1671
M147230	B08	14723001	1671
M147317	B08	14731793	1671
M147397	B08	14739712	1671
M147444	B08	14744474	1671
M147600	B08	14760002	1671
M147768	B08	14776831	1671
M147810	B08	14781053	1671
M147850	B08	14785043	1671
M147953	B08	14795314	1672
M147981	B08	14798169	1672
M148217	B08	14821793	1672
M148243	B08	14824303	1672
M148299	B08	14829965	1672
M148349	B08	14834917	1672
M148461	B08	14846187	1672
M148876	B08	14887684	1673
M149360	B08	14936046	1673
M149540	B08	14954069	1674
M149648	B08	14964843	1674
M149767	B08	14976776	1674
M149816	B08	14981638	1674
M149839	B08	14983974	1674
M149868	B08	14986855	1674

M149995	B08	14999575	1674
M150356	B08	15035641	1675
M150575	B08	15057588	1675
M151709	B08	15170958	1675
M151746	B08	15174683	1675
M151772	B08	15177279	1675
M151870	B08	15187095	1676
M152079	B08	15207995	1676
M152099	B08	15209975	1676
M152208	B08	15220816	1676
M152234	B08	15223477	1676
M152290	B08	15229011	1676
M152383	B08	15238350	1676
M152541	B08	15254100	1677
M152865	B08	15286599	1677
M153148	B08	15314898	1677
M153179	B08	15317975	1677
M154088	B08	15408858	1677
M155887	B08	15588731	1678
M156212	B08	15621206	1678
M157823	B08	15782306	1679
M157835	B08	15783570	1679
M159161	B08	15916183	1679
M164035	B08	16403540	1680
M164217	B08	16421726	1680
M164662	B08	16466226	1680
M165000	B08	16500028	1681
M165389	B08	16538983	1681
M165815	B08	16581578	1682
M165969	B08	16596958	1682
M166259	B08	16625914	1682
M168643	B08	16864338	1683
M168677	B08	16867779	1683
M173822	B08	17382235	1684
M175028	B08	17502862	1684
M176303	B08	17630383	1685
M176387	B08	17638749	1685
M176446	B08	17644664	1685
M176564	B08	17656435	1685
M176711	B08	17671138	1685
M176928	B08	17692846	1685
M177116	B08	17711604	1686
M177185	B08	17718537	1686
M177298	B08	17729815	1686
M177772	B08	17777211	1687
M178108	B08	17810834	1687
M178201	B08	17820106	1687

M178288	B08	17828831	1687
M178542	B08	17854291	1687
M178725	B08	17872580	1687
M178931	B08	17893199	1687
M179107	B08	17910758	1687
M179269	B08	17926940	1688
M179283	B08	17928365	1688
M179397	B08	17939792	1688
M179546	B08	17954629	1688
M179635	B08	17963526	1688
M179647	B08	17964734	1688
M179703	B08	17970332	1688
M179820	B08	17982074	1688
M180219	B08	18021947	1689
M180366	B08	18036694	1689
M180558	B08	18055883	1689
M180630	B08	18063036	1689
M180655	B08	18065557	1689
M180736	B08	18073646	1689
M180861	B08	18086165	1689
M181019	B08	18101902	1690
M181220	B08	18122089	1690
M181373	B08	18137353	1691
M181596	B08	18159634	1691
M181938	B08	18193816	1691
M182414	B08	18241448	1691
M182482	B08	18248228	1691
M182838	B08	18283879	1692
M183103	B08	18310319	1692
M183278	B08	18327822	1692
M183433	B08	18343339	1692
M183843	B08	18384351	1692
M184216	B08	18421620	1692
M184523	B08	18452353	1693
M184787	B08	18478765	1693
M185059	B08	18505994	1694
M185210	B08	18521014	1694
M185427	B08	18542760	1694
M185469	B08	18546914	1694
M185582	B08	18558210	1694
M185804	B08	18580433	1694
M185936	B08	18593604	1694
M185950	B08	18595081	1694
M186007	B08	18600752	1694
M186080	B08	18608097	1694
M186781	B08	18678165	1695
M187228	B08	18722845	1695

M187265	B08	18726502	1695
M188558	B08	18855804	1696
M188775	B08	18877595	1696
M188967	B08	18896790	1696
M189088	B08	18908872	1696
M189381	B08	18938167	1696
M198840	B08	19884086	1697
M198929	B08	19892949	1697
M202469	B08	20246973	1698
M203039	B08	20303901	1698
M240143	B08	24014353	1699
M240180	B08	24018098	1699
M240495	B08	24049583	1700
M240544	B08	24054404	1700
M241079	B08	24107941	1701
M241143	B08	24114392	1701
M241409	B08	24140989	1701
M241438	B08	24143898	1701
M241479	B08	24147902	1701
M241739	B08	24173977	1702
M241803	B08	24180311	1702
M241877	B08	24187726	1703
M243023	B08	24302310	1703
M246662	B08	24666217	1704
M247331	B08	24733115	1704
M247432	B08	24743263	1704
M247497	B08	24749739	1705
M247578	B08	24757854	1705
M249096	B08	24909650	1705
M260603	B08	26060348	1706
M262150	B08	26215000	1706
M272816	B08	27281665	1707
M273033	B08	27303378	1707
M277479	B08	27747902	1708
M277601	B08	27760167	1708
M278254	B08	27825451	1708
M278324	B08	27832466	1708
M278453	B08	27845341	1708
M278466	B08	27846655	1709
M278504	B08	27850427	1709
M278513	B08	27851319	1710
M278531	B08	27853141	1710
M278612	B08	27861266	1710
M295886	B08	29588652	1711
M295940	B08	29594093	1711
M296214	B08	29621401	1712
M296282	B08	29628221	1712

M296491	B08	29649115	1713
M296585	B08	29658574	1713
M311900	B08	31190062	1714
M312683	B08	31268376	1714
M359215	B08	35921578	1715
M359677	B08	35967769	1715
M363224	B08	36322451	1716
M364179	B08	36417954	1716
M365880	B08	36588046	1717
M367233	B08	36723398	1717
M384878	B08	38487862	1718
M385221	B08	38522135	1718
M386176	B08	38617697	1718
M388646	B08	38864615	1719
M389470	B08	38947048	1719
M389985	B08	38998564	1719
M395677	B08	39567720	1720
M396453	B08	39645326	1720
M405084	B08	40508471	1721
M406523	B08	40652320	1721
M425920	B08	42592028	1722
M426811	B08	42681110	1722
M427888	B08	42788810	1723
M428472	B08	42847247	1723
M428500	B08	42850005	1723
M428837	B08	42883742	1723
M429051	B08	42905185	1723
M429132	B08	42913222	1723
M429259	B08	42925940	1723
M429868	B08	42986835	1724
M429947	B08	42994710	1724
M430282	B08	43028201	1725
M430311	B08	43031189	1725
M430501	B08	43050169	1726
M430602	B08	43060252	1726
M430682	B08	43068262	1727
M431039	B08	43103961	1727
M431442	B08	43144278	1728
M431525	B08	43152515	1728
M431977	B08	43197791	1729
M432312	B08	43231271	1729
M432560	B08	43256031	1729
M432947	B08	43294788	1729
M432976	B08	43297640	1729
M433071	B08	43307129	1730
M433112	B08	43311226	1730
M433183	B08	43318339	1730

M433446	B08	43344693	1730
M433491	B08	43349156	1730
M433782	B08	43378240	1730
M434842	B08	43484267	1731
M434944	B08	43494489	1731
M435252	B08	43525269	1731
M435648	B08	43564832	1731
M436430	B08	43643051	1732
M437083	B08	43708345	1732
M440615	B08	44061571	1733
M440789	B08	44078906	1733
M441286	B08	44128696	1733
M441350	B08	44135032	1734
M441444	B08	44144419	1734
M441622	B08	44162274	1735
M441697	B08	44169766	1735
M441829	B08	44182955	1735
M442084	B08	44208455	1735
M442887	B08	44288781	1736
M442903	B08	44290367	1736
M443144	B08	44314426	1737
M443302	B08	44330218	1737
M443500	B08	44350040	1738
M443605	B08	44360506	1738
M443866	B08	44386685	1738
M443926	B08	44392636	1739
M444141	B08	44414114	1739
M444180	B08	44418078	1739
M444270	B08	44427032	1739
M444298	B08	44429804	1739
M444346	B08	44434687	1739
M444579	B08	44457919	1740
M444623	B08	44462320	1740
M444795	B08	44479514	1740
M444796	B08	44479604	1740
M444833	B08	44483358	1741
M444911	B08	44491156	1741
M444978	B08	44497876	1742
M445008	B08	44500827	1742
M447953	B08	44795379	1743
M448061	B08	44806123	1743
M448168	B08	44816860	1743
M448191	B08	44819136	1743
M448452	B08	44845217	1743
M456443	B08	45644301	1744
M456582	B08	45658292	1744
M459223	B08	45922383	1745

M460385	B08	46038533	1745
M460813	B08	46081386	1745
M460845	B08	46084580	1746
M460958	B08	46095868	1746
M461041	B08	46104171	1746
M461115	B08	46111574	1746
M461193	B08	46119333	1746
M461350	B08	46135088	1746
M462301	B08	46230190	1746
M463705	B08	46370571	1747
M464344	B08	46434469	1747
M464418	B08	46441899	1748
M464549	B08	46454959	1748
M465284	B08	46528406	1749
M465402	B08	46540263	1749
M465445	B08	46544547	1749
M465566	B08	46556600	1749
M465688	B08	46568870	1749
M465845	B08	46584548	1749
M465964	B08	46596430	1750
M466034	B08	46603477	1750
M466039	B08	46603988	1750
M466116	B08	46611661	1750
M466265	B08	46626527	1750
M466322	B08	46632246	1750
M466380	B08	46638017	1750
M466511	B08	46651158	1750
M466576	B08	46657656	1750
M466618	B08	46661865	1750
M466782	B08	46678237	1751
M466914	B08	46691477	1751
M467006	B08	46700663	1751
M467168	B08	46716826	1752
M467240	B08	46724001	1752
M467571	B08	46757123	1752
M467628	B08	46762869	1752
M472946	B08	47294636	1753
M474329	B08	47432991	1753
M474334	B08	47433409	1753
M474354	B08	47435462	1753
M474389	B08	47438918	1753
M474473	B08	47447354	1753
M474483	B08	47448350	1753
M474885	B08	47488535	1754
M474888	B08	47488879	1754
M477496	B08	47749648	1755
M478232	B08	47823224	1755

M480882	B08	48088251	1756
M480982	B08	48098249	1756
M481017	B08	48101712	1756
M481138	B08	48113894	1756
M481149	B08	48114958	1756
M481545	B08	48154519	1757
M481597	B08	48159778	1757
M481761	B08	48176159	1758
M481897	B08	48189786	1758
M482015	B08	48201525	1758
M482070	B08	48207067	1758
M482189	B08	48218903	1759
M482203	B08	48220373	1759
M482547	B08	48254739	1759
M482583	B08	48258368	1759
M482614	B08	48261485	1759
M482682	B08	48268247	1759
M482726	B08	48272699	1759
M482870	B08	48287027	1759
M482918	B08	48291810	1759
M484057	B08	48405706	1760
M484102	B08	48410267	1760
M484116	B08	48411674	1760
M484184	B08	48418492	1760
M484220	B08	48422072	1760
M484408	B08	48440873	1760
M484483	B08	48448330	1760
M484498	B08	48449858	1760
M493269	B08	49326917	1761
M493345	B08	49334539	1761
M493484	B08	49348482	1762
M493575	B08	49357540	1762
M493643	B08	49364335	1762
M493677	B08	49367746	1762
M493797	B08	49379712	1763
M493872	B08	49387233	1763
M494224	B08	49422447	1764
M494255	B08	49425577	1764
M495801	B08	49580180	1764
M495868	B08	49586823	1765
M495980	B08	49598007	1765
M496032	B08	49603253	1766
M496245	B08	49624543	1766
M496601	B08	49660171	1767
M496622	B08	49662215	1767
M497065	B08	49706541	1768
M497101	B08	49710136	1768

M497136	B08	49713611	1768
M497190	B08	49719011	1768
M498079	B08	49807963	1768
M498222	B08	49822274	1768
M501969	B08	50196948	1769
M502965	B08	50296526	1769
M504809	B08	50480915	1770
M504822	B08	50482236	1770
M504879	B08	50487922	1770
M505215	B08	50521551	1770
M505256	B08	50525691	1771
M506254	B08	50625438	1771
M507452	B08	50745263	1772
M507472	B08	50747216	1772
M507505	B08	50750505	1772
M507538	B08	50753859	1772
M507607	B08	50760752	1772
M507633	B08	50763355	1772
M509266	B08	50926653	1773
M509371	B08	50937133	1773
M509530	B08	50953077	1774
M509542	B08	50954255	1774
M509561	B08	50956119	1774
M509598	B08	50959805	1774
M510257	B08	51025755	1775
M510278	B08	51027840	1775
M510446	B08	51044619	1775
M510587	B08	51058764	1775
M511702	B08	51170258	1776
M511748	B08	51174832	1776
M511784	B08	51178424	1776
M512182	B08	51218270	1777
M512237	B08	51223799	1777
M512393	B08	51239371	1778
M512469	B08	51246955	1778
M512487	B08	51248787	1778
M512530	B08	51253054	1778
M512588	B08	51258852	1778
M512942	B08	51294247	1779
M512986	B08	51298695	1779
M513000	B08	51300070	1779
M513064	B08	51306449	1779
M513775	B08	51377598	1780
M513922	B08	51392246	1780
M514478	B08	51447818	1781
M514493	B08	51449309	1781
M514567	B08	51456747	1782

M514647	B08	51464722	1782
M514651	B08	51465119	1782
M514679	B08	51467953	1782
M514703	B08	51470386	1782
M514983	B08	51498389	1783
M515050	B08	51505061	1783
M515085	B08	51508583	1784
M515104	B08	51510468	1784
M515141	B08	51514134	1784
M515576	B08	51557621	1785
M515592	B08	51559254	1785
M515650	B08	51565099	1785
M515664	B08	51566492	1785
M515717	B08	51571708	1785
M515772	B08	51577248	1786
M515851	B08	51585119	1786
M515894	B08	51589437	1786
M516017	B08	51601713	1786
M516809	B08	51680935	1787
M516870	B08	51687077	1787
M517058	B08	51705845	1788
M517112	B08	51711212	1788
M517188	B08	51718882	1789
M517505	B08	51750537	1789
M517826	B08	51782654	1789
M517842	B08	51784228	1789
M517900	B08	51790064	1789
M517963	B08	51796341	1789
M517980	B08	51798098	1789
M518049	B08	51804985	1789
M518097	B08	51809779	1789
M518141	B08	51814164	1789
M518154	B08	51815404	1789
M518193	B08	51819369	1789
M518233	B08	51823335	1789
M518258	B08	51825862	1789
M518273	B08	51827373	1789
M518290	B08	51829027	1789
M518364	B08	51836492	1789
M518392	B08	51839216	1789
M518472	B08	51847217	1789
M518509	B08	51850972	1790
M518641	B08	51864117	1790
M518677	B08	51867719	1790
M518786	B08	51878679	1790
M518855	B08	51885526	1790
M518881	B08	51888128	1790

M519505	B08	51950541	1791
M519573	B08	51957373	1791
M519619	B08	51961966	1791
M519673	B08	51967351	1791
M519852	B08	51985278	1792
M519894	B08	51989491	1792
M519948	B08	51994813	1792
M520060	B08	52006044	1792
M521103	B08	52110393	1793
M521173	B08	52117354	1793
M521286	B08	52128660	1793
M521395	B08	52139502	1793
M521440	B08	52144037	1793
M521457	B08	52145755	1793
M521504	B08	52150499	1793
M521557	B08	52155730	1793
M521578	B08	52157899	1793
M521633	B08	52163337	1793
M522151	B08	52215123	1793
M522246	B08	52224613	1793
M522299	B08	52229905	1793
M522359	B08	52235987	1794
M522469	B08	52246969	1794
M522589	B08	52258988	1794
M522611	B08	52261160	1794
M522678	B08	52267880	1794
M522725	B08	52272568	1794
M522877	B08	52287797	1794
M522998	B08	52299871	1794
M523338	B08	52333805	1794
M523356	B08	52335634	1794
M523612	B08	52361277	1795
M523778	B08	52377816	1795
M523783	B08	52378386	1795
M523954	B08	52395496	1795
M523973	B08	52397372	1795
M524087	B08	52408718	1795
M524120	B08	52412000	1795
M524153	B08	52415391	1795
M524327	B08	52432723	1796
M524361	B08	52436163	1796
M524614	B08	52461482	1796
M524649	B08	52464939	1796
M524769	B08	52476977	1797
M524909	B08	52490959	1797
M524931	B08	52493124	1798
M524940	B08	52494027	1798

M524993	B08	52499310	1798
M525035	B08	52503528	1798
M525089	B08	52508991	1799
M525145	B08	52514549	1799
M525264	B08	52526472	1799
M525277	B08	52527707	1799
M525344	B08	52534410	1799
M525371	B08	52537148	1799
M525383	B08	52538345	1799
M525432	B08	52543277	1799
M525466	B08	52546674	1799
M525524	B08	52552407	1799
M525570	B08	52557050	1799
M526043	B08	52604336	1799
M526077	B08	52607741	1799
M526274	B08	52627465	1799
M526608	B08	52660821	1800
M526635	B08	52663501	1800
M526728	B08	52672850	1800
M526753	B08	52675319	1801
M526811	B08	52681129	1801
M526942	B08	52694238	1802
M527002	B08	52700248	1802
M527091	B08	52709186	1802
M527203	B08	52720375	1802
M527289	B08	52728961	1802
M527309	B08	52730968	1802
M527352	B08	52735259	1802
M528244	B08	52824458	1803
M528322	B08	52832272	1803
M8079	BS00363	8079	1804
M33894	BS00363	33894	1804
M4515	BS00403	4515	1805
M108012	BS00403	108012	1805
M5130	BS00629	5130	1806
M37676	BS00629	37676	1806
M47824	BS00629	47824	1806
M18849	BS00707	18849	1807
M45365	BS00707	45365	1807
M201042	C01	201042	1808
M288982	C01	288982	1808
M459599	C01	459599	1809
M471720	C01	471720	1809
M694980	C01	694980	1810
M719699	C01	719699	1810
M124033	C01	1240336	1811
M126301	C01	1263011	1811

M126940	C01	1269407	1811
M272900	C01	2729008	1812
M273241	C01	2732416	1812
M274180	C01	2741809	1813
M274420	C01	2744207	1813
M274728	C01	2747288	1814
M275108	C01	2751081	1814
M275562	C01	2755620	1815
M275786	C01	2757868	1815
M275873	C01	2758730	1816
M276203	C01	2762036	1816
M276568	C01	2765682	1817
M276671	C01	2766719	1817
M281583	C01	2815837	1818
M281906	C01	2819063	1818
M282313	C01	2823139	1818
M282598	C01	2825989	1818
M350286	C01	3502867	1819
M350314	C01	3503142	1819
M354643	C01	3546432	1819
M363812	C01	3638125	1819
M364018	C01	3640185	1819
M364214	C01	3642148	1819
M367466	C01	3674663	1820
M368991	C01	3689910	1820
M376563	C01	3765639	1821
M377665	C01	3776653	1821
M400356	C01	4003562	1822
M401332	C01	4013323	1822
M411398	C01	4113988	1822
M412660	C01	4126605	1822
M417756	C01	4177561	1823
M418002	C01	4180023	1823
M427119	C01	4271197	1824
M427555	C01	4275550	1824
M428336	C01	4283363	1824
M428817	C01	4288178	1825
M431398	C01	4313983	1825
M432176	C01	4321760	1825
M433628	C01	4336283	1825
M438921	C01	4389218	1825
M477713	C01	4777133	1826
M479446	C01	4794468	1826
M562213	C01	5622137	1827
M563109	C01	5631094	1827
M563242	C01	5632422	1827
M564306	C01	5643067	1828

M580270	C01	5802705	1828
M583045	C01	5830451	1829
M584188	C01	5841886	1829
M584888	C01	5848889	1829
M585206	C01	5852063	1829
M585436	C01	5854362	1829
M605292	C01	6052920	1830
M606236	C01	6062367	1830
M606459	C01	6064591	1830
M606840	C01	6068405	1830
M607899	C01	6078994	1830
M608326	C01	6083269	1830
M609512	C01	6095124	1831
M609937	C01	6099373	1831
M610075	C01	6100758	1831
M625187	C01	6251873	1831
M625709	C01	6257099	1832
M625912	C01	6259121	1832
M627785	C01	6277854	1833
M628749	C01	6287496	1833
M629637	C01	6296371	1833
M631338	C01	6313389	1833
M632171	C01	6321710	1834
M632664	C01	6326646	1834
M713343	C01	7133433	1835
M714428	C01	7144281	1835
M732354	C01	7323542	1836
M734331	C01	7343316	1836
M735175	C01	7351754	1836
M736309	C01	7363093	1837
M739445	C01	7394457	1837
M743359	C01	7433595	1837
M745443	C01	7454432	1838
M745775	C01	7457753	1838
M747433	C01	7474338	1838
M750883	C01	7508838	1839
M751617	C01	7516172	1839
M752546	C01	7525461	1839
M754998	C01	7549988	1839
M766607	C01	7666079	1840
M770361	C01	7703614	1840
M771531	C01	7715314	1840
M773053	C01	7730539	1840
M773566	C01	7735660	1840
M775708	C01	7757081	1840
M775908	C01	7759086	1840
M776153	C01	7761534	1840

M776736	C01	7767363	1840
M776987	C01	7769874	1840
M803810	C01	8038102	1841
M803948	C01	8039487	1841
M804999	C01	8049999	1841
M806038	C01	8060384	1841
M806555	C01	8065559	1841
M808196	C01	8081964	1841
M809369	C01	8093690	1841
M810051	C01	8100512	1841
M810108	C01	8101082	1841
M812881	C01	8128811	1842
M813224	C01	8132242	1842
M813951	C01	8139517	1842
M814141	C01	8141415	1842
M814978	C01	8149784	1842
M816312	C01	8163126	1842
M817688	C01	8176887	1842
M819435	C01	8194355	1843
M834626	C01	8346266	1843
M834702	C01	8347022	1843
M841586	C01	8415865	1844
M841753	C01	8417539	1844
M906765	C01	9067656	1845
M908679	C01	9086792	1845
M912344	C01	9123447	1846
M916783	C01	9167831	1846
M923155	C01	9231551	1846
M923597	C01	9235970	1846
M923831	C01	9238317	1846
M924071	C01	9240710	1846
M924783	C01	9247838	1846
M925282	C01	9252827	1846
M927259	C01	9272590	1846
M928016	C01	9280164	1846
M929524	C01	9295241	1847
M930999	C01	9309996	1847
M932515	C01	9325158	1848
M933871	C01	9338713	1848
M935007	C01	9350075	1848
M935979	C01	9359796	1848
M936763	C01	9367631	1848
M940749	C01	9407499	1848
M948103	C01	9481033	1848
M979206	C01	9792063	1849
M981070	C01	9810708	1849
M982426	C01	9824268	1849

M984172	C01	9841724	1849
M984486	C01	9844862	1849
M998270	C01	9982708	1850
M100865	C01	10086513	1850
M101678	C01	10167879	1851
M101780	C01	10178082	1851
M101798	C01	10179845	1851
M103535	C01	10353564	1852
M103802	C01	10380265	1852
M103900	C01	10390082	1852
M104756	C01	10475648	1852
M105102	C01	10510241	1853
M105112	C01	10511203	1853
M105535	C01	10553585	1853
M105693	C01	10569318	1853
M105734	C01	10573437	1853
M105810	C01	10581054	1853
M105826	C01	10582687	1854
M105845	C01	10584581	1854
M105908	C01	10590808	1855
M105934	C01	10593406	1855
M106170	C01	10617029	1855
M106237	C01	10623781	1855
M106251	C01	10625127	1855
M116934	C01	11693450	1856
M117255	C01	11725582	1856
M117434	C01	11743445	1856
M117449	C01	11744978	1856
M117626	C01	11762622	1856
M117786	C01	11778678	1856
M117807	C01	11780773	1856
M117860	C01	11786000	1856
M117903	C01	11790335	1856
M117982	C01	11798202	1856
M118034	C01	11803475	1856
M118094	C01	11809489	1856
M118202	C01	11820247	1857
M118244	C01	11824414	1857
M118251	C01	11825150	1858
M118311	C01	11831152	1858
M118381	C01	11838170	1858
M118426	C01	11842692	1858
M118473	C01	11847313	1858
M118767	C01	11876735	1858
M131960	C01	13196020	1859
M132517	C01	13251753	1859
M132540	C01	13254009	1859

M136332	C01	13633228	1860
M136432	C01	13643287	1860
M136458	C01	13645841	1860
M136522	C01	13652247	1860
M137828	C01	13782813	1861
M138266	C01	13826639	1861
M138339	C01	13833965	1861
M138380	C01	13838068	1861
M138432	C01	13843292	1861
M141215	C01	14121513	1862
M141326	C01	14132670	1862
M141382	C01	14138296	1863
M141430	C01	14143008	1863
M141457	C01	14145716	1863
M141494	C01	14149447	1863
M141924	C01	14192415	1863
M142299	C01	14229913	1863
M142328	C01	14232851	1863
M142670	C01	14267047	1864
M143005	C01	14300515	1864
M143138	C01	14313816	1864
M143360	C01	14336052	1864
M143400	C01	14340071	1864
M143957	C01	14395784	1864
M144232	C01	14423261	1864
M144295	C01	14429532	1865
M144311	C01	14431125	1865
M144423	C01	14442383	1865
M144432	C01	14443243	1865
M144508	C01	14450827	1865
M144564	C01	14456433	1865
M149699	C01	14969920	1866
M150974	C01	15097429	1866
M151003	C01	15100311	1866
M151085	C01	15108554	1866
M151198	C01	15119878	1866
M151298	C01	15129889	1866
M151489	C01	15148904	1867
M151545	C01	15154532	1867
M151663	C01	15166317	1867
M151732	C01	15173271	1867
M151795	C01	15179570	1867
M151861	C01	15186135	1867
M151894	C01	15189493	1867
M151968	C01	15196850	1867
M152152	C01	15215244	1867
M152331	C01	15233121	1867

M152600	C01	15260048	1867
M152840	C01	15284085	1867
M152938	C01	15293801	1867
M153077	C01	15307760	1867
M153249	C01	15324933	1868
M153417	C01	15341764	1868
M153761	C01	15376133	1868
M154574	C01	15457454	1868
M154603	C01	15460328	1868
M154626	C01	15462633	1868
M154841	C01	15484162	1869
M154915	C01	15491584	1869
M154975	C01	15497506	1869
M155076	C01	15507615	1869
M155248	C01	15524867	1869
M155403	C01	15540363	1869
M155429	C01	15542981	1869
M155578	C01	15557842	1869
M155683	C01	15568328	1869
M155805	C01	15580592	1869
M156407	C01	15640707	1869
M156584	C01	15658476	1870
M156622	C01	15662279	1870
M156665	C01	15666531	1870
M156698	C01	15669841	1870
M156730	C01	15673038	1870
M156762	C01	15676262	1870
M157083	C01	15708323	1870
M157240	C01	15724041	1870
M165289	C01	16528911	1871
M165536	C01	16553656	1871
M165601	C01	16560125	1871
M165631	C01	16563137	1871
M165710	C01	16571096	1871
M165753	C01	16575392	1871
M165847	C01	16584720	1871
M166005	C01	16600510	1871
M166354	C01	16635458	1871
M175663	C01	17566314	1872
M176984	C01	17698464	1872
M193610	C01	19361037	1873
M194775	C01	19477564	1873
M200202	C01	20020263	1874
M201368	C01	20136873	1874
M212628	C01	21262865	1875
M213891	C01	21389117	1875
M224737	C01	22473789	1876

M225886	C01	22588659	1876
M245583	C01	24558340	1877
M246159	C01	24615947	1877
M247013	C01	24701364	1877
M249975	C01	24997591	1878
M250213	C01	25021355	1878
M277612	C01	27761227	1879
M278136	C01	27813641	1879
M279179	C01	27917910	1879
M324199	C01	32419901	1880
M324255	C01	32425520	1880
M324380	C01	32438047	1880
M324546	C01	32454659	1880
M324573	C01	32457311	1880
M324710	C01	32471078	1880
M324871	C01	32487114	1880
M324938	C01	32493809	1880
M325290	C01	32529045	1880
M325464	C01	32546431	1880
M325975	C01	32597554	1881
M326152	C01	32615216	1881
M326344	C01	32634410	1882
M327209	C01	32720964	1882
M327523	C01	32752329	1882
M327639	C01	32763975	1882
M327930	C01	32793039	1882
M342069	C01	34206973	1883
M342986	C01	34298619	1883
M356433	C01	35643371	1884
M356585	C01	35658507	1884
M356677	C01	35667701	1884
M380029	C01	38002945	1885
M380331	C01	38033185	1885
M381683	C01	38168330	1886
M383208	C01	38320887	1886
M405398	C01	40539850	1887
M405486	C01	40548682	1887
M405515	C01	40551552	1887
M405601	C01	40560103	1887
M405682	C01	40568262	1888
M405906	C01	40590666	1888
M406092	C01	40609239	1888
M406225	C01	40622579	1888
M414211	C01	41421187	1889
M414325	C01	41432548	1889
M414723	C01	41472391	1889
M414913	C01	41491320	1889

M415220	C01	41522018	1889
M415388	C01	41538892	1890
M415415	C01	41541589	1890
M415461	C01	41546121	1890
M415463	C01	41546355	1890
M415491	C01	41549160	1890
M415511	C01	41551168	1890
M415602	C01	41560231	1890
M416430	C01	41643098	1890
M416676	C01	41667606	1891
M416912	C01	41691242	1891
M416977	C01	41697760	1891
M417029	C01	41702919	1892
M417144	C01	41714464	1892
M417186	C01	41718663	1892
M417267	C01	41726779	1893
M417309	C01	41730963	1893
M417331	C01	41733144	1893
M417484	C01	41748469	1894
M417653	C01	41765370	1894
M417715	C01	41771558	1894
M418093	C01	41809389	1894
M418125	C01	41812521	1894
M418214	C01	41821423	1894
M418288	C01	41828847	1895
M419712	C01	41971290	1895
M419788	C01	41978881	1895
M423840	C01	42384051	1896
M423978	C01	42397836	1896
M424533	C01	42453350	1896
M426491	C01	42649191	1897
M426549	C01	42654987	1897
M426700	C01	42670066	1897
M426740	C01	42674076	1897
M426813	C01	42681338	1898
M426850	C01	42685038	1898
M427994	C01	42799448	1898
M427997	C01	42799782	1898
M428030	C01	42803059	1898
M428090	C01	42809085	1898
M428441	C01	42844127	1899
M428493	C01	42849340	1899
M428576	C01	42857663	1899
M428596	C01	42859613	1899
M428701	C01	42870176	1899
M428767	C01	42876768	1899
M428810	C01	42881092	1899

M428840	C01	42884098	1899
M429008	C01	42900866	1899
M429029	C01	42902957	1900
M429059	C01	42905979	1900
M429157	C01	42915709	1901
M429284	C01	42928484	1901
M429411	C01	42941134	1902
M429428	C01	42942810	1902
M431857	C01	43185747	1903
M432064	C01	43206441	1903
M432138	C01	43213830	1904
M432156	C01	43215662	1904
M432307	C01	43230728	1904
M432437	C01	43243786	1904
M432565	C01	43256508	1904
M436355	C01	43635557	1905
M436375	C01	43637549	1905
M436562	C01	43656267	1905
M908	C02	908	1906
M49844	C02	49844	1906
M85891	C02	85891	1906
M98069	C02	98069	1906
M103379	C02	103379	1906
M114840	C02	114840	1907
M116173	C02	116173	1907
M122113	C02	122113	1908
M140299	C02	140299	1908
M173094	C02	173094	1908
M175421	C02	175421	1908
M186170	C02	186170	1908
M195034	C02	195034	1908
M247733	C02	247733	1909
M248837	C02	248837	1909
M254863	C02	254863	1909
M258695	C02	258695	1909
M305010	C02	305010	1910
M333820	C02	333820	1910
M366950	C02	366950	1910
M370722	C02	370722	1910
M441882	C02	441882	1910
M450224	C02	450224	1910
M456685	C02	456685	1910
M471906	C02	471906	1911
M474597	C02	474597	1911
M494517	C02	494517	1911
M498282	C02	498282	1911
M501486	C02	501486	1911

M506176	C02	506176	1911
M516733	C02	516733	1912
M522664	C02	522664	1912
M528569	C02	528569	1913
M535786	C02	535786	1913
M594252	C02	594252	1914
M608520	C02	608520	1914
M616198	C02	616198	1914
M623471	C02	623471	1914
M659349	C02	659349	1915
M662376	C02	662376	1915
M691868	C02	691868	1915
M716643	C02	716643	1916
M791895	C02	791895	1916
M795227	C02	795227	1916
M796920	C02	796920	1917
M806210	C02	806210	1917
M810906	C02	810906	1918
M898371	C02	898371	1918
M923358	C02	923358	1918
M965668	C02	965668	1919
M968440	C02	968440	1919
M982149	C02	982149	1919
M990186	C02	990186	1919
M112306	C02	1123061	1919
M112496	C02	1124960	1919
M112602	C02	1126022	1920
M114284	C02	1142845	1920
M114880	C02	1148805	1920
M115599	C02	1155992	1920
M117323	C02	1173239	1920
M135415	C02	1354150	1921
M136810	C02	1368103	1921
M137060	C02	1370602	1921
M137349	C02	1373498	1921
M138754	C02	1387549	1921
M139062	C02	1390624	1921
M139688	C02	1396881	1921
M217142	C02	2171423	1922
M221520	C02	2215201	1922
M222069	C02	2220693	1923
M222439	C02	2224396	1923
M275465	C02	2754654	1924
M275879	C02	2758798	1924
M281594	C02	2815949	1924
M283642	C02	2836422	1924
M300648	C02	3006484	1925

M302671	C02	3026718	1925
M384072	C02	3840724	1926
M386106	C02	3861067	1926
M429911	C02	4299116	1927
M431484	C02	4314844	1927
M432937	C02	4329377	1927
M433345	C02	4333454	1927
M434420	C02	4344205	1927
M434841	C02	4348412	1928
M435276	C02	4352765	1928
M435316	C02	4353166	1928
M458892	C02	4588920	1929
M459347	C02	4593472	1929
M461202	C02	4612025	1929
M461570	C02	4615706	1929
M462305	C02	4623053	1929
M463409	C02	4634098	1929
M463425	C02	4634253	1929
M463900	C02	4639001	1930
M464514	C02	4645143	1930
M493584	C02	4935843	1931
M508646	C02	5086463	1931
M515154	C02	5151543	1932
M521043	C02	5210439	1932
M522256	C02	5222569	1932
M524492	C02	5244925	1933
M524576	C02	5245767	1933
M528660	C02	5286606	1934
M529381	C02	5293811	1934
M529532	C02	5295328	1934
M531659	C02	5316597	1934
M539576	C02	5395767	1934
M555918	C02	5559182	1935
M556415	C02	5564155	1935
M557220	C02	5572202	1935
M562318	C02	5623185	1935
M563725	C02	5637259	1935
M564015	C02	5640150	1935
M565098	C02	5650982	1935
M565313	C02	5653132	1935
M565799	C02	5657994	1936
M571622	C02	5716226	1936
M571873	C02	5718731	1937
M572772	C02	5727724	1937
M580660	C02	5806605	1937
M646978	C02	6469785	1938
M648005	C02	6480050	1938

M649404	C02	6494047	1938
M650442	C02	6504427	1938
M650961	C02	6509615	1938
M651925	C02	6519250	1938
M652124	C02	6521243	1938
M653402	C02	6534020	1938
M653587	C02	6535878	1938
M661525	C02	6615252	1938
M662468	C02	6624682	1938
M664531	C02	6645316	1939
M665666	C02	6656665	1939
M666472	C02	6664720	1939
M666636	C02	6666364	1939
M668539	C02	6685399	1940
M669784	C02	6697846	1940
M672049	C02	6720498	1940
M673814	C02	6738147	1940
M674000	C02	6740001	1941
M674992	C02	6749927	1941
M697880	C02	6978807	1942
M698315	C02	6983150	1942
M703534	C02	7035346	1942
M703727	C02	7037271	1942
M705854	C02	7058547	1942
M707455	C02	7074558	1943
M707970	C02	7079706	1943
M708343	C02	7083430	1943
M708474	C02	7084744	1943
M712867	C02	7128671	1944
M714196	C02	7141967	1944
M715524	C02	7155246	1944
M716073	C02	7160730	1944
M725773	C02	7257731	1944
M726262	C02	7262620	1945
M728259	C02	7282592	1945
M729981	C02	7299816	1946
M731221	C02	7312217	1946
M734854	C02	7348546	1947
M738504	C02	7385046	1947
M798898	C02	7988986	1948
M800150	C02	8001507	1948
M116951	C02	11695153	1949
M117295	C02	11729538	1949
M171815	C02	17181541	1950
M171938	C02	17193898	1950
M172081	C02	17208190	1951
M172118	C02	17211833	1951

M172183	C02	17218306	1951
M198525	C02	19852566	1952
M198652	C02	19865296	1952
M198753	C02	19875335	1952
M198900	C02	19890026	1952
M199103	C02	19910382	1952
M199151	C02	19915106	1953
M199221	C02	19922188	1953
M199252	C02	19925241	1954
M199462	C02	19946249	1954
M199497	C02	19949795	1955
M199785	C02	19978583	1955
M199807	C02	19980749	1955
M199998	C02	19999899	1955
M200127	C02	20012793	1955
M200445	C02	20044599	1956
M200731	C02	20073128	1956
M200776	C02	20077645	1956
M200911	C02	20091121	1957
M201011	C02	20101137	1957
M223555	C02	22355574	1958
M223843	C02	22384398	1958
M223912	C02	22391259	1958
M223971	C02	22397192	1958
M223993	C02	22399374	1958
M224400	C02	22440027	1959
M224537	C02	22453741	1959
M224794	C02	22479444	1959
M224946	C02	22494618	1959
M225113	C02	22511370	1959
M225236	C02	22523612	1960
M225340	C02	22534098	1960
M225561	C02	22556170	1960
M225716	C02	22571672	1960
M225794	C02	22579466	1960
M226194	C02	22619487	1960
M262282	C02	26228287	1961
M262785	C02	26278545	1961
M267897	C02	26789704	1962
M267927	C02	26792703	1962
M268012	C02	26801278	1962
M268078	C02	26807831	1963
M268159	C02	26815951	1963
M268252	C02	26825260	1963
M268398	C02	26839860	1964
M268713	C02	26871331	1964
M268728	C02	26872855	1964

M268850	C02	26885048	1964
M269030	C02	26903092	1964
M269177	C02	26917774	1965
M269285	C02	26928515	1965
M269384	C02	26938470	1966
M269396	C02	26939609	1966
M269475	C02	26947542	1966
M269536	C02	26953662	1966
M270079	C02	27007917	1967
M270188	C02	27018844	1967
M270328	C02	27032865	1967
M275461	C02	27546196	1968
M275581	C02	27558156	1968
M276445	C02	27644524	1969
M277853	C02	27785348	1969
M277972	C02	27797244	1969
M278205	C02	27820512	1970
M278207	C02	27820766	1970
M278341	C02	27834198	1970
M278468	C02	27846884	1970
M278553	C02	27855367	1970
M278850	C02	27885017	1971
M278995	C02	27899503	1971
M279138	C02	27913858	1972
M279249	C02	27924989	1972
M279569	C02	27956912	1973
M279716	C02	27971629	1973
M279854	C02	27985446	1973
M281014	C02	28101448	1974
M281073	C02	28107397	1974
M281138	C02	28113892	1974
M281342	C02	28134230	1974
M281402	C02	28140256	1974
M281596	C02	28159635	1974
M281721	C02	28172187	1974
M281742	C02	28174294	1974
M281898	C02	28189810	1974
M282541	C02	28254119	1975
M282559	C02	28255975	1975
M282729	C02	28272942	1976
M282853	C02	28285398	1976
M282960	C02	28296007	1976
M283127	C02	28312760	1976
M283205	C02	28320559	1976
M283500	C02	28350051	1977
M283548	C02	28354864	1977
M284164	C02	28416475	1978

M284249	C02	28424977	1978
M284272	C02	28427247	1978
M284373	C02	28437390	1978
M284444	C02	28444437	1978
M284728	C02	28472831	1978
M284832	C02	28483249	1978
M285502	C02	28550285	1979
M285678	C02	28567822	1979
M285987	C02	28598776	1979
M286361	C02	28636193	1980
M286520	C02	28652090	1980
M286624	C02	28662492	1980
M286649	C02	28664982	1980
M286722	C02	28672236	1980
M286976	C02	28697645	1981
M287179	C02	28717947	1981
M287184	C02	28718439	1981
M287297	C02	28729784	1981
M287714	C02	28771402	1982
M288013	C02	28801324	1982
M288019	C02	28801947	1982
M288380	C02	28838025	1982
M288815	C02	28881591	1983
M289261	C02	28926157	1983
M289281	C02	28928191	1984
M289500	C02	28950071	1984
M289506	C02	28950622	1984
M289715	C02	28971562	1984
M289786	C02	28978602	1985
M289953	C02	28995367	1985
M290405	C02	29040545	1986
M290511	C02	29051113	1986
M290568	C02	29056849	1986
M291001	C02	29100151	1987
M291503	C02	29150364	1987
M291516	C02	29151613	1987
M291916	C02	29191630	1987
M292214	C02	29221444	1988
M292324	C02	29232490	1988
M300008	C02	30000839	1989
M301300	C02	30130060	1989
M303856	C02	30385644	1990
M304608	C02	30460866	1990
M347911	C02	34791128	1991
M349249	C02	34924972	1991
M353991	C02	35399179	1992
M355583	C02	35558344	1992

M356694	C02	35669404	1993
M357829	C02	35782927	1993
M362580	C02	36258099	1994
M363734	C02	36373409	1994
M386960	C02	38696015	1995
M387662	C02	38766214	1995
M423278	C02	42327802	1996
M423346	C02	42334632	1996
M423393	C02	42339346	1996
M423418	C02	42341832	1996
M423439	C02	42343982	1996
M423459	C02	42345981	1996
M423705	C02	42370558	1997
M425142	C02	42514255	1997
M426244	C02	42624452	1998
M426395	C02	42639530	1998
M426991	C02	42699116	1998
M427042	C02	42704266	1998
M427267	C02	42726775	1998
M427328	C02	42732860	1998
M431607	C02	43160798	1999
M431908	C02	43190855	1999
M432009	C02	43200944	1999
M432341	C02	43234121	1999
M432366	C02	43236633	1999
M432707	C02	43270796	1999
M432956	C02	43295620	1999
M433077	C02	43307789	1999
M433116	C02	43311633	1999
M433216	C02	43321667	2000
M433335	C02	43333524	2000
M442860	C02	44286062	2001
M444456	C02	44445692	2001
M447130	C02	44713069	2002
M447189	C02	44718940	2002
M451491	C02	45149114	2003
M452278	C02	45227838	2003
M452394	C02	45239427	2003
M452591	C02	45259179	2003
M452716	C02	45271662	2003
M452944	C02	45294473	2004
M453136	C02	45313697	2004
M453193	C02	45319366	2004
M453459	C02	45345987	2004
M453517	C02	45351712	2004
M453551	C02	45355102	2004
M453608	C02	45360818	2004

M453635	C02	45363554	2004
M453738	C02	45373806	2004
M453783	C02	45378396	2004
M453837	C02	45383726	2004
M453837	C02	45383742	2004
M453978	C02	45397824	2004
M454085	C02	45408591	2004
M454162	C02	45416220	2004
M454247	C02	45424705	2004
M454392	C02	45439207	2004
M454425	C02	45442507	2004
M493417	C02	49341703	2005
M494025	C02	49402524	2005
M494057	C02	49405735	2005
M494326	C02	49432670	2006
M494331	C02	49433150	2006
M496415	C02	49641558	2007
M496421	C02	49642121	2007
M496459	C02	49645992	2007
M497514	C02	49751456	2008
M497604	C02	49760446	2008
M497680	C02	49768089	2009
M497799	C02	49779900	2009
M498021	C02	49802171	2010
M498185	C02	49818533	2010
M498575	C02	49857591	2010
M498827	C02	49882780	2011
M498862	C02	49886253	2011
M499059	C02	49905959	2011
M499064	C02	49906408	2011
M520243	C02	52024351	2012
M521410	C02	52141024	2012
M521659	C02	52165990	2012
M527886	C02	52788630	2013
M528030	C02	52803077	2013
M830015	C03	830015	2014
M894157	C03	894157	2014
M900654	C03	900654	2014
M905958	C03	905958	2014
M144161	C03	1441619	2015
M144806	C03	1448065	2015
M167381	C03	1673813	2016
M167795	C03	1677957	2016
M167987	C03	1679878	2017
M179177	C03	1791774	2017
M181262	C03	1812623	2017
M181283	C03	1812830	2017

M181457	C03	1814577	2017
M183562	C03	1835627	2018
M184832	C03	1848321	2018
M186946	C03	1869469	2019
M187490	C03	1874905	2019
M188124	C03	1881249	2020
M188600	C03	1886004	2020
M189473	C03	1894735	2021
M189830	C03	1898308	2021
M190281	C03	1902819	2021
M190733	C03	1907333	2021
M289964	C03	2899647	2022
M290703	C03	2907033	2022
M291395	C03	2913950	2023
M291819	C03	2918192	2023
M292070	C03	2920704	2024
M292388	C03	2923887	2024
M292817	C03	2928175	2025
M293330	C03	2933306	2025
M304138	C03	3041385	2026
M311804	C03	3118048	2026
M318566	C03	3185664	2027
M319062	C03	3190621	2027
M322091	C03	3220916	2027
M582325	C03	5823256	2028
M597960	C03	5979600	2028
M604069	C03	6040698	2029
M604336	C03	6043367	2029
M604941	C03	6049416	2029
M605826	C03	6058262	2030
M606836	C03	6068368	2030
M610728	C03	6107284	2030
M611362	C03	6113625	2030
M611654	C03	6116545	2030
M611880	C03	6118802	2030
M613530	C03	6135300	2030
M614608	C03	6146088	2030
M619376	C03	6193769	2030
M619536	C03	6195362	2030
M828471	C03	8284717	2031
M830047	C03	8300473	2031
M970817	C03	9708178	2032
M971600	C03	9716000	2032
M972390	C03	9723909	2033
M972619	C03	9726192	2033
M973190	C03	9731900	2033
M975990	C03	9759909	2033

M977706	C03	9777067	2034
M978317	C03	9783172	2034
M983867	C03	9838679	2035
M985135	C03	9851351	2035
M985821	C03	9858212	2035
M111899	C03	11189952	2036
M113200	C03	11320092	2036
M113268	C03	11326897	2036
M113280	C03	11328062	2036
M115162	C03	11516254	2037
M115539	C03	11553914	2037
M115650	C03	11565089	2038
M115806	C03	11580637	2038
M115927	C03	11592793	2038
M115965	C03	11596592	2038
M116000	C03	11600083	2038
M116088	C03	11608828	2038
M116134	C03	11613478	2038
M122936	C03	12293652	2039
M122959	C03	12295953	2039
M123222	C03	12322215	2039
M124306	C03	12430688	2040
M124331	C03	12433139	2040
M137539	C03	13753946	2041
M137689	C03	13768913	2041
M153954	C03	15395418	2042
M153996	C03	15399622	2042
M154378	C03	15437869	2043
M154598	C03	15459862	2043
M154693	C03	15469382	2043
M154716	C03	15471616	2043
M154848	C03	15484843	2043
M154984	C03	15498409	2043
M155223	C03	15522391	2044
M155355	C03	15535557	2044
M155359	C03	15535923	2044
M155402	C03	15540202	2045
M155461	C03	15546179	2045
M155565	C03	15556528	2045
M155654	C03	15565439	2045
M156134	C03	15613406	2046
M156327	C03	15632738	2046
M156457	C03	15645703	2046
M156476	C03	15647604	2046
M156546	C03	15654680	2046
M156582	C03	15658292	2046
M156682	C03	15668238	2047

M156732	C03	15673291	2047
M156845	C03	15684571	2048
M156915	C03	15691525	2048
M157243	C03	15724386	2049
M157364	C03	15736483	2049
M157570	C03	15757081	2050
M157979	C03	15797919	2050
M158213	C03	15821335	2051
M158274	C03	15827442	2051
M158374	C03	15837469	2051
M158392	C03	15839260	2051
M168160	C03	16816080	2052
M168243	C03	16824324	2052
M168303	C03	16830367	2052
M168485	C03	16848537	2052
M169006	C03	16900673	2053
M169030	C03	16903055	2053
M169555	C03	16955509	2053
M169700	C03	16970059	2053
M169743	C03	16974387	2053
M169792	C03	16979239	2053
M169874	C03	16987437	2053
M170166	C03	17016646	2054
M170371	C03	17037139	2054
M170509	C03	17050931	2054
M170642	C03	17064252	2054
M170763	C03	17076309	2055
M171840	C03	17184077	2055
M172135	C03	17213532	2055
M172157	C03	17215740	2055
M172490	C03	17249004	2056
M172653	C03	17265341	2056
M172706	C03	17270608	2056
M172939	C03	17293932	2056
M173372	C03	17337254	2056
M173447	C03	17344721	2056
M177227	C03	17722777	2057
M177272	C03	17727292	2057
M177345	C03	17734553	2058
M177467	C03	17746766	2058
M178502	C03	17850218	2058
M180024	C03	18002494	2059
M180293	C03	18029388	2059
M182527	C03	18252796	2060
M182602	C03	18260232	2060
M182951	C03	18295198	2060
M183087	C03	18308770	2060

M183121	C03	18312143	2060
M183131	C03	18313143	2060
M183164	C03	18316465	2061
M183191	C03	18319127	2061
M183604	C03	18360483	2062
M183870	C03	18387028	2062
M183882	C03	18388233	2063
M183934	C03	18393440	2063
M184043	C03	18404358	2064
M184392	C03	18439245	2064
M184459	C03	18445991	2064
M184716	C03	18471696	2065
M184735	C03	18473521	2065
M184865	C03	18486582	2066
M184976	C03	18497607	2066
M185106	C03	18510619	2067
M185190	C03	18519068	2067
M185388	C03	18538889	2068
M185475	C03	18547511	2068
M185629	C03	18562909	2069
M185714	C03	18571499	2069
M185781	C03	18578165	2069
M185818	C03	18581891	2069
M185836	C03	18583661	2069
M185899	C03	18589957	2069
M232939	C03	23293979	2070
M233263	C03	23326309	2070
M233693	C03	23369309	2070
M233758	C03	23375853	2070
M236392	C03	23639215	2071
M236509	C03	23650984	2071
M236560	C03	23656037	2071
M236676	C03	23667615	2071
M241182	C03	24118286	2072
M241248	C03	24124856	2072
M241374	C03	24137467	2072
M241604	C03	24160460	2073
M242036	C03	24203642	2073
M242116	C03	24211646	2073
M242173	C03	24217385	2073
M242233	C03	24223384	2073
M243152	C03	24315295	2073
M253793	C03	25379370	2074
M253822	C03	25382242	2074
M254069	C03	25406978	2074
M254106	C03	25410616	2074
M254136	C03	25413632	2074

M254150	C03	25415086	2074
M254169	C03	25416980	2074
M258251	C03	25825191	2075
M258262	C03	25826281	2075
M259039	C03	25903946	2075
M259149	C03	25914946	2075
M259190	C03	25919026	2075
M259227	C03	25922703	2075
M259399	C03	25939989	2076
M259533	C03	25953338	2076
M259715	C03	25971573	2076
M260725	C03	26072554	2076
M260970	C03	26097001	2076
M261590	C03	26159047	2077
M262049	C03	26204924	2077
M262259	C03	26225926	2078
M262325	C03	26232567	2078
M262362	C03	26236239	2078
M262476	C03	26247631	2078
M262541	C03	26254123	2078
M262591	C03	26259161	2079
M262946	C03	26294614	2079
M270287	C03	27028799	2080
M270467	C03	27046777	2080
M272147	C03	27214776	2081
M272183	C03	27218338	2081
M272239	C03	27223971	2082
M272240	C03	27224045	2082
M272316	C03	27231641	2083
M273669	C03	27366950	2083
M273758	C03	27375845	2084
M273810	C03	27381080	2084
M273854	C03	27385405	2085
M273991	C03	27399141	2085
M274904	C03	27490480	2086
M276125	C03	27612551	2086
M296444	C03	29644412	2087
M296543	C03	29654380	2087
M296633	C03	29663363	2087
M296685	C03	29668569	2087
M296768	C03	29676821	2087
M296864	C03	29686487	2087
M298378	C03	29837863	2088
M298407	C03	29840780	2088
M298500	C03	29850048	2088
M298590	C03	29859067	2088
M298896	C03	29889628	2088

M298901	C03	29890105	2088
M298986	C03	29898690	2088
M309279	C03	30927914	2089
M310757	C03	31075755	2089
M312150	C03	31215005	2090
M312946	C03	31294694	2090
M312976	C03	31297687	2090
M313065	C03	31306537	2091
M313181	C03	31318183	2091
M313228	C03	31322840	2092
M313317	C03	31331732	2092
M313401	C03	31340137	2093
M314949	C03	31494987	2093
M316115	C03	31611542	2094
M316872	C03	31687259	2094
M316940	C03	31694075	2094
M316956	C03	31695638	2094
M317538	C03	31753857	2094
M317751	C03	31775164	2095
M317941	C03	31794129	2095
M317969	C03	31796923	2095
M318766	C03	31876668	2096
M318819	C03	31881984	2096
M318957	C03	31895774	2096
M331298	C03	33129820	2097
M332737	C03	33273702	2097
M340388	C03	34038804	2098
M341057	C03	34105710	2098
M341141	C03	34114146	2098
M341587	C03	34158719	2098
M344372	C03	34437288	2099
M344442	C03	34444231	2099
M344605	C03	34460588	2099
M344845	C03	34484520	2099
M345110	C03	34511036	2099
M345244	C03	34524429	2100
M345373	C03	34537350	2100
M345444	C03	34544468	2100
M345558	C03	34555852	2100
M345686	C03	34568632	2100
M349063	C03	34906390	2101
M349153	C03	34915341	2101
M349271	C03	34927157	2101
M349418	C03	34941899	2102
M349492	C03	34949217	2102
M349627	C03	34962758	2102
M349850	C03	34985082	2103

M349872	C03	34987214	2103
M350005	C03	35000531	2103
M350099	C03	35009979	2103
M350184	C03	35018487	2103
M355400	C03	35540079	2104
M355600	C03	35560021	2104
M355736	C03	35573639	2104
M355766	C03	35576640	2104
M355894	C03	35589495	2105
M356177	C03	35617768	2105
M356377	C03	35637723	2105
M356984	C03	35698467	2106
M358066	C03	35806689	2106
M358144	C03	35814451	2106
M363282	C03	36328214	2107
M363753	C03	36375374	2107
M365509	C03	36550916	2108
M365683	C03	36568305	2108
M366020	C03	36602026	2108
M366040	C03	36604040	2108
M366089	C03	36608900	2108
M366922	C03	36692289	2108
M366946	C03	36694645	2109
M367071	C03	36707152	2109
M367081	C03	36708156	2110
M367142	C03	36714223	2110
M367232	C03	36723263	2111
M367271	C03	36727180	2111
M367323	C03	36732364	2112
M367352	C03	36735270	2112
M367717	C03	36771744	2113
M367735	C03	36773591	2113
M368151	C03	36815101	2114
M368225	C03	36822577	2114
M368262	C03	36826278	2114
M368369	C03	36836991	2115
M368624	C03	36862474	2115
M368663	C03	36866355	2116
M368773	C03	36877304	2116
M369647	C03	36964750	2117
M369767	C03	36976742	2117
M369828	C03	36982899	2117
M370378	C03	37037815	2117
M370511	C03	37051198	2117
M370604	C03	37060443	2117
M370651	C03	37065194	2117
M370680	C03	37068092	2118

M370826	C03	37082671	2118
M370930	C03	37093002	2119
M371108	C03	37110840	2119
M371151	C03	37115145	2119
M371179	C03	37117977	2119
M371273	C03	37127329	2119
M372004	C03	37200454	2120
M372038	C03	37203866	2120
M372169	C03	37216987	2120
M372385	C03	37238523	2121
M372773	C03	37277352	2121
M372809	C03	37280922	2122
M374408	C03	37440867	2122
M380393	C03	38039376	2123
M380887	C03	38088723	2123
M380985	C03	38098508	2123
M381121	C03	38112164	2123
M381180	C03	38118095	2123
M381314	C03	38131451	2123
M422815	C03	42281520	2124
M423534	C03	42353469	2124
M445238	C03	44523845	2125
M445500	C03	44550072	2125
M446794	C03	44679412	2125
M494403	C03	49440378	2126
M494799	C03	49479936	2126
M513033	C03	51303369	2127
M514095	C03	51409503	2127
M514106	C03	51410688	2128
M514426	C03	51442646	2128
M527206	C03	52720696	2129
M527396	C03	52739687	2129
M527528	C03	52752864	2129
M527552	C03	52755230	2129
M528014	C03	52801450	2130
M528183	C03	52818393	2130
M528269	C03	52826973	2131
M528314	C03	52831409	2131
M528355	C03	52835523	2132
M528477	C03	52847776	2132
M528527	C03	52852713	2133
M528549	C03	52854967	2133
M528832	C03	52883256	2134
M530263	C03	53026311	2134
M532849	C03	53284989	2135
M533896	C03	53389674	2135
M533945	C03	53394546	2136

M533996	C03	53399601	2136
M534082	C03	53408271	2136
M571542	C03	57154208	2137
M571711	C03	57171135	2137
M572032	C03	57203213	2138
M572552	C03	57255216	2138
M572592	C03	57259211	2138
M572793	C03	57279347	2139
M572886	C03	57288645	2139
M573291	C03	57329155	2139
M573355	C03	57335558	2139
M573398	C03	57339831	2139
M576648	C03	57664844	2140
M578143	C03	57814333	2140
M578553	C03	57855382	2141
M579879	C03	57987915	2141
M580051	C03	58005153	2141
M580051	C03	58005178	2141
M580165	C03	58016539	2142
M580323	C03	58032384	2142
M585403	C03	58540337	2143
M585551	C03	58555100	2143
M585969	C03	58596937	2144
M586043	C03	58604312	2144
M586368	C03	58636806	2145
M586454	C03	58645488	2145
M587391	C03	58739157	2146
M587648	C03	58764880	2146
M588089	C03	58808975	2146
M588250	C03	58825013	2146
M588408	C03	58840802	2146
M588649	C03	58864985	2147
M588930	C03	58893024	2147
M589186	C03	58918670	2147
M589528	C03	58952806	2147
M589573	C03	58957357	2147
M589671	C03	58967133	2147
M590164	C03	59016432	2147
M590218	C03	59021882	2147
M590247	C03	59024765	2147
M590299	C03	59029999	2148
M590333	C03	59033335	2148
M590352	C03	59035299	2148
M590598	C03	59059814	2149
M591093	C03	59109317	2149
M591497	C03	59149790	2149
M591698	C03	59169801	2149

M591808	C03	59180836	2149
M592183	C03	59218354	2149
M592227	C03	59222720	2150
M592310	C03	59231084	2150
M606405	C03	60640516	2151
M606422	C03	60642286	2151
M607098	C03	60709831	2152
M607139	C03	60713918	2152
M608480	C03	60848005	2152
M608971	C03	60897109	2153
M609056	C03	60905670	2153
M609160	C03	60916023	2153
M609802	C03	60980209	2154
M610015	C03	61001554	2154
M610683	C03	61068337	2154
M615595	C03	61559534	2155
M615678	C03	61567848	2155
M615943	C03	61594335	2155
M616087	C03	61608797	2155
M616206	C03	61620629	2155
M616284	C03	61628409	2155
M616714	C03	61671437	2155
M616745	C03	61674539	2155
M619167	C03	61916741	2156
M620318	C03	62031806	2156
M620390	C03	62039081	2156
M620683	C03	62068324	2156
M625944	C03	62594437	2157
M625986	C03	62598608	2157
M626047	C03	62604793	2157
M626088	C03	62608883	2157
M626191	C03	62619147	2158
M626218	C03	62621888	2158
M626333	C03	62633352	2158
M626978	C03	62697802	2158
M627028	C03	62702851	2158
M628109	C03	62810958	2159
M628668	C03	62866896	2159
M628746	C03	62874650	2159
M628894	C03	62889458	2160
M629110	C03	62911076	2160
M629354	C03	62935468	2160
M629990	C03	62999082	2160
M633949	C03	63394977	2161
M634165	C03	63416541	2161
M634414	C03	63441418	2161
M634549	C03	63454927	2161

M634633	C03	63463357	2161
M634740	C03	63474068	2161
M634914	C03	63491401	2161
M635233	C03	63523386	2162
M635323	C03	63532363	2162
M636944	C03	63694496	2163
M636968	C03	63696821	2163
M637124	C03	63712431	2164
M637302	C03	63730241	2164
M637421	C03	63742101	2164
M637544	C03	63754438	2164
M637617	C03	63761788	2164
M637736	C03	63773617	2164
M637958	C03	63795841	2165
M638116	C03	63811643	2165
M641598	C03	64159876	2166
M641628	C03	64162802	2166
M641933	C03	64193339	2167
M642284	C03	64228452	2167
M642812	C03	64281266	2167
M643800	C03	64380060	2168
M643972	C03	64397268	2168
M645034	C03	64503405	2168
M645323	C03	64532336	2168
M646526	C03	64652670	2169
M646576	C03	64657657	2169
M646595	C03	64659550	2169
M647008	C03	64700897	2169
M647096	C03	64709670	2169
M650282	C03	65028293	2170
M650291	C03	65029138	2170
M650375	C03	65037564	2170
M651158	C03	65115875	2170
M651216	C03	65121610	2170
M651228	C03	65122861	2170
M651620	C03	65162082	2170
M653273	C03	65327316	2171
M653296	C03	65329637	2171
M653344	C03	65334491	2171
M653412	C03	65341237	2171
M653638	C03	65363816	2171
M653698	C03	65369873	2172
M653707	C03	65370796	2172
M653762	C03	65376209	2172
M653870	C03	65387097	2172
M654024	C03	65402411	2173
M654127	C03	65412782	2173

M654192	C03	65419276	2174
M654293	C03	65429365	2174
M654331	C03	65433152	2174
M654515	C03	65451571	2174
M654645	C03	65464556	2175
M654684	C03	65468440	2175
M654724	C03	65472443	2175
M101851	C04	1018519	2176
M102558	C04	1025585	2176
M103440	C04	1034401	2176
M103716	C04	1037163	2176
M106337	C04	1063376	2176
M107753	C04	1077538	2176
M108438	C04	1084387	2176
M108844	C04	1088449	2176
M109608	C04	1096086	2176
M110045	C04	1100452	2176
M110459	C04	1104595	2176
M111134	C04	1111341	2176
M111373	C04	1113732	2176
M111721	C04	1117214	2176
M112535	C04	1125354	2176
M116984	C04	1169845	2176
M202731	C04	2027312	2177
M206730	C04	2067307	2177
M242149	C04	2421493	2178
M242919	C04	2429193	2178
M244651	C04	2446514	2179
M251557	C04	2515575	2179
M259334	C04	2593348	2179
M259787	C04	2597871	2180
M261301	C04	2613019	2180
M275773	C04	2757734	2180
M276652	C04	2766521	2181
M277441	C04	2774412	2181
M277566	C04	2775661	2181
M289681	C04	2896810	2181
M290077	C04	2900771	2181
M290554	C04	2905544	2181
M290994	C04	2909940	2181
M292490	C04	2924903	2181
M299940	C04	2999408	2182
M310701	C04	3107017	2182
M310921	C04	3109217	2182
M331062	C04	3310627	2183
M343638	C04	3436383	2183
M343981	C04	3439810	2183

M346081	C04	3460818	2183
M348463	C04	3484636	2184
M349091	C04	3490919	2184
M350975	C04	3509757	2184
M355880	C04	3558808	2184
M356278	C04	3562788	2184
M399322	C04	3993221	2185
M399545	C04	3995452	2185
M399912	C04	3999129	2185
M400148	C04	4001489	2185
M400981	C04	4009811	2185
M402040	C04	4020409	2185
M404219	C04	4042197	2185
M404452	C04	4044529	2185
M420474	C04	4204745	2186
M420541	C04	4205415	2186
M421173	C04	4211739	2186
M434540	C04	4345400	2186
M470726	C04	4707263	2187
M475132	C04	4751329	2187
M509826	C04	5098260	2188
M512059	C04	5120598	2188
M512505	C04	5125058	2188
M514594	C04	5145940	2188
M550004	C04	5500045	2189
M552408	C04	5524087	2189
M553905	C04	5539056	2190
M555424	C04	5554248	2190
M555958	C04	5559586	2190
M569067	C04	5690677	2190
M569650	C04	5696500	2190
M570571	C04	5705717	2191
M571061	C04	5710611	2191
M591168	C04	5911687	2192
M591450	C04	5914504	2192
M592698	C04	5926987	2193
M598200	C04	5982000	2193
M602914	C04	6029142	2193
M605374	C04	6053749	2193
M606322	C04	6063229	2193
M637334	C04	6373344	2194
M639028	C04	6390280	2194
M657264	C04	6572647	2195
M657549	C04	6575495	2195
M658302	C04	6583027	2195
M659238	C04	6592383	2196
M659993	C04	6599933	2196

M661973	C04	6619738	2196
M662675	C04	6626759	2196
M673920	C04	6739205	2196
M675207	C04	6752072	2196
M679904	C04	6799046	2197
M685407	C04	6854070	2197
M685818	C04	6858180	2197
M717010	C04	7170105	2198
M727954	C04	7279546	2198
M728302	C04	7283029	2198
M728745	C04	7287456	2198
M730797	C04	7307970	2199
M731942	C04	7319428	2199
M732480	C04	7324809	2199
M739189	C04	7391896	2200
M739376	C04	7393769	2200
M739993	C04	7399939	2200
M740454	C04	7404549	2200
M740671	C04	7406712	2200
M763372	C04	7633722	2201
M764096	C04	7640965	2201
M764480	C04	7644804	2201
M765408	C04	7654088	2201
M768243	C04	7682430	2201
M769329	C04	7693297	2202
M770075	C04	7700750	2202
M770289	C04	7702896	2202
M771154	C04	7711545	2203
M771639	C04	7716394	2203
M772414	C04	7724147	2203
M861493	C04	8614939	2204
M873973	C04	8739731	2204
M108235	C04	10823589	2205
M108386	C04	10838662	2205
M108569	C04	10856908	2205
M108578	C04	10857833	2205
M108664	C04	10866482	2205
M132127	C04	13212798	2206
M132144	C04	13214457	2206
M132841	C04	13284193	2207
M132919	C04	13291912	2207
M133162	C04	13316232	2208
M133323	C04	13332364	2208
M169224	C04	16922455	2209
M170363	C04	17036366	2209
M183317	C04	18331784	2210
M183737	C04	18373772	2210

M185464	C04	18546475	2211
M186313	C04	18631384	2211
M192739	C04	19273996	2212
M193633	C04	19363384	2212
M200757	C04	20075729	2213
M201657	C04	20165784	2213
M219948	C04	21994805	2214
M221079	C04	22107980	2214
M283581	C04	28358185	2215
M284729	C04	28472934	2215
M293207	C04	29320757	2216
M294605	C04	29460527	2216
M302154	C04	30215491	2217
M303652	C04	30365222	2217
M306669	C04	30666968	2218
M308235	C04	30823505	2218
M331241	C04	33124153	2219
M331304	C04	33130487	2219
M331865	C04	33186536	2219
M331917	C04	33191780	2219
M332017	C04	33201741	2219
M332123	C04	33212320	2219
M332182	C04	33218206	2219
M332608	C04	33260866	2219
M332706	C04	33270614	2220
M332755	C04	33275517	2220
M332837	C04	33283799	2220
M332941	C04	33294151	2220
M333330	C04	33333015	2221
M333520	C04	33352010	2221
M333649	C04	33364964	2221
M333794	C04	33379419	2221
M333937	C04	33393792	2221
M334338	C04	33433854	2222
M334349	C04	33434981	2222
M334717	C04	33471768	2222
M343729	C04	34372995	2223
M343988	C04	34398816	2223
M344060	C04	34406009	2223
M344075	C04	34407506	2223
M344215	C04	34421517	2223
M344237	C04	34423794	2223
M344969	C04	34496959	2223
M345139	C04	34513976	2223
M345381	C04	34538184	2224
M346800	C04	34680029	2224
M394235	C04	39423587	2225

M394545	C04	39454581	2225
M407681	C04	40768151	2226
M407758	C04	40775869	2226
M407992	C04	40799250	2226
M410095	C04	41009586	2227
M410732	C04	41073244	2227
M419990	C04	41999006	2228
M420215	C04	42021501	2228
M420603	C04	42060379	2228
M420848	C04	42084809	2228
M421174	C04	42117410	2228
M422465	C04	42246576	2229
M423207	C04	42320760	2229
M423284	C04	42328497	2229
M423421	C04	42342183	2229
M423497	C04	42349732	2229
M424032	C04	42403206	2229
M434372	C04	43437274	2230
M434407	C04	43440718	2230
M434503	C04	43450360	2231
M434543	C04	43454348	2231
M446912	C04	44691287	2232
M447270	C04	44727050	2232
M447359	C04	44735959	2232
M448032	C04	44803296	2232
M449952	C04	44995252	2233
M449989	C04	44998927	2233
M450195	C04	45019518	2233
M460043	C04	46004320	2234
M460709	C04	46070998	2234
M460901	C04	46090163	2234
M461109	C04	46110988	2234
M461199	C04	46119908	2234
M461265	C04	46126576	2234
M461312	C04	46131213	2234
M461900	C04	46190087	2235
M462013	C04	46201366	2235
M462168	C04	46216836	2236
M462333	C04	46233321	2236
M462638	C04	46263814	2237
M462739	C04	46273940	2237
M462834	C04	46283439	2237
M462858	C04	46285836	2237
M463362	C04	46336200	2238
M463533	C04	46353334	2238
M463704	C04	46370436	2239
M464024	C04	46402484	2239

M464202	C04	46420291	2239
M464977	C04	46497759	2240
M465752	C04	46575248	2240
M465811	C04	46581184	2240
M466029	C04	46602945	2240
M466237	C04	46623730	2240
M466544	C04	46654473	2240
M466563	C04	46656342	2241
M466592	C04	46659207	2241
M466664	C04	46666408	2241
M466685	C04	46668535	2241
M466725	C04	46672582	2241
M466868	C04	46686804	2241
M467046	C04	46704611	2242
M467102	C04	46710217	2242
M467278	C04	46727819	2243
M467291	C04	46729113	2243
M467520	C04	46752035	2243
M467556	C04	46755617	2244
M467694	C04	46769437	2244
M467923	C04	46792386	2245
M468661	C04	46866143	2245
M501697	C04	50169725	2246
M501734	C04	50173415	2246
M501779	C04	50177900	2246
M501792	C04	50179266	2246
M501885	C04	50188578	2246
M501918	C04	50191831	2247
M503110	C04	50311078	2247
M503503	C04	50350302	2247
M503729	C04	50372976	2248
M503844	C04	50384487	2248
M510289	C04	51028911	2249
M510365	C04	51036534	2249
M510444	C04	51044483	2249
M510466	C04	51046674	2249
M510554	C04	51055424	2249
M520711	C04	52071145	2250
M521461	C04	52146153	2250
M523050	C04	52305050	2251
M523088	C04	52308884	2251
M523124	C04	52312467	2252
M523658	C04	52365855	2252
M523726	C04	52372649	2252
M524583	C04	52458366	2252
M528641	C04	52864159	2253
M528794	C04	52879409	2253

M529068	C04	52906827	2253
M537162	C04	53716239	2254
M537176	C04	53717610	2254
M537250	C04	53725063	2255
M537354	C04	53735489	2255
M537391	C04	53739122	2255
M537418	C04	53741816	2255
M537447	C04	53744742	2255
M540956	C04	54095653	2256
M541856	C04	54185640	2256
M543081	C04	54308102	2257
M543365	C04	54336520	2257
M543455	C04	54345551	2257
M544066	C04	54406695	2257
M544087	C04	54408775	2257
M544112	C04	54411260	2257
M544132	C04	54413237	2257
M544180	C04	54418000	2257
M544330	C04	54433023	2257
M544367	C04	54436747	2257
M544458	C04	54445823	2258
M544506	C04	54450625	2258
M544563	C04	54456396	2258
M544608	C04	54460879	2258
M545072	C04	54507298	2259
M545658	C04	54565883	2259
M1400	C05	1400	2260
M4880	C05	4880	2260
M34435	C05	34435	2260
M38849	C05	38849	2260
M41714	C05	41714	2260
M47019	C05	47019	2260
M49282	C05	49282	2260
M55576	C05	55576	2260
M56708	C05	56708	2260
M62496	C05	62496	2260
M67382	C05	67382	2260
M71379	C05	71379	2260
M74213	C05	74213	2260
M78972	C05	78972	2260
M81232	C05	81232	2260
M91140	C05	91140	2260
M95815	C05	95815	2260
M105636	C05	105636	2260
M127815	C05	127815	2260
M142158	C05	142158	2260
M145419	C05	145419	2260

M147108	C05	147108	2261
M157034	C05	157034	2261
M178537	C05	178537	2261
M194551	C05	194551	2261
M199254	C05	199254	2261
M201196	C05	201196	2261
M204624	C05	204624	2261
M206364	C05	206364	2261
M253015	C05	253015	2261
M295449	C05	295449	2261
M826615	C05	826615	2262
M844876	C05	844876	2262
M989961	C05	989961	2263
M108433	C05	1084335	2263
M108916	C05	1089161	2263
M109083	C05	1090836	2263
M109465	C05	1094657	2264
M110264	C05	1102643	2264
M111629	C05	1116292	2264
M125393	C05	1253933	2264
M132062	C05	1320620	2265
M133099	C05	1330996	2265
M135397	C05	1353973	2265
M136041	C05	1360412	2265
M136288	C05	1362883	2265
M137299	C05	1372992	2265
M138347	C05	1383470	2266
M138726	C05	1387267	2266
M143884	C05	1438843	2266
M145410	C05	1454107	2266
M146003	C05	1460034	2266
M146163	C05	1461631	2266
M146525	C05	1465251	2266
M149737	C05	1497372	2266
M154091	C05	1540916	2266
M154369	C05	1543697	2267
M155441	C05	1554416	2267
M158744	C05	1587441	2267
M159504	C05	1595044	2267
M160418	C05	1604185	2267
M184644	C05	1846448	2268
M184795	C05	1847952	2268
M232874	C05	2328744	2269
M237265	C05	2372650	2269
M238388	C05	2383881	2270
M249218	C05	2492187	2270
M252077	C05	2520775	2270

M271074	C05	2710741	2271
M271371	C05	2713712	2271
M271376	C05	2713761	2271
M293053	C05	2930531	2272
M295936	C05	2959363	2272
M296489	C05	2964893	2272
M344907	C05	3449076	2273
M345257	C05	3452575	2273
M345355	C05	3453557	2273
M358728	C05	3587287	2274
M364875	C05	3648752	2274
M365282	C05	3652829	2274
M373850	C05	3738500	2275
M373858	C05	3738586	2275
M381436	C05	3814362	2275
M389665	C05	3896657	2275
M393160	C05	3931607	2276
M393489	C05	3934894	2276
M395731	C05	3957310	2276
M475198	C05	4751987	2277
M475237	C05	4752378	2277
M619177	C05	6191776	2278
M620024	C05	6200247	2278
M656504	C05	6565049	2279
M659148	C05	6591485	2279
M704295	C05	7042955	2280
M717587	C05	7175877	2280
M717741	C05	7177416	2280
M718295	C05	7182957	2281
M718390	C05	7183907	2281
M723984	C05	7239842	2282
M724919	C05	7249192	2282
M727289	C05	7272898	2283
M737192	C05	7371921	2283
M737344	C05	7373446	2283
M737546	C05	7375464	2284
M738171	C05	7381712	2284
M738207	C05	7382076	2284
M738828	C05	7388287	2285
M740301	C05	7403017	2285
M740643	C05	7406439	2286
M741305	C05	7413056	2286
M742265	C05	7422653	2286
M743920	C05	7439208	2286
M744894	C05	7448949	2286
M747259	C05	7472590	2286
M748593	C05	7485939	2287

M749125	C05	7491250	2287
M749752	C05	7497525	2288
M750869	C05	7508694	2288
M752632	C05	7526326	2289
M754340	C05	7543408	2289
M755758	C05	7557589	2290
M756753	C05	7567533	2290
M762869	C05	7628699	2290
M771065	C05	7710656	2290
M771268	C05	7712685	2290
M771681	C05	7716819	2290
M771721	C05	7717217	2291
M771879	C05	7718791	2291
M772399	C05	7723993	2291
M773494	C05	7734947	2291
M943386	C05	9433865	2292
M947062	C05	9470623	2292
M947860	C05	9478604	2292
M948113	C05	9481138	2293
M948504	C05	9485041	2293
M949331	C05	9493317	2293
M949488	C05	9494884	2294
M954292	C05	9542922	2294
M956247	C05	9562476	2294
M960732	C05	9607329	2295
M964162	C05	9641623	2295
M968356	C05	9683563	2296
M969000	C05	9690005	2296
M971794	C05	9717940	2297
M973958	C05	9739586	2297
M974174	C05	9741749	2297
M987286	C05	9872864	2297
M990600	C05	9906000	2298
M991776	C05	9917766	2298
M993110	C05	9931103	2298
M995375	C05	9953755	2298
M995624	C05	9956248	2298
M100349	C05	10034907	2299
M100580	C05	10058078	2299
M100933	C05	10093352	2299
M101421	C05	10142183	2300
M101480	C05	10148032	2300
M116923	C05	11692350	2301
M117639	C05	11763913	2301
M118352	C05	11835255	2301
M122738	C05	12273819	2302
M124146	C05	12414674	2302

M125632	C05	12563272	2303
M125717	C05	12571764	2303
M126040	C05	12604068	2304
M126077	C05	12607743	2304
M126430	C05	12643079	2304
M126603	C05	12660337	2305
M126768	C05	12676808	2305
M126886	C05	12688649	2305
M127141	C05	12714123	2306
M127159	C05	12715941	2306
M127186	C05	12718657	2306
M127261	C05	12726156	2307
M127319	C05	12731924	2307
M127843	C05	12784308	2308
M128212	C05	12821264	2308
M128246	C05	12824629	2309
M128338	C05	12833802	2309
M128421	C05	12842194	2309
M128472	C05	12847292	2309
M134493	C05	13449390	2310
M134855	C05	13485589	2310
M135027	C05	13502739	2310
M143667	C05	14366796	2311
M143765	C05	14376544	2311
M143914	C05	14391496	2311
M143984	C05	14398437	2312
M144078	C05	14407825	2312
M144104	C05	14410420	2313
M144167	C05	14416783	2313
M144258	C05	14425865	2314
M144607	C05	14460753	2314
M146553	C05	14655330	2315
M147596	C05	14759624	2315
M147756	C05	14775623	2316
M147847	C05	14784713	2316
M147964	C05	14796414	2316
M147983	C05	14798311	2316
M148755	C05	14875578	2317
M148811	C05	14881152	2317
M148925	C05	14892566	2317
M148964	C05	14896442	2318
M149036	C05	14903660	2318
M149207	C05	14920779	2318
M149493	C05	14949394	2318
M149607	C05	14960720	2318
M149706	C05	14970663	2318
M149753	C05	14975300	2318

M151770	C05	15177057	2319
M151814	C05	15181476	2319
M152254	C05	15225470	2320
M152294	C05	15229433	2320
M152448	C05	15244888	2320
M152489	C05	15248942	2320
M152571	C05	15257110	2320
M152711	C05	15271195	2321
M152744	C05	15274439	2321
M152769	C05	15276951	2322
M152857	C05	15285738	2322
M152916	C05	15291628	2322
M153157	C05	15315766	2322
M154841	C05	15484172	2323
M155007	C05	15500714	2323
M155175	C05	15517529	2324
M155365	C05	15536530	2324
M155542	C05	15554298	2324
M177570	C05	17757085	2325
M178171	C05	17817144	2325
M184964	C05	18496423	2326
M185461	C05	18546173	2326
M195643	C05	19564309	2327
M195781	C05	19578167	2327
M196048	C05	19604896	2328
M196450	C05	19645076	2328
M222671	C05	22267158	2329
M223419	C05	22341952	2329
M223780	C05	22378040	2329
M247847	C05	24784714	2330
M249440	C05	24944091	2330
M261162	C05	26116284	2331
M261913	C05	26191313	2331
M269015	C05	26901542	2332
M269537	C05	26953778	2332
M276172	C05	27617258	2333
M277127	C05	27712742	2333
M277590	C05	27759033	2333
M303671	C05	30367138	2334
M304495	C05	30449585	2334
M310877	C05	31087719	2335
M311369	C05	31136951	2335
M312446	C05	31244668	2335
M321163	C05	32116377	2336
M321614	C05	32161487	2336
M333742	C05	33374298	2337
M334750	C05	33475092	2337

M356526	C05	35652665	2338
M357401	C05	35740150	2338
M360932	C05	36093261	2339
M360946	C05	36094669	2339
M397405	C05	39740592	2340
M397421	C05	39742149	2340
M397506	C05	39750622	2340
M397535	C05	39753501	2341
M397619	C05	39761983	2341
M397682	C05	39768208	2341
M397975	C05	39797560	2341
M398021	C05	39802123	2341
M407260	C05	40726076	2342
M407296	C05	40729688	2342
M407903	C05	40790334	2342
M408012	C05	40801290	2342
M408109	C05	40810952	2342
M408277	C05	40827720	2342
M408704	C05	40870405	2343
M408871	C05	40887193	2343
M408902	C05	40890213	2343
M409146	C05	40914694	2343
M413753	C05	41375392	2344
M415226	C05	41522606	2344
M418166	C05	41816686	2345
M418826	C05	41882627	2345
M420253	C05	42025385	2346
M421361	C05	42136118	2346
M424098	C05	42409805	2347
M424132	C05	42413262	2347
M434903	C05	43490344	2348
M435015	C05	43501548	2348
M435143	C05	43514372	2348
M436775	C05	43677577	2349
M436811	C05	43681133	2349
M437023	C05	43702332	2349
M438325	C05	43832583	2349
M438353	C05	43835392	2349
M438418	C05	43841880	2350
M439106	C05	43910665	2350
M440206	C05	44020644	2351
M441255	C05	44125553	2351
M441287	C05	44128707	2351
M441836	C05	44183683	2352
M441849	C05	44184961	2352
M441874	C05	44187489	2353
M441936	C05	44193632	2353

M442119	C05	44211911	2354
M442144	C05	44214423	2354
M442258	C05	44225815	2355
M442952	C05	44295242	2355
M443405	C05	44340591	2356
M443421	C05	44342170	2356
M443439	C05	44343935	2356
M443617	C05	44361726	2356
M443901	C05	44390182	2356
M443902	C05	44390200	2356
M443936	C05	44393638	2356
M444010	C05	44401028	2356
M444083	C05	44408318	2356
M444194	C05	44419472	2356
M444708	C05	44470879	2357
M444774	C05	44477431	2357
M444944	C05	44494442	2357
M454250	C05	45425034	2358
M454966	C05	45496670	2358
M454983	C05	45498327	2358
M455018	C05	45501864	2358
M455029	C05	45502916	2358
M455117	C05	45511729	2358
M455290	C05	45529006	2359
M455303	C05	45530334	2359
M455312	C05	45531205	2359
M455343	C05	45534359	2359
M455704	C05	45570472	2360
M455902	C05	45590214	2360
M456968	C05	45696814	2360
M458703	C05	45870319	2361
M458774	C05	45877414	2361
M458784	C05	45878447	2361
M458800	C05	45880013	2361
M458870	C05	45887030	2362
M458996	C05	45899613	2362
M459119	C05	45911931	2362
M459188	C05	45918856	2362
M461759	C05	46175991	2363
M461892	C05	46189204	2363
M462299	C05	46229972	2363
M35552	C06	35552	2364
M159551	C06	159551	2364
M371482	C06	371482	2365
M412749	C06	412749	2365
M837931	C06	837931	2366
M997367	C06	997367	2366

M759525	C06	7595251	2367
M760163	C06	7601639	2367
M761021	C06	7610215	2367
M762540	C06	7625409	2367
M765473	C06	7654739	2367
M765902	C06	7659026	2367
M768968	C06	7689688	2367
M769296	C06	7692969	2367
M769638	C06	7696383	2367
M770619	C06	7706195	2367
M771608	C06	7716086	2367
M772177	C06	7721775	2367
M774739	C06	7747392	2367
M776677	C06	7766774	2368
M777144	C06	7771440	2368
M777157	C06	7771571	2368
M781194	C06	7811945	2368
M782631	C06	7826312	2368
M784046	C06	7840463	2368
M784352	C06	7843521	2368
M784516	C06	7845162	2368
M786950	C06	7869504	2368
M791288	C06	7912885	2368
M792513	C06	7925137	2368
M821000	C06	8210004	2369
M821554	C06	8215549	2369
M823360	C06	8233602	2369
M824565	C06	8245657	2370
M825074	C06	8250746	2370
M826364	C06	8263642	2370
M828493	C06	8284933	2370
M830295	C06	8302952	2370
M830469	C06	8304699	2370
M831077	C06	8310772	2370
M831839	C06	8318393	2370
M832716	C06	8327162	2370
M832915	C06	8329156	2370
M833623	C06	8336232	2370
M836041	C06	8360418	2370
M837346	C06	8373463	2370
M838797	C06	8387971	2370
M839581	C06	8395812	2370
M840546	C06	8405468	2370
M844043	C06	8440439	2371
M848102	C06	8481025	2371
M854063	C06	8540639	2371
M949531	C06	9495315	2372

M951721	C06	9517214	2372
M951980	C06	9519801	2372
M952385	C06	9523856	2372
M952909	C06	9529096	2372
M999601	C06	9996013	2373
M100023	C06	10002356	2373
M100138	C06	10013894	2373
M100484	C06	10048493	2373
M100927	C06	10092778	2373
M101006	C06	10100673	2373
M101205	C06	10120546	2373
M101329	C06	10132962	2373
M101422	C06	10142231	2373
M101504	C06	10150413	2373
M101547	C06	10154764	2373
M101582	C06	10158266	2374
M101724	C06	10172463	2374
M101805	C06	10180582	2374
M101953	C06	10195312	2374
M101978	C06	10197824	2374
M101995	C06	10199500	2374
M102015	C06	10201550	2374
M102065	C06	10206561	2374
M102129	C06	10212947	2374
M102163	C06	10216330	2374
M102302	C06	10230231	2374
M102440	C06	10244076	2374
M102568	C06	10256800	2374
M102659	C06	10265959	2374
M102736	C06	10273676	2374
M102878	C06	10287809	2374
M102970	C06	10297017	2374
M103053	C06	10305304	2375
M103150	C06	10315030	2375
M103265	C06	10326534	2375
M103570	C06	10357044	2375
M103827	C06	10382785	2375
M103884	C06	10388452	2375
M104006	C06	10400685	2375
M104353	C06	10435319	2375
M104535	C06	10453577	2375
M104651	C06	10465143	2375
M104915	C06	10491585	2376
M105039	C06	10503993	2376
M105471	C06	10547117	2376
M105601	C06	10560118	2376
M105651	C06	10565128	2376

M105687	C06	10568778	2376
M105866	C06	10586681	2376
M106106	C06	10610612	2376
M106337	C06	10633787	2376
M106514	C06	10651432	2376
M106633	C06	10663351	2377
M106709	C06	10670924	2377
M106914	C06	10691438	2377
M107165	C06	10716555	2378
M108458	C06	10845803	2378
M125371	C06	12537100	2379
M125511	C06	12551124	2379
M125923	C06	12592377	2379
M126288	C06	12628891	2379
M126635	C06	12663550	2380
M127253	C06	12725326	2380
M127541	C06	12754185	2380
M128193	C06	12819340	2380
M128643	C06	12864396	2381
M128889	C06	12888982	2381
M128952	C06	12895219	2381
M129093	C06	12909344	2381
M129158	C06	12915874	2381
M129253	C06	12925383	2381
M129423	C06	12942318	2381
M130018	C06	13001870	2381
M130176	C06	13017660	2381
M130238	C06	13023885	2381
M130282	C06	13028244	2382
M130570	C06	13057010	2382
M130700	C06	13070075	2382
M130897	C06	13089792	2383
M131608	C06	13160829	2383
M132012	C06	13201217	2383
M132402	C06	13240244	2383
M133415	C06	13341533	2384
M133583	C06	13358360	2384
M133825	C06	13382534	2384
M134134	C06	13413446	2384
M134233	C06	13423363	2384
M134464	C06	13446488	2384
M134621	C06	13462170	2384
M134659	C06	13465984	2384
M135002	C06	13500255	2384
M135151	C06	13515189	2385
M135473	C06	13547337	2385
M135650	C06	13565089	2385

M135700	C06	13570052	2385
M135840	C06	13584046	2385
M136002	C06	13600280	2385
M136194	C06	13619400	2385
M136329	C06	13632927	2385
M136501	C06	13650198	2385
M136723	C06	13672311	2385
M139822	C06	13982243	2386
M139916	C06	13991613	2386
M140021	C06	14002193	2386
M140188	C06	14018855	2386
M143026	C06	14302651	2387
M143069	C06	14306991	2387
M143754	C06	14375434	2387
M148298	C06	14829896	2388
M148328	C06	14832816	2388
M149791	C06	14979199	2388
M162329	C06	16232926	2389
M162669	C06	16266910	2389
M163587	C06	16358712	2389
M173417	C06	17341781	2390
M173742	C06	17374281	2390
M188321	C06	18832180	2391
M188411	C06	18841136	2391
M189057	C06	18905700	2391
M191053	C06	19105332	2392
M191137	C06	19113715	2392
M191427	C06	19142760	2393
M191486	C06	19148681	2393
M192878	C06	19287825	2393
M193120	C06	19312059	2394
M193324	C06	19332440	2394
M193536	C06	19353666	2394
M193798	C06	19379837	2394
M193937	C06	19393765	2394
M194065	C06	19406584	2395
M194100	C06	19410014	2395
M194277	C06	19427739	2395
M194295	C06	19429555	2395
M195598	C06	19559836	2395
M202715	C06	20271503	2396
M203500	C06	20350098	2396
M204184	C06	20418484	2396
M204280	C06	20428003	2396
M205305	C06	20530590	2397
M205591	C06	20559108	2397
M205702	C06	20570294	2397

M206093	C06	20609300	2397
M206496	C06	20649684	2397
M206616	C06	20661630	2397
M206825	C06	20682523	2397
M206987	C06	20698766	2398
M208512	C06	20851240	2398
M213534	C06	21353435	2399
M213559	C06	21355981	2399
M214146	C06	21414612	2399
M214172	C06	21417246	2399
M214265	C06	21426575	2399
M214356	C06	21435668	2399
M218085	C06	21808589	2400
M218212	C06	21821201	2400
M220042	C06	22004235	2401
M220427	C06	22042716	2401
M220567	C06	22056712	2401
M220583	C06	22058366	2401
M221145	C06	22114590	2401
M221256	C06	22125645	2401
M221301	C06	22130193	2401
M221353	C06	22135359	2401
M221406	C06	22140666	2402
M221441	C06	22144112	2402
M221492	C06	22149239	2402
M221721	C06	22172176	2402
M222219	C06	22221906	2402
M222249	C06	22224944	2402
M222465	C06	22246550	2402
M222529	C06	22252990	2402
M222675	C06	22267563	2402
M222966	C06	22296650	2402
M223048	C06	22304854	2403
M223098	C06	22309829	2403
M227076	C06	22707682	2404
M227729	C06	22772934	2404
M228404	C06	22840464	2404
M234546	C06	23454627	2405
M234591	C06	23459191	2405
M234755	C06	23475537	2405
M234775	C06	23477564	2405
M234899	C06	23489936	2405
M235105	C06	23510523	2406
M235158	C06	23515819	2406
M235253	C06	23525323	2406
M235380	C06	23538087	2406
M235950	C06	23595025	2406

M236206	C06	23620606	2406
M236241	C06	23624138	2406
M236500	C06	23650051	2406
M236621	C06	23662187	2406
M238332	C06	23833298	2407
M238466	C06	23846677	2407
M238574	C06	23857487	2407
M238597	C06	23859785	2407
M238710	C06	23871040	2407
M238882	C06	23888201	2407
M239349	C06	23934992	2407
M239588	C06	23958873	2407
M239683	C06	23968342	2407
M239700	C06	23970080	2407
M239778	C06	23977805	2407
M239897	C06	23989766	2407
M239902	C06	23990264	2407
M239937	C06	23993708	2408
M240200	C06	24020023	2408
M240612	C06	24061207	2408
M240739	C06	24073936	2408
M241054	C06	24105442	2408
M241536	C06	24153669	2408
M246375	C06	24637536	2409
M247881	C06	24788129	2409
M249415	C06	24941564	2410
M249458	C06	24945870	2410
M249694	C06	24969495	2410
M250494	C06	25049469	2410
M250530	C06	25053002	2410
M250626	C06	25062686	2410
M251005	C06	25100522	2410
M252655	C06	25265596	2411
M252687	C06	25268770	2411
M253056	C06	25305685	2411
M256137	C06	25613751	2412
M256144	C06	25614471	2412
M257107	C06	25710756	2412
M257169	C06	25716936	2412
M257477	C06	25747718	2413
M258963	C06	25896363	2413
M268038	C06	26803805	2414
M268391	C06	26839188	2414
M268435	C06	26843537	2414
M269275	C06	26927517	2414
M273448	C06	27344827	2415
M273633	C06	27363370	2415

M273662	C06	27366208	2415
M273782	C06	27378213	2415
M274916	C06	27491666	2416
M275764	C06	27576406	2416
M275880	C06	27588098	2416
M275922	C06	27592246	2416
M275993	C06	27599363	2416
M276032	C06	27603238	2416
M276129	C06	27612949	2416
M276206	C06	27620662	2416
M276356	C06	27635640	2416
M276390	C06	27639004	2416
M276442	C06	27644252	2416
M276501	C06	27650139	2416
M276538	C06	27653812	2417
M276649	C06	27664948	2417
M276810	C06	27681092	2417
M276979	C06	27697983	2417
M277188	C06	27718882	2417
M278929	C06	27892901	2418
M279212	C06	27921275	2418
M279280	C06	27928036	2418
M279770	C06	27977014	2418
M279894	C06	27989404	2418
M283796	C06	28379621	2419
M283810	C06	28381043	2419
M285039	C06	28503933	2419
M285081	C06	28508162	2419
M285105	C06	28510507	2419
M285139	C06	28513964	2420
M285187	C06	28518722	2420
M285239	C06	28523939	2420
M285486	C06	28548611	2420
M285924	C06	28592485	2420
M286177	C06	28617795	2420
M286243	C06	28624338	2420
M286579	C06	28657981	2420
M286692	C06	28669216	2420
M286738	C06	28673800	2420
M291778	C06	29177800	2421
M291918	C06	29191882	2421
M291980	C06	29198005	2421
M292032	C06	29203221	2421
M308763	C06	30876392	2422
M308843	C06	30884368	2422
M309106	C06	30910659	2422
M309495	C06	30949597	2422

M309520	C06	30952064	2422
M309570	C06	30957001	2422
M309806	C06	30980681	2422
M309990	C06	30999044	2422
M310051	C06	31005127	2422
M310293	C06	31029380	2422
M310345	C06	31034504	2422
M320390	C06	32039096	2423
M320474	C06	32047452	2423
M326171	C06	32617147	2424
M326193	C06	32619301	2424
M326239	C06	32623911	2424
M326278	C06	32627848	2424
M326388	C06	32638862	2425
M326395	C06	32639552	2425
M326530	C06	32653015	2425
M326611	C06	32661152	2425
M326987	C06	32698754	2425
M327239	C06	32723932	2425
M327244	C06	32724478	2425
M327918	C06	32791821	2425
M327963	C06	32796329	2425
M332848	C06	33284870	2426
M333069	C06	33306905	2426
M333226	C06	33322634	2426
M333265	C06	33326572	2426
M333448	C06	33344849	2427
M333487	C06	33348787	2427
M334974	C06	33497461	2427
M335005	C06	33500510	2427
M335039	C06	33503982	2427
M335814	C06	33581415	2428
M335954	C06	33595466	2428
M336112	C06	33611247	2428
M336561	C06	33656108	2428
M336740	C06	33674078	2428
M337264	C06	33726436	2428
M337501	C06	33750189	2429
M337689	C06	33768940	2429
M337983	C06	33798308	2429
M338032	C06	33803256	2429
M338219	C06	33821905	2429
M338385	C06	33838543	2430
M338675	C06	33867576	2430
M339432	C06	33943278	2430
M339488	C06	33948864	2430
M339539	C06	33953975	2430

M339584	C06	33958424	2430
M339671	C06	33967183	2430
M339745	C06	33974598	2430
M339984	C06	33998427	2430
M339993	C06	33999387	2431
M340240	C06	34024092	2431
M341855	C06	34185584	2432
M343118	C06	34311802	2432
M343213	C06	34321346	2432
M343939	C06	34393970	2433
M344089	C06	34408969	2433
M344115	C06	34411588	2433
M357109	C06	35710960	2434
M357129	C06	35712916	2434
M359148	C06	35914823	2435
M359167	C06	35916793	2435
M359203	C06	35920353	2435
M359256	C06	35925654	2435
M359449	C06	35944940	2435
M359539	C06	35953957	2435
M359581	C06	35958180	2435
M362001	C06	36200118	2436
M362164	C06	36216495	2436
M362265	C06	36226598	2436
M362342	C06	36234289	2436
M362366	C06	36236646	2436
M362372	C06	36237269	2436
M362438	C06	36243896	2436
M362506	C06	36250692	2436
M362805	C06	36280518	2436
M362831	C06	36283123	2436
M362896	C06	36289644	2436
M366297	C06	36629720	2437
M367833	C06	36783388	2437
M367857	C06	36785707	2437
M368167	C06	36816764	2438
M368918	C06	36891832	2438
M368991	C06	36899155	2439
M369142	C06	36914219	2439
M369176	C06	36917602	2439
M369352	C06	36935266	2440
M369375	C06	36937552	2440
M369546	C06	36954603	2440
M369671	C06	36967153	2440
M369686	C06	36968648	2440
M369972	C06	36997218	2440
M370031	C06	37003195	2440

M370306	C06	37030681	2440
M370439	C06	37043990	2440
M370629	C06	37062903	2441
M370940	C06	37094082	2441
M373135	C06	37313529	2442
M373227	C06	37322704	2442
M373247	C06	37324796	2442
M373899	C06	37389925	2442
M373985	C06	37398518	2443
M374794	C06	37479448	2443
M375213	C06	37521382	2443
M375255	C06	37525518	2443
M375344	C06	37534404	2443
M375386	C06	37538649	2443
M376683	C06	37668364	2444
M376784	C06	37678463	2444
M379153	C06	37915394	2445
M379210	C06	37921048	2445
M379254	C06	37925494	2445
M379458	C06	37945862	2446
M380177	C06	38017780	2446
M381456	C06	38145678	2447
M381571	C06	38157108	2447
M382944	C06	38294450	2447
M383032	C06	38303244	2447
M384432	C06	38443286	2448
M384595	C06	38459572	2448
M385198	C06	38519873	2448
M385219	C06	38521939	2448
M385257	C06	38525781	2448
M385293	C06	38529375	2448
M385377	C06	38537724	2448
M385391	C06	38539150	2449
M385476	C06	38547698	2449
M385504	C06	38550487	2450
M385665	C06	38566568	2450
M385712	C06	38571261	2450
M386358	C06	38635858	2451
M386393	C06	38639385	2451
M386462	C06	38646202	2451
M386842	C06	38684274	2452
M386868	C06	38686820	2452
M387147	C06	38714777	2452
M387380	C06	38738044	2453
M387703	C06	38770320	2453
M387794	C06	38779441	2453
M387896	C06	38789663	2453

M387947	C06	38794714	2453
M387972	C06	38797234	2453
M388594	C06	38859439	2453
M388707	C06	38870744	2453
M389140	C06	38914068	2454
M389176	C06	38917654	2454
M389282	C06	38928204	2454
M389351	C06	38935161	2454
M389539	C06	38953901	2455
M389606	C06	38960643	2455
M389630	C06	38963027	2455
M389664	C06	38966482	2455
M389716	C06	38971603	2455
M389868	C06	38986886	2455
M389888	C06	38988856	2455
M389939	C06	38993969	2455
M389970	C06	38997016	2455
M390149	C06	39014912	2455
M390880	C06	39088046	2455
M392098	C06	39209826	2456
M392158	C06	39215886	2456
M392412	C06	39241232	2457
M392507	C06	39250798	2457
M392678	C06	39267804	2457
M393465	C06	39346578	2457
M394000	C06	39400089	2457
M402053	C06	40205325	2458
M402124	C06	40212423	2458
M402163	C06	40216343	2459
M402180	C06	40218054	2459
M402215	C06	40221563	2459
M402401	C06	40240114	2459
M402401	C06	40240167	2459
M402490	C06	40249003	2459
M402558	C06	40255843	2459
M163514	C07	1635144	2460
M169613	C07	1696137	2460
M719770	C07	7197702	2461
M722844	C07	7228446	2461
M102679	C07	10267918	2462
M103341	C07	10334159	2462
M111382	C07	11138272	2463
M112320	C07	11232043	2463
M112474	C07	11247488	2464
M113538	C07	11353864	2464
M113948	C07	11394805	2464
M114541	C07	11454141	2465

M114970	C07	11497034	2465
M122205	C07	12220548	2466
M122937	C07	12293701	2466
M123000	C07	12300081	2466
M123126	C07	12312681	2466
M123278	C07	12327843	2466
M123573	C07	12357349	2466
M123612	C07	12361216	2466
M123655	C07	12365557	2466
M123710	C07	12371050	2466
M123760	C07	12376089	2466
M123827	C07	12382781	2467
M123908	C07	12390854	2467
M124093	C07	12409342	2467
M124275	C07	12427576	2468
M124702	C07	12470280	2468
M125544	C07	12554433	2469
M125836	C07	12583672	2469
M126005	C07	12600595	2469
M126013	C07	12601322	2469
M126111	C07	12611126	2470
M126253	C07	12625385	2470
M126375	C07	12637599	2471
M126496	C07	12649653	2471
M126798	C07	12679809	2471
M127219	C07	12721990	2471
M127325	C07	12732531	2471
M127469	C07	12746961	2471
M127649	C07	12764911	2471
M127675	C07	12767547	2471
M127791	C07	12779166	2471
M129340	C07	12934061	2472
M129772	C07	12977289	2472
M130351	C07	13035119	2473
M130374	C07	13037415	2473
M130541	C07	13054197	2473
M130912	C07	13091228	2474
M131006	C07	13100628	2474
M131226	C07	13122646	2474
M131322	C07	13132262	2474
M131479	C07	13147934	2475
M131732	C07	13173287	2475
M131917	C07	13191732	2476
M132045	C07	13204549	2476
M146429	C07	14642921	2477
M146565	C07	14656563	2477
M150470	C07	15047059	2478

M151192	C07	15119285	2478
M181540	C07	18154007	2479
M181610	C07	18161001	2479
M187711	C07	18771154	2480
M188922	C07	18892225	2480
M192143	C07	19214300	2481
M192540	C07	19254046	2481
M193046	C07	19304626	2481
M193151	C07	19315117	2481
M193318	C07	19331834	2481
M222478	C07	22247841	2482
M224066	C07	22406639	2482
M226864	C07	22686464	2483
M226998	C07	22699831	2483
M227437	C07	22743770	2483
M227915	C07	22791553	2484
M227988	C07	22798820	2484
M228087	C07	22808760	2485
M228133	C07	22813382	2485
M228193	C07	22819311	2485
M228216	C07	22821622	2486
M228273	C07	22827385	2486
M228539	C07	22853929	2486
M228853	C07	22885308	2486
M228881	C07	22888168	2486
M228913	C07	22891303	2486
M228947	C07	22894748	2486
M228977	C07	22897753	2486
M229012	C07	22901227	2486
M229042	C07	22904283	2486
M229393	C07	22939304	2486
M229571	C07	22957107	2486
M229597	C07	22959738	2486
M229736	C07	22973631	2486
M229806	C07	22980698	2486
M229888	C07	22988818	2487
M229901	C07	22990118	2487
M229929	C07	22992917	2487
M229964	C07	22996428	2487
M230046	C07	23004663	2487
M230278	C07	23027897	2487
M230322	C07	23032227	2487
M230384	C07	23038413	2487
M230507	C07	23050762	2487
M230740	C07	23074047	2487
M230848	C07	23084807	2487
M230923	C07	23092342	2487

M230954	C07	23095463	2487
M231241	C07	23124106	2487
M235120	C07	23512043	2488
M235214	C07	23521455	2488
M235506	C07	23550680	2488
M236471	C07	23647150	2488
M244132	C07	24413203	2489
M244296	C07	24429610	2489
M244725	C07	24472516	2489
M245677	C07	24567734	2489
M245776	C07	24577600	2490
M245914	C07	24591470	2490
M246066	C07	24606608	2490
M246133	C07	24613395	2490
M246211	C07	24621182	2491
M246456	C07	24645679	2491
M246694	C07	24669465	2491
M246793	C07	24679328	2491
M247043	C07	24704316	2491
M247283	C07	24728340	2491
M247381	C07	24738111	2491
M247420	C07	24742059	2491
M247612	C07	24761261	2491
M247662	C07	24766244	2491
M247810	C07	24781068	2491
M247995	C07	24799575	2492
M248036	C07	24803661	2492
M248129	C07	24812975	2492
M248194	C07	24819497	2492
M251298	C07	25129878	2493
M252090	C07	25209086	2493
M252856	C07	25285685	2493
M264684	C07	26468465	2494
M264797	C07	26479717	2494
M264923	C07	26492369	2494
M280164	C07	28016466	2495
M280759	C07	28075994	2495
M285791	C07	28579110	2496
M285857	C07	28585762	2496
M285887	C07	28588703	2496
M286103	C07	28610375	2496
M286130	C07	28613015	2496
M286154	C07	28615446	2496
M286309	C07	28630981	2496
M288862	C07	28886251	2497
M288998	C07	28899899	2497
M289117	C07	28911753	2497

M289236	C07	28923608	2497
M289382	C07	28938211	2497
M289571	C07	28957171	2497
M289766	C07	28976655	2497
M289845	C07	28984519	2497
M289981	C07	28998130	2497
M305258	C07	30525869	2498
M305273	C07	30527328	2498
M305374	C07	30537496	2498
M305502	C07	30550273	2498
M305619	C07	30561923	2498
M306082	C07	30608266	2499
M306149	C07	30614912	2499
M307416	C07	30741678	2499
M307479	C07	30747994	2499
M307646	C07	30764620	2499
M309523	C07	30952306	2500
M309703	C07	30970374	2500
M309874	C07	30987483	2500
M309987	C07	30998770	2500
M311781	C07	31178186	2501
M312676	C07	31267658	2501
M312753	C07	31275333	2501
M312810	C07	31281073	2502
M313043	C07	31304333	2502
M314036	C07	31403654	2502
M314386	C07	31438615	2502
M315820	C07	31582075	2503
M315951	C07	31595131	2503
M316079	C07	31607900	2503
M316150	C07	31615030	2504
M316212	C07	31621260	2504
M316252	C07	31625211	2504
M317218	C07	31721836	2504
M317314	C07	31731406	2504
M317514	C07	31751464	2504
M317538	C07	31753802	2504
M317607	C07	31760712	2504
M317749	C07	31774980	2504
M317824	C07	31782498	2505
M318001	C07	31800136	2505
M318093	C07	31809384	2505
M318095	C07	31809596	2505
M318172	C07	31817293	2505
M318247	C07	31824733	2505
M318257	C07	31825795	2505
M318408	C07	31840838	2505

M318444	C07	31844460	2505
M318499	C07	31849976	2505
M333702	C07	33370226	2506
M333719	C07	33371992	2506
M333778	C07	33377834	2506
M333819	C07	33381922	2506
M333835	C07	33383568	2506
M333880	C07	33388021	2506
M333900	C07	33390078	2506
M335559	C07	33555953	2507
M335603	C07	33560342	2507
M335644	C07	33564466	2507
M335664	C07	33566482	2507
M335757	C07	33575721	2507
M335874	C07	33587427	2507
M335919	C07	33591935	2507
M335934	C07	33593491	2507
M335998	C07	33599827	2507
M336117	C07	33611780	2507
M336370	C07	33637028	2508
M337772	C07	33777201	2508
M340150	C07	34015002	2509
M340162	C07	34016216	2509
M341367	C07	34136789	2509
M350164	C07	35016454	2510
M350187	C07	35018739	2510
M350210	C07	35021039	2510
M351883	C07	35188306	2511
M352810	C07	35281025	2511
M352859	C07	35285905	2511
M352959	C07	35295978	2511
M353080	C07	35308011	2511
M353144	C07	35314466	2511
M353184	C07	35318403	2511
M353206	C07	35320681	2511
M353357	C07	35335786	2511
M353624	C07	35362464	2512
M353684	C07	35368407	2512
M353712	C07	35371283	2512
M353850	C07	35385013	2513
M353873	C07	35387370	2513
M354852	C07	35485286	2513
M354865	C07	35486576	2513
M354983	C07	35498300	2513
M355026	C07	35502667	2513
M355166	C07	35516618	2513
M355440	C07	35544091	2513

M355522	C07	35552211	2514
M355696	C07	35569626	2514
M355771	C07	35577164	2515
M356642	C07	35664215	2515
M356741	C07	35674183	2516
M356843	C07	35684327	2516
M356876	C07	35687652	2516
M356908	C07	35690892	2516
M357109	C07	35710939	2517
M357140	C07	35714052	2517
M357176	C07	35717649	2517
M357293	C07	35729374	2517
M357445	C07	35744520	2517
M357454	C07	35745435	2517
M357600	C07	35760066	2517
M357618	C07	35761801	2517
M357657	C07	35765744	2517
M358401	C07	35840147	2517
M358423	C07	35842320	2517
M358708	C07	35870877	2517
M363026	C07	36302643	2518
M363047	C07	36304798	2518
M363993	C07	36399374	2518
M364469	C07	36446929	2518
M364571	C07	36457146	2518
M364585	C07	36458592	2518
M364701	C07	36470161	2519
M364729	C07	36472940	2519
M364814	C07	36481428	2519
M364818	C07	36481846	2519
M365424	C07	36542414	2519
M365478	C07	36547879	2519
M367259	C07	36725958	2520
M367328	C07	36732817	2520
M367388	C07	36738831	2520
M367431	C07	36743154	2521
M367468	C07	36746847	2521
M369005	C07	36900559	2521
M369027	C07	36902717	2521
M369099	C07	36909925	2522
M369153	C07	36915346	2522
M371557	C07	37155799	2523
M372571	C07	37257189	2523
M372619	C07	37261981	2523
M372755	C07	37275557	2523
M372789	C07	37278969	2523
M372942	C07	37294246	2523

M372960	C07	37296081	2523
M374734	C07	37473401	2524
M375784	C07	37578495	2524
M380635	C07	38063512	2525
M380656	C07	38065654	2525
M380764	C07	38076472	2525
M380996	C07	38099661	2525
M381079	C07	38107995	2525
M381159	C07	38115912	2525
M381320	C07	38132036	2525
M381687	C07	38168724	2525
M381711	C07	38171140	2525
M381770	C07	38177058	2525
M381787	C07	38178764	2525
M381903	C07	38190320	2526
M382058	C07	38205850	2526
M382213	C07	38221313	2526
M382288	C07	38228838	2526
M393042	C07	39304290	2527
M393085	C07	39308540	2527
M393096	C07	39309652	2527
M393152	C07	39315258	2527
M393223	C07	39322354	2527
M393356	C07	39335630	2528
M393640	C07	39364027	2528
M393675	C07	39367521	2528
M393732	C07	39373280	2528
M393737	C07	39373724	2528
M393845	C07	39384501	2528
M393865	C07	39386527	2528
M393906	C07	39390625	2528
M393981	C07	39398132	2528
M395769	C07	39576905	2529
M395804	C07	39580406	2529
M395992	C07	39599215	2530
M396146	C07	39614680	2530
M396190	C07	39619005	2530
M396265	C07	39626513	2530
M396337	C07	39633700	2530
M398244	C07	39824438	2531
M398256	C07	39825612	2531
M398396	C07	39839660	2531
M398482	C07	39848212	2531
M399630	C07	39963039	2531
M399638	C07	39963889	2531
M399787	C07	39978741	2531
M399794	C07	39979437	2531

M399820	C07	39982027	2531
M399874	C07	39987474	2532
M399922	C07	39992223	2532
M399966	C07	39996607	2532
M401174	C07	40117448	2532
M401245	C07	40124528	2532
M401293	C07	40129367	2532
M401333	C07	40133344	2532
M402202	C07	40220298	2533
M402256	C07	40225687	2533
M402390	C07	40239088	2533
M402494	C07	40249422	2533
M402575	C07	40257546	2534
M403142	C07	40314276	2534
M403304	C07	40330409	2534
M403440	C07	40344079	2534
M404144	C07	40414461	2534
M408189	C07	40818944	2535
M409573	C07	40957322	2535
M409751	C07	40975184	2535
M409787	C07	40978767	2535
M409831	C07	40983114	2536
M409933	C07	40993317	2536
M410030	C07	41003016	2536
M410056	C07	41005603	2536
M410172	C07	41017299	2536
M410289	C07	41028926	2536
M410310	C07	41031029	2537
M410500	C07	41050076	2537
M410556	C07	41055635	2538
M410588	C07	41058825	2538
M410704	C07	41070481	2538
M410721	C07	41072199	2538
M410811	C07	41081127	2538
M410913	C07	41091314	2538
M410931	C07	41093194	2538
M411009	C07	41100957	2539
M411230	C07	41123054	2539
M411290	C07	41129006	2539
M411339	C07	41133979	2539
M411963	C07	41196380	2539
M413182	C07	41318214	2540
M413275	C07	41327525	2540
M413335	C07	41333565	2540
M413456	C07	41345662	2540
M413511	C07	41351168	2540
M417173	C07	41717339	2541

M417273	C07	41727391	2541
M417320	C07	41732029	2541
M417820	C07	41782036	2541
M418154	C07	41815457	2542
M418176	C07	41817661	2542
M418239	C07	41823976	2542
M418278	C07	41827811	2542
M418373	C07	41837369	2542
M418464	C07	41846450	2542
M418474	C07	41847425	2542
M418634	C07	41863414	2542
M418730	C07	41873083	2542
M418847	C07	41884797	2542
M418964	C07	41896475	2542
M419091	C07	41909162	2542
M419103	C07	41910321	2542
M419154	C07	41915458	2542
M419220	C07	41922028	2542
M419240	C07	41924020	2542
M419291	C07	41929118	2542
M419351	C07	41935195	2542
M419493	C07	41949377	2542
M419611	C07	41961144	2542
M419650	C07	41965039	2542
M419880	C07	41988069	2543
M419901	C07	41990189	2543
M419962	C07	41996227	2543
M420142	C07	42014245	2544
M420429	C07	42042986	2544
M421638	C07	42163888	2544
M426073	C07	42607342	2545
M426210	C07	42621065	2545
M426255	C07	42625595	2545
M427072	C07	42707266	2545
M427229	C07	42722912	2545
M427568	C07	42756895	2546
M427600	C07	42760098	2546
M427632	C07	42763264	2546
M428590	C07	42859005	2546
M428717	C07	42871778	2546
M428753	C07	42875317	2546
M428797	C07	42879745	2546
M429086	C07	42908683	2546
M429158	C07	42915872	2546
M429337	C07	42933789	2547
M429345	C07	42934567	2547
M429716	C07	42971692	2547

M429884	C07	42988416	2547
M430294	C07	43029446	2547
M432453	C07	43245349	2548
M432667	C07	43266740	2548
M432738	C07	43273843	2548
M432860	C07	43286005	2548
M432917	C07	43291739	2548
M432984	C07	43298479	2548
M436203	C07	43620366	2549
M436502	C07	43650278	2549
M438169	C07	43816934	2550
M438267	C07	43826744	2550
M438410	C07	43841064	2551
M438685	C07	43868534	2551
M439893	C07	43989391	2551
M440374	C07	44037492	2552
M440405	C07	44040585	2552
M441208	C07	44120811	2552
M441234	C07	44123421	2552
M441339	C07	44133928	2552
M441813	C07	44181370	2553
M441854	C07	44185467	2553
M441886	C07	44188625	2554
M441944	C07	44194409	2554
M441970	C07	44197020	2554
M442024	C07	44202449	2554
M442118	C07	44211843	2554
M442236	C07	44223630	2555
M442244	C07	44224476	2555
M442283	C07	44228331	2555
M442291	C07	44229190	2555
M442449	C07	44244946	2555
M442469	C07	44246931	2555
M442503	C07	44250392	2555
M442550	C07	44255005	2555
M442565	C07	44256508	2555
M442614	C07	44261445	2555
M442641	C07	44264140	2555
M443797	C07	44379797	2555
M444855	C07	44485571	2556
M444923	C07	44492330	2556
M444952	C07	44495276	2556
M445012	C07	44501278	2556
M445084	C07	44508433	2557
M445168	C07	44516814	2557
M445423	C07	44542318	2557
M445440	C07	44544000	2557

M446314	C07	44631450	2557
M446624	C07	44662438	2557
M446643	C07	44664354	2557
M446681	C07	44668118	2557
M446707	C07	44670769	2558
M446713	C07	44671335	2558
M446945	C07	44694525	2559
M447013	C07	44701396	2559
M447198	C07	44719888	2559
M447282	C07	44728227	2559
M449541	C07	44954195	2560
M451000	C07	45100035	2560
M451069	C07	45106989	2560
M451225	C07	45122568	2561
M451240	C07	45124011	2561
M453521	C07	45352110	2562
M453594	C07	45359423	2562
M453629	C07	45362981	2562
M453760	C07	45376057	2562
M453999	C07	45399935	2563
M454278	C07	45427871	2563
M454301	C07	45430140	2564
M454552	C07	45455240	2564
M454582	C07	45458295	2564
M454963	C07	45496339	2564
M455018	C07	45501872	2564
M455556	C07	45555607	2564
M455779	C07	45577975	2564
M455879	C07	45587911	2564
M460142	C07	46014207	2565
M460262	C07	46026260	2565
M460511	C07	46051192	2565
M460674	C07	46067497	2565
M462956	C07	46295695	2566
M462993	C07	46299335	2566
M463854	C07	46385401	2566
M463865	C07	46386526	2566
M463934	C07	46393449	2566
M464038	C07	46403832	2566
M464447	C07	46444735	2566
M464554	C07	46455433	2566
M464748	C07	46474863	2567
M465699	C07	46569940	2567
M466379	C07	46637983	2568
M467597	C07	46759702	2568
M467676	C07	46767659	2569
M467701	C07	46770116	2569

M467759	C07	46775931	2570
M467833	C07	46783331	2570
M468369	C07	46836915	2571
M468533	C07	46853341	2571
M468565	C07	46856503	2571
M469396	C07	46939684	2572
M469528	C07	46952803	2572
M471290	C07	47129098	2573
M471894	C07	47189415	2573
M471937	C07	47193724	2573
M472028	C07	47202810	2573
M472050	C07	47205097	2573
M472056	C07	47205692	2573
M472094	C07	47209420	2573
M472611	C07	47261151	2574
M472704	C07	47270414	2574
M472763	C07	47276313	2574
M472797	C07	47279700	2575
M472900	C07	47290097	2575
M472939	C07	47293991	2575
M473988	C07	47398867	2576
M474070	C07	47407047	2576
M474385	C07	47438549	2576
M474395	C07	47439560	2576
M474674	C07	47467426	2576
M474924	C07	47492421	2576
M474974	C07	47497416	2576
M475133	C07	47513343	2576
M475286	C07	47528622	2577
M475367	C07	47536720	2577
M475580	C07	47558047	2578
M475722	C07	47572232	2578
M475756	C07	47575636	2578
M477068	C07	47706833	2578
M477144	C07	47714436	2578
M477249	C07	47724932	2579
M477256	C07	47725636	2579
M477795	C07	47779517	2579
M488712	C07	48871250	2580
M489046	C07	48904690	2580
M489258	C07	48925807	2581
M489456	C07	48945606	2581
M489493	C07	48949398	2581
M489580	C07	48958078	2582
M489625	C07	48962547	2582
M489669	C07	48966991	2582
M489710	C07	48971079	2582

M489783	C07	48978318	2582
M489836	C07	48983638	2582
M489856	C07	48985608	2582
M489885	C07	48988547	2582
M489960	C07	48996044	2582
M434753	C08	434753	2583
M488082	C08	488082	2583
M319101	C08	3191013	2584
M331967	C08	3319678	2584
M519454	C08	5194544	2585
M533840	C08	5338405	2585
M537521	C08	5375218	2586
M543359	C08	5433593	2586
M578458	C08	5784585	2587
M591020	C08	5910205	2587
M639764	C08	6397643	2588
M651785	C08	6517858	2588
M655189	C08	6551891	2588
M677087	C08	6770879	2589
M692763	C08	6927637	2589
M730426	C08	7304264	2590
M740314	C08	7403147	2590
M762102	C08	7621021	2591
M766137	C08	7661372	2591
M783311	C08	7833113	2592
M786211	C08	7862115	2592
M799096	C08	7990962	2593
M799556	C08	7995569	2593
M810961	C08	8109618	2593
M952467	C08	9524678	2594
M962793	C08	9627935	2594
M972653	C08	9726531	2595
M979632	C08	9796326	2595
M106647	C08	10664703	2596
M107470	C08	10747021	2596
M117854	C08	11785421	2597
M118608	C08	11860836	2597
M119291	C08	11929118	2597
M121421	C08	12142124	2598
M122162	C08	12216249	2598
M122883	C08	12288380	2598
M133660	C08	13366015	2599
M134847	C08	13484746	2599
M135143	C08	13514300	2599
M186520	C08	18652038	2600
M186686	C08	18668695	2600
M186917	C08	18691785	2600

M187060	C08	18706078	2601
M187235	C08	18723542	2601
M187572	C08	18757283	2601
M187665	C08	18766500	2601
M187771	C08	18777182	2601
M187909	C08	18790977	2602
M188005	C08	18800539	2602
M188030	C08	18803035	2602
M188209	C08	18820963	2602
M188278	C08	18827812	2603
M188465	C08	18846519	2603
M188553	C08	18855313	2603
M188685	C08	18868577	2603
M188714	C08	18871476	2603
M192251	C08	19225168	2604
M192580	C08	19258099	2604
M194133	C08	19413319	2605
M194233	C08	19423319	2605
M194283	C08	19428328	2605
M194413	C08	19441399	2606
M194455	C08	19445563	2606
M194527	C08	19452712	2606
M194620	C08	19462011	2606
M194931	C08	19493141	2606
M207975	C08	20797571	2607
M209309	C08	20930920	2607
M226085	C08	22608544	2608
M227332	C08	22733299	2608
M227406	C08	22740605	2608
M227446	C08	22744642	2608
M227467	C08	22746759	2608
M227540	C08	22754049	2609
M227681	C08	22768138	2609
M227718	C08	22771811	2610
M227805	C08	22780528	2610
M227866	C08	22786604	2611
M227875	C08	22787582	2611
M227919	C08	22791995	2611
M227974	C08	22797485	2611
M227982	C08	22798290	2611
M228171	C08	22817104	2611
M232275	C08	23227522	2612
M232297	C08	23229761	2612
M232562	C08	23256227	2612
M232622	C08	23262201	2612
M232863	C08	23286351	2612
M233159	C08	23315900	2612

M233534	C08	23353473	2613
M235072	C08	23507248	2613
M235088	C08	23508822	2613
M235317	C08	23531767	2614
M235552	C08	23555263	2614
M235601	C08	23560125	2614
M236006	C08	23600675	2614
M236020	C08	23602089	2614
M240223	C08	24022325	2615
M240273	C08	24027356	2615
M240380	C08	24038065	2616
M240419	C08	24041989	2616
M240658	C08	24065818	2617
M240738	C08	24073877	2617
M248729	C08	24872986	2618
M248731	C08	24873155	2618
M248782	C08	24878202	2618
M248847	C08	24884785	2618
M248938	C08	24893869	2618
M249012	C08	24901231	2618
M249133	C08	24913313	2619
M249174	C08	24917435	2619
M258329	C08	25832931	2620
M258340	C08	25834069	2620
M258448	C08	25844871	2620
M259184	C08	25918486	2621
M259215	C08	25921551	2621
M259257	C08	25925756	2621
M259324	C08	25932487	2621
M260116	C08	26011655	2622
M260522	C08	26052205	2622
M260703	C08	26070374	2622
M260958	C08	26095836	2622
M261110	C08	26111071	2622
M261220	C08	26122023	2622
M261705	C08	26170550	2622
M267391	C08	26739158	2623
M267435	C08	26743598	2623
M267557	C08	26755796	2624
M267578	C08	26757873	2624
M267922	C08	26792277	2625
M267998	C08	26799840	2625
M268135	C08	26813508	2625
M268214	C08	26821497	2625
M268464	C08	26846487	2625
M268578	C08	26857856	2625
M268758	C08	26875834	2625

M268779	C08	26877987	2625
M268947	C08	26894785	2625
M269125	C08	26912599	2625
M269281	C08	26928143	2625
M269324	C08	26932400	2625
M269393	C08	26939328	2625
M269492	C08	26949279	2625
M269610	C08	26961002	2626
M269699	C08	26969970	2626
M269826	C08	26982614	2627
M269884	C08	26988465	2627
M269935	C08	26993538	2627
M270079	C08	27007928	2627
M270272	C08	27027236	2627
M270525	C08	27052599	2627
M274150	C08	27415067	2628
M275381	C08	27538138	2628
M275472	C08	27547201	2628
M275865	C08	27586579	2629
M275873	C08	27587392	2629
M275923	C08	27592372	2630
M275989	C08	27598928	2630
M276007	C08	27600772	2630
M276041	C08	27604193	2631
M276091	C08	27609140	2631
M276354	C08	27635417	2632
M276412	C08	27641288	2632
M276495	C08	27649576	2632
M276589	C08	27658957	2632
M277859	C08	27785928	2632
M287006	C08	28700656	2633
M287384	C08	28738463	2633
M287644	C08	28764455	2633
M287673	C08	28767335	2633
M287696	C08	28769616	2633
M287917	C08	28791778	2633
M287955	C08	28795599	2633
M288107	C08	28810774	2633
M288301	C08	28830176	2633
M288411	C08	28841133	2633
M288435	C08	28843548	2633
M289064	C08	28906470	2634
M289161	C08	28916173	2634
M289242	C08	28924257	2634
M289415	C08	28941507	2634
M289479	C08	28947917	2634
M296870	C08	29687036	2635

M297180	C08	29718061	2635
M297267	C08	29726719	2636
M297305	C08	29730528	2636
M297383	C08	29738353	2636
M297482	C08	29748288	2637
M297536	C08	29753688	2637
M297553	C08	29755385	2638
M297601	C08	29760158	2638
M298652	C08	29865250	2639
M299612	C08	29961243	2639
M299927	C08	29992712	2639
M300097	C08	30009718	2639
M300146	C08	30014607	2640
M300165	C08	30016501	2640
M300296	C08	30029662	2641
M300414	C08	30041455	2641
M301108	C08	30110818	2642
M301138	C08	30113800	2642
M301170	C08	30117052	2642
M301264	C08	30126420	2642
M301401	C08	30140136	2642
M301988	C08	30198840	2643
M302100	C08	30210035	2643
M302395	C08	30239562	2643
M302462	C08	30246264	2643
M302554	C08	30255460	2643
M302567	C08	30256725	2643
M303016	C08	30301612	2643
M305539	C08	30553975	2644
M306011	C08	30601190	2644
M306066	C08	30606645	2644
M306960	C08	30696076	2644
M306991	C08	30699105	2644
M307107	C08	30710732	2644
M307853	C08	30785329	2645
M307863	C08	30786352	2645
M307898	C08	30789857	2645
M311344	C08	31134482	2646
M312221	C08	31222152	2646
M312244	C08	31224400	2646
M312365	C08	31236583	2646
M312370	C08	31237035	2646
M312601	C08	31260104	2646
M312793	C08	31279381	2646
M312864	C08	31286443	2646
M312881	C08	31288109	2646
M313182	C08	31318234	2647

M314777	C08	31477779	2647
M314864	C08	31486439	2648
M315001	C08	31500174	2648
M315190	C08	31519007	2648
M315204	C08	31520489	2648
M315548	C08	31554844	2648
M316366	C08	31636627	2648
M317626	C08	31762610	2649
M317650	C08	31765053	2649
M318600	C08	31860047	2649
M323314	C08	32331479	2650
M323345	C08	32334537	2650
M324780	C08	32478033	2651
M325400	C08	32540097	2651
M326318	C08	32631847	2651
M326486	C08	32648604	2652
M326599	C08	32659940	2652
M326956	C08	32695662	2652
M327299	C08	32729927	2653
M327449	C08	32744912	2653
M327516	C08	32751677	2654
M327559	C08	32755919	2654
M327592	C08	32759252	2655
M327691	C08	32769125	2655
M327739	C08	32773962	2655
M327981	C08	32798111	2656
M328099	C08	32809951	2656
M328162	C08	32816241	2656
M328199	C08	32819991	2657
M328540	C08	32854022	2657
M331891	C08	33189197	2658
M331963	C08	33196343	2658
M332011	C08	33201122	2658
M332144	C08	33214471	2659
M332197	C08	33219738	2659
M332253	C08	33225351	2660
M332449	C08	33244910	2660
M332721	C08	33272184	2660
M341335	C08	34133527	2661
M341780	C08	34178086	2661
M343308	C08	34330855	2662
M343515	C08	34351575	2662
M343813	C08	34381318	2662
M344505	C08	34450556	2662
M344788	C08	34478829	2662
M345313	C08	34531324	2663
M345411	C08	34541105	2663

M345585	C08	34558546	2663
M345619	C08	34561970	2663
M345665	C08	34566502	2663
M345731	C08	34573186	2663
M345754	C08	34575473	2663
M346190	C08	34619096	2663
M346249	C08	34624974	2663
M347720	C08	34772053	2664
M347896	C08	34789610	2664
M351748	C08	35174869	2665
M351804	C08	35180460	2665
M351872	C08	35187276	2666
M351961	C08	35196147	2666
M352132	C08	35213241	2667
M352173	C08	35217325	2667
M352956	C08	35295641	2667
M353594	C08	35359440	2667
M354091	C08	35409148	2668
M354130	C08	35413045	2668
M355914	C08	35591484	2669
M357239	C08	35723944	2669
M365660	C08	36566004	2670
M366640	C08	36664074	2670
M369069	C08	36906948	2671
M369145	C08	36914543	2671
M369218	C08	36921833	2672
M369260	C08	36926000	2672
M369341	C08	36934155	2672
M369431	C08	36943147	2673
M369469	C08	36946976	2673
M369509	C08	36950931	2674
M369589	C08	36958962	2674
M369681	C08	36968128	2675
M369721	C08	36972144	2675
M369886	C08	36988668	2675
M370144	C08	37014404	2676
M370263	C08	37026328	2676
M370296	C08	37029659	2676
M370380	C08	37038010	2676
M370447	C08	37044707	2676
M370475	C08	37047534	2676
M370499	C08	37049936	2676
M370572	C08	37057237	2677
M370698	C08	37069878	2677
M370782	C08	37078265	2677
M370986	C08	37098660	2678
M371022	C08	37102277	2678

M371433	C08	37143363	2679
M371476	C08	37147648	2679
M371729	C08	37172907	2680
M371747	C08	37174702	2680
M371794	C08	37179435	2680
M371827	C08	37182714	2681
M371868	C08	37186896	2681
M371929	C08	37192985	2681
M372160	C08	37216084	2682
M372250	C08	37225006	2682
M372319	C08	37231948	2682
M374804	C08	37480497	2683
M375001	C08	37500168	2683
M375027	C08	37502748	2684
M375544	C08	37554440	2684
M379596	C08	37959676	2685
M379714	C08	37971410	2685
M379755	C08	37975589	2685
M380452	C08	38045200	2686
M380511	C08	38051110	2686
M381008	C08	38100819	2687
M381074	C08	38107442	2687
M381607	C08	38160712	2687
M381648	C08	38164810	2687
M383407	C08	38340718	2688
M383442	C08	38344299	2688
M383481	C08	38348111	2689
M383535	C08	38353584	2689
M383787	C08	38378739	2690
M384911	C08	38491191	2690
M384924	C08	38492405	2690
M385055	C08	38505566	2691
M385098	C08	38509841	2691
M385277	C08	38527799	2691
M387852	C08	38785266	2692
M388378	C08	38837897	2692
M388590	C08	38859061	2692
M388786	C08	38878609	2692
M393783	C08	39378369	2693
M393901	C08	39390164	2693
M394060	C08	39406043	2694
M394089	C08	39408960	2694
M394110	C08	39411086	2694
M394154	C08	39415451	2694
M394171	C08	39417124	2694
M397140	C08	39714030	2695
M398185	C08	39818586	2695

M398305	C08	39830519	2695
M398418	C08	39841803	2696
M398512	C08	39851212	2696
M398681	C08	39868193	2697
M399672	C08	39967218	2697
M399780	C08	39978036	2697
M402442	C08	40244204	2698
M402483	C08	40248319	2698
M407566	C08	40756603	2699
M408079	C08	40807908	2699
M408801	C08	40880181	2699
M408821	C08	40882192	2700
M408832	C08	40883223	2700
M415349	C08	41534993	2701
M416171	C08	41617189	2701
M416221	C08	41622125	2701
M416272	C08	41627237	2701
M416284	C08	41628494	2701
M416395	C08	41639514	2701
M416433	C08	41643371	2701
M416524	C08	41652477	2701
M416658	C08	41665811	2702
M416673	C08	41667304	2702
M422449	C08	42244923	2703
M423543	C08	42354378	2703
M423684	C08	42368454	2704
M425001	C08	42500148	2704
M426062	C08	42606230	2705
M426575	C08	42657581	2705
M426703	C08	42670373	2706
M426776	C08	42677655	2706
M428202	C08	42820271	2707
M428271	C08	42827161	2707
M428279	C08	42827927	2708
M428338	C08	42833816	2708
M428391	C08	42839123	2709
M428526	C08	42852616	2709
M428555	C08	42855526	2710
M428605	C08	42860574	2710
M428740	C08	42874085	2711
M428859	C08	42885912	2711
M428885	C08	42888599	2711
M429234	C08	42923498	2712
M429350	C08	42935005	2712
M429393	C08	42939366	2713
M429573	C08	42957383	2713
M429746	C08	42974632	2713

M430057	C08	43005769	2714
M430180	C08	43018079	2714
M229684	C09	229684	2715
M279571	C09	279571	2715
M281752	C09	281752	2715
M290032	C09	290032	2715
M350345	C09	350345	2715
M116159	C09	1161594	2716
M116874	C09	1168744	2716
M117071	C09	1170718	2717
M127652	C09	1276521	2717
M127868	C09	1278684	2718
M128044	C09	1280444	2718
M161996	C09	1619965	2719
M164767	C09	1647673	2719
M165540	C09	1655404	2719
M165781	C09	1657811	2719
M203199	C09	2031997	2720
M204378	C09	2043787	2720
M205135	C09	2051354	2720
M226955	C09	2269554	2721
M229305	C09	2293058	2721
M229750	C09	2297507	2721
M231003	C09	2310033	2721
M232485	C09	2324855	2721
M237400	C09	2374007	2722
M240583	C09	2405839	2722
M240819	C09	2408195	2722
M242383	C09	2423839	2722
M242691	C09	2426916	2722
M243142	C09	2431422	2722
M243606	C09	2436066	2722
M243955	C09	2439554	2722
M244392	C09	2443926	2722
M244709	C09	2447093	2722
M244986	C09	2449864	2722
M250753	C09	2507533	2722
M251068	C09	2510681	2722
M267760	C09	2677608	2723
M267858	C09	2678580	2723
M325786	C09	3257862	2724
M326690	C09	3266903	2724
M327314	C09	3273148	2724
M328479	C09	3284798	2724
M328692	C09	3286925	2724
M329642	C09	3296423	2724
M329670	C09	3296707	2724

M330516	C09	3305167	2724
M373077	C09	3730773	2725
M373176	C09	3731763	2725
M379607	C09	3796070	2725
M380118	C09	3801185	2725
M381107	C09	3811077	2725
M383977	C09	3839779	2725
M387904	C09	3879045	2726
M388330	C09	3883306	2726
M405127	C09	4051274	2727
M405230	C09	4052306	2727
M407863	C09	4078631	2728
M409418	C09	4094187	2728
M410011	C09	4100119	2728
M412932	C09	4129325	2728
M419355	C09	4193554	2728
M422670	C09	4226705	2728
M423021	C09	4230217	2728
M450337	C09	4503373	2729
M451269	C09	4512695	2729
M451479	C09	4514793	2729
M451835	C09	4518357	2729
M462589	C09	4625893	2730
M464229	C09	4642298	2730
M465213	C09	4652138	2730
M474771	C09	4747715	2730
M474987	C09	4749872	2730
M476662	C09	4766623	2730
M477223	C09	4772236	2730
M477826	C09	4778269	2730
M478530	C09	4785305	2730
M496760	C09	4967608	2731
M497531	C09	4975314	2731
M498989	C09	4989894	2731
M517584	C09	5175847	2732
M526252	C09	5262528	2732
M577357	C09	5773575	2733
M579103	C09	5791030	2733
M629198	C09	6291984	2734
M629611	C09	6296117	2734
M678624	C09	6786241	2735
M678961	C09	6789618	2735
M679882	C09	6798824	2735
M680768	C09	6807686	2735
M680995	C09	6809959	2735
M758587	C09	7585878	2736
M759374	C09	7593747	2736

M759780	C09	7597801	2736
M764619	C09	7646198	2736
M765644	C09	7656444	2736
M766849	C09	7668493	2736
M776277	C09	7762771	2737
M780385	C09	7803856	2737
M780494	C09	7804941	2737
M785254	C09	7852541	2737
M785701	C09	7857018	2737
M789013	C09	7890132	2737
M789670	C09	7896703	2737
M790837	C09	7908371	2737
M792168	C09	7921684	2737
M792901	C09	7929010	2738
M795541	C09	7955410	2738
M799971	C09	7999719	2738
M869038	C09	8690380	2739
M875806	C09	8758066	2739
M876121	C09	8761214	2739
M876451	C09	8764515	2739
M880833	C09	8808338	2739
M881833	C09	8818335	2739
M882409	C09	8824097	2739
M882878	C09	8828788	2739
M883631	C09	8836316	2739
M883886	C09	8838867	2739
M884420	C09	8844209	2739
M885658	C09	8856581	2740
M886481	C09	8864813	2740
M889370	C09	8893704	2741
M889699	C09	8896998	2741
M890172	C09	8901729	2742
M892408	C09	8924083	2742
M892574	C09	8925745	2742
M894250	C09	8942505	2743
M894419	C09	8944191	2743
M894736	C09	8947360	2743
M894955	C09	8949552	2743
M895526	C09	8955263	2743
M896148	C09	8961488	2743
M896575	C09	8965750	2743
M896741	C09	8967411	2743
M897075	C09	8970751	2744
M897984	C09	8979840	2744
M898520	C09	8985206	2745
M899902	C09	8999024	2745
M900494	C09	9004946	2745

M901421	C09	9014214	2745
M914071	C09	9140719	2745
M916031	C09	9160315	2746
M931962	C09	9319628	2746
M959023	C09	9590232	2747
M959979	C09	9599797	2747
M960615	C09	9606152	2747
M963547	C09	9635478	2747
M964055	C09	9640556	2747
M964380	C09	9643801	2747
M964390	C09	9643902	2747
M964682	C09	9646828	2747
M966087	C09	9660872	2747
M968095	C09	9680951	2748
M968450	C09	9684503	2748
M970287	C09	9702873	2749
M971281	C09	9712818	2749
M976429	C09	9764295	2749
M987705	C09	9877057	2750
M989430	C09	9894303	2750
M989579	C09	9895791	2750
M105357	C09	10535708	2751
M105419	C09	10541994	2751
M105644	C09	10564410	2751
M105661	C09	10566139	2751
M116056	C09	11605683	2752
M116210	C09	11621004	2752
M116213	C09	11621320	2752
M117325	C09	11732513	2753
M117345	C09	11734589	2753
M118796	C09	11879637	2753
M164860	C09	16486082	2754
M166064	C09	16606423	2754
M166381	C09	16638130	2754
M166440	C09	16644065	2754
M166645	C09	16664540	2755
M166761	C09	16676131	2755
M166925	C09	16692578	2755
M167259	C09	16725991	2755
M167339	C09	16733970	2755
M167448	C09	16744838	2755
M167629	C09	16762993	2756
M168006	C09	16800628	2756
M168029	C09	16802902	2757
M168111	C09	16811169	2757
M168265	C09	16826551	2757
M168308	C09	16830871	2757

M168385	C09	16838562	2758
M168563	C09	16856364	2758
M168596	C09	16859622	2758
M168686	C09	16868670	2758
M168761	C09	16876109	2759
M168851	C09	16885181	2759
M169256	C09	16925628	2760
M169288	C09	16928876	2760
M169407	C09	16940709	2761
M169442	C09	16944228	2761
M169526	C09	16952653	2761
M176216	C09	17621698	2762
M177369	C09	17736955	2762
M193002	C09	19300220	2763
M193243	C09	19324394	2763
M193287	C09	19328780	2763
M193645	C09	19364537	2763
M193745	C09	19374504	2763
M193850	C09	19385032	2764
M193976	C09	19397661	2764
M194459	C09	19445981	2765
M194538	C09	19453879	2765
M195789	C09	19578914	2766
M195923	C09	19592389	2766
M196275	C09	19627544	2767
M196444	C09	19644420	2767
M196741	C09	19674110	2768
M196793	C09	19679318	2768
M196847	C09	19684712	2768
M196925	C09	19692573	2768
M197214	C09	19721496	2768
M197420	C09	19742019	2769
M197544	C09	19754477	2769
M197626	C09	19762666	2770
M197633	C09	19763314	2770
M197682	C09	19768276	2770
M197741	C09	19774114	2771
M197885	C09	19788574	2771
M198146	C09	19814659	2772
M198340	C09	19834081	2772
M198885	C09	19888585	2772
M199002	C09	19900244	2772
M199366	C09	19936625	2773
M199593	C09	19959367	2773
M199959	C09	19995937	2773
M201097	C09	20109754	2774
M201162	C09	20116274	2774

M201328	C09	20132825	2774
M201507	C09	20150765	2774
M201543	C09	20154314	2774
M201591	C09	20159106	2774
M201719	C09	20171970	2774
M201895	C09	20189543	2774
M202013	C09	20201335	2774
M202279	C09	20227922	2774
M202311	C09	20231138	2774
M202391	C09	20239130	2774
M202607	C09	20260786	2775
M202744	C09	20274489	2775
M203045	C09	20304505	2776
M203078	C09	20307827	2776
M203136	C09	20313642	2776
M203391	C09	20339163	2776
M203589	C09	20358961	2777
M203724	C09	20372445	2777
M205964	C09	20596423	2778
M206037	C09	20603703	2778
M206062	C09	20606281	2778
M253127	C09	25312738	2779
M254269	C09	25426939	2779
M258454	C09	25845436	2780
M260048	C09	26004897	2780
M265657	C09	26565778	2781
M267248	C09	26724827	2781
M304980	C09	30498081	2782
M305268	C09	30526890	2782
M306402	C09	30640294	2782
M306501	C09	30650147	2782
M307654	C09	30765475	2783
M308032	C09	30803207	2783
M308408	C09	30840862	2784
M309146	C09	30914670	2784
M309698	C09	30969826	2785
M310793	C09	31079368	2785
M311143	C09	31114378	2785
M313980	C09	31398002	2786
M315539	C09	31553912	2786
M316535	C09	31653502	2787
M317499	C09	31749911	2787
M318964	C09	31896442	2788
M320538	C09	32053808	2788
M323388	C09	32338839	2789
M323661	C09	32366187	2789
M328202	C09	32820240	2790

M329081	C09	32908133	2790
M343782	C09	34378216	2791
M344688	C09	34468827	2791
M352468	C09	35246843	2792
M353641	C09	35364147	2792
M364549	C09	36454998	2793
M364920	C09	36492008	2793
M409192	C09	40919219	2794
M410724	C09	41072488	2794
M413098	C09	41309859	2795
M413336	C09	41333682	2795
M416155	C09	41615549	2796
M416187	C09	41618779	2796
M416260	C09	41626090	2796
M417523	C09	41752309	2796
M432670	C09	43267075	2797
M434075	C09	43407593	2797
M444623	C09	44462369	2798
M444736	C09	44473680	2798
M444746	C09	44474687	2798
M445095	C09	44509525	2799
M445343	C09	44534383	2799
M445621	C09	44562195	2800
M445638	C09	44563890	2800
M445902	C09	44590274	2801
M446029	C09	44602967	2801
M450631	C09	45063174	2802
M450653	C09	45065308	2802
M450846	C09	45084609	2802
M450928	C09	45092878	2802
M450985	C09	45098597	2802
M451480	C09	45148085	2803
M451690	C09	45169039	2803
M451730	C09	45173007	2803
M451771	C09	45177173	2803
M451874	C09	45187463	2803
M452046	C09	45204663	2803
M452455	C09	45245533	2803
M452526	C09	45252696	2803
M452697	C09	45269732	2803
M452874	C09	45287455	2803
M452985	C09	45298569	2803
M453895	C09	45389509	2804
M454148	C09	45414892	2804
M468983	C09	46898382	2805
M469026	C09	46902640	2805
M469396	C09	46939688	2806

M469524	C09	46952463	2806
M471444	C09	47144474	2807
M471456	C09	47145648	2807
M481996	C09	48199635	2808
M482819	C09	48281964	2808
M485057	C09	48505739	2809
M485099	C09	48509977	2809
M496007	C09	49600796	2810
M496033	C09	49603353	2810
M496141	C09	49614159	2810
M496237	C09	49623742	2810
M496310	C09	49631055	2810
M501516	C09	50151651	2811
M501924	C09	50192438	2811
M501931	C09	50193154	2811
M501986	C09	50198656	2811
M502960	C09	50296011	2811
M502994	C09	50299498	2811
M503744	C09	50374482	2812
M503756	C09	50375604	2812
M503820	C09	50382087	2812
M505718	C09	50571815	2813
M505745	C09	50574556	2813
M506250	C09	50625001	2813
M506298	C09	50629844	2813
M506331	C09	50633172	2813
M506710	C09	50671038	2813
M506808	C09	50680816	2813
M506960	C09	50696092	2814
M506974	C09	50697404	2814
M507013	C09	50701351	2814
M507035	C09	50703582	2814
M507092	C09	50709240	2814
M507115	C09	50711503	2814
M508116	C09	50811667	2814
M508196	C09	50819677	2814
M508444	C09	50844474	2814
M508454	C09	50845433	2814
M508473	C09	50847371	2814
M513384	C09	51338417	2815
M513447	C09	51344713	2815
M513485	C09	51348556	2815
M513550	C09	51355016	2815
M513645	C09	51364569	2815
M513661	C09	51366194	2815
M521821	C09	52182198	2816
M522918	C09	52291848	2816

M525743	C09	52574353	2817
M525802	C09	52580243	2817
M525854	C09	52585415	2817
M526029	C09	52602919	2817
M526271	C09	52627104	2817
M526322	C09	52632206	2817
M526338	C09	52633809	2817
M526800	C09	52680072	2817
M527250	C09	52725098	2818
M527278	C09	52727870	2818
M527309	C09	52730908	2818
M527656	C09	52765693	2818
M528177	C09	52817700	2818
M528267	C09	52826753	2818
M528671	C09	52867191	2818
M528787	C09	52878704	2818
M528829	C09	52882900	2818
M528909	C09	52890988	2819
M530333	C09	53033320	2819
M530437	C09	53043768	2819
M530538	C09	53053829	2820
M530580	C09	53058080	2820
M531023	C09	53102336	2820
M531387	C09	53138709	2820
M531511	C09	53151161	2820
M532039	C09	53203904	2820
M532105	C09	53210578	2820
M532772	C09	53277285	2821
M532791	C09	53279180	2821
M532841	C09	53284152	2821
M532901	C09	53290156	2821
M533060	C09	53306066	2821
M535242	C09	53524268	2822
M536482	C09	53648296	2822
M536777	C09	53677762	2822
M536794	C09	53679427	2822
M536852	C09	53685259	2823
M536899	C09	53689968	2823
M536937	C09	53693720	2823
M536957	C09	53695757	2823
M536991	C09	53699182	2823
M537004	C09	53700419	2823
M537351	C09	53735197	2823
M537429	C09	53742950	2823
M539307	C09	53930782	2824
M539341	C09	53934106	2824
M541096	C09	54109671	2825

M541272	C09	54127278	2825
M544073	C09	54407365	2826
M544108	C09	54410850	2826
M544207	C09	54420759	2826
M544504	C09	54450412	2826
M544583	C09	54458339	2826
M544701	C09	54470155	2826
M545350	C09	54535038	2826
M545370	C09	54537030	2826
M9317	Scaffold00434	9317	2827
M24540	Scaffold00434	24540	2827
M154641	Scaffold00434	154641	2827
M44796	Scaffold00709	44796	2828
M59721	Scaffold00709	59721	2828
M17632	Scaffold00883	17632	2829
M27044	Scaffold00883	27044	2829
M3131	Scaffold00919	3131	2830
M15469	Scaffold00919	15469	2830
M17988	Scaffold00970	17988	2831
M40375	Scaffold00970	40375	2831
M1107	Scaffold01200	1107	2832
M11891	Scaffold01200	11891	2832
M13402	Scaffold01200	13402	2832
M4573	Scaffold01326	4573	2833
M7952	Scaffold01326	7952	2833

Table S7a Haplotype trend regression analysi

First Marker	Chromos	Position	Block #
M9620644	B07	9620644	1362
M4410527	B07	4410527	1331
M3684925	B07	3684925	1320
M4425980	B07	4425980	1332
M25650458	B07	25650458	1461
M7738513	B07	7738513	1353
M8034787	B07	8034787	1356
M7764181	B07	7764181	1354
M5986754	B07	5986754	1347
M13208012	B07	13208012	1383
M5375029	B07	5375029	1343
M8334946	B07	8334946	1357
M4330165	B07	4330165	1330
M10650500	B07	10650500	1369
M32749694	B07	32749694	1498
M6270507	B07	6270507	1350
M34418225	B07	34418225	1505
M13393388	B07	13393388	1387
M4245547	B07	4245547	1328
M3691385	B07	3691385	1321
M5336932	B07	5336932	1342
M35894652	B07	35894652	1511
M8960409	B07	8960409	1359
M6250296	B07	6250296	1349
M24355519	B07	24355519	1450
M9673714	B07	9673714	1363
M4858426	B07	4858426	1334
M14356051	B07	14356051	1406
M13753632	B07	13753632	1392
M38408159	B07	38408159	1532
M14429375	B07	14429375	1408
M11907439	B07	11907439	1376
M5130	BS00629	5130	1811
M8804511	B07	8804511	1358
M36982693	B07	36982693	1521
M6350914	B07	6350914	1351
M22623449	B07	22623449	1442
M4977141	B07	4977141	1337
M5587919	B07	5587919	1345
M14805048	B01	14805048	75
M27843689	B07	27843689	1476
M42163883	B07	42163883	1543
M13346230	B07	13346230	1386
M4886169	B07	4886169	1335

**M14543176**	**B07**	**14543176**	**1411**
**M3648675**	**B07**	**3648675**	**1319**
**M2890321**	**B07**	**2890321**	**1308**
**M38866415**	**B07**	**38866415**	**1535**
**M33540194**	**B07**	**33540194**	**1501**
**M3896075**	**B07**	**3896075**	**1325**
**M3574719**	**B07**	**3574719**	**1318**
**M32176325**	**B07**	**32176325**	**1495**
**M22802362**	**B07**	**22802362**	**1444**
**M30889160**	**B07**	**30889160**	**1489**
**M12449285**	**B07**	**12449285**	**1379**
**M24416151**	**B07**	**24416151**	**1451**
**M26796805**	**B07**	**26796805**	**1471**
**M14499710**	**B07**	**14499710**	**1410**
M22013632	B07	22013632	1436
M12382897	B07	12382897	1377
M5816614	B07	5816614	1346
M3209185	B07	3209185	1314
M9090041	B07	9090041	1360
M2613822	B07	2613822	1302
M27636453	B07	27636453	1474
M22040928	B07	22040928	1437
M3723297	B07	3723297	1322
M37953127	B07	37953127	1529
M26358342	B07	26358342	1468
M22105894	B07	22105894	1438
M33432684	B07	33432684	1500
M34903569	B07	34903569	1507
M26107973	B07	26107973	1466
M28688749	B07	28688749	1479
M49440378	C03	49440378	2131
M32557616	B07	32557616	1497
M13298784	B07	13298784	1385
M26295602	B07	26295602	1467
M34183723	B06	34183723	1237
M41748469	C01	41748469	1899
M25312738	C09	25312738	2785
M24041170	B07	24041170	1449
M11806929	B07	11806929	1375
M25227598	B07	25227598	1458
M25059617	B07	25059617	1456
M3732620	B07	3732620	1323
M14158134	B07	14158134	1401
M14121635	B07	14121635	1399
M13574887	B07	13574887	1390
M36142212	B07	36142212	1514
M38928725	B07	38928725	1536

M27974412	B07	27974412	1477
M14165179	B07	14165179	1402
M8079	BS00363	8079	1809
M12410651	B07	12410651	1378
M3217705	B07	3217705	1315
M14270427	B07	14270427	1404
M42328690	B07	42328690	1545
M32820240	C09	32820240	2796
M13460542	B07	13460542	1388
M34906390	C03	34906390	2106
M37122804	B07	37122804	1522
M27798399	B07	27798399	1475
M24606538	B07	24606538	1453
M4524587	B07	4524587	1333
M33783360	B07	33783360	1502
M31553797	B02	31553797	318
M33753316	B06	33753316	1233
M13620201	B07	13620201	1391
M22985367	B07	22985367	1445
M2757087	B07	2757087	1304
M34894969	B06	34894969	1244
M36120054	B06	36120054	1249
M42560894	B07	42560894	1548
M33287828	B06	33287828	1232
M37093002	C03	37093002	2124
M26976672	B07	26976672	1472
M19385032	C09	19385032	2770
M35540079	C03	35540079	2109
M42228584	B02	42228584	405
M14565234	B07	14565234	1412
M37315865	B07	37315865	1523
M34368458	B06	34368458	1239
M42071854	B07	42071854	1542
M25542671	B07	25542671	1460
M2790013	B07	2790013	1305
M34952666	B06	34952666	1245
M34064890	B07	34064890	1503
M51410688	C03	51410688	2133
M34180286	B06	34180286	1236
M37200454	C03	37200454	2125
M36328214	C03	36328214	2112
M44641713	B02	44641713	429
M38168330	C01	38168330	1891
M13526624	B07	13526624	1389
M34765721	B06	34765721	1241
M18849	BS00707	18849	1812
M10090715	B07	10090715	1365

M32997333	B07	32997333	1499
M38609726	B07	38609726	1533
M3458408	B07	3458408	1316
M4142720	B07	4142720	1326
M18572234	B07	18572234	1424
M34649849	B06	34649849	1240
M37312224	B02	37312224	354
M11933197	B01	11933197	59
M10199983	B07	10199983	1366
M25891999	B07	25891999	1464
M22417108	B07	22417108	1440
M34829560	B06	34829560	1242
M26060839	B07	26060839	1465
M39050115	B07	39050115	1538
M25372764	B07	25372764	1459
M33233243	B01	33233243	158
M25845436	C09	25845436	2786
M34233026	B07	34233026	1504
M39299217	B07	39299217	1540
M33918504	B06	33918504	1235
M35698467	C03	35698467	2111
M17621698	C09	17621698	2768
M266832	B08	266832	1552
M26565778	C09	26565778	2787
M35440174	B06	35440174	1246
M2876182	B07	2876182	1307
M13820139	B07	13820139	1394
M31128252	B07	31128252	1490
M44431720	B02	44431720	424
M36866355	C03	36866355	2121
M31325852	B07	31325852	1491
M36732364	C03	36732364	2117
M36577292	B07	36577292	1518
M52835523	C03	52835523	2137
M14320800	B07	14320800	1405
M37654974	B07	37654974	1526
M42204113	B07	42204113	1544
M26792277	C08	26792277	2630
M38780127	B07	38780127	1534
M14664349	B07	14664349	1413
M36767097	B07	36767097	1519
M2283575	B07	2283575	1297
M26472722	B07	26472722	1469
M2744186	B07	2744186	1303
M2335626	B08	2335626	1569
M29411190	B07	29411190	1482
M3177797	B07	3177797	1313

M14409976	B07	14409976	1407
M26628405	B07	26628405	1470
M19627544	C09	19627544	2773
M4190599	B07	4190599	1327
M19939992	B07	19939992	1433
M2885324	B08	2885324	1573
M31896442	C09	31896442	2794
M5419919	B07	5419919	1344
M38469050	B02	38469050	364
M14134089	B07	14134089	1400
M41726779	C01	41726779	1898
M16511341	B07	16511341	1417
M31351274	B07	31351274	1492
M18865754	B07	18865754	1427
M2548458	B08	2548458	1571
M19965171	B07	19965171	1434
M11607341	B07	11607341	1373
M38477768	B02	38477768	365
M10902883	B07	10902883	1370
M43370206	B07	43370206	1549
M34999235	B07	34999235	1508
M36708156	C03	36708156	2115
M1644118	B07	1644118	1293
M37785238	B02	37785238	359
M30361755	B07	30361755	1486
M12568910	B07	12568910	1381
M30525555	B07	30525555	1487
M36723263	C03	36723263	2116
M36211916	B07	36211916	1515
M11718692	B07	11718692	1374
M37599152	B07	37599152	1525
M37062903	C06	37062903	2446
M34941899	C03	34941899	2107
M16259212	B07	16259212	1415
M11068381	B07	11068381	1371
M7954677	B07	7954677	1355
M5120118	B07	5120118	1338
M3105431	B07	3105431	1312
M37906384	B07	37906384	1528
M25679789	B07	25679789	1462
M23610656	B07	23610656	1447
M11207412	B07	11207412	1372
M5540515	B08	5540515	1602
M10352268	B07	10352268	1367
M13782626	B07	13782626	1393
M30765475	C09	30765475	2789
M31952043	B07	31952043	1494

M38189169	B07	38189169	1530
M10146008	B04	10146008	824
M36810946	B07	36810946	1520
M13906702	B07	13906702	1396
M31531980	B07	31531980	1493
M31306537	C03	31306537	2096
M14896442	C05	14896442	2323
M2989939	B07	2989939	1310
M35846019	B07	35846019	1510
M4903573	B07	4903573	1336
M19926846	B07	19926846	1432
M2907300	B07	2907300	1309
M4105171	B08	4105171	1584
M38039376	C03	38039376	2128
M22447228	B07	22447228	1441
M14807931	B07	14807931	1414
M35961	B05	35961	900
M3369219	B08	3369219	1577
M2857721	B07	2857721	1306
M35990485	B07	35990485	1512
M32852834	B06	32852834	1231
M13366015	C08	13366015	2604
M9108511	B07	9108511	1361
M37068092	C03	37068092	2123
M31876668	C03	31876668	2101
M14190734	B07	14190734	1403
M42196431	B02	42196431	404
M20260786	C09	20260786	2781
M2293789	B07	2293789	1298
M37890194	B06	37890194	1259
M627148	B06	627148	1109
M11054517	B04	11054517	828
M11261642	B04	11261642	830
M35058860	B07	35058860	1509
M24807426	B07	24807426	1454
M33883467	B06	33883467	1234
M38864615	B08	38864615	1723
M35246843	C09	35246843	2798
M23877164	B07	23877164	1448
M17779767	B07	17779767	1423
M41300441	B02	41300441	396
M36091214	B07	36091214	1513
M38127795	B06	38127795	1261
M40919219	C09	40919219	2800
M25109886	B07	25109886	1457
M36454998	C09	36454998	2799
M41309859	C09	41309859	2801

M4260462	B07	4260462	1329
M13883806	B07	13883806	1395
M46175991	C05	46175991	2368
M16451555	B07	16451555	1416
M3544729	B07	3544729	1317
M31398002	C09	31398002	2792
M1389679	B07	1389679	1292
M2456105	B07	2456105	1299
M35589495	C03	35589495	2110
M9717940	C05	9717940	2302
M16940709	C09	16940709	2767
M27385995	B03	27385995	711
M30022816	B07	30022816	1485
M686267	B08	686267	1555
M18878013	B07	18878013	1428
M7867523	B04	7867523	802
M44518614	B02	44518614	428
M51303369	C03	51303369	2132
M45430140	C07	45430140	2569
M8164815	B04	8164815	808
M36836991	C03	36836991	2120
M3858613	B07	3858613	1324
M14043418	B07	14043418	1397
M44562195	C09	44562195	2806
M22684390	B07	22684390	1443
M18546475	C04	18546475	2216
M2568196	B07	2568196	1300
M37398518	C06	37398518	2448
M20358961	C09	20358961	2783
M45870319	C05	45870319	2366
M37238523	C03	37238523	2126
M41088028	B02	41088028	393
M7173948	B07	7173948	1352
M8009782	B06	8009782	1177
M41667606	C01	41667606	1896
M35643371	C01	35643371	1889
M161315	B07	161315	1278
M36439544	B07	36439544	1517
M19774114	C09	19774114	2777
M45389509	C09	45389509	2810
M37682553	B02	37682553	357
M42290787	B02	42290787	407
M20304505	C09	20304505	2782
M34852143	B06	34852143	1243
M9641655	B04	9641655	821
M38002945	C01	38002945	1890
M19742019	C09	19742019	2775

M32338839	C09	32338839	2795
M30969826	C09	30969826	2791
M11989714	B04	11989714	840
M38985286	B07	38985286	1537
M1801063	B02	1801063	264
M44390803	B02	44390803	423
M30840862	C09	30840862	2790
M42370558	C02	42370558	2002
M36694645	C03	36694645	2114
M14302651	C06	14302651	2392
M25865828	B07	25865828	1463
M14434855	B07	14434855	1409
M24434503	B07	24434503	1452
M34038804	C03	34038804	2103
M31653502	C09	31653502	2793
M38548053	B02	38548053	368
M18817226	B07	18817226	1426
M1635144	C07	1635144	2465
M26136857	B03	26136857	701
M42281520	C03	42281520	2129
M9368498	B04	9368498	817
M29054822	B07	29054822	1481
M41903911	B02	41903911	401
M6958133	B03	6958133	597
M36771744	C03	36771744	2118
M12684597	B08	12684597	1650
M19445981	C09	19445981	2771
M13328693	B01	13328693	69
M921648	B07	921648	1289
M36262742	B02	36262742	347
M2723479	B08	2723479	1572
M34524429	C03	34524429	2105
M31486439	C08	31486439	2653
M739480	B07	739480	1286
M10048869	B04	10048869	823
M16691656	B07	16691656	1419
M35713245	B06	35713245	1247
M13031360	B07	13031360	1382
M36899155	C06	36899155	2444
M5874541	B08	5874541	1607
M42763252	B02	42763252	409
M16802902	C09	16802902	2763
M1114958	B02	1114958	255
M42756895	C07	42756895	2551
M45529006	C05	45529006	2364
M29746104	B03	29746104	719
M39527058	B06	39527058	1273

M2017947	B07	2017947	1295
M8210004	C06	8210004	2374
M20899047	B03	20899047	672
M44383347	B02	44383347	422
M3383200	B04	3383200	762
M8098716	B04	8098716	806
M38281726	B02	38281726	362
M39132880	B02	39132880	372
M36200118	C06	36200118	2441
M28136916	B03	28136916	715
M34206973	C01	34206973	1888
M32827754	B06	32827754	1230
M37966600	B06	37966600	1260
M44590274	C09	44590274	2807
M38145678	C06	38145678	2452
M32759252	C08	32759252	2660
M23085300	B06	23085300	1194
M10774068	B01	10774068	51
M13284193	C04	13284193	2212
M19977205	B07	19977205	1435
M18827812	C08	18827812	2608
M38527738	B02	38527738	367
M3041391	B07	3041391	1311
M36816764	C06	36816764	2443
M6160904	B07	6160904	1348
M44365369	B02	44365369	421
M9726531	C08	9726531	2600
M36566004	C08	36566004	2675
M1939371	B08	1939371	1565
M5131694	B06	5131694	1154
M44485571	C07	44485571	2561
M16876109	C09	16876109	2765
M347358	B07	347358	1281
M41803232	B02	41803232	400
M13275269	B07	13275269	1384
M944660	B07	944660	1290
M47994969	B05	47994969	1091
M43841880	C05	43841880	2355
M43588846	B02	43588846	417
M16945549	B07	16945549	1421
M3536914	B04	3536914	766
M40568262	C01	40568262	1893
M34437288	C03	34437288	2104
M19814659	C09	19814659	2778
M41702919	C01	41702919	1897
M13897578	B08	13897578	1665
M30128893	B03	30128893	723

M1019711	B04	1019711	739
M1203940	B04	1203940	741
M26684089	B03	26684089	706
M34378216	C09	34378216	2797
M44175233	B02	44175233	419
M40216343	C06	40216343	2464
M12415594	B08	12415594	1647
M8881536	B04	8881536	810
M14470374	B03	14470374	637
M39663398	B02	39663398	375
M20596423	C09	20596423	2784
M28889443	B07	28889443	1480
M33284870	C06	33284870	2431
M14096078	B07	14096078	1398
M15035641	B08	15035641	1679
M31190062	B08	31190062	1718
M19578914	C09	19578914	2772
M38571948	B02	38571948	369
M37668364	C06	37668364	2449
M59029999	C03	59029999	2153
M30471682	B01	30471682	119
M1816697	B04	1816697	750
M24107941	B08	24107941	1705
M36314323	B06	36314323	1251
M64652670	C03	64652670	2174
M114290	B08	114290	1551
M39824438	C07	39824438	2536
M31340137	C03	31340137	2098
M12845988	B01	12845988	66
M12663550	C06	12663550	2385
M776657	B07	776657	1287
M1251793	B08	1251793	1560
M40978865	B02	40978865	390
M49311335	B05	49311335	1097
M37264761	B02	37264761	352
M49600796	C09	49600796	2816
M42263076	B02	42263076	406
M36996898	B01	36996898	220
M10212939	B04	10212939	825
M34985082	C03	34985082	2108
M2724435	B02	2724435	268
M41668019	B02	41668019	399
M37431703	B07	37431703	1524
M32377523	B07	32377523	1496
M38460057	B02	38460057	363
M34393970	C06	34393970	2438
M668992	B07	668992	1284

M13212798	C04	13212798	2211
M4908887	B08	4908887	1592
M41009586	C04	41009586	2232
M39033319	B06	39033319	1267
M2577402	B07	2577402	1301
M32633487	B01	32633487	146
M27555639	B03	27555639	712
M19460986	B07	19460986	1430
M19936625	C09	19936625	2779
M43450360	C04	43450360	2236
M34305703	B06	34305703	1238
M31322840	C03	31322840	2097
M44225815	C05	44225815	2360
M32819991	C08	32819991	2662
M35921578	B08	35921578	1719
M39688822	B02	39688822	376
M24997591	C01	24997591	1883
M48335613	B02	48335613	458
M11433216	B04	11433216	831
M23624138	C06	23624138	2411
M35950495	B01	35950495	198
M41334003	B02	41334003	397
M2115952	B08	2115952	1566
M1848505	B07	1848505	1294
M22153301	B07	22153301	1439
M14124831	B01	14124831	73
M39403858	B06	39403858	1272
M24666217	B08	24666217	1708
M6469309	B08	6469309	1620
M42327802	C02	42327802	2001
M2710059	B03	2710059	569
M13068207	B01	13068207	68
M44509525	C09	44509525	2805
M8384495	B04	8384495	809
M14229676	B05	14229676	993
M45735401	B02	45735401	433
M5912668	B01	5912668	32
M1363606	B02	1363606	258
M41135893	B02	41135893	395
M12813465	B08	12813465	1652
M6558570	B04	6558570	796
M19906491	B05	19906491	1022
M27047106	B03	27047106	708
M3684033	B06	3684033	1139
M34330855	C08	34330855	2667
M38730205	B06	38730205	1265
M13982243	C06	13982243	2391

M31380347	B03	31380347	726
M30557343	B07	30557343	1488
M5597822	B08	5597822	1604
M33716294	B01	33716294	165
M5840952	B04	5840952	788
M3871630	B03	3871630	578
M4665827	B08	4665827	1591
M6503076	B03	6503076	593
M38220204	B07	38220204	1531
M14122422	B08	14122422	1669
M13643658	B08	13643658	1663
M12563272	C05	12563272	2308
M46370571	B08	46370571	1751
M58596937	C03	58596937	2149
M21701497	B04	21701497	881
M3004299	B03	3004299	574
M17619688	B07	17619688	1422
M22473789	C01	22473789	1881
M44183683	C05	44183683	2357
M27832215	B01	27832215	92
M49802171	C02	49802171	2015
M42409805	C05	42409805	2352
M5375218	C08	5375218	2591
M5718731	C02	5718731	1942
M47376530	B02	47376530	446
M6428008	B04	6428008	793
M3045455	B05	3045455	921
M50887956	B02	50887956	482
M35179888	B01	35179888	189
M36756334	B06	36756334	1253
M39833731	B07	39833731	1541
M42788810	B08	42788810	1727
M33344849	C06	33344849	2432
M19114301	B01	19114301	80
M8891443	B01	8891443	43
M5005932	B08	5005932	1594
M30498081	C09	30498081	2788
M30274920	B01	30274920	117
M26504813	B03	26504813	704
M692437	B07	692437	1285
M39734874	B02	39734874	378
M28372098	B07	28372098	1478
M44187489	C05	44187489	2358
M42518037	B07	42518037	1547
M916338	B08	916338	1557
M32729927	C08	32729927	2658
M6685399	C02	6685399	1945

M16922455	C04	16922455	2214
M44483358	B08	44483358	1745
M15719744	B05	15719744	1008
M11964467	B04	11964467	839
M28143185	B01	28143185	96
M17632	Scaffold0	17632	2835
M42592028	B08	42592028	1726
M29751536	B07	29751536	1484
M36674201	B02	36674201	349
M44462369	C09	44462369	2804
M16486082	C09	16486082	2760
M5559182	C02	5559182	1940
M29629136	B01	29629136	112
M16925628	C09	16925628	2766
M39902789	B02	39902789	380
M3931607	C05	3931607	2281
M1005400	B01	1005400	7
M42165677	B02	42165677	403
M11605683	C09	11605683	2758
M45122568	C07	45122568	2566
M725722	B02	725722	249
M23364813	B07	23364813	1446
M33362389	B01	33362389	162
M36084763	B06	36084763	1248
M2508295	B08	2508295	1570
M49698927	B02	49698927	472
M34789671	B01	34789671	188
M35914823	C06	35914823	2440
M10663351	C06	10663351	2382
M39162344	B07	39162344	1539
M45063174	C09	45063174	2808
M28306086	B01	28306086	97
M12220548	C07	12220548	2471
M1407738	B05	1407738	906
M4271015	B08	4271015	1585
M17757085	C05	17757085	2330
M2710741	C05	2710741	2276
M4288178	C01	4288178	1830
M36943147	C08	36943147	2678
M52788630	C02	52788630	2018
M9144375	B04	9144375	813
M34650124	B01	34650124	184
M41318214	C07	41318214	2545
M3672616	B04	3672616	770
M2056233	B04	2056233	756
M39977474	B02	39977474	383
M3799240	B08	3799240	1582

M14398437	C05	14398437	2317
M40414301	B02	40414301	389
M8075709	B04	8075709	805
M7188605	B04	7188605	799
M46939688	C09	46939688	2812
M3006484	C02	3006484	1930
M46328735	B02	46328735	436
M14655330	C05	14655330	2320
M37732265	B02	37732265	358
M27921218	B01	27921218	93
M1830670	B03	1830670	561
M2575475	B03	2575475	567
M9499999	B04	9499999	820
M17181541	C02	17181541	1955
M9702873	C09	9702873	2755
M3861591	B02	3861591	272
M1138219	B05	1138219	903
M48428052	B02	48428052	459
M13028244	C06	13028244	2387
M45644301	B08	45644301	1748
M13922652	B05	13922652	990
M38443286	C06	38443286	2453
M44954195	C07	44954195	2565
M147108	C05	147108	2266
M18471696	C03	18471696	2070
M32891100	B01	32891100	153
M27223971	C03	27223971	2087
M1645525	B03	1645525	559
M38791649	B06	38791649	1266
M33750189	C06	33750189	2434
M50696092	C09	50696092	2820
M48623257	B02	48623257	462
M52476977	B08	52476977	1802
M572986	B03	572986	535
M37280043	B02	37280043	353
M41007274	B02	41007274	391
M10456762	B07	10456762	1368
M1236043	B04	1236043	742
M28465	B07	28465	1275
M24749739	B08	24749739	1709
M43317599	B02	43317599	416
M43050169	B08	43050169	1730
M5569445	B08	5569445	1603
M9123447	C01	9123447	1851
M36765989	B01	36765989	215
M32419901	C01	32419901	1885
M45887030	C05	45887030	2367

M28928191	C02	28928191	1989
M13975759	B08	13975759	1667
M9835186	B07	9835186	1364
M7394457	C01	7394457	1842
M58016539	C03	58016539	2147
M31858864	B06	31858864	1223
M29463684	B03	29463684	718
M32097985	B01	32097985	131
M329155	B07	329155	1280
M26803805	C06	26803805	2419
M5550926	B06	5550926	1159
M30083891	B03	30083891	721
M7035319	B01	7035319	38
M37915394	C06	37915394	2450
M57154208	C03	57154208	2142
M34668941	B01	34668941	185
M2730897	B05	2730897	919
M13485527	B08	13485527	1657
M608375	B04	608375	735
M51340881	B02	51340881	484
M13089792	C06	13089792	2388
M3088282	B08	3088282	1575
M62619147	C03	62619147	2163
M41096283	B02	41096283	394
M5193758	B03	5193758	591
M41421187	C01	41421187	1894
M29100151	C02	29100151	1992
M5000130	B08	5000130	1593
M30529558	B01	30529558	120
M55130161	B02	55130161	522
M12427576	C07	12427576	2473
M46422912	B02	46422912	438
M33129820	C03	33129820	2102
M37332091	B02	37332091	355
M44502782	B02	44502782	427
M39269299	B04	39269299	893
M30718368	B02	30718368	315
M12276175	B01	12276175	61
M27214776	C03	27214776	2086
M1289000	B04	1289000	743
M34648028	B07	34648028	1506
M2930531	C05	2930531	2277
M1842764	B04	1842764	752
M26226600	B01	26226600	85
M39990837	B02	39990837	384
M39987474	C07	39987474	2537
M5170208	B08	5170208	1600

M15276951	C05	15276951	2327
M21623043	B04	21623043	879
M26938470	C02	26938470	1971
M36650397	B04	36650397	892
M40629327	B04	40629327	896
M10018966	B01	10018966	49
M9459749	B04	9459749	819
M10716555	C06	10716555	2383
M12554954	B01	12554954	64
M32876052	B01	32876052	152
M5019973	B08	5019973	1595
M35188306	C07	35188306	2516
M42923011	B02	42923011	413
M37280922	C03	37280922	2127
M38457015	B01	38457015	235
M47144474	C09	47144474	2813
M6266702	B08	6266702	1615
M29040545	C02	29040545	1991
M16838562	C09	16838562	2764
M18706078	C08	18706078	2606
M42855526	C08	42855526	2716
M30952306	C07	30952306	2505
M35119643	B03	35119643	734
M49432670	C02	49432670	2011
M24014353	B08	24014353	1703
M17010722	B04	17010722	862
M5313559	B04	5313559	783
M32592031	B01	32592031	145
M33875912	B01	33875912	170
M38133095	B02	38133095	361
M39868193	C08	39868193	2703
M12091578	B01	12091578	60
M27491666	C06	27491666	2421
M33218797	B02	33218797	332
M10833730	B04	10833730	826
M63523386	C03	63523386	2167
M27421040	B07	27421040	1473
M35726499	B01	35726499	194
M13133454	B08	13133454	1655
M16566661	B07	16566661	1418
M40768151	C04	40768151	2231
M9680951	C09	9680951	2754
M44523845	C03	44523845	2130
M3013972	B03	3013972	575
M6244476	B01	6244476	34
M38439147	B06	38439147	1262
M5773327	B04	5773327	786

M21994805	C04	21994805	2219
M37714850	B07	37714850	1527
M4085530	B06	4085530	1145
M26901542	C05	26901542	2337
M4732823	B06	4732823	1153
M34638796	B01	34638796	183
M2352984	B03	2352984	565
M56336123	B02	56336123	529
M3474161	B04	3474161	763
M42941134	C01	42941134	1907
M183082	B02	183082	246
M26664675	B01	26664675	86
M5971766	B08	5971766	1610
M10469789	B01	10469789	50
M30594057	B05	30594057	1031
M3870713	B06	3870713	1142
M31134482	C08	31134482	2651
M7239842	C05	7239842	2287
M30345015	B01	30345015	118
M9160315	C09	9160315	2752
M2005534	B03	2005534	563
M33771700	B01	33771700	168
M1910386	B06	1910386	1116
M10535708	C09	10535708	2757
M19273996	C04	19273996	2217
M40391218	B02	40391218	388
M43068262	B08	43068262	1731
M12488519	B07	12488519	1380
M26520731	B03	26520731	705
M39423587	C04	39423587	2230
M34185584	C06	34185584	2437
M19174831	B03	19174831	658
M13476963	B05	13476963	976
M50374482	C09	50374482	2818
M12345264	B01	12345264	62
M1349402	B04	1349402	745
M43622081	B02	43622081	418
M52235987	B08	52235987	1799
M41816686	C05	41816686	2350
M32722142	B01	32722142	147
M9375856	B04	9375856	818
M19535283	B07	19535283	1431
M20698766	C06	20698766	2403
M49734576	B05	49734576	1098
M19300220	C09	19300220	2769
M14954069	B08	14954069	1678
M9906000	C05	9906000	2303

M39639263	B02	39639263	374
M1936466	B04	1936466	755
M2597871	C04	2597871	2185
M32333672	B01	32333672	136
M5364452	B06	5364452	1157
M3408180	B08	3408180	1578
M2916777	B03	2916777	572
M887289	B07	887289	1288
M11764219	B04	11764219	835
M11584824	B01	11584824	56
M29785501	B03	29785501	720
M10491585	C06	10491585	2381
M28756145	B06	28756145	1204
M4363749	B04	4363749	771
M27952085	B01	27952085	94
M36588046	B08	36588046	1721
M46755617	C04	46755617	2249
M51850972	B08	51850972	1795
M18605546	B07	18605546	1425
M35642001	B01	35642001	193
M4397017	B08	4397017	1586
M2842230	B03	2842230	570
M59222720	C03	59222720	2155
M7947	B05	7947	898
M5194544	C08	5194544	2590
M64159876	C03	64159876	2171
M31519247	B06	31519247	1221
M32609950	B03	32609950	729
M32331479	C08	32331479	2655
M36629720	C06	36629720	2442
M94994	B08	94994	1550
M31722006	B03	31722006	727
M1410661	B04	1410661	748
M7983538	B04	7983538	804
M29654346	B06	29654346	1215
M3580046	B08	3580046	1580
M3209003	B04	3209003	760
M7707902	B08	7707902	1624
M12824629	C05	12824629	2314
M13011147	B04	13011147	845
M32440996	B01	32440996	138
M27695402	B02	27695402	303
M10885507	B03	10885507	612
M12675498	B01	12675498	65
M16762993	C09	16762993	2762
M463601	B08	463601	1554
M42855735	B02	42855735	411

M908	C02	908	1911
M5582555	B06	5582555	1161
M9319555	B04	9319555	815
M47491450	B02	47491450	449
M847953	B03	847953	541
M10031972	B04	10031972	822
M13316232	C04	13316232	2213
M7929010	C09	7929010	2744
M46898382	C09	46898382	2811
M33731302	B01	33731302	166
M15595140	B04	15595140	856
M37381335	B06	37381335	1257
M5285231	B07	5285231	1341
M45425034	C05	45425034	2363
M50642337	B05	50642337	1104
M50926653	B08	50926653	1777
M9315727	B01	9315727	45
M35652665	C05	35652665	2343
M6097734	B04	6097734	791
M10305304	C06	10305304	2380
M44288781	B08	44288781	1740
M46158218	B02	46158218	435
M7557589	C05	7557589	2295
M48505739	C09	48505739	2815
M45294473	C02	45294473	2009
M53284989	C03	53284989	2140
M17094424	B04	17094424	863
M35924293	B04	35924293	890
M19105332	C06	19105332	2397
M11659175	B01	11659175	57
M53394546	C03	53394546	2141
M43677577	C05	43677577	2354
M28636193	C02	28636193	1985
M1578367	B08	1578367	1562
M24621182	C07	24621182	2496
M31775164	C03	31775164	2100
M52801450	C03	52801450	2135
M38618720	B06	38618720	1264
M29530246	B07	29530246	1483
M6095124	C01	6095124	1836
M5657994	C02	5657994	1941
M9351376	B04	9351376	816
M33729003	B05	33729003	1034
M3754196	B08	3754196	1581
M13340711	B05	13340711	973
M27385405	C03	27385405	2090
M42372522	B07	42372522	1546

M19564309	C05	19564309	2332
M34180877	B01	34180877	172
M20020263	C01	20020263	1879
M8415865	C01	8415865	1849
M19949795	C02	19949795	1960
M28700656	C08	28700656	2638
M1316317	B02	1316317	256
M33189197	C08	33189197	2663
M4615318	B04	4615318	773
M32597554	C01	32597554	1886
M34991297	B02	34991297	339
M27586579	C08	27586579	2634
M28493608	B01	28493608	98
M4402814	B04	4402814	772
M33270614	C04	33270614	2225
M40539850	C01	40539850	1892
M49341703	C02	49341703	2010
M1726869	B06	1726869	1114
M41055635	C07	41055635	2543
M43321667	C02	43321667	2005
M1021738	B08	1021738	1558
M2922689	B03	2922689	573
M33304694	B01	33304694	160
M64193339	C03	64193339	2172
M29479873	B01	29479873	111
M31990187	B01	31990187	130
M27761227	C01	27761227	1884
M53277285	C09	53277285	2827
M19361037	C01	19361037	1878
M20531562	B04	20531562	876
M3587287	C05	3587287	2279
M3131	Scaffold0	3131	2836
M34306807	B01	34306807	177
M3908232	B06	3908232	1143
M7304264	C08	7304264	2595
M8690380	C09	8690380	2745
M2228300	B05	2228300	913
M5211073	B08	5211073	1601
M44449681	B02	44449681	425
M32110984	B02	32110984	321
M28824910	B01	28824910	105
M56070965	B02	56070965	528
M14660005	B04	14660005	847
M39406043	C08	39406043	2699
M47477830	B02	47477830	448
M26563383	B04	26563383	885
M9317	Scaffold0	9317	2833

M34709556	B01	34709556	186
M42902957	C01	42902957	1905
M51756636	B02	51756636	490
M4365668	B06	4365668	1148
M28771402	C02	28771402	1987
M39687787	B06	39687787	1274
M15757081	C03	15757081	2055
M838432	B02	838432	251
M51430496	B02	51430496	485
M10353564	C01	10353564	1857
M5824491	B08	5824491	1606
M35174869	C08	35174869	2670
M19762666	C09	19762666	2776
M38031980	B02	38031980	360
M1821729	B04	1821729	751
M24187726	B08	24187726	1707
M57664844	C03	57664844	2145
M434753	C08	434753	2588
M5383026	B06	5383026	1158
M15806903	B04	15806903	857
M15575958	B04	15575958	855
M427814	B08	427814	1553
M51377598	B08	51377598	1785
M26116284	C05	26116284	2336
M43495429	B05	43495429	1063
M5728731	B08	5728731	1605
M5890169	B08	5890169	1608
M4535506	B01	4535506	26
M9262562	B04	9262562	814
M32634410	C01	32634410	1887
M39318498	B06	39318498	1270
M22534490	B03	22534490	674
M32169701	B01	32169701	134
M47740948	B05	47740948	1088
M36964750	C03	36964750	2122
M31611542	C03	31611542	2099
M3553309	B04	3553309	767
M33201216	B01	33201216	157
M10034907	C05	10034907	2304
M44340591	C05	44340591	2361
M44995252	C04	44995252	2238
M11838384	B03	11838384	618
M17726585	B04	17726585	867
M43245349	C07	43245349	2553
M7833113	C08	7833113	2597
M35624786	B01	35624786	192
M32397351	B01	32397351	137

M57855382	C03	57855382	2146
M350390	B02	350390	247
M58636806	C03	58636806	2150
M6220356	B08	6220356	1613
M36608573	B01	36608573	209
M5220242	B07	5220242	1339
M13147934	C07	13147934	2480
M5047277	B08	5047277	1597
M31281073	C07	31281073	2507
M43144278	B08	43144278	1732
M41615549	C09	41615549	2802
M40205325	C06	40205325	2463
M18496423	C05	18496423	2331
M14224110	B03	14224110	635
M46526787	B02	46526787	440
M4634867	B04	4634867	774
M32648604	C08	32648604	2657
M60640516	C03	60640516	2156
M17722777	C03	17722777	2062
M4581624	B08	4581624	1589
M57279347	C03	57279347	2144
M16091173	B04	16091173	860
M2926238	B08	2926238	1574
M32768591	B01	32768591	149
M1503486	B05	1503486	908
M41538892	C01	41538892	1895
M31215005	C03	31215005	2095
M33098779	B02	33098779	329
M4729427	B03	4729427	587
M18771154	C07	18771154	2485
M12660337	C05	12660337	2310
M1514285	B05	1514285	909
M16870367	B01	16870367	78
M51579739	B02	51579739	487
M25681481	B01	25681481	83
M33765192	B01	33765192	167
M5162758	B08	5162758	1599
M33547438	B01	33547438	163
M65369873	C03	65369873	2177
M40756603	C08	40756603	2705
M36640724	B01	36640724	211
M11695153	C02	11695153	1954
M2928175	C03	2928175	2030
M1200001	B08	1200001	1559
M47558047	C07	47558047	2583
M36968128	C08	36968128	2680
M32224785	B01	32224785	135

M63694496	C03	63694496	2168
M17037047	B05	17037047	1021
M52660821	B08	52660821	1805
M3562531	B04	3562531	768
M65464556	C03	65464556	2180
M33333015	C04	33333015	2226
M41665811	C08	41665811	2708
M33311330	B04	33311330	889
M38914068	C06	38914068	2459
M17249004	C03	17249004	2061
M46410775	B02	46410775	437
M16769955	B07	16769955	1420
M60980209	C03	60980209	2159
M11408692	B01	11408692	55
M408904	B07	408904	1282
M4967608	C09	4967608	2737
M36628308	B01	36628308	210
M2421493	C04	2421493	2183
M32448723	B01	32448723	139
M247733	C02	247733	1914
M16581578	B08	16581578	1686
M15456213	B05	15456213	1003
M52361277	B08	52361277	1800
M52720696	C03	52720696	2134
M8927545	B04	8927545	812
M35669404	C02	35669404	1998
M26301267	B03	26301267	702
M43437274	C04	43437274	2235
M43790584	B05	43790584	1069
M40691614	B05	40691614	1048
M28906470	C08	28906470	2639
M7307970	C04	7307970	2204
M11793280	B04	11793280	836
M34584629	B01	34584629	182
M43267075	C09	43267075	2803
M21262865	C01	21262865	1880
M34531324	C08	34531324	2668
M19312059	C06	19312059	2399
M51447818	B08	51447818	1786
M40452409	B05	40452409	1047
M1639320	B03	1639320	558
M40240630	B02	40240630	386
M58864985	C03	58864985	2152
M43028201	B08	43028201	1729
M13194433	B04	13194433	846
M4503373	C09	4503373	2735
M7824100	B05	7824100	938

M39172208	B05	39172208	1043
M2415666	B03	2415666	566
M29811163	B01	29811163	114
M11478899	B03	11478899	617
M5078136	B08	5078136	1598
M6109149	B04	6109149	792
M10142183	C05	10142183	2305
M12714123	C05	12714123	2311
M5098260	C04	5098260	2193
M22304854	C06	22304854	2408
M22808760	C07	22808760	2490
M44188625	C07	44188625	2559
M30385644	C02	30385644	1995
M28736339	B03	28736339	716
M8924093	B08	8924093	1628
M33225351	C08	33225351	2665
M8393475	B03	8393475	601
M32137267	B01	32137267	132
M32562473	B01	32562473	143
M12784308	C05	12784308	2313
M31762610	C08	31762610	2654
M49873198	B05	49873198	1102
M37219821	B06	37219821	1256
M24160460	C03	24160460	2078
M42874085	C08	42874085	2717
M39471130	B01	39471130	240
M52852713	C03	52852713	2138
M915900	B03	915900	543
M42986835	B08	42986835	1728
M33525870	B03	33525870	730
M49082885	B02	49082885	464
M57203213	C03	57203213	2143
M3246967	B04	3246967	761
M34205328	B01	34205328	174
M32726709	B01	32726709	148
M1672913	B08	1672913	1563
M42384051	C01	42384051	1901
M39728691	B01	39728691	244
M1123836	B04	1123836	740
M42937432	B02	42937432	414
M32537494	B01	32537494	142
M1383470	C05	1383470	2271
M12861707	B05	12861707	963
M48199635	C09	48199635	2814
M21648643	B04	21648643	880
M19441399	C08	19441399	2611
M34085824	B03	34085824	732

M11394022	B03	11394022	615
M20044599	C02	20044599	1961
M6240178	B08	6240178	1614
M46084580	B08	46084580	1750
M17209917	B03	17209917	648
M36550916	C03	36550916	2113
M36478463	B06	36478463	1252
M38340718	C08	38340718	2693
M15540202	C03	15540202	2050
M29152117	B01	29152117	110
M51816835	B02	51816835	492
M18855804	B08	18855804	1700
M11836713	B01	11836713	58
M6404489	B08	6404489	1617
M16948447	B05	16948447	1020
M31769341	B01	31769341	127
M5429357	B01	5429357	30
M16232926	C06	16232926	2394
M39703381	B02	39703381	377
M5248694	B07	5248694	1340
M51135527	B05	51135527	1107
M16929647	B05	16929647	1019
M40252539	B02	40252539	387
M51338417	C09	51338417	2821
M12726156	C05	12726156	2312
M9495315	C06	9495315	2377
M32798111	C08	32798111	2661
M8160710	B04	8160710	807
M14366796	C05	14366796	2316
M43643051	B08	43643051	1736
M49430713	B02	49430713	468
M47129098	C07	47129098	2578
M6799046	C04	6799046	2202
M25939989	C03	25939989	2081
M12898556	B01	12898556	67
M39104904	B02	39104904	371
M46836915	C07	46836915	2576
M14719474	B08	14719474	1675
M5359978	B03	5359978	592
M27982966	B02	27982966	306
M32345428	B06	32345428	1228
M29588652	B08	29588652	1715
M18002494	C03	18002494	2064
M44223630	C07	44223630	2560
M4177561	C01	4177561	1828
M17016646	C03	17016646	2059
M25825191	C03	25825191	2080

M46891397	B02	46891397	444
M49751456	C02	49751456	2013
M15613406	C03	15613406	2051
M31626789	B06	31626789	1222
M63712431	C03	63712431	2169
M27563060	B03	27563060	713
M36211610	B04	36211610	891
M31087719	C05	31087719	2340
M43542566	B05	43542566	1065
M65419276	C03	65419276	2179
M17988	Scaffold0	17988	2837
M19571416	B03	19571416	665
M4559405	B06	4559405	1149
M4172072	B05	4172072	924
M26259161	C03	26259161	2084
M8146587	B05	8146587	940
M10590808	C01	10590808	1860
M44305813	B02	44305813	420
M42649191	C01	42649191	1902
M12537100	C06	12537100	2384
M19674110	C09	19674110	2774
M18360483	C03	18360483	2067
M9996013	C06	9996013	2378
M15177057	C05	15177057	2324
M15517529	C05	15517529	2329
M1879533	B04	1879533	753
M32478033	C08	32478033	2656
M36274189	B07	36274189	1516
M2209132	B08	2209132	1567
M42246576	C04	42246576	2234
M3581751	B06	3581751	1137
M4093186	B08	4093186	1583
M41828847	C01	41828847	1900
M47605500	B02	47605500	451
M23414075	B03	23414075	681
M42025385	C05	42025385	2351
M29644412	C03	29644412	2092
M26060348	B08	26060348	1710
M2934989	B04	2934989	759
M46596430	B08	46596430	1754
M15225470	C05	15225470	2325
M46775931	C07	46775931	2575
M5360171	B06	5360171	1156
M14304563	B01	14304563	74
M27885017	C02	27885017	1976
M9877057	C09	9877057	2756
M641409	B02	641409	248

M4499714	B02	4499714	276
M15668238	C03	15668238	2052
M36128172	B06	36128172	1250
M20496364	B04	20496364	875
M27617258	C05	27617258	2338
M29221444	C02	29221444	1993
M7391059	B04	7391059	800
M37547406	B06	37547406	1258
M26332067	B03	26332067	703
M20271503	C06	20271503	2401
M22102589	B04	22102589	883
M11103905	B01	11103905	53
M12938020	B04	12938020	844
M1325604	B03	1325604	551
M45570472	C05	45570472	2365
M35406417	B01	35406417	191
M30210130	B03	30210130	724
M12430688	C03	12430688	2045
M44746991	B02	44746991	430
M9838679	C03	9838679	2040
M29649115	B08	29649115	1717
M46678117	B02	46678117	441
M12934061	C07	12934061	2477
M38949825	B02	38949825	370
M3291116	B01	3291116	19
M22771811	C08	22771811	2615
M5926987	C04	5926987	2198
M37652733	B01	37652733	227
M14134097	B08	14134097	1670
M52071145	C04	52071145	2255
M23454627	C06	23454627	2410
M31888028	B02	31888028	319
M34547273	B01	34547273	179
M37313529	C06	37313529	2447
M3948605	B03	3948605	580
M28634649	B06	28634649	1202
M9220831	B01	9220831	44
M14138296	C01	14138296	1868
M22131626	B06	22131626	1190
M13782813	C01	13782813	1866
M34970384	B02	34970384	338
M22709145	B03	22709145	676
M22248097	B02	22248097	298
M48176159	B08	48176159	1762
M20109754	C09	20109754	2780
M60709831	C03	60709831	2157
M54791691	B02	54791691	515

M26840242	B04	26840242	886
M27066259	B03	27066259	709
M32861786	B01	32861786	151
M33330252	B01	33330252	161
M11732513	C09	11732513	2759
M17341781	C06	17341781	2395
M7406439	C05	7406439	2291
M39543120	B02	39543120	373
M16937	B05	16937	899
M30215491	C04	30215491	2222
M22523612	C02	22523612	1965
M10145239	B02	10145239	291
M45500480	B02	45500480	431
M25424399	B03	25424399	694
M1331482	B02	1331482	257
M42034434	B02	42034434	402
M1018519	C04	1018519	2181
M36048510	B02	36048510	346
M12638826	B05	12638826	957
M15999026	B05	15999026	1009
M25379370	C03	25379370	2079
M36188039	B01	36188039	204
M716643	C02	716643	1921
M17208190	C02	17208190	1956
M44020644	C05	44020644	2356
M34249862	B01	34249862	175
M42014245	C07	42014245	2549
M1414999	B08	1414999	1561
M17692339	B04	17692339	866
M5784585	C08	5784585	2592
M46752178	B05	46752178	1085
M3521133	B04	3521133	765
M16214338	B03	16214338	645
M24857700	B07	24857700	1455
M1541940	B02	1541940	262
M6447712	B08	6447712	1619
M1303670	B04	1303670	744
M23869545	B03	23869545	686
M46216836	C04	46216836	2241
M51557621	B08	51557621	1790
M27635417	C08	27635417	2637
M35402866	B02	35402866	343
M9836652	B08	9836652	1631
M32638862	C06	32638862	2430
M42915709	C01	42915709	1906
M38042081	B05	38042081	1039
M28358185	C04	28358185	2220

M7133986	B03	7133986	598
M2754654	C02	2754654	1929
M63795841	C03	63795841	2170
M2428485	B02	2428485	266
M9067656	C01	9067656	1850
M6810238	B01	6810238	35
M58739157	C03	58739157	2151
M45148085	C09	45148085	2809
M11507409	B04	11507409	832
M44713069	C02	44713069	2007
M2055650	B03	2055650	564
M17240057	B03	17240057	649
M42949803	B02	42949803	415
M1705261	B05	1705261	911
M13205920	B08	13205920	1656
M26917774	C02	26917774	1970
M27846655	B08	27846655	1713
M46034416	B02	46034416	434
M1915073	B01	1915073	12
M32575820	B01	32575820	144
M1815484	B03	1815484	560
M1367319	B04	1367319	746
M9295241	C01	9295241	1852
M3738500	C05	3738500	2280
M33679585	B01	33679585	164
M3940842	B03	3940842	579
M236551	B07	236551	1279
M39802625	B02	39802625	379
M1478076	B02	1478076	261
M8284717	C03	8284717	2036
M5871481	B05	5871481	930
M11692350	C05	11692350	2306
M7197702	C07	7197702	2466
M35710960	C06	35710960	2439
M52725098	C09	52725098	2824
M14829896	C06	14829896	2393
M42827927	C08	42827927	2714
M40508471	B08	40508471	1725
M37644910	B02	37644910	356
M38481552	B02	38481552	366
M15782306	B08	15782306	1683
M49421167	B02	49421167	467
M22594162	B04	22594162	884
M13627714	B08	13627714	1662
M7586321	B02	7586321	286
M36183376	B01	36183376	203
M36322451	B08	36322451	1720

M64380060	C03	64380060	2173
M10975452	B04	10975452	827
M23512043	C07	23512043	2493
M54862298	B02	54862298	517
M47714663	B02	47714663	453
M1094657	C05	1094657	2269
M26228287	C02	26228287	1966
M11101134	B05	11101134	952
M4509546	B08	4509546	1588
M13753946	C03	13753946	2046
M837931	C06	837931	2371
M15484162	C01	15484162	1874
M13682201	B08	13682201	1664
M35399179	C02	35399179	1997
M35343479	B01	35343479	190
M5244925	C02	5244925	1938
M8909148	B04	8909148	811
M2765682	C01	2765682	1822
M7485939	C05	7485939	2292
M27956912	C02	27956912	1978
M2383881	C05	2383881	2275
M41375392	C05	41375392	2349
M8997488	B02	8997488	289
M44694525	C07	44694525	2564
M7621021	C08	7621021	2596
M19426842	B02	19426842	294
M16500028	B08	16500028	1685
M18832180	C06	18832180	2396
M36146749	B01	36146749	201
M14311948	B08	14311948	1673
M1261244	B01	1261244	9
M9860243	B01	9860243	48
M10167879	C01	10167879	1856
M33774256	B03	33774256	731
M36935266	C06	36935266	2445
M42923498	C08	42923498	2718
M21353435	C06	21353435	2404
M27243765	B03	27243765	710
M19405247	B07	19405247	1429
M55761741	B02	55761741	526
M4204745	C04	4204745	2191
M6428298	B06	6428298	1166
M37480497	C08	37480497	2688
M5976561	B06	5976561	1162
M32489472	B06	32489472	1229
M20875588	B03	20875588	671
M38074193	B05	38074193	1040

M44691287	C04	44691287	2237
M18283879	B08	18283879	1696
M14306655	B03	14306655	636
M25763797	B03	25763797	698
M65327316	C03	65327316	2176
M10823589	C04	10823589	2210
M44457919	B08	44457919	1744
M17823624	B04	17823624	868
M20414396	B01	20414396	81
M36888188	B06	36888188	1254
M19326993	B03	19326993	660
M896420	B04	896420	738
M49641558	C02	49641558	2012
M12657412	B05	12657412	958
M12766997	B03	12766997	627
M12738308	B05	12738308	961
M69473	B07	69473	1276
M37097413	B01	37097413	221
M32039096	C06	32039096	2428
M31582075	C07	31582075	2508
M10546315	B03	10546315	609
M28011049	B01	28011049	95
M12191107	B06	12191107	1181
M35959145	B01	35959145	199
M27653812	C06	27653812	2422
M38635858	C06	38635858	2456
M15254100	B08	15254100	1681
M27851319	B08	27851319	1714
M3840724	C02	3840724	1931
M39196651	B06	39196651	1268
M21992408	B04	21992408	882
M7766774	C06	7766774	2373
M15271195	C05	15271195	2326
M17881332	B03	17881332	653
M44475587	B02	44475587	426
M51838152	B02	51838152	493
M11138272	C07	11138272	2468
M51684015	B02	51684015	488
M7469412	B02	7469412	284
M36818502	B01	36818502	217
M1679878	C03	1679878	2022
M6397643	C08	6397643	2593
M52779833	B02	52779833	500
M2101276	B04	2101276	757
M122113	C02	122113	1913
M13053020	B08	13053020	1653
M2332510	B05	2332510	914

M5539056	C04	5539056	2195
M25975699	B03	25975699	700
M36258099	C02	36258099	1999
M23212781	B06	23212781	1195
M26780196	B01	26780196	87
M24118286	C03	24118286	2077
M48570484	B02	48570484	461
M14992384	B05	14992384	996
M50571815	C09	50571815	2819
M3041385	C03	3041385	2031
M15416354	B04	15416354	852
M1469208	B03	1469208	555
M7140307	B04	7140307	798
M40870405	C05	40870405	2348
M33071469	B05	33071469	1033
M39314942	B06	39314942	1269
M3672299	B03	3672299	577
M35745521	B01	35745521	195
M54933260	B02	54933260	519
M2848655	B03	2848655	571
M4609957	B01	4609957	27
M2338678	B05	2338678	915
M37057237	C08	37057237	2682
M40882192	C08	40882192	2706
M22644690	B06	22644690	1192
M44470879	C05	44470879	2362
M37155799	C07	37155799	2528
M3916242	B06	3916242	1144
M20058967	B05	20058967	1024
M22004235	C06	22004235	2406
M51508583	B08	51508583	1789
M36930480	B01	36930480	219
M45399935	C07	45399935	2568
M43676804	B05	43676804	1067
M45922383	B08	45922383	1749
M20230862	B04	20230862	871
M27093034	B01	27093034	88
M12864396	C06	12864396	2386
M18699676	B02	18699676	293
M8573734	B08	8573734	1626
M490036	B07	490036	1283
M27644524	C02	27644524	1974
M44497876	B08	44497876	1746
M27546196	C02	27546196	1973
M15538378	B04	15538378	854
M12230352	B08	12230352	1646
M47901649	B05	47901649	1089

M5175847	C09	5175847	2738
M43197791	B08	43197791	1733
M38738044	C06	38738044	2458
M23340729	B03	23340729	680
M11162711	B08	11162711	1635
M42562558	B02	42562558	408
M31468005	B06	31468005	1218
M39909939	B02	39909939	381
M47738651	B02	47738651	454
M49377832	B02	49377832	466
M2125959	B05	2125959	912
M28766854	B06	28766854	1205
M15656982	B06	15656982	1184
M42933789	C07	42933789	2552
M3528992	B02	3528992	269
M49788428	B02	49788428	473
M25918486	C08	25918486	2626
M6317612	B06	6317612	1165
M27722847	B01	27722847	91
M1605347	B01	1605347	10
M5809862	B04	5809862	787
M23695279	B03	23695279	683
M22247841	C07	22247841	2487
M49422447	B08	49422447	1768
M31615030	C07	31615030	2509
M20075729	C04	20075729	2218
M20413575	B04	20413575	874
M20797571	C08	20797571	2612
M13497756	B08	13497756	1658
M7986242	B05	7986242	939
M6505726	B04	6505726	795
M810906	C02	810906	1923
M49706541	B08	49706541	1772
M8293560	B01	8293560	39
M62810958	C03	62810958	2164
M15047059	C07	15047059	2483
M6459485	B04	6459485	794
M46656342	C04	46656342	2246
M3993221	C04	3993221	2190
M27109856	B01	27109856	89
M28881591	C02	28881591	1988
M6539471	B08	6539471	1621
M17515464	B01	17515464	79
M35362464	C07	35362464	2517
M46775424	B02	46775424	443
M12602275	B08	12602275	1649
M34372995	C04	34372995	2228

M39753501	C05	39753501	2346
M1380530	B04	1380530	747
M21808589	C06	21808589	2405
M35807668	B02	35807668	345
M48886234	B05	48886234	1094
M6058262	C03	6058262	2035
M11300293	B03	11300293	613
M39241232	C06	39241232	2462
M37098660	C08	37098660	2683
M44037492	C07	44037492	2557
M16701331	B01	16701331	77
M37182714	C08	37182714	2686
M34187072	B01	34187072	173
M12142124	C08	12142124	2603
M33555953	C07	33555953	2512
M52883256	C03	52883256	2139
M754260	B08	754260	1556
M28325919	B02	28325919	310
M471906	C02	471906	1916
M41999006	C04	41999006	2233
M19142760	C06	19142760	2398
M59059814	C03	59059814	2154
M39374580	B06	39374580	1271
M25261866	B03	25261866	693
M1196710	B03	1196710	547
M38696015	C02	38696015	2000
M13341533	C06	13341533	2389
M32231518	B06	32231518	1225
M24799575	C07	24799575	2497
M42787608	B02	42787608	410
M7042955	C05	7042955	2285
M18154007	C07	18154007	2484
M30927914	C03	30927914	2094
M2185175	B07	2185175	1296
M28701516	B06	28701516	1203
M51226823	B05	51226823	1108
M11927408	B08	11927408	1644
M18331784	C04	18331784	2215
M24413203	C07	24413203	2494
M37483619	B01	37483619	225
M33999387	C06	33999387	2436
M14082294	B08	14082294	1668
M49840193	B05	49840193	1101
M1156952	B03	1156952	546
M50435153	B02	50435153	478
M3970060	B05	3970060	923
M7508838	C01	7508838	1844

M791865	B06	791865	1111
M10919017	B01	10919017	52
M44162274	B08	44162274	1739
M15134210	B04	15134210	850
M12604068	C05	12604068	2309
M48442113	B02	48442113	460
M10582687	C01	10582687	1859
M17346128	B03	17346128	650
M12161014	B04	12161014	841
M30553975	C08	30553975	2649
M4299116	C02	4299116	1932
M9576448	B05	9576448	945
M27820512	C02	27820512	1975
M14410420	C05	14410420	2318
M36757680	B02	36757680	351
M38378739	C08	38378739	2695
M34880918	B03	34880918	733
M13311096	B05	13311096	972
M49807771	B02	49807771	474
M4777133	C01	4777133	1831
M17566314	C01	17566314	1877
M36815101	C03	36815101	2119
M35033769	B02	35033769	341
M36950931	C08	36950931	2679
M38550487	C06	38550487	2455
M8893704	C09	8893704	2747
M2229453	B01	2229453	13
M6786241	C09	6786241	2741
M5040898	B08	5040898	1596
M11268568	B01	11268568	54
M5301969	B04	5301969	782
M33785837	B01	33785837	169
M2615928	B03	2615928	568
M18252796	C03	18252796	2065
M5268261	B06	5268261	1155
M38487862	B08	38487862	1722
M38434755	B01	38434755	234
M13733941	B05	13733941	985
M2332983	B01	2332983	14
M4924210	B03	4924210	588
M36696991	B01	36696991	212
M21458383	B06	21458383	1187
M48031343	B05	48031343	1092
M50953077	B08	50953077	1778
M49956270	B05	49956270	1103
M51239371	B08	51239371	1783
M49494533	B02	49494533	469

M7182957	C05	7182957	2286
M1400	C05	1400	2265
M48909217	B05	48909217	1095
M6823219	B01	6823219	36
M33113434	B02	33113434	330
M7497525	C05	7497525	2293
M14775623	C05	14775623	2321
M35008538	B02	35008538	340
M38045200	C08	38045200	2691
M35409148	C08	35409148	2673
M2031997	C09	2031997	2726
M21035998	B05	21035998	1026
M51705845	B08	51705845	1793
M11462064	B03	11462064	616
M305010	C02	305010	1915
M4650155	B06	4650155	1151
M13049855	B05	13049855	967
M29013309	B06	29013309	1209
M2865109	B05	2865109	920
M19604896	C05	19604896	2333
M6469785	C02	6469785	1943
M841059	B03	841059	540
M26159047	C03	26159047	2082
M10071430	B02	10071430	290
M23227522	C08	23227522	2617
M1255330	B03	1255330	549
M19925241	C02	19925241	1959
M7666079	C01	7666079	1845
M28697645	C02	28697645	1986
M4888488	B05	4888488	925
M496956	B05	496956	901
M27369538	B01	27369538	90
M4540787	B03	4540787	585
M459599	C01	459599	1814
M37618073	B01	37618073	226
M36371765	B01	36371765	207
M19214300	C07	19214300	2486
M1337580	B03	1337580	552
M41039501	B02	41039501	392
M26839860	C02	26839860	1969
M20047351	B05	20047351	1023
M32751677	C08	32751677	2659
M6279571	B08	6279571	1616
M30111816	B03	30111816	722
M13698264	B05	13698264	982
M52712349	B02	52712349	496
M406930	B03	406930	533

M15437869	C03	15437869	2048
M926678	B01	926678	6
M28582412	B02	28582412	314
M2766521	C04	2766521	2186
M28948686	B01	28948686	108
M1107	Scaffold0	1107	2838
M51950541	B08	51950541	1796
M44061571	B08	44061571	1737
M15395418	C03	15395418	2047
M17630383	B08	17630383	1689
M50348460	B02	50348460	475
M4670233	B06	4670233	1152
M3555215	B01	3555215	20
M14817476	B03	14817476	638
M11527520	B04	11527520	833
M10735258	B03	10735258	611
M34370126	B01	34370126	178
M52432723	B08	52432723	1801
M16414275	B03	16414275	646
M22355574	C02	22355574	1963
M6059173	B04	6059173	790
M14425865	C05	14425865	2319
M56512940	B02	56512940	530
M43185747	C01	43185747	1908
M8717667	B03	8717667	602
M16548981	B01	16548981	76
M30247851	B03	30247851	725
M4573	Scaffold0	4573	2839
M13935931	B08	13935931	1666
M7322689	B03	7322689	599
M23251445	B03	23251445	679
M1360227	B05	1360227	905
M46686385	B05	46686385	1084
M54195117	B02	54195117	510
M34772053	C08	34772053	2669
M18538889	C03	18538889	2073
M6373344	C04	6373344	2199
M48218903	B08	48218903	1763
M35710939	C07	35710939	2522
M28205703	B06	28205703	1200
M1874870	B08	1874870	1564
M3096790	B03	3096790	576
M24049583	B08	24049583	1704
M40726076	C05	40726076	2347
M19915106	C02	19915106	1958
M4610735	B03	4610735	586
M3586808	B04	3586808	769

M28177817	B02	28177817	307
M43490344	C05	43490344	2353
M28923119	B01	28923119	107
M28849284	B06	28849284	1207
M34757499	B01	34757499	187
M1102409	B03	1102409	545
M50372976	C04	50372976	2253
M32246160	B06	32246160	1226
M3802837	B02	3802837	271
M9309991	B08	9309991	1630
M31923371	B02	31923371	320
M16403540	B08	16403540	1684
M9433865	C05	9433865	2297
M4515	BS00403	4515	1810
M1574084	B03	1574084	556
M53053829	C09	53053829	2826
M5570173	B06	5570173	1160
M8182956	B06	8182956	1178
M36752804	B01	36752804	214
M32922489	B01	32922489	154
M50416901	B02	50416901	476
M12129995	B03	12129995	623
M4026129	B02	4026129	273
M957920	B05	957920	902
M43307129	B08	43307129	1734
M6592383	C04	6592383	2201
M10412345	B03	10412345	608
M20091121	C02	20091121	1962
M14887684	B08	14887684	1677
M51680935	B08	51680935	1792
M24024946	B03	24024946	690
M27747902	B08	27747902	1712
M36093261	C05	36093261	2344
M8440439	C06	8440439	2376
M2803896	B06	2803896	1126
M28883874	B06	28883874	1208
M42881544	B02	42881544	412
M19852566	C02	19852566	1957
M52824458	B08	52824458	1808
M36736760	B01	36736760	213
M11907888	B03	11907888	619
M12713330	B05	12713330	960
M50711422	B02	50711422	481
M14642921	C07	14642921	2482
M4861421	B04	4861421	775
M19406584	C06	19406584	2400
M13243059	B05	13243059	970

M40818944	C07	40818944	2540
M47768998	B02	47768998	455
M28978602	C02	28978602	1990
M47528622	C07	47528622	2582
M1084227	B02	1084227	254
M7323542	C01	7323542	1841
M32116377	C05	32116377	2341
M43841064	C07	43841064	2556
M43005769	C08	43005769	2720
M33015753	B02	33015753	326
M48958078	C07	48958078	2587
M18388233	C03	18388233	2068
M52493124	B08	52493124	1803
M31318234	C08	31318234	2652
M8004147	B02	8004147	288
M6140370	B06	6140370	1164
M8985206	C09	8985206	2751
M3187029	B06	3187029	1132
M22707682	C06	22707682	2409
M58540337	C03	58540337	2148
M22440027	C02	22440027	1964
M23811871	B03	23811871	685
M12200264	B03	12200264	624
M46767659	C07	46767659	2574
M13078010	B03	13078010	630
M371482	C06	371482	2370
M54612389	B02	54612389	513
M5497423	B01	5497423	31
M49001056	B05	49001056	1096
M39810326	B04	39810326	894
M7454432	C01	7454432	1843
M922459	B03	922459	544
M12273819	C05	12273819	2307
M43703147	B05	43703147	1068
M41339833	B02	41339833	398
M51718882	B08	51718882	1794
M3185664	C03	3185664	2032
M9590232	C09	9590232	2753
M44314426	B08	44314426	1741
M18012583	B04	18012583	869
M49348482	B08	49348482	1766
M6942753	B05	6942753	934
M4348412	C02	4348412	1933
M15109512	B04	15109512	849
M39574536	B01	39574536	242
M39378369	C08	39378369	2698
M6770879	C08	6770879	2594

M33883433	C07	33883433	2514
M26468465	C07	26468465	2499
M48925807	C07	48925807	2586
M4625893	C09	4625893	2736
M94318	B02	94318	245
M17711604	B08	17711604	1690
M52187273	B02	52187273	495
M1846448	C05	1846448	2273
M65402411	C03	65402411	2178
M32505021	B01	32505021	141
M378298	B01	378298	2
M44211911	C05	44211911	2359
M397981	B03	397981	532
M3191013	C08	3191013	2589
M9481138	C05	9481138	2298
M52312467	C04	52312467	2257
M2899647	C03	2899647	2027
M8825754	B01	8825754	42
M46441899	B08	46441899	1752
M33581415	C06	33581415	2433
M7717217	C05	7717217	2296
M14826078	B03	14826078	639
M15187095	B08	15187095	1680
M29603192	B06	29603192	1213
M15484172	C05	15484172	2328
M36176492	B01	36176492	202
M5622137	C01	5622137	1832
M44286062	C02	44286062	2006
M9246118	B08	9246118	1629
M1470417	B05	1470417	907
M9692990	B01	9692990	47
M45902777	B05	45902777	1076
M13064451	B08	13064451	1654
M8878643	B05	8878643	943
M6695012	B03	6695012	595
M31782498	C07	31782498	2510
M16819476	B05	16819476	1017
M1201855	B03	1201855	548
M29621401	B08	29621401	1716
M1187294	B01	1187294	8
M8090442	B03	8090442	600
M38684274	C06	38684274	2457
M1543697	C05	1543697	2272
M31183688	B06	31183688	1216
M16864338	B08	16864338	1687
M38305889	B01	38305889	232
M796920	C02	796920	1922

M21388466	B02	21388466	296
M15324933	C01	15324933	1873
M2609959	B02	2609959	267
M15583795	B05	15583795	1006
M15900320	B03	15900320	644
M36746055	B02	36746055	350
M23671249	B02	23671249	301
M26739158	C08	26739158	2628
M18101902	B08	18101902	1694
M32031117	B03	32031117	728
M35579543	B02	35579543	344
M22591257	B02	22591257	299
M34749255	B02	34749255	336
M28513964	C06	28513964	2425
M11785421	C08	11785421	2602
M6565049	C05	6565049	2284
M16897481	B05	16897481	1018
M32935532	B01	32935532	155
M7526326	C05	7526326	2294
M34133527	C08	34133527	2666
M39788091	B05	39788091	1044
M32256667	B06	32256667	1227
M15522391	C03	15522391	2049
M1891142	B04	1891142	754
M4854378	B01	4854378	28
M7800896	B06	7800896	1174
M5773575	C09	5773575	2739
M42939366	C08	42939366	2719
M23292258	B06	23292258	1196
M47398867	C07	47398867	2581
M33370226	C07	33370226	2511
M42624452	C02	42624452	2003
M53433106	B02	53433106	506
M27231641	C03	27231641	2088
M18137353	B08	18137353	1695
M15724386	C03	15724386	2054
M2650930	B05	2650930	917
M2815837	C01	2815837	1823
M37143363	C08	37143363	2684
M7898905	B04	7898905	803
M5744260	B04	5744260	785
M5286606	C02	5286606	1939
M53524268	C09	53524268	2828
M4214778	B03	4214778	582
M15878722	B04	15878722	859
M826615	C05	826615	2267
M10267918	C07	10267918	2467

M1240336	C01	1240336	1816
M39740592	C05	39740592	2345
M965668	C02	965668	1924
M56690885	B02	56690885	531
M34538184	C04	34538184	2229
M10266620	B08	10266620	1634
M51863469	B02	51863469	494
M41534993	C08	41534993	2707
M24637536	C06	24637536	2414
M11860441	B04	11860441	837
M23293979	C03	23293979	2075
M8942505	C09	8942505	2749
M26281150	B05	26281150	1029
M28272942	C02	28272942	1981
M27490480	C03	27490480	2091
M588163	B03	588163	536
M38539150	C06	38539150	2454
M8194355	C01	8194355	1848
M7673034	B05	7673034	937
M38264314	B01	38264314	231
M8856581	C09	8856581	2746
M18678165	B08	18678165	1699
M771716	B01	771716	5
M3858161	B06	3858161	1141
M14219190	B08	14219190	1672
M19664852	B03	19664852	666
M830015	C03	830015	2019
M2990891	B06	2990891	1129
M50805497	B05	50805497	1105
M55963616	B02	55963616	527
M15250614	B05	15250614	1000
M39084687	B05	39084687	1042
M51025755	B08	51025755	1779
M6933512	B06	6933512	1168
M893990	B03	893990	542
M55365765	B02	55365765	524
M5157568	B04	5157568	780
M49803582	B05	49803582	1100
M28350051	C02	28350051	1982
M6272798	B05	6272798	932
M12893254	B05	12893254	965
M14121513	C01	14121513	1867
M46370436	C04	46370436	2244
M28579110	C07	28579110	2501
M46528406	B08	46528406	1753
M42670373	C08	42670373	2712
M748491	B06	748491	1110

M34574437	B01	34574437	181
M54651645	B02	54651645	514
M13191732	C07	13191732	2481
M24022325	C08	24022325	2620
M3006630	B01	3006630	16
M46704611	C04	46704611	2247
M27946860	B02	27946860	305
M18505994	B08	18505994	1698
M30198840	C08	30198840	2648
M20530590	C06	20530590	2402
M15684571	C03	15684571	2053
M2377055	B02	2377055	265
M21534123	B06	21534123	1188
M28810928	B01	28810928	104
M27415067	C08	27415067	2633
M31178186	C07	31178186	2506
M49692022	B02	49692022	471
M2677608	C09	2677608	2729
M43160798	C02	43160798	2004
M18674047	B03	18674047	655
M46514281	B02	46514281	439
M13449390	C05	13449390	2315
M39567720	B08	39567720	1724
M38953901	C06	38953901	2460
M18021947	B08	18021947	1693
M10394589	B05	10394589	949
M14969920	C01	14969920	1871
M34809547	B02	34809547	337
M46235291	B05	46235291	1079
M5911687	C04	5911687	2197
M46004320	C04	46004320	2239
M42839836	B05	42839836	1054
M1291400	B03	1291400	550
M2269554	C09	2269554	2727
M4172685	B02	4172685	274
M30490292	B04	30490292	887
M3416295	B08	3416295	1579
M4051274	C09	4051274	2733
M2347810	B06	2347810	1122
M601786	B03	601786	537
M13512754	B05	13512754	979
M27281665	B08	27281665	1711
M31979889	B01	31979889	129
M35216714	B02	35216714	342
M7078104	B06	7078104	1171
M31513931	B06	31513931	1220
M7004417	B04	7004417	797

M33374298	C05	33374298	2342
M2685267	B05	2685267	918
M552186	B03	552186	534
M13081824	B05	13081824	968
M1461683	B02	1461683	260
M7375464	C05	7375464	2289
M40244204	C08	40244204	2704
M20246973	B08	20246973	1702
M294311	B01	294311	1
M46899859	B02	46899859	445
M37502748	C08	37502748	2689
M3688993	B02	3688993	270
M14515405	B06	14515405	1182
M4373314	B01	4373314	25
M39304290	C07	39304290	2532
M11901349	B04	11901349	838
M8577703	B05	8577703	941
M1126022	C02	1126022	1925
M14429532	C01	14429532	1870
M22836408	B06	22836408	1193
M13876203	B05	13876203	989
M3377116	B06	3377116	1136
M28781548	B06	28781548	1206
M50427751	B02	50427751	477
M13790489	B05	13790489	988
M32895502	B02	32895502	324
M44392636	B08	44392636	1743
M23993708	C06	23993708	2413
M39576905	C07	39576905	2534
M49683876	B02	49683876	470
M26961002	C08	26961002	2631
M33090194	B02	33090194	328
M41815457	C07	41815457	2547
M28101448	C02	28101448	1979
M50196948	B08	50196948	1773
M691263	B03	691263	538
M35977515	B01	35977515	200
M10616447	B05	10616447	950
M7403032	B08	7403032	1622
M4980537	B04	4980537	776
M36906948	C08	36906948	2676
M36921833	C08	36921833	2677
M3502867	C01	3502867	1824
M27007917	C02	27007917	1972
M17763064	B03	17763064	651
M51456747	B08	51456747	1787
M43484267	B08	43484267	1735

M3480976	B04	3480976	764
M26789704	C02	26789704	1967
M36743154	C07	36743154	2526
M50634367	B02	50634367	480
M9507189	B01	9507189	46
M8038102	C01	8038102	1846
M13536171	B01	13536171	70
M11339048	B03	11339048	614
M51171070	B02	51171070	483
M15821335	C03	15821335	2056
M9683563	C05	9683563	2301
M55098383	B02	55098383	520
M4601516	B06	4601516	1150
M2171423	C02	2171423	1927
M29026522	B06	29026522	1210
M12673150	B03	12673150	626
M27732195	B03	27732195	714
M54109671	C09	54109671	2831
M9494884	C05	9494884	2299
M659349	C02	659349	1920
M38551576	B01	38551576	237
M42820271	C08	42820271	2713
M25609010	B06	25609010	1197
M12070979	B03	12070979	620
M28141809	B06	28141809	1198
M51294247	B08	51294247	1784
M39930425	B02	39930425	382
M6936026	B01	6936026	37
M52182198	C09	52182198	2822
M53528124	B02	53528124	507
M38418619	B01	38418619	233
M52890988	C09	52890988	2825
M25129878	C07	25129878	2498
M30110818	C08	30110818	2647
M61559534	C03	61559534	2160
M22140666	C06	22140666	2407
M48405706	B08	48405706	1764
M10664703	C08	10664703	2601
M2143283	B06	2143283	1120
M5830451	C01	5830451	1834
M30542916	B01	30542916	121
M46159914	B05	46159914	1078
M16664540	C09	16664540	2761
M52574353	C09	52574353	2823
M2436074	B06	2436074	1124
M42368454	C08	42368454	2710
M3329179	B06	3329179	1134

M29303857	B06	29303857	1212
M7040382	B06	7040382	1170
M7454308	B04	7454308	801
M15228080	B03	15228080	640
M51571943	B02	51571943	486
M22608544	C08	22608544	2613
M12631588	B04	12631588	843
M11454141	C07	11454141	2470
M42839123	C08	42839123	2715
M1320620	C05	1320620	2270
M35213241	C08	35213241	2672
M11941206	B08	11941206	1645
M28254119	C02	28254119	1980
M17651732	B04	17651732	865
M37216084	C08	37216084	2687
M27028799	C03	27028799	2085
M860950	B04	860950	737
M8970751	C09	8970751	2750
M1445883	B02	1445883	259
M54846056	B02	54846056	516
M5001323	B03	5001323	589
M2920704	C03	2920704	2029
M12350835	B05	12350835	956
M4639001	C02	4639001	1935
M35016454	C07	35016454	2515
M25832931	C08	25832931	2625
M7477041	B05	7477041	936
M38348111	C08	38348111	2694
M23578962	B03	23578962	682
M34293499	B01	34293499	176
M5004021	B04	5004021	777
M38015049	B01	38015049	230
M36909925	C07	36909925	2527
M2729384	B01	2729384	15
M2220693	C02	2220693	1928
M4387881	B03	4387881	584
M8706359	B01	8706359	41
M1441619	C03	1441619	2020
M33279274	B01	33279274	159
M13611977	B05	13611977	981
M9607329	C05	9607329	2300
M3729563	B01	3729563	21
M46190087	C04	46190087	2240
M3364652	B06	3364652	1135
M37399988	B01	37399988	224
M7711545	C04	7711545	2208
M2374007	C09	2374007	2728

M22891303	C07	22891303	2491
M39209826	C06	39209826	2461
M7677067	B08	7677067	1623
M43136869	B05	43136869	1059
M41031029	C07	41031029	2542
M30753677	B02	30753677	316
M42607342	C07	42607342	2550
M38505566	C08	38505566	2696
M6530828	B02	6530828	283
M28379621	C06	28379621	2424
M13965490	B05	13965490	991
M27913858	C02	27913858	1977
M986855	B06	986855	1112
M42904665	B05	42904665	1055
M39335630	C07	39335630	2533
M38922335	B01	38922335	238
M18562909	C03	18562909	2074
M6978807	C02	6978807	1947
M30876392	C06	30876392	2427
M7950439	B02	7950439	287
M7388287	C05	7388287	2290
M28713448	B01	28713448	102
M9524678	C08	9524678	2599
M13633228	C01	13633228	1865
M47488535	B08	47488535	1758
M24065818	C08	24065818	2622
M51028911	C04	51028911	2254
M11516254	C03	11516254	2042
M43213830	C01	43213830	1909
M49326917	B08	49326917	1765
M46014207	C07	46014207	2570
M49335673	B02	49335673	465
M7585878	C09	7585878	2742
M26011655	C08	26011655	2627
M9989081	B08	9989081	1633
M11997810	B05	11997810	953
M15314381	B03	15314381	641
M90572	B07	90572	1277
M2310155	B08	2310155	1568
M15506769	B04	15506769	853
M28461317	B02	28461317	312
M39594419	B01	39594419	243
M9325158	C01	9325158	1853
M16816080	C03	16816080	2057
M5500045	C04	5500045	2194
M13207017	B05	13207017	969
M38100819	C08	38100819	2692

M4345550	B01	4345550	24
M12248205	B03	12248205	625
M13091228	C07	13091228	2479
M34555734	B01	34555734	180
M44795379	B08	44795379	1747
M13035119	C07	13035119	2478
M37198066	B01	37198066	222
M17599873	B04	17599873	864
M50554395	B02	50554395	479
M3385274	B05	3385274	922
M20396340	B06	20396340	1186
M22934619	B03	22934619	677
M35674183	C07	35674183	2521
M12637599	C07	12637599	2476
M36725958	C07	36725958	2525
M1161594	C09	1161594	2722
M43018695	B05	43018695	1057
M44135032	B08	44135032	1738
M29037964	B06	29037964	1211
M1694576	B02	1694576	263
M38063512	C07	38063512	2530
M37049970	B06	37049970	1255
M23353473	C08	23353473	2618
M33002238	B01	33002238	156
M28258953	B02	28258953	309
M19398366	B03	19398366	662
M14165086	B08	14165086	1671
M51498389	B08	51498389	1788
M24558340	C01	24558340	1882
M12293652	C03	12293652	2044
M30000839	C02	30000839	1994
M37959676	C08	37959676	2690
M23126820	B03	23126820	678
M50191831	C04	50191831	2252
M33637028	C07	33637028	2513
M13723159	B05	13723159	984
M54445823	C04	54445823	2263
M11605914	B08	11605914	1641
M516733	C02	516733	1917
M13554488	B08	13554488	1659
M22267158	C05	22267158	2334
M37720754	B01	37720754	228
M1170718	C09	1170718	2723
M7982443	B06	7982443	1176
M13196020	C01	13196020	1864
M35906351	B01	35906351	196
M3310627	C04	3310627	2188

M4588920	C02	4588920	1934
M24577600	C07	24577600	2495
M36771096	B01	36771096	216
M13622850	B08	13622850	1661
M51577248	B08	51577248	1791
M6277854	C01	6277854	1838
M10106990	B03	10106990	604
M52791665	B02	52791665	501
M52694238	B08	52694238	1807
M39082799	B01	39082799	239
M40850723	B05	40850723	1050
M2747288	C01	2747288	1819
M46678237	B08	46678237	1755
M28773199	B01	28773199	103
M12093441	B03	12093441	621
M47774859	B02	47774859	456
M6291984	C09	6291984	2740
M11189952	C03	11189952	2041
M2755620	C01	2755620	1820
M29320757	C04	29320757	2221
M33618024	B02	33618024	334
M31671778	B01	31671778	125
M2758730	C01	2758730	1821
M16428988	B04	16428988	861
M55577827	B02	55577827	525
M1881249	C03	1881249	2025
M32460239	B04	32460239	888
M12834786	B03	12834786	628
M24038065	C08	24038065	2621
M7128671	C02	7128671	1949
M14795314	B08	14795314	1676
M9041720	B05	9041720	944
M46295695	C07	46295695	2571
M9982708	C01	9982708	1855
M32617147	C06	32617147	2429
M16319388	B05	16319388	1012
M28550285	C02	28550285	1984
M18345958	B03	18345958	654
M9696701	B05	9696701	946
M3879045	C09	3879045	2732
M29726719	C08	29726719	2641
M16467587	B05	16467587	1015
M43628610	B05	43628610	1066
M11766677	B08	11766677	1643
M51985278	B08	51985278	1797
M22791553	C07	22791553	2489
M30367138	C05	30367138	2339

M52826973	C03	52826973	2136
M63394977	C03	63394977	2166
M13354384	B05	13354384	974
M43210346	B04	43210346	897
M22963374	B02	22963374	300
M42967286	B05	42967286	1056
M18050105	B04	18050105	870
M62889458	C03	62889458	2165
M40220298	C07	40220298	2538
M1071272	B02	1071272	253
M4751987	C05	4751987	2282
M54892634	B02	54892634	518
M33832487	B02	33832487	335
M43620366	C07	43620366	2554
M22786604	C08	22786604	2616
M6519408	B03	6519408	594
M10193797	B03	10193797	606
M1354150	C02	1354150	1926
M32187108	B06	32187108	1224
M25016114	B05	25016114	1028
M17734553	C03	17734553	2063
M42681338	C01	42681338	1903
M47294636	B08	47294636	1757
M2729008	C01	2729008	1817
M16090016	B05	16090016	1011
M50745263	B08	50745263	1776
M49747943	B05	49747943	1099
M46666403	B05	46666403	1083
M24941564	C06	24941564	2415
M46497759	C04	46497759	2245
M47631436	B02	47631436	452
M6740001	C02	6740001	1946
M812826	B03	812826	539
M14037344	B01	14037344	72
M20883231	B04	20883231	878
M7272898	C05	7272898	2288
M44471474	B05	44471474	1074
M52675319	B08	52675319	1806
M13502307	B05	13502307	978
M31723571	B01	31723571	126
M37945862	C06	37945862	2451
M32539978	B02	32539978	323
M1670537	B06	1670537	1113
M53803009	B02	53803009	509
M36246070	B01	36246070	205
M52722446	B02	52722446	497
M13267095	B05	13267095	971

M1402869	B03	1402869	554
M31489563	B06	31489563	1219
M694980	C01	694980	1815
M36470161	C07	36470161	2524
M10158266	C06	10158266	2379
M38456612	B06	38456612	1263
M31604900	B01	31604900	124
M2027312	C04	2027312	2182
M21602054	B06	21602054	1189
M40426761	B05	40426761	1046
M4497891	B08	4497891	1587
M33172672	B02	33172672	331
M5129718	B03	5129718	590
M6083318	B01	6083318	33
M13770244	B05	13770244	987
M1787135	B04	1787135	749
M43528581	B05	43528581	1064
M23074047	C07	23074047	2492
M27872780	B02	27872780	304
M9911584	B08	9911584	1632
M46336200	C04	46336200	2243
M6575108	B06	6575108	1167
M16397910	B05	16397910	1013
M2132524	B04	2132524	758
M18652038	C08	18652038	2605
M23639215	C03	23639215	2076
M29687036	C08	29687036	2640
M21180152	B01	21180152	82
M23833298	C06	23833298	2412
M25647813	B03	25647813	697
M20752953	B03	20752953	667
M26225926	C03	26225926	2083
M62594437	C03	62594437	2162
M25613751	C06	25613751	2417
M46637983	C07	46637983	2573
M3311895	B06	3311895	1133
M1673813	C03	1673813	2021
M44508433	C07	44508433	2562
M47405030	B02	47405030	447
M13973645	B05	13973645	992
M29119288	B03	29119288	717
M55116373	B02	55116373	521
M40257546	C07	40257546	2539
M46024327	B05	46024327	1077
M24784714	C05	24784714	2335
M6422182	B08	6422182	1618
M34791128	C02	34791128	1996

M7512393	B02	7512393	285
M41717339	C07	41717339	2546
M6257099	C01	6257099	1837
M5041869	B04	5041869	778
M38208206	B05	38208206	1041
M42844127	C01	42844127	1904
M37473401	C07	37473401	2529
M12948945	B05	12948945	966
M7348546	C02	7348546	1952
M16049409	B05	16049409	1010
M24872986	C08	24872986	2623
M19413319	C08	19413319	2610
M43097696	B05	43097696	1058
M27344827	C06	27344827	2420
M30608266	C07	30608266	2504
M25992204	B01	25992204	84
M52772420	B02	52772420	499
M3783620	B06	3783620	1140
M40888083	B05	40888083	1051
M15148904	C01	15148904	1872
M53701981	B02	53701981	508
M46727819	C04	46727819	2248
M2404175	B06	2404175	1123
M36522807	B01	36522807	208
M15211363	B05	15211363	999
M17926940	B08	17926940	1692
M54407365	C09	54407365	2832
M43275703	B05	43275703	1061
M46479334	B05	46479334	1081
M25635761	B03	25635761	696
M46939684	C07	46939684	2577
M38537497	B01	38537497	236
M8614939	C04	8614939	2209
M37238870	B01	37238870	223
M5151543	C02	5151543	1937
M60897109	C03	60897109	2158
M5643067	C01	5643067	1833
M43193942	B05	43193942	1060
M7133433	C01	7133433	1840
M37713458	B05	37713458	1037
M41382273	B05	41382273	1052
M6321710	C01	6321710	1839
M30178342	B01	30178342	115
M30259168	B01	30259168	116
M13527638	B05	13527638	980
M594252	C02	594252	1919
M8245657	C06	8245657	2375

M25473631	B03	25473631	695
M11581996	B04	11581996	834
M17382235	B08	17382235	1688
M13705563	B05	13705563	983
M6796913	B03	6796913	596
M15633727	B05	15633727	1007
M52110393	B08	52110393	1798
M29611237	B06	29611237	1214
M33580241	B02	33580241	333
M16712831	B02	16712831	292
M11466571	B08	11466571	1639
M6191776	C05	6191776	2283
M49603253	B08	49603253	1770
M53225307	B02	53225307	504
M37777096	B01	37777096	229
M2741809	C01	2741809	1818
M12382781	C07	12382781	2472
M22505504	B03	22505504	673
M8128811	C01	8128811	1847
M7074558	C02	7074558	1948
M44350040	B08	44350040	1742
M7988986	C02	7988986	1953
M42813967	B05	42813967	1053
M46692118	B02	46692118	442
M17076309	C03	17076309	2060
M3995116	B01	3995116	23
M7860893	B06	7860893	1175
M3671412	B06	3671412	1138
M15532402	B05	15532402	1004
M7479611	B06	7479611	1173
M10129490	B03	10129490	605
M1278684	C09	1278684	2724
M1869469	C03	1869469	2024
M35187276	C08	35187276	2671
M50525691	B08	50525691	1775
M3127847	B01	3127847	18
M17855855	B03	17855855	652
M3196514	B08	3196514	1576
M30562014	B01	30562014	122
M54211104	B02	54211104	511
M40750882	B05	40750882	1049
M42606230	C08	42606230	2711
M48154519	B08	48154519	1761
M32244315	B02	32244315	322
M11538327	B08	11538327	1640
M3449076	C05	3449076	2278
M954285	B07	954285	1291

M26755796	C08	26755796	2629
M16527444	B05	16527444	1016
M19291952	B03	19291952	659
M15120649	B05	15120649	998
M29837863	C03	29837863	2093
M46836480	B05	46836480	1086
M21846102	B02	21846102	297
M11820247	C01	11820247	1862
M17777211	B08	17777211	1691
M28496471	B02	28496471	313
M24173977	B08	24173977	1706
M26882315	B03	26882315	707
M53716239	C04	53716239	2259
M12169025	B04	12169025	842
M2622402	B06	2622402	1125
M20942811	B05	20942811	1025
M27082091	B02	27082091	302
M50169725	C04	50169725	2251
M31852562	B01	31852562	128
M13411712	B05	13411712	975
M4094362	B06	4094362	1146
M54308102	C04	54308102	2262
M46474863	C07	46474863	2572
M18510619	C03	18510619	2072
M8529616	B01	8529616	40
M10510241	C01	10510241	1858
M28886251	C07	28886251	2502
M35385013	C07	35385013	2518
M6645316	C02	6645316	1944
M10381975	B05	10381975	948
M1749924	B01	1749924	11
M12237331	B05	12237331	954
M28416991	B02	28416991	311
M28195235	B06	28195235	1199
M7633722	C04	7633722	2206
M47261151	C07	47261151	2579
M11565089	C03	11565089	2043
M4644621	B02	4644621	278
M23888823	B03	23888823	687
M29794992	B01	29794992	113
M33433854	C04	33433854	2227
M1619965	C09	1619965	2725
M34160819	B01	34160819	171
M52508991	B08	52508991	1804
M23531767	C08	23531767	2619
M61916741	C03	61916741	2161
M20850512	B04	20850512	877

M10648112	B03	10648112	610
M40983114	C07	40983114	2541
M36302643	C07	36302643	2523
M6040698	C03	6040698	2034
M20309392	B06	20309392	1185
M44518509	B05	44518509	1075
M6469793	B05	6469793	933
M44670769	C07	44670769	2563
M12121671	B03	12121671	622
M29177800	C06	29177800	2426
M4707263	C04	4707263	2192
M4167994	B03	4167994	581
M4592424	B08	4592424	1590
M51072697	B08	51072697	1780
M53930782	C09	53930782	2830
M28624569	B01	28624569	100
M43816934	C07	43816934	2555
M6052920	C01	6052920	1835
M5823256	C03	5823256	2033
M8903629	B03	8903629	603
M25265596	C06	25265596	2416
M47973112	B05	47973112	1090
M2328744	C05	2328744	2274
M15845930	B04	15845930	858
M46542013	B05	46542013	1082
M7595251	C06	7595251	2372
M19148512	B03	19148512	657
M49379712	B08	49379712	1767
M55204156	B02	55204156	523
M18452353	B08	18452353	1697
M24609957	B03	24609957	691
M28416475	C02	28416475	1983
M1553784	B05	1553784	910
M3006080	B06	3006080	1130
M9723909	C03	9723909	2038
M12678735	B05	12678735	959
M22226670	B05	22226670	1027
M39512649	B01	39512649	241
M33124153	C04	33124153	2224
M2961430	B06	2961430	1128
M53168240	B02	53168240	503
M49882780	C02	49882780	2016
M54507298	C04	54507298	2264
M15348284	B05	15348284	1001
M47540167	B02	47540167	450
M28665339	B01	28665339	101
M13606305	B08	13606305	1660

M114840	C02	114840	1912
M7990962	C08	7990962	2598
M36439744	B02	36439744	348
M53386833	B02	53386833	505
M36906340	B01	36906340	218
M3834407	B01	3834407	22
M32973415	B02	32973415	325
M598344	B01	598344	4
M13515189	C06	13515189	2390
M229684	C09	229684	2721
M52864159	C04	52864159	2258
M25927955	B03	25927955	699
M1020283	B02	1020283	252
M28843351	B01	28843351	106
M10251597	B03	10251597	607
M1836519	B03	1836519	562
M39996778	B02	39996778	385
M12611126	C07	12611126	2475
M49586823	B08	49586823	1769
M7170105	C04	7170105	2203
M41988069	C07	41988069	2548
M51040974	B05	51040974	1106
M3023041	B06	3023041	1131
M27592372	C08	27592372	2635
M5202807	B04	5202807	781
M13266608	B03	13266608	632
M53725063	C04	53725063	2260
M51716949	B02	51716949	489
M28535035	B01	28535035	99
M44181370	C07	44181370	2558
M51784255	B02	51784255	491
M16528911	C01	16528911	1876
M51218270	B08	51218270	1782
M31442978	B01	31442978	123
M50151651	C09	50151651	2817
M3765639	C01	3765639	1826
M15373933	B04	15373933	851
M2288905	B06	2288905	1121
M39841803	C08	39841803	2702
M30525869	C07	30525869	2503
M4417336	B02	4417336	275
M5097208	B04	5097208	779
M20078425	B02	20078425	295
M53074118	B02	53074118	502
M26982614	C08	26982614	2632
M1198158	B05	1198158	904
M24628537	B03	24628537	692

M7299816	C02	7299816	1951
M39599215	C07	39599215	2535
M23760049	B03	23760049	684
M5363361	B04	5363361	784
M18486582	C03	18486582	2071
M5448049	B02	5448049	279
M13487962	B05	13487962	977
M47724932	C07	47724932	2584
M11439685	B08	11439685	1637
M27375845	C03	27375845	2089
M47552968	B05	47552968	1087
M1971930	B06	1971930	1117
M20380180	B04	20380180	873
M13657337	B03	13657337	634
M6182714	B08	6182714	1612
M3111599	B01	3111599	17
M24913313	C08	24913313	2624
M8707231	B05	8707231	942
M544310	B01	544310	3
M15558557	B05	15558557	1005
M40261735	B05	40261735	1045
M41100957	C07	41100957	2544
M65028293	C03	65028293	2175
M13255932	B03	13255932	631
M35552211	C07	35552211	2519
M19518688	B03	19518688	664
M4003562	C01	4003562	1827
M44128345	B05	44128345	1073
M11452740	B08	11452740	1638
M11247488	C07	11247488	2469
M9792063	C01	9792063	1854
M4935843	C02	4935843	1936
M48927792	B02	48927792	463
M32489455	B05	32489455	1032
M27901946	B05	27901946	1030
M32164852	B01	32164852	133
M32456119	B01	32456119	140
M15658476	C01	15658476	1875
M18790977	C08	18790977	2607
M22686464	C07	22686464	2488
M47749648	B08	47749648	1759
M38190320	C07	38190320	2531
M15083773	B05	15083773	997
M29748288	C08	29748288	2642
M33214471	C08	33214471	2664
M39834660	C08	39834660	2701
M11245124	B04	11245124	829

M48088251	B08	48088251	1760
M12794154	B08	12794154	1651
M46792386	C04	46792386	2250
M16425040	B05	16425040	1014
M12590264	B08	12590264	1648
M22754049	C08	22754049	2614
M35577164	C07	35577164	2520
M11272740	B08	11272740	1636
M699050	B04	699050	736
M40385787	B04	40385787	895
M14339686	B08	14339686	1674
M30794771	B02	30794771	317
M24007948	B03	24007948	689
M8901729	C09	8901729	2748
M13431735	B03	13431735	633
M13744368	B05	13744368	986
M36353701	B01	36353701	206
M48871250	C07	48871250	2585
M14875578	C05	14875578	2322
M5868938	B04	5868938	789
M34643015	B05	34643015	1036
M46412883	B05	46412883	1080
M15588731	B08	15588731	1682
M49768089	C02	49768089	2014
M20355357	B04	20355357	872
M4271197	C01	4271197	1829
M201042	C01	201042	1813
M11693450	C01	11693450	1861
M48296979	B02	48296979	457
M7382523	B05	7382523	935
M4231861	B03	4231861	583
M51170258	B08	51170258	1781
M9708178	C03	9708178	2037
M3257862	C09	3257862	2730
M20761911	B03	20761911	668
M52305050	C04	52305050	2256
M38785266	C08	38785266	2697
M5568053	B02	5568053	281
M12763918	B05	12763918	962
M3484636	C04	3484636	2189
M6162569	B08	6162569	1611
M7391896	C04	7391896	2205
M39714030	C08	39714030	2700
M28618982	B06	28618982	1201
M35935009	B01	35935009	197
M2446514	C04	2446514	2184
M46716826	B08	46716826	1756

M22618837	B03	22618837	675
M27604193	C08	27604193	2636
M16900673	C03	16900673	2058
M37014404	C08	37014404	2681
M5198965	B05	5198965	927
M11825150	C01	11825150	1863
M5915468	B08	5915468	1609
M9862731	B05	9862731	947
M5547725	B02	5547725	280
M23907971	B03	23907971	688
M30029662	C08	30029662	2646
M5705717	C04	5705717	2196
M12866341	B05	12866341	964
M2069683	B06	2069683	1118
M15399228	B03	15399228	642
M17098083	B03	17098083	647
M13829993	B01	13829993	71
M19884086	B08	19884086	1701
M18316465	C03	18316465	2066
M1894712	B06	1894712	1115
M11085340	B05	11085340	951
M6039450	B05	6039450	931
M35591484	C08	35591484	2674
M37172907	C08	37172907	2685
M1894735	C03	1894735	2026
M30785329	C08	30785329	2650
M43928299	B05	43928299	1071
M2136271	B06	2136271	1119
M25747718	C06	25747718	2418
M7993400	B08	7993400	1625
M14453268	B05	14453268	994
M6572647	C04	6572647	2200
M47279700	C07	47279700	2580
M33838543	C06	33838543	2435
M19471682	B03	19471682	663
M54095653	C04	54095653	2261
M29755385	C08	29755385	2643
M2942723	B06	2942723	1127
M30014607	C08	30014607	2645
M53685259	C09	53685259	2829
M35552	C06	35552	2369
M49660171	B08	49660171	1771
M37967557	B05	37967557	1038
M12995754	B03	12995754	629
M48194370	B05	48194370	1093
M43857162	B05	43857162	1070
M2353087	B05	2353087	916

M42244923	C08	42244923	2709
M26807831	C02	26807831	1968
M6961017	B06	6961017	1169
M7693297	C04	7693297	2207
M4108839	B06	4108839	1147
M8772931	B06	8772931	1180
M8736510	B06	8736510	1179
M2913950	C03	2913950	2028
M12377827	B01	12377827	63
M33053990	B02	33053990	327
M34445482	B05	34445482	1035
M5259292	B01	5259292	29
M22237512	B06	22237512	1191
M8649899	B08	8649899	1627
M3730773	C09	3730773	2731
M45149114	C02	45149114	2008
M1835627	C03	1835627	2023
M4532942	B02	4532942	277
M15020795	B04	15020795	848
M54544680	B02	54544680	512
M31381657	B06	31381657	1217
M30666968	C04	30666968	2223
M45352110	C07	45352110	2567
M28976132	B01	28976132	109
M20812694	B03	20812694	669
M27892901	C06	27892901	2423
M7762771	C09	7762771	2743
M5778958	B05	5778958	929
M28242914	B02	28242914	308
M7262620	C02	7262620	1950
M14267047	C01	14267047	1869
M12309627	B05	12309627	955
M5603816	B02	5603816	282
M52024351	C02	52024351	2017
M15408010	B03	15408010	643
M7173589	B06	7173589	1172
M14564080	B06	14564080	1183
M14488471	B05	14488471	995
M11674080	B08	11674080	1642
M831087	B02	831087	250
M1592238	B03	1592238	557
M19120941	B03	19120941	656
M19370606	B03	19370606	661
M28016466	C07	28016466	2500
M18404358	C03	18404358	2069
M20846124	B03	20846124	670
M50480915	B08	50480915	1774

M45718833	B02	45718833	432
M52760767	B02	52760767	498
M44796	Scaffold0	44796	2834
M4078631	C09	4078631	2734
M5032999	B05	5032999	926
M1380812	B03	1380812	553
M3674663	C01	3674663	1825
M989961	C05	989961	2268
M15375529	B05	15375529	1002
M32792940	B01	32792940	150
M43635557	C01	43635557	1910
M29865250	C08	29865250	2644
M43417777	B05	43417777	1062
M12554433	C07	12554433	2474
M528569	C02	528569	1918
M44009981	B05	44009981	1072
M6047775	B06	6047775	1163
M9777067	C03	9777067	2039
M19225168	C08	19225168	2609
M46263814	C04	46263814	2242
M2999408	C04	2999408	2187
M5246929	B05	5246929	928

s of selected lines of YWDH population using the SVS package. Markers in bold are significa

**Markers Used**	**# Haploty**
**M9620644, M9626667, M9633923, M9635330, M9635946, M9663678, M96673**	2
**M4410527, M4417927**	3
**M3684925, M3688477, M3688602**	2
**M4425980, M4446769**	2
**M25650458, M25672161**	2
**M7738513, M7739795**	2
**M8034787, M8041457, M8157260**	2
**M7764181, M7851747**	2
**M5986754, M6040076, M6135212**	2
**M13208012, M13255297**	2
**M5375029, M5385024**	2
**M8334946, M8346459, M8348762**	2
**M4330165, M4333627, M4364280, M4375360, M4384358, M4400270**	2
**M10650500, M10652124**	2
**M32749694, M32887688**	2
**M6270507, M6274968**	2
**M34418225, M34428500**	2
**M13393388, M13405718**	2
**M4245547, M4249695**	2
**M3691385, M3703432**	2
**M5336932, M5344225, M5346221**	2
**M35894652, M35895908, M35919366, M35960799**	2
**M8960409, M9009012, M9046170, M9056660, M9076141**	4
**M6250296, M6255733**	2
**M24355519, M24402049**	2
**M9673714, M9828155**	2
**M4858426, M4872894, M4880373, M4881713**	2
**M14356051, M14401683, M14407236**	2
**M13753632, M13764641, M13773252**	2
**M38408159, M38437375, M38449589, M38475936, M38491516, M38496011**	2
**M14429375, M14429651**	2
**M11907439, M12056431**	2
**M5130, M37676, M47824**	2
**M8804511, M8817488, M8825429, M8831804, M8834714, M8865302, M88817**	2
**M36982693, M37032943**	2
**M6350914, M6353539, M6447544, M6453499, M6459432, M6475334**	2
**M22623449, M22632702**	2
**M4977141, M5098427**	2
**M5587919, M5593641, M5597100, M5599294, M5607279, M5629997, M56459**	4
**M14805048, M14806351**	2
**M27843689, M27899870**	3
**M42163883, M42189846**	2
**M13346230, M13361598**	2
**M4886169, M4888013, M4889900**	2

**M14543176, M14559957**	**2**
**M3648675, M3651510, M3661374**	**2**
**M2890321, M2892609, M2897369**	**2**
**M38866415, M38883275, M38894581**	**2**
**M33540194, M33563219, M33597959, M33619544, M33685264**	**2**
**M3896075, M3899258, M3907760, M3961259, M3990739**	**2**
**M3574719, M3579830, M3584128, M3604244, M3610790, M3615196, M36207**	**3**
**M32176325, M32292314**	**2**
**M22802362, M22836628, M22839381, M22855580, M22897089**	**2**
**M30889160, M31000819**	**2**
**M12449285, M12474743**	**2**
**M24416151, M24426748**	**2**
**M26796805, M26807908, M26817656, M26833712**	**2**
**M14499710, M14508930, M14511360**	**2**
M22013632, M22019498, M22028505	2
M12382897, M12387854, M12391072, M12397149, M12401457	3
M5816614, M5818319, M5869541, M5873056, M5896271, M5938587	2
M3209185, M3211415	2
M9090041, M9093663	2
M2613822, M2620423, M2625251	2
M27636453, M27675377, M27686794, M27696325, M27737413, M27741278, M	2
M22040928, M22048268	2
M3723297, M3726699	2
M37953127, M37963331, M37994032, M38036328, M38073768, M38103645, M	2
M26358342, M26367620, M26371853, M26384379	2
M22105894, M22134136	2
M33432684, M33505791	2
M34903569, M34944669, M34952462	2
M26107973, M26122853, M26157699, M26173949, M26187655, M26193813, M	2
M28688749, M28693540, M28699182, M28751672, M28845439	2
M49440378, M49479936	2
M32557616, M32602721	2
M13298784, M13319570	2
M26295602, M26323600	2
M34183723, M34298866	3
M41748469, M41765370, M41771558, M41809389, M41812521, M41821423	2
M25312738, M25426939	2
M24041170, M24128771	2
M11806929, M11809097	2
M25227598, M25239205	2
M25059617, M25063793, M25100098	2
M3732620, M3733606, M3735196, M3746223	3
M14158134, M14163354	2
M14121635, M14128042, M14129952	2
M13574887, M13574967	2
M36142212, M36176587	2
M38928725, M38942161	2

M27974412, M28064024, M28114210	3
M14165179, M14167588	2
M8079, M33894	2
M12410651, M12411928	2
M3217705, M3218205	3
M14270427, M14289565	3
M42328690, M42351367	2
M32820240, M32908133	2
M13460542, M13477779	2
M34906390, M34915341, M34927157	3
M37122804, M37141015, M37149131, M37251190, M37273288	2
M27798399, M27806664	2
M24606538, M24615253, M24743180, M24763505	2
M4524587, M4534748, M4558002, M4565029, M4573699, M4584605, M458873	4
M33783360, M33825318, M33935619	2
M31553797, M31561834	3
M33753316, M33778678	2
M13620201, M13635456, M13678201	2
M22985367, M22997056, M23134557	2
M2757087, M2767383	2
M34894969, M34899582, M34905698, M34923700, M34924710, M34929868	3
M36120054, M36124534	2
M42560894, M42562724, M42583170, M42626907, M42639995	2
M33287828, M33295187	2
M37093002, M37110840, M37115145, M37117977, M37127329	2
M26976672, M26999919, M27025466, M27025887, M27033912, M27036469, M	2
M19385032, M19397661	2
M35540079, M35560021, M35573639, M35576640	2
M42228584, M42230478, M42237548, M42242719, M42248982	2
M14565234, M14569860, M14605048, M14610245, M14620942, M14630339, M	2
M37315865, M37319621, M37322564, M37348456	2
M34368458, M34370845, M34376247	2
M42071854, M42095776, M42130323, M42149765	2
M25542671, M25564895	2
M2790013, M2793286, M2809385	2
M34952666, M34964161, M34977923, M34989952	2
M34064890, M34136017, M34211281, M34223395	2
M51410688, M51442646	3
M34180286, M34180945	2
M37200454, M37203866, M37216987	2
M36328214, M36375374	2
M44641713, M44670285, M44679019, M44685904, M44696424, M44702782, M	2
M38168330, M38320887	2
M13526624, M13533440, M13536932, M13552189	2
M34765721, M34769224	2
M18849, M45365	2
M10090715, M10096933, M10111765	2

M32997333, M33153329	3
M38609726, M38664784, M38685085, M38707046, M38750201	2
M3458408, M3464312, M3478653, M3521161, M3524033, M3536333, M354127	2
M4142720, M4165071, M4168981	3
M18572234, M18595514, M18602509	2
M34649849, M34754574, M34760882	2
M37312224, M37315617, M37320144, M37327266	2
M11933197, M11933958, M11959296, M12009600, M12032858, M12043151, M	2
M10199983, M10202247, M10216164, M10315737, M10346664	2
M25891999, M25907031, M25938889, M25982214, M25985993, M26001317, M	2
M22417108, M22422614	2
M34829560, M34838020	2
M26060839, M26091584, M26102527	2
M39050115, M39114685, M39156169	2
M25372764, M25438374, M25456641, M25526524	2
M33233243, M33257782	4
M25845436, M26004897	2
M34233026, M34253632, M34268602, M34293399, M34339424, M34380073, M	2
M39299217, M39302593, M39308177	2
M33918504, M33940590, M33947026	2
M35698467, M35806689, M35814451	2
M17621698, M17736955	2
M266832, M269361, M270765, M273211, M302688, M306257, M319093, M378	2
M26565778, M26724827	2
M35440174, M35443778	2
M2876182, M2882226	2
M13820139, M13844766	3
M31128252, M31246995, M31267384, M31269352	2
M44431720, M44434393	2
M36866355, M36877304	2
M31325852, M31343113	2
M36732364, M36735270	2
M36577292, M36606896, M36614197, M36627946, M36657162, M36690458, M	2
M52835523, M52847776	2
M14320800, M14325719	3
M37654974, M37682863, M37690121, M37703271, M37710315	2
M42204113, M42239793, M42248580, M42252962, M42262610, M42295647	2
M26792277, M26799840, M26813508, M26821497, M26846487, M26857856, M	3
M38780127, M38818094, M38828300	3
M14664349, M14676891, M14678291	2
M36767097, M36775283	2
M2283575, M2287097	2
M26472722, M26490411, M26551713	2
M2744186, M2747731	2
M2335626, M2337528, M2357295, M2390911, M2396759, M2398827, M240995	2
M29411190, M29422837, M29427064, M29482905	2
M3177797, M3186072	2

M14409976, M14413289, M14417128	2
M26628405, M26653240, M26682963, M26689194, M26694681, M26748836, M	2
M19627544, M19644420	2
M4190599, M4242261	2
M19939992, M19954637	2
M2885324, M2914691	2
M31896442, M32053808	3
M5419919, M5429834, M5432975, M5460458, M5489473, M5503966	3
M38469050, M38473304, M38475495	2
M14134089, M14136614	3
M41726779, M41730963, M41733144	2
M16511341, M16537145	3
M31351274, M31358635, M31388258, M31417079, M31471931, M31505537	2
M18865754, M18869261	2
M2548458, M2550034, M2553867, M2555181, M2558658, M2562607, M261656	2
M19965171, M19973111	2
M11607341, M11607718, M11614228, M11678738, M11679296, M11693008	2
M38477768, M38479773	2
M10902883, M10904507, M10907333, M10929033, M10931651, M10952963, M	2
M43370206, M43431034	2
M34999235, M35005985, M35037530	3
M36708156, M36714223	2
M1644118, M1651312	2
M37785238, M37794859, M37907192	2
M30361755, M30486924, M30510485, M30517906, M30520630	2
M12568910, M12570149, M12581586, M12585456, M12593782	2
M30525555, M30534860, M30546920	3
M36723263, M36727180	2
M36211916, M36219061, M36247671	3
M11718692, M11732392, M11746071, M11749470, M11764435	2
M37599152, M37608115, M37625244	2
M37062903, M37094082	3
M34941899, M34949217, M34962758	2
M16259212, M16380265, M16385485, M16387674, M16389355, M16393601, M	2
M11068381, M11070086, M11073709, M11086004, M11093266, M11095740, M	2
M7954677, M7960368, M7979125, M7983899, M7990215, M7991972, M799443	2
M5120118, M5150614, M5159029	3
M3105431, M3115534, M3134469, M3138560, M3155275, M3161270	2
M37906384, M37930199, M37943642	3
M25679789, M25695860, M25737621, M25786640, M25816855, M25836597, M	2
M23610656, M23613342, M23634892, M23636568, M23639395, M23671740, M	2
M11207412, M11265736, M11272585, M11273996, M11281634, M11286688, M	2
M5540515, M5546167, M5551543, M5562965, M5566004	2
M10352268, M10375422, M10380820, M10382457, M10384901, M10390648, M	2
M13782626, M13790772, M13814008	2
M30765475, M30803207	2
M31952043, M32025496, M32090504	2

M38189169, M38198414, M38204710	2
M10146008, M10163829, M10185164	2
M36810946, M36826988, M36857397, M36897122, M36950300	3
M13906702, M13918465, M13929715, M13943671	2
M31531980, M31611702	2
M31306537, M31318183	2
M14896442, M14903660, M14920779, M14949394, M14960720, M14970663, M	2
M2989939, M2992321, M2997727, M3006361, M3013409	2
M35846019, M35868600, M35886800	2
M4903573, M4907864, M4929349, M4930994, M4953550, M4955654, M497018	2
M19926846, M19928147, M19930835, M19934771	2
M2907300, M2912838, M2914255, M2921662, M2925419	4
M4105171, M4147134, M4150485, M4216042, M4219796, M4259970, M426159	3
M38039376, M38088723, M38098508, M38112164, M38118095, M38131451	2
M22447228, M22470923, M22495815	2
M14807931, M14808170	2
M35961, M41996, M50221, M77852	2
M3369219, M3373650, M3375712, M3380619	2
M2857721, M2875647	3
M35990485, M36042144	2
M32852834, M32854701, M32930222	2
M13366015, M13484746, M13514300	3
M9108511, M9113881, M9120526, M9129160, M9130337, M9249826, M925120	3
M37068092, M37082671	3
M31876668, M31881984, M31895774	2
M14190734, M14191683, M14195629	2
M42196431, M42201533	2
M20260786, M20274489	2
M2293789, M2452880	2
M37890194, M37895398, M37950852	2
M627148, M636755, M705838, M712580, M720197, M725211, M727739, M729	3
M11054517, M11148718, M11163256, M11211952	2
M11261642, M11272360, M11286808, M11354612, M11376804, M11379528, M	2
M35058860, M35149435, M35180085, M35202629, M35210521	2
M24807426, M24811307, M24818501, M24821260, M24828107, M24835388, M	2
M33883467, M33896929, M33905590	2
M38864615, M38947048, M38998564	2
M35246843, M35364147	2
M23877164, M24013512	2
M17779767, M17793047, M17812561	2
M41300441, M41314853, M41329037	2
M36091214, M36136513	3
M38127795, M38133512, M38138298, M38158059, M38165622, M38173752, M	2
M40919219, M41072488	2
M25109886, M25111463, M25134447, M25140154, M25148185	2
M36454998, M36492008	2
M41309859, M41333682	2

M4260462, M4263463, M4268250, M4279463, M4306582, M4314335	2
M13883806, M13890042, M13902514	3
M46175991, M46189204, M46229972	2
M16451555, M16455135	2
M3544729, M3555072, M3563846	2
M31398002, M31553912	2
M1389679, M1394306, M1395576	2
M2456105, M2458716, M2461857, M2503950, M2522992, M2533499, M255150	2
M35589495, M35617768, M35637723	2
M9717940, M9739586, M9741749, M9872864	4
M16940709, M16944228, M16952653	2
M27385995, M27451427, M27462373, M27489977, M27508802, M27521547, M	2
M30022816, M30043328, M30092920, M30108594, M30126354, M30152701, M	3
M686267, M743751, M746359, M750732, M752274	2
M18878013, M18884442, M18889349, M18939019	2
M7867523, M7871367	2
M44518614, M44529589, M44614341, M44635451	2
M51303369, M51409503	2
M45430140, M45455240, M45458295, M45496339, M45501872, M45555607, M	2
M8164815, M8179461, M8188555, M8192340, M8310416	2
M36836991, M36862474	3
M3858613, M3875285, M3876847	2
M14043418, M14055815, M14064382, M14084392	2
M44562195, M44563890	2
M22684390, M22699159, M22721772, M22755774	2
M18546475, M18631384	3
M2568196, M2573551	2
M37398518, M37479448, M37521382, M37525518, M37534404, M37538649	2
M20358961, M20372445	2
M45870319, M45877414, M45878447, M45880013	2
M37238523, M37277352	2
M41088028, M41089476	2
M7173948, M7182157, M7193048, M7198114, M7201411	2
M8009782, M8026683, M8034193, M8044689, M8074669, M8077102, M808092	2
M41667606, M41691242, M41697760	2
M35643371, M35658507, M35667701	2
M161315, M166812	2
M36439544, M36456649, M36485875, M36521972, M36533475, M36546681, M	2
M19774114, M19788574	2
M45389509, M45414892	3
M37682553, M37696296	2
M42290787, M42296460, M42302123, M42311775, M42316330, M42321784, M	2
M20304505, M20307827, M20313642, M20339163	2
M34852143, M34854826, M34857991, M34862702	2
M9641655, M9643643, M9667444	2
M38002945, M38033185	3
M19742019, M19754477	2

M32338839, M32366187	2
M30969826, M31079368, M31114378	3
M11989714, M12044289	2
M38985286, M38989918, M38996533, M39002033, M39010106	2
M1801063, M1809516, M1821331, M1828987, M1837449, M1840421, M193696	2
M44390803, M44393373, M44395213, M44397744, M44401517, M44406389, M	2
M30840862, M30914670	2
M42370558, M42514255	2
M36694645, M36707152	2
M14302651, M14306991, M14375434	4
M25865828, M25871615	3
M14434855, M14438226, M14439677, M14443910, M14445209	2
M24434503, M24452406, M24466185, M24507778, M24545305, M24563473, M	2
M34038804, M34105710, M34114146, M34158719	2
M31653502, M31749911	2
M38548053, M38559460, M38562787, M38564406	3
M18817226, M18819418, M18853064, M18857516	2
M1635144, M1696137	2
M26136857, M26148479, M26154076, M26164278, M26167382, M26190938, M	2
M42281520, M42353469	2
M9368498, M9369846, M9372567	2
M29054822, M29183587, M29201718	2
M41903911, M42012109	2
M6958133, M6965407, M6968929, M6973197, M6975276, M6995896, M702202	2
M36771744, M36773591	2
M12684597, M12703771, M12721439, M12732174, M12734830, M12735548, M	2
M19445981, M19453879	2
M13328693, M13481574	2
M921648, M924431, M927151	2
M36262742, M36409682, M36421902	2
M2723479, M2739573, M2747406, M2753326, M2761697, M2770604, M284474	3
M34524429, M34537350, M34544468, M34555852, M34568632	2
M31486439, M31500174, M31519007, M31520489, M31554844, M31636627	3
M739480, M747281	2
M10048869, M10051770	2
M16691656, M16715708, M16720709, M16730973, M16738820	2
M35713245, M35789815, M35832364	2
M13031360, M13032117, M13117339, M13173392, M13188246	2
M36899155, M36914219, M36917602	2
M5874541, M5886184	2
M42763252, M42766782	2
M16802902, M16811169, M16826551, M16830871	2
M1114958, M1121983, M1142364, M1145860, M1151635	2
M42756895, M42760098, M42763264, M42859005, M42871778, M42875317, M	2
M45529006, M45530334, M45531205, M45534359	2
M29746104, M29753345, M29764529, M29771340	2
M39527058, M39529304, M39666424, M39670996, M39674264, M39676512, M	2

M2017947, M2020011, M2024313	2
M8210004, M8215549, M8233602	2
M20899047, M20924948, M20933863, M20949250, M20958522, M20965658, M	2
M44383347, M44388605	2
M3383200, M3466724, M3469548	2
M8098716, M8113219, M8118961	2
M38281726, M38286730, M38418771, M38424772	3
M39132880, M39190939	2
M36200118, M36216495, M36226598, M36234289, M36236646, M36237269, M	2
M28136916, M28158865, M28180520	3
M34206973, M34298619	2
M32827754, M32842826, M32845663	2
M37966600, M38084492, M38125095	2
M44590274, M44602967	2
M38145678, M38157108, M38294450, M38303244	2
M32759252, M32769125, M32773962	2
M23085300, M23119316, M23160665	2
M10774068, M10806829, M10810228, M10813123, M10818376, M10824568, M	2
M13284193, M13291912	2
M19977205, M19982234, M20001204, M20013599	2
M18827812, M18846519, M18855313, M18868577, M18871476	2
M38527738, M38533036	2
M3041391, M3057173, M3061861, M3066981, M3070284, M3073536, M309569	2
M36816764, M36891832	2
M6160904, M6168049, M6182213, M6189644, M6195646, M6199437, M620964	2
M44365369, M44368138, M44375915	2
M9726531, M9796326	2
M36566004, M36664074	3
M1939371, M1941023, M1946165, M1979050, M1980563, M1984342, M199995	2
M5131694, M5195200, M5196644, M5198947, M5202332, M5203471, M520692	3
M44485571, M44492330, M44495276, M44501278	3
M16876109, M16885181	2
M347358, M405104	2
M41803232, M41815449, M41837530	2
M13275269, M13279502, M13280473	2
M944660, M951722	2
M47994969, M48000614, M48005776	2
M43841880, M43910665	2
M43588846, M43591184, M43607298	2
M16945549, M16946771	2
M3536914, M3537887, M3545960	2
M40568262, M40590666, M40609239, M40622579	2
M34437288, M34444231, M34460588, M34484520, M34511036	2
M19814659, M19834081, M19888585, M19900244	2
M41702919, M41714464, M41718663	2
M13897578, M13899583, M13911630	2
M30128893, M30141654, M30144343, M30163370	2

M1019711, M1028832, M1034678, M1037487, M1072714, M1072792, M107748	2
M1203940, M1211937, M1214690, M1218137	2
M26684089, M26688576, M26704956, M26708644, M26713889, M26720308, M	2
M34378216, M34468827	2
M44175233, M44179012, M44253886, M44271625, M44273713	2
M40216343, M40218054, M40221563, M40240114, M40240167, M40249003, M	2
M12415594, M12421038, M12454133	2
M8881536, M8895163, M8904038	2
M14470374, M14471853, M14485422, M14497712, M14505429, M14512114, M	4
M39663398, M39664140, M39666764	2
M20596423, M20603703, M20606281	2
M28889443, M28930917, M28971966	2
M33284870, M33306905, M33322634, M33326572	2
M14096078, M14103469, M14115264	2
M15035641, M15057588, M15170958, M15174683, M15177279	2
M31190062, M31268376	2
M19578914, M19592389	2
M38571948, M38689011, M38691130	3
M37668364, M37678463	2
M59029999, M59033335, M59035299	2
M30471682, M30482873, M30503603, M30518172	3
M1816697, M1818677	2
M24107941, M24114392, M24140989, M24143898, M24147902	3
M36314323, M36471047	3
M64652670, M64657657, M64659550, M64700897, M64709670	2
M114290, M258208, M259553, M262234	2
M39824438, M39825612, M39839660, M39848212, M39963039, M39963889, M	2
M31340137, M31494987	2
M12845988, M12854502	2
M12663550, M12725326, M12754185, M12819340	2
M776657, M777497, M796106, M823752, M826702, M834153, M835499, M839	2
M1251793, M1254440, M1282974, M1308868, M1309924, M1313050, M139324	2
M40978865, M40989113, M41000245	2
M49311335, M49350631, M49351963, M49367382, M49369600	2
M37264761, M37269018	2
M49600796, M49603353, M49614159, M49623742, M49631055	2
M42263076, M42265855, M42273508, M42284189	2
M36996898, M37003156, M37005771, M37013740, M37019838, M37032296, M	3
M10212939, M10237383, M10243883	2
M34985082, M34987214, M35000531, M35009979, M35018487	2
M2724435, M2726823, M2735091, M2761466	2
M41668019, M41753581, M41758434, M41773472	2
M37431703, M37437010, M37485572, M37540680, M37560165, M37586614	2
M32377523, M32526585	3
M38460057, M38464247, M38464861, M38466196	2
M34393970, M34408969, M34411588	2
M668992, M684011	2

M13212798, M13214457	2
M4908887, M4919017	2
M41009586, M41073244	2
M39033319, M39041607, M39049713, M39056057, M39069888, M39139656, M	2
M2577402, M2579953, M2587660	2
M32633487, M32638341, M32640025, M32665160, M32671938, M32695415, M	2
M27555639, M27559123	2
M19460986, M19498943	2
M19936625, M19959367, M19995937	2
M43450360, M43454348	2
M34305703, M34313245, M34343528	2
M31322840, M31331732	2
M44225815, M44295242	3
M32819991, M32854022	3
M35921578, M35967769	3
M39688822, M39693572, M39698297	3
M24997591, M25021355	2
M48335613, M48341193, M48344371, M48359945, M48363568, M48378806, M	2
M11433216, M11443348, M11445545, M11451097, M11462016, M11473475, M	2
M23624138, M23650051, M23662187	2
M35950495, M35956459	3
M41334003, M41336814	2
M2115952, M2121797	2
M1848505, M1851174	2
M22153301, M22156087, M22174119, M22189848, M22208256, M22217940, M	2
M14124831, M14166073, M14221657, M14271332	2
M39403858, M39410037, M39424942, M39439514, M39444416, M39451079, M	2
M24666217, M24733115, M24743263	3
M6469309, M6474707, M6476783, M6479575, M6483292, M6486013, M649478	2
M42327802, M42334632, M42339346, M42341832, M42343982, M42345981	3
M2710059, M2717393	2
M13068207, M13070310, M13077298, M13093852, M13097781, M13103152, M	2
M44509525, M44534383	3
M8384495, M8400568	2
M14229676, M14254069, M14275175, M14277189, M14278273, M14283446	3
M45735401, M45788455	2
M5912668, M5916981, M5956808, M5959948, M5983887, M5989966, M600212	2
M1363606, M1430622	3
M41135893, M41158268, M41167431, M41262102, M41288139, M41292616	2
M12813465, M12824260, M12847388, M12866438, M12897811, M12915463, M	2
M6558570, M6561845, M6567527	2
M19906491, M19969677, M19986879, M19993268, M20011242, M20041396	2
M27047106, M27055199, M27060505	2
M3684033, M3704208, M3708949, M3710666, M3730304, M3739890	2
M34330855, M34351575, M34381318, M34450556, M34478829	2
M38730205, M38738548, M38741639, M38745459, M38773416, M38777591	2
M13982243, M13991613, M14002193, M14018855	2

M31380347, M31414319, M31420530	2
M30557343, M30557989, M30591759, M30626135, M30650941, M30672411, M	2
M5597822, M5604678, M5649715, M5651441, M5677643, M5679138, M568414	2
M33716294, M33726559	2
M5840952, M5844680, M5848600	2
M3871630, M3875921, M3878721, M3937587	2
M4665827, M4669761, M4681747, M4687666, M4735424	2
M6503076, M6506295, M6512553	3
M38220204, M38237601, M38253879, M38260848, M38266585, M38281822, M	2
M14122422, M14128841	2
M13643658, M13651845, M13654356	2
M12563272, M12571764	2
M46370571, M46434469	4
M58596937, M58604312	2
M21701497, M21708783, M21720268, M21775779	2
M3004299, M3013819	2
M17619688, M17720902, M17748998, M17752431, M17773728	3
M22473789, M22588659	2
M44183683, M44184961	3
M27832215, M27854645	2
M49802171, M49818533, M49857591	2
M42409805, M42413262	2
M5375218, M5433593	2
M5718731, M5727724, M5806605	2
M47376530, M47377865, M47378632, M47383655, M47387575	2
M6428008, M6428717, M6433911, M6436936	2
M3045455, M3046901, M3051568, M3059989, M3061644, M3063484, M306878	2
M50887956, M50962442, M50967375, M50973170, M50982125	2
M35179888, M35192286, M35196497, M35197395	2
M36756334, M36760566, M36860251, M36860561, M36865721, M36876367	3
M39833731, M39853776	3
M42788810, M42847247, M42850005, M42883742, M42905185, M42913222, M	2
M33344849, M33348787, M33497461, M33500510, M33503982	2
M19114301, M19221569	2
M8891443, M8893533, M8897899, M8910663, M8920372, M8928364, M896288	2
M5005932, M5018558	3
M30498081, M30526890, M30640294, M30650147	3
M30274920, M30296135, M30300028, M30324106	2
M26504813, M26506034, M26507040, M26513163	2
M692437, M717362	2
M39734874, M39764237, M39783251, M39785092	2
M28372098, M28464265, M28489658, M28502515, M28510205, M28517059	3
M44187489, M44193632	2
M42518037, M42521032, M42522350, M42523614, M42527085, M42530384, M	2
M916338, M919744, M1014332, M1019205	2
M32729927, M32744912	2
M6685399, M6697846, M6720498, M6738147	2

M16922455, M17036366	3
M44483358, M44491156	2
M15719744, M15721217, M15728071, M15739885, M15773747	3
M11964467, M11970533	2
M28143185, M28194102, M28221795, M28226017, M28264722	2
M17632, M27044	2
M42592028, M42681110	2
M29751536, M29791338, M29829989, M29904301	2
M36674201, M36674530, M36730256, M36732531, M36734693, M36738371, M	3
M44462369, M44473680, M44474687	2
M16486082, M16606423, M16638130, M16644065	2
M5559182, M5564155, M5572202, M5623185, M5637259, M5640150, M565098	2
M29629136, M29662615, M29771720	2
M16925628, M16928876	2
M39902789, M39908231	2
M3931607, M3934894, M3957310	2
M1005400, M1024278, M1163858	2
M42165677, M42177752	2
M11605683, M11621004, M11621320	2
M45122568, M45124011	2
M725722, M730257, M735824, M790443, M802040, M804918	3
M23364813, M23395155, M23418558, M23474307, M23524495	2
M33362389, M33365029, M33509463	2
M36084763, M36087628, M36091190, M36095161, M36118845	2
M2508295, M2523382, M2530207, M2530811, M2543533, M2545017	2
M49698927, M49714490, M49719710, M49728644, M49735044, M49747575, M	2
M34789671, M34799822	2
M35914823, M35916793, M35920353, M35925654, M35944940, M35953957, M	3
M10663351, M10670924, M10691438	2
M39162344, M39178737, M39192548, M39199935, M39206241, M39224208	2
M45063174, M45065308, M45084609, M45092878, M45098597	2
M28306086, M28334713, M28372568, M28392532, M28397811, M28407265, M	2
M12220548, M12293701, M12300081, M12312681, M12327843, M12357349, M	2
M1407738, M1408651, M1410725, M1412282, M1425285, M1429893	2
M4271015, M4316874, M4317053, M4319044, M4323541, M4324906, M432876	3
M17757085, M17817144	2
M2710741, M2713712, M2713761	2
M4288178, M4313983, M4321760, M4336283, M4389218	4
M36943147, M36946976	2
M52788630, M52803077	2
M9144375, M9163204	2
M34650124, M34653410	2
M41318214, M41327525, M41333565, M41345662, M41351168	2
M3672616, M3673162, M3786612, M3797581	3
M2056233, M2065012, M2074284	2
M39977474, M39979547	2
M3799240, M3801394, M3804821, M3818507, M3820437, M3822134, M382424	3

M14398437, M14407825	2
M40414301, M40417333, M40426310, M40462621, M40467952, M40474270, M	2
M8075709, M8076970, M8079391	2
M7188605, M7191186	2
M46939688, M46952463	3
M3006484, M3026718	2
M46328735, M46329673, M46367246, M46376927, M46386003, M46389911	2
M14655330, M14759624	3
M37732265, M37736721	3
M27921218, M27936807	3
M1830670, M1834978	2
M2575475, M2579010, M2584267	2
M9499999, M9504288	2
M17181541, M17193898	2
M9702873, M9712818, M9764295	2
M3861591, M3878700, M3950115, M4005960, M4012616, M4020736	2
M1138219, M1167673, M1173459, M1177154	2
M48428052, M48440915	2
M13028244, M13057010, M13070075	3
M45644301, M45658292	2
M13922652, M13928688, M13939315, M13948702	2
M38443286, M38459572, M38519873, M38521939, M38525781, M38529375, M	2
M44954195, M45100035, M45106989	2
M147108, M157034, M178537, M194551, M199254, M201196, M204624, M206	3
M18471696, M18473521	2
M32891100, M32896158, M32899663	3
M27223971, M27224045	2
M1645525, M1651064, M1652086	2
M38791649, M38798304, M38810535, M38819693, M38833822, M38949108	2
M33750189, M33768940, M33798308, M33803256, M33821905	2
M50696092, M50697404, M50701351, M50703582, M50709240, M50711503, M	2
M48623257, M48627630, M48630596, M48644326	3
M52476977, M52490959	2
M572986, M574059, M575190, M578392	2
M37280043, M37286296, M37295822, M37298641, M37300155, M37300407	2
M41007274, M41029664	2
M10456762, M10458261, M10467973, M10568831, M10574212, M10580897, M	2
M1236043, M1273808	2
M28465, M32839	2
M24749739, M24757854, M24909650	2
M43317599, M43320291	2
M43050169, M43060252	2
M5569445, M5570907	2
M9123447, M9167831, M9231551, M9235970, M9238317, M9240710, M924783	2
M36765989, M36769420	2
M32419901, M32425520, M32438047, M32454659, M32457311, M32471078, M	2
M45887030, M45899613, M45911931, M45918856	2

M28928191, M28950071, M28950622, M28971562	2
M13975759, M13980405, M14013593, M14016083, M14021237, M14023139, M	2
M9835186, M9847165, M9869086, M9873951, M9874908, M9878563, M989178	2
M7394457, M7433595	2
M58016539, M58032384	2
M31858864, M31865157, M31867383	2
M29463684, M29483736	2
M32097985, M32119641	2
M329155, M332544	2
M26803805, M26839188, M26843537, M26927517	2
M5550926, M5554135, M5554616, M5566549	2
M30083891, M30087480, M30096388, M30107350	2
M7035319, M7055336, M7151210	2
M37915394, M37921048, M37925494	2
M57154208, M57171135	2
M34668941, M34679523	2
M2730897, M2806845, M2814995, M2821280	2
M13485527, M13487427, M13492807	2
M608375, M630579, M638062, M648755, M674488, M681257, M692904	2
M51340881, M51343422	2
M13089792, M13160829, M13201217, M13240244	2
M3088282, M3091862, M3094442, M3106691, M3110569, M3112992, M311719	3
M62619147, M62621888, M62633352, M62697802, M62702851	2
M41096283, M41101743, M41119193, M41126812	2
M5193758, M5197532, M5200814, M5203399, M5217883, M5226735, M528679	2
M41421187, M41432548, M41472391, M41491320, M41522018	2
M29100151, M29150364, M29151613, M29191630	2
M5000130, M5002670	2
M30529558, M30536994	2
M55130161, M55134924, M55136101, M55142771, M55145248, M55151665, M	2
M12427576, M12470280	2
M46422912, M46425734, M46428275, M46437067, M46450075, M46459441, M	2
M33129820, M33273702	2
M37332091, M37336598, M37340639	2
M44502782, M44504372, M44507049, M44510024	2
M39269299, M39375377	2
M30718368, M30718912, M30722457, M30725132, M30730199	2
M12276175, M12292214, M12307337, M12311773	2
M27214776, M27218338	2
M1289000, M1294935	2
M34648028, M34696604, M34721163, M34760652, M34787570, M34797004, M	2
M2930531, M2959363, M2964893	2
M1842764, M1875789	2
M26226600, M26235572	2
M39990837, M39993766	2
M39987474, M39992223, M39996607, M40117448, M40124528, M40129367, M	3
M5170208, M5181326	2

M15276951, M15285738, M15291628, M15315766	2
M21623043, M21642479, M21645217	2
M26938470, M26939609, M26947542, M26953662	2
M36650397, M36679704	2
M40629327, M40739879	2
M10018966, M10023001, M10028538, M10038670, M10042046, M10044497, M	2
M9459749, M9462015, M9475421, M9479072	2
M10716555, M10845803	2
M12554954, M12602111, M12611382, M12636756, M12644588, M12648974	2
M32876052, M32883515, M32884789	2
M5019973, M5024297, M5033676, M5034950	2
M35188306, M35281025, M35285905, M35295978, M35308011, M35314466, M	3
M42923011, M42930483, M42936421	2
M37280922, M37440867	2
M38457015, M38459007	2
M47144474, M47145648	2
M6266702, M6270631	2
M29040545, M29051113, M29056849	2
M16838562, M16856364, M16859622, M16868670	2
M18706078, M18723542, M18757283, M18766500, M18777182	3
M42855526, M42860574	2
M30952306, M30970374, M30987483, M30998770	2
M35119643, M35141915	3
M49432670, M49433150	2
M24014353, M24018098	2
M17010722, M17018645, M17021571, M17071437, M17074580, M17076386, M	2
M5313559, M5318684	2
M32592031, M32595414	3
M33875912, M34032714	2
M38133095, M38135989, M38138065, M38140974, M38164363	2
M39868193, M39967218, M39978036	2
M12091578, M12111833, M12135970, M12173588, M12182878, M12204443, M	2
M27491666, M27576406, M27588098, M27592246, M27599363, M27603238, M	2
M33218797, M33292048, M33369727, M33375022, M33378654	3
M10833730, M10836371, M10909797, M10915625, M10925614, M10942343, M	2
M63523386, M63532363	2
M27421040, M27464119, M27476862, M27494614, M27510148, M27522473	3
M35726499, M35732445, M35737902	2
M13133454, M13135922	2
M16566661, M16600932, M16632877, M16633083, M16645210, M16674335	2
M40768151, M40775869, M40799250	2
M9680951, M9684503	2
M44523845, M44550072, M44679412	2
M3013972, M3017930, M3021281, M3042242	2
M6244476, M6254648, M6259234, M6267711, M6277698, M6278688, M628343	2
M38439147, M38451743	2
M5773327, M5782051, M5782938, M5785301	2

M21994805, M22107980	3
M37714850, M37716741, M37721143, M37738747, M37748143, M37757823, M	2
M4085530, M4091616	3
M26901542, M26953778	3
M4732823, M4740531, M4744061, M4747976, M4836842, M4839796	2
M34638796, M34646498	2
M2352984, M2403157, M2408058, M2410241	2
M56336123, M56338248, M56347910, M56362382, M56388290, M56482390, M	2
M3474161, M3476840	2
M42941134, M42942810	2
M183082, M341431	2
M26664675, M26679087	2
M5971766, M5986282, M5987412, M5988480, M5998778, M6077510, M60828O	2
M10469789, M10484375	2
M30594057, M30604697	2
M3870713, M3876079, M3877583, M3881911, M3887367	2
M31134482, M31222152, M31224400, M31236583, M31237035, M31260104, M	2
M7239842, M7249192	2
M30345015, M30374506, M30382054, M30401674, M30422755, M30450037	3
M9160315, M9319628	3
M2005534, M2009672, M2014310, M2039054, M2040864, M2044994, M204965	2
M33771700, M33772253	2
M1910386, M1929385, M1931591	2
M10535708, M10541994, M10564410, M10566139	2
M19273996, M19363384	2
M40391218, M40396037	2
M43068262, M43103961	2
M12488519, M12492401, M12501087, M12505936, M12522458, M12560324	2
M26520731, M26674225, M26677224	2
M39423587, M39454581	2
M34185584, M34311802, M34321346	3
M19174831, M19185281, M19206884, M19209567, M19209938, M19211422, M	2
M13476963, M13477664, M13479887, M13481994	2
M50374482, M50375604, M50382087	2
M12345264, M12348503, M12360747, M12369147	2
M1349402, M1352422	2
M43622081, M43625499, M43642181, M43646589, M43647916, M43652252	2
M52235987, M52246969, M52258988, M52261160, M52267880, M52272568, M	2
M41816686, M41882627	2
M32722142, M32723994	2
M9375856, M9455817	2
M19535283, M19545848	3
M20698766, M20851240	2
M49734576, M49741538	2
M19300220, M19324394, M19328780, M19364537, M19374504	2
M14954069, M14964843, M14976776, M14981638, M14983974, M14986855, M	2
M9906000, M9917766, M9931103, M9953755, M9956248	2

M39639263, M39641354	2
M1936466, M1942220, M1950329, M1959472, M1962320, M1979988, M198410	2
M2597871, M2613019, M2757734	3
M32333672, M32375537, M32387409, M32392498	2
M5364452, M5368716, M5370151	2
M3408180, M3411267, M3411630	2
M2916777, M2920779	2
M887289, M896074	2
M11764219, M11766013, M11768608	2
M11584824, M11596720, M11629259	2
M29785501, M29931395	3
M10491585, M10503993, M10547117, M10560118, M10565128, M10568778, M	2
M28756145, M28760416	2
M4363749, M4372466, M4374944, M4380197, M4389654, M4394737	2
M27952085, M27955992, M27970076	2
M36588046, M36723398	2
M46755617, M46769437	2
M51850972, M51864117, M51867719, M51878679, M51885526, M51888128	2
M18605546, M18614673, M18622731, M18625582, M18628978, M18646207, M	2
M35642001, M35654448, M35667316, M35671897, M35706916, M35715130, M	2
M4397017, M4411898, M4414063, M4429877, M4441413, M4445549, M445179	2
M2842230, M2845816	3
M59222720, M59231084	2
M7947, M12456	2
M5194544, M5338405	2
M64159876, M64162802	3
M31519247, M31529606, M31588600, M31610005	4
M32609950, M32715855	2
M32331479, M32334537	2
M36629720, M36783388, M36785707	2
M94994, M97645	3
M31722006, M31870570	2
M1410661, M1414830	2
M7983538, M7992286, M8000230, M8010724	2
M29654346, M29656888, M29756374	2
M3580046, M3582096, M3584772, M3591032, M3599583, M3627146, M371875	2
M3209003, M3210752, M3211687	2
M7707902, M7731925, M7772154, M7783900, M7789960	2
M12824629, M12833802, M12842194, M12847292	3
M13011147, M13013437, M13014788, M13166162	2
M32440996, M32444047, M32447202	3
M27695402, M27796295, M27848624	2
M10885507, M10886506	2
M12675498, M12722132, M12750623, M12760740, M12760907, M12783455, M	2
M16762993, M16800628	3
M463601, M466297, M478308, M481752, M486868, M493023, M497735, M501	2
M42855735, M42861157, M42866444, M42870370	2

M908, M49844, M85891, M98069, M103379	2
M5582555, M5586284, M5593623, M5613584	4
M9319555, M9328291, M9332014, M9335540, M9339695	2
M47491450, M47494682	2
M847953, M853513	2
M10031972, M10035646, M10036188	3
M13316232, M13332364	2
M7929010, M7955410, M7999719	2
M46898382, M46902640	2
M33731302, M33744518	2
M15595140, M15600258, M15606236	2
M37381335, M37393009, M37419305, M37422898, M37427006, M37431857, M	2
M5285231, M5290135	2
M45425034, M45496670, M45498327, M45501864, M45502916, M45511729	2
M50642337, M50645477	2
M50926653, M50937133	2
M9315727, M9329347, M9332063, M9348792, M9364263, M9367966, M937853	2
M35652665, M35740150	2
M6097734, M6098685, M6102296, M6105092	2
M10305304, M10315030, M10326534, M10357044, M10382785, M10388452, M	2
M44288781, M44290367	2
M46158218, M46250349, M46279629, M46281125, M46284871, M46288027, M	2
M7557589, M7567533, M7628699, M7710656, M7712685, M7716819	2
M48505739, M48509977	2
M45294473, M45313697, M45319366, M45345987, M45351712, M45355102, M	2
M53284989, M53389674	3
M17094424, M17100613, M17102776, M17110200, M17122647	2
M35924293, M35996879	2
M19105332, M19113715	2
M11659175, M11687549, M11800009, M11814600	2
M53394546, M53399601, M53408271	2
M43677577, M43681133, M43702332, M43832583, M43835392	2
M28636193, M28652090, M28662492, M28664982, M28672236	3
M1578367, M1582553	3
M24621182, M24645679, M24669465, M24679328, M24704316, M24728340, M	2
M31775164, M31794129, M31796923	2
M52801450, M52818393	3
M38618720, M38620203, M38714905, M38725278	3
M29530246, M29603748, M29628570, M29675610, M29681103	2
M6095124, M6099373, M6100758, M6251873	3
M5657994, M5716226	3
M9351376, M9357080	2
M33729003, M33741567, M33848289	3
M3754196, M3770340, M3772766, M3780282, M3785165, M3786372, M378748	2
M13340711, M13343553, M13350626, M13353099	2
M27385405, M27399141	2
M42372522, M42415403, M42460842	3

M19564309, M19578167	2
M34180877, M34183285	2
M20020263, M20136873	2
M8415865, M8417539	2
M19949795, M19978583, M19980749, M19999899, M20012793	2
M28700656, M28738463, M28764455, M28767335, M28769616, M28791778, M	2
M1316317, M1324348, M1328920	2
M33189197, M33196343, M33201122	2
M4615318, M4619325, M4622989	2
M32597554, M32615216	2
M34991297, M34998015, M34998977, M35006514	2
M27586579, M27587392	2
M28493608, M28498559	3
M4402814, M4405324, M4540735	2
M33270614, M33275517, M33283799, M33294151	2
M40539850, M40548682, M40551552, M40560103	2
M49341703, M49402524, M49405735	2
M1726869, M1727197, M1785889, M1790904, M1856864, M1857380, M186071	2
M41055635, M41058825, M41070481, M41072199, M41081127, M41091314, M	2
M43321667, M43333524	2
M1021738, M1026661, M1030446, M1034149, M1038003, M1042722, M104421	2
M2922689, M2928400	3
M33304694, M33326696	2
M64193339, M64228452, M64281266	2
M29479873, M29519178, M29550265, M29594374, M29597230, M29610731	2
M31990187, M32000968	2
M27761227, M27813641, M27917910	2
M53277285, M53279180, M53284152, M53290156, M53306066	2
M19361037, M19477564	2
M20531562, M20537861, M20670448	2
M3587287, M3648752, M3652829	4
M3131, M15469	2
M34306807, M34308227, M34326095, M34328880, M34357559	2
M3908232, M3913828	2
M7304264, M7403147	2
M8690380, M8758066, M8761214, M8764515, M8808338, M8818335, M882409	2
M2228300, M2320627, M2323312	2
M5211073, M5227342, M5252591, M5285239, M5327627	4
M44449681, M44451822	2
M32110984, M32112908, M32120214, M32148919, M32150503, M32202932, M	3
M28824910, M28839707	2
M56070965, M56073117, M56074983, M56082056, M56097990, M56099602, M	2
M14660005, M14664605, M14682171, M14684118	2
M39406043, M39408960, M39411086, M39415451, M39417124	2
M47477830, M47480602	2
M26563383, M26696258	2
M9317, M24540, M154641	2

M34709556, M34714774, M34722783, M34725168, M34728718, M34735450	2
M42902957, M42905979	2
M51756636, M51769113	2
M4365668, M4442323, M4448291, M4452281, M4453727, M4459794, M446665	2
M28771402, M28801324, M28801947, M28838025	2
M39687787, M39689568, M39692632, M39694147, M39696843, M39701050, M	2
M15757081, M15797919	2
M838432, M848754, M857538, M861981, M884545, M898920, M905276, M915	2
M51430496, M51434650, M51436184, M51443768, M51528644, M51541075, M	2
M10353564, M10380265, M10390082, M10475648	2
M5824491, M5829963, M5854802, M5862251, M5869726	3
M35174869, M35180460	2
M19762666, M19763314, M19768276	2
M38031980, M38045289	2
M1821729, M1827480, M1834972	3
M24187726, M24302310	2
M57664844, M57814333	3
M434753, M488082	2
M5383026, M5466195, M5498894, M5513175, M5539062, M5542742	2
M15806903, M15822663	2
M15575958, M15579274, M15580824	3
M427814, M436263, M442260, M446469	2
M51377598, M51392246	3
M26116284, M26191313	2
M43495429, M43501227, M43502180, M43507229, M43515185	3
M5728731, M5799742, M5807213, M5810294	2
M5890169, M5893171, M5904656, M5907096	3
M4535506, M4537374, M4541030, M4552799, M4554509, M4560521, M456481	2
M9262562, M9273651, M9289513	2
M32634410, M32720964, M32752329, M32763975, M32793039	2
M39318498, M39322663, M39337105, M39339868	3
M22534490, M22567508, M22572348, M22572361, M22587842, M22601796, M	2
M32169701, M32174862, M32210995	2
M47740948, M47871959, M47879747, M47888687, M47892673, M47899199	4
M36964750, M36976742, M36982899, M37037815, M37051198, M37060443, M	2
M31611542, M31687259, M31694075, M31695638, M31753857	2
M3553309, M3555402	2
M33201216, M33203454, M33204939, M33210106	2
M10034907, M10058078, M10093352	2
M44340591, M44342170, M44343935, M44361726, M44390182, M44390200, M	2
M44995252, M44998927, M45019518	2
M11838384, M11841936, M11846606, M11850103, M11853985, M11862186, M	2
M17726585, M17729678, M17733634, M17743073, M17744645, M17750748, M	2
M43245349, M43266740, M43273843, M43286005, M43291739, M43298479	2
M7833113, M7862115	3
M35624786, M35630156, M35637102	2
M32397351, M32405842	3

M57855382, M57987915, M58005153, M58005178	2
M350390, M354922	2
M58636806, M58645488	2
M6220356, M6222394, M6224075	2
M36608573, M36618412, M36627839	2
M5220242, M5236878, M5243049	2
M13147934, M13173287	2
M5047277, M5062816, M5063629	2
M31281073, M31304333, M31403654, M31438615	2
M43144278, M43152515	2
M41615549, M41618779, M41626090, M41752309	3
M40205325, M40212423	2
M18496423, M18546173	3
M14224110, M14227260, M14236877, M14244658, M14245973, M14259213, M	2
M46526787, M46532745, M46541321, M46547522, M46635502, M46654284, M	2
M4634867, M4638915, M4645527, M4651342, M4668089, M4731293, M476475	3
M32648604, M32659940, M32695662	2
M60640516, M60642286	2
M17722777, M17727292	2
M4581624, M4583539	2
M57279347, M57288645, M57329155, M57335558, M57339831	2
M16091173, M16097890, M16104577, M16112077, M16114313, M16142484, M	2
M2926238, M2926959, M2929004, M2938189, M2941837, M2943726, M294597	3
M32768591, M32780971	2
M1503486, M1511469	2
M41538892, M41541589, M41546121, M41546355, M41549160, M41551168, M	2
M31215005, M31294694, M31297687	3
M33098779, M33112018	2
M4729427, M4739230, M4746700, M4751133	2
M18771154, M18892225	2
M12660337, M12676808, M12688649	2
M1514285, M1516939, M1539110	2
M16870367, M16909881	2
M51579739, M51657744	2
M25681481, M25800447	2
M33765192, M33769205	2
M5162758, M5167369	2
M33547438, M33672569, M33672757	2
M65369873, M65370796, M65376209, M65387097	2
M40756603, M40807908, M40880181	2
M36640724, M36663955, M36667050, M36676358, M36678043, M36682280, M	2
M11695153, M11729538	2
M2928175, M2933306	2
M1200001, M1236759	2
M47558047, M47572232, M47575636, M47706833, M47714436	4
M36968128, M36972144, M36988668	2
M32224785, M32294777, M32305451, M32312583	2

M63694496, M63696821	2
M17037047, M17078559, M17086859, M17090060	2
M52660821, M52663501, M52672850	2
M3562531, M3563991	2
M65464556, M65468440, M65472443	2
M33333015, M33352010, M33364964, M33379419, M33393792	2
M41665811, M41667304	2
M33311330, M33423127	2
M38914068, M38917654, M38928204, M38935161	2
M17249004, M17265341, M17270608, M17293932, M17337254, M17344721	2
M46410775, M46415170	2
M16769955, M16803017, M16829242, M16832920, M16851087, M16855851, M	2
M60980209, M61001554, M61068337	2
M11408692, M11410552, M11472605, M11494708, M11531032, M11559366, M	2
M408904, M418435	2
M4967608, M4975314, M4989894	2
M36628308, M36630284, M36632911	2
M2421493, M2429193	2
M32448723, M32454832	2
M247733, M248837, M254863, M258695	2
M16581578, M16596958, M16625914	2
M15456213, M15492024, M15528832	2
M52361277, M52377816, M52378386, M52395496, M52397372, M52408718, M	2
M52720696, M52739687, M52752864, M52755230	2
M8927545, M8937564	2
M35669404, M35782927	2
M26301267, M26327649, M26329641	2
M43437274, M43440718	2
M43790584, M43822455	3
M40691614, M40703002	2
M28906470, M28916173, M28924257, M28941507, M28947917	2
M7307970, M7319428, M7324809	2
M11793280, M11802029	3
M34584629, M34588455	2
M43267075, M43407593	2
M21262865, M21389117	2
M34531324, M34541105, M34558546, M34561970, M34566502, M34573186, M	3
M19312059, M19332440, M19353666, M19379837, M19393765	2
M51447818, M51449309	2
M40452409, M40468554, M40476330, M40488279, M40491689, M40518758	2
M1639320, M1641715	2
M40240630, M40243208, M40245333, M40246659	2
M58864985, M58893024, M58918670, M58952806, M58957357, M58967133, M	2
M43028201, M43031189	2
M13194433, M13205690, M13228033, M13233732	2
M4503373, M4512695, M4514793, M4518357	3
M7824100, M7837353, M7847047, M7862278, M7869729, M7875295, M788013	3

M39172208, M39196045	2
M2415666, M2419326, M2523980, M2572296	4
M29811163, M29816126, M29828701, M29835777	3
M11478899, M11488247	2
M5078136, M5088597	2
M6109149, M6116965	2
M10142183, M10148032	2
M12714123, M12715941, M12718657	2
M5098260, M5120598, M5125058, M5145940	2
M22304854, M22309829	2
M22808760, M22813382, M22819311, M22821622, M22827385, M22853929, M	2
M44188625, M44194409, M44197020, M44202449, M44211843	2
M30385644, M30460866	2
M28736339, M28799806, M28878315	4
M8924093, M8928779, M8936254, M8939178, M8944631, M8971560, M898521	2
M33225351, M33244910, M33272184	2
M8393475, M8433740	2
M32137267, M32157442	2
M32562473, M32572038	3
M12784308, M12821264	3
M31762610, M31765053, M31860047	2
M49873198, M49913462	2
M37219821, M37235365, M37264382, M37265649, M37333759, M37341359	3
M24160460, M24203642, M24211646, M24217385, M24223384, M24315295	2
M42874085, M42885912, M42888599	2
M39471130, M39474397, M39488945	2
M52852713, M52854967	2
M915900, M919670	3
M42986835, M42994710	2
M33525870, M33564260, M33678952	3
M49082885, M49093668, M49115290, M49116863, M49119293, M49121640, M	2
M57203213, M57255216, M57259211	2
M3246967, M3248911, M3252232, M3258713, M3263485, M3264317, M327533	2
M34205328, M34223076, M34242451	2
M32726709, M32740329, M32756071	2
M1672913, M1788704, M1809670	3
M42384051, M42397836, M42453350	2
M39728691, M39740149, M39746004, M39750428, M39756414	2
M1123836, M1129880, M1135521, M1160013	2
M42937432, M42943951	2
M32537494, M32542047, M32544087	4
M1383470, M1387267, M1438843, M1454107, M1460034, M1461631, M146525	3
M12861707, M12862527	2
M48199635, M48281964	2
M21648643, M21652516	2
M19441399, M19445563, M19452712, M19462011, M19493141	2
M34085824, M34227435	2

M11394022, M11413354, M11441141	2
M20044599, M20073128, M20077645	2
M6240178, M6249187	2
M46084580, M46095868, M46104171, M46111574, M46119333, M46135088, M	2
M17209917, M17236397	2
M36550916, M36568305, M36602026, M36604040, M36608900, M36692289	3
M36478463, M36481292, M36483670, M36484942, M36489681, M36493840, M	2
M38340718, M38344299	2
M15540202, M15546179, M15556528, M15565439	3
M29152117, M29184992	2
M51816835, M51819897, M51821557, M51824025, M51828203, M51830396	2
M18855804, M18877595, M18896790, M18908872, M18938167	2
M11836713, M11871006	3
M6404489, M6408137, M6414226, M6416380	2
M16948447, M16978455	2
M31769341, M31793644	3
M5429357, M5432233, M5457167, M5462493, M5464307, M5467360, M547315	2
M16232926, M16266910, M16358712	2
M39703381, M39710622, M39715134	2
M5248694, M5253550, M5255173, M5267010	2
M51135527, M51151071, M51151964, M51183142, M51184575, M51187586, M	3
M16929647, M16933089, M16938973	2
M40252539, M40348727, M40348883, M40352849, M40356535, M40370581, M	3
M51338417, M51344713, M51348556, M51355016, M51364569, M51366194	2
M12726156, M12731924	2
M9495315, M9517214, M9519801, M9523856, M9529096	2
M32798111, M32809951, M32816241	2
M8160710, M8162404	2
M14366796, M14376544, M14391496	3
M43643051, M43708345	2
M49430713, M49435213, M49445037, M49447990, M49448410	2
M47129098, M47189415, M47193724, M47202810, M47205097, M47205692, M	2
M6799046, M6854070, M6858180	2
M25939989, M25953338, M25971573, M26072554, M26097001	2
M12898556, M12904284, M12928102, M12940343, M12949014, M12956236, M	3
M39104904, M39121873, M39130380	2
M46836915, M46853341, M46856503	2
M14719474, M14723001, M14731793, M14739712, M14744474, M14760002, M	2
M5359978, M5369822, M5435619, M5435786, M5472002, M5473596, M547689	2
M27982966, M27996600, M28005620, M28007285, M28013047, M28020213, M	2
M32345428, M32348230, M32350847	3
M29588652, M29594093	2
M18002494, M18029388	2
M44223630, M44224476, M44228331, M44229190, M44244946, M44246931, M	2
M4177561, M4180023	2
M17016646, M17037139, M17050931, M17064252	2
M25825191, M25826281, M25903946, M25914946, M25919026, M25922703	2

M46891397, M46893878	2
M49751456, M49760446	2
M15613406, M15632738, M15645703, M15647604, M15654680, M15658292	2
M31626789, M31634655	2
M63712431, M63730241, M63742101, M63754438, M63761788, M63773617	2
M27563060, M27569839, M27574169, M27575053, M27579317, M27587602, M	2
M36211610, M36286312	3
M31087719, M31136951, M31244668	2
M43542566, M43546934, M43563771, M43567323, M43571969, M43582230, M	2
M65419276, M65429365, M65433152, M65451571	2
M17988, M40375	3
M19571416, M19576177, M19584457, M19592196	2
M4559405, M4560427, M4583491	2
M4172072, M4175152, M4178668, M4181965, M4193061, M4200576, M420419	3
M26259161, M26294614	2
M8146587, M8163918, M8201197, M8267432, M8285493, M8287388, M829719	2
M10590808, M10593406, M10617029, M10623781, M10625127	3
M44305813, M44341122, M44347664	2
M42649191, M42654987, M42670066, M42674076	2
M12537100, M12551124, M12592377, M12628891	2
M19674110, M19679318, M19684712, M19692573, M19721496	2
M18360483, M18387028	2
M9996013, M10002356, M10013894, M10048493, M10092778, M10100673, M1	2
M15177057, M15181476	2
M15517529, M15536530, M15554298	2
M1879533, M1886457	2
M32478033, M32540097, M32631847	2
M36274189, M36309200, M36310266, M36322946, M36340286, M36349641, M	2
M2209132, M2209958, M2213970, M2257460, M2277905, M2292429, M229503	2
M42246576, M42320760, M42328497, M42342183, M42349732, M42403206	2
M3581751, M3599036, M3606120, M3613620, M3618892, M3620539, M362254	3
M4093186, M4101023, M4103836	2
M41828847, M41971290, M41978881	3
M47605500, M47609200, M47613129, M47620627	2
M23414075, M23422926, M23434191, M23441001, M23445057, M23499037, M	2
M42025385, M42136118	2
M29644412, M29654380, M29663363, M29668569, M29676821, M29686487	2
M26060348, M26215000	2
M2934989, M2950418, M2970373, M2977537, M2978653	2
M46596430, M46603477, M46603988, M46611661, M46626527, M46632246, M	3
M15225470, M15229433, M15244888, M15248942, M15257110	2
M46775931, M46783331	2
M5360171, M5364035	2
M14304563, M14312729, M14365130, M14378429, M14379065, M14384247, M	2
M27885017, M27899503	2
M9877057, M9894303, M9895791	2
M641409, M642914, M649687, M657251, M661521, M666133, M680834, M683	2

M4499714, M4507776, M4508830, M4523353, M4528272	2
M15668238, M15673291	2
M36128172, M36131619, M36257472	3
M20496364, M20513042	2
M27617258, M27712742, M27759033	2
M29221444, M29232490	2
M7391059, M7393585	2
M37547406, M37547996, M37555771, M37598813	2
M26332067, M26334690, M26338552, M26341834, M26348999, M26352145, M	2
M20271503, M20350098, M20418484, M20428003	3
M22102589, M22173786	3
M11103905, M11252576	2
M12938020, M12939009, M12950929	2
M1325604, M1330921	2
M45570472, M45590214, M45696814	2
M35406417, M35410211, M35412567	2
M30210130, M30221394, M30236989, M30240614	3
M12430688, M12433139	2
M44746991, M44759732, M44764181, M44808775, M44810094, M44814120, M	2
M9838679, M9851351, M9858212	2
M29649115, M29658574	2
M46678117, M46681365	2
M12934061, M12977289	2
M38949825, M38958463, M38961989, M38966440, M38969033, M38974179, M	2
M3291116, M3392837	4
M22771811, M22780528	2
M5926987, M5982000, M6029142, M6053749, M6063229	2
M37652733, M37656012	3
M14134097, M14137805, M14161040, M14161415	2
M52071145, M52146153	2
M23454627, M23459191, M23475537, M23477564, M23489936, M23510523, M	2
M31888028, M31904559	2
M34547273, M34550178	2
M37313529, M37322704, M37324796, M37389925	2
M3948605, M3952221, M3965544	2
M28634649, M28645988, M28656599, M28661136, M28667424, M28673062	3
M9220831, M9287736, M9295649, M9302733, M9311508	5
M14138296, M14143008, M14145716, M14149447, M14192415, M14229913, M	4
M22131626, M22175633	2
M13782813, M13826639, M13833965, M13838068, M13843292	2
M34970384, M34973169, M34975464, M34983344, M34985303	2
M22709145, M22711993, M22722878, M22737640, M22747370	2
M22248097, M22399538	2
M48176159, M48189786, M48201525, M48207067	2
M20109754, M20116274, M20132825, M20150765, M20154314, M20159106, M	2
M60709831, M60713918, M60848005	3
M54791691, M54805243, M54808113, M54843116	2

M26840242, M26893531	3
M27066259, M27066699, M27114038, M27130663, M27159682, M27195440, M	2
M32861786, M32865736, M32866343	2
M33330252, M33332738	2
M11732513, M11734589, M11879637	2
M17341781, M17374281	2
M7406439, M7413056, M7422653, M7439208, M7448949, M7472590	2
M39543120, M39629065, M39632521	3
M16937, M22280	2
M30215491, M30365222	3
M22523612, M22534098, M22556170, M22571672, M22579466, M22619487	2
M10145239, M10147194	2
M45500480, M45655909	2
M25424399, M25427950, M25434403, M25439218, M25442517, M25445400, M	3
M1331482, M1339725, M1342826	2
M42034434, M42067401, M42158231	3
M1018519, M1025585, M1034401, M1037163, M1063376, M1077538, M108438	3
M36048510, M36107287	2
M12638826, M12642978, M12645041	2
M15999026, M16007412, M16027193, M16039652	2
M25379370, M25382242, M25406978, M25410616, M25413632, M25415086, M	2
M36188039, M36239932	2
M716643, M791895, M795227	2
M17208190, M17211833, M17218306	2
M44020644, M44125553, M44128707	4
M34249862, M34252401, M34253635, M34259646	2
M42014245, M42042986, M42163888	2
M1414999, M1562334, M1563635, M1567515, M1571626, M1574662	3
M17692339, M17705299	2
M5784585, M5910205	2
M46752178, M46775985, M46786535, M46812417	3
M3521133, M3529889	2
M16214338, M16215748, M16220539, M16222471, M16234428	2
M24857700, M24884893, M24891069, M24915947, M24936287, M24952804, M	2
M1541940, M1592019, M1592470, M1690041	2
M6447712, M6449304	2
M1303670, M1321547, M1333193	2
M23869545, M23873564	2
M46216836, M46233321	3
M51557621, M51559254, M51565099, M51566492, M51571708	3
M27635417, M27641288, M27649576, M27658957, M27785928	3
M35402866, M35435984, M35438932, M35444479, M35450661, M35548190	2
M9836652, M9843972, M9855441, M9861460, M9867330, M9869178	2
M32638862, M32639552, M32653015, M32661152, M32698754, M32723932, M	2
M42915709, M42928484	2
M38042081, M38066509	2
M28358185, M28472934	2

M7133986, M7146325, M7288012, M7289219, M7293546	2
M2754654, M2758798, M2815949, M2836422	2
M63795841, M63811643	2
M2428485, M2429738, M2439083, M2557987	2
M9067656, M9086792	2
M6810238, M6814413	2
M58739157, M58764880, M58808975, M58825013, M58840802	2
M45148085, M45169039, M45173007, M45177173, M45187463, M45204663, M	2
M11507409, M11513364	2
M44713069, M44718940	2
M2055650, M2059412, M2081402	2
M17240057, M17242429, M17255681, M17266552, M17269298, M17290974, M	3
M42949803, M43009217, M43021003, M43027355, M43081350	2
M1705261, M1710289, M1742698, M1822303, M1826963, M1832197, M183530	3
M13205920, M13219458, M13246508	2
M26917774, M26928515	2
M27846655, M27850427	2
M46034416, M46117715, M46117913	2
M1915073, M1918371, M1920519, M1925848, M1930933, M1936054, M194952	2
M32575820, M32583369	2
M1815484, M1820016, M1822313, M1825147, M1828787	3
M1367319, M1367922	2
M9295241, M9309996	2
M3738500, M3738586, M3814362, M3896657	2
M33679585, M33686013, M33694312	2
M3940842, M3943875	2
M236551, M245328	2
M39802625, M39806574, M39809638, M39815364, M39823189, M39828605, M	2
M1478076, M1482824	3
M8284717, M8300473	3
M5871481, M5921485, M5923374, M5931844, M5947493, M5990444, M599393	2
M11692350, M11763913, M11835255	2
M7197702, M7228446	2
M35710960, M35712916	2
M52725098, M52727870, M52730908, M52765693, M52817700, M52826753, M	3
M14829896, M14832816, M14979199	3
M42827927, M42833816	2
M40508471, M40652320	3
M37644910, M37650208, M37656022, M37657741, M37659098, M37676974	2
M38481552, M38484119, M38486434, M38492693, M38494908, M38496187, M	3
M15782306, M15783570, M15916183	3
M49421167, M49427300	2
M22594162, M22598588	2
M13627714, M13636140	2
M7586321, M7594495, M7619739, M7624568, M7631920	2
M36183376, M36186754	2
M36322451, M36417954	3

M64380060, M64397268, M64503405, M64532336	3
M10975452, M10992050, M10998186, M11007222	2
M23512043, M23521455, M23550680, M23647150	2
M54862298, M54867200, M54876551, M54878280, M54882266, M54884157	2
M47714663, M47727615	2
M1094657, M1102643, M1116292, M1253933	2
M26228287, M26278545	2
M11101134, M11116327, M11120135, M11128401, M11151481, M11153534	2
M4509546, M4512880, M4517292, M4527550, M4529275, M4545351, M455677	2
M13753946, M13768913	2
M837931, M997367	2
M15484162, M15491584, M15497506, M15507615, M15524867, M15540363, M	2
M13682201, M13688402, M13696681	2
M35399179, M35558344	2
M35343479, M35358021	2
M5244925, M5245767	2
M8909148, M8916223, M8920344, M8924564	2
M2765682, M2766719	2
M7485939, M7491250	2
M27956912, M27971629, M27985446	2
M2383881, M2492187, M2520775	4
M41375392, M41522606	2
M8997488, M9005534, M9009442	2
M44694525, M44701396, M44719888, M44728227	2
M7621021, M7661372	2
M19426842, M19488794, M19518529, M19538205	2
M16500028, M16538983	2
M18832180, M18841136, M18905700	2
M36146749, M36169881	2
M14311948, M14315273, M14319796	2
M1261244, M1306026, M1308113, M1313583, M1317853, M1319074, M135397	2
M9860243, M9863352, M9875365, M9882789, M9894726, M9917908, M992125	2
M10167879, M10178082, M10179845	2
M33774256, M33887245	2
M36935266, M36937552, M36954603, M36967153, M36968648, M36997218, M	3
M42923498, M42935005	2
M21353435, M21355981, M21414612, M21417246, M21426575, M21435668	2
M27243765, M27267828, M27279473, M27281497, M27293336, M27295573, M	3
M19405247, M19407382, M19434263, M19437093	2
M55761741, M55764873, M55789796, M55790323, M55801163, M55803239, M	2
M4204745, M4205415, M4211739, M4345400	3
M6428298, M6457201, M6465755, M6483703, M6508927, M6515278, M651867	2
M37480497, M37500168	2
M5976561, M6022189, M6025319	2
M32489472, M32495410, M32498658, M32506805, M32513164, M32554954, M	2
M20875588, M20880187	2
M38074193, M38085471, M38090918, M38095854, M38136172, M38165864, M	2

M44691287, M44727050, M44735959, M44803296	2
M18283879, M18310319, M18327822, M18343339, M18384351, M18421620	2
M14306655, M14316056, M14465533	2
M25763797, M25804401, M25853289, M25866868, M25889569, M25898371, M	2
M65327316, M65329637, M65334491, M65341237, M65363816	2
M10823589, M10838662, M10856908, M10857833, M10866482	2
M44457919, M44462320, M44479514, M44479604	2
M17823624, M17838654, M17844525, M17849282, M17855076, M17876324, M	2
M20414396, M20513268, M20549285	2
M36888188, M36890588, M36894310, M37006770, M37043803, M37046444	2
M19326993, M19353238, M19356307, M19358264	2
M896420, M924181	2
M49641558, M49642121, M49645992	2
M12657412, M12662544, M12669225, M12671822, M12674810	2
M12766997, M12770932, M12777393, M12783121, M12784832, M12787094, M	2
M12738308, M12743083, M12749373	2
M69473, M78569, M80394, M82669	2
M37097413, M37099059	2
M32039096, M32047452	2
M31582075, M31595131, M31607900	2
M10546315, M10587076, M10633331, M10635952	3
M28011049, M28063131	3
M12191107, M12220597	2
M35959145, M35965001, M35968452	3
M27653812, M27664948, M27681092, M27697983, M27718882	2
M38635858, M38639385, M38646202	2
M15254100, M15286599, M15314898, M15317975, M15408858	2
M27851319, M27853141, M27861266	2
M3840724, M3861067	2
M39196651, M39199846, M39205616, M39249135, M39250307, M39258158, M	2
M21992408, M22073017	3
M7766774, M7771440, M7771571, M7811945, M7826312, M7840463, M784352	2
M15271195, M15274439	2
M17881332, M17887328	3
M44475587, M44479393, M44485076	2
M51838152, M51844556, M51853635	2
M11138272, M11232043	2
M51684015, M51695380, M51699817, M51710426	2
M7469412, M7488979	2
M36818502, M36831690, M36834378, M36901789	2
M1679878, M1791774, M1812623, M1812830, M1814577, M1818790	3
M6397643, M6517858, M6551891	2
M52779833, M52783675	2
M2101276, M2103030, M2124052	2
M122113, M140299, M173094, M175421, M186170, M195034	2
M13053020, M13055134	2
M2332510, M2335970	2

M5539056, M5554248, M5559586, M5690677, M5696500	2
M25975699, M26127388, M26134899	2
M36258099, M36373409	3
M23212781, M23252078, M23274313, M23282884	2
M26780196, M26851285	3
M24118286, M24124856, M24137467	2
M48570484, M48571482, M48575202, M48581326, M48586812, M48598768, M	3
M14992384, M14999894, M15006174, M15019725, M15024053, M15025260, M	2
M50571815, M50574556, M50625001, M50629844, M50633172, M50671038, M	2
M3041385, M3118048	4
M15416354, M15432698, M15433984, M15438555, M15493670, M15495552	2
M1469208, M1478270	2
M7140307, M7153841, M7168948, M7175431	2
M40870405, M40887193, M40890213, M40914694	2
M33071469, M33113310, M33158204, M33159102, M33174177, M33178612, M	2
M39314942, M39315431	3
M3672299, M3676477	3
M35745521, M35750793, M35755972, M35758032	2
M54933260, M54940408, M54959931, M54962426, M54965546, M54975778, M	2
M2848655, M2854738, M2870739, M2873535, M2874319, M2879133, M288718	2
M4609957, M4610755, M4617798, M4627028, M4628088, M4630111, M469130	2
M2338678, M2343133	2
M37057237, M37069878, M37078265	3
M40882192, M40883223	2
M22644690, M22663820, M22674724, M22678354, M22693726, M22694645	2
M44470879, M44477431, M44494442	2
M37155799, M37257189, M37261981, M37275557, M37278969, M37294246, M	2
M3916242, M3919230, M3940121, M3942812, M3944300, M3947671, M401705	2
M20058967, M20067129, M20070930, M20080034, M20093497	2
M22004235, M22042716, M22056712, M22058366, M22114590, M22125645, M	2
M51508583, M51510468, M51514134	3
M36930480, M36933256, M36936253	2
M45399935, M45427871	2
M43676804, M43684444, M43686784, M43691642	2
M45922383, M46038533, M46081386	3
M20230862, M20319848, M20327741	2
M27093034, M27100569	2
M12864396, M12888982, M12895219, M12909344, M12915874, M12925383, M	3
M18699676, M18702167	2
M8573734, M8589946, M8604394, M8612202, M8621444, M8630356	2
M490036, M499864, M514152, M550983, M612253, M619876, M635743	2
M27644524, M27785348, M27797244	2
M44497876, M44500827	2
M27546196, M27558156	2
M15538378, M15552345, M15564182	2
M12230352, M12253275, M12254021, M12259701, M12270492, M12285973, M	2
M47901649, M47903264, M47944909, M47960845	2

M5175847, M5262528	2
M43197791, M43231271, M43256031, M43294788, M43297640	2
M38738044, M38770320, M38779441, M38789663, M38794714, M38797234, M	2
M23340729, M23346664, M23347889, M23376307, M23382768, M23398300	2
M11162711, M11250332	3
M42562558, M42674521, M42681507	4
M31468005, M31475424	2
M39909939, M39919193, M39924944	2
M47738651, M47745931	2
M49377832, M49378288, M49386217, M49389944, M49393748	2
M2125959, M2126744	2
M28766854, M28777455	2
M15656982, M15768733	3
M42933789, M42934567, M42971692, M42988416, M43029446	2
M3528992, M3630576, M3642460, M3647610, M3686228	2
M49788428, M49793341	2
M25918486, M25921551, M25925756, M25932487	2
M6317612, M6361448	2
M27722847, M27746557, M27761931	3
M1605347, M1608470, M1665208, M1716356, M1716410, M1717612, M172398	2
M5809862, M5812337, M5817354, M5832572	2
M23695279, M23701201, M23725650, M23730458, M23736144, M23747371	2
M22247841, M22406639	3
M49422447, M49425577, M49580180	2
M31615030, M31621260, M31625211, M31721836, M31731406, M31751464, M	2
M20075729, M20165784	2
M20413575, M20425632, M20441649, M20445876, M20449666, M20450698, M	2
M20797571, M20930920	2
M13497756, M13528582	2
M7986242, M7991410, M7999306, M8008786, M8014467, M8019337, M804249	2
M6505726, M6537628	2
M810906, M898371, M923358	4
M49706541, M49710136, M49713611, M49719011, M49807963, M49822274	3
M8293560, M8379088	2
M62810958, M62866896, M62874650	3
M15047059, M15119285	2
M6459485, M6461003, M6465224, M6466028, M6472536, M6475117	3
M46656342, M46659207, M46666408, M46668535, M46672582, M46686804	2
M3993221, M3995452, M3999129, M4001489, M4009811, M4020409, M404219	2
M27109856, M27123445, M27132868, M27156097, M27173322, M27267595	2
M28881591, M28926157	3
M6539471, M6541350, M6545795	2
M17515464, M17572249	2
M35362464, M35368407, M35371283	2
M46775424, M46882109, M46885944, M46887763	2
M12602275, M12608880, M12613833, M12615135, M12616668, M12636409	3
M34372995, M34398816, M34406009, M34407506, M34421517, M34423794, M	3

M39753501, M39761983, M39768208, M39797560, M39802123	2
M1380530, M1400514	2
M21808589, M21821201	2
M35807668, M35819855, M35820191	2
M48886234, M48889985	2
M6058262, M6068368, M6107284, M6113625, M6116545, M6118802, M613530	2
M11300293, M11312539, M11332541	2
M39241232, M39250798, M39267804, M39346578, M39400089	2
M37098660, M37102277	2
M44037492, M44040585, M44120811, M44123421, M44133928	2
M16701331, M16756956	2
M37182714, M37186896, M37192985	2
M34187072, M34197253	2
M12142124, M12216249, M12288380	3
M33555953, M33560342, M33564466, M33566482, M33575721, M33587427, M	3
M52883256, M53026311	3
M754260, M768255, M770964, M913709	3
M28325919, M28338237, M28340986, M28344266, M28357848, M28366190	2
M471906, M474597, M494517, M498282, M501486, M506176	2
M41999006, M42021501, M42060379, M42084809, M42117410	3
M19142760, M19148681, M19287825	2
M59059814, M59109317, M59149790, M59169801, M59180836, M59218354	3
M39374580, M39384782	3
M25261866, M25351138, M25362065, M25376258, M25387901, M25396489, M	2
M1196710, M1199151	3
M38696015, M38766214	2
M13341533, M13358360, M13382534, M13413446, M13423363, M13446488, M	2
M32231518, M32237928	2
M24799575, M24803661, M24812975, M24819497	3
M42787608, M42793769, M42817059, M42845762	2
M7042955, M7175877, M7177416	3
M18154007, M18161001	2
M30927914, M31075755	2
M2185175, M2189942, M2192549	3
M28701516, M28704633, M28715943	2
M51226823, M51237160, M51249119, M51253280	3
M11927408, M11936799	2
M18331784, M18373772	2
M24413203, M24429610, M24472516, M24567734	4
M37483619, M37487345, M37491082, M37495178, M37508952	2
M33999387, M34024092	2
M14082294, M14088413, M14101375	2
M49840193, M49849066	2
M1156952, M1183506, M1186413, M1195950	2
M50435153, M50438514, M50444534, M50455785, M50462004, M50465786, M	2
M3970060, M3971324, M3974712, M3981273	2
M7508838, M7516172, M7525461, M7549988	2

M791865, M799031, M806168, M810501, M813245, M833456, M842937, M861	2
M10919017, M10929954, M10943025, M11004663, M11013273, M11022759, M	3
M44162274, M44169766, M44182955, M44208455	2
M15134210, M15153927, M15270547, M15272541, M15281226, M15287633	3
M12604068, M12607743, M12643079	2
M48442113, M48458180, M48462534, M48464678, M48468753, M48471673, M	3
M10582687, M10584581	2
M17346128, M17347550, M17351903	2
M12161014, M12162530	2
M30553975, M30601190, M30606645, M30696076, M30699105, M30710732	2
M4299116, M4314844, M4329377, M4333454, M4344205	2
M9576448, M9589256, M9634042, M9634134, M9641267, M9645495, M964974	2
M27820512, M27820766, M27834198, M27846884, M27855367	2
M14410420, M14416783	2
M36757680, M36760637	2
M38378739, M38491191, M38492405	2
M34880918, M34973210	2
M13311096, M13312615, M13319169	2
M49807771, M49831409	2
M4777133, M4794468	2
M17566314, M17698464	3
M36815101, M36822577, M36826278	2
M35033769, M35036138, M35183644, M35184081, M35189523	2
M36950931, M36958962	2
M38550487, M38566568, M38571261	2
M8893704, M8896998	2
M2229453, M2235264, M2239506, M2241983, M2245057, M2283837, M231264	3
M6786241, M6789618, M6798824, M6807686, M6809959	2
M5040898, M5042716	2
M11268568, M11299016, M11300015, M11336829, M11341036, M11351634, M	2
M5301969, M5311550	2
M33785837, M33792120, M33804879, M33839589	2
M2615928, M2620362, M2639798	3
M18252796, M18260232, M18295198, M18308770, M18312143, M18313143	2
M5268261, M5281637, M5345085, M5350550, M5356179	2
M38487862, M38522135, M38617697	3
M38434755, M38437565, M38439669	2
M13733941, M13734514	2
M2332983, M2335389, M2340233, M2341678, M2438766, M2441399, M244618	2
M4924210, M4944097, M4951140, M4955755, M4957422, M4961832, M496490	2
M36696991, M36700769	2
M21458383, M21512561	2
M48031343, M48035952	2
M50953077, M50954255, M50956119, M50959805	2
M49956270, M50011366, M50019216, M50032892, M50039905, M50058170	2
M51239371, M51246955, M51248787, M51253054, M51258852	2
M49494533, M49499077, M49599594, M49652769	2

M7182957, M7183907	2
M1400, M4880, M34435, M38849, M41714, M47019, M49282, M55576, M5670[illegible]	3
M48909217, M48910344, M48918040, M48953332	2
M6823219, M6825243, M6857968, M6864442, M6878325, M6891454, M690145	2
M33113434, M33116028, M33139432	2
M7497525, M7508694	2
M14775623, M14784713, M14796414, M14798311	2
M35008538, M35015420	3
M38045200, M38051110	2
M35409148, M35413045	2
M2031997, M2043787, M2051354	2
M21035998, M21065092, M21166454	2
M51705845, M51711212	2
M11462064, M11465377	2
M305010, M333820, M366950, M370722, M441882, M450224, M456685	2
M4650155, M4656930, M4662272	2
M13049855, M13060295	2
M29013309, M29014451	2
M2865109, M2867789, M2990565, M2993480, M3007937	2
M19604896, M19645076	2
M6469785, M6480050, M6494047, M6504427, M6509615, M6519250, M652124	3
M841059, M841882	3
M26159047, M26204924	2
M10071430, M10084187, M10098822, M10100216, M10113790	2
M23227522, M23229761, M23256227, M23262201, M23286351, M23315900	2
M1255330, M1267729, M1268863, M1274023, M1277151, M1284296	2
M19925241, M19946249	2
M7666079, M7703614, M7715314, M7730539, M7735660, M7757081, M775908	2
M28697645, M28717947, M28718439, M28729784	2
M4888488, M4937369, M4939392, M4947054, M4949559, M4958029, M497874	3
M496956, M504336, M512733, M519645, M523594	2
M27369538, M27378471, M27397518, M27410234, M27423832, M27472990	2
M4540787, M4556914, M4562557, M4562668, M4564834, M4577393, M458655	2
M459599, M471720	2
M37618073, M37619898, M37622741, M37626580, M37630120	2
M36371765, M36383481	2
M19214300, M19254046, M19304626, M19315117, M19331834	3
M1337580, M1348119	3
M41039501, M41059389, M41062620, M41068840, M41079702	2
M26839860, M26871331, M26872855, M26885048, M26903092	3
M20047351, M20050959	2
M32751677, M32755919	2
M6279571, M6299473	2
M30111816, M30125890	2
M13698264, M13699783, M13702025	2
M52712349, M52716059, M52719774	2
M406930, M534039, M535783, M538768	2

M15437869, M15459862, M15469382, M15471616, M15484843, M15498409	2
M926678, M976868, M988918, M992122	2
M28582412, M28585520	2
M2766521, M2774412, M2775661, M2896810, M2900771, M2905544, M290994	2
M28948686, M28955283	2
M1107, M11891, M13402	3
M51950541, M51957373, M51961966, M51967351	2
M44061571, M44078906, M44128696	2
M15395418, M15399622	2
M17630383, M17638749, M17644664, M17656435, M17671138, M17692846	3
M50348460, M50356482, M50408005	2
M4670233, M4681423, M4689484, M4697950, M4703528	2
M3555215, M3558787, M3562675, M3567764, M3580104, M3593917, M359770	2
M14817476, M14819764	2
M11527520, M11528291, M11536509	2
M10735258, M10814303, M10830830	2
M34370126, M34381557	2
M52432723, M52436163, M52461482, M52464939	2
M16414275, M16432882, M16454960, M16471322, M16475648, M16477203, M	2
M22355574, M22384398, M22391259, M22397192, M22399374	2
M6059173, M6071117, M6081159	2
M14425865, M14460753	4
M56512940, M56517919, M56520116, M56569112, M56572096, M56576814, M	2
M43185747, M43206441	3
M8717667, M8726232, M8732271, M8738599, M8742930, M8753649, M876873	2
M16548981, M16625959	2
M30247851, M30355055	3
M4573, M7952	2
M13935931, M13949747	2
M7322689, M7333237, M7335828	2
M23251445, M23256175, M23264916, M23319257	3
M1360227, M1367582, M1369545	2
M46686385, M46687495	3
M54195117, M54198718, M54202691	2
M34772053, M34789610	2
M18538889, M18547511	2
M6373344, M6390280	2
M48218903, M48220373, M48254739, M48258368, M48261485, M48268247, M	2
M35710939, M35714052, M35717649, M35729374, M35744520, M35745435, M	3
M28205703, M28211169, M28219626, M28224500, M28264485	2
M1874870, M1885957, M1889808, M1898882, M1901279, M1908321, M191496	2
M3096790, M3101250, M3130880, M3217865, M3244665, M3254418	2
M24049583, M24054404	2
M40726076, M40729688, M40790334, M40801290, M40810952, M40827720	4
M19915106, M19922188	2
M4610735, M4613326, M4616502, M4650150, M4655410, M4673850, M469488	2
M3586808, M3587862, M3588772, M3594212, M3600273	2

M28177817, M28197291, M28230618	2
M43490344, M43501548, M43514372	3
M28923119, M28925560, M28931594	2
M28849284, M28859668, M28865883	2
M34757499, M34758714	2
M1102409, M1105491, M1111223, M1119961, M1127648	2
M50372976, M50384487	2
M32246160, M32249802	2
M3802837, M3855881, M3855902	2
M9309991, M9320016, M9325685, M9333204, M9340447, M9344310, M936798	2
M31923371, M31938222, M31994708, M32005932, M32069397	2
M16403540, M16421726, M16466226	2
M9433865, M9470623, M9478604	2
M4515, M108012	3
M1574084, M1575300, M1584251	3
M53053829, M53058080, M53102336, M53138709, M53151161, M53203904, M	3
M5570173, M5581063	3
M8182956, M8191485, M8340798	3
M36752804, M36754852	2
M32922489, M32926180	2
M50416901, M50419770	2
M12129995, M12141463, M12198707	2
M4026129, M4032981	2
M957920, M1069921, M1112391	2
M43307129, M43311226, M43318339, M43344693, M43349156, M43378240	2
M6592383, M6599933, M6619738, M6626759, M6739205, M6752072	2
M10412345, M10525710	2
M20091121, M20101137	2
M14887684, M14936046	2
M51680935, M51687077	2
M24024946, M24025128, M24030918, M24146322	4
M27747902, M27760167, M27825451, M27832466, M27845341	3
M36093261, M36094669	2
M8440439, M8481025, M8540639	2
M2803896, M2818639, M2914689	2
M28883874, M28896140, M28899879, M28904442, M28909079, M28925351, M	2
M42881544, M42894011, M42897390, M42904405, M42908288	2
M19852566, M19865296, M19875335, M19890026, M19910382	2
M52824458, M52832272	2
M36736760, M36738540	2
M11907888, M11909298, M11913076, M11919486, M11925243, M11930636, M	3
M12713330, M12718028	2
M50711422, M50848560	2
M14642921, M14656563	2
M4861421, M4885687, M4887407, M4890386, M4910329, M4945138, M495429	3
M19406584, M19410014, M19427739, M19429555, M19559836	2
M13243059, M13248690	2

M40818944, M40957322, M40975184, M40978767	2
M47768998, M47771864	2
M28978602, M28995367	2
M47528622, M47536720	2
M1084227, M1087184	2
M7323542, M7343316, M7351754	2
M32116377, M32161487	2
M43841064, M43868534, M43989391	3
M43005769, M43018079	3
M33015753, M33019681	2
M48958078, M48962547, M48966991, M48971079, M48978318, M48983638, M	2
M18388233, M18393440	2
M52493124, M52494027, M52499310, M52503528	2
M31318234, M31477779	2
M8004147, M8122789	3
M6140370, M6286009, M6286434, M6291287, M6294368	2
M8985206, M8999024, M9004946, M9014214, M9140719	3
M3187029, M3190021, M3200668, M3214306, M3228062, M3293033, M329506	2
M22707682, M22772934, M22840464	2
M58540337, M58555100	3
M22440027, M22453741, M22479444, M22494618, M22511370	3
M23811871, M23841109, M23863946	2
M12200264, M12204593, M12229180	2
M46767659, M46770116	2
M13078010, M13099310, M13103016, M13111526, M13121127, M13143013, M	2
M371482, M412749	2
M54612389, M54647448	3
M5497423, M5614699	2
M49001056, M49015939, M49023095, M49039726	2
M39810326, M39836890, M39918505	2
M7454432, M7457753, M7474338	2
M922459, M1000269, M1003573, M1008519, M1012993, M1022493, M1032441	2
M12273819, M12414674	2
M43703147, M43704266, M43717017	2
M41339833, M41345779, M41352022, M41367194, M41372976	2
M51718882, M51750537, M51782654, M51784228, M51790064, M51796341, M	3
M3185664, M3190621, M3220916	3
M9590232, M9599797, M9606152, M9635478, M9640556, M9643801, M964390	3
M44314426, M44330218	2
M18012583, M18019022, M18037709, M18042470, M18045750, M18046976	2
M49348482, M49357540, M49364335, M49367746	2
M6942753, M7003877, M7008503, M7051507	2
M4348412, M4352765, M4353166	2
M15109512, M15111670, M15122797	2
M39574536, M39576742	2
M39378369, M39390164	2
M6770879, M6927637	2

M33883433, M34015002, M34016216	5
M26468465, M26479717, M26492369	2
M48925807, M48945606, M48949398	2
M4625893, M4642298, M4652138, M4747715, M4749872, M4766623, M477223	2
M94318, M97250, M98613, M102007, M103374, M107397	2
M17711604, M17718537, M17729815	2
M52187273, M52194153	2
M1846448, M1847952	2
M65402411, M65412782	3
M32505021, M32507729, M32510802	2
M378298, M415256, M416231, M422308, M425753, M428298, M432529, M438	2
M44211911, M44214423	2
M397981, M403153	2
M3191013, M3319678	2
M9481138, M9485041, M9493317	2
M52312467, M52365855, M52372649, M52458366	2
M2899647, M2907033	3
M8825754, M8830222, M8835420, M8866442, M8873191, M8884576, M888837	2
M46441899, M46454959	2
M33581415, M33595466, M33611247, M33656108, M33674078, M33726436	3
M7717217, M7718791, M7723993, M7734947	2
M14826078, M14837470, M14839210, M14846945, M14850750, M14935532, M	3
M15187095, M15207995, M15209975, M15220816, M15223477, M15229011, M	3
M29603192, M29608315	2
M15484172, M15500714	2
M36176492, M36180628	2
M5622137, M5631094, M5632422	2
M44286062, M44445692	2
M9246118, M9293914	3
M1470417, M1495251, M1495978	3
M9692990, M9721704, M9725863	2
M45902777, M46010583, M46019860	3
M13064451, M13068730, M13070196, M13075552, M13080676, M13098220, M	3
M8878643, M8885041, M8888037, M8891430, M8892441, M8894454, M890276	2
M6695012, M6701096, M6708011, M6756131, M6766576, M6771148, M679469	3
M31782498, M31800136, M31809384, M31809596, M31817293, M31824733, M	2
M16819476, M16828398	2
M1201855, M1204113, M1228775, M1232362, M1234428	2
M29621401, M29628221	3
M1187294, M1189746	2
M8090442, M8093211	2
M38684274, M38686820, M38714777	2
M1543697, M1554416, M1587441, M1595044, M1604185	3
M31183688, M31222982, M31225349	3
M16864338, M16867779	2
M38305889, M38351774	2
M796920, M806210	2

M21388466, M21460828	2
M15324933, M15341764, M15376133, M15457454, M15460328, M15462633	2
M2609959, M2626260	2
M15583795, M15589712, M15602748	2
M15900320, M16000027	3
M36746055, M36748556, M36755071	2
M23671249, M23782049	2
M26739158, M26743598	2
M18101902, M18122089	2
M32031117, M32183069	3
M35579543, M35613453, M35639444, M35646336, M35669512, M35722842, M	2
M22591257, M22628129	2
M34749255, M34755677, M34779137, M34783032, M34791150, M34795407, M	2
M28513964, M28518722, M28523939, M28548611, M28592485, M28617795, M	2
M11785421, M11860836, M11929118	2
M6565049, M6591485	4
M16897481, M16908612, M16909919	2
M32935532, M32938476, M32943306, M32976056	2
M7526326, M7543408	2
M34133527, M34178086	2
M39788091, M39812300, M39838054, M39842765, M39852769, M39858046, M	2
M32256667, M32259732, M32268062, M32284173, M32304699, M32325401, M	2
M15522391, M15535557, M15535923	2
M1891142, M1892837	2
M4854378, M4867711, M5000542	2
M7800896, M7804047, M7826673, M7842699, M7852785, M7857031	3
M5773575, M5791030	2
M42939366, M42957383, M42974632	2
M23292258, M23298360, M23315655	2
M47398867, M47407047, M47438549, M47439560, M47467426, M47492421, M	2
M33370226, M33371992, M33377834, M33381922, M33383568, M33388021, M	2
M42624452, M42639530, M42699116, M42704266, M42726775, M42732860	2
M53433106, M53435253, M53438813, M53441475, M53445141, M53446972, M	2
M27231641, M27366950	3
M18137353, M18159634, M18193816, M18241448, M18248228	2
M15724386, M15736483	2
M2650930, M2657954, M2680864	2
M2815837, M2819063, M2823139, M2825989	2
M37143363, M37147648	2
M7898905, M7927187, M7932952, M7934936, M7947502, M7955828, M795809	2
M5744260, M5750722, M5755308	2
M5286606, M5293811, M5295328, M5316597, M5395767	2
M53524268, M53648296, M53677762, M53679427	3
M4214778, M4228762	3
M15878722, M16013828, M16029721	2
M826615, M844876	3
M10267918, M10334159	2

M1240336, M1263011, M1269407	2
M39740592, M39742149, M39750622	2
M965668, M968440, M982149, M990186, M1123061, M1124960	4
M56690885, M56709498	2
M34538184, M34680029	2
M10266620, M10268358, M10274107, M10281100, M10327849, M10330276, M	2
M51863469, M51863852, M51872971, M51880910	2
M41534993, M41617189, M41622125, M41627237, M41628494, M41639514, M	2
M24637536, M24788129	2
M11860441, M11877827	2
M23293979, M23326309, M23369309, M23375853	3
M8942505, M8944191, M8947360, M8949552, M8955263, M8961488, M896575	2
M26281150, M26384038	2
M28272942, M28285398, M28296007, M28312760, M28320559	2
M27490480, M27612551	2
M588163, M590233, M592207	3
M38539150, M38547698	2
M8194355, M8346266, M8347022	2
M7673034, M7678763, M7696981, M7713857, M7719166, M7724307, M772736	3
M38264314, M38266991, M38293904	2
M8856581, M8864813	2
M18678165, M18722845, M18726502	2
M771716, M786925, M794326, M804145, M806085, M818105, M821670, M824	2
M3858161, M3858374, M3867003	2
M14219190, M14246462, M14255590, M14260000, M14264846	2
M19664852, M19668583, M19674183, M19690203	2
M830015, M894157, M900654, M905958	2
M2990891, M2999823	2
M50805497, M50868598	2
M55963616, M55971003, M55976847, M55980854, M55984812, M55996924, M	2
M15250614, M15253491, M15261535, M15270744, M15271623, M15281417, M	2
M39084687, M39136200	2
M51025755, M51027840, M51044619, M51058764	4
M6933512, M6936819, M6945829	2
M893990, M897284, M899802, M903291, M914773	2
[truncated: 277,841 more chars]
